# Item difficulty index, discrimination index, and reliability of the 26 health professions licensing examinations in 2022, Korea: a psychometric study

**DOI:** 10.3352/jeehp.2023.20.31

**Published:** 2023-11-22

**Authors:** Yoon Hee Kim, Bo Hyun Kim, Joonki Kim, Bokyoung Jung, Sangyoung Bae

**Affiliations:** Research and Development Division, Korea Health Personnel Licensing Examination Institute, Seoul, Korea; Hallym University, Korea

**Keywords:** Educational measurement, Health occupations, Licensure, Reproducibility of results, Republic of Korea

## Abstract

**Purpose:**

This study presents item analysis results of the 26 health personnel licensing examinations managed by the Korea Health Personnel Licensing Examination Institute (KHPLEI) in 2022.

**Methods:**

The item difficulty index, item discrimination index, and reliability were calculated. The item discrimination index was calculated using a discrimination index based on the upper and lower 27% rule and the item-total correlation.

**Results:**

Out of 468,352 total examinees, 418,887 (89.4%) passed. The pass rates ranged from 27.3% for health educators level 1 to 97.1% for oriental medical doctors. Most examinations had a high average difficulty index, albeit to varying degrees, ranging from 61.3% for prosthetists and orthotists to 83.9% for care workers. The average discrimination index based on the upper and lower 27% rule ranged from 0.17 for oriental medical doctors to 0.38 for radiological technologists. The average item-total correlation ranged from 0.20 for oriental medical doctors to 0.38 for radiological technologists. The Cronbach α, as a measure of reliability, ranged from 0.872 for health educators-level 3 to 0.978 for medical technologists. The correlation coefficient between the average difficulty index and average discrimination index was -0.2452 (P=0.1557), that between the average difficulty index and the average item-total correlation was 0.3502 (P=0.0392), and that between the average discrimination index and the average item-total correlation was 0.7944 (P<0.0001).

**Conclusion:**

This technical report presents the item analysis results and reliability of the recent examinations by the KHPLEI, demonstrating an acceptable range of difficulty index and discrimination index values, as well as good reliability.

## Graphical abstract

[Fig f1-jeehp-20-31]

## Introduction

### Background

The Korea Health Personnel Licensing Examination Institute (KHPLEI) conducts national health personnel licensing examinations every year to assess whether candidates of health professionals have the minimal competency to practice medical and health care in the field. These national licensing examinations should consist of appropriate items that assess candidates for their competencies. After the examinations, it is essential to analyze the items’ difficulty, discrimination, and reliability and evaluate their appropriateness. If certain items show too high or low difficulty and discrimination index, the content of those items should be rechecked. The results of those analyses can be reflected in subsequent examinations.

### Objectives

The examination results were analyzed for item difficulty, discrimination, and reliability to evaluate the appropriateness of the items in 26 health personnel licensing examinations in Korea. The correlation between the average item difficulty index and the average item discrimination index was also assessed.

## Methods

### Ethics statement

This was not a human population study, but an analysis of the test results. Therefore, neither approval by the institutional review board nor obtainment of informed consent was required.

### Study design

This was a descriptive psychometric study based on examinees’ responses to the licensing examinations.

### Setting

Licensing examinations for 26 health professions from January to February 2022 were included for item analysis based on the classical test theory.

### Variables

The items’ difficulty index, discrimination index, and reliability were variables.

### Data source/measurement

Based on classical test theory, item difficulty, discrimination, and test reliability were calculated from the data. The item difficulty of each item was calculated as follows: P=(number of examines of correct choice)/(number of all examinees). It ranged from 0 to 1. The discrimination index was calculated using 2 commonly used methods. The first one was a method to find the difference in difficulty between the top 27% group and the bottom 27% group; this is called the upper and lower 27% rule method. The second method involved finding the correlation coefficients between items and the total score. The discrimination index and test reliability based on classical test theory were calculated only for tests with more than 100 examinees.

### Bias

There was no bias in selecting data. All data were included.

### Study size

Sample size estimation was not necessary because all data were included.

### Statistical methods

The item analysis based on classical test theory was done using IBM SPSS ver. 21.0 (IBM Corp.) The correlation analysis was done using DBSTAT ver. 5.0 (DBSTAT Co.).

## Results

### Pass rates

Table 1 shows the results of the 26 national licensure examinations for healthcare professionals administered in 2022 (Supplement 1). The analysis covered 36 examinations, including those that are administered more than once a year. The number of examinees ranged from as few as 11 to as many as 96,541. Among them, the examinations for midwives and health educators level 1 and the rehabilitation counselors level 2 had fewer than 100 examinees, so only the difficulty based on the classical test theory was analyzed, while other analyses, such as the discrimination and reliability according to classical test theory, were omitted. The pass rate varied from 27.3% to 97.1%. The pass rate of the health educator level 1 examination was less than 50%, which was markedly lower than those of other professions. The pass rates of physicians, dentists, midwives, nurses, oriental medical doctors, pharmacists, rehabilitation counselors, and the 38th, 39th, and 41st examinations for care workers were over 90%.

### Item analysis

The results of the item analysis of the examinations conducted in 2022 are presented in Table 2. Reliability was very high for all professions, with the lowest reliability shown by a Cronbach α value of 0.872 for the health educators level 3 examination. The correlation coefficient between the average difficulty index and the average discrimination index was -0.2452 (P=0.1557). The correlation coefficient between the average difficulty index and the average item-total correlation was 0.3502 (P=0.0392). The correlation coefficient between the average discrimination index and average item-total correlation was 0.7944 (P<0.0001).

## Discussion

### Key results

In 2022, 26 health professions licensing examinations displayed pass rates ranging from 27.3% to 97.1%. The examinee numbers varied widely, from 11 to 96,541. Average difficulty indexes ranged from 61.3% to 83.9%. The average item-total correlation ranged from 0.20 to 0.38. Overall reliability was high, with the lowest Cronbach α value being 0.872.

### Interpretation

For the analysis results based on the classical test theory, if the average difficulty is less than 50% to 60%, it is interpreted as moderate, 60% to 70% as somewhat easy, 70% to 80% as easy, and 80% or more as very easy [1]. According to these interpretations, the difficulty indexes of the above examinations were in the range of easy (9), somewhat easy (17), and moderate (12). For medical personnel, including physicians, dentists, nurses, and oriental medical doctors, the difficulty indexes were all in the somewhat easy category. Their pass rates were all over 90%. The Korean government controlled the school admission capacity for those professionals. The minimum requirement for them was not excessively high, and those examinees performed at a high level.

For the discriminant power, if the average discrimination index is less than 0.25, it is interpreted as a test with low discriminant power, 0.25 to 0.30 as a test with average discriminant power, and 0.30 or more as a test with good discriminant power [2]. According to this interpretation, 8 examinations had low discrimination power, 14 had average discrimination power, and 13 had good discrimination power. Regarding the item-total correlations, 7 examinations had low values, 11 had average values, and 17 had good values. For reliability, we found that all tests had Cronbach α values equal to or greater than 0.872, indicating high reliability across all examinations.

### Comparison with previous studies

Some studies have published item analyses of Korea’s health personnel licensing examinations. Investigations of the difficulty and discrimination indexes of the 64th (2000) and 65th (2001) Korean Medical Licensing Examinations (KMLE), based on classical test theory, revealed values of 71.9±21.7 and 68.3±23.5, and 0.22±0.11 and 0.18±0.13, respectively [3].

In addition to item analysis, the proportions of question items, according to their cognitive domain levels and types of multiple choice questions (MCQs), and the contents of medical knowledge of the KMLE conducted in 1992 and 1993 were explored. In 1992 and 1993, recall-level question items constituted 68.0% of all MCQ question items. The proportions of problem-solving level question items were only 7.7% in 1992 and 11.1% in 1993. The predominant types of MCQs were “best answer type” and “one correct answer type,” comprising 40.7% and 30.9%, respectively, in 1992, and 35.0% and 32.0%, respectively, in 1993 [4]. However, in 2022, problem-solving level question items constituted 55.3% of all MCQ question items, while recall-level question items accounted for only 6.0% [5].

For the nursing licensing examination, the outcomes of the 330-item examination, administered to 12,024 examinees in January 2004, were analyzed. According to classical test theory, the analysis of the items revealed a prevalence of easy items with a difficulty level of 0.7 or higher, and the correlation coefficient between item-total scores ranged from 0.2 to 0.3, indicating moderate discrimination [6]. Notably, there was a limited number of item analyses for the 26 personnel licensing examinations, likely due to challenges in data accessibility.

### Limitation

There were no data in the item analysis for each item. A further analysis of the information for each item would help understand the item’s qualities.

### Generalizability

The data only reflected an item analysis of health personnel licensing examinations in Korea.

### Suggestion for further study

Item response theory needs item analysis to find the more precise and stable item characteristics [7]. The item parameters based on item response theory are invariant and independent of the examinees’ characteristics. With those items, a tailored test, including computerized adaptive testing, can be achieved, where the item’s difficulty can adapt to the examinee’s ability. This method can enhance test efficiency and precision.

### Conclusion

The above results of the national health personnel licensing examinations conducted in 2022 showed an acceptable range of difficulty index values, discrimination index values, and reliability, although 8 out of the 25 examinations’ difficulty indexes were low discrimination. This suggests that all examinations administered by the KHPLEI fulfill their purpose—namely, assessing the minimum competency of health professionals to perform in their fields.

## Figures and Tables

**Figure f1-jeehp-20-31:**
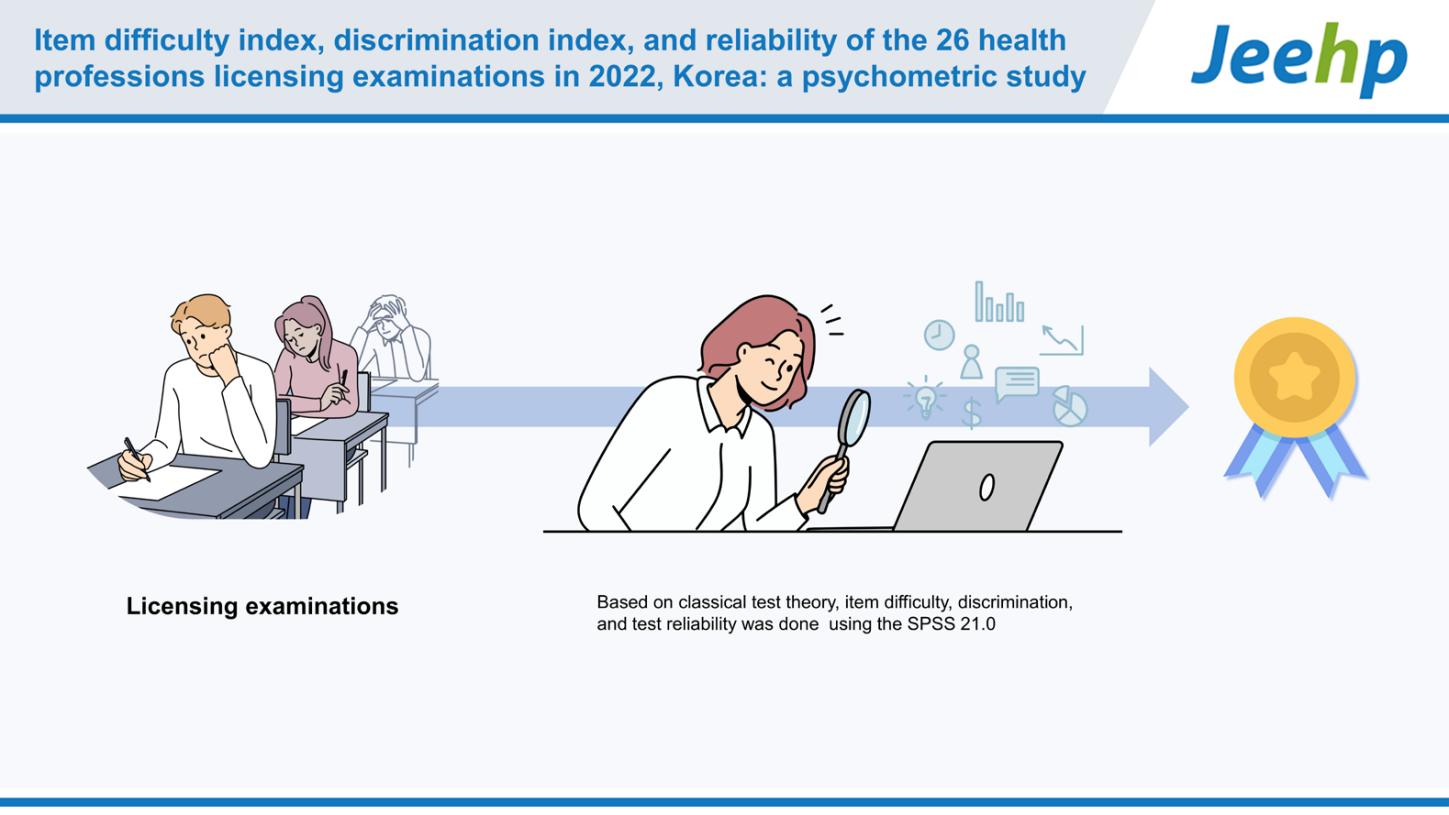


**Table 1.** Pass rate of examinees of the 26 national health professions licensing examinations of Korea in 2022 (as of the date of the successful applicant announcement)

Job titles	No. of examinees	No. of the successful candidate	Pass rate (%)	Date of examination (year/month/date)
Assistive technology professionals (4th)	248	167	67.3	2022/2/12
Care workers (38th)	85,148	77,830	91.4	2022/2/19
Care workers (39th)	93,860	85,509	91.1	2022/5/14
Care workers (40th)	80,116	69,958	87.3	2022/8/6
Care workers (41th)	96,541	87,005	90.1	2022/11/5
Dental hygienists (50th)	5,575	4,575	82.1	2022/12/11
Dental technicians (50th)	1,057	867	82	2022/11/26
Dentists (74th)	767	708	92.3	2022/1/14
Dietitians (46th)	5,398	3,629	67.2	2022/12/17
Emergency medical technicians–level 1 (28th)	1,736	1,530	88.1	2022/11/26
Emergency medical technicians–level 2 (28th)	1,148	966	84.1	2022/11/26
Health educators–level 1 (13th)	11	3	27.3	2022/2/12
Health educators–level 2 (13th)	111	69	62.2	2022/2/12
Health educators–level 3 (13th)	1,105	642	58.1	2022/2/12
Health information managers (39th)	2,745	1,516	55.2	2022/12/3
Herbal pharmacists (23th)	144	119	82.6	2022/1/7
Medical technologists (50th)	2,917	2,561	87.8	2022/12/11
Midwives (33th)	12	11	91.7	2022/1/14
Nurses (62th)	24,175	23,362	96.6	2022/1/21
Nurse assistants (first-half)	22,075	18,198	82.4	2022/3/19
Nurse assistants (second-half)	17,840	14,812	83	2022/9/24
Occupational therapists (50th)	1,995	1,577	79	2022/12/3
Optometrists (35th)	1,619	1,213	74.9	2022/12/17
Oriental medical doctors (77th)	753	731	97.1	2022/1/14
Oriental medicine dispenser (29th)	-	-	-	
Pharmacists (73th)	1,993	1,840	92.3	2022/1/21
Physical therapists (50th)	5,430	4,677	86.7	2022/12/11
Physicians (86th)^a)^	6,043a)	5,786	95.7	2022/1/6–7
Prosthetists and orthotists (23th)	160	83	51.9	2022/11/26
Radiological technologists (50th)	2,609	1,958	75	2022/12/17
Rehabilitation counselors–level 1 (6th)	412	376	91.9	2022/2/12
Rehabilitation counselors–level 2 (6th)	49	45	91.8	2022/2/12
Rehabilitation counselors–level 3 (6th)	-	-	-	2022/2/12
Sanitary technicians (44th)	8,221	5,019	61.1	2022/11/29
Speech-language pathologists–level 1 (11th)	946	559	59.1	2022/12/3
Speech-language pathologists–level 2 (11th)	1,436	986	68.7	2022/12/3

a) This reflects those who waived the 85th practical examination and took either the 86th first-half practical examination or the second-half practical examination.

**Table 2.** Difficulty index, upper and lower group discrimination index, item-total correlation, and reliability index presented as Cronbach α for 26 health professions licensing examinations in 2022, Korea

Job title	No. of subjects	No. of items	Difficulty index (%)	Upper and lower group discrimination^a)^	Item-total correlation^a)^	Reliability Cronbach α
Assistive technology professionals (4th)	2	170	64.4±24.3	0.23±0.15	0.20±0.12	0.893
Care workers (38th-AM)	2	80	82.2±15.2	0.27±0.14	0.32±0.08	0.903
Care workers (38th-PM)	2	80	81.8±13.5	0.27±0.12	0.33±0.07	0.906
Care workers(39th-AM)	2	80	83.9±13.0	0.27±.014	0.36±0.08	0.92
Care workers (39th-PM)	2	80	82.3±14.0	0.29±0.14	0.35±0.10	0.919
Care workers (40th-AM)	2	80	81.4±14.0	0.30±0.14	0.35±0.09	0.919
Care workers (40th-PM)	2	80	78.3±16.8	0.33±0.15	0.36±0.11	0.925
Care workers (41th-AM)	2	80	81.4±16.3	0.29±0.14	0.35±0.09	0.916
Care workers (41th-PM)	2	80	82.6±12.5	0.29±0.13	0.34±0.09	0.916
Dental hygienists (50th)	2	200	72.3±17.3	0.29±0.12	0.30±0.10	0.952
Dental technicians (50th)	3	205	70.8±19.7	0.30±0.13	0.31±0.11	0.955
Dentists (74th)	13	364	78.3±19.1	0.19±0.12	0.22±0.09	0.949
Dietitians (46th)	4	220	67.0±17.9	0.37±0.17	0.33±0.12	0.966
Emergency medical technicians–level 1 (28th)	5	230	74.4±18.6	0.26±0.13	0.26±0.10	0.945
Emergency medical technicians–level 2 (28th)	5	140	66.7±21.4	0.24±0.13	0.23±0.10	0.892
Health educators–level 1 (13th)	3	60	64.2±29.7	-	-	-
Health educators–level 2 (13th)	8	180	62.4±23.5	0.27±0.17	0.25±0.13	0.918
Health educators–level 3 (13th)	4	110	62.3±22.1	0.27±0.16	0.26±0.12	0.872
Health information managers (39th)	3	230	63.0±21.8	0.33±0.17	0.29±0.13	0.959
Medical technologists (50th)	4	280	80.6±13.2	0.32±0.15	0.37±0.12	0.978
Midwives (33th)	4	165	74.2±23.9	-	-	-
Nurses (62th)	8	295	78.9±19.9	0.18±0.13	0.23±0.10	0.934
Nurse assistants (1st half)	4	100	73.9±20.3	0.34±0.18	0.36±0.13	0.932
Nurse assistants (2nd half)	4	100	76.2±18.3	0.34±0.16	0.37±0.13	0.935
Occupational therapists (50th)	4	240	74.8±18.0	0.28±0.15	0.29±0.11	0.959
Optometrists (35th)	4	250	71.9±16.4	0.38±0.15	0.37±0.12	0.975
Oriental medical doctors (77th)	11	340	76.8±22.0	0.17±0.11	0.20±0.10	0.931
Oriental medicine pharmacists (23th)	3	250	69.8±21.9	0.29±0.17	0.32±0.18	0.963
Pharmacists (73th)	4	350	79.2±23.3	0.19±0.11	0.24±0.11	0.948
Physical therapists (50th)	5	260	76.1±18.1	0.28±0.14	0.32±0.14	0.965
Physicians (86th)	3	320	78.1±20.6	0.20±0.14	0.24±0.11	0.949
Prosthetists and orthotists (23th)	7	210	61.3±19.2	0.33±0.17	0.28±0.13	0.950
Radiological technologists (50th)	4	250	72.8±17.4	0.38±0.16	0.37±0.13	0.976
Rehabilitation counselors–level 1 (6th)	7	120	75.4±19.1	0.24±0.16	0.27±0.11	0.886
Rehabilitation counselors–level 2 (6th)	7	150	81.0±17.7	-	-	-
Sanitary technicians (44th)	6	220	64.9±20.6	0.32±0.17	0.29±0.13	0.954
Speech-language pathologists–level 1 (11th)	6	140	62.9±23.1	0.26±0.15	0.25±0.12	0.893
Speech-language pathologists–level 2 (11th)	5	150	66.5±19.5	0.36±0.17	0.34±0.13	0.947

Values are presented as number or mean±standard deviation unless otherwise stated.

a) Discrimination index and reliability were not calculated for health professions with fewer than 100 candidates.

## Supplementary materials

Supplement 1. Item analysis results of 26 health professions licensing examinations administered during late 2022 and early 2023.

Supplement 2. Audio recording of the abstract.
