# Supplementary material for: Item difficulty index, discrimination index, and reliability of the 26 health professions licensing examinations in 2022, Korea: a psychometric study
Source: J Educ Eval Health Prof. 2023 Nov 22;20:31. doi: 10.3352/jeehp.2023.20.31 (PMC11959405; doi:10.3352/jeehp.2023.20.31)
Supplement: Supplementary file 1 — Supplement 1. Item analysis results of 26 health professions licensing examinations administered during late 2022 and early 2023. [file jeehp-20-31_Suppl1.zip › 2022│Γ╡╡ ╗≤╣▌▒Γ ░ú╚ú┴╢╣1⁄2╗τ ▒╣░í╜├╟Φ ║╨╝«░ß░·.pdf]

2022년도 상반기
간호조무사 국가시험
문항분석 결과

국민이 신뢰하고 감동하는 시험평가기관
한국보건의료인국가시험원
KOREA HEALTH PERSONNEL LICENSING EXAMINATION INSTITUTE

일반 용어 정의

☐ **평균**

- 집단에서의 대표적 경향 값으로 전체 값을 더하여 총 응시자로 나눈 값

☐ **표준편차**

- 평균과 각 점수의 차이인 편차들의 평균으로 점수가 흩어져 분포되어 있는 정도

☐ **추정난이도**

- 문항개발자가 예측한 정답률

☐ **검사이론**

- 검사와 검사를 구성하고 있는 문항의 양호도를 분석 및 평가하는 방법을 정의한 이론체계
- 대표적으로 고전검사이론과 문항반응이론이 있음

고전검사이론 용어 정의

□ **고전검사이론(Classical Test Theory; CTT)**

- 검사의 질을 분석하는 검사이론 중 한 가지로 19세기 말부터 전개되어 현재까지 주로 사용되고 있는 검사이론임
- 고전검사이론에 의한 문항과 응시자 능력 추정치는 다음과 같음

○ **문항난이도**

· 검사 문항의 쉽고 어려운 정도를 나타내는 지수
· 난이도 지수는 총 반응 수에 대한 정답 반응 수의 비율로 문항의 정답률임
· 문항난이도는 0~100까지의 값을 가짐
· 난이도 값이 큰 경우, 쉬운 문항으로 '난이도가 낮다'라고 해석하며, 난이도 값이 작은 경우, 어려운 문항으로 '난이도가 높다'라고 해석함

○ **문항변별도**

· 각 문항이 응시자의 능력 수준을 변별할 수 있는 정도를 나타내는 지수
· 문항변별도는 −1~+1까지의 값을 가지며, 1에 가까울수록 변별력 크다고 해석함
· 일반적으로 문항변별도가 0.3 이상이면 우수한 문항으로 평가함
· 구하는 방식에는 '상하위집단 구분법', '문항-총점 상관계수' 등이 있음
  1) **변별도 1(상하위구분법):** 상위 27%와 하위 27% 집단의 난이도 차이를 구하는 방식
  2) **변별도 2(상관계수법):** 문항-총점과의 상관계수로 구하는 방식

○ **신뢰도**

· 시험이 평가하고자 하는 것을 일관성 있게 측정하는가로 시험이 오차없이 정확하게 측정한 정도를 의미함
· 국시원에서는 문항의 내적일관성(Cronbach α)으로 신뢰도를 추정하며 1에 가까울수록 신뢰도가 높다고 해석함

문항반응이론 용어 정의

□ **문항반응이론(Item Response Theory; IRT)**

- 고전검사이론과 같이 검사의 질을 분석하는 검사이론 중 한 가지로 20세기 초에 제안됨
- 문항분석과 응시자의 능력을 추정하는데 전제 및 방법에서 고전검사이론과 차이를 보이며, 문항반응이론에 의한 문항과 응시자 능력 추정치는 다음과 같음

○ **문항난이도**

· 검사 문항의 쉽고 어려운 정도를 나타내는 지수
· 난이도 지수는 응시자의 50%가 정답을 맞힐 것으로 기대되는 능력수준임
· 난이도의 범위는 무한하나 일반적으로 -2.0 이하는 매우 쉬운 것으로, +2.0 이상은 매우 어려운 것으로 해석함

○ **문항변별도**

· 각 문항이 응시자의 능력 수준을 변별할 수 있는 정도를 나타내는 지수
· 능력이 증가함에 따라 정답을 맞힐 확률이 얼마나 변화하는지를 나타내는 지수로 지수가 클수록 변별력이 크다고 해석함
· 변별도의 범위는 무한하나 일반적으로 0.0 이하는 변별력이 없는 것으로 해석하며, 0.65 이상이면 우수한 문항으로 해석함

○ **검사정보함수**

· 검사에 대한 반응으로부터 구할 수 있는 정보량을 나타내는 그래프로, 검사가 응시자의 능력을 얼마나 정확히 추정하는가에 대해 알려줌
· 검사정보함수는 측정오차의 분산과 역의 관계를 가짐
· 검사정보함수에서 최대정보를 가지는 지점의 능력수준에 해당하는 응시자의 능력을 가장 정확히 추정할 수 있다고 해석함

목 차

I. 시행 결과 ········· 6
1. 시험 현황 ········· 7
1) 시험명 ········· 7
2) 시험시행일 ········· 7
3) 응시현황 ········· 7
4) 과목별 문항 수, 배점 및 과락 점수 ········· 7
2. 합격률과 평균성적 ········· 7
1) 합격 및 불합격 현황 ········· 7
2) 과목별 과락자수 내역 ········· 7
3) 전회 대비 합격률과 평균성적 ········· 8

II. 문항분석 결과 ········· 9
1. 성적 ········· 10
1) 전체 성적분포도 ········· 10
2) 과목별 성적분포도 ········· 11
2. 난이도와 변별도 ········· 12
1) 전체 난이도와 변별도 ········· 12
2) 과목별 난이도와 변별도 ········· 15
3) 지식수준별 난이도와 변별도 ········· 26
4) 자료유형별 난이도와 변별도 ········· 34
3. 난이도와 변별도 간 산포도 ········· 40
1) 전체 난이도와 변별도 간 산포도 ········· 40
2) 과목별 난이도와 변별도 간 산포도 ········· 40
4. 신뢰도 분석 ········· 43

I. 시행 결과

1. 시험 현황

1) 시험명: 2022년도 하반기 간호조무사 자격시험

2) 시험시행일: 2022년 3월 19일

3) 응시현황

응시대상자수	결시자수	부정행위자수	응시자 준수사항 위반자 수		응시자수 (%)
			휴대폰 소지등	신분증 미지참	
23,197	1,103	-	-	-	22,075 (95.2)

4) 과목별 문항 수, 배점 및 과락 점수

교 시	과 목 명	문제 수	배점	총점	합격자 점수기준	
					과락기준	총점 합격기준
1교시	1. 기초간호학 개요	35	1	35	14	60점 이상
2교시	2. 보건간호학 개요	15	1	15	6	
3교시	3. 공중보건학개론	20	1	20	8	
4교시	4. 실기	30	1	30	12	
계		100		100		

2. 합격률과 평균성적

1) 합격 및 불합격 현황

합격자수 (%)	불합격자수(%)				채점보류자수
	평락	과락	기권	계	
18,198(82.4)	3,784(17.2)	93(0.,)	-	3,877(17.6)	19

2) 과목별 과락자수 내역

과목명 / 과락자수	기초간호학 개요	보건간호학 개요	공중보건학 개론	실기
과목별 과락자 수	-	16	76	1
2과목 과락자 수	-			

3) 전회 대비 합격률과 평균성적

회차	년도	합격률(%)	평균성적	표준편차	백분율 환산점수
2020년 상반기	2020	84.8	74.9	13.3	74.9
2020년 하반기	2020	91.9	81.8	12.8	81.8
2021년 상반기	2021	85.7	76.4	14.1	76.4
2021년 하반기	2021	87.0	77.6	13.8	77.6
**2022년 상반기**	**2022**	**82.4**	**73.9**	**14.1**	**73.9**

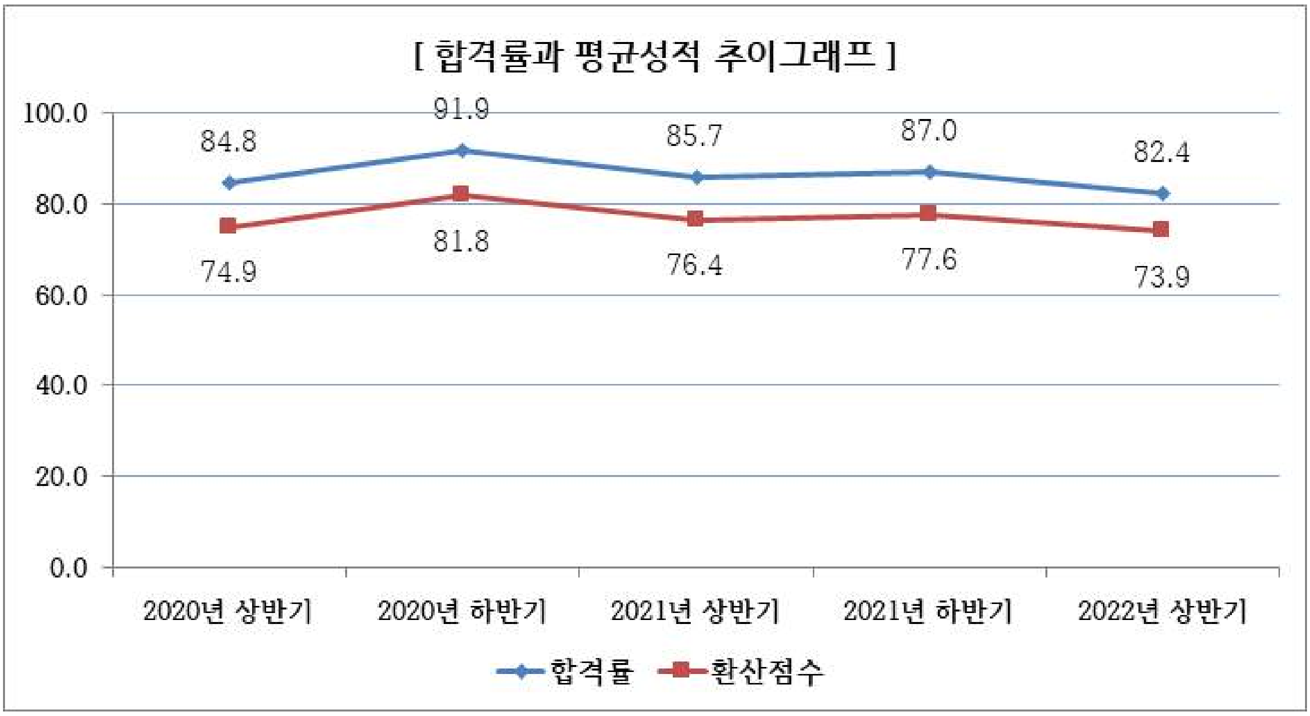

해석

- 전년 대비 합격률은 4.6 감소하였으며, 백분율 환산점수는 3.7 점 감소함
- 표준편차는 0.3 증가함

Ⅱ. 문항분석 결과

1. 성적

1) 전체 성적분포도

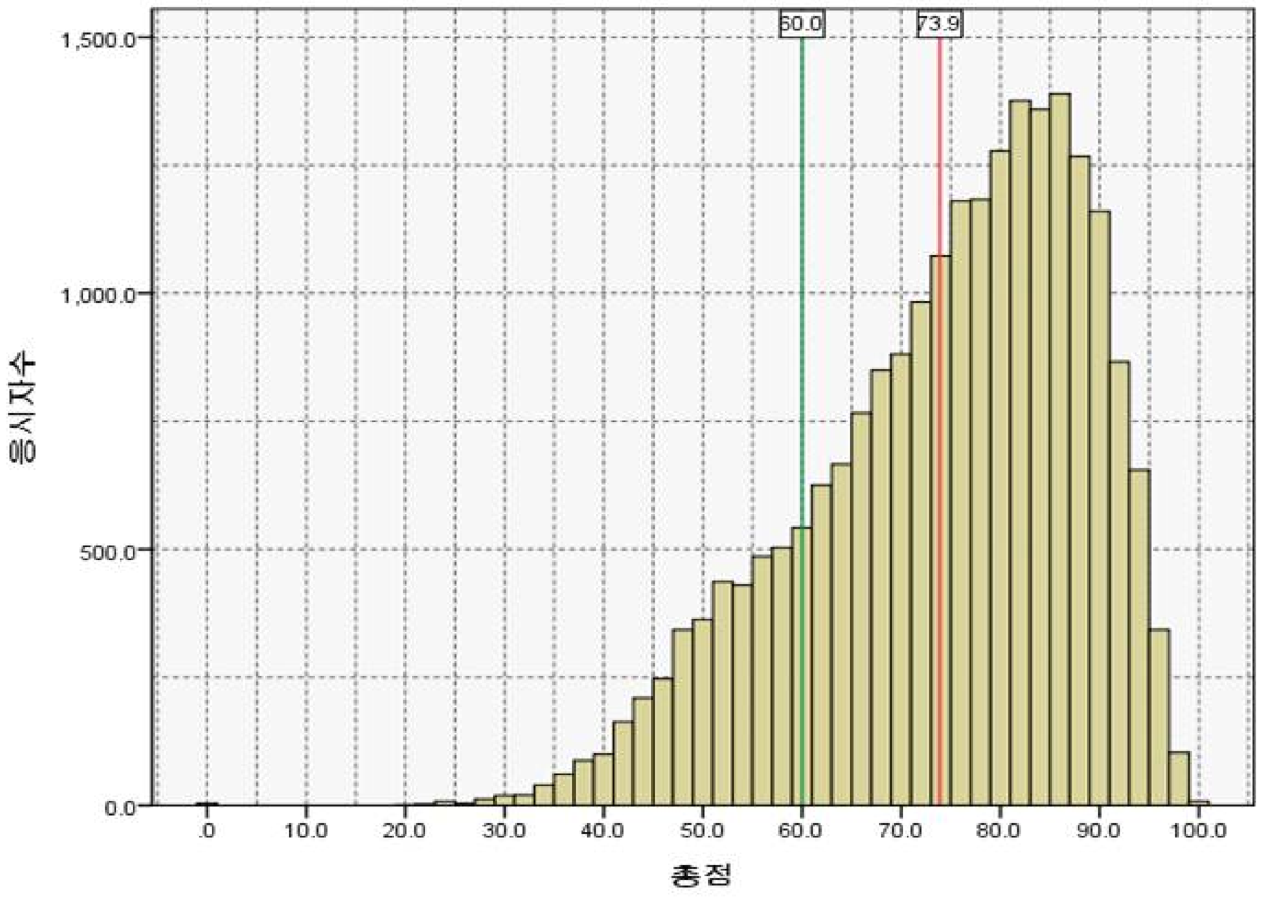

응시자	총점	합격선	평균성적	표준편차
22,094※	100	60	73.9	14.1

※ 22,094명은 응시자(22,075)에 채점보류자(19명)를 포함한 수치임

2) 과목별 성적분포도

가) 기초간호학개요

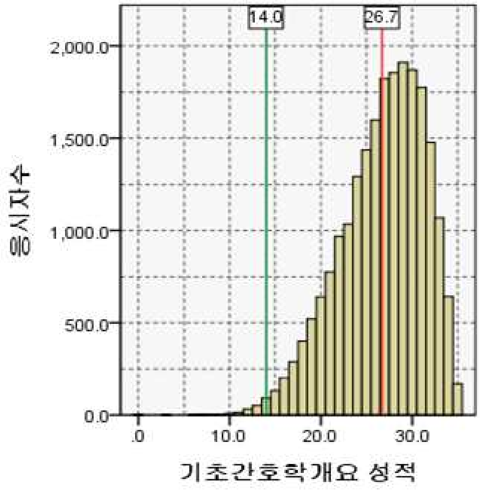

총점	과락선	평균성적	표준편차
35	14	26.7	4.6

나) 보건간호학개요

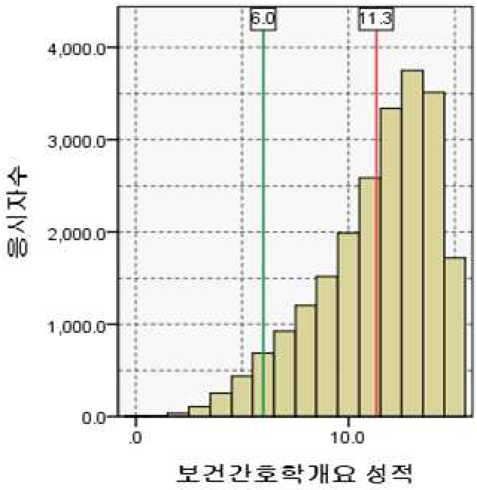

총점	과락선	평균성적	표준편차
15	6	11.3	2.7

다) 공중보건학개론

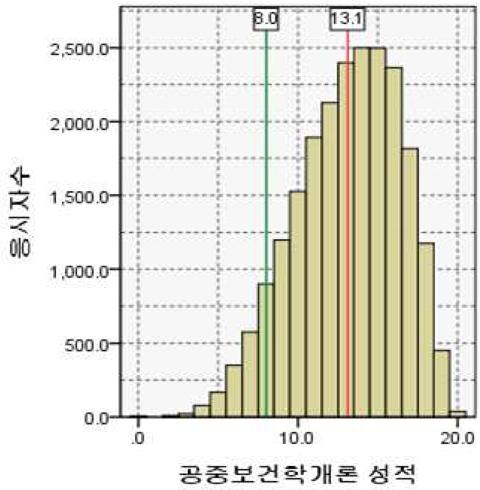

총점	과락선	평균성적	표준편차
20	8	13.1	3.3

실기

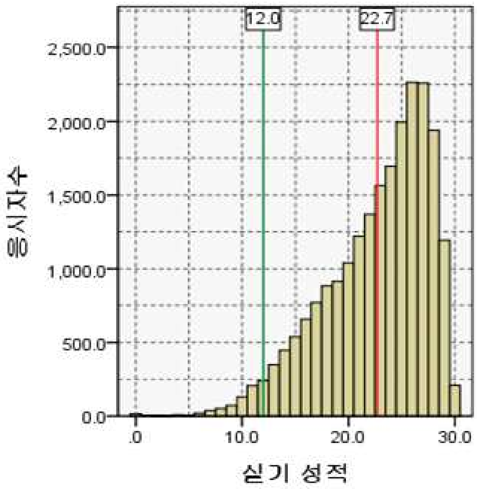

총점	과락선	평균성적	표준편차
30	12	22.7	4.9

2. 난이도와 변별도

1) 전체 난이도와 변별도

가) 전회 대비 전체 난이도와 변별도

회차	난이도		변별도1		변별도2	
	평균	표준편차	평균	표준편차	평균	표준편차
2020년 상반기	74.9	19.7	0.31	0.17	0.35	0.13
2020년 하반기	81.8	14.8	0.30	0.17	0.36	0.13
2021년 상반기	76.4	17.1	0.33	0.16	0.37	0.12
2021년 하반기	77.6	16.0	0.33	0.18	0.36	0.13
**2022년 상반기**	**73.9**	**20.3**	**0.34**	**0.18**	**0.36**	**0.13**

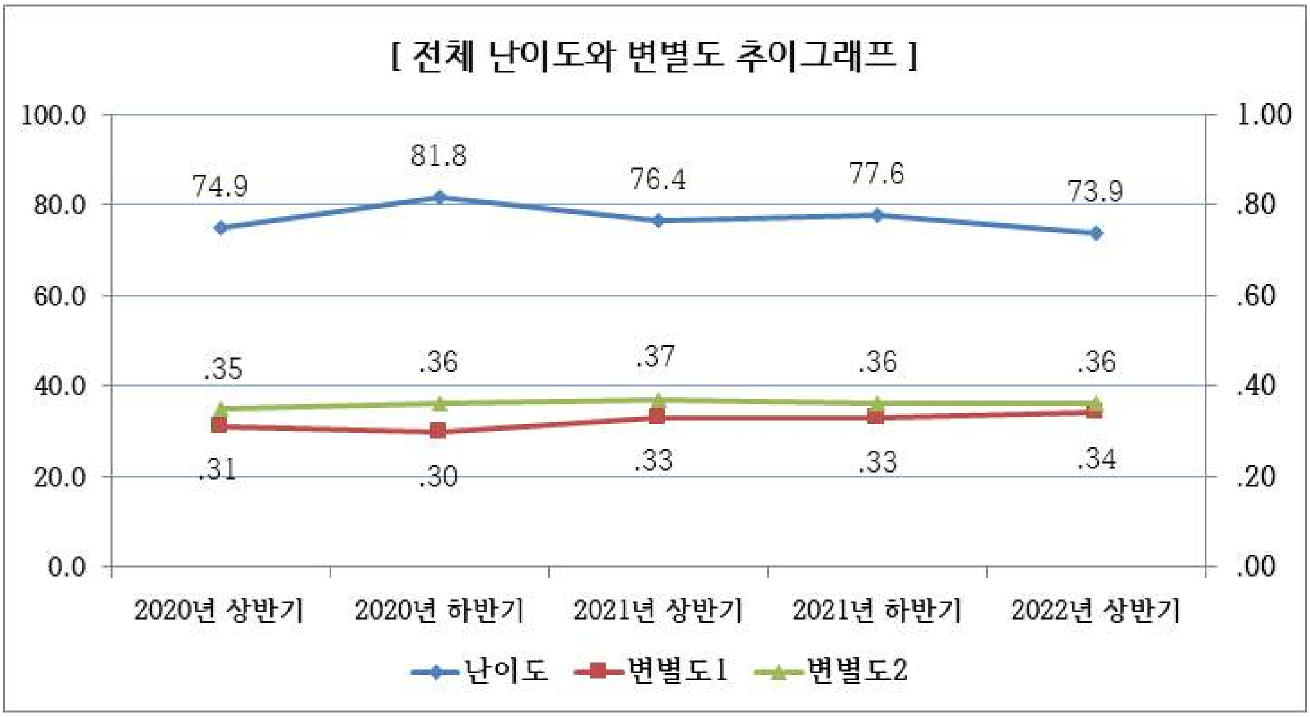

해석

- 전년 대비 난이도 지수는 3.7 감소함
- 변별도 1 지수는 .01 증가하였으며, 변별도 2 지수는 변화 없음

나) 전체 난이도와 변별도 분포도 및 비율분석

(1) 전체 난이도 분포도 및 비율분석

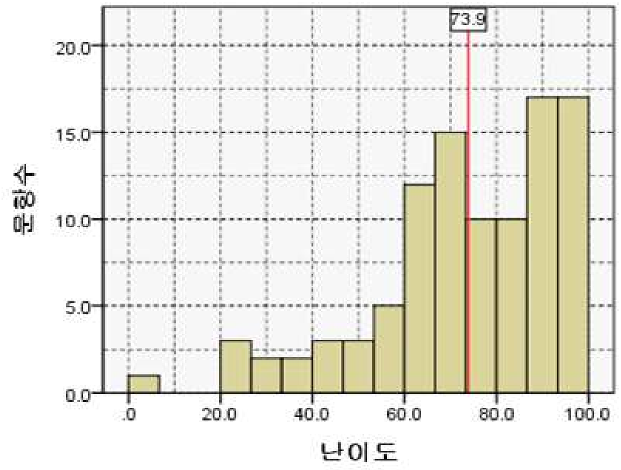

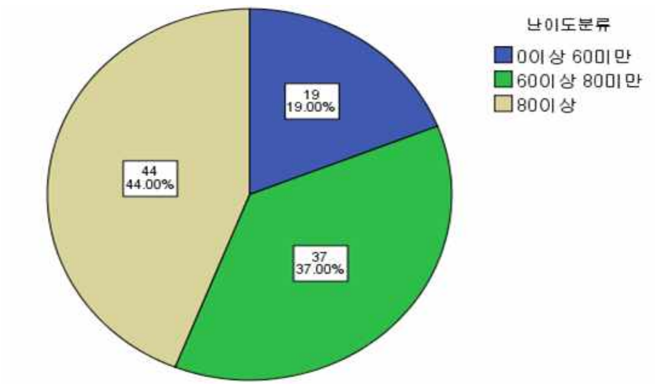

총점	난이도	표준편차
100	73.9	20.3

난이도	문항수	비율(%)
0~60미만	19	19.0
60~80미만	37	37.0
80~100	44	44.0
전체	100	100.0

(2) 전체 변별도1 분포도 및 비율분석

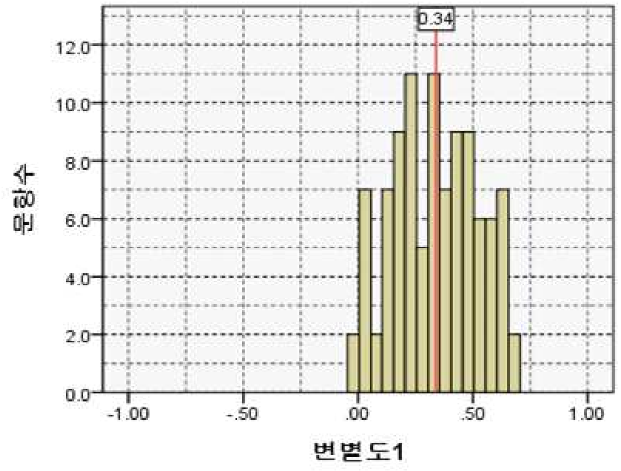

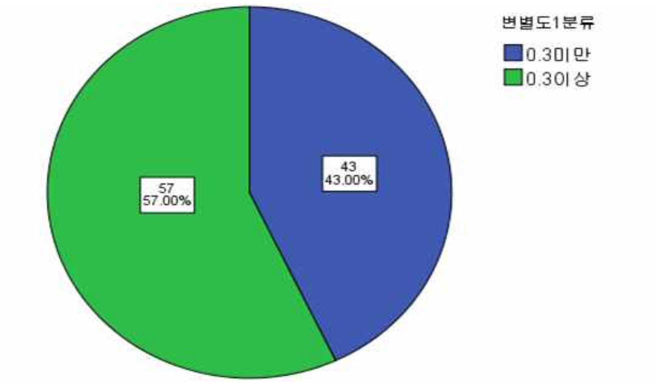

총점	변별도1	표준편차
100	.34	.18

변별도1	문항수	비율(%)
0.3미만	43	43.0
0.3이상	57	57.0
전체	100	100.0

(3) 전체 변별도2 분포도 및 비율분석

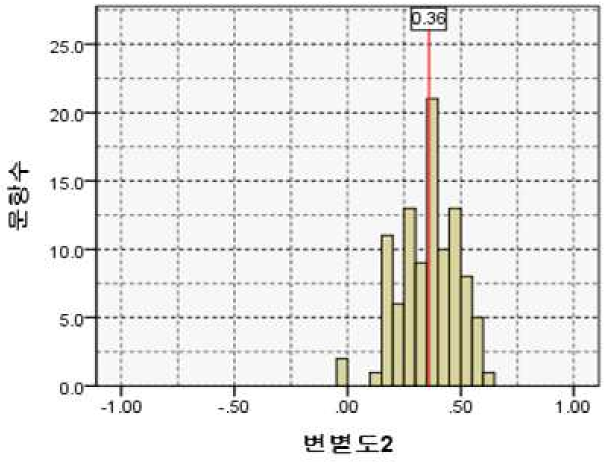

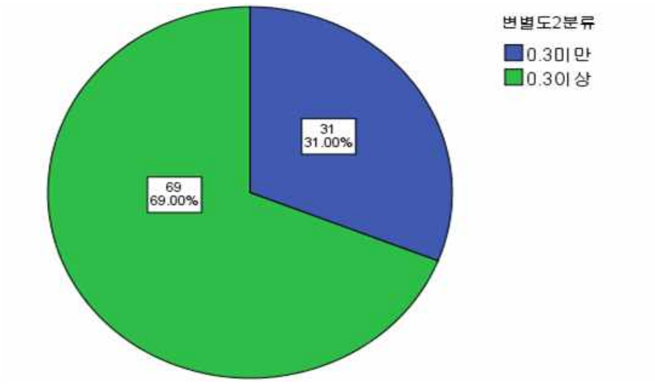

총점	변별도2	표준편차	변별도2	문항수	비율(%)
100	.36	.13	0.3미만	31	31.0
			0.3이상	69	69.0
			전체	100	100.0

해석

- 난이도 지수가 80에서 100 사이인 문항이 전체 100문항 중 44문항으로 가장 많았으며, 차례로 60이상 80미만인 문항이 37문항, 60미만인 문항이 19문항인 것으로 나타남
- 변별도1 지수를 기준으로 분류하였을 때, 0.3미만인 문항이 43문항으로 0.3이상인 문항이 57문항인 것에 비해 더 적게 나타남
- 변별도2 지수를 기준으로 분류하였을 때, 0.3미만인 문항이 31문항으로 0.3이상인 문항이 69문항인 것에 비해 더 적게 나타남

2) 과목별 난이도와 변별도

가) 전회 대비 과목별 난이도와 변별도

(1) 전회 대비 기초간호학개요 난이도와 변별도

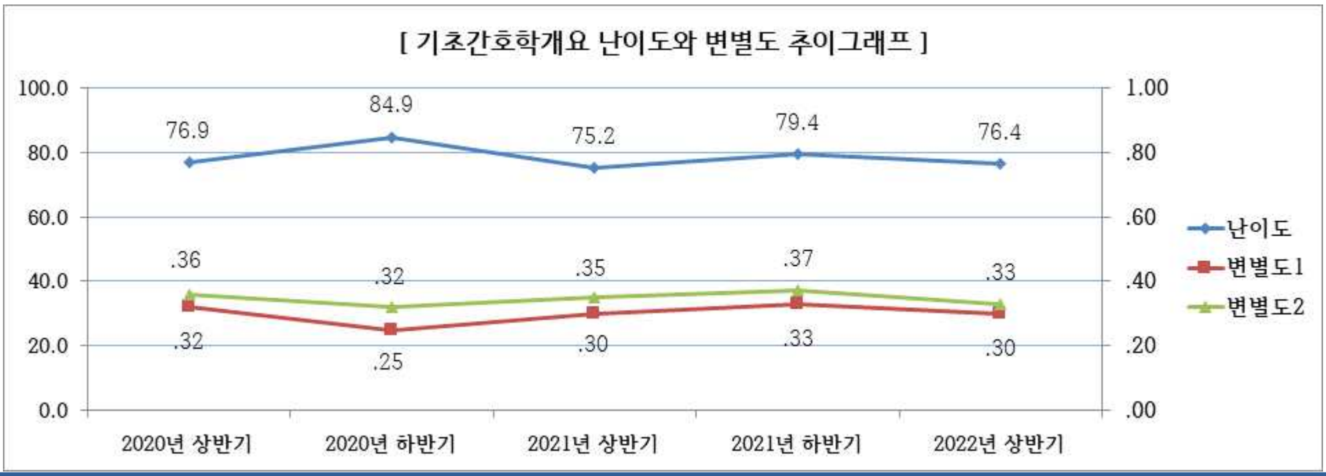

회차	난이도		변별도1		변별도2	
	평균	표준편차	평균	표준편차	평균	표준편차
2020년 상반기	76.9	19.3	0.32	0.16	0.36	0.12
2020년 하반기	84.9	14.4	0.25	0.17	0.32	0.11
2021년 상반기	75.2	20.6	0.30	0.15	0.35	0.10
2021년 하반기	79.4	16.1	0.33	0.20	0.37	0.11
**2022년 상반기**	**76.4**	**22.0**	**0.30**	**0.19**	**0.33**	**0.12**

(2) 전회 대비 보건간호학개요 난이도와 변별도

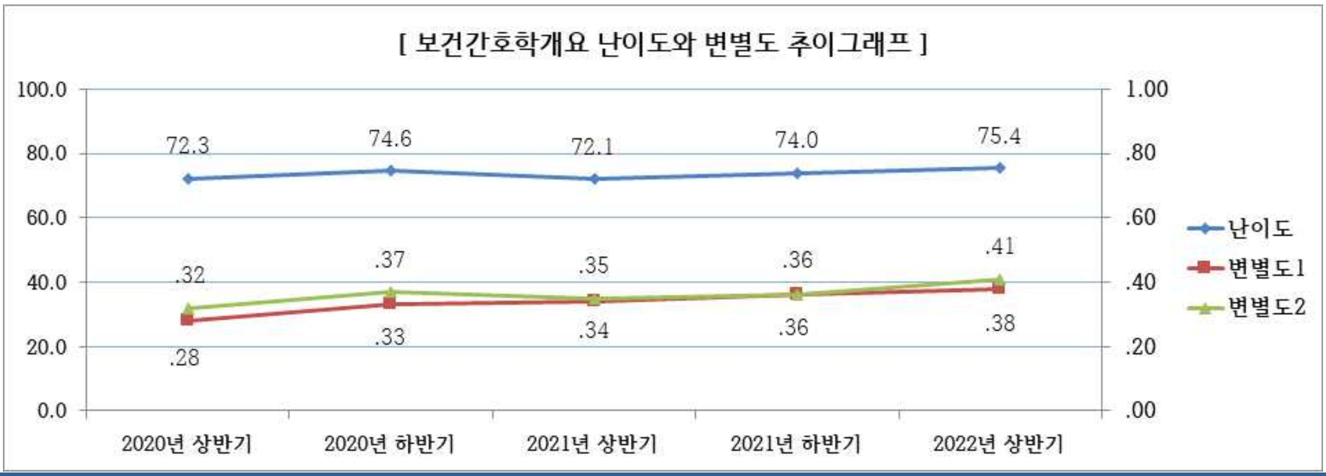

회차	난이도		변별도1		변별도2	
	평균	표준편차	평균	표준편차	평균	표준편차
2020년 상반기	72.3	20.0	0.28	0.16	0.32	0.14
2020년 하반기	74.6	20.0	0.33	0.17	0.37	0.13
2021년 상반기	72.1	20.6	0.34	0.15	0.35	0.11
2021년 하반기	74.0	11.7	0.36	0.18	0.36	0.15
**2022년 상반기**	**75.4**	**18.1**	**0.38**	**0.16**	**0.41**	**0.10**

(3) 전회 대비 공중보건학개론 난이도와 변별도

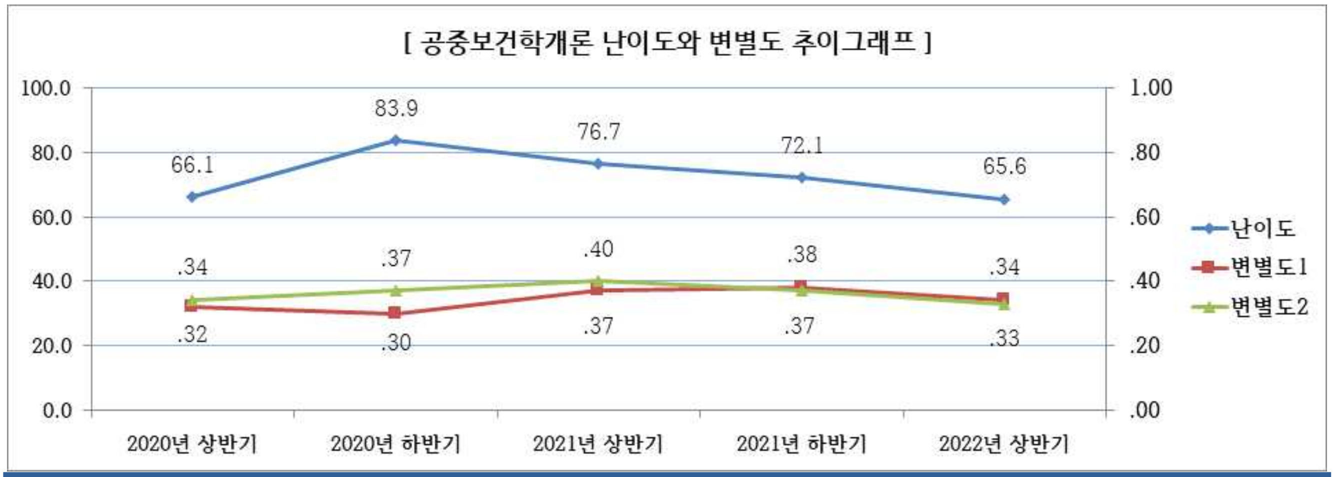

회차	난이도		변별도1		변별도2	
	평균	표준편차	평균	표준편차	평균	표준편차
2020년 상반기	66.1	24.0	0.32	0.19	0.34	0.16
2020년 하반기	83.9	11.3	0.30	0.18	0.37	0.13
2021년 상반기	76.7	13.4	0.37	0.18	0.40	0.14
2021년 하반기	72.1	12.4	0.38	0.17	0.37	0.14
**2022년 상반기**	**65.6**	**22.1**	**0.34**	**0.18**	**0.33**	**0.14**

(4) 전회 대비 실기 난이도와 변별도

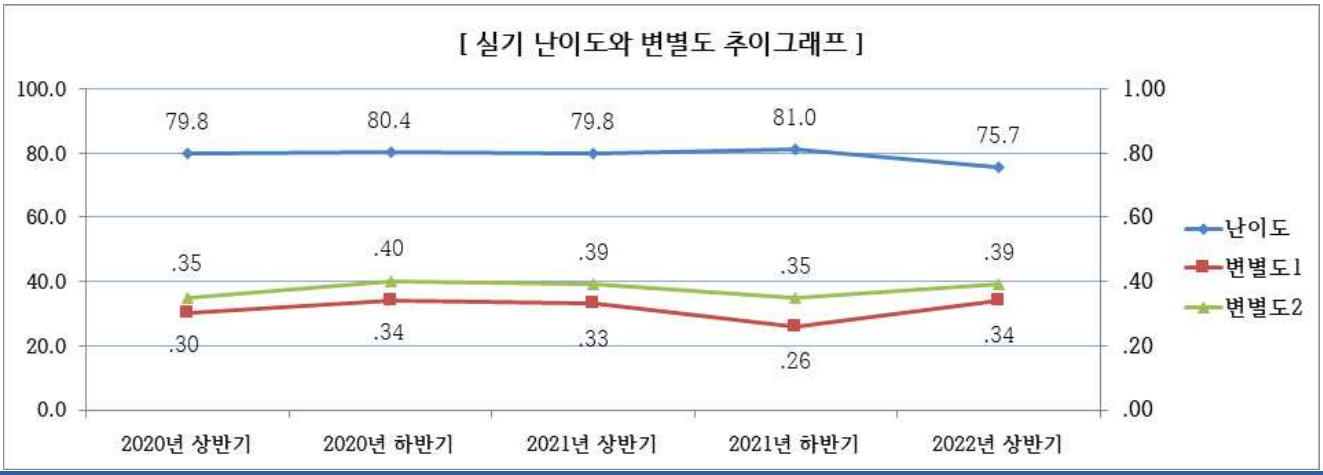

회차	난이도		변별도1		변별도2	
	평균	표준편차	평균	표준편차	평균	표준편차
2020년 상반기	79.8	15.1	0.30	0.17	0.35	0.12
2020년 하반기	80.4	13.5	0.34	0.17	0.40	0.13
2021년 상반기	79.8	12.6	0.33	0.16	0.39	0.14
2021년 하반기	81.0	19.1	0.26	0.16	0.35	0.13
**2022년 상반기**	**75.7**	**17.6**	**0.34**	**0.18**	**0.39**	**0.13**

해석

- 전회 대비 기초간호학개요, 공중보건학개론, 실기 과목의 난이도 지수는 각각 3.0, 6.5, 5.3 감소하였으며, 보건간호학개론 과목의 난이도 지수는 4.1 증가함
- 기초간호학개요 과목의 변별도 1 지수 및 변별도 2 지수는 각각 .03, .04 감소함
- 보건간호학개요 과목의 변별도 1 지수 및 변별도 2 지수는 각각 .02, .05 증가함
- 공중보건학개론 과목의 변별도 1 지수 및 변별도 2 지수는 각각 .04 증가함
- 실기 과목의 변별도 1 지수 및 변별도 2 지수는 각각 .08, .04 증가함

나) 과목별 난이도와 변별도 분포도 및 비율분석

(1) 기초간호학개요 난이도와 변별도 분포도 및 비율분석

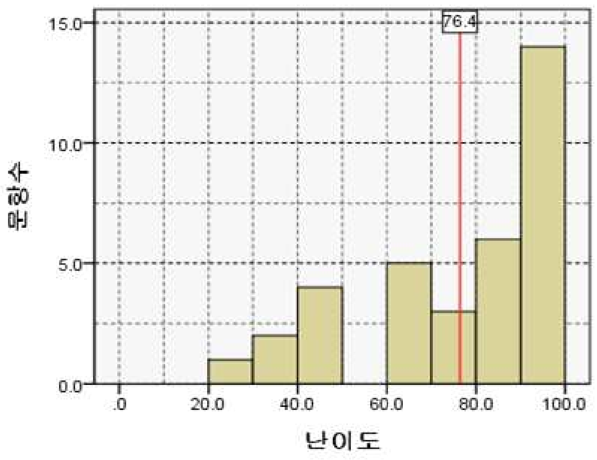

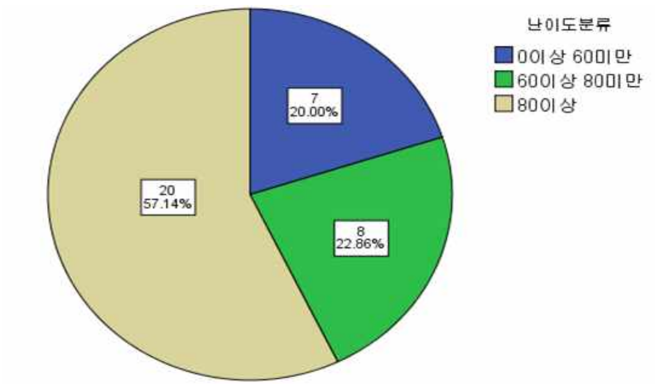

총점	난이도	표준편차
35	76.4	22.0

난이도	문항수	비율(%)
0~60미만	7	20.0
60~80미만	8	22.9
80~100	20	57.1
전체	35	100.0

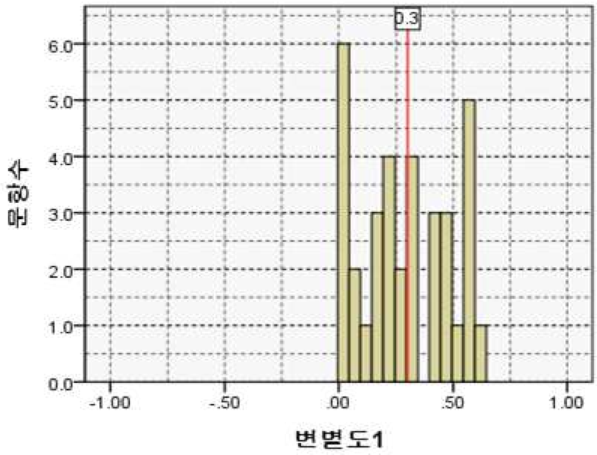

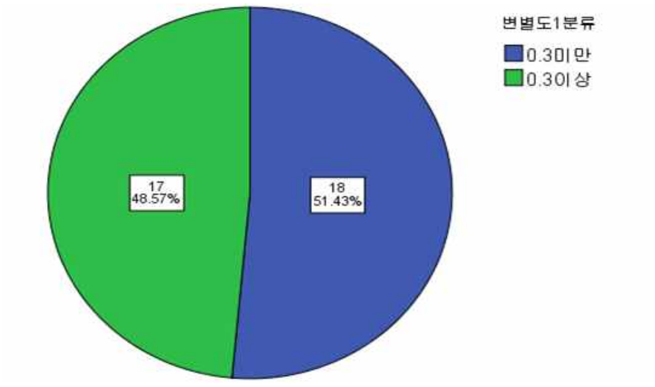

총점	변별도1	표준편차
35	.30	.19

변별도1	문항수	비율(%)
0.3미만	18	51.4
0.3이상	17	48.6
전체	35	100.0

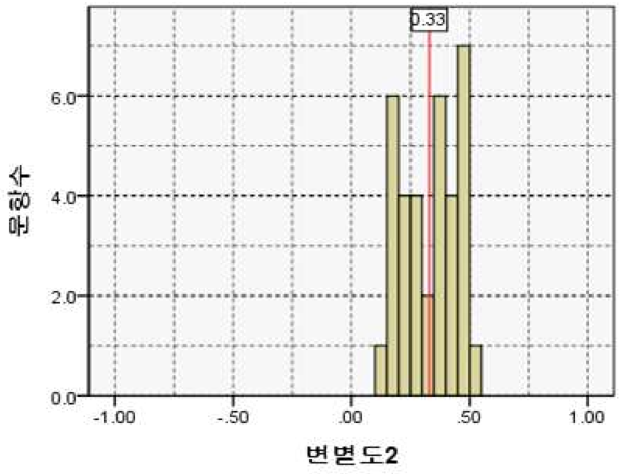

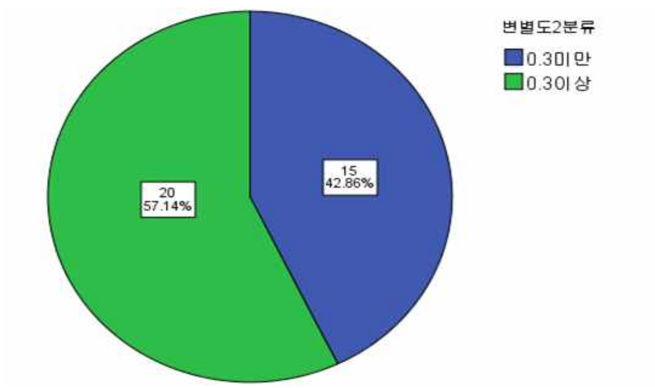

총점	변별도2	표준편차
35	.33	.12

변별도2	문항수	비율(%)
0.3미만	15	42.9
0.3이상	20	57.1
전체	35	100.0

해석

- 기초간호학개요 과목에서 난이도 지수가 80에서 100 사이인 문항이 전체 35문항 중 20문항으로 가장 많았으며, 다음으로 60이상 80미만인 문항이 8문항, 60미만인 문항이 7문항 인 것으로 나타남
- 변별도1 지수를 기준으로 분류하였을 때, 0.3미만인 문항이 18문항으로 0.3이상인 문항이 17문항인 것에 비해 더 많게 나타남
- 변별도2 지수를 기준으로 분류하였을 때, 0.3미만인 문항이 15문항으로 0.3이상인 문항이 20문항인 것에 비해 더 적게 나타남

(2) 보건간호학개요 난이도와 변별도 분포도 및 비율분석

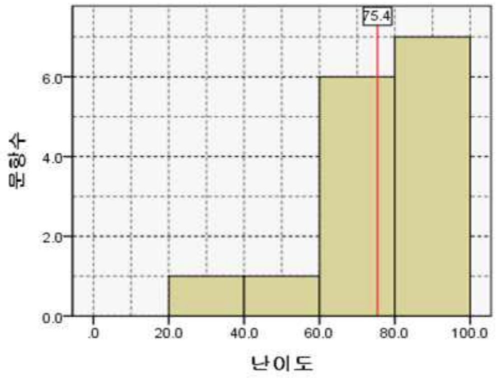

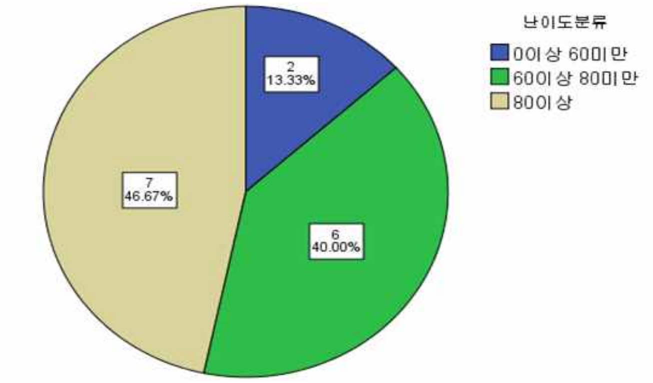

총점	난이도	표준편차
15	75.4	18.1

난이도	문항수	비율(%)
0~60미만	2	13.3
60~80미만	6	40.0
80~100	7	46.7
전체	15	100.0

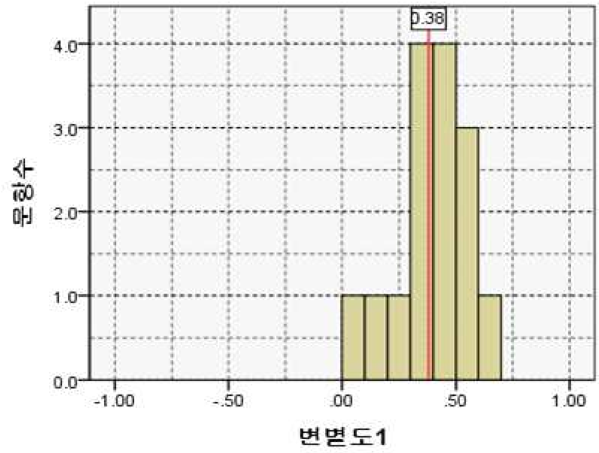

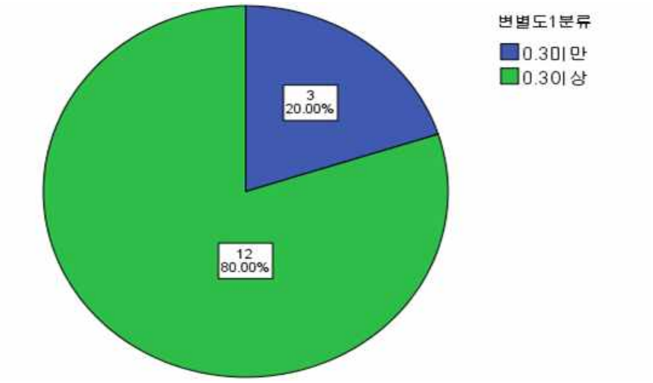

총점	변별도1	표준편차
15	.38	.16

변별도1	문항수	비율(%)
0.3미만	3	20.0
0.3이상	12	80.0
전체	15	100.0

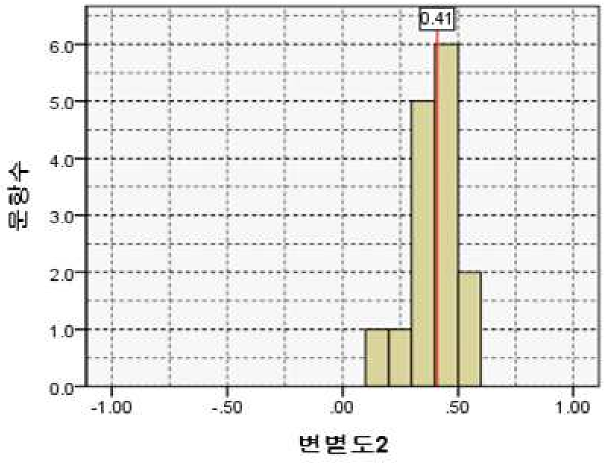

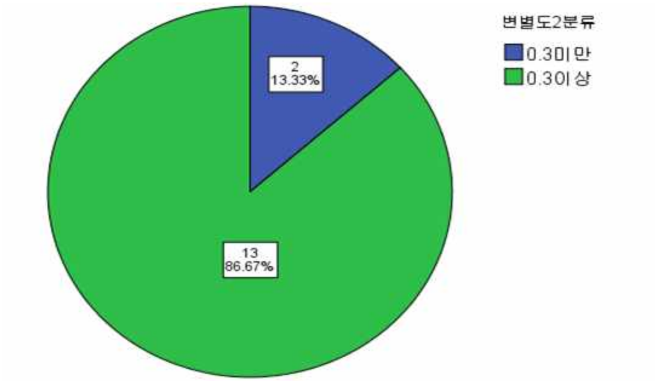

총점	변별도2	표준편차
15	.41	.10

변별도2	문항수	비율(%)
0.3미만	2	13.3
0.3이상	13	86.7
전체	15	100.0

해석

- 보건간호학개요 과목에서 난이도 지수가 80 에서 100 사이인 문항이 전체 15 문항 중 7 문항이었으며, 다음으로 60 이상 80 미만인 문항이 6 문항, 60 미만인 문항이 2 문항 인 것으로 나타남
- 변별도 1 지수를 기준으로 분류하였을 때, 0.3 미만인 문항이 3 문항으로 0.3 이상인 문항이 12 문항인 것에 비해 더 적게 나타남
- 변별도 2 지수를 기준으로 분류하였을 때, 0.3 미만인 문항이 2 문항으로 0.3 이상인 문항이 13 문항인 것에 비해 더 적게 나타남

(3) 공중보건학개론 난이도와 변별도 분포도 및 비율분석

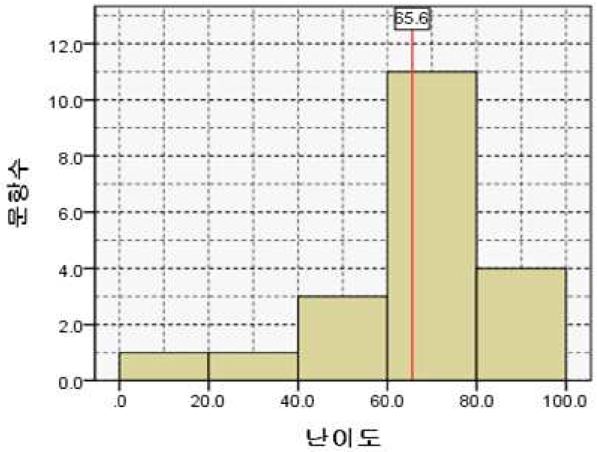

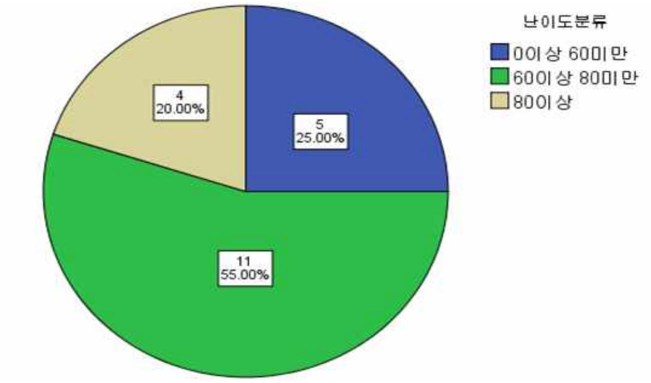

총점	난이도	표준편차
20	65.6	22.1

난이도	문항수	비율(%)
0~60미만	5	25.0
60~80미만	11	55.0
80~100	4	20.0
전체	20	100.0

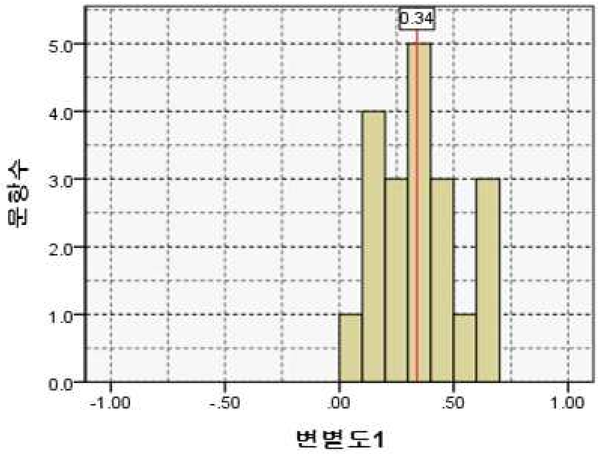

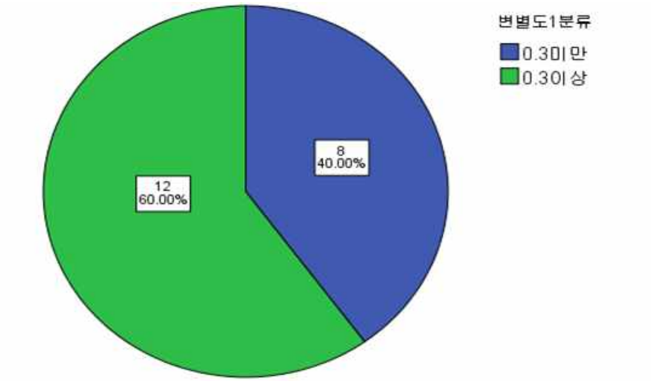

총점	변별도1	표준편차
20	.34	.18

변별도1	문항수	비율(%)
0.3미만	8	40.0
0.3이상	12	60.0
전체	20	100.0

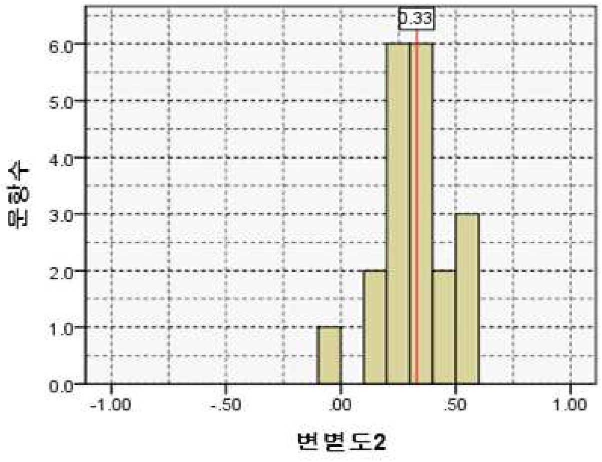

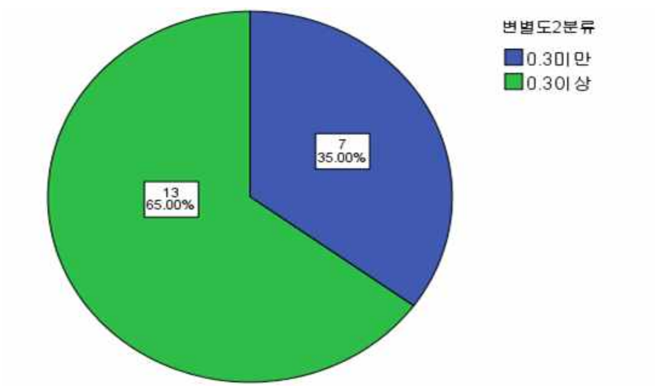

총점	변별도2	표준편차
20	.33	.14

변별도2	문항수	비율(%)
0.3미만	7	35.0
0.3이상	13	65.0
전체	20	100.0

해석

- 공중보건학개론 과목에서 난이도 지수가 80에서 100사이인 문항이 전체 20문항 중 4문항으로 나타났으며, 다음으로 60에서 80 사이인 문항이 11문항, 60미만인 문항이 5문항으로 나타남
- 변별도1 지수를 기준으로 분류하였을 때, 0.3미만인 문항이 8문항으로 0.3이상인 문항이 12 문항인 것에 비해 더 적게 나타남
- 변별도2 지수를 기준으로 분류하였을 때, 0.3미만인 문항이 7문항으로 0.3이상인 문항이 13문항인 것에 비해 더 적게 나타남

(4) 실기 난이도와 변별도 분포도 및 비율분석

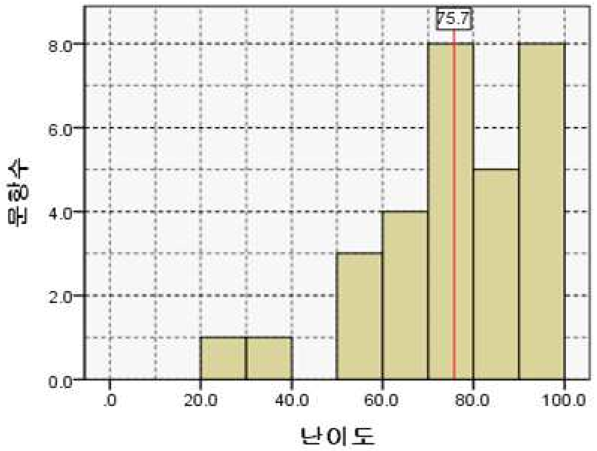

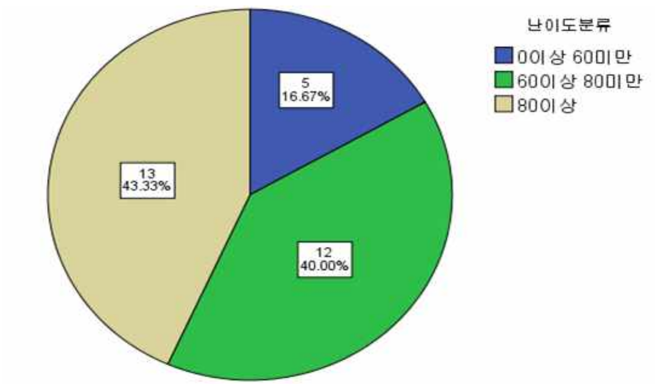

총점	난이도	표준편차	난이도	문항수	비율(%)
30	75.7	17.6	0~60미만	5	16.7
			60~80미만	12	40.0
			80~100	13	43.3
			전체	30	100.0

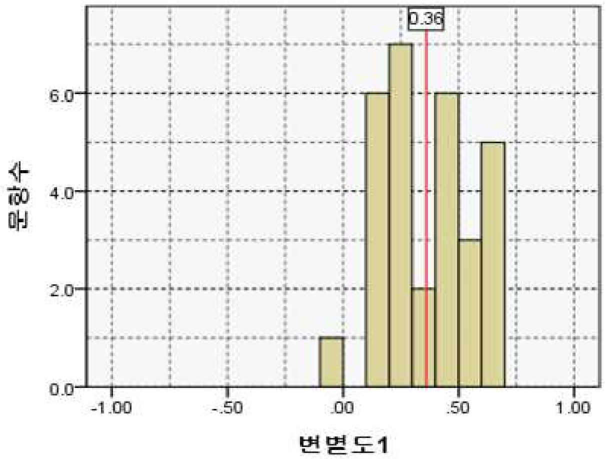

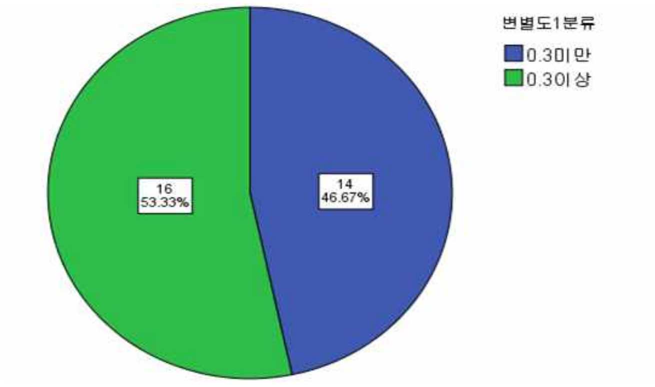

총점	변별도1	표준편차	변별도1	문항수	비율(%)
30	.36	.19	0.3미만	14	46.7
			0.3이상	16	53.3
			전체	30	100.0

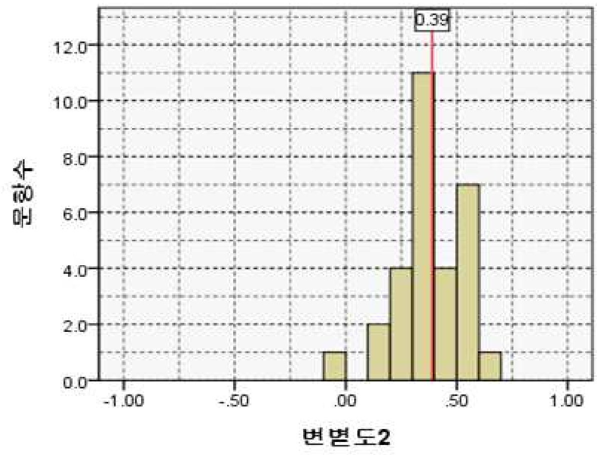

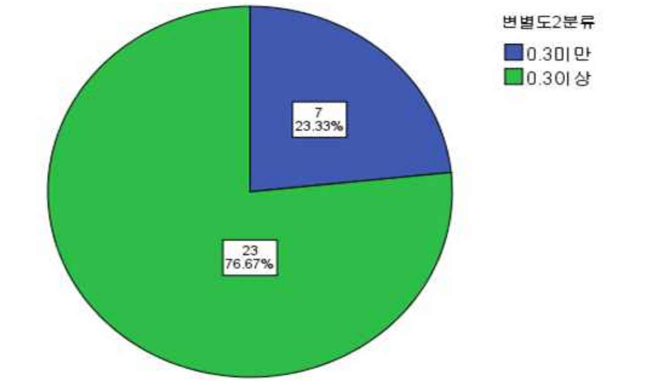

총점	변별도2	표준편차
30	.39	.13

변별도2	문항수	비율(%)
0.3미만	7	23.3
0.3이상	23	76.7
전체	30	100.0

해석

- 실기 과목에서 난이도 지수가 60 이상 80 미만인 문항이 전체 30 문항 중 13 문항으로 가장 많았으며, 다음으로 80 에서 100 사이인 문항 12 문항, 60 미만인 문항이 5 문항 인 것으로 나타남
- 변별도 1 지수를 기준으로 분류하였을 때, 0.3 미만인 문항이 14 문항으로 0.3 이상인 문항이 16 문항인 것에 비해 적게 나타남
- 변별도 2 지수를 기준으로 분류하였을 때, 0.3 미만인 문항이 7 문항으로 0.3 이상인 문항이 23 문항인 것에 비해 더 적게 나타남

3) 지식수준별 난이도와 변별도

가) 전회 대비 지식수준별 난이도와 변별도

(1) 전회 대비 암기형 난이도와 변별도

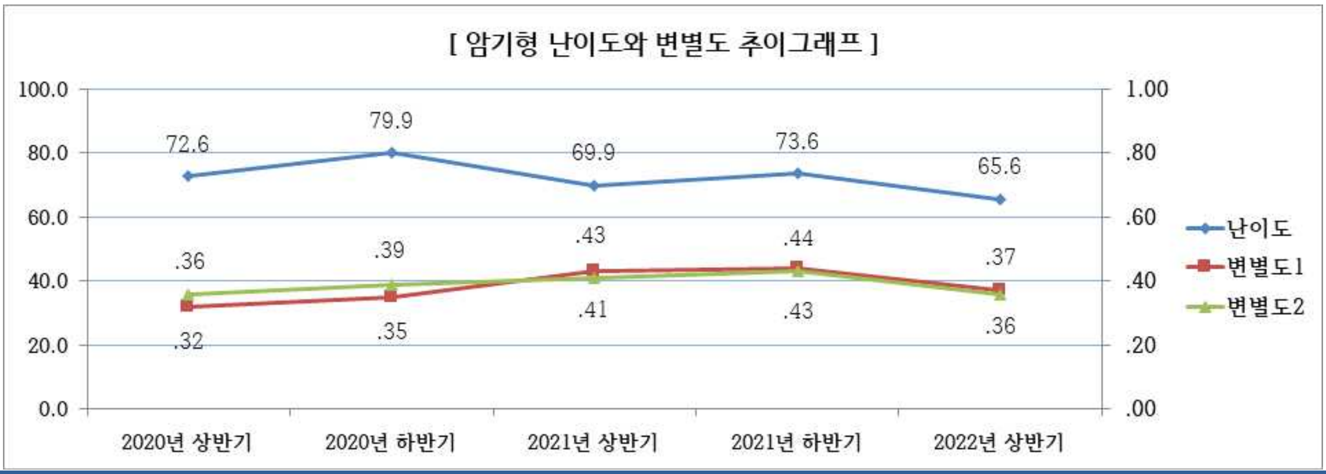

회차	난이도		변별도1		변별도2	
	평균	표준편차	평균	표준편차	평균	표준편차
2020년 하반기	79.9	12.7	0.35	0.17	0.39	0.12
2021년 상반기	69.9	17.2	0.43	0.15	0.41	0.11
2021년 하반기	73.6	14.5	0.44	0.17	0.43	0.10
2021년 하반기	73.6	14.5	0.44	0.17	0.43	0.10
**2022년 상반기**	**65.6**	**21.8**	**0.37**	**0.19**	**0.36**	**0.15**

(2) 전회 대비 해석형 난이도와 변별도

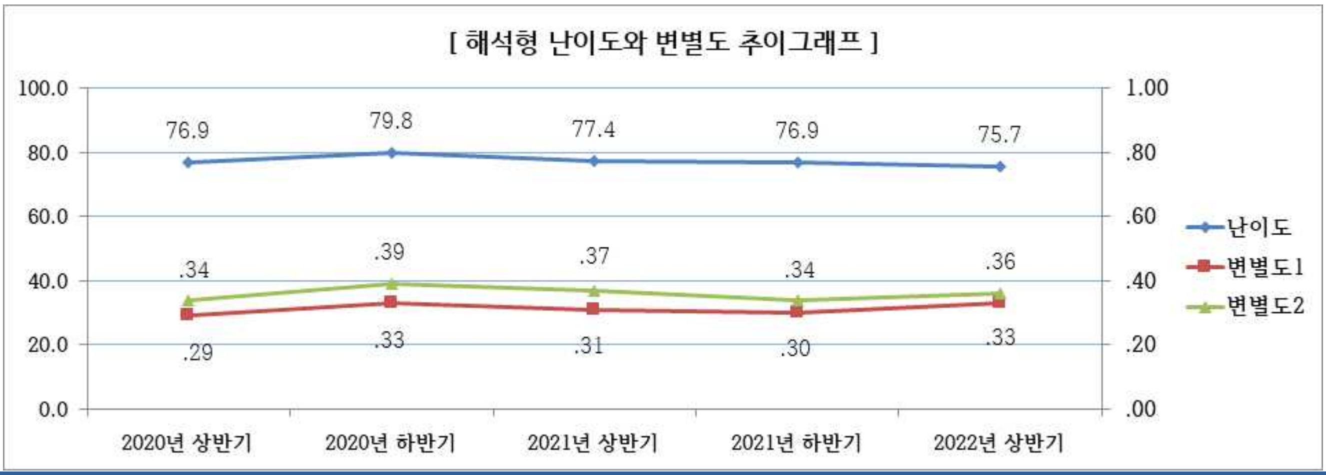

회차	난이도		변별도1		변별도2	
	평균	표준편차	평균	표준편차	평균	표준편차
2020년 상반기	76.9	19.8	0.29	0.17	0.34	0.13
2020년 하반기	79.8	16.3	0.33	0.17	0.39	0.12
2021년 상반기	77.4	16.8	0.31	0.14	0.37	0.12
2021년 하반기	76.9	17.7	0.30	0.18	0.34	0.13
**2022년 상반기**	**75.7**	**19.3**	**0.33**	**0.18**	**0.36**	**0.15**

(3) 전회 대비 해결형 난이도와 변별도

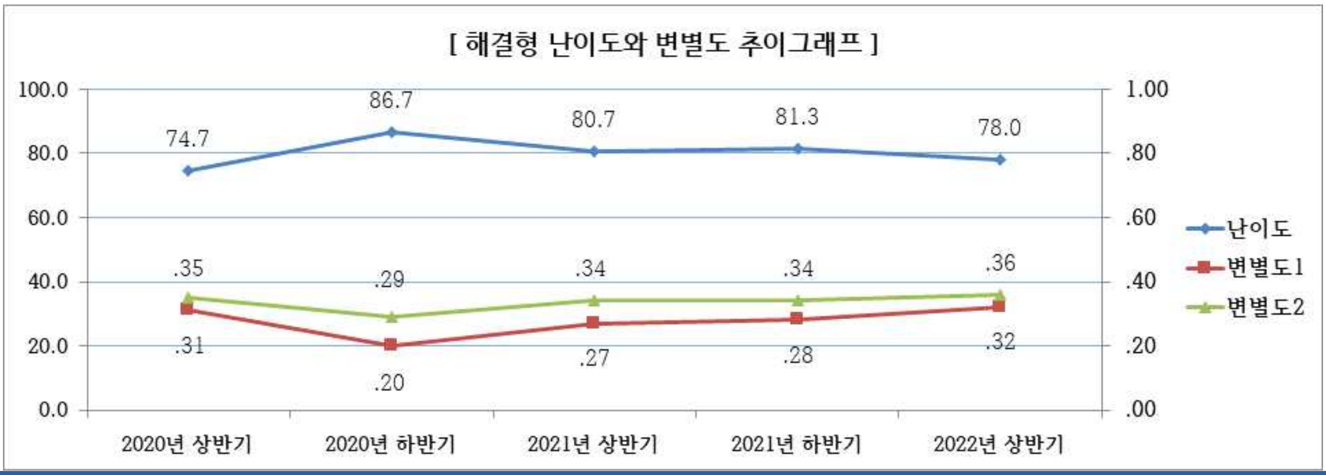

회차	난이도		변별도1		변별도2	
	평균	표준편차	평균	표준편차	평균	표준편차
2020년 상반기	**74.7**	17.9	0.31	0.15	0.35	0.12
2020년 하반기	86.7	13.9	0.20	0.15	0.29	0.11
2021년 상반기	80.7	16.2	0.27	0.16	0.34	0.13
2021년 하반기	81.3	14.3	0.28	0.16	0.34	0.12
2022년 상반기	78.0	19.3	0.32	0.19	0.36	0.13

해석

- 전회 대비 암기형, 해결형, 해석형 문항의 난이도 지수는 각각 8.0, 1.2, 3.3 감소함
- 암기형 문항의 변별도 1 지수와 변별도 2 지수는 각각 .07 감소함
- 해석형 문항의 변별도 1 지수와 변별도 2 지수는 각각 .03, .02 증가함
- 해결형 문항의 변별도 1 지수와 변별도 2 지수는 각각 .04, .02 증가함

나) 지식수준별 난이도와 변별도 분포도 및 비율분석

(1) 암기형 난이도와 변별도 분포도 및 비율분석

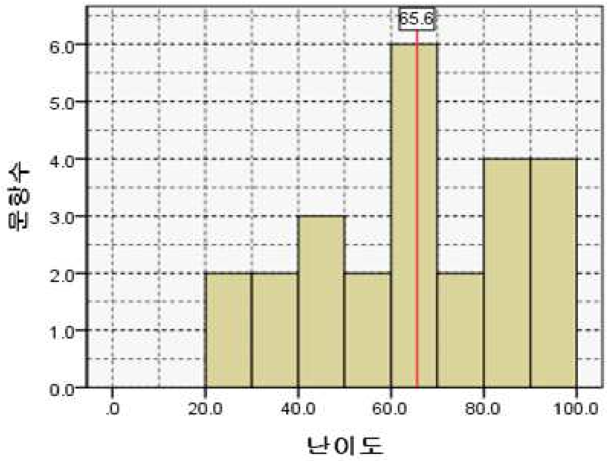

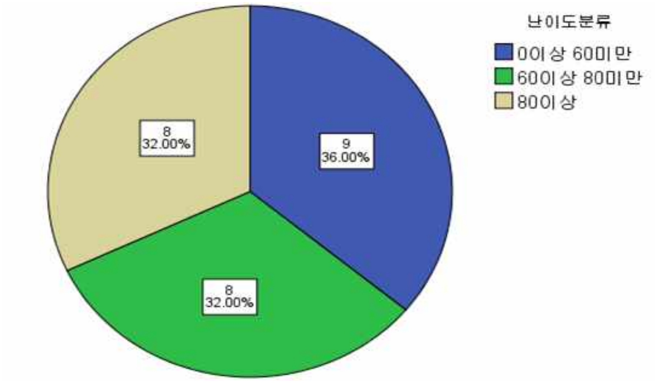

총점	난이도	표준편차
25	65.6	21.8

난이도	문항수	비율(%)
0~60미만	9	36.0
60~80미만	8	32.0
80~100	8	32.0
전체	25	100.0

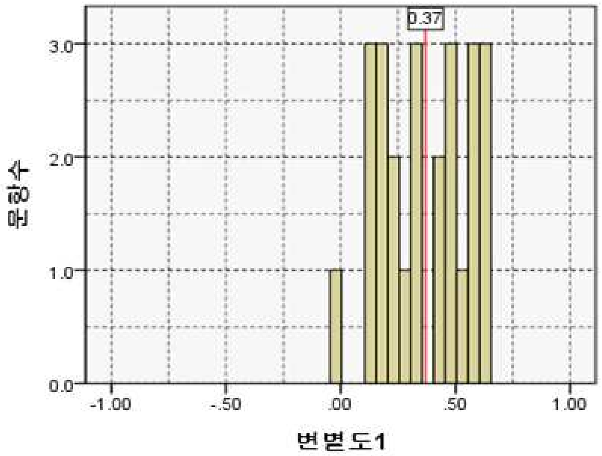

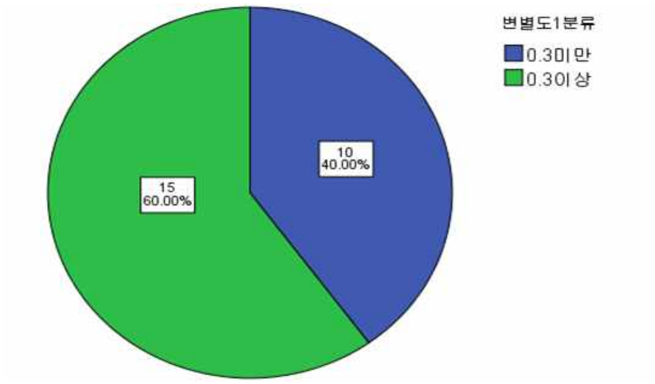

총점	변별도1	표준편차
25	.37	.19

변별도1	문항수	비율(%)
0.3미만	10	40.0
0.3이상	15	60.0
전체	25	100.0

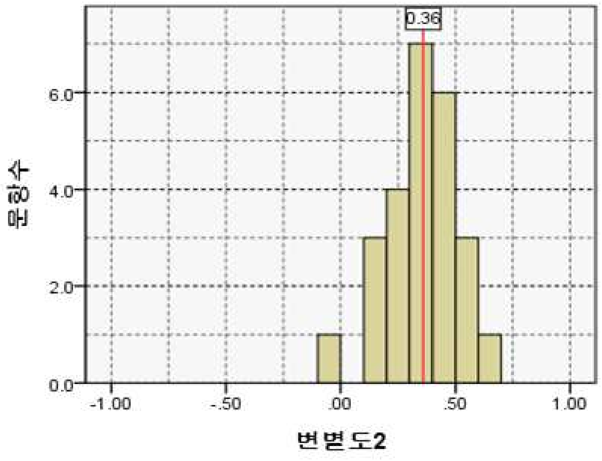

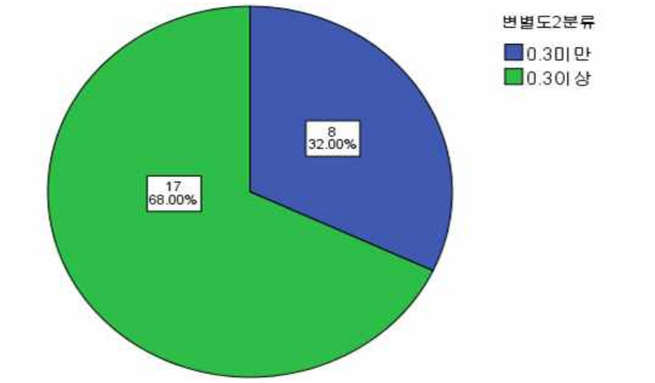

총점	변별도2	표준편차
25	.36	.15

변별도2	문항수	비율(%)
0.3미만	8	32.0
0.3이상	17	68.0
전체	25	100.0

해석

- 암기형 문항에서 난이도 지수가 80에서 100 사이인 문항이 전체 25문항 중 8문항 이였으며, 다음으로 60 이상 80 미만인 문항이 8문항, 60 미만인 문항이 9문항인 것으로 나타남
- 변별도1 지수를 기준으로 분류하였을 때, 0.3 미만인 문항이 10문항으로 0.3 이상인 문항이 15문항인 것에 비해 더 적게 나타남
- 변별도2 지수를 기준으로 분류하였을 때, 0.3 미만인 문항이 8문항으로 0.3 이상인 문항이 17문항인 것에 비해 더 적게 나타남

(2) 해석형 난이도와 변별도 분포도 및 비율분석

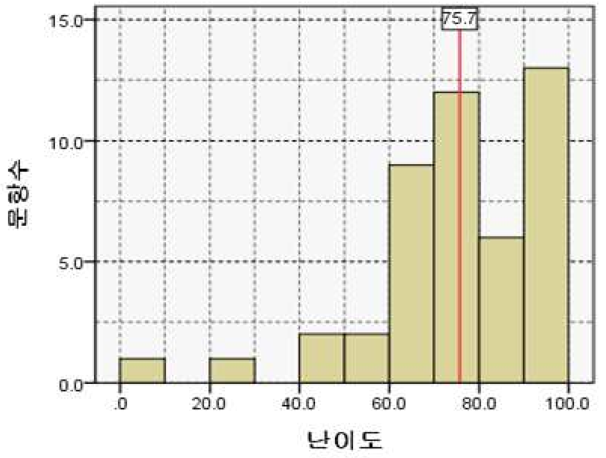

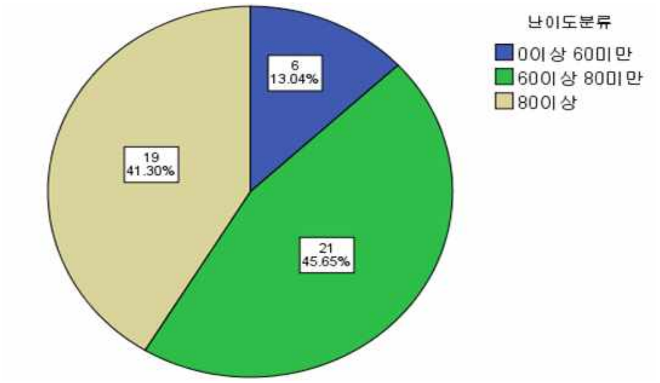

총점	난이도	표준편차
46	75.7	19.3

난이도	문항수	비율(%)
0~60미만	6	13.0
60~80미만	21	45.7
80~100	19	41.3
전체	46	100.0

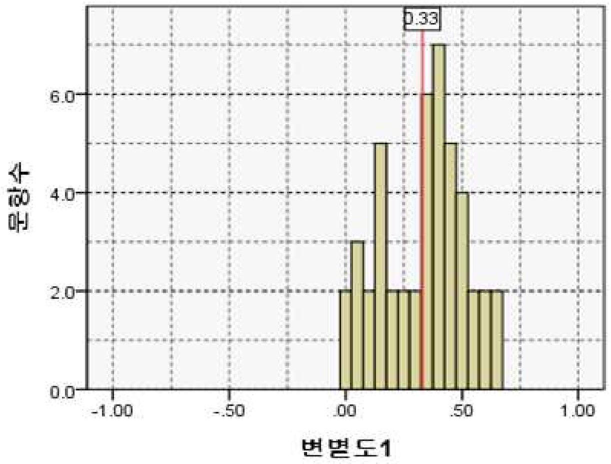

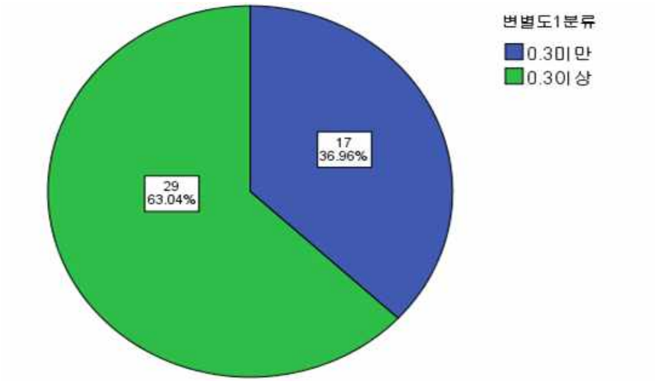

총점	변별도1	표준편차
46	.33	.18

변별도1	문항수	비율(%)
0.3미만	17	37.0
0.3이상	29	63.0
전체	46	100.0

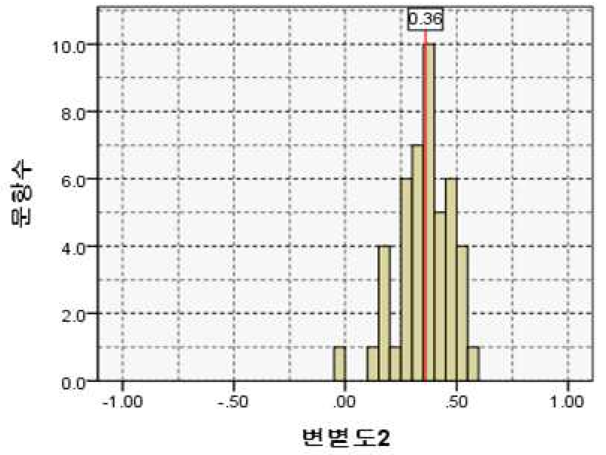

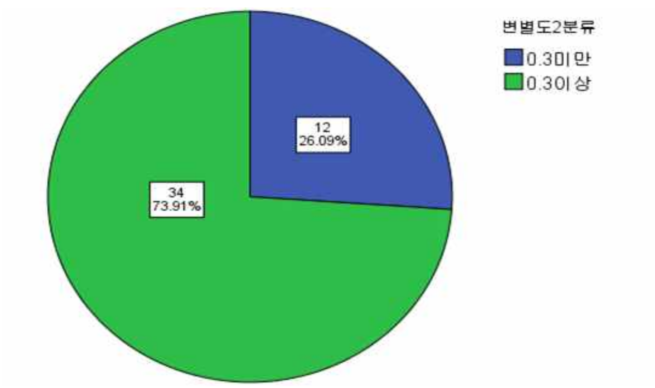

총점	변별도2	표준편차
46	.36	.12

변별도2	문항수	비율(%)
0.3미만	12	26.1
0.3이상	34	73.9
전체	46	100.0

해석

- 해석형 문항에서 난이도 지수가 80에서 100 사이인 문항이 전체 46문항 중 19문항이었으며, 다음으로 60이상 80미만인 문항이 21문항, 60미만인 문항이 6문항인 것으로 나타남
- 변별도1 지수를 기준으로 분류하였을 때, 0.3미만인 문항이 17문항으로 0.3 이상인 문항이 29문항인 것에 비해 더 적게 나타남
- 변별도2 지수를 기준으로 분류하였을 때, 0.3미만인 문항이 12문항으로 0.3 이상인 문항이 34문항인 것에 비해 더 적게 나타남

(3) 해결형 난이도와 변별도 분포도 및 비율분석

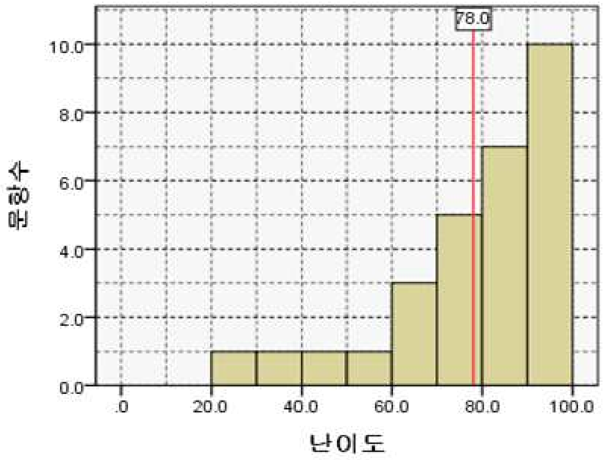

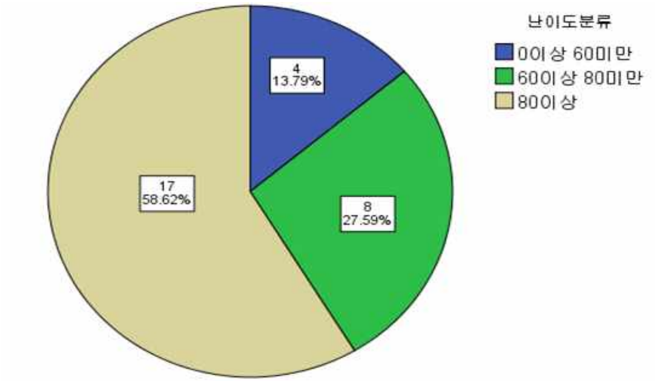

총점	난이도	표준편차
29	78.0	19.3

난이도	문항수	비율(%)
0~60미만	4	13.8
60~80미만	8	27.6
80~100	17	58.6
전체	29	100.0

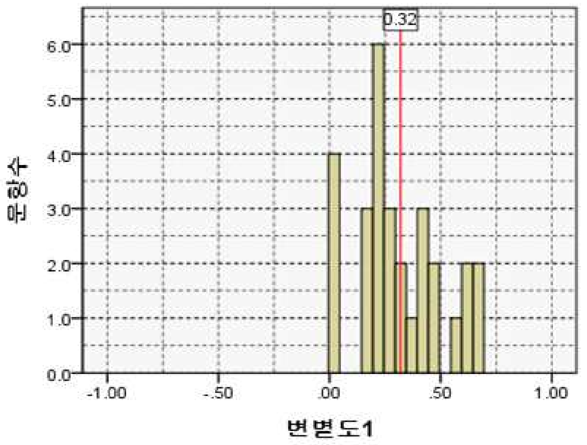

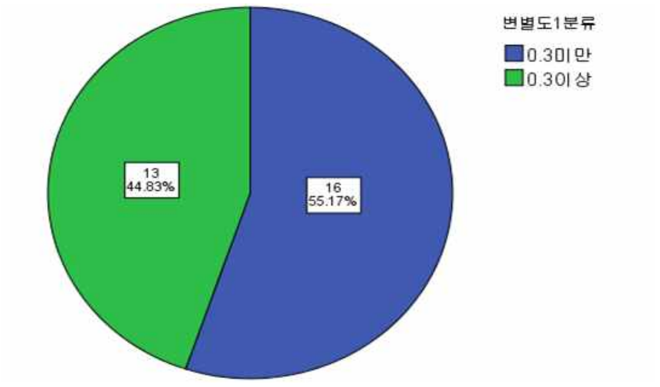

총점	변별도1	표준편차
29	.32	.19

변별도1	문항수	비율(%)
0.3미만	16	55.2
0.3이상	13	44.8
전체	29	100.0

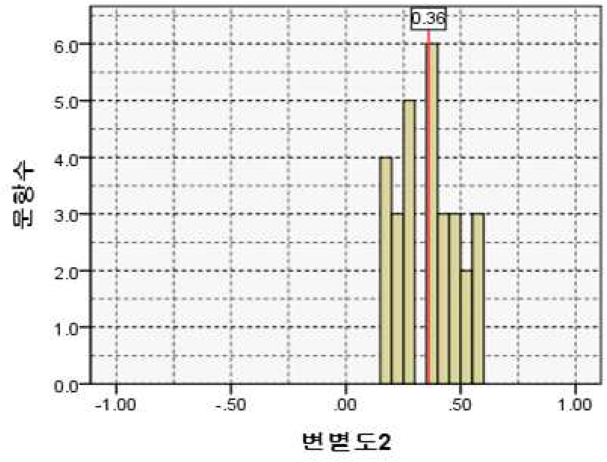

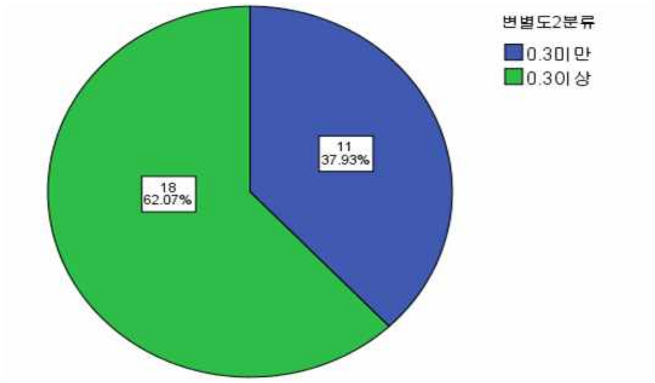

총점	변별도2	표준편차
29	.36	.13

변별도2	문항수	비율(%)
0.3미만	11	37.9
0.3이상	18	62.1
전체	29	100.0

해석

- 해결형 문항에서 난이도 지수가 80에서 100 사이인 문항이 전체 29문항 중 17문항으로 가장 많았으며, 다음으로 60이상 80미만인 문항이 8문항, 60미만인 문항이 4문항인 것으로 나타남
- 변별도1 지수를 기준으로 분류하였을 때, 0.3미만인 문항이 16문항으로 0.3이상인 문항이 13문항인 것에 비해 더 많게 나타남
- 변별도2 지수를 기준으로 분류하였을 때, 0.3미만인 문항이 11문항으로 0.3이상인 문항이 18문항인 것에 비해 더 적게 나타남

4) 자료유형별 난이도와 변별도

가) 전회 대비 자료유형별 난이도와 변별도

(1) 전회 대비 텍스트형 난이도와 변별도

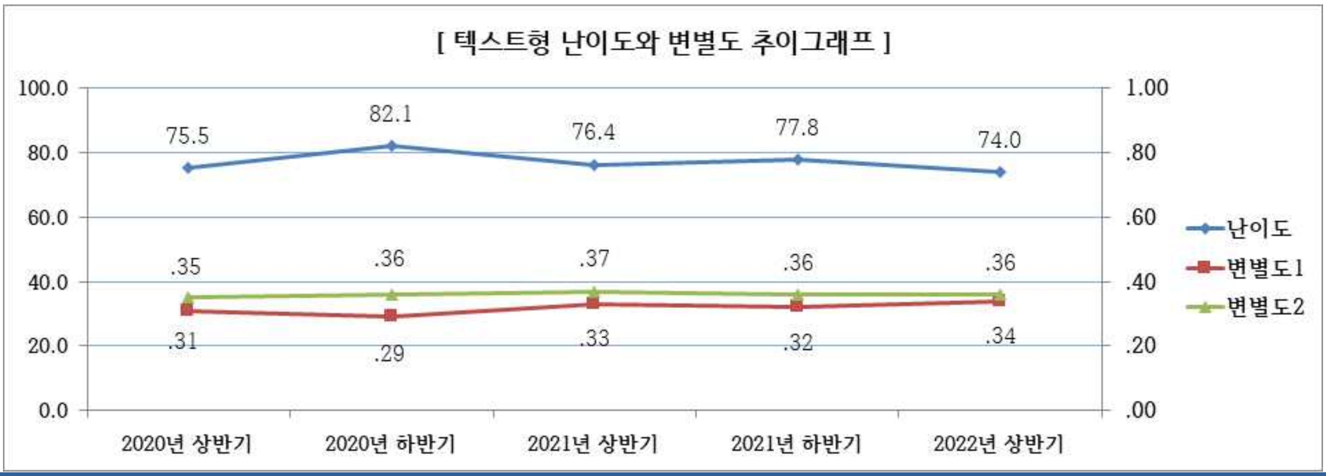

회차	난이도		변별도1		변별도2	
	평균	표준편차	평균	표준편차	평균	표준편차
2020년 상반기	75.5	19.4	0.31	0.17	0.35	0.13
2020년 하반기	82.1	14.6	0.29	0.17	0.36	0.13
2021년 상반기	76.4	17.1	0.33	0.16	0.37	0.12
2021년 하반기	77.8	16.0	0.32	0.18	0.36	0.13
**2022년 상반기**	**74.0**	**20.1**	**0.34**	**0.19**	**0.36**	**0.13**

(2) 전회 대비 자료제시형 난이도와 변별도

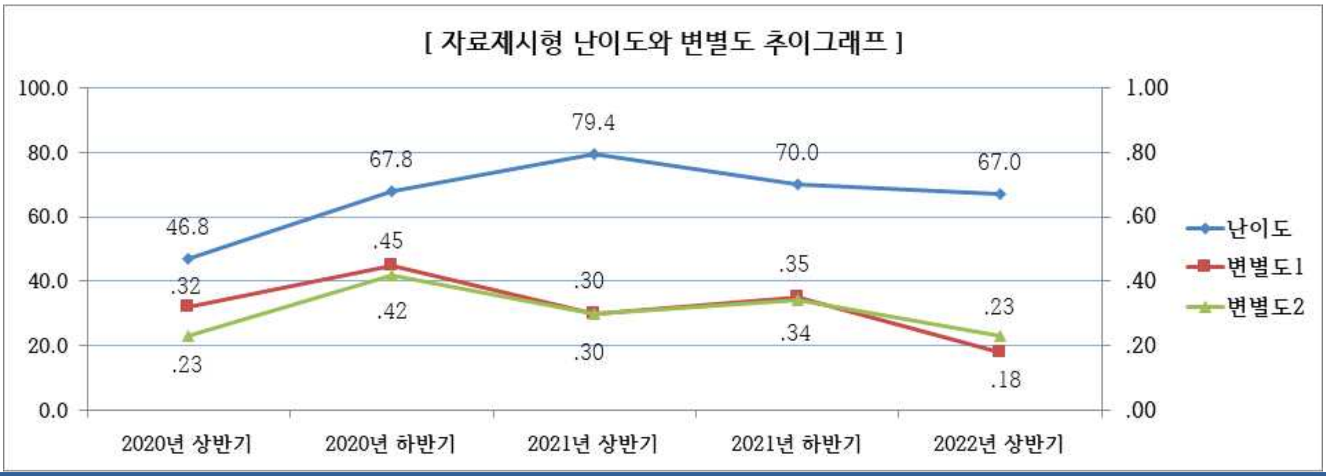

회차	난이도		변별도1		변별도2	
	평균	표준편차	평균	표준편차	평균	표준편차
2020년 상반기	46.8	8.1	0.32	0.28	0.23	0.00
2020년 하반기	67.8	22.9	0.45	0.14	0.42	0.01
2021년 상반기	79.4	22.6	0.30	0.30	0.30	0.17
2021년 하반기	70.0	21.7	0.35	0.10	0.34	0.00
**2022년 상반기**	**67.0**	**40.6**	**0.18**	**0.08**	**0.23**	**0.07**

해석

- 전회 대비 텍스트형, 자료제시형 문항의 난이도 지수는 각각 3.8, 3.0 감소함
- 텍스트형의 변별도 1 지수는 .02 증가하였으며, 변별도 2 지수는 변화없음
- 자료제시형의 변별도 1 지수와 변별도 2 지수는 각각 .17, .11 감소함

나) 자료유형별 난이도와 변별도 분포도 및 비율분석

(1) 텍스트형 난이도와 변별도 분포도 및 비율분석

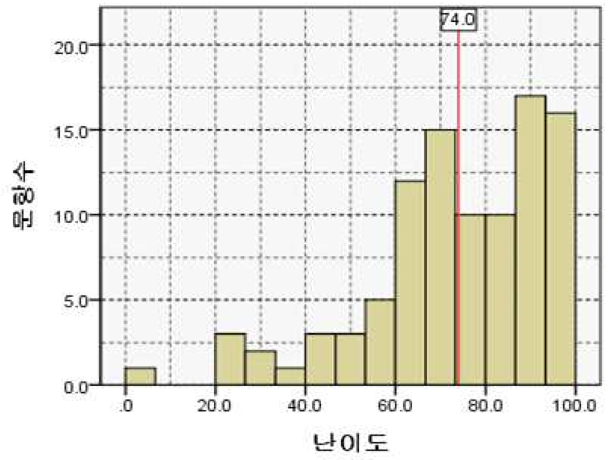

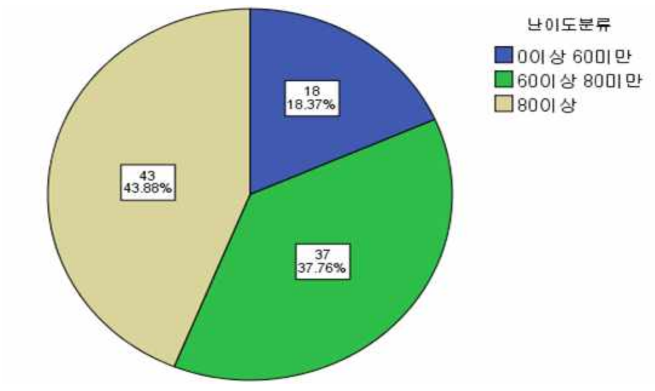

총점	난이도	표준편차
98	74.0	20.1

난이도	문항수	비율(%)
0~60미만	18	18.4
60~80미만	37	37.8
80~100	43	43.9
전체	98	100.0

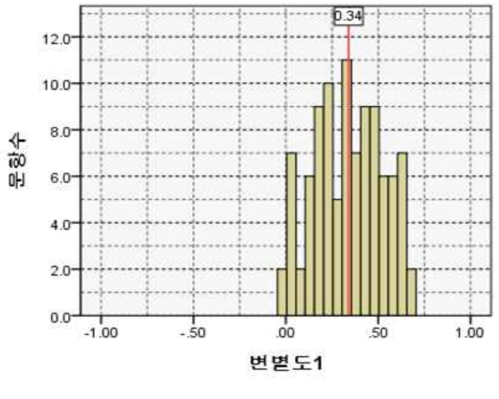

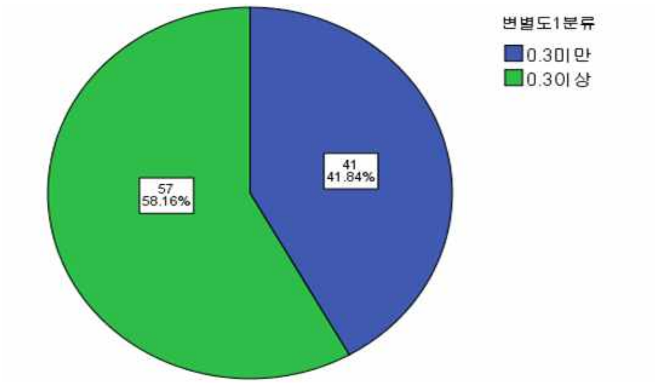

총점	변별도1	표준편차
98	.34	.19

변별도1	문항수	비율(%)
0.3미만	41	41.8
0.3이상	57	58.2
전체	98	100.0

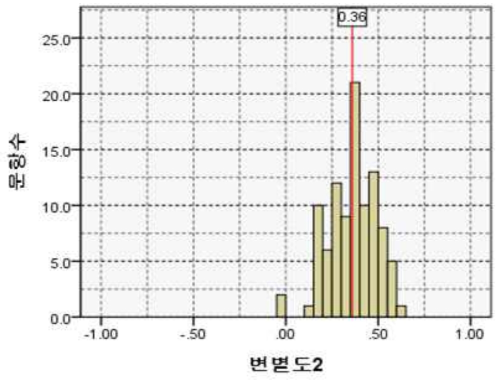

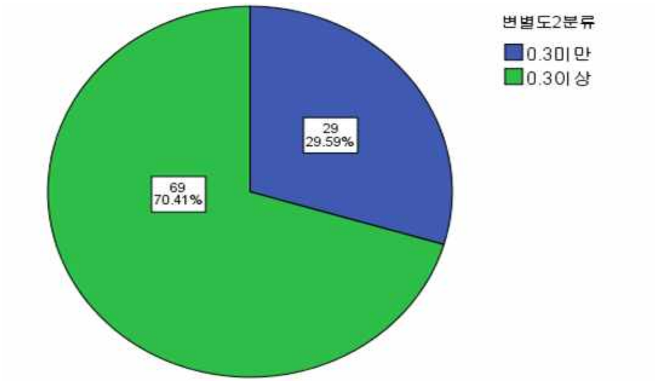

총점	변별도2	표준편차
98	.36	.13

변별도2	문항수	비율(%)
0.3미만	29	29.6
0.3이상	69	70.4
전체	98	100.0

해석

- 텍스트형 문항에서 난이도 지수가 80 에서 100 사이인 문항이 전체 98 문항 중 43 문항으로 가장 많았으며, 다음으로 60 이상 80 미만인 문항이 37 문항, 60 미만인 문항이 13 문항인 것으로 나타남
- 변별도 1 지수를 기준으로 분류하였을 때, 0.3 미만인 문항이 41 문항으로 0.3 이상인 문항이 57 문항으로 더 적게 나타남
- 변별도 2 지수를 기준으로 분류하였을 때, 0.3 미만인 문항이 29 문항으로 0.3 이상인 문항이 69 문항에 비해 더 적게 나타남

(2) 자료제시형 난이도와 변별도 분포도 및 비율분석

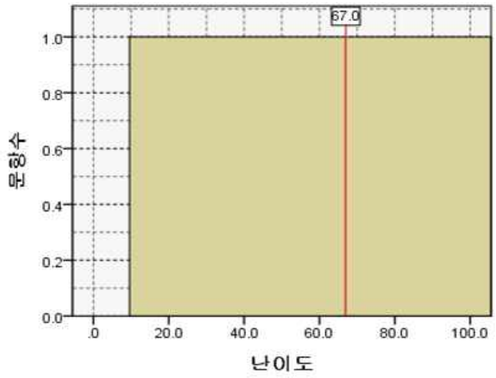

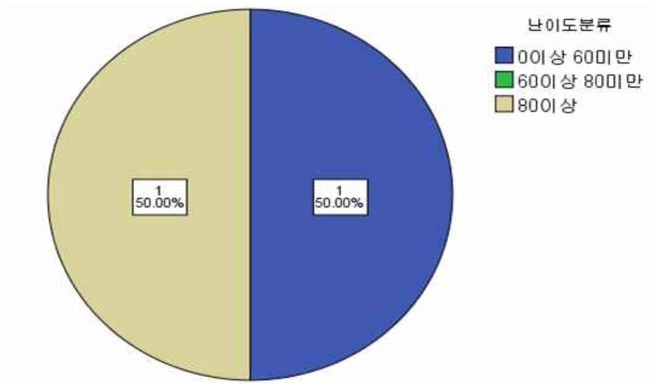

총점	난이도	표준편차
2	67.0	40.6

난이도	문항수	비율(%)
0~60미만	1	50.0
60~80미만	-	-
80~100	1	50.0
전체	2	100.0

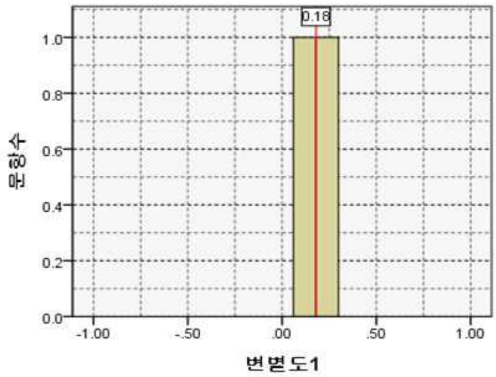

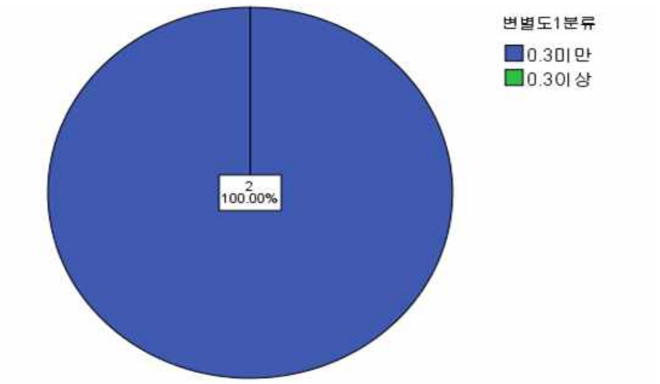

총점	변별도1	표준편차
2	.18	.08

변별도1	문항수	비율(%)
0.3미만	2	100.0
0.3이상	-	-
전체	2	100.0

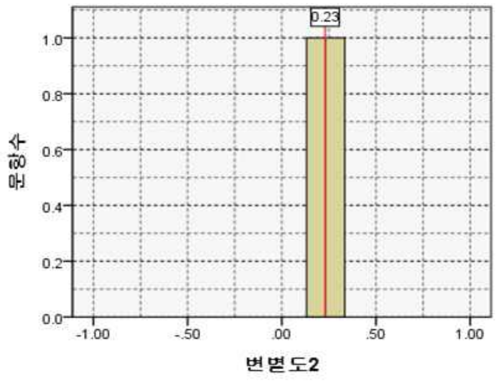

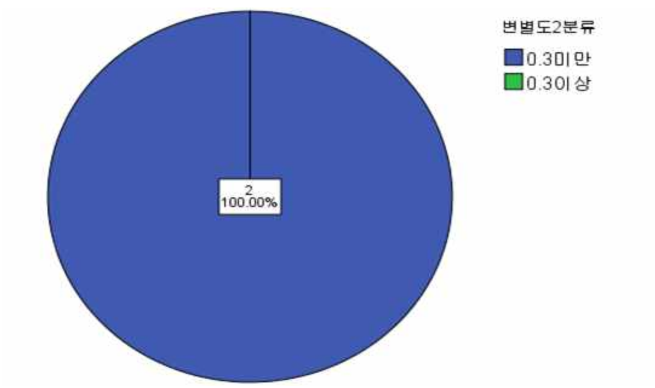

총점	변별도2	표준편차
2	.23	.07

변별도2	문항수	비율(%)
0.3미만	2	100.0
0.3이상	-	-
전체	2	100.0

해석

- 자료제시형 문항의 난이도 지수가 80에서 100사이인 문항이 1문항, 60이하인 문항이 1문항인 것으로 나타남
- 변별도1 지수를 기준으로 분류하였을 때, 0.3 미만인 문항이 2문항으로 나타남
- 변별도2 지수를 기준으로 분류하였을 때, 0.3미만인 문항이 2문항으로 나타남

3. 난이도와 변별도 간 산포도

1) 전체 난이도와 변별도 간 산포도

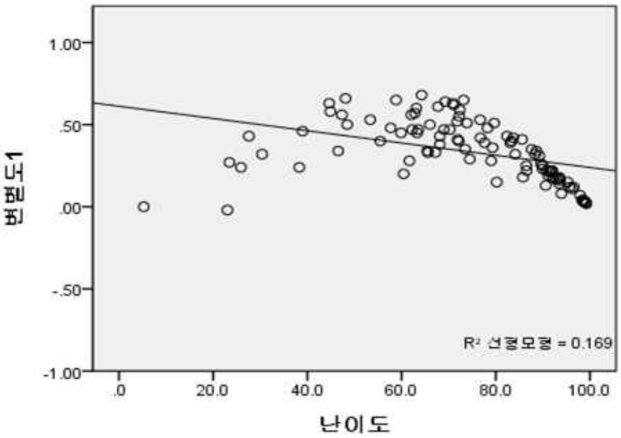

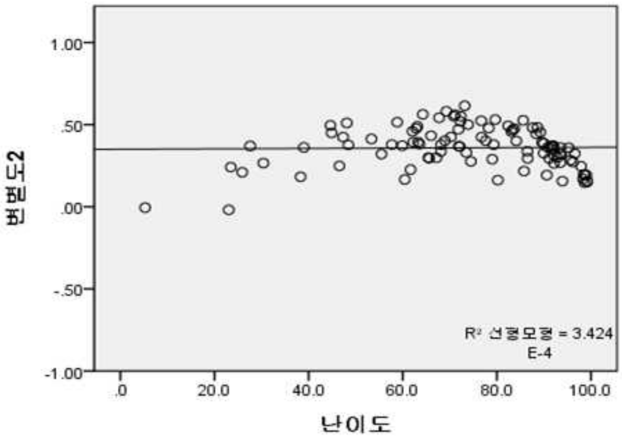

해석

- 전체 문항을 대상으로 난이도와 변별도1 지수 간 상관은 -.411*로 문항 난이도가 쉬울수록 변별력이 낮아지는 것으로 나타남
- 난이도와 변별도2 지수 간 상관은 .019로 문항 난이도와 변별력 간 관련성이 없는 것으로 나타남

2) 과목별 난이도와 변별도 간 산포도

가) 기초간호학개요 난이도와 변별도 간 산포도

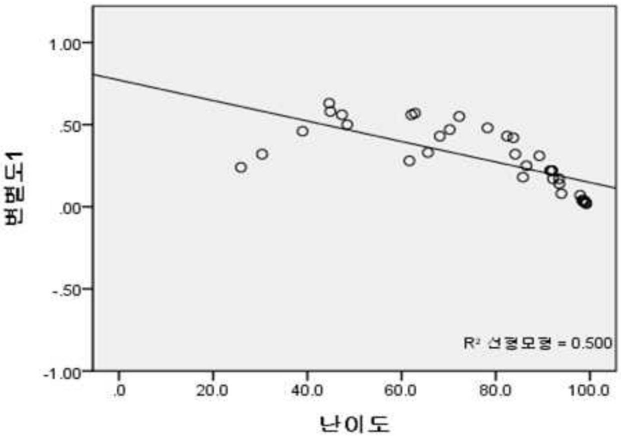

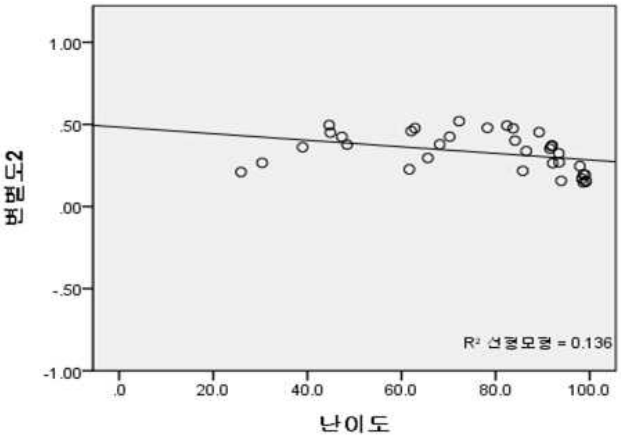

해석
- 기초간호학개론 과목 문항을 대상으로 난이도와 변별도1 지수 간 상관은 -.707*로 문항 난이도가 쉬울수록 변별력이 낮아지는 것으로 나타남<br>- 난이도와 변별도2 지수 간 상관은 -.369*로 문항 난이도가 쉬울수록 변별력이 낮아지는 것으로 나타남

나) 보건간호학개요 난이도와 변별도 간 산포도

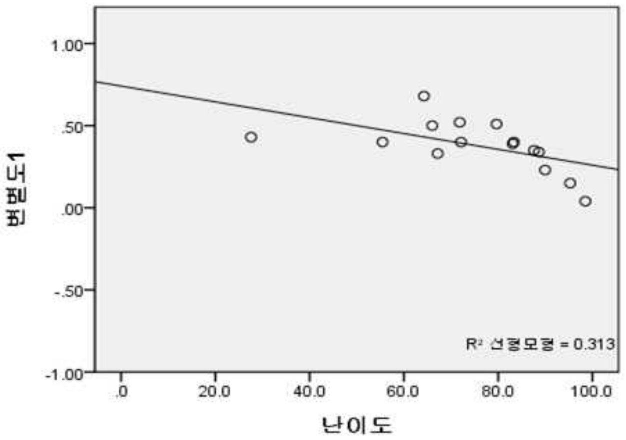

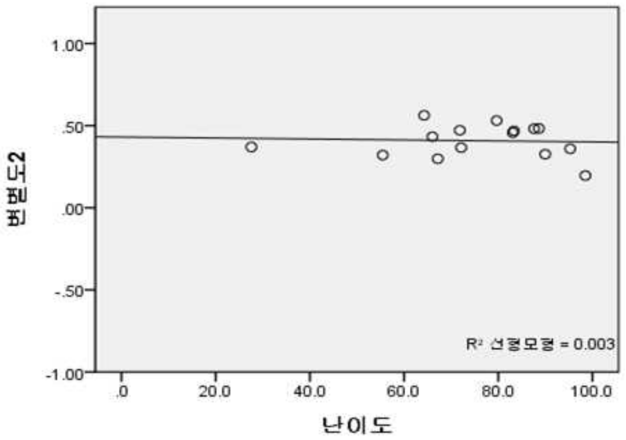

해석
- 보건간호학개요 과목 문항을 대상으로 난이도와 변별도1 지수 간 상관은 -.559*로 문항 난이도가 쉬울수록 변별력이 낮아지는 것으로 나타남<br>- 난이도와 변별도2 지수 간 상관은 -.054로 문항 난이도와 변별력 간 관련성이 없는 것으로 나타남

다) 공중보건학개론 난이도와 변별도 간 산포도

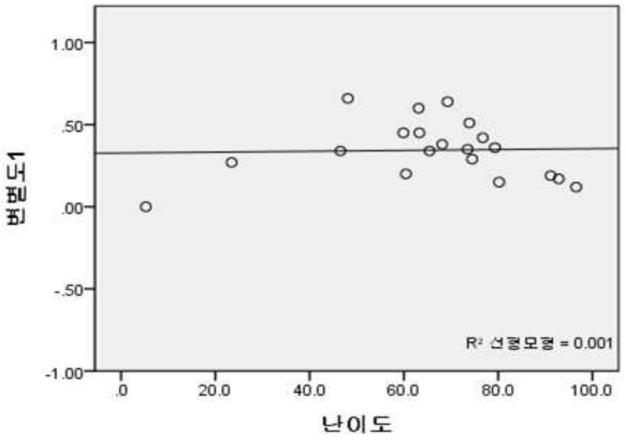

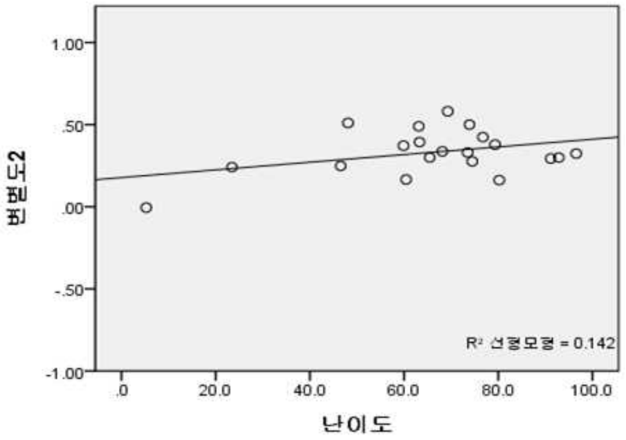

해석

- 공중보건학개론 과목 문항을 대상으로 난이도와 변별도1 지수 간 상관은 .099로 문항 난이도와 변별력 간 관련성이 없는 것으로 나타남
- 난이도와 변별도2 지수 간 상관은 .377로 문항 난이도와 변별력 간 관련성이 낮은 것으로 나타남

라) 실기 난이도와 변별도 간 산포도

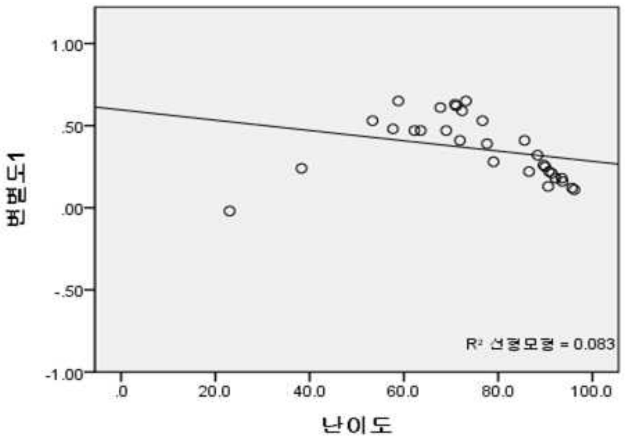

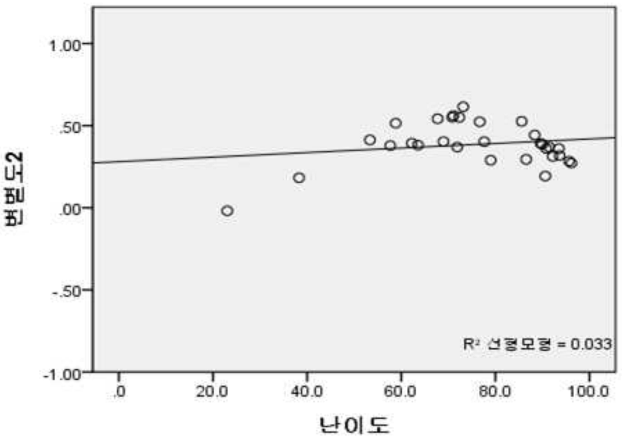

해석

- 실기 과목 문항을 대상으로 난이도와 변별도1 지수 간 상관은 -.288로 문항 난이도와 변별력 간 관련성이 낮은 것으로 나타남
- 난이도와 변별도2 지수 간 상관은 .183으로 문항 난이도와 변별력 간 관련성이 낮은 것으로 나타남

4. 신뢰도 분석

과목명	문항수	2020년 상반기	2020년 하반기	2021년 상반기	2021년 하반기	2022년 상반기
**전체**	**100**	**.925**	**.931**	**.933**	**.931**	**.932**
기초간호학개요	35	.833	.786	.808	.843	**.805**
보건간호학개요	15	.595	.661	.656	.664	**.740**
공중보건학개론	20	.692	.751	.775	.737	**.697**
실기	30	.798	.838	.830	.792	**.832**

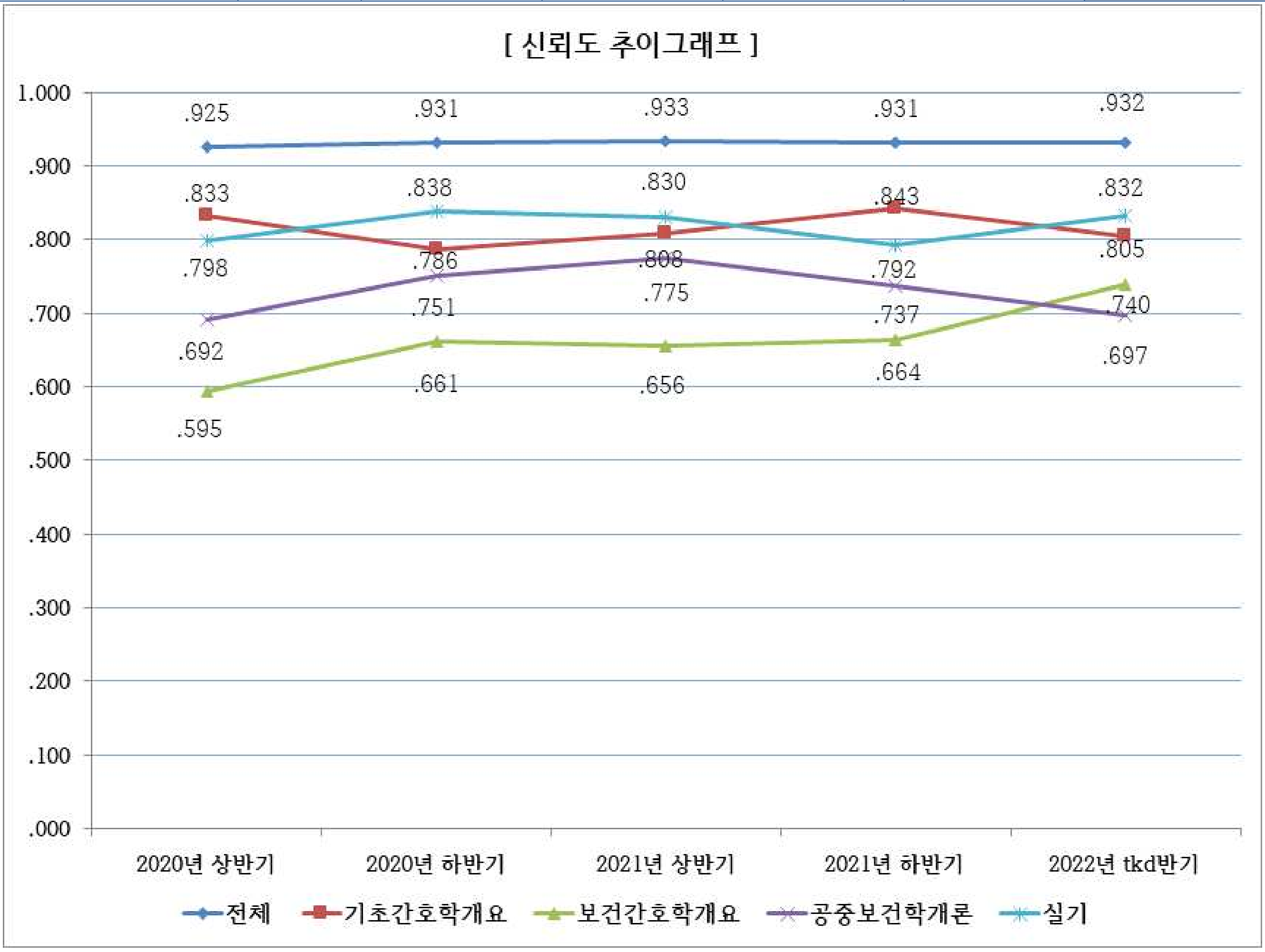

해석

- 간호조무사 자격시험 전체 문항에서 일관되게 해당 영역을 측정하고 있는 것으로 나타남
- 시험 전체, 보건간호학 개요, 실기 과목을 대상으로 했을 때, 전회대비 신뢰도는 각각 .001, .076, .040 감소함
- 기초간호학개요 과목, 공중보건학개론 과목을 대상으로 했을 때, 전회 대비 신뢰도는 각각 .038, .040 증가함.
