# Supplementary material for: Item difficulty index, discrimination index, and reliability of the 26 health professions licensing examinations in 2022, Korea: a psychometric study
Source: J Educ Eval Health Prof. 2023 Nov 22;20:31. doi: 10.3352/jeehp.2023.20.31 (PMC11959405; doi:10.3352/jeehp.2023.20.31)
Supplement: Supplementary file 1 — Supplement 1. Item analysis results of 26 health professions licensing examinations administered during late 2022 and early 2023. [file jeehp-20-31_Suppl1.zip › 2022│Γ╡╡ ┴a11╚╕ 1▒▐ ╛≡╛ε└τ╚░╗τ ▒╣░í╜├╟Φ ║╨╝«░ß░·.pdf]

2022년도 제11회
1급 언어재활사 국가시험
문항분석 결과

국민이 신뢰하고 감동하는 시험평가기관
한국보건의료인국가시험원
KOREA HEALTH PERSONNEL LICENSING EXAMINATION INSTITUTE

일반 용어 정의

□ **평균**

- 집단에서의 대표적 경향 값으로 전체 값을 더하여 총 응시자로 나눈 값

□ **표준편차**

- 평균과 각 점수의 차이인 편차들의 평균으로 점수가 흩어져 분포되어 있는 정도

□ **추정난이도**

- 문항개발자가 예측한 정답률

□ **검사이론**

- 검사와 검사를 구성하고 있는 문항의 양호도를 분석 및 평가하는 방법을 정의한 이론체계
- 대표적으로 고전검사이론과 문항반응이론이 있음

고전검사이론 용어 정의

□ **고전검사이론(Classical Test Theory; CTT)**

- 검사의 질을 분석하는 검사이론 중 한 가지로 19세기 말부터 전개되어 현재까지 주로 사용되고 있는 검사이론임
- 고전검사이론에 의한 문항과 응시자 능력 추정치는 다음과 같음

○ **문항난이도**

· 검사 문항의 쉽고 어려운 정도를 나타내는 지수
· 난이도 지수는 총 반응 수에 대한 정답 반응 수의 비율로 문항의 정답률임
· 문항난이도는 0~100까지의 값을 가짐
· 난이도 값이 큰 경우, 쉬운 문항으로 '난이도가 낮다'라고 해석하며, 난이도 값이 작은 경우, 어려운 문항으로 '난이도가 높다'라고 해석함

○ **문항변별도**

· 각 문항이 응시자의 능력 수준을 변별할 수 있는 정도를 나타내는 지수
· 문항변별도는 -1~+1까지의 값을 가지며, 1에 가까울수록 변별력 크다고 해석함
· 일반적으로 문항변별도가 0.3 이상이면 우수한 문항으로 평가함
· 구하는 방식에는 '상하위집단 구분법', '문항-총점 상관계수' 등이 있음
1) **변별도 1(상하위구분법):** 상위 27%와 하위 27% 집단의 난이도 차이를 구하는 방식
2) **변별도 2(상관계수법):** 문항-총점과의 상관계수로 구하는 방식

○ **신뢰도**

· 시험이 평가하고자 하는 것을 일관성 있게 측정하는가로 시험이 오차없이 정확하게 측정한 정도를 의미함
· 국시원에서는 문항의 내적일관성(Cronbach α)으로 신뢰도를 추정하며 1에 가까울수록 신뢰도가 높다고 해석함

문항반응이론 용어 정의

□ **문항반응이론(Item Response Theory; IRT)**

- 고전검사이론과 같이 검사의 질을 분석하는 검사이론 중 한 가지로 20세기 초에 제안됨
- 문항분석과 응시자의 능력을 추정하는데 전제 및 방법에서 고전검사이론과 차이를 보이며, 문항반응이론에 의한 문항과 응시자 능력 추정치는 다음과 같음

○ **문항난이도**

· 검사 문항의 쉽고 어려운 정도를 나타내는 지수

· 난이도 지수는 응시자의 50%가 정답을 맞힐 것으로 기대되는 능력수준임

· 난이도의 범위는 무한하나 일반적으로 -2.0 이하는 매우 쉬운 것으로, +2.0 이상은 매우 어려운 것으로 해석함

○ **문항변별도**

· 각 문항이 응시자의 능력 수준을 변별할 수 있는 정도를 나타내는 지수

· 능력이 증가함에 따라 정답을 맞힐 확률이 얼마나 변화하는지를 나타내는 지수로 지수가 클수록 변별력이 크다고 해석함

· 변별도의 범위는 무한하나 일반적으로 0.0 이하는 변별력이 없는 것으로 해석하며, 0.65 이상이면 우수한 문항으로 해석함

○ **검사정보함수**

· 검사에 대한 반응으로부터 구할 수 있는 정보량을 나타내는 그래프로, 검사가 응시자의 능력을 얼마나 정확히 추정하는가에 대해 알려줌

· 검사정보함수는 측정오차의 분산과 역의 관계를 가짐

· 검사정보함수에서 최대정보를 가지는 지점의 능력수준에 해당하는 응시자의 능력을 가장 정확히 추정할 수 있다고 해석함

목 차

I. 시행 결과 ······ 6
1. 시험 현황 ······ 7
1) 시험명 ······ 7
2) 시험시행일 ······ 7
3) 응시현황 ······ 7
4) 과목별 문항 수, 배점 및 과락 점수 ······ 7
2. 합격률과 평균성적 ······ 7
1) 합격 및 불합격 현황 ······ 7
2) 과목별 과락자수 내역 ······ 7
3) 전회 대비 합격률과 평균성적 ······ 8

II. 문항분석 결과 ······ 9
1. 성적 ······ 10
1) 전체 성적분포도 ······ 10
2) 과목별 성적분포도 ······ 11
2. 난이도와 변별도 ······ 13
1) 전체 난이도와 변별도 ······ 13
2) 과목별 난이도와 변별도 ······ 16
3) 지식수준별 난이도와 변별도 ······ 32
4) 자료유형별 난이도와 변별도 ······ 40
3. 난이도와 변별도 간 산포도 ······ 47
1) 전체 난이도와 변별도 간 산포도 ······ 47
2) 과목별 난이도와 변별도 간 산포도 ······ 47
4. 신뢰도 분석 ······ 50

Ⅰ. 시행 결과

1. 시험 현황

1) 시험명: 2022년도 제11회 1급 언어재활사 국가시험

2) 시험시행일: 2022년 12월 3일

3) 응시현황

응시대상자수	결시자수	부정행위자수	응시자 준수사항 위반자 수		응시자수 (%)
			휴대폰 소지	신분증 미지참	
1,004	54	0	0	0	946 (94.2)

4) 과목별 문항 수, 배점 및 과락 점수

교 시	과 목 명	문제 수	배점	총점	합격자 점수기준	
					과목 과락기준	총점 합격기준
1교시	신경언어장애	24	1	24	9.6	84.0
1교시	언어발달장애	24	1	24	9.6	
1교시	유창성장애	24	1	24	9.6	
2교시	음성장애	24	1	24	9.6	
2교시	조음음운장애	24	1	24	9.6	
2교시	언어재활현장실무	20	1	20	8.0	
계		140		140		

2. 합격률과 평균성적

1) 합격 및 불합격 현황

합격자수 (%)	불합격자수(%)				채점보류자수
	평락	과락	기권	계	
559 (59.1)	366 (38.7)	20 (2.1)	1 (0.1)	387 (40.9)	2 (00.0)

2) 과목별 과락자수 내역

과목명 / 과락자수	신경언어 장애	언어발달 장애	유창성장애	음성장애	조음음운 장애	언어재활 현장실무
과목별 과락자 수	3	2	1	2	11	1
전과목 과락자 수	-					

3) 전회 대비 합격률과 평균성적

회차	년도	합격률(%)	평균성적	표준편차	백분율 환산점수
제7회	2018	42.7	82.1	14.1	58.6
제8회	2019	77.7	96.3	14.5	68.7
제9회	2020	67.6	91.6	15.1	65.5
제10회	2021	74.0	95.2	13.5	68.0
**제11회**	**2022**	**59.1**	**88.0**	**14.9**	**62.8**

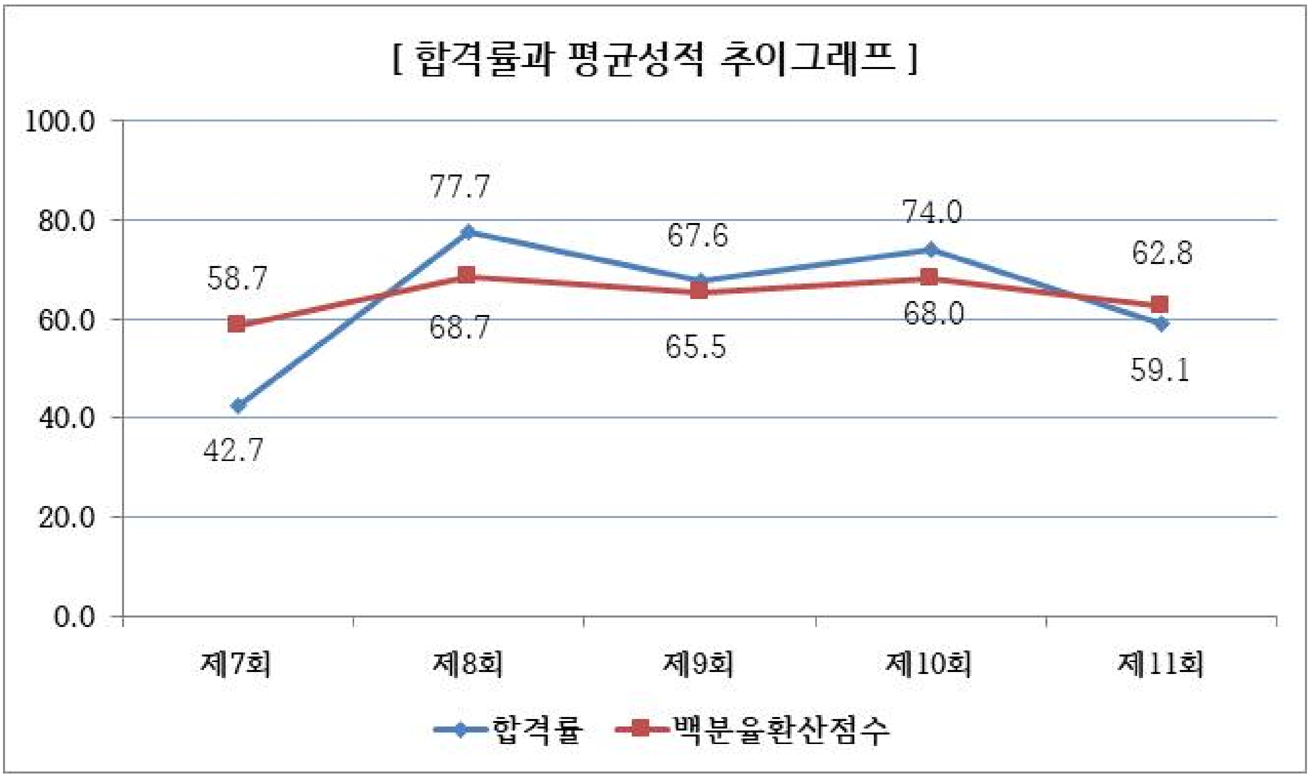

해석

- 전년 대비 합격률은 14.9%, 백분율 환산점수는 5.2 점 감소함
- 표준편차는 1.4 점 증가함

II. 문항분석 결과

1. 성적

1) 전체 성적분포도

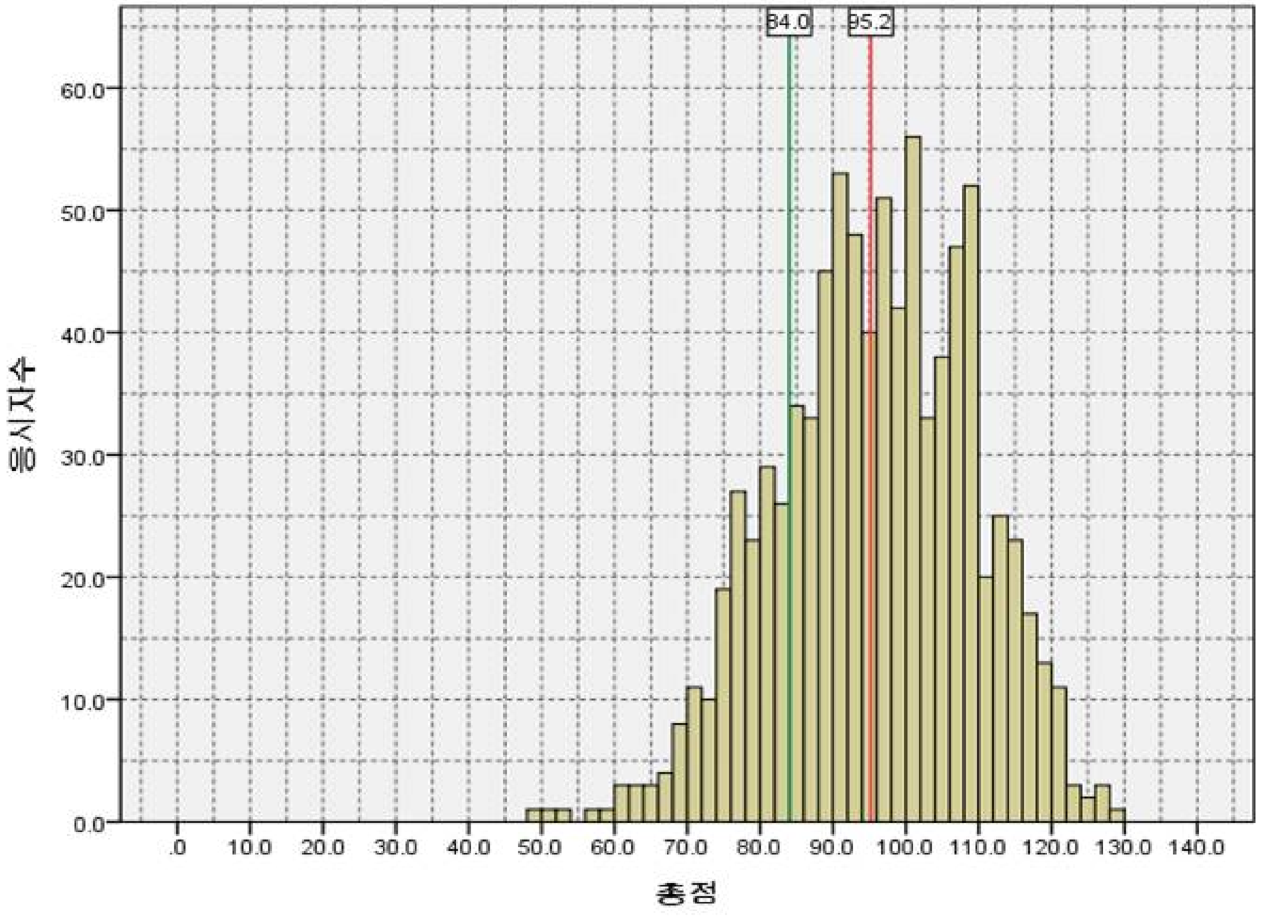

응시자	총점	합격선	평균성적	표준편차
947※	140	84	88.0	14.9

※ 947명은 전체응시자(946명)에서 채점보류자수(2명)를 더하고 기권자(1명)를 제외한 수치임

2) 과목별 성적분포도

가) 신경언어장애

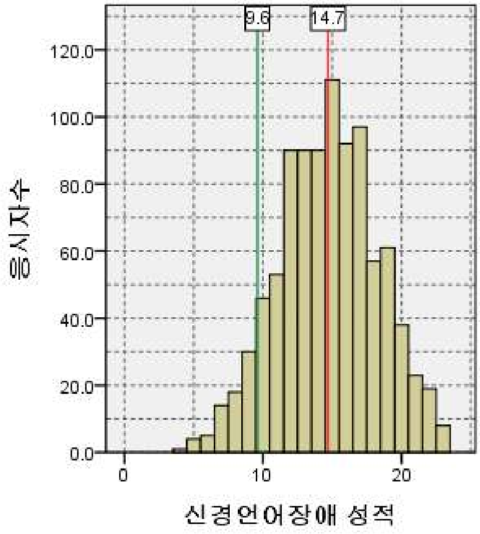

총점	과락선	평균성적	표준편차
24	9.6	14.7	3.6

나) 언어발달장애

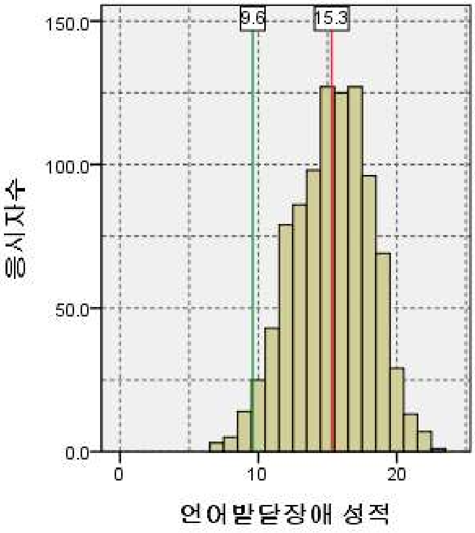

총점	과락선	평균성적	표준편차
24	9.6	15.3	2.8

다) 유창성장애

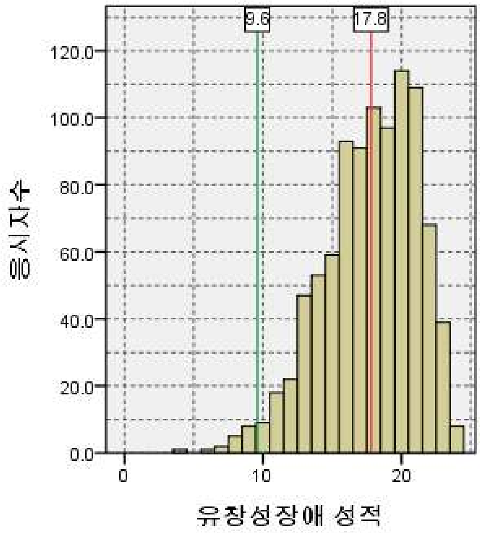

총점	과락선	평균성적	표준편차
24	9.6	17.8	3.4

음성장애

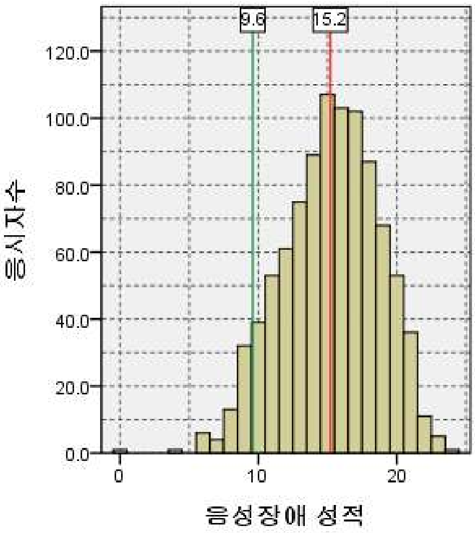

총점	과락선	평균성적	표준편차
24	9.6	15.2	3.5

라) 조음음운장애

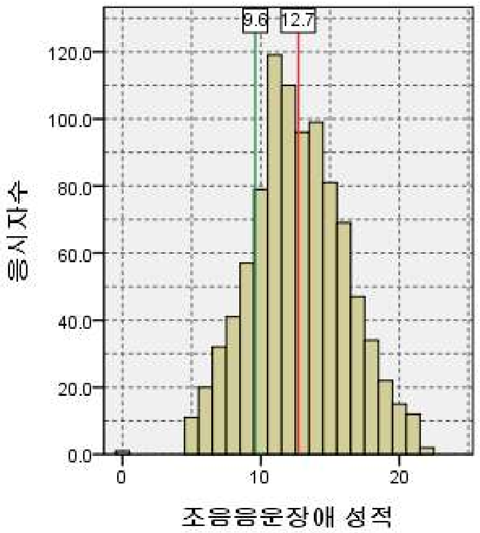

총점	과락선	평균성적	표준편차
24	9.6	12.7	3.5

마) 언어재활현장실무

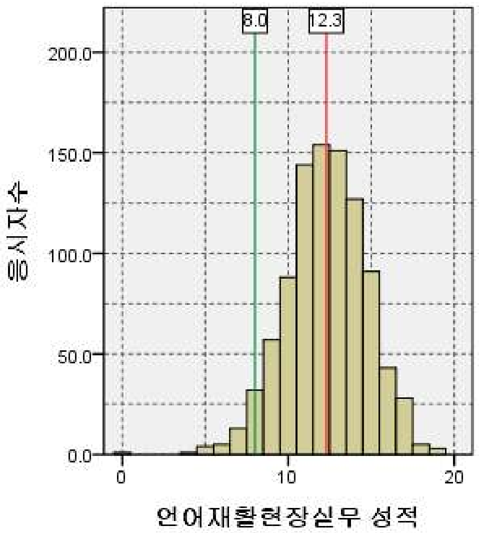

총점	과락선	평균성적	표준편차
20	8.0	12.3	2.4

2. 난이도와 변별도

1) 전체 난이도와 변별도

가) 전회 대비 전체 난이도와 변별도

회차	난이도		변별도1		변별도2	
	평균	표준편차	평균	표준편차	평균	표준편차
**제7회**	58.7	23.6	.24	.14	.23	.11
제8회	68.8	22.1	.25	.14	.25	.11
제9회	65.5	22.5	.27	.16	.25	.12
제10회	68.0	23.1	.24	.15	.23	.11
**제11회**	**62.9**	**23.1**	**.26**	**.15**	**.25**	**.12**

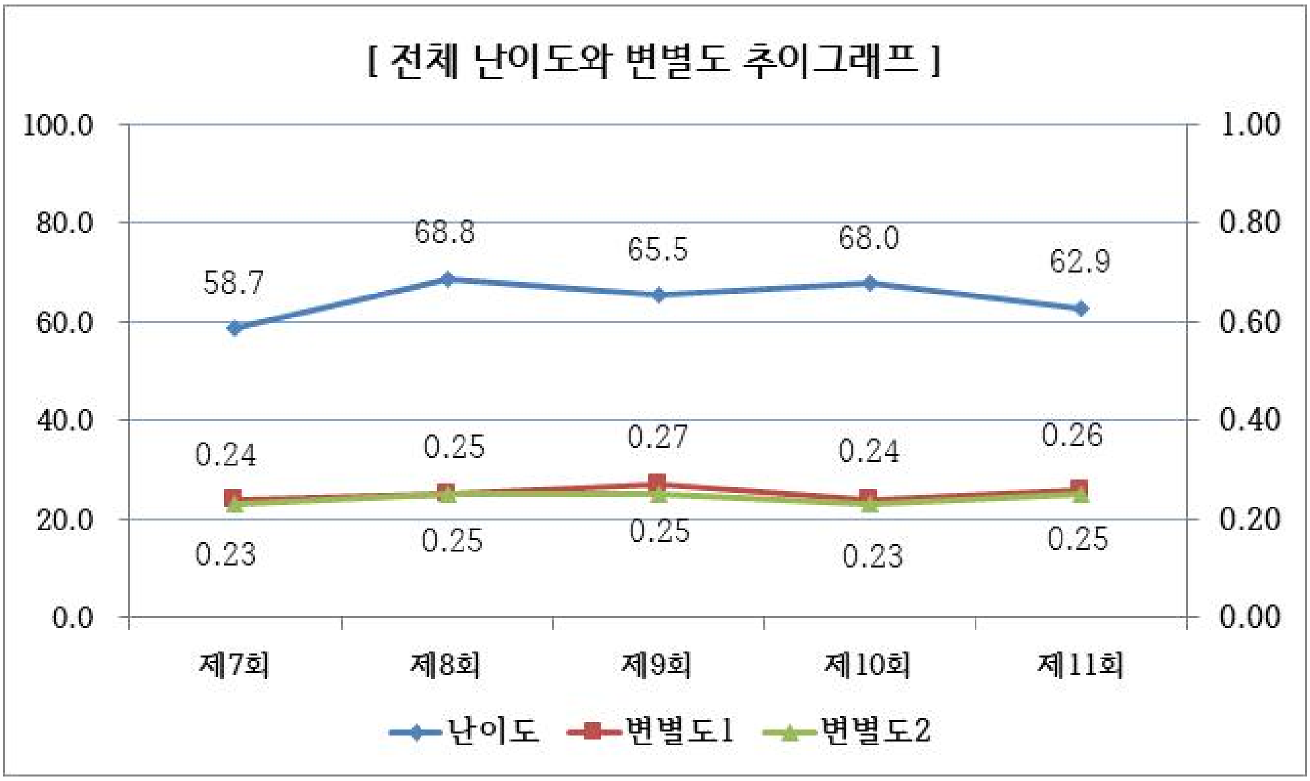

해석

- 전년 대비 난이도 지수는 5.1 감소함
- 변별도 1 ,2 지수 각각 .02, .02 증가함

나) 전체 난이도와 변별도 분포도 및 비율분석

(1) 전체 난이도 분포도 및 비율분석

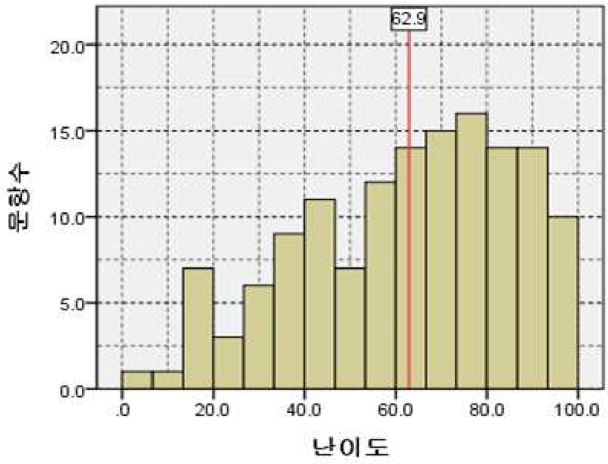

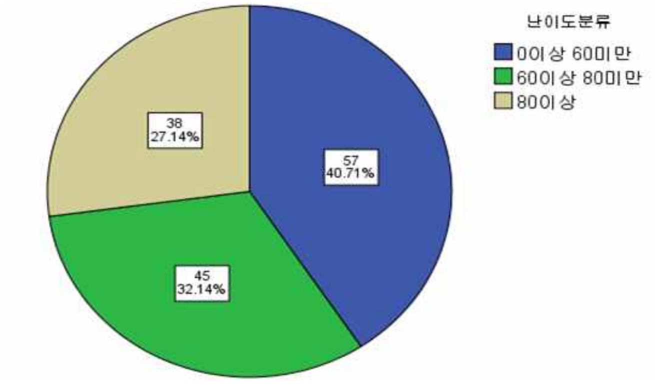

총점	난이도	표준편차
140	62.9	23.1

난이도	문항수	비율(%)
0~60미만	57	40.7
60~80미만	45	32.1
80~100	38	27.1
전체	140	100.0

(2) 전체 변별도1 분포도 및 비율분석

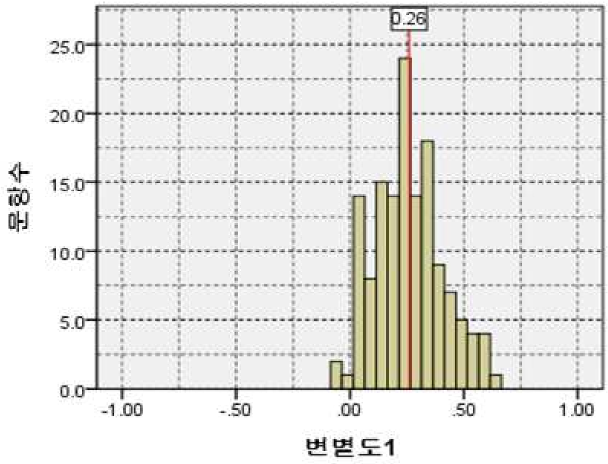

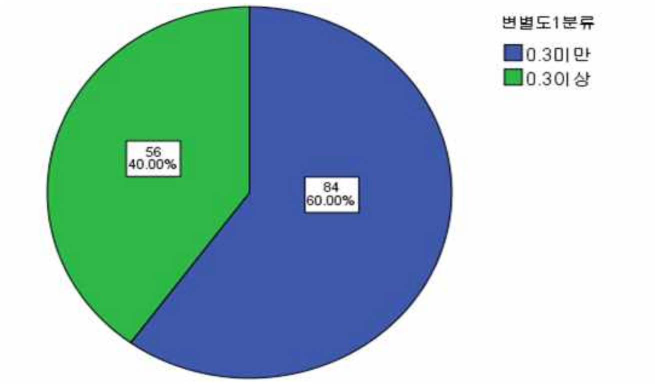

총점	변별도1	표준편차
140	.26	.15

변별도1	문항수	비율(%)
0.3미만	84	60.0
0.3이상	56	40.0
전체	140	100.0

(3) 전체 변별도2 분포도 및 비율분석

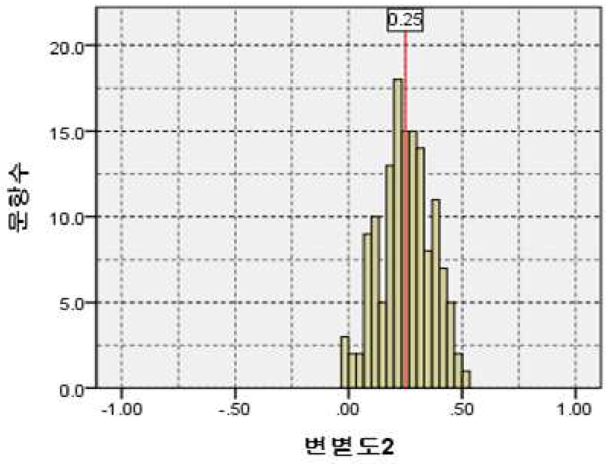

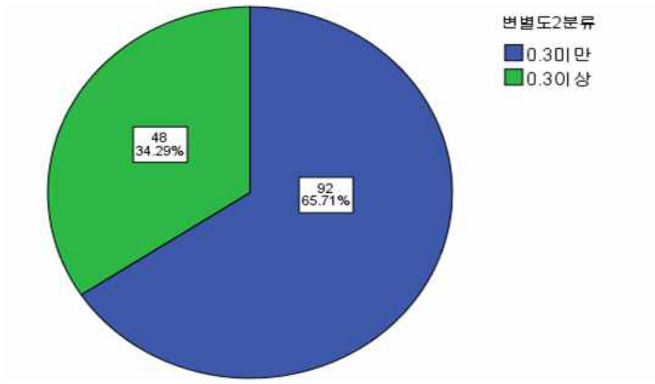

총점	변별도2	표준편차
140	.25	.12

변별도2	문항수	비율(%)
0.3미만	92	65.7
0.3이상	48	34.3
전체	140	100.0

해석

- 난이도 지수가 60 미만인 문항이 전체 140 문항 중 57 문항이었으며, 다음으로 60 에서 80 사이인 문항이 45 문항, 80 에서 100 사이인 문항이 38 문항인 것으로 나타남
- 변별도 1 지수를 기준으로 분류하였을 때, 0.3 미만인 문항이 84 문항으로 0.3 이상인 문항이 56 문항인 것에 비해 더 많이 나타남
- 변별도 2 지수를 기준으로 분류하였을 때, 0.3 미만인 문항이 92 문항으로 0.3 이상인 문항이 48 문항인 것에 비해 더 많이 나타남

2) 과목별 난이도와 변별도

가) 전회 대비 과목별 난이도와 변별도

(1) 전회 대비 신경언어장애 난이도와 변별도

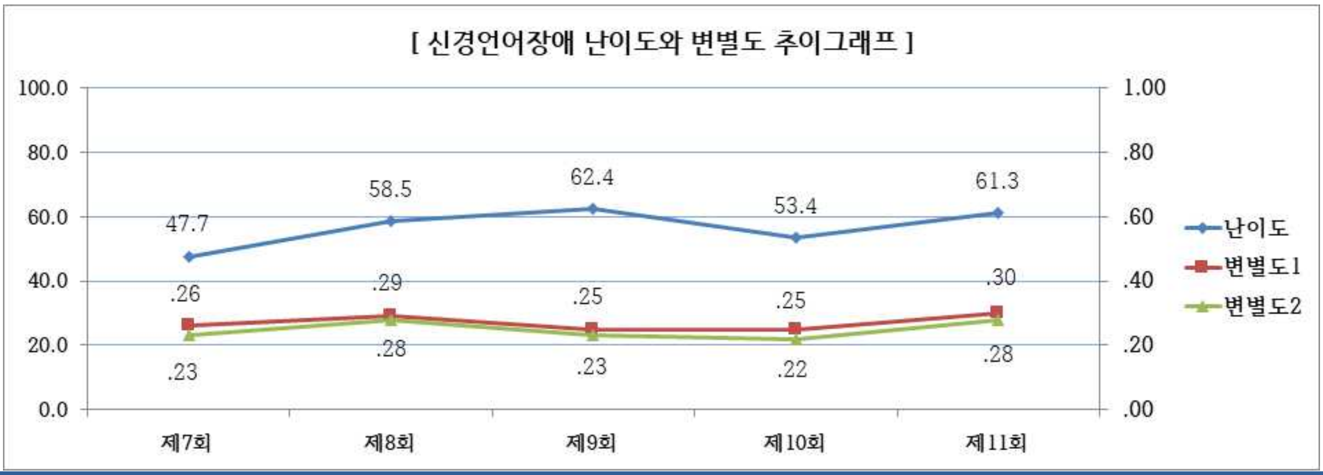

회차	난이도		변별도1		변별도2	
	평균	표준편차	평균	표준편차	평균	표준편차
제7회	47.7	19.7	.26	.13	.23	.11
제8회	58.5	19.9	.29	.13	.28	.08
제9회	62.4	20.7	.25	.15	.23	.10
제10회	53.4	26.6	.25	.13	.22	.11
**제11회**	**61.3**	**21.8**	**.30**	**.12**	**.28**	**.09**

(2) 전회 대비 언어발달장애 난이도와 변별도

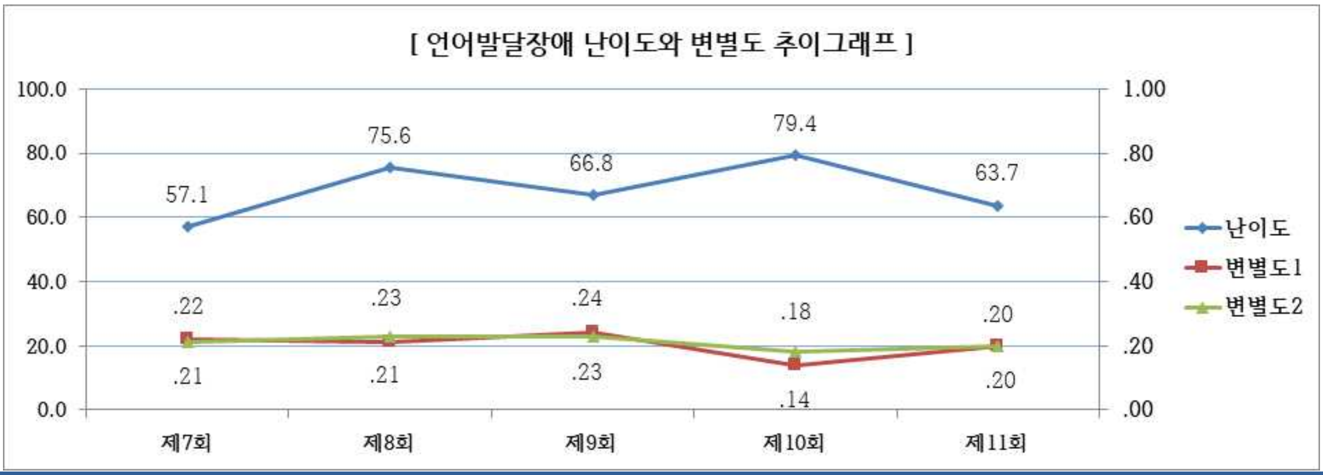

회차	난이도		변별도1		변별도2	
	평균	표준편차	평균	표준편차	평균	표준편차
제7회	57.1	24.7	.22	.16	.21	.13
제8회	75.6	22.4	.21	.13	.23	.11
제9회	66.8	24.3	.24	.14	.23	.09
제10회	79.4	24.5	.14	.12	.18	.11
**제11회**	**63.7**	**25.3**	**.20**	**.15**	**.20**	**.12**

(3) 전회 대비 유창성장애 난이도와 변별도

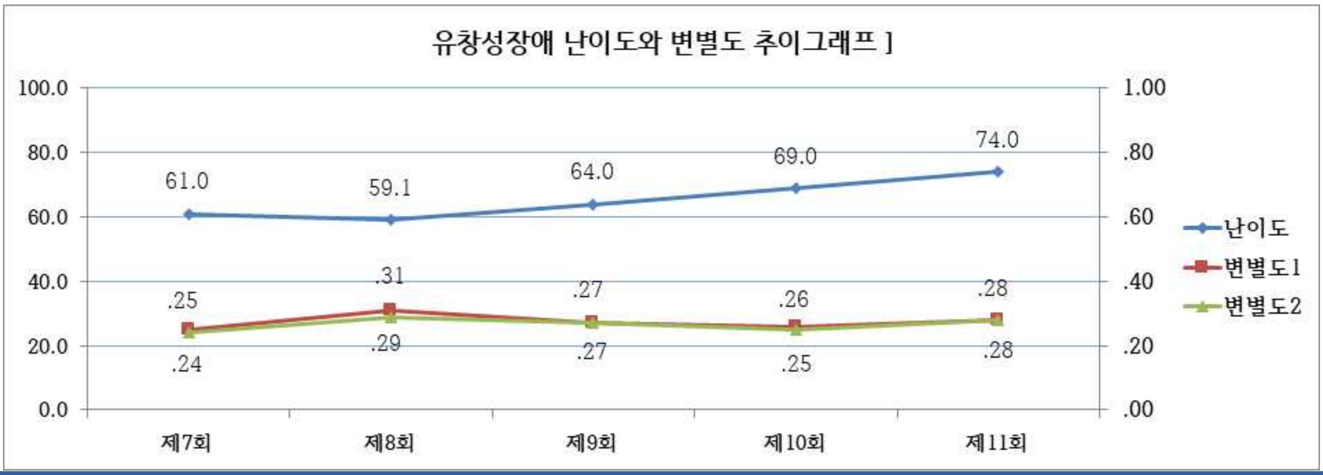

회차	난이도		변별도1		변별도2	
	평균	표준편차	평균	표준편차	평균	표준편차
제7회	61.0	23.0	.25	.13	.24	.09
제8회	59.1	20.7	.31	.11	.29	.08
제9회	64.0	24.6	.27	.15	.27	.11
제10회	69.0	21.9	.26	.15	.25	.11
**제11회**	**74.0**	**17.2**	**.28**	**.13**	**.28**	**.09**

(4) 전회 대비 음성장애 난이도와 변별도

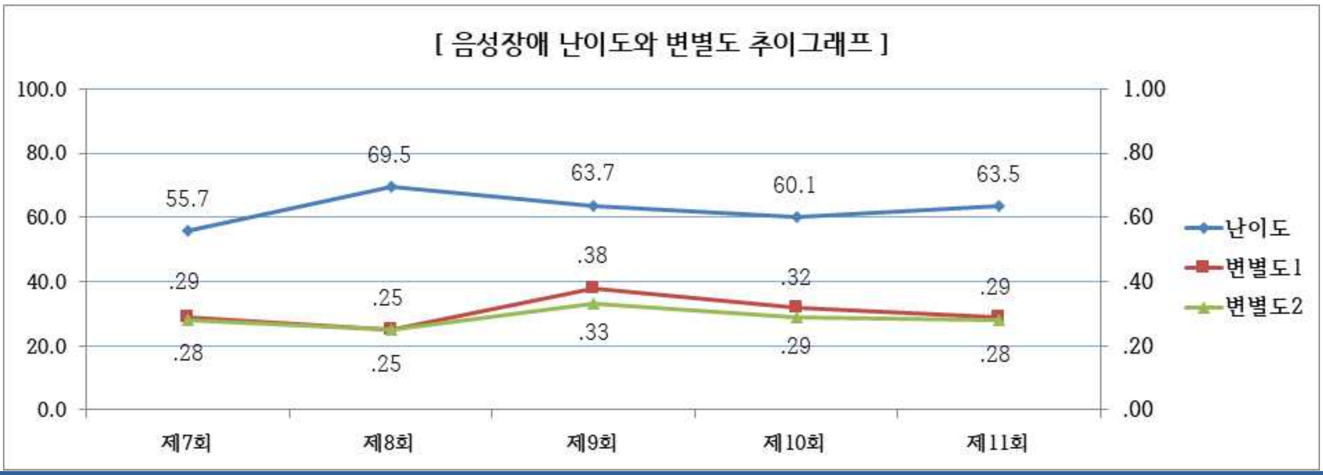

회차	난이도		변별도1		변별도2	
	평균	표준편차	평균	표준편차	평균	표준편차
제7회	55.7	24.8	.29	.14	.28	.10
제8회	69.5	21.4	.25	.13	.25	.09
제9회	63.7	13.9	.38	.12	.33	.08
제10회	60.1	18.5	.32	.12	.29	.09
**제11회**	**63.5**	**24.4**	**.29**	**.15**	**.28**	**.12**

(5) 전회 대비 조음음운장애 난이도와 변별도

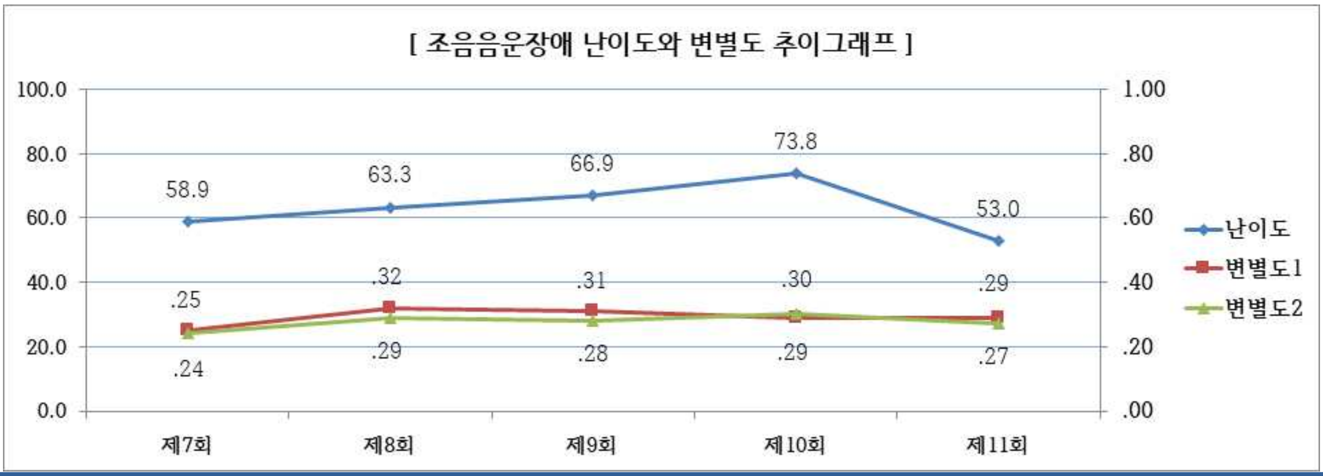

회차	난이도		변별도1		변별도2	
	평균	표준편차	평균	표준편차	평균	표준편차
제7회	58.9	21.7	.25	.14	.24	.12
제8회	63.3	18.6	.32	.11	.29	.09
제9회	66.9	22.1	.31	.19	.28	.14
제10회	73.8	15.5	.29	.13	.30	.09
**제11회**	**53.0**	**23.7**	**.29**	**.18**	**.27**	**.15**

(6) 전회 대비 언어재활현장실무 난이도와 변별도

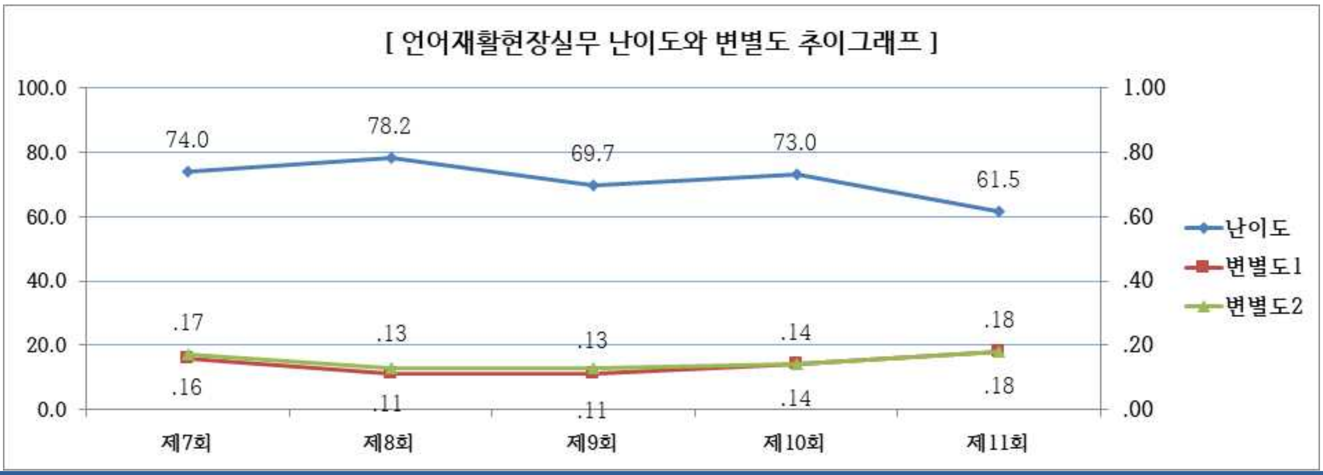

회차	난이도		변별도1		변별도2	
	평균	표준편차	평균	표준편차	평균	표준편차
제7회	74.0	22.4	.16	.14	.17	.12
제8회	78.2	26.3	.11	.13	.13	.11
제9회	69.7	30.0	.11	.12	.13	.09
제10회	73.0	21.1	.14	.11	.14	.09
**제11회**	**61.5**	**22.7**	**.18**	**.12**	**.18**	**.09**

해석

- 전회 대비 신경언어장애, 유창성장애, 음성장애 과목의 난이도 지수는 각각 7.9, 5.0, 3.4 증가함
- 언어발달장애, 조음음운장애, 언어재활현장실무 과목의 난이도 지수는 각각 15.7, 20.8, 11.5 감소함
- 신경언어장애, 유창성장애, 언어발달장애, 언어재활현장실무 과목의 변별도 1 지수는 0.05, 0.06, 0.02, 0.04 증가하였으며, 음성장애 과목의 변별도 1 지수는 각각 0.03 감소함,
- 조음음운장애 과목의 변별도 1 지수는 변화 없음
- 신경언어장애, 언어발달장애, 유창성장애, 언어재활현장실무 과목의 변별도 2 지수는 각각 0.06, 0.02, 0.03, 0.04 증가함
- 음성장애, 조음음운장애 과목의 변별도 2 지수는 각각 0.01, 0.03 감소함

나) 과목별 난이도와 변별도 분포도 및 비율분석

(1) 신경언어장애 난이도와 변별도 분포도 및 비율분석

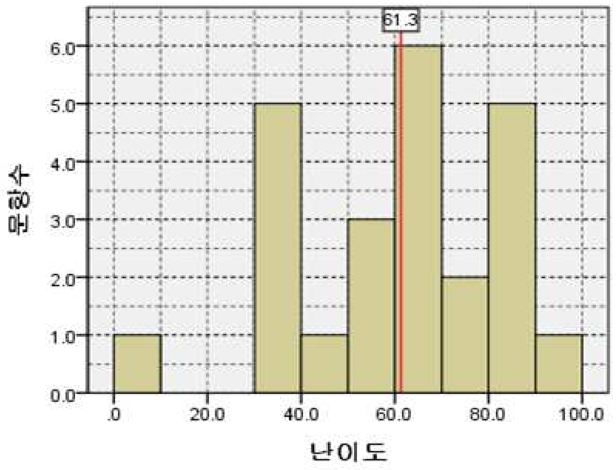

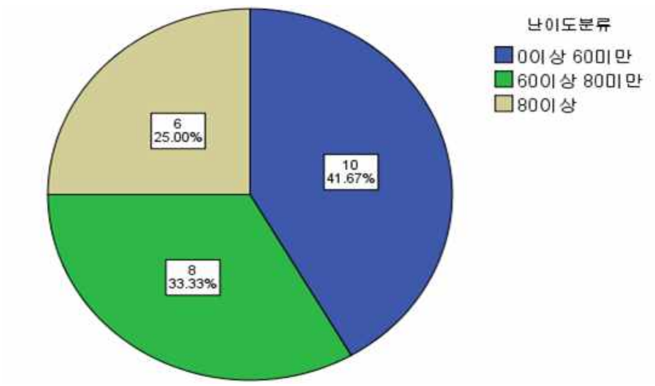

총점	난이도	표준편차
24	61.3	21.8

난이도	문항수	비율(%)
0~60미만	10	41.7
60~80미만	8	33.3
80~100	6	25.0
전체	24	100.0

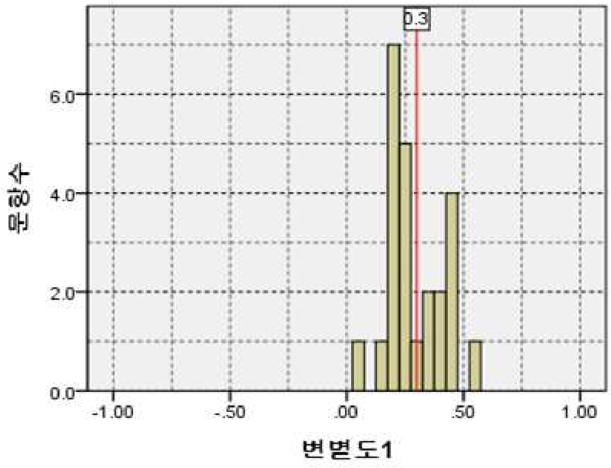

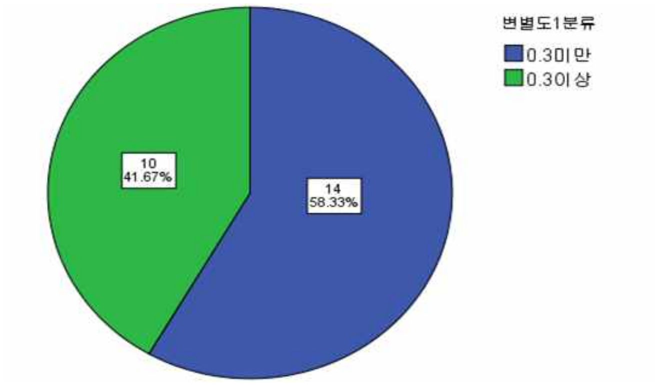

총점	변별도1	표준편차
24	.30	.12

변별도1	문항수	비율(%)
0.3미만	14	58.3
0.3이상	10	41.7
전체	24	100.0

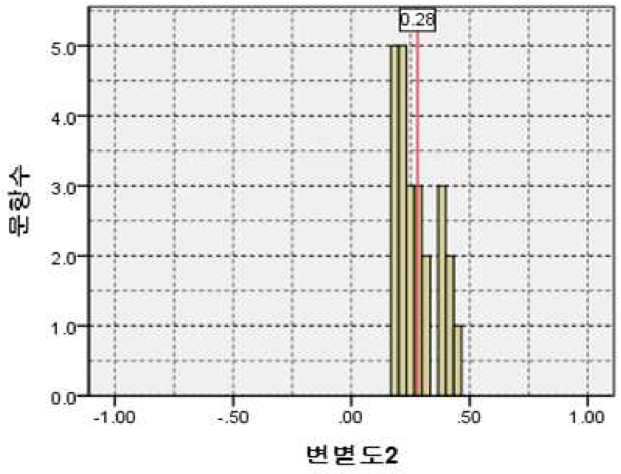

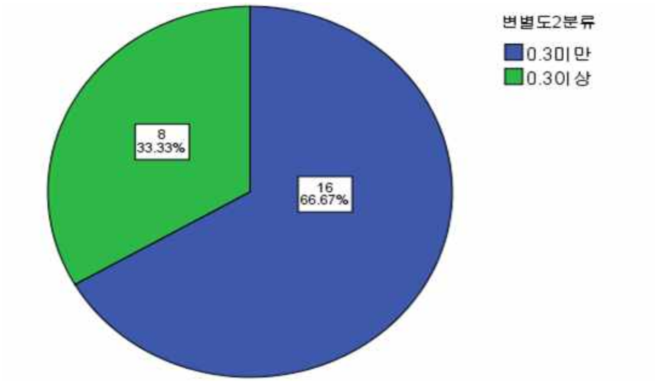

총점	변별도2	표준편차	변별도2	문항수	비율(%)
24	.28	.09	0.3미만	16	66.7
			0.3이상	8	33.3
			전체	24	100.0

해석

- 신경언어장애 과목에서 난이도 지수가 60 미만인 문항이 전체 24 문항 중 10 항이었으며, 다음으로 80 에서 100 사이의 문항이 8 문항, 60 이상 80 미만인 문항이 6 문항인 것으로 나타남
- 변별도 1 지수를 기준으로 분류하였을 때, 0.3 미만인 문항이 14 문항으로 0.3 이상인 문항이 10 문항인 것에 비해 더 많게 나타남
- 변별도 2 지수를 기준으로 분류하였을 때, 0.3 미만인 문항이 16 문항으로 0.3 이상인 문항이 8 문항인 것에 비해 더 많게 나타남

(2) 언어발달장애 난이도와 변별도 분포도 및 비율분석

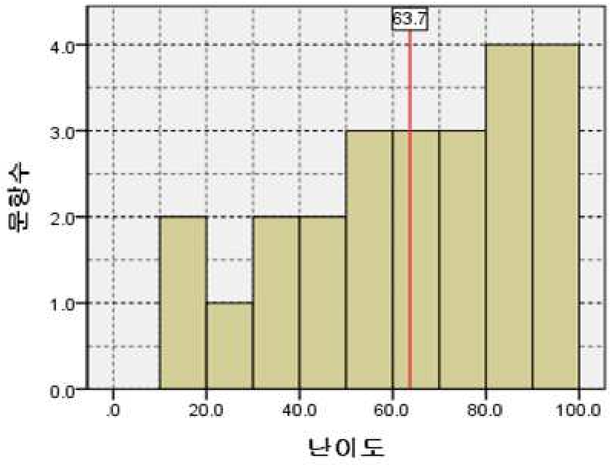

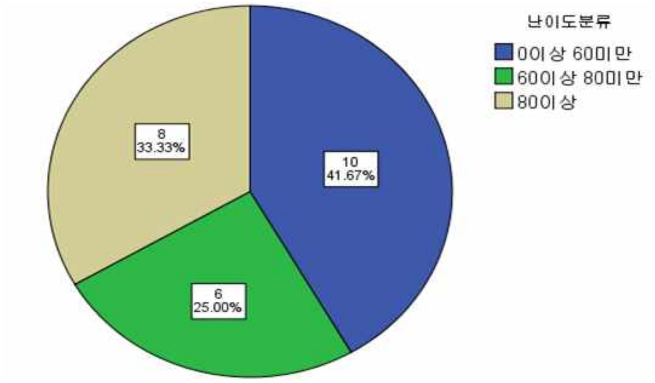

총점	난이도	표준편차
24	63.7	25.3

난이도	문항수	비율(%)
0~60미만	10	41.7
60~80미만	6	25.0
80~100	8	33.3
전체	24	100.0

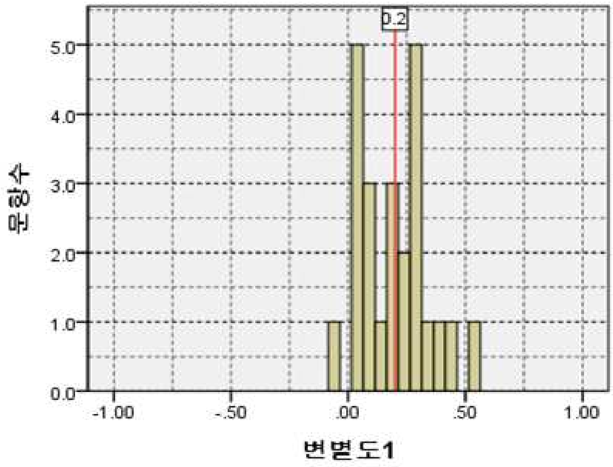

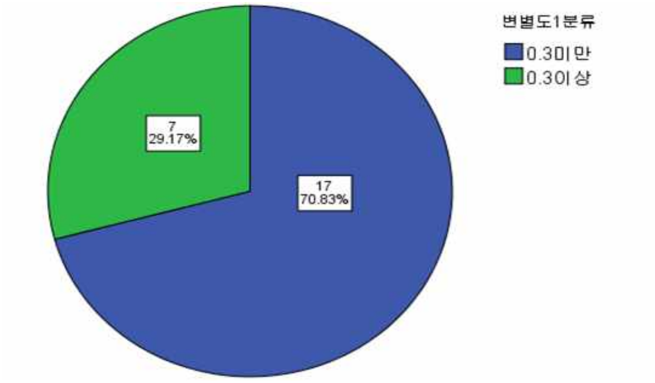

총점	변별도1	표준편차
24	.20	.15

변별도1	문항수	비율(%)
0.3미만	17	70.8
0.3이상	7	29.2
전체	24	100.0

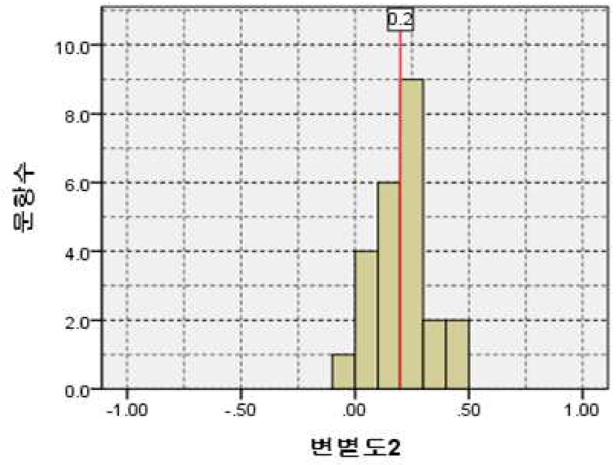

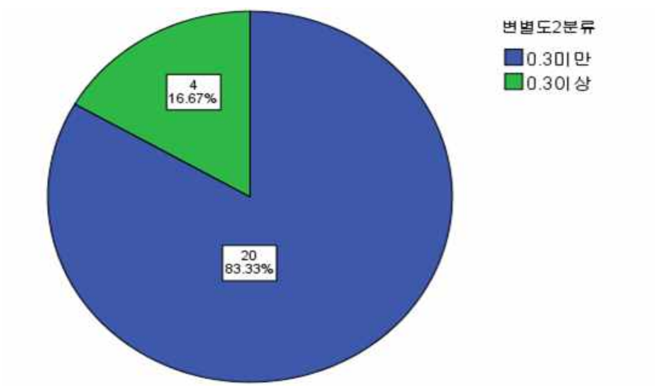

총점	변별도2	표준편차
24	.20	.12

변별도2	문항수	비율(%)
0.3미만	20	83.3
0.3이상	4	16.7
전체	24	100.0

해석

- 언어발달장애 과목에서 난이도 지수가 60 미만인 문항이 전체 24문항 중 10문항이었으며, 60에서 80 미만인 문항이 6문항, 80에서 100사이 8문항으로 나타남
- 변별도1 지수를 기준으로 분류하였을 때, 0.3미만인 문항이 17문항으로 0.3이상인 문항이 7문항인 것에 비해 더 많게 나타남
- 변별도2 지수를 기준으로 분류하였을 때, 0.3미만인 문항이 20문항으로 0.3이상인 문항이 4문항인 것에 비해 더 많게 나타남

(3) 유창성장애 난이도와 변별도 분포도 및 비율분석

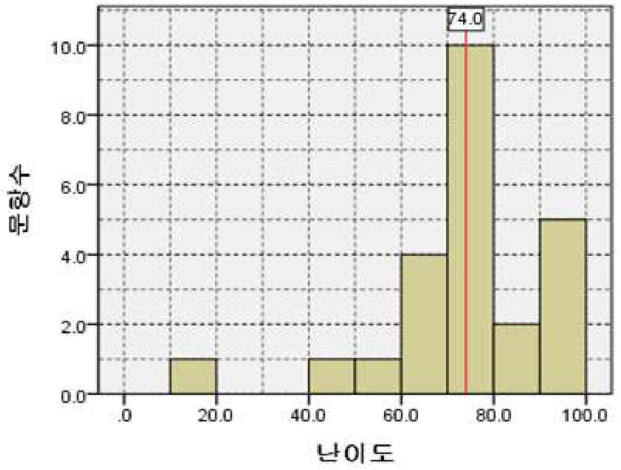

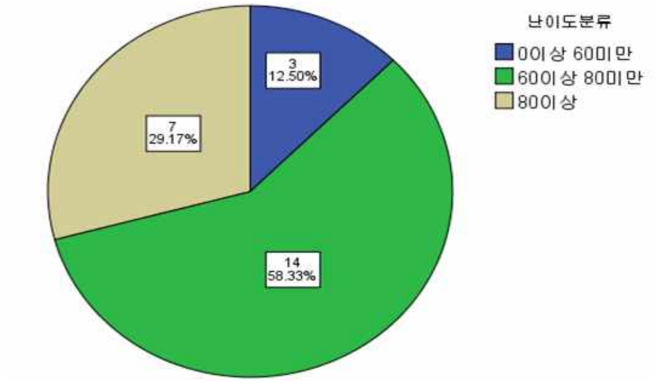

총점	난이도	표준편차
24	74.0	17.2

난이도	문항수	비율(%)
0~60미만	3	12.5
60~80미만	14	58.3
80~100	7	29.2
전체	24	100.0

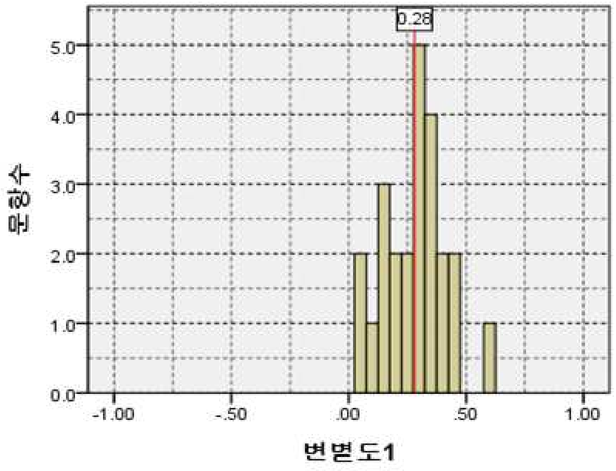

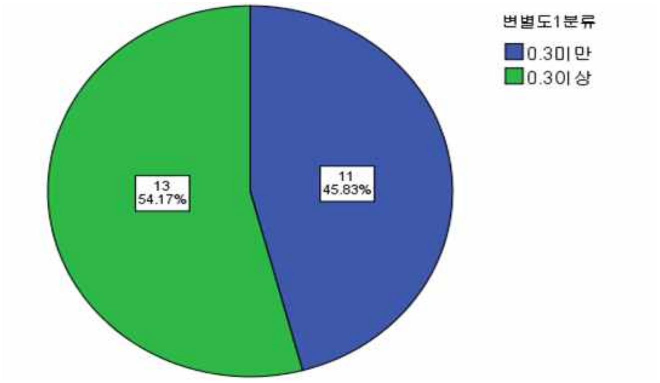

총점	변별도1	표준편차
24	.28	.13

변별도1	문항수	비율(%)
0.3미만	11	45.8
0.3이상	13	54.2
전체	24	100.0

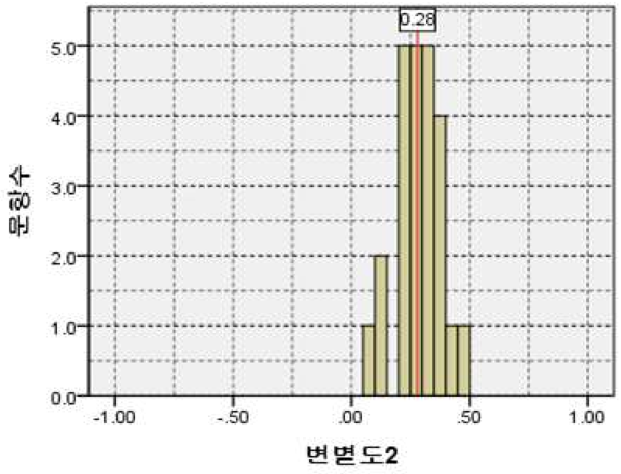

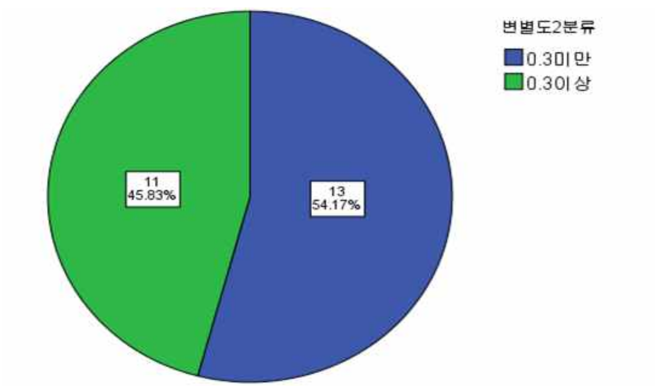

총점	변별도2	표준편차
24	.28	.09

변별도2	문항수	비율(%)
0.3미만	13	54.2
0.3이상	11	45.8
전체	24	100.0

해석

- 유창성장애 과목에서 난이도 지수가 60 미만인 문항이 전체 24 문항 중 3 문항으로 나타났으며, 다음으로 60 이상 80 미만인 문항이 14 문항, 80 에서 100 사이인 문항이 7 문항인 것으로 나타남
- 변별도 1 지수를 기준으로 분류하였을 때, 0.3 미만인 문항이 11 문항으로 0.3 이상인 문항이 13 문항인 것에 비해 더 적게 나타남
- 변별도 2 지수를 기준으로 분류하였을 때, 0.3 미만인 문항이 13 문항으로 0.3 이상인 문항이 11 문항인 것에 비해 더 많게 나타남

(4) 음성장애 난이도와 변별도 분포도 및 비율분석

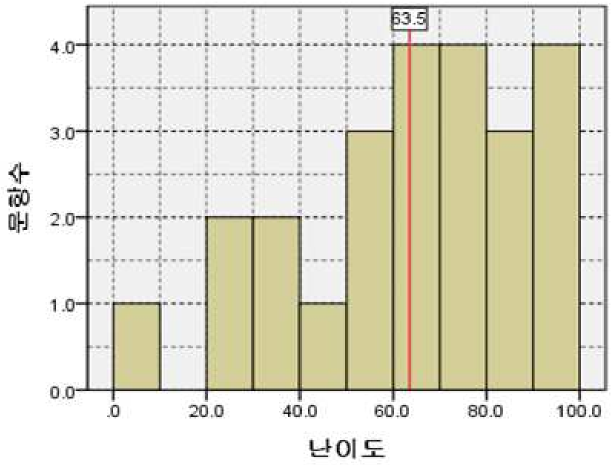

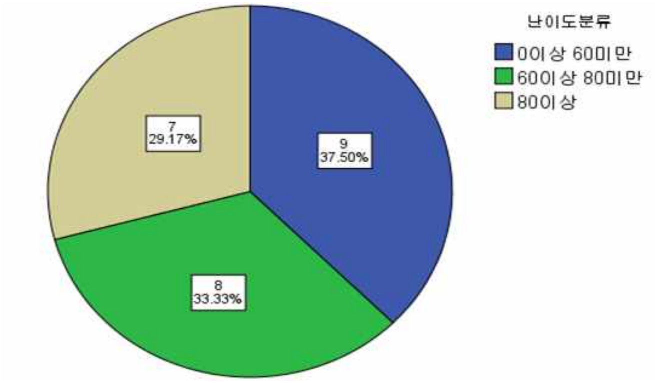

총점	난이도	표준편차
24	63.5	24.4

난이도	문항수	비율(%)
0~60미만	9	37.5
60~80미만	8	33.3
80~100	7	29.2
전체	24	100.0

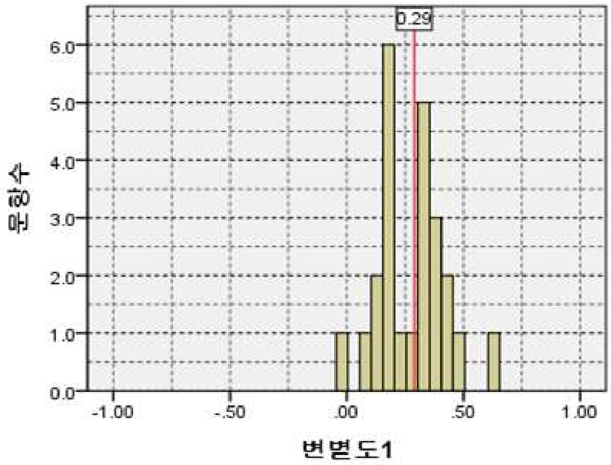

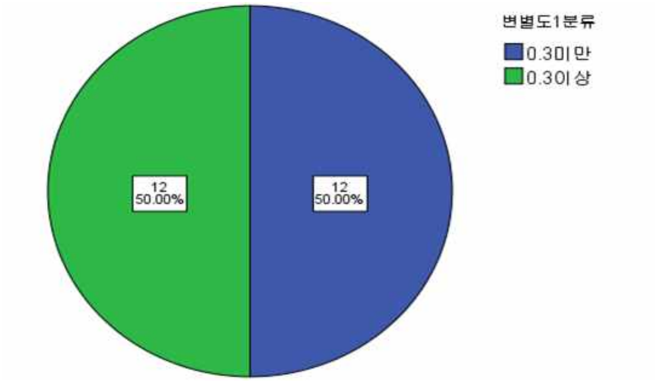

총점	변별도1	표준편차
24	.29	.15

변별도1	문항수	비율(%)
0.3미만	12	50.0
0.3이상	12	50.0
전체	24	100.0

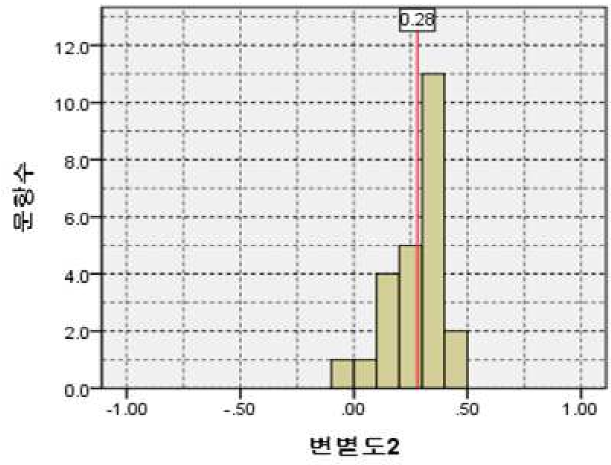

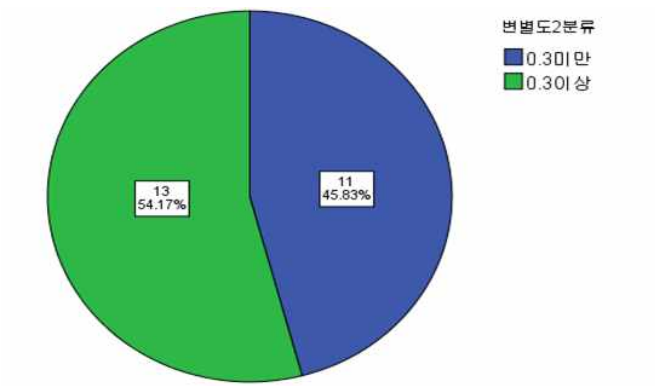

총점	변별도2	표준편차
24	.28	.12

변별도2	문항수	비율(%)
0.3미만	11	45.8
0.3이상	13	54.2
전체	24	100.0

해석

- 음성장애 과목에서 난이도 지수가 60 미만인 문항이 전체 24 문항 중 9 문항이었으며, 60 이상 80 미만인 문항이 8 문항, 80 에서 100 사이인 문항이 7 문항인 것으로 나타남
- 변별도 1 지수를 기준으로 분류하였을 때, 0.3 미만인 문항이 12 문항, 0.3 이상인 문항이 12 문항으로 같게 나타남
- 변별도 2 지수를 기준으로 분류하였을 때, 0.3 미만인 문항이 11 문항으로 0.3 이상인 문항이 13 문항인 것에 비해 더 적게 나타남

(5) 조음음운장애 난이도와 변별도 분포도 및 비율분석

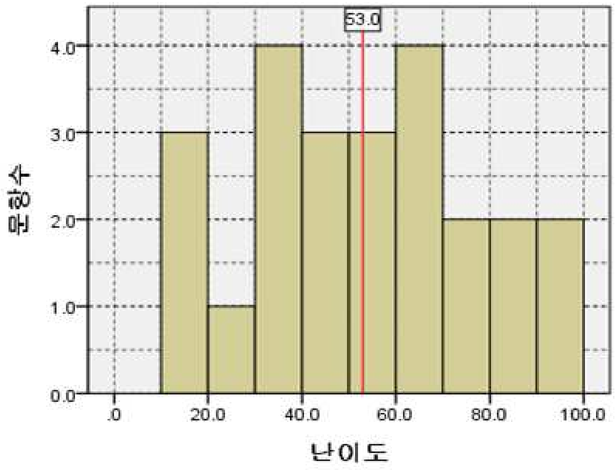

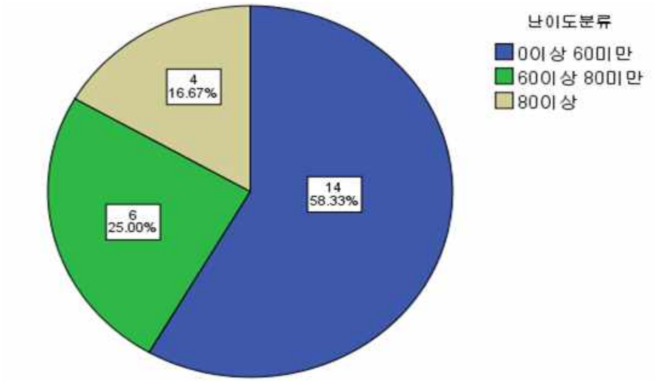

총점	난이도	표준편차
24	53.0	23.7

난이도	문항수	비율(%)
0~60미만	14	58.3
60~80미만	6	25.0
80~100	4	16.7
전체	24	100.0

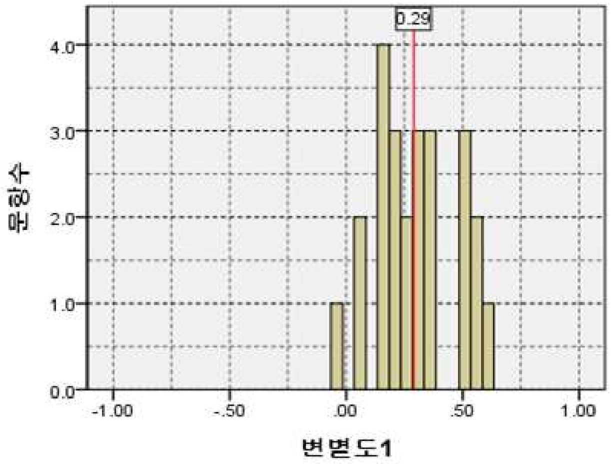

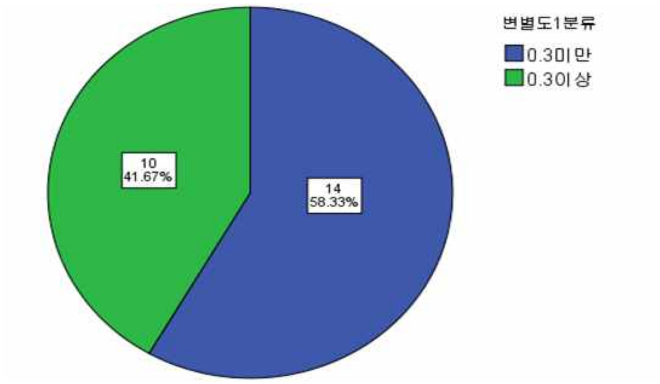

총점	변별도1	표준편차
24	.29	.18

변별도1	문항수	비율(%)
0.3미만	14	58.3
0.3이상	10	41.7
전체	24	100.0

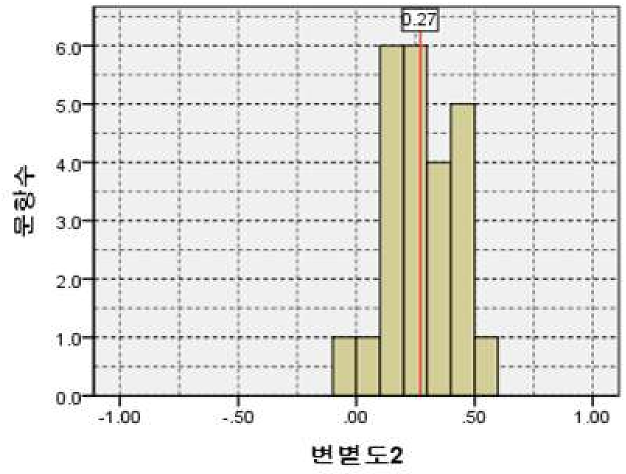

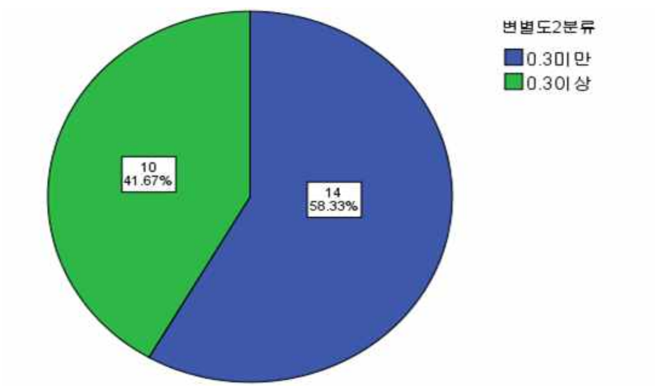

총점	변별도2	표준편차
24	.27	.15

변별도2	문항수	비율(%)
0.3미만	14	58.3
0.3이상	10	41.7
전체	24	100.0

해석

- 조음음운장애 과목에서 난이도 지수가 60 미만인 문항이 전체 24 문항 중 14 문항, 60 이상 80 미만인 문항이 6 문항, 80 에서 100 사이인 문항이 4 문항인 것으로 나타남
- 변별도 1 지수를 기준으로 분류하였을 때, 0.3 미만인 문항이 14 문항으로 0.3 이상인 문항이 10 문항인 것에 비해 더 많게 나타남
- 변별도 2 지수를 기준으로 분류하였을 때, 0.3 미만인 문항이 14 문항으로 0.3 이상인 문항이 10 문항인 것에 비해 더 많게 나타남

(6) 언어재활현장실무 난이도와 변별도 분포도 및 비율분석

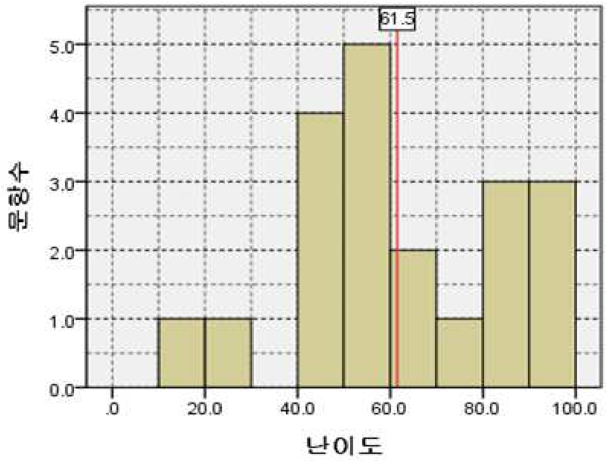

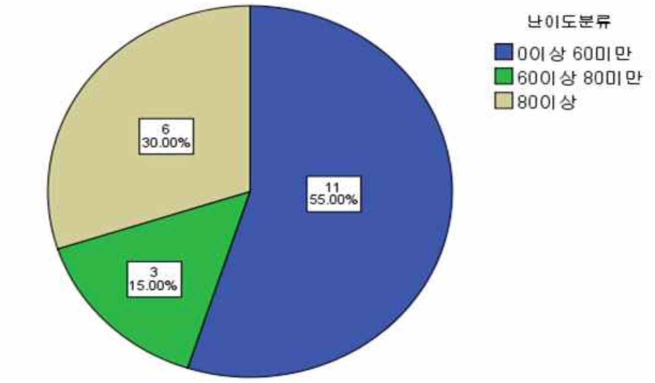

총점	난이도	표준편차
20	61.5	22.7

난이도	문항수	비율(%)
0~60미만	11	55.0
60~80미만	3	15.0
80~100	6	30.0
전체	20	100.0

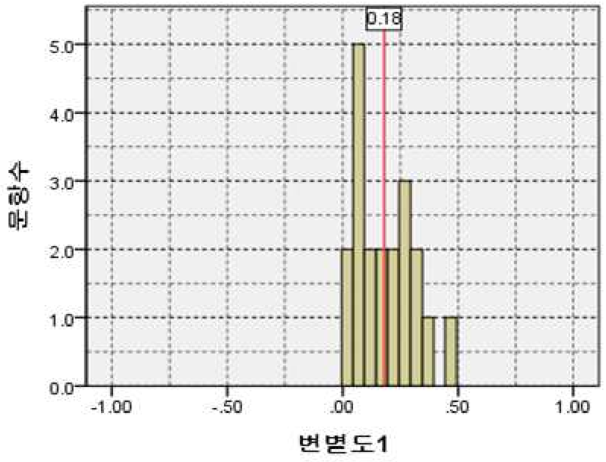

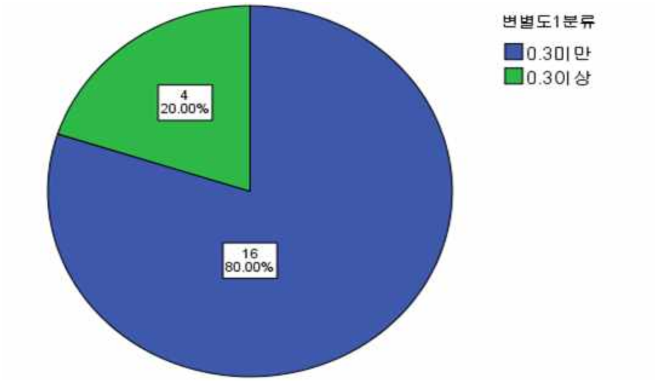

총점	변별도1	표준편차
20	.18	.12

변별도1	문항수	비율(%)
0.3미만	16	80.0
0.3이상	4	20.0
전체	20	100.0

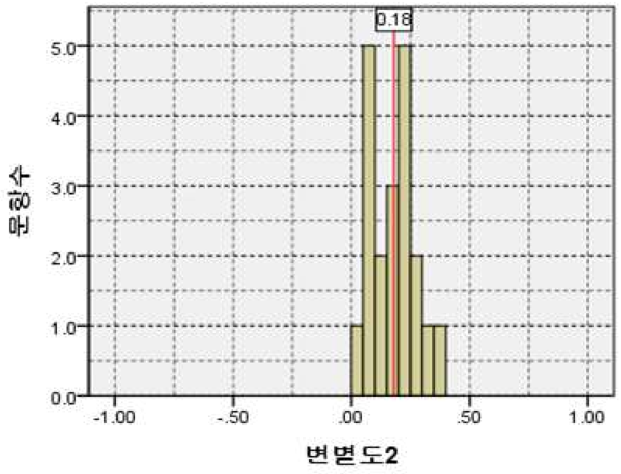

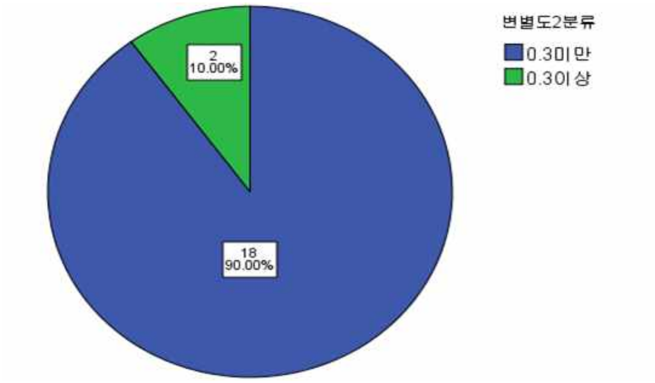

총점	변별도2	표준편차
20	.18	.09

변별도2	문항수	비율(%)
0.3미만	18	90.0
0.3이상	2	10.0
전체	20	100.0

해석

- 언어재활현장실무 과목에서 난이도 지수가 60 미만인 문항이 전체 20 문항 중 11 문항, 60 이상 80 미만인 문항이 3 문항, 80 에서 100 사이인 문항이 6 문항인 것으로 나타남
- 변별도 1 지수를 기준으로 분류하였을 때, 0.3 미만인 문항이 16 문항으로 0.3 이상인 문항이 4 문항인 것에 비해 더 많게 나타남
- 변별도 2 지수를 기준으로 분류하였을 때, 0.3 미만인 문항이 18 문항으로 0.3 이상인 문항이 2 문항인 것에 비해 더 많게 나타남

3) 지식수준별 난이도와 변별도

가) 전회 대비 지식수준별 난이도와 변별도

(1) 전회 대비 암기형 난이도와 변별도

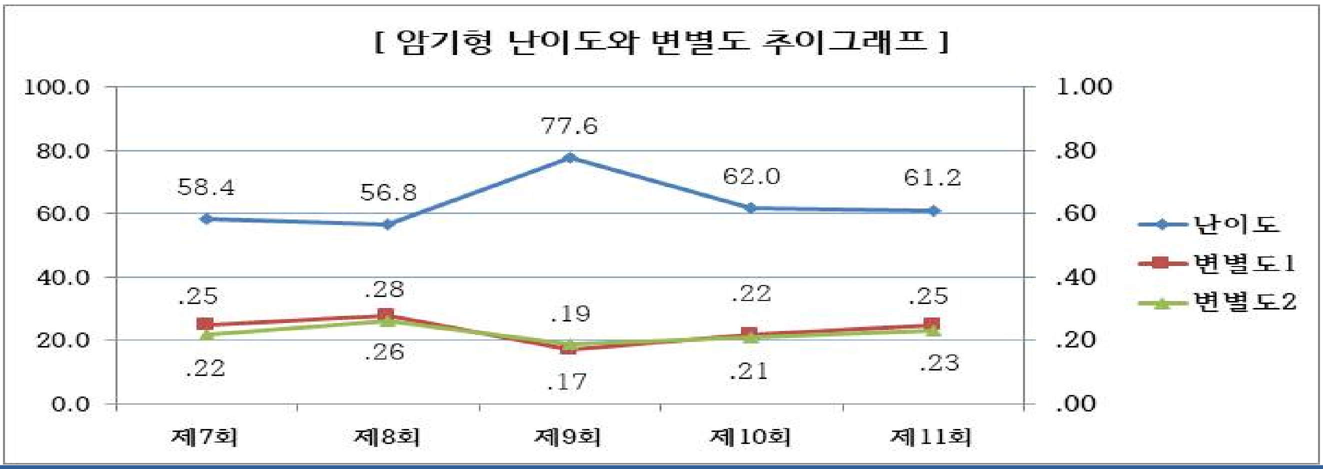

회차	난이도		변별도1		변별도2	
	평균	표준편차	평균	표준편차	평균	표준편차
제7회	58.4	23.3	.25	.16	.22	.13
제8회	56.8	23.8	.28	.15	.24	.12
제9회	77.6	23.1	.17	.13	.19	.10
제10회	62.0	23.6	.22	.15	.21	.12
**제11회**	61.2	19.5	.25	.13	.23	.11

(2) 전회 대비 해석형 난이도와 변별도

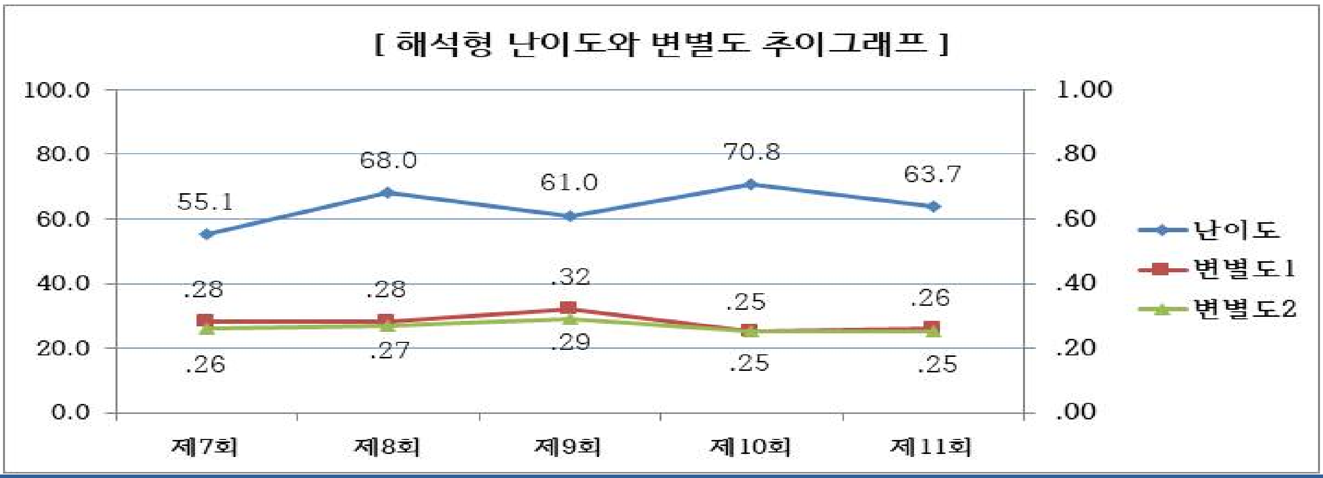

회차	난이도		변별도1		변별도2	
	평균	표준편차	평균	표준편차	평균	표준편차
제7회	55.1	22.7	.28	.14	.26	.12
제8회	68.0	20.8	.28	.15	.27	.12
제9회	61.0	22.4	.32	.17	.29	.12
제10회	70.8	21.8	.25	.15	.25	.12
**제11회**	63.7	26.2	.26	.16	.25	.12

(3) 전회 대비 해결형 난이도와 변별도

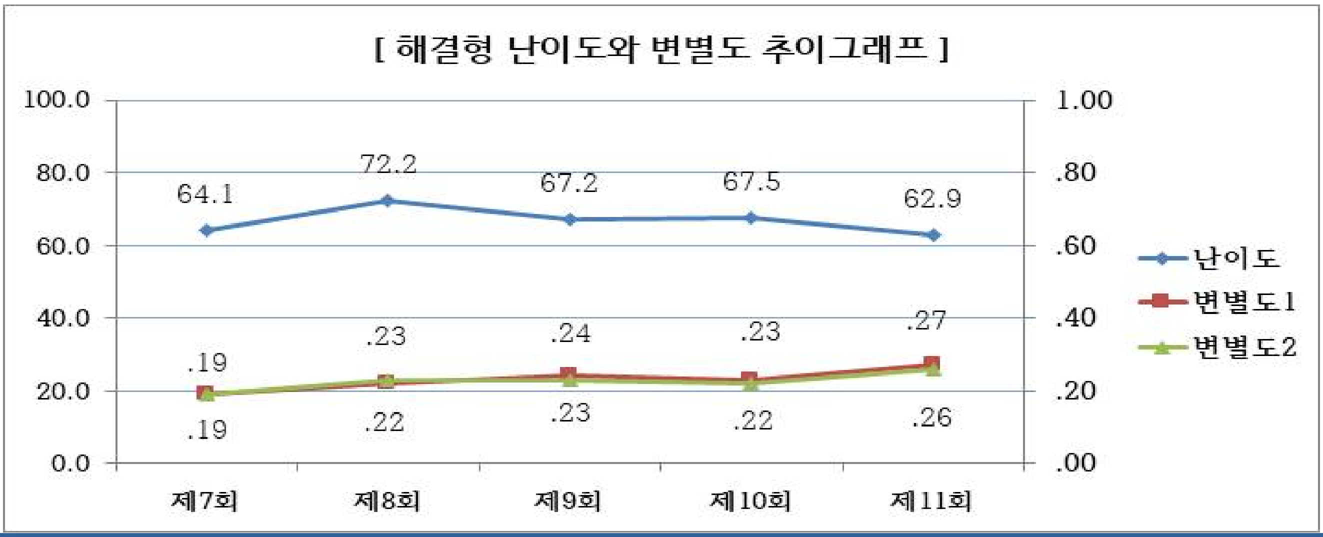

회차	난이도		변별도1		변별도2	
	평균	표준편차	평균	표준편차	평균	표준편차
제7회	64.1	24.6	.19	.12	.19	.10
제8회	72.2	22.1	.22	.12	.23	.09
제9회	67.2	21.7	.24	.15	.23	.11
제10회	67.5	24.1	.23	.14	.22	.10
**제11회**	**62.9**	**21.7**	**.27**	**.15**	**.26**	**.12**

해석

- 전회 대비 암기형, 해석형, 해결형 문항의 난이도 지수는 각각 0.8, 7.1, 4.6 증가함
- 변별도 1 지수의 경우 암기형, 해석형, 해결형 문항에서 각각 0.03, 0.01, 0.04 증가함
- 변별도 2 지수의 경우 암기형, 해결형 문항에서 각각 0.02, 0.04 증가하였으며, 해석형 문항에서는 변화가 없음

나) 지식수준별 난이도와 변별도 분포도 및 비율분석

(1) 암기형 난이도와 변별도 분포도 및 비율분석

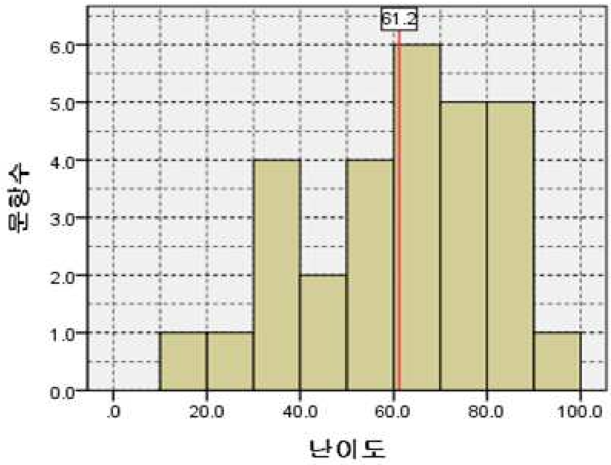

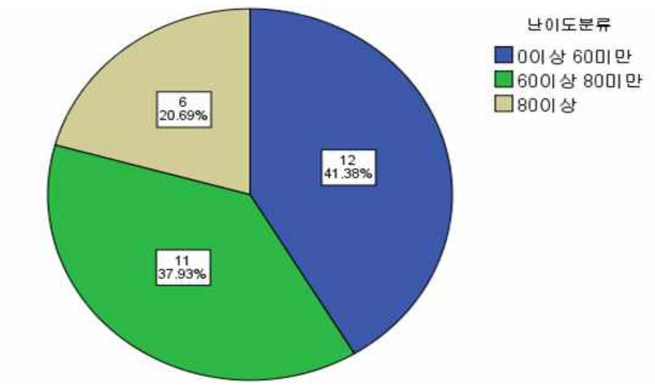

총점	난이도	표준편차
29	61.2	19.5

난이도	문항수	비율(%)
0~60미만	12	41.4
60~80미만	11	37.9
80~100	6	20.7
전체	29	100.0

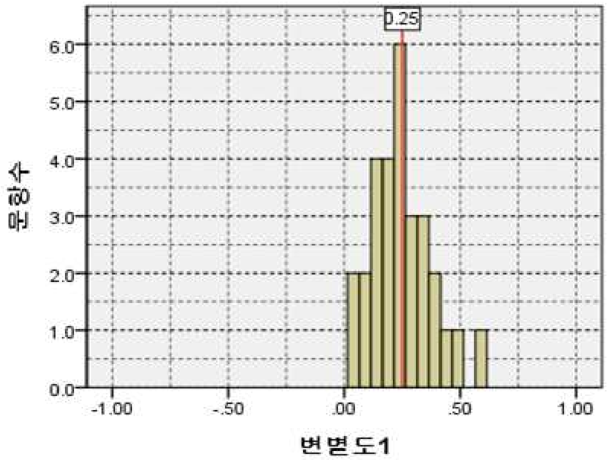

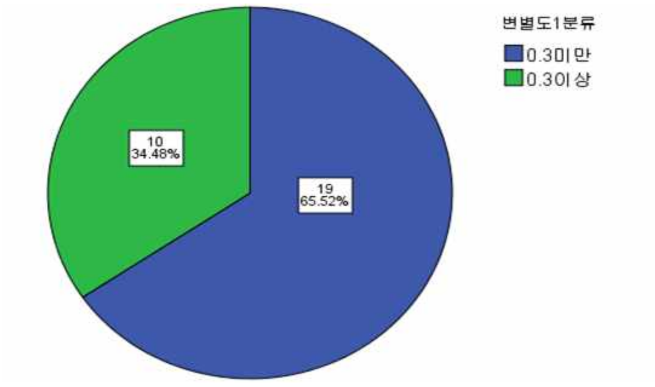

총점	변별도1	표준편차
29	.25	.13

변별도1	문항수	비율(%)
0.3미만	19	65.5
0.3이상	10	34.5
전체	29	100.0

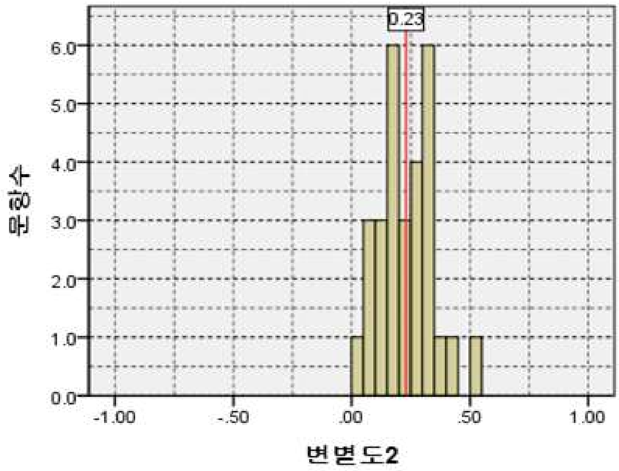

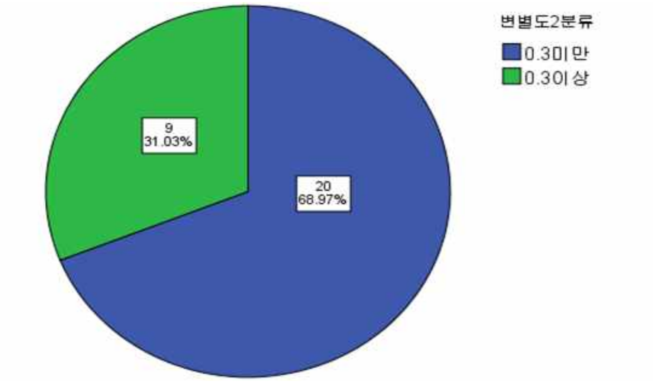

총점	변별도2	표준편차
29	.23	.11

변별도2	문항수	비율(%)
0.3미만	20	69.0
0.3이상	9	31.0
전체	29	100.0

해석

- 암기형 문항에서 난이도 지수가 60 미만인 문항이 전체 29 문항 중 12 문항, 60 이상 80 미만인 문항이 11 문항, 80 에서 100 사이인 문항이 6 문항인 것으로 나타남
- 변별도 1 지수를 기준으로 분류하였을 때, 0.3 미만인 문항이 19 문항으로 0.3 이상인 문항이 10 문항인 것에 비해 더 많이 나타남
- 변별도 2 지수를 기준으로 분류하였을 때, 0.3 미만인 문항이 20 문항으로 0.3 이상인 문항이 9 문항인 것에 비해 더 많이 나타남

(2) 해석형 난이도와 변별도 분포도 및 비율분석

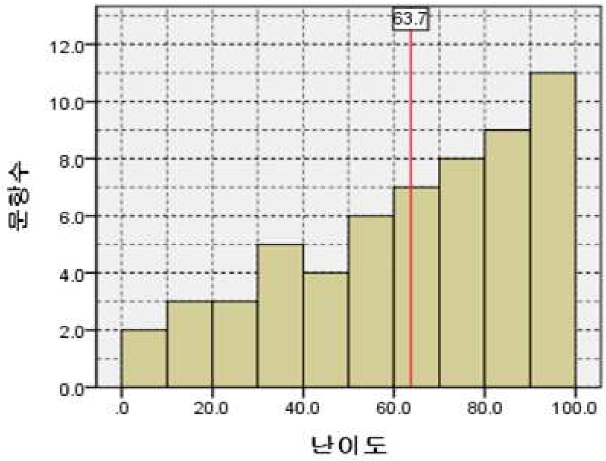

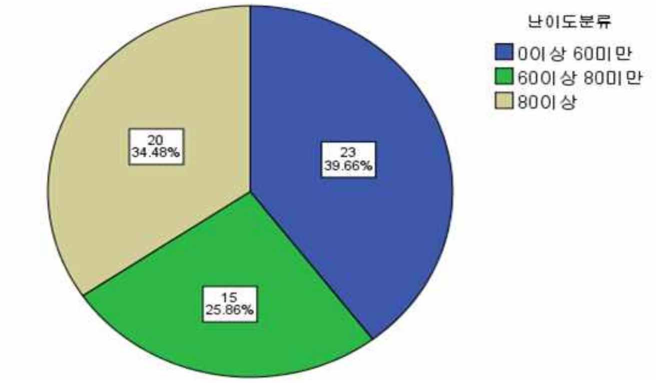

총점	난이도	표준편차
58	63.7	26.2

난이도	문항수	비율(%)
0~60미만	23	39.7
60~80미만	15	25.9
80~100	20	34.5
전체	58	100.0

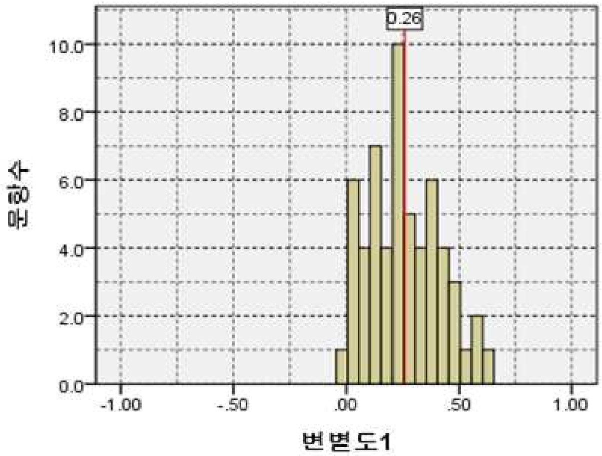

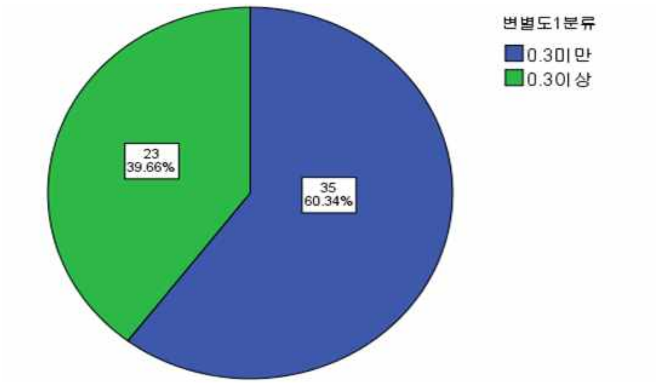

총점	변별도1	표준편차
58	.26	.16

변별도1	문항수	비율(%)
0.3미만	35	60.3
0.3이상	23	39.7
전체	58	100.0

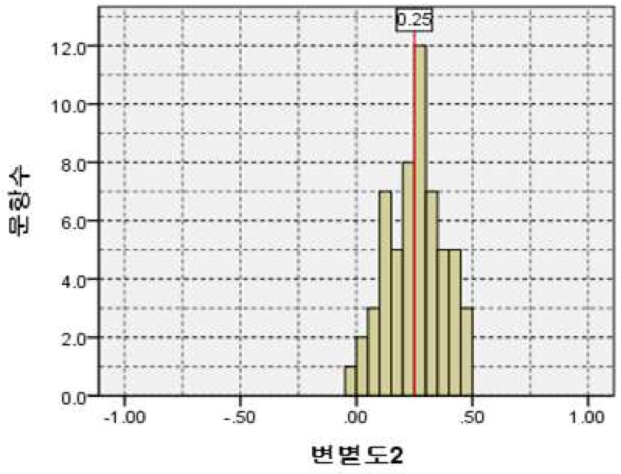

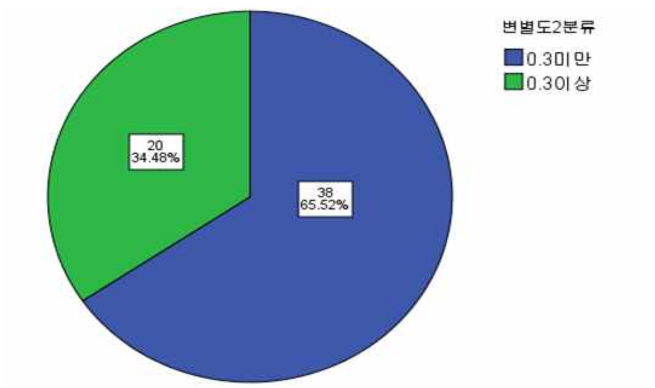

총점	변별도2	표준편차	변별도2	문항수	비율(%)
58	.25	.12	0.3미만	38	65.5
			0.3이상	20	34.5
			전체	58	100.0

해석

- 해석형 문항에서 난이도 지수가 60 미만인 문항이 전체 58 문항 중 23 문항이었으며, 60 이상 80 미만인 문항이 15 문항, 80 에서 100 사이인 문항이 20 문항 순으로 나타남
- 변별도 1 지수를 기준으로 분류하였을 때, 0.3 미만인 문항이 35 문항, 0.3 이상인 문항이 23 문항인 것에 비해 더 많게 나타남
- 변별도 2 지수를 기준으로 분류하였을 때, 0.3 미만인 문항이 38 문항으로 0.3 이상인 문항이 20 문항인 것에 비해 더 많게 나타남

(3) 해결형 난이도와 변별도 분포도 및 비율분석

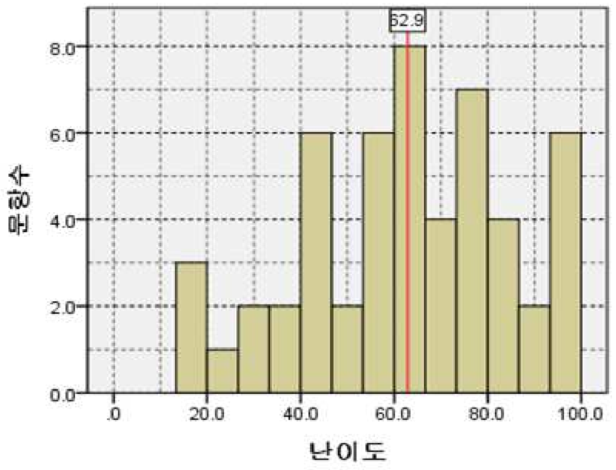

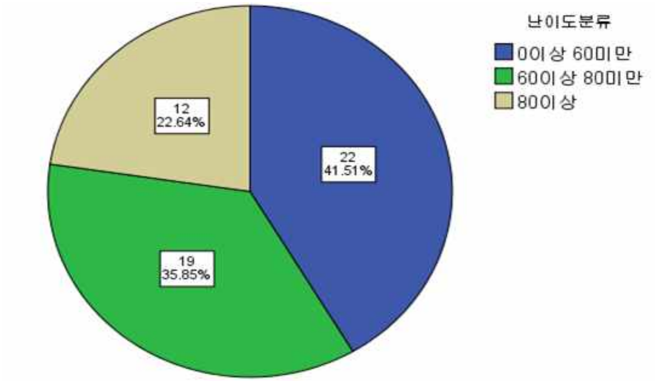

총점	난이도	표준편차
53	62.9	21.7

난이도	문항수	비율(%)
0~60미만	22	41.5
60~80미만	19	35.8
80~100	12	22.6
전체	53	100.0

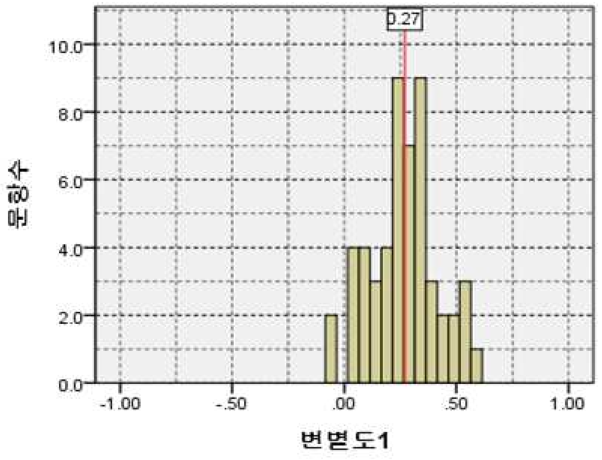

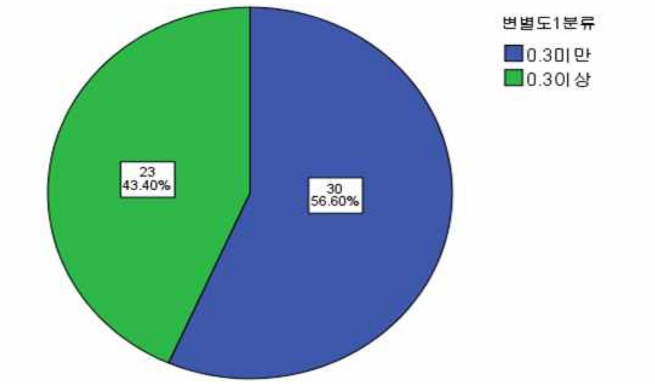

총점	변별도1	표준편차
53	.27	.15

변별도1	문항수	비율(%)
0.3미만	30	56.6
0.3이상	23	43.4
전체	53	100.0

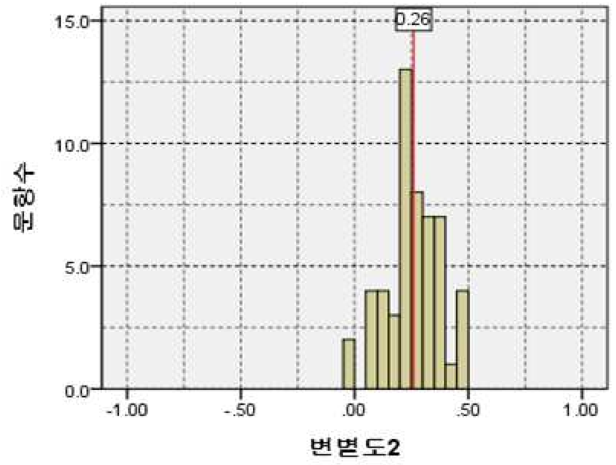

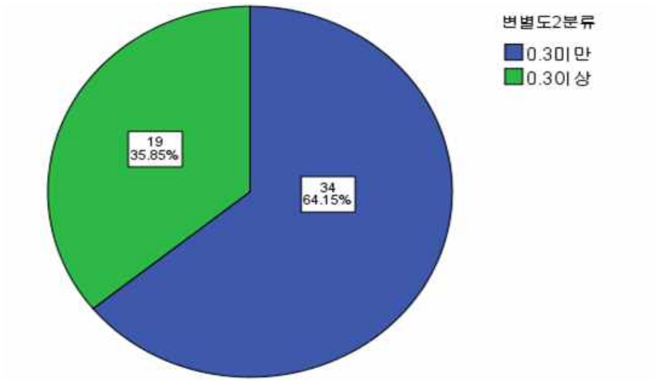

총점	변별도2	표준편차
53	.26	.12

변별도2	문항수	비율(%)
0.3미만	34	64.2
0.3이상	19	35.8
전체	53	100.0

해석

- 해결형 문항에서 난이도 지수가 60 미만이 문항이 전체 53 문항 중 22 문항이었으며, 60 이상 80 미만인 문항이 19 문항, 80 에서 100 사이인 문항이 12 문항인 것으로 나타남
- 변별도 1 지수를 기준으로 분류하였을 때, 0.3 미만인 문항이 30 문항으로 0.3 이상인 문항이 23 문항인 것에 비해 더 많게 나타남
- 변별도 2 지수를 기준으로 분류하였을 때, 0.3 미만인 문항이 34 문항으로 0.3 이상인 문항이 19 문항인 것에 비해 더 많게 나타남

4) 자료유형별 난이도와 변별도

가) 전회 대비 자료유형별 난이도와 변별도

(1) 전회 대비 텍스트형 난이도와 변별도

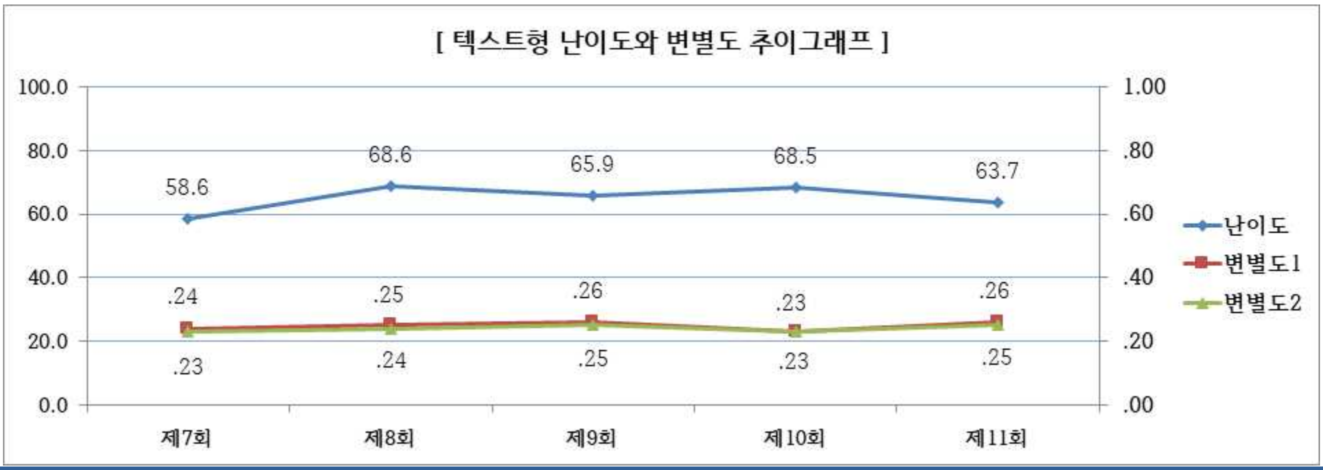

회차	난이도		변별도1		변별도2	
	평균	표준편차	평균	표준편차	평균	표준편차
제7회	58.6	23.8	.24	.14	.23	.11
제8회	68.6	22.0	.25	.14	.24	.11
제9회	65.9	22.7	.26	.17	.25	.12
제10회	68.5	22.9	.23	.14	.23	.11
**제11회**	**63.7**	**22.6**	**.26**	**.15**	**.25**	**.12**

(2) 전회 대비 자료제시형 난이도와 변별도

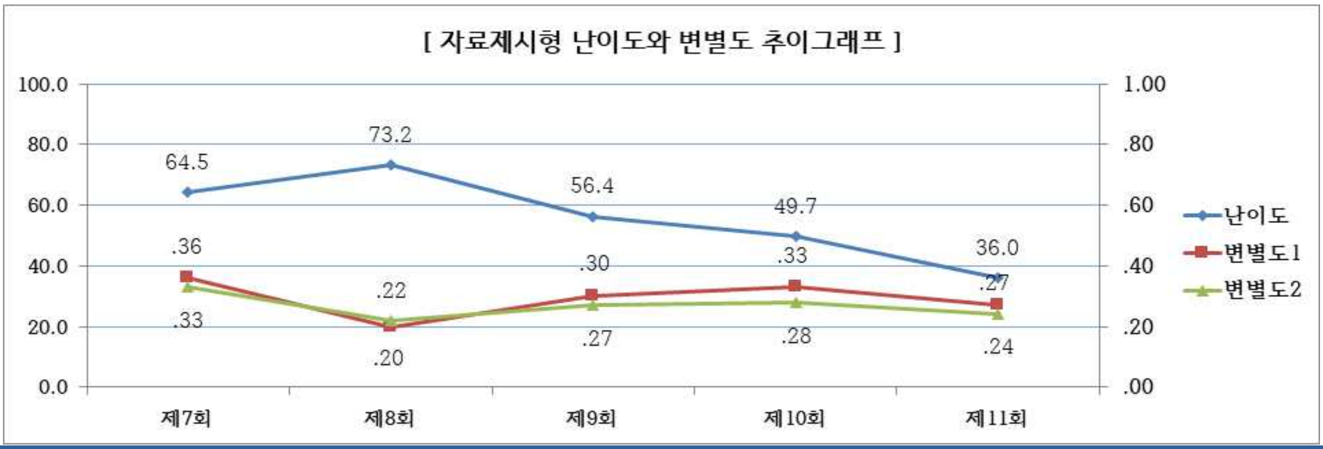

회차	난이도		변별도1		변별도2	
	평균	표준편차	평균	표준편차	평균	표준편차
제7회	64.5	5.9	.36	.06	.33	.09
제8회	73.2	29.2	.20	.14	.22	.09
제9회	56.4	19.6	.30	.15	.27	.13
제10회	49.7	24.6	.33	.21	.28	.15
**제11회**	**36.0**	**27.4**	**.27**	**.20**	**.24**	**.18**

해석	
	- 전회 대비 텍스트형 문항과 자료제시형 문항의 난이도 지수는 각각 4.8, 13.7 감소함<br>- 변별도 1 지수의 경우 텍스트형 문항에서 0.03 증가하였으며, 자료제시형 문항에서 0.06 감소함<br>- 변별도 2 지수의 경우 텍스트형 문항에서 0.02 증가하였으며, 자료제시형 문항에서 0.04 감소함

나) 자료유형별 난이도와 변별도 분포도 및 비율분석

(1) 텍스트형 난이도와 변별도 분포도 및 비율분석

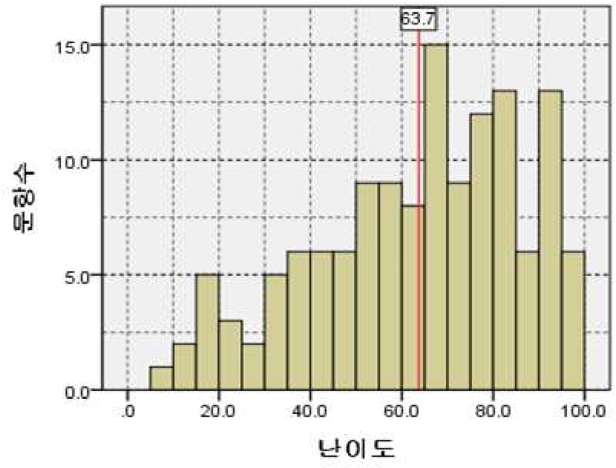

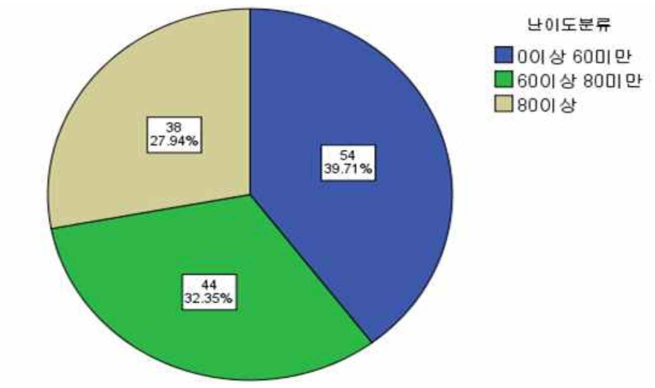

총점	난이도	표준편차	난이도	문항수	비율(%)
136	63.7	22.6	0~60미만	54	39.7
			60~80미만	44	32.4
			80~100	38	27.9
			전체	136	100.0

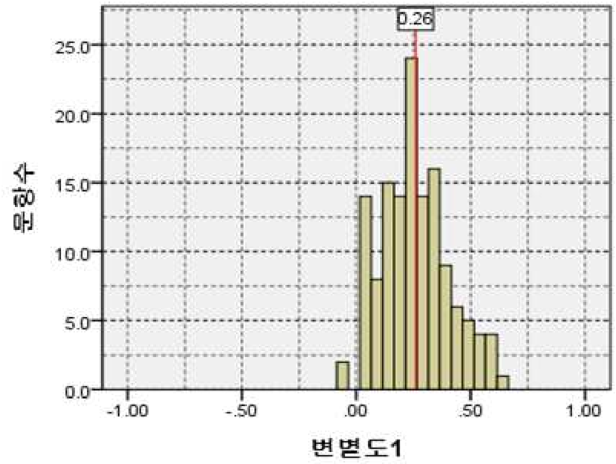

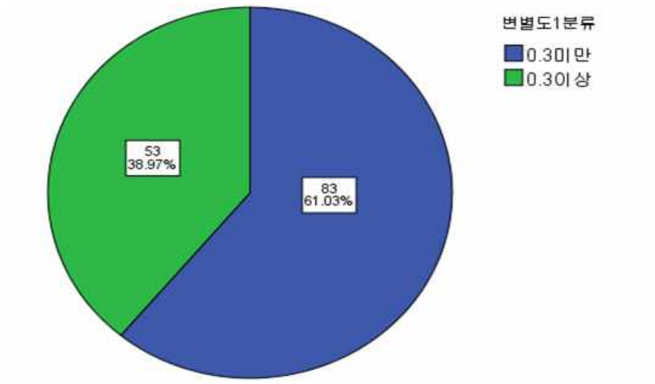

총점	변별도1	표준편차	변별도1	문항수	비율(%)
136	.26	.15	0.3미만	83	61.0
			0.3이상	53	39.0
			전체	136	100.0

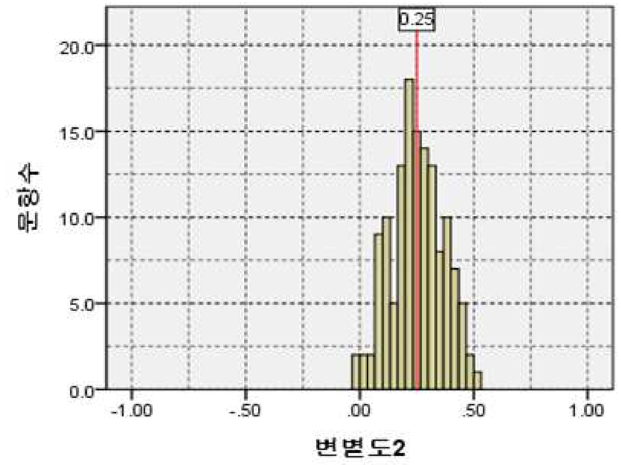

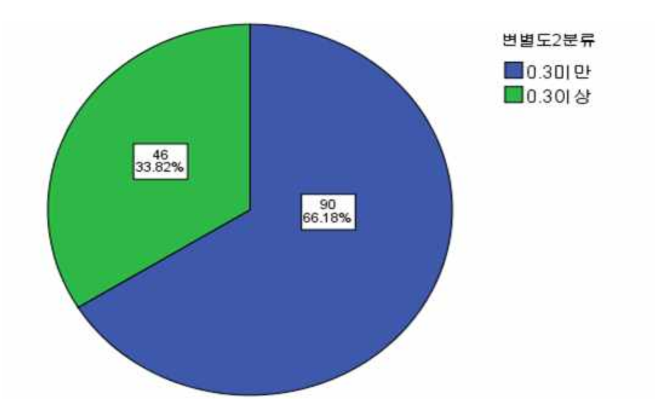

총점	변별도2	표준편차
136	.25	.12

변별도2	문항수	비율(%)
0.3미만	90	66.2
0.3이상	46	33.8
전체	136	100.0

해석

- 텍스트형 문항에서 난이도 지수가 60 미만인 문항이 전체 136 문항 중 54 문항이었으며, 60 이상 80 미만인 문항이 44 문항, 80 에서 100 사이인 문항이 38 문항인 것으로 나타남
- 변별도 1 지수를 기준으로 분류하였을 때, 0.3 미만인 문항이 83 문항으로 0.3 이상인 문항이 53 문항인 것에 비해 더 많게 나타남
- 변별도 2 지수를 기준으로 분류하였을 때, 0.3 미만인 문항이 90 문항으로 0.3 이상인 문항이 46 문항인 것에 비해 더 많게 나타남

(2) 자료제시형 난이도와 변별도 분포도 및 비율분석

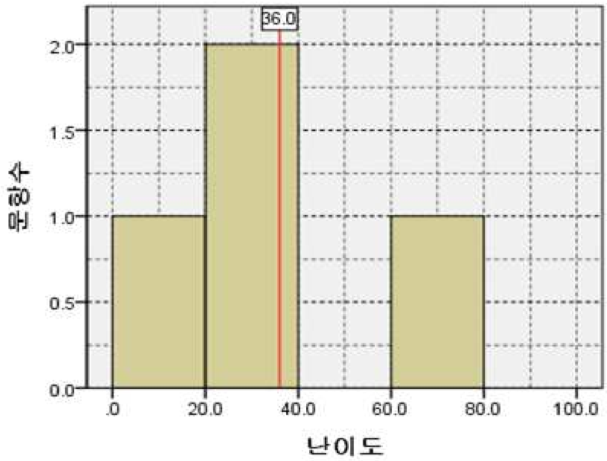

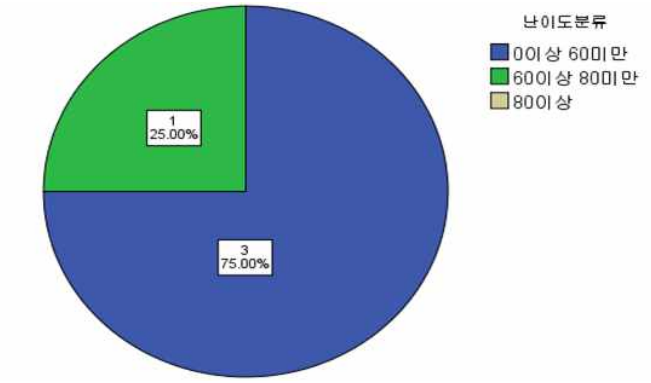

총점	난이도	표준편차	난이도	문항수	비율(%)
4	36.0	27.4	0~60미만	3	75.0
			60~80미만	1	25.0
			80~100	-	-
			전체	4	100.0

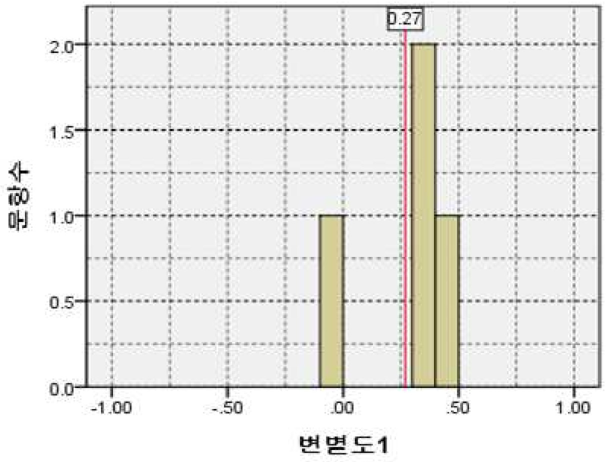

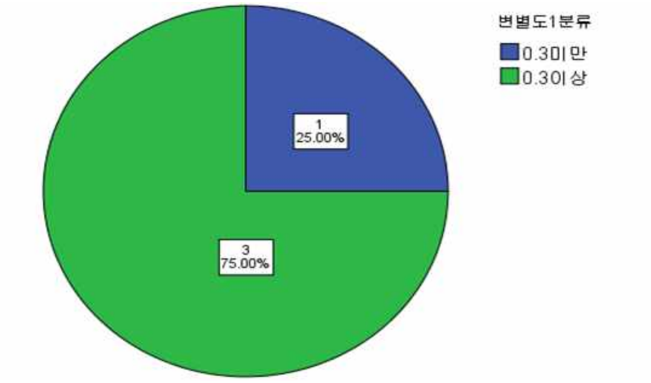

총점	변별도1	표준편차	변별도1	문항수	비율(%)
4	.27	.20	0.3미만	1	25.0
			0.3이상	3	75.0
			전체	4	100.0

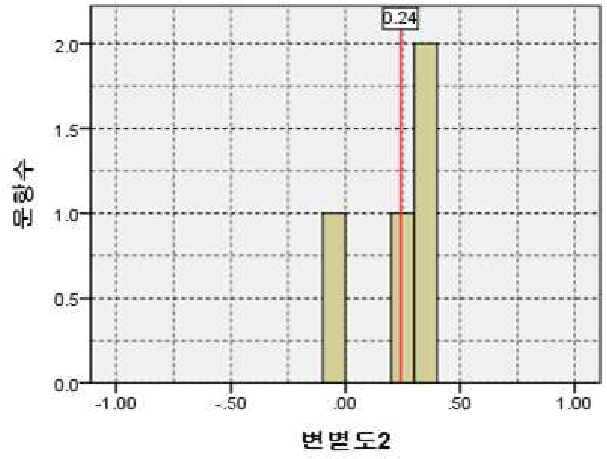

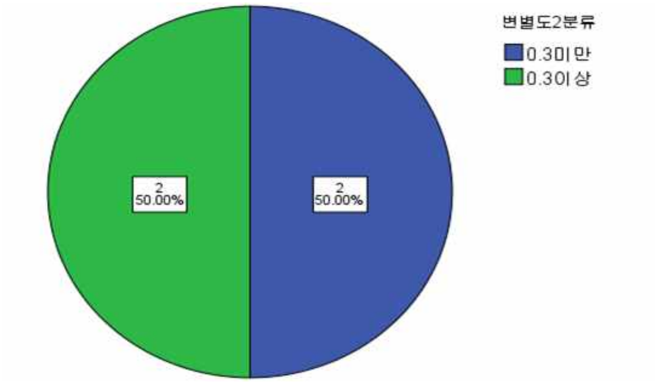

총점	변별도2	표준편차
4	.24	.18

변별도2	문항수	비율(%)
0.3미만	2	50.0
0.3이상	2	50.0
전체	4	100.0

해석

- 자료제시 문항은 난이도 지수가 60 미만인 문항이 전체 4 문항 중 3 문항, 60 이상 80 미만인 문항이 1 문항인 것으로 나타남
- 변별도 1 지수를 기준으로 분류하였을 때, 0.3 미만인 문항이 1 문항으로 0.3 이상인 문항이 3 문항으로 더 적게 나타남
- 변별도 2 지수를 기준으로 분류하였을 때, 0.3 미만인 문항이 2 문항으로 0.3 이상인 문항이 2 문항인 것으로 나타남

3. 난이도와 변별도 간 산포도

1) 전체 난이도와 변별도 간 산포도

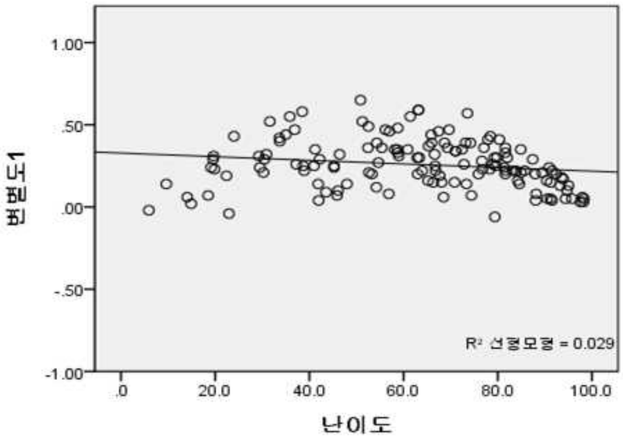

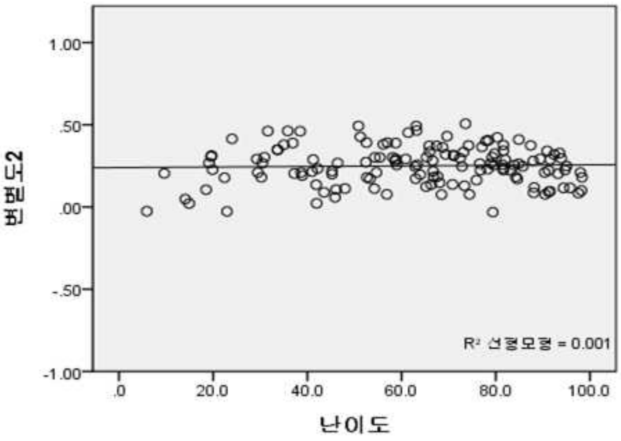

해석

- 난이도와 변별도 1 지수 간 상관은 -.171*로 난이도 지수가 높을수록 변별력이 낮아지는 것으로 나타남
- 난이도와 변별도 2 지수 간 상관은 .033 로 난이도와 변별력 간 관련성이 없는 것으로 나타남

2) 과목별 난이도와 변별도 간 산포도

가) 신경언어장애 난이도와 변별도 간 산포도

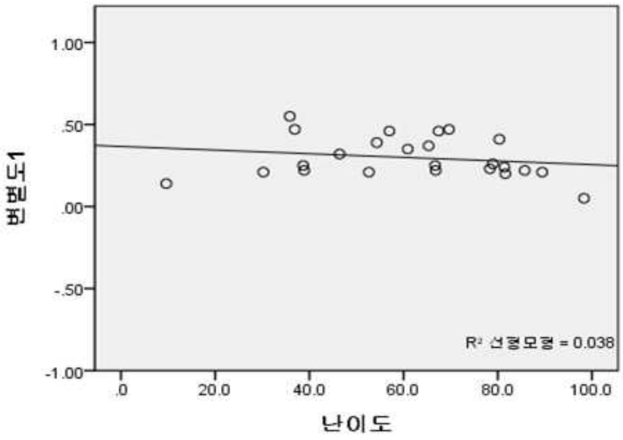

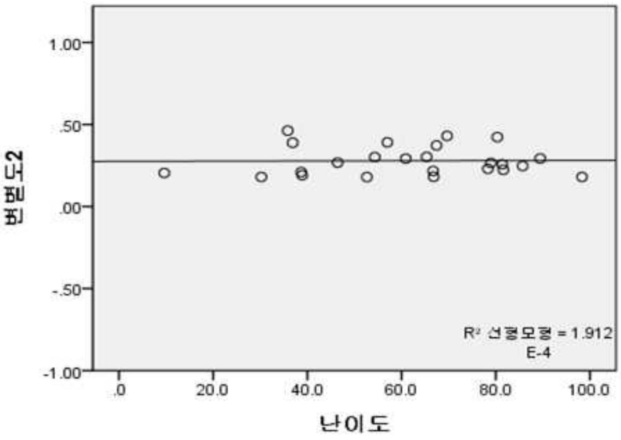

해석

- 난이도와 변별도 1 지수 간 상관은 -.195 로 난이도와 변별력 간 관련성이 낮은 것으로 나타남
- 난이도와 변별도 2 지수 간 상관은 .014 로 난이도와 변별력 간 관련성이 없는 것으로 나타남

나) 언어발달장애 난이도와 변별도 간 산포도

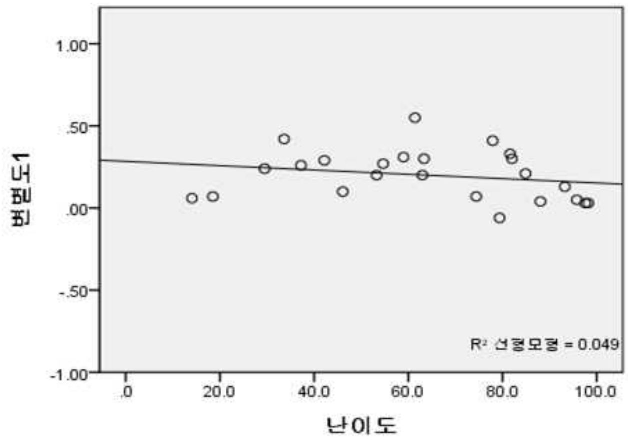

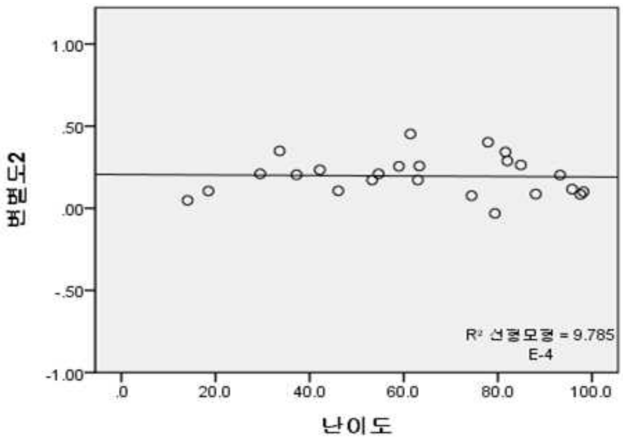

해석

- 난이도와 변별도 1 지수 간 상관은 -.221 로 난이도와 변별력 간 관련성이 낮은 것으로 나타남
- 난이도와 변별도 2 지수 간 상관은 -.031 로 난이도와 변별력 간 관련성이 없는 것으로 나타남

다) 유창성장애 난이도와 변별도 간 산포도

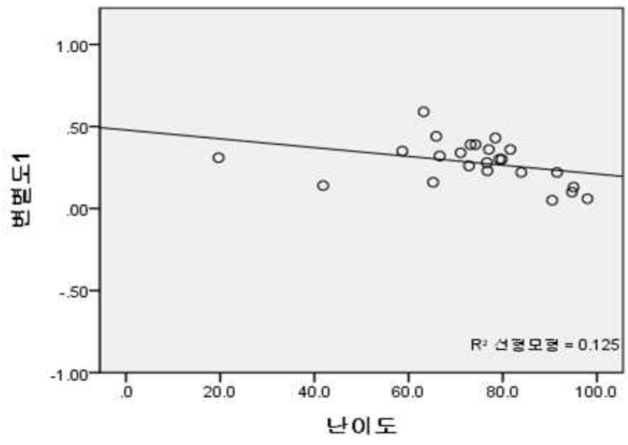

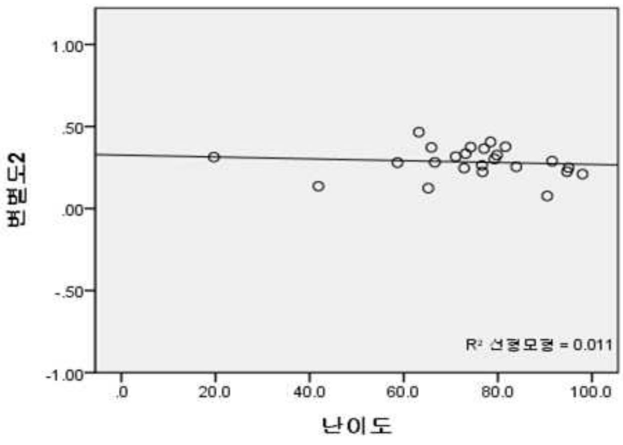

해석

- 난이도와 변별도 1 지수 간 상관은 -.353 으로 난이도와 변별력 간 관련성이 낮은 것으로 나타남
- 난이도와 변별도 2 지수 간 상관은 -.107 로 난이도와 변별력 간 관련성이 낮은 것으로 나타남

라) 음성장애 난이도와 변별도 간 산포도

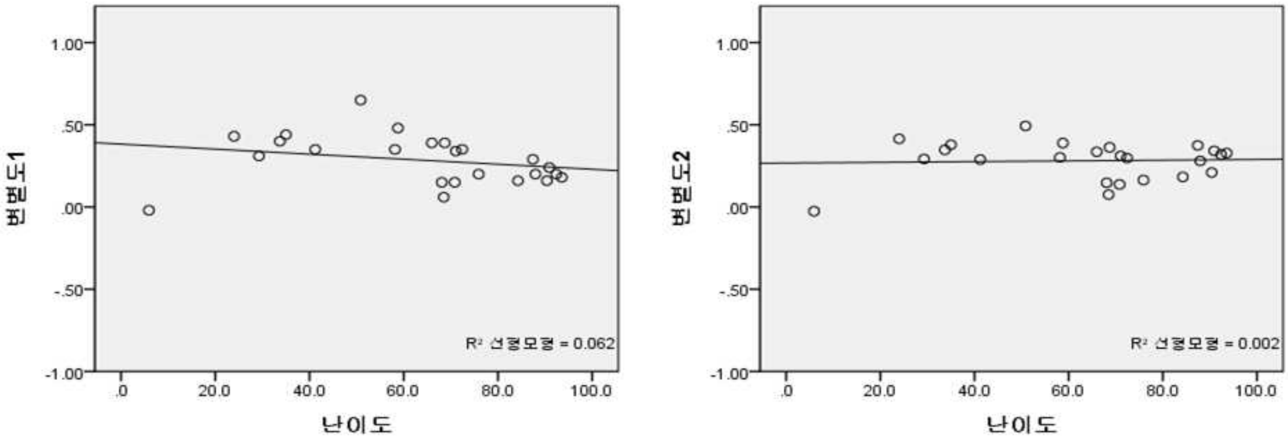

해석

- 난이도와 변별도 1 지수 간 상관은 -.250 으로 난이도와 변별력 간 관련성이 낮은 것으로 나타남
- 난이도와 변별도 2 지수 간 상관은 .045 로 난이도와 변별력 간 관련성이 없는 것으로 나타남

마) 조음음운장애 난이도와 변별도 간 산포도

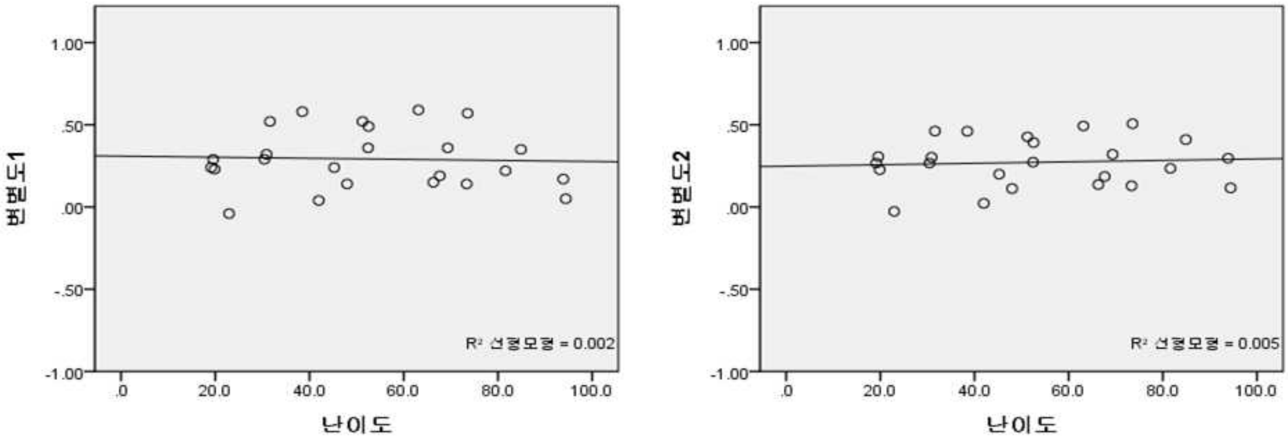

해석

- 난이도와 변별도1 지수 간 상관은 -.043로 난이도와 변별력 간 관련성이 없는 것으로 나타남
- 난이도와 변별도2 지수 간 상관은 .071로 난이도와 변별력 간 관련성이 없는 것으로 나타남

바) 언어재활현장실무 난이도와 변별도 간 산포도

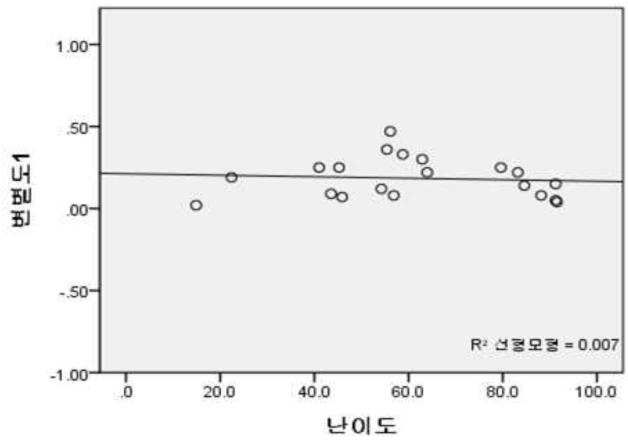

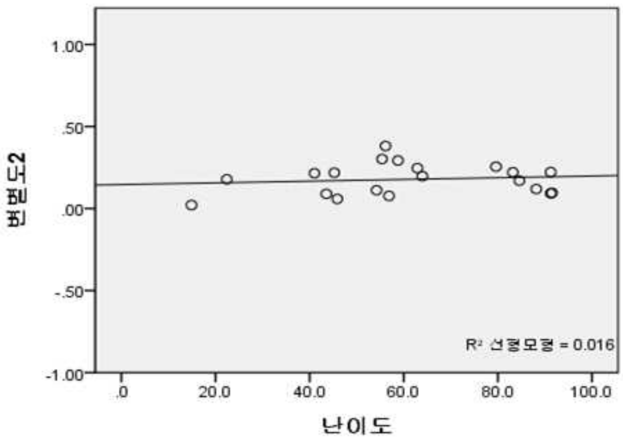

해석

- 난이도와 변별도1 지수 간 상관은 -.086으로 난이도와 변별력 간 관련성이 없는 것으로 나타남
- 난이도와 변별도2 지수 간 상관은 .126로 난이도와 변별력 간 관련성이 낮은 것으로 나타남

4. 신뢰도 분석

과목명	문항수	제7회	제8회	제9회	제10회	제11회
전체	140	.874	.895	.898	.879	.893
신경언어장애	24	.556	.659	.555	.551	.670
언어발달장애	24	.536	.584	.601	.436	.509
유창성장애	24	.593	.678	.654	.595	.684
음성장애	24	.686	.625	.757	.670	.684
조음음운장애	24	.580	.675	.695	.697	.632
언어재활현장실무	20	.389	.285	.260	.295	.377

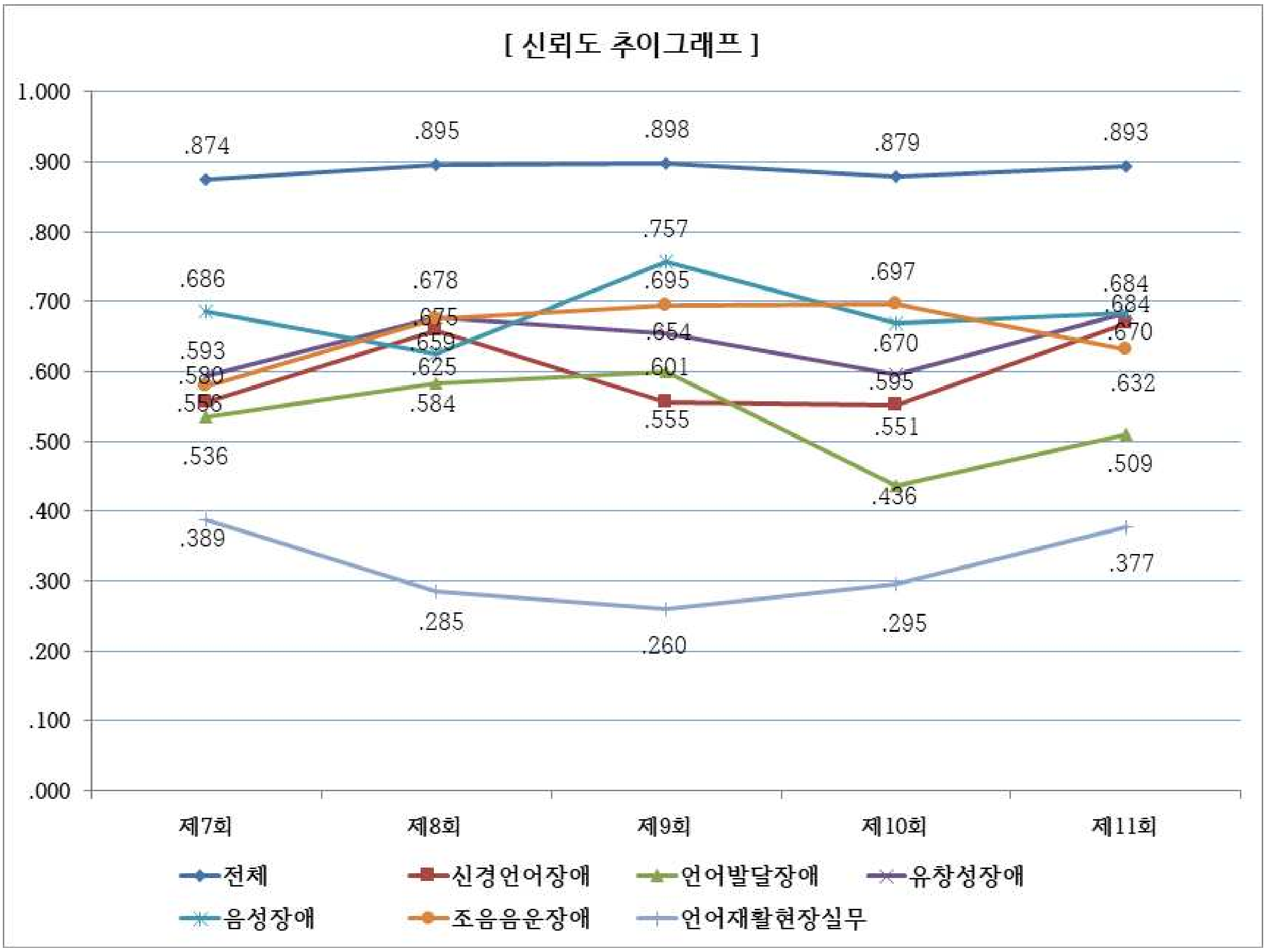

해석

- 1급 언어재활사 국가시험 전체 신뢰도는 작년대비 0.14 증가하였으며, 과목에 따라 신뢰도의 차이가 있는 것으로 나타남
- 전회 대비 신뢰도는 신경언어장애, 언어발달장애, 유창성장애, 음성장애, 언어재활현장실무 과목 문항을 대상으로 했을 때 각각 .119, .073, .089, .014, .082 증가함.
- 언어재활현장실무 과목 문항을 대상으로 하는 신뢰도는 전회 대비 다소 증가하였으나, 전체적으로 낮은 신뢰도를 보이고 있음
- 조음음운장애 과목 문항을 대상으로 했을 때는 각각 .065 감소함
