# Supplementary material for: Item difficulty index, discrimination index, and reliability of the 26 health professions licensing examinations in 2022, Korea: a psychometric study
Source: J Educ Eval Health Prof. 2023 Nov 22;20:31. doi: 10.3352/jeehp.2023.20.31 (PMC11959405; doi:10.3352/jeehp.2023.20.31)
Supplement: Supplementary file 1 — Supplement 1. Item analysis results of 26 health professions licensing examinations administered during late 2022 and early 2023. [file jeehp-20-31_Suppl1.zip › 2022│Γ╡╡ ┴a13╚╕ ║╕░╟▒│└░╗τ 2▒▐ ▒╣░í╜├╟Φ ║╨╝«░ß░·.pdf]

2022년도 제13회
보건교육사 2급 국가시험
문항분석 결과

국민이 신뢰하고 감동하는 시험평가기관
한국보건의료인국가시험원
KOREA HEALTH PERSONNEL LICENSING EXAMINATION INSTITUTE

일반 용어 정의

□ **평균**
- 집단에서의 대표적 경향값으로 전체 값을 더하여 총 응시자로 나눈 값

□ **표준편차**
- 평균과 각 점수의 차이인 편차들의 평균으로 점수가 흩어져 분포되어 있는 정도

□ **추정난이도**
- 문항개발자가 예측한 정답률

□ **검사이론**
- 검사와 검사를 구성하고 있는 문항의 양호도를 분석 및 평가하는 방법을 정의한 이론체계
- 대표적으로 고전검사이론과 문항반응이론이 있음

고전검사이론 용어 정의

□ 고전검사이론(Classical Test Theory; CTT)

- 검사의 질을 분석하는 검사이론 중 한 가지로 19세기 말부터 전개되어 현재까지 주로 사용되고 있는 검사이론임
- 고전검사이론에 의한 문항과 응시자 능력 추정치는 다음과 같음

○ **문항난이도**

· 검사 문항의 쉽고 어려운 정도를 나타내는 지수
· 난이도 지수는 총 반응 수에 대한 정답 반응 수의 비율로 문항의 정답률임
· 문항난이도는 0~100까지의 값을 가짐
· 난이도 값이 큰 경우, 쉬운 문항으로 '난이도가 낮다'라고 해석하며, 난이도 값이 작은 경우, 어려운 문항으로 '난이도가 높다'라고 해석함

○ **문항변별도**

· 각 문항이 응시자의 능력 수준을 변별할 수 있는 정도를 나타내는 지수
· 문항변별도는 −1~+1까지의 값을 가지며, 1에 가까울수록 변별력 크다고 해석함
· 일반적으로 문항변별도가 0.3 이상이면 우수한 문항으로 평가함
· 구하는 방식에는 '상하위집단 구분법', '문항-총점 상관계수' 등이 있음
  1) **변별도 1(상하위구분법):** 상위 27%와 하위 27% 집단의 난이도 차이를 구하는 방식
  2) **변별도 2(상관계수법):** 문항-총점과의 상관계수로 구하는 방식

○ **신뢰도**

· 시험이 평가하고자 하는 것을 일관성 있게 측정하는가로 시험이 오차없이 정확하게 측정한 정도를 의미함
· 국시원에서는 문항의 내적일관성(Cronbach α)으로 신뢰도를 추정하며 1에 가까울수록 신뢰도가 높다고 해석함

목 차

I. 시행 결과 ······ 6

1. 시험 현황 ······ 7

1) 시험명 ······ 7

2) 시험시행일 ······ 7

3) 응시현황 ······ 7

4) 과목별 문항 수, 배점 및 과락 점수 ······ 7

2. 합격률과 평균성적 ······ 7

1) 합격 및 불합격 현황 ······ 7

2) 과목별 과락자수 내역 ······ 7

3) 전회 대비 합격률과 평균성적 ······ 8

II. 문항분석 결과 ······ 9

1. 성적 ······ 10

1) 전체 성적분포도 ······ 10

2) 과목별 성적분포도 ······ 11

2. 난이도와 변별도 ······ 13

1) 전체 난이도와 변별도 ······ 13

2) 과목별 난이도와 변별도 ······ 16

3) 지식수준별 난이도와 변별도 ······ 37

3. 난이도와 변별도 간 산포도 ······ 45

1) 전체 난이도와 변별도 간 산포도 ······ 45

2) 과목별 난이도와 변별도 간 산포도 ······ 45

4. 신뢰도 분석 ······ 50

Ⅰ. 시행 결과

1. 시험 현황

1) 시험명: 2022년도 제12회 보건교육사 2급 국가시험

2) 시험시행일: 2022년 2월 12일

3) 응시현황

응시대상자수	결시자수	부정행위자수	응시자 준수사항 위반자 수		응시자수 (%)
			휴대폰 소지	신분증 미지참	
153	42	-	-	-	111 (72.5)

4) 과목별 문항 수, 배점 및 과락 점수

교 시	과 목 명	문제수	배점	총점	합격자 점수기준	
					과목 과락기준	총점 합격기준
1교시	보건프로그램 개발 및 평가	25	1	25	10.0	108.0
	보건교육방법론	20	1	20	8.0	
	보건사업관리	20	1	20	8.0	
	보건의료법규	20	1	20	8.0	
2교시	조사방법론	25	1	25	10.0	
	보건의사소통	25	1	25	10.0	
	보건학	20	1	20	8.0	
	보건교육학	25	1	25	10.0	
계		180		180		

2. 합격률과 평균성적

1) 합격 및 불합격 현황

합격자수 (%)	불합격자수(%)				채점보류자수
	평락	과락	기권	계	
69 (62.2)	41 (36.9)	1 (0.9)	- (0.0)	42 (37.8)	0 (0.0)

2) 과목별 과락자수 내역

과목명 / 과락자수	보건프로그램개발 및 평가	보건교육방법론	보건사업관리	보건의료법규	조사방법론	보건의사소통	보건학	보건교육학
과목별 과락자 수	-	-	-	-	1	-	-	-
전과목 과락자 수	-							

3) 전회 대비 합격률과 평균성적

회차	년도	합격률(%)	평균성적	표준편차	백분율 환산점수
제9회	2017	57.9	112.9	23.6	62.7
제10회	2018	60.9	112.1	18.2	62.3
제11회	2019	47.6	108.4	20.3	60.2
제12회	2021	53.7	109.6	18.7	60.9
**제13회**	**2022**	**62.2**	**112.3**	**19.4**	**62.4**

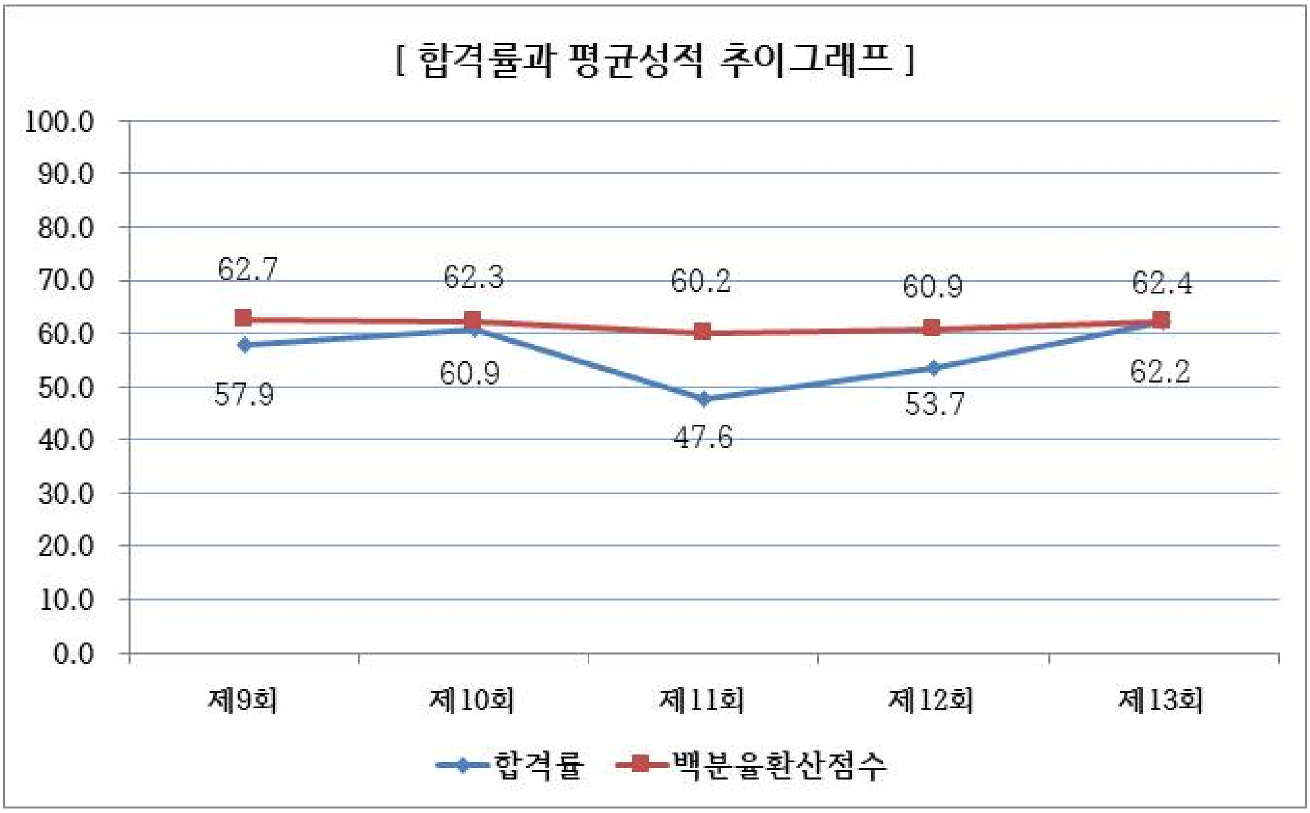

해석

- 전년 대비 합격률은 8.5%, 백분율 환산점수는 1.5 점 증가함
- 표준편차는 0.7 증가함

※ 2021년 이전까지는 응시자 수가 100명 이하로 난이도와 변별도 외 분석은 시행하지 않고 문항분석표만 제공하였으나, 2022년 응시자 수가 100명이 넘어 분석을 시행함.

II. 문항분석 결과

1. 성적

1) 전체 성적분포도

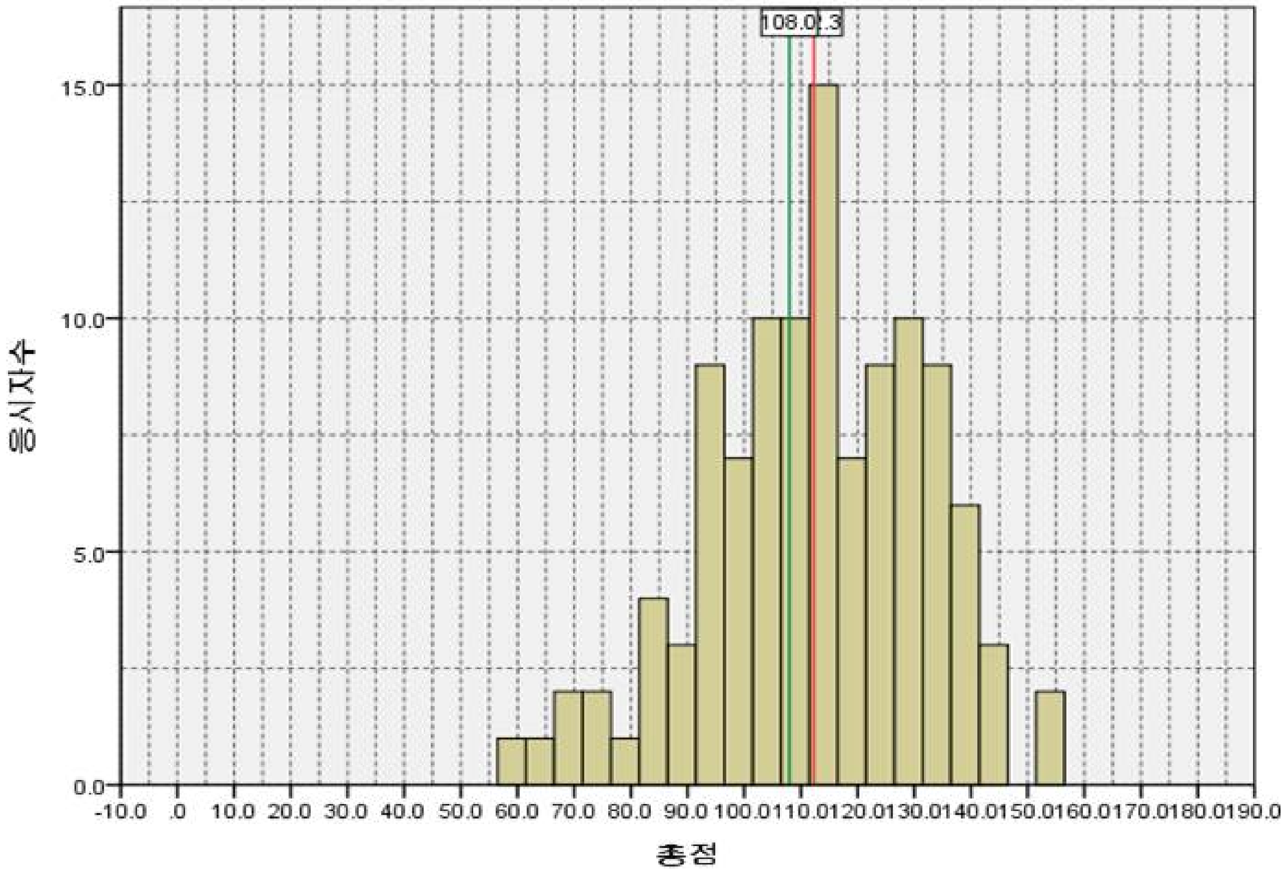

응시자	총점	합격선	평균성적	표준편차
111	180	108	112.3	19.4

2) 과목별 성적분포도

가) 보건프로그램 개발 및 평가

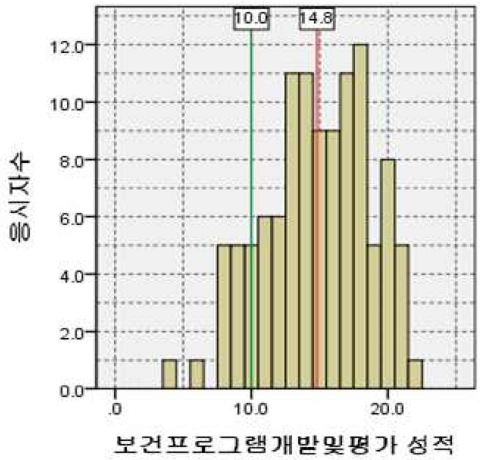

**총점**	과락기준	평균성적	표준편차
25	10	14.8	3.8

나) 보건교육방법론

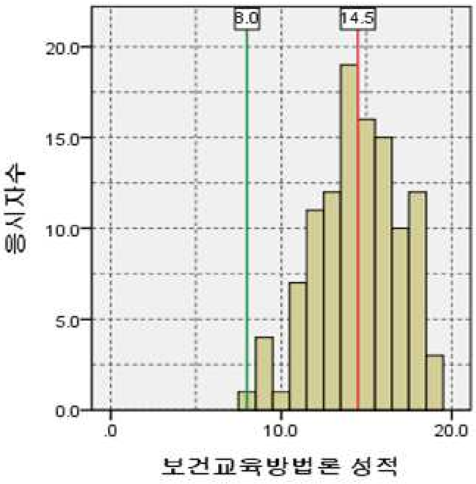

총점	과락기준	평균성적	표준편차
20	8	14.5	2.5

다) 보건사업관리

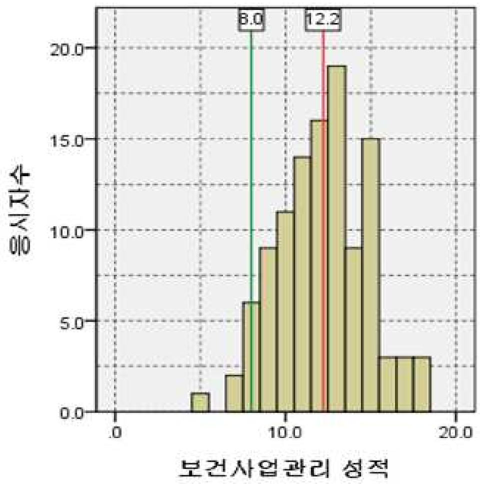

**총점**	과락기준	평균성적	표준편차
20	8	12.2	2.6

라) 보건의료법규

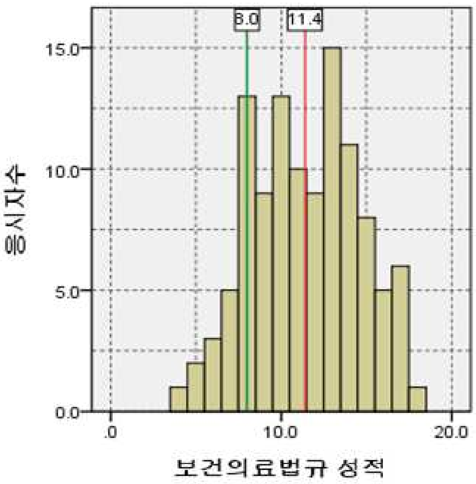

총점	과락기준	평균성적	표준편차
20	8	11.4	3.2

마) 조사방법론

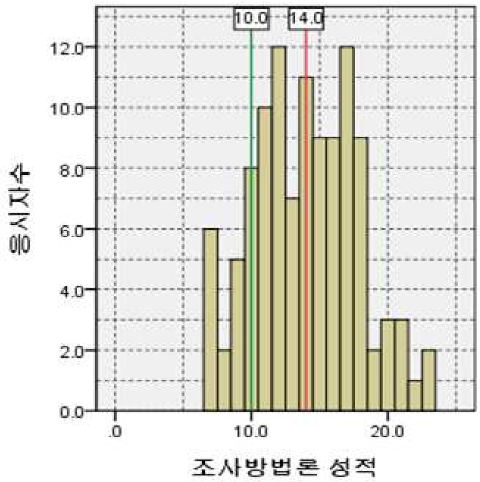

총점	과락기준	평균성적	표준편차
25	10	14.0	3.8

바) 보건의사소통

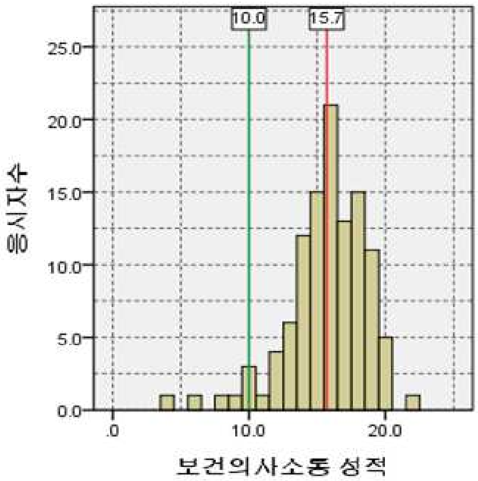

총점	과락기준	평균성적	표준편차
25	10	15.7	2.9

사) 보건학

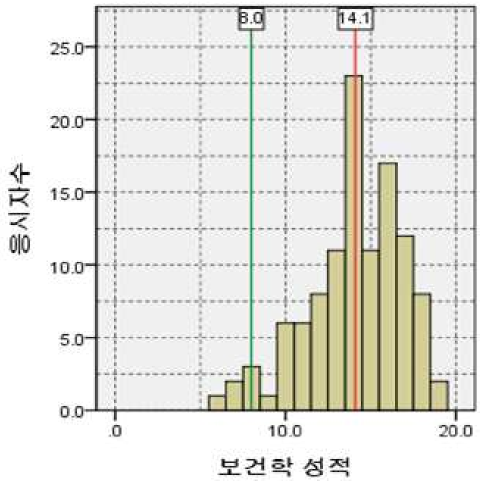

총점	과락기준	평균성적	표준편차
20	8	14.1	2.8

아) 보건교육학

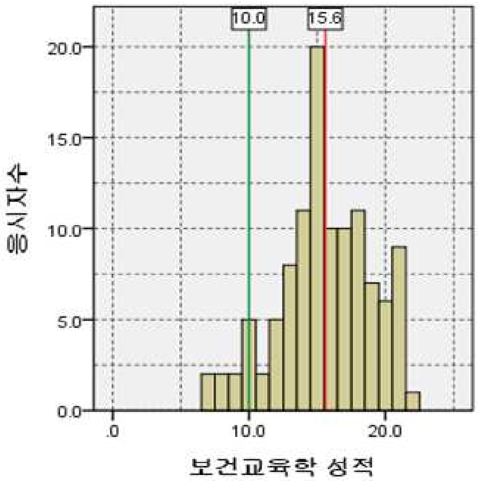

총점	과락기준	평균성적	표준편차
25	10	15.6	3.4

2. 난이도와 변별도

1) 전체 난이도와 변별도

가) 전회 대비 전체 난이도와 변별도

회차	난이도		변별도1		변별도2	
	평균	표준편차	평균	표준편차	평균	표준편차
제9회	62.7	23.0	.32	.25		
제10회	62.3	24.3	.24	.22		
제11회	60.2	23.7	.28	.20		
제12회	60.9	24.1	.25	.21	.24	.17
**제13회**	**62.4**	23.5	**.27**	.17	.25	.13

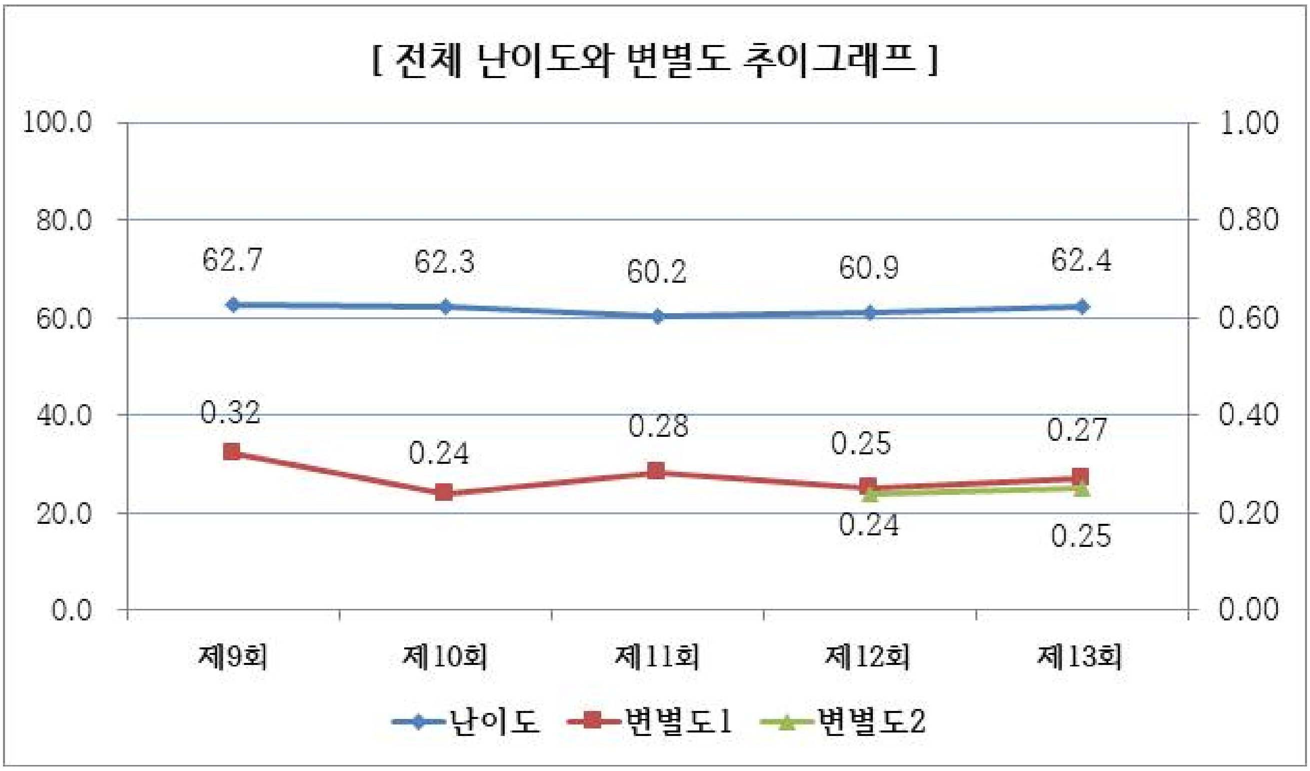

해석

- 전년 대비 난이도 지수는 1.5 증가함
- 변별도 1, 2 지수는 각각 0.02, 0.01 상승함

나) 전체 난이도와 변별도 분포도 및 비율분석

(1) 전체 난이도 분포도 및 비율분석

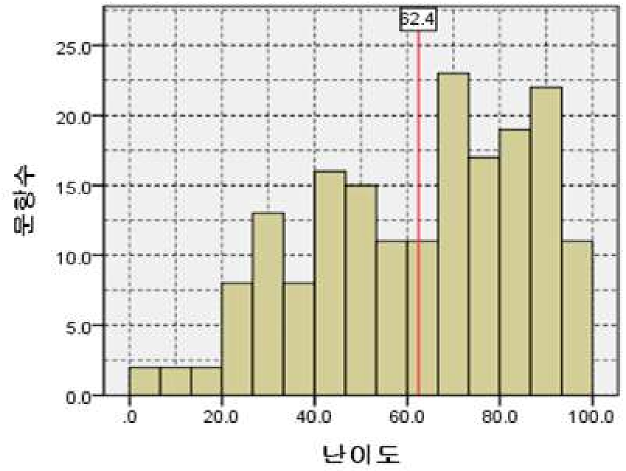

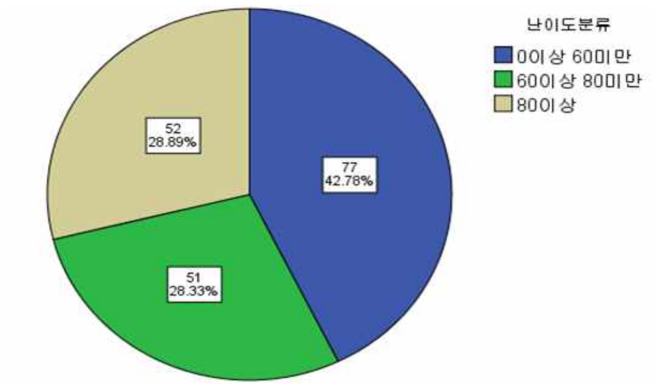

총점	난이도	표준편차
180	62.4	23.5

난이도	문항수	비율(%)
0~60미만	77	42.8
60~80미만	51	28.3
80~100	52	28.9
전체	180	100.0

(2) 전체 변별도1 분포도 및 비율분석

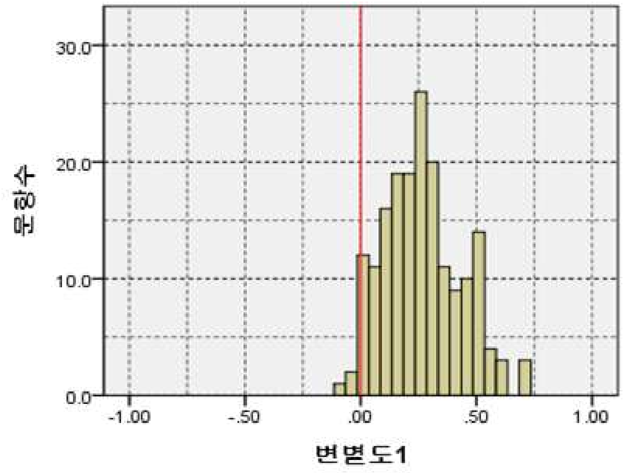

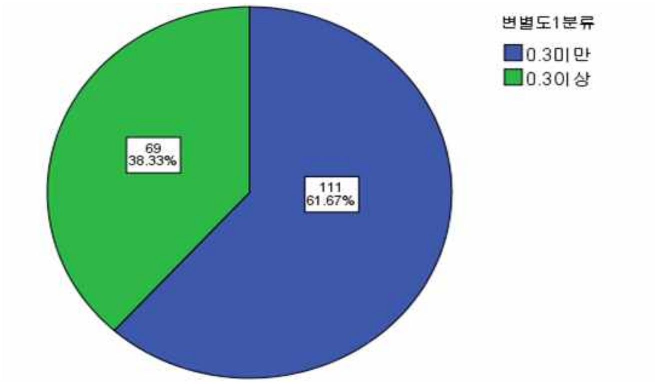

총점	변별도1	표준편차
180	.27	.17

변별도1	문항수	비율(%)
0.3미만	111	61.7
0.3이상	69	38.3
전체	180	100.0

(3) 전체 변별도2 분포도 및 비율분석

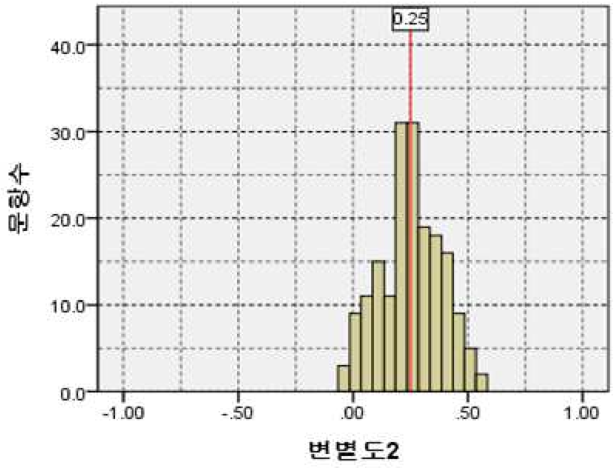

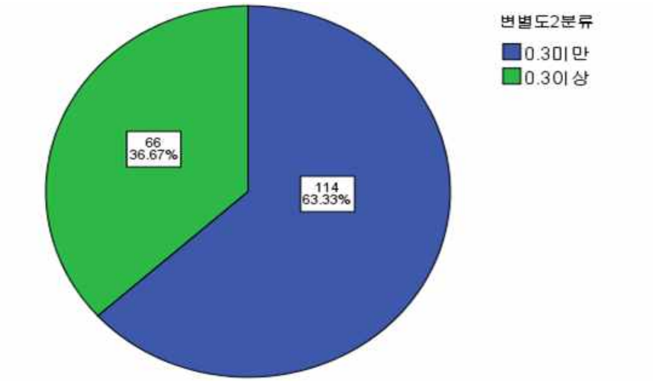

총점	변별도2	표준편차
180	.25	.13

변별도2	문항수	비율(%)
0.3미만	114	63.3
0.3이상	66	36.7
전체	180	100.0

해석

- 난이도 지수가 80에서 100 사이인 문항이 전체 180 중 52문항으로 나타났으며, 60이상 80미만인 문항이 51문항, 60미만인 문항이 77문항인 것으로 나타남
- 변별도1 지수를 기준으로 분류하였을 때, 0.3미만인 문항이 111항으로 0.3이상인 문항이 69문항인 것에 비해 더 많이 나타남
- 변별도2 지수를 기준으로 분류하였을 때, 0.3미만인 문항이 114문항으로 0.3이상인 문항이 66문항인 것에 비해 더 많이 나타남

2) 과목별 난이도와 변별도

가) 전회 대비 과목별 난이도와 변별도

(1) 전회 대비 보건프로그램 개발 및 평가 난이도와 변별도

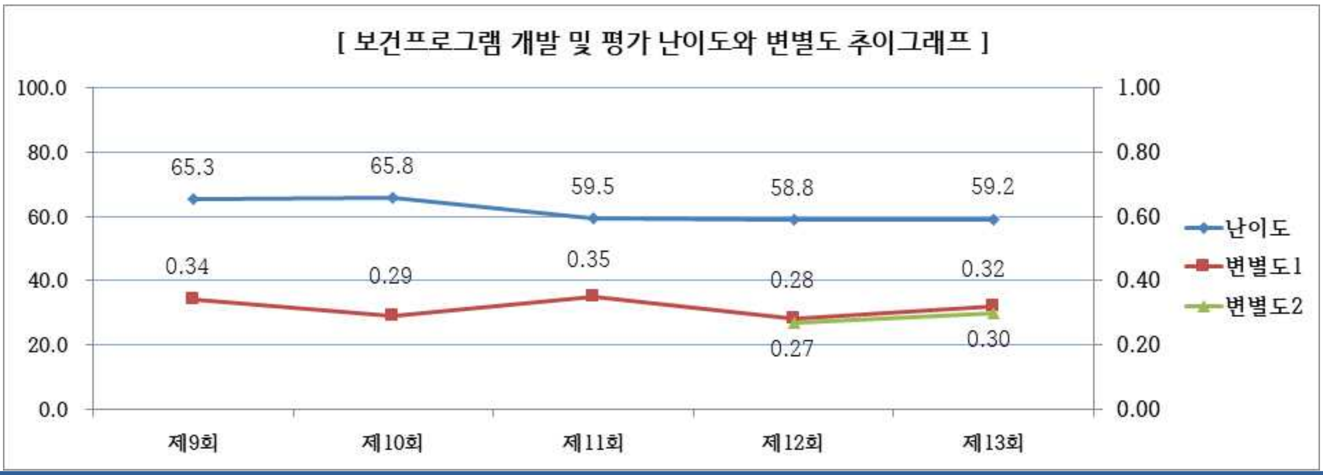

회차	난이도		변별도1		변별도2	
	평균	표준편차	평균	표준편차	평균	표준편차
제9회	65.3	17.5	.34	.20		
제10회	65.8	20.3	.29	.23		
제11회	59.5	15.4	.35	.20		
제12회	58.8	25.4	.28	.24	.27	.20
**제13회**	**59.2**	**22.6**	**.32**	**.16**	**.30**	**.11**

(2) 전회 대비 보건교육방법론 난이도와 변별도

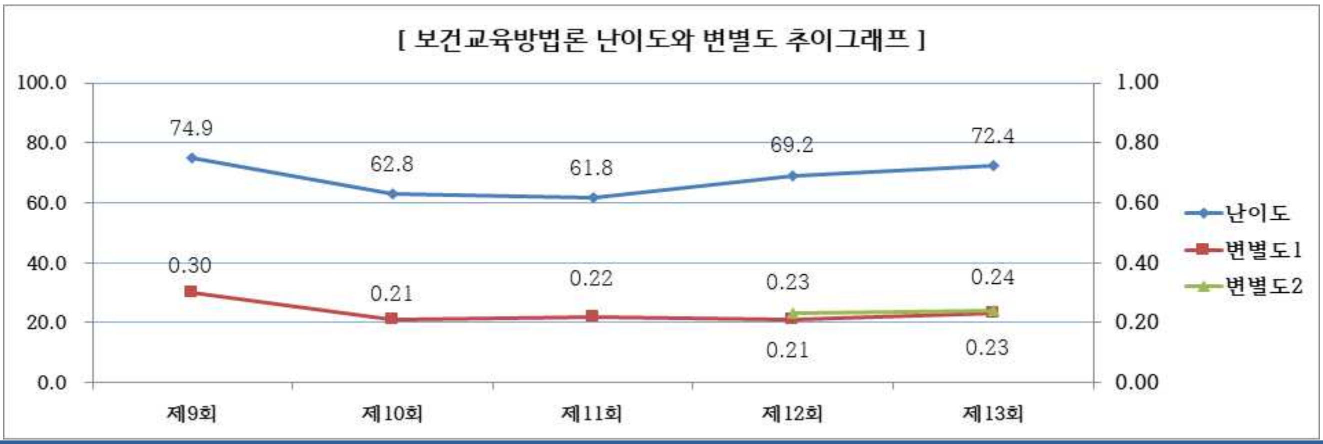

회차	난이도		변별도1		변별도2	
	평균	표준편차	평균	표준편차	평균	표준편차
제9회	74.9	21.7	.30	.31		
제10회	62.8	23.9	.21	.20		
제11회	61.8	26.9	.22	.16		
제12회	69.2	22.3	.21	.24	.23	.20
**제13회**	**72.4**	**21.4**	**.23**	**.18**	**.24**	**.15**

(3) 전회 대비 보건사업관리 난이도와 변별도

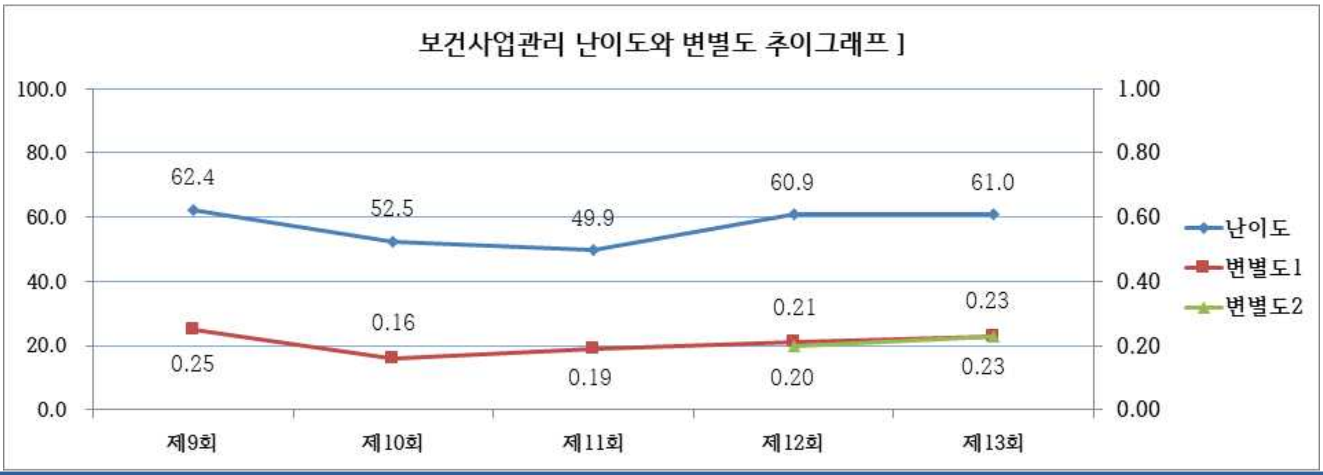

회차	난이도		변별도1		변별도2	
	평균	표준편차	평균	표준편차	평균	표준편차
제9회	62.4	25.0	.25	.22		
제10회	52.5	27.2	.16	.20		
제11회	49.9	28.6	.19	.18		
제12회	60.9	22.1	.21	.22	.20	.18
**제13회**	**61.0**	**27.9**	**.23**	**.15**	**.23**	**.13**

(4) 전회 대비 보건의료법규 난이도와 변별도

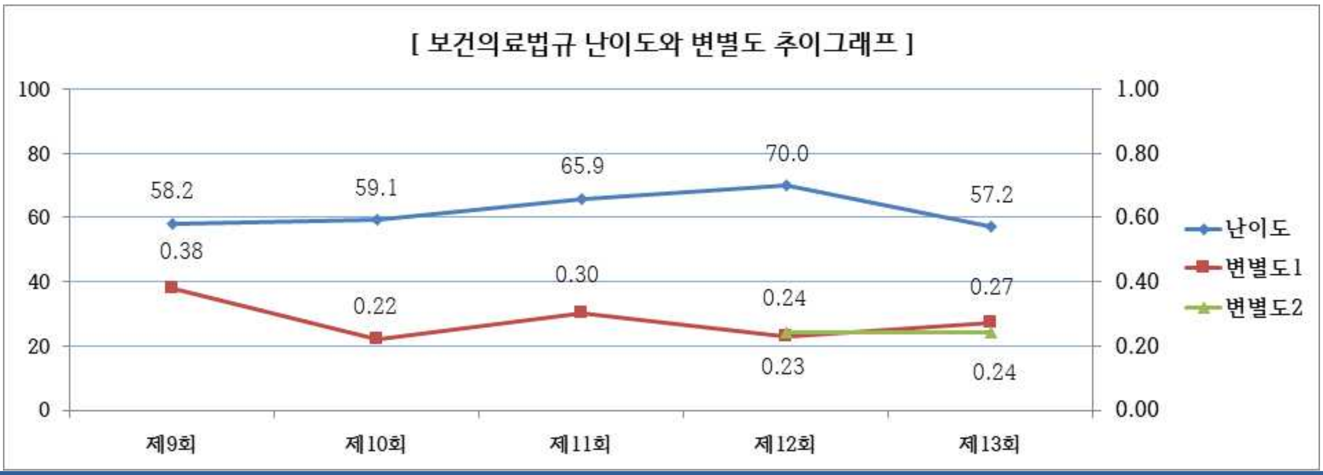

회차	난이도		변별도1		변별도2	
	평균	표준편차	평균	표준편차	평균	표준편차
제9회	58.2	20.4	.38	.22		
제10회	59.1	23.5	.22	.24		
제11회	65.9	20.4	.30	.16		
제12회	70.0	21.1	.23	.17	.24	.12
**제13회**	**57.2**	**20.5**	**.27**	**.19**	**.24**	**.15**

(5) 전회 대비 조사방법론 난이도와 변별도

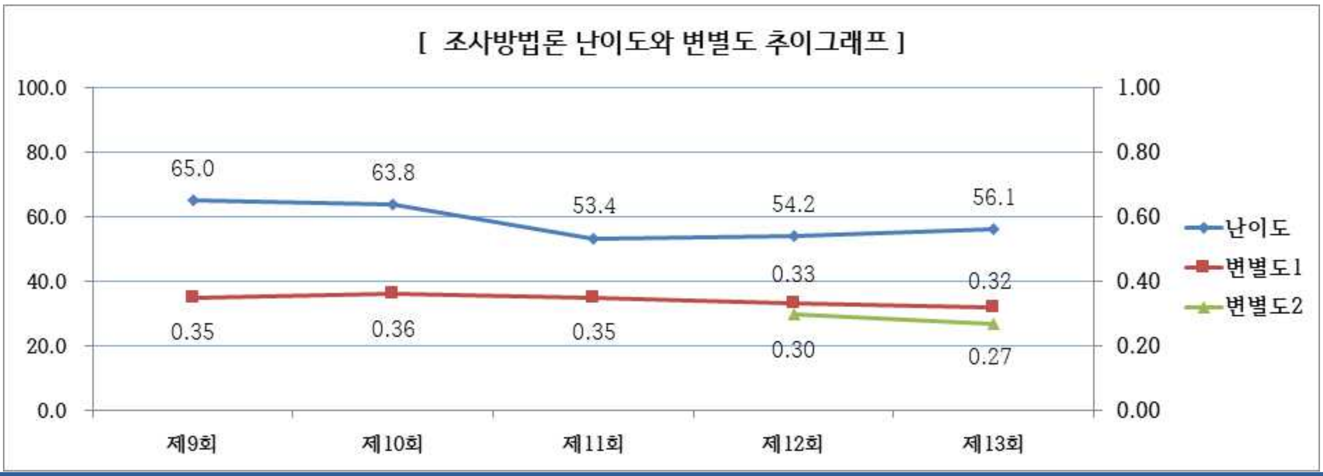

회차	난이도		변별도1		변별도2	
	평균	표준편차	평균	표준편차	평균	표준편차
제9회	65.0	22.4	.35	.17		
제10회	63.8	21.4	.36	.19		
제11회	53.4	20.3	.35	.27		
제12회	54.2	22.2	.33	.22	.30	.16
**제13회**	**56.1**	**22.1**	**.32**	**.17**	**.27**	**.14**

(6) 전회 대비 보건의사소통 난이도와 변별도

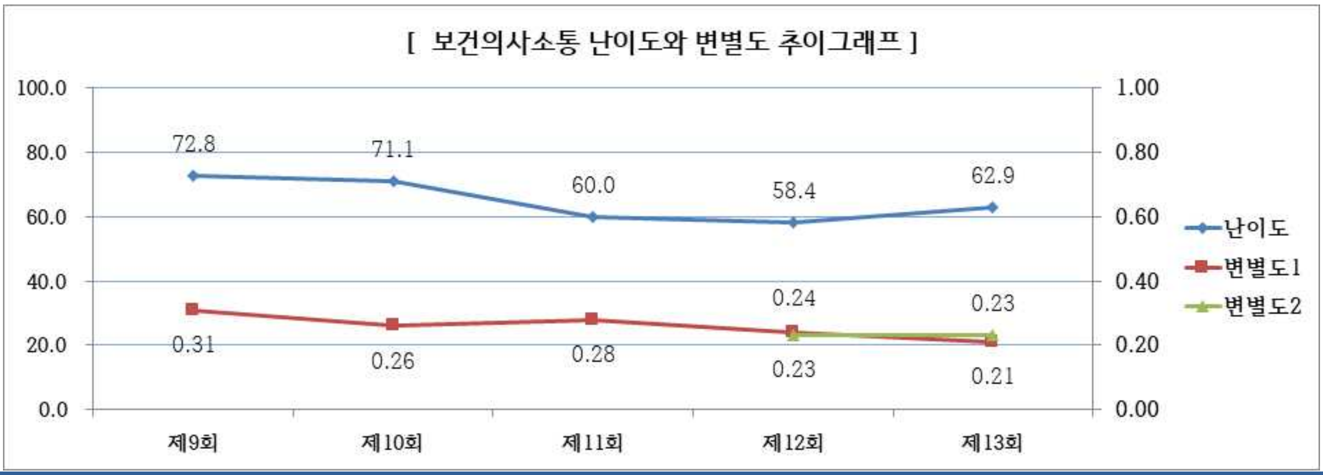

회차	난이도		변별도1		변별도2	
	평균	표준편차	평균	표준편차	평균	표준편차
제9회	72.8	17.4	.31	.26		
제10회	71.1	18.8	.26	.19		
제11회	60.0	25.2	.28	.21		
제12회	58.4	26.3	.24	.21	.23	.17
**제13회**	**62.9**	**26.8**	**.21**	**.15**	**.23**	**.13**

(7) 전회 대비 보건학 난이도와 변별도

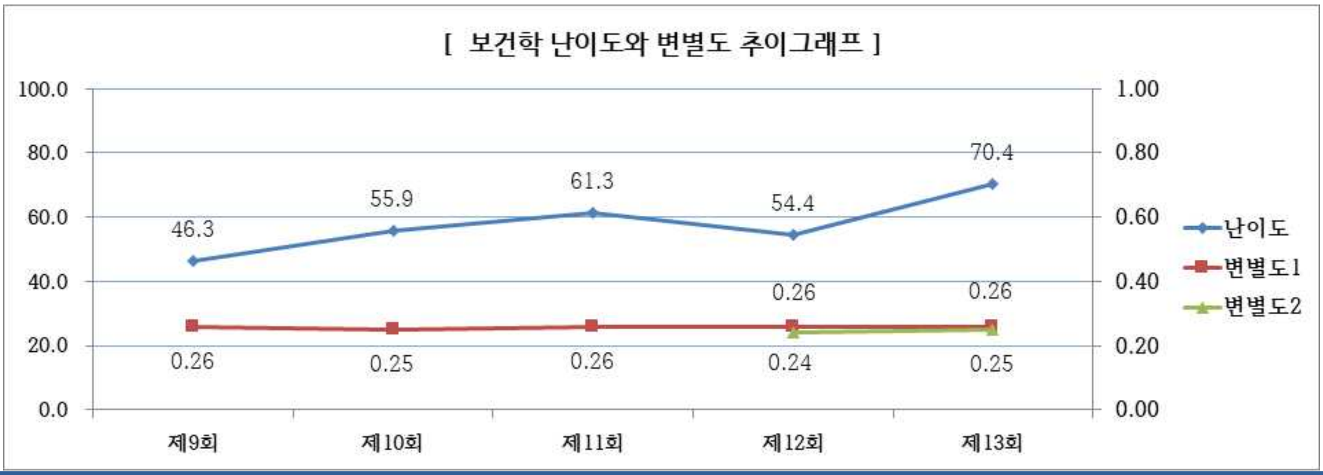

회차	난이도		변별도1		변별도2	
	평균	표준편차	평균	표준편차	평균	표준편차
제9회	46.3	22.8	.26	.33		
제10회	55.9	25.7	.25	.18		
제11회	61.3	22.2	.26	.16		
제12회	54.4	22.1	.26	.20	.24	.16
**제13회**	**70.4**	**19.4**	**.26**	**.18**	**.25**	**.15**

(8) 전회 대비 보건교육학 난이도와 변별도

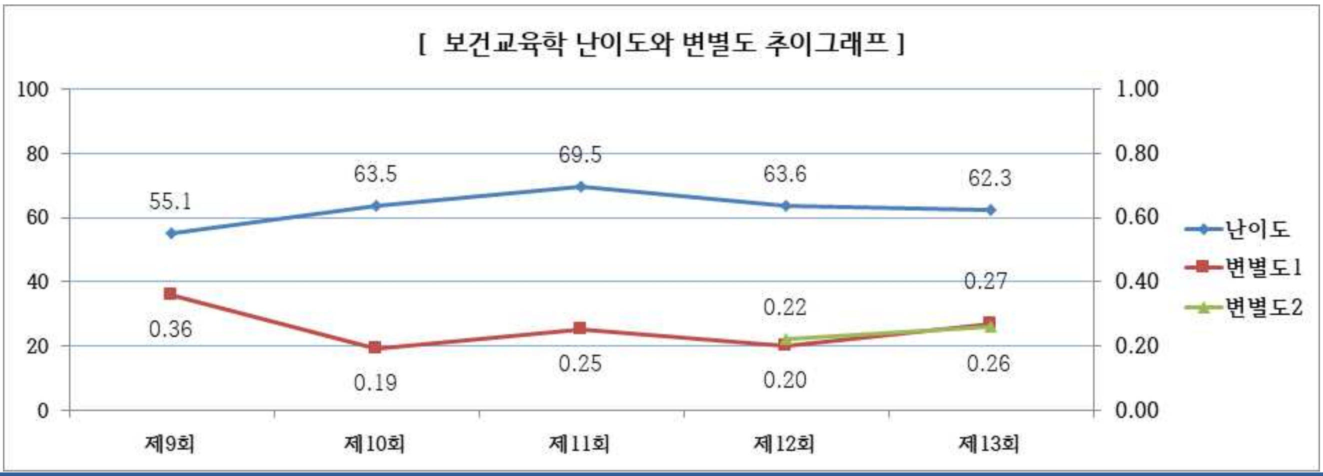

회차	난이도		변별도1		변별도2	
	평균	표준편차	평균	표준편차	평균	표준편차
제9회	55.1	23.3	.36	.20		
제10회	63.5	28.2	.19	.22		
제11회	69.5	23.6	.25	.17		
제12회	63.6	27.8	.20	.22	.22	.17
**제13회**	**62.3**	**24.0**	**.27**	**.15**	**.26**	**.12**

해석

- 전회 대비 보건프로그램 개발 및 평가, 보건교육방법론, 보건사업관리, 조사방법론, 보건의사소통, 보건학 과목의 난이도 지수는 각각 0.4, 3.2, 0.1, 1.9, 4.5, 16.0 증가하였으며, 보건의료법규, 보건교육학 과목의 난이도 지수는 각각 12.8, 1.3 감소함
- 보건프로그램 개발 및 평가, 교육보건방법론, 보건사업관리, 보건의료법규, 보건교육학 과목의 변별도 1 지수는 각각 0.04, 0.02, 0.02, 0.04, 0.07 증가하였으며, 조사방법론, 보건의사소통 과목의 변별도 1 지수는 각각 0.01, 0.03 감소함
- 보건학 과목의 변별도 1 지수는 변화 없음
- 보건프로그램 개발 및 평가, 보건교육학, 보건사업관리, 보건학, 보건교육학 과목의 변별도 2 지수는 각각 0.03, 0.01, 0.03, 0.01, 0.04 증가하였으며, 조사방법론 과목의 변별도 2 지수는 0.03 감소함.
- 보건의료법규, 보건의사소통 과목의 변별도 2 지수는 변화 없음

나) 과목별 난이도와 변별도 분포도 및 비율분석

(1) 보건프로그램 개발 및 평가 난이도와 변별도 분포도 및 비율분석

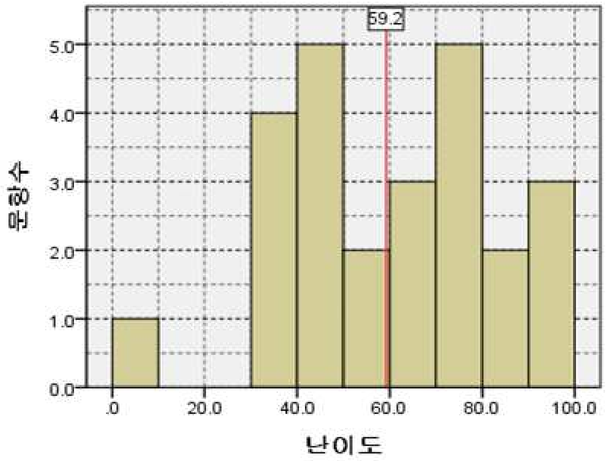

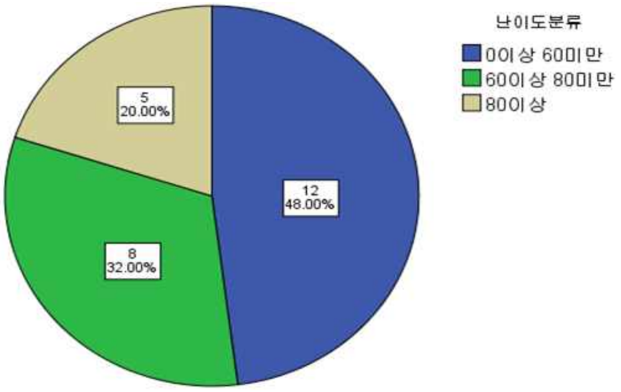

총점	난이도	표준편차	난이도	문항수	비율(%)
25	59.2	22.6	0~60미만	12	48.0
			60~80미만	8	32.0
			80~100	5	20.0
			전체	25	100.0

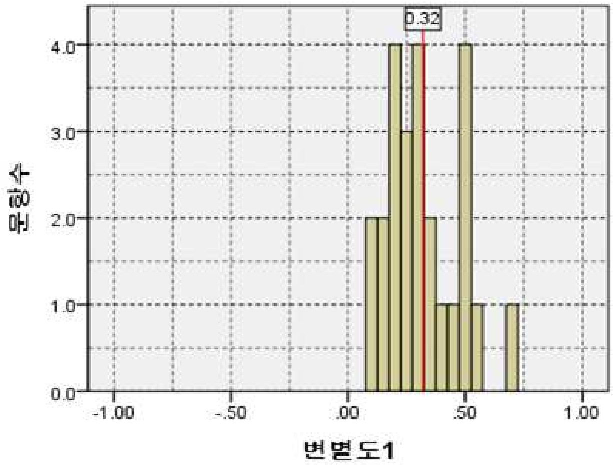

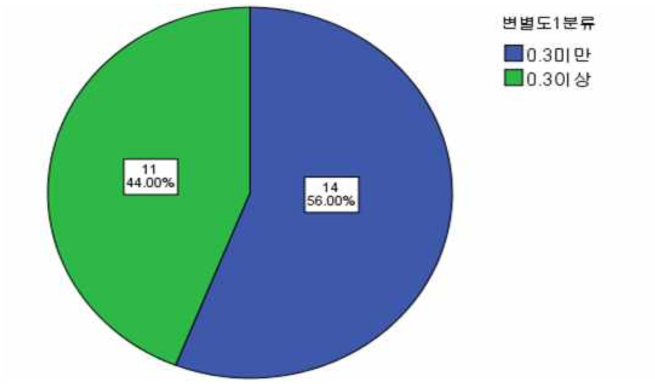

총점	변별도1	표준편차	변별도1	문항수	비율(%)
25	.32	.16	0.3미만	14	56.0
			0.3이상	11	44.0
			전체	25	100.0

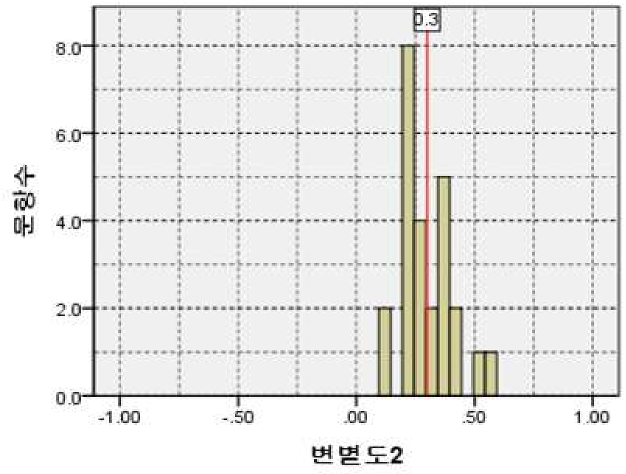

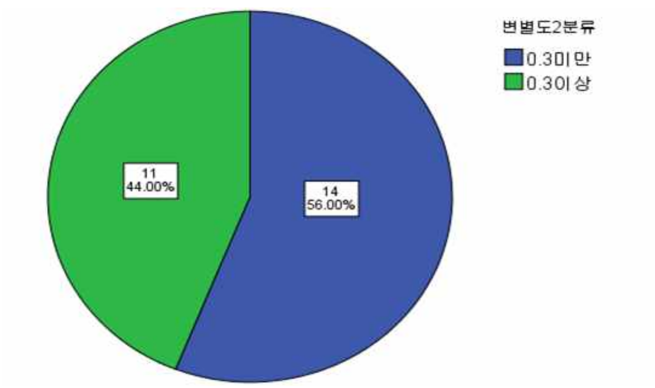

총점	변별도2	표준편차
25	.30	.11

변별도2	문항수	비율(%)
0.3미만	14	56.0
0.3이상	11	44.0
전체	25	100.0

해석

- 보건프로그램 개발 및 평가 과목에서 난이도 지수가 80 에서 100 사이인 문항이 전체 25 문항 중 5 문항으로 가장 적었으며, 다음으로 60 이상 80 미만인 문항이 8 문항, 60 미만인 문항은 12 문항으로 나타남
- 변별도 1 지수를 기준으로 분류하였을 때, 0.3 미만인 문항이 14 문항으로 0.3 이상인 문항이 11 문항인 것에 비해 더 많이 나타남
- 변별도 2 지수를 기준으로 분류하였을 때, 0.3 미만인 문항이 14 문항으로 0.3 이상인 문항이 11 문항인 것에 비해 더 많이 나타남

(2) 보건교육방법론 난이도와 변별도 분포도 및 비율분석

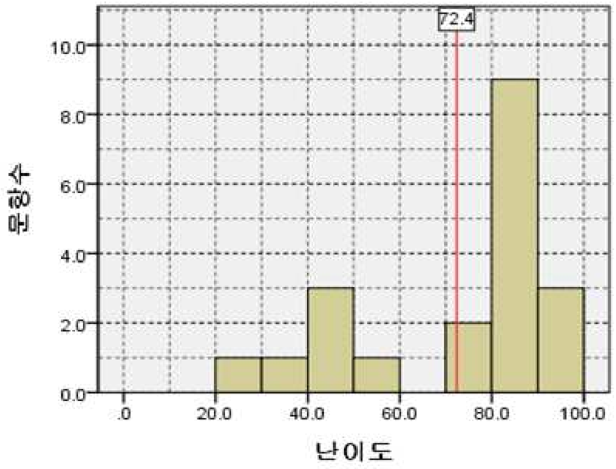

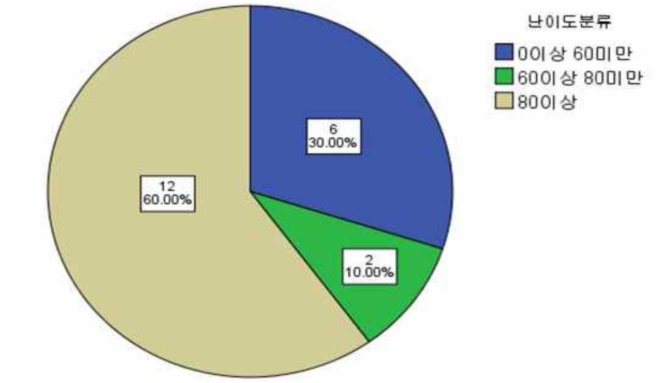

총점	난이도	표준편차
20	72.4	21.4

난이도	문항수	비율(%)
0~60미만	6	30.0
60~80미만	2	10.0
80~100	12	60.0
전체	20	100.0

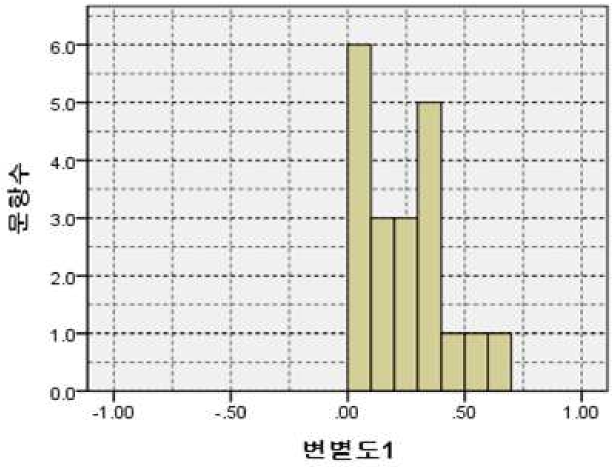

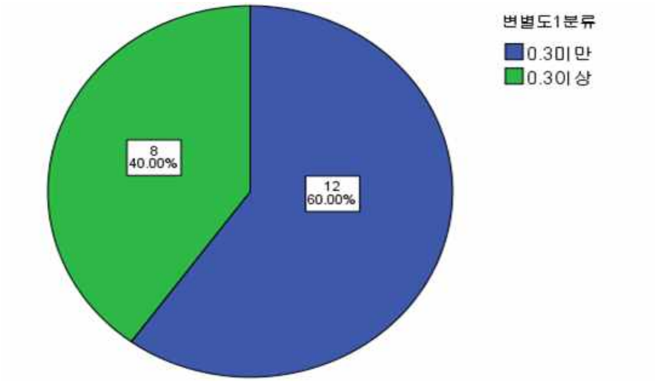

총점	변별도1	표준편차
20	.23	.18

변별도1	문항수	비율(%)
0.3미만	12	60.0
0.3이상	8	40.0
전체	20	100.0

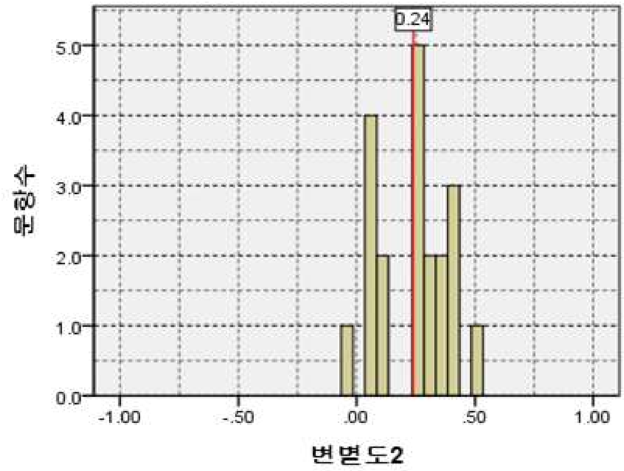

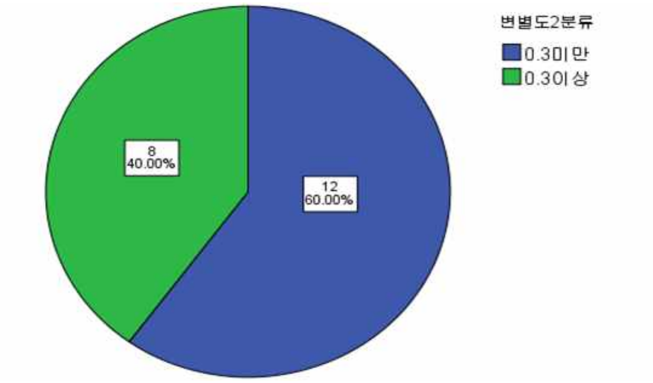

총점	변별도2	표준편차	변별도2	문항수	비율(%)
20	.24	.15	0.3미만	12	60.0
			0.3이상	8	40.0
			전체	20	100.0

해석

- 보건교육방법론 과목에서 난이도 지수가 80에서 100 사이인 문항이 전체 20문항 중 12문항으로 나타났으며, 다음으로 60이상 80미만인 문항이 2문항, 60미만인 문항은 6문항으로 나타남
- 변별도1 지수를 기준으로 분류하였을 때, 0.3미만인 문항이 12문항, 0.3이상인 문항이 8문항인 것에 비해 더 많이 나타남
- 변별도2 지수를 기준으로 분류하였을 때, 0.3미만인 문항이 12문항으로 0.3이상인 문항이 8문항인 것에 비해 더 많이 나타남

(3) 보건사업관리 난이도와 변별도 분포도 및 비율분석

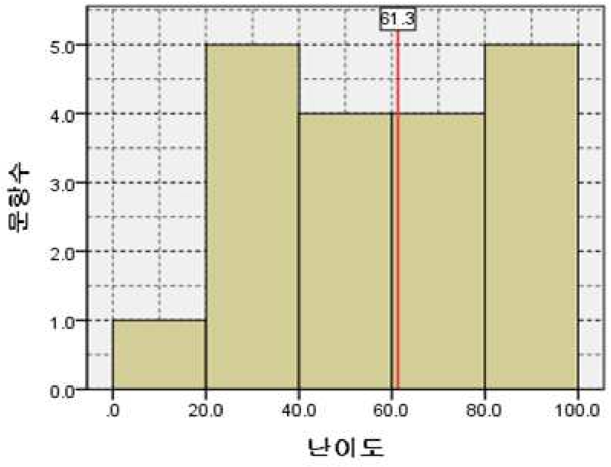

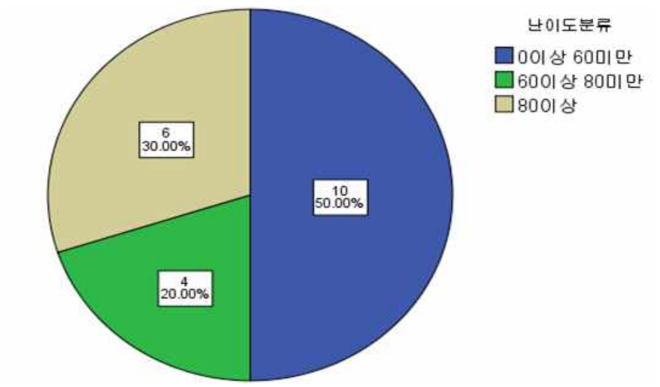

총점	난이도	표준편차	난이도	문항수	비율(%)
20	61.0	27.9	0~60미만	10	50.0
			60~80미만	4	20.0
			80~100	6	30.0
			전체	20	100.0

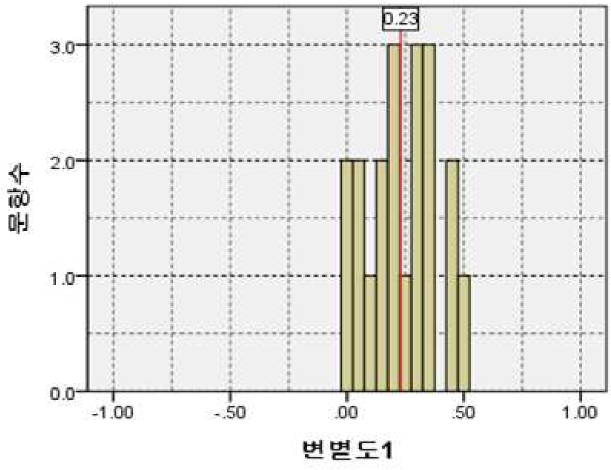

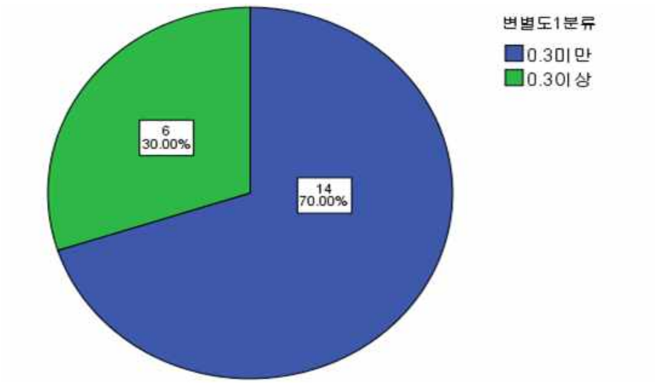

총점	변별도1	표준편차	변별도1	문항수	비율(%)
20	.23	.15	0.3미만	14	70.0
			0.3이상	6	30.0
			전체	20	100.0

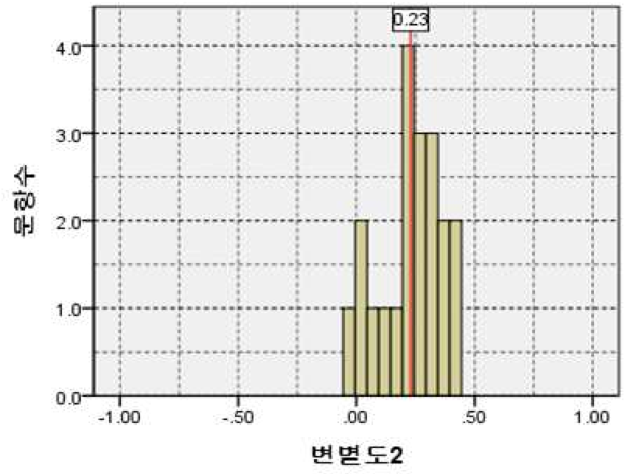

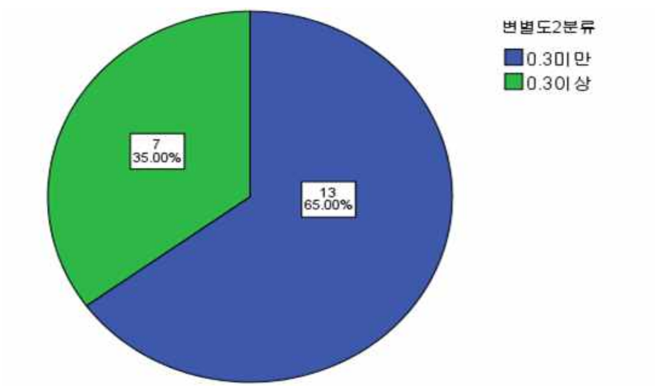

총점	변별도2	표준편차
20	.23	.13

변별도2	문항수	비율(%)
0.3미만	13	65.0
0.3이상	7	35.0
전체	20	100.0

해석

- 보건사업관리 과목에서 난이도 지수가 80 에서 100 사이인 문항이 전체 20 문항 중 6 문항으로 나타났으며, 다음으로 60 이상 80 미만인 문항이 4 문항, 60 미만인 문항은 10 문항으로 나타남
- 변별도 1 지수를 기준으로 분류하였을 때, 0.3 미만인 문항이 14 문항으로 0.3 이상인 문항이 6 문항인 것에 비해 더 많이 나타남
- 변별도 2 지수를 기준으로 분류하였을 때, 0.3 미만인 문항이 13 문항으로 0.3 이상인 문항이 7 문항인 것에 비해 더 많이 나타남

(4) 보건의료법규 난이도와 변별도 분포도 및 비율분석

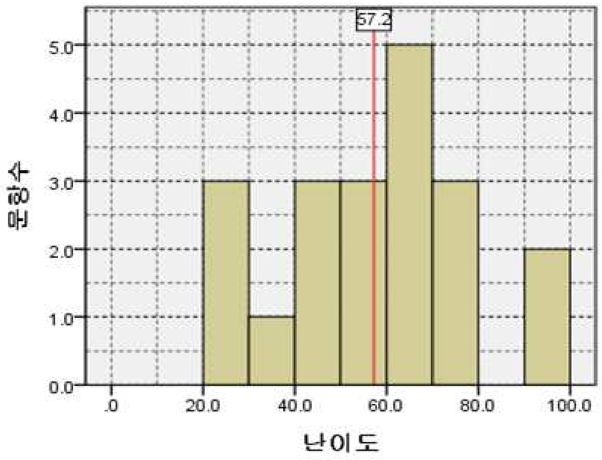

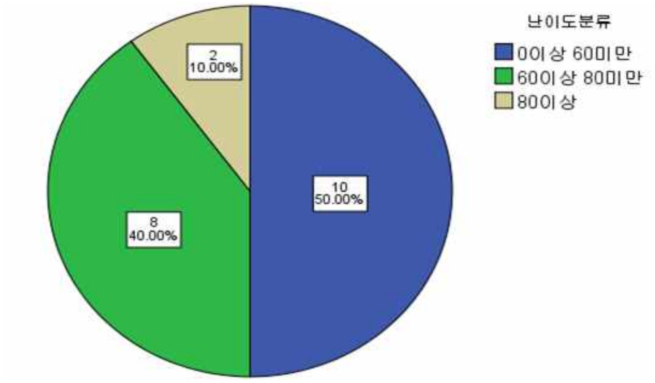

총점	난이도	표준편차
20	57.2	20.5

난이도	문항수	비율(%)
0~60미만	10	50.0
60~80미만	8	40.0
80~100	2	10.0
전체	20	100.0

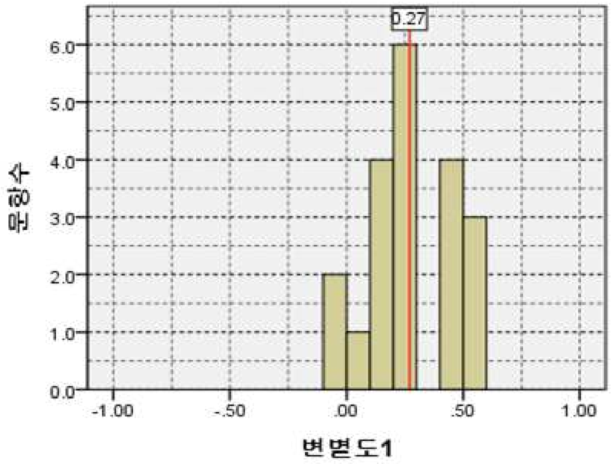

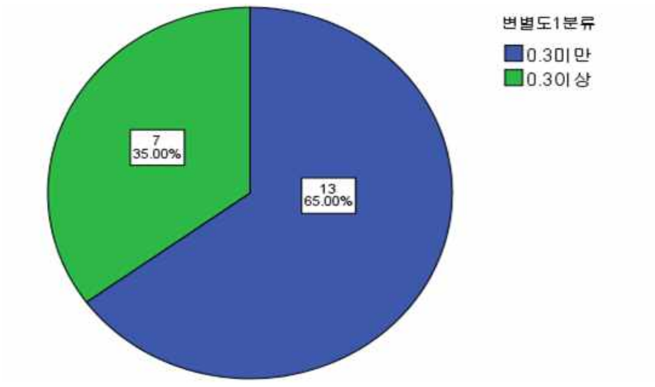

총점	변별도1	표준편차
20	.27	.19

변별도1	문항수	비율(%)
0.3미만	13	65.0
0.3이상	7	35.0
전체	20	100.0

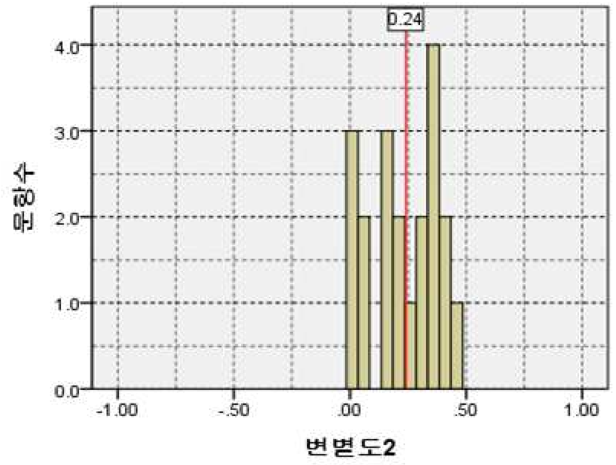

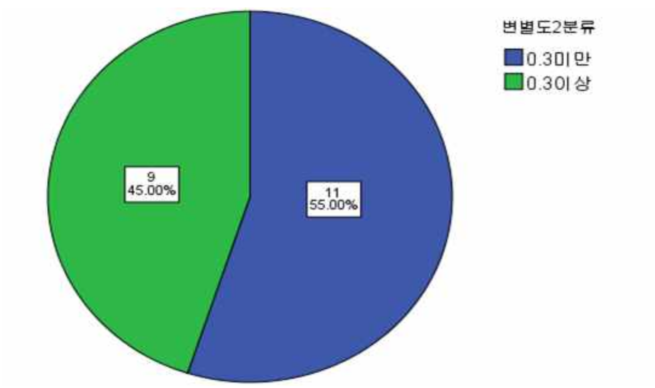

총점	변별도2	표준편차
20	.24	.15

변별도2	문항수	비율(%)
0.3미만	11	55.0
0.3이상	9	45.0
전체	20	100.0

해석

- 보건의료법규 과목에서 난이도 지수가 80에서 100 사이인 문항이 전체 20문항 중 2문항으로 나타났으며, 다음으로 60이상 80미만인 문항이 8문항, 60미만인 문항은 10문항으로 나타남
- 변별도1 지수를 기준으로 분류하였을 때, 0.3미만인 문항이 13문항으로 0.3이상인 문항이 7문항인 것에 비해 더 많이 나타남
- 변별도2 지수를 기준으로 분류하였을 때, 0.3미만인 문항이 11문항으로 0.3이상인 문항이 9문항인 것에 비해 더 많이 나타남

(5) 조사방법론 난이도와 변별도 분포도 및 비율분석

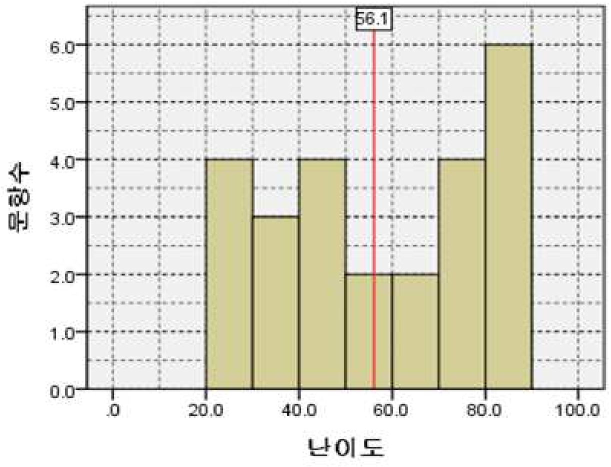

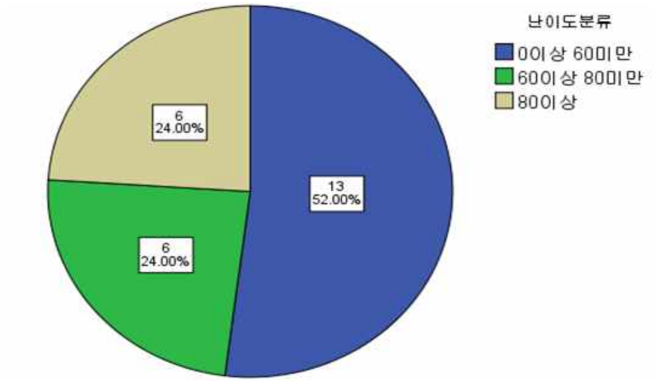

총점	난이도	표준편차
25	56.1	22.1

난이도	문항수	비율(%)
0~60미만	13	52.0
60~80미만	6	24.0
80~100	6	24.0
전체	25	100.0

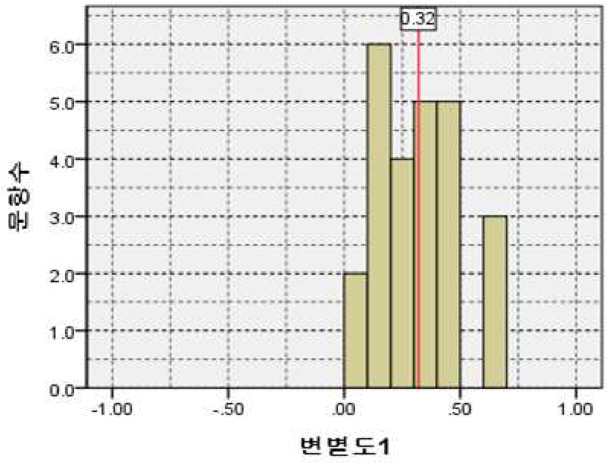

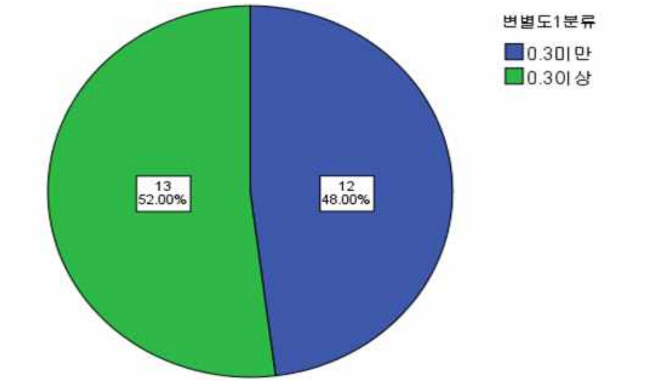

총점	변별도1	표준편차
25	.32	.17

변별도1	문항수	비율(%)
0.3미만	12	48.0
0.3이상	13	52.0
전체	25	100.0

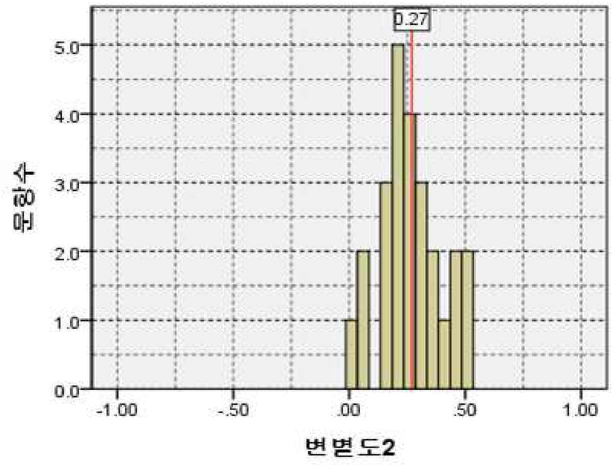

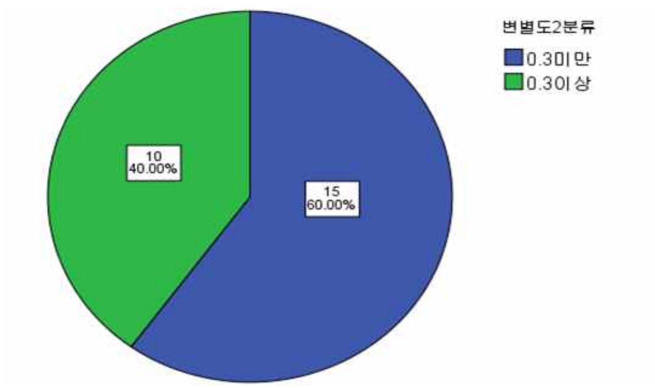

총점	변별도2	표준편차
25	.27	.14

변별도2	문항수	비율(%)
0.3미만	15	60.0
0.3이상	10	40.0
전체	25	100.0

해석

- 조사방법론 과목에서 난이도 지수가 80에서 100 사이인 문항이 전체 25문항 중 6문항으로 가장 적었으며, 다음으로 60이상 80미만인 문항이 6문항, 60 미만인 문항은 13문항으로 나타남
- 변별도1 지수를 기준으로 분류하였을 때, 0.3미만인 문항이 12문항으로 0.3 이상인 문항이 13문항인 것에 비해 더 적게 나타남
- 변별도2 지수를 기준으로 분류하였을 때, 0.3미만인 문항이 15문항으로 0.3 이상인 문항이 10문항인 것에 비해 더 많이 나타남

(6) 보건의사소통 난이도와 변별도 분포도 및 비율분석

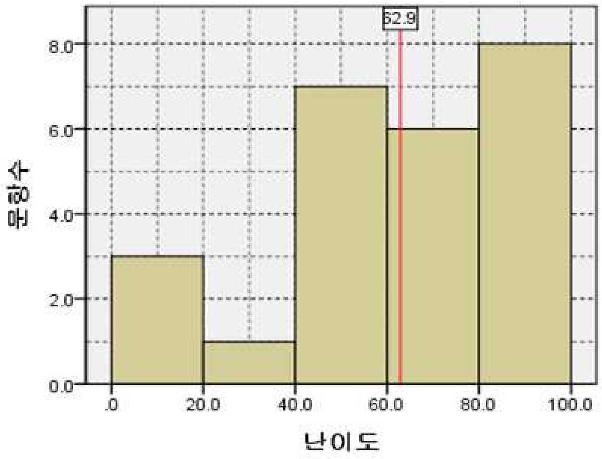

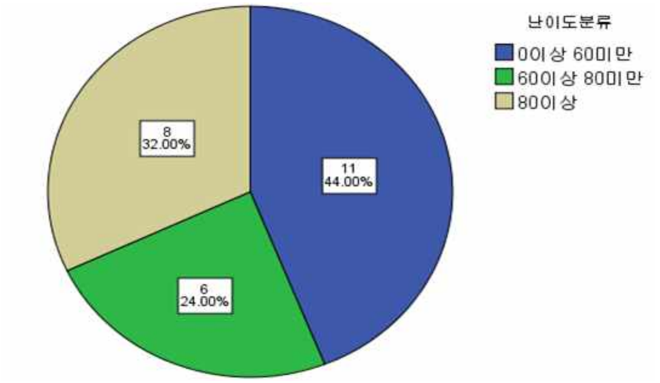

총점	난이도	표준편차
25	62.9	26.8

난이도	문항수	비율(%)
0~60미만	11	44.0
60~80미만	6	24.0
80~100	8	32.0
전체	25	100.0

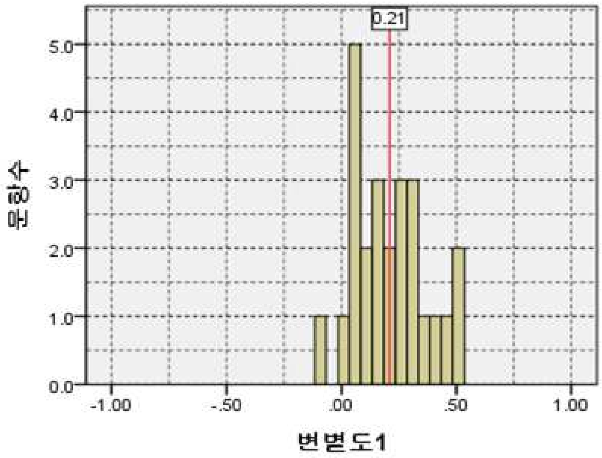

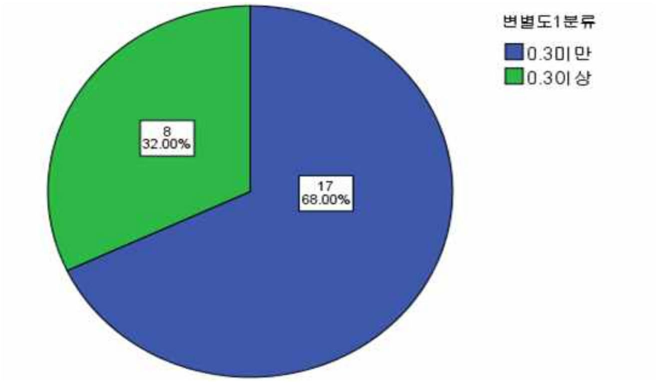

총점	변별도1	표준편차
25	.21	.15

변별도1	문항수	비율(%)
0.3미만	17	68.0
0.3이상	8	32.0
전체	25	100.0

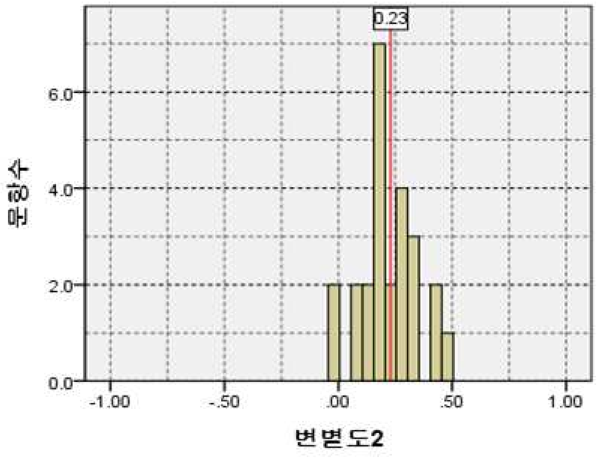

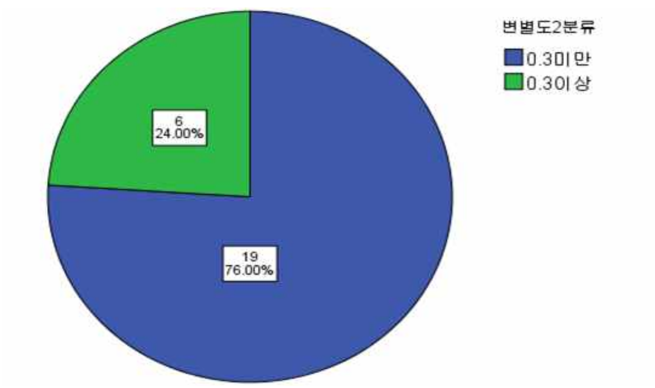

총점	변별도2	표준편차
25	.23	.13

변별도2	문항수	비율(%)
0.3미만	19	76.0
0.3이상	6	24.0
전체	25	100.0

해석

- 보건의사소통 과목에서 난이도 지수가 80 에서 100 사이인 문항이 전체 25 문항 중 8 문항으로 나타났으며, 다음으로 60 이상 80 미만인 문항이 6 문항, 60 미만인 문항은 11 문항으로 나타남
- 변별도 1 지수를 기준으로 분류하였을 때, 0.3 미만인 문항이 17 문항, 0.3 이상인 문항이 8 문항인 것에 비해 더 많이 나타남
- 변별도 2 지수를 기준으로 분류하였을 때, 0.3 미만인 문항이 19 문항으로 0.3 이상인 문항이 6 문항인 것에 비해 더 많이 나타남

(7) 보건학 난이도와 변별도 분포도 및 비율분석

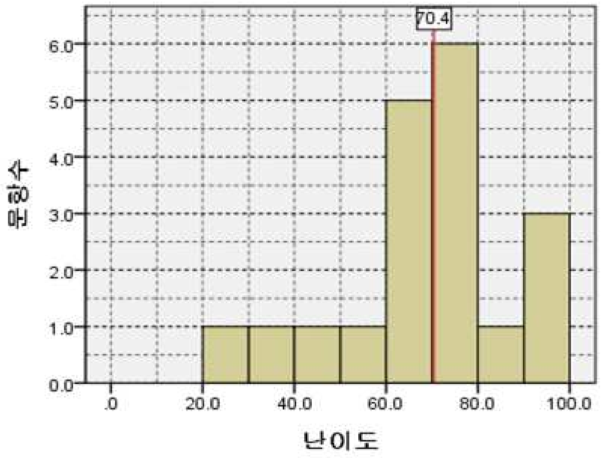

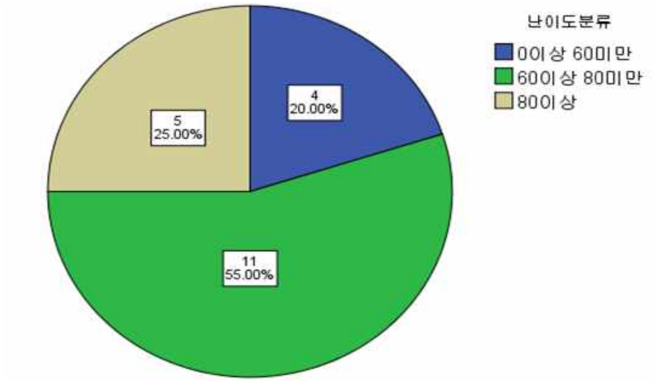

총점	난이도	표준편차
20	70.4	19.4

난이도	문항수	비율(%)
0~60미만	4	20.0
60~80미만	11	55.0
80~100	5	25.0
전체	20	100.0

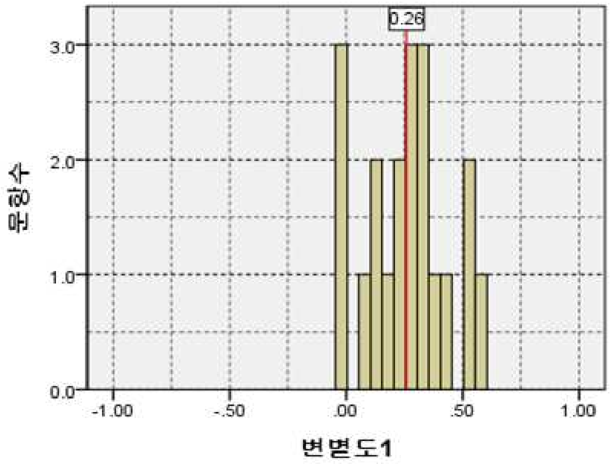

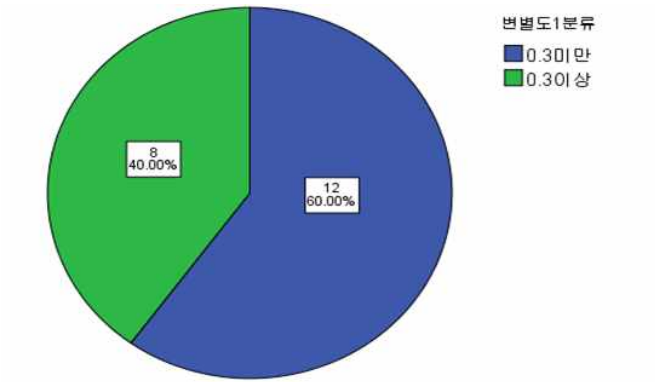

총점	변별도1	표준편차
20	.26	.18

변별도1	문항수	비율(%)
0.3미만	12	60.0
0.3이상	8	40.0
전체	20	100.0

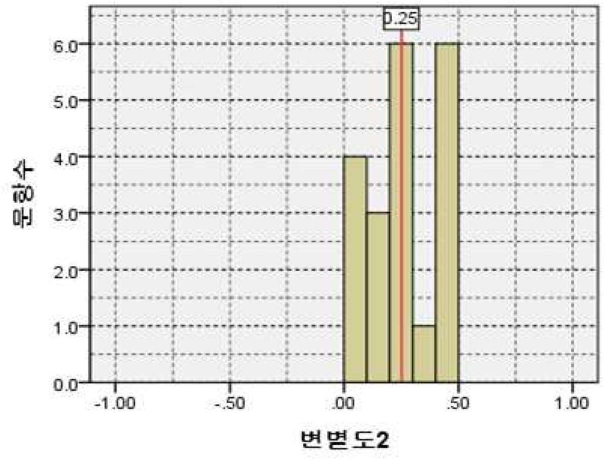

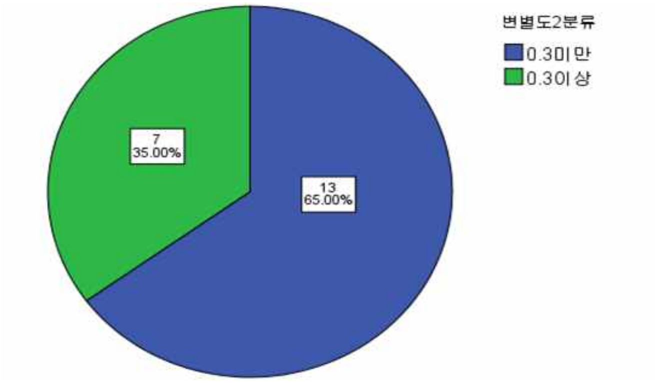

총점	변별도2	표준편차
20	.25	.15

변별도2	문항수	비율(%)
0.3미만	13	65.0
0.3이상	7	35.0
전체	20	100.0

해석

- 보건학 과목에서 난이도 지수가 80 에서 100 사이인 문항이 전체 20 문항 중 5 문항으로 나타났으며, 다음으로 60 이상 80 미만인 문항이 11 문항, 60 미만인 문항은 4 문항으로 나타남
- 변별도 1 지수를 기준으로 분류하였을 때, 0.3 미만인 문항이 12 문항으로 0.3 이상인 문항이 8 문항인 것에 비해 더 많이 나타남
- 변별도 2 지수를 기준으로 분류하였을 때, 0.3 미만인 문항이 13 문항으로 0.3 이상인 문항이 7 문항인 것에 비해 더 많이 나타남

(8) 보건교육학 난이도와 변별도 분포도 및 비율분석

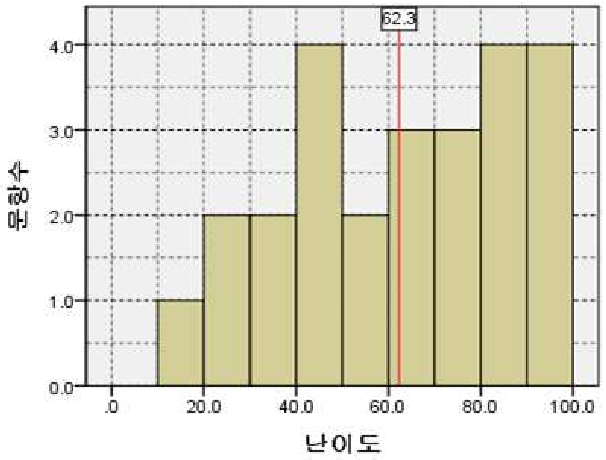

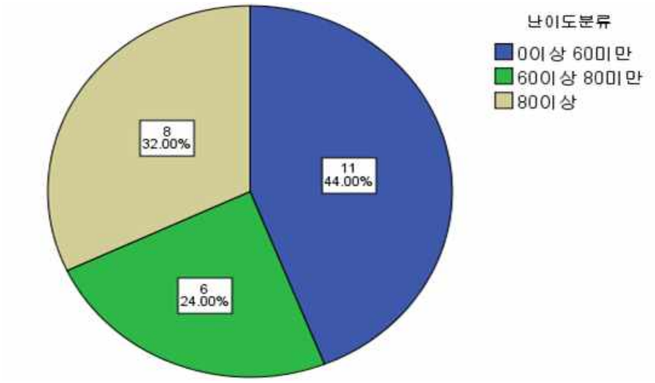

총점	난이도	표준편차
25	62.3	24.0

난이도	문항수	비율(%)
0~60미만	11	44.0
60~80미만	6	24.0
80~100	8	32.0
전체	25	100.0

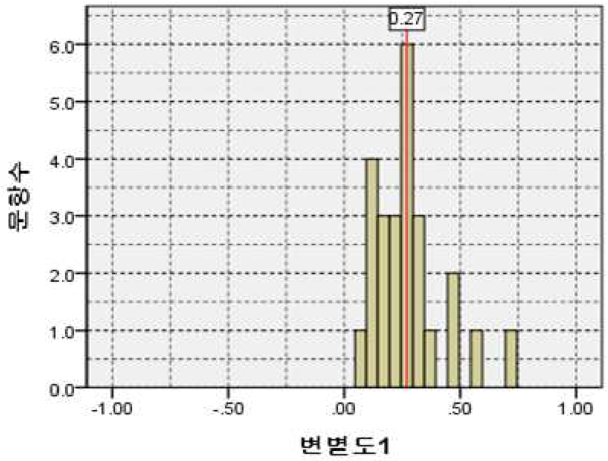

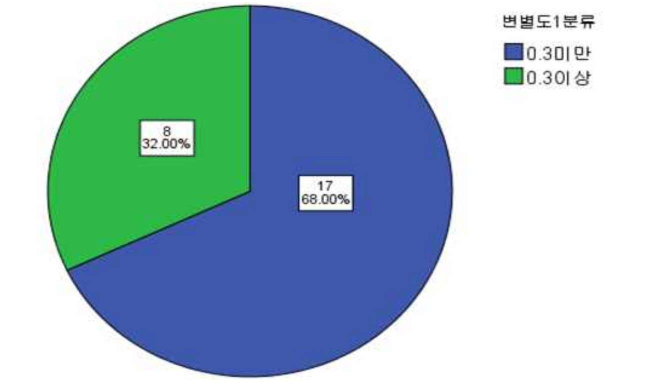

총점	변별도1	표준편차
25	.27	.15

변별도1	문항수	비율(%)
0.3미만	17	68.0
0.3이상	8	32.0
전체	25	100.0

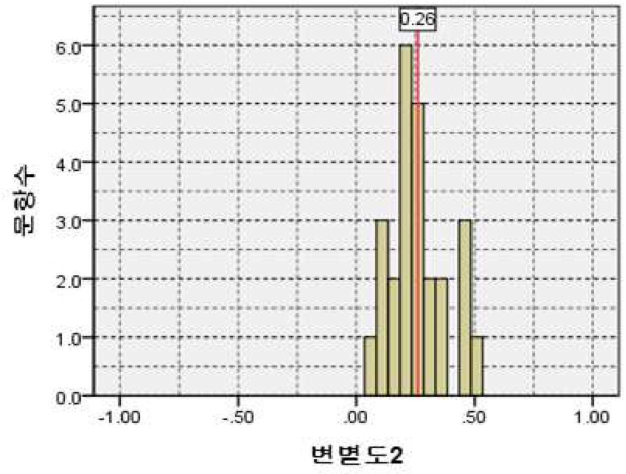

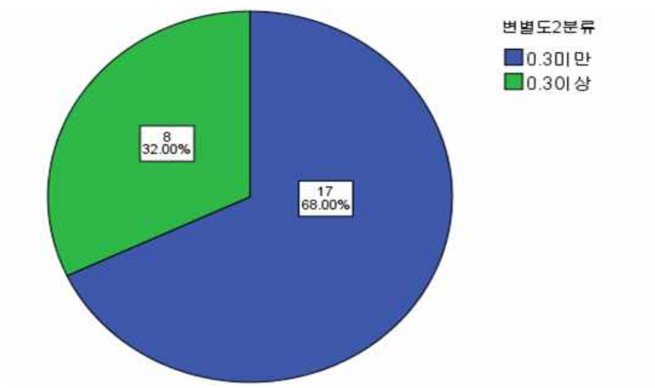

총점	변별도2	표준편차
25	.26	.12

변별도2	문항수	비율(%)
0.3미만	17	68.0
0.3이상	8	32.0
전체	25	100.0

해석

- 보건교육학 과목에서 난이도 지수가 80 에서 100 사이인 문항이 전체 25 문항 중 8 문항으로 나타났으며, 다음으로 60 이상 80 미만인 문항이 6 문항, 60 미만인 문항은 11 문항으로 나타남
- 변별도 1 지수를 기준으로 분류하였을 때, 0.3 미만인 문항이 17 문항으로 0.3 이상인 문항이 8 문항인 것에 비해 더 많이 나타남
- 변별도 2 지수를 기준으로 분류하였을 때, 0.3 미만인 문항이 17 문항으로 0.3 이상인 문항이 8 문항인 것에 비해 더 많이 나타남

3) 지식수준별 난이도와 변별도

가) 전회 대비 지식수준별 난이도와 변별도

(1) 전회 대비 암기형 난이도와 변별도

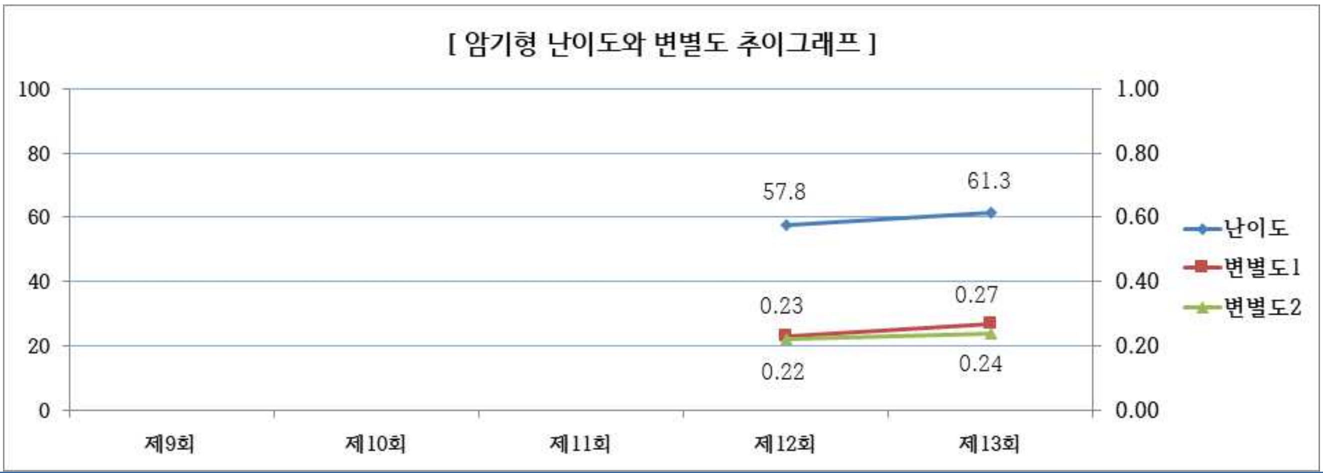

회차	난이도		변별도1		변별도2	
	평균	표준편차	평균	표준편차	평균	표준편차
제9회						
제10회						
제11회						
제12회	57.8	23.8	.23	.21	.22	.16
**제13회**	**61.3**	**21.1**	**.27**	**.16**	**.24**	**.13**

(2) 전회 대비 해석형 난이도와 변별도

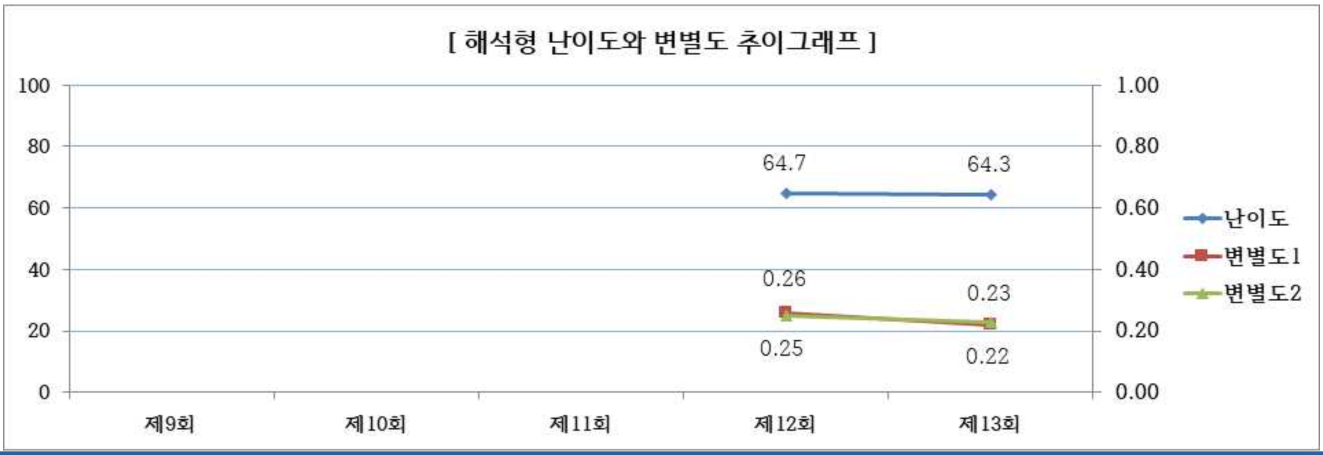

회차	난이도		변별도1		변별도2	
	평균	표준편차	평균	표준편차	평균	표준편차
제9회						
제10회						
제11회						
제12회	64.7	24.5	.26	.20	.25	.17
**제13회**	**64.3**	**26.2**	**.22**	**.15**	**.23**	**.13**

(3) 전회 대비 해결형 난이도와 변별도

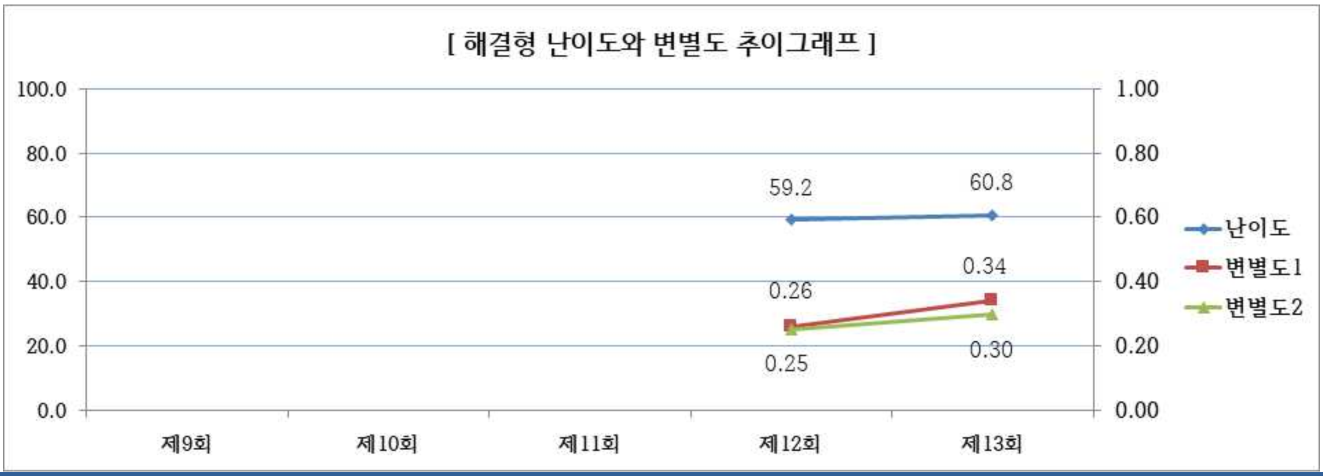

회차	난이도		변별도1		변별도2	
	평균	표준편차	평균	표준편차	평균	표준편차
제9회						
제10회						
제11회						
제12회	59.2	23.9	.26	.23	.25	.19
**제13회**	**60.8**	**22.1**	**.34**	**.18**	**.30**	**.13**

해석

- 전회 대비 암기형, 해결형 문항의 난이도 지수는 각각 3.5, 1.6 증가하였으며, 해석형 문항의 난이도 지수는 0.4 감소함
- 변별도 1 지수의 경우 암기형, 해결형 문항에서는 각각 0.04, 0.08 증가하였으나, 해석형 문항에서는 0.04 감소함
- 변별도 2 지수는 암기형, 해결형 문항에서는 각각 0.02,0.05 증가하였으나, 해석형 문항에서 0.02 감소함

나) 지식수준별 난이도와 변별도 분포도 및 비율분석

(1) 암기형 난이도와 변별도 분포도 및 비율분석

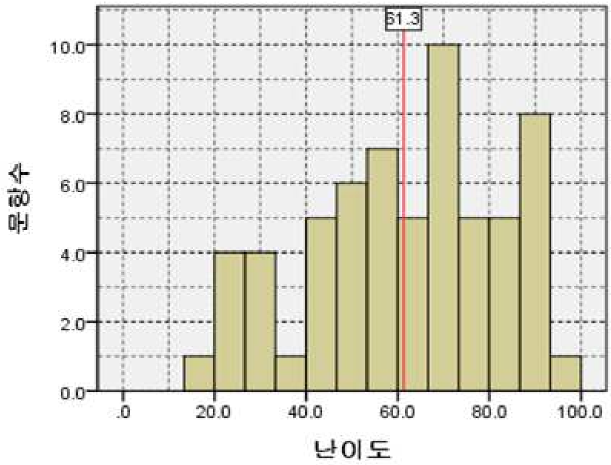

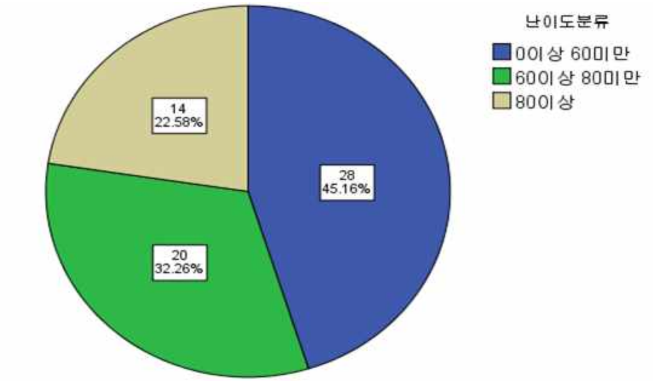

총점	난이도	표준편차
62	61.3	21.1

난이도	문항수	비율(%)
0~60미만	28	45.2
60~80미만	20	32.3
80~100	14	22.6
전체	62	100.0

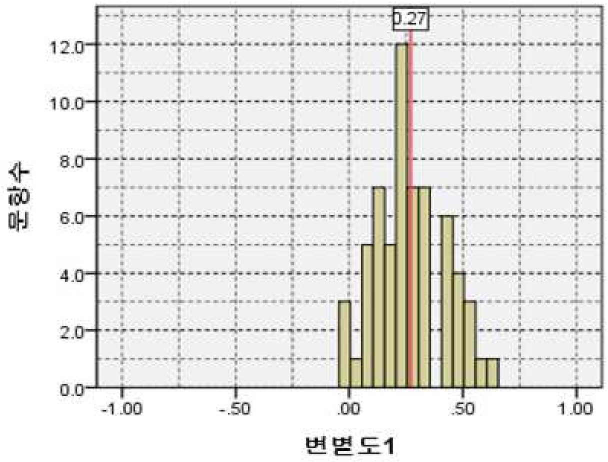

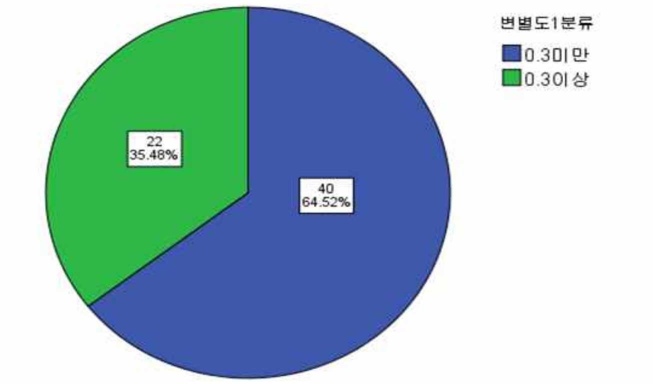

총점	변별도1	표준편차
62	.27	.16

변별도1	문항수	비율(%)
0.3미만	40	64.5
0.3이상	22	35.5
전체	62	100.0

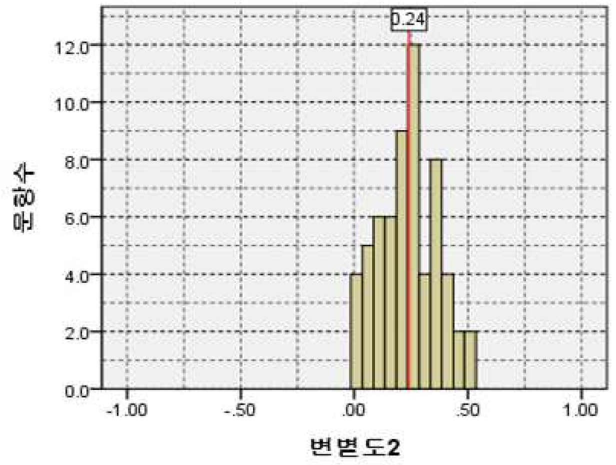

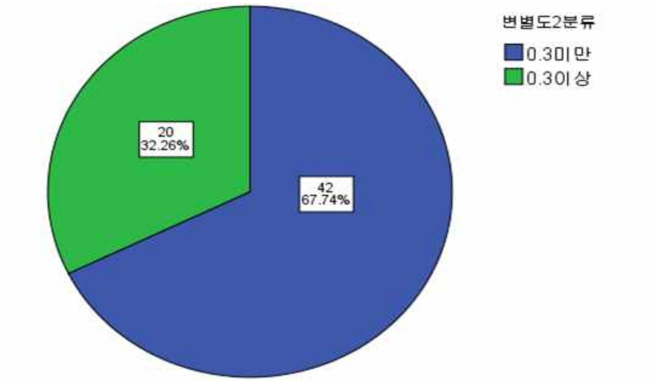

총점	변별도2	표준편차
62	.24	.13

변별도2	문항수	비율(%)
0.3미만	42	67.7
0.3이상	20	32.3
전체	62	100.0

해석

- 암기형에서 난이도 지수가 80 에서 100 사이인 문항이 전체 62 문항 중 14 문항으로 가장 적었으며, 다음으로 60 이상 80 미만인 문항이 20 문항, 60 미만인 문항은 28 문항으로 나타남
- 변별도 1 지수를 기준으로 분류하였을 때, 0.3 미만인 문항이 40 문항으로 0.3 이상인 문항이 22 문항인 것에 비해 더 많이 나타남
- 변별도 2 지수를 기준으로 분류하였을 때, 0.3 미만인 문항이 42 문항으로 0.3 이상인 문항이 20 문항인 것에 비해 더 많이 나타남

(2) 해석형 난이도와 변별도 분포도 및 비율분석

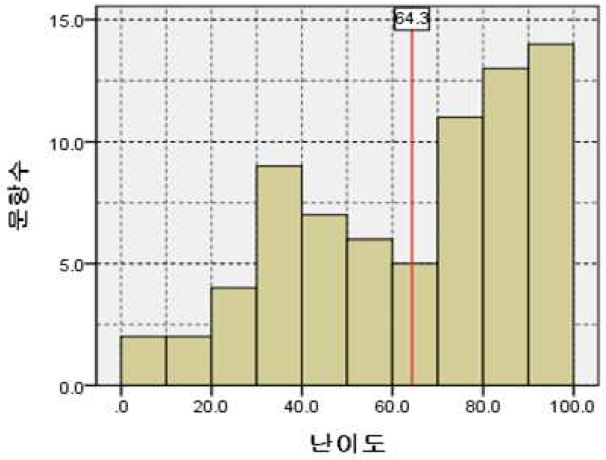

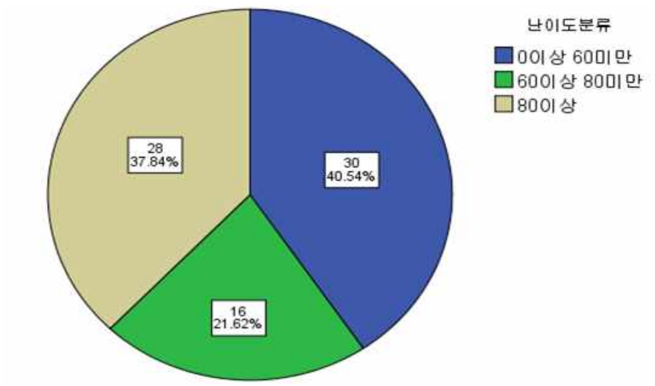

총점	난이도	표준편차
74	64.3	26.2

난이도	문항수	비율(%)
0~60미만	30	40.5
60~80미만	16	21.6
80~100	28	37.8
전체	74	100.0

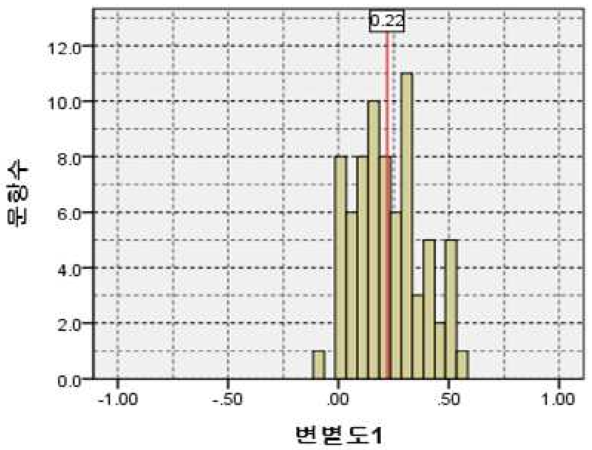

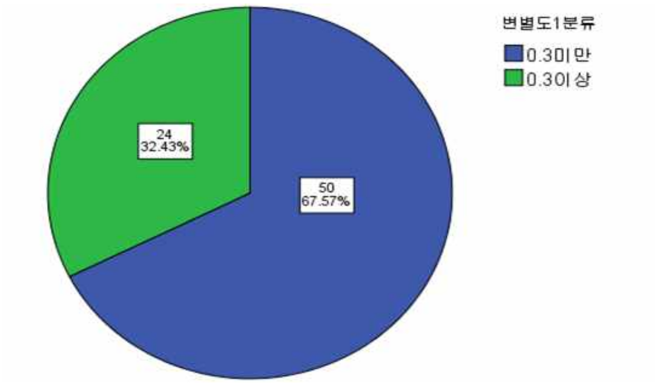

총점	변별도1	표준편차
74	.22	.15

변별도1	문항수	비율(%)
0.3미만	50	67.6
0.3이상	24	32.4
전체	74	100.0

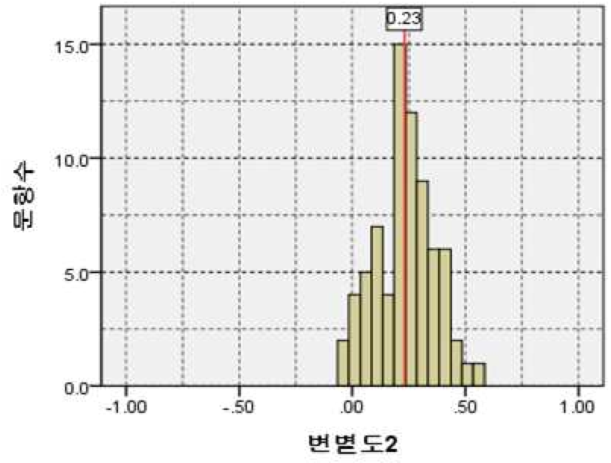

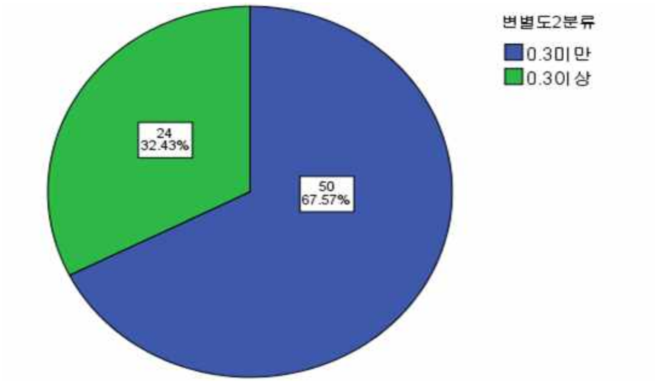

총점	변별도2	표준편차
74	.23	.13

변별도2	문항수	비율(%)
0.3미만	50	67.6
0.3이상	24	32.4
전체	74	100.0

해석

- 해석형에서 난이도 지수가 80에서 100 사이인 문항이 전체 74문항 중 28문항으로 나타났으며, 다음으로 60 이상 80 미만인 문항이 16문항, 60 미만인 문항은 30문항으로 나타남
- 변별도1 지수를 기준으로 분류하였을 때, 0.3 미만인 문항이 50문항, 0.3 이상인 문항이 24문항인 것에 비해 더 많이 나타남
- 변별도2 지수를 기준으로 분류하였을 때, 0.3 미만인 문항이 50문항으로 0.3 이상인 문항이 24문항인 것에 비해 더 많이 나타남

(3) 해결형 난이도와 변별도 분포도 및 비율분석

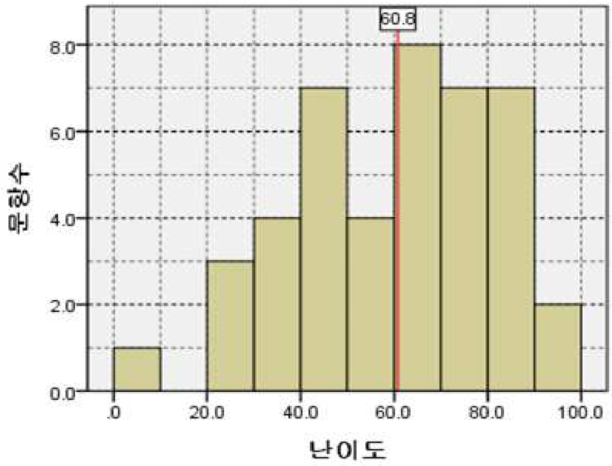

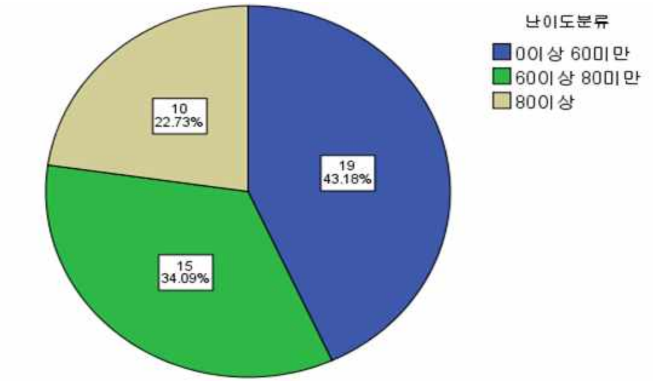

총점	난이도	표준편차
44	60.8	22.1

난이도	문항수	비율(%)
0~60미만	19	43.2
60~80미만	15	34.1
80~100	10	22.7
전체	44	100.0

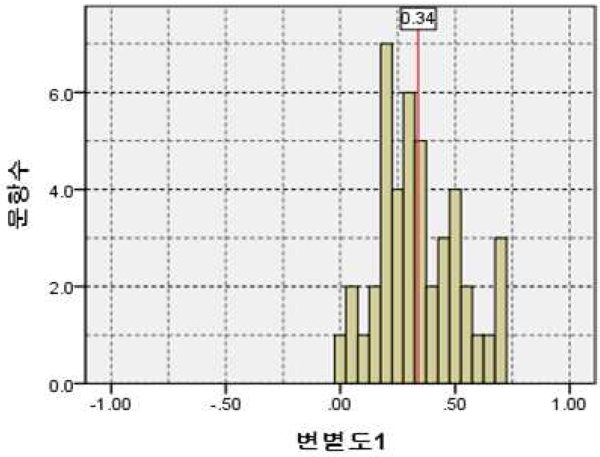

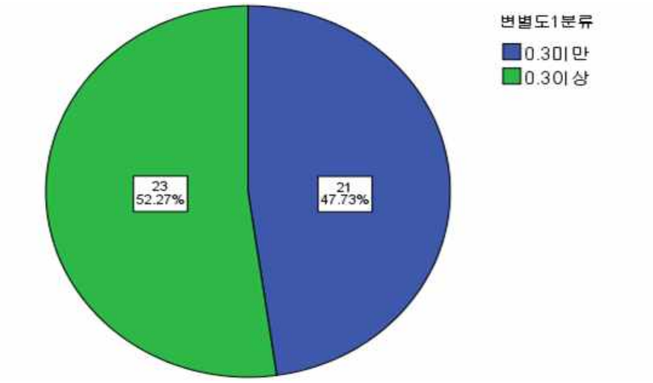

총점	변별도1	표준편차
44	.34	.18

변별도1	문항수	비율(%)
0.3미만	21	47.7
0.3이상	23	52.3
전체	44	100.0

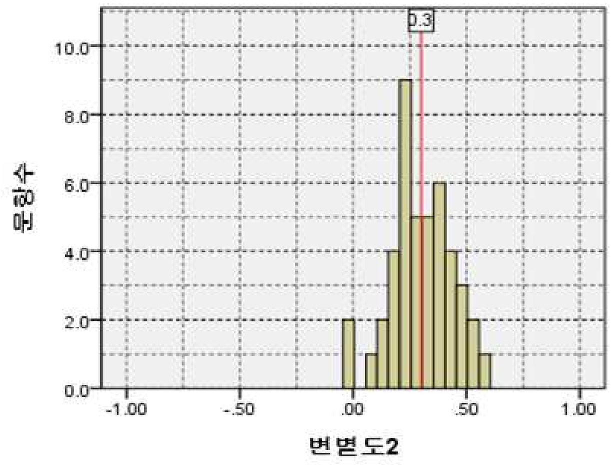

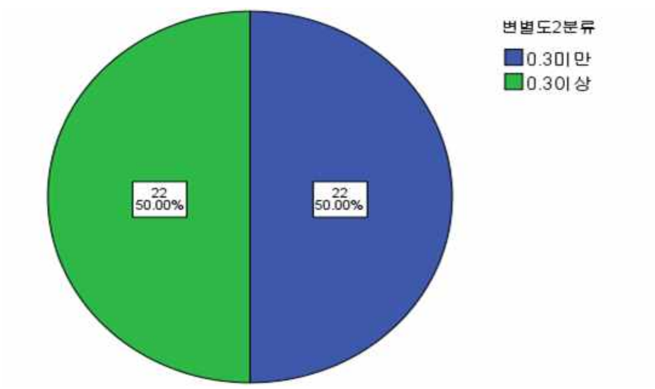

총점	변별도2	표준편차
44	.30	.13

변별도2	문항수	비율(%)
0.3미만	22	50.0
0.3이상	22	50.0
전체	44	100.0

해석

- 해결형에서 난이도 지수가 80에서 100 사이인 문항이 전체 44문항 중 10문항으로 나타났으며, 다음으로 60 이상 80 미만인 문항이 15문항, 60 미만인 문항은 19문항으로 나타남
- 변별도 1 지수를 기준으로 분류하였을 때, 0.3 미만인 문항이 21문항으로 0.3 이상인 문항이 23문항인 것에 비해 더 적게 나타남
- 변별도 2 지수를 기준으로 분류하였을 때, 0.3 미만인 문항이 22문항으로 0.3 이상인 문항과 같게 나타남

3. 난이도와 변별도 간 산포도

1) 전체 난이도와 변별도 간 산포도

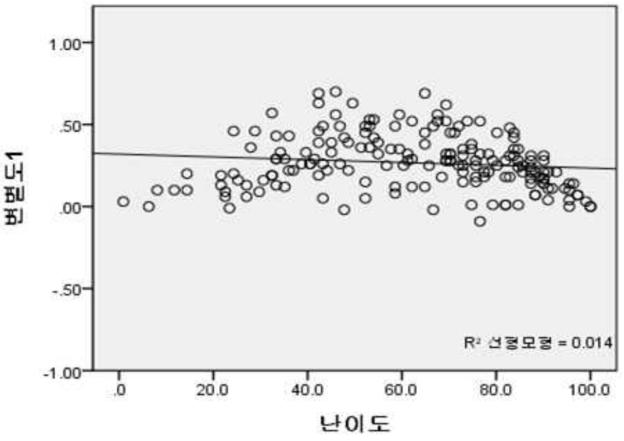

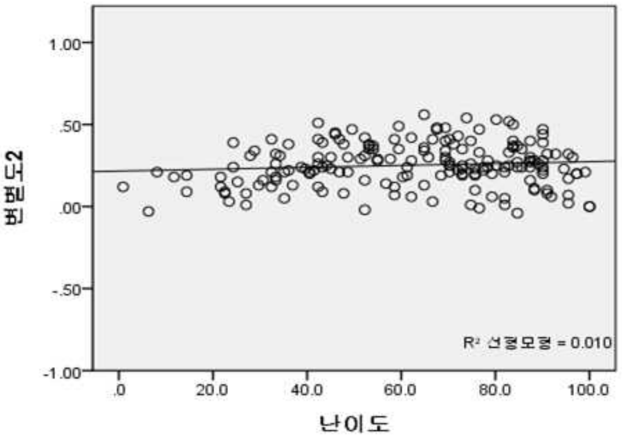

해석

- 난이도 지수와 변별도1 지수 간 상관은 -.119로 난이도 지수와 변별력의 관련성이 낮은 것으로 나타남
- 난이도 지수와 변별도2 지수 간 상관은 .102로 난이도 지수와 변별력의 관련성이 낮은 것으로 나타남

2) 과목별 난이도와 변별도 간 산포도

가) 보건프로그램 개발 및 평가 난이도와 변별도 간 산포도

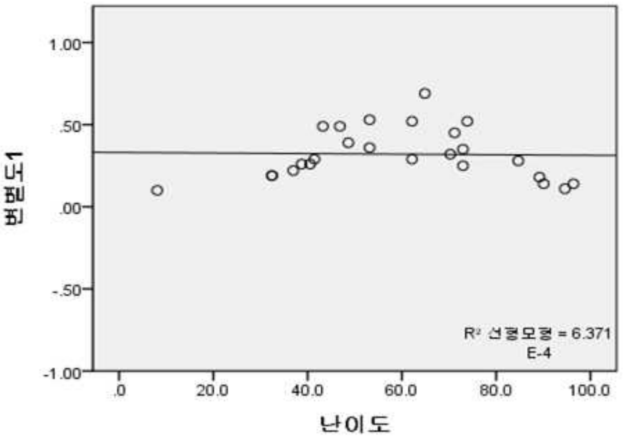

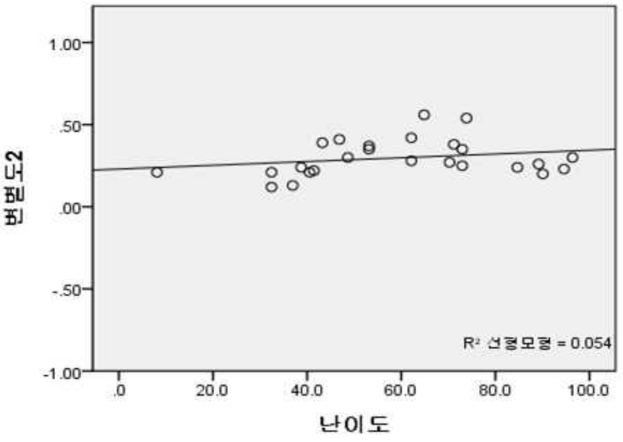

해석

- 난이도 지수와 변별도 1 지수 간 상관은 -.025 로 난이도 지수와 변별력의 관련성이 없는 것으로 나타남
- 난이도 지수와 변별도 2 지수 간 상관은 .233 으로 난이도 지수와 변별력의 관련성이 낮은 것으로 나타남

나) 보건교육방법론 난이도와 변별도 간 산포도

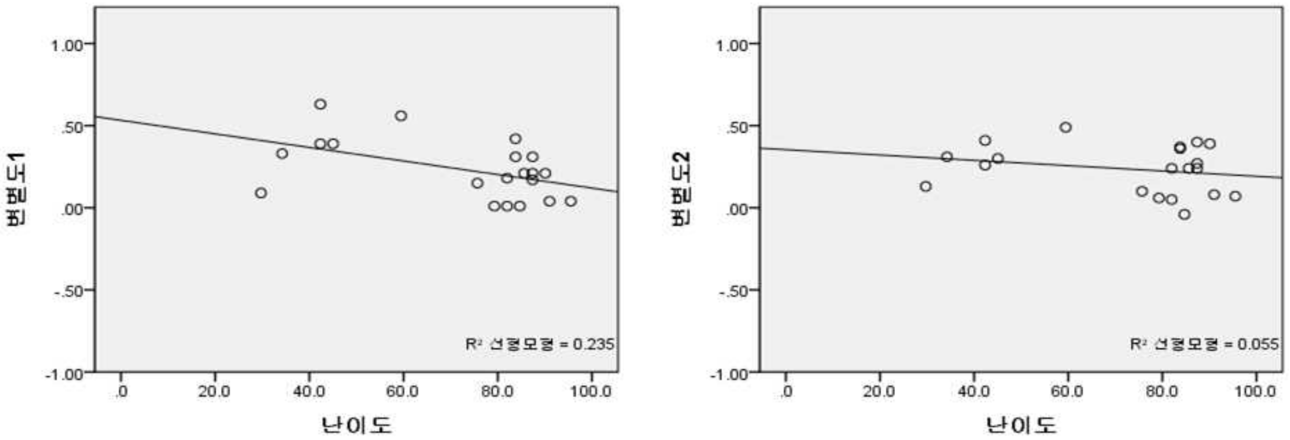

해석

- 난이도 지수와 변별도 1 지수 간 상관은 -.485*로 문항 난이도가 쉬울수록 변별력이 낮아지는 것으로 나타남
- 난이도 지수와 변별도 2 지수 간 상관은 -.235 로 난이도 지수와 변별력의 관련성이 낮은 것으로 나타남

다) 보건사업관리 난이도와 변별도 간 산포도

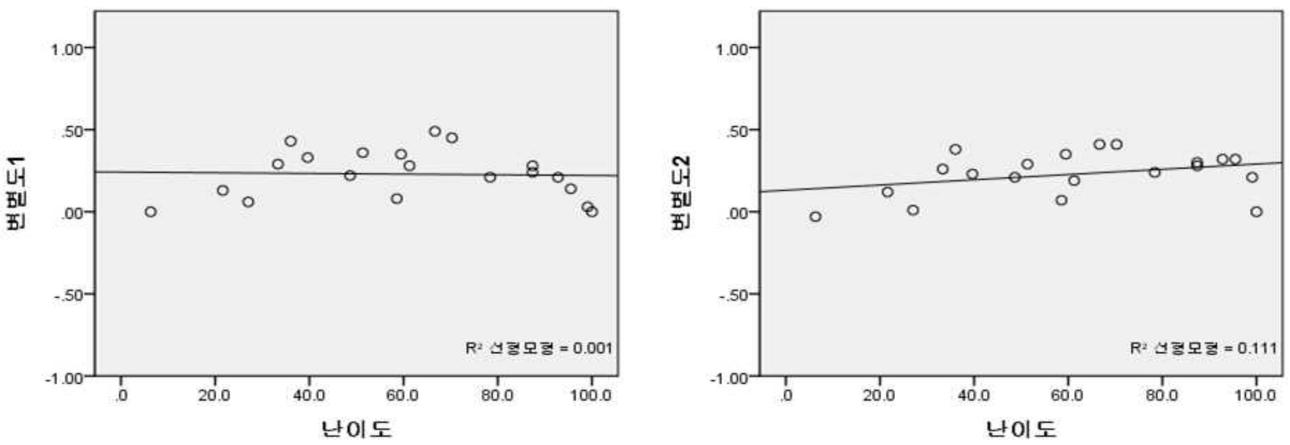

해석

- 난이도 지수와 변별도 1 지수 간 상관은 -.038 로 난이도 지수와 변별력의 관련성이 없는 것으로 나타남
- 난이도 지수와 변별도 2 지수 간 상관은 .333 으로 난이도 지수와 변별력의 관련성이 낮은 것으로 나타남

라) 보건의료법규 난이도와 변별도 간 산포도

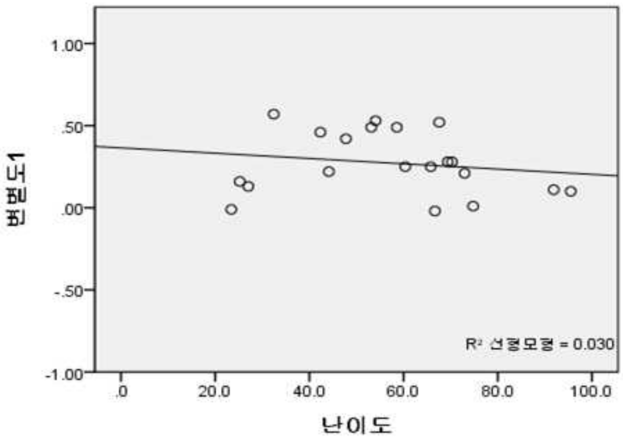

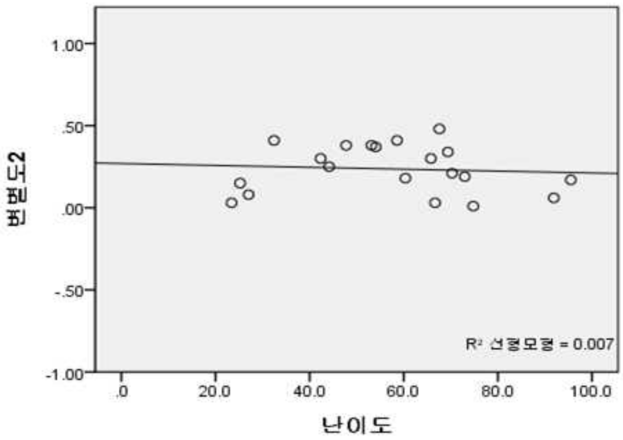

해석

- 난이도 지수와 변별도 1 지수 간 상관은 -.173 으로 난이도 지수와 변별력의 관련성이 낮은 것으로 나타남
- 난이도 지수와 변별도 2 지수 간 상관은 -.081 로 난이도 지수와 변별력의 관련성이 없는 것으로 나타남

마) 조사방법론 난이도와 변별도 간 산포도

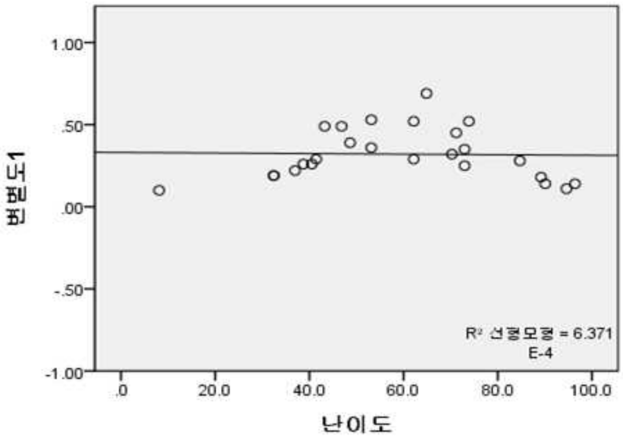

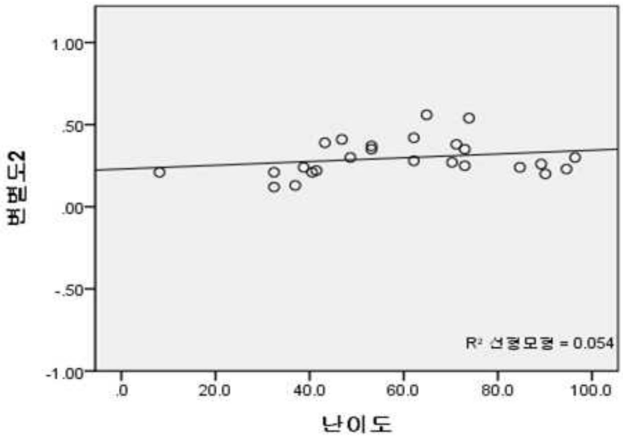

해석

- 난이도 지수와 변별도1 지수 간 상관은 -.025로 난이도 지수와 변별력의 관련성이 없는 것으로 나타남
- 난이도 지수와 변별도2 지수 간 상관은 .233으로 난이도 지수와 변별력의 관련성이 낮은 것으로 나타남

바) 보건의사소통 난이도와 변별도 간 산포도

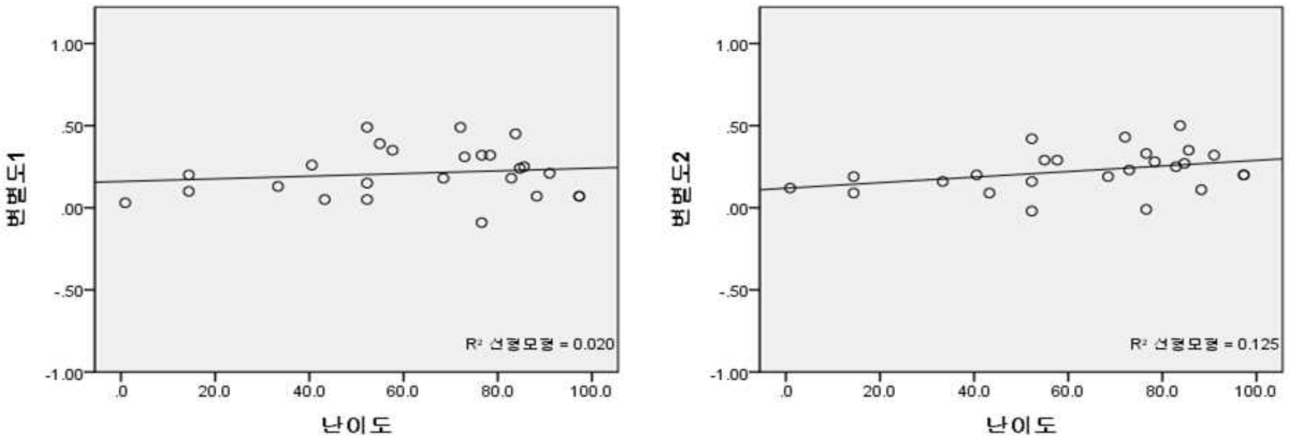

해석

- 난이도 지수와 변별도1 지수 간 상관은 .141으로 난이도 지수와 변별력의 관련성이 낮은 것으로 나타남
- 난이도 지수와 변별도2 지수 간 상관은 .354으로 난이도 지수와 변별력의 관련성이 낮은 것으로 나타남

사) 보건학 난이도와 변별도 간 산포도

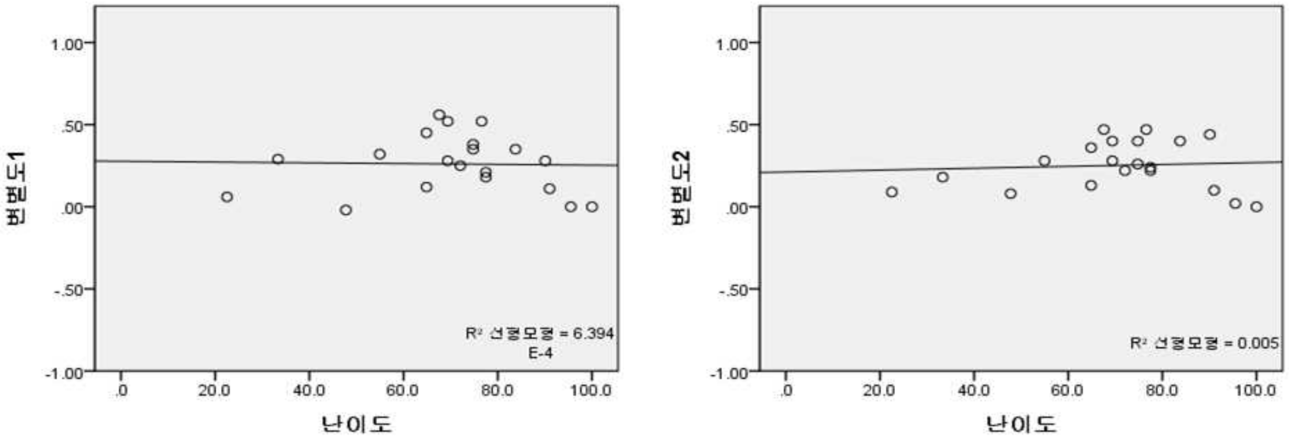

해석

- 난이도 지수와 변별도1 지수 간 상관은 -.025로 난이도 지수와 변별력의 관련성이 없는 것으로 나타남
- 난이도 지수와 변별도2 지수 간 상관은 .074로 난이도 지수와 변별력의 관련성이 없는 것으로 나타남

아) 보건교육학 난이도와 변별도 간 산포도

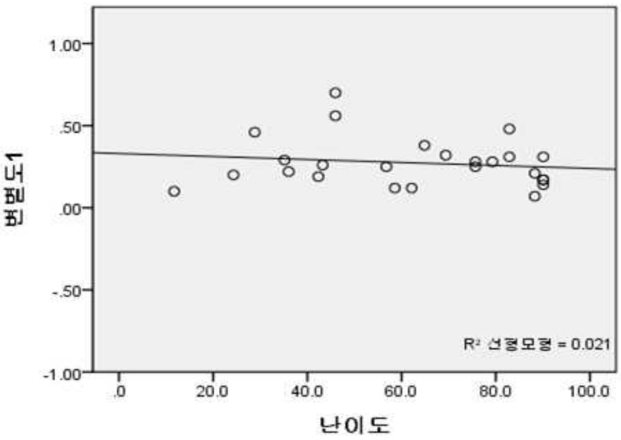

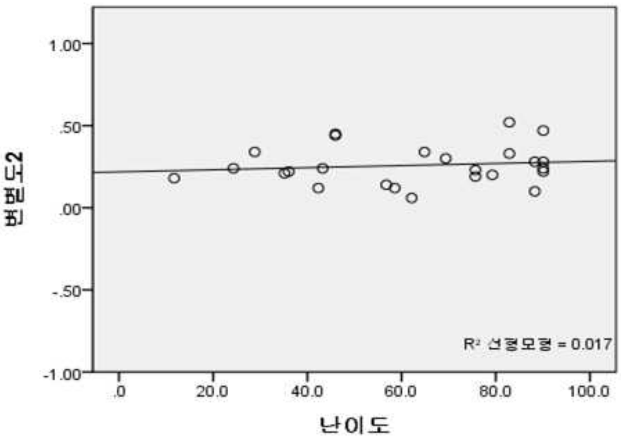

해석

- 난이도 지수와 변별도1 지수 간 상관은 -.145로 난이도 지수와 변별력의 관련성이 낮은 것으로 나타남
- 난이도 지수와 변별도2 지수 간 상관은 .129로 난이도 지수와 변별력의 관련성이 낮은 것으로 나타남

4. 신뢰도 분석

과목명	문항수	제9회	제10회	제11회	제12회	제13회
전체	180				.911	**.918**
보건프로그램 개발 및 평가	25				.662	**.699**
보건교육방법론	20				.395	**.514**
보건사업관리	20				.450	**.544**
보건의료법규	20				.563	**.620**
조사방법론	25				.743	**.680**
보건의사소통	25				.533	**.541**
보건학	20				.635	**.581**
보건교육학	25				.494	**.640**

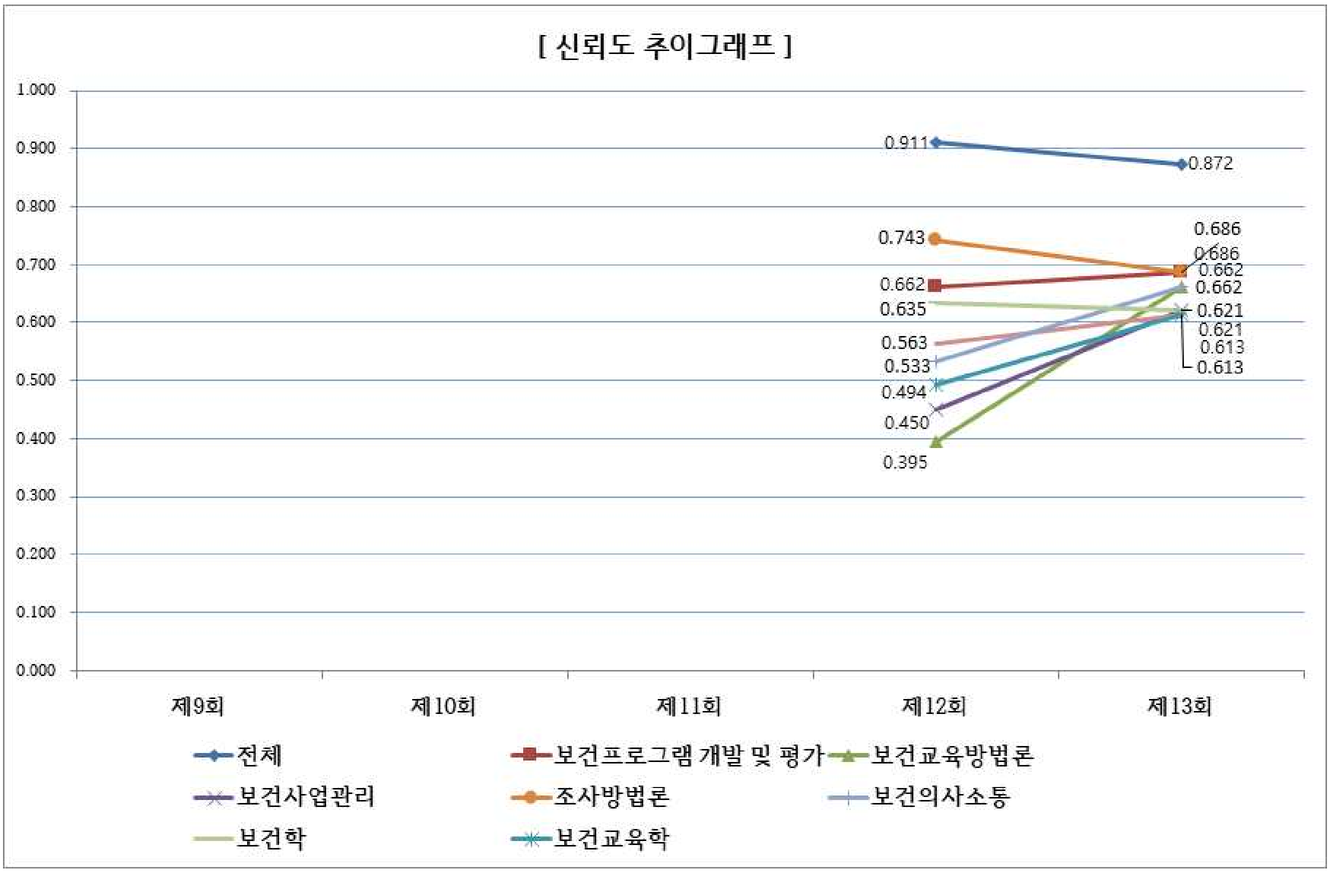

해석

- 전회 대비 보건교육사 2급 국가시험 문항의 신뢰도는 .039 감소함
- 전회 대비 보건프로그램 개발 및 평가 과목 문항의 신뢰도는 .024 증가함
- 보건교육방법론 과목 문항의 신뢰도는 .267 증가함
- 보건사업관리 과목 문항의 신뢰도는 .171 증가함
- 전회 대비 보건의료법규 과목 문항의 신뢰도는 .050 증가함
- 조사방법론 과목 문항의 신뢰도는 .057 감소함
- 보건의사소통 과목 문항의 신뢰도는 .129 증가함
- 전회 대비 보건학 과목 문항의 신뢰도는 .014 감소함
- 전회 대비 보건교육학 과목 문항의 신뢰도는 .119 증가함
