# Supplementary material for: Item difficulty index, discrimination index, and reliability of the 26 health professions licensing examinations in 2022, Korea: a psychometric study
Source: J Educ Eval Health Prof. 2023 Nov 22;20:31. doi: 10.3352/jeehp.2023.20.31 (PMC11959405; doi:10.3352/jeehp.2023.20.31)
Supplement: Supplementary file 1 — Supplement 1. Item analysis results of 26 health professions licensing examinations administered during late 2022 and early 2023. [file jeehp-20-31_Suppl1.zip › 2022│Γ╡╡ ┴a13╚╕ ║╕░╟▒│└░╗τ 3▒▐ ▒╣░í╜├╟Φ ║╨╝«░ß░·.pdf]

# 2022년도 제13회
# 보건교육사 3급 국가시험
# 문항분석 결과

국민이 신뢰하고 감동하는 시험평가기관
한국보건의료인국가시험원
KOREA HEALTH PERSONNEL LICENSING EXAMINATION INSTITUTE

# 일반 용어 정의

☐ **평균**
- 집단에서의 대표적 경향값으로 전체 값을 더하여 총 응시자로 나눈 값

☐ **표준편차**
- 평균과 각 점수의 차이인 편차들의 평균으로 점수가 흩어져 분포되어 있는 정도

☐ **추정난이도**
- 문항개발자가 예측한 정답률

☐ **검사이론**
- 검사와 검사를 구성하고 있는 문항의 양호도를 분석 및 평가하는 방법을 정의한 이론체계
- 대표적으로 고전검사이론과 문항반응이론이 있음

## 고전검사이론 용어 정의

□ **고전검사이론(Classical Test Theory; CTT)**

- 검사의 질을 분석하는 검사이론 중 한 가지로 19세기 말부터 전개되어 현재까지 주로 사용되고 있는 검사이론임
- 고전검사이론에 의한 문항과 응시자 능력 추정치는 다음과 같음

○ **문항난이도**

· 검사 문항의 쉽고 어려운 정도를 나타내는 지수
· 난이도 지수는 총 반응 수에 대한 정답 반응 수의 비율로 문항의 정답률임
· 문항난이도는 0~100까지의 값을 가짐
· 난이도 값이 큰 경우, 쉬운 문항으로 '난이도가 낮다'라고 해석하며, 난이도 값이 작은 경우, 어려운 문항으로 '난이도가 높다'라고 해석함

○ **문항변별도**

· 각 문항이 응시자의 능력 수준을 변별할 수 있는 정도를 나타내는 지수
· 문항변별도는 −1~+1까지의 값을 가지며, 1에 가까울수록 변별력 크다고 해석함
· 일반적으로 문항변별도가 0.3 이상이면 우수한 문항으로 평가함
· 구하는 방식에는 '상하위집단 구분법', '문항-총점 상관계수' 등이 있음
  1) **변별도 1(상하위구분법):** 상위 27%와 하위 27% 집단의 난이도 차이를 구하는 방식
  2) **변별도 2(상관계수법):** 문항-총점과의 상관계수로 구하는 방식

○ **신뢰도**

· 시험이 평가하고자 하는 것을 일관성 있게 측정하는가로 시험이 오차없이 정확하게 측정한 정도를 의미함
· 국시원에서는 문항의 내적일관성(Cronbach $\alpha$)으로 신뢰도를 추정하며 1에 가까울수록 신뢰도가 높다고 해석함

# 목 차


I. 시행 결과 ········ 6

1. 시험 현황 ········ 7

1) 시험명 ········ 7

2) 시험시행일 ········ 7

3) 응시현황 ········ 7

4) 과목별 문항 수, 배점 및 과락 점수 ········ 7

2. 합격률과 평균성적 ········ 7

1) 합격 및 불합격 현황 ········ 7

2) 과목별 과락자수 내역 ········ 7

3) 전회 대비 합격률과 평균성적 ········ 8

II. 문항분석 결과 ········ 9

1. 성적 ········ 10

1) 전체 성적분포도 ········ 10

2) 과목별 성적분포도 ········ 11

2. 난이도와 변별도 ········ 12

1) 전체 난이도와 변별도 ········ 15

2) 과목별 난이도와 변별도 ········ 15

3) 지식수준별 난이도와 변별도 ········ 26

3. 난이도와 변별도 간 산포도 ········ 34

1) 전체 난이도와 변별도 간 산포도 ········ 34

2) 과목별 난이도와 변별도 간 산포도 ········ 34

4. 신뢰도 분석 ········ 37

# I. 시행 결과

## 1. 시험 현황

1) 시험명: 2022년도 제12회 보건교육사 3급 국가시험

2) 시험시행일: 2022년 2월 12일

3) 응시현황

| 응시대상자수 | 결시자수 | 부정행위자수 | 응시자 준수사항 위반자 수 | | 응시자수 (%) |
|---|---|---|---|---|---|
| | | | 휴대폰 소지 | 신분증 미지참 | |
| 1,394 | 288 | - | - | - | 1,105 (79.3) |

4) 과목별 문항 수, 배점 및 과락 점수

| 교 시 | 과 목 명 | 문제 수 | 배점 | 총점 | 합격자 점수기준 | |
|---|---|---|---|---|---|---|
| | | | | | 과목 과락기준 | 총점 합격기준 |
| 1교시 | 보건프로그램 개발 및 평가 | 30 | 1 | 30 | 12 | 66 |
| | 보건학 | 30 | 1 | 30 | 12 | |
| | 보건교육학 | 30 | 1 | 30 | 12 | |
| | 보건의료법규 | 20 | 1 | 20 | 8 | |
| 계 | | 110 | | 110 | | |

## 2. 합격률과 평균성적

1) 합격 및 불합격 현황

| 합격자수 (%) | 불합격자수(%) | | | | 채점보류자수 |
|---|---|---|---|---|---|
| | 평락 | 과락 | 기권 | 계 | |
| 642 (58.1) | 448 (40.5) | 15 (1.4) | - (0.0) | 469 (41.9) | 1 (0.0) |

2) 과목별 과락자수 내역

| 과목명 / 과락자수 | 보건프로그램 개발 및 평가 | 보건학 | 보건교육학 | 보건의료법규 |
|---|---|---|---|---|
| 과목별 과락자 수 | - | - | - | 15 |
| 전과목 과락자 수 | - | | | |

3) 전회 대비 합격률과 평균성적

| 회차 | 년도 | 합격률(%) | 평균성적 | 표준편차 | 백분율 환산점수 |
|---|---|---|---|---|---|
| 제9회 | 2017 | 74.9 | 72.5 | 11.7 | 65.9 |
| 제10회 | 2018 | 62.6 | 68.9 | 12.7 | 62.6 |
| 제11회 | 2019 | 50.1 | 64.9 | 12.1 | 59.0 |
| 제12회 | 2021 | 67.4 | 70.4 | 12.1 | 64.0 |
| **제13회** | **2022** | **58.1** | **68.5** | **12.3** | **62.3** |

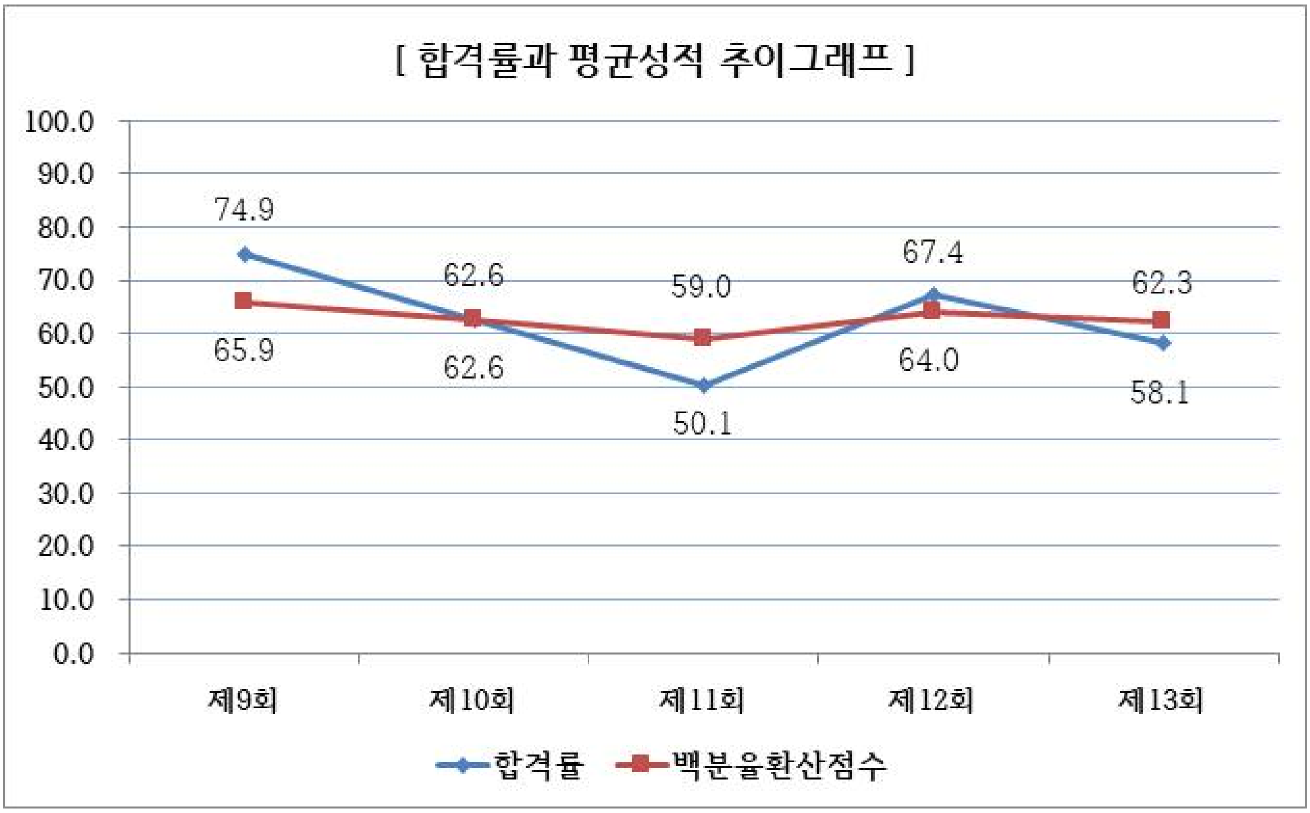


**해석**

- 전년 대비 합격률은 9.3%, 백분율 환산점수는 1.7 점 감소함
- 표준편차는 0.2 증가함

# Ⅱ. 문항분석 결과

## 1. 성적

### 1) 전체 성적분포도

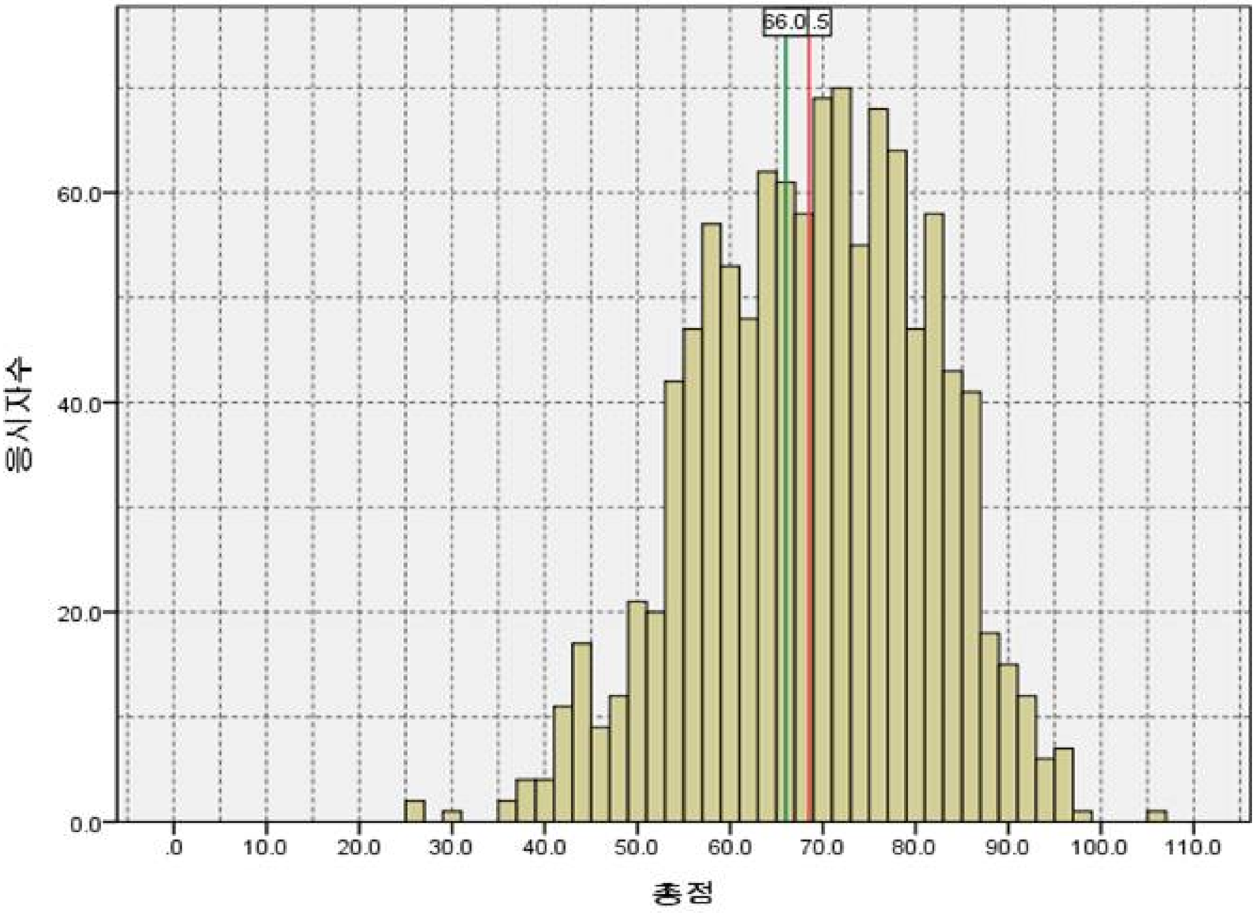


| 응시자 | 총점 | 합격선 | 평균성적 | 표준편차 |
|---|---|---|---|---|
| 1,406* | 110 | 66 | 68.5 | 12.3 |

*응시자 1,405명에 채점보류자 1인을 포함한 숫자임

2) 과목별 성적분포도

가) 보건프로그램 개발 및 평가

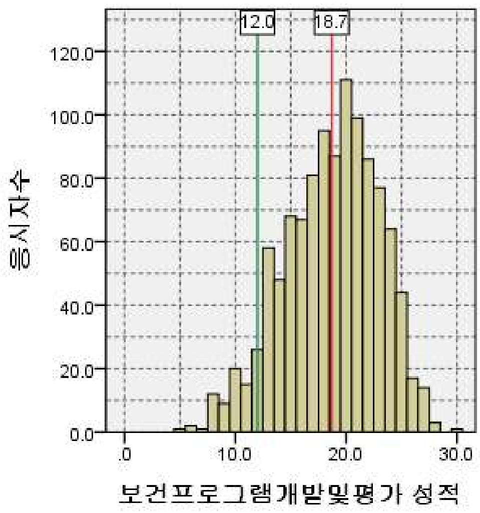


| 총점 | 과락기준 | 평균성적 | 표준편차 |
|---|---|---|---|
| 30 | 12 | 18.7 | 4.3 |

나) 보건학

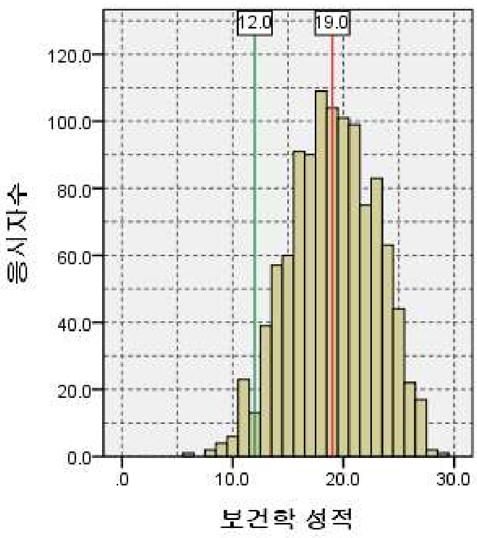


| 총점 | 과락기준 | 평균성적 | 표준편차 |
|---|---|---|---|
| 30 | 12 | 19.0 | 3.9 |

다) 보건교육학

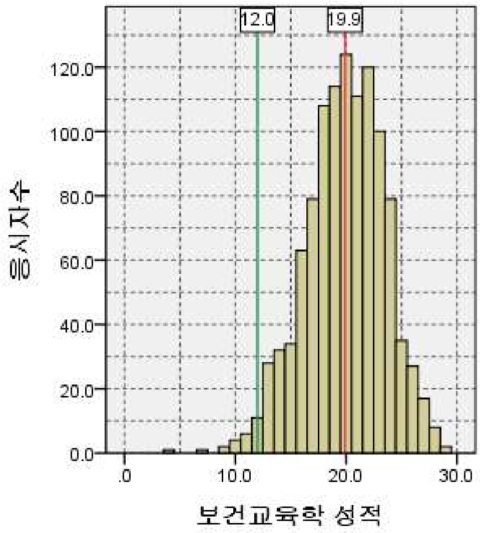


| 총점 | 과락기준 | 평균성적 | 표준편차 |
|---|---|---|---|
| 30 | 12 | 19.9 | 3.6 |

보건의료법규

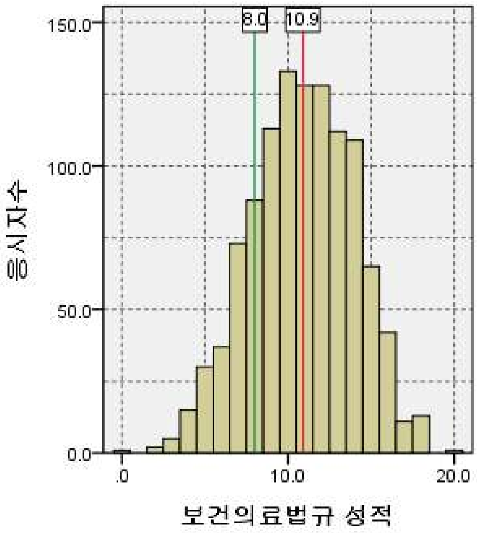


| 총점 | 과락기준 | 평균성적 | 표준편차 |
|---|---|---|---|
| 20 | 8 | 10.9 | 3.1 |

## 2. 난이도와 변별도

### 1) 전체 난이도와 변별도

가) 전회 대비 전체 난이도와 변별도

| 회차 | 난이도 | | 변별도1 | | 변별도2 | |
|---|---|---|---|---|---|---|
| | 평균 | 표준편차 | 평균 | 표준편차 | 평균 | 표준편차 |
| 제9회 | 65.9 | 20.6 | 0.24 | 0.15 | 0.22 | 0.14 |
| 제10회 | 62.6 | 20.5 | 0.27 | 0.13 | 0.23 | 0.11 |
| 제11회 | 59.0 | 24.1 | 0.27 | 0.15 | 0.25 | 0.12 |
| 제12회 | 64.0 | 23.1 | 0.27 | 0.14 | 0.26 | 0.11 |
| **제13회** | **62.3** | **22.1** | **0.27** | **0.16** | **0.26** | **0.12** |

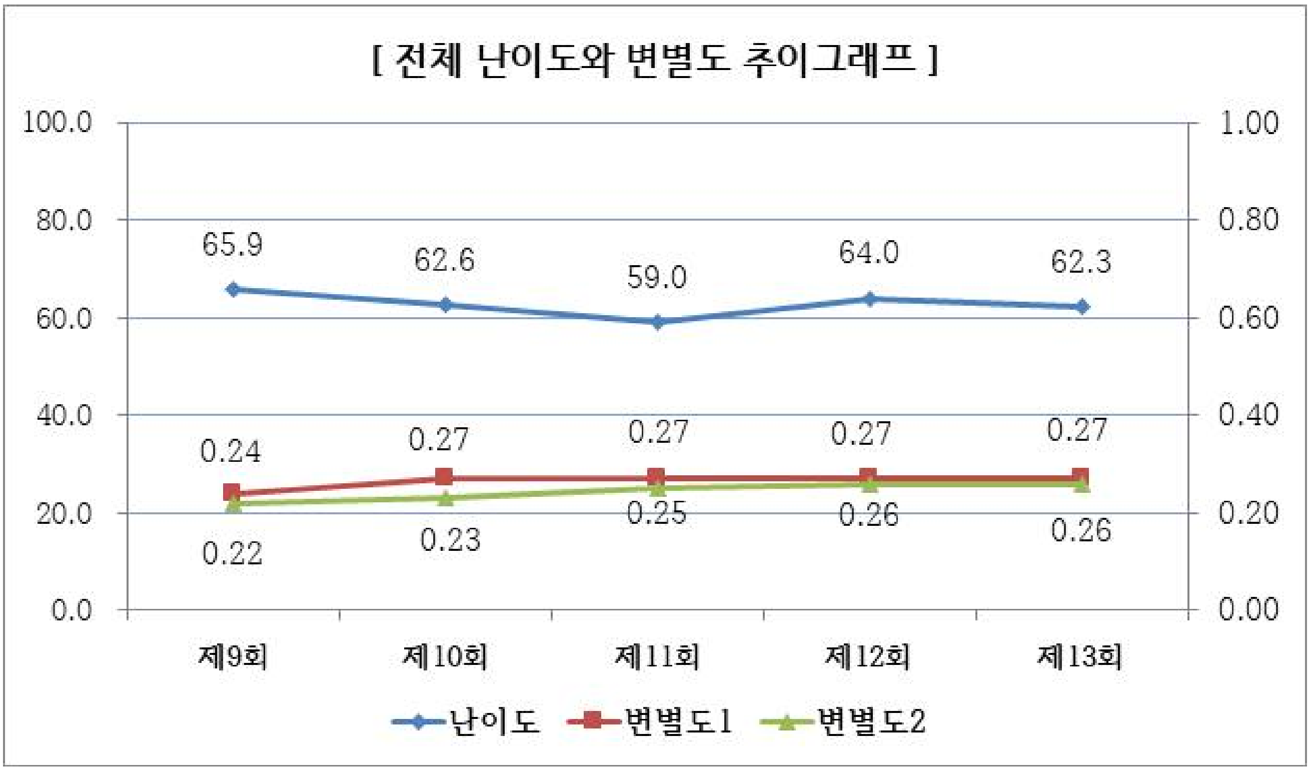


**해석**

- 전년 대비 난이도 지수는 1.7 감소함
- 변별도 1, 2 지수 모두 변화 없음

## 나) 전체 난이도와 변별도 분포도 및 비율분석

### (1) 전체 난이도 분포도 및 비율분석

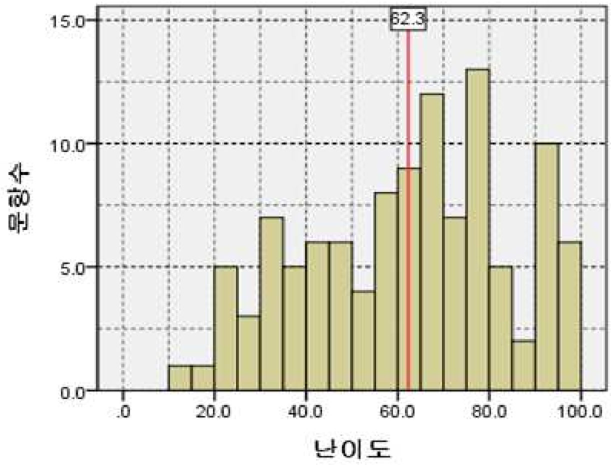


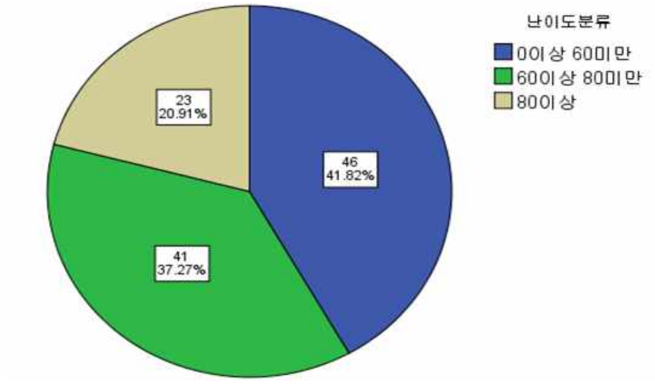


| 총점 | 난이도 | 표준편차 |
|---|---|---|
| 110 | 62.3 | 22.1 |

| 난이도 | 문항수 | 비율(%) |
|---|---|---|
| 0~60미만 | 46 | 41.8 |
| 60~80미만 | 41 | 37.3 |
| 80~100 | 23 | 20.9 |
| 전체 | 110 | 100.0 |

### (2) 전체 변별도1 분포도 및 비율분석

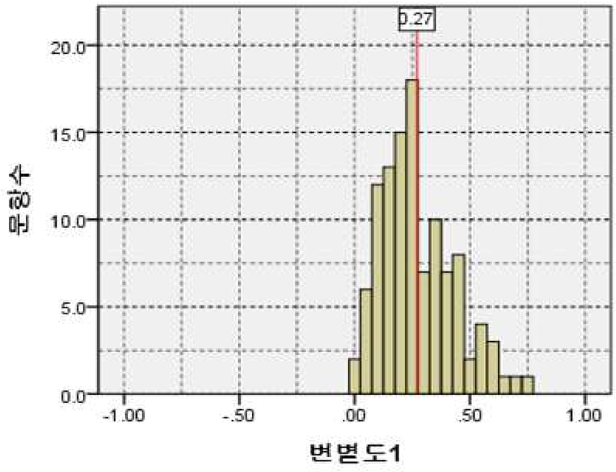


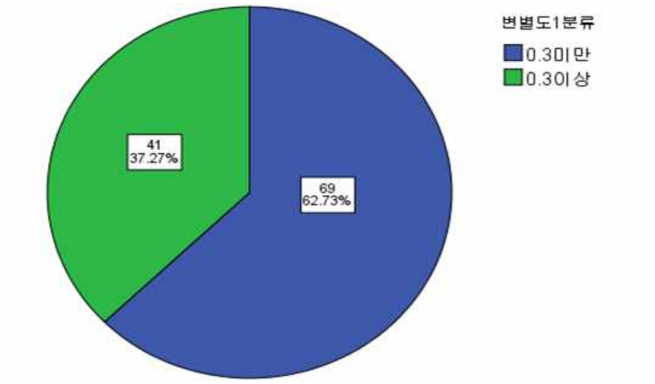


| 총점 | 변별도1 | 표준편차 |
|---|---|---|
| 110 | .27 | .16 |

| 변별도1 | 문항수 | 비율(%) |
|---|---|---|
| 0.3미만 | 69 | 62.7 |
| 0.3이상 | 41 | 37.3 |
| 전체 | 110 | 100.0 |

(3) 전체 변별도2 분포도 및 비율분석

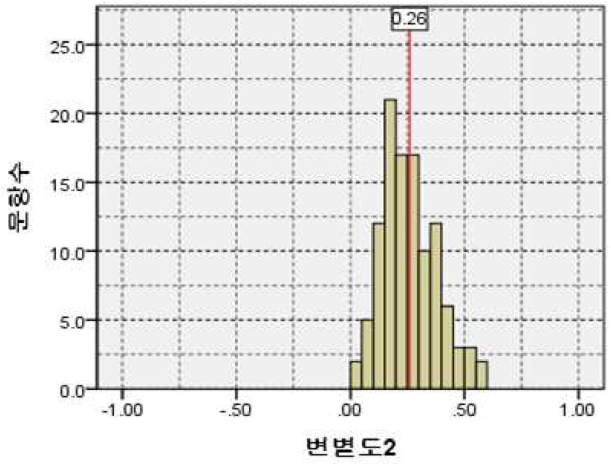


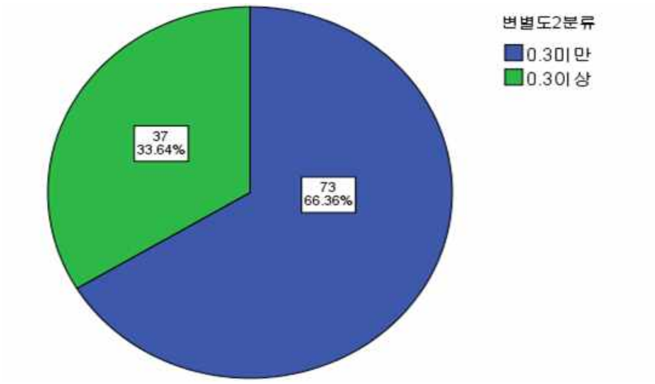


| 총점 | 변별도2 | 표준편차 | 변별도2 | 문항수 | 비율(%) |
|---|---|---|---|---|---|
| 110 | .26 | .12 | 0.3미만 | 73 | 66.4 |
| | | | 0.3이상 | 37 | 33.6 |
| | | | 전체 | 110 | 100.0 |

**해석**

- 난이도 지수가 80 에서 100 사이인 문항이 전체 110 중 23 문항으로 가장 적었으며, 60 이상 80 미만인 문항이 41 문항, 60 미만인 문항이 46 문항인 것으로 나타남
- 변별도 1 지수를 기준으로 분류하였을 때, 0.3 미만인 문항이 69 문항으로 0.3 이상인 문항이 41 문항인 것에 비해 더 많이 나타남
- 변별도 2 지수를 기준으로 분류하였을 때, 0.3 미만인 문항이 73 문항으로 0.3 이상인 문항이 37 문항인 것에 비해 더 많이 나타남

2) 과목별 난이도와 변별도

가) 전회 대비 과목별 난이도와 변별도

(1) 전회 대비 보건프로그램 개발 및 평가 난이도와 변별도

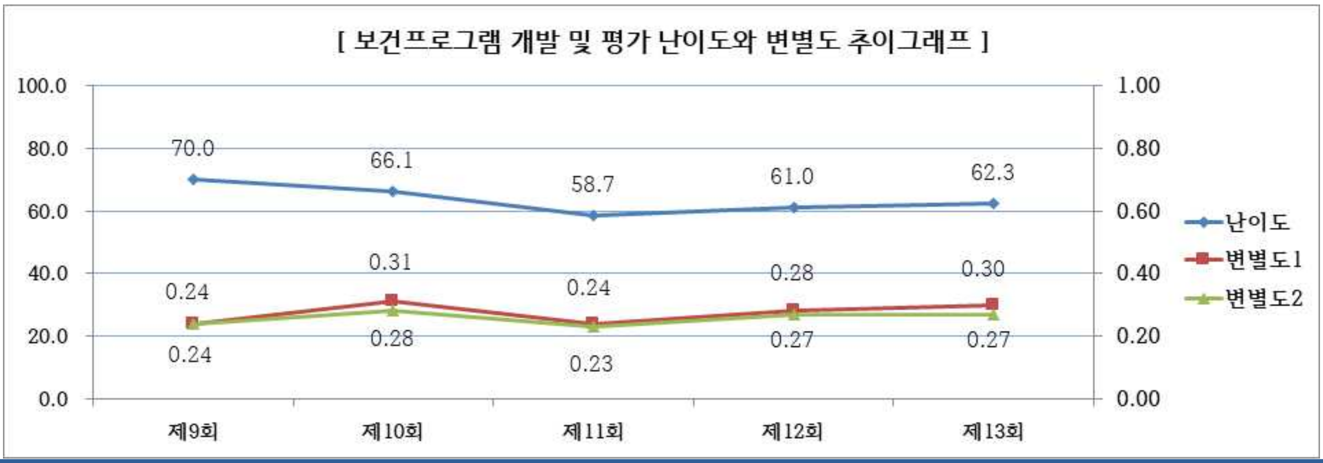


| 회차 | 난이도 | | 변별도1 | | 변별도2 | |
|---|---|---|---|---|---|---|
| | 평균 | 표준편차 | 평균 | 표준편차 | 평균 | 표준편차 |
| 제9회 | 70.0 | 19.5 | 0.24 | 0.18 | 0.24 | 0.16 |
| 제10회 | 66.1 | 19.3 | 0.31 | 0.13 | 0.28 | 0.12 |
| 제11회 | 58.7 | 26.1 | 0.24 | 0.13 | 0.23 | 0.11 |
| 제12회 | 61.0 | 25.7 | 0.28 | 0.15 | 0.27 | 0.11 |
| **제13회** | **62.3** | **18.3** | **0.30** | **0.17** | **0.27** | **0.14** |

(2) 전회 대비 보건학 난이도와 변별도

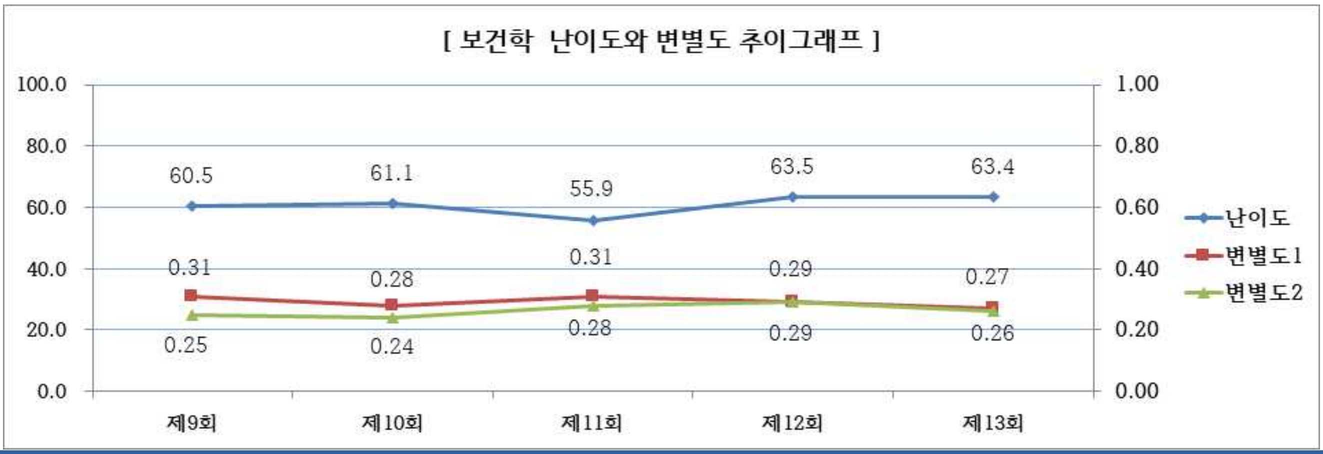


| 회차 | 난이도 | | 변별도1 | | 변별도2 | |
|---|---|---|---|---|---|---|
| | 평균 | 표준편차 | 평균 | 표준편차 | 평균 | 표준편차 |
| 제9회 | 60.5 | 15.7 | 0.31 | 0.14 | 0.25 | 0.11 |
| 제10회 | 61.1 | 18.3 | 0.28 | 0.13 | 0.24 | 0.12 |
| 제11회 | 55.9 | 22.2 | 0.31 | 0.18 | 0.28 | 0.14 |
| 제12회 | 63.5 | 24.0 | 0.29 | 0.14 | 0.29 | 0.11 |
| **제13회** | **63.4** | **23.2** | **0.27** | **0.15** | **0.26** | **0.12** |

(3) 전회 대비 보건교육학 난이도와 변별도

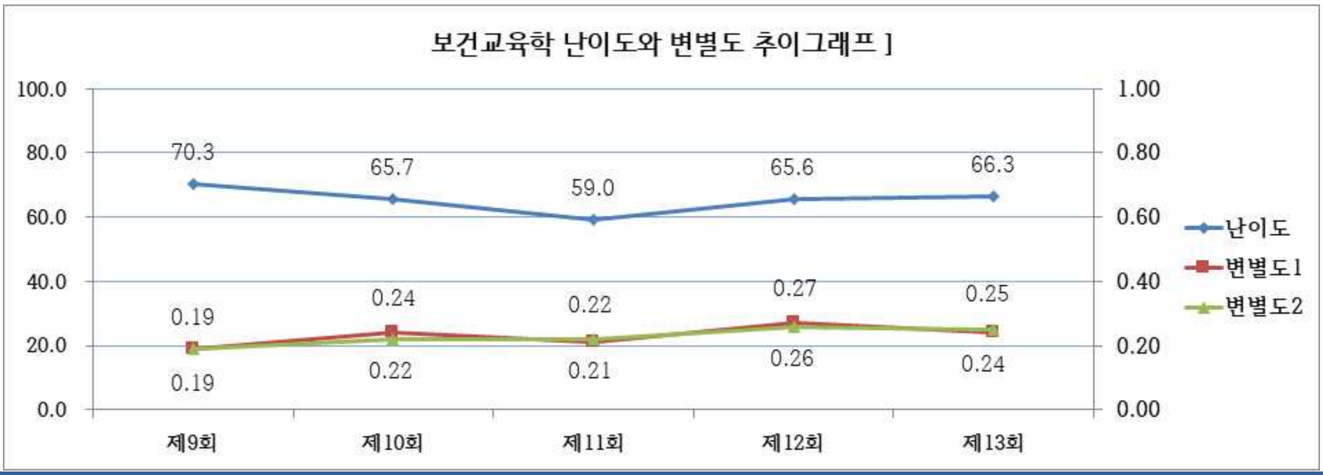


| 회차 | 난이도 | | 변별도1 | | 변별도2 | |
|---|---|---|---|---|---|---|
| | 평균 | 표준편차 | 평균 | 표준편차 | 평균 | 표준편차 |
| 제9회 | 70.3 | 23.5 | 0.19 | 0.15 | 0.19 | 0.14 |
| 제10회 | 65.7 | 21.8 | 0.24 | 0.11 | 0.22 | 0.08 |
| 제11회 | 59.0 | 27.1 | 0.21 | 0.14 | 0.22 | 0.12 |
| 제12회 | 65.6 | 21.2 | 0.27 | 0.13 | 0.26 | 0.10 |
| **제13회** | **66.3** | **23.8** | **0.24** | **0.16** | **0.25** | **0.11** |

(4) 전회 대비 보건의료법규 난이도와 변별도

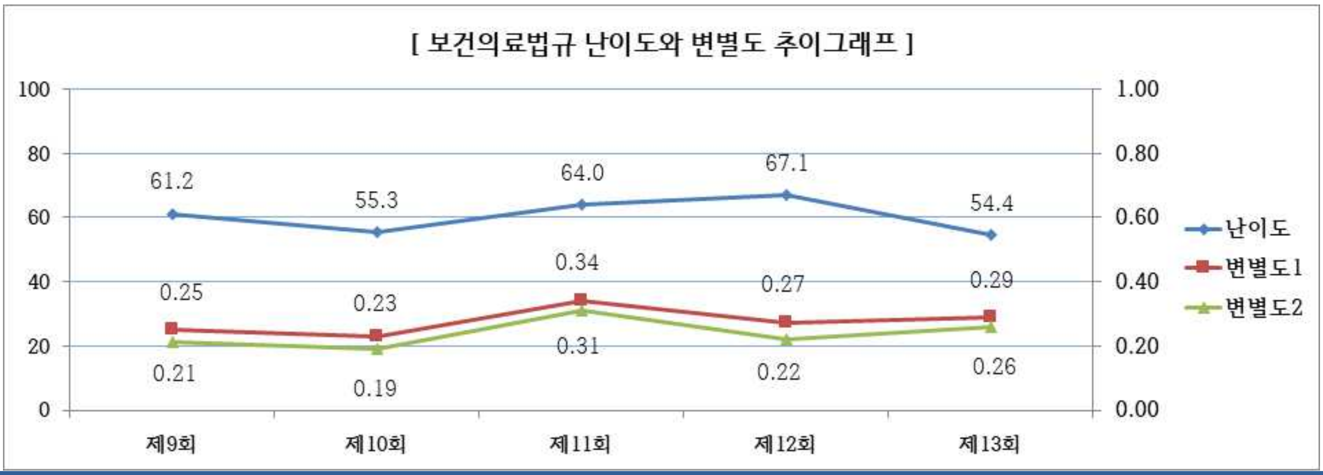


| 회차 | 난이도 | | 변별도1 | | 변별도2 | |
|---|---|---|---|---|---|---|
| | 평균 | 표준편차 | 평균 | 표준편차 | 평균 | 표준편차 |
| 제9회 | 61.2 | 22.7 | 0.25 | 0.12 | 0.21 | 0.10 |
| 제10회 | 55.3 | 22.8 | 0.23 | 0.14 | 0.19 | 0.12 |
| 제11회 | 64.0 | 19.1 | 0.34 | 0.13 | 0.31 | 0.08 |
| 제12회 | 67.1 | 21.2 | 0.27 | 0.13 | 0.22 | 0.10 |
| **제13회** | **54.4** | **22.3** | **0.29** | **0.14** | **0.26** | **0.10** |

**해석**

- 전회 대비 보건프로그램 개발 및 평가, 보건교육학 과목의 난이도 지수는 각각 1.3, 0.7 증가하였으며, 보건학, 보건의료법규 과목의 난이도 지수는 각각 0.1, 12.7 감소함
- 보건프로그램 개발 및 평가, 보건의료법규 과목의 변별도 1 지수는 각각 0.02 증가하였으며, 보건학, 보건교육학 과목의 변별도 1 지수는 각각 0.02, 0.03 감소함
- 보건프로그램 개발 및 평가 과목의 변별도 2 지수는 변화 없었으며, 보건학, 보건교육학 과목의 변별도 2 지수는 각각 0.03, 0.01 감소함. 보건의료법규 과목의 변별도 2 지수는 0.04 증가함

## 나) 과목별 난이도와 변별도 분포도 및 비율분석

### (1) 보건프로그램 개발 및 평가 난이도와 변별도 분포도 및 비율분석

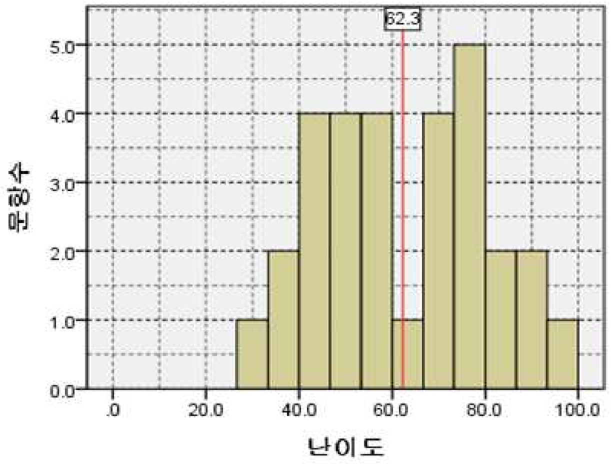


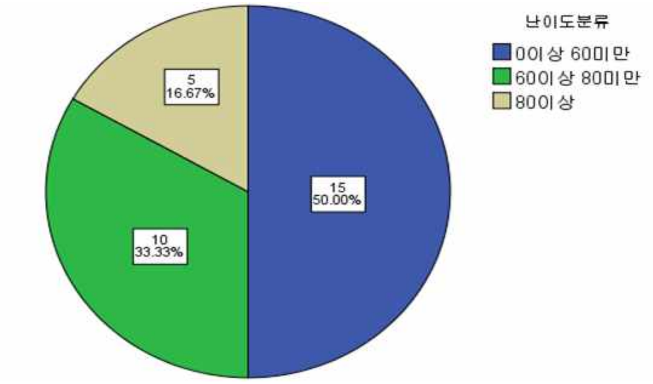


| 총점 | 난이도 | 표준편차 |
|---|---|---|
| 30 | 62.3 | 18.3 |

| 난이도 | 문항수 | 비율(%) |
|---|---|---|
| 0~60미만 | 15 | 50.0 |
| 60~80미만 | 10 | 33.3 |
| 80~100 | 5 | 16.7 |
| 전체 | 30 | 100.0 |

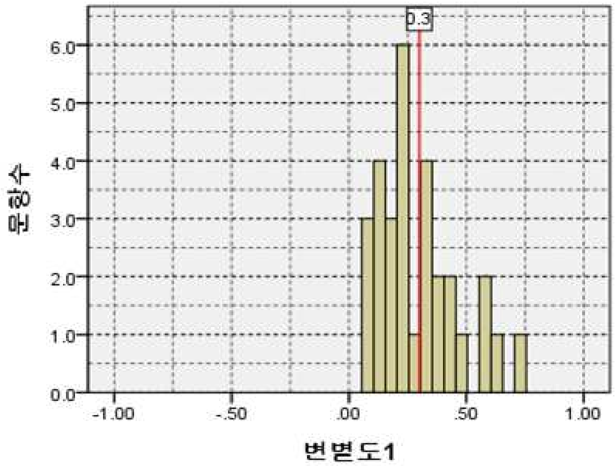


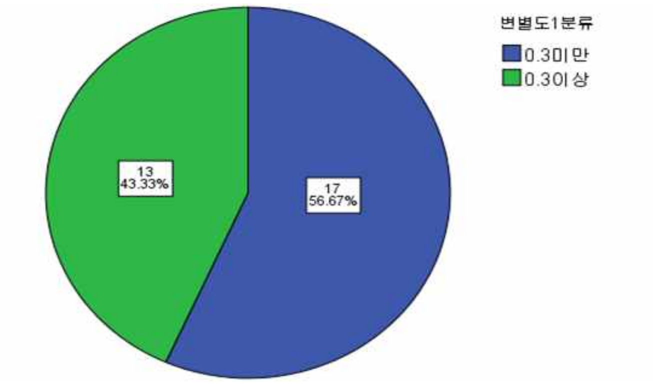


| 총점 | 변별도1 | 표준편차 |
|---|---|---|
| 30 | .30 | .17 |

| 변별도1 | 문항수 | 비율(%) |
|---|---|---|
| 0.3미만 | 17 | 56.7 |
| 0.3이상 | 13 | 43.3 |
| 전체 | 30 | 100.0 |

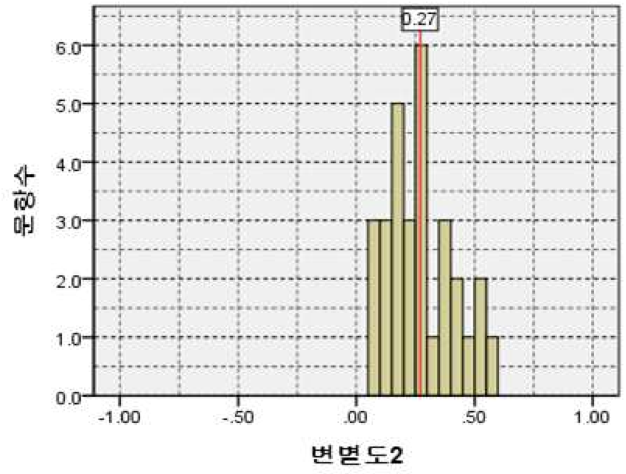


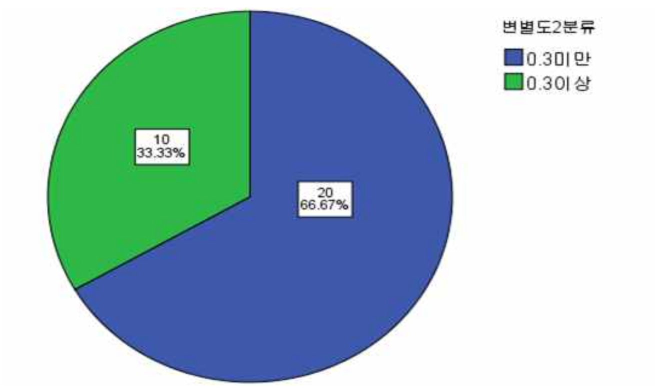


| 총점 | 변별도2 | 표준편차 |
|---|---|---|
| 30 | .27 | .14 |

| 변별도2 | 문항수 | 비율(%) |
|---|---|---|
| 0.3미만 | 20 | 66.7 |
| 0.3이상 | 10 | 33.3 |
| 전체 | 30 | 100.0 |

**해석**

- 보건프로그램 개발 및 평가 과목에서 난이도 지수가 80 에서 100 사이인 문항이 전체 30 문항 중 5 문항으로 가장 적었으며, 다음으로 60 이상 80 미만인 문항이 10 문항, 60 미만인 문항은 15 문항으로 나타남
- 변별도 1 지수를 기준으로 분류하였을 때, 0.3 미만인 문항이 17 문항으로 0.3 이상인 문항이 13 문항인 것에 비해 더 많이 나타남
- 변별도 2 지수를 기준으로 분류하였을 때, 0.3 미만인 문항이 20 문항으로 0.3 이상인 문항이 10 문항인 것에 비해 더 많이 나타남

(2) 보건학 난이도와 변별도 분포도 및 비율분석

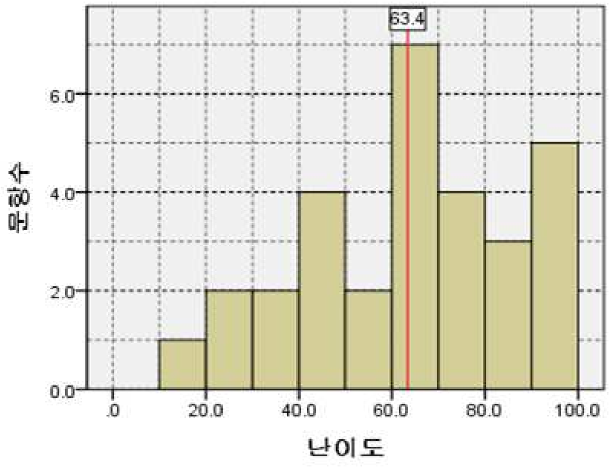


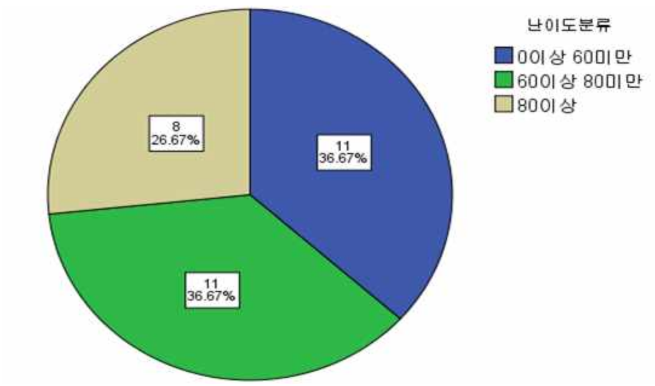


| 총점 | 난이도 | 표준편차 |
|---|---|---|
| 30 | 63.4 | 23.2 |

| 난이도 | 문항수 | 비율(%) |
|---|---|---|
| 0~60미만 | 11 | 36.7 |
| 60~80미만 | 11 | 36.7 |
| 80~100 | 8 | 26.7 |
| 전체 | 30 | 100.0 |

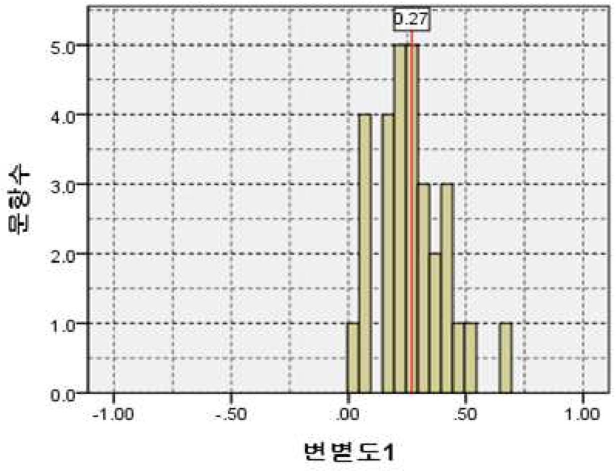


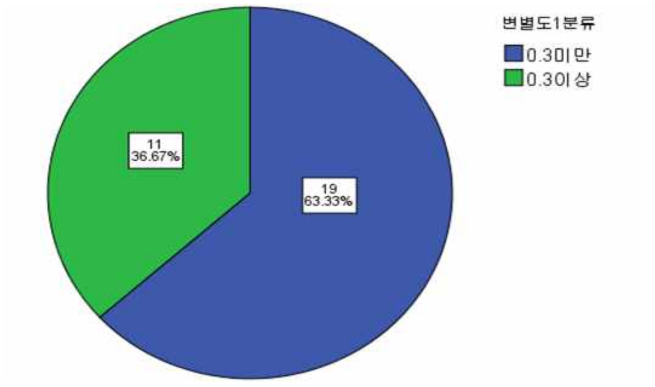


| 총점 | 변별도1 | 표준편차 |
|---|---|---|
| 30 | .27 | .15 |

| 변별도1 | 문항수 | 비율(%) |
|---|---|---|
| 0.3미만 | 19 | 63.3 |
| 0.3이상 | 11 | 36.7 |
| 전체 | 30 | 100.0 |

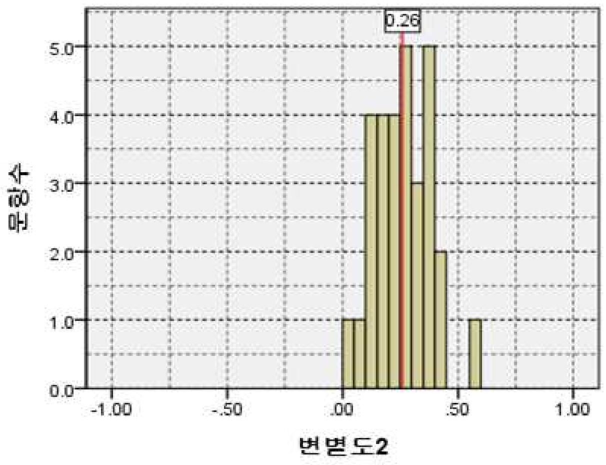


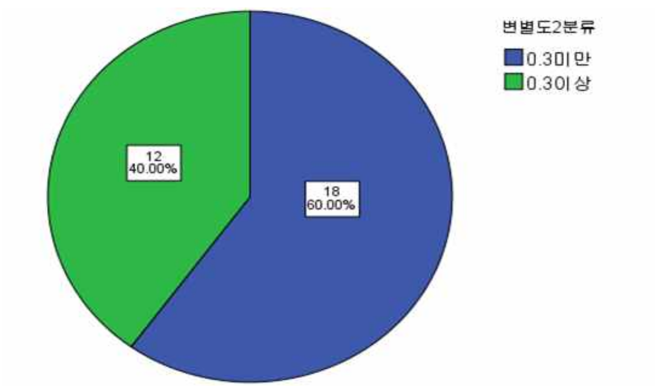


| 총점 | 변별도2 | 표준편차 |
|---|---|---|
| 30 | .26 | .12 |

| 변별도2 | 문항수 | 비율(%) |
|---|---|---|
| 0.3미만 | 18 | 60.0 |
| 0.3이상 | 12 | 40.0 |
| 전체 | 30 | 100.0 |

**해석**

- 보건학 과목에서 난이도 지수가 80에서 100 사이인 문항이 전체 30문항 중 8문항으로 나타났으며, 다음으로 60 이상 80 미만인 문항이 11문항, 60 미만인 문항은 11문항으로 나타남
- 변별도1 지수를 기준으로 분류하였을 때, 0.3 미만인 문항이 19문항, 0.3 이상인 문항이 11문항인 것에 비해 더 많이 나타남
- 변별도2 지수를 기준으로 분류하였을 때, 0.3 미만인 문항이 18문항으로 0.3 이상인 문항이 12문항인 것에 비해 더 많이 나타남

(3) 보건교육학 난이도와 변별도 분포도 및 비율분석

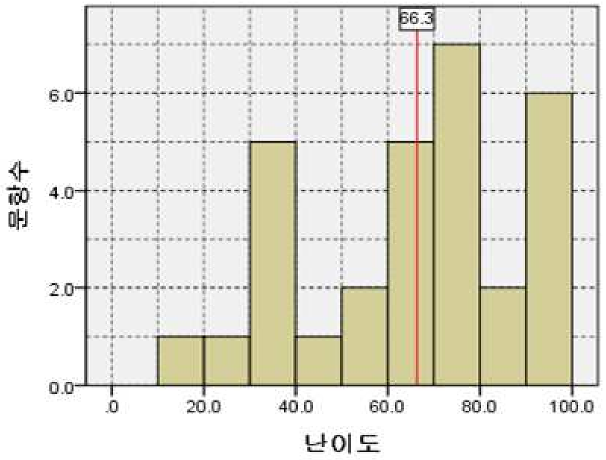


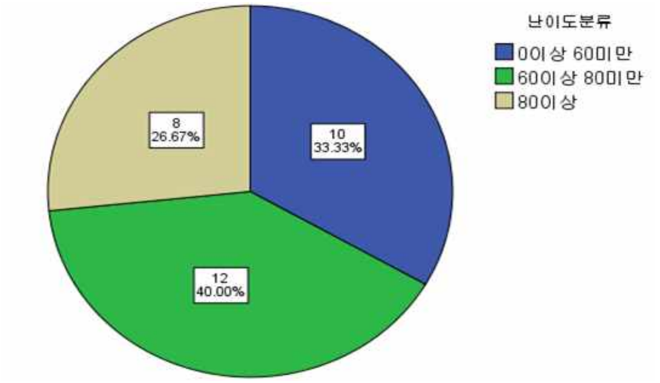


| 총점 | 난이도 | 표준편차 |
|---|---|---|
| 30 | 66.3 | 23.8 |

| 난이도 | 문항수 | 비율(%) |
|---|---|---|
| 0~60미만 | 10 | 33.3 |
| 60~80미만 | 12 | 40.0 |
| 80~100 | 8 | 26.7 |
| 전체 | 30 | 100.0 |

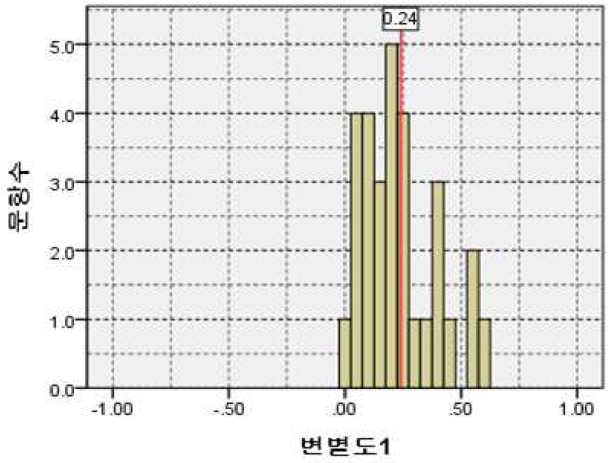


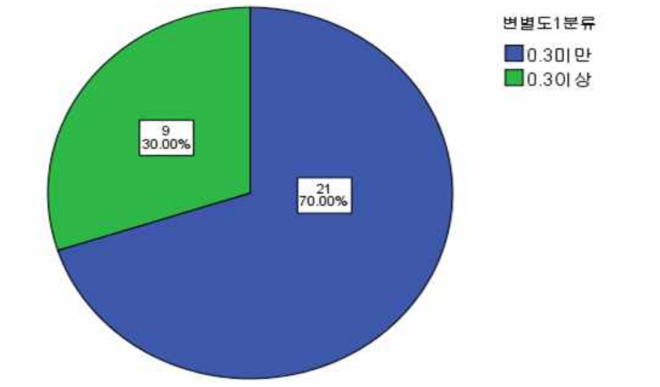


| 총점 | 변별도1 | 표준편차 |
|---|---|---|
| 30 | .24 | .16 |

| 변별도1 | 문항수 | 비율(%) |
|---|---|---|
| 0.3미만 | 21 | 70.0 |
| 0.3이상 | 9 | 30.0 |
| 전체 | 30 | 100.0 |

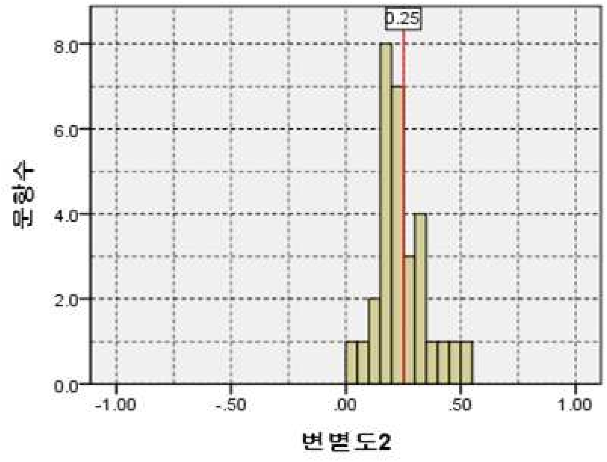


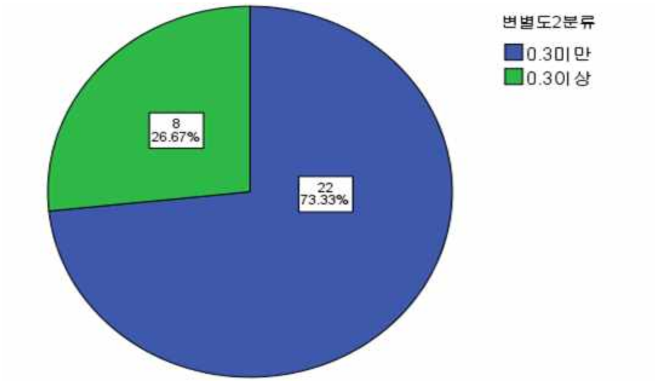


| 총점 | 변별도2 | 표준편차 |
|---|---|---|
| 30 | .25 | .11 |

| 변별도2 | 문항수 | 비율(%) |
|---|---|---|
| 0.3미만 | 22 | 73.3 |
| 0.3이상 | 8 | 26.7 |
| 전체 | 30 | 100.0 |

**해석**

- 보건교육학 과목에서 난이도 지수가 80에서 100 사이인 문항이 전체 30문항 중 8문항으로 나타났으며, 다음으로 60이상 80미만인 문항이 12문항, 60미만인 문항은 10문항으로 나타남
- 변별도1 지수를 기준으로 분류하였을 때, 0.3미만인 문항이 21문항으로 0.3 이상인 문항이 9문항인 것에 비해 더 많이 나타남
- 변별도2 지수를 기준으로 분류하였을 때, 0.3미만인 문항이 22문항으로 0.3 이상인 문항이 8문항인 것에 비해 더 많이 나타남

(4) 보건의료법규 난이도와 변별도 분포도 및 비율분석

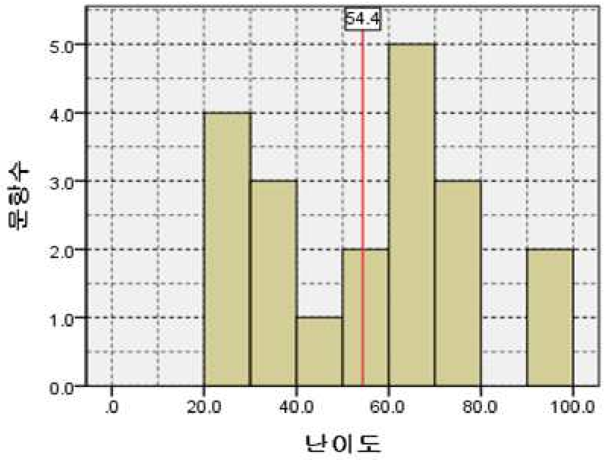


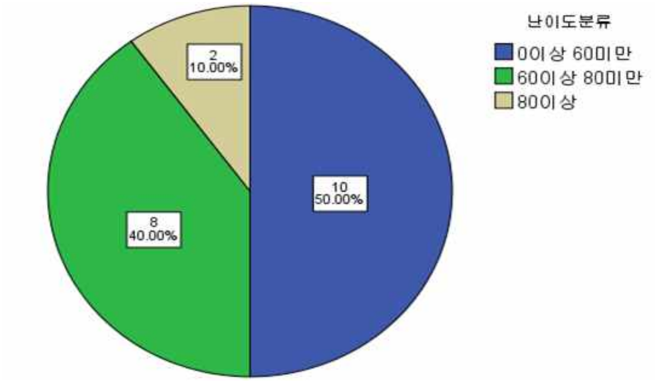


| 총점 | 난이도 | 표준편차 | 난이도 | 문항수 | 비율(%) |
|---|---|---|---|---|---|
| 20 | 54.4 | 22.3 | 0~60미만 | 10 | 50.0 |
| | | | 60~80미만 | 8 | 40.0 |
| | | | 80~100 | 2 | 10.0 |
| | | | 전체 | 20 | 100.0 |

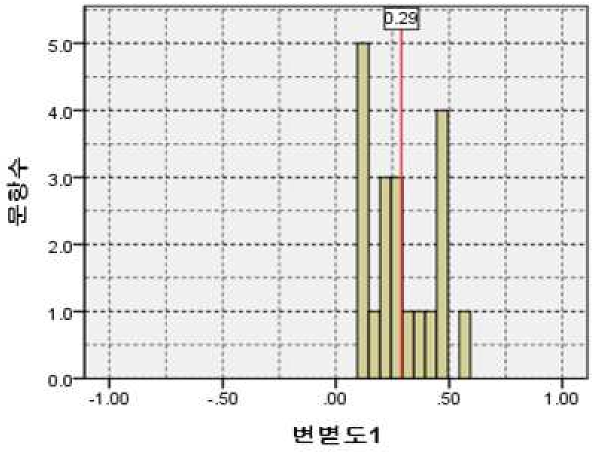


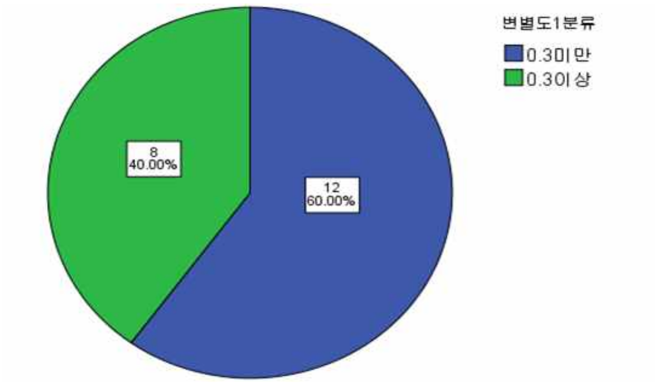


| 총점 | 변별도1 | 표준편차 | 변별도1 | 문항수 | 비율(%) |
|---|---|---|---|---|---|
| 20 | .29 | .14 | 0.3미만 | 12 | 60.0 |
| | | | 0.3이상 | 8 | 40.0 |
| | | | 전체 | 20 | 100.0 |

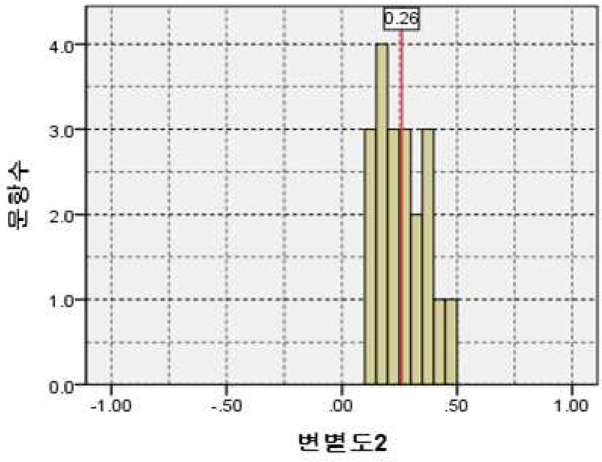


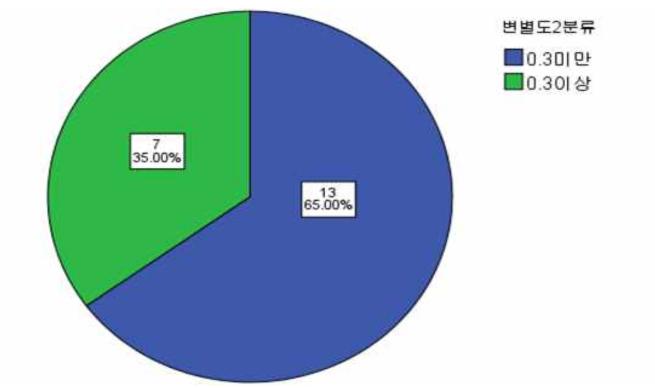


| 총점 | 변별도2 | 표준편차 |
|---|---|---|
| 20 | .26 | .10 |

| 변별도2 | 문항수 | 비율(%) |
|---|---|---|
| 0.3미만 | 13 | 65.0 |
| 0.3이상 | 7 | 35.0 |
| 전체 | 20 | 100.0 |

**해석**

- 보건의료법규 과목에서 난이도 지수가 80 에서 100 사이인 문항이 전체 20 문항 중 2 문항으로 나타났으며, 다음으로 60 이상 80 미만인 문항이 8 문항, 60 미만인 문항은 10 문항으로 나타남
- 변별도 1 지수를 기준으로 분류하였을 때, 0.3 미만인 문항이 12 문항으로 0.3 이상인 문항이 8 문항인 것에 비해 더 많이 나타남
- 변별도 2 지수를 기준으로 분류하였을 때, 0.3 미만인 문항이 13 문항으로 0.3 이상인 문항이 7 문항인 것에 비해 더 많이 나타남

### 3) 지식수준별 난이도와 변별도

#### 가) 전회 대비 지식수준별 난이도와 변별도

(1) 전회 대비 암기형 난이도와 변별도

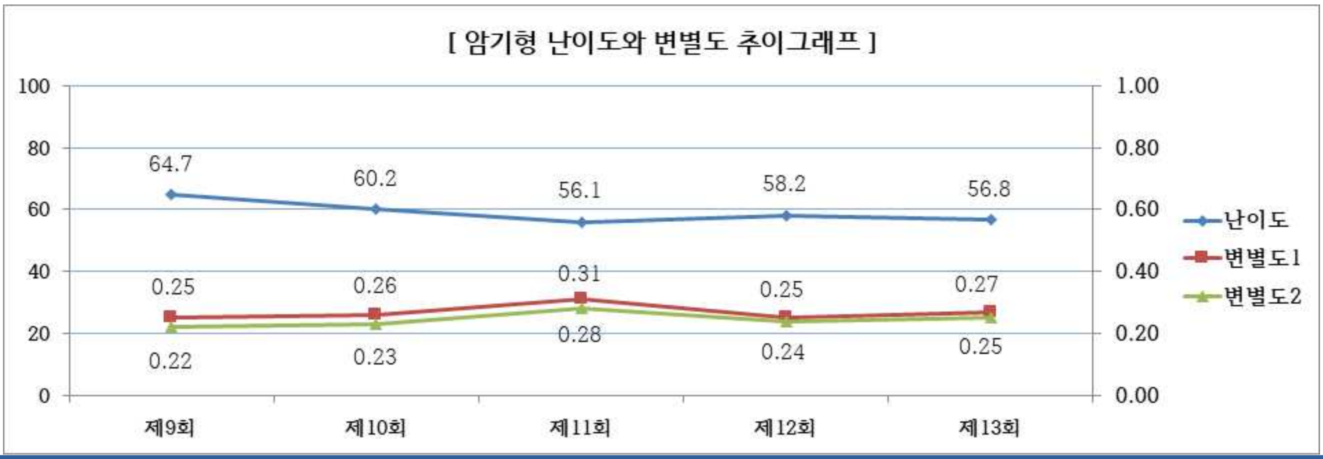


| 회차 | 난이도 | | 변별도1 | | 변별도2 | |
|---|---|---|---|---|---|---|
| | 평균 | 표준편차 | 평균 | 표준편차 | 평균 | 표준편차 |
| 제9회 | 64.7 | 20.1 | 0.25 | 0.12 | 0.22 | 0.11 |
| 제10회 | 60.2 | 23.2 | 0.26 | 0.13 | 0.23 | 0.10 |
| 제11회 | 56.1 | 23.5 | 0.31 | 0.13 | 0.28 | 0.10 |
| 제12회 | 58.2 | 23.4 | 0.25 | 0.14 | 0.24 | 0.12 |
| **제13회** | **56.8** | **22.8** | **0.27** | **0.15** | **0.25** | **0.11** |

(2) 전회 대비 해석형 난이도와 변별도

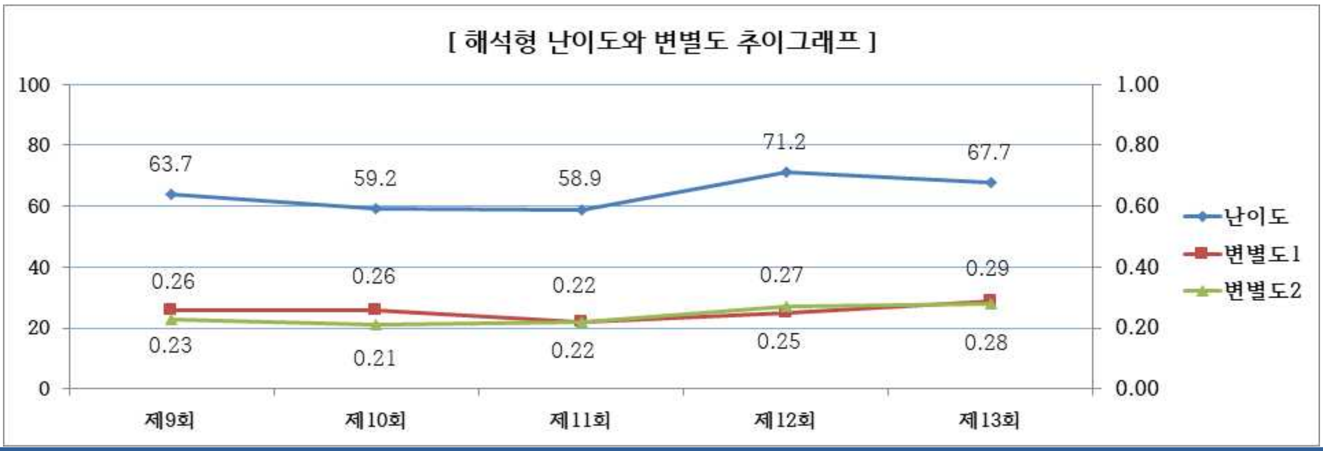


| 회차 | 난이도 | | 변별도1 | | 변별도2 | |
|---|---|---|---|---|---|---|
| | 평균 | 표준편차 | 평균 | 표준편차 | 평균 | 표준편차 |
| 제9회 | 63.7 | 24.0 | 0.26 | 0.18 | 0.23 | 0.14 |
| 제10회 | 59.2 | 17.1 | 0.26 | 0.13 | 0.21 | 0.13 |
| 제11회 | 58.9 | 26.3 | 0.22 | 0.17 | 0.22 | 0.14 |
| 제12회 | 71.2 | 22.4 | 0.25 | 0.12 | 0.27 | 0.09 |
| **제13회** | **67.7** | **18.4** | **0.29** | **0.17** | **0.28** | **0.13** |

(3) 전회 대비 해결형 난이도와 변별도

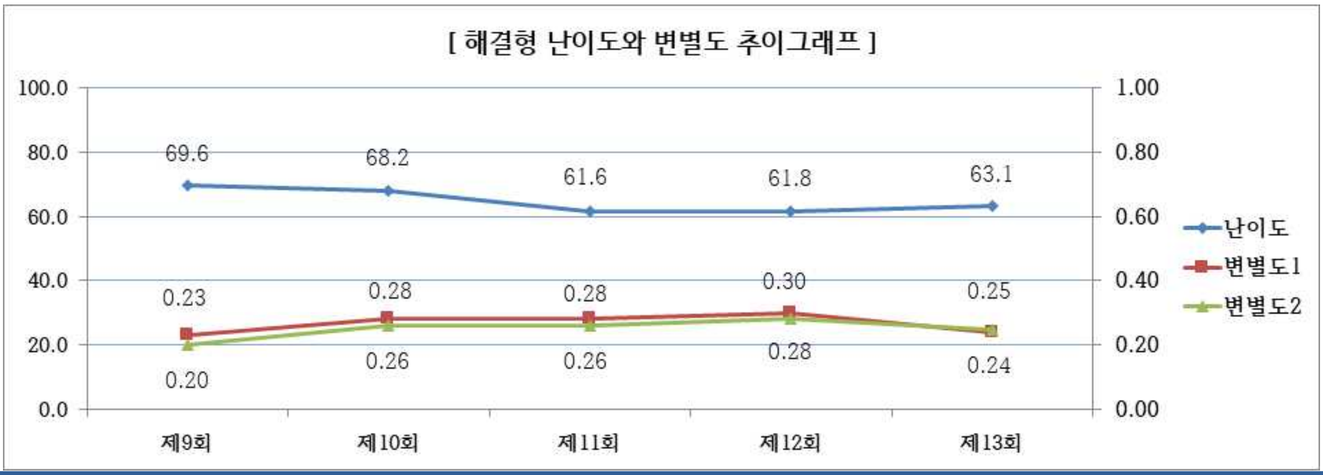


| 회차 | 난이도 | | 변별도1 | | 변별도2 | |
|---|---|---|---|---|---|---|
| | 평균 | 표준편차 | 평균 | 표준편차 | 평균 | 표준편차 |
| 제9회 | 69.6 | 18.0 | 0.23 | 0.17 | 0.20 | 0.15 |
| 제10회 | 68.2 | 19.8 | 0.28 | 0.12 | 0.26 | 0.10 |
| 제11회 | 61.6 | 22.4 | 0.28 | 0.14 | 0.26 | 0.10 |
| 제12회 | 61.8 | 21.9 | 0.30 | 0.15 | 0.28 | 0.11 |
| **제13회** | **63.1** | **25.3** | **0.24** | **0.16** | **0.25** | **0.11** |

**해석**

- 전회 대비 암기형, 해석형 문항의 난이도 지수는 각각 1.4, 3.5 감소하였으며, 해결형 문항의 난이도 지수는 1.3 증가함
- 변별도 1 지수의 경우 암기형, 해석형 문항에서는 각각 0.02, 0.04 증가하였으나, 해결형 문항에서는 0.06 감소함
- 변별도 2 지수는 암기형, 해석형 문항에서는 각각 0.01 증가하였으나, 해결형 문항에서 0.03 감소함

## 나) 지식수준별 난이도와 변별도 분포도 및 비율분석

### (1) 암기형 난이도와 변별도 분포도 및 비율분석

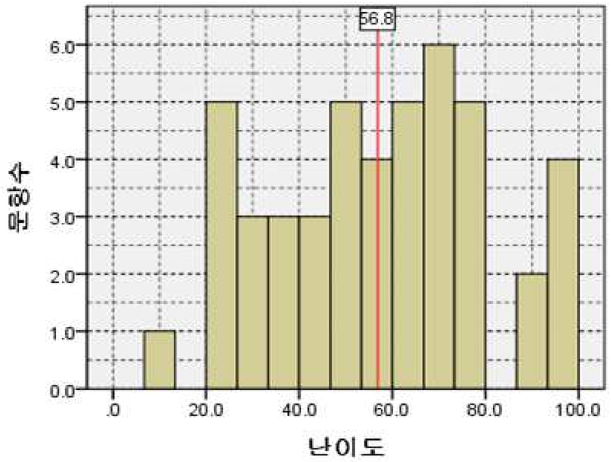


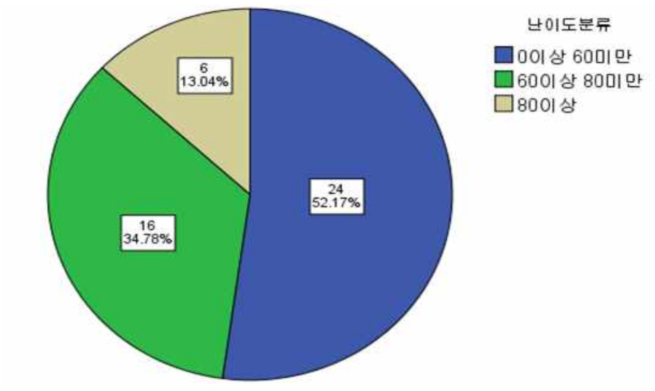


| 총점 | 난이도 | 표준편차 |
|---|---|---|
| 46 | 56.8 | 22.8 |

| 난이도 | 문항수 | 비율(%) |
|---|---|---|
| 0~60미만 | 24 | 52.2 |
| 60~80미만 | 16 | 34.8 |
| 80~100 | 6 | 13.0 |
| 전체 | 46 | 100.0 |

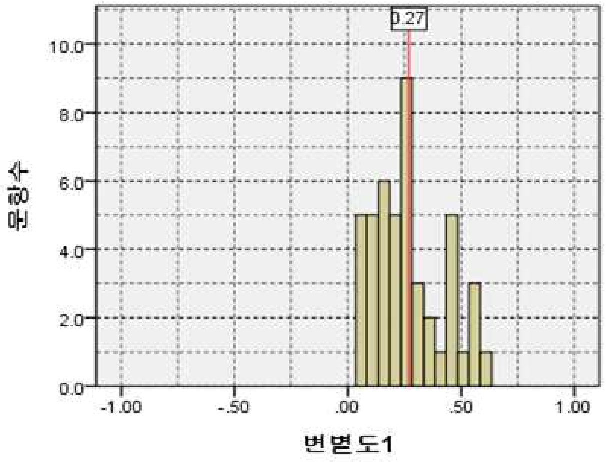


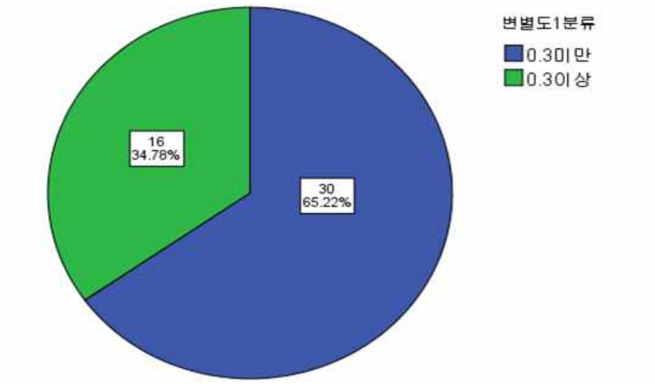


| 총점 | 변별도1 | 표준편차 |
|---|---|---|
| 46 | .27 | .15 |

| 변별도1 | 문항수 | 비율(%) |
|---|---|---|
| 0.3미만 | 30 | 65.2 |
| 0.3이상 | 16 | 34.8 |
| 전체 | 46 | 100.0 |

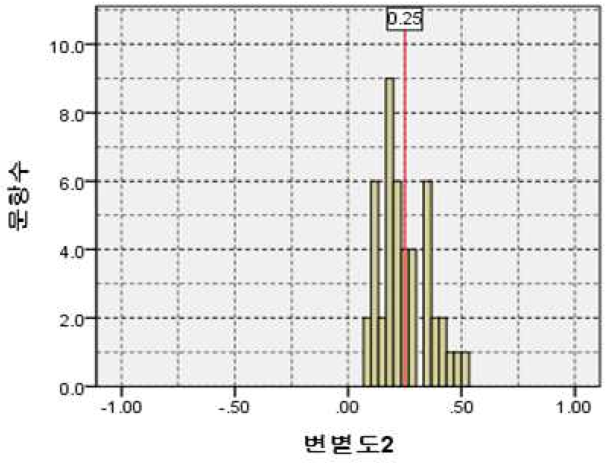


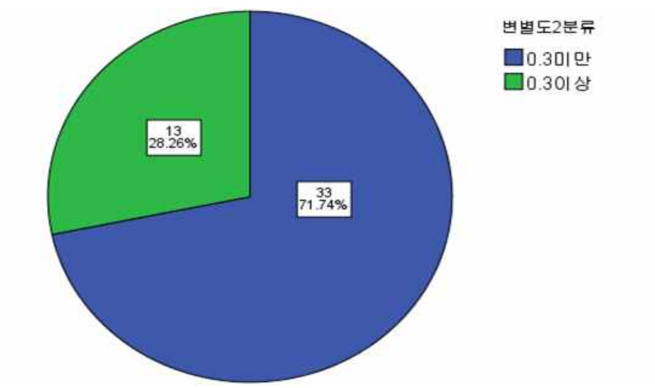


| 총점 | 변별도2 | 표준편차 |
|---|---|---|
| 46 | .25 | .11 |

| 변별도2 | 문항수 | 비율(%) |
|---|---|---|
| 0.3미만 | 33 | 71.7 |
| 0.3이상 | 13 | 28.3 |
| 전체 | 46 | 100.0 |

**해석**

- 암기형에서 난이도 지수가 80 에서 100 사이인 문항이 전체 46 문항 중 6 문항으로 가장 적었으며, 다음으로 60 이상 80 미만인 문항이 16 문항, 60 미만인 문항은 24 문항으로 나타남
- 변별도 1 지수를 기준으로 분류하였을 때, 0.3 미만인 문항이 30 문항으로 0.3 이상인 문항이 16 문항인 것에 비해 더 많이 나타남
- 변별도 2 지수를 기준으로 분류하였을 때, 0.3 미만인 문항이 33 문항으로 0.3 이상인 문항이 13 문항인 것에 비해 더 많이 나타남

(2) 해석형 난이도와 변별도 분포도 및 비율분석

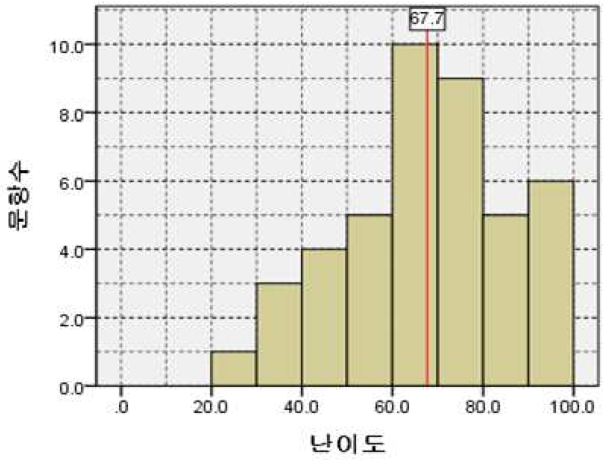


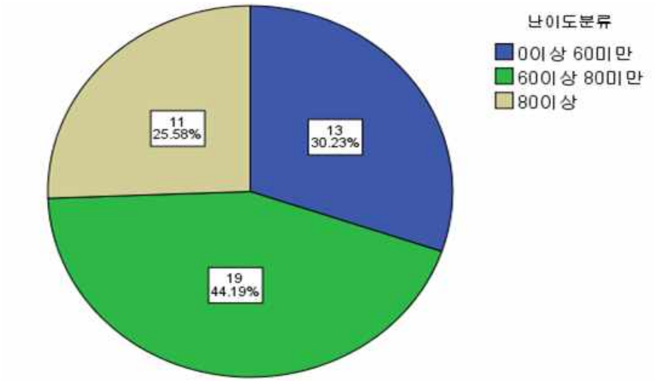


| 총점 | 난이도 | 표준편차 |
|---|---|---|
| 43 | 67.7 | 18.4 |

| 난이도 | 문항수 | 비율(%) |
|---|---|---|
| 0~60미만 | 13 | 30.2 |
| 60~80미만 | 19 | 44.2 |
| 80~100 | 11 | 25.6 |
| 전체 | 43 | 100.0 |

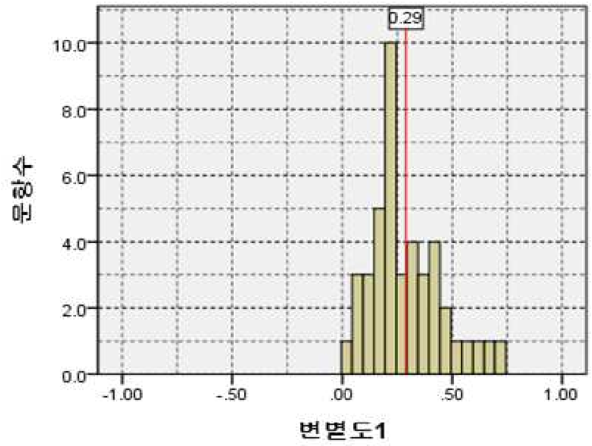


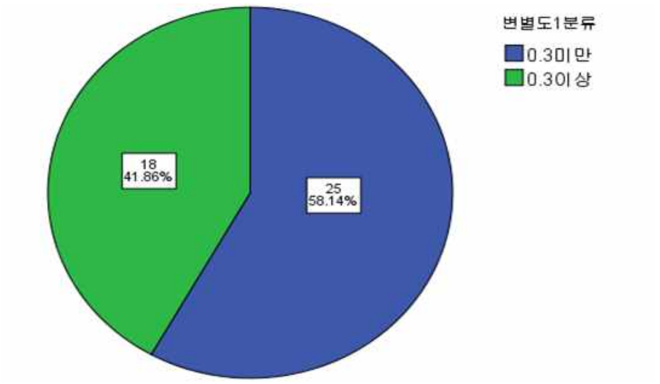


| 총점 | 변별도1 | 표준편차 |
|---|---|---|
| 43 | .29 | .17 |

| 변별도1 | 문항수 | 비율(%) |
|---|---|---|
| 0.3미만 | 25 | 58.1 |
| 0.3이상 | 18 | 41.9 |
| 전체 | 43 | 100.0 |

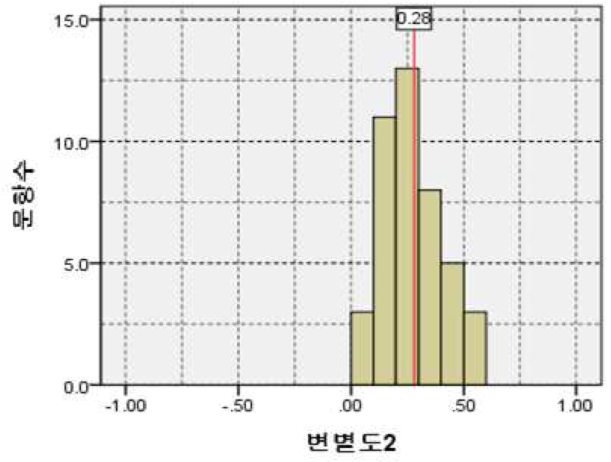


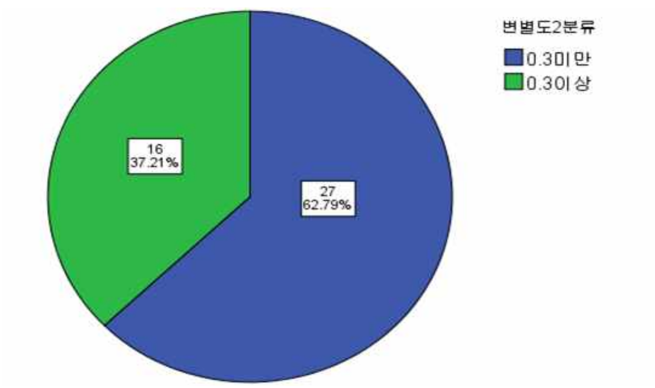


| 총점 | 변별도2 | 표준편차 |
|---|---|---|
| 43 | .28 | .13 |

| 변별도2 | 문항수 | 비율(%) |
|---|---|---|
| 0.3미만 | 27 | 62.8 |
| 0.3이상 | 16 | 37.2 |
| 전체 | 43 | 100.0 |

**해석**

- 해석형에서 난이도 지수가 80 에서 100 사이인 문항이 전체 40 문항 중 11 문항으로 나타났으며, 다음으로 60 이상 80 미만인 문항이 19 문항, 60 미만인 문항은 13 문항으로 나타남
- 변별도 1 지수를 기준으로 분류하였을 때, 0.3 미만인 문항이 25 문항, 0.3 이상인 문항이 18 문항인 것에 비해 더 많이 나타남
- 변별도 2 지수를 기준으로 분류하였을 때, 0.3 미만인 문항이 27 문항으로 0.3 이상인 문항이 16 문항인 것에 비해 더 많이 나타남

(3) 해결형 난이도와 변별도 분포도 및 비율분석

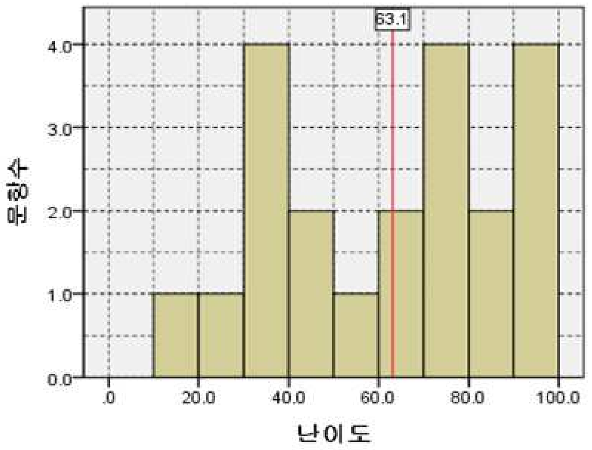


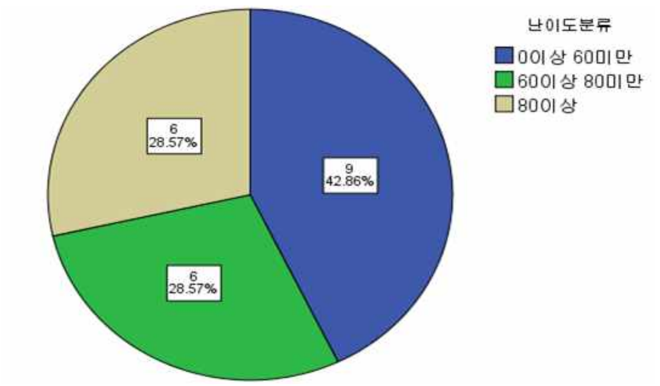


| 총점 | 난이도 | 표준편차 | 난이도 | 문항수 | 비율(%) |
|---|---|---|---|---|---|
| 21 | 63.1 | 25.3 | 0~60미만 | 9 | 42.9 |
| | | | 60~80미만 | 6 | 28.6 |
| | | | 80~100 | 6 | 28.6 |
| | | | 전체 | 21 | 100.0 |

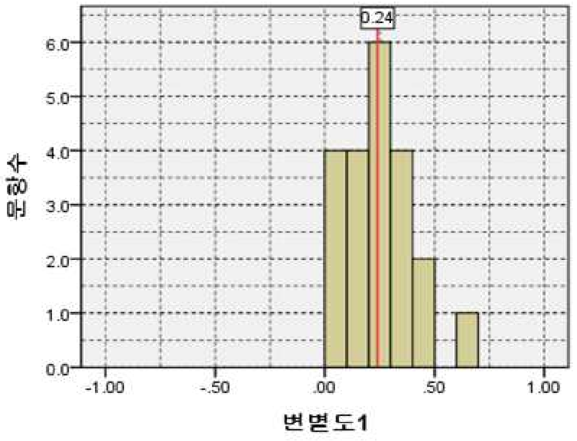


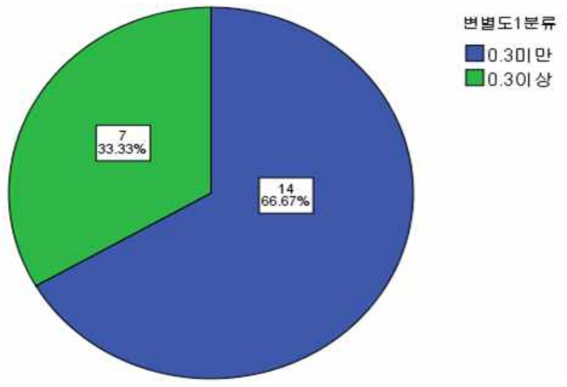


| 총점 | 변별도1 | 표준편차 | 변별도1 | 문항수 | 비율(%) |
|---|---|---|---|---|---|
| 21 | .24 | .16 | 0.3미만 | 14 | 66.7 |
| | | | 0.3이상 | 7 | 33.3 |
| | | | 전체 | 21 | 100.0 |

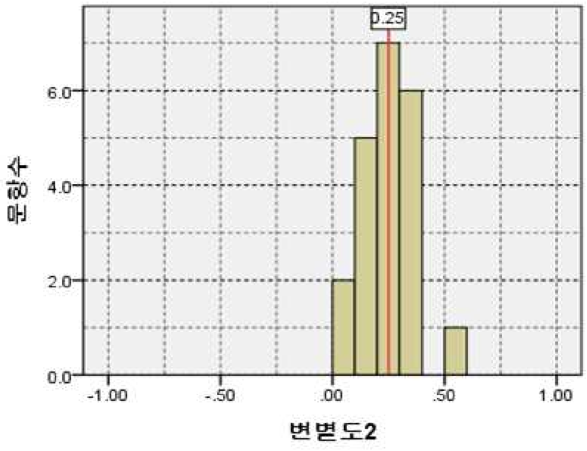


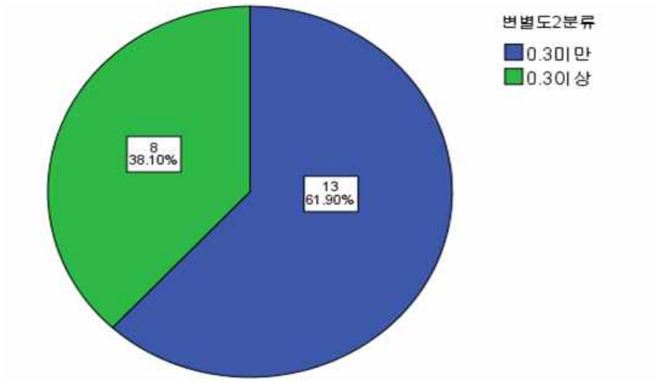


| 총점 | 변별도2 | 표준편차 |
|---|---|---|
| 21 | .25 | .11 |

| 변별도2 | 문항수 | 비율(%) |
|---|---|---|
| 0.3미만 | 13 | 61.9 |
| 0.3이상 | 8 | 38.1 |
| 전체 | 21 | 100.0 |

**해석**

- 해결형에서 난이도 지수가 80 에서 100 사이인 문항이 전체 21 문항 중 6 문항으로 나타났으며, 다음으로 60 이상 80 미만인 문항이 6 문항, 60 미만인 문항은 9 문항으로 나타남
- 변별도 1 지수를 기준으로 분류하였을 때, 0.3 미만인 문항이 14 문항으로 0.3 이상인 문항이 7 문항인 것에 비해 더 많이 나타남
- 변별도 2 지수를 기준으로 분류하였을 때, 0.3 미만인 문항이 13 문항으로 0.3 이상인 문항이 8 문항인 것에 비해 더 많이 나타남

## 3. 난이도와 변별도 간 산포도

### 1) 전체 난이도와 변별도 간 산포도

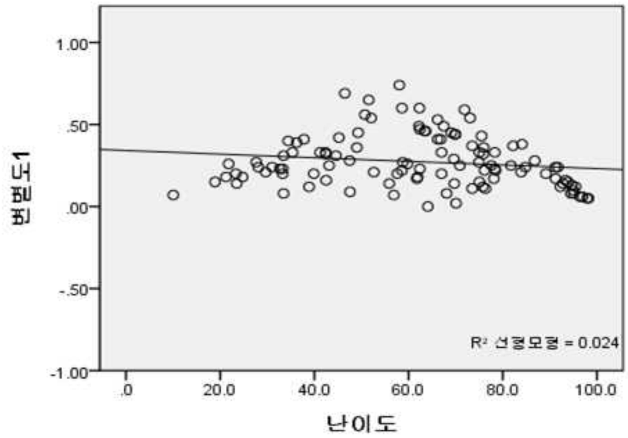


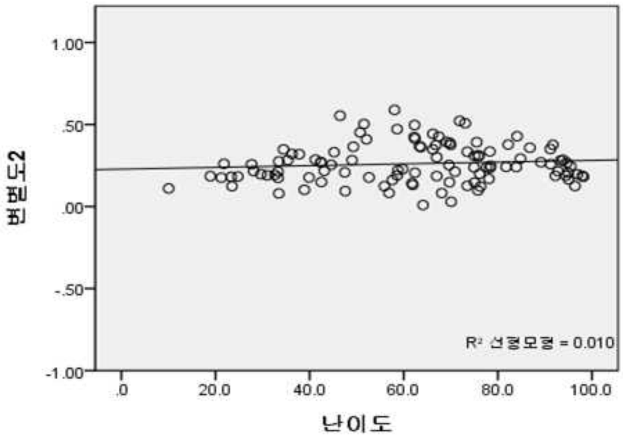


**해석**

- 난이도 지수와 변별도1 지수 간 상관은 -.154로 난이도 지수와 변별력의 관련성이 낮은 것으로 나타남
- 난이도 지수와 변별도2 지수 간 상관은 .100으로 난이도 지수와 변별력의 관련성이 낮은 것으로 나타남

### 2) 과목별 난이도와 변별도 간 산포도

#### 가) 보건프로그램 개발 및 평가 난이도와 변별도 간 산포도

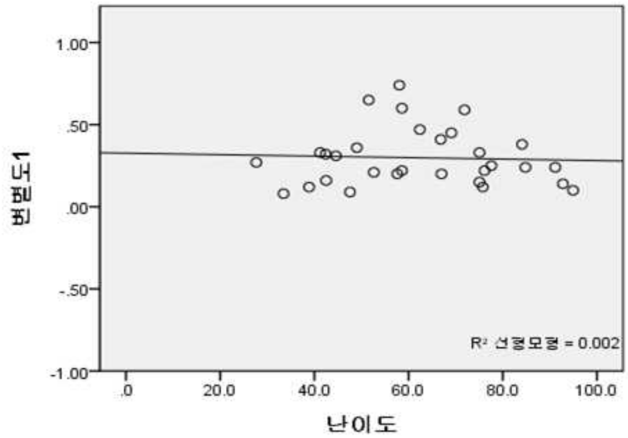


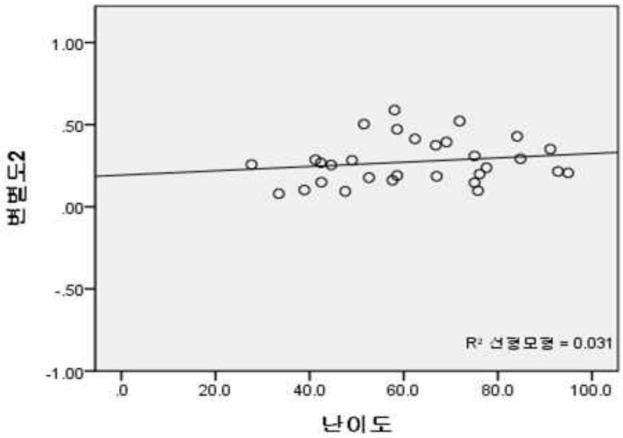

**해석**

- 난이도 지수와 변별도1 지수 간 상관은 -.047로 난이도 지수와 변별력의 관련성이 없는 것으로 나타남
- 난이도 지수와 변별도2 지수 간 상관은 .175로 난이도 지수와 변별력의 관련성이 낮은 것으로 나타남

나) 보건학 난이도와 변별도 간 산포도

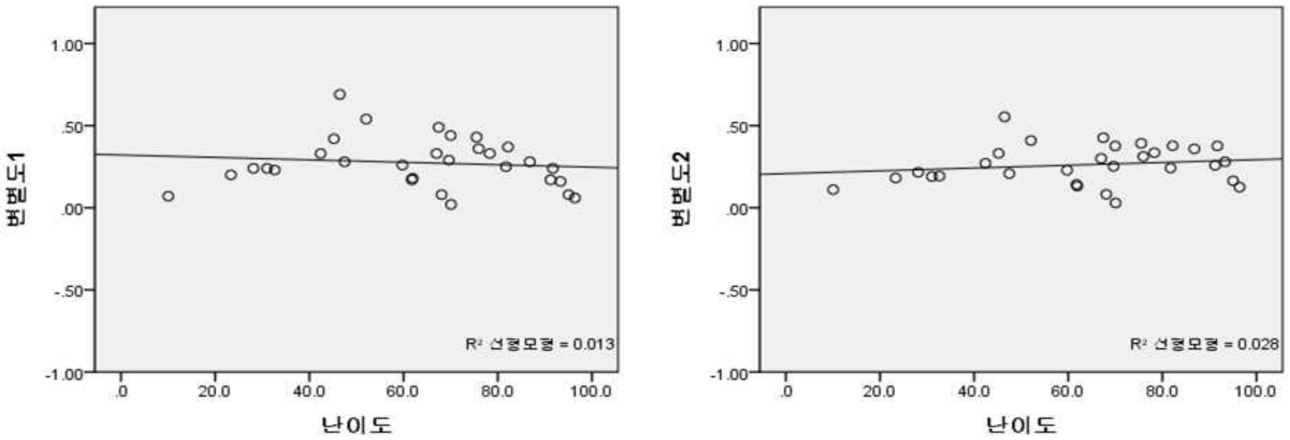


**해석**

- 난이도 지수와 변별도1 지수 간 상관은 -.113으로 난이도 지수와 변별력의 관련성이 낮은 것으로 나타남
- 난이도 지수와 변별도2 지수 간 상관은 .166으로 난이도 지수와 변별력의 고나련성이 낮은 것으로 나타남

다) 보건교육학 난이도와 변별도 간 산포도

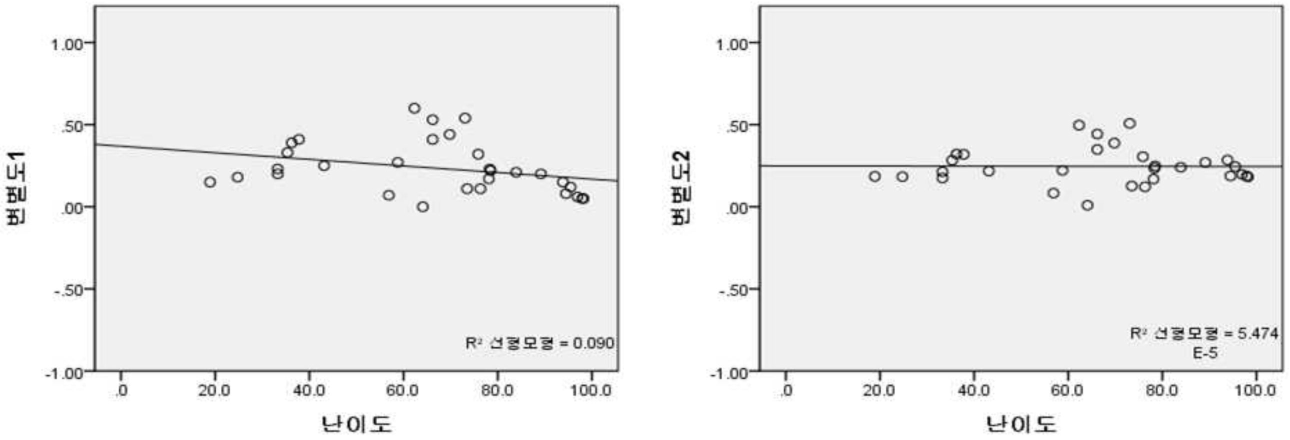

**해석**

- 난이도 지수와 변별도 1 지수 간 상관은 -.300 으로 난이도 지수와 변별력의 관련성이 낮은 것으로 나타남
- 난이도 지수와 변별도 2 지수 간 상관은 -.007 로 난이도 지수와 변별력의 관련성이 없는 것으로 나타남

라) 보건의료법규 난이도와 변별도 간 산포도

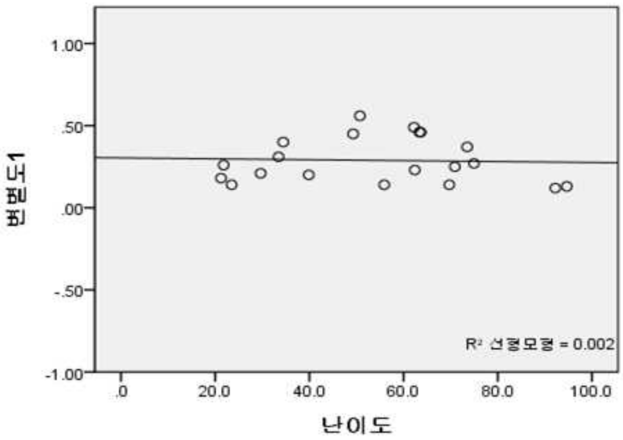


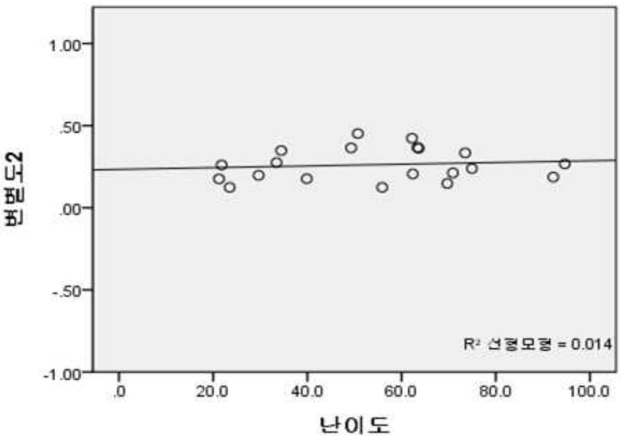


**해석**

- 난이도 지수와 변별도 1 지수 간 상관은 .045 로 난이도 지수와 변별력의 관련성이 없는 것으로 나타남
- 난이도 지수와 변별도 2 지수 간 상관은 .117 로 난이도 지수와 변별력의 관련성이 낮은 것으로 나타남

## 4. 신뢰도 분석

| 과목명 | 문항수 | 제9회 | 제10회 | 제11회 | 제12회 | 제13회 |
|---|---|---|---|---|---|---|
| 전체 | 110 | .862 | .876 | .869 | .875 | .872 |
| 보건프로그램 개발 및 평가 | 30 | .664 | .742 | .609 | .699 | .686 |
| 보건학 | 30 | .715 | .681 | .712 | .716 | .662 |
| 보건교육학 | 30 | .567 | .642 | .561 | .651 | .621 |
| 보건의료법규 | 20 | .542 | .526 | .707 | .514 | .613 |

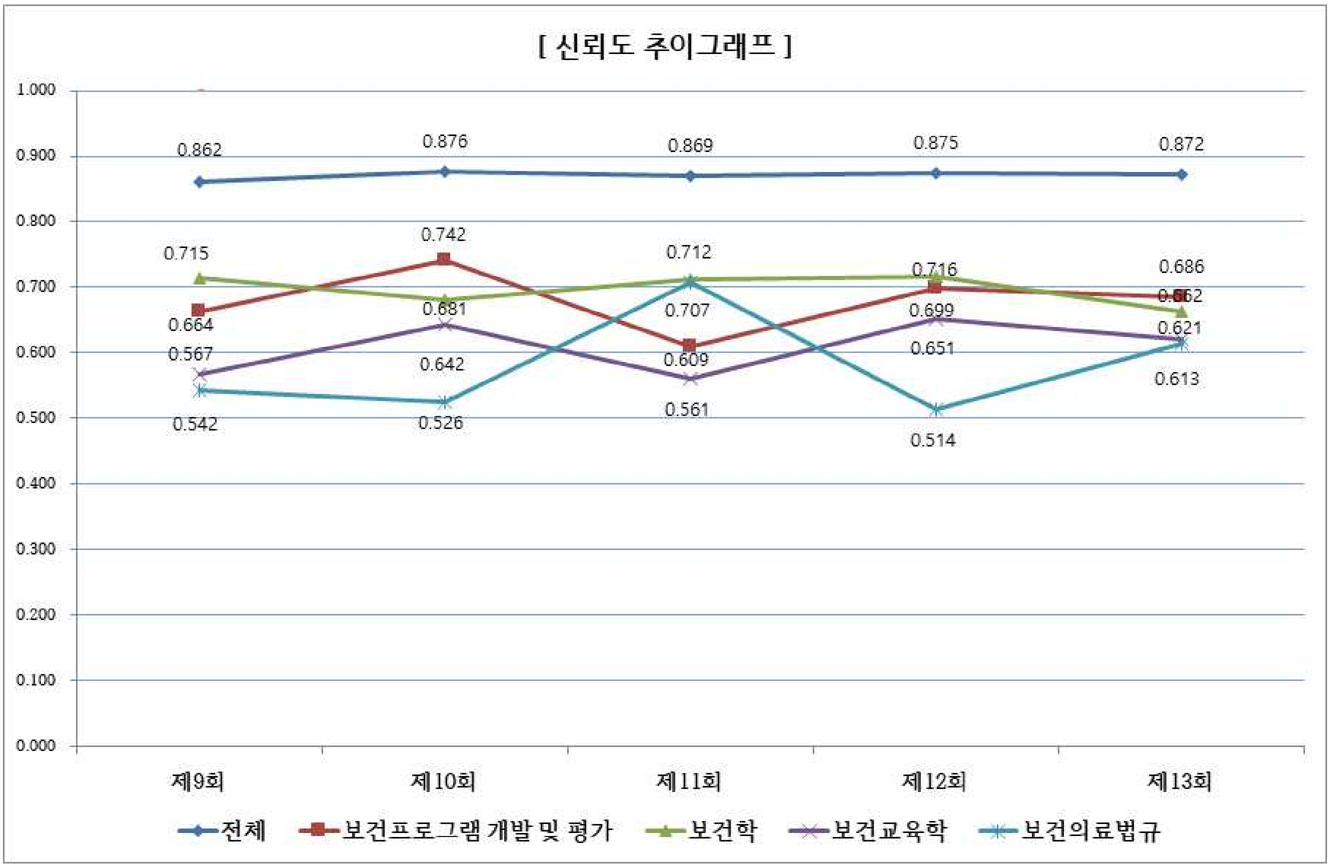


**해석**

- 전회 대비 전체문항의 신뢰도는 .007 감소함
- 전회 대비 보건프로그램 개발 및 평가 문항의 신뢰도는 .133 감소함
- 전회 대비 보건학 과목 문항의 신뢰도는 .031 증가함
- 전회 대비 보건교육학 과목 문항의 신뢰도는 .081 감소함
- 전회 대비 보건의료법규 과목 문항의 신뢰도는 .181 증가함
