# Supplementary material for: Item difficulty index, discrimination index, and reliability of the 26 health professions licensing examinations in 2022, Korea: a psychometric study
Source: J Educ Eval Health Prof. 2023 Nov 22;20:31. doi: 10.3352/jeehp.2023.20.31 (PMC11959405; doi:10.3352/jeehp.2023.20.31)
Supplement: Supplementary file 1 — Supplement 1. Item analysis results of 26 health professions licensing examinations administered during late 2022 and early 2023. [file jeehp-20-31_Suppl1.zip › 2022│Γ╡╡ ┴a23╚╕ └╟┴÷║╕┴╢▒Γ▒Γ╗τ ▒╣░í╜├╟Φ ║╨╝«░ß░·.pdf]

2022년도 제23회
의지·보조기기사 국가시험
문항분석 결과

국민이 신뢰하고 감동하는 시험평가기관
한국보건의료인국가시험원
KOREA HEALTH PERSONNEL LICENSING EXAMINATION INSTITUTE

일반 용어 정의

□ **평균**

- 집단에서의 대표적 경향값으로 전체 값을 더하여 총 응시자로 나눈 값

□ **표준편차**

- 평균과 각 점수의 차이인 편차들의 평균으로 점수가 흩어져 분포되어 있는 정도

□ **검사이론**

- 검사와 검사를 구성하고 있는 문항의 양호도를 분석 및 평가하는 방법을 정의한 이론체계
- 대표적으로 고전검사이론과 문항반응이론이 있음

고전검사이론 용어 정의

□ **고전검사이론(Classical Test Theory; CTT)**

- 검사의 질을 분석하는 검사이론 중 한 가지로 19세기 말부터 전개되어 현재까지 주로 사용되고 있는 검사이론임
- 고전검사이론에 의한 문항과 응시자 능력 추정치는 다음과 같음

○ **문항난이도**

· 검사 문항의 쉽고 어려운 정도를 나타내는 지수
· 난이도 지수는 총 반응 수에 대한 정답 반응 수의 비율로 문항의 정답률임
· 문항난이도는 0~100까지의 값을 가짐
· 난이도 값이 큰 경우, 쉬운 문항으로 '난이도가 낮다'라고 해석하며, 난이도 값이 작은 경우, 어려운 문항으로 '난이도가 높다'라고 해석함

○ **문항변별도**

· 각 문항이 응시자의 능력 수준을 변별할 수 있는 정도를 나타내는 지수
· 문항변별도는 -1~+1까지의 값을 가지며, 1에 가까울수록 변별력 크다고 해석함
· 일반적으로 문항변별도가 0.3 이상이면 우수한 문항으로 평가함
· 구하는 방식에는 '상하위집단 구분법', '문항-총점 상관계수' 등이 있음
  1) **변별도 1(상하위구분법):** 상위 27%와 하위 27% 집단의 난이도 차이를 구하는 방식
  2) **변별도 2(상관계수법):** 문항-총점과의 상관계수로 구하는 방식

○ **신뢰도**

· 시험이 평가하고자 하는 것을 일관성 있게 측정하는가로 시험이 오차없이 정확하게 측정한 정도를 의미함
· 국시원에서는 문항의 내적일관성(Cronbach α)으로 신뢰도를 추정하며 1에 가까울수록 신뢰도가 높다고 해석함

목 차

I. 시행 결과 ········· 5

1. 시험 현황 ········· 6

1) 시험명 ········· 6

2) 시험시행일 ········· 6

3) 응시현황 ········· 6

4) 과목별 문항 수, 배점 및 과락 점수 ········· 6

2. 합격률과 평균성적 ········· 6

1) 필기시험 합격 및 불합격 현황 ········· 6

2) 실기시험 합격 및 불합격 현황 ········· 7

3) 과목별 과락자수 내역 ········· 7

4) 전회 대비 합격률과 평균성적 ········· 7

II. 문항분석 결과 ········· 9

1. 성적 ········· 10

1) 전체 성적분포도 ········· 10

2) 과목별 성적분포도 ········· 11

2. 난이도와 변별도 ········· 13

1) 전체 난이도와 변별도 ········· 13

2) 과목별 난이도와 변별도 ········· 16

3) 지식수준별 난이도와 변별도 ········· 34

4) 자료유형별 난이도와 변별도 ········· 42

3. 난이도와 변별도 간 산포도 ········· 48

1) 전체 난이도와 변별도 간 산포도 ········· 48

2) 과목별 난이도와 변별도 간 산포도 ········· 48

4. 신뢰도 분석 ········· 53

I. 시행 결과

1. 시험 현황

1) 시험명: 2022년도 제23회 의지보조기기사 국가시험

2) 시험시행일: 2022년 11월 26일

3) 응시현황

응시대상자수	결시자수	부정행위자수	응시자 준수사항 위반자 수		응시자수 (%)
			휴대폰 소지	신분증 미지참	
165	5	0	0	0	160 (97.0)

4) 과목별 문항 수, 배점 및 과락 점수

교 시	과 목 명	문제 수	배점	총점	합격자 점수기준	
					과목 과락기준	총점 합격기준
1교시	보건의료 관계 법규	20	1	20	8점 미만	126점 이상
	운동·생체역학	20	1	20	8점 미만	
	재활공학·재료학	20	1	20	8점 미만	
	보조기학	50	1	50	20점 미만	
2교시	해부·생리학	20	1	20	8점 미만	
	재활의학	30	1	30	12점 미만	
	의지학	50	1	50	20점 미만	
실기시험		2	50	100	60점 미만	60점 이상
계		212		310		

2. 합격률과 평균성적

1) 필기시험 합격 및 불합격 현황

합격자수 (%)	불합격자수(%)				채점보류자수
	평락	과락	기권	계	
83 (51.9)	76 (47.5)	0 (0.0)	1 (0.6)	77 (48.1)	0 (00.0)

2) 실기시험 합격 및 불합격 현황

합격자수 (%)	결시자수	불합격자수(%)			
		평락	과락	기권	계
80 (97.6)	0 (0.0)	0 (0.0)	2 (2.44)	0 (0.0)	2 (2.44)

※ 실기시험 응시자의 경우 필기시험 합격자(83명) 중 미응시자(1명)을 제외한 수

3) 과목별 과락자수 내역

과목명 / 과락자수	보건의료 관계법규	운동· 생체역학	재활공학 ·재료학	보조기학	해부· 생리학	재활의학	의지학	실기시험
과목별 과락자 수	-	-	-	-	-	-	-	2
전과목 과락자 수	-							

4) 전회 대비 합격률과 평균성적

회차	년도	합격률(%)	평균성적	표준편차	백분율 환산점수
제19회	2018	51.2	174.3	66.8	62.2
제20회	2019	44.0	157.5	69.3	50.8
제21회	2020	61.0	181.7	69.7	58.6
제22회	2021	46.8	169.9	68.7	54.8
**제23회**	**2022**	**50.0**	**170.0**	**67.2**	**54.8**

※ 310점 만점(실기포함)

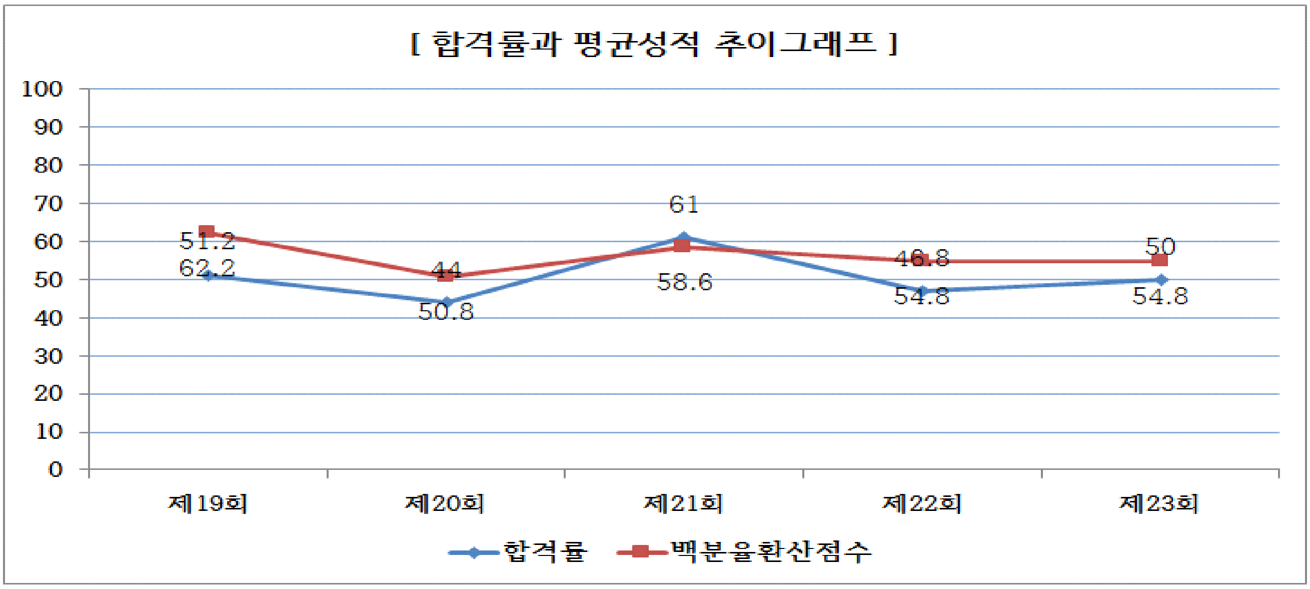

4) 전체 성적분포도

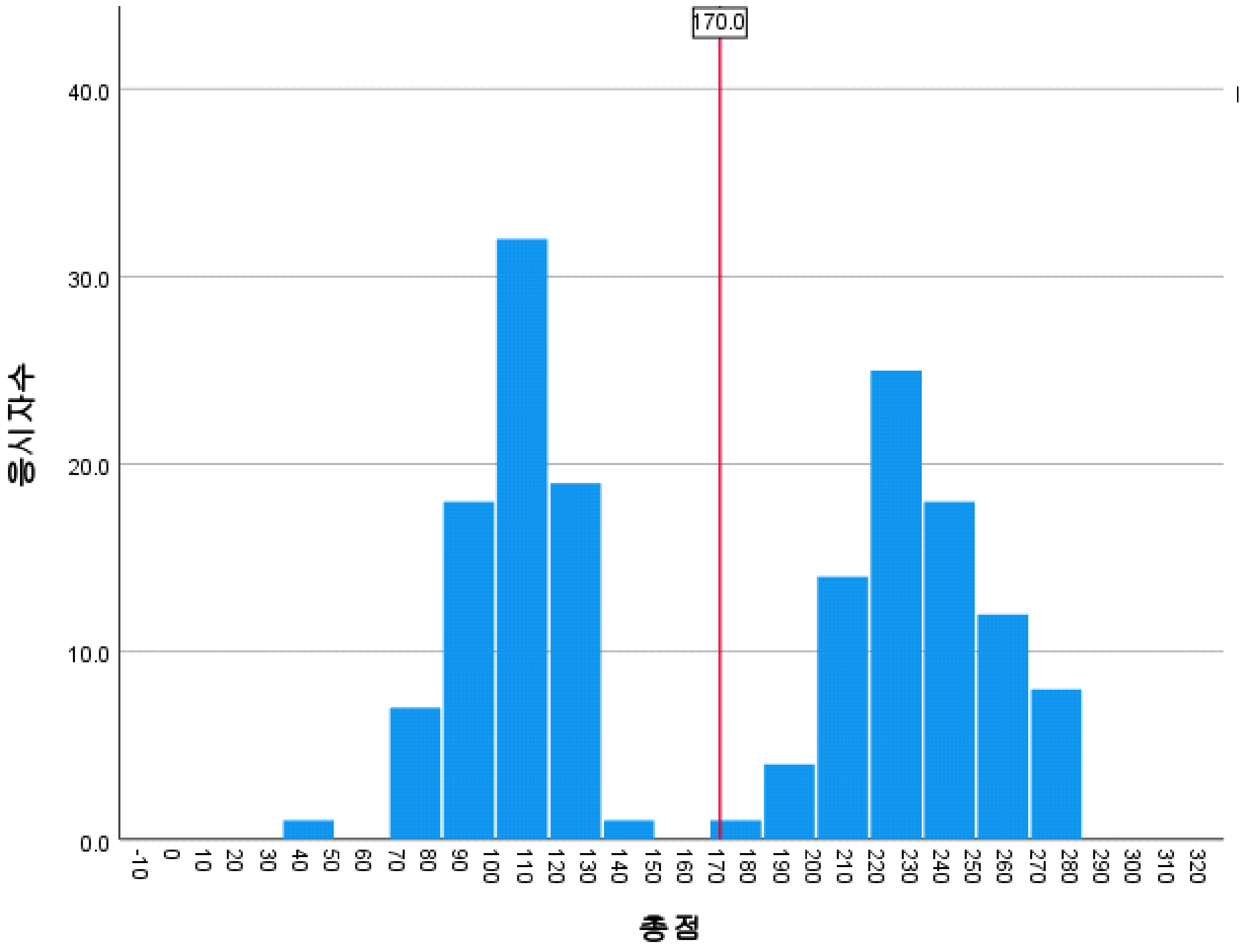

※ 필기시험 불합격자와 필기시험에 합격했으나 실기시험에 응시하지 않은 인원의 실기성적을 포함하지 않음

해석

- 전년 대비 합격률은 3.2% 증가하였으며, 백분율 환산점수는 동일함

Ⅱ. 문항분석 결과

1. 성적

1) 전체 성적분포도

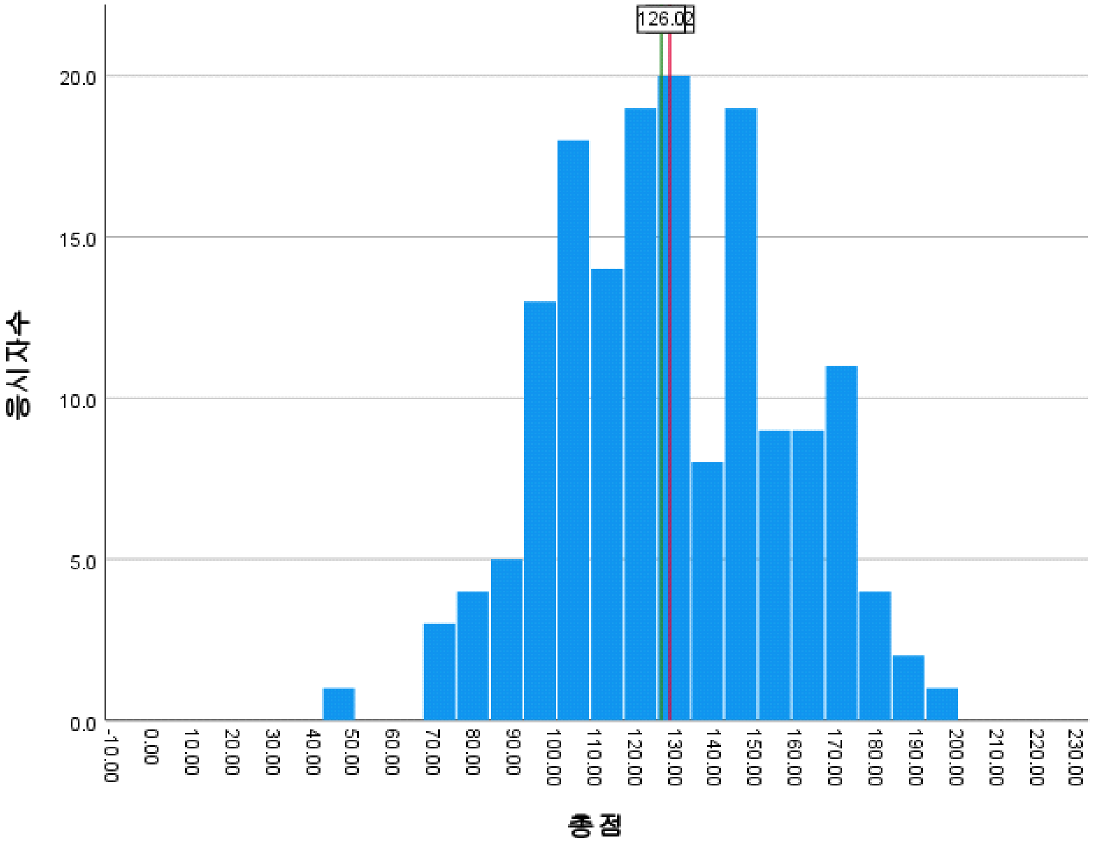

응시자	총점	합격선	평균성적	표준편차
160	210	126	128.2	28.5

※ 실기시험 점수 미포함

2) 과목별 성적분포도

가) 보건의료관계법규

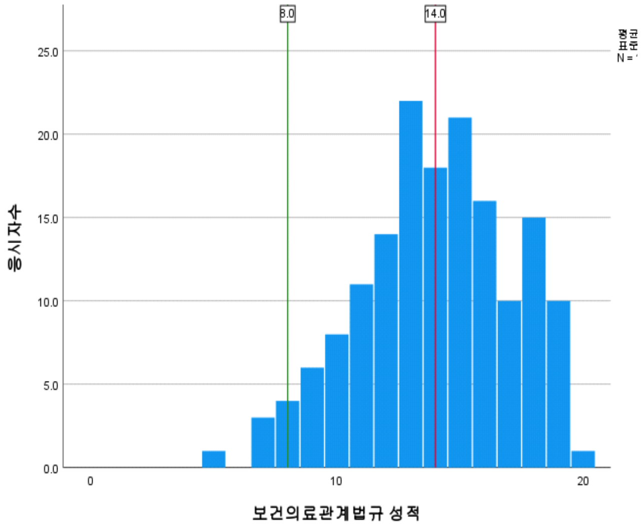

총점	과락선	평균성적	표준편차
20	8	14.0	3.1

나) 운동·생체역학

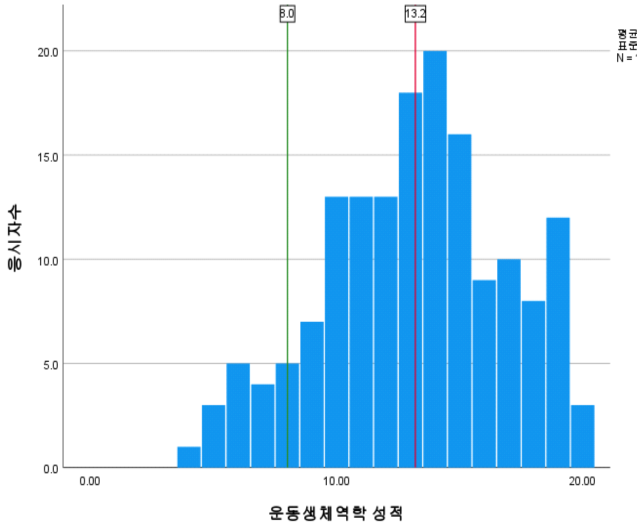

총점	과락선	평균성적	표준편차
20	8	13.2	3.7

다) 재활공학·재료학

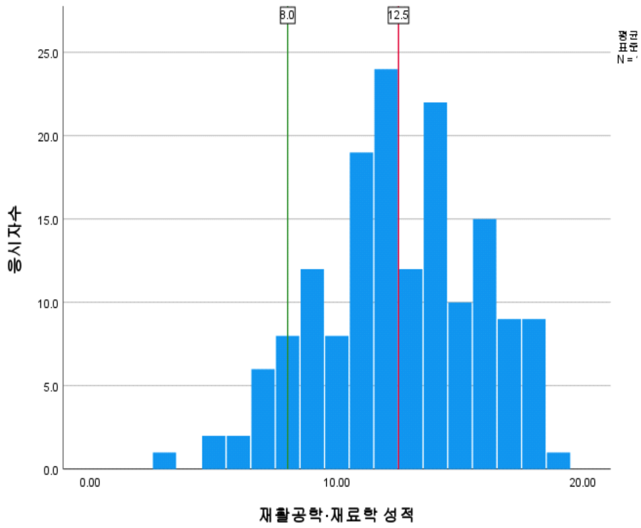

총점	과락선	평균성적	표준편차
20	8	12.5	3.2

라) 보조기학

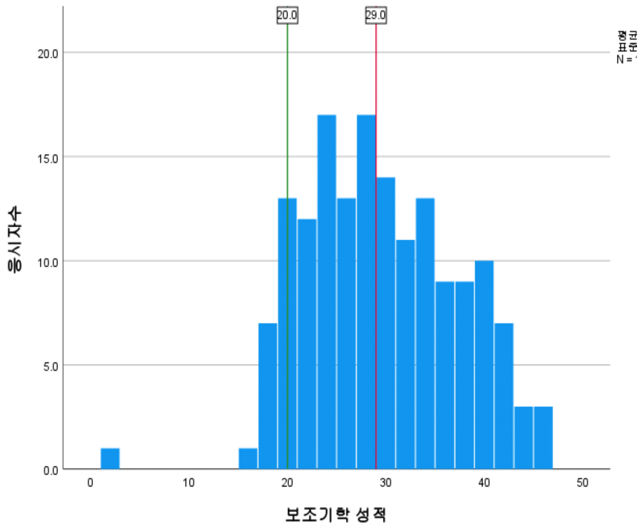

총점	과락선	평균성적	표준편차
50	20	29.0	7.6

마) 해부·생리학

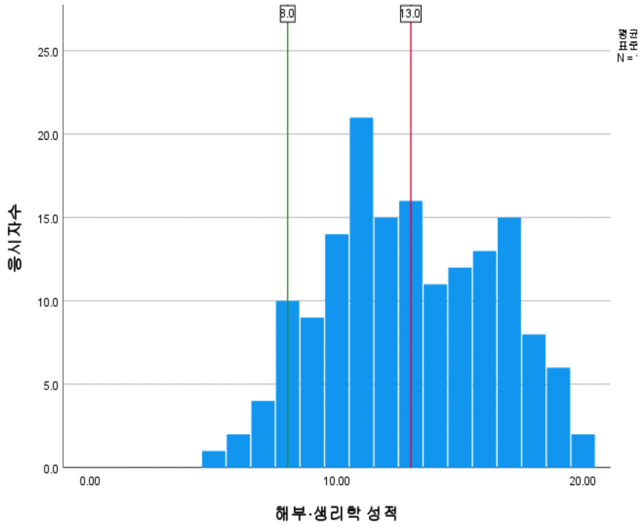

총점	과락선	평균성적	표준편차
20	8	13.0	3.4

바) 재활의학

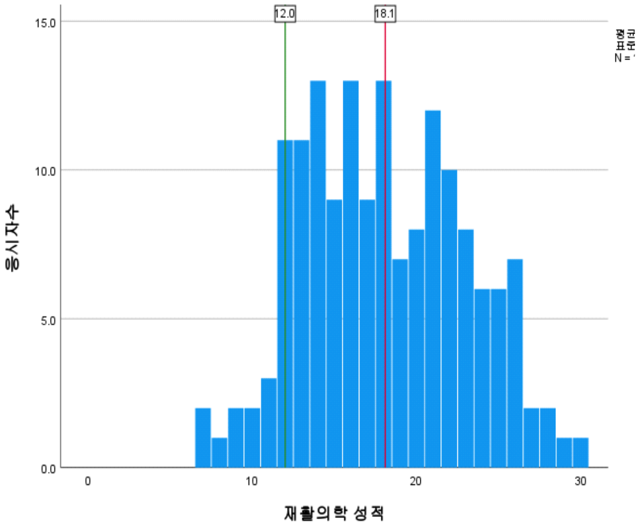

총점	과락선	평균성적	표준편차
30	12	18.1	5.0

사) 의지학

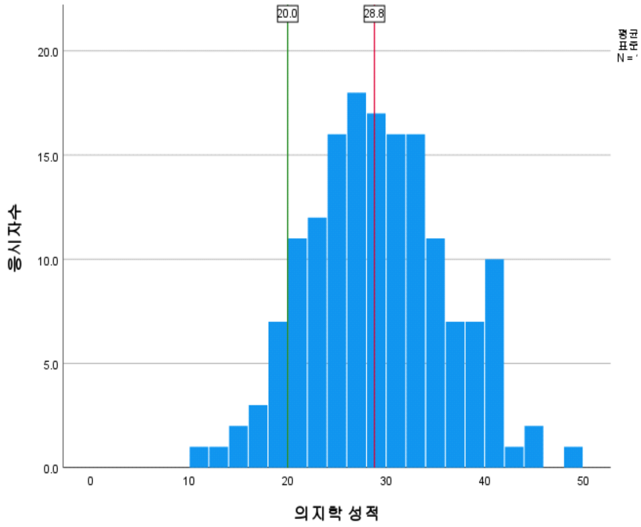

총점	과락선	평균성적	표준편차
50	20	28.8	7.1

2. 난이도와 변별도

1) 전체 난이도와 변별도

가) 전회 대비 전체 난이도와 변별도

회차	난이도		변별도1		변별도2	
	평균	표준편차	평균	표준편차	평균	표준편차
제19회	62.2	19.6	.33	.20	.29	.14
제20회	58.2	19.8	.38	.20	.32	.16
제21회	62.8	19.1	.37	.19	.32	.14
제22회	61.9	18.5	.34	.17	.31	.13
**제23회**	**61.3**	**19.2**	**.33**	**.17**	**.28**	**.13**

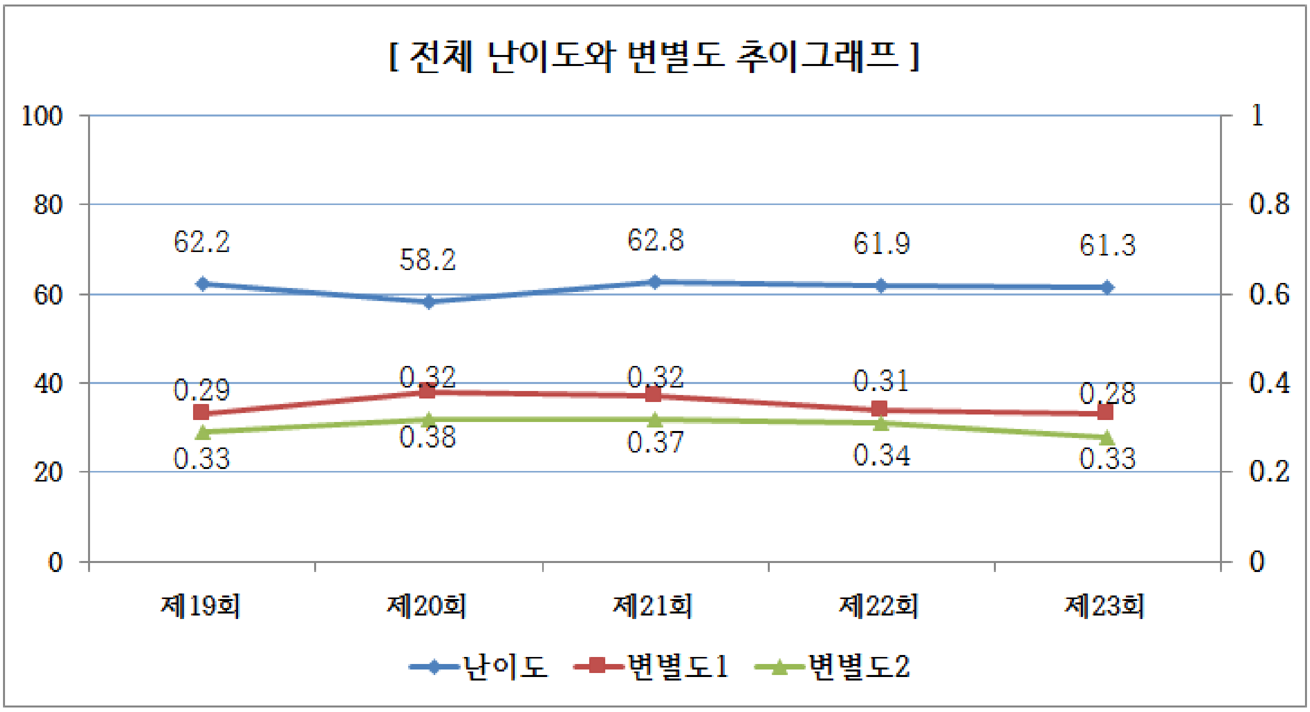

해석

- 전년 대비 난이도 지수는 0.6 감소함
- 변별도 1 지수는 .01, 변별도 2 지수는 .03 감소함

나) 전체 난이도와 변별도 분포도 및 비율분석

(1) 전체 난이도 분포도 및 비율분석

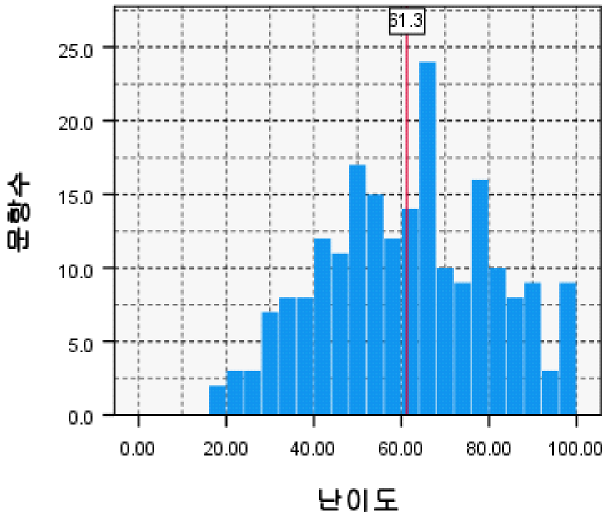

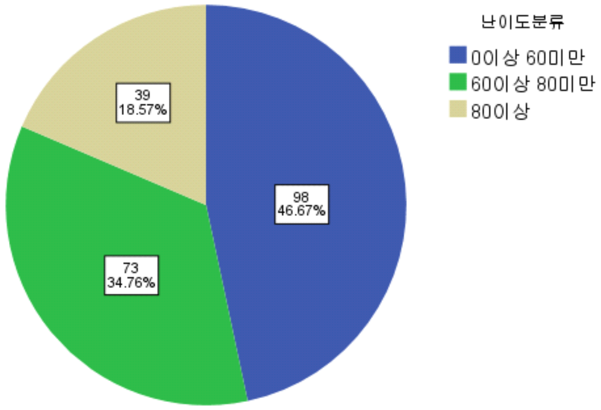

총점	난이도	표준편차
210	61.3	19.3

난이도	문항수	비율(%)
0~60미만	98	46.7
60~80미만	73	34.8
80~100	39	18.6
전체	210	100.0

(2) 전체 변별도1 분포도 및 비율분석

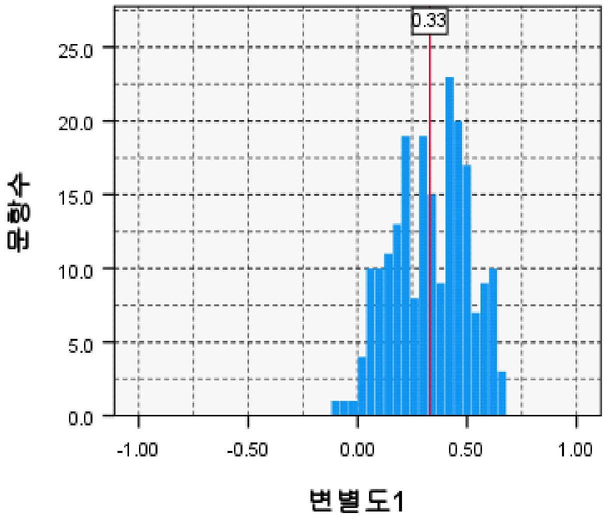

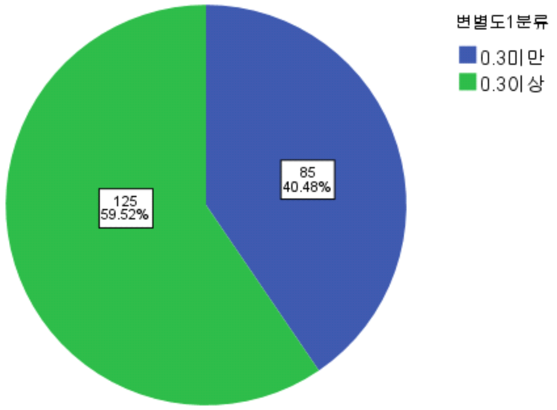

총점	변별도1	표준편차
210	.33	.17

변별도1	문항수	비율(%)
0.3미만	85	40.5
0.3이상	125	59.5
전체	210	100.0

(3) 전체 변별도2 분포도 및 비율분석

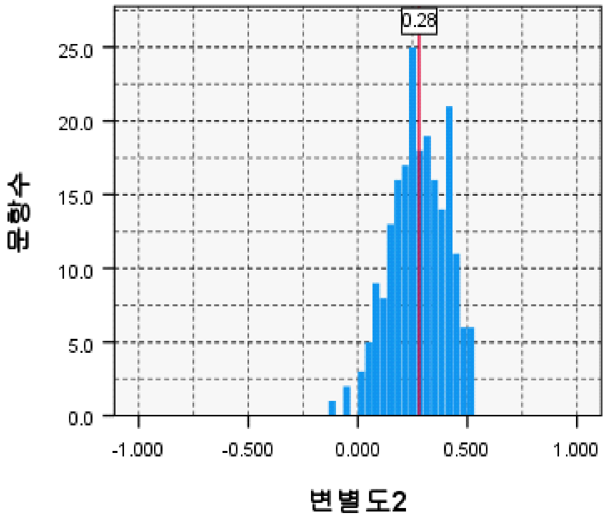

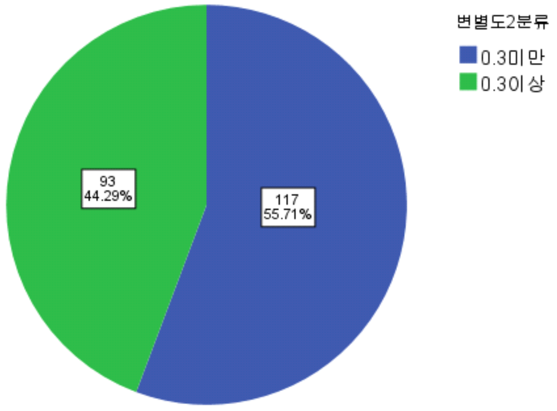

총점	변별도2	표준편차
210	.28	.13

변별도2	문항수	비율(%)
0.3미만	117	55.7
0.3이상	93	44.3
전체	210	100.0

해석

- 난이도 지수가 0에서 60 미만인 문항이 전체 210문항 중 98문항으로 가장 많았으며, 차례로 60이상 80미만인 문항이 73문항, 80에서 100사이인 문항이 39문항인 것으로 나타남
- 변별도1 지수를 기준으로 분류하였을 때, 0.3미만인 문항이 85문항으로 0.3 이상인 문항이 125문항인 것에 비해 더 적게 나타남
- 변별도2 지수를 기준으로 분류하였을 때, 0.3미만인 문항이 117문항으로 0.3이상인 문항이 93문항인 것에 비해 더 적게 나타남

2) 과목별 난이도와 변별도

가) 전회 대비 과목별 난이도와 변별도

(1) 전회 대비 보건의료관계법규 난이도와 변별도

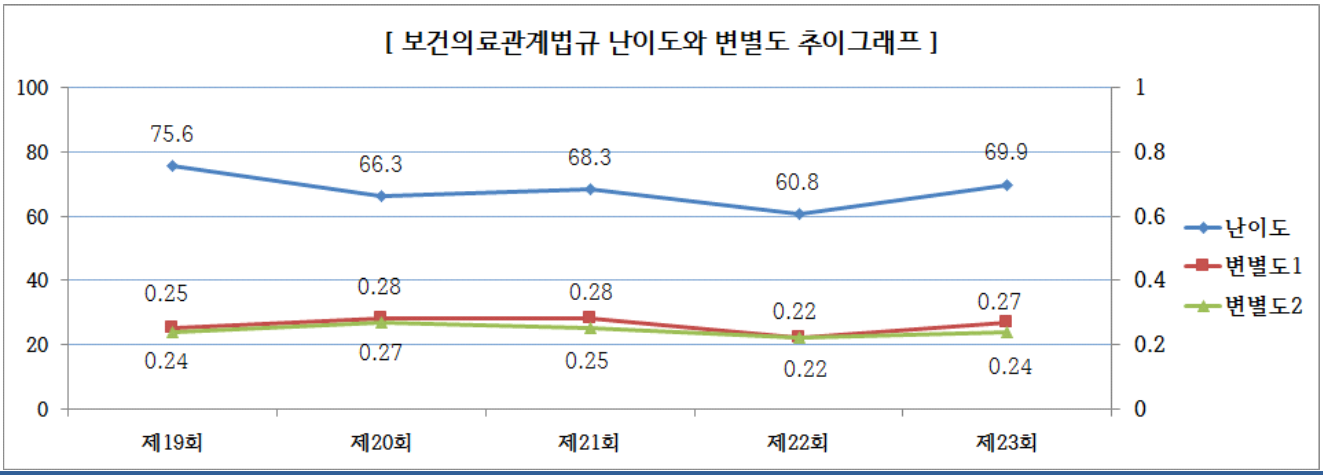

회차	난이도		변별도1		변별도2	
	평균	표준편차	평균	표준편차	평균	표준편차
제19회	75.6	16.5	.25	.19	.24	.12
제20회	66.3	20.8	.28	.14	.27	.12
제21회	68.3	17.9	.28	.17	.25	.13
제22회	60.8	24.2	.22	.14	.22	.14
**제23회**	**69.9**	**15.8**	**.27**	**.14**	**.24**	**.11**

(2) 전회 대비 운동·생체역학 난이도와 변별도

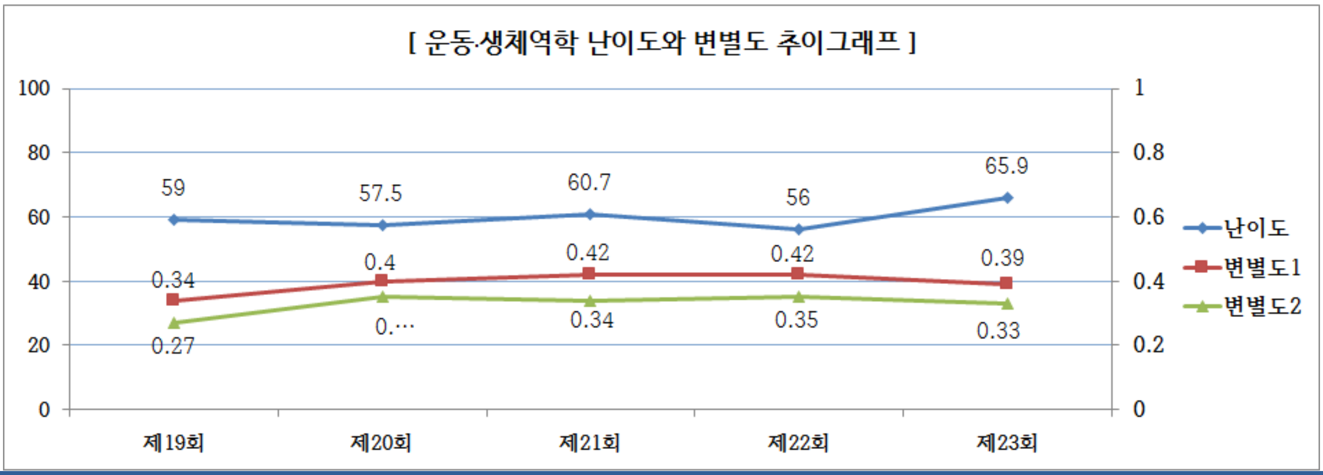

회차	난이도		변별도1		변별도2	
	평균	표준편차	평균	표준편차	평균	표준편차
제19회	59.0	19.4	.34	.19	.27	.15
제20회	57.5	19.5	.40	.21	.35	.17
제21회	60.7	15.5	.42	.18	.34	.14
제22회	56.0	10.8	.42	.17	.35	.14
**제23회**	**65.9**	**18.1**	**.39**	**.16**	**.33**	**.12**

(3) 전회 대비 재활공학·재료학 난이도와 변별도

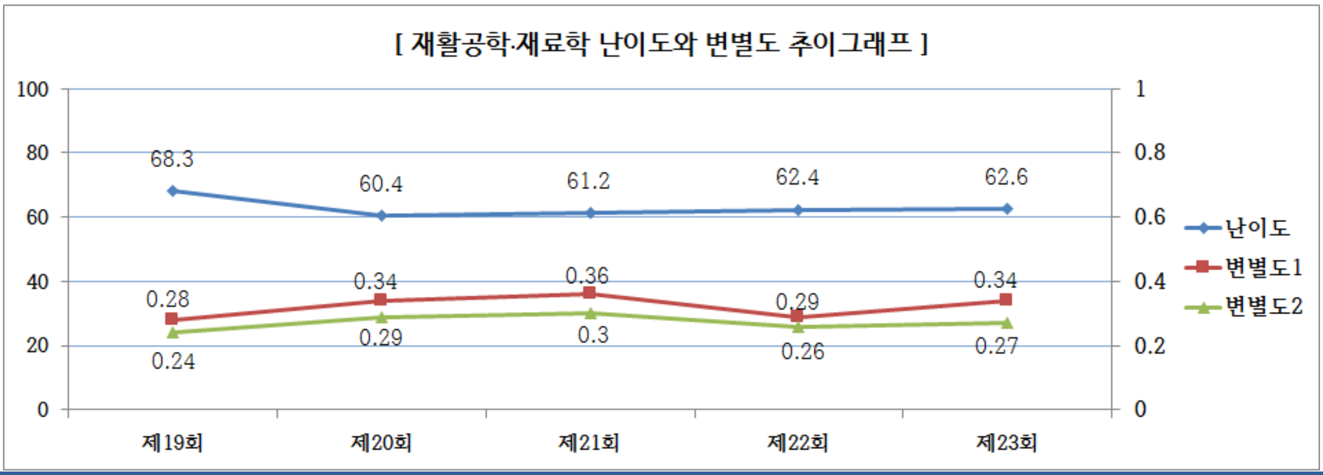

회차	난이도		변별도1		변별도2	
	평균	표준편차	평균	표준편차	평균	표준편차
제19회	68.3	13.9	.28	.20	.24	.14
제20회	60.4	19.0	.34	.17	.29	.13
제21회	61.2	15.6	.36	.19	.30	.13
제22회	62.4	20.5	.29	.16	.26	.14
**제23회**	**62.6**	**19.8**	**.34**	**.16**	**.27**	**.10**

(4) 전회 대비 보조기학 난이도와 변별도

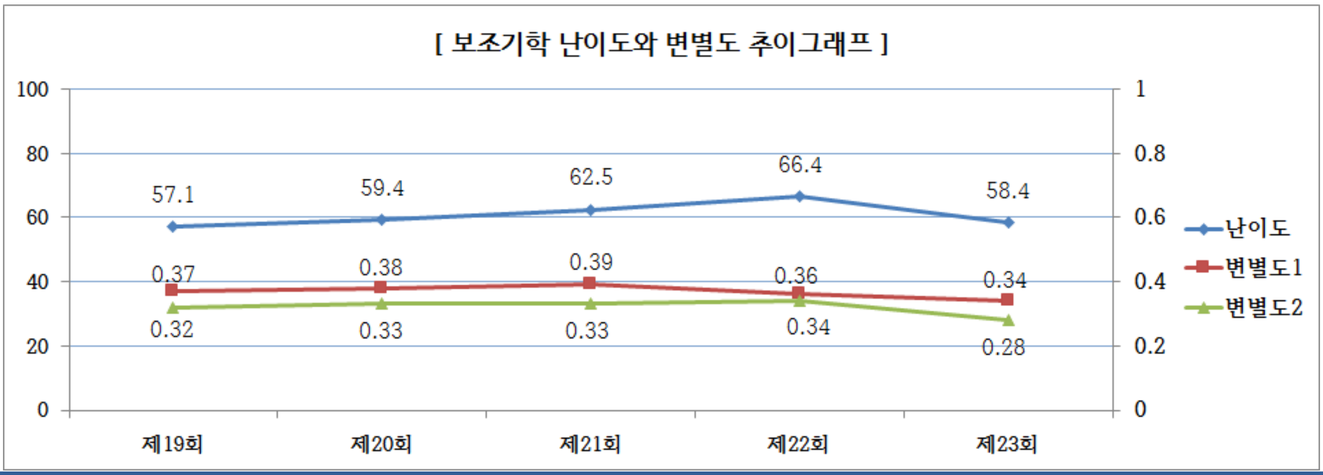

회차	난이도		변별도1		변별도2	
	평균	표준편차	평균	표준편차	평균	표준편차
제19회	57.1	21.0	.37	.22	.32	.16
제20회	59.4	19.7	.38	.22	.33	.18
제21회	62.5	19.0	.39	.18	.33	.13
제22회	66.4	18.5	.36	.17	.34	.12
**제23회**	**58.4**	**17.8**	**.34**	**.18**	**.28**	**.14**

(5) 전회 대비 해부·생리학 난이도와 변별도

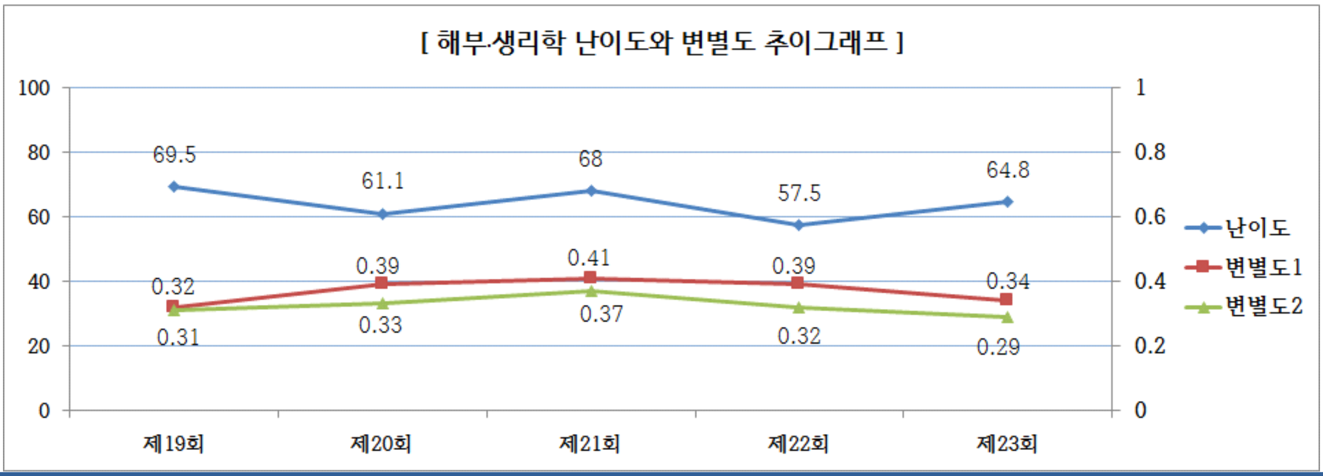

회차	난이도		변별도1		변별도2	
	평균	표준편차	평균	표준편차	평균	표준편차
제19회	69.5	16.4	.32	.18	.31	.12
제20회	61.1	15.9	.39	.15	.33	.13
제21회	68.0	18.8	.41	.22	.37	.16
제22회	57.5	18.2	.39	.14	.32	.12
**제23회**	**64.8**	**20.2**	**.34**	**.20**	**.29**	**.14**

(6) 전회 대비 재활의학 난이도와 변별도

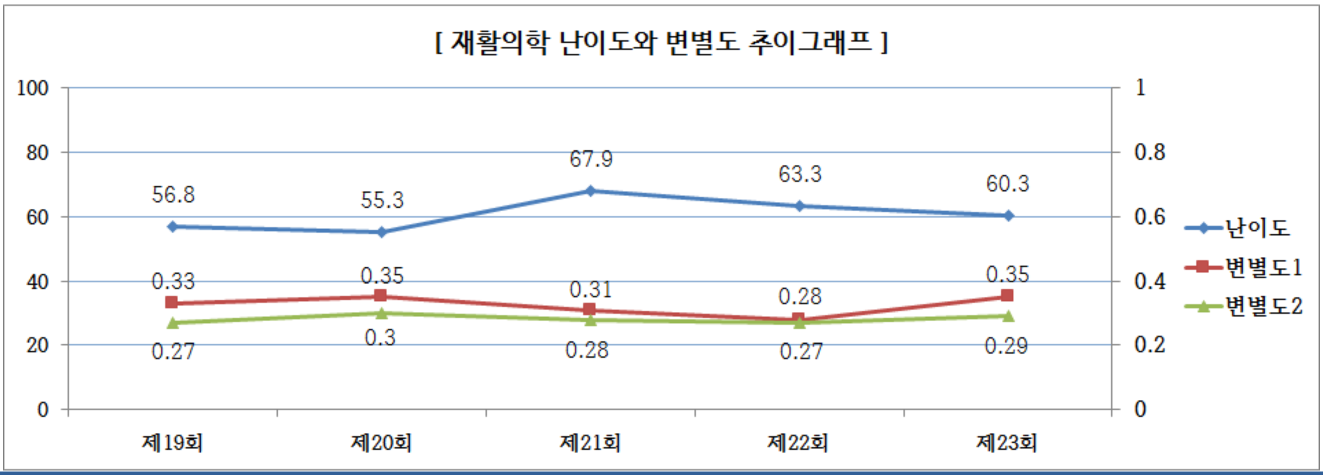

회차	난이도		변별도1		변별도2	
	평균	표준편차	평균	표준편차	평균	표준편차
제19회	56.8	16.8	.33	.19	.27	.14
제20회	55.3	19.6	.35	.22	.30	.17
제21회	67.9	16.5	.31	.20	.28	.14
제22회	63.3	17.9	.28	.15	.27	.12
**제23회**	**60.3**	**17.2**	**.35**	**.16**	**.29**	**.11**

(7) 전회 대비 의지학 난이도와 변별도

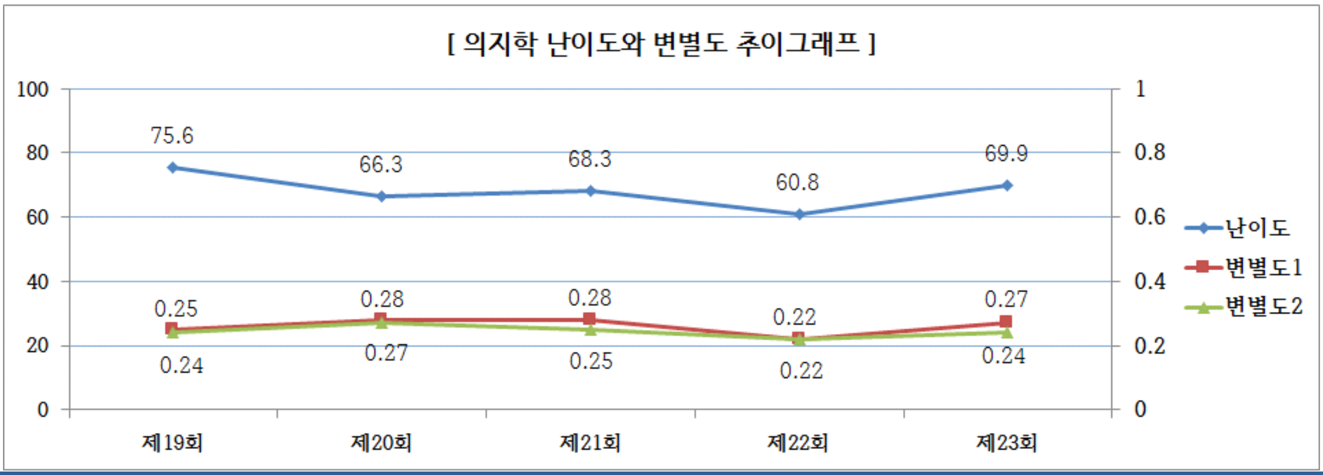

회차	난이도		변별도1		변별도2	
	평균	표준편차	평균	표준편차	평균	표준편차
제19회	60.9	20.0	.35	.18	.31	.13
제20회	53.5	20.0	.43	.20	.36	.16
제21회	57.1	21.7	.39	.16	.34	.12
제22회	61.2	17.7	.38	.17	.33	.14
**제23회**	**57.5**	**21.0**	**.31**	**.17**	**.27**	**.12**

해석

- 전회 대비 보건의료관계법규 과목의 난이도 지수는 9.1, 변별도 1 지수는 .05, 변별도 2 지수는 .02 증가함
- 운동·생체역학 과목의 난이도 지수는 9.9 증가, 변별도 1 지수는 .03, 변별도 2 지수는 .02 감소함
- 재활공학·재료학 과목의 난이도 지수는 0.2, 변별도 1 지수는 .05, 변별도 2 지수는 .01 증가함
- 보조기학 과목의 난이도 지수는 8.0, 변별도 1 지수는 .02, 변별도 2 지수는 .06 감소함
- 해부생리학 과목의 난이도 지수는 7.3 증가, 변별도 1 지수와 변별도 2 지수는 각각 .05, .03 감소함
- 재활의학 과목의 난이도 지수는 3.0 감소, 변별도 1 지수와 변별도 2 지수는 각각 .07, .02 증가함
- 의지학 과목의 난이도 지수는 3.7, 변별도 1 지수는 .07, 변별도 2 지수는 .06 감소함

나) 과목별 난이도와 변별도 분포도 및 비율분석

(1) 보건의료 관계 법규 난이도와 변별도 분포도 및 비율분석

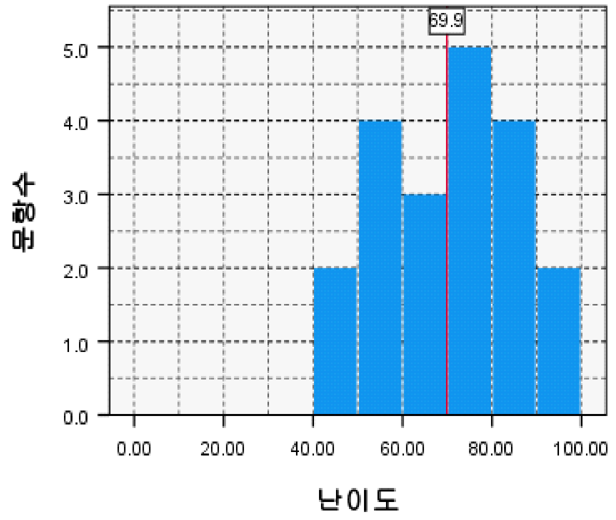

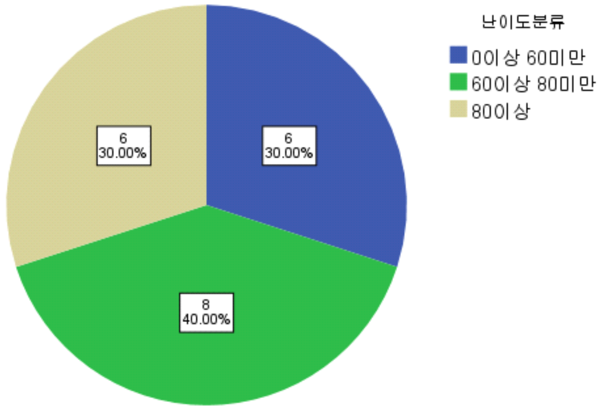

총점	난이도	표준편차
20	69.9	16.2

난이도	문항수	비율(%)
0~60미만	6	30.0
60~80미만	8	40.0
80~100	6	30.0
전체	20	100.0

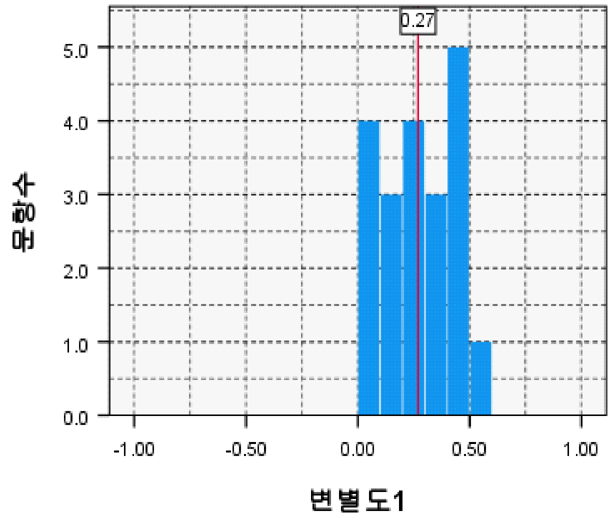

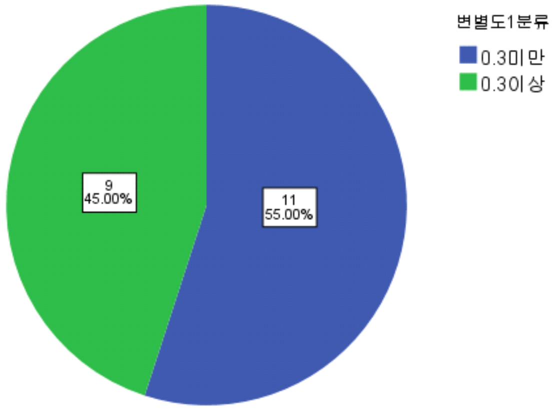

총점	변별도1	표준편차
20	.27	.14

변별도1	문항수	비율(%)
0.3미만	11	55.0
0.3이상	9	45.0
전체	20	100.0

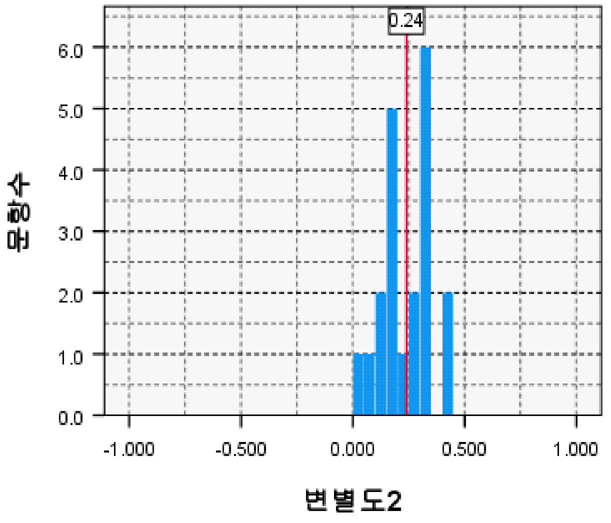

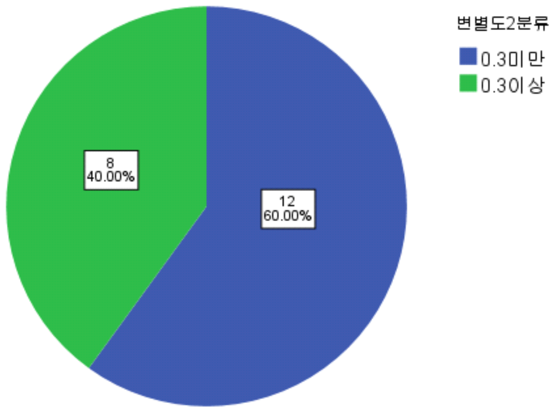

총점	변별도2	표준편차
20	.24	.11

변별도2	문항수	비율(%)
0.3미만	12	60.0
0.3이상	8	40.0
전체	20	100.0

해석

- 보건의료 관계 법규 과목에서 난이도 지수가 60에서 80 미만인 문항이 전체 20문항 중 8문항으로 가장 많았으며, 다음으로 0에서 60 미만인 문항이 6문항, 80에서 100 사이인 문항이 6문항으로 나타남
- 변별도1 지수를 기준으로 분류하였을 때, 0.3미만인 문항이 11문항으로 0.3 이상인 문항이 9문항인 것에 비해 더 많이 나타남
- 변별도2 지수를 기준으로 분류하였을 때, 0.3미만인 문항이 12문항으로 0.3 이상인 문항이 8문항인 것에 비해 더 많이 나타남

(2) 운동·생체역학 난이도와 변별도 분포도 및 비율분석

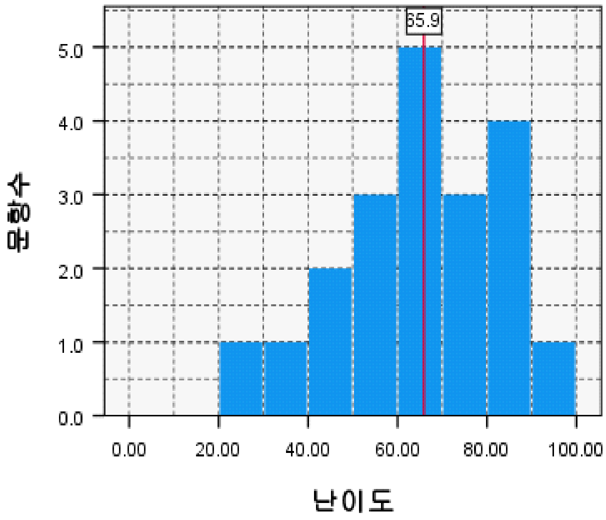

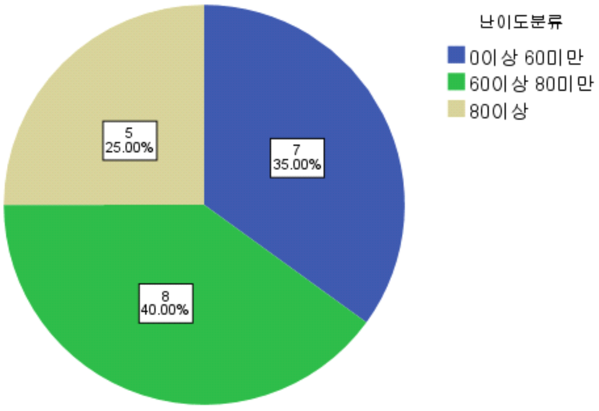

총점	난이도	표준편차
20	65.9	18.6

난이도	문항수	비율(%)
0~60미만	7	35.0
60~80미만	8	40.0
80~100	5	25.0
전체	20	100.0

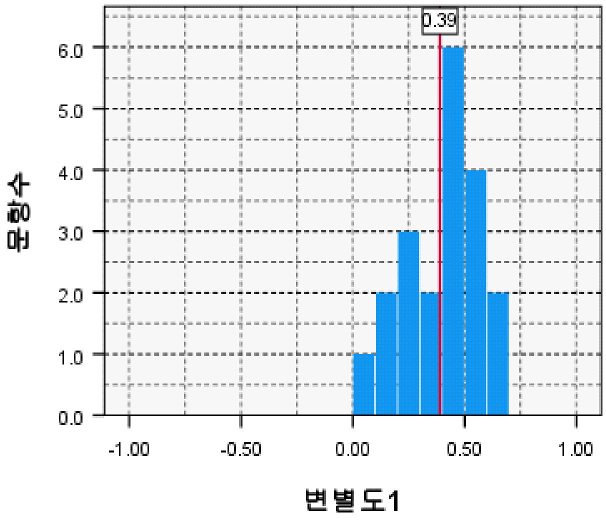

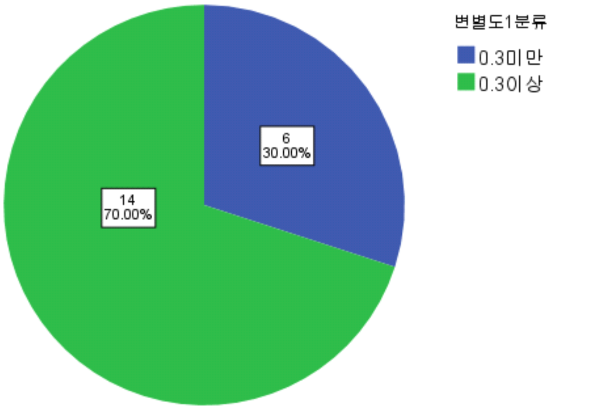

총점	변별도1	표준편차
20	.39	.17

변별도1	문항수	비율(%)
0.3미만	6	30.0
0.3이상	14	70.0
전체	20	100.0

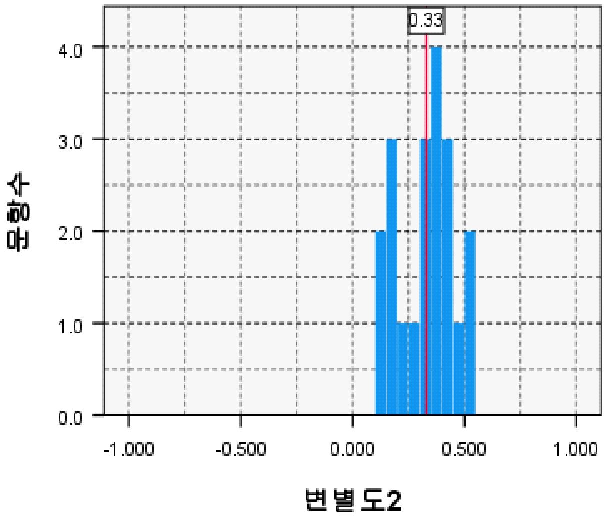

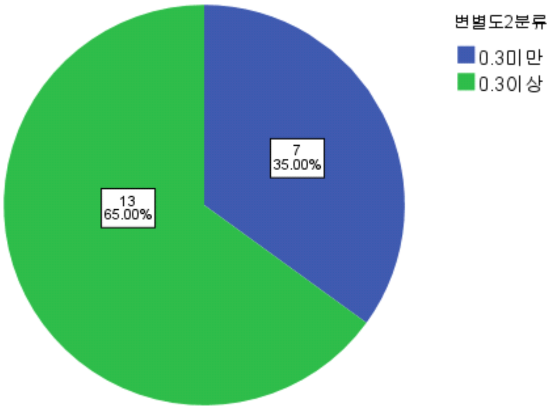

총점	변별도2	표준편차
20	.33	.12

변별도2	문항수	비율(%)
0.3미만	7	35.0
0.3이상	13	65.0
전체	20	100.0

해석

- 운동·생체역학 과목에서 난이도 지수가 60 이상 80 미만인 문항이 전체 20 문항 중 8 문항으로 가장 많았으며, 다음으로 0 에서 60 미만인 문항이 7 문항, 80 에서 100 사이인 문항이 5 문항으로 나타남
- 변별도 1 지수를 기준으로 분류하였을 때, 0.3 미만인 문항이 6 문항으로 0.3 이상인 문항이 14 문항인 것에 비해 더 적게 나타남
- 변별도 2 지수를 기준으로 분류하였을 때, 0.3 미만인 문항이 7 문항으로 0.3 이상인 문항이 13 문항인 것에 비해 더 적게 나타남

(3) 재활공학·재료공학 난이도와 변별도 분포도 및 비율분석

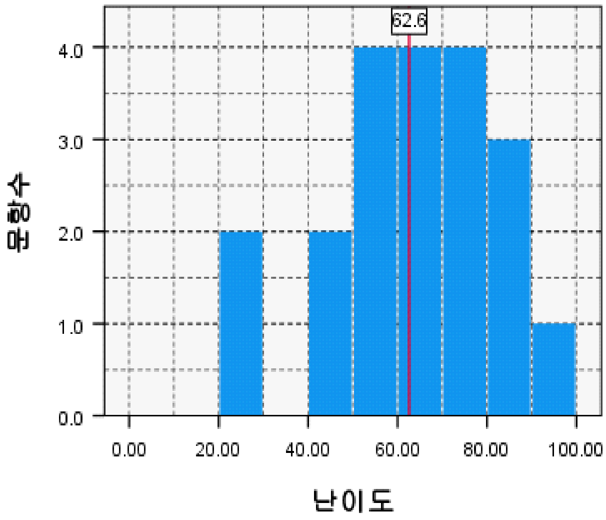

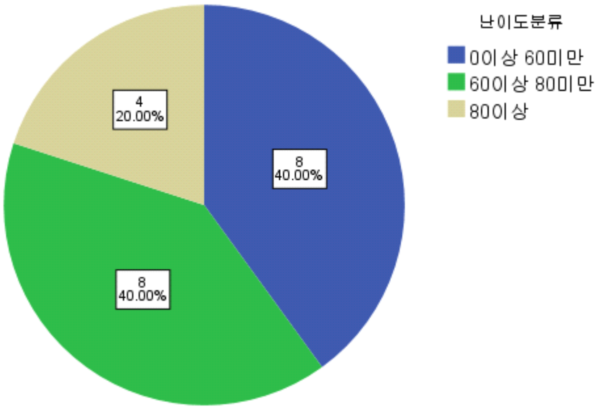

총점	난이도	표준편차
20	62.6	20.3

난이도	문항수	비율(%)
0~60미만	8	40.0
60~80미만	8	40.0
80~100	4	20.0
전체	20	100.0

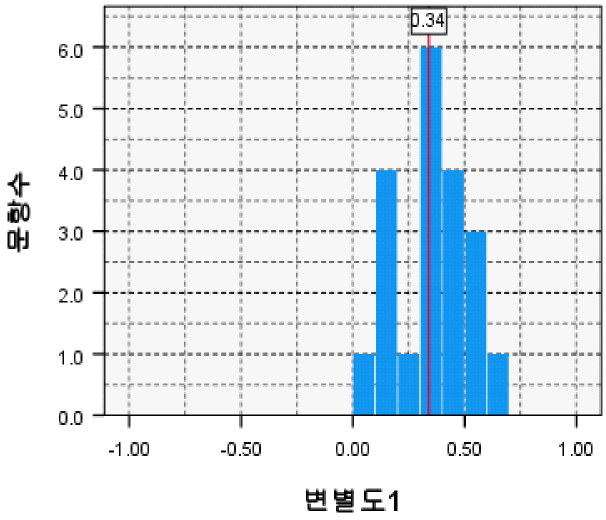

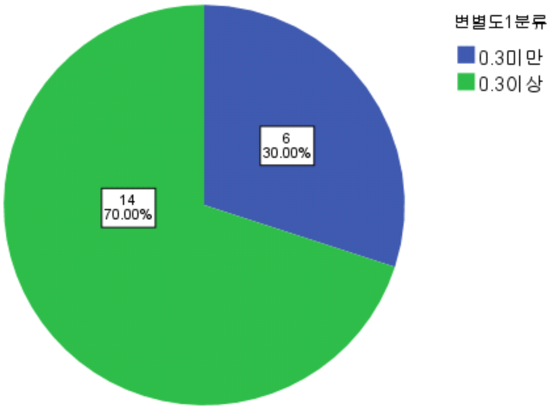

총점	변별도1	표준편차
20	.34	.16

변별도1	문항수	비율(%)
0.3미만	6	30.0
0.3이상	14	70.0
전체	20	100.0

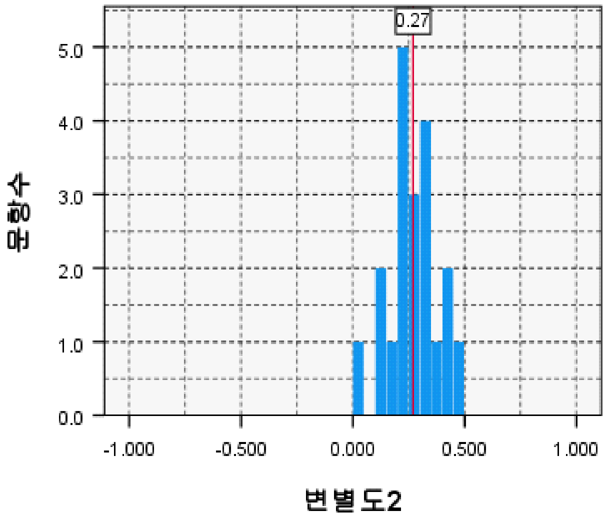

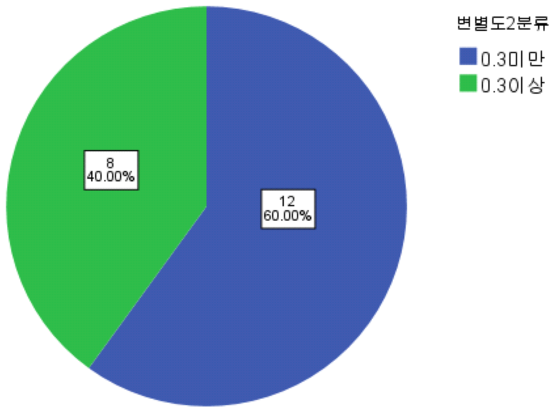

총점	변별도2	표준편차
20	.27	.11

변별도2	문항수	비율(%)
0.3미만	12	60.0
0.3이상	8	40.0
전체	20	100.0

해석

- 재활공학·재료학 과목에서 난이도 지수가 0 에서 60 미만인 문항이 전체 20 문항 중 8 문항, 60 이상에서 80 미만인 문항이 8 문항으로 가장 많았으며, 다음으로 80 에서 100 사이인 문항이 4 문항으로 나타남
- 변별도 1 지수를 기준으로 분류하였을 때, 0.3 미만인 문항이 6 문항으로 0.3 이상인 문항이 14 문항인 것에 비해 더 적게 나타남
- 변별도 2 지수를 기준으로 분류하였을 때, 0.3 미만인 문항이 12 문항으로 0.3 이상인 문항이 8 문항인 것에 비해 더 많이 나타남

(4) 보조기학 난이도와 변별도 분포도 및 비율분석

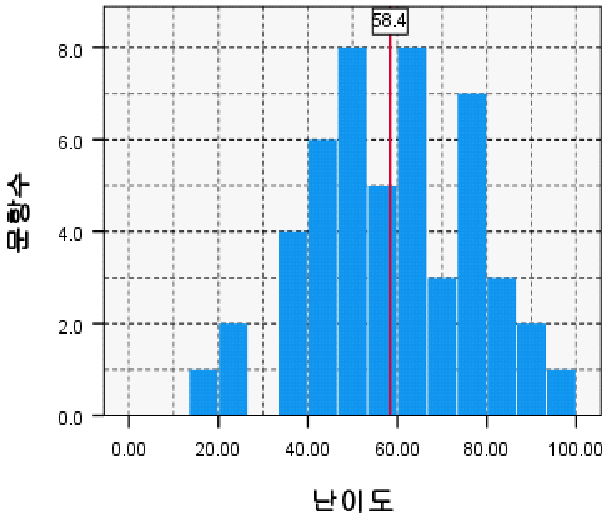

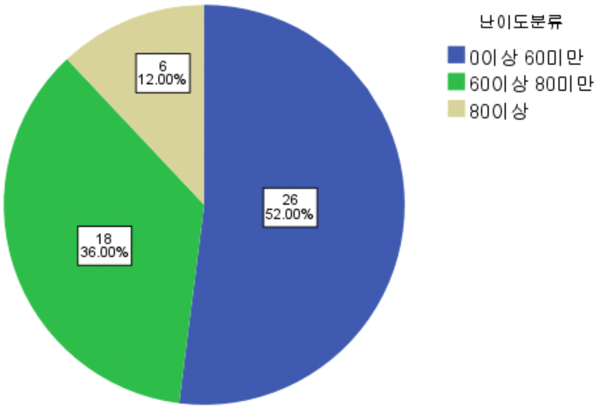

총점	난이도	표준편차
50	58.4	18.0

난이도	문항수	비율(%)
0~60미만	26	52.0
60~80미만	18	36.0
80~100	6	12.0
전체	50	100.0

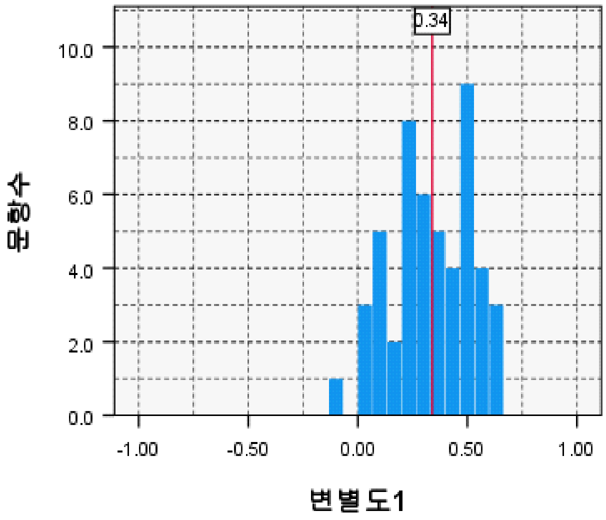

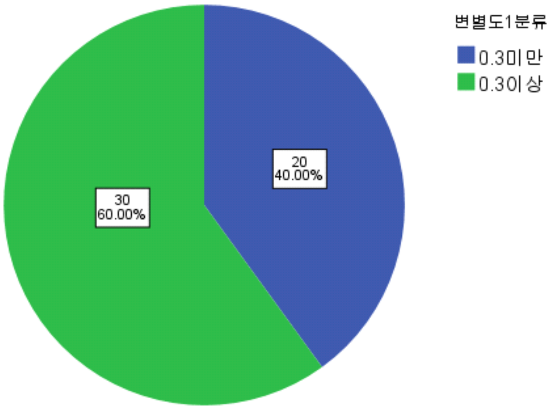

총점	변별도1	표준편차
50	.34	.18

변별도1	문항수	비율(%)
0.3미만	20	40.0
0.3이상	30	60.0
전체	50	100.0

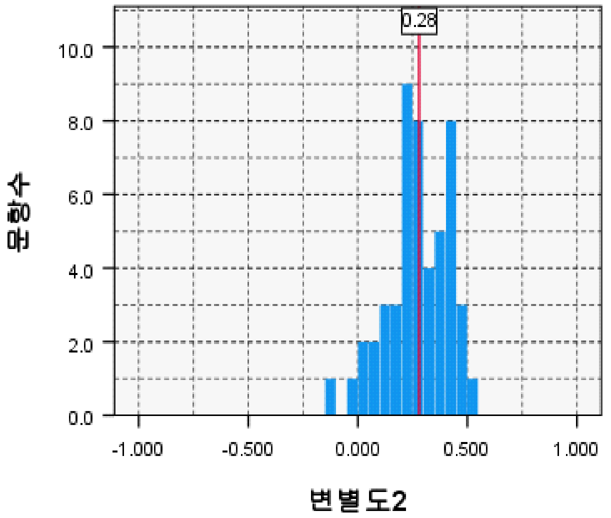

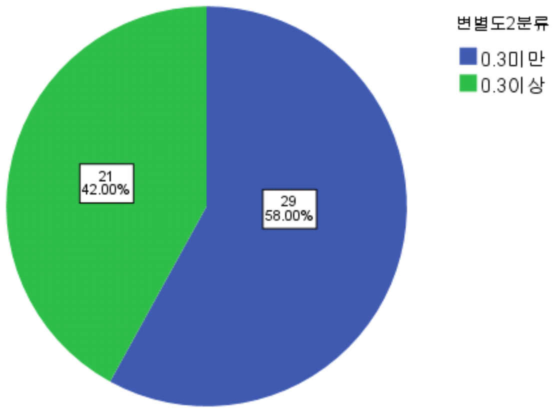

총점	변별도2	표준편차
50	.28	.14

변별도2	문항수	비율(%)
0.3미만	29	58.0
0.3이상	21	42.0
전체	50	100.0

해석

.

- 보조기학 과목에서 난이도 지수가 0에서 60미만인 문항이 전체 50문항 중 26문항으로 가장 많았으며, 다음으로 60이상 80 미만인 문항이이 18문항, 80에서 100 사이인 문항이 6문항으로 나타남
- 변별도1 지수를 기준으로 분류하였을 때, 0.3미만인 문항이 20문항으로 0.3이상인 문항이 30문항인 것에 비해 더 적게 나타남
- 변별도2 지수를 기준으로 분류하였을 때, 0.3미만인 문항이 29문항으로 0.3이상인 문항이 21문항인 것에 비해 더 많이 나타남

(5) 해부·생리학 난이도와 변별도 분포도 및 비율분석

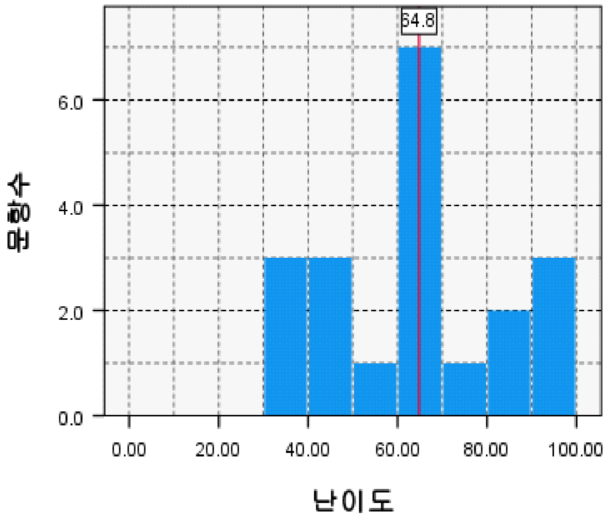

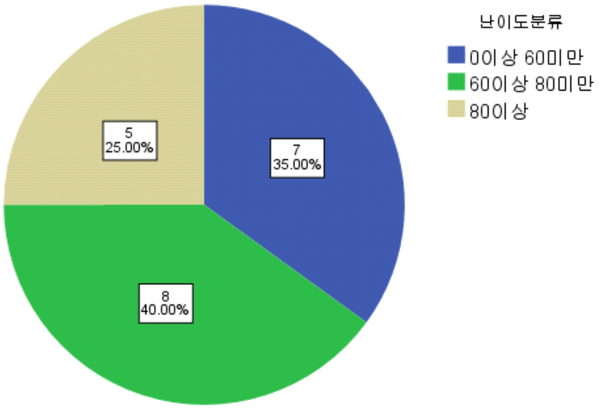

총점	난이도	표준편차
20	62.8	20.8

난이도	문항수	비율(%)
0~60미만	7	35.0
60~80미만	8	40.0
80~100	5	25.0
전체	20	100.0

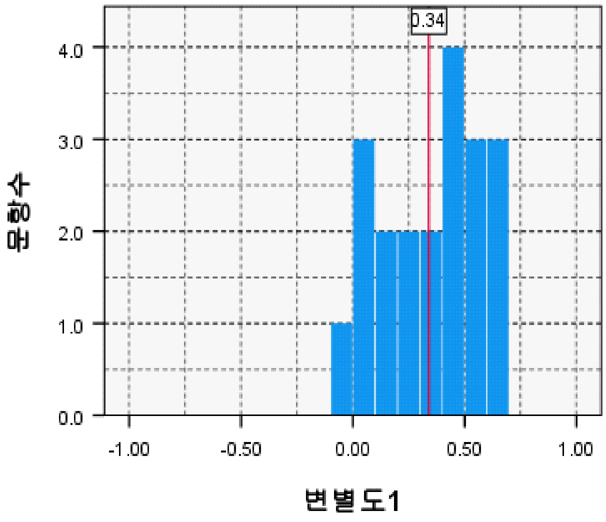

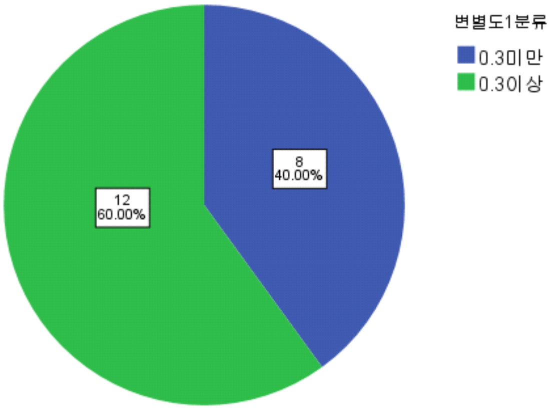

총점	변별도1	표준편차
20	.34	.21

변별도1	문항수	비율(%)
0.3미만	8	40.0
0.3이상	12	60.0
전체	20	100.0

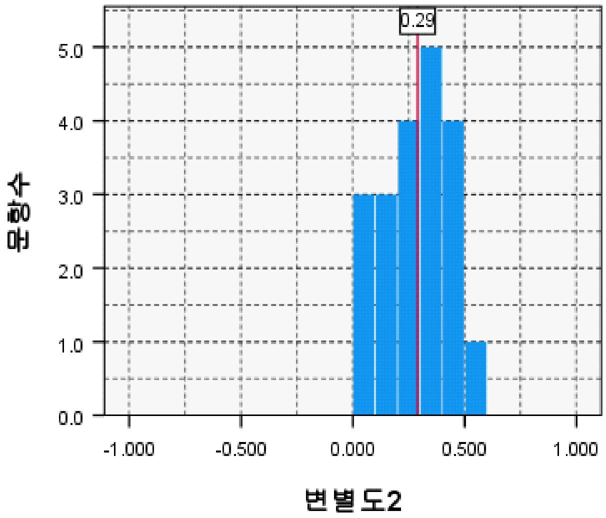

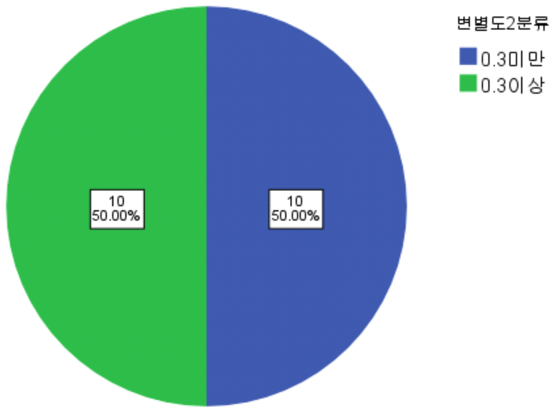

총점	변별도2	표준편차
20	.29	.15

변별도2	문항수	비율(%)
0.3미만	10	50.0
0.3이상	10	50.0
전체	20	100.0

해석

- 해부·생리학 과목에서 난이도 지수가 60에서 80 사이인 문항이 전체 20문항 중 8문항으로 가장 많았으며, 다음으로 0에서 60 미만인 문항이 7문항, 80이상 100미만인 문항이 5문항으로 나타남
- 변별도1 지수를 기준으로 분류하였을 때, 0.3미만인 문항이 8문항으로 0.3이상인 문항이 12문항인 것에 비해 더 적게 나타남
- 변별도2 지수를 기준으로 분류하였을 때, 0.3미만인 문항이 10문항으로 0.3이상인 문항이 10문항인 것으로 동일하게 나타남

(6) 재활의학 난이도와 변별도 분포도 및 비율분석

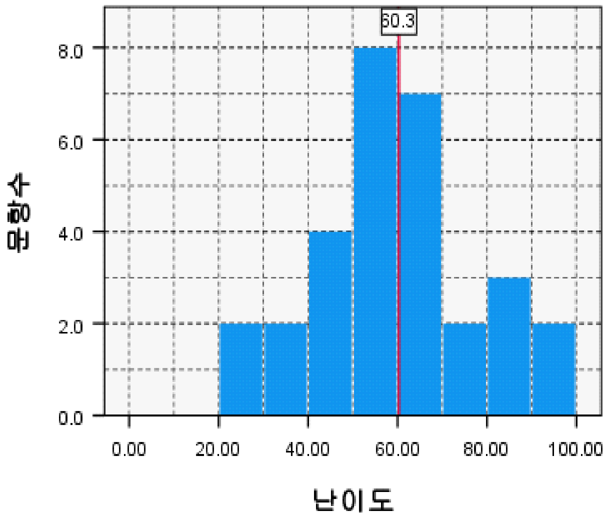

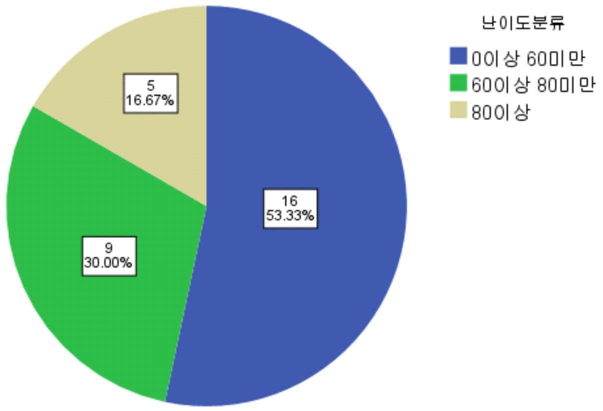

총점	난이도	표준편차
30	60.3	17.5

난이도	문항수	비율(%)
0~60미만	16	53.3
60~80미만	9	30.0
80~100	5	16.7
전체	30	100.0

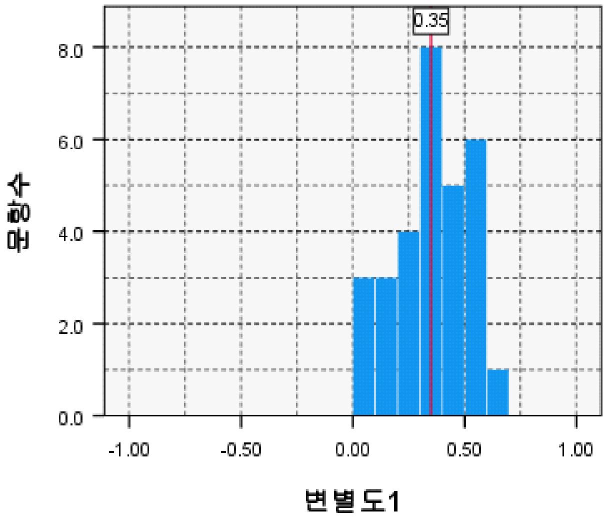

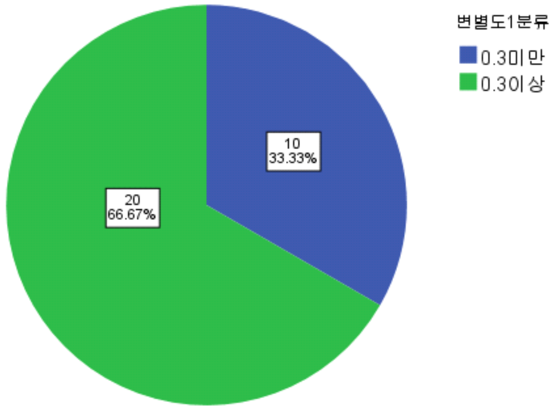

총점	변별도1	표준편차
30	.35	.16

변별도1	문항수	비율(%)
0.3미만	10	33.3
0.3이상	20	66.7
전체	30	100.0

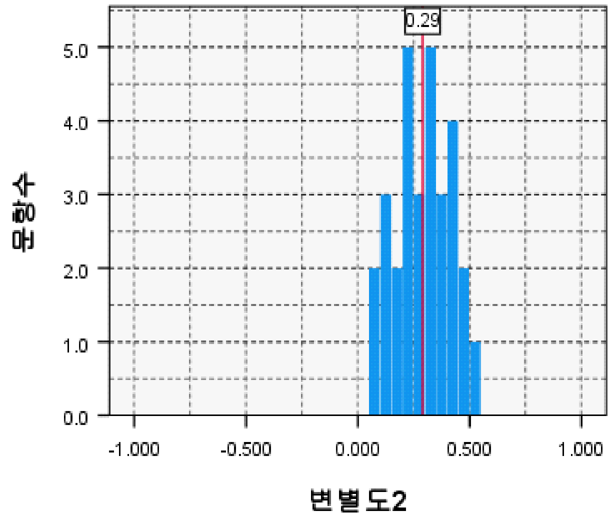

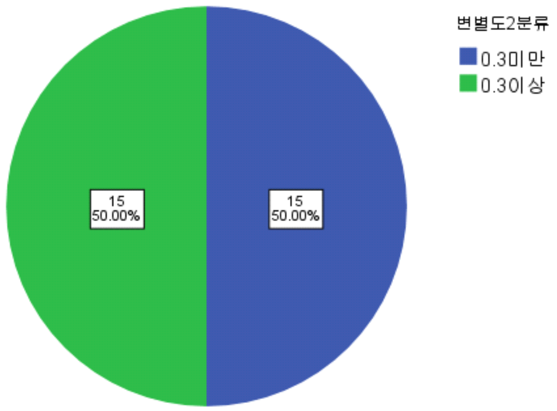

총점	변별도2	표준편차
30	.29	.12

변별도2	문항수	비율(%)
0.3미만	15	50.0
0.3이상	15	50.0
전체	30	100.0

해석

- 재활의학 과목에서 난이도 지수가 0 이상 60 미만인 문항이 전체 30 문항 중 16 문항으로 가장 많았으며, 다음으로 60 에서 80 사이인 문항이 9 문항, 80 에서 100 미만인 문항이 5 문항으로 나타남
- 변별도 1 지수를 기준으로 분류하였을 때, 0.3 미만인 문항이 10 문항으로 0.3 이상인 문항이 20 문항인 것에 비해 더 많이 나타남
- 변별도 2 지수를 기준으로 분류하였을 때, 0.3 미만인 문항이 15 문항으로 0.3 이상인 문항이 15 문항인 것에 비해 동일하게 나타남

(7) 의지학 난이도와 변별도 분포도 및 비율분석

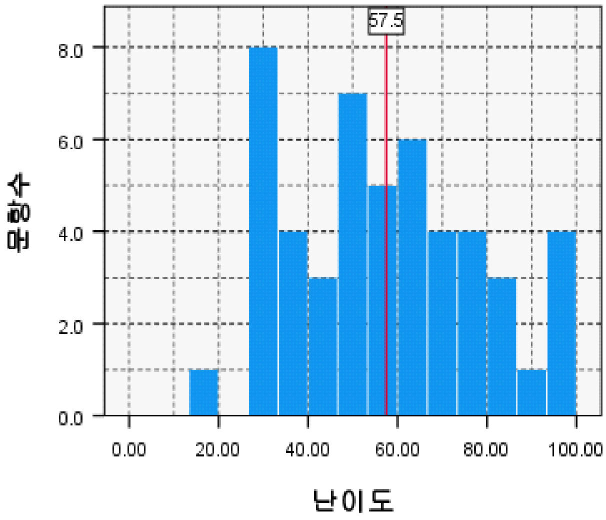

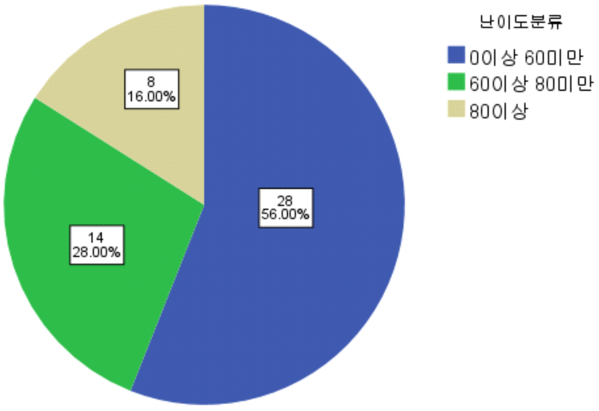

총점	난이도	표준편차
50	57.5	21.2

난이도	문항수	비율(%)
0~60미만	28	56.0
60~80미만	14	28.0
80~100	8	16.0
전체	50	100.0

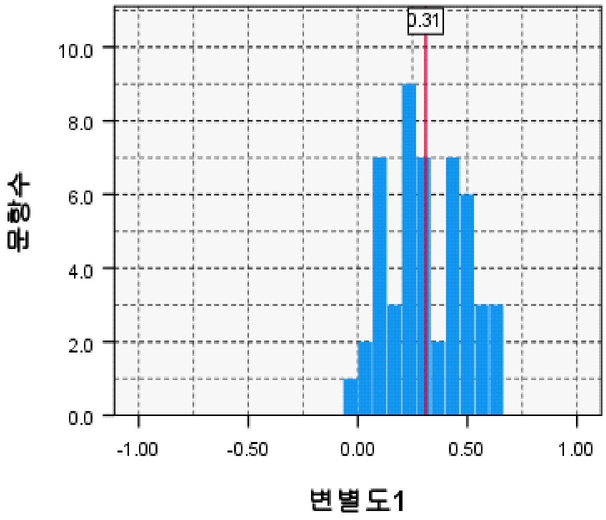

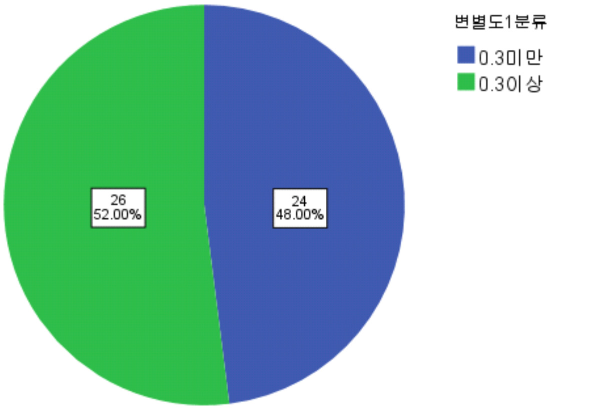

총점	변별도1	표준편차
50	.31	.17

변별도1	문항수	비율(%)
0.3미만	24	48.0
0.3이상	26	52.0
전체	50	100.0

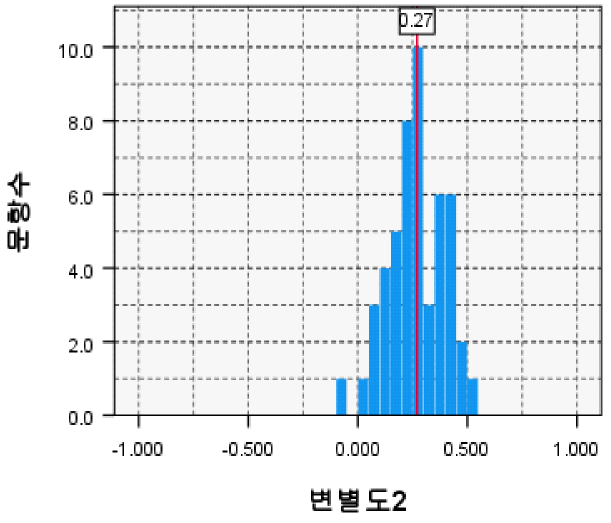

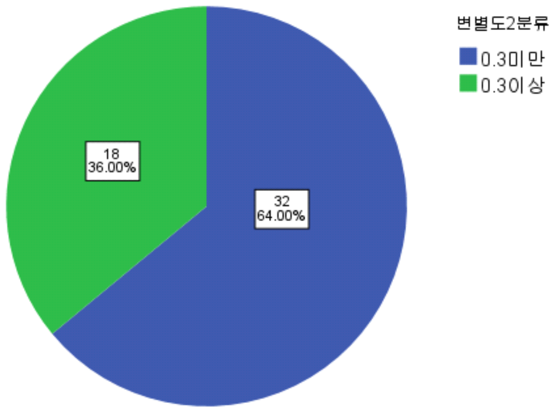

총점	변별도2	표준편차
50	.27	.12

변별도2	문항수	비율(%)
0.3미만	32	64.0
0.3이상	18	36.0
전체	50	100.0

해석

- 의지학 과목에서 난이도 지수가 0에서 60사이인 문항이 전체 50문항 중 28문항으로 가장 많았으며, 다음으로 60이상 80미만인 문항이 14문항, 80에서 100 사이인 문항이 8문항으로 나타남
- 변별도1 지수를 기준으로 분류하였을 때, 0.3미만인 문항이 24문항으로 0.3이상인 문항이 26문항인 것에 비해 더 적게 나타남
- 변별도2 지수를 기준으로 분류하였을 때, 0.3미만인 문항이 32문항으로 0.3이상인 문항이 18문항인 것에 비해 더 많이 나타남

3) 지식수준별 난이도와 변별도

가) 전회 대비 지식수준별 난이도와 변별도

(1) 전회 대비 암기형 난이도와 변별도

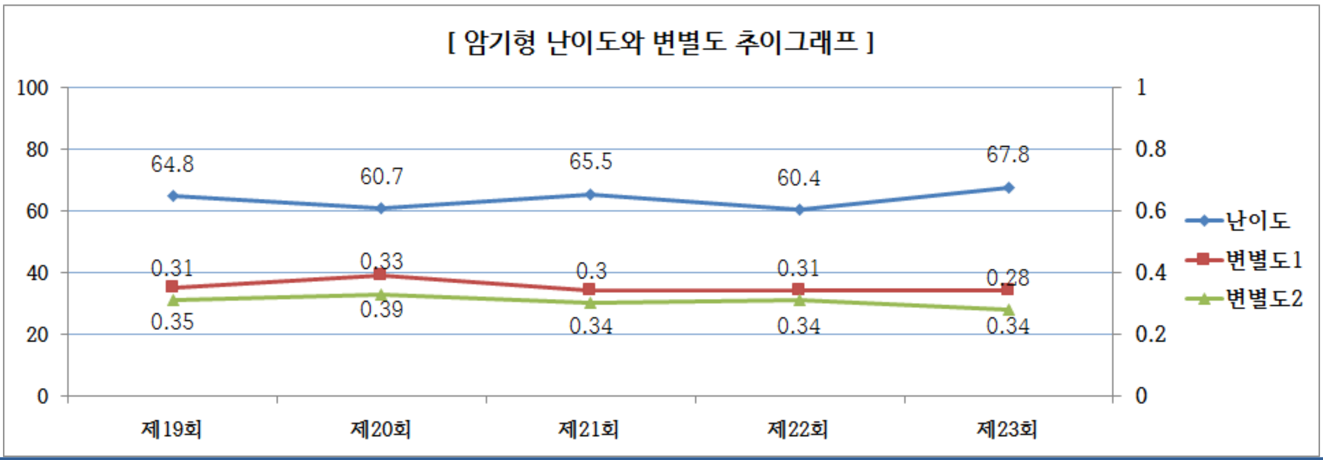

회차	난이도		변별도1		변별도2	
	평균	표준편차	평균	표준편차	평균	표준편차
제19회	64.8	19.7	.35	.20	.31	.14
제20회	60.7	18.8	.39	.19	.33	.15
제21회	65.5	19.9	.34	.21	.30	.15
제22회	60.4	20.8	.34	.17	.31	.14
**제23회**	**67.8**	**17.5**	**.34**	**.17**	**.28**	**.12**

(2) 전회 대비 해석형 난이도와 변별도

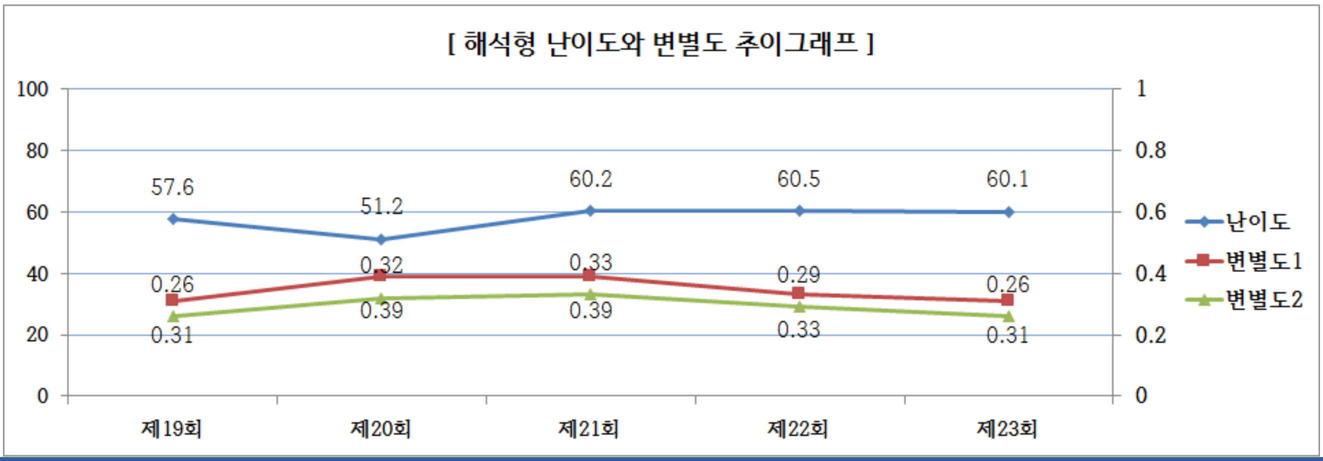

회차	난이도		변별도1		변별도2	
	평균	표준편차	평균	표준편차	평균	표준편차
제19회	57.6	20.1	.31	.21	.26	.16
제20회	51.2	18.6	.39	.20	.32	.17
제21회	60.2	18.5	.39	.18	.33	.14
제22회	60.5	18.4	.33	.16	.29	.12
**제23회**	**60.1**	**19.5**	**.31**	**.18**	**.26**	**.13**

(3) 전회 대비 해결형 난이도와 변별도

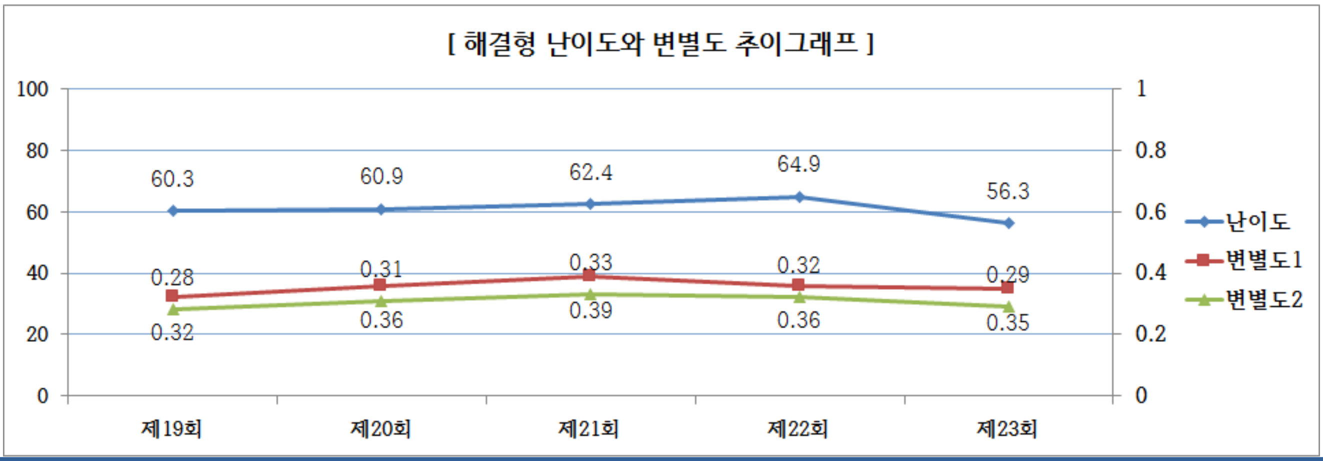

회차	난이도		변별도1		변별도2	
	평균	표준편차	평균	표준편차	평균	표준편차
제19회	60.3	18.4	.32	.19	.28	.14
제20회	60.9	20.5	.36	.22	.31	.18
제21회	62.4	18.3	.39	.17	.33	.12
제22회	64.9	15.6	.36	.18	.32	.14
**제23회**	**56.3**	**18.8**	**.35**	**.17**	**.29**	**.13**

해석

- 전회 대비 암기형 문항의 난이도 지수는 7.4 증가하였으며, 변별도 1 지수는 동일 하였으며 변별도 2 지수는 .03 감소함
- 해석형 문항의 난이도 지수는 .4, 변별도 1 지수는 .02, 변별도 2 지수는 .03 감소함
- 해결형 문항의 난이도 지수는 8.6, 변별도 1 지수는 .01, 변별도 2 지수는 .03 감소함

나) 지식수준별 난이도와 변별도 분포도 및 비율분석

(1) 암기형 난이도와 변별도 분포도 및 비율분석

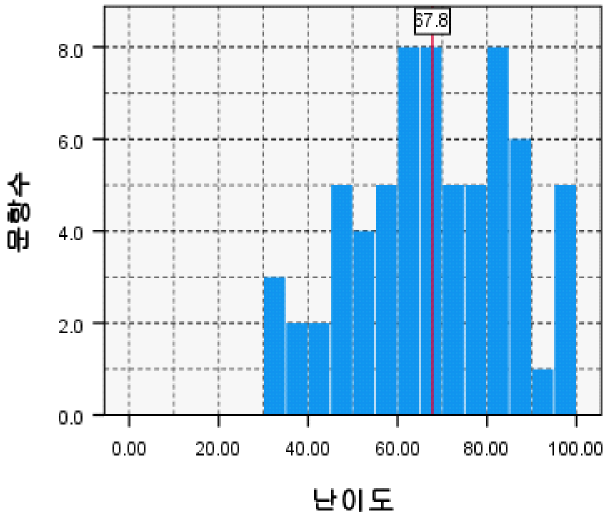

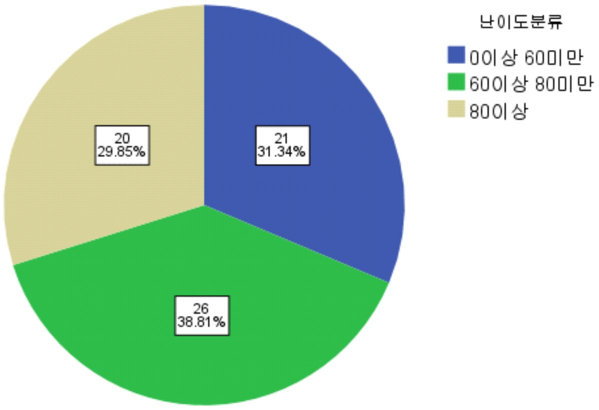

총점	난이도	표준편차	난이도	문항수	비율(%)
67	67.8	17.7	0~60미만	21	31.3
			60~80미만	26	38.8
			80~100	20	29.9
			전체	67	100.0

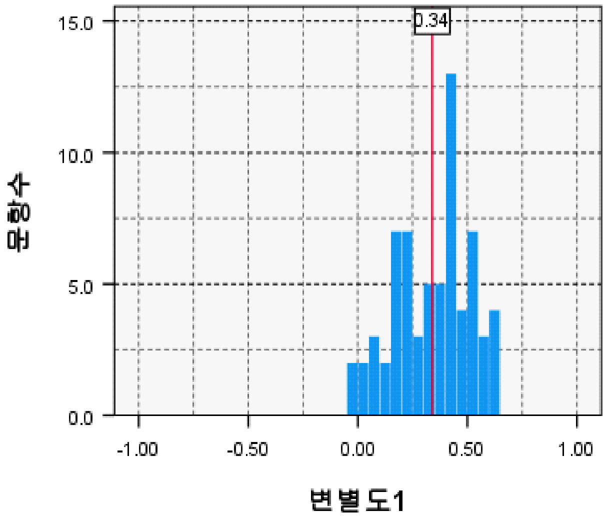

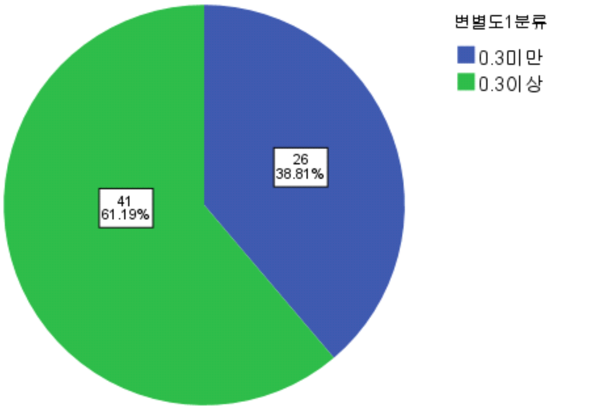

총점	변별도1	표준편차	변별도1	문항수	비율(%)
67	.34	.17	0.3미만	26	38.8
			0.3이상	41	61.2
			전체	67	100.0

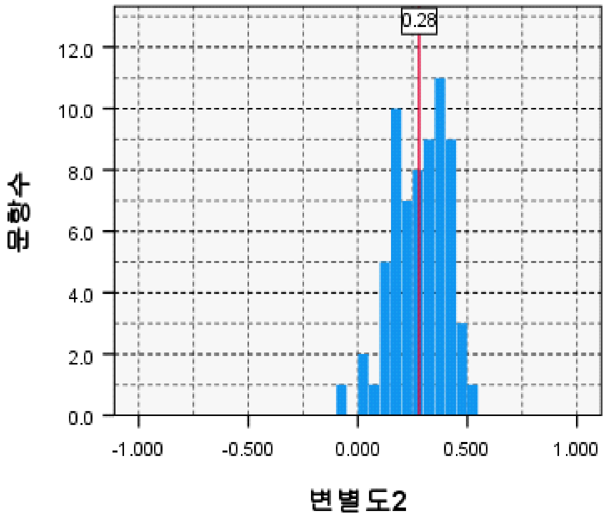

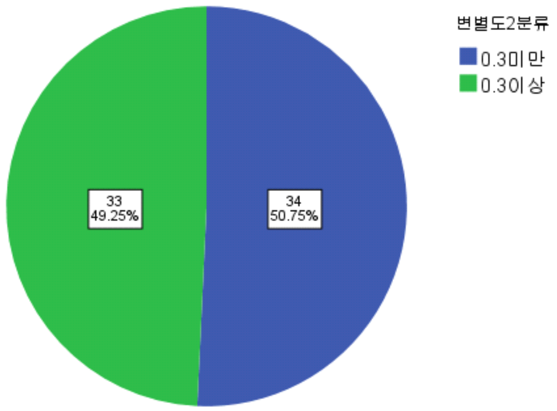

총점	변별도2	표준편차
67	.28	.12

변별도2	문항수	비율(%)
0.3미만	34	50.7
0.3이상	33	49.3
전체	67	100.0

해석

- 암기형 문항에서 난이도 지수가 60 이상 80 미만인 문항이 전체 67 문항 중 26 문항으로 가장 많았으며, 다음으로 0 에서 60 미만인 문항이 21 문항, 80 에서 100 사이인 문항이 20 문항으로 나타남
- 변별도 1 지수를 기준으로 분류하였을 때, 0.3 미만인 문항이 26 문항으로 0.3 이상인 문항이 41 문항인 것에 비해 더 적게 나타남
- 변별도 2 지수를 기준으로 분류하였을 때, 0.3 미만인 문항이 34 문항으로 0.3 이상인 문항이 33 문항인 것에 비해 더 많이 나타남

(2) 해석형 난이도와 변별도 분포도 및 비율분석

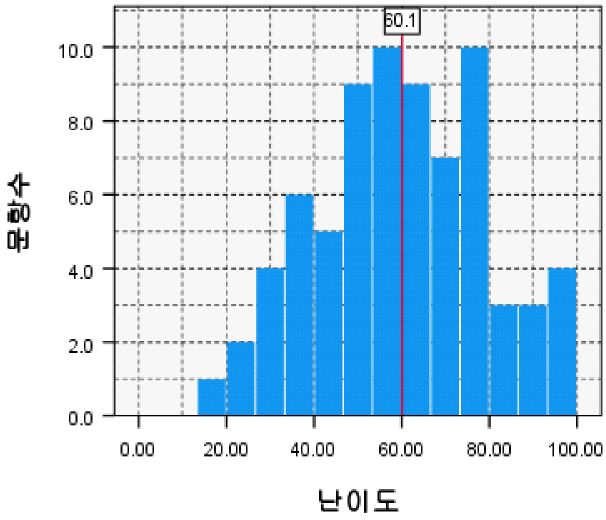

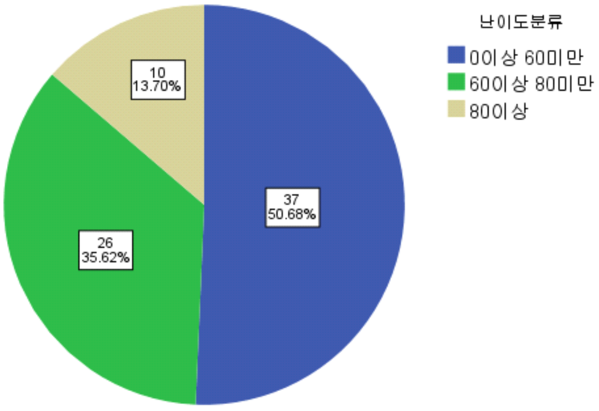

총점	난이도	표준편차
73	60.1	19.6

난이도	문항수	비율(%)
0~60미만	37	50.7
60~80미만	26	35.6
80~100	10	13.7
전체	73	100.0

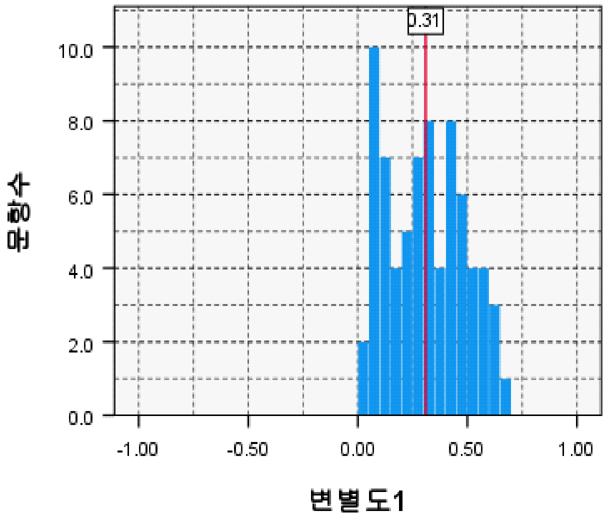

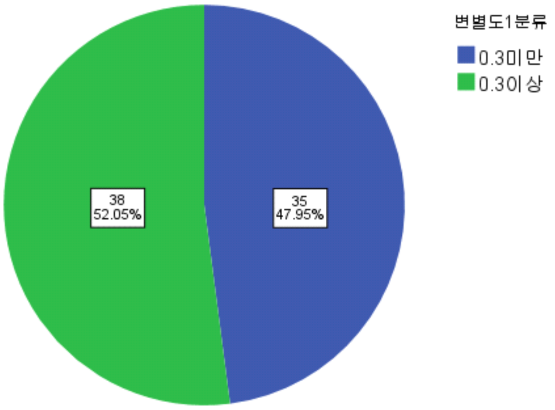

총점	변별도1	표준편차
73	0.31	0.18

변별도1	문항수	비율(%)
0.3미만	35	47.9
0.3이상	38	52.1
전체	73	100.0

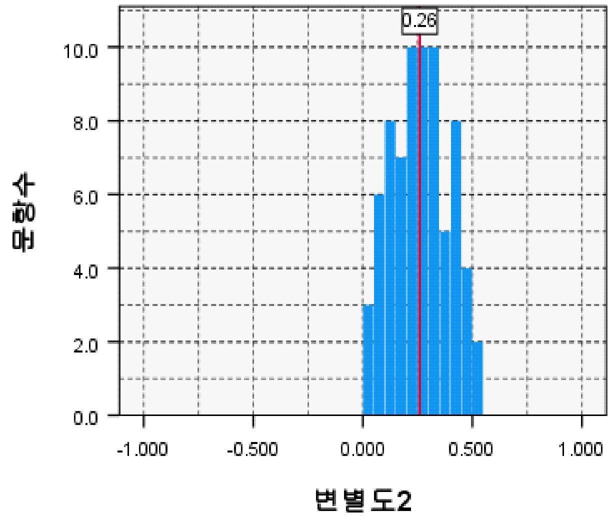

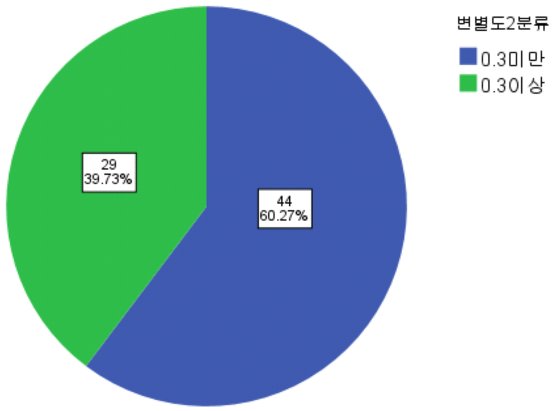

총점	변별도2	표준편차
73	.26	.13

변별도2	문항수	비율(%)
0.3미만	44	60.3
0.3이상	29	39.7
전체	73	100.0

해석

- 해석형 문항에서 난이도 지수가 0 에서 60 미만인 문항이 전체 73 문항 중 37 문항으로 가장 많았으며, 다음으로 60 이상 80 미만인 문항이 26 문항, 80 에서 100 사이인 문항이 10 문항으로 나타남
- 변별도 1 지수를 기준으로 분류하였을 때, 0.3 미만인 문항이 35 문항으로 0.3 이상인 문항이 38 문항인 것에 비해 더 적게 나타남
- 변별도 2 지수를 기준으로 분류하였을 때, 0.3 미만인 문항이 44 문항으로 0.3 이상인 문항이 29 문항인 것에 비해 더 많이 나타남

(3) 해결형 난이도와 변별도 분포도 및 비율분석

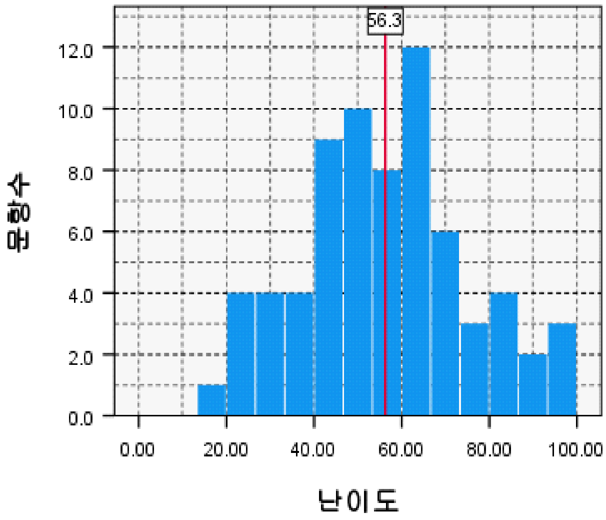

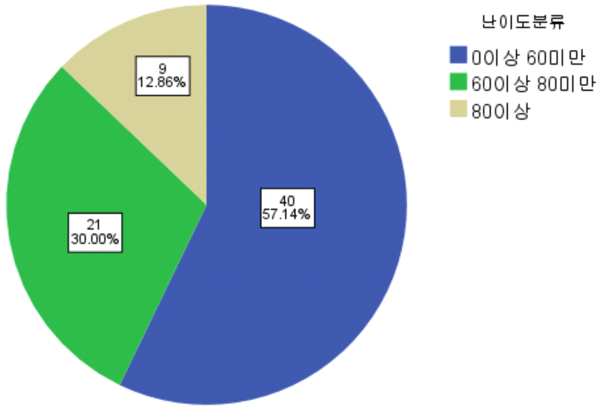

총점	난이도	표준편차
70	56.3	18.9

난이도	문항수	비율(%)
0~60미만	40	57.1
60~80미만	21	30.0
80~100	9	12.9
전체	70	100.0

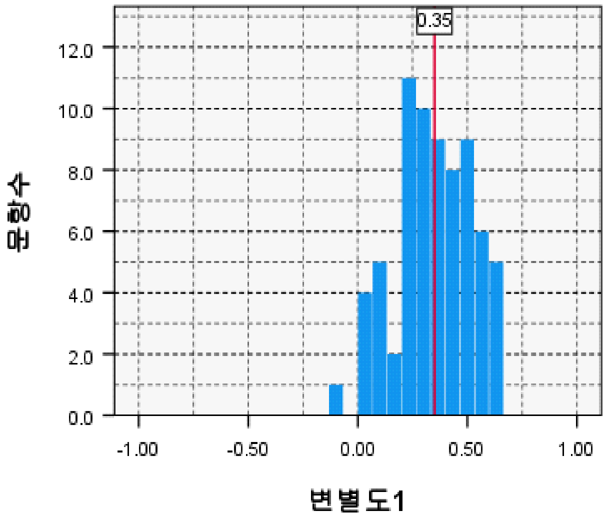

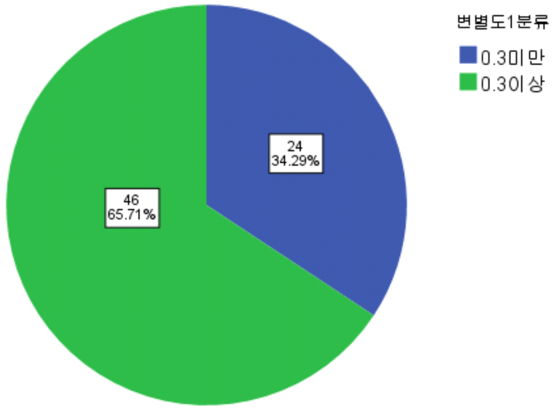

총점	변별도1	표준편차
70	.35	.17

변별도1	문항수	비율(%)
0.3미만	24	34.3
0.3이상	46	65.7
전체	70	100.0

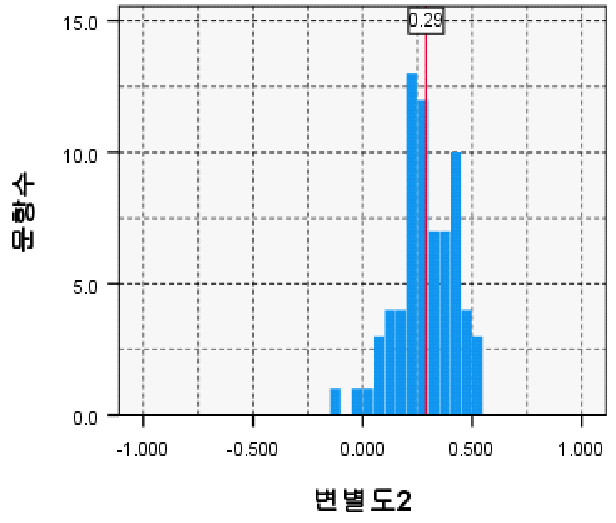

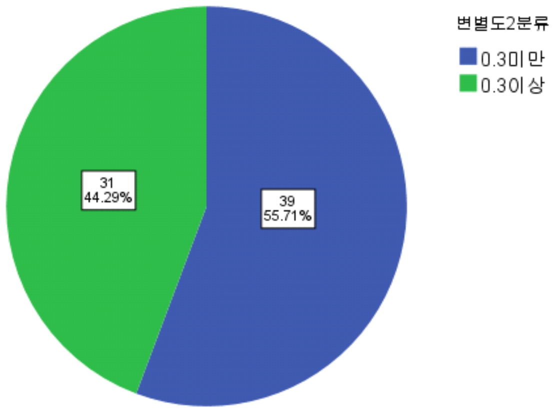

총점	변별도2	표준편차
70	.29	.13

변별도2	문항수	비율(%)
0.3미만	39	55.7
0.3이상	31	44.3
전체	70	100.0

해석

- 해결형 문항에서 난이도 지수가 0 이상 60 미만인 문항이 전체 70 문항 중 40 문항으로 가장 많았으며, 다음으로 60 에서 80 미만인 문항이 21 문항, 80 에서 100 사이인 문항이 9 문항으로 나타남
- 변별도 1 지수를 기준으로 분류하였을 때, 0.3 미만인 문항이 24 문항으로 0.3 이상인 문항이 46 문항인 것에 비해 더 적게 나타남
- 변별도 2 지수를 기준으로 분류하였을 때, 0.3 미만인 문항이 39 문항으로 0.3 이상인 문항이 31 문항인 것에 비해 더 많이 나타남

4) 자료유형별 난이도와 변별도

가) 전회 대비 자료유형별 난이도와 변별도

(1) 전회 대비 텍스트형 난이도와 변별도

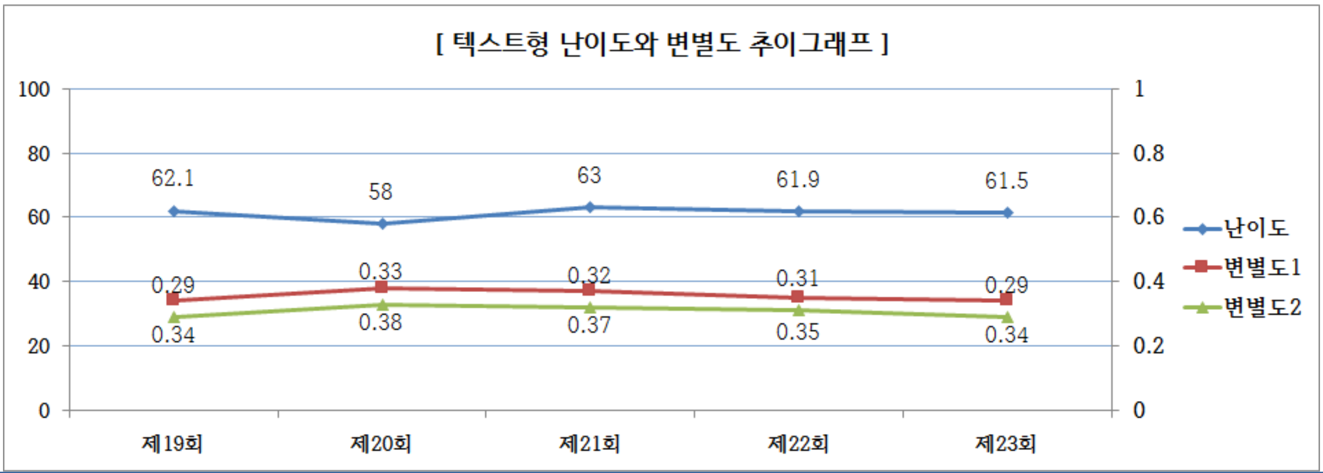

회차	난이도		변별도1		변별도2	
	평균	표준편차	평균	표준편차	평균	표준편차
제19회	62.1	19.4	.34	.20	.29	.14
제20회	58.0	20.0	.38	.20	.33	.16
제21회	63.0	19.1	.37	.19	.32	.14
제22회	61.9	17.9	.35	.17	.31	.13
**제23회**	**61.5**	**18.7**	**.34**	**.17**	**.29**	**.12**

(2) 전회 대비 자료제시형 난이도와 변별도

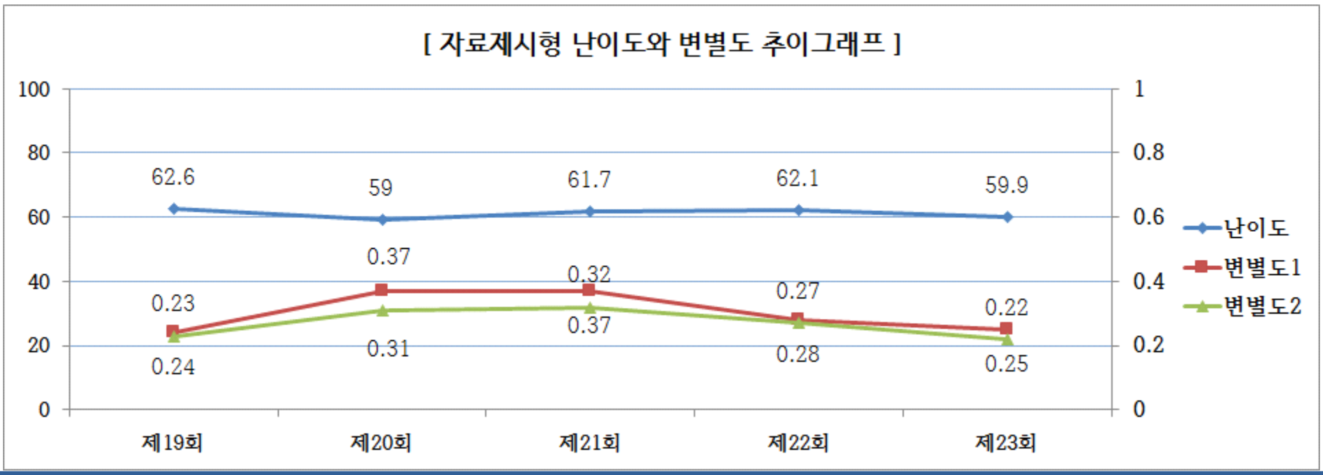

회차	난이도		변별도1		변별도2	
	평균	표준편차	평균	표준편차	평균	표준편차
제19회	62.6	21.4	.24	.13	.23	.12
제20회	59.0	18.7	.37	.20	.31	.16
제21회	61.7	19.0	.37	.18	.32	.13
제22회	62.1	22.2	.28	.15	.27	.12
**제23회**	**59.9**	**22.0**	**.25**	**.16**	**.22**	**.13**

해석

- 전회 대비 텍스트형 문항의 난이도 지수는 0.4, 변별도 1 지수는 .01, 변별도 2 지수는 .02 감소함
- 자료제시형 문항의 난이도 지수는 2.2, 변별도 1 시주는 .03, 변별도 2 지수는 .05 감소함

나) 자료유형별 난이도와 변별도 분포도 및 비율분석

(1) 텍스트형 난이도와 변별도 분포도 및 비율분석

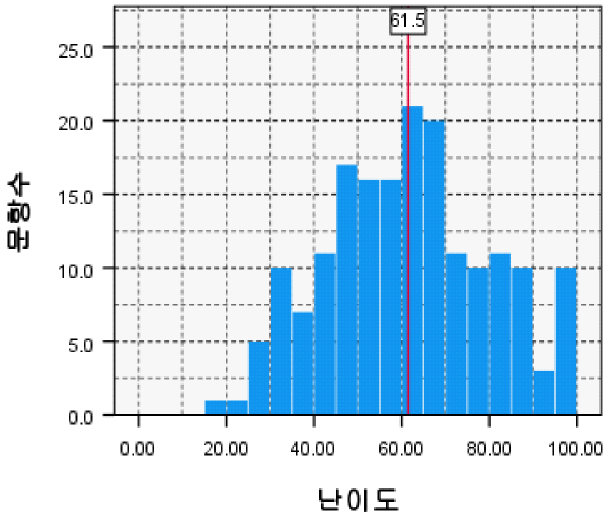

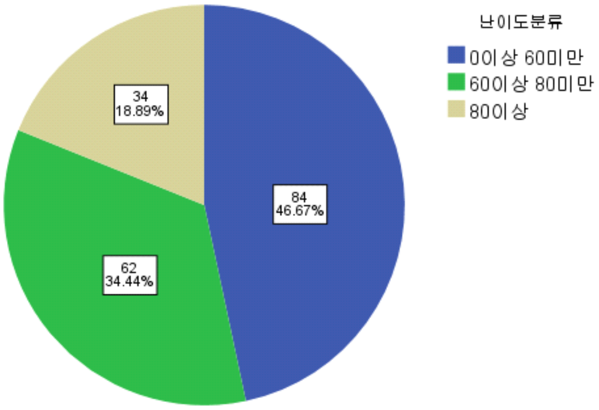

총점	난이도	표준편차
180	61.5	18.8

난이도	문항수	비율(%)
0~60미만	84	46.7
60~80미만	62	34.4
80~100	34	18.9
전체	180	100.0

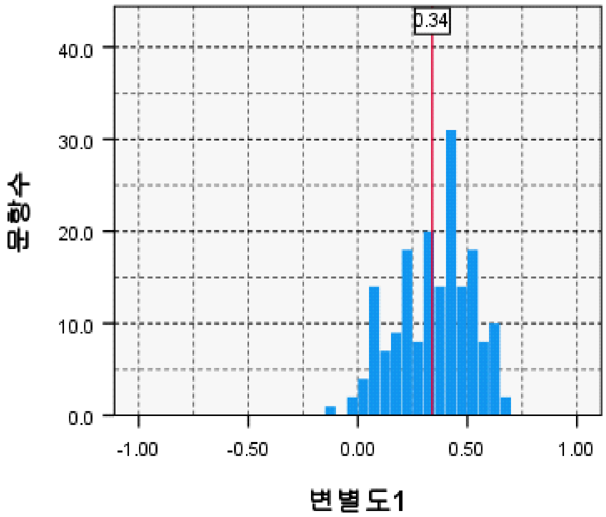

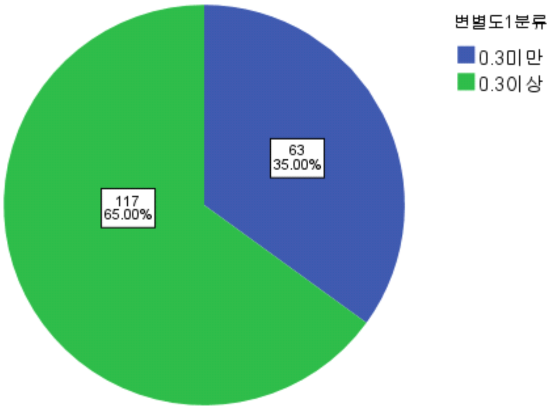

총점	변별도1	표준편 차
180	.34	.17

변별도1	문항수	비율(%)
0.3미만	63	35.0
0.3이상	117	65.0
전체	181	100.0

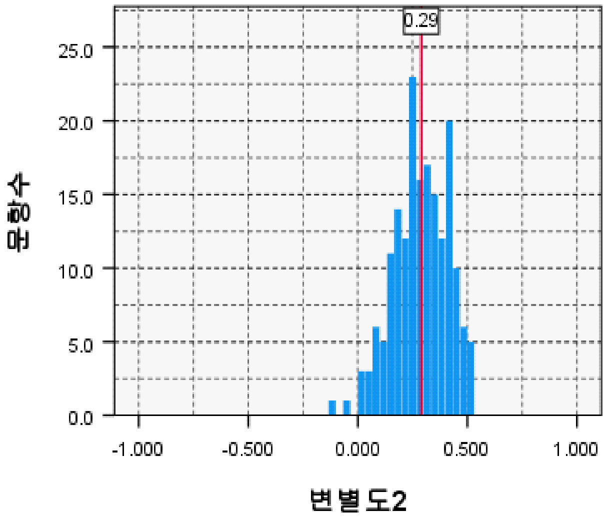

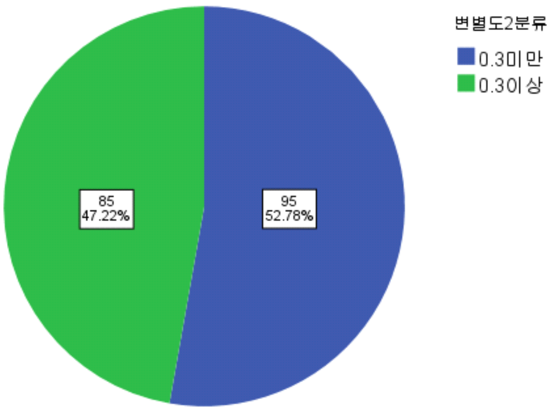

총점	변별도2	표준편차
180	.29	.12

변별도2	문항수	비율(%)
0.3미만	95	52.8
0.3이상	85	47.2
전체	180	100.0

해석

- 텍스트형 문항에서 난이도 지수가 0 에서 60 미만인 문항인 문항이 전체 180 문항 중 84 문항으로 가장 많았으며, 다음으로 60 이상 80 미만인 문항이 62 문항, 80 에서 100 사이인 문항이 34 문항인 것으로 나타남
- 변별도 1 지수를 기준으로 분류하였을 때, 0.3 미만인 문항이 63 문항으로 0.3 이상인 문항이 117 문항인 것에 비해 더 적게 나타남
- 변별도 2 지수를 기준으로 분류하였을 때, 0.3 미만인 문항이 95 문항으로 0.3 이상인 문항이 85 문항인 것에 비해 더 적게 나타남

(2) 자료제시형 난이도와 변별도 분포도 및 비율분석

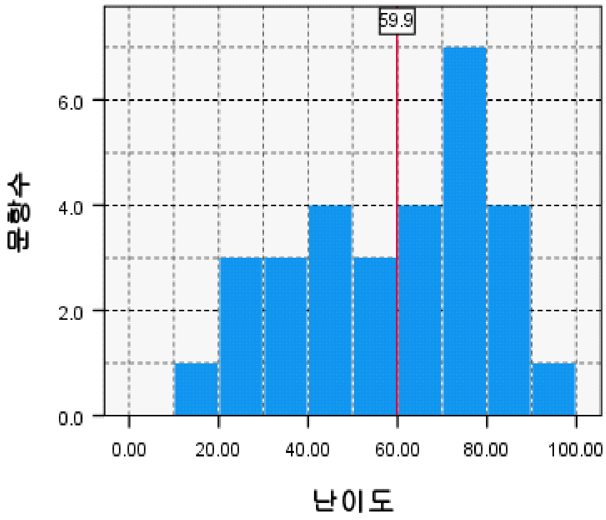

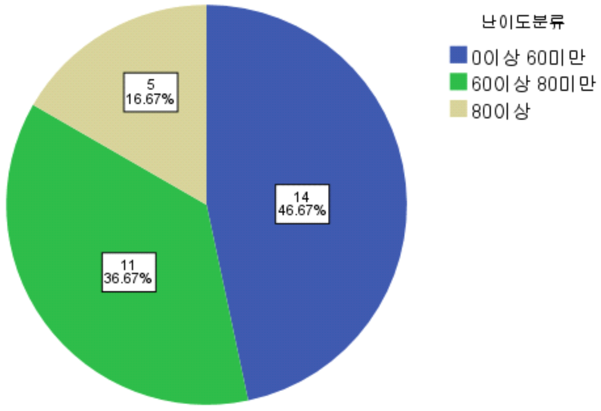

총점	난이도	표준편차
30	59.9	22.3

난이도	문항수	비율(%)
0~60미만	14	46.7
60~80미만	11	36.7
80~100	5	16.6
전체	30	100.0

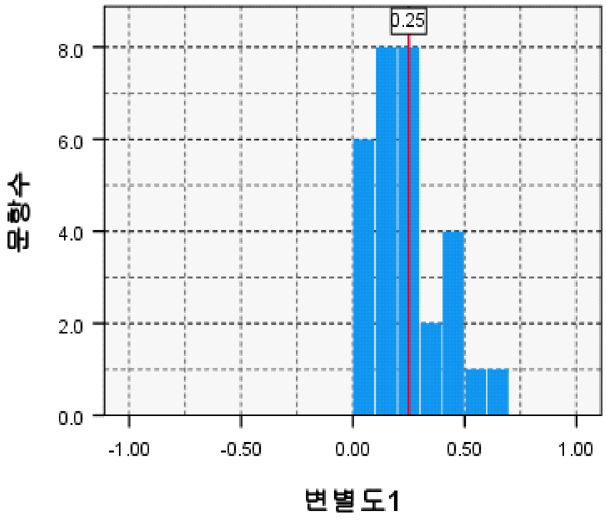

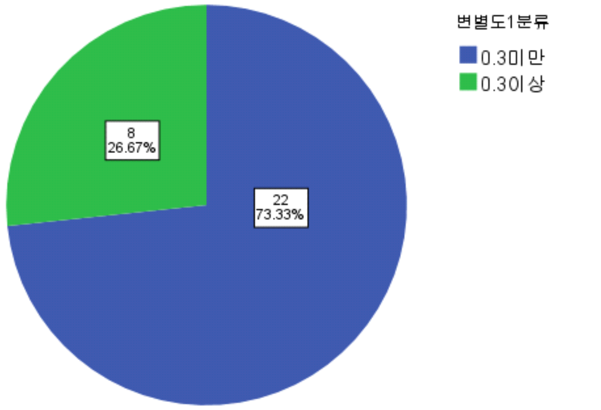

총점	변별도1	표준편차
30	.25	.16

변별도1	문항수	비율(%)
0.3미만	22	73.3
0.3이상	8	26.7
전체	30	100.0

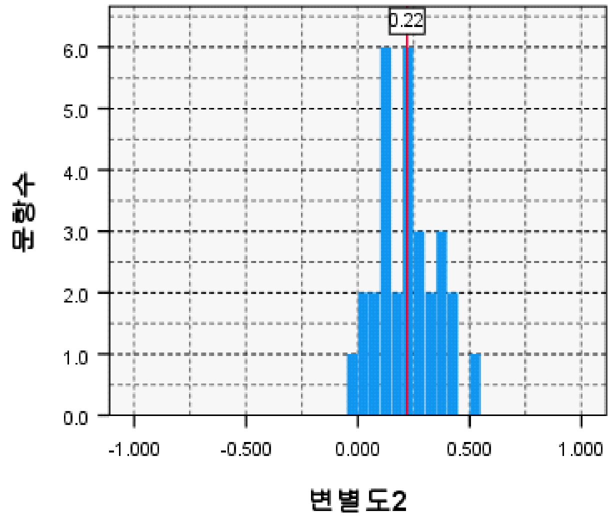

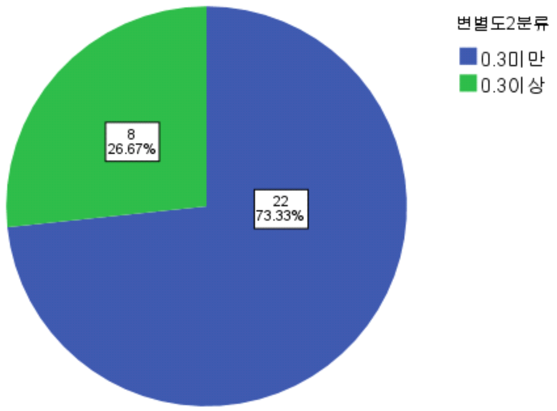

총점	변별도2	표준편차
30	.22	.13

변별도2	문항수	비율(%)
0.3미만	22	73.3
0.3이상	8	26.7
전체	30	100.0

해석

- 자료제시형 문항에서 난이도 지수가 0 에서 60 미만인 문항이 전체 30 문항 중 14 문항으로 가장 많았으며, 다음으로 60 이상 80 미만인 문항이 11 문항, 80 에서 100 사이인 문항이 5 문항인 것으로 나타남
- 변별도 1 지수를 기준으로 분류하였을 때, 0.3 미만인 문항이 22 문항으로 0.3 이상인 문항이 8 문항인 것에 비해 더 많게 나타남
- 변별도 2 지수를 기준으로 분류하였을 때, 0.3 미만인 문항이 22 문항으로 0.3 이상인 문항이 8 문항인 것에 비해 더 많게 나타남

3. 난이도와 변별도 간 산포도

1) 전체 난이도와 변별도 간 산포도

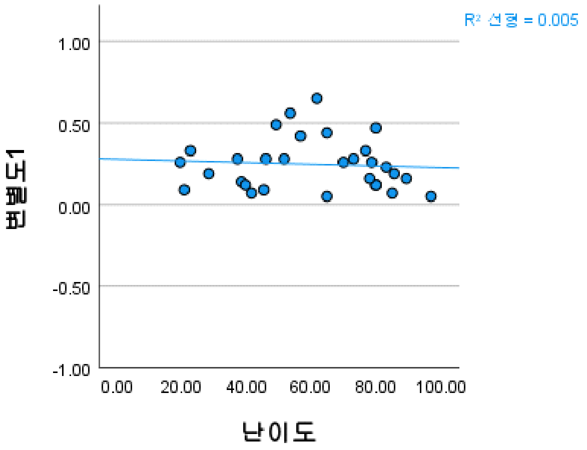

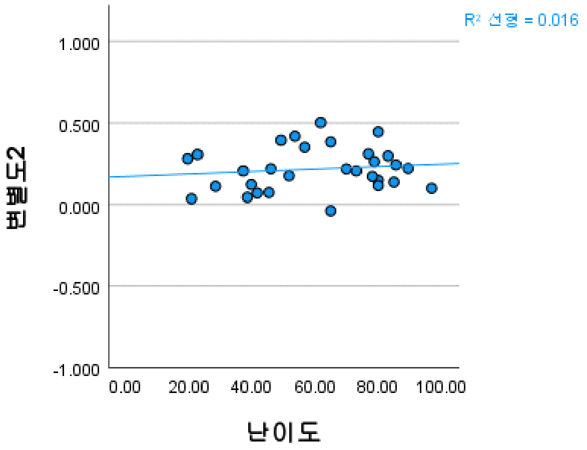

해석

- 전체 문항을 대상으로 난이도와 변별도1 지수 간 상관은 -.072로 문항 난이도와 변별도 간 관련성이 없는 것으로 나타남
- 난이도와 변별도2 지수 간 상관은 0.128로 문항 난이도와 변별도 간 관련성이 낮은 것으로 나타남

2) 과목별 난이도와 변별도 간 산포도

가) 보건의료관계법규 난이도와 변별도 간 산포도

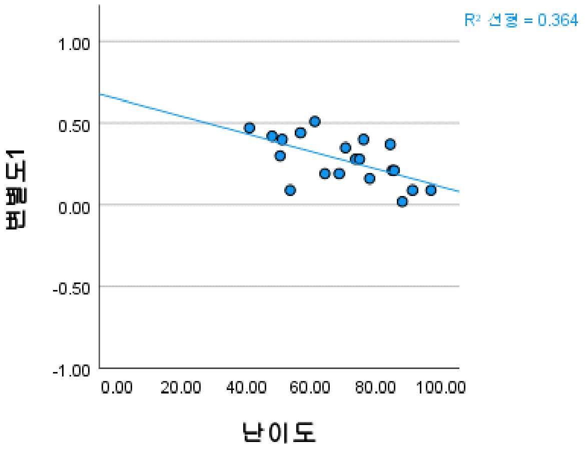

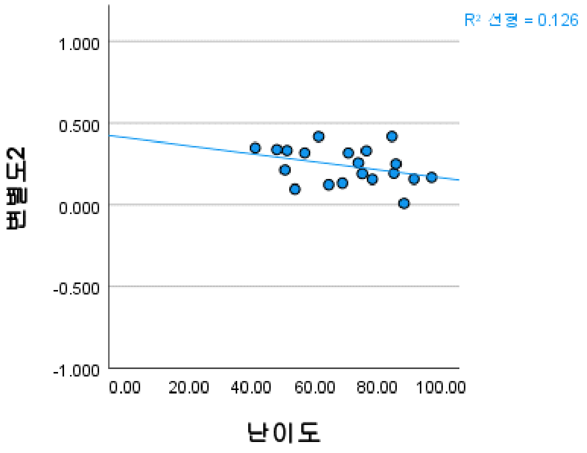

해석

- 보건의료관계법규 과목을 대상으로 난이도와 변별도1 지수 간 상관은 -.604**로 난이도 지수가 높을수록 변별력이 낮아지는 것으로 나타남
- 난이도와 변별도2 지수 간 상관은 -.355로 문항 난이도와 변별도 간 관련성이 낮은 것으로 나타남

나) 운동·생체역학 난이도와 변별도 간 산포도

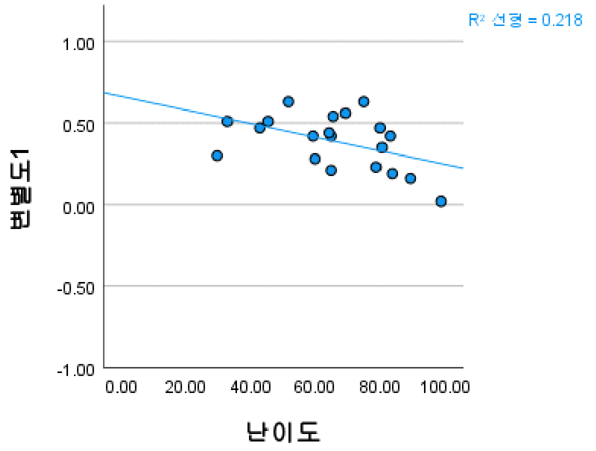

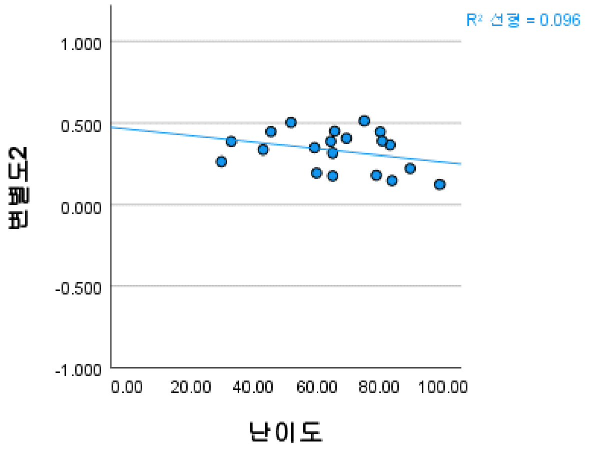

해석

- 운동·생체역학 과목을 대상으로 난이도와 변별도1 지수 간 상관은 -.467*로 난이도 지수가 높을수록 변별력이 낮아지는 것으로 나타남
- 난이도와 변별도2 지수 간 상관은 -.309로 문항 난이도와 변별도 간 관련성이 낮은 것으로 나타남

다) 재활공학·재료학 난이도와 변별도 간 산포도

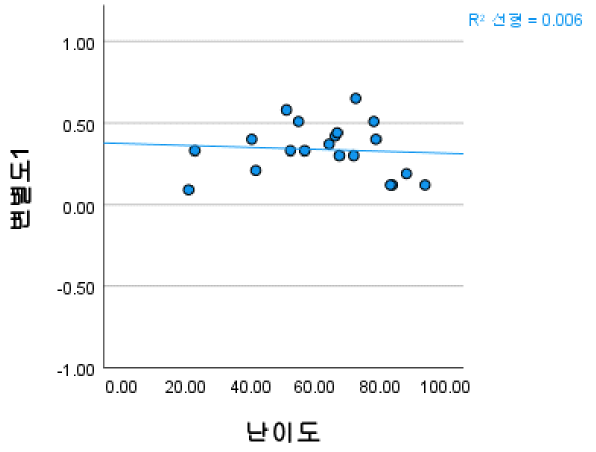

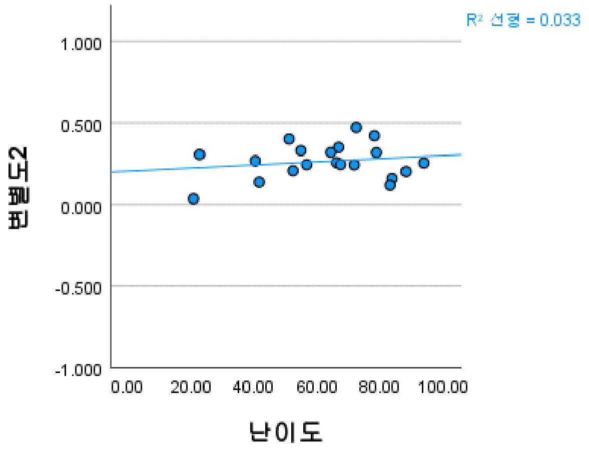

해석

- 재활공학·재료학 과목을 대상으로 난이도와 변별도 1 지수 간 상관은 -.077 으로 문항 난이도와 변별도 간 관련성이 없는 것으로 나타남
- 난이도와 변별도 2 지수 간 상관은 .182 로 문항 난이도와 변별도 간 관련성이 없는 것으로 나타남

라) 보조기학 난이도와 변별도 간 산포도

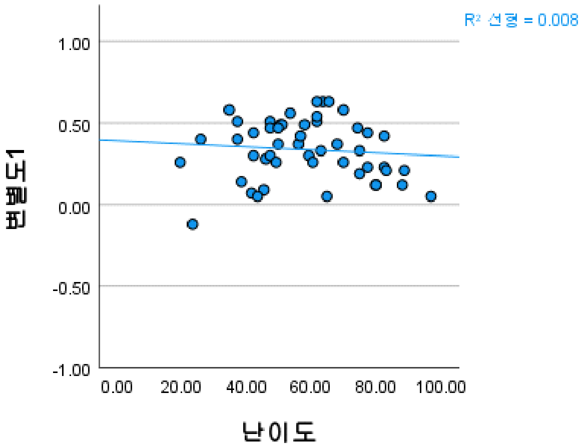

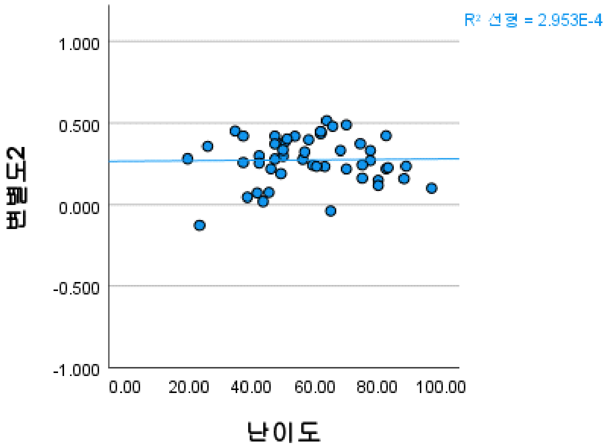

해석

- 보조기학 과목을 대상으로 난이도와 변별도 1 지수 간 상관은 -.092 로 문항 난이도와 변별도 간 관련성이 없는 것으로 나타남
- 난이도와 변별도 2 지수 간 상관은 .017 로 문항 난이도와 변별도 간 관련성이 없는 것으로 나타남

마) 해부·생리학 난이도와 변별도 간 산포도

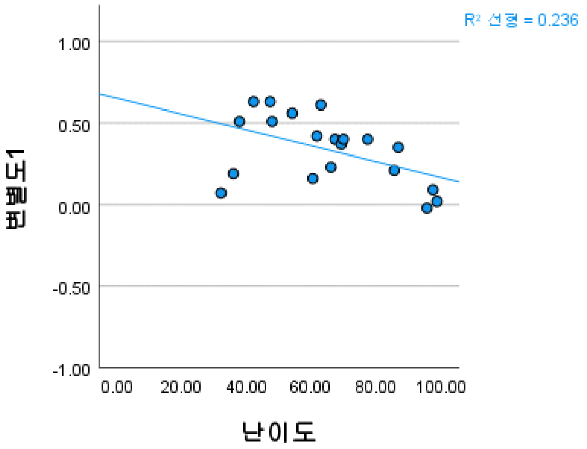

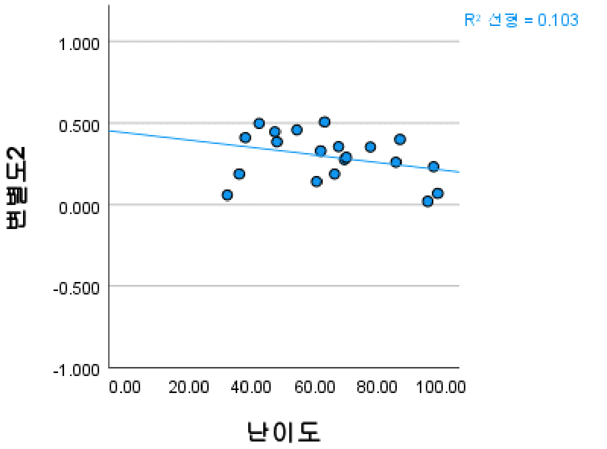

해석

- 해부·생리학 과목을 대상으로 난이도와 변별도 1 지수 간 상관은 -.486*으로 난이도 지수가 높을수록 변별력이 낮아지는 것으로 나타남
- 난이도와 변별도 2 지수 간 상관은 .321 로 문항 난이도와 변별도 간 관련성이 낮은 것으로 나타남

바) 재활의학 난이도와 변별도 간 산포도

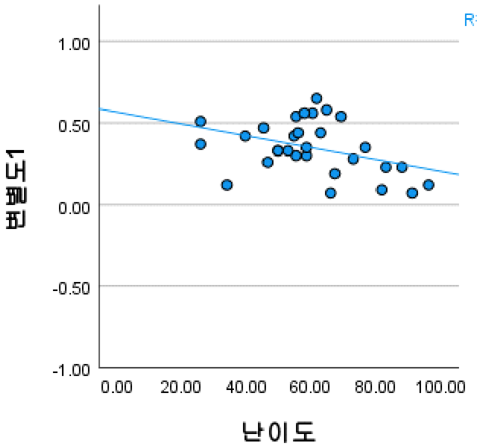

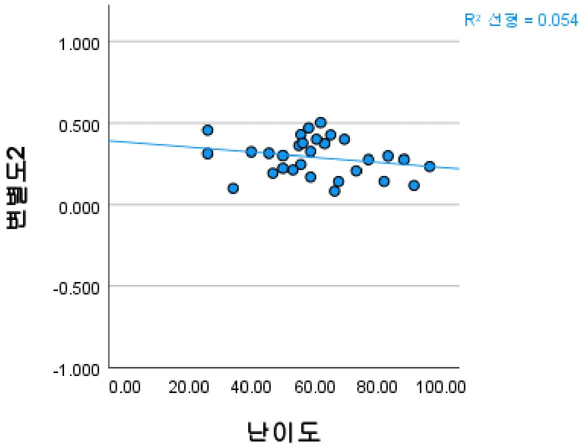

해석

- 재활의학 과목을 대상으로 난이도와 변별도 1 지수 간 상관은 -.389*로 난이도 지수가 높을수록 변별력이 낮아지는 것으로 나타남
- 난이도와 변별도 2 지수 간 상관은 -.232 로 문항 난이도와 변별도 간 관련성이 낮은 것으로 나타남

사) 의지학 난이도와 변별도 간 산포도

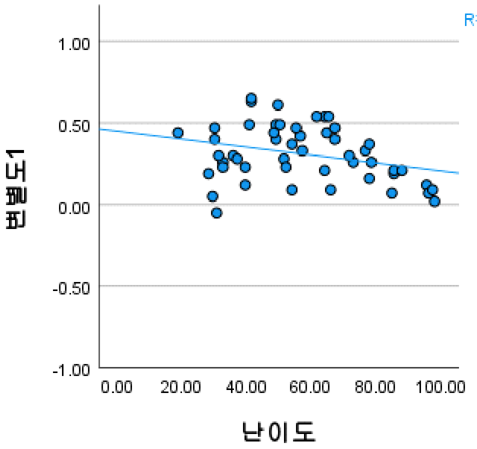

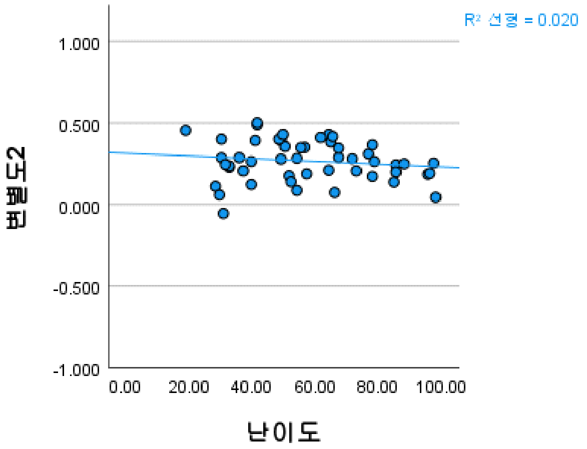

해석

- 의지학 과목을 대상으로 난이도와 변별도1 지수 간 상관은 -.298로 문항 난이도와 변별도 간 관련성이 낮은 것으로 나타남
- 난이도와 변별도2 지수 간 상관은 -.143로 문항 난이도와 변별도 간 관련성이 없는 것으로 나타남

4. 신뢰도 분석

과목명	문항수	제19회	제20회	제21회	제22회	제23회
전체	210	.953	.963	.961	.958	**.950**
보건의료 관계 법규	20	.666	.603	.675	.638	**.644**
운동·생체역학	20	.650	.763	.736	.769	**.757**
재활공학·재료학	20	.626	.640	.691	.602	**.661**
보조기학	50	.861	.864	.872	.874	**.833**
해부·생리학	20	.713	.721	.768	.712	**.715**
재활의학	30	.724	.769	.730	.702	**.768**
의지학	50	.860	.893	.874	.873	**.818**

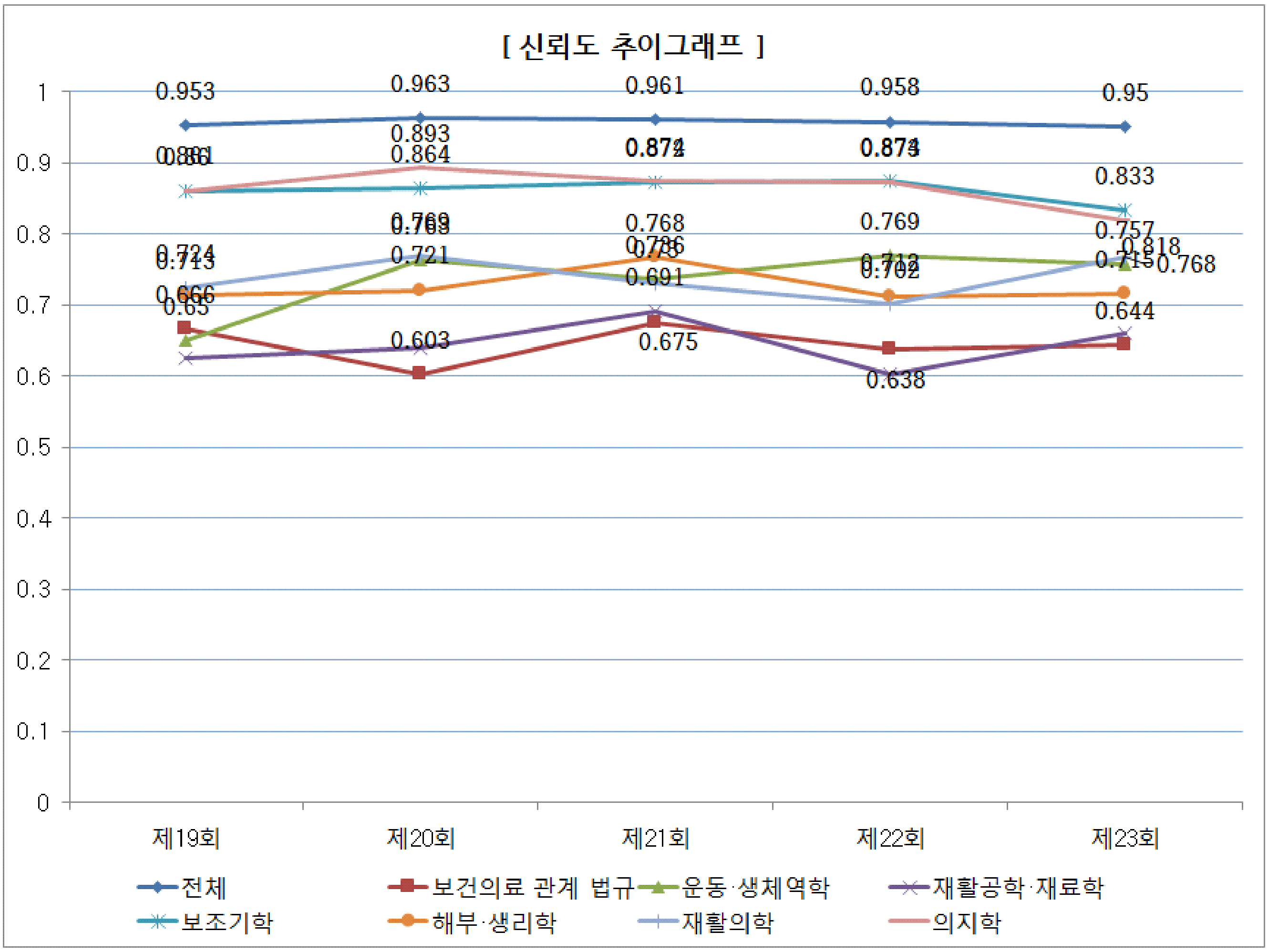

해석

- 전회 대비 신뢰도는 전체문항과 운동·생체역학, 보조기학, 의지학 과목의 경우 각각 .008, .012, .041, .0551 감소함
- 보건이료관계법규, 재활공학·재료학, 해부·생리학, 재활의학 과목의 경우 전년대비 각각 .006, .059, .003, .066 증가함

○ 분석결과 관련 문의 : 한국보건의료인국가시험원 연구개발본부 배상영 책임연구원
Tel : 02-2087-8955, FAX : 02-2087-8885
E-mail : bsy0601@kuksiwon.or.kr
