# Supplementary material for: Item difficulty index, discrimination index, and reliability of the 26 health professions licensing examinations in 2022, Korea: a psychometric study
Source: J Educ Eval Health Prof. 2023 Nov 22;20:31. doi: 10.3352/jeehp.2023.20.31 (PMC11959405; doi:10.3352/jeehp.2023.20.31)
Supplement: Supplementary file 1 — Supplement 1. Item analysis results of 26 health professions licensing examinations administered during late 2022 and early 2023. [file jeehp-20-31_Suppl1.zip › 2022│Γ╡╡ ┴a23╚╕ ╟╤╛α╗τ ▒╣░í╜├╟Φ ║╨╝«░ß░·.pdf]

2022년도 제23회
한약사 국가시험
문항분석 결과

국민이 신뢰하고 감동하는 시험평가기관
한국보건의료인국가시험원
KOREA HEALTH PERSONNEL LICENSING EXAMINATION INSTITUTE

일반 용어 정의

□ **평균**

- 집단에서의 대표적 경향값으로 전체 값을 더하여 총 응시자로 나눈 값

□ **표준편차**

- 평균과 각 점수의 차이인 편차들의 평균으로 점수가 흩어져 분포되어 있는 정도

□ **추정난이도**

- 문항개발자가 예측한 정답률

□ **검사이론**

- 검사와 검사를 구성하고 있는 문항의 양호도를 분석 및 평가하는 방법을 정의한 이론체계
- 대표적으로 고전검사이론과 문항반응이론이 있음

고전검사이론 용어 정의

□ 고전검사이론(Classical Test Theory; CTT)

- 검사의 질을 분석하는 검사이론 중 한 가지로 19세기 말부터 전개되어 현재까지 주로 사용되고 있는 검사이론임
- 고전검사이론에 의한 문항과 응시자 능력 추정치는 다음과 같음

○ **문항난이도**

· 검사 문항의 쉽고 어려운 정도를 나타내는 지수

· 난이도 지수는 총 반응 수에 대한 정답 반응 수의 비율로 문항의 정답률임

· 문항난이도는 0~100까지의 값을 가짐

· 난이도 값이 큰 경우, 쉬운 문항으로 '난이도가 낮다'라고 해석하며, 난이도 값이 작은 경우, 어려운 문항으로 '난이도가 높다'라고 해석함

○ **문항변별도**

· 각 문항이 응시자의 능력 수준을 변별할 수 있는 정도를 나타내는 지수

· 문항변별도는 -1~+1까지의 값을 가지며, 1에 가까울수록 변별력 크다고 해석함

· 일반적으로 문항변별도가 0.3 이상이면 우수한 문항으로 평가함

· 구하는 방식에는 '상하위집단 구분법', '문항-총점 상관계수' 등이 있음

1) **변별도 1(상하위구분법):** 상위 27%와 하위 27% 집단의 난이도 차이를 구하는 방식

2) **변별도 2(상관계수법):** 문항-총점과의 상관계수로 구하는 방식

○ **신뢰도**

· 시험이 평가하고자 하는 것을 일관성 있게 측정하는가로 시험이 오차없이 정확하게 측정한 정도를 의미함

· 국시원에서는 문항의 내적일관성(Cronbach α)으로 신뢰도를 추정하며 1에 가까울수록 신뢰도가 높다고 해석함

목 차

I. 시행 결과 ······ 6

1. 시험 현황 ······ 7

1) 시험명 ······ 7

2) 시험시행일 ······ 7

3) 응시현황 ······ 7

4) 과목별 문항 수, 배점 및 과락 점수 ······ 7

2. 합격률과 평균성적 ······ 7

1) 합격 및 불합격 현황 ······ 7

2) 과목별 과락자수 내역 ······ 7

3) 전회 대비 합격률과 평균성적 ······ 8

II. 문항분석 결과 ······ 9

1. 성적 ······ 10

1) 전체 성적분포도 ······ 10

2) 과목별 성적분포도 ······ 11

2. 난이도와 변별도 ······ 12

1) 전체 난이도와 변별도 ······ 12

2) 과목별 난이도와 변별도 ······ 15

3) 지식수준별 난이도와 변별도 ······ 25

3. 난이도와 변별도 간 산포도 ······ 33

1) 전체 난이도와 변별도 간 산포도 ······ 33

2) 과목별 난이도와 변별도 간 산포도 ······ 33

4. 신뢰도 분석 ······ 36

I. 시행 결과

1. 시험 현황

1) 시험명: 2022년도 제23회 한약사 국가시험

2) 시험시행일: 2022년 1월 19일

3) 응시현황

응시대상자수	결시자수	부정행위자수	응시자 준수사항 위반자 수		응시자수 (%)
			휴대폰 소지	신분증 미지참	
148	3	0	0	0	※144 (97.3)

※채점보류자수(1명) 제외

4) 과목별 문항 수, 배점 및 과락 점수

교 시	과 목 명	문제 수	배점	총점	합격자 점수기준	
					과목 과락기준	총점 합격기준
1교시	한약학 기초	110	1	110	44점 미만	150점 이상
1교시	보건•의약 관계 법규	30	1	30	12점 미만	
2교시	한약학 응용	110	1	110	44점 미만	
계		250		250		

2. 합격률과 평균성적

1) 합격 및 불합격 현황

합격자수 (%)	불합격자수(%)				채점보류자수
	평락	과락	기권	계	
119 (82.6)	24 (16.7)	0 (0.0)	1 (0.7)	25 (17.4)	1

2) 과목별 과락자수 내역

과목명 / 과락자수	과목1	과목2	과목3	과목4
과목별 과락자 수	0	0	0	0
전과목 과락자 수	0			

3) 전회 대비 합격률과 평균성적

회차	년도	합격률(%)	평균성적	표준편차	백분율 환산점수
제19회	2018	90.9	191.6	30.1	76.6
제20회	2019	81.1	172.9	31.9	69.2
제21회	2020	86.3	182.2	30.8	72.9
제22회	2021	84.6	174.6	28.8	69.8
제23회	2022	82.6	173.7	33.4	69.5

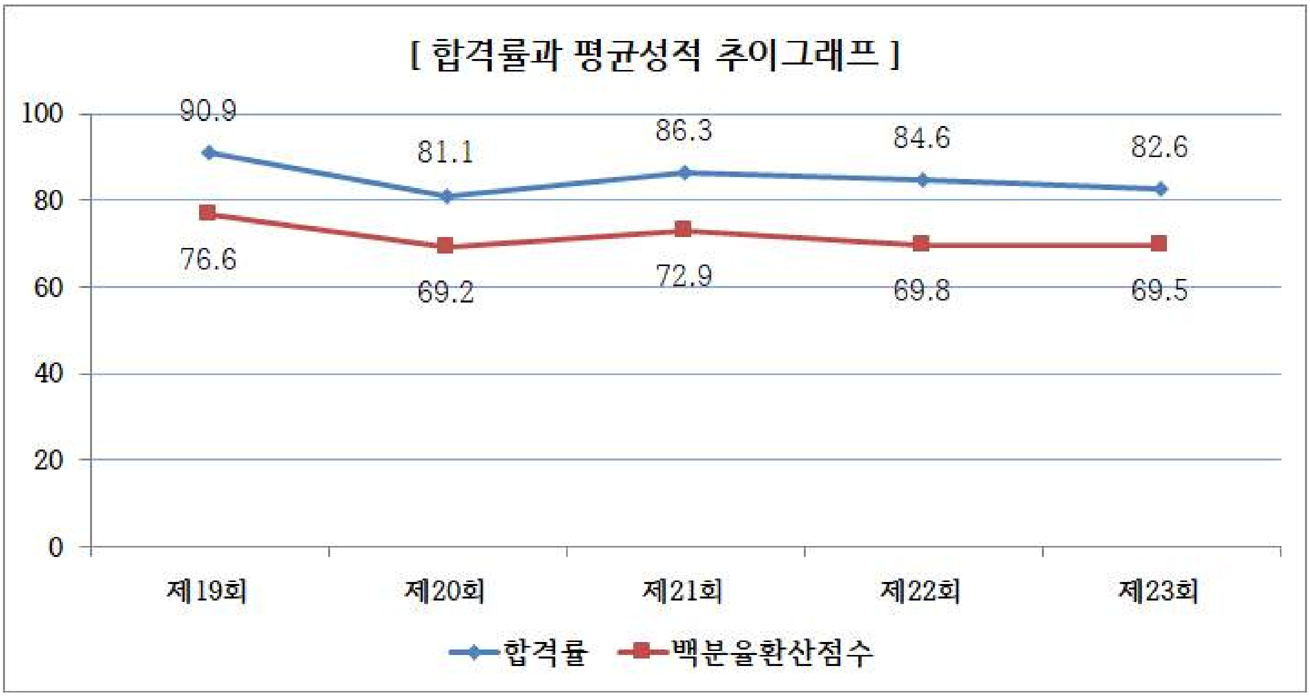

해석

- 전년 대비 합격률은 2.0% 감소하고, 백분율 환산점수는 0.3 감소함

Ⅱ. 문항분석 결과

1. 성적

1) 전체 성적분포도

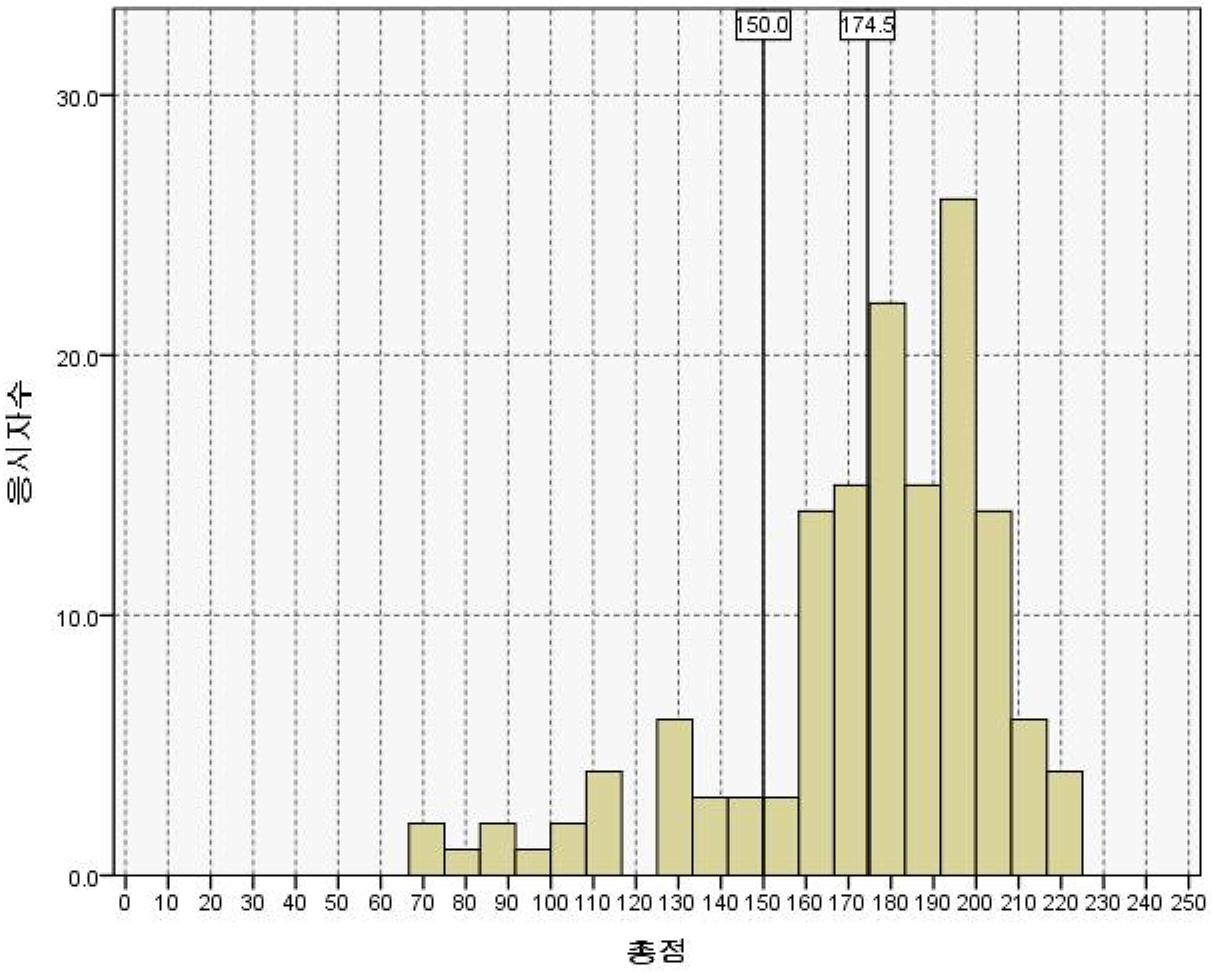

응시자	총점	합격선	평균성적	표준편차
※143	250	150	174.5	31.8

※응시자준수사항 위반자(1명) 및 기권자(1명) 제외

2) 과목별 성적분포도

가) 한약학기초

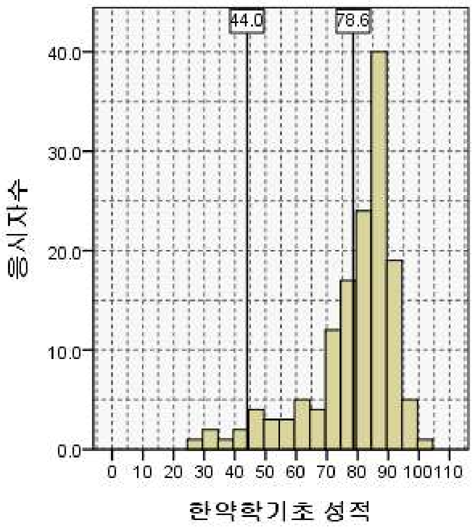

총점	과락선	평균성적	표준편차
110	44	78.6	14.7

나) 보건의약관계법규

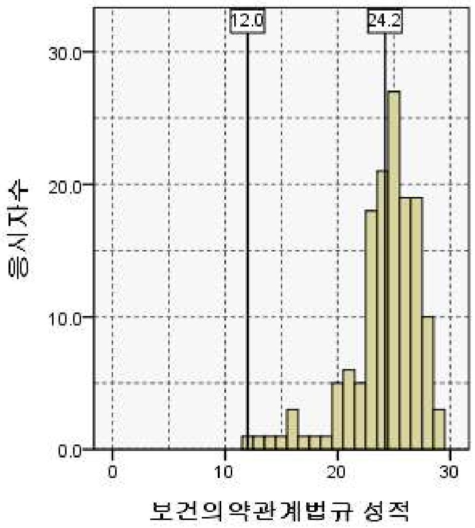

총점	과락선	평균성적	표준편차
30	12	24.2	3.2

다) 한약학응용

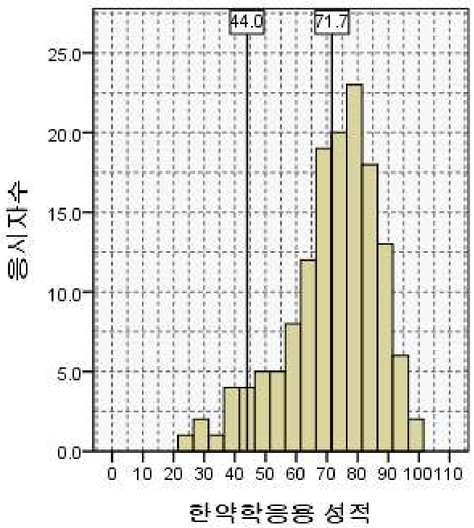

총점	과락선	평균성적	표준편차
110	44	71.7	15.4

2. 난이도와 변별도

1) 전체 난이도와 변별도

가) 전회 대비 전체 난이도와 변별도

회차	난이도		변별도1		변별도2	
	평균	표준편차	평균	표준편차	평균	표준편차
제19회	76.7	19.0	.27	.14	.33	.15
제20회	69.2	21.1	.31	.17	.31	.16
제21회	72.9	20.7	.29	.17	.31	.15
제22회	69.8	22.5	.27	.27	.29	.16
제23회	69.8	21.9	.29	.17	.32	.18

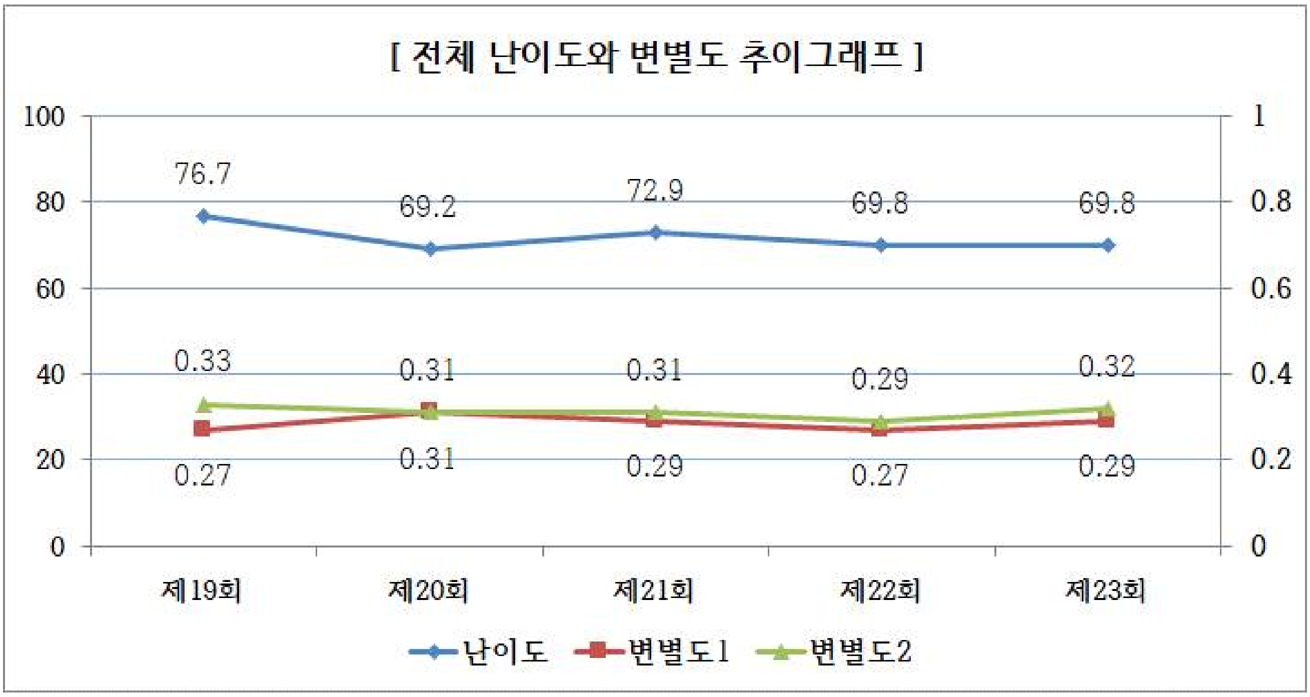

해석

- 전년 대비 난이도 지수는 변하지 않음
- 변별도 1 지수는 .02 증가함
- 변별도 2 지수는 .03 증가함

나) 전체 난이도와 변별도 분포도 및 비율분석

(1) 전체 난이도 분포도 및 비율분석

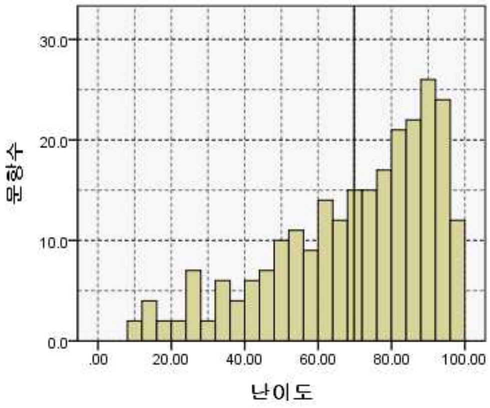

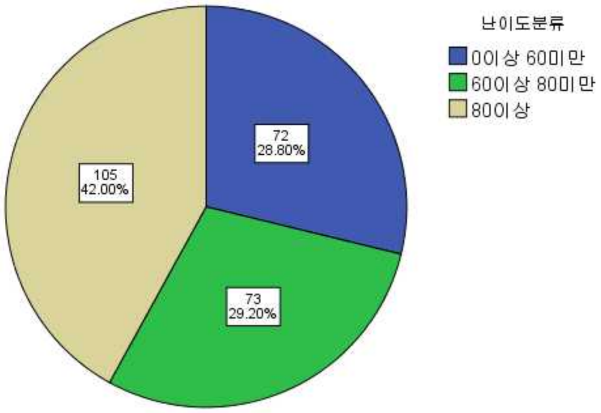

총점	난이도	표준편차
250	69.8	21.9

난이도	문항수	비율(%)
0~60미만	72	28.8
60~80미만	73	29.2
80~100	105	42.0
전체	250	100.0

(2) 전체 변별도1 분포도 및 비율분석

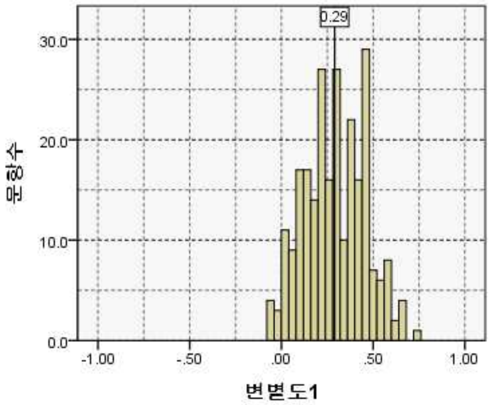

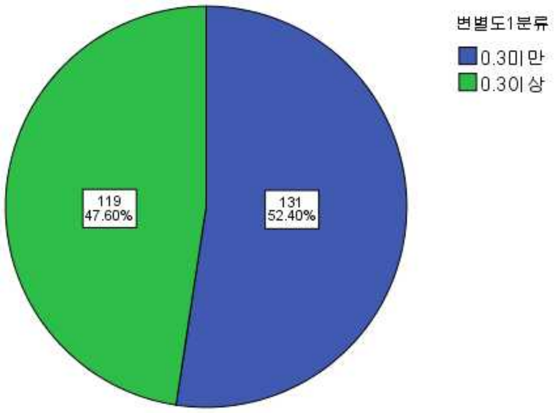

총점	변별도1	표준편차
250	.29	.17

변별도1	문항수	비율(%)
0.3미만	131	52.4
0.3이상	119	47.6
전체	250	100.0

(3) 전체 변별도2 분포도 및 비율분석

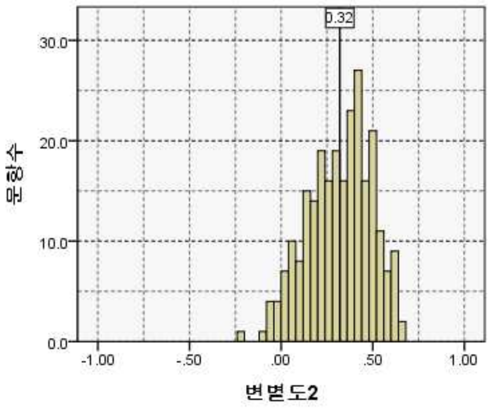

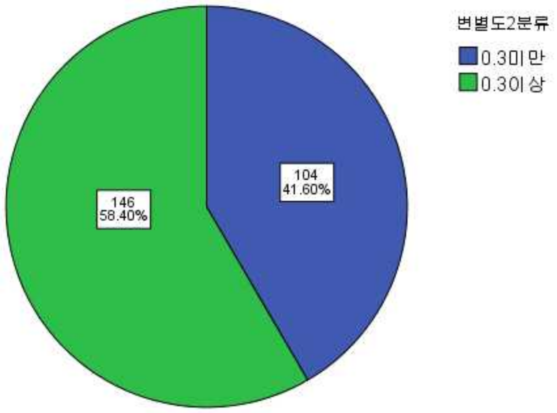

총점	변별도2	표준편차
250	.32	.18

변별도2	문항수	비율(%)
0.3미만	104	41.6
0.3이상	146	58.4
전체	250	100.0

해석

- 난이도 지수가 80 에서 100 사이인 문항이 전체 250 문항 중 105 문항이었으며, 60 이상 80 미만인 문항이 73 문항, 60 미만인 문항이 72 문항인 것으로 나타남
- 변별도 1 지수를 기준으로 분류하였을 때, 0.3 미만인 문항이 131 문항으로 0.3 이상인 문항이 119 문항인 것에 비해 더 많이 나타남
- 변별도 2 지수를 기준으로 분류하였을 때, 0.3 미만인 문항이 104 문항으로 0.3 이상인 문항이 146 문항인 것에 비해 더 적게 나타남

2) 과목별 난이도와 변별도

가) 전회 대비 과목별 난이도와 변별도

(1) 전회 대비 한약학 기초 난이도와 변별도

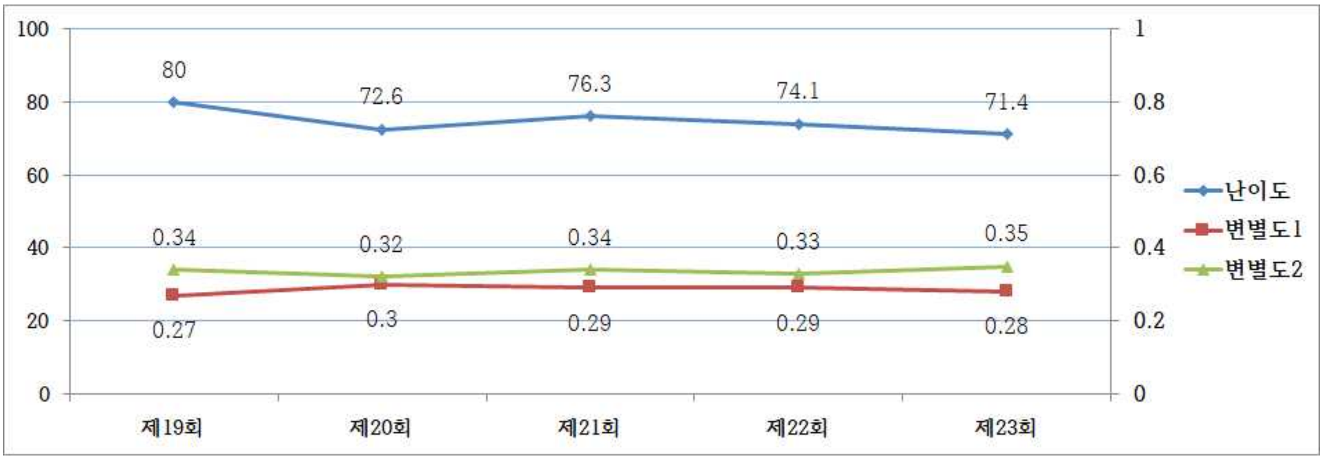

회차	난이도		변별도1		변별도2	
	평균	표준편차	평균	표준편차	평균	표준편차
제19회	80.0	17.5	.27	.14	.34	.15
제20회	72.6	21.4	.30	.17	.32	.16
제21회	76.3	10.7	.29	.15	.34	.13
제22회	74.1	19.3	.29	.14	.33	.14
제23회	71.4	23.4	.28	.16	.35	.18

해석

- 전회 대비 한약학 기초 과목의 난이도 지수는 2.7 감소함
- 전회 대비 한약학 기초 과목의 변별도 1 지수는 .01 감소함
- 전회 대비 한약학 기초 과목의 변별도 2 지수는 .02 증가함

(2) 전회 대비 보건·의약관계법규 난이도와 변별도

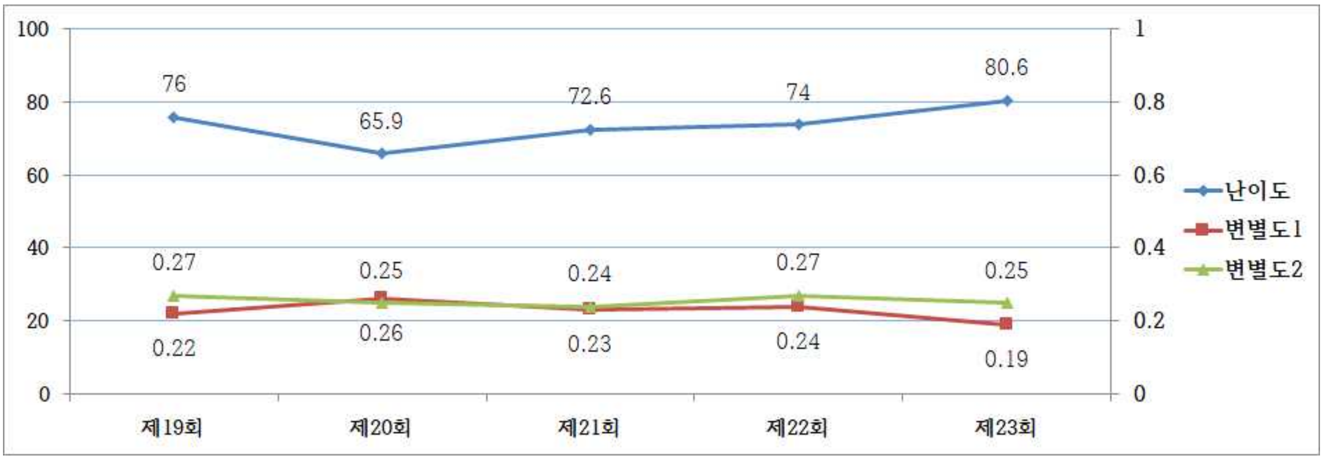

회차	난이도		변별도1		변별도2	
	평균	표준편차	평균	표준편차	평균	표준편차
제19회	76.0	19.7	.22	.12	.27	.12
제20회	65.9	21.0	.26	.18	.25	.16
제21회	72.6	19.9	.23	.16	.24	.15
제22회	74.0	21.5	.24	.17	.27	.16
제23회	80.6	19.7	.19	.15	.25	.18

해석

- 전회 대비 보건·의약관계법규 과목의 난이도 지수는 6.6 증가함
- 전회 대비 보건·의약관계법규 과목의 변별도 1 지수는 .05 감소함
- 전회 대비 보건·의약관계법규 과목의 변별도 2 지수는 .02 감소함

(3) 전회 대비 한약학 응용 난이도와 변별도

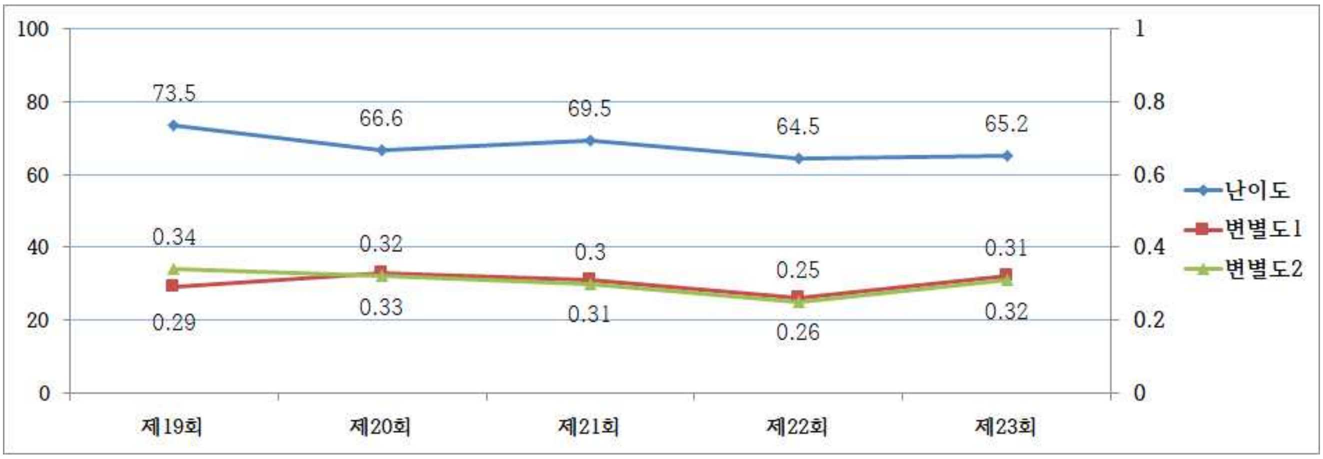

회차	난이도		변별도1		변별도2	
	평균	표준편차	평균	표준편차	평균	표준편차
제19회	73.5	19.9	.29	.15	.34	.15
제20회	66.6	20.5	.33	.17	.32	.15
제21회	69.5	20.5	.31	.18	.30	.16
제22회	64.5	24.6	.26	.19	.25	.17
제23회	65.2	19.7	.32	.17	.31	.16

해석

- 전회 대비 한약학 응용 과목의 난이도 지수는 0.7 증가함
- 전회 대비 한약학 응용 과목의 변별도 1 지수는 .06 증가함
- 전회 대비 한약학 응용 과목의 변별도 2 지수는 .06 증가함

나) 과목별 난이도와 변별도 분포도 및 비율분석

(1) 한약학 기초 난이도와 변별도 분포도 및 비율분석

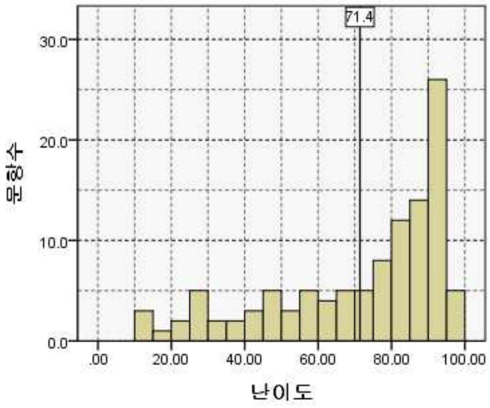

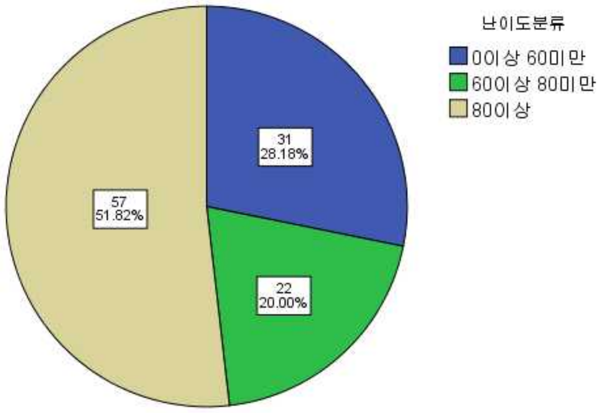

총점	난이도	표준편차
110	71.4	23.4

난이도	문항수	비율(%)
0~60미만	31	28.2
60~80미만	22	20.0
80~100	57	51.8
전체	110	100.0

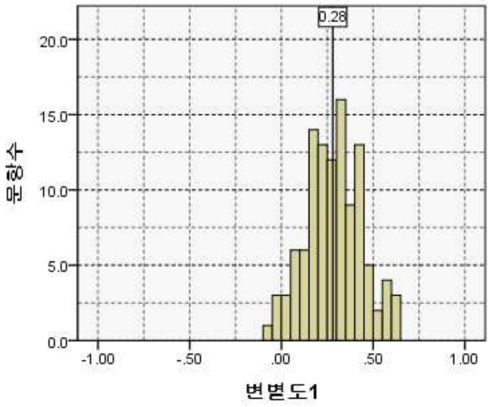

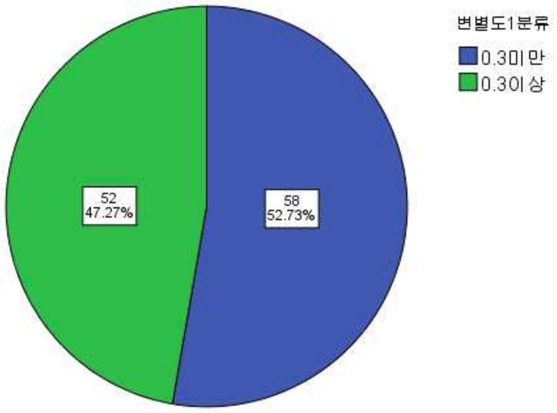

총점	변별도1	표준편차
110	.28	.16

변별도1	문항수	비율(%)
0.3미만	58	52.7
0.3이상	52	47.3
전체	110	100.0

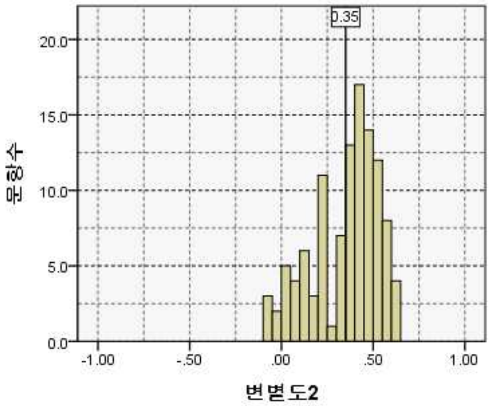

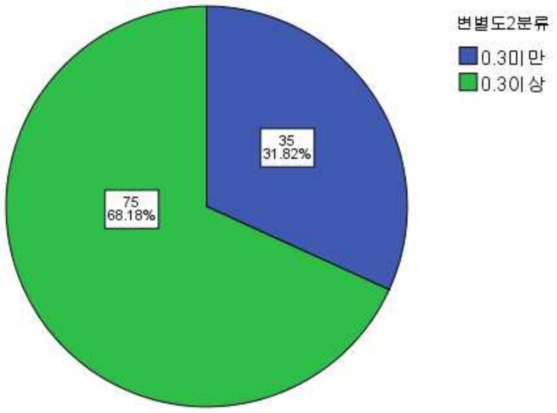

총점	변별도2	표준편차
110	.35	.18

변별도2	문항수	비율(%)
0.3미만	35	31.8
0.3이상	75	68.2
전체	110	100.0

해석

- 한약학 기초 과목에서 난이도 지수가 80 에서 100 사이인 문항이 전체 110 문항 중 57 문항이었으며, 60 이상 80 미만인 문항이 22 문항, 60 미만인 문항이 31 문항으로 나타남
- 변별도 1 지수를 기준으로 분류하였을 때, 0.3 미만인 문항이 58 문항으로 0.3 이상인 문항이 52 문항인 것에 비해 더 많이 나타남
- 변별도 2 지수를 기준으로 분류하였을 때, 0.3 미만인 문항이 35 문항으로 0.3 이상인 문항이 75 문항인 것에 비해 더 적게 나타남

(2) 보건·의약관계법규 난이도와 변별도 분포도 및 비율분석

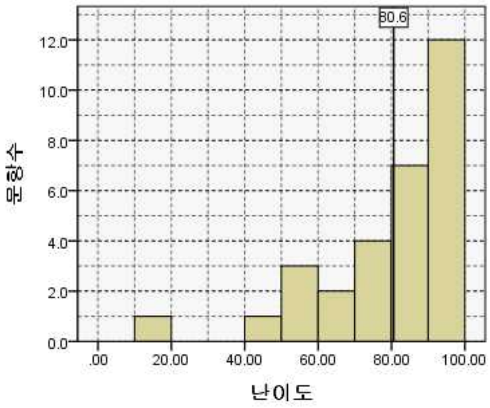

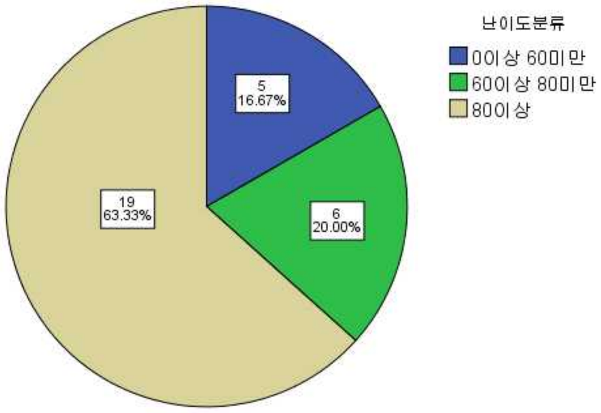

총점	난이도	표준편차
30	80.6	19.7

난이도	문항수	비율(%)
0~60미만	5	16.7
60~80미만	6	20.0
80~100	19	63.3
전체	30	100.0

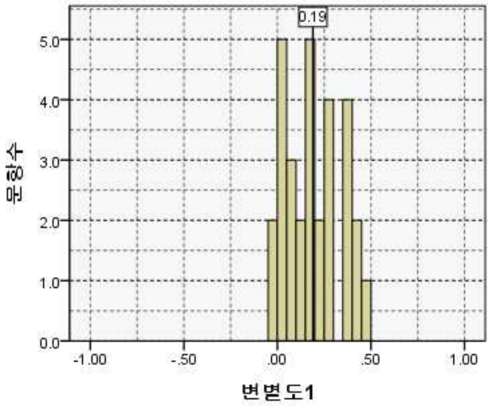

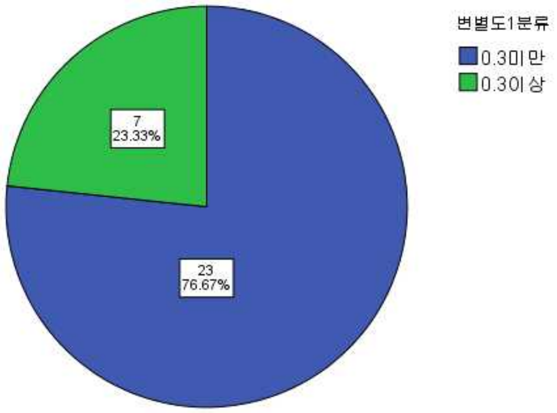

총점	변별도1	표준편차
30	.19	.15

변별도1	문항수	비율(%)
0.3미만	23	76.7
0.3이상	7	23.3
전체	30	100.0

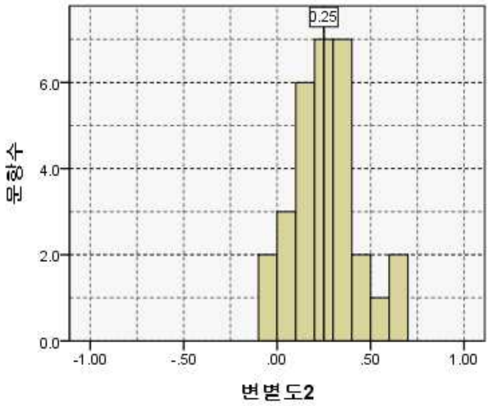

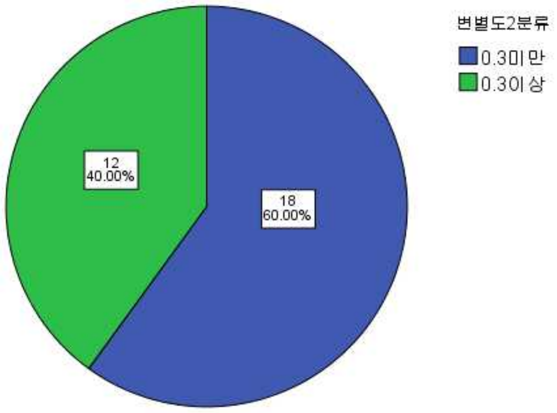

총점	변별도2	표준편차
30	.25	.18

변별도2	문항수	비율(%)
0.3미만	18	60.0
0.3이상	12	40.0
전체	30	100.0

해석

- 보건·의약관계법규 과목에서 난이도 지수가 80 에서 100 사이인 문항이 전체 30 문항 중 19 문항이었으며, 60 이상 80 미만인 문항이 6 문항, 60 미만인 문항이 5 문항으로 나타남
- 변별도 1 지수를 기준으로 분류하였을 때, 0.3 미만인 문항이 23 문항으로 0.3 이상인 문항이 7 문항인 것에 비해 더 많이 나타남
- 변별도 2 지수를 기준으로 분류하였을 때, 0.3 미만인 문항이 18 문항으로 0.3 이상인 문항이 12 문항인 것과 비해 더 많이 나타남

(3) 한약학 응용 난이도와 변별도 분포도 및 비율분석

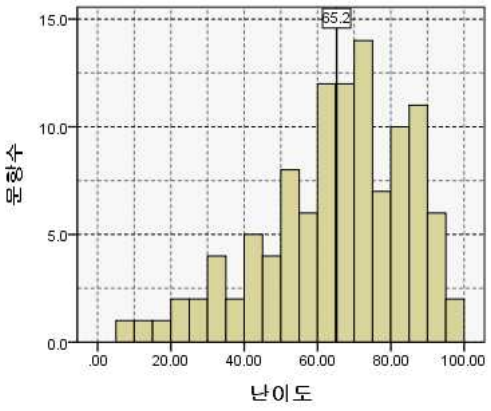

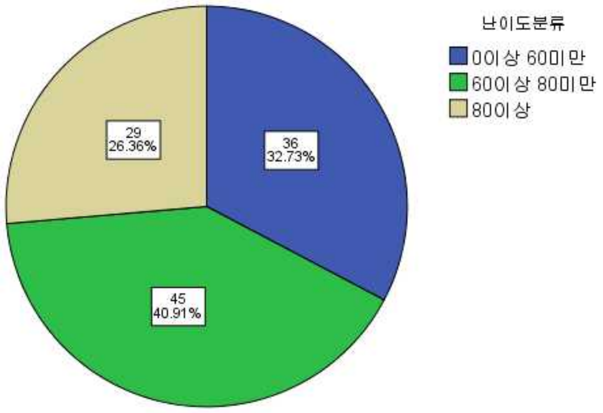

총점	난이도	표준편차
110	65.2	19.7

난이도	문항수	비율(%)
0~60미만	36	32.7
60~80미만	45	40.9
80~100	29	26.4
전체	110	100.0

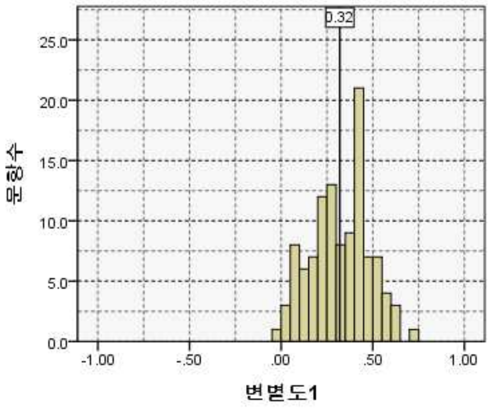

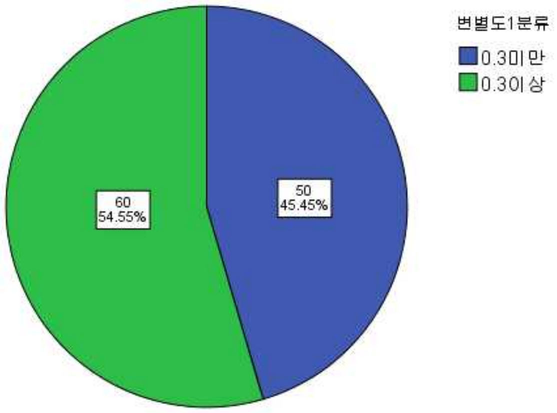

총점	변별도1	표준편차
110	.32	.17

변별도1	문항수	비율(%)
0.3미만	50	45.5
0.3이상	60	54.5
전체	110	100.0

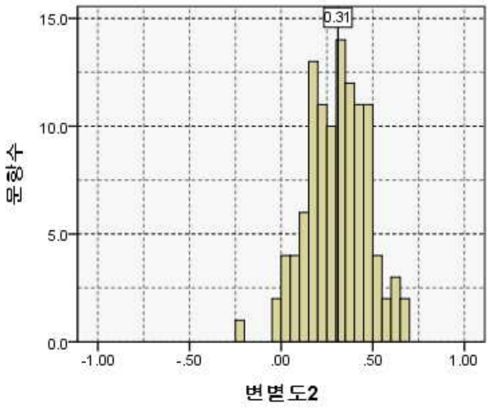

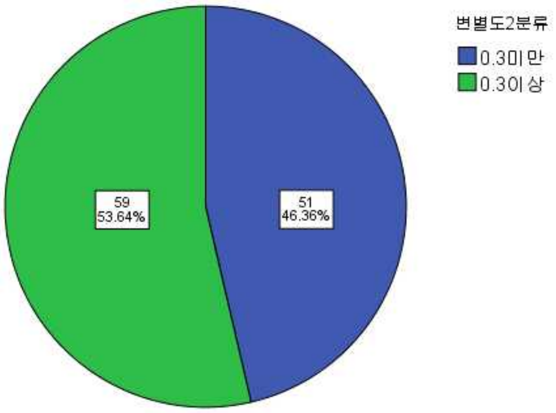

총점	변별도2	표준편차
110	.31	.16

변별도2	문항수	비율(%)
0.3미만	51	46.4
0.3이상	59	53.6
전체	110	100.0

해석

- 한약학 응용 과목에서 난이도 지수가 80에서 100 사이인 문항이 전체 110문항 중 29문항이었으며, 60이상 80미만인 문항이 45문항, 60 미만인 문항이 36문항으로 나타남
- 변별도1 지수를 기준으로 분류하였을 때, 0.3미만인 문항이 50문항으로 0.3이상인 문항이 60문항인 것에 비해 더 적게 나타남
- 변별도2 지수를 기준으로 분류하였을 때, 0.3미만인 문항이 51문항으로 0.3이상인 문항이 59문항인 것에 비해 더 적게 나타남

3) 지식수준별 난이도와 변별도

가) 전회 대비 지식수준별 난이도와 변별도

(1) 전회 대비 암기형 난이도와 변별도

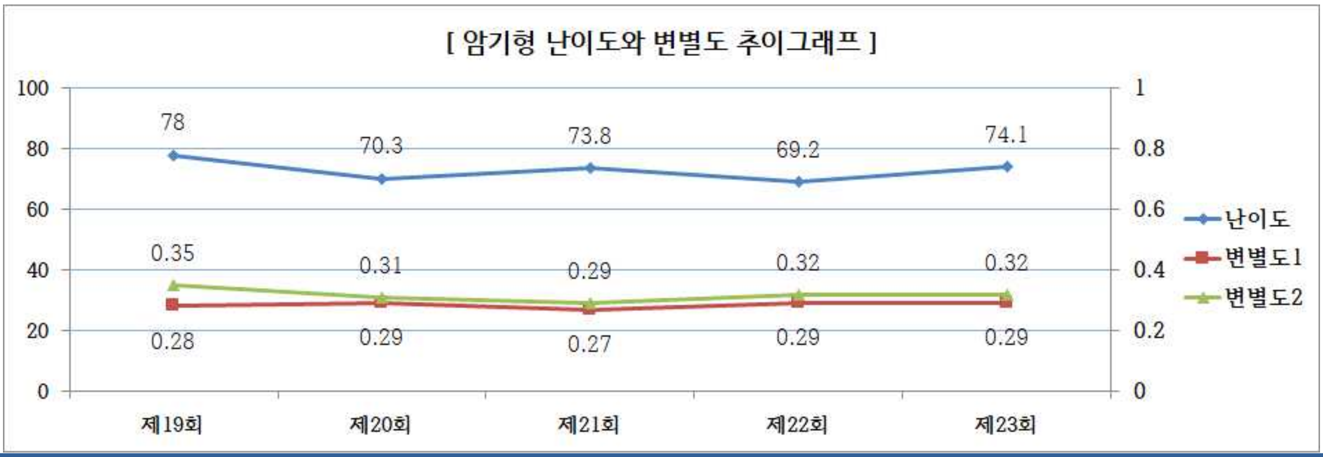

회차	난이도		변별도1		변별도2	
	평균	표준편차	평균	표준편차	평균	표준편차
제19회	78.0	19.0	.28	.15	.35	.14
제20회	70.3	21.9	.29	.17	.31	.16
제21회	73.8	20.5	.27	.15	.29	.14
제22회	69.2	25.0	.29	.16	.32	.15
제23회	74.1	19.9	.29	.18	.32	.17

(2) 전회 대비 해석형 난이도와 변별도

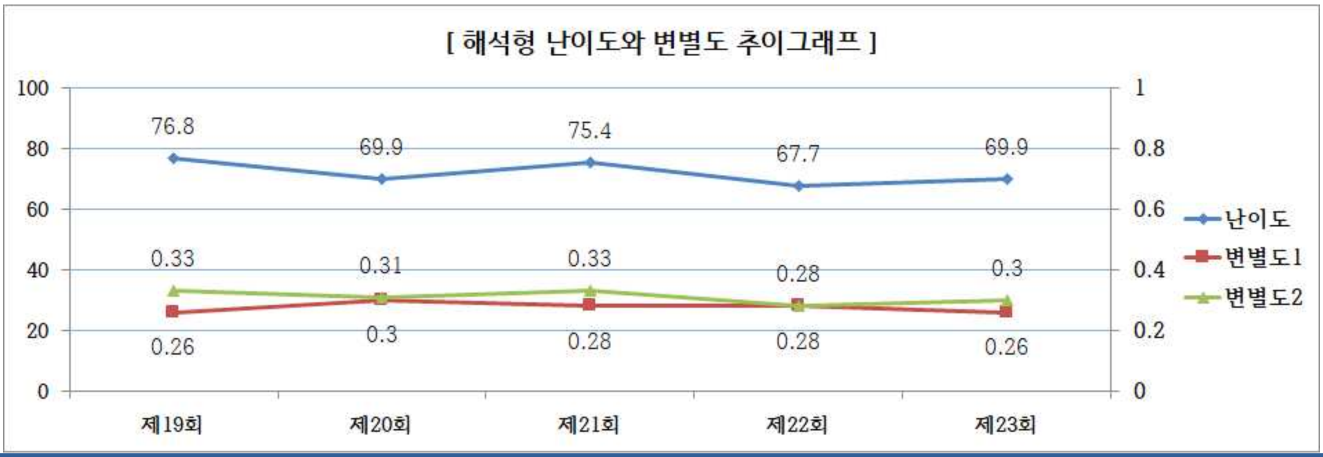

회차	난이도		변별도1		변별도2	
	평균	표준편차	평균	표준편차	평균	표준편차
제19회	76.8	20.1	.26	.14	.33	.15
제20회	69.9	20.5	.30	.18	.31	.17
제21회	75.4	20.5	.28	.16	.33	.15
제22회	67.7	23.2	.28	.18	.28	.17
제23회	69.9	22.8	.26	.16	.30	.17

(3) 전회 대비 해결형 난이도와 변별도

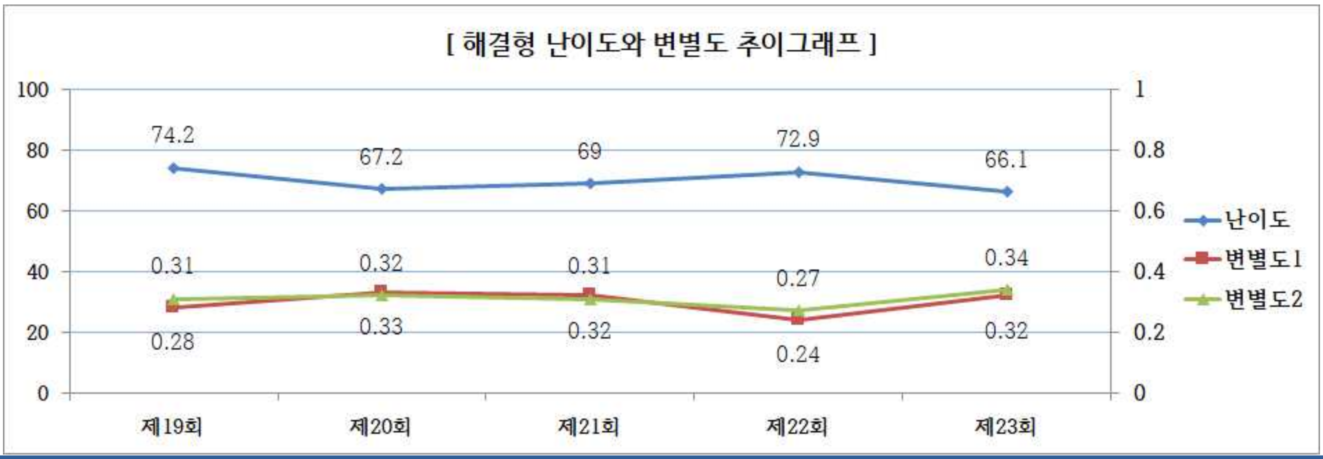

회차	난이도		변별도1		변별도2	
	평균	표준편차	평균	표준편차	평균	표준편차
제19회	74.2	16.6	.28	.14	.31	.16
제20회	67.2	21.2	.33	.17	.32	.14
제21회	69.0	20.9	.32	.18	.31	.16
제22회	72.9	19.1	.24	.16	.27	.16
제23회	66.1	21.7	.32	.16	.34	.19

해석

- 전회 대비 암기형 문항의 난이도 지수는 4.9 증가하였고, 해석형 문항의 난이도 지수는 2.2 증가하였으며, 해결형 문항의 난이도 지수는 6.8 감소함
- 변별도 1 지수는 암기형 문항에서 변하지 않았으며 해석형 문항에서는 .02 감소하였고, 해결형 문항에서는 .08 증가함
- 변별도 2 지수는 암기형 문항에서 변하지 않았고, 해석형 문항에서는 .02 증가하였고, 해결형 문항에서는 .07 증가함

나) 지식수준별 난이도와 변별도 분포도 및 비율분석

(1) 암기형 난이도와 변별도 분포도 및 비율분석

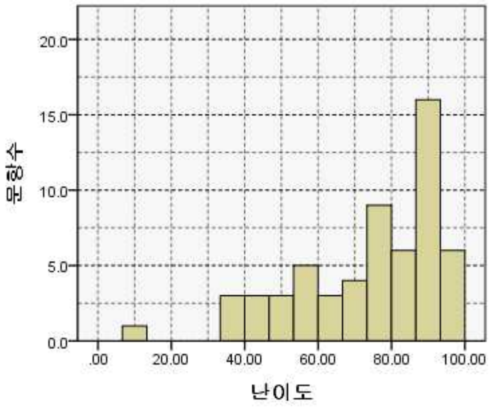

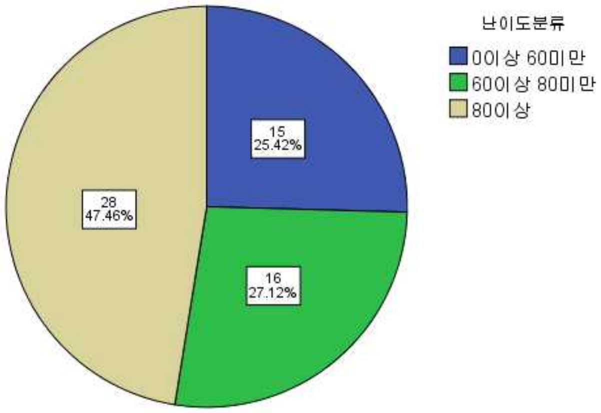

총점	난이도	표준편차
59	74.1	19.9

난이도	문항수	비율(%)
0~60미만	15	25.4
60~80미만	16	27.1
80~100	28	47.5
전체	59	100.0

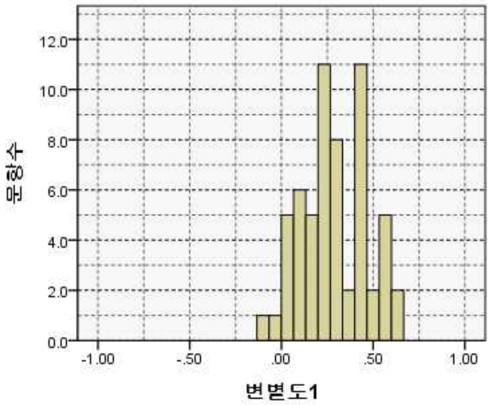

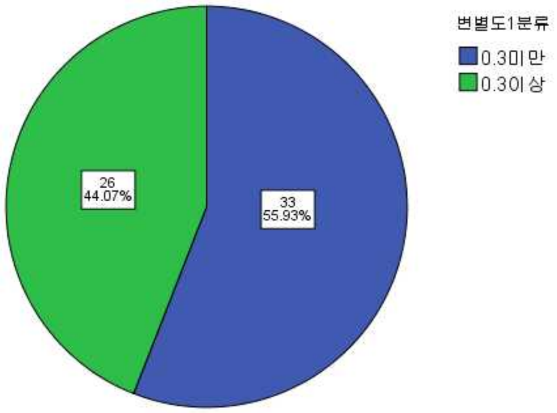

총점	변별도1	표준편차
59	.29	.18

변별도1	문항수	비율(%)
0.3미만	33	55.9
0.3이상	26	44.1
전체	59	100.0

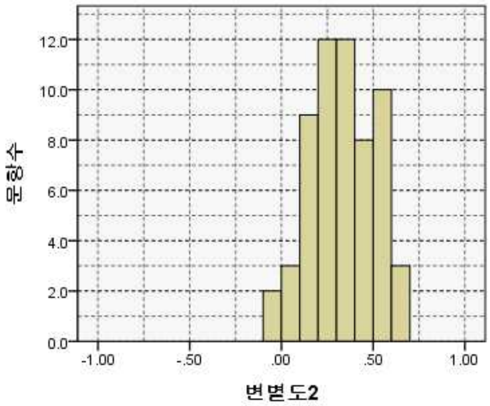

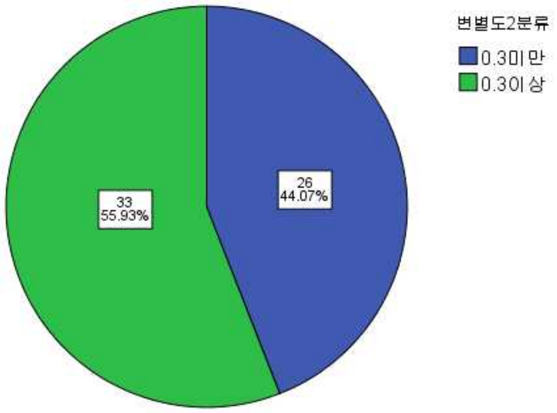

총점	변별도2	표준편차
59	.33	.17

변별도2	문항수	비율(%)
0.3미만	26	44.1
0.3이상	33	55.9
전체	59	100.0

해석

- 암기형 문항에서 난이도 지수가 80 에서 100 사이인 문항이 전체 59 문항 중 28 문항이었으며, 60 이상 80 미만인 문항이 16 문항, 60 미만인 문항이 15 문항인 것으로 나타남
- 변별도 1 지수를 기준으로 분류하였을 때, 0.3 미만인 문항이 33 문항으로 0.3 이상인 문항이 26 문항인 것에 비해 더 많이 나타남
- 변별도 2 지수를 기준으로 분류하였을 때, 0.3 미만인 문항이 26 문항으로 0.3 이상인 문항이 33 문항인 것에 비해 더 적게 나타남

(2) 해석형 난이도와 변별도 분포도 및 비율분석

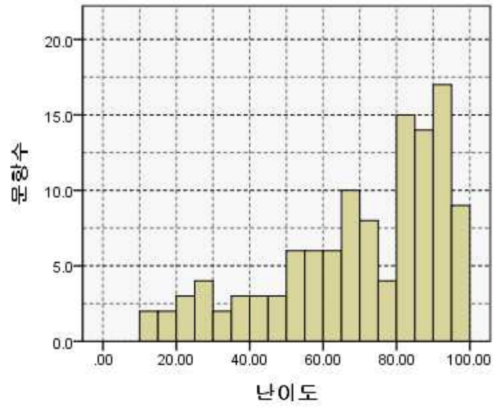

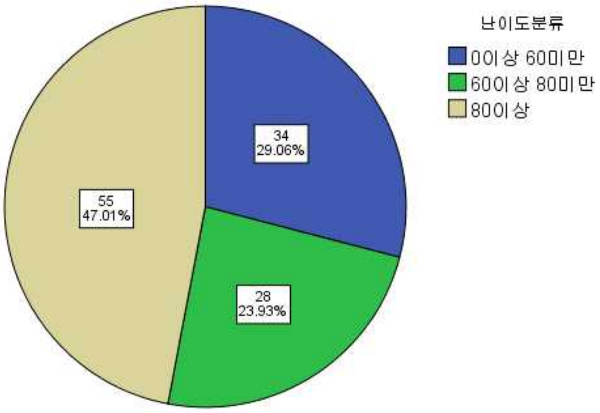

총점	난이도	표준편차	난이도	문항수	비율(%)
117	69.9	22.8	0~60미만	34	29.1
			60~80미만	28	23.9
			80~100	55	47.0
			전체	117	100.0

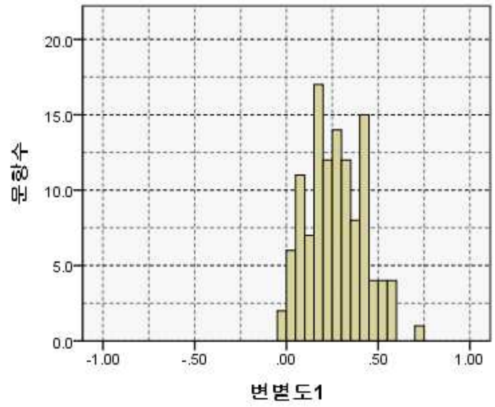

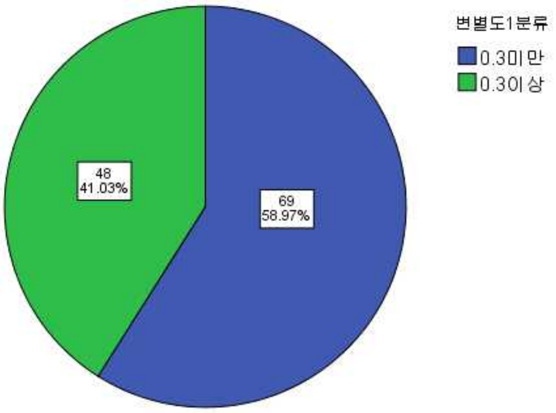

총점	변별도1	표준편차	변별도1	문항수	비율(%)
117	.26	.16	0.3미만	69	59.0
			0.3이상	48	41.0
			전체	117	100.0

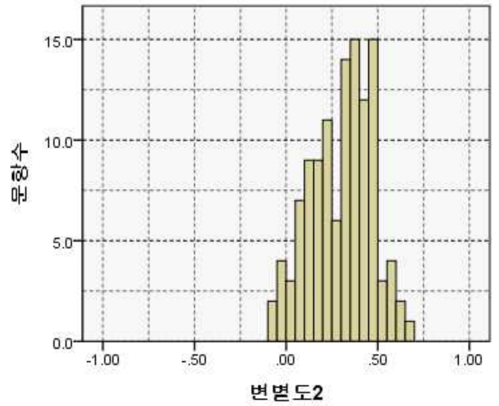

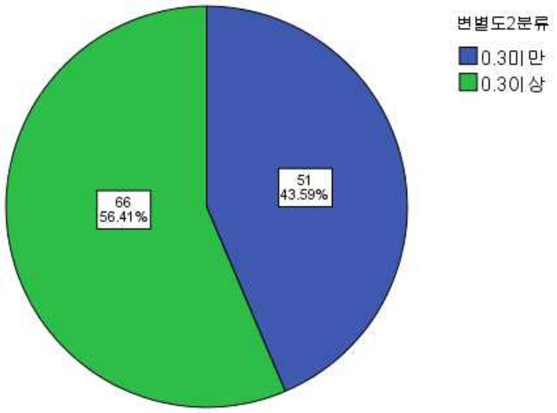

총점	변별도2	표준편차
117	.30	.17

변별도2	문항수	비율(%)
0.3미만	51	43.6
0.3이상	66	56.4
전체	117	100.0

해석

- 해석형 문항에서 난이도 지수가 80 에서 100 사이인 문항이 전체 117 문항 중 55 문항이었으며, 60 이상 80 미만인 문항이 28 문항, 60 미만인 문항이 34 문항인 것으로 나타남
- 변별도 1 지수를 기준으로 하였을 때, 0.3 미만인 문항이 69 문항으로 0.3 이상인 문항이 48 문항인 것에 비해 더 많이 나타남
- 변별도 2 지수를 기준으로 분류하였을 때, 0.3 미만인 문항이 51 문항으로 0.3 이상인 문항이 66 문항인 것에 비해 더 적게 나타남

(3) 해결형 난이도와 변별도 분포도 및 비율분석

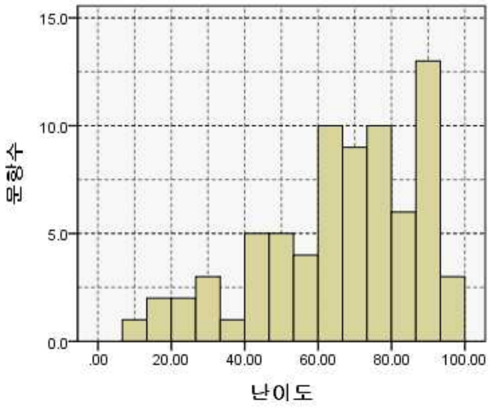

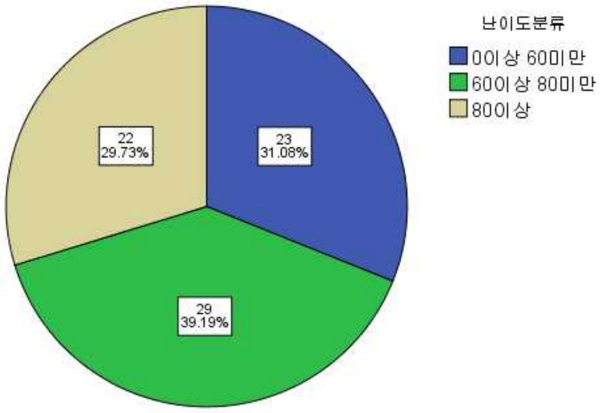

총점	난이도	표준편차
74	66.1	21.7

난이도	문항수	비율(%)
0~60미만	23	31.1
60~80미만	29	39.2
80~100	22	29.7
전체	74	100.0

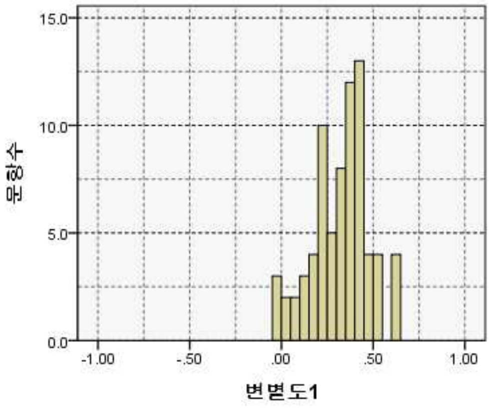

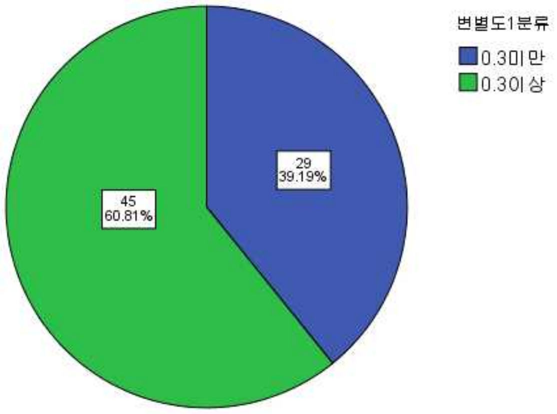

총점	변별도1	표준편차
74	.32	.16

변별도1	문항수	비율(%)
0.3미만	29	39.2
0.3이상	45	60.8
전체	74	100.0

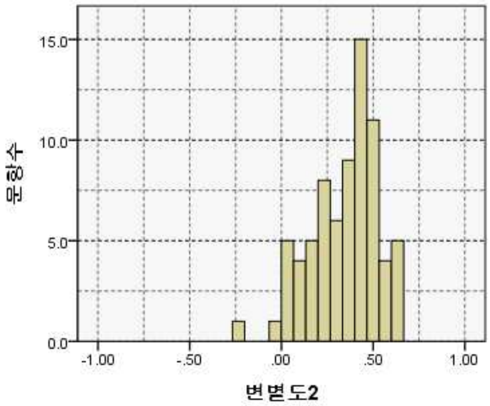

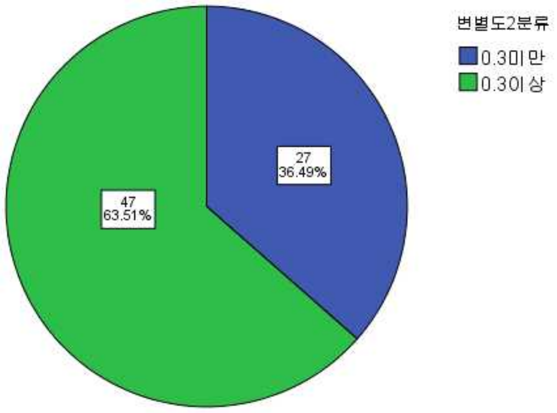

총점	변별도2	표준편차	변별도2	문항수	비율(%)
74	.34	.19	0.3미만	27	36.5
			0.3이상	47	63.5
			전체	74	100.0

해석

- 해결형 문항에서 난이도 지수가 80에서 100 사이인 문항이 전체 74문항 중 22문항이었으며, 60 이상 80 미만인 문항이 29문항, 60 미만인 문항이 23문항인 것으로 나타남
- 변별도 1 지수를 기준으로 분류 하였을 때, 0.3 미만인 문항이 29문항으로 0.3 이상인 문항이 45문항인 것에 비해 더 적게 나타남
- 변별도 2 지수를 기준으로 분류하였을 때, 0.3 미만인 문항이 27문항으로 0.3 이상인 문항이 47문항인 것에 비해 더 적게 나타남

3. 난이도와 변별도 간 산포도

1) 전체 난이도와 변별도 간 산포도

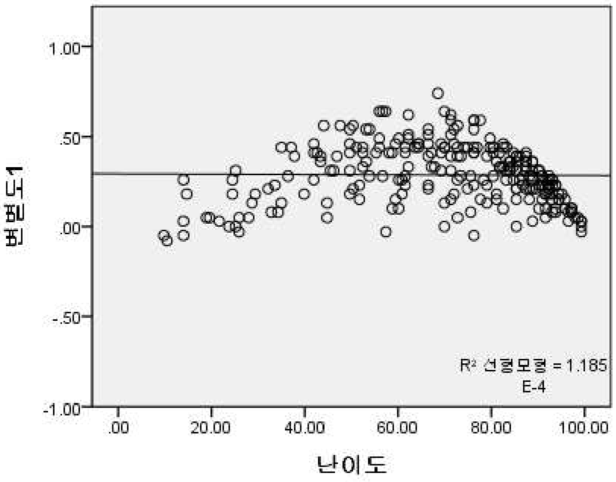

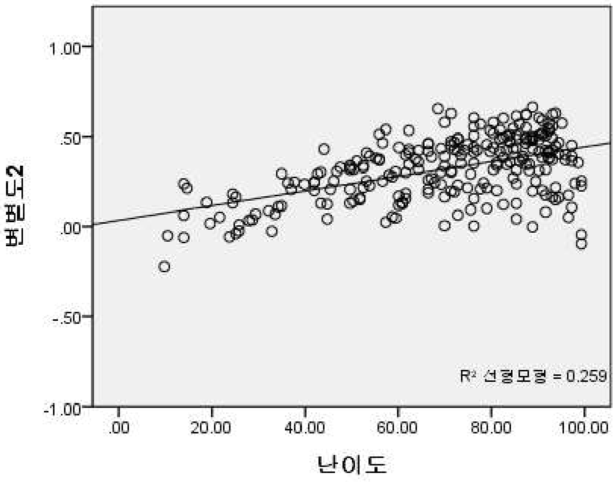

해석

- 난이도 지수와 변별도 1 지수 간 상관은 -.011로 관련성이 없는 것으로 나타남
- 난이도 지수와 변별도 2 지수 간 상관은 .509*로 난이도 지수가 높을수록 변별력이 높아지는 것으로 나타남

2) 과목별 난이도와 변별도 간 산포도

가) 한약학 기초 난이도와 변별도 간 산포도

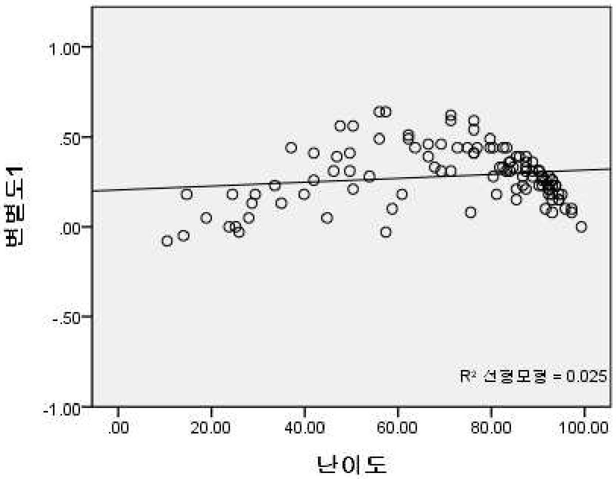

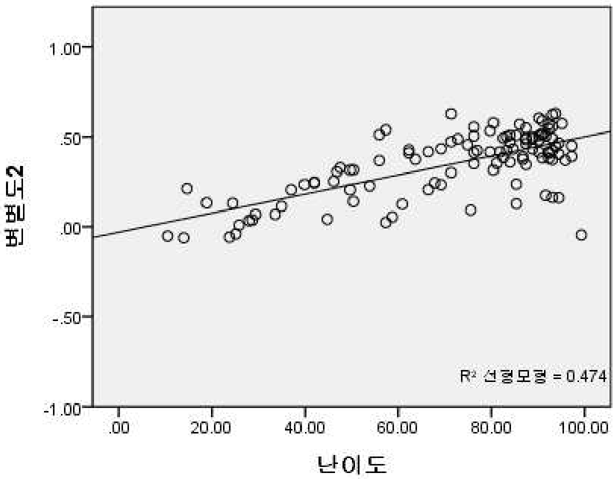

해석

- 난이도 지수와 변별도 1 지수 간 상관은 .159 로 관련성이 없는 것으로 나타남
- 난이도 지수와 변별도 2 지수 간 상관은 .688*로 난이도 지수가 높을수록 변별력이 높아지는 것으로 나타남

나) 보건·의약관계법규 난이도와 변별도 간 산포도

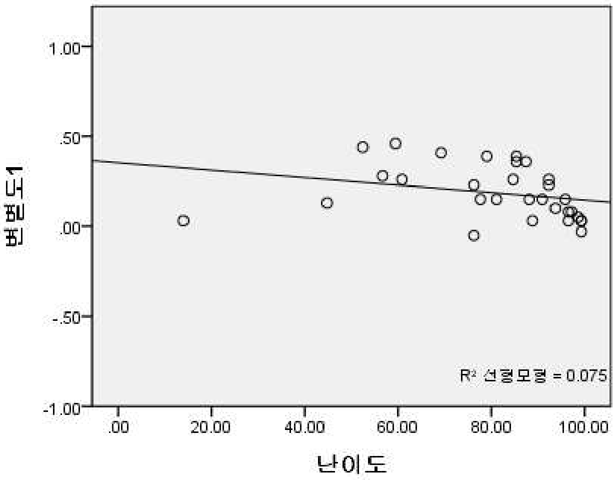

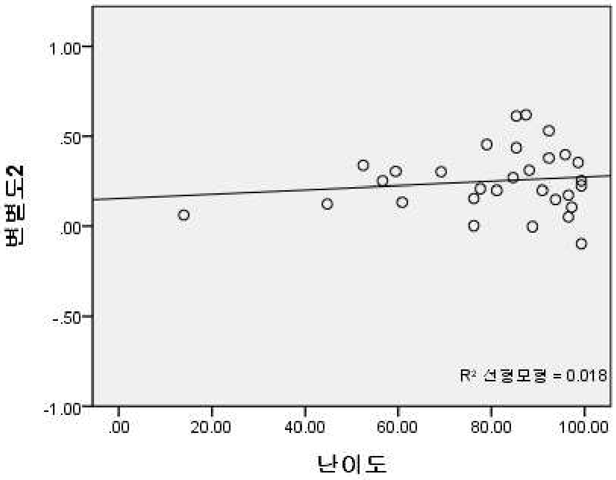

해석

- 난이도 지수와 변별도 1 지수 간 상관은 -.275 로 관련성이 없는 것으로 나타남
- 난이도 지수와 변별도 2 지수 간 상관은 .133 으로 관련성이 없는 것으로 나타남

다) 한약학 응용 난이도와 변별도 간 산포도

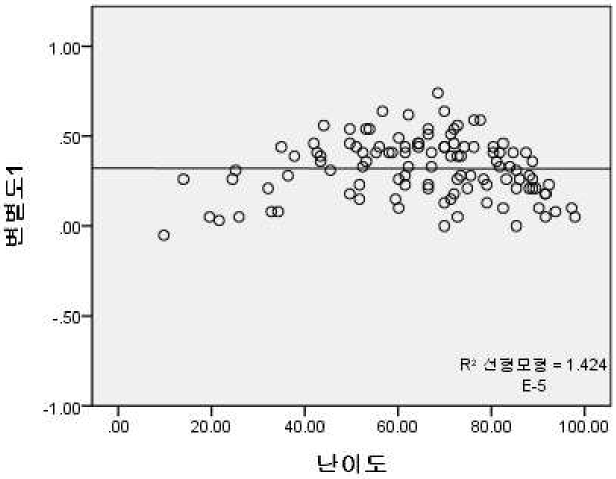

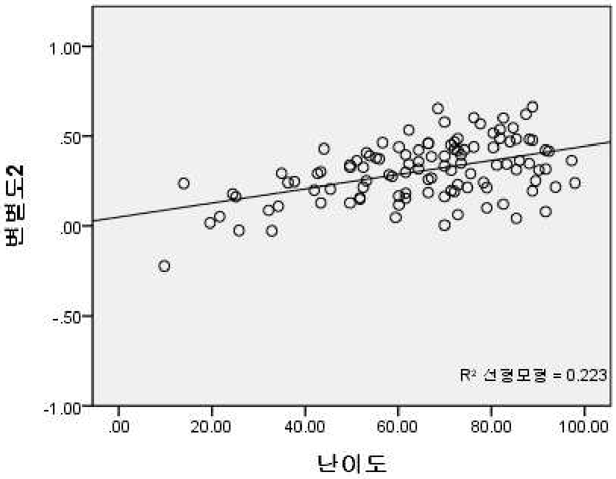

해석

- 난이도 지수와 변별도1 지수 간 상관은 -.004로 관련성이 없는 것으로 나타남
- 난이도 지수와 변별도2 지수 간 상관은 .472*로 난이도 지수가 높을수록 변별력이 높아지는 것으로 나타남

4. 신뢰도 분석

과목명	문항수	제19회	제20회	제21회	제22회	제23회
전체	250	.964	.962	.963	.955	.963
한약학 기초	110	.928	.924	.933	.927	.932
보건·의약관계법규	30	.681	.686	.642	.729	.665
한약학 응용	110	.927	.924	.917	.880	.920

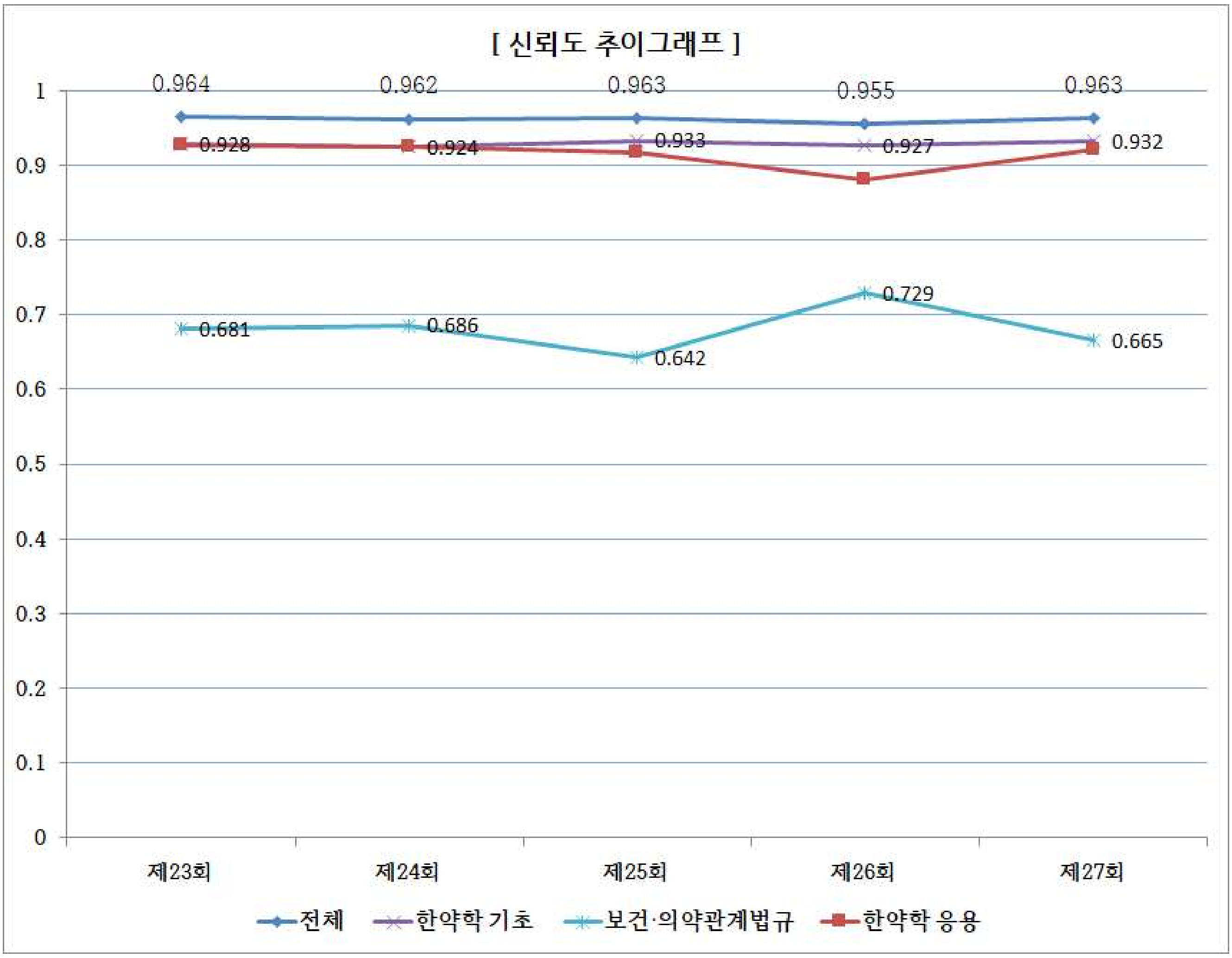

해석

- 전회 대비 전체 문항의 신뢰도는 .008 증가함
- 전회 대비 한약학 기초 과목 문항의 신뢰도는 .005 증가함
- 전회 대비 보건·의약관계법규 과목 문항의 신뢰도는 .064 감소함
- 전회 대비 한약학 응용 과목 문항의 신뢰도는 .040 증가함

○ 분석결과 관련 문의 : 한국보건의료인국가시험원 연구개발본부 김준기 전임연구원
Tel : 02-2087-8956, FAX : 02-2087-8885
E-mail : tontates@kuksiwon.or.kr
