# Supplementary material for: Item difficulty index, discrimination index, and reliability of the 26 health professions licensing examinations in 2022, Korea: a psychometric study
Source: J Educ Eval Health Prof. 2023 Nov 22;20:31. doi: 10.3352/jeehp.2023.20.31 (PMC11959405; doi:10.3352/jeehp.2023.20.31)
Supplement: Supplementary file 1 — Supplement 1. Item analysis results of 26 health professions licensing examinations administered during late 2022 and early 2023. [file jeehp-20-31_Suppl1.zip › 2022│Γ╡╡ ┴a28╚╕ 1▒▐ └└▒▐▒╕┴╢╗τ ▒╣░í╜├╟Φ ║╨╝«░ß░·.pdf]

2022년도 제28회
1급 응급구조사 국가시험
문항분석 결과

국민이 신뢰하고 감동하는 시험평가기관

한국보건의료인국가시험원

KOREA HEALTH PERSONNEL LICENSING EXAMINATION INSTITUTE

일반 용어 정의

□ **평균**

- 집단에서의 대표적 경향값으로 전체 값을 더하여 총 응시자로 나눈 값

□ **표준편차**

- 평균과 각 점수의 차이인 편차들의 평균으로 점수가 흩어져 분포되어 있는 정도

□ **추정난이도**

- 문항개발자가 예측한 정답률

□ **검사이론**

- 검사와 검사를 구성하고 있는 문항의 양호도를 분석 및 평가하는 방법을 정의한 이론체계
- 대표적으로 고전검사이론과 문항반응이론이 있음

고전검사이론 용어 정의

□ **고전검사이론(Classical Test Theory; CTT)**

- 검사의 질을 분석하는 검사이론 중 한 가지로 19세기 말부터 전개되어 현재까지 주로 사용되고 있는 검사이론임
- 고전검사이론에 의한 문항과 응시자 능력 추정치는 다음과 같음

○ **문항난이도**

· 검사 문항의 쉽고 어려운 정도를 나타내는 지수
· 난이도 지수는 총 반응 수에 대한 정답 반응 수의 비율로 문항의 정답률임
· 문항난이도는 0~100까지의 값을 가짐
· 난이도 값이 큰 경우, 쉬운 문항으로 '난이도가 낮다'라고 해석하며, 난이도 값이 작은 경우, 어려운 문항으로 '난이도가 높다'라고 해석함

○ **문항변별도**

· 각 문항이 응시자의 능력 수준을 변별할 수 있는 정도를 나타내는 지수
· 문항변별도는 -1~+1까지의 값을 가지며, 1에 가까울수록 변별력 크다고 해석함
· 일반적으로 문항변별도가 0.3 이상이면 우수한 문항으로 평가함
· 구하는 방식에는 '상하위집단 구분법', '문항-총점 상관계수' 등이 있음
  1) 변별도 1(상하위구분법): 상위 27%와 하위 27% 집단의 난이도 차이를 구하는 방식
  2) 변별도 2(상관계수법): 문항-총점과의 상관계수로 구하는 방식

○ **신뢰도**

· 시험이 평가하고자 하는 것을 일관성 있게 측정하는가로 시험이 오차없이 정확하게 측정한 정도를 의미함
· 국시원에서는 문항의 내적일관성(Cronbach α)으로 신뢰도를 추정하며 1에 가까울수록 신뢰도가 높다고 해석함

목 차

I. 시행 결과 ······ 6
1. 시험 현황 ······ 7
1) 시험명 ······ 7
2) 시험시행일 ······ 7
3) 응시현황 ······ 7
4) 과목별 문항 수, 배점 및 과락 점수 ······ 7
2. 합격률과 평균성적 ······ 7
1) 합격 및 불합격 현황 ······ 7
2) 과목별 과락자수 내역 ······ 8
3) 전회 대비 합격률과 평균성적 ······ 8

II. 문항분석 결과 ······ 9
1. 성적 ······ 10
1) 전체 성적분포도 ······ 10
2) 과목별 성적분포도 ······ 11
2. 난이도와 변별도 ······ 13
1) 전체 난이도와 변별도 ······ 13
2) 과목별 난이도와 변별도 ······ 16
3) 지식수준별 난이도와 변별도 ······ 31
3. 난이도와 변별도 간 산포도 ······ 39
1) 전체 난이도와 변별도 간 산포도 ······ 39
2) 과목별 난이도와 변별도 간 산포도 ······ 39
4. 신뢰도 분석 ······ 43

I. 시행 결과

1. 시험 현황

1) 시험명: 2022년도 제28회 1급 응급구조사 국가시험

2) 시험시행일: 2022년 11월 26일

3) 응시현황

구분	응시대상자수	결시자수	부정행위자수	응시자 준수사항 위반자 수		응시자수 (%)
				휴대폰 소지	신분증 미지참	
실기	1,753	17	0	0	0	1,736 (99.0)
필기	1,753	47	0	0	0	1,705 (97.3)
종합	1,753	※16	0	0	0	1,736 (99.0)

※실기•필기 모두 결시

4) 과목별 문항 수, 배점 및 과락 점수

교 시	과 목 명	문제 수	배점	총점	합격자 점수기준	
					과목 과락기준	총점 합격기준
1교시	기초의학	30	1	30	12점 미만	174점 이상
1교시	응급환자관리	40	1	40	16점 미만	
1교시	전문응급처치학총론	30	1	30	12점 미만	
1교시	응급의료관련법령	20	1	20	8점 미만	
2교시	전문응급처치학각론	110	1	110	44점 미만	
	실기시험	3	20	60	36점 미만	
계		233		290		

2. 합격률과 평균성적

1) 합격 및 불합격 현황

구분	합격자수 (%)	불합격자수(%)				채점보류자수
		평락	과락	기권	계	
실기	1,690 (97.4)	-	46 (2.7)	-	46 (2.7)	-
필기	1,530 (89.7)	93 (5.5)	82 (4.8)	-	175 (10.3)	1
종합	1,530 (88.1)	※124 (7.1)	82 (4.7)	-	206 (11.9)	1

※실기•필기 합산점수 평락

2) 과목별 과락자수 내역

과목명 / 과락자수	기초의학	응급환자관리	전문응급 처치학총론	응급의료 관련법령	전문응급 처치학각론	실기시험
과목별 과락자 수	59	2	-	16	-	10
전과목 과락자 수	82※					

※2과목 과락자수 5명

3) 전회 대비 합격률과 평균성적

회차	년도	합격률(%)	평균성적	표준편차	백분율 환산점수
제24회	2018	88.3	215.5	25.8	74.3
제25회	2019	88.1	213.1	26.2	73.4
제26회	2020	85.9	209.2	26.7	72.1
제27회	2021	75.3	205.6	24.4	70.9
제28회	2022	88.1	222.9	26.9	76.9

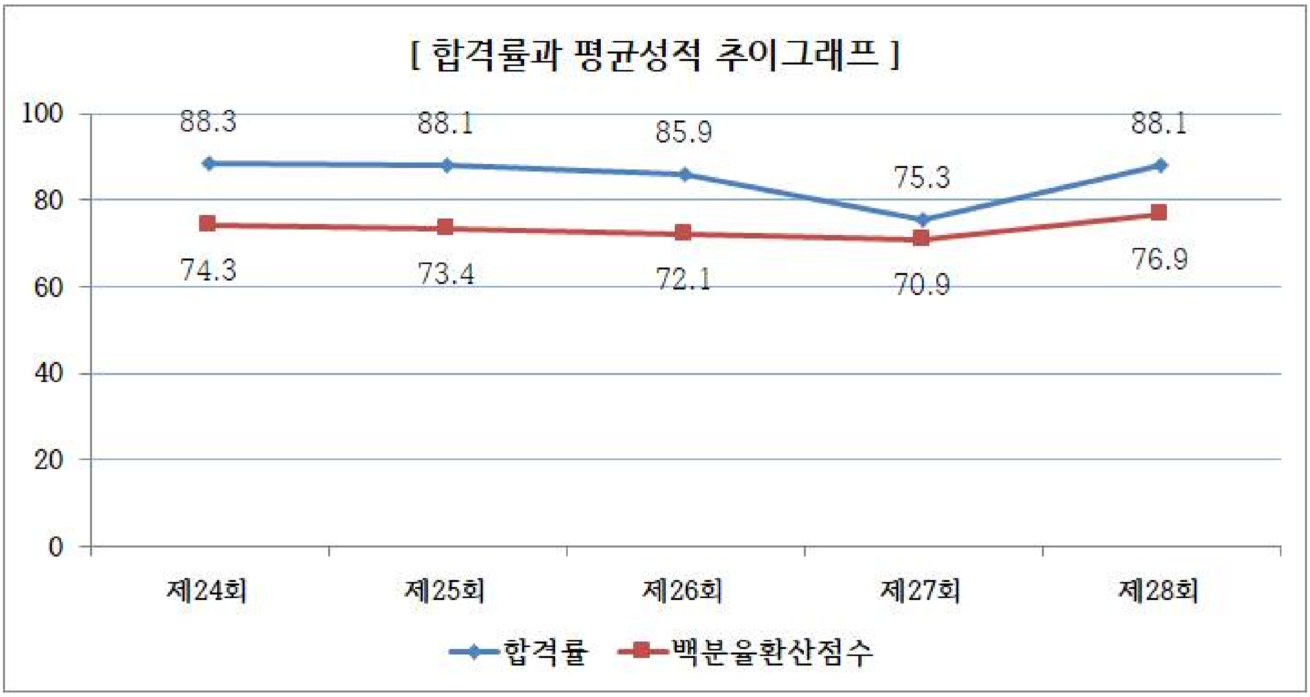

해석

- 전년 대비 합격률은 12.8% 증가하고, 백분율 환산점수는 6.0 점 증가함

Ⅱ. 문항분석 결과

1. 성적

1) 전체 성적분포도

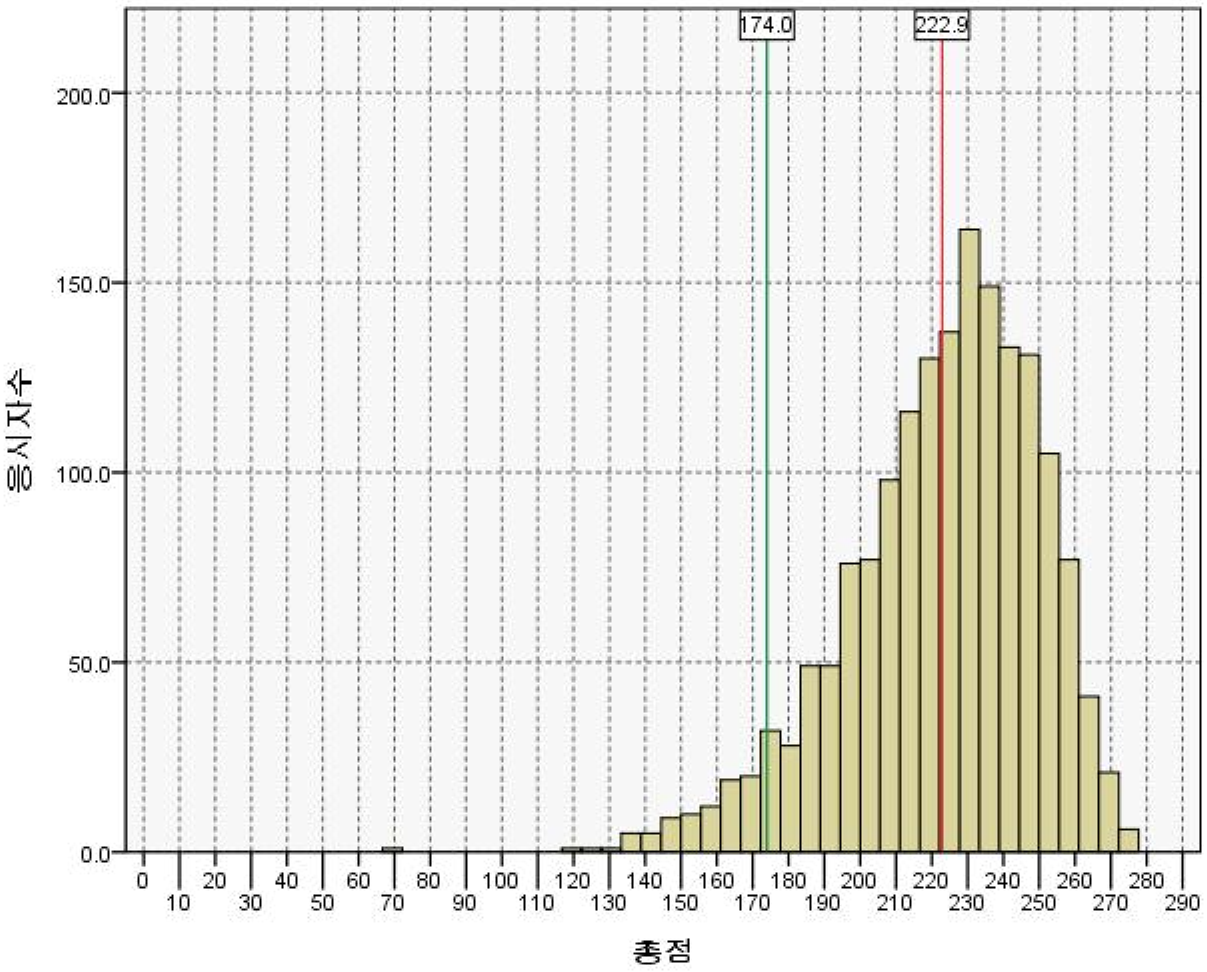

응시자	총점	합격선	평균성적	표준편차
1,705	290	174	222.9	26.9

2) 과목별 성적분포도

가) 기초의학

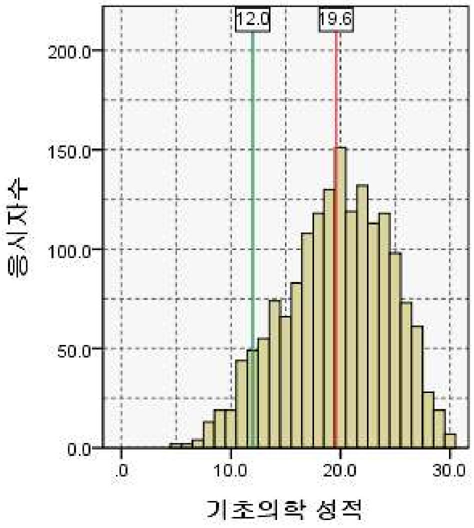

총점	과락선	평균성적	표준편차
30	12	19.6	4.8

나) 응급환자관리

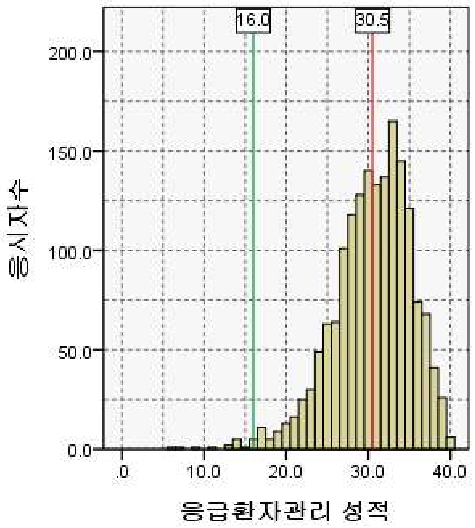

총점	과락선	평균성적	표준편차
40	16	30.5	4.8

다) 전문응급처치학총론

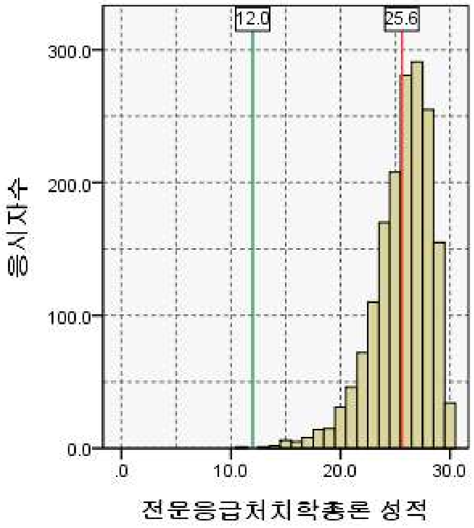

총점	과락선	평균성적	표준편차
30	12	25.6	2.7

라) 응급의료관련법령

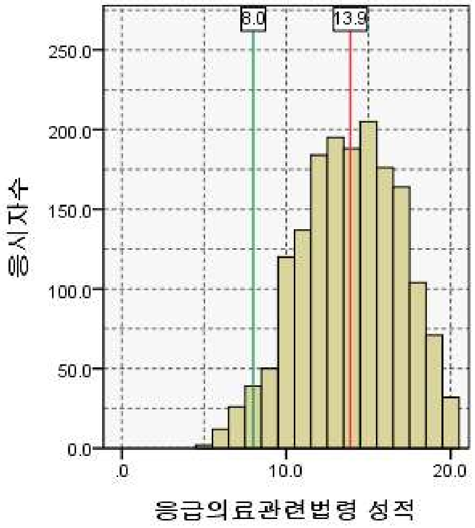

총점	과락선	평균성적	표준편차
20	8	13.9	3.1

마) 전문응급처치학각론

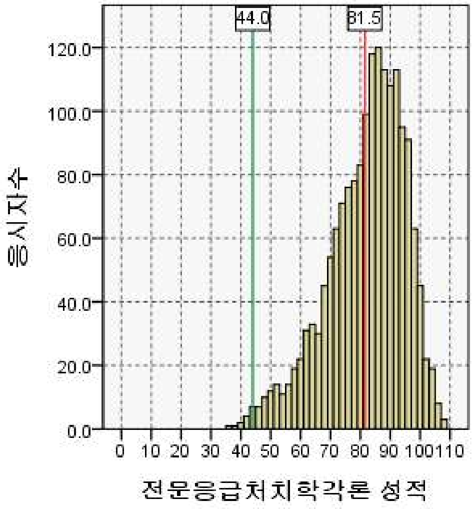

총점	과락선	평균성적	표준편차
110	44	81.5	12.9

2. 난이도와 변별도

1) 전체 난이도와 변별도

가) 전회 대비 전체 난이도와 변별도

회차	난이도		변별도1		변별도2	
	평균	표준편차	평균	표준편차	평균	표준편차
제24회	71.8	19.1	.23	.12	.23	.10
제25회	70.5	19.0	.24	.12	.24	.10
제26회	68.9	20.3	.25	.13	.25	.10
제27회	67.1	22.7	.23	.12	.22	.10
제28회	74.4	18.6	.26	.13	.26	.10

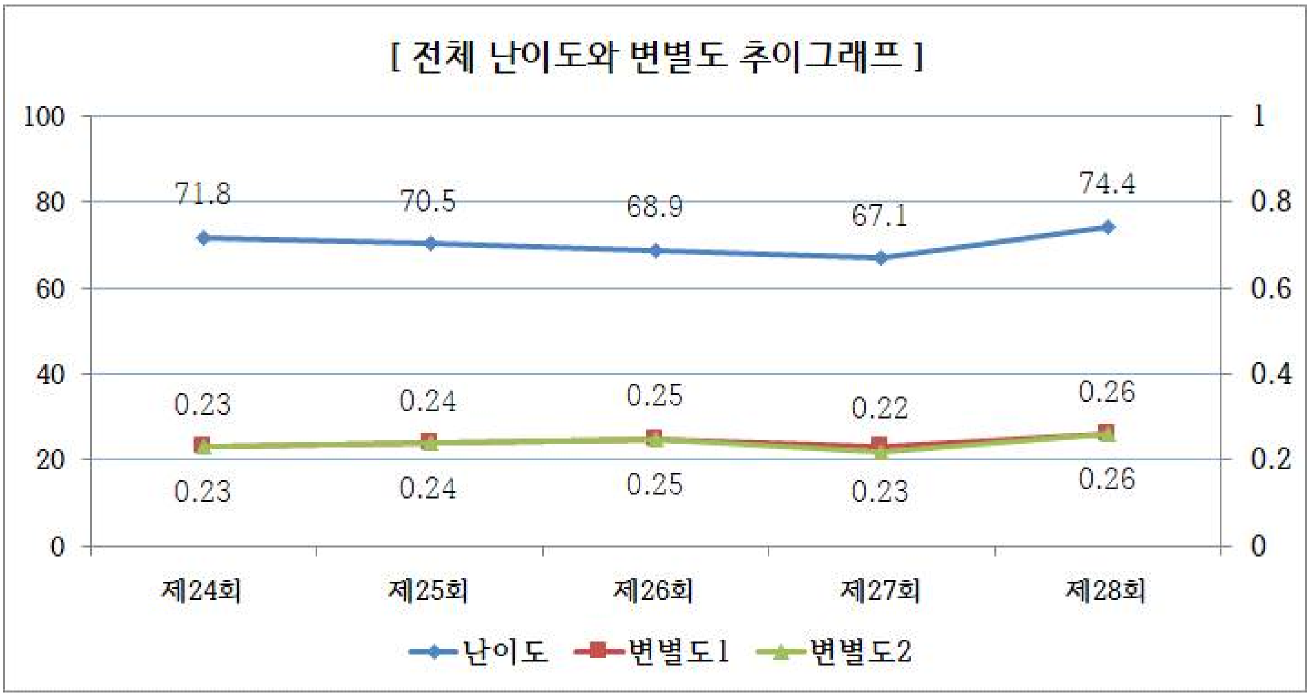

해석

- 전년 대비 난이도 지수는 7.3 증가함
- 변별도 1 지수는 .03 증가함
- 변별도 2 지수는 .04 증가함

나) 전체 난이도와 변별도 분포도 및 비율분석

(1) 전체 난이도 분포도 및 비율분석

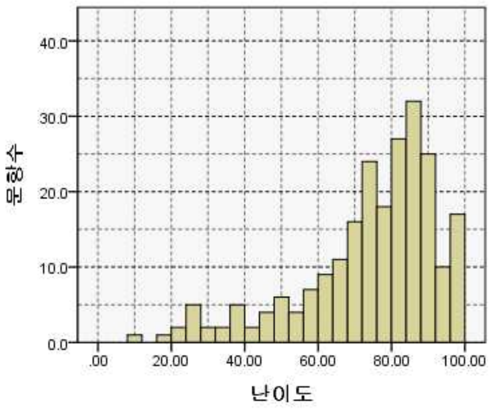

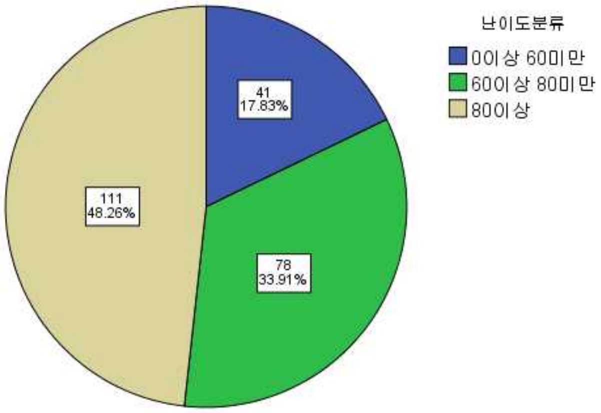

총점	난이도	표준편차
230	74.4	18.5

난이도	문항수	비율(%)
0~60미만	41	17.8
60~80미만	78	33.9
80~100	111	48.3
전체	230	100.0

(2) 전체 변별도1 분포도 및 비율분석

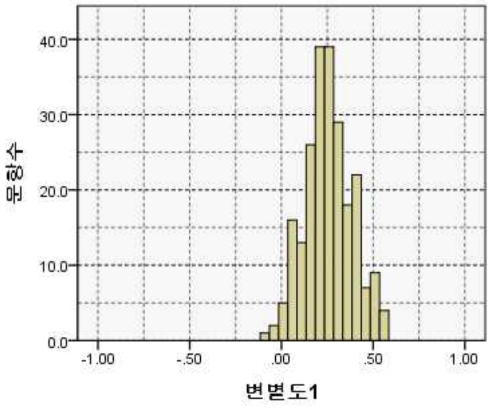

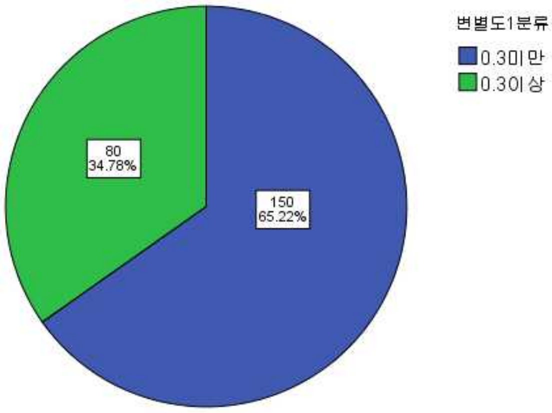

총점	변별도1	표준편차
230	.26	.13

변별도1	문항수	비율(%)
0.3미만	150	65.2
0.3이상	80	34.8
전체	230	100.0

(3) 전체 변별도2 분포도 및 비율분석

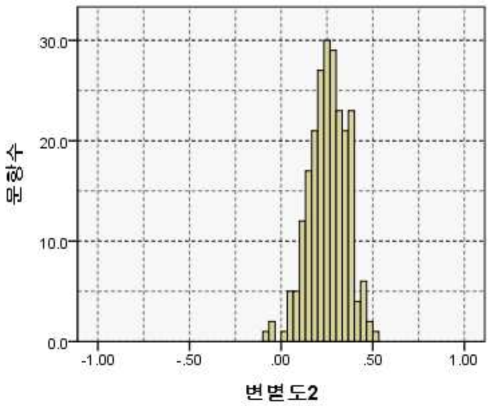

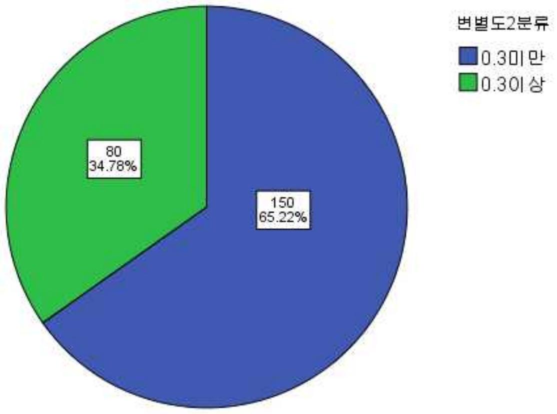

총점	변별도2	표준편차
230	.26	.10

변별도2	문항수	비율(%)
0.3미만	150	65.2
0.3이상	80	34.8
전체	230	100.0

해석

- 난이도 지수가 80에서 100사이인 문항이 전체 230문항 중 111문항이었으며, 60이상 80미만인 문항이 78문항, 60미만인 문항이 41문항인 것으로 나타남
- 변별도1 지수를 기준으로 분류하였을 때, 0.3 미만인 문항이 150문항으로 0.3 이상인 문항이 80문항인 것에 비해 더 많이 나타남
- 변별도2 지수를 기준으로 분류하였을 때, 0.3 미만인 문항이 150문항으로 0.3 이상인 문항이 80문항인 것에 비해 더 많이 나타남

2) 과목별 난이도와 변별도

가) 전회 대비 과목별 난이도와 변별도

(1) 전회 대비 기초의학 난이도와 변별도

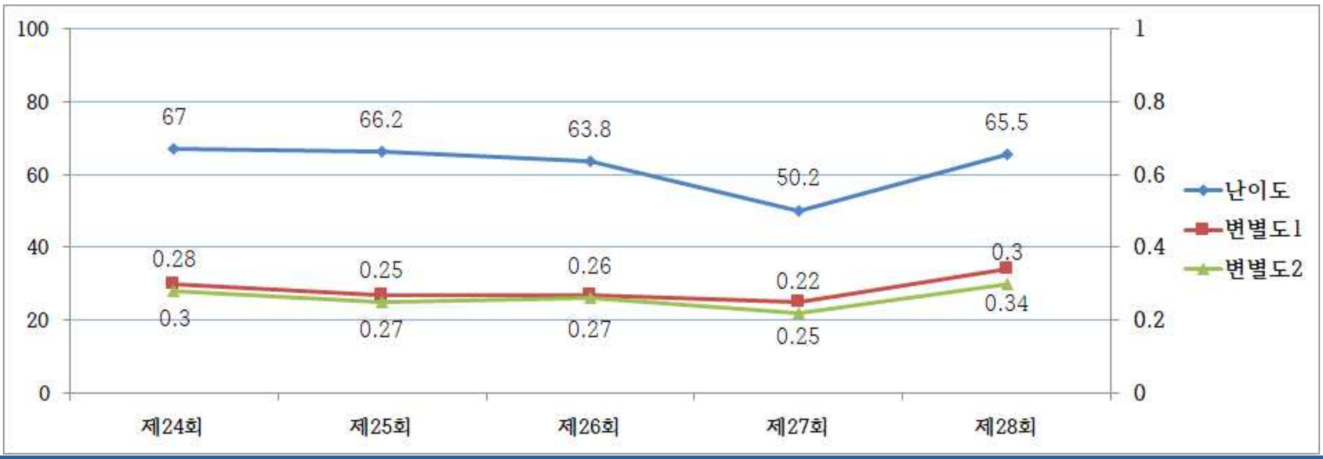

회차	난이도		변별도1		변별도2	
	평균	표준편차	평균	표준편차	평균	표준편차
제24회	67.0	18.8	.30	.11	.28	.08
제25회	66.2	16.5	.27	.12	.25	.10
제26회	63.8	24.8	.27	.14	.26	.10
제27회	50.2	22.3	.25	.12	.22	.09
제28회	65.5	19.1	.34	.11	.30	.08

해석

- 전회 대비 기초의학 과목의 난이도 지수는 15.3 증가함
- 전회 대비 기초의학 과목의 변별도 1 지수는 .09 증가함
- 전회 대비 기초의학 과목의 변별도 2 지수는 .08 증가함

(2) 전회 대비 응급환자관리 난이도와 변별도

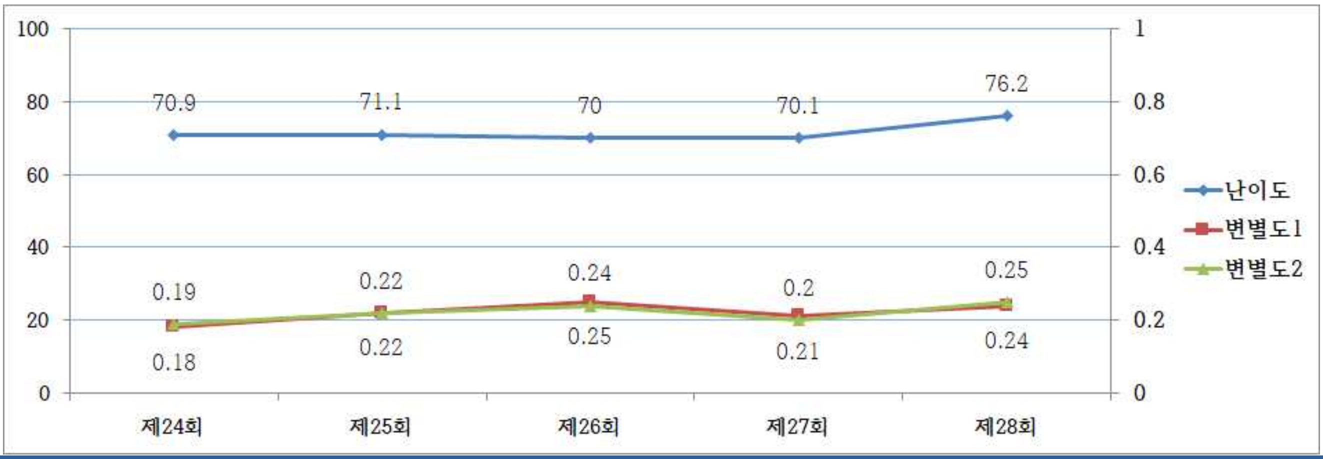

회차	난이도		변별도1		변별도2	
	평균	표준편차	평균	표준편차	평균	표준편차
제24회	70.9	24.3	.18	.11	.19	.09
제25회	71.1	19.3	.22	.12	.22	.11
제26회	70.0	20.3	.25	.13	.24	.09
제27회	70.1	23.7	.21	.12	.20	.09
제28회	76.2	16.6	.24	.10	.25	.09

해석

- 전회 대비 응급환자관리 과목의 난이도 지수는 5.1 증가함
- 전회 대비 응급환자관리 과목의 변별도 1 지수는 .03 증가함
- 전회 대비 응급환자관리 과목의 변별도 2 지수는 .05 증가함

(3) 전회 대비 전문응급처치학총론 난이도와 변별도

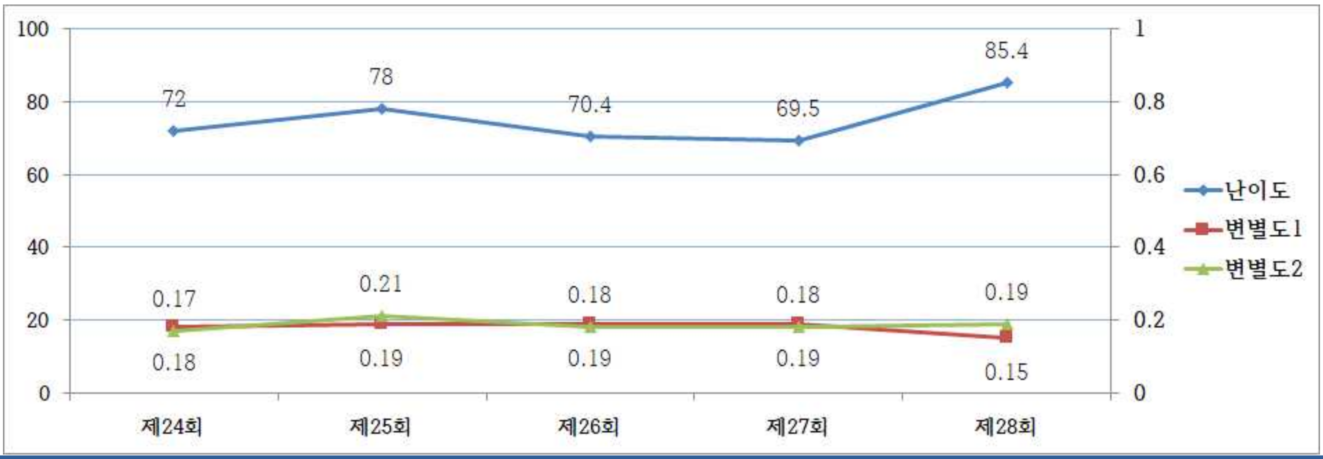

회차	난이도		변별도1		변별도2	
	평균	표준편차	평균	표준편차	평균	표준편차
제24회	72.0	19.5	.18	.13	.17	.10
제25회	78.0	17.2	.19	.10	.21	.09
제26회	70.4	19.7	.19	.12	.18	.09
제27회	69.5	21.9	.19	.12	.18	.10
제28회	85.4	13.9	.15	.12	.19	.12

해석

- 전회 대비 전문응급처치학총론 과목의 난이도 지수는 15.9 증가함
- 전회 대비 전문응급처치학총론 과목의 변별도 1 지수는 .04 감소함
- 전회 대비 전문응급처치학총론 과목의 변별도 2 지수는 .01 증가함

(4) 전회 대비 전문응급처치학각론 난이도와 변별도

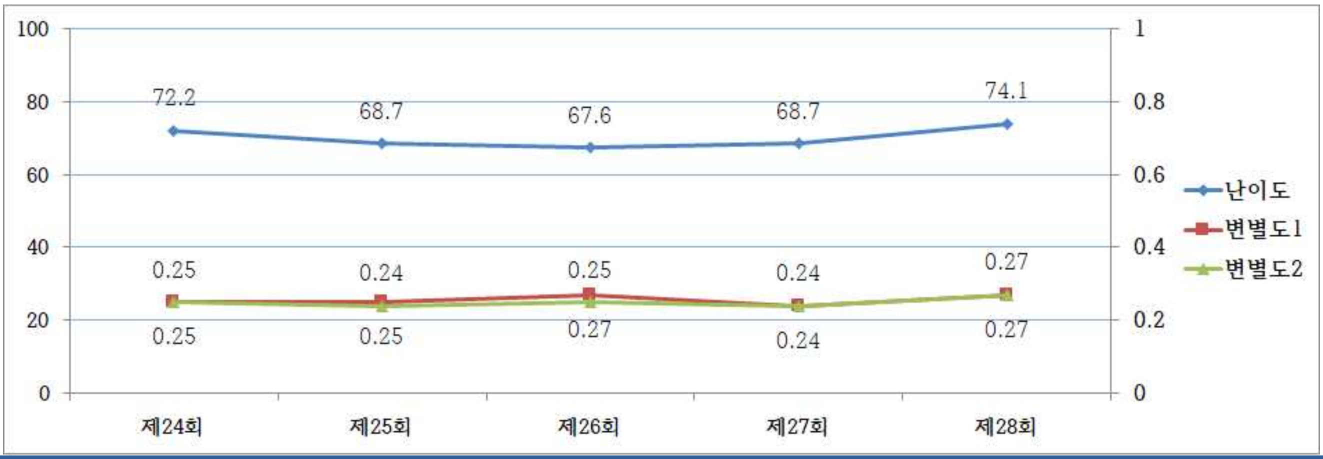

회차	난이도		변별도1		변별도2	
	평균	표준편차	평균	표준편차	평균	표준편차
제24회	72.2	17.3	.25	.11	.25	.10
제25회	68.7	20.1	.25	.12	.24	.10
제26회	67.6	19.9	.27	.13	.25	.10
제27회	68.7	21.6	.24	.12	.24	.10
제28회	74.1	19.2	.27	.13	.27	.10

해석

- 전회 대비 전문응급처치학각론 과목의 난이도 지수는 5.4 증가함
- 전회 대비 전문응급처치학각론 과목의 변별도 1 지수는 .03 증가함
- 전회 대비 전문응급처치학각론 과목의 변별도 2 지수는 .03 증가함

(5) 전회 대비 응급의료관련법령 난이도와 변별도

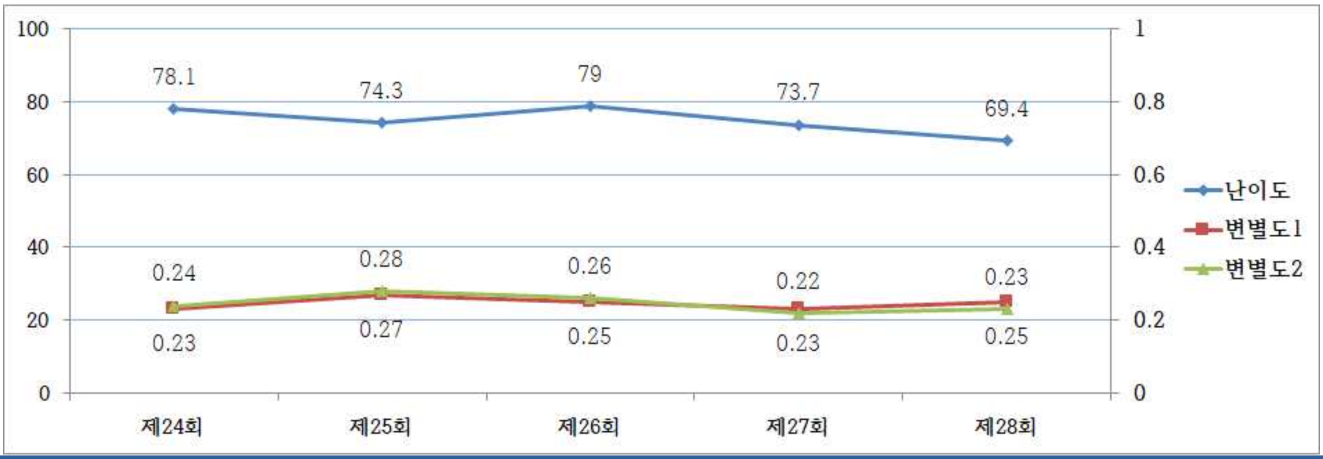

회차	난이도		변별도1		변별도2	
	평균	표준편차	평균	표준편차	평균	표준편차
제24회	78.1	16.2	.23	.13	.24	.10
제25회	74.3	16.2	.27	.12	.28	.09
제26회	79.0	12.9	.25	.13	.26	.09
제27회	73.7	18.5	.23	.12	.22	.07
제28회	69.4	16.3	.25	.10	.23	.08

해석

- 전회 대비 응급의료관련법령 과목의 난이도 지수는 4.3 감소함
- 전회 대비 응급의료관련법령 과목의 변별도 1 지수는 .02 증가함
- 전회 대비 응급의료관련법령 과목의 변별도 2 지수는 .01 증가함

나) 과목별 난이도와 변별도 분포도 및 비율분석

(1) 기초의학 난이도와 변별도 분포도 및 비율분석

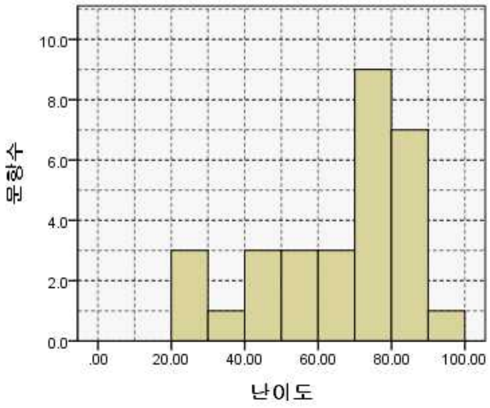

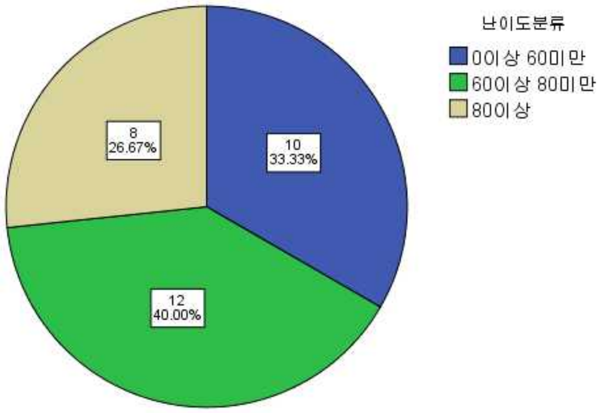

총점	난이도	표준편차
30	65.5	19.1

난이도	문항수	비율(%)
0~60미만	10	33.3
60~80미만	12	40.0
80~100	8	26.7
전체	30	100.0

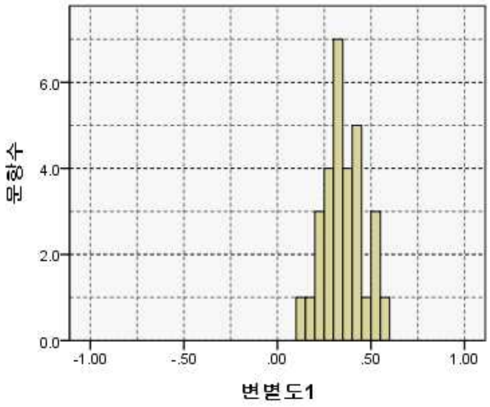

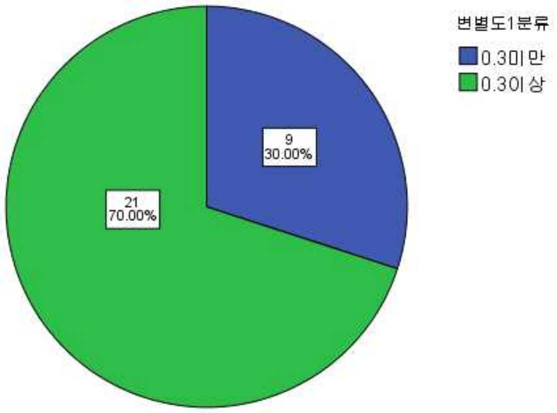

총점	변별도1	표준편차
30	.34	.11

변별도1	문항수	비율(%)
0.3미만	9	30.0
0.3이상	21	70.0
전체	30	100.0

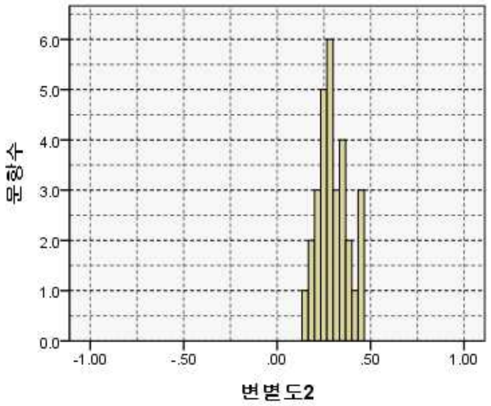

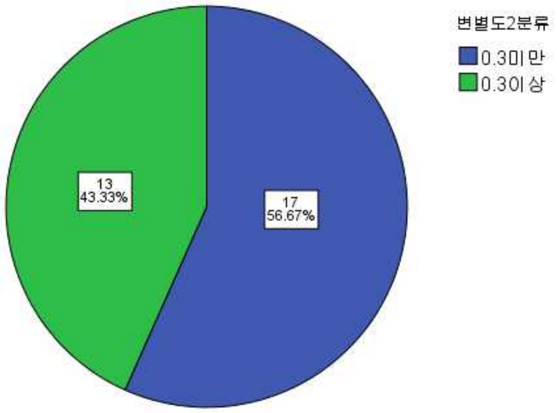

총점	변별도2	표준편차
30	.30	.08

변별도2	문항수	비율(%)
0.3미만	17	56.7
0.3이상	13	43.3
전체	30	100.0

해석

- 기초의학 과목에서 난이도 지수가 80 에서 100 사이인 문항이 전체 30 문항 중 8 문항이었으며, 60 이상 80 미만인 문항이 12 문항, 60 미만인 문항이 10 문항으로 나타남
- 변별도 1 지수를 기준으로 분류하였을 때, 0.3 미만인 문항이 9 문항으로 0.3 이상인 문항이 21 문항인 것에 비해 더 적게 나타남
- 변별도 2 지수를 기준으로 분류하였을 때, 0.3 미만인 문항이 17 문항으로 0.3 이상인 문항이 13 문항인 것에 비해 더 많이 나타남

(2) 응급환자관리 난이도와 변별도 분포도 및 비율분석

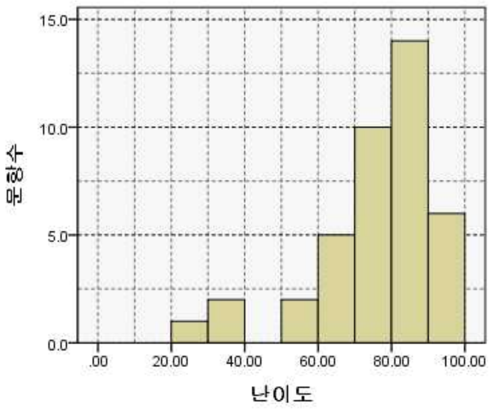

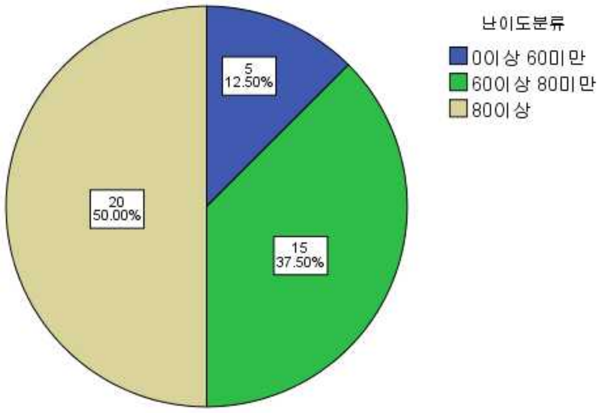

총점	난이도	표준편차
40	76.2	16.6

난이도	문항수	비율(%)
0~60미만	5	12.5
60~80미만	15	37.5
80~100	20	50.0
전체	40	100.0

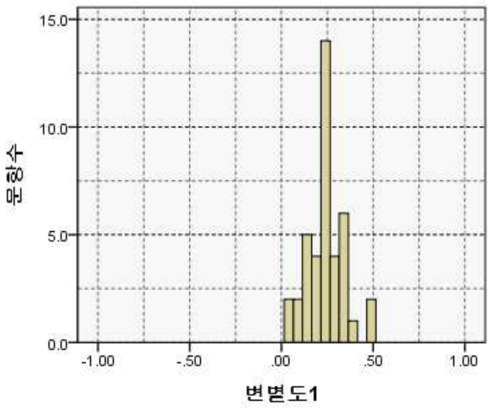

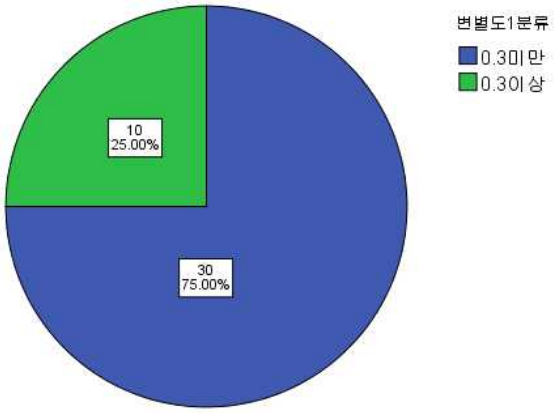

총점	변별도1	표준편차
40	.24	.10

변별도1	문항수	비율(%)
0.3미만	30	75.0
0.3이상	10	25.0
전체	40	100.0

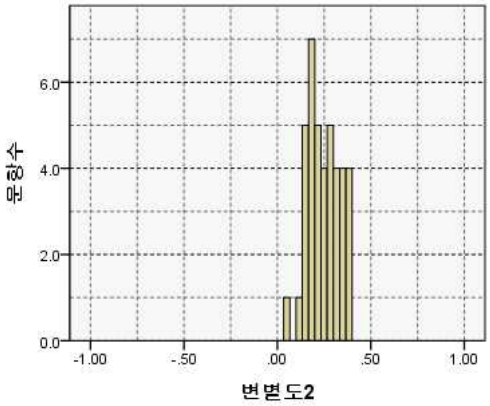

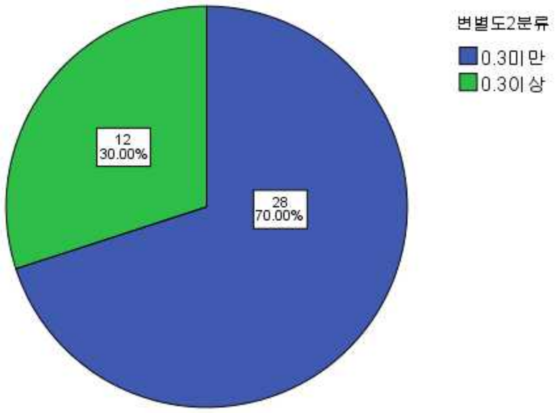

총점	변별도2	표준편차
40	.25	.09

변별도2	문항수	비율(%)
0.3미만	28	70.0
0.3이상	12	30.0
전체	40	100.0

해석

- 응급환자관리 과목에서 난이도 지수가 80 에서 100 사이인 문항이 전체 40 문항 중 20 문항이었으며, 60 이상 80 미만인 문항이 15 문항, 60 미만인 문항이 5 문항으로 나타남
- 변별도 1 지수를 기준으로 분류하였을 때, 0.3 미만인 문항이 30 문항으로 0.3 이상인 문항이 10 문항인 것에 비해 더 많이 나타남
- 변별도 2 지수를 기준으로 분류하였을 때, 0.3 미만인 문항이 28 문항으로 0.3 이상인 문항이 12 문항인 것에 비해 더 많이 나타남

(3) 전문응급처치학총론 난이도와 변별도 분포도 및 비율분석

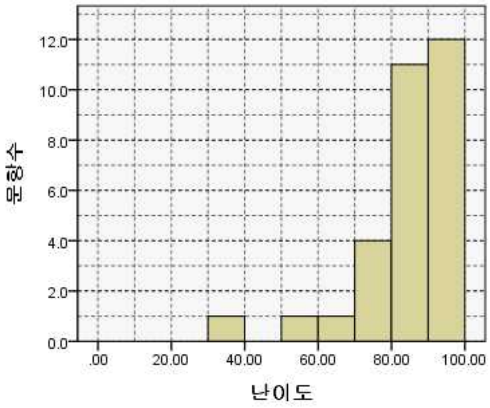

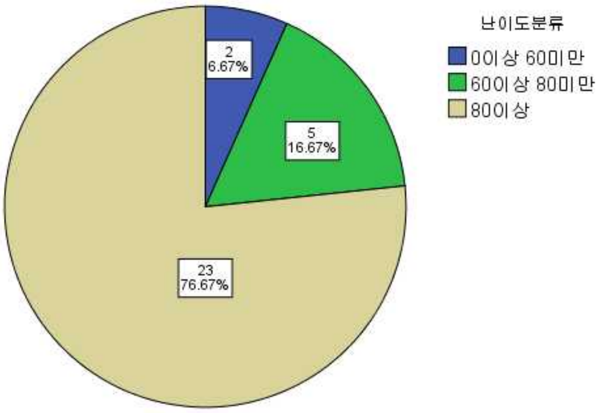

총점	난이도	표준편차
30	85.4	13.9

난이도	문항수	비율(%)
0~60미만	2	6.7
60~80미만	5	16.7
80~100	23	76.7
전체	30	100.0

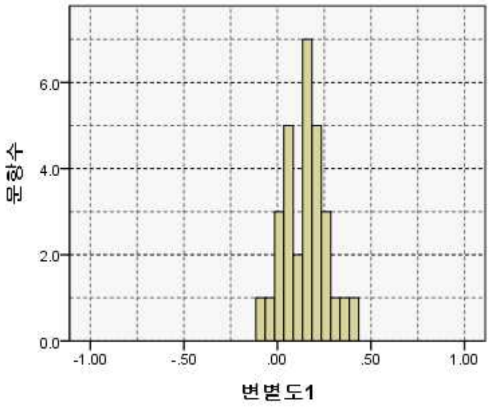

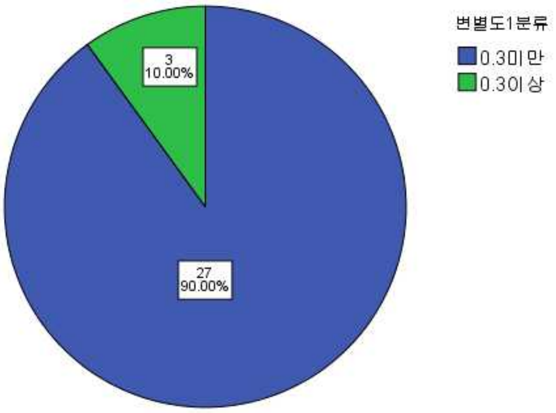

총점	변별도1	표준편차
30	.15	.12

변별도1	문항수	비율(%)
0.3미만	27	90.0
0.3이상	3	10.0
전체	30	100.0

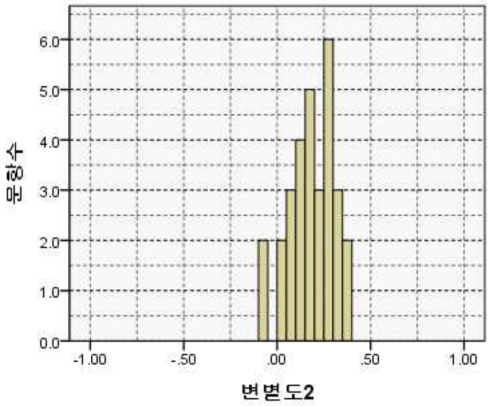

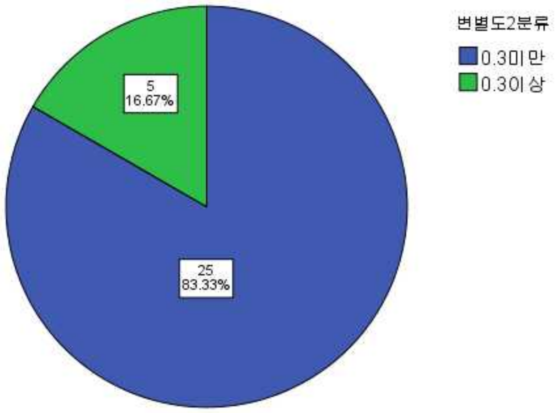

총점	변별도2	표준편차
30	.19	.12

변별도2	문항수	비율(%)
0.3미만	25	83.3
0.3이상	5	16.7
전체	30	100.0

해석

- 전문응급처치학총론 과목에서 난이도 지수가 80 에서 100 사이인 문항이 전체 30 문항 중 23 문항이었으며, 60 이상 80 미만인 문항이 5 문항, 60 미만인 문항이 2 문항으로 나타남
- 변별도 1 지수를 기준으로 분류하였을 때, 0.3 미만인 문항이 27 문항으로 0.3 이상인 문항이 3 문항인 것에 비해 더 많이 나타남
- 변별도 2 지수를 기준으로 분류하였을 때, 0.3 미만인 문항이 25 문항으로 0.3 이상인 문항이 5 문항인 것에 비해 더 많이 나타남

(4) 전문응급처치학각론 난이도와 변별도 분포도 및 비율분석

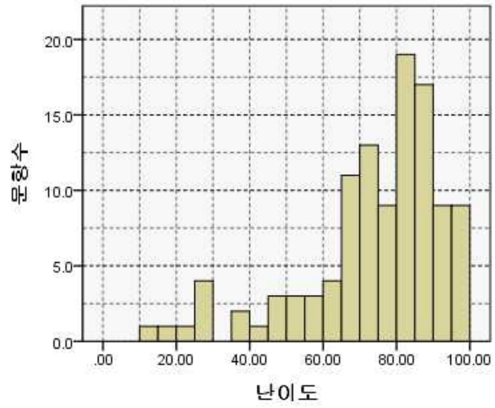

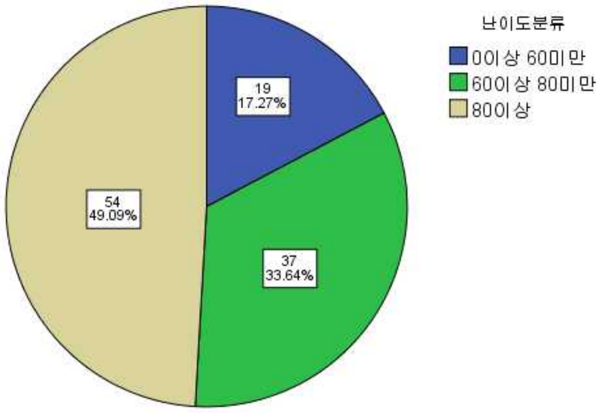

총점	난이도	표준편차
110	74.1	19.2

난이도	문항수	비율(%)
0~60미만	19	17.3
60~80미만	37	33.6
80~100	54	49.1
전체	110	100.0

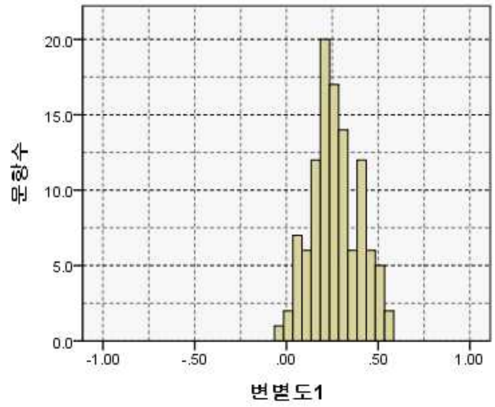

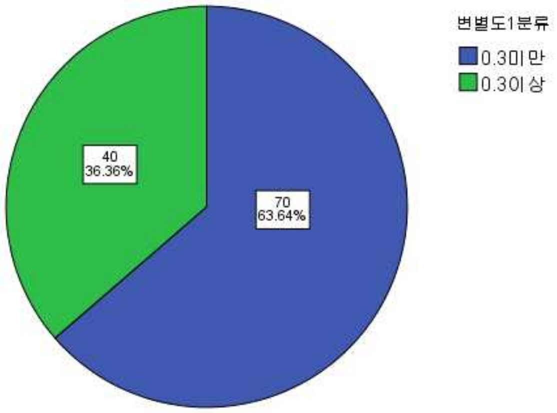

총점	변별도1	표준편차
110	.27	.13

변별도1	문항수	비율(%)
0.3미만	70	63.6
0.3이상	40	36.4
전체	110	100.0

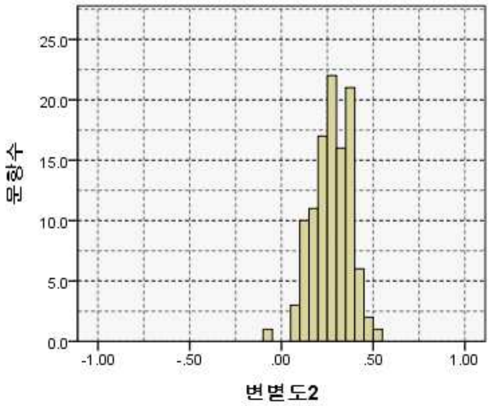

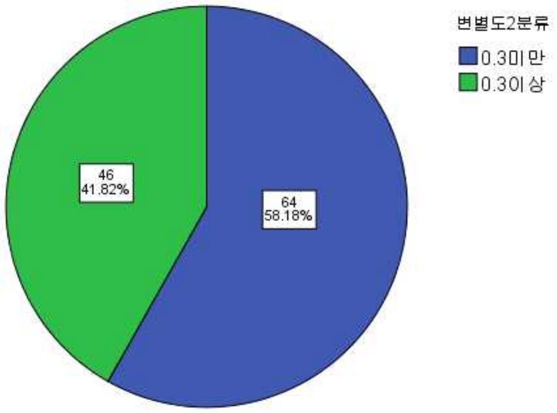

총점	변별도2	표준편차
110	.27	.10

변별도2	문항수	비율(%)
0.3미만	64	58.2
0.3이상	46	41.8
전체	110	100.0

해석

- 응급의료관련법령 과목에서 난이도 지수가 80 에서 100 사이인 문항이 전체 110 문항 중 54 문항이었으며, 60 이상 80 미만인 문항이 37 문항, 60 미만인 문항이 19 문항으로 나타남
- 변별도 1 지수를 기준으로 분류하였을 때, 0.3 미만인 문항이 70 문항으로 0.3 이상인 문항이 40 문항인 것에 비해 더 많이 나타남
- 변별도 2 지수를 기준으로 분류하였을 때, 0.3 미만인 문항이 64 문항으로 0.3 이상인 문항이 46 문항인 것에 비해 더 많이 나타남

(5) 응급의료관련법령 난이도와 변별도 분포도 및 비율분석

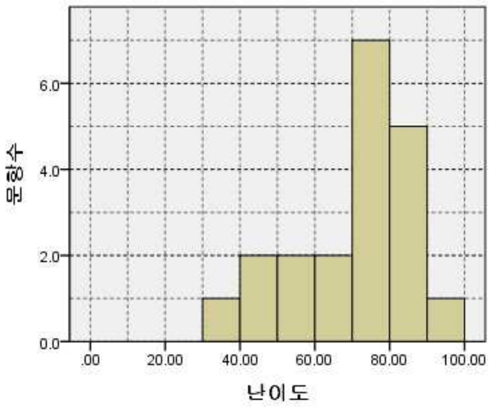

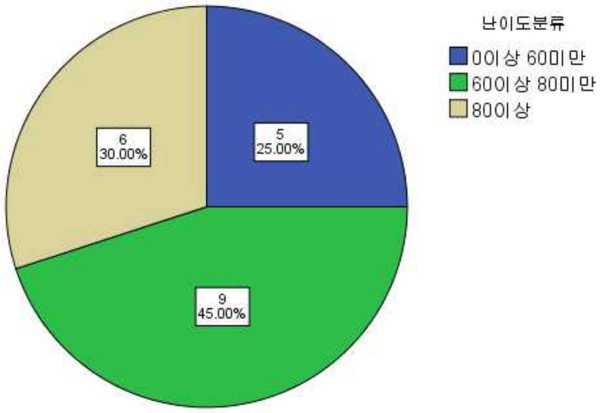

총점	난이도	표준편차	난이도	문항수	비율(%)
20	69.4	16.3	0~60미만	5	25.0
			60~80미만	9	45.0
			80~100	6	30.0
			전체	20	100.0

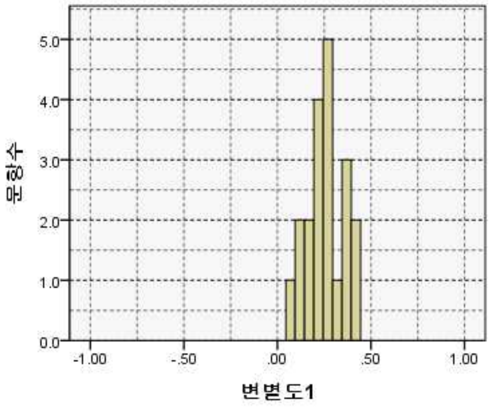

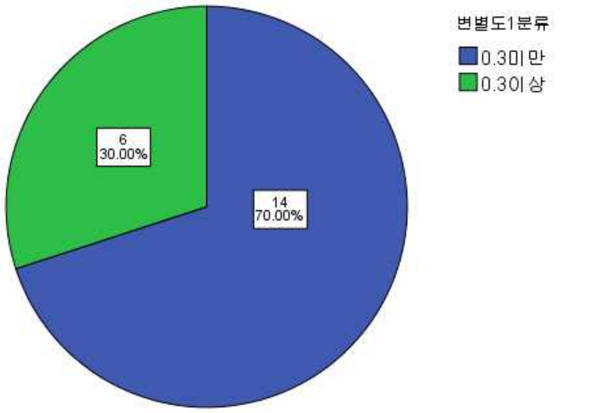

총점	변별도1	표준편차	변별도1	문항수	비율(%)
20	.25	.10	0.3미만	14	70.0
			0.3이상	6	30.0
			전체	20	100.0

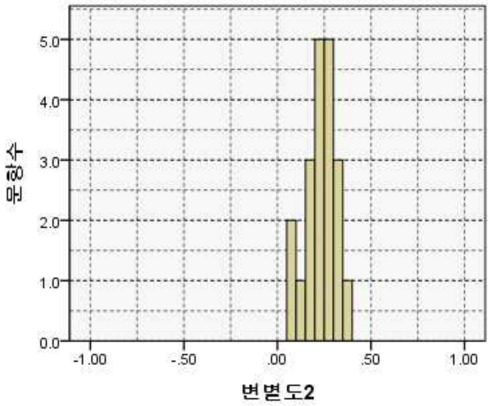

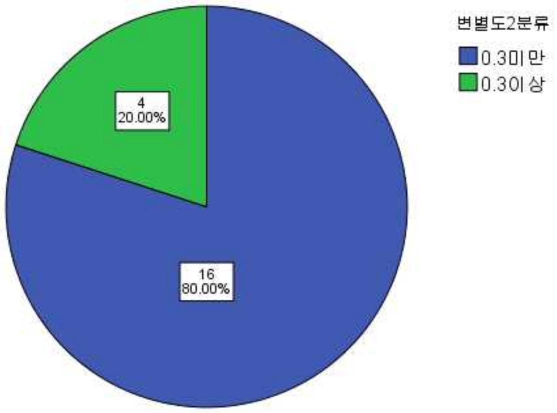

총점	변별도2	표준편차
20	.23	.08

변별도2	문항수	비율(%)
0.3미만	16	80.0
0.3이상	4	20.0
전체	20	100.0

해석

- 전문응급처치학각론 과목에서 난이도 지수가 80 에서 100 사이인 문항이 전체 20 문항 중 6 문항이었으며, 60 이상 80 미만인 문항이 9 문항, 60 미만인 문항이 5 문항으로 나타남
- 변별도 1 지수를 기준으로 분류하였을 때, 0.3 미만인 문항이 14 문항으로 0.3 이상인 문항이 6 문항인 것에 비해 더 많이 나타남
- 변별도 2 지수를 기준으로 분류하였을 때, 0.3 미만인 문항이 16 문항으로 0.3 이상인 문항이 4 문항인 것에 비해 더 많이 나타남

3) 지식수준별 난이도와 변별도

가) 전회 대비 지식수준별 난이도와 변별도

(1) 전회 대비 암기형 난이도와 변별도

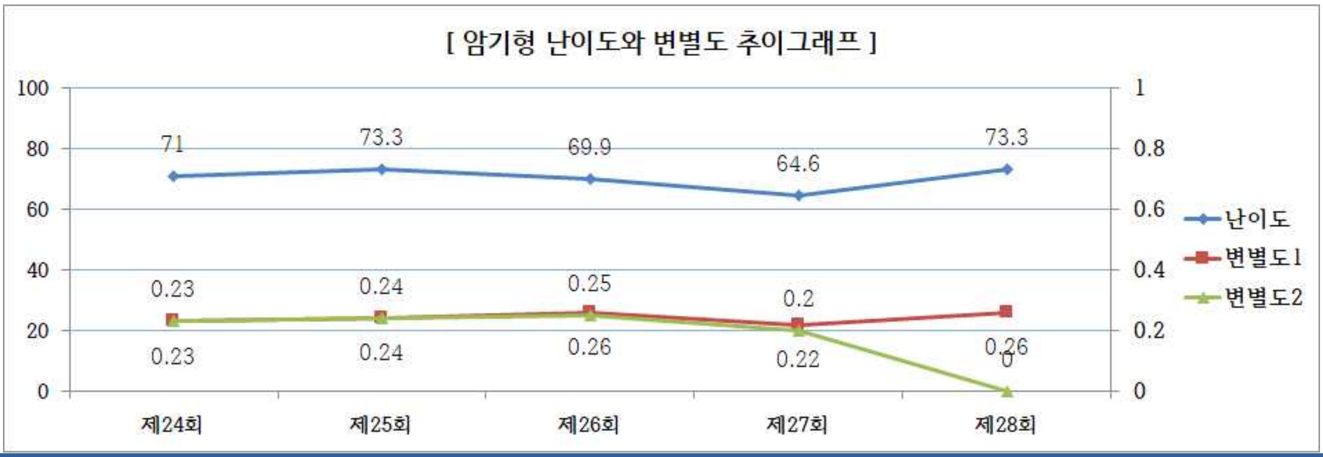

회차	난이도		변별도1		변별도2	
	평균	표준편차	평균	표준편차	평균	표준편차
제24회	71.0	21.0	.23	.13	.23	.10
제25회	73.3	18.3	.24	.12	.24	.09
제26회	69.9	20.6	.26	.13	.25	.09
제27회	64.6	23.8	.22	.11	.20	.09
제28회	73.3	18.9	.26	.13	.27	.10

(2) 전회 대비 해석형 난이도와 변별도

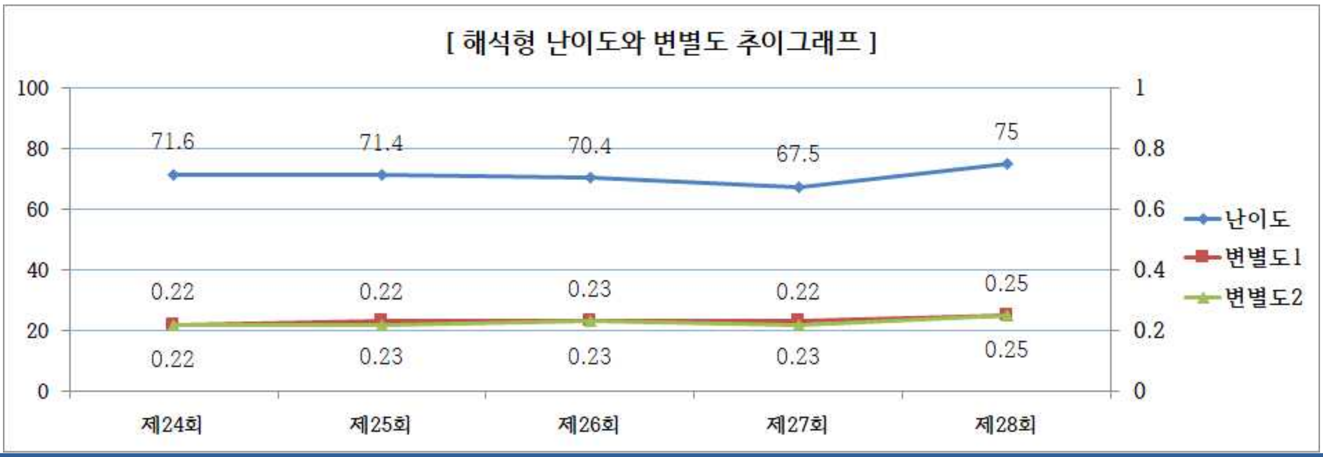

회차	난이도		변별도1		변별도2	
	평균	표준편차	평균	표준편차	평균	표준편차
제24회	71.6	19.1	.22	.11	.22	.09
제25회	71.4	18.1	.23	.12	.22	.11
제26회	70.4	20.7	.23	.15	.23	.11
제27회	67.5	23.0	.23	.13	.22	.11
제28회	75.0	18.4	.25	.13	.25	.10

(3) 전회 대비 해결형 난이도와 변별도

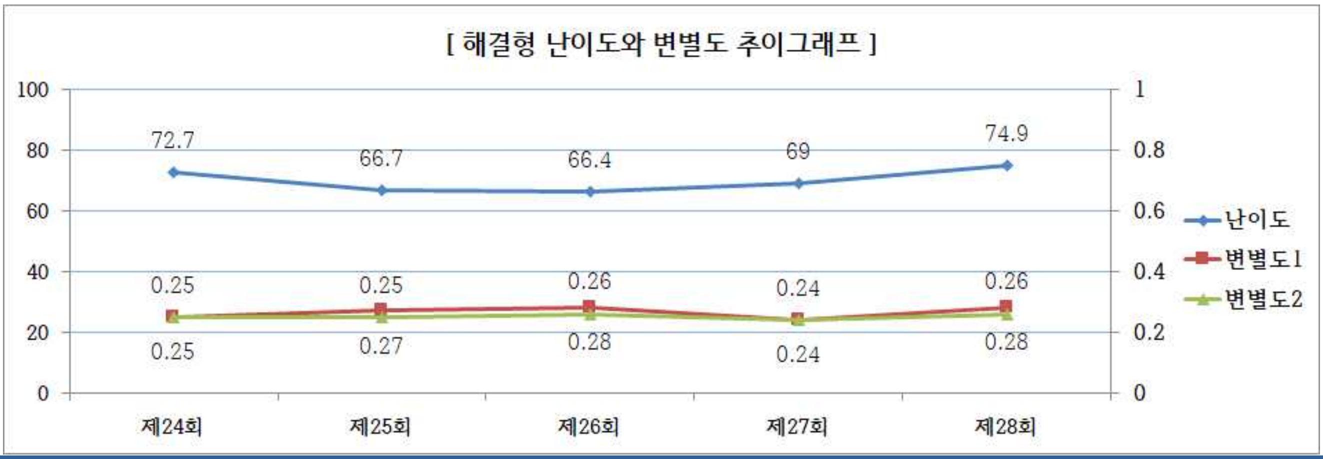

회차	난이도		변별도1		변별도2	
	평균	표준편차	평균	표준편차	평균	표준편차
제24회	72.7	17.3	.25	.11	.25	.10
제25회	66.7	20.2	.27	.11	.25	.10
제26회	66.4	19.7	.28	.11	.26	.08
제27회	69.0	21.2	.24	.11	.24	.09
제28회	74.9	18.5	.28	.13	.26	.12

해석

- 전회 대비 암기형 문항의 난이도 지수는 8.7 증가하였으며, 해석형 문항의 난이도 지수는 7.5 증가하였고, 해결형 문항의 난이도 지수는 5.9 증가함
- 변별도 1 지수는 암기형 문항에서 .04 증가하였으며, 해석형 문항에서 .02 증가하였고, 해결형 문항에서 .04 증가함
- 변별도 2 지수는 암기형 문항에서 .07 증가하였으며, 해석형 문항에서 .03 증가하였고, 해결형 문항에서 .02 증가함

나) 지식수준별 난이도와 변별도 분포도 및 비율분석

(1) 암기형 난이도와 변별도 분포도 및 비율분석

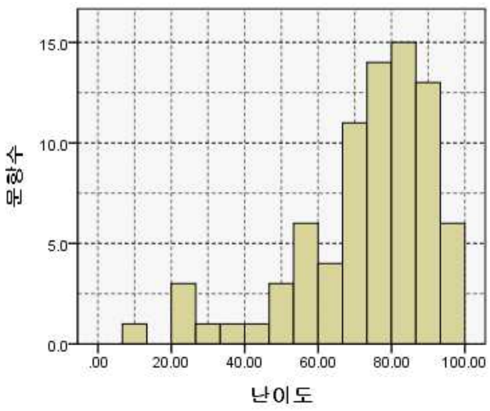

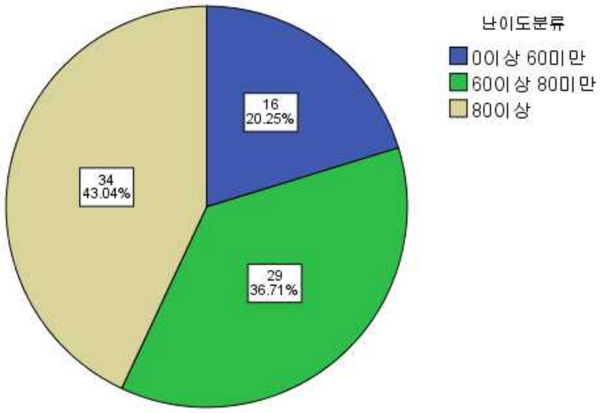

총점	난이도	표준편차
79	73.3	18.9

난이도	문항수	비율(%)
0~60미만	16	20.3
60~80미만	29	36.7
80~100	34	43.0
전체	79	100.0

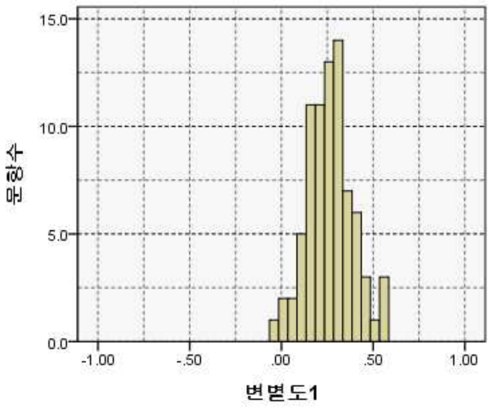

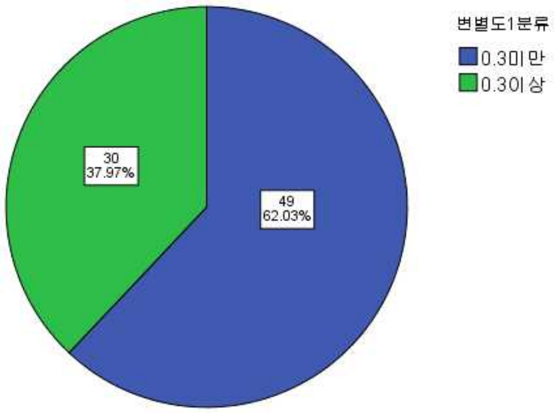

총점	변별도1	표준편차
79	.26	.13

변별도1	문항수	비율(%)
0.3미만	49	62.0
0.3이상	30	38.0
전체	79	100.0

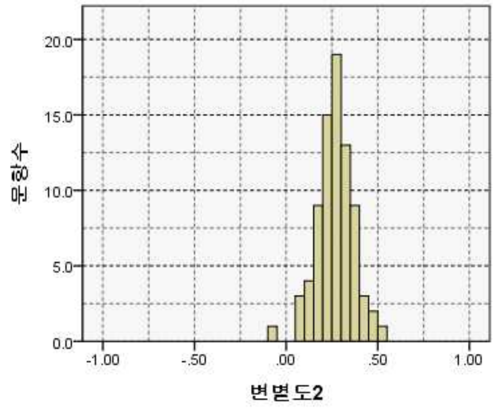

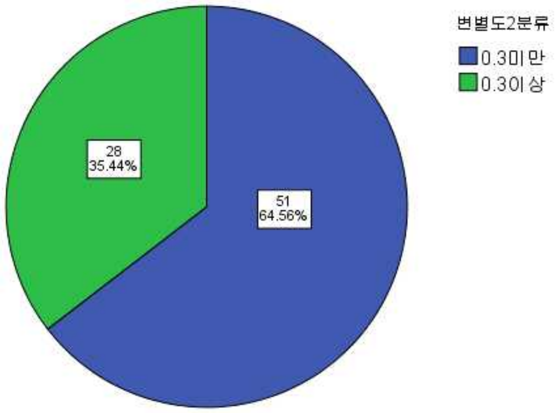

총점	변별도2	표준편차
79	.27	.10

변별도2	문항수	비율(%)
0.3미만	51	64.6
0.3이상	28	35.4
전체	79	100.0

해석

- 암기형 문항에서 난이도 지수가 80 에서 100 사이인 문항이 전체 79 문항 중 34 문항이었으며, 60 이상 80 미만인 문항이 29 문항, 60 미만인 문항이 16 문항인 것으로 나타남
- 변별도 1 지수를 기준으로 하였을 때, 0.3 미만인 문항이 49 문항으로 0.3 이상인 문항이 30 문항인 것에 비해 더 많이 나타남
- 변별도 2 지수를 기준으로 분류하였을 때, 0.3 미만인 문항이 51 문항으로 0.3 이상인 문항이 28 문항인 것에 비해 더 많이 나타남

(2) 해석형 난이도와 변별도 분포도 및 비율분석

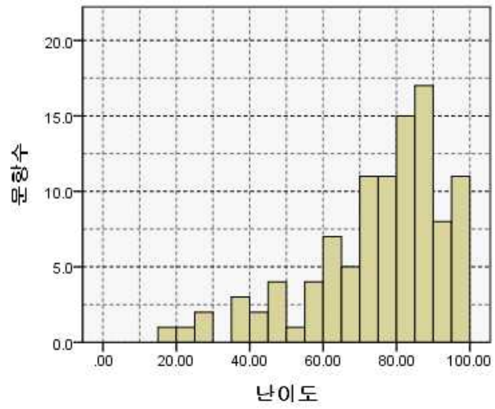

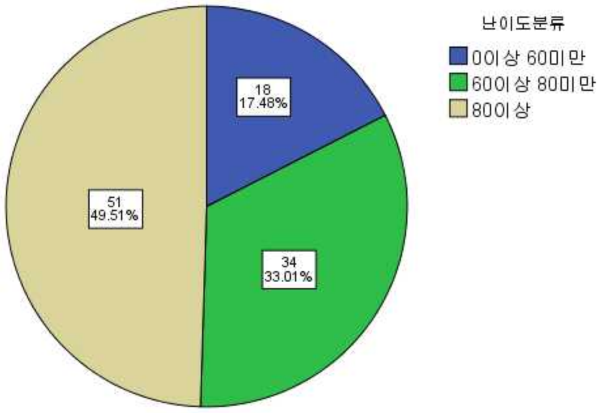

총점	난이도	표준편차	난이도	문항수	비율(%)
103	75.0	18.4	0~60미만	18	17.5
			60~80미만	34	33.0
			80~100	51	49.5
			전체	103	100.0

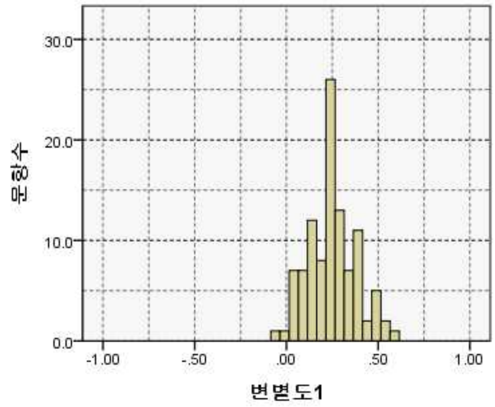

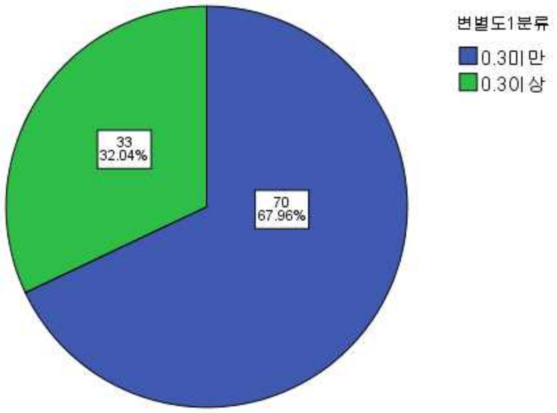

총점	변별도1	표준편차	변별도1	문항수	비율(%)
103	.25	.13	0.3미만	70	68.0
			0.3이상	33	32.0
			전체	103	100.0

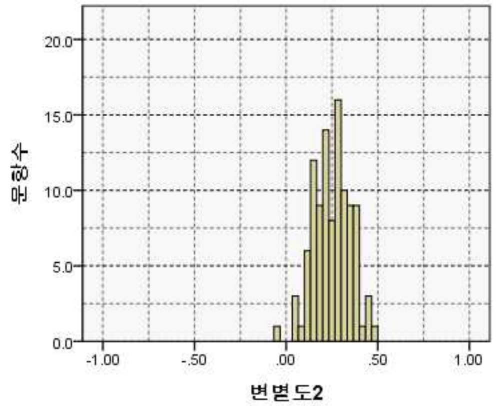

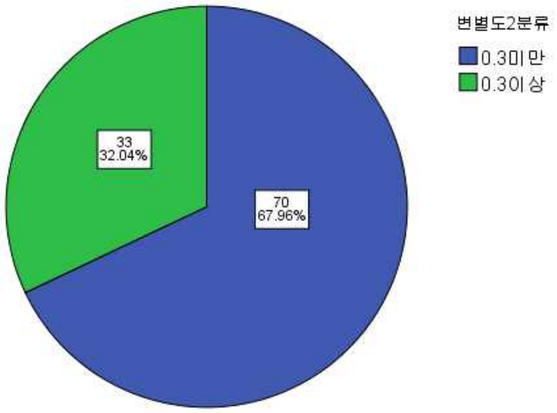

총점	변별도2	표준편차
103	.25	.10

변별도2	문항수	비율(%)
0.3미만	70	68.0
0.3이상	33	32.0
전체	103	100.0

해석

- 해석형 문항에서 난이도 지수가 80 에서 100 사이인 문항이 전체 103 문항 중 51 문항이었으며, 60 이상 80 미만인 문항이 34 문항, 60 미만인 문항이 18 문항인 것으로 나타남
- 변별도 1 지수를 기준으로 하였을 때, 0.3 미만인 문항이 70 문항으로 0.3 이상인 문항이 33 문항인 것에 비해 더 많이 나타남
- 변별도 2 지수를 기준으로 분류하였을 때, 0.3 미만인 문항이 70 문항으로 0.3 이상인 문항이 33 문항인 것에 비해 더 많이 나타남

(3) 해결형 난이도와 변별도 분포도 및 비율분석

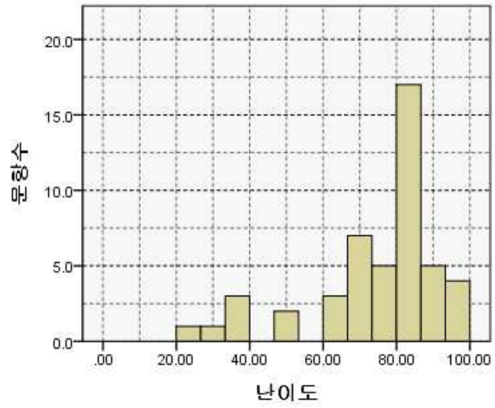

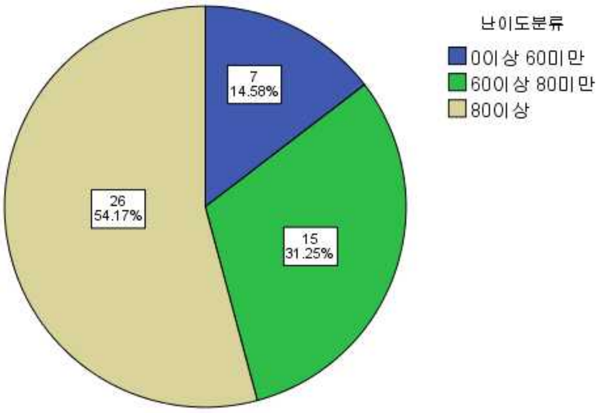

총점	난이도	표준편차
48	74.9	18.5

난이도	문항수	비율(%)
0~60미만	7	14.6
60~80미만	15	31.3
80~100	26	54.2
전체	48	100.0

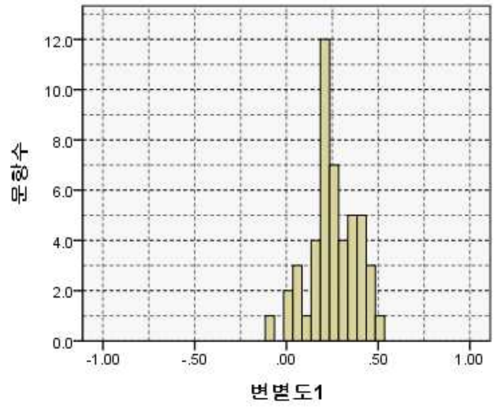

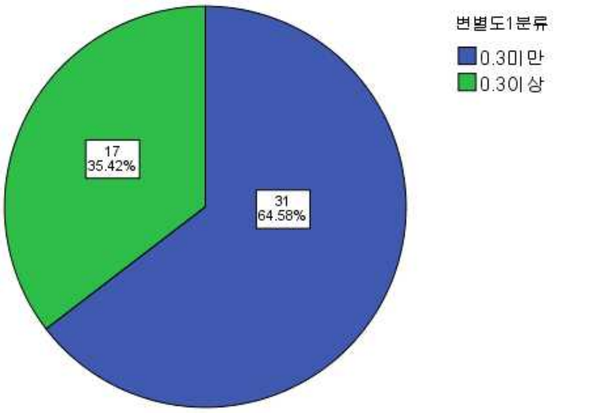

총점	변별도1	표준편차
48	.25	.13

변별도1	문항수	비율(%)
0.3미만	31	64.6
0.3이상	17	35.4
전체	48	100.0

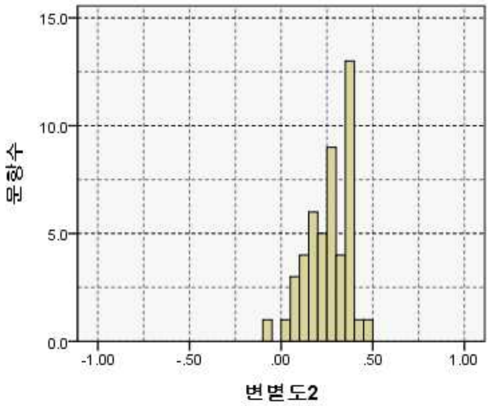

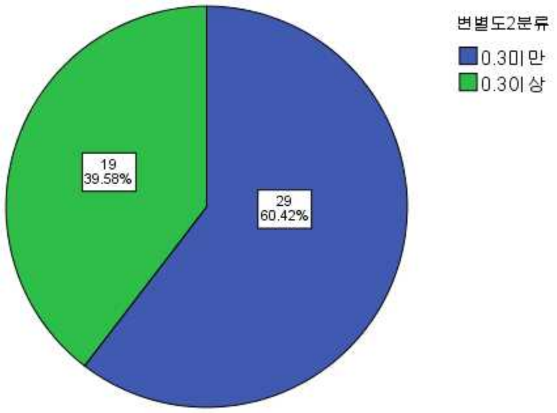

총점	변별도2	표준편차
48	.26	.12

변별도2	문항수	비율(%)
0.3미만	29	60.4
0.3이상	19	39.6
전체	48	100.0

해석

- 해결형 문항에서 난이도 지수가 80에서 100 사이인 문항이 전체 48문항 중 26문항이었으며, 60 이상 80 미만인 문항이 15문항, 60 미만인 문항이 7문항인 것으로 나타남
- 변별도 1 지수를 기준으로 하였을 때, 0.3 미만인 문항이 31문항으로 0.3 이상인 문항이 17문항인 것에 비해 더 많이 나타남
- 변별도 2 지수를 기준으로 분류하였을 때, 0.3 미만인 문항이 29문항으로 0.3 이상인 문항이 19문항인 것에 비해 더 많이 나타남

3. 난이도와 변별도 간 산포도

1) 전체 난이도와 변별도 간 산포도

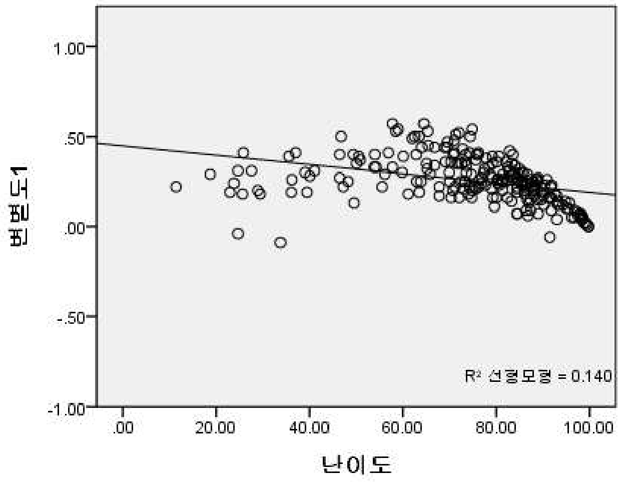

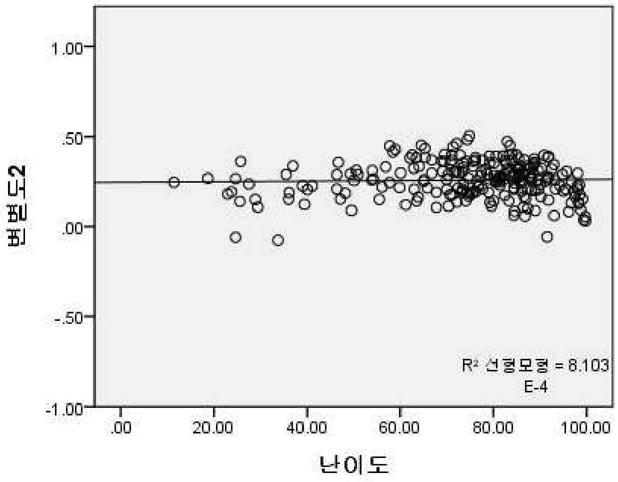

해석

- 난이도 지수와 변별도 1 지수 간 상관은 -.374*로 난이도 지수가 높을수록 변별력이 낮아지는 것으로 나타남
- 난이도 지수와 변별도 2 지수 간 상관은 .028 로 관련성이 없는 것으로 나타남

2) 과목별 난이도와 변별도 간 산포도

가) 기초의학 난이도와 변별도 간 산포도

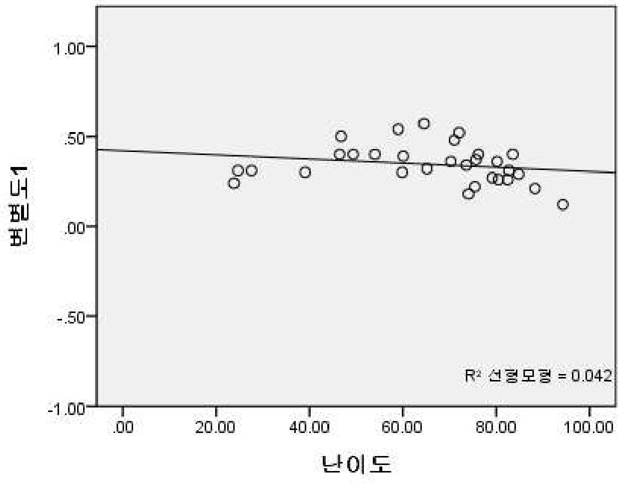

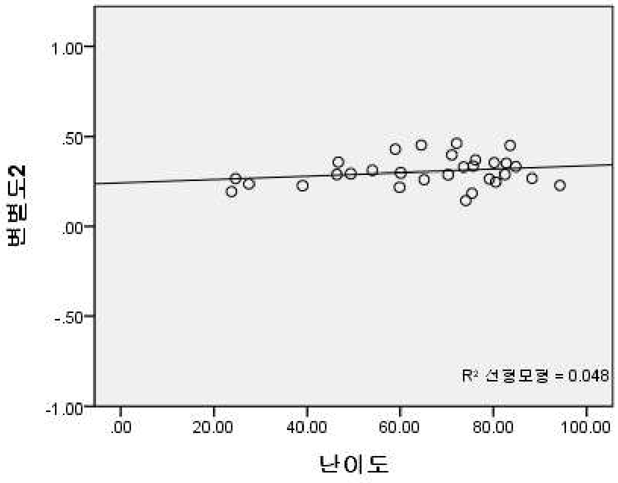

해석

- 난이도 지수와 변별도 1 지수 간 상관은 -.206 으로 관련성이 없는 것으로 나타남
- 난이도 지수와 변별도 2 지수 간 상관은 .219 로 관련성이 없는 것으로 나타남

나) 응급환자관리 난이도와 변별도 간 산포도

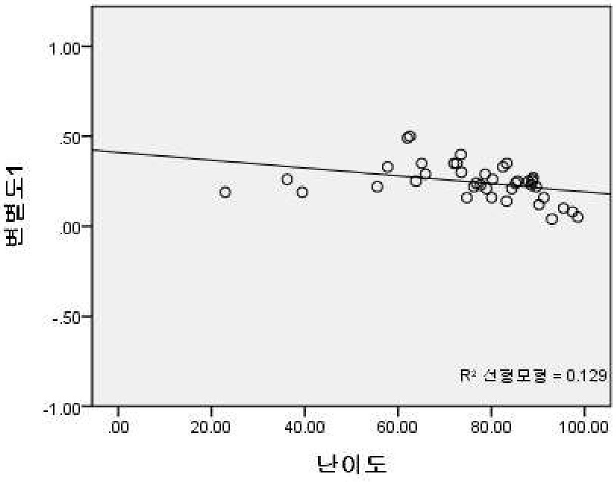

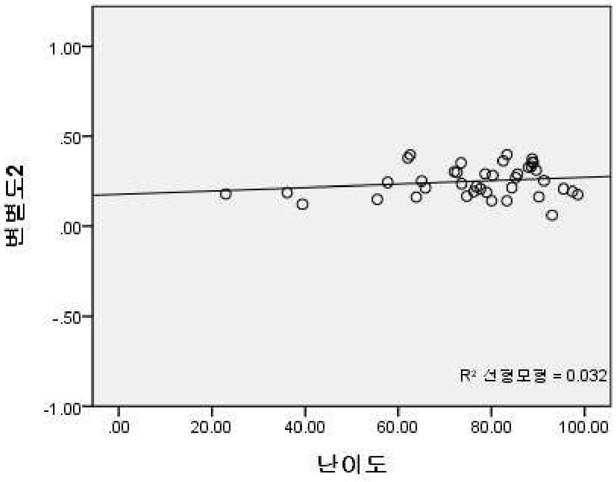

해석

- 난이도 지수와 변별도 1 지수 간 상관은 -.359*로 난이도 지수가 높을수록 변별력이 낮아지는 것으로 나타남
- 난이도 지수와 변별도 2 지수 간 상관은 -.180 으로 관련성이 없는 것으로 나타남

다) 전문응급처치학총론 난이도와 변별도 간 산포도

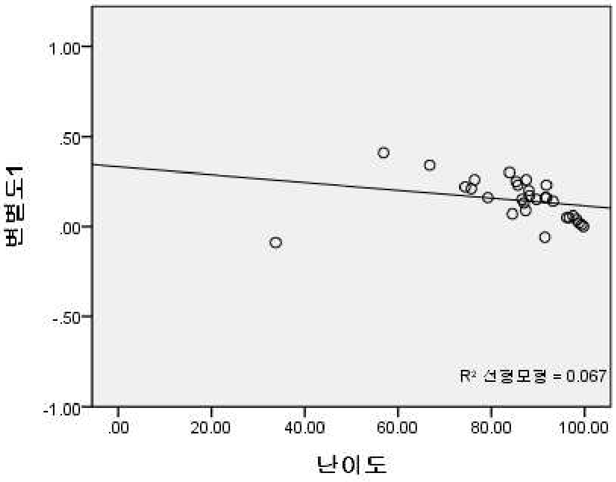

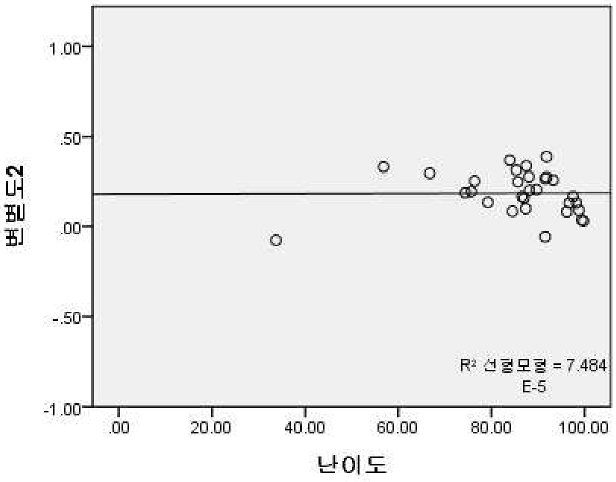

해석

- 난이도 지수와 변별도 1 지수 간 상관은 -.258 로 관련성이 없는 것으로 나타남
- 난이도 지수와 변별도 2 지수 간 상관은 .009 로 관련성이 없는 것으로 나타남

라) 전문응급처치학각론 난이도와 변별도 간 산포도

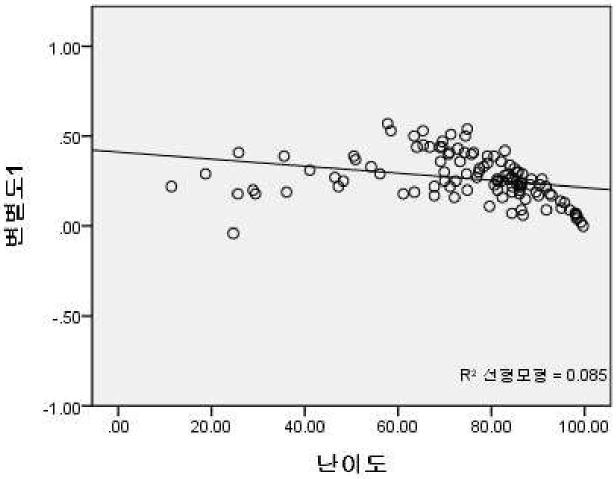

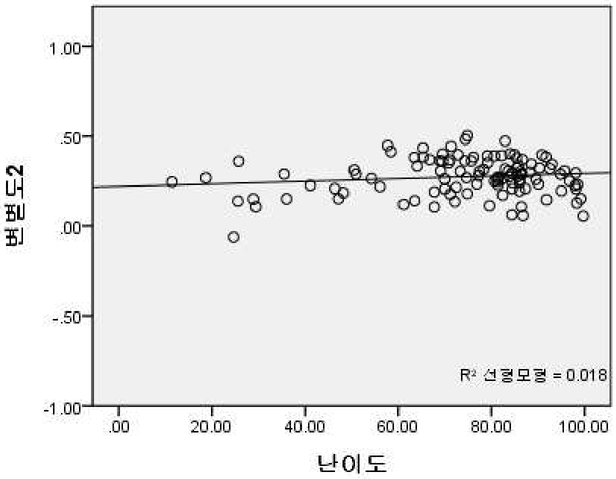

해석

- 난이도 지수와 변별도 1 지수 간 상관은 -.292*으로 난이도 지수가 높을수록 변별력이 낮아지는 것으로 나타남
- 난이도 지수와 변별도 2 지수 간 상관은 .134 로 관련성이 없는 것으로 나타남

마) 응급의료관련법령 난이도와 변별도 간 산포도

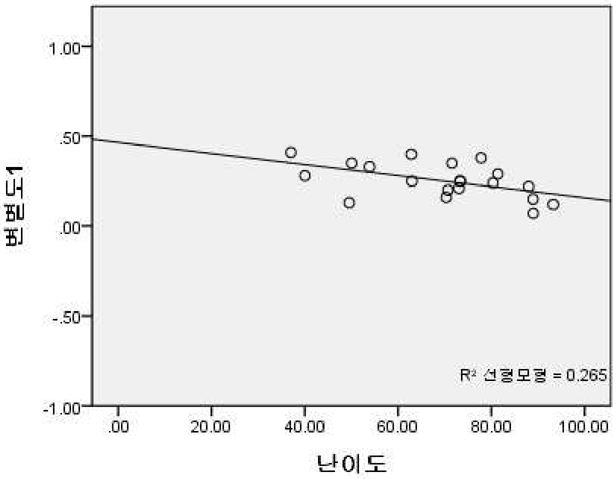

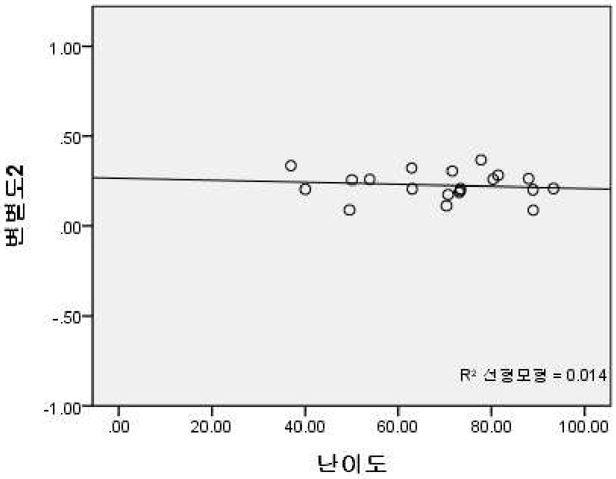

해석

- 난이도 지수와 변별도1 지수 간 상관은 -.515*로 난이도 지수가 높을수록 변별력이 낮아지는 것으로 나타남
- 난이도 지수와 변별도2 지수 간 상관은 -.117로 관련성이 없는 것으로 나타남

4. 신뢰도 분석

과목명	문항수	제24회	제25회	제26회	제27회	제28회
전체	230	.932	.934	.938	.926	.945
기초의학	30	.736	.688	.718	.644	.780
응급환자관리	40	.617	.701	.734	.684	.746
전문응급처치학총론	30	.527	.625	.558	.564	.586
전문응급처치학각론	110	.887	.879	.891	.876	.905
응급의료관련법령	20	.642	.691	.670	.583	.630

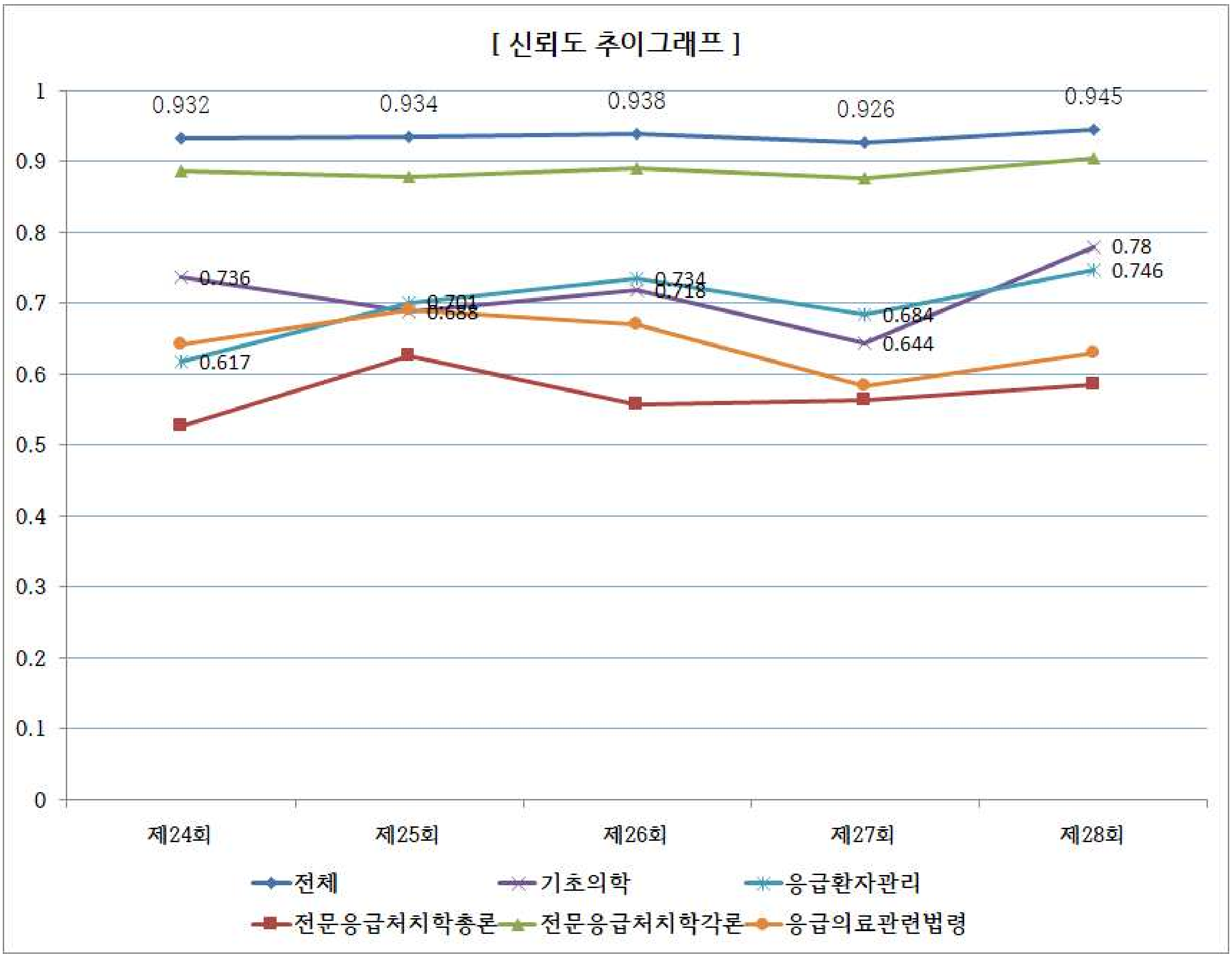

해석

- 전회 대비 전체문항의 신뢰도는 .019 증가함
- 전회 대비 기초의학과목 문항의 신뢰도는 .136 증가함
- 전회 대비 응급환자관리 과목 문항의 신뢰도는 .062 증가함
- 전회 대비 전문응급처치학총론 과목 문항의 신뢰도는 .022 증가함
- 전회 대비 전문응급처치학각론 과목 문항의 신뢰도는 .029 증가함
- 전회 대비 응급의료관련법령 과목 문항의 신뢰도는 .047 증가함

○ 분석결과 관련 문의 : 한국보건의료인국가시험원 연구개발본부 김준기 전임연구원
Tel : 02-2087-8956, FAX : 02-2087-8885
E-mail : tontates@kuksiwon.or.kr
