# Supplementary material for: Item difficulty index, discrimination index, and reliability of the 26 health professions licensing examinations in 2022, Korea: a psychometric study
Source: J Educ Eval Health Prof. 2023 Nov 22;20:31. doi: 10.3352/jeehp.2023.20.31 (PMC11959405; doi:10.3352/jeehp.2023.20.31)
Supplement: Supplementary file 1 — Supplement 1. Item analysis results of 26 health professions licensing examinations administered during late 2022 and early 2023. [file jeehp-20-31_Suppl1.zip › 2022│Γ╡╡ ┴a28╚╕ 2▒▐ └└▒▐▒╕┴╢╗τ ▒╣░í╜├╟Φ ║╨╝«░ß░·.pdf]

# 2022년도 제28회
# 2급 응급구조사 국가시험
# 문항분석 결과

국민이 신뢰하고 감동하는 시험평가기관
한국보건의료인국가시험원
KOREA HEALTH PERSONNEL LICENSING EXAMINATION INSTITUTE

## 일반 용어 정의

□ **평균**

- 집단에서의 대표적 경향값으로 전체 값을 더하여 총 응시자로 나눈 값

□ **표준편차**

- 평균과 각 점수의 차이인 편차들의 평균으로 점수가 흩어져 분포되어 있는 정도

□ **추정난이도**

- 문항개발자가 예측한 정답률

□ **검사이론**

- 검사와 검사를 구성하고 있는 문항의 양호도를 분석 및 평가하는 방법을 정의한 이론체계
- 대표적으로 고전검사이론과 문항반응이론이 있음

## 고전검사이론 용어 정의

□ **고전검사이론(Classical Test Theory; CTT)**

- 검사의 질을 분석하는 검사이론 중 한 가지로 19세기 말부터 전개되어 현재까지 주로 사용되고 있는 검사이론임
- 고전검사이론에 의한 문항과 응시자 능력 추정치는 다음과 같음

○ **문항난이도**

· 검사 문항의 쉽고 어려운 정도를 나타내는 지수
· 난이도 지수는 총 반응 수에 대한 정답 반응 수의 비율로 문항의 정답률임
· 문항난이도는 0~100까지의 값을 가짐
· 난이도 값이 큰 경우, 쉬운 문항으로 '난이도가 낮다'라고 해석하며, 난이도 값이 작은 경우, 어려운 문항으로 '난이도가 높다'라고 해석함

○ **문항변별도**

· 각 문항이 응시자의 능력 수준을 변별할 수 있는 정도를 나타내는 지수
· 문항변별도는 -1~+1까지의 값을 가지며, 1에 가까울수록 변별력 크다고 해석함
· 일반적으로 문항변별도가 0.3 이상이면 우수한 문항으로 평가함
· 구하는 방식에는 '상하위집단 구분법', '문항-총점 상관계수' 등이 있음
  1) **변별도 1(상하위구분법):** 상위 27%와 하위 27% 집단의 난이도 차이를 구하는 방식
  2) **변별도 2(상관계수법):** 문항-총점과의 상관계수로 구하는 방식

○ **신뢰도**

· 시험이 평가하고자 하는 것을 일관성 있게 측정하는가로 시험이 오차없이 정확하게 측정한 정도를 의미함
· 국시원에서는 문항의 내적일관성(Cronbach $\alpha$)으로 신뢰도를 추정하며 1에 가까울수록 신뢰도가 높다고 해석함

# 목 차

I. 시행 결과 ···· 6
1. 시험 현황 ···· 7
1) 시험명 ···· 7
2) 시험시행일 ···· 7
3) 응시현황 ···· 7
4) 과목별 문항 수, 배점 및 과락 점수 ···· 7
2. 합격률과 평균성적 ···· 7
1) 합격 및 불합격 현황 ···· 7
2) 과목별 과락자수 내역 ···· 8
3) 전회 대비 합격률과 평균성적 ···· 8

II. 문항분석 결과 ···· 9
1. 성적 ···· 10
1) 전체 성적분포도 ···· 10
2) 과목별 성적분포도 ···· 11
2. 난이도와 변별도 ···· 13
1) 전체 난이도와 변별도 ···· 13
2) 과목별 난이도와 변별도 ···· 16
3) 지식수준별 난이도와 변별도 ···· 31
3. 난이도와 변별도 간 산포도 ···· 39
1) 전체 난이도와 변별도 간 산포도 ···· 39
2) 과목별 난이도와 변별도 간 산포도 ···· 39
4. 신뢰도 분석 ···· 43

# I. 시행 결과

## 1. 시험 현황

1) 시험명: 2022도 제28회 2급 응급구조사 국가시험

2) 시험시행일: 2022년 12월 26일

3) 응시현황

| 구분 | 응시대상자수 | 결시자수 | 부정행위자수 | 응시자 준수사항 위반자 수 | | 응시자수 (%) |
|---|---|---|---|---|---|---|
| | | | | 휴대폰 소지 | 신분증 미지참 | |
| 실기 | 1,188 | 37 | 0 | 1 | 0 | 1,150 (96.8) |
| 필기 | 1,188 | 107 | 0 | 0 | 0 | **1,079 (90.8) |
| 종합 | 1,188 | *37 | 0 | 1 | 0 | 1,148 (96.6) |

*실기•필기 모두 결시
**채점보류자 제외

4) 과목별 문항 수, 배점 및 과락 점수

| 교 시 | 과 목 | 문제 수 | 배점 | 총점 | 합격자 점수기준 | |
|---|---|---|---|---|---|---|
| | | | | | 과목 과락기준 | 총점 합격기준 |
| 1교시 | 기본응급처치학총론 | 20 | 1 | 20 | 8점 미만 | 120점 이상 |
| 1교시 | 기본응급환자관리 | 20 | 1 | 20 | 8점 미만 | |
| 1교시 | 응급의료관련법령 | 20 | 1 | 20 | 8점 미만 | |
| 2교시 | 기본응급처치학각론 | 60 | 1 | 60 | 24점 미만 | |
| 2교시 | 응급의료장비 | 20 | 1 | 20 | 8점 미만 | |
| | 실기시험 | 3 | 20 | 60 | 36점 미만 | |
| 계 | | 143 | | 200 | | |

## 2. 합격률과 평균성적

1) 합격 및 불합격 현황

| 구분 | 합격자수 (%) | 불합격자수(%) | | | | 채점보류자수 |
|---|---|---|---|---|---|---|
| | | 평락 | 과락 | 기권 | 계 | |
| 실기 | 1,094 (95.1) | 0 (0.0) | 56 (4.9) | 0 (0.0) | 56 (4.9) | - |
| 필기 | 966 (89.5) | 90 (8.3) | 22 (2.0) | 1 (0.1) | 113 (10.5) | 2 |
| 종합 | 966 (84.1) | 159 (13.9) | 22※ (1.9) | 1 (0.1) | 182 (15.9) | 2 |

※최종결과는 합격자 결정 기준에 따라 필기점수와 실기점수를 합산

2) 과목별 과락자수 내역

| 과목명 / 과락자수 | 기본응급 처치학총론 | 기본응급 환자관리 | 응급의료 관련법령 | 기본응급 처치학각론 | 응급의료장비 | 실기시험 |
|---|---|---|---|---|---|---|
| 과목별 과락자 수 | 1 | 1 | 7 | 1 | 11 | 1 |
| 전과목 과락자 수 | 22 | | | | | |

3) 전회 대비 합격률과 평균성적

| 회차 | 년도 | 합격률(%) | 평균성적 | 표준편차 | 백분율 환산점수 |
|---|---|---|---|---|---|
| 제24회 | 2018 | 83.1 | 145.1 | 15.9 | 72.6 |
| 제25회 | 2019 | 89.4 | 148.2 | 14.7 | 74.1 |
| 제26회 | 2020 | 88.0 | 146.7 | 14.4 | 73.4 |
| 제27회 | 2021 | 75.8 | 138.4 | 16.6 | 69.2 |
| 제28회 | 2022 | 84.1 | 142.0 | 17.0 | 71.0 |

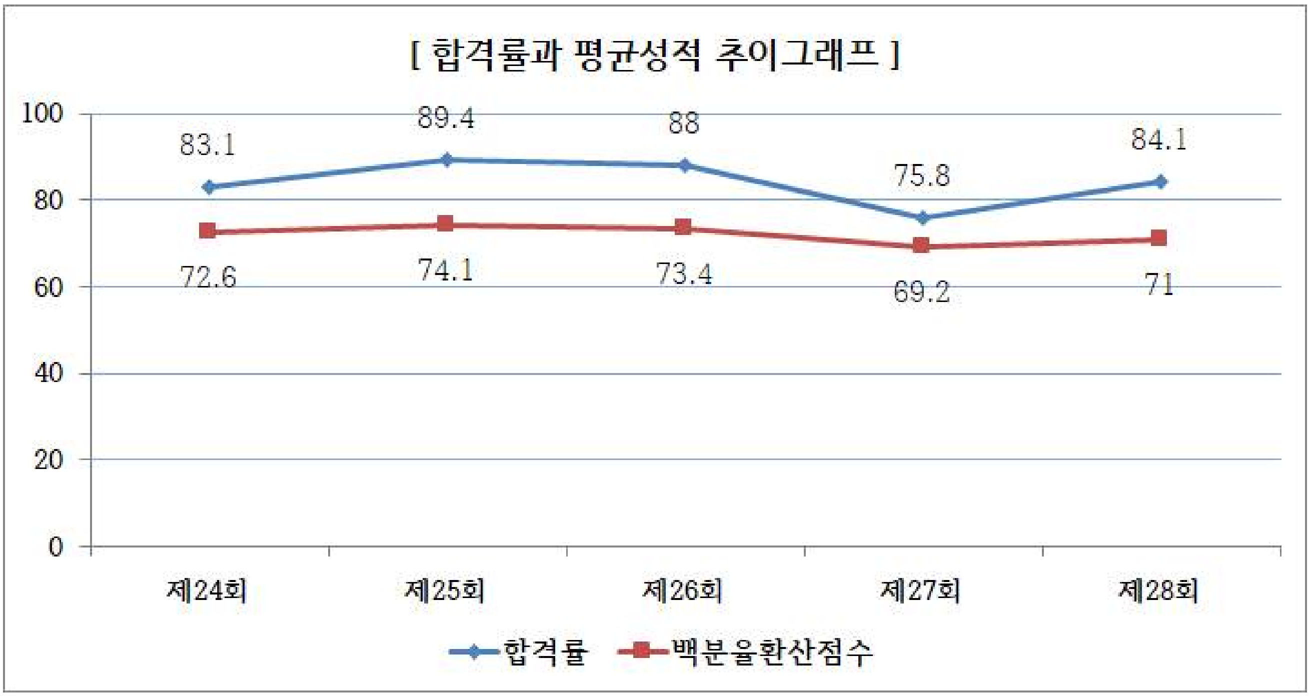


**해석**

- 전년 대비 합격률은 8.3% 증가하고, 백분율 환산점수는 1.8 점 증가함

# II. 문항분석 결과

## 1. 성적

1) 전체 성적분포도

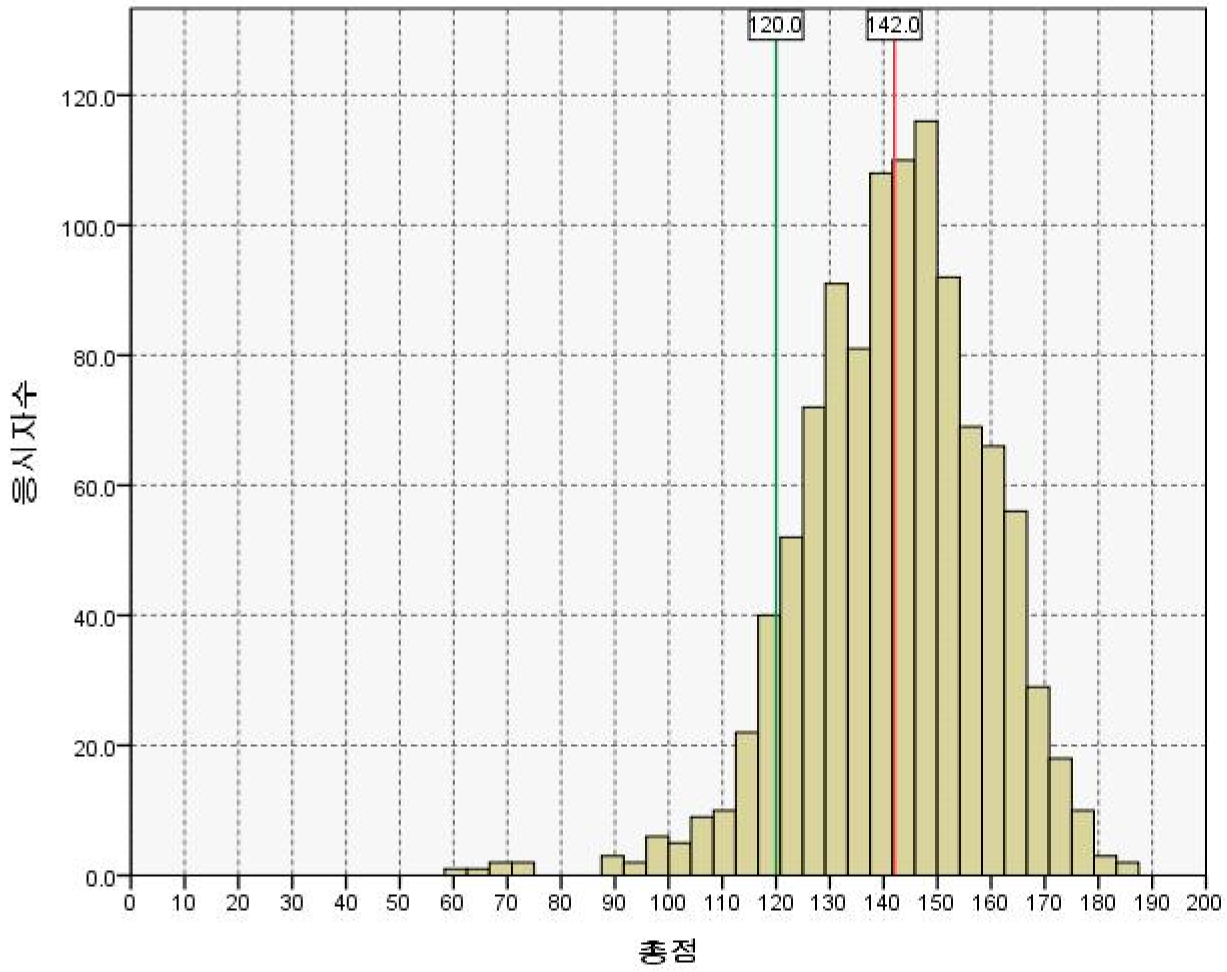


| 응시자 | 총점 | 합격선 | 평균성적 | 표준편차 |
|---|---|---|---|---|
| 1079 | 200 | 120 | 142.0 | 17.0 |

2) 과목별 성적분포도

가) 기본응급처치학총론

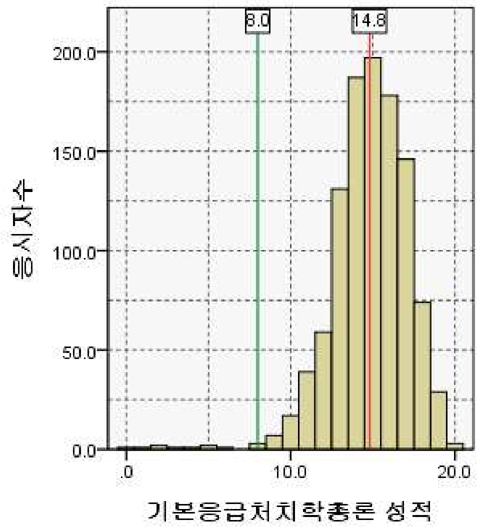


| 총점 | 과락선 | 평균성적 | 표준편차 |
|---|---|---|---|
| 20 | 8 | 14.8 | 2.3 |

나) 기본응급환자관리

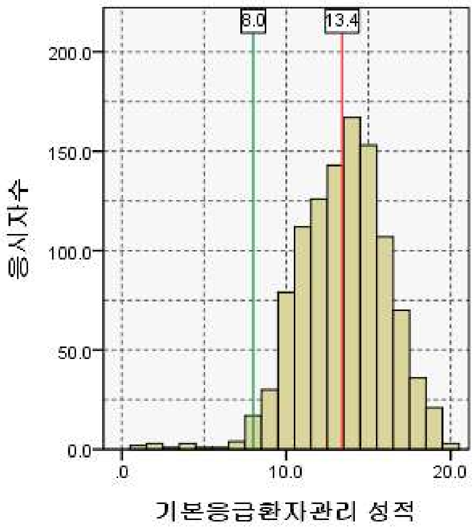


| 총점 | 과락선 | 평균성적 | 표준편차 |
|---|---|---|---|
| 20 | 8 | 13.4 | 2.7 |

다) 응급의료관련법령

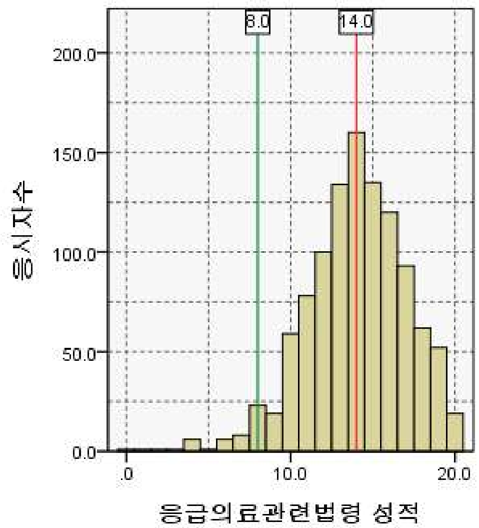


| 총점 | 과락선 | 평균성적 | 표준편차 |
|---|---|---|---|
| 20 | 8 | 14.0 | 3.0 |

라) 기본응급처치학각론

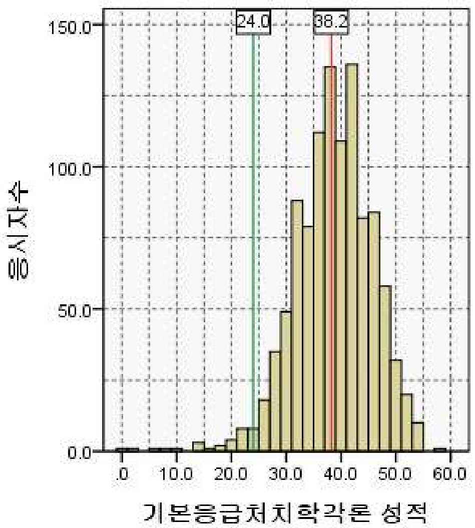


| 총점 | 과락선 | 평균성적 | 표준편차 |
|---|---|---|---|
| 60 | 24 | 38.2 | 7.1 |

마) 응급의료장비

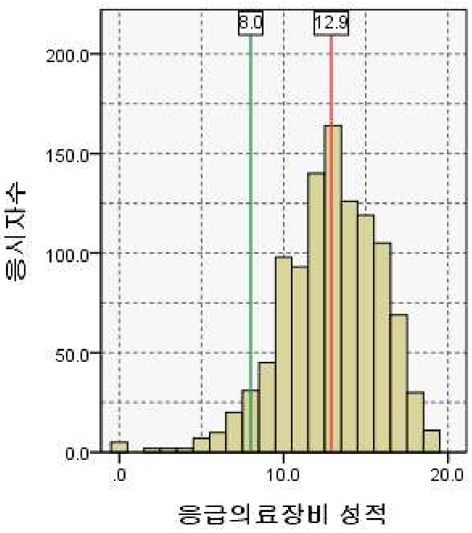


| 총점 | 과락선 | 평균성적 | 표준편차 |
|---|---|---|---|
| 20 | 8 | 12.9 | 3.0 |

## 2. 난이도와 변별도

### 1) 전체 난이도와 변별도

#### 가) 전회 대비 전체 난이도와 변별도

| 회차 | 난이도 | | 변별도1 | | 변별도2 | |
|---|---|---|---|---|---|---|
| | 평균 | 표준편차 | 평균 | 표준편차 | 평균 | 표준편차 |
| 제24회 | 68.8 | 20.0 | .22 | .12 | .20 | .09 |
| 제25회 | 70.3 | 19.8 | .20 | .10 | .18 | .09 |
| 제26회 | 70.0 | 21.4 | .19 | .12 | .19 | .10 |
| 제27회 | 64.4 | 22.5 | .23 | .14 | .21 | .10 |
| 제28회 | 66.7 | 21.4 | .24 | .13 | .23 | .10 |

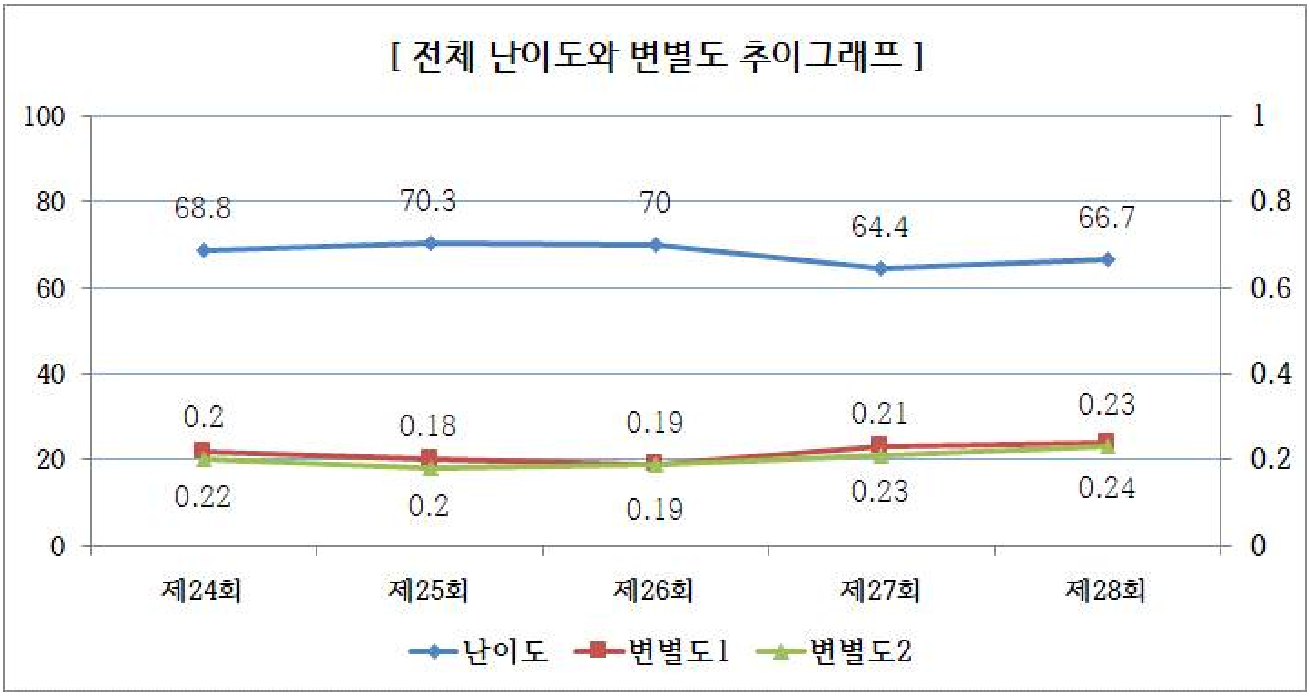


**해석**

- 전년 대비 난이도 지수는 2.3 증가함
- 변별도 1 지수는 .01 증가함
- 변별도 2 지수는 .02 증가함

## 나) 전체 난이도와 변별도 분포도 및 비율분석

### (1) 전체 난이도 분포도 및 비율분석

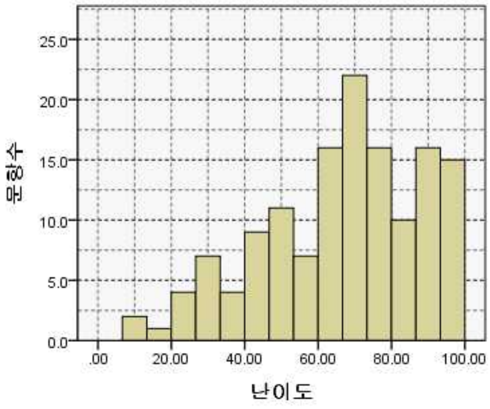


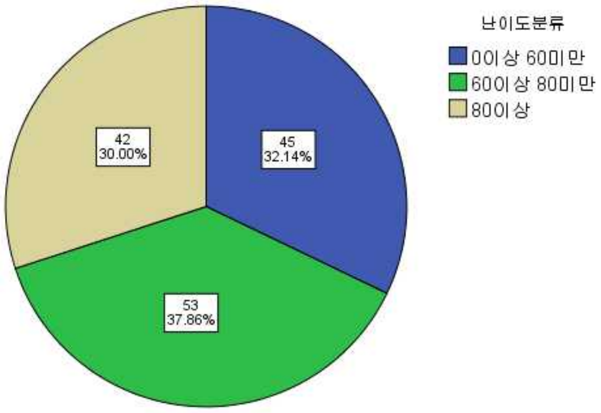


| 총점 | 난이도 | 표준편차 |
|---|---|---|
| 140 | 66.7 | 21.4 |

| 난이도 | 문항수 | 비율(%) |
|---|---|---|
| 0~60미만 | 45 | 32.1 |
| 60~80미만 | 53 | 37.9 |
| 80~100 | 42 | 30.0 |
| 전체 | 140 | 100.0 |

### (2) 전체 변별도1 분포도 및 비율분석

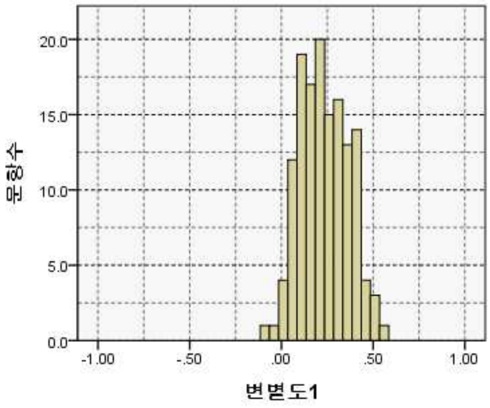


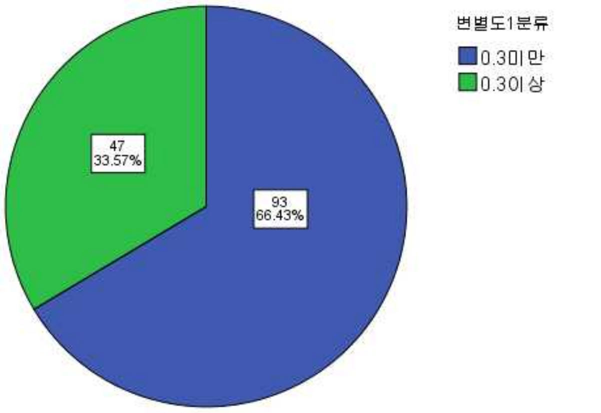


| 총점 | 변별도1 | 표준편차 |
|---|---|---|
| 140 | .24 | .13 |

| 변별도1 | 문항수 | 비율(%) |
|---|---|---|
| 0.3미만 | 93 | 66.4 |
| 0.3이상 | 47 | 33.6 |
| 전체 | 140 | 100.0 |

(3) 전체 변별도2 분포도 및 비율분석

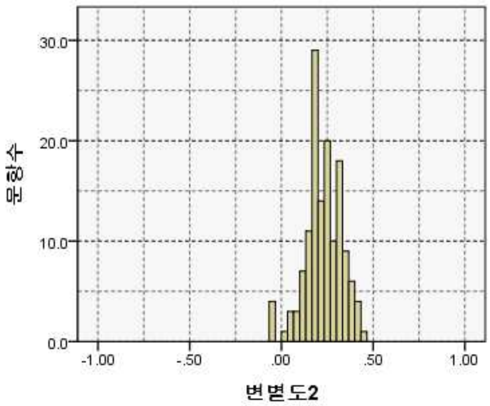


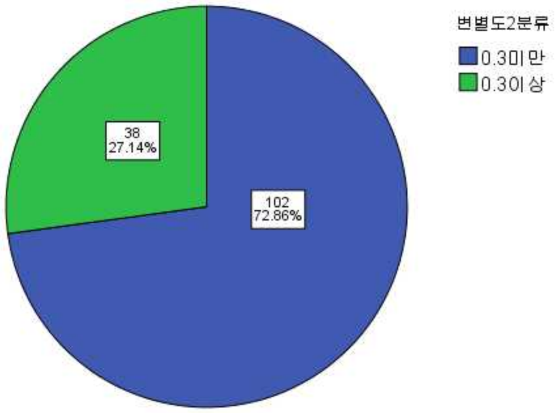


| 총점 | 변별도2 | 표준편차 |
|---|---|---|
| 140 | .23 | .10 |

| 변별도2 | 문항수 | 비율(%) |
|---|---|---|
| 0.3미만 | 102 | 72.9 |
| 0.3이상 | 38 | 27.1 |
| 전체 | 140 | 100.0 |

**해석**

- 난이도 지수가 80 에서 100 사이인 문항이 전체 140 문항 중 42 문항이었으며, 60 이상 80 미만인 문항이 53 문항, 60 미만인 문항이 45 문항인 것으로 나타남
- 변별도 1 지수를 기준으로 분류하였을 때, 0.3 미만인 문항이 93 문항으로 0.3 이상인 문항이 47 문항인 것에 비해 더 많이 나타남
- 변별도 2 지수를 기준으로 분류하였을 때, 0.3 미만인 문항이 102 문항으로 0.3 이상인 문항이 38 문항인 것에 비해 더 많이 나타남

2) 과목별 난이도와 변별도

가) 전회 대비 과목별 난이도와 변별도

(1) 전회 대비 기본응급처치학총론 난이도와 변별도

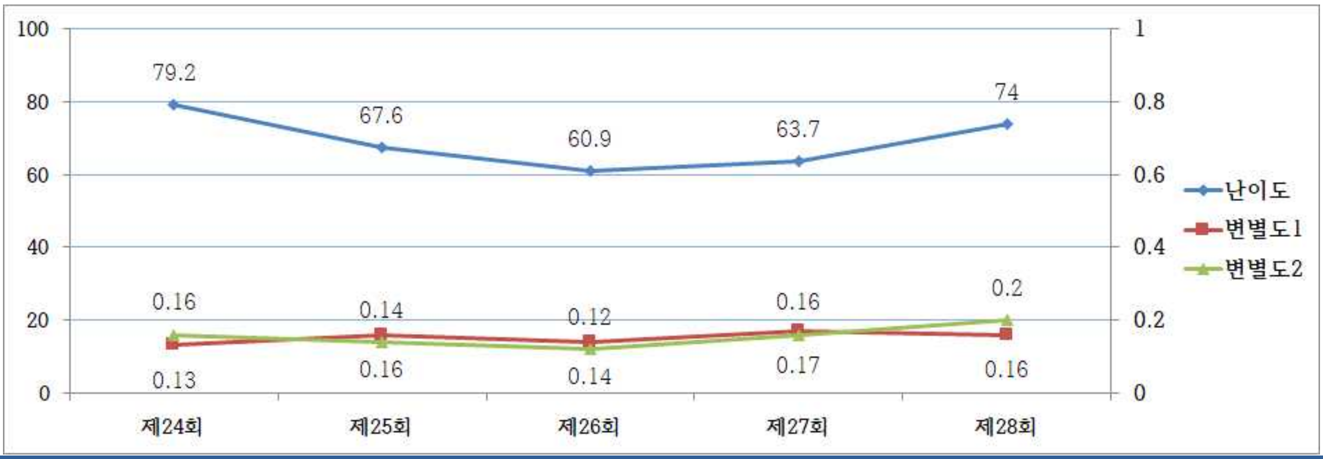


| 회차 | 난이도 | | 변별도1 | | 변별도2 | |
|---|---|---|---|---|---|---|
| | 평균 | 표준편차 | 평균 | 표준편차 | 평균 | 표준편차 |
| 제24회 | 79.2 | 21.3 | .13 | .09 | .16 | .07 |
| 제25회 | 67.6 | 23.7 | .16 | .09 | .14 | .07 |
| 제26회 | 60.9 | 26.3 | .14 | .09 | .12 | .08 |
| 제27회 | 63.7 | 31.0 | .17 | .13 | .16 | .10 |
| 제28회 | 74.0 | 24.5 | .16 | .12 | .20 | .07 |

**해석**

- 전회 대비 기본응급처치학총론 과목의 난이도 지수는 10.3 증가함
- 전회 대비 기본응급처치학총론 과목의 변별도 1 지수는 .01 감소함
- 전회 대비 기본응급처치학총론 과목의 변별도 2 지수는 .04 증가함

(2) 전회 대비 기본응급환자관리 난이도와 변별도

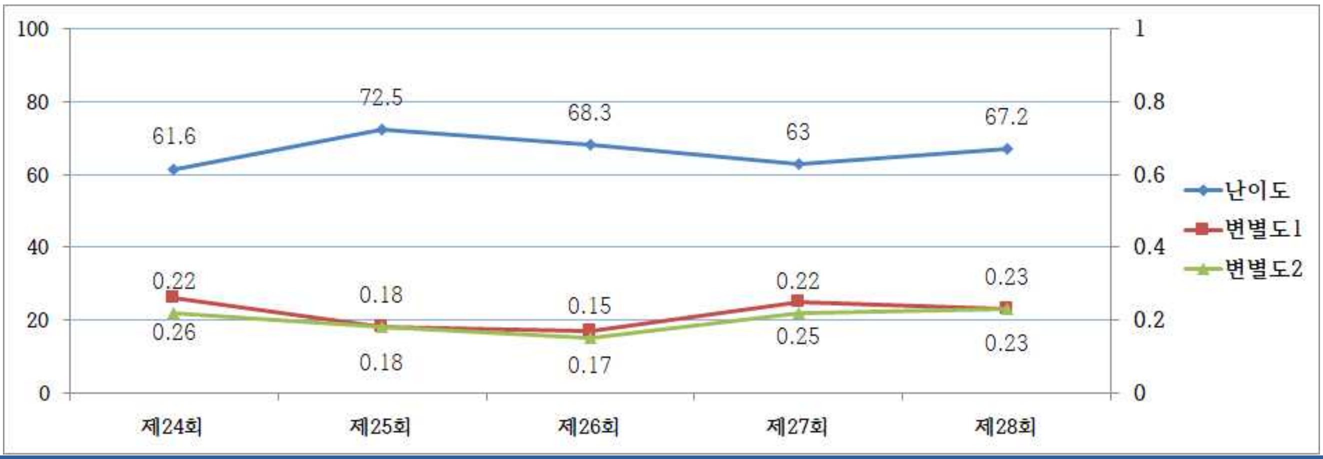


| 회차 | 난이도 | | 변별도1 | | 변별도2 | |
|---|---|---|---|---|---|---|
| | 평균 | 표준편차 | 평균 | 표준편차 | 평균 | 표준편차 |
| 제24회 | 61.6 | 21.6 | .26 | .12 | .22 | .10 |
| 제25회 | 72.5 | 20.6 | .18 | .09 | .18 | .09 |
| 제26회 | 68.3 | 24.9 | .17 | .11 | .15 | .08 |
| 제27회 | 63.0 | 21.8 | .25 | .14 | .22 | .09 |
| 제28회 | 67.2 | 23.0 | .23 | .11 | .23 | .07 |

**해석**

- 전회 대비 기본응급환자관리 과목의 난이도 지수는 4.2 증가함
- 전회 대비 기본응급환자관리 과목의 변별도 1 지수는 .02 감소함
- 전회 대비 기본응급환자관리 과목의 변별도 2 지수는 .01 증가함

(3) 전회 대비 응급의료관련법령 난이도와 변별도

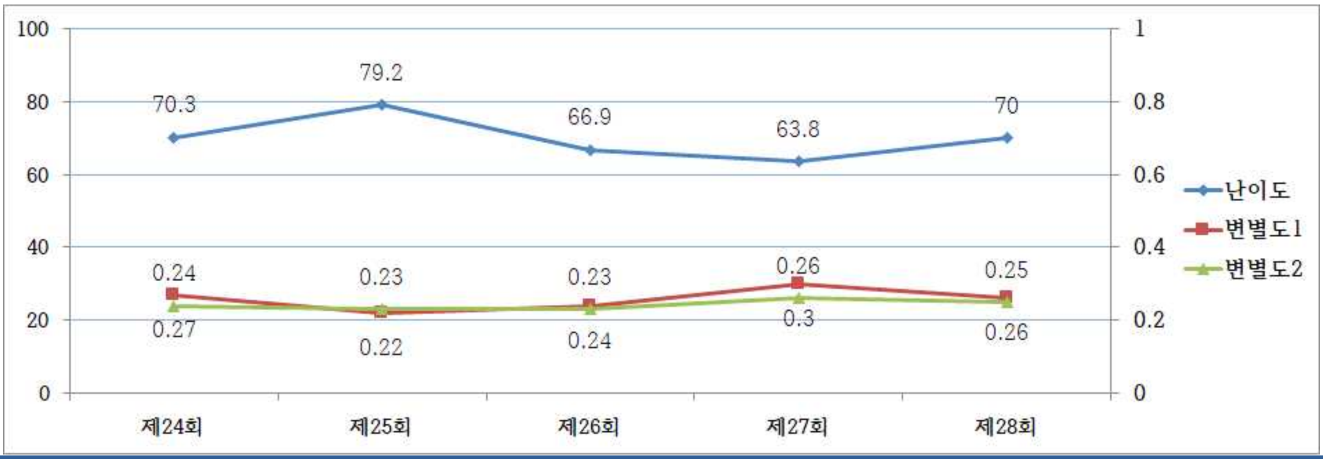


| 회차 | 난이도 | | 변별도1 | | 변별도2 | |
|---|---|---|---|---|---|---|
| | 평균 | 표준편차 | 평균 | 표준편차 | 평균 | 표준편차 |
| 제24회 | 70.3 | 17.9 | .27 | .10 | .24 | .06 |
| 제25회 | 79.2 | 13.0 | .22 | .11 | .23 | .09 |
| 제26회 | 66.9 | 22.1 | .24 | .11 | .23 | .06 |
| 제27회 | 63.8 | 18.1 | .30 | .10 | .26 | .06 |
| 제28회 | 70.0 | 18.1 | .26 | .10 | .25 | .06 |

**해석**

- 전회 대비 응급의료관련법령 과목의 난이도 지수는 6.2 증가함
- 전회 대비 응급의료관련법령 과목의 변별도 1 지수는 .04 감소함
- 전회 대비 응급의료관련법령 과목의 변별도 2 지수는 .01 감소함

(4) 전회 대비 기본응급처치학각론 난이도와 변별도

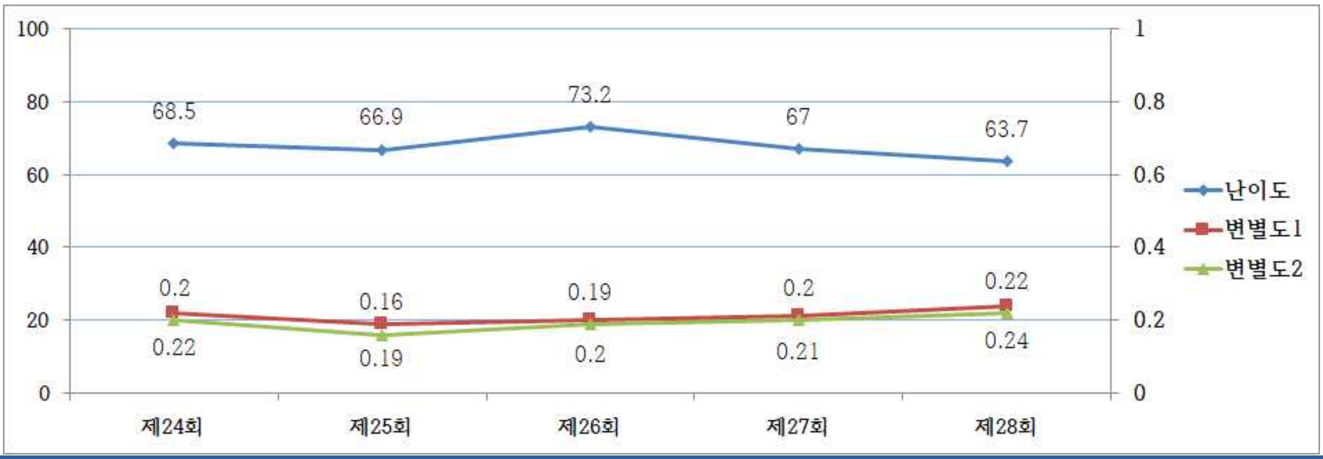


| 회차 | 난이도 | | 변별도1 | | 변별도2 | |
|---|---|---|---|---|---|---|
| | 평균 | 표준편차 | 평균 | 표준편차 | 평균 | 표준편차 |
| 제24회 | 68.5 | 19.8 | .22 | .12 | .20 | .10 |
| 제25회 | 66.9 | 21.3 | .19 | .11 | .16 | .08 |
| 제26회 | 73.2 | 18.0 | .20 | .14 | .19 | .11 |
| 제27회 | 67.0 | 20.7 | .21 | .15 | .20 | .11 |
| 제28회 | 63.7 | 20.6 | .24 | .14 | .22 | .12 |

**해석**

- 전회 대비 기본응급처치학각론 과목의 난이도 지수는 3.3 감소함
- 전회 대비 기본응급처치학각론 과목의 변별도 1 지수는 .03 증가함
- 전회 대비 기본응급처치학각론 과목의 변별도 2 지수는 .02 증가함

(5) 전회 대비 응급의료장비 난이도와 변별도

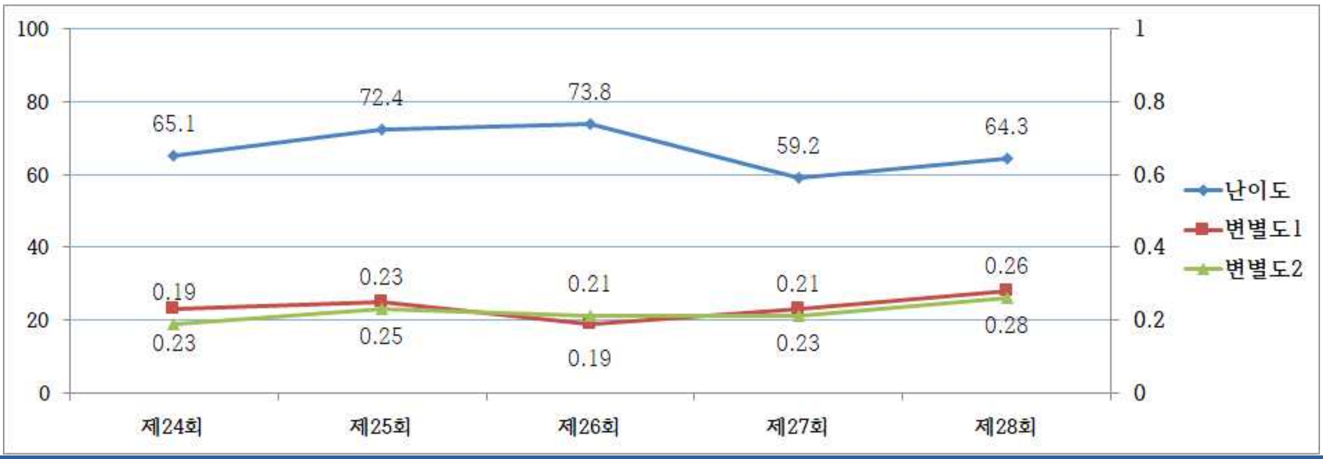


| 회차 | 난이도 | | 변별도1 | | 변별도2 | |
|---|---|---|---|---|---|---|
| | 평균 | 표준편차 | 평균 | 표준편차 | 평균 | 표준편차 |
| 제24회 | 65.1 | 16.9 | .23 | .11 | .19 | .08 |
| 제25회 | 72.4 | 13.2 | .25 | .11 | .23 | .10 |
| 제26회 | 73.8 | 19.9 | .19 | .10 | .21 | .08 |
| 제27회 | 59.2 | 23.4 | .23 | .15 | .21 | .12 |
| 제28회 | 64.3 | 21.8 | .28 | .14 | .26 | .11 |

**해석**

- 전회 대비 응급의료장비 과목의 난이도 지수는 5.1 증가함
- 전회 대비 응급의료장비 과목의 변별도 1 지수는 .05 증가함
- 전회 대비 응급의료장비 과목의 변별도 2 지수는 .05 증가함

## 나) 과목별 난이도와 변별도 분포도 및 비율분석

### (1) 기본응급처치학총론 난이도와 변별도 분포도 및 비율분석

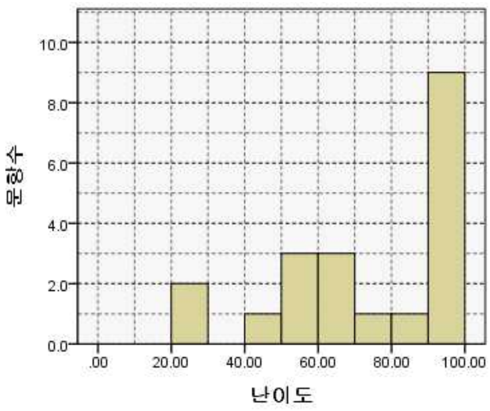


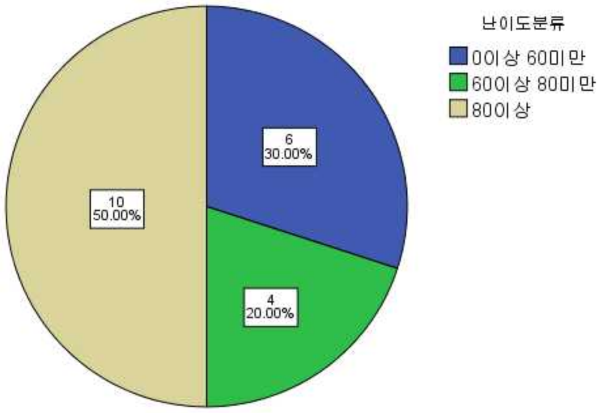


| 총점 | 난이도 | 표준편차 |
|---|---|---|
| 20 | 74.0 | 24.5 |

| 난이도 | 문항수 | 비율(%) |
|---|---|---|
| 0~60미만 | 6 | 30.0 |
| 60~80미만 | 4 | 20.0 |
| 80~100 | 10 | 50.0 |
| 전체 | 20 | 100.0 |

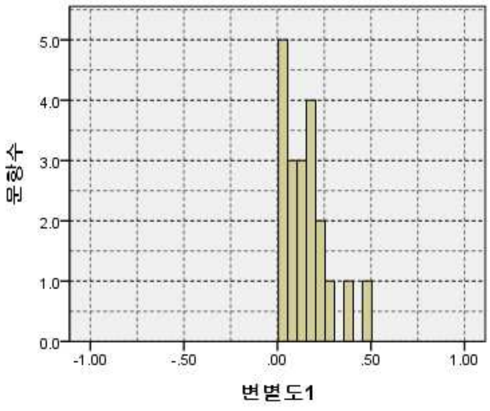


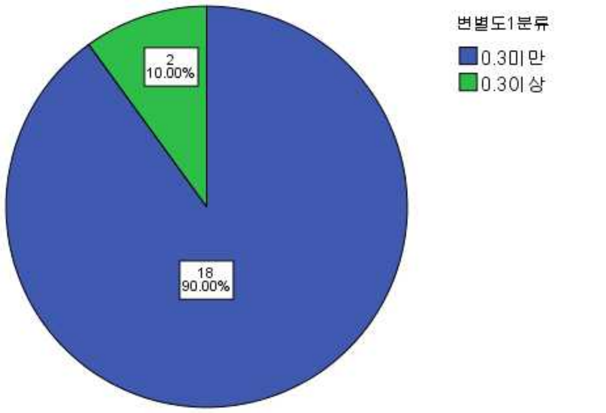


| 총점 | 변별도1 | 표준편차 |
|---|---|---|
| 20 | .16 | .12 |

| 변별도1 | 문항수 | 비율(%) |
|---|---|---|
| 0.3미만 | 18 | 90.0 |
| 0.3이상 | 2 | 10.0 |
| 전체 | 20 | 100.0 |

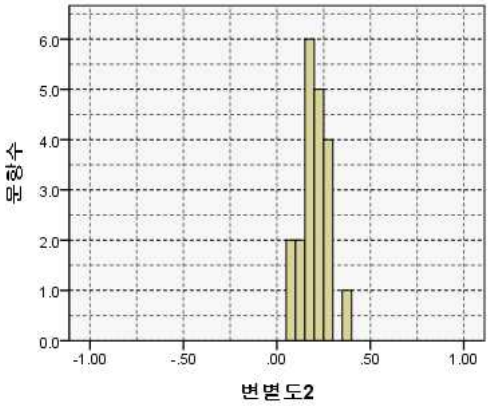


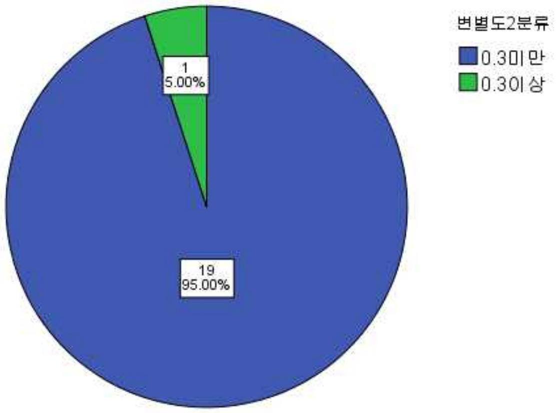


| 총점 | 변별도2 | 표준편차 |
|---|---|---|
| 20 | .20 | .07 |

| 변별도2 | 문항수 | 비율(%) |
|---|---|---|
| 0.3미만 | 19 | 95.0 |
| 0.3이상 | 1 | 5.0 |
| 전체 | 20 | 100.0 |

**해석**

- 기본응급처치학총론 과목에서 난이도 지수가 80 에서 100 사이인 문항이 전체 20 문항 중 10 문항이었으며, 60 이상 80 미만인 문항이 4 문항, 60 미만인 문항이 6 문항으로 나타남
- 변별도 1 지수를 기준으로 분류하였을 때, 0.3 미만인 문항이 18 문항으로 0.3 이상인 문항이 2 문항인 것에 비해 더 많이 나타남
- 변별도 2 지수를 기준으로 분류하였을 때, 0.3 미만인 문항이 19 문항으로 0.3 이상인 문항이 1 문항인 것에 비해 더 많이 나타남

(2) 기본응급환자관리 난이도와 변별도 분포도 및 비율분석

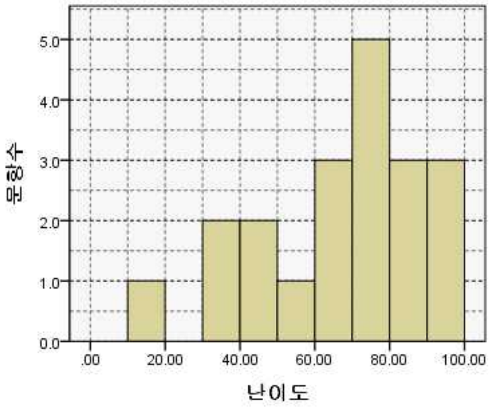


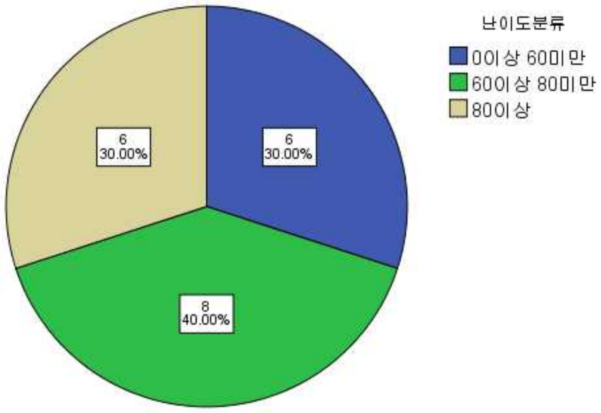


| 총점 | 난이도 | 표준편차 |
|---|---|---|
| 20 | 67.2 | 23.0 |

| 난이도 | 문항수 | 비율(%) |
|---|---|---|
| 0~60미만 | 6 | 30.0 |
| 60~80미만 | 8 | 40.0 |
| 80~100 | 6 | 30.0 |
| 전체 | 20 | 100.0 |

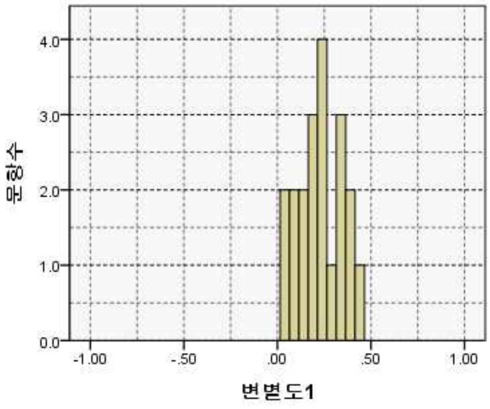


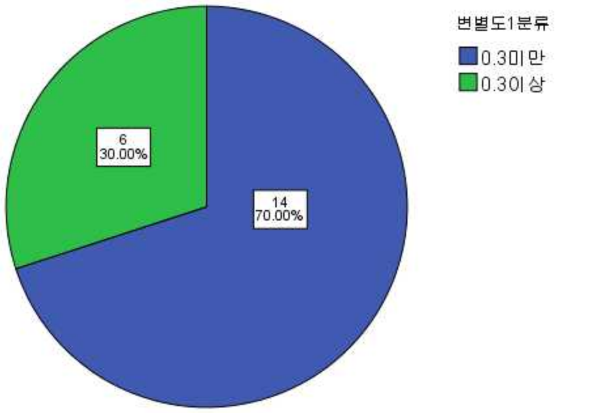


| 총점 | 변별도1 | 표준편차 |
|---|---|---|
| 20 | .23 | .11 |

| 변별도1 | 문항수 | 비율(%) |
|---|---|---|
| 0.3미만 | 14 | 70.0 |
| 0.3이상 | 6 | 30.0 |
| 전체 | 20 | 100.0 |

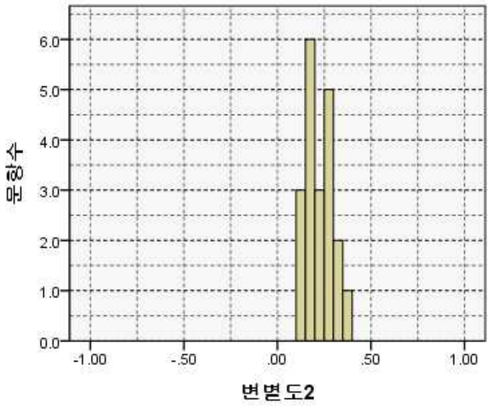


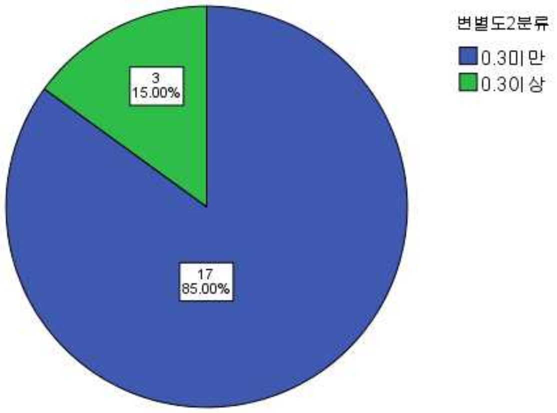


| 총점 | 변별도2 | 표준편차 |
|---|---|---|
| 20 | .23 | .07 |

| 변별도2 | 문항수 | 비율(%) |
|---|---|---|
| 0.3미만 | 17 | 85.0 |
| 0.3이상 | 3 | 15.0 |
| 전체 | 20 | 100.0 |

**해석**

- 기본응급환자관리 과목에서 난이도 지수가 80에서 100 사이인 문항이 전체 20문항 중 6문항이었으며, 60이상 80미만인 문항이 8문항, 60 미만인 문항이 6문항으로 나타남
- 변별도1 지수를 기준으로 분류하였을 때, 0.3미만인 문항이 14문항으로 0.3 이상인 문항이 6문항인 것에 비해 더 많이 나타남
- 변별도2 지수를 기준으로 분류하였을 때, 0.3미만인 문항이 17문항으로 0.3 이상인 문항이 3문항인 것에 비해 더 많이 나타남

(3) 응급의료관련법령 난이도와 변별도 분포도 및 비율분석

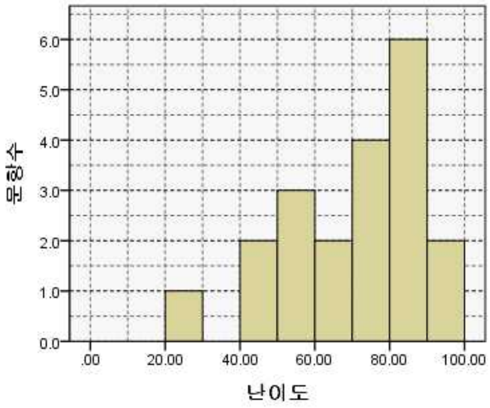


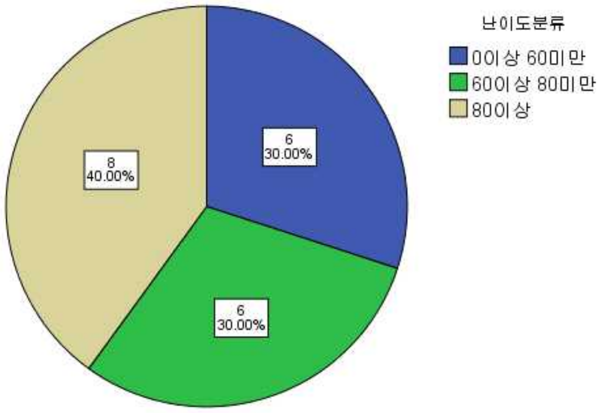


| 총점 | 난이도 | 표준편차 |
|---|---|---|
| 20 | 70.0 | 18.1 |

| 난이도 | 문항수 | 비율(%) |
|---|---|---|
| 0~60미만 | 6 | 30.0 |
| 60~80미만 | 6 | 30.0 |
| 80~100 | 8 | 40.0 |
| 전체 | 20 | 100.0 |

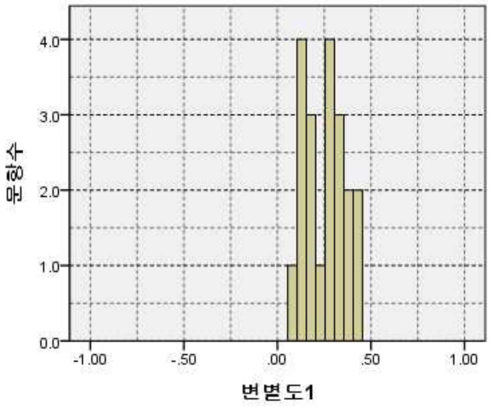


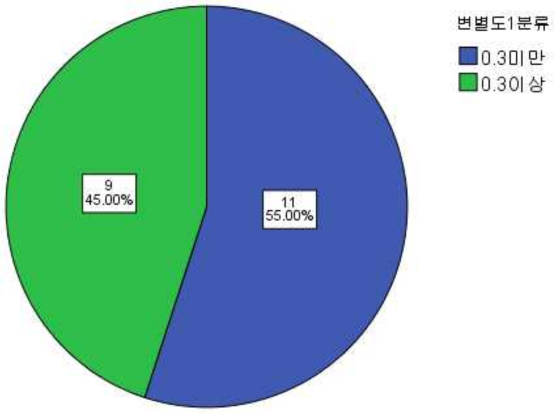


| 총점 | 변별도1 | 표준편차 |
|---|---|---|
| 20 | .26 | .10 |

| 변별도1 | 문항수 | 비율(%) |
|---|---|---|
| 0.3미만 | 11 | 55.0 |
| 0.3이상 | 9 | 45.0 |
| 전체 | 20 | 100.0 |

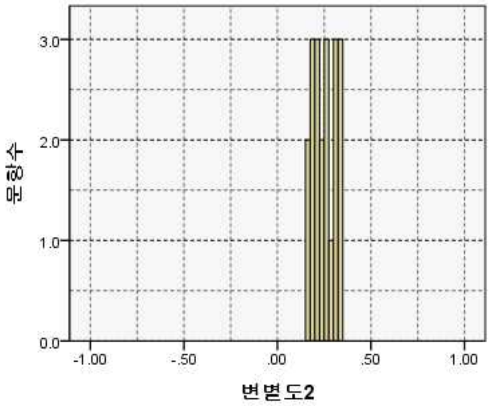


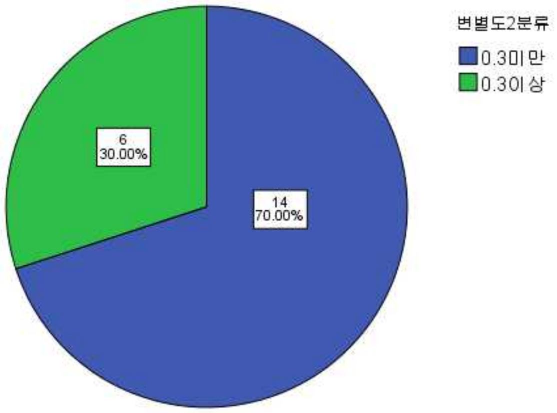


| 총점 | 변별도2 | 표준편차 | 변별도2 | 문항수 | 비율(%) |
|---|---|---|---|---|---|
| 20 | .25 | .06 | 0.3미만 | 14 | 70.0 |
| | | | 0.3이상 | 6 | 30.0 |
| | | | 전체 | 20 | 100.0 |

**해석**

- 응급의료관련법령 과목에서 난이도 지수가 80에서 100 사이인 문항이 전체 20문항 중 8문항이었으며, 60이상 80미만인 문항이 6문항, 60 미만인 문항이 6문항으로 나타남
- 변별도1 지수를 기준으로 분류하였을 때, 0.3미만인 문항이 11문항으로 0.3이상인 문항이 9문항인 것과 동일하게 나타남
- 변별도2 지수를 기준으로 분류하였을 때, 0.3미만인 문항이 14문항으로 0.3이상인 문항이 6문항인 것에 비해 더 많이 나타남

(4) 기본응급처치학각론 난이도와 변별도 분포도 및 비율분석

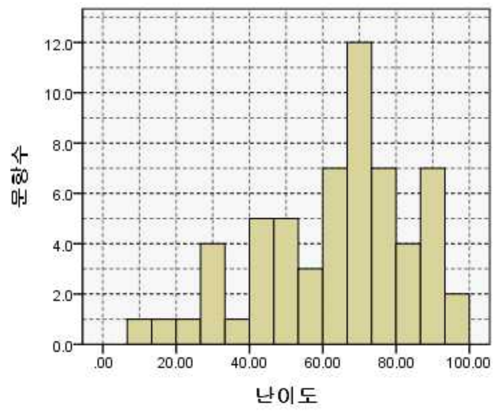


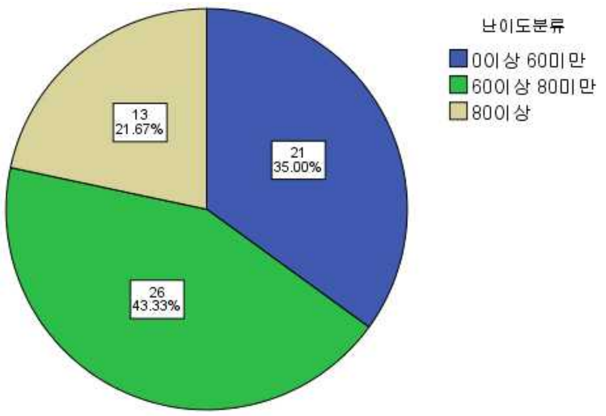


| 총점 | 난이도 | 표준편차 | 난이도 | 문항수 | 비율(%) |
|---|---|---|---|---|---|
| 60 | 63.7 | 20.6 | 0~60미만 | 21 | 35.0 |
| | | | 60~80미만 | 26 | 43.3 |
| | | | 80~100 | 13 | 21.7 |
| | | | 전체 | 60 | 100.0 |

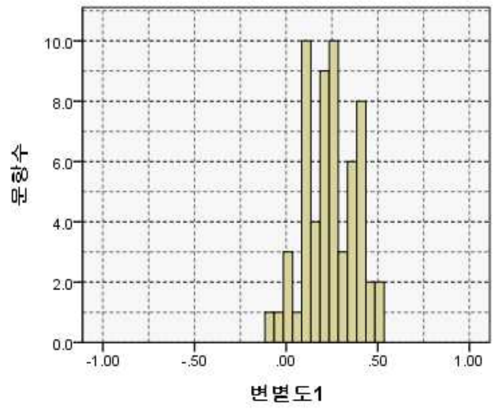


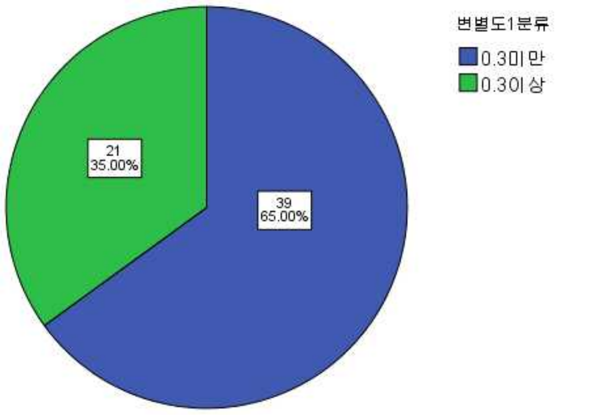


| 총점 | 변별도1 | 표준편차 | 변별도1 | 문항수 | 비율(%) |
|---|---|---|---|---|---|
| 60 | .24 | .14 | 0.3미만 | 39 | 65.0 |
| | | | 0.3이상 | 21 | 35.0 |
| | | | 전체 | 60 | 100.0 |

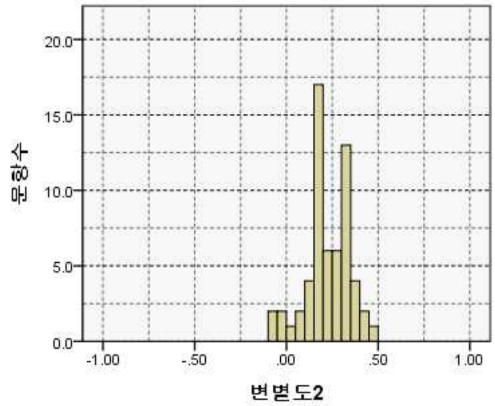


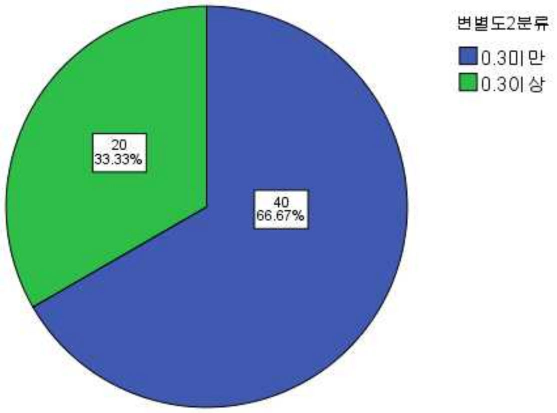


| 총점 | 변별도2 | 표준편차 |
|---|---|---|
| 60 | .22 | .12 |

| 변별도2 | 문항수 | 비율(%) |
|---|---|---|
| 0.3미만 | 40 | 66.7 |
| 0.3이상 | 20 | 33.3 |
| 전체 | 60 | 100.0 |

**해석**

- 기본응급처치학각론 과목에서 난이도 지수가 80 에서 100 사이인 문항이 전체 60 문항 중 13 문항이었으며, 60 이상 80 미만인 문항이 26 문항, 60 미만인 문항이 21 문항으로 나타남
- 변별도 1 지수를 기준으로 분류하였을 때, 0.3 미만인 문항이 39 문항으로 0.3 이상인 문항이 21 문항인 것에 비해 더 많이 나타남
- 변별도 2 지수를 기준으로 분류하였을 때, 0.3 미만인 문항이 40 문항으로 0.3 이상인 문항이 20 문항인 것에 비해 더 많이 나타남

(5) 응급의료장비 난이도와 변별도 분포도 및 비율분석

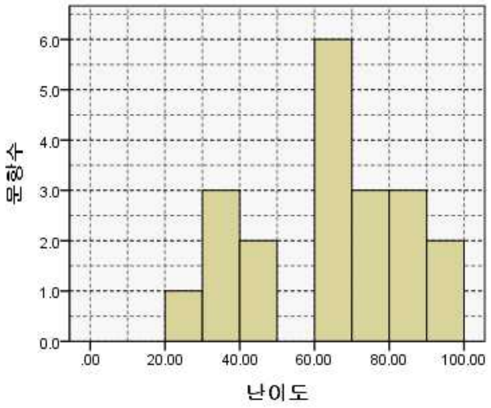


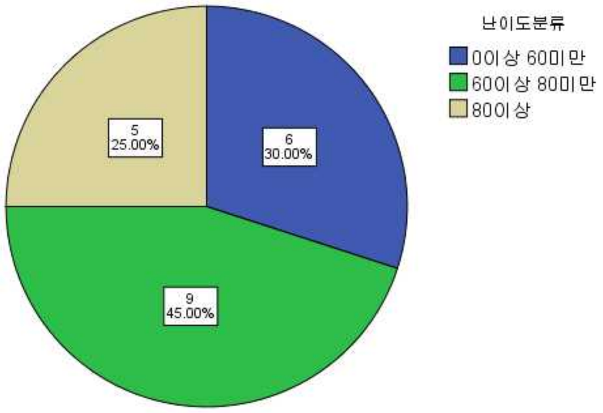


| 총점 | 난이도 | 표준편차 |
|---|---|---|
| 20 | 64.3 | 21.8 |

| 난이도 | 문항수 | 비율(%) |
|---|---|---|
| 0~60미만 | 6 | 30.0 |
| 60~80미만 | 9 | 45.0 |
| 80~100 | 5 | 25.0 |
| 전체 | 20 | 100.0 |

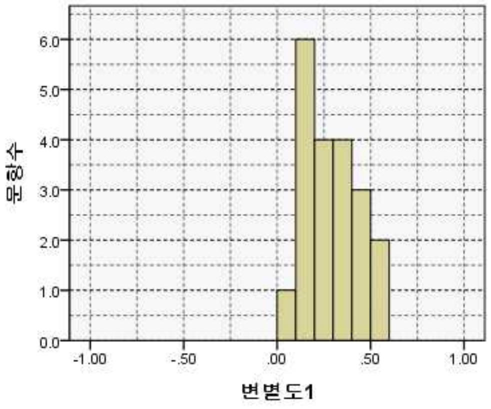


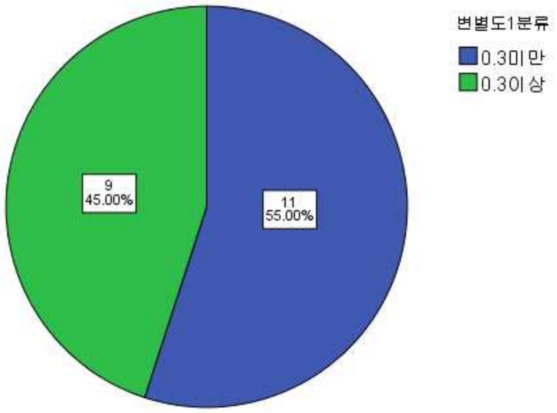


| 총점 | 변별도1 | 표준편차 |
|---|---|---|
| 20 | .28 | .14 |

| 변별도1 | 문항수 | 비율(%) |
|---|---|---|
| 0.3미만 | 11 | 55.0 |
| 0.3이상 | 9 | 45.0 |
| 전체 | 20 | 100.0 |

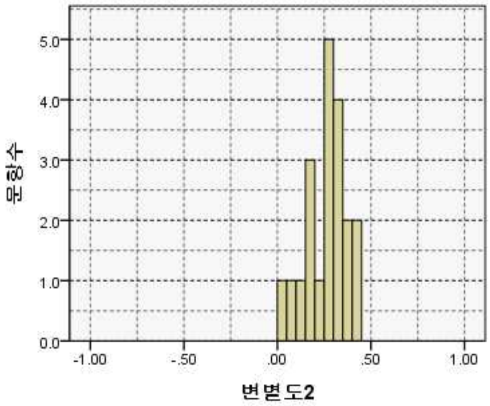


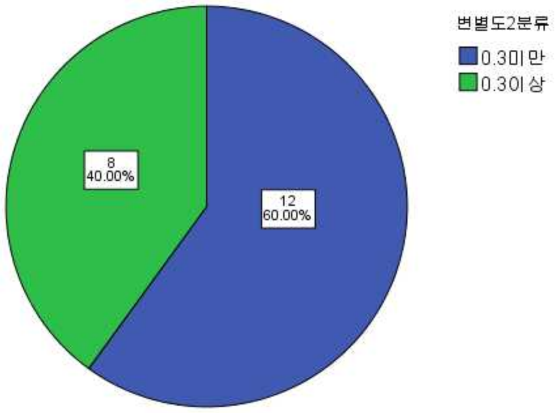


| 총점 | 변별도2 | 표준편차 |
|---|---|---|
| 20 | .26 | .11 |

| 변별도2 | 문항수 | 비율(%) |
|---|---|---|
| 0.3미만 | 12 | 60.0 |
| 0.3이상 | 8 | 40.0 |
| 전체 | 20 | 100.0 |

**해석**

- 응급의료장비 과목에서 난이도 지수가 80에서 100 사이인 문항이 전체 20문항 중 5문항이었으며, 60이상 80미만인 문항이 9문항, 60 미만인 문항이 6문항으로 나타남
- 변별도1 지수를 기준으로 분류하였을 때, 0.3미만인 문항이 11문항으로 0.3이상인 문항이 9문항인 것에 비해 더 많이 나타남
- 변별도2 지수를 기준으로 분류하였을 때, 0.3미만인 문항이 12문항으로 0.3이상인 문항이 8문항인 것에 비해 더 많이 나타남

## 3) 지식수준별 난이도와 변별도

### 가) 전회 대비 지식수준별 난이도와 변별도

#### (1) 전회 대비 암기형 난이도와 변별도

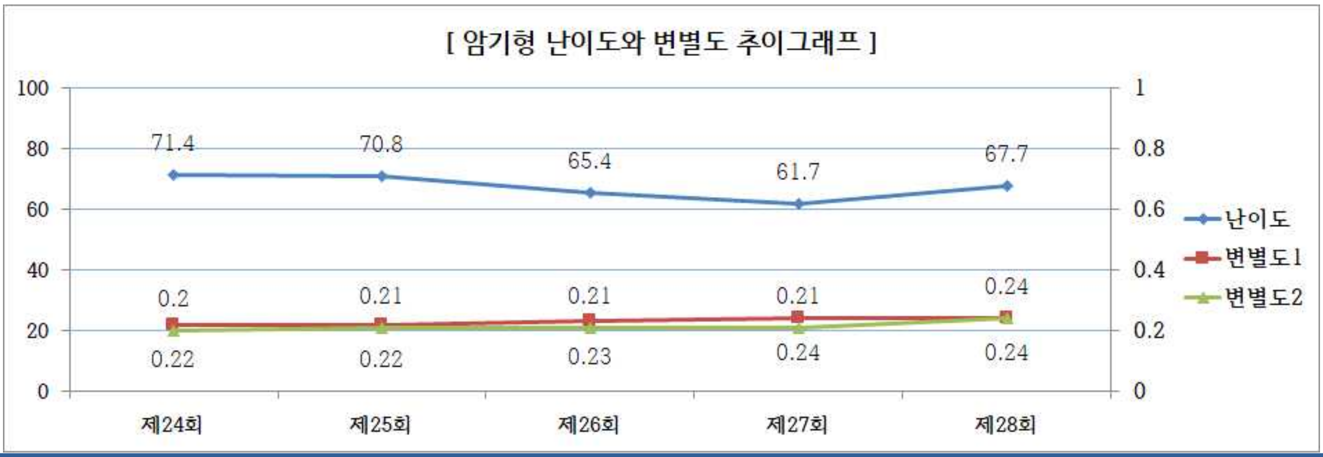


| 회차 | 난이도 | | 변별도1 | | 변별도2 | |
|---|---|---|---|---|---|---|
| | 평균 | 표준편차 | 평균 | 표준편차 | 평균 | 표준편차 |
| 제24회 | 71.4 | 18.7 | .22 | .11 | .20 | .08 |
| 제25회 | 70.8 | 19.3 | .22 | .11 | .21 | .09 |
| 제26회 | 65.4 | 22.4 | .23 | .12 | .21 | .08 |
| 제27회 | 61.7 | 24.5 | .24 | .13 | .21 | .10 |
| 제28회 | 67.7 | 22.2 | .24 | .12 | .24 | .08 |

#### (2) 전회 대비 해석형 난이도와 변별도

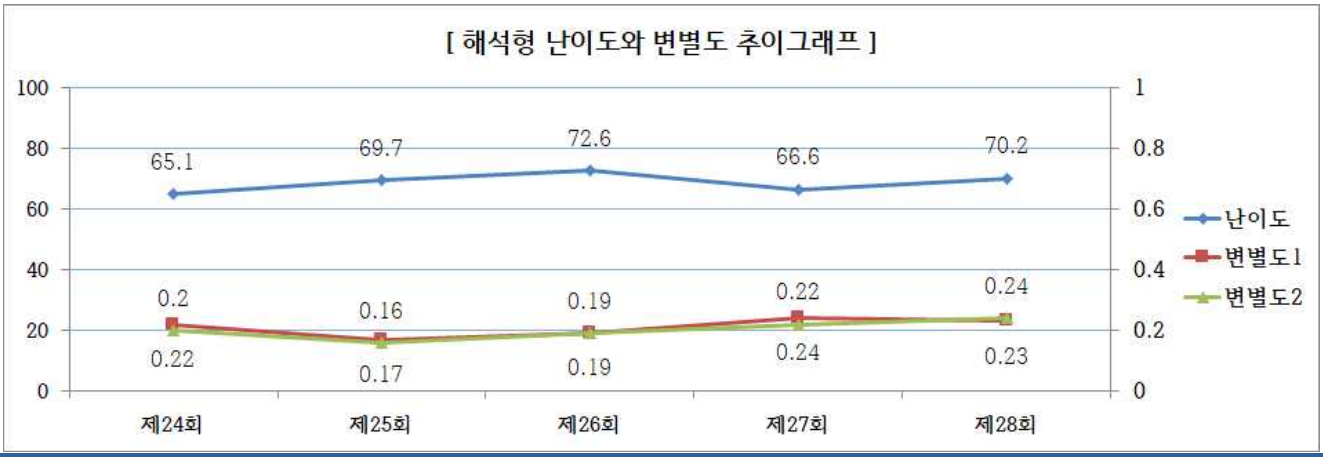


| 회차 | 난이도 | | 변별도1 | | 변별도2 | |
|---|---|---|---|---|---|---|
| | 평균 | 표준편차 | 평균 | 표준편차 | 평균 | 표준편차 |
| 제24회 | 65.1 | 21.8 | .22 | .13 | .20 | .09 |
| 제25회 | 69.7 | 19.4 | .17 | .09 | .16 | .08 |
| 제26회 | 72.6 | 21.1 | .19 | .12 | .19 | .09 |
| 제27회 | 66.6 | 20.4 | .24 | .16 | .22 | .11 |
| 제28회 | 70.2 | 19.2 | .23 | .14 | .24 | .09 |

(3) 전회 대비 해결형 난이도와 변별도

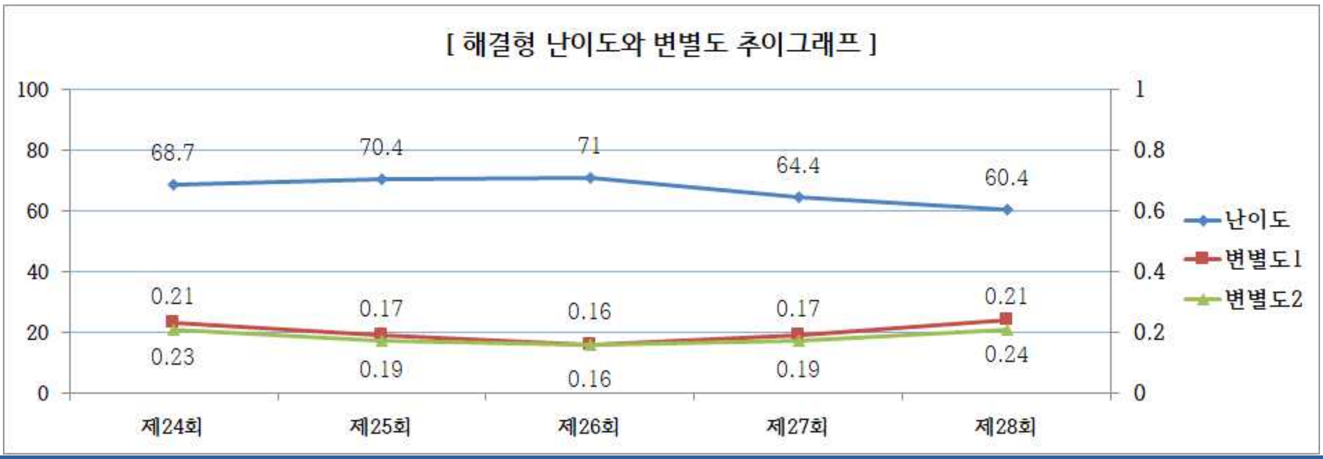


| 회차 | 난이도 | | 변별도1 | | 변별도2 | |
|---|---|---|---|---|---|---|
| | 평균 | 표준편차 | 평균 | 표준편차 | 평균 | 표준편차 |
| 제24회 | 68.7 | 19.8 | .23 | .12 | .21 | .09 |
| 제25회 | 70.4 | 21.0 | .19 | .11 | .17 | .09 |
| 제26회 | 71.0 | 20.5 | .16 | .12 | .16 | .11 |
| 제27회 | 64.4 | 23.3 | .19 | .12 | .17 | .10 |
| 제28회 | 60.4 | 22.3 | .24 | .15 | .21 | .13 |

**해석**

- 전회 대비 암기형 문항의 난이도 지수는 6.0 증가하였으며, 해석형 문항의 난이도 지수는 3.6 증가하였고 해결형 문항의 난이도 지수는 4.0 감소함
- 변별도 1 지수는 암기형 문항에서 변하지 않았고, 해석형 문항에서 .01 감소하였으며, 해결형 문항에서 .05 증가함
- 변별도 2 지수는 암기형 문항에서 .03 증가하였고, 해석형 문항에서 .02 증가하였으며, 해결형 문항에서 .04 증가함

## 나) 지식수준별 난이도와 변별도 분포도 및 비율분석

### (1) 암기형 난이도와 변별도 분포도 및 비율분석

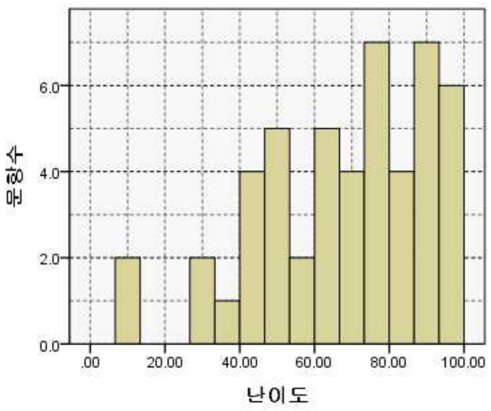


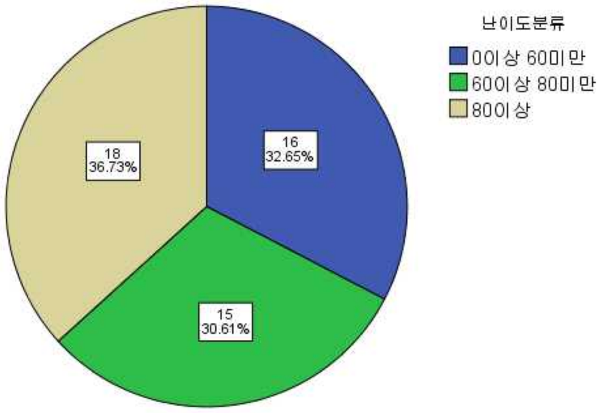


| 총점 | 난이도 | 표준편차 |
|---|---|---|
| 49 | 67.7 | 22.2 |

| 난이도 | 문항수 | 비율(%) |
|---|---|---|
| 0~60미만 | 16 | 32.7 |
| 60~80미만 | 15 | 30.6 |
| 80~100 | 18 | 36.7 |
| 전체 | 49 | 100.0 |

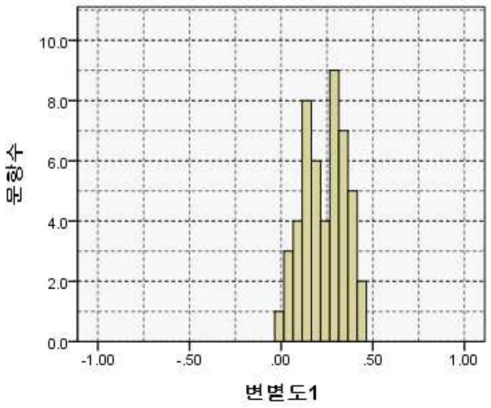


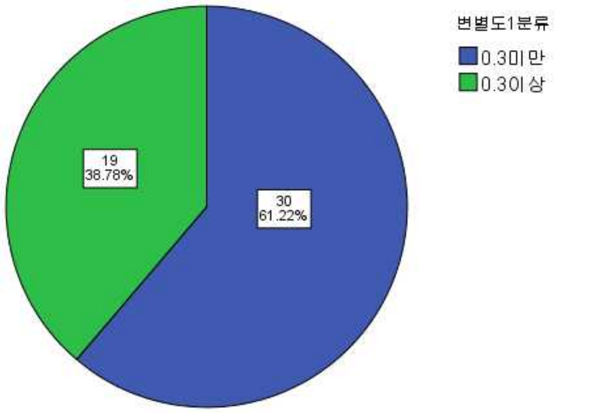


| 총점 | 변별도1 | 표준편차 |
|---|---|---|
| 49 | .24 | .12 |

| 변별도1 | 문항수 | 비율(%) |
|---|---|---|
| 0.3미만 | 30. | 61.2 |
| 0.3이상 | 19 | 38.8 |
| 전체 | 49 | 100.0 |

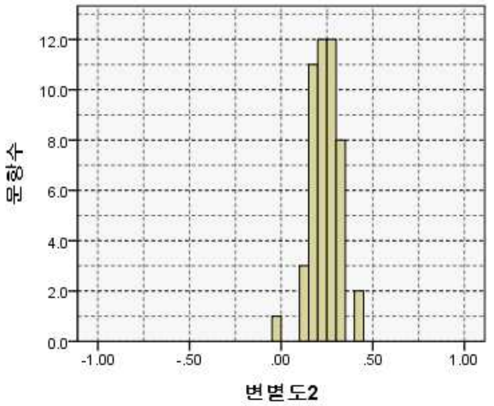


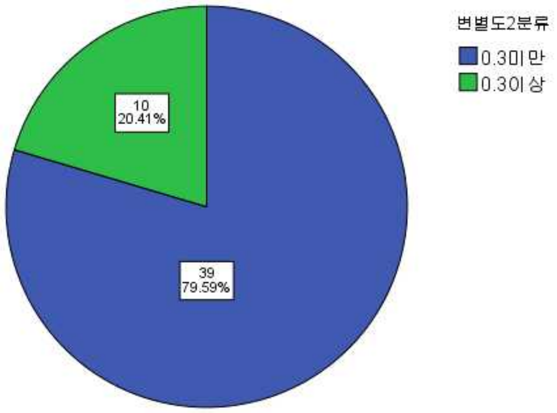


| 총점 | 변별도2 | 표준편차 | 변별도2 | 문항수 | 비율(%) |
|---|---|---|---|---|---|
| 49 | .24 | .08 | 0.3미만 | 39 | 79.6 |
| | | | 0.3이상 | 10 | 20.4 |
| | | | 전체 | 49 | 100.0 |

**해석**

- 암기형 문항에서 난이도 지수가 80 에서 100 사이인 문항이 전체 49 문항 중 18 문항이었으며, 60 이상 80 미만인 문항이 15 문항, 60 미만인 문항이 16 문항인 것으로 나타남
- 변별도 1 지수를 기준으로 하였을 때, 0.3 미만인 문항이 30 문항으로 0.3 이상인 문항이 19 문항인 것에 비해 더 많이 나타남
- 변별도 2 지수를 기준으로 분류하였을 때, 0.3 미만인 문항이 39 문항으로 0.3 이상인 문항이 10 문항인 것에 비해 더 많이 나타남

(2) 해석형 난이도와 변별도 분포도 및 비율분석

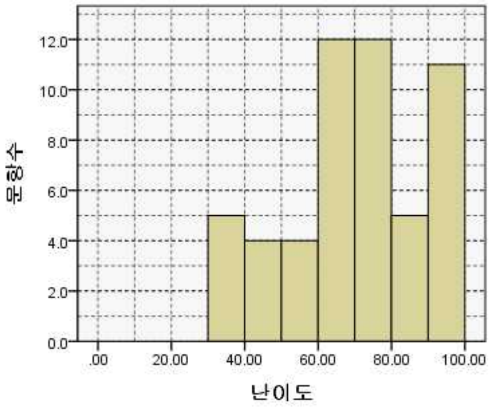


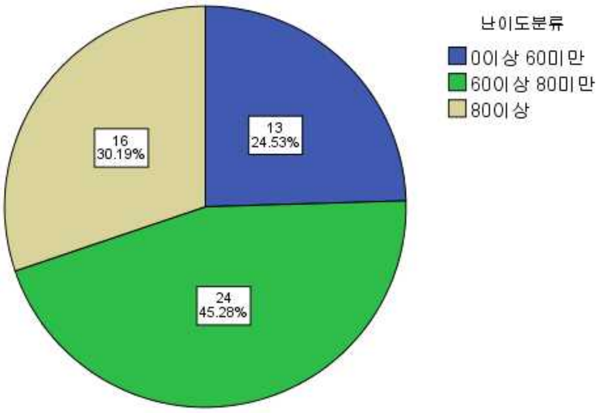


| 총점 | 난이도 | 표준편차 |
|---|---|---|
| 53 | 70.2 | 19.2 |

| 난이도 | 문항수 | 비율(%) |
|---|---|---|
| 0~60미만 | 13 | 24.5 |
| 60~80미만 | 24 | 45.3 |
| 80~100 | 16 | 30.2 |
| 전체 | 53 | 100.0 |

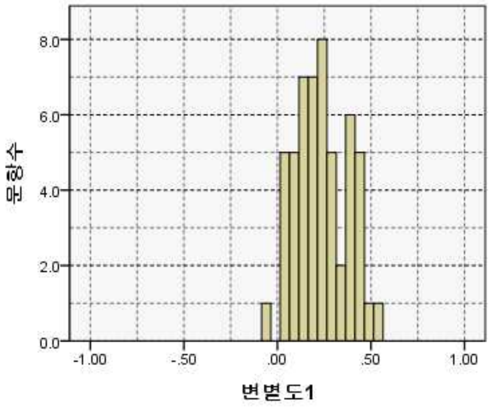


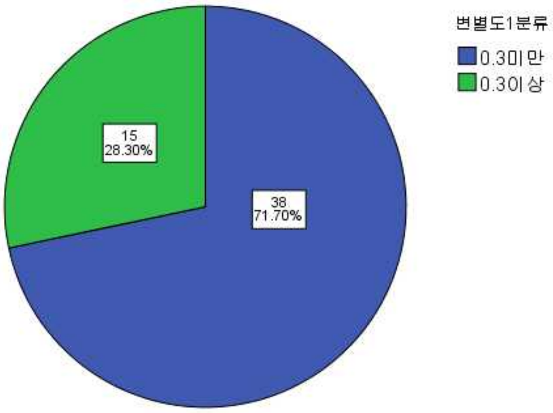


| 총점 | 변별도1 | 표준편차 |
|---|---|---|
| 53 | .23 | .14 |

| 변별도1 | 문항수 | 비율(%) |
|---|---|---|
| 0.3미만 | 38 | 71.7 |
| 0.3이상 | 15 | 28.3 |
| 전체 | 53 | 100.0 |

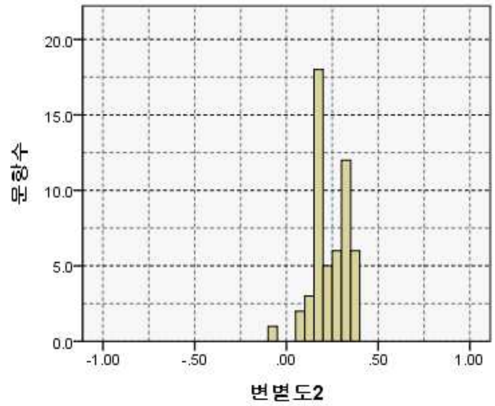


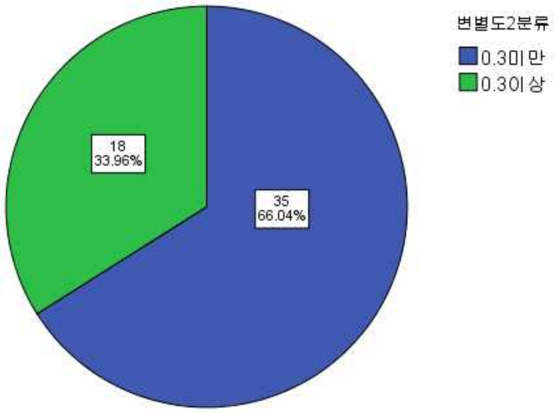


| 총점 | 변별도2 | 표준편차 |
|---|---|---|
| 53 | .24 | .09 |

| 변별도2 | 문항수 | 비율(%) |
|---|---|---|
| 0.3미만 | 35 | 66.0 |
| 0.3이상 | 18 | 34.0 |
| 전체 | 53 | 100.0 |

**해석**

- 해석형 문항에서 난이도 지수가 80 에서 100 사이인 문항이 전체 53 문항 중 16 문항이었으며, 60 이상 80 미만인 문항이 24 문항, 60 미만인 문항이 13 문항인 것으로 나타남
- 변별도 1 지수를 기준으로 하였을 때, 0.3 미만인 문항이 38 문항으로 0.3 이상인 문항이 15 문항인 것에 비해 더 많이 나타남
- 변별도 2 지수를 기준으로 분류하였을 때, 0.3 미만인 문항이 35 문항으로 0.3 이상인 문항이 18 문항인 것에 비해 더 많이 나타남

(3) 해결형 난이도와 변별도 분포도 및 비율분석

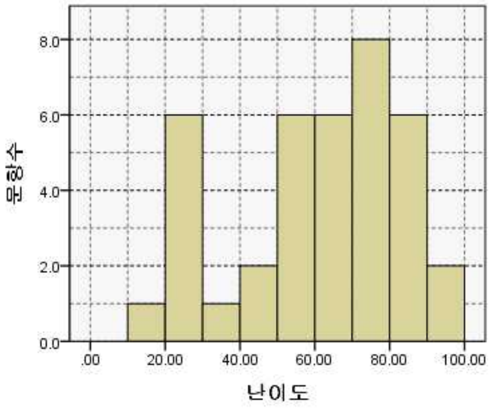


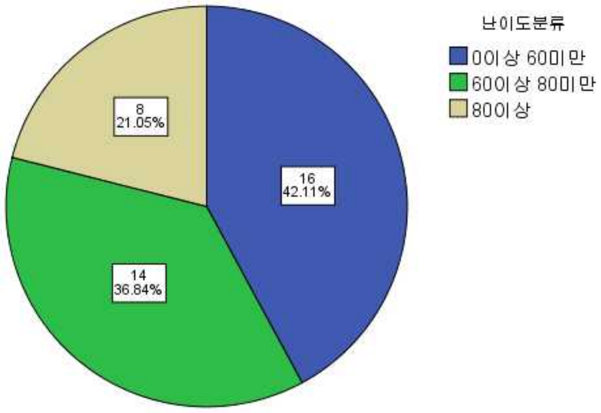


| 총점 | 난이도 | 표준편차 |
|---|---|---|
| 38 | 60.4 | 22.3 |

| 난이도 | 문항수 | 비율(%) |
|---|---|---|
| 0~60미만 | 16 | 42.1 |
| 60~80미만 | 14 | 36.8 |
| 80~100 | 8 | 21.1 |
| 전체 | 38 | 100.0 |

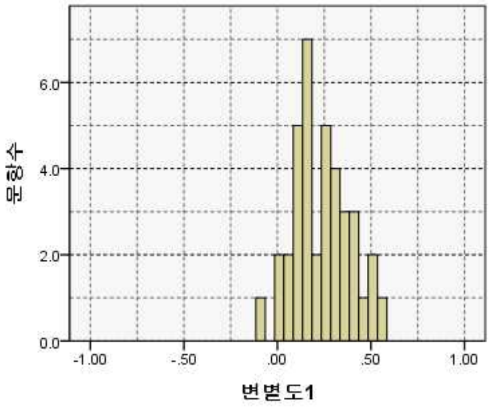


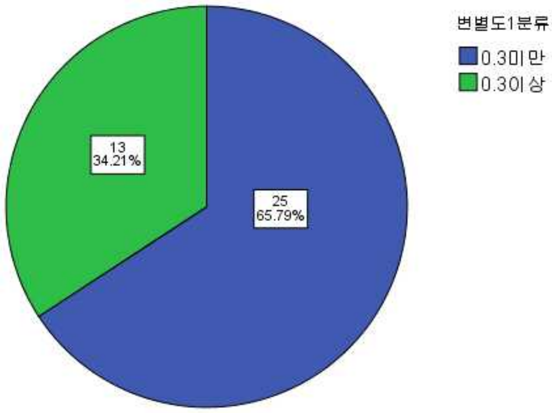


| 총점 | 변별도1 | 표준편차 |
|---|---|---|
| 38 | .24 | .15 |

| 변별도1 | 문항수 | 비율(%) |
|---|---|---|
| 0.3미만 | 25 | 65.8 |
| 0.3이상 | 13 | 34.2 |
| 전체 | 38 | 100.0 |

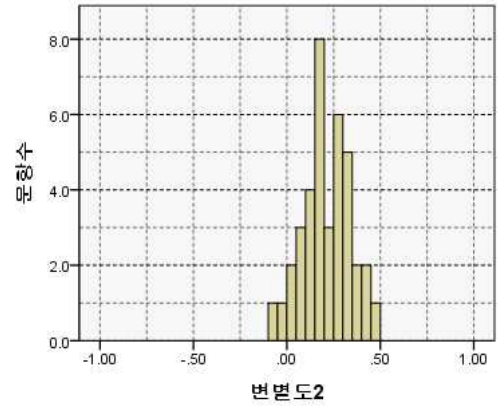


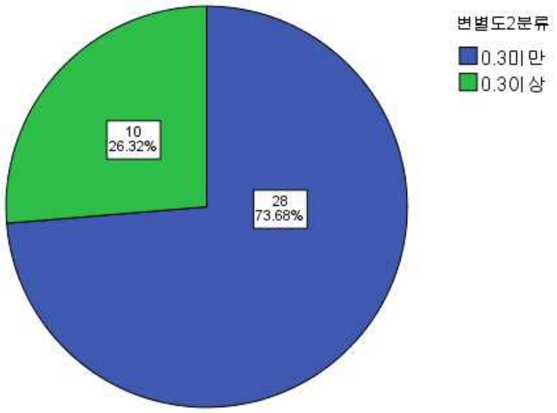


| 총점 | 변별도2 | 표준편차 |
|---|---|---|
| 38 | .21 | .13 |

| 변별도2 | 문항수 | 비율(%) |
|---|---|---|
| 0.3미만 | 28 | 73.7 |
| 0.3이상 | 10 | 26.3 |
| 전체 | 38 | 100.0 |

**해석**

- 해결형 문항에서 난이도 지수가 80에서 100 사이인 문항이 전체 38문항 중 8문항이었으며, 60이상 80미만인 문항이 14문항, 60미만인 문항이 16문항인 것으로 나타남
- 변별도1 지수를 기준으로 하였을 때, 0.3미만인 문항이 25문항으로 0.3이상인 문항이 13문항인 것에 비해 더 많이 나타남
- 변별도2 지수를 기준으로 분류하였을 때, 0.3미만인 문항이 28문항으로 0.3이상인 문항이 10문항인 것에 비해 더 많이 나타남

## 3. 난이도와 변별도 간 산포도

### 1) 전체 난이도와 변별도 간 산포도

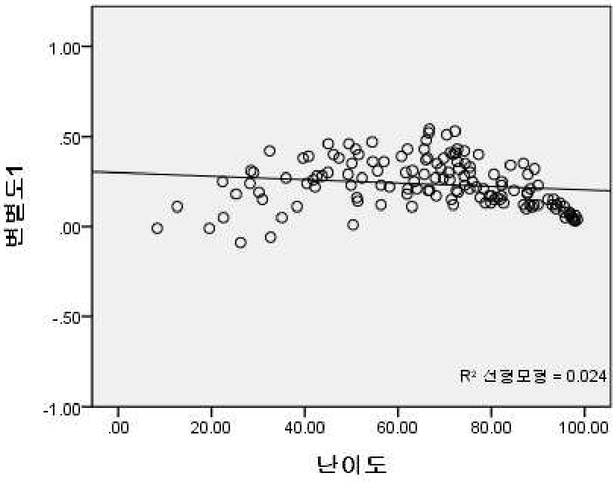


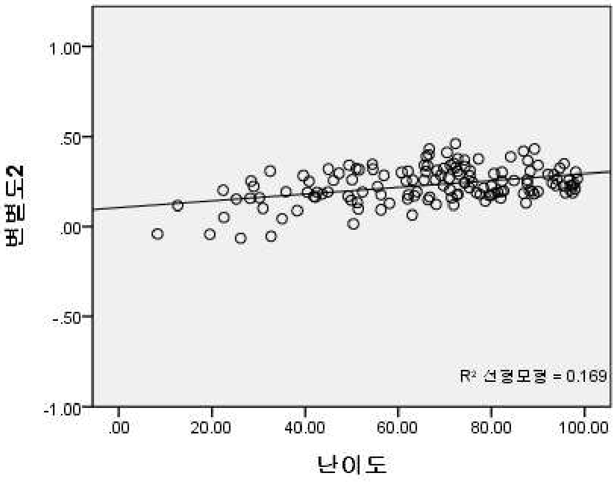


**해석**

- 난이도 지수와 변별도 1 지수 간 상관은 -.154 로 관련성이 없는 것으로 나타남
- 난이도 지수와 변별도 2 지수 간 상관은 .411*로 난이도 지수가 높을수록 변별력이 높아지는 것으로 나타남

### 2) 난이도와 변별도 간 산포도

#### 가) 기본응급처치학총론 난이도와 변별도 간 산포도

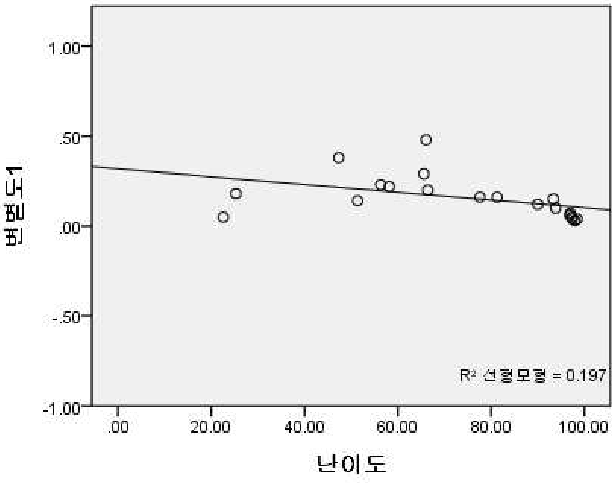


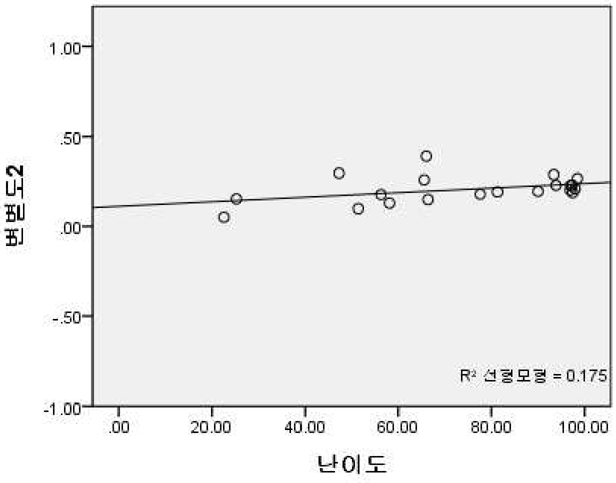

**해석**

- 난이도 지수와 변별도 1 지수 간 상관은 -.443 으로 관련성이 낮은 것으로 나타남
- 난이도 지수와 변별도 2 지수 간 상관은 -.418 로 관련성이 낮은 것으로 나타남

나) 기본응급환자관리 난이도와 변별도 간 산포도

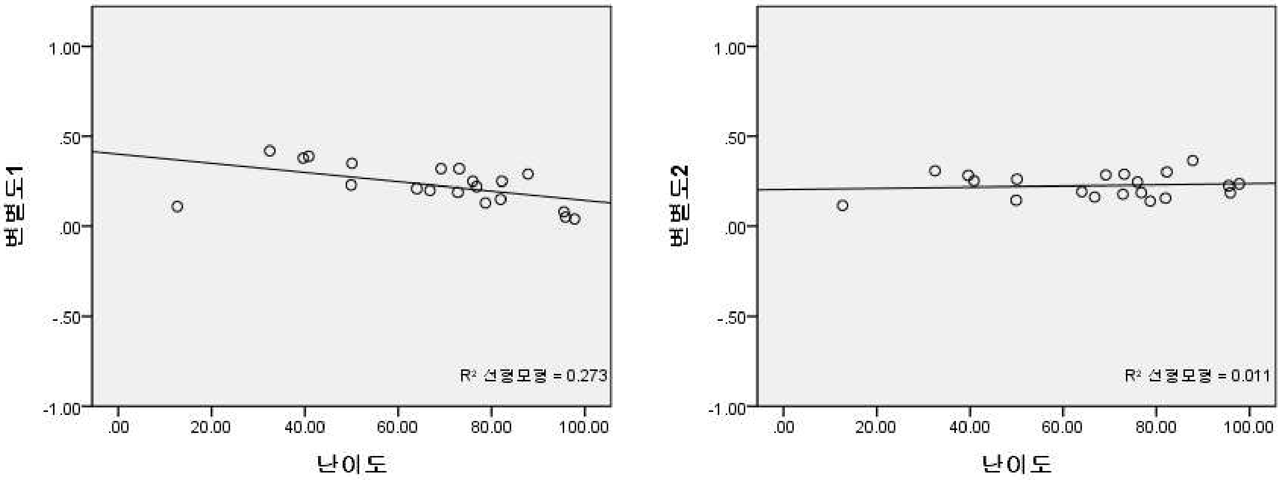


**해석**

- 난이도 지수와 변별도 1 지수 간 상관은 -.522*로 난이도 지수가 높을수록 변별력이 낮아지는 것으로 나타남
- 난이도 지수와 변별도 2 지수 간 상관은 -.104 로 관련성이 없는 것으로 나타남

다) 응급의료관련법령 난이도와 변별도 간 산포도

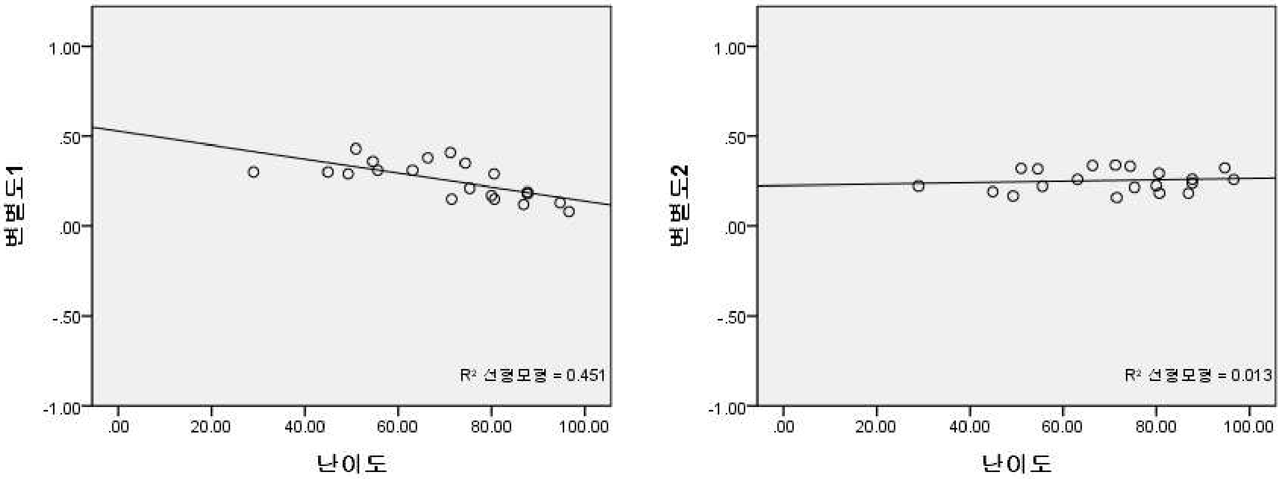

**해석**

- 난이도 지수와 변별도 1 지수 간 상관은 -.672*로 난이도 지수가 높을수록 변별력이 낮아지는 것으로 나타남
- 난이도 지수와 변별도 2 지수 간 상관은 -.016 으로 관련성이 없는 것으로 나타남

라) 기본응급처치학각론 난이도와 변별도 간 산포도

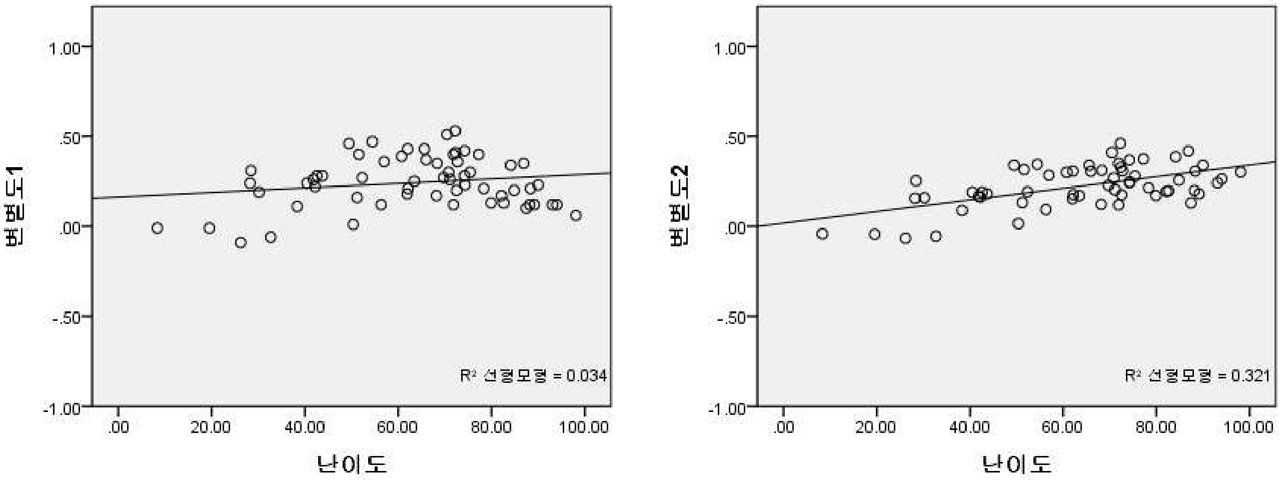


**해석**

- 난이도 지수와 변별도 1 지수 간 상관은 -.183 으로 관련성이 없는 것으로 나타남
- 난이도 지수와 변별도 2 지수 간 상관은 .566*으로 난이도 지수가 높을수록 변별력이 높아지는 것으로 나타남

마) 응급의료장비 난이도와 변별도 간 산포도

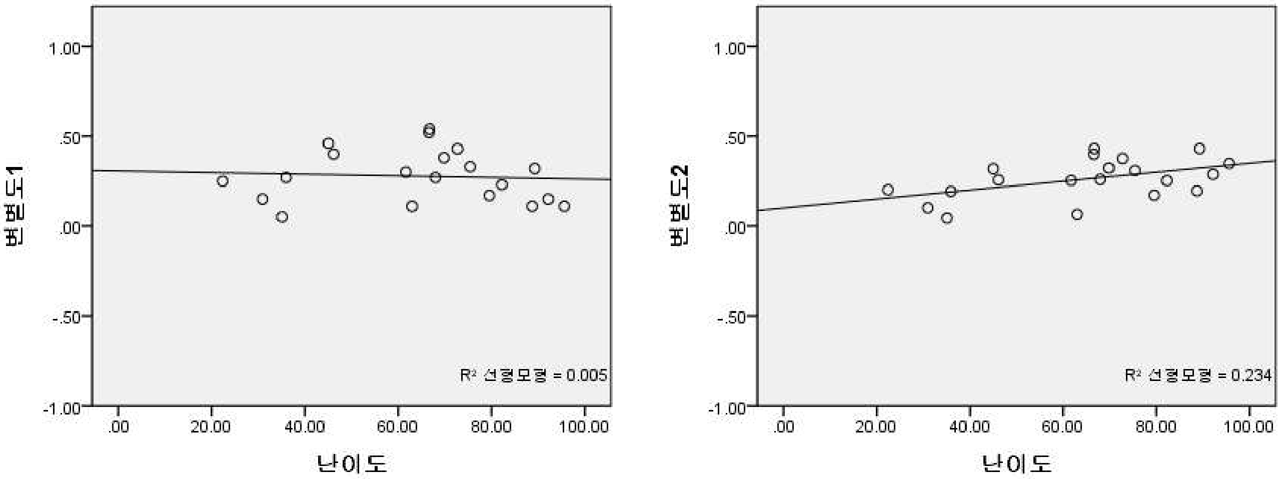

**해석**

- 난이도 지수와 변별도1 지수 간 상관은 -.068로 관련성이 없는 것으로 나타남
- 난이도 지수와 변별도2 지수 간 상관은 .484*로 난이도 지수가 높을수록 변별력이 높아지는 것으로 나타남

## 4. 신뢰도 분석

| 과목명 | 문항수 | 제24회 | 제25회 | 제26회 | 제27회 | 제27회 |
|---|---|---|---|---|---|---|
| 전체 | 140 | .873 | .847 | .847 | .876 | .892 |
| 기본응급처치학총론 | 20 | .446 | .382 | .259 | .443 | .532 |
| 기본응급환자관리 | 20 | .577 | .452 | .405 | .547 | .563 |
| 응급의료관련법령 | 20 | .663 | .661 | .629 | .670 | .648 |
| 기본응급처치학각론 | 60 | .747 | .681 | .731 | .749 | .784 |
| 응급의료장비 | 20 | .479 | .593 | .548 | .546 | .617 |

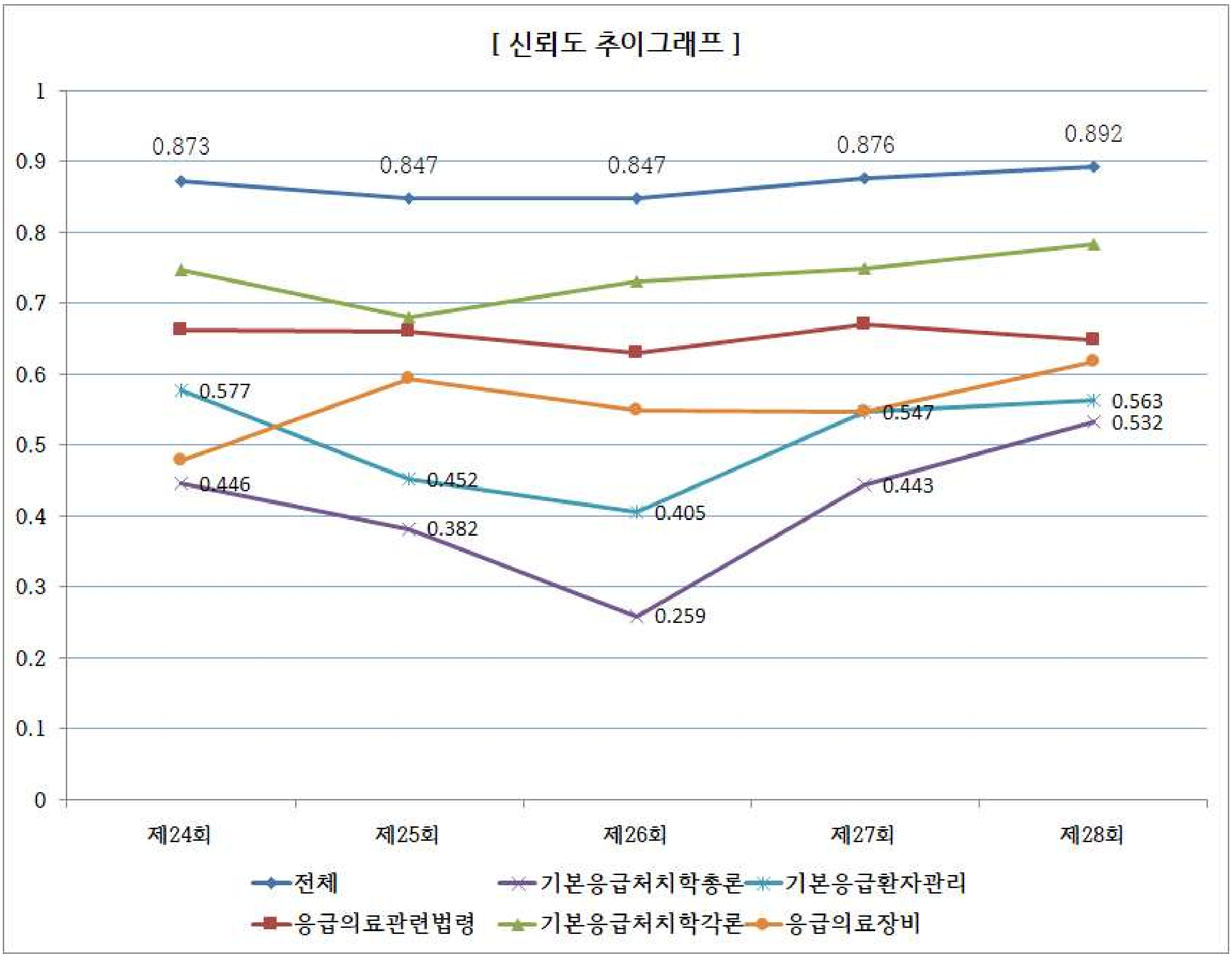

**해석**

- 전회 대비 전체문항의 신뢰도는 .016 증가함
- 전회 대비 기본응급처치학총론과목 문항의 신뢰도는 .089 증가함
- 전회 대비 기본응급환자관리 과목 문항의 신뢰도는 .016 증가함
- 전회 대비 응급의료관련법령 과목 문항의 신뢰도는 .022 감소함
- 전회 대비 기본응급처치학각론 과목 문항의 신뢰도는 .035 증가함
- 전회 대비 응급의료장비 과목 문항의 신뢰도는 .071 증가함

○ 분석결과 관련 문의 : 한국보건의료인국가시험원 연구개발본부 김준기 전임연구원
Tel : 02-2087-8956, FAX : 02-2087-8885
E-mail : tontates@kuksiwon.or.kr
