# Supplementary material for: Item difficulty index, discrimination index, and reliability of the 26 health professions licensing examinations in 2022, Korea: a psychometric study
Source: J Educ Eval Health Prof. 2023 Nov 22;20:31. doi: 10.3352/jeehp.2023.20.31 (PMC11959405; doi:10.3352/jeehp.2023.20.31)
Supplement: Supplementary file 1 — Supplement 1. Item analysis results of 26 health professions licensing examinations administered during late 2022 and early 2023. [file jeehp-20-31_Suppl1.zip › 2022│Γ╡╡ ┴a35╚╕ ╛╚░μ╗τ ▒╣░í╜├╟Φ ║╨╝«░ß░·.pdf]

2022년도 제35회 안경사 국가시험 문항분석 결과

국민이 신뢰하고 감동하는 시험평가기관
한국보건의료인국가시험원
KOREA HEALTH PERSONNEL LICENSING EXAMINATION INSTITUTE

일반 용어 정의

□ **평균**
- 집단에서의 대표적 경향값으로 전체 값을 더하여 총 응시자로 나눈 값

□ **표준편차**
- 평균과 각 점수의 차이인 편차들의 평균으로 점수가 흩어져 분포되어 있는 정도

□ **추정난이도**
- 문항개발자가 예측한 정답률

□ **검사이론**
- 검사와 검사를 구성하고 있는 문항의 양호도를 분석 및 평가하는 방법을 정의한 이론체계
- 대표적으로 고전검사이론과 문항반응이론이 있음

고전검사이론 용어 정의

□ **고전검사이론(Classical Test Theory; CTT)**

- 검사의 질을 분석하는 검사이론 중 한 가지로 19세기 말부터 전개되어 현재까지 주로 사용되고 있는 검사이론임
- 고전검사이론에 의한 문항과 응시자 능력 추정치는 다음과 같음

○ **문항난이도**

· 검사 문항의 쉽고 어려운 정도를 나타내는 지수
· 난이도 지수는 총 반응 수에 대한 정답 반응 수의 비율로 문항의 정답률임
· 문항난이도는 0~100까지의 값을 가짐
· 난이도 값이 큰 경우, 쉬운 문항으로 '난이도가 낮다'라고 해석하며, 난이도 값이 작은 경우, 어려운 문항으로 '난이도가 높다'라고 해석함

○ **문항변별도**

· 각 문항이 응시자의 능력 수준을 변별할 수 있는 정도를 나타내는 지수
· 문항변별도는 -1~+1까지의 값을 가지며, 1에 가까울수록 변별력 크다고 해석함
· 일반적으로 문항변별도가 0.3 이상이면 우수한 문항으로 평가함
· 구하는 방식에는 '상하위집단 구분법', '문항-총점 상관계수' 등이 있음
  1) 변별도 1(상하위구분법): 상위 27%와 하위 27% 집단의 난이도 차이를 구하는 방식
  2) 변별도 2(상관계수법): 문항-총점과의 상관계수로 구하는 방식

○ **신뢰도**

· 시험이 평가하고자 하는 것을 일관성 있게 측정하는가로 시험이 오차없이 정확하게 측정한 정도를 의미함
· 국시원에서는 문항의 내적일관성(Cronbach α)으로 신뢰도를 추정하며 1에 가까울수록 신뢰도가 높다고 해석함

목 차

I. 시행 결과 ···················· 6
1. 시험 현황 ···················· 7
1) 시험명 ···················· 7
2) 시험시행일 ···················· 7
3) 응시현황 ···················· 7
4) 과목별 문항 수, 배점 및 과락 점수 ···················· 7
2. 합격률과 평균성적 ···················· 7
1) 합격 및 불합격 현황 ···················· 7
2) 과목별 과락자수 내역 ···················· 7
3) 전회 대비 합격률과 평균성적 ···················· 8

II. 문항분석 결과 ···················· 10
1. 성적 ···················· 11
1) 전체 성적분포도 ···················· 11
2) 과목별 성적분포도 ···················· 12
2. 난이도와 변별도 ···················· 13
1) 전체 난이도와 변별도 ···················· 13
2) 과목별 난이도와 변별도 ···················· 16
3) 지식수준별 난이도와 변별도 ···················· 28
3. 난이도와 변별도 간 산포도 ···················· 37
1) 전체 난이도와 변별도 간 산포도 ···················· 37
2) 과목별 난이도와 변별도 간 산포도 ···················· 38
4. 신뢰도 분석 ···················· 41

I. 시행 결과

1. 시험 현황

1) 시험명: 2022년도 제35회 안경사 국가시험

2) 시험시행일: 2022년 12월 17일

3) 응시현황

응시대상자 수	결시자 수	부정행위자 수	응시자 준수사항 위반자 수		응시자 수 (%)
			휴대폰 소지	신분증 미지참	
1,712	92	0	0	0	1,619 (94.6%)

※ 1,619명은 응시대상자(1,712명)에서 결시자수(92명) 및 채점보류자수(1명)를 제외한 수치임

4) 과목별 문항 수, 배점 및 과락 점수

교 시	과 목 명	문제 수	배점	총점	합격자 점수기준	
					과목 과락기준	총점 합격기준
1교시	시광학이론	85	1	85	34점 미만	114점 이상
2교시	의료관계법규	20	1	20	8점 미만	
2교시	시광학응용	85	1	85	34점 미만	
3교시	실기시험	60	1	60	36점 미만	
계		250		250		

2. 합격률과 평균성적

1) 합격 및 불합격 현황

합격자 수 (%)	불합격자 수(%)					채점보류자 수
	평락	과락	실기탈락	기권	계	
1,213 (74.9)	390 (24.09)	0 (0.0)	13 (0.80)	3 (0.19)	406 (25.1)	1

2) 과목별 과락자수 내역

과락자 수 \ 과목명	시광학이론	의료관계법규	시광학응용	실기시험
과목별 과락자 수	0	0	0	0
전과목 과락자 수	0			

3) 전회 대비 합격률과 평균성적

회차	년도	합격률(%)	평균성적	표준편차	백분율 환산점수
제31회	2018	76.8	172.4	46.8	69.0
제32회	2019	73.3	170.5	48.4	68.2
제33회	2020	69.9	163.9	48.8	65.6
제34회	2021	71.0	163.4	50.4	65.4
**제35회**	**2022**	**74.9**	**171.8**	**51.0**	**68.7**

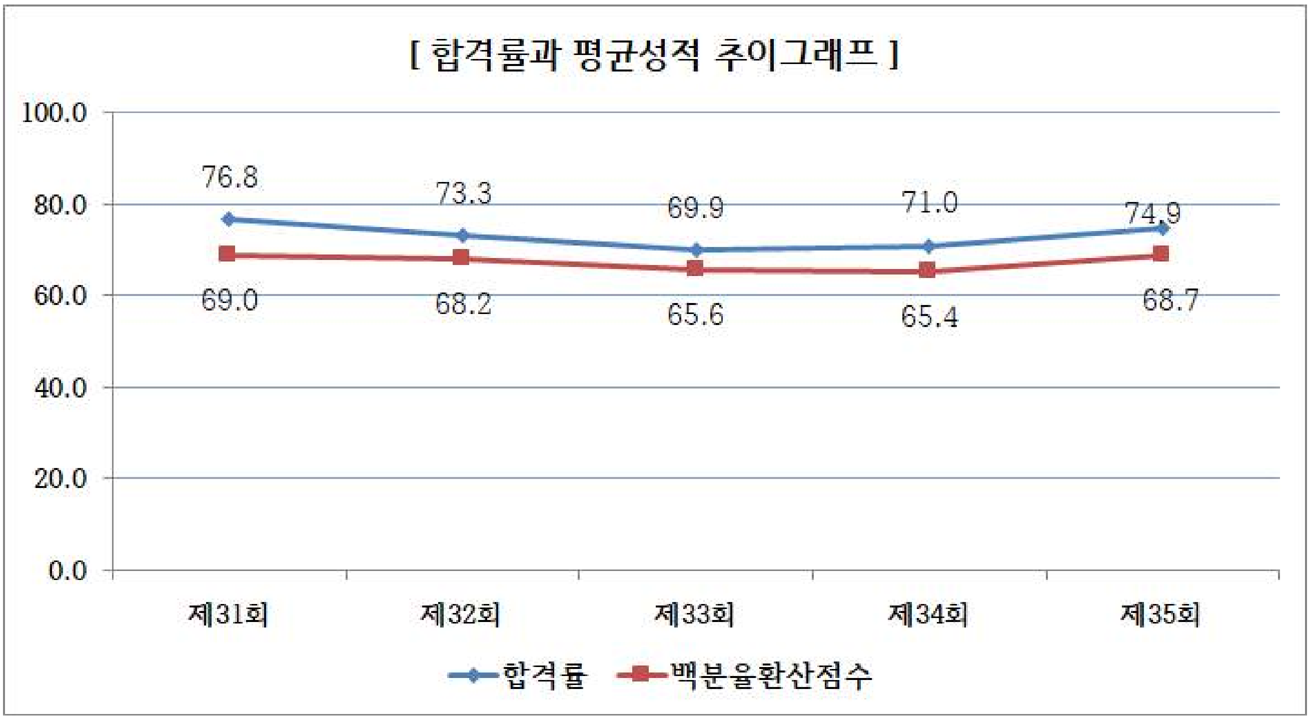

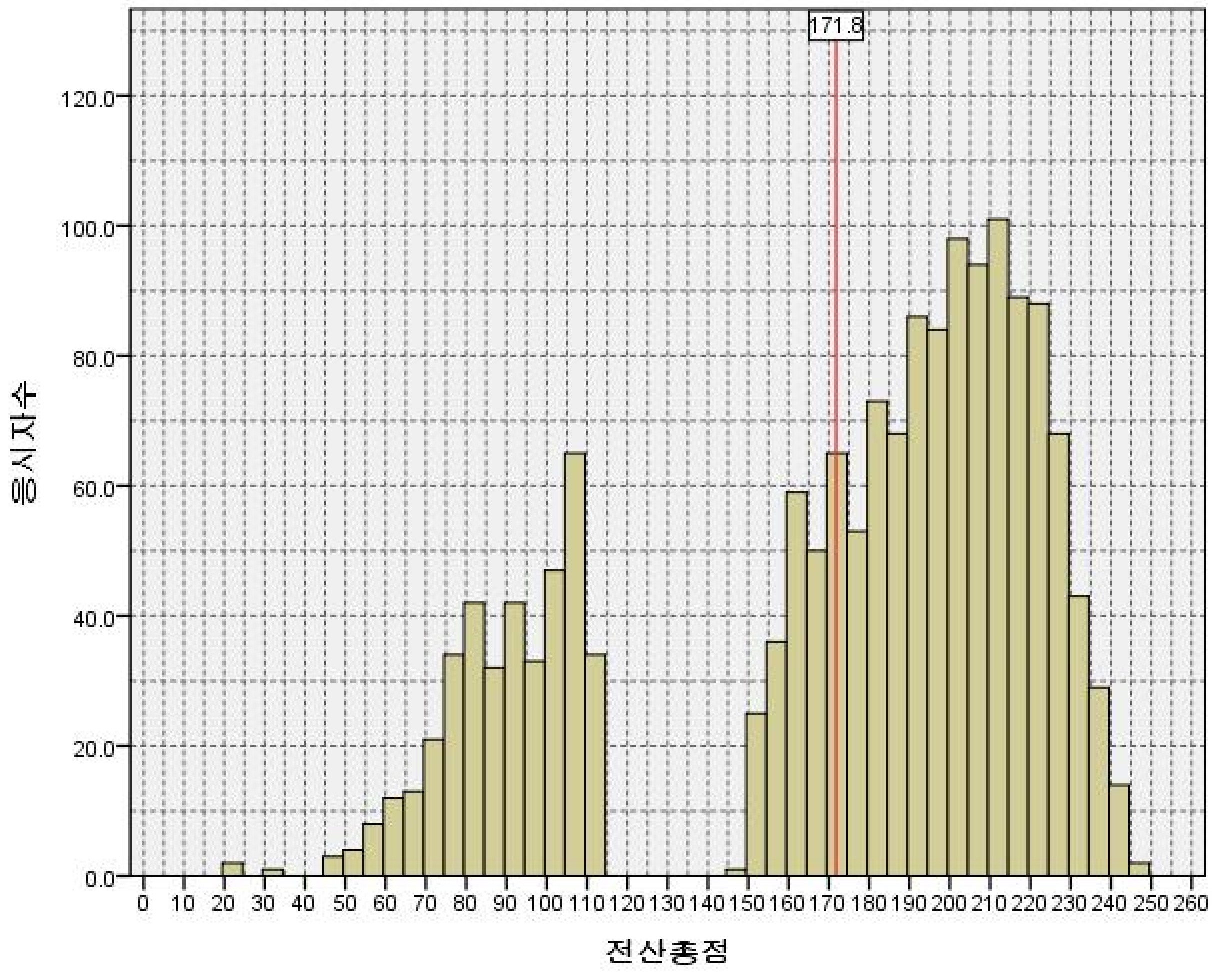

응시자	총점	합격선	평균성적	표준편차
1,619	250		171.8	51.0

※ 필기시험 불합격자의 실기성적을 포함하지 않음

※ 1,619명은 응시자(1,620명)에서 채점보류자(1명)를 제외한 수치임

해석

- 전년대비 합격률은 3.9% 증가하였고, 백분율 환산점수는 3.3 점 증가함

Ⅱ. 문항분석 결과

1. 성적

1) 전체 성적분포도

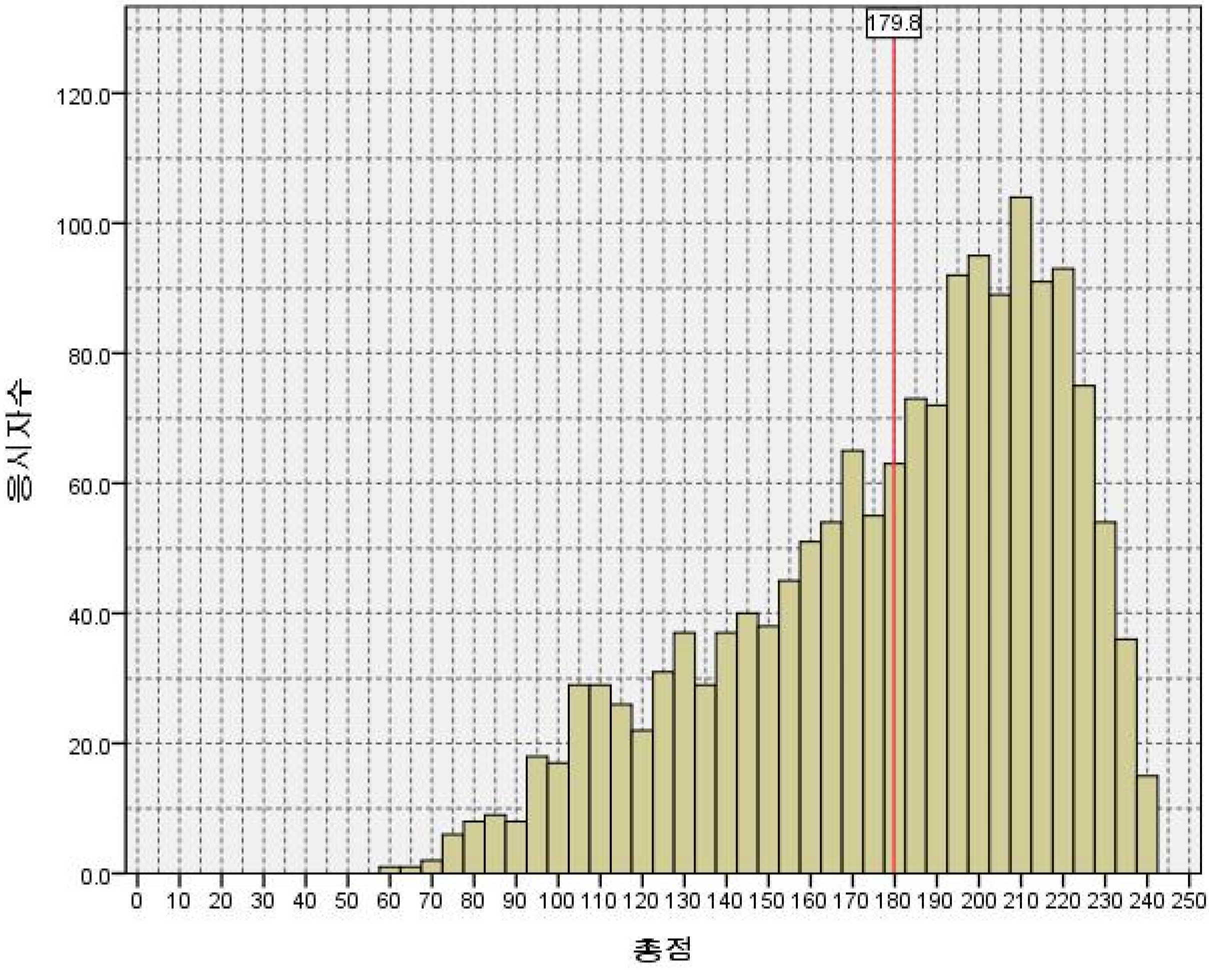

응시자	총점	합격선	평균성적	표준편차
1,617	250		179.8	39.2

※ 필기시험 불합격자의 실기성적을 포함함

※ 1,617명은 응시자(1,620명)에서 기권자(3명)를 제외한 수치임

2) 과목별 성적분포도(※ 필기형 실기 포함)

가) 시광학이론

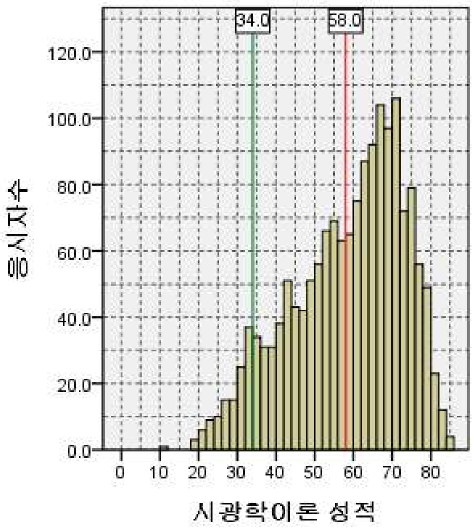

총점	과락선	평균성적	표준편차
85	34	58.0	14.5

나) 의료관계법규

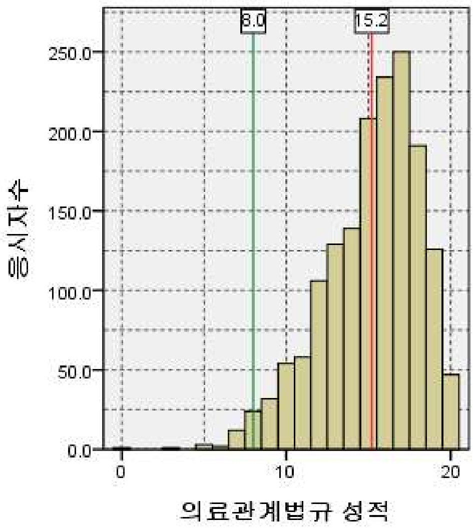

총점	과락선	평균성적	표준편차
20	8	15.2	2.9

다) 시광학응용

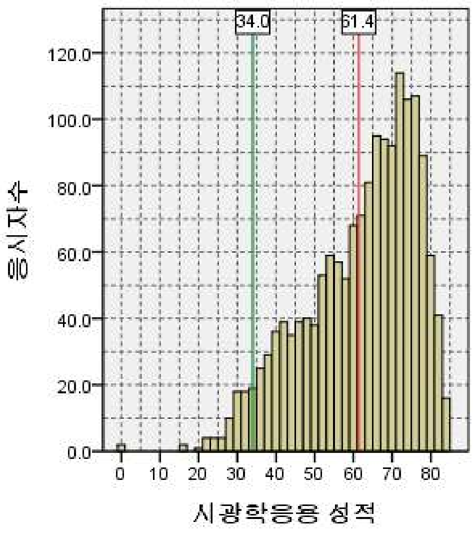

총점	과락선	평균성적	표준편차
85	34	61.4	14.3

라) 실기시험

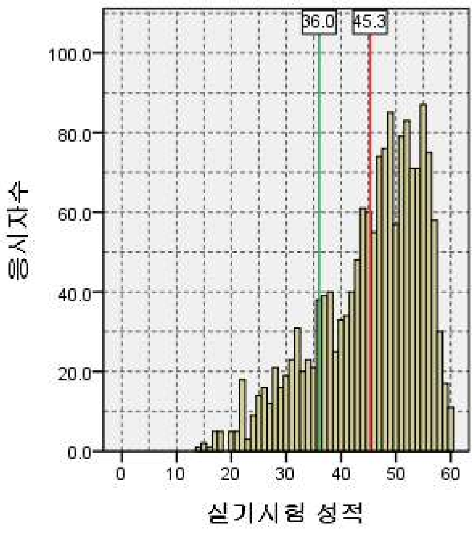

총점	과락선	평균성적	표준편차
60	36	45.3	9.6

2. 난이도와 변별도

1) 전체 난이도와 변별도

가) 전회 대비 전체 난이도와 변별도

회차	난이도		변별도1		변별도2	
	평균	표준편차	평균	표준편차	평균	표준편차
제31회	71.5	17.0	.36	.15	.35	.11
제32회	70.5	16.4	.39	.14	.37	.11
제33회	68.7	17.8	.37	.14	.35	.11
제34회	69.1	18.0	.37	.14	.36	.12
**제35회**	**71.9**	**16.4**	**.38**	**.15**	**.37**	**.12**

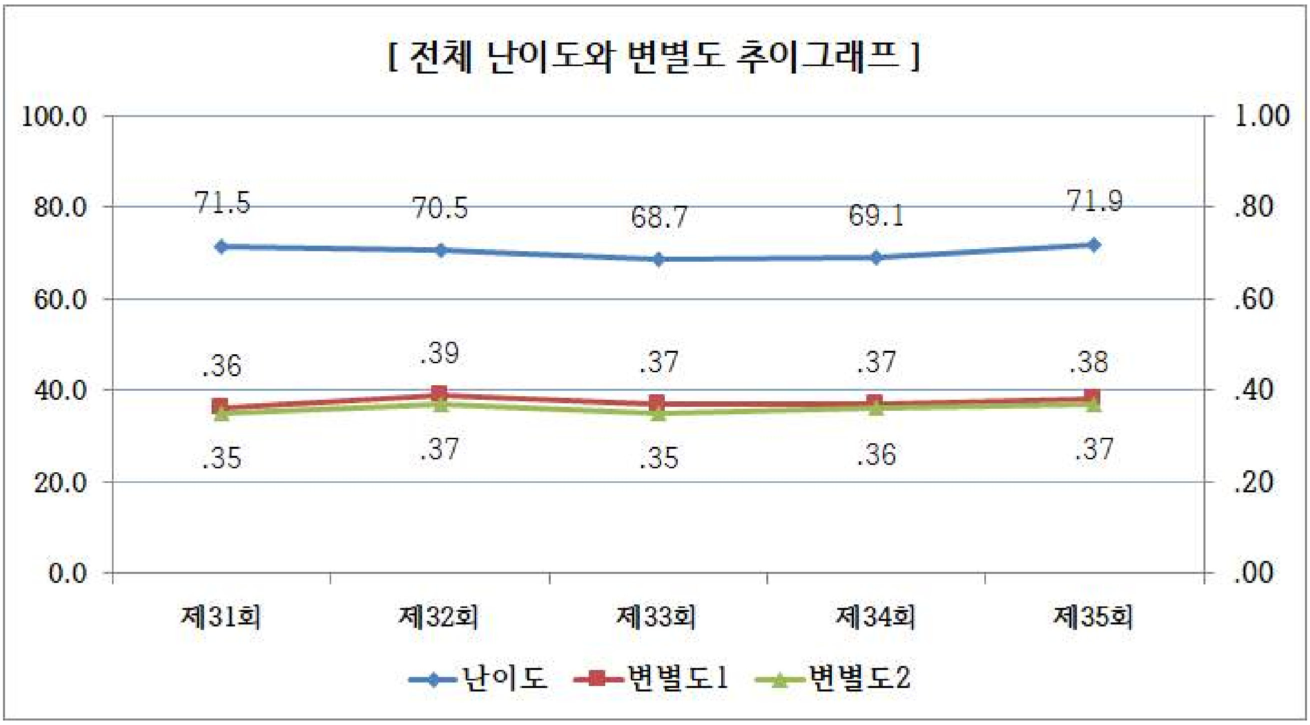

해석

- 전회 대비 난이도 지수는 2.8 증가함
- 변별도 1 지수와 변별도 2 지수는 각각 .01 씩 증가함

나) 전체 난이도와 변별도 분포도 및 비율분석

(1) 전체 난이도 분포도 및 비율분석

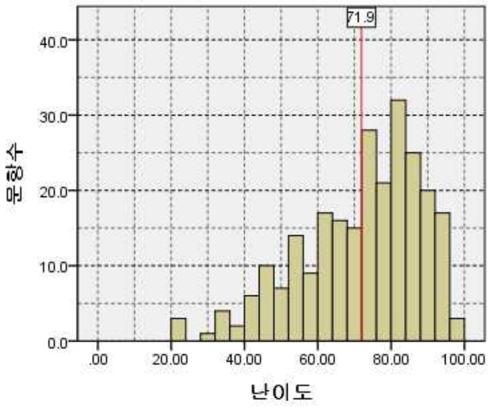

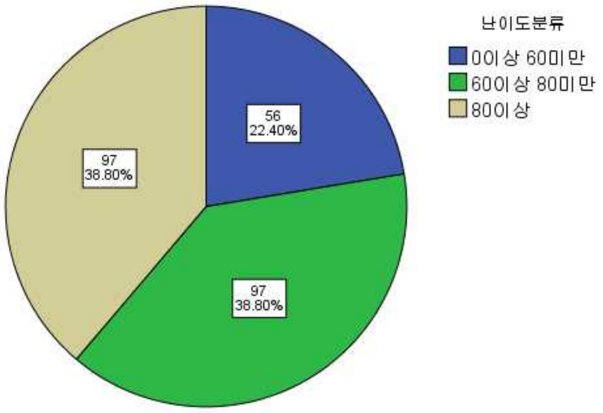

총점	난이도	표준편차
250	71.9	16.4

난이도	문항수	비율(%)
0~60미만	56	22.4
60~80미만	97	38.8
80~100	97	38.8
전체	250	100.0

(2) 전체 변별도1 분포도 및 비율분석

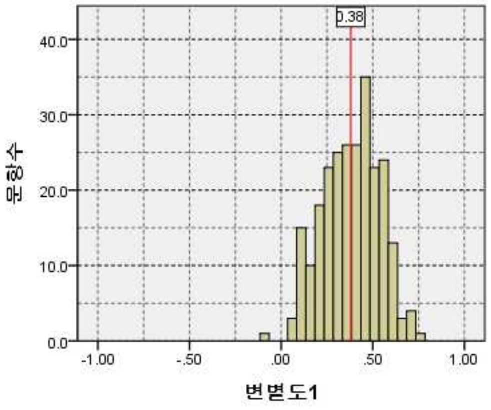

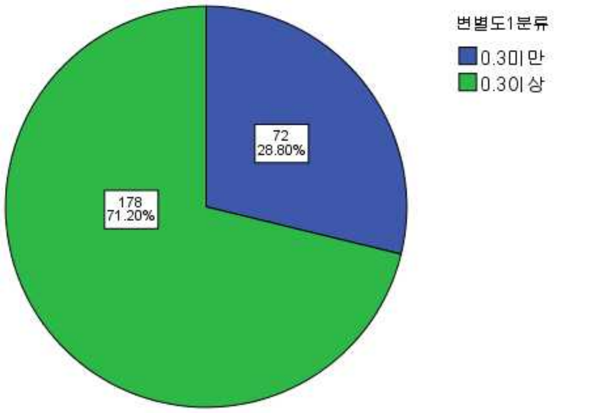

총점	변별도1	표준편차
250	.38	.15

변별도1	문항수	비율(%)
0.3미만	72	28.8
0.3이상	178	71.2
전체	250	100.0

(3) 전체 변별도2 분포도 및 비율분석

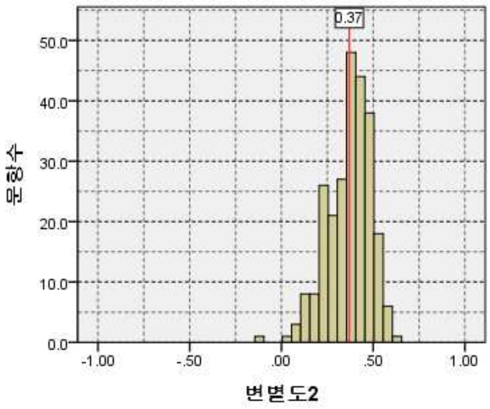

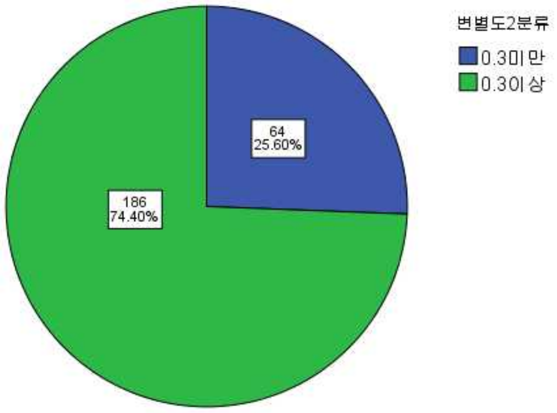

총점	변별도2	표준편차	변별도2	문항수	비율(%)
250	.37	.12	0.3미만	64	25.6
			0.3이상	186	74.4
			전체	250	100.0

해석

- 전체 250문항 중 난이도 지수가 60이상 80미만인 문항과 80이상인 문항이 각각 97문항으로 가장 많았으며, 60미만인 문항이 56문항으로 나타남
- 변별도1 지수를 기준으로 분류하였을 때, 0.3미만인 문항이 72문항으로 0.3 이상인 문항이 178문항인 것에 비해 더 적게 나타남
- 변별도2 지수를 기준으로 분류하였을 때, 0.3미만인 문항이 64문항으로 0.3 이상인 문항이 186문항인 것에 비해 더 적게 나타남

2) 과목별 난이도와 변별도

가) 전회 대비 과목별 난이도와 변별도

(1) 전회 대비 시광학이론 난이도와 변별도

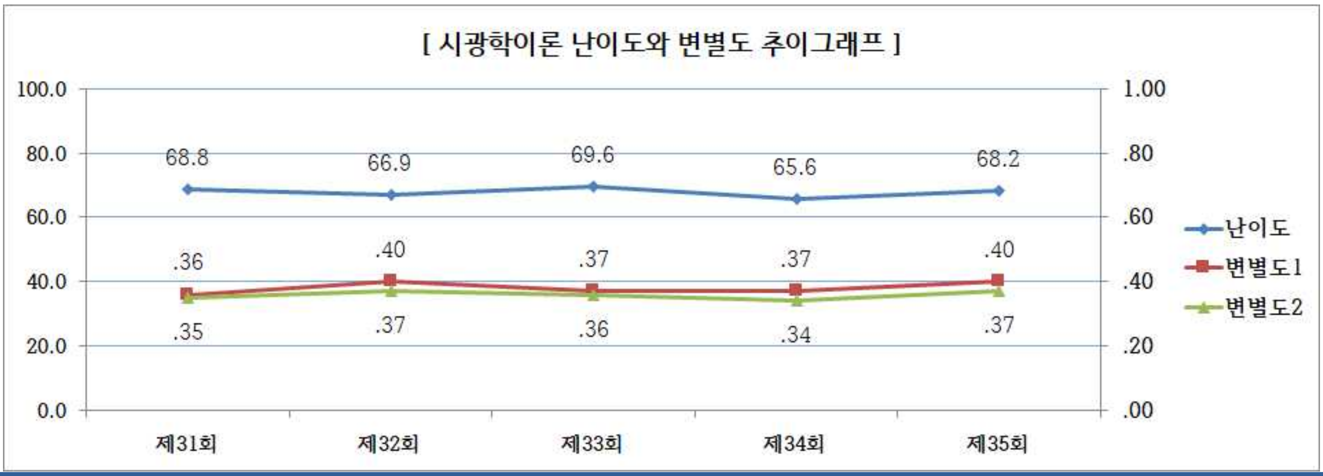

회차	난이도		변별도1		변별도2	
	평균	표준편차	평균	표준편차	평균	표준편차
제31회	68.8	18.9	.36	.15	.35	.11
제32회	66.9	17.0	.40	.13	.37	.10
제33회	69.6	19.3	.37	.14	.36	.10
제34회	65.6	19.5	.37	.14	.34	.12
**제35회**	**68.2**	**16.7**	**.40**	**.14**	**.37**	**.11**

해석

- 전회 대비 시광학이론 과목의 난이도 지수는 2.6 증가함
- 전회 대비 시광학이론 과목의 변별도 1 지수는 .03 증가함
- 전회 대비 시광학이론 과목의 변별도 2 지수는 .03 증가함

(2) 전회 대비 의료관계법규 난이도와 변별도

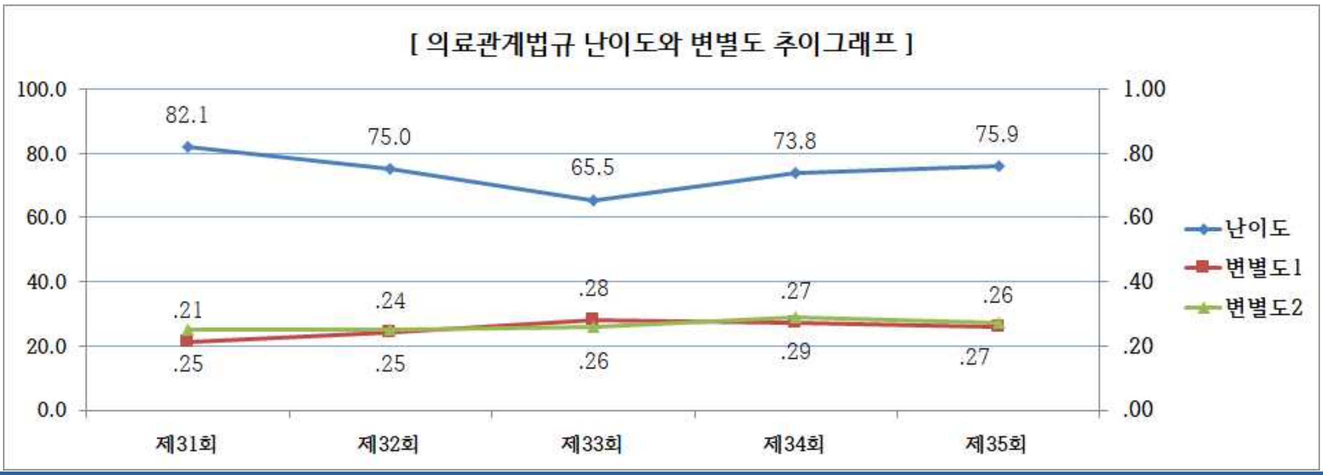

회차	난이도		변별도1		변별도2	
	평균	표준편차	평균	표준편차	평균	표준편차
제31회	82.1	13.1	.21	.13	.25	.10
제32회	75.0	16.4	.24	.12	.25	.11
제33회	65.5	23.9	.28	.12	.26	.09
제34회	73.8	19.5	.27	.14	.29	.11
**제35회**	**75.9**	**14.7**	**.26**	**.14**	**.27**	**.12**

해석

- 전회 대비 의료관계법규 과목의 난이도 지수는 2.1 증가함
- 전회 대비 의료관계법규 과목의 변별도 1 지수는 .01 감소함
- 전회 대비 의료관계법규 과목의 변별도 2 지수는 .02 감소함

(3) 전회 대비 시광학응용 난이도와 변별도

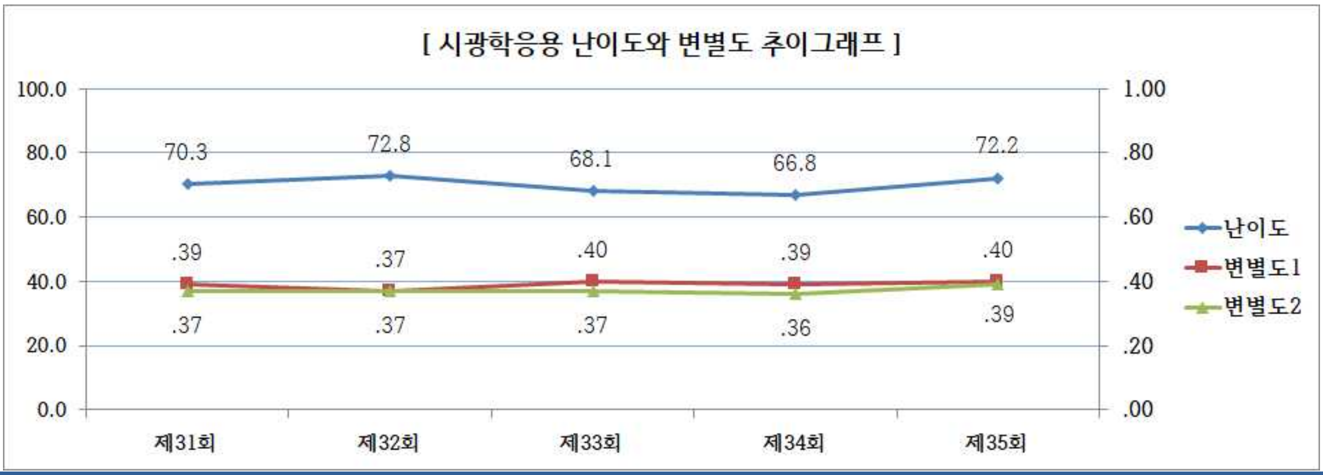

회차	난이도		변별도1		변별도2	
	평균	표준편차	평균	표준편차	평균	표준편차
제31회	70.3	17.2	.39	.14	.37	.10
제32회	72.8	17.1	.37	.16	.37	.11
제33회	68.1	15.3	.40	.14	.37	.13
제34회	66.8	17.2	.39	.14	.36	.11
**제35회**	**72.2**	**16.6**	**.40**	**.17**	**.39**	**.12**

해석

- 전회 대비 시광학응용 과목의 난이도 지수는 5.4 증가함
- 전회 대비 시광학응용 과목의 변별도 1 지수는 .01 증가함
- 전회 대비 시광학응용 과목의 변별도 2 지수는 .03 증가함

(4) 전회 대비 실기시험 난이도와 변별도

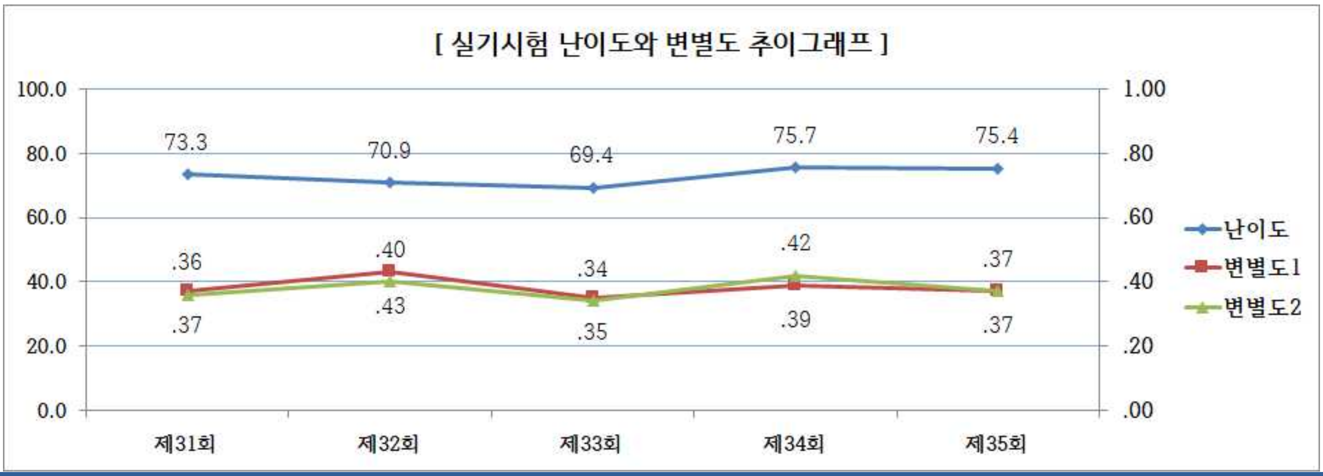

회차	난이도		변별도1		변별도2	
	평균	표준편차	평균	표준편차	평균	표준편차
제31회	73.3	13.6	.37	.13	.36	.10
제32회	70.9	13.6	.43	.11	.40	.08
제33회	69.4	16.8	.35	.14	.34	.11
제34회	75.7	14.3	.39	.14	.42	.10
**제35회**	**75.4**	**15.2**	**.37**	**.14**	**.37**	**.10**

해석

- 전회 대비 실기시험 과목의 난이도 지수는 0.3 감소함
- 전회 대비 실기시험 과목의 변별도 1 지수는 .02 감소함
- 전회 대비 실기시험 과목의 변별도 2 지수는 .05 감소함

나) 과목별 난이도와 변별도 분포도 및 비율분석

(1) 시광학이론 난이도와 변별도 분포도 및 비율분석

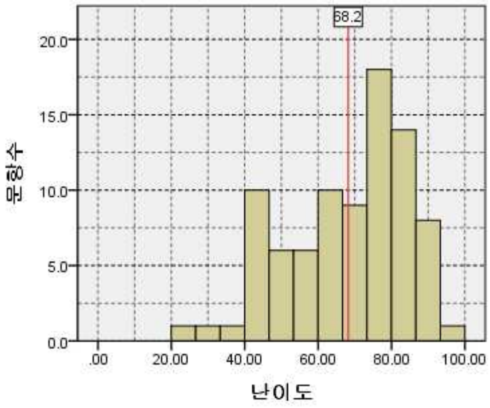

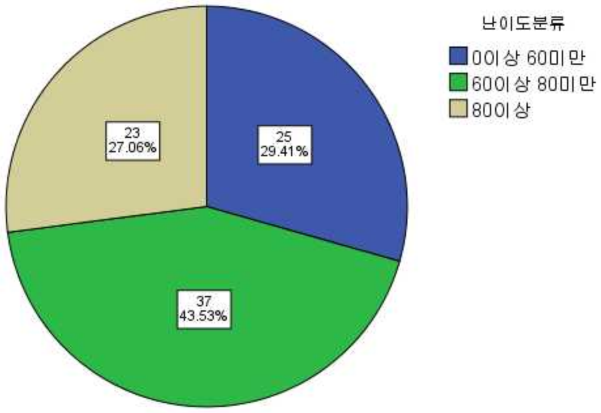

총점	난이도	표준편차
85	68.2	16.7

난이도	문항수	비율(%)
0~60미만	25	29.4
60~80미만	37	43.5
80~100	23	27.1
전체	85	100.0

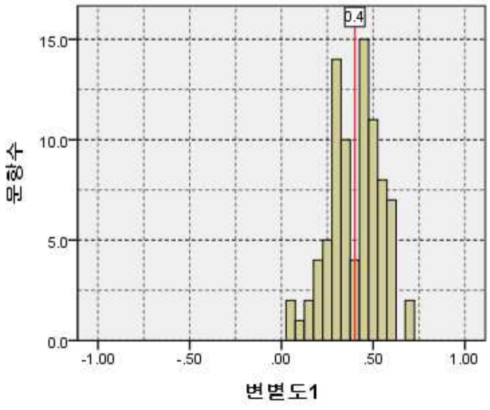

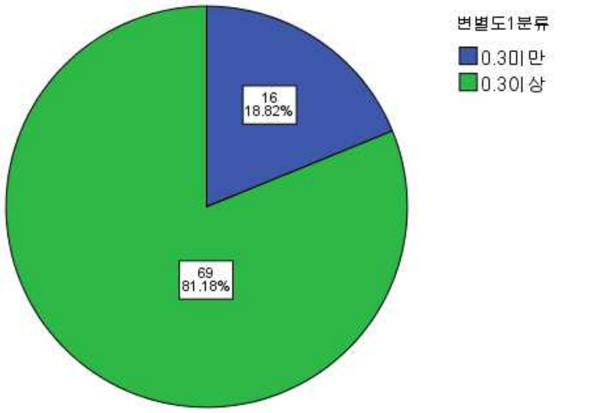

총점	변별도1	표준편차
85	.40	.14

변별도1	문항수	비율(%)
0.3미만	16	18.8
0.3이상	69	81.2
전체	85	100.0

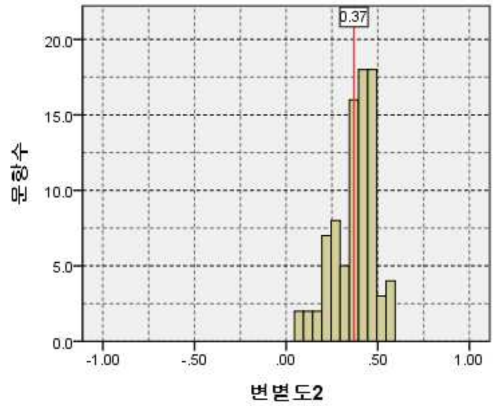

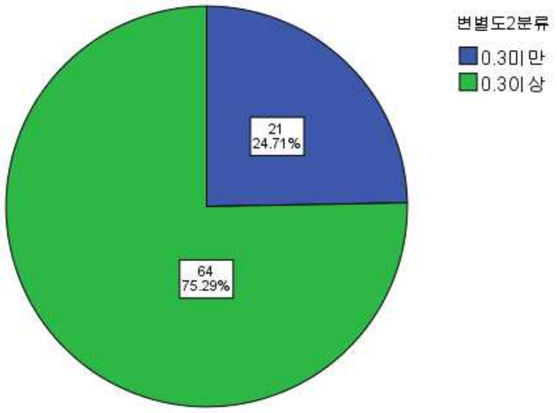

총점	변별도2	표준편차
85	.37	.11

변별도2	문항수	비율(%)
0.3미만	21	24.7
0.3이상	64	75.3
전체	85	100.0

해석

- 시광학이론 과목에서 난이도 지수가 60 이상 80 미만인 문항이 37 문항으로 가장 많았으며, 60 미만인 문항이 25 문항, 80 이상인 문항이 23 문항으로 나타남
- 변별도 1 지수를 기준으로 분류하였을 때, 0.3 미만인 문항이 16 문항으로 0.3 이상인 문항이 69 문항인 것에 비해 더 적게 나타남
- 변별도 2 지수를 기준으로 분류하였을 때, 0.3 미만인 문항이 21 문항으로 0.3 이상인 문항이 64 문항인 것에 비해 더 적게 나타남

(2) 의료관계법규 난이도와 변별도 분포도 및 비율분석

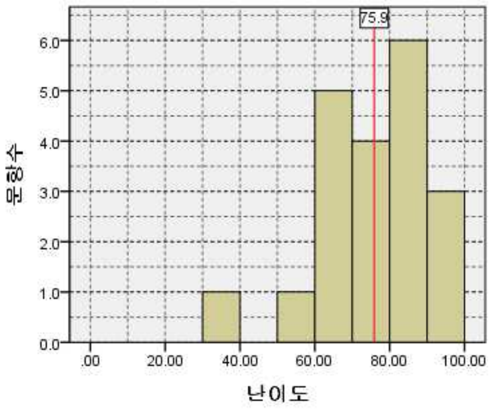

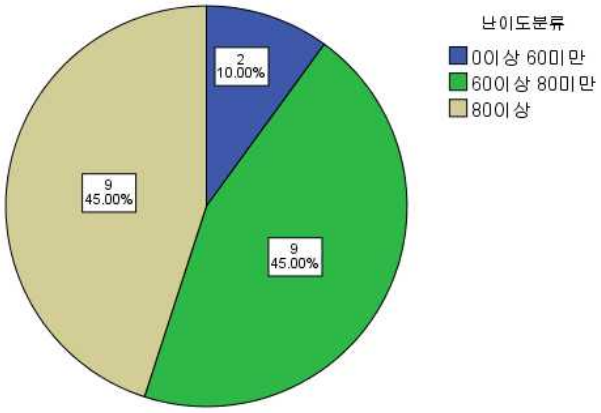

총점	난이도	표준편차
20	75.9	14.7

난이도	문항수	비율(%)
0~60미만	2	10.0
60~80미만	9	45.0
80~100	9	45.0
전체	20	100.0

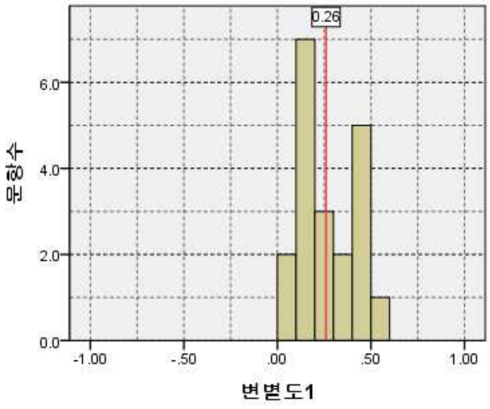

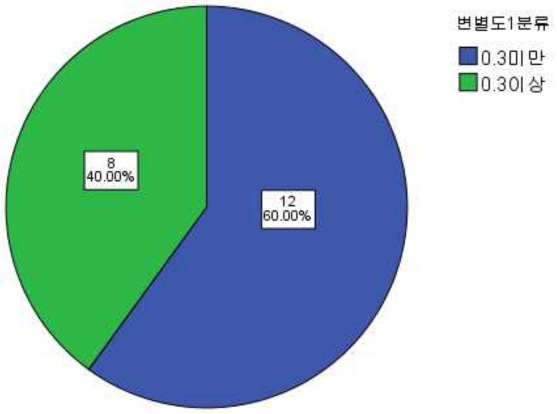

총점	변별도1	표준편차
20	.26	.14

변별도1	문항수	비율(%)
0.3미만	12	60.0
0.3이상	8	40.0
전체	20	100.0

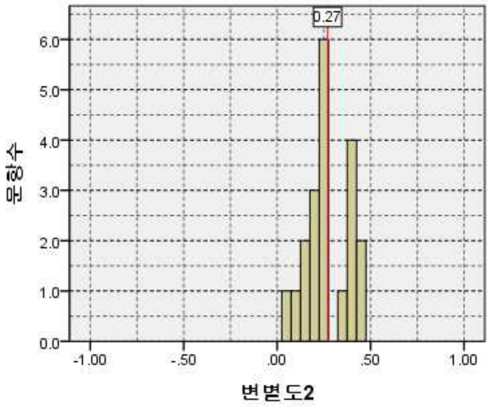

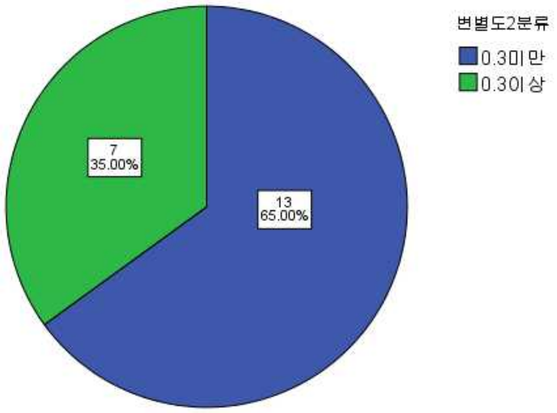

총점	변별도2	표준편차
20	.27	.12

변별도2	문항수	비율(%)
0.3미만	13	65.0
0.3이상	7	35.0
전체	20	100.0

해석

- 의료관계법규 과목에서 난이도 지수가 60 이상 80 미만인 문항과 80 이상인 문항이 각각 9 문항으로 가장 많았으며, 60 미만인 문항이 2 문항으로 나타남
- 변별도 1 지수를 기준으로 분류하였을 때, 0.3 미만인 문항이 12 문항으로 0.3 이상인 문항이 8 문항인 것에 비해 더 많이 나타남
- 변별도 2 지수를 기준으로 분류하였을 때, 0.3 미만인 문항이 13 문항으로 0.3 이상인 문항이 7 문항인 것에 비해 더 많이 나타남

(3) 시광학응용 난이도와 변별도 분포도 및 비율분석

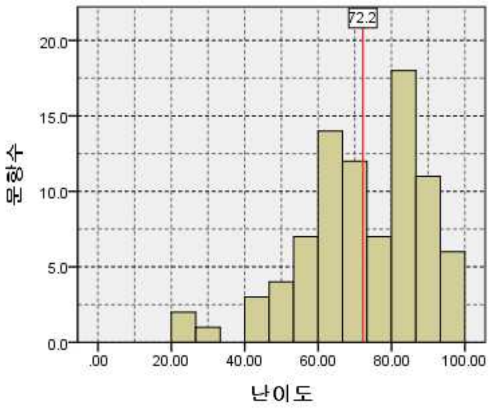

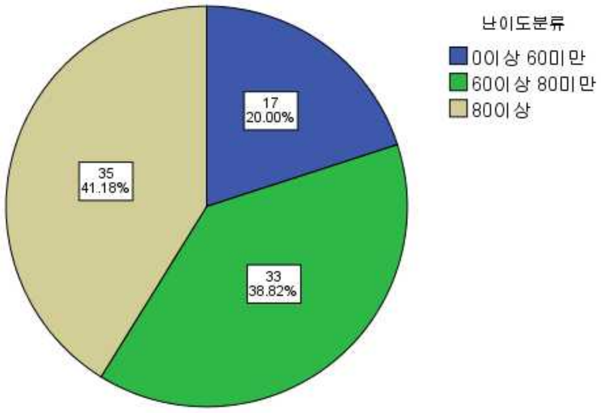

총점	난이도	표준편차	난이도	문항수	비율(%)
85	72.2	16.6	0~60미만	17	20.0
			60~80미만	33	38.8
			80~100	35	41.2
			전체	85	100.0

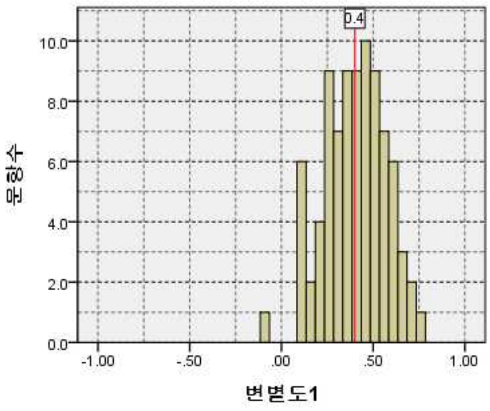

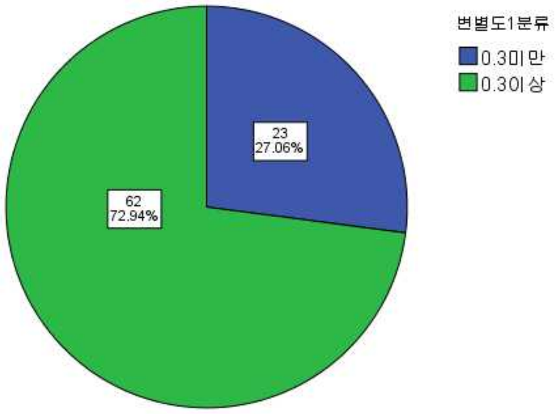

총점	변별도1	표준편차	변별도1	문항수	비율(%)
85	.40	.17	0.3미만	23	27.1
			0.3이상	62	72.9
			전체	85	100.0

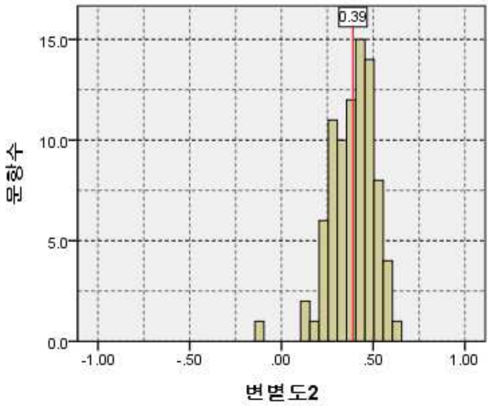

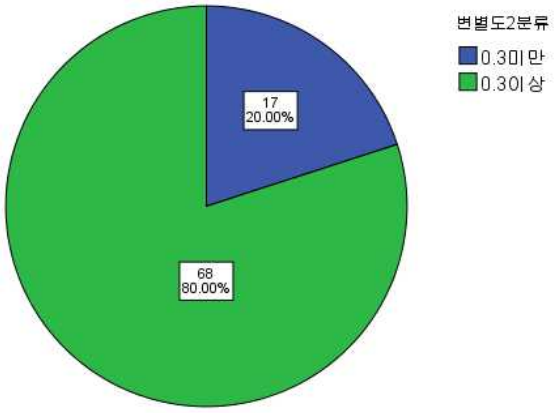

총점	변별도2	표준편차	변별도2	문항수	비율(%)
85	.39	.12	0.3미만	17	20.0
			0.3이상	68	80.0
			전체	85	100.0

해석

- 시광학응용 과목에서 난이도 지수가 80 이상인 문항이 35 문항으로 가장 많았으며, 60 이상 80 미만인 문항이 33 문항, 60 미만인 문항이 17 문항으로 나타남
- 변별도 1 지수를 기준으로 분류하였을 때, 0.3 미만인 문항이 23 문항으로 0.3 이상인 문항이 62 문항인 것에 비해 더 적게 나타남
- 변별도 2 지수를 기준으로 분류하였을 때, 0.3 미만인 문항이 17 문항으로 0.3 이상인 문항이 68 문항인 것에 비해 더 적게 나타남

(4) 실기시험 난이도와 변별도 분포도 및 비율분석

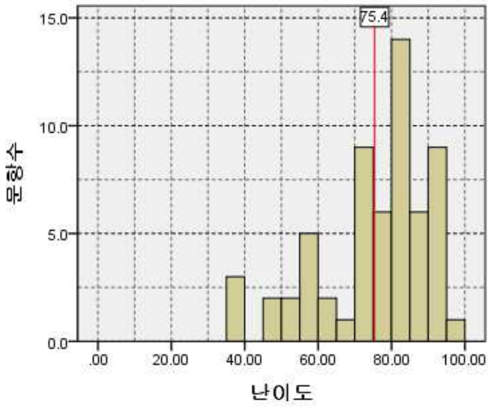

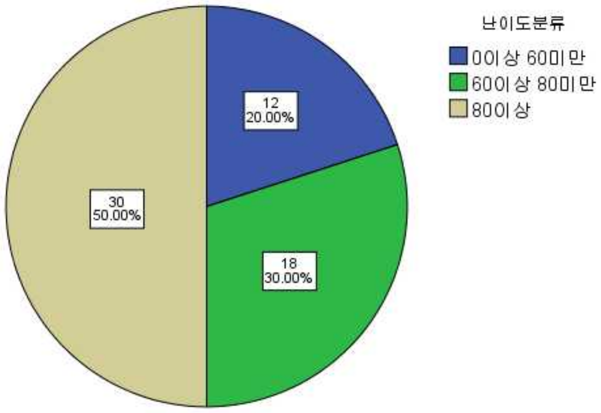

총점	난이도	표준편차
60	75.4	15.2

난이도	문항수	비율(%)
0~60미만	12	20.0
60~80미만	18	30.0
80~100	30	50.0
전체	60	100.0

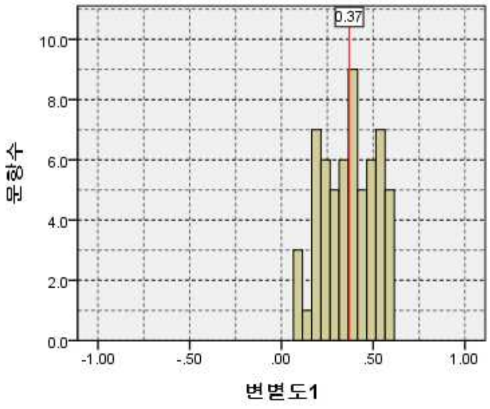

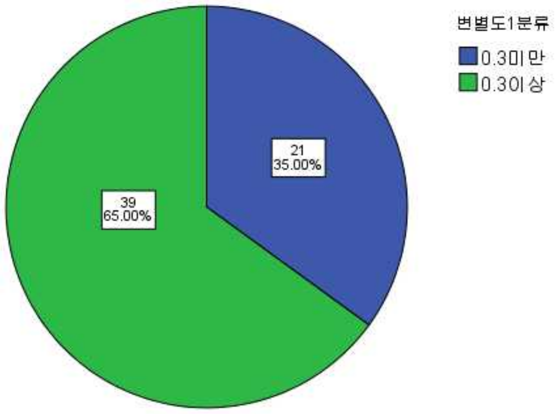

총점	변별도1	표준편차
60	.37	.14

변별도1	문항수	비율(%)
0.3미만	21	35.0
0.3이상	39	65.0
전체	60	100.0

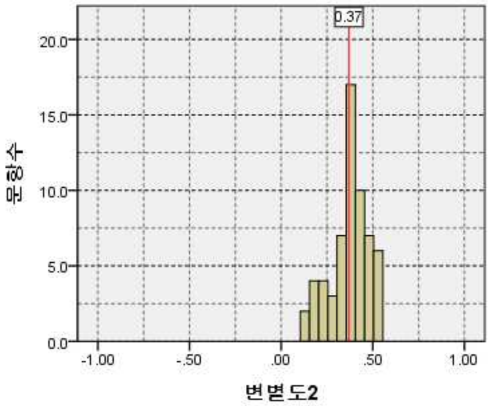

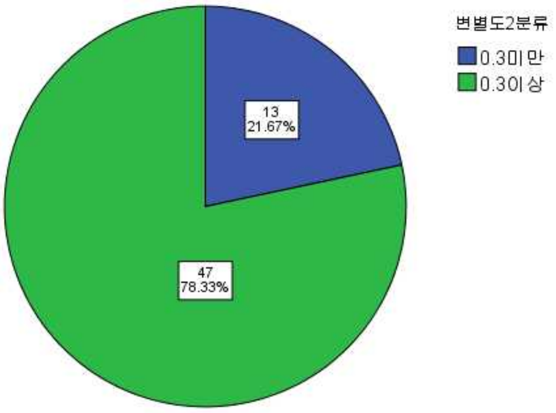

총점	변별도2	표준편차
60	.37	.10

변별도2	문항수	비율(%)
0.3미만	13	21.7
0.3이상	47	78.3
전체	60	100.0

해석

- 실기시험 과목에서 난이도 지수가 80 이상인 문항이 30 문항으로 가장 많았으며, 60 이상 80 미만인 문항이 18 문항, 60 미만인 문항이 12 문항으로 나타남
- 변별도 1 지수를 기준으로 분류하였을 때, 0.3 미만인 문항이 21 문항으로 0.3 이상인 문항이 39 문항인 것에 비해 더 적게 나타남
- 변별도 2 지수를 기준으로 분류하였을 때, 0.3 미만인 문항이 13 문항으로 0.3 이상인 문항이 47 문항인 것에 비해 더 적게 나타남

3) 지식수준별 난이도와 변별도

가) 전회 대비 지식수준별 난이도와 변별도

(1) 전회 대비 암기형 난이도와 변별도

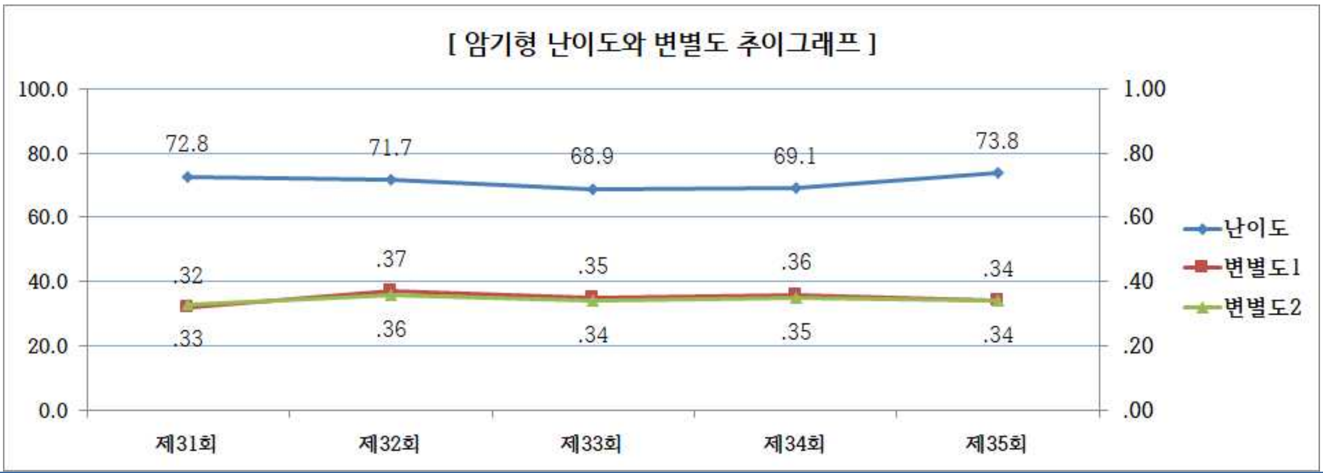

회차	난이도		변별도1		변별도2	
	평균	표준편차	평균	표준편차	평균	표준편차
제31회	72.8	18.7	.32	.14	.33	.11
제32회	71.7	17.2	.37	.14	.36	.11
제33회	68.9	19.5	.35	.14	.34	.11
제34회	69.1	18.4	.36	.14	.35	.11
**제35회**	**73.8**	**15.8**	**.34**	**.15**	**.34**	**.11**

해석

- 전회 대비 암기형 문항의 난이도 지수는 4.7 증가함
- 전회 대비 암기형 문항의 변별도 1 지수는 .02 감소함
- 전회 대비 암기형 문항의 변별도 2 지수는 .01 감소함

(2) 전회 대비 해석형 난이도와 변별도

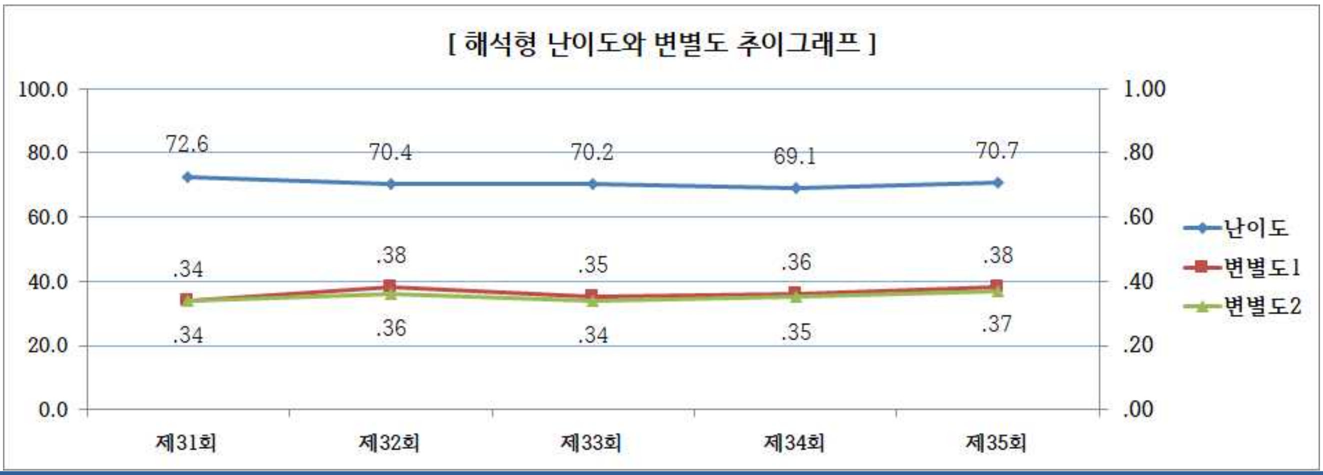

회차	난이도		변별도1		변별도2	
	평균	표준편차	평균	표준편차	평균	표준편차
제31회	72.6	16.2	.34	.15	.34	.11
제32회	70.4	16.3	.38	.14	.36	.10
제33회	70.2	18.3	.35	.15	.34	.12
제34회	69.1	19.0	.36	.14	.35	.11
**제35회**	**70.7**	**17.9**	**.38**	**.15**	**.37**	**.12**

해석

- 전회 대비 해석형 문항의 난이도 지수는 1.6 증가함
- 전회 대비 해석형 문항의 변별도 1 지수는 .02 증가함
- 전회 대비 해석형 문항의 변별도 2 지수는 .02 증가함

(3) 전회 대비 해결형 난이도와 변별도

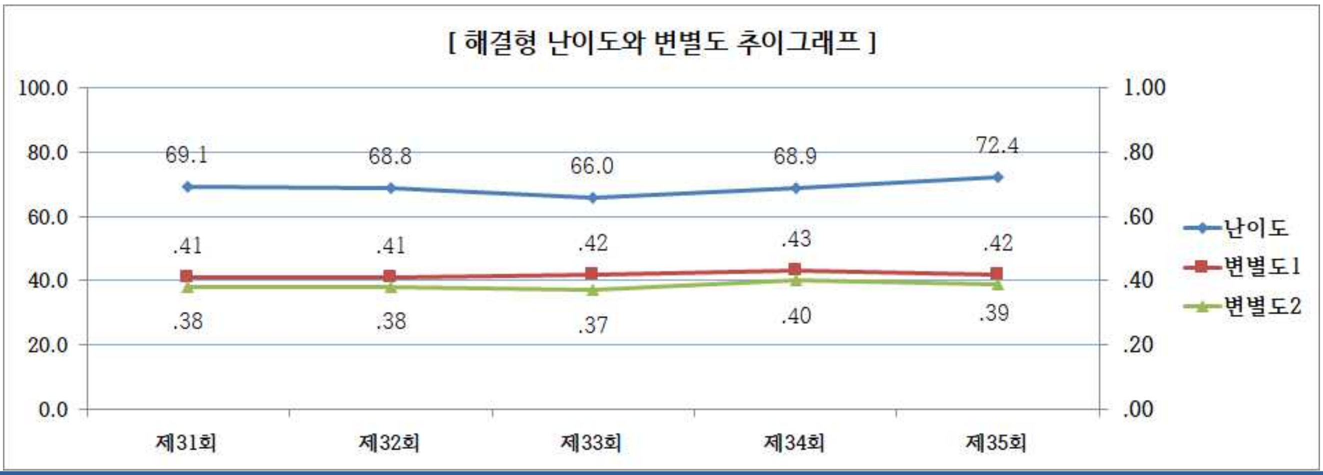

회차	난이도		변별도1		변별도2	
	평균	표준편차	평균	표준편차	평균	표준편차
제31회	69.1	16.1	.41	.14	.38	.10
제32회	68.8	15.3	.41	.15	.38	.11
제33회	66.0	13.6	.42	.13	.37	.11
제34회	68.9	15.1	.43	.14	.40	.12
**제35회**	**72.4**	**13.7**	**.42**	**.15**	**.39**	**.11**

해석

- 전회 대비 해결형 문항의 난이도 지수는 3.5 증가함
- 전회 대비 해결형 문항의 변별도 1 지수는 .01 감소함
- 전회 대비 해결형 문항의 변별도 2 지수는 .01 감소함

나) 지식수준별 난이도와 변별도 분포도 및 비율분석

(1) 암기형 난이도와 변별도 분석도 및 비율분석

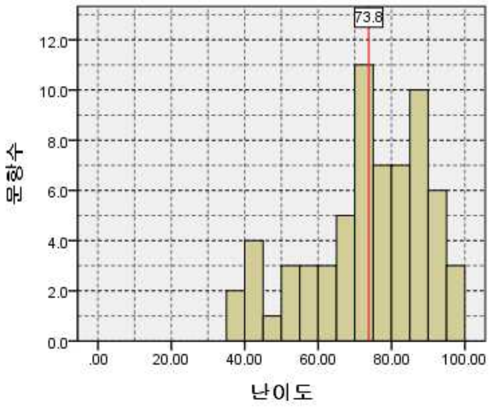

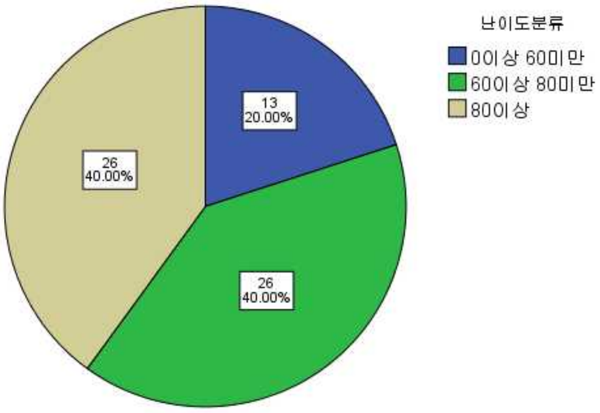

총점	난이도	표준편차
65	73.8	15.8

난이도	문항수	비율(%)
0~60미만	13	20.0
60~80미만	26	40.0
80~100	26	40.0
전체	65	100.0

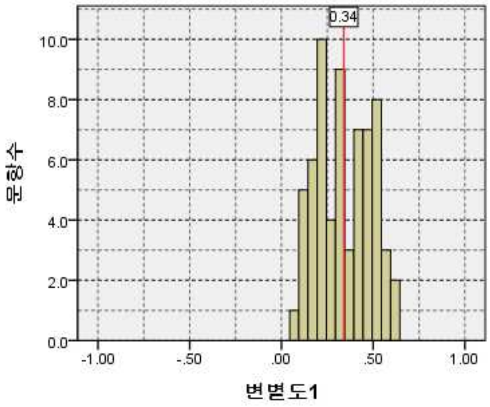

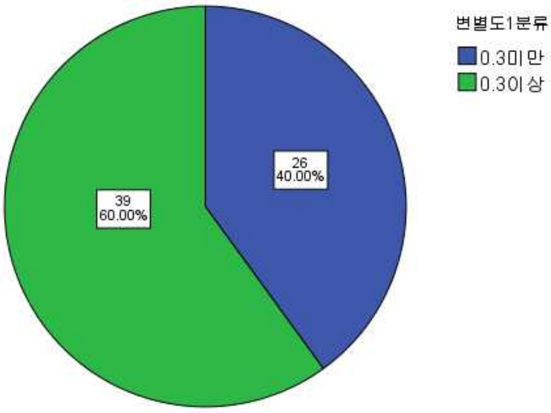

총점	변별도1	표준편차
65	.34	.15

변별도1	문항수	비율(%)
0.3미만	26	40.0
0.3이상	39	60.0
전체	65	100.0

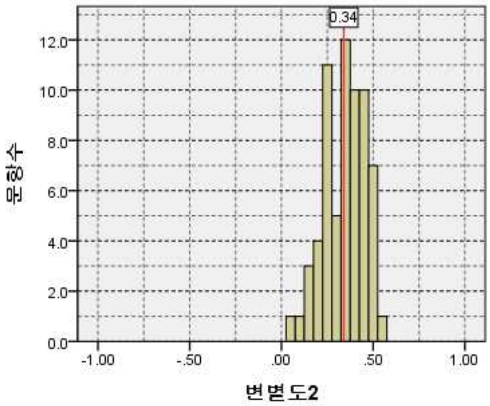

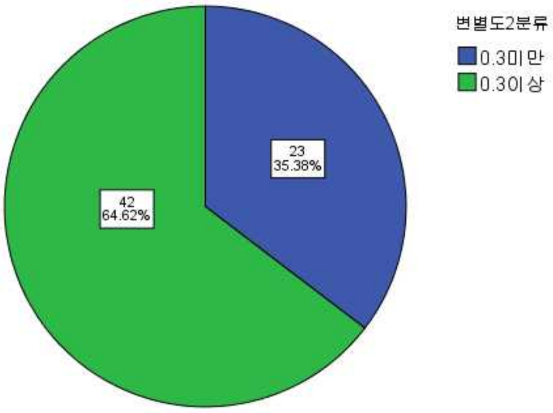

총점	변별도2	표준편차	변별도2	문항수	비율(%)
65	.34	.11	0.3미만	23	35.4
			0.3이상	42	64.6
			전체	65	100.0

해석

- 암기형 문항에서 난이도 지수가 60 이상 80 미만인 문항과 80 이상인 문항이 각각 26 문항으로 가장 많았으며, 60 미만인 문항이 13 문항으로 나타남
- 변별도 1 지수를 기준으로 분류하였을 때, 0.3 미만인 문항이 26 문항으로 0.3 이상인 문항이 39 문항인 것에 비해 더 적게 나타남
- 변별도 2 지수를 기준으로 분류하였을 때, 0.3 미만인 문항이 23 문항으로 0.3 이상인 문항이 42 문항인 것에 비해 더 적게 나타남

(2) 해석형 난이도와 변별도 분포도 및 비율분석

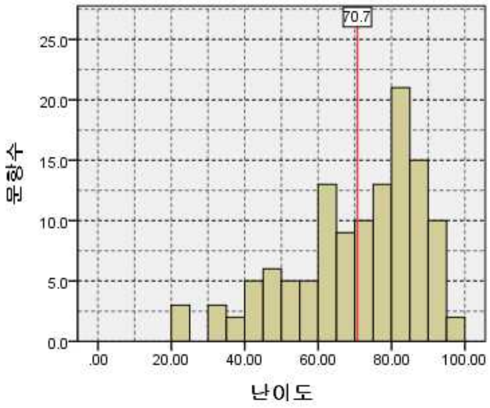

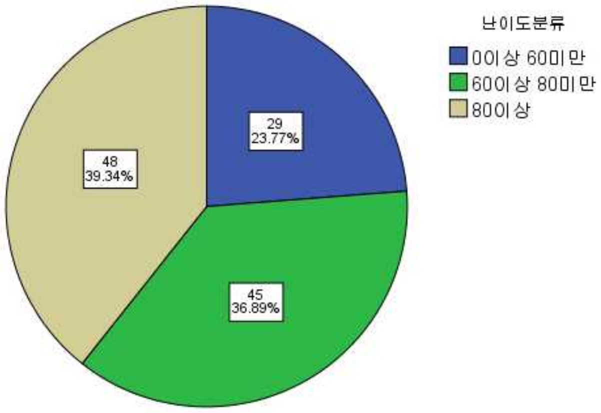

총점	난이도	표준편차
122	70.7	17.9

난이도	문항수	비율(%)
0~60미만	29	23.8
60~80미만	45	36.9
80~100	48	39.3
전체	122	100.0

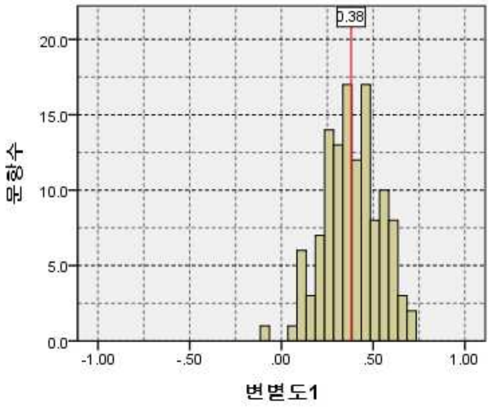

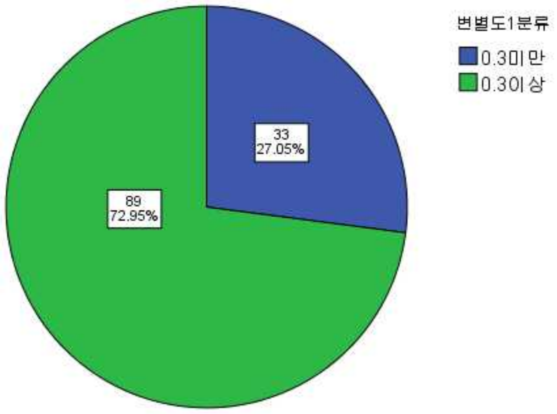

총점	변별도1	표준편차
122	.38	.15

변별도1	문항수	비율(%)
0.3미만	33	27.0
0.3이상	89	73.0
전체	122	100.0

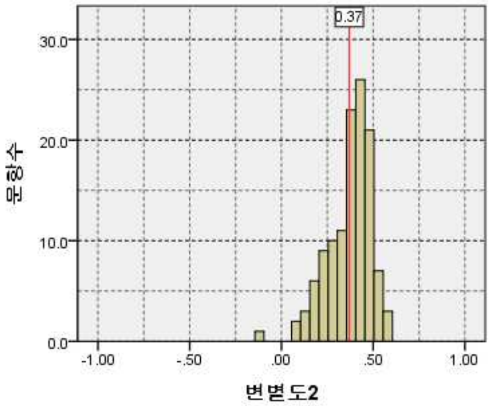

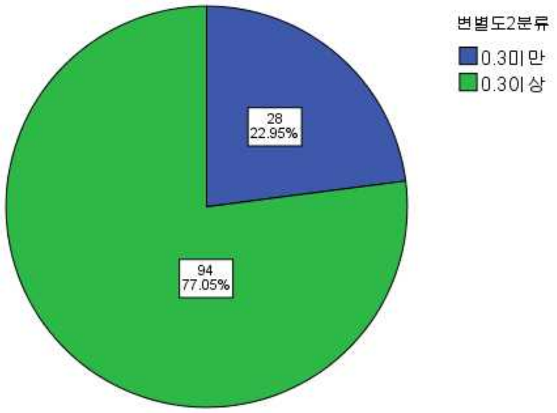

총점	변별도2	표준편차
122	.37	.12

변별도2	문항수	비율(%)
0.3미만	28	23.0
0.3이상	94	77.0
전체	122	100.0

해석

- 해석형 문항에서 난이도 지수가 80 이상인 문항이 48 문항으로 가장 많았으며, 60 이상 80 미만인 문항이 45 문항, 60 미만인 문항이 29 문항으로 나타남
- 변별도 1 지수를 기준으로 분류하였을 때, 0.3 미만인 문항이 33 문항으로 0.3 이상인 문항이 89 문항인 것에 비해 더 적게 나타남
- 변별도 2 지수를 기준으로 분류하였을 때, 0.3 미만인 문항이 28 문항으로 0.3 이상인 문항이 94 문항인 것에 비해 더 적게 나타남

(3) 해결형 난이도와 변별도 분포도 및 비율분석

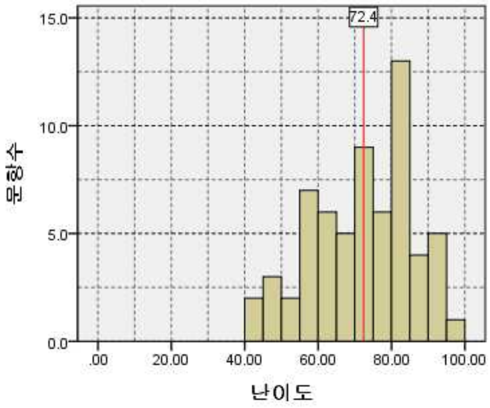

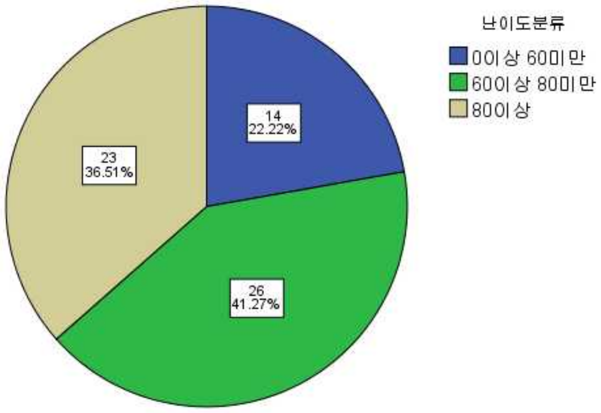

총점	난이도	표준편차
63	72.4	13.7

난이도	문항수	비율(%)
0~60미만	14	22.2
60~80미만	26	41.3
80~100	23	36.5
전체	63	100.0

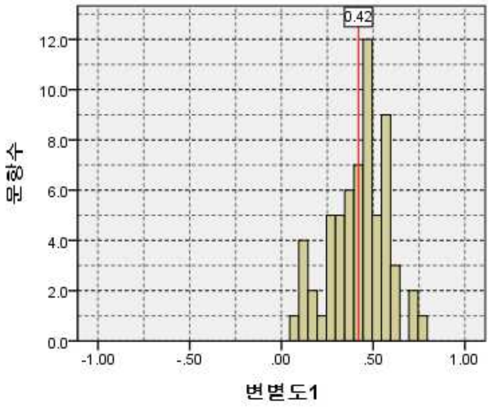

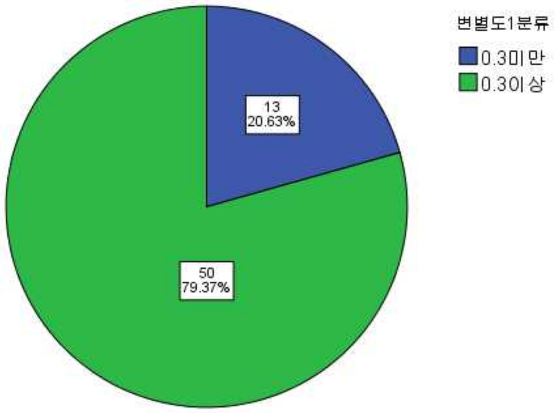

총점	변별도1	표준편차
63	.42	.15

변별도1	문항수	비율(%)
0.3미만	13	20.6
0.3이상	50	79.4
전체	63	100.0

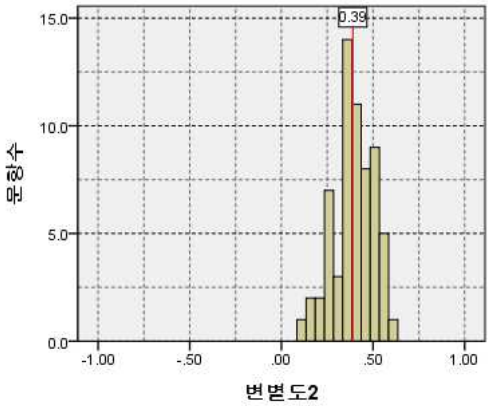

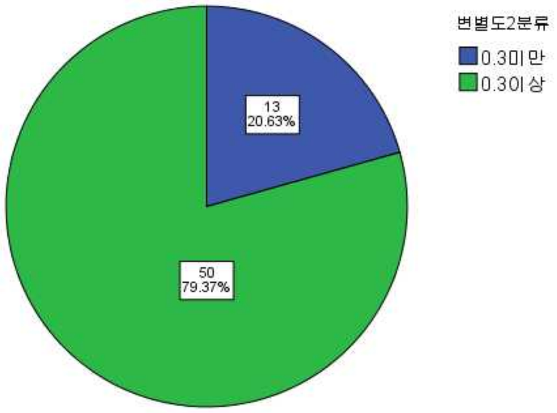

총점	변별도2	표준편차	변별도2	문항수	비율(%)
63	.39	.11	0.3미만	13	20.6
			0.3이상	50	79.4
			전체	63	100.0

해석

- 해결형 문항에서 난이도 지수가 60 이상 80 미만인 문항이 26 문항으로 가장 많았으며, 80 이상인 문항이 23 문항, 60 미만인 문항이 14 문항으로 나타남
- 변별도 1 지수를 기준으로 분류하였을 때, 0.3 미만인 문항이 13 문항으로 0.3 이상인 문항이 50 문항인 것에 비해 더 적게 나타남
- 변별도 2 지수를 기준으로 분류하였을 때, 0.3 미만인 문항이 13 문항으로 0.3 이상인 문항이 50 문항인 것에 비해 더 적게 나타남

3. 난이도와 변별도 간 산포도

1) 전체 난이도와 변별도 간 산포도

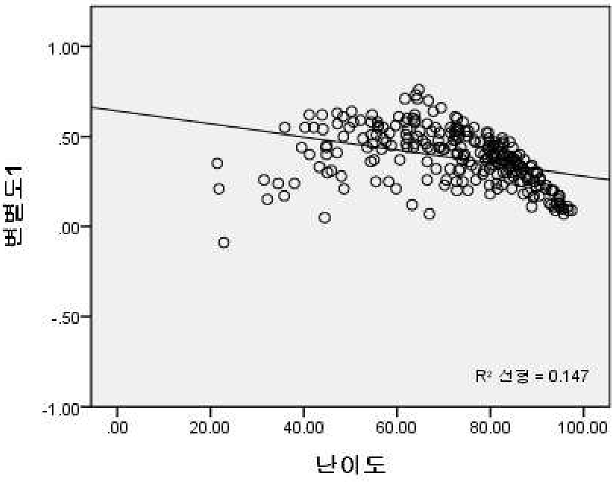

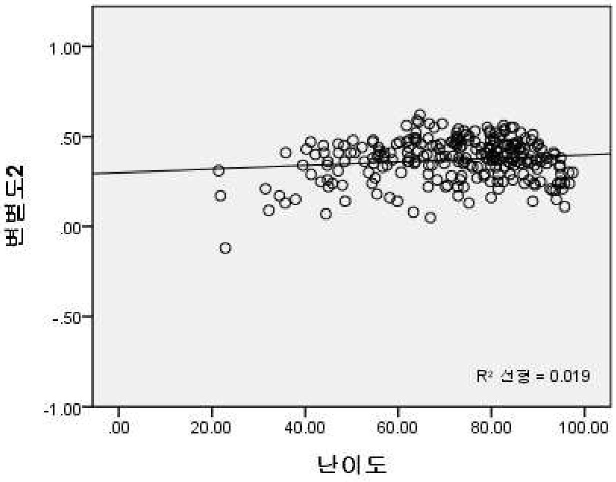

해석

- 전체 문항을 대상으로 한 난이도 지수와 변별도 1 지수 간 상관은 -.384**으로 난이도 지수가 높을수록 변별력이 낮아지는 것으로 나타남
- 난이도 지수와 변별도 2 지수 간 상관은 .138*로 난이도 지수가 높을수록 변별력이 높아지는 것으로 나타남

2) 과목별 난이도와 변별도 간 산포도

가) 시광학이론 난이도와 변별도 간 산포도

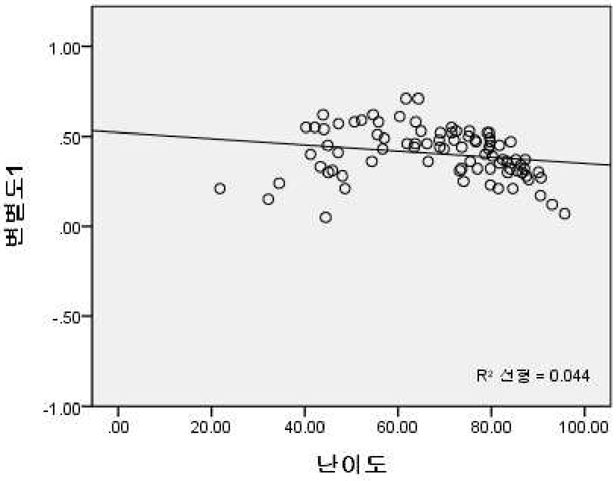

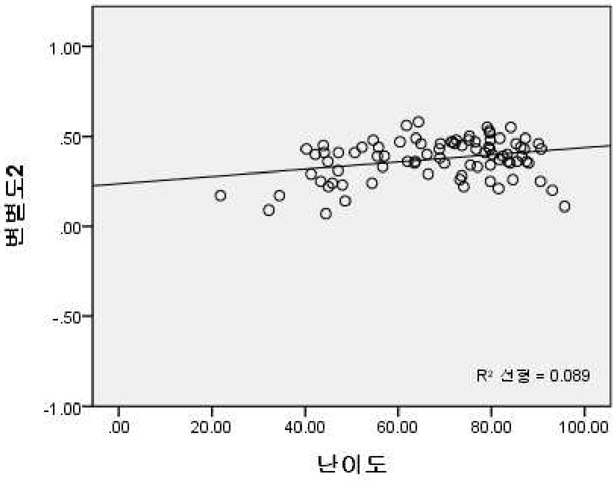

해석

- 시광학이론 과목 문항을 대상으로 한 난이도 지수와 변별도 1 지수 간 상관은 -.209 로 관련성이 없는 것으로 나타남
- 난이도 지수와 변별도 2 지수 간 상관은 .299**으로 난이도 지수가 높을수록 변별력이 높아지는 것으로 나타남

나) 의료관계법규 난이도와 변별도 간 산포도

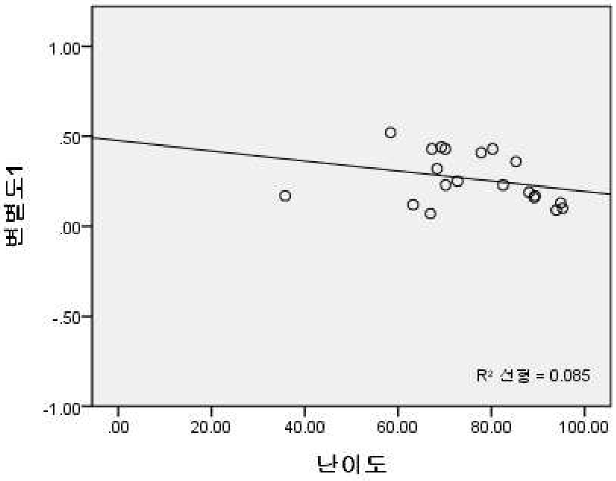

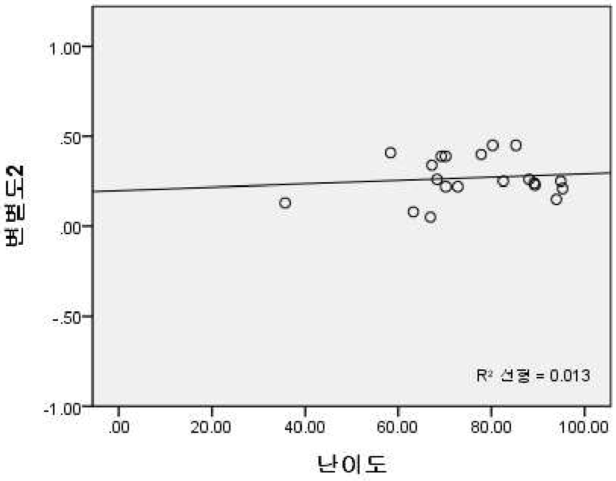

해석

- 의료관계법규 과목 문항을 대상으로 한 난이도 지수와 변별도 1 지수 간 상관은 -.292 로 관련성이 없는 것으로 나타남
- 난이도 지수와 변별도 2 지수 간 상관은 .115 로 관련성이 없는 것으로 나타남

다) 시광학응용 난이도와 변별도 간 산포도

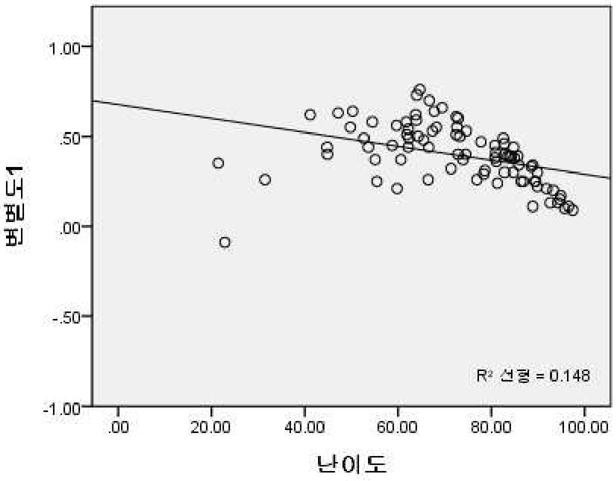

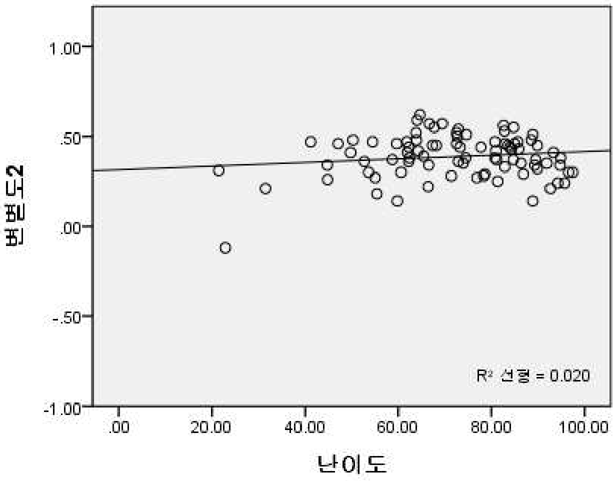

해석

- 시광학응용 과목 문항을 대상으로 한 난이도 지수와 변별도 1 지수 간 상관은 -.385**로 난이도 지수가 높을수록 변별력이 낮아지는 것으로 나타남
- 난이도 지수와 변별도 2 지수 간 상관은 .141 로 관련성이 없는 것으로 나타남

라) 실기시험 난이도와 변별도 간 산포도

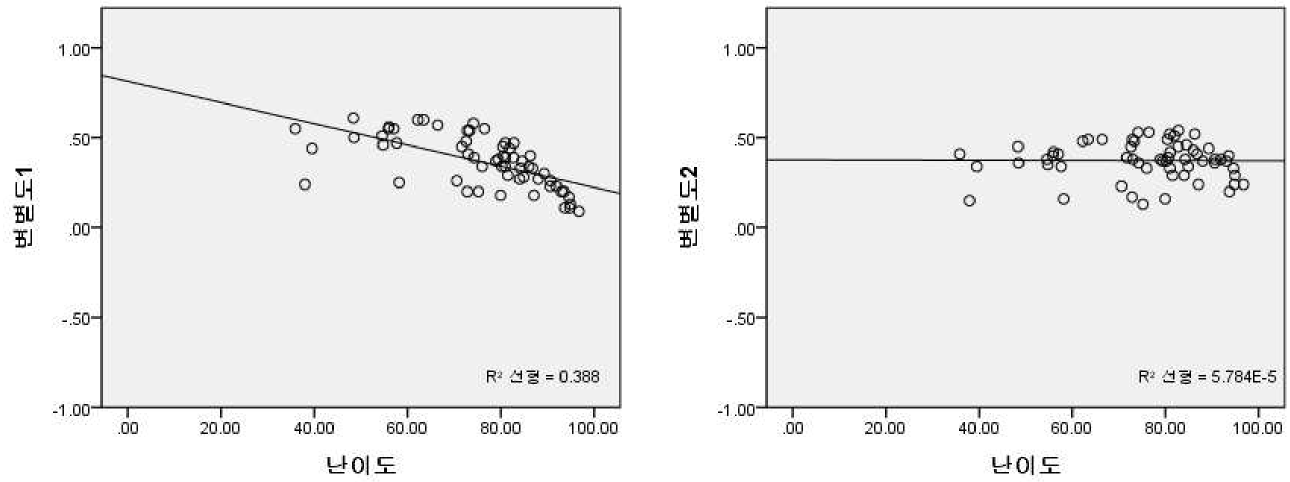

해석

- 실기시험 과목 문항을 대상으로 한 난이도 지수와 변별도1 지수 간 상관은 -.623**로 난이도 지수가 높을수록 변별력이 낮아지는 것으로 나타남
- 난이도 지수와 변별도2 지수 간 상관은 -.008로 관련성이 없는 것으로 나타남

4. 신뢰도 분석

과목명	문항수	제31회	제32회	제33회	제34회	제35회
전체	250	.973	.976	.972	.974	**.975**
시광학이론	85	.925	.933	.928	.921	**.935**
의료관계법규	20	.664	.644	.653	.676	**.656**
시광학응용	85	.933	.937	.932	.926	**.939**
실기시험	60	.905	.922	.886	.928	**.908**

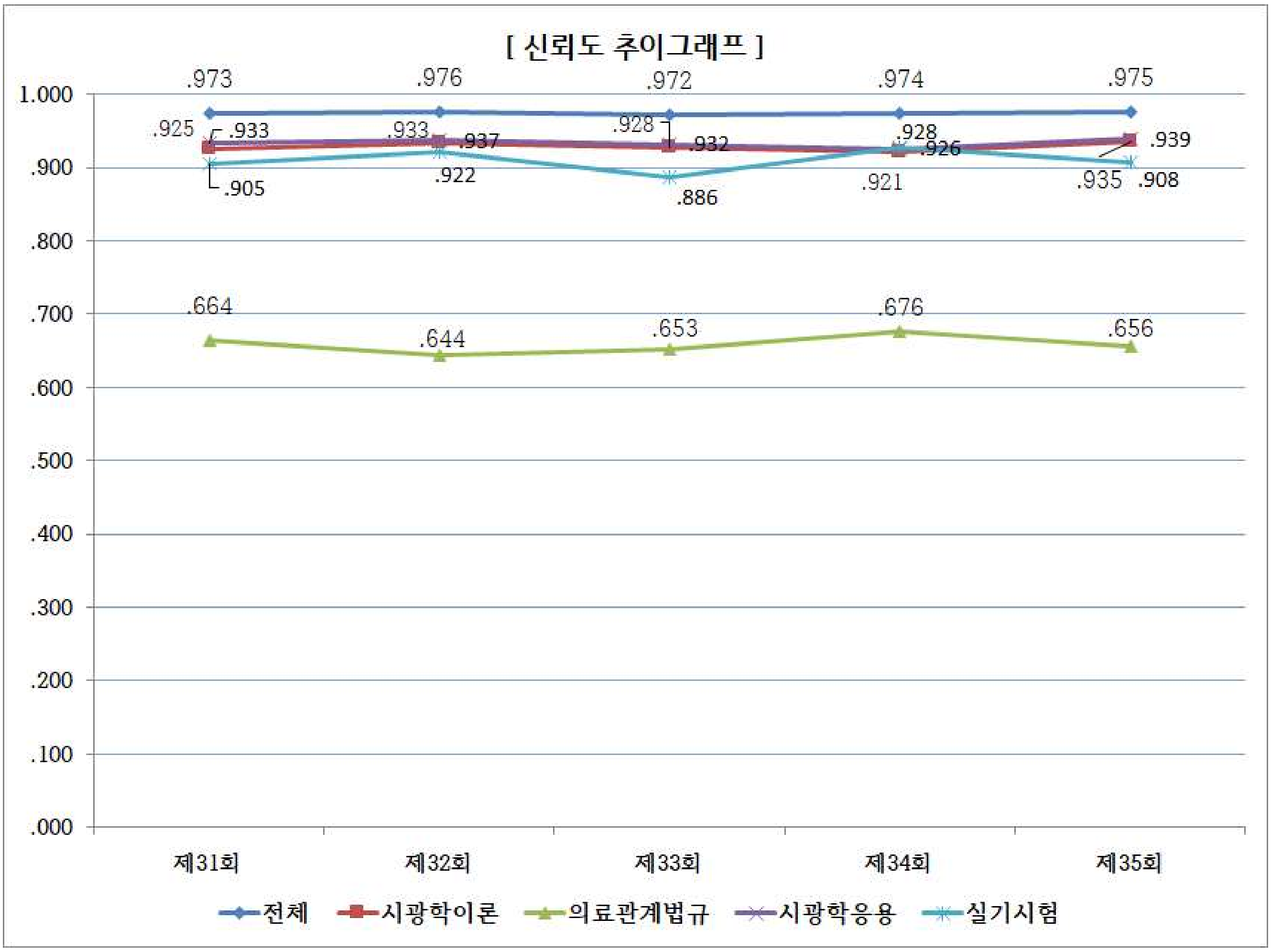

해석

- 전회 대비 전체 문항의 신뢰도는 .001 증가함
- 전회 대비 시광학이론 과목 문항의 신뢰도는 .014 증가함
- 전회 대비 의료관계법규 과목 문항의 신뢰도는 .020 감소함
- 전회 대비 시광학응용 과목 문항의 신뢰도는 .013 증가함
- 전회 대비 실기시험 과목 문항의 신뢰도는 .020 감소함

○ 분석결과 관련 문의 : 한국보건의료인국가시험원 연구개발본부 김보현 전임연구원
Tel : 02-2087-8954, FAX : 02-2087-8885
E-mail : kimbohyun@kuksiwon.or.kr
