# Supplementary material for: Item difficulty index, discrimination index, and reliability of the 26 health professions licensing examinations in 2022, Korea: a psychometric study
Source: J Educ Eval Health Prof. 2023 Nov 22;20:31. doi: 10.3352/jeehp.2023.20.31 (PMC11959405; doi:10.3352/jeehp.2023.20.31)
Supplement: Supplementary file 1 — Supplement 1. Item analysis results of 26 health professions licensing examinations administered during late 2022 and early 2023. [file jeehp-20-31_Suppl1.zip › 2022│Γ╡╡ ┴a39╚╕ ║╕░╟└╟╖ß┴ñ║╕░n╕«╗τ ▒╣░í╜├╟Φ ║╨╝«░ß░·.pdf]

2022년도 제39회
보건의료정보관리사 국가시험
문항분석 결과

국민이 신뢰하고 감동하는 시험평가기관
한국보건의료인국가시험원
KOREA HEALTH PERSONNEL LICENSING EXAMINATION INSTITUTE

일반 용어 정의

□ **평균**

- 집단에서의 대표적 경향값으로 전체 값을 더하여 총 응시자로 나눈 값

□ **표준편차**

- 평균과 각 점수의 차이인 편차들의 평균으로 점수가 흩어져 분포되어 있는 정도

□ **추정난이도**

- 문항개발자가 예측한 정답률

□ **검사이론**

- 검사와 검사를 구성하고 있는 문항의 양호도를 분석 및 평가하는 방법을 정의한 이론체계
- 대표적으로 고전검사이론과 문항반응이론이 있음

고전검사이론 용어 정의

□ **고전검사이론(Classical Test Theory; CTT)**

- 검사의 질을 분석하는 검사이론 중 한 가지로 19세기 말부터 전개되어 현재까지 주로 사용되고 있는 검사이론임
- 고전검사이론에 의한 문항과 응시자 능력 추정치는 다음과 같음

○ **문항난이도**

· 검사 문항의 쉽고 어려운 정도를 나타내는 지수
· 난이도 지수는 총 반응 수에 대한 정답 반응 수의 비율로 문항의 정답률임
· 문항난이도는 0~100까지의 값을 가짐
· 난이도 값이 큰 경우, 쉬운 문항으로 '난이도가 낮다'라고 해석하며, 난이도 값이 작은 경우, 어려운 문항으로 '난이도가 높다'라고 해석함

○ **문항변별도**

· 각 문항이 응시자의 능력 수준을 변별할 수 있는 정도를 나타내는 지수
· 문항변별도는 -1~+1까지의 값을 가지며, 1에 가까울수록 변별력 크다고 해석함
· 일반적으로 문항변별도가 0.3 이상이면 우수한 문항으로 평가함
· 구하는 방식에는 '상하위집단 구분법', '문항-총점 상관계수' 등이 있음
  1) **변별도 1(상하위구분법):** 상위 27%와 하위 27% 집단의 난이도 차이를 구하는 방식
  2) **변별도 2(상관계수법):** 문항-총점과의 상관계수로 구하는 방식

○ **신뢰도**

· 시험이 평가하고자 하는 것을 일관성 있게 측정하는가로 시험이 오차없이 정확하게 측정한 정도를 의미함
· 국시원에서는 문항의 내적일관성(Cronbach α)으로 신뢰도를 추정하며 1에 가까울수록 신뢰도가 높다고 해석함

목 차

I. 시행 결과 ······ 6
1. 시험 현황 ······ 7
1) 시험명 ······ 7
2) 시험시행일 ······ 7
3) 응시현황 ······ 7
4) 과목별 문항 수, 배점 및 과락 점수 ······ 7
2. 합격률과 평균성적 ······ 7
1) 합격 및 불합격 현황 ······ 7
2) 과목별 과락자수 내역 ······ 7
3) 전회 대비 합격률과 평균성적 ······ 8

II. 문항분석 결과 ······ 10
1. 성적 ······ 11
1) 전체 성적분포도 ······ 11
2) 과목별 성적분포도 ······ 12
2. 난이도와 변별도 ······ 13
1) 전체 난이도와 변별도 ······ 13
2) 과목별 난이도와 변별도 ······ 16
3) 지식수준별 난이도와 변별도 ······ 25
3. 난이도와 변별도 간 산포도 ······ 34
1) 전체 난이도와 변별도 간 산포도 ······ 34
2) 과목별 난이도와 변별도 간 산포도 ······ 35
4. 신뢰도 분석 ······ 37

I. 시행 결과

1. 시험 현황

1) 시험명: 2022년도 제39회 보건의료정보관리사 국가시험

2) 시험시행일: 2022년 12월 3일

3) 응시현황

응시대상자 수	결시자 수	부정행위자 수	응시자 준수사항 위반자 수		응시자 수 (%)
			휴대폰 소지	신분증 미지참	
2,902	157	0	0	0	2,745 (94.6)

4) 과목별 문항 수, 배점 및 과락 점수

교 시	과 목 명	문제 수	배점	총점	합격자 점수기준	
					과목 과락기준	총점 합격기준
1교시	보건의료정보관리학1	102	1	102	68점 미만	114점 이상
2교시	보건의료정보관리학2	68	1	68		
2교시	의료관계법규	20	1	20	8점 미만	
3교시	실기시험	40	1	40	24점 미만	
계		230		230		

※ 1교시 보건의료정보관리학1, 2교시 보건의료정보관리학2는 동일과목임

2. 합격률과 평균성적

1) 합격 및 불합격 현황

합격자 수 (%)	불합격자 수(%)					채점보류자 수
	평락	과락	실기탈락	기권	계	
1,516 (55.2)	1,149 (41.86)	1 (0.04)	68 (2.48)	11 (0.40)	1,229 (44.8)	0 (0.00)

2) 과목별 과락자수 내역

과목명 / 과락자 수	보건의료정보관리학	의료관계법규	실기시험
과목별 과락자 수	0	1	0
전과목 과락자 수	0		

3) 전회 대비 합격률과 평균성적

회차	년도	합격률(%)	평균성적	표준편차	백분율 환산점수
제35회	2018	31.1	129.1	36.7	56.1
제36회	2019	68.5	152.4	40.2	66.3
제37회	2020	45.5	128.1	40.6	55.7
제38회	2021	40.2	122.0	40.7	53.1
**제39회**	**2022**	**55.2**	**135.4**	**39.6**	**58.9**

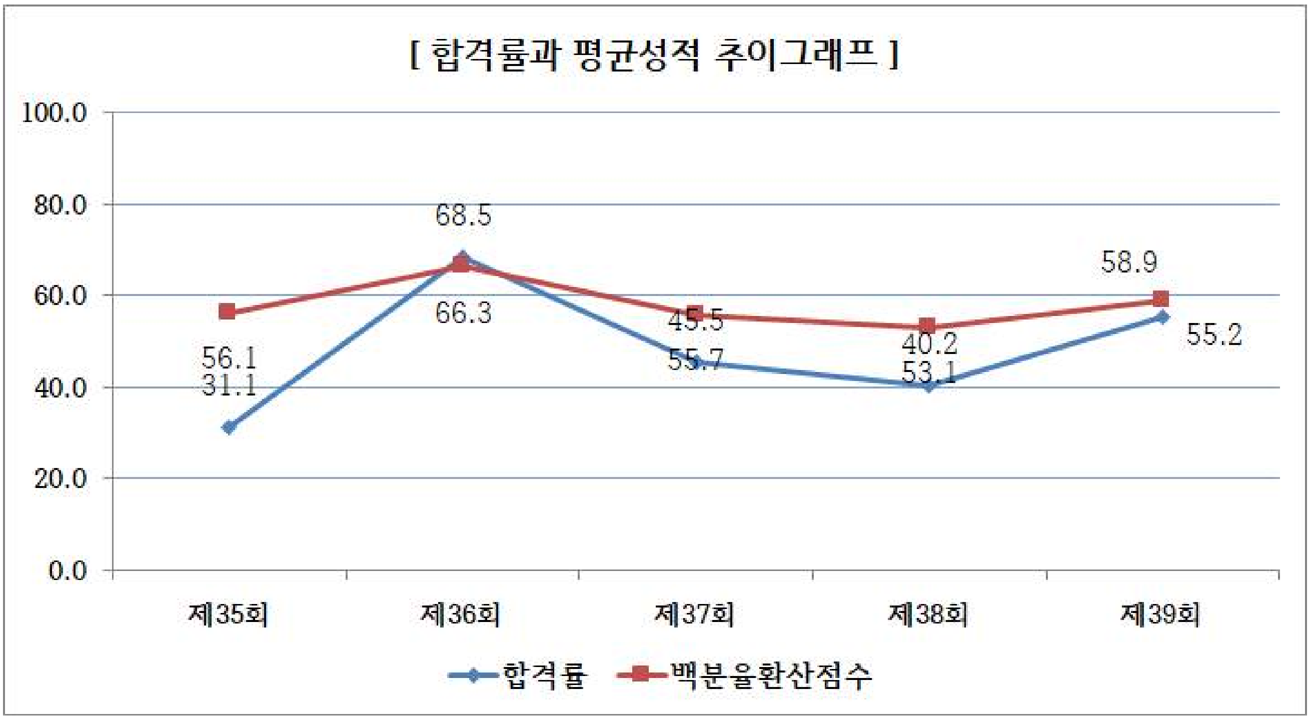

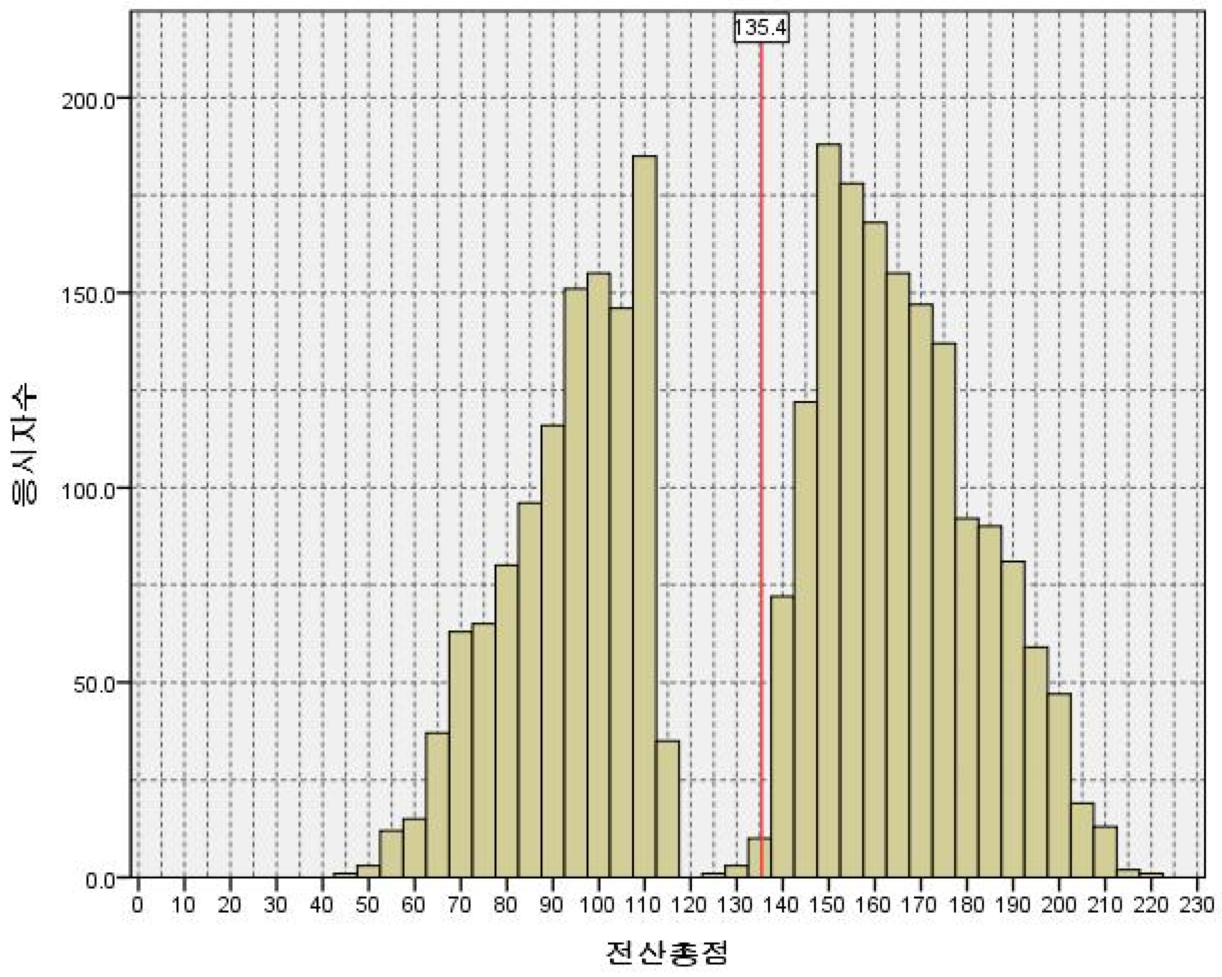

응시자	총점	합격선	평균성적	표준편차
2,745	230		135.4	39.6

※ 2,745명은 전체응시자(2,745명)에서 채점보류자(0명)를 제외한 수치임

※ 필기시험 불합격자의 실기성적을 포함하지 않음

해석

- 전년대비 합격률은 15.0%, 백분율 환산점수는 5.8 점 증가함

II. 문항분석 결과

1. 성적

1) 전체 성적분포도

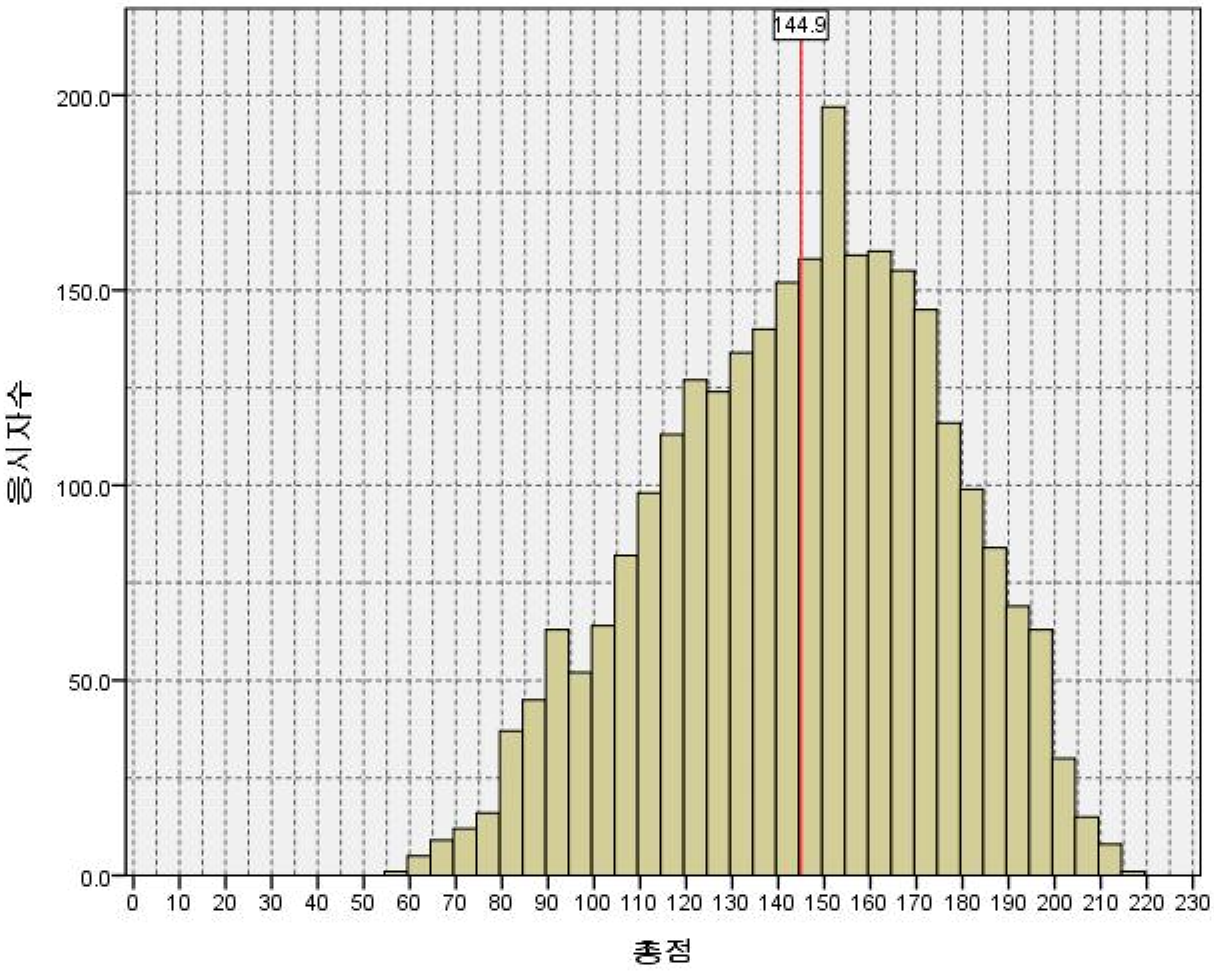

응시자	총점	합격선	평균성적	표준편차
2,734	230		144.9	30.7

※ 2,734명은 전체응시자(2,745명)에서 기권자(11명)를 제외한 수치임

※ 필기시험 불합격자의 실기성적을 포함함

2) 과목별 성적분포도(※ 필기형 실기 포함)

가) 보건의료정보관리학

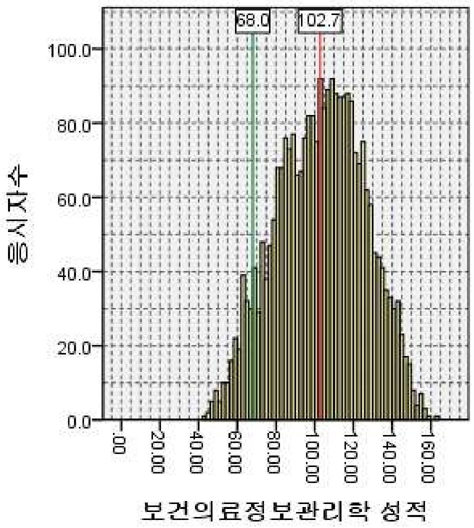

총점	과락선	평균성적	표준편차
170	68	102.7	22.8

나) 의료관계법규

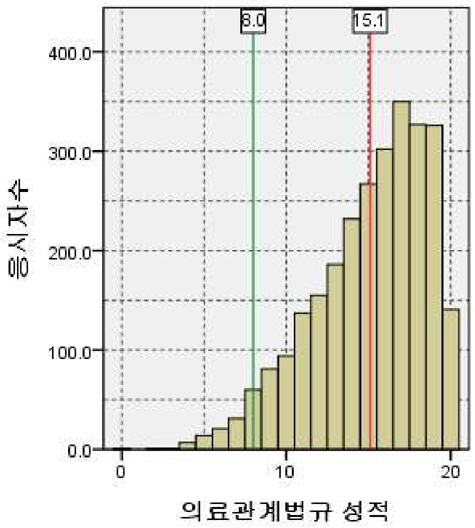

총점	과락선	평균성적	표준편차
20	8	15.1	3.5

다) 실기시험

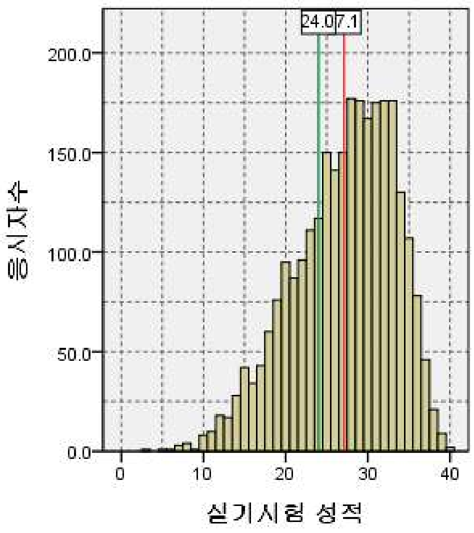

총점	과락선	평균성적	표준편차
40	24	27.1	6.2

2. 난이도와 변별도

1) 전체 난이도와 변별도

가) 전회 대비 전체 난이도와 변별도

회차	난이도		변별도1		변별도2	
	평균	표준편차	평균	표준편차	평균	표준편차
제35회	59.4	21.1	.32	.15	.28	.12
제36회	68.8	19.5	.35	.17	.33	.13
제37회	60.2	20.4	.35	.17	.31	.13
제38회	58.1	19.1	.36	.17	.30	.13
**제39회**	**63.0**	**21.8**	**.33**	**.17**	**.29**	**.13**

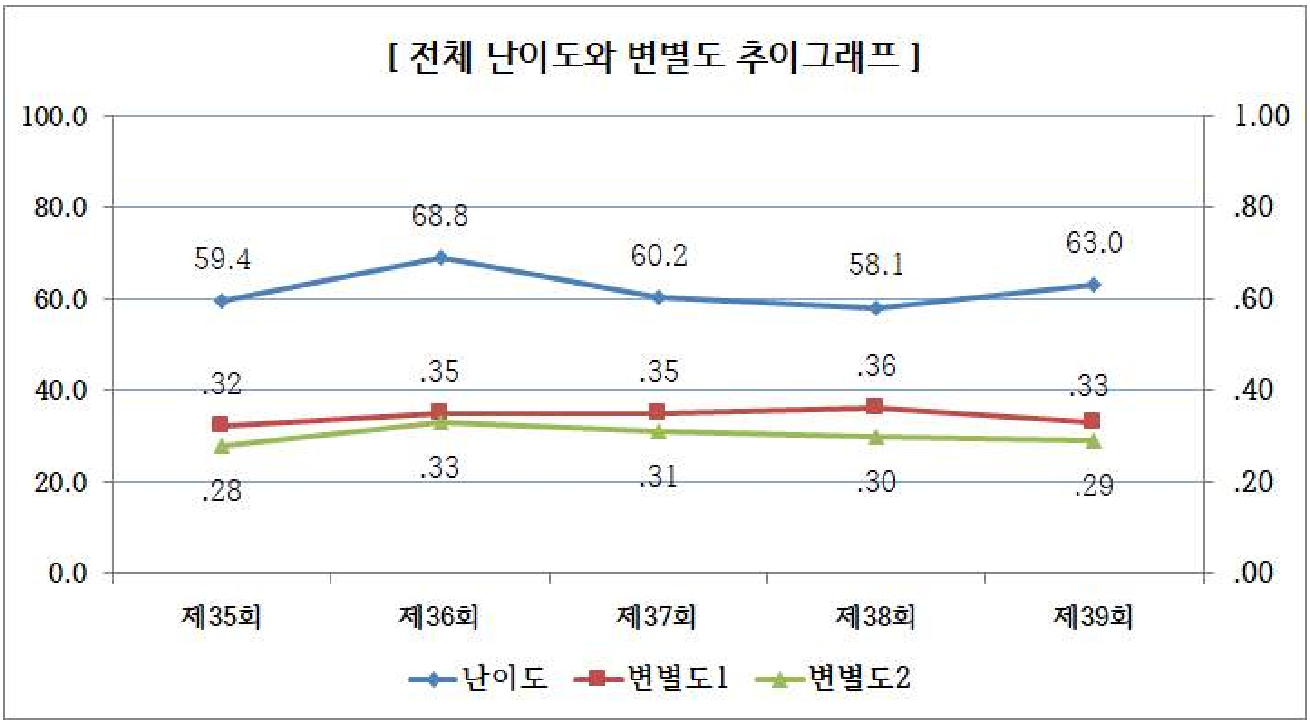

해석

- 전회 대비 난이도 지수는 4.9 증가함
- 변별도 1 지수는 .03 감소함
- 변별도 2 지수는 .01 감소함

나) 전체 난이도와 변별도 분포도 및 비율분석

(1) 전체 난이도 분포도 및 비율분석

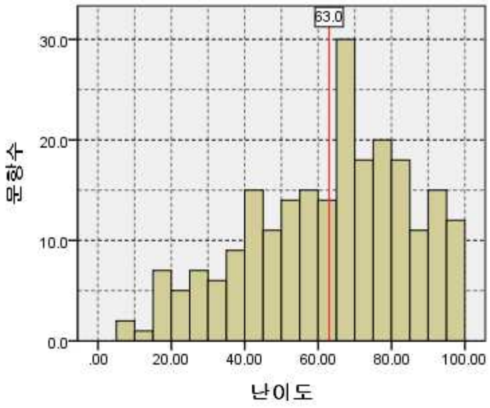

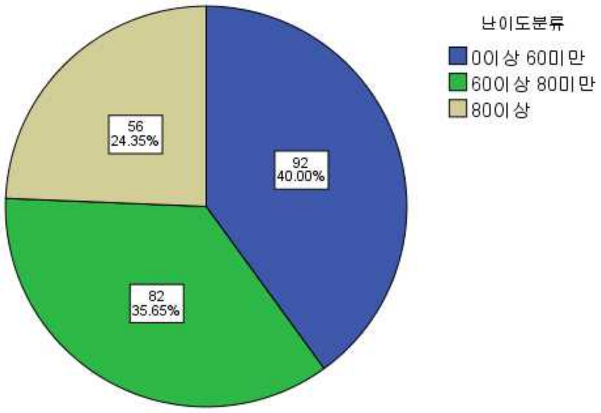

총점	난이도	표준편차
230	63.0	21.8

난이도	문항수	비율(%)
0~60미만	92	40.0
60~80미만	82	35.7
80~100	56	24.3
전체	230	100.0

(2) 전체 변별도1 분포도 및 비율분석

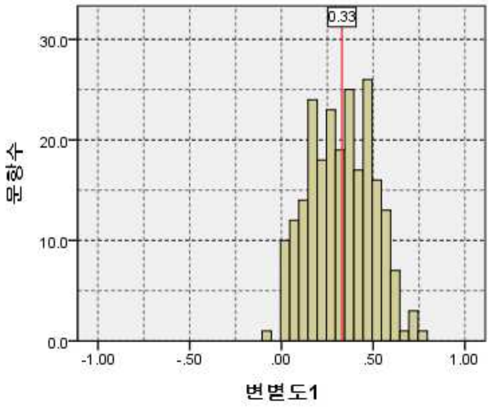

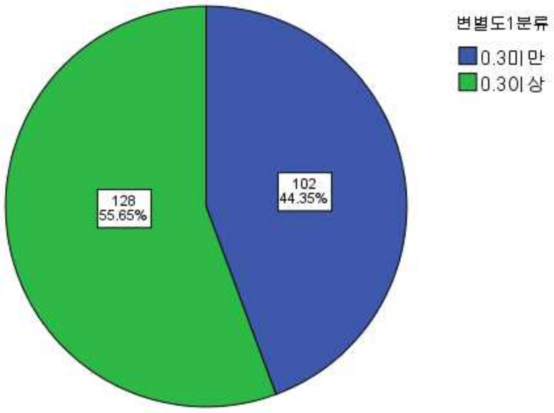

총점	변별도1	표준편차
230	.33	.17

변별도1	문항수	비율(%)
0.3미만	102	44.3
0.3이상	128	55.7
전체	230	100.0

(3) 전체 변별도2 분포도 및 비율분석

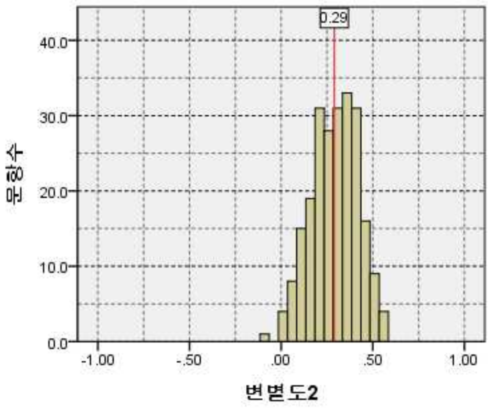

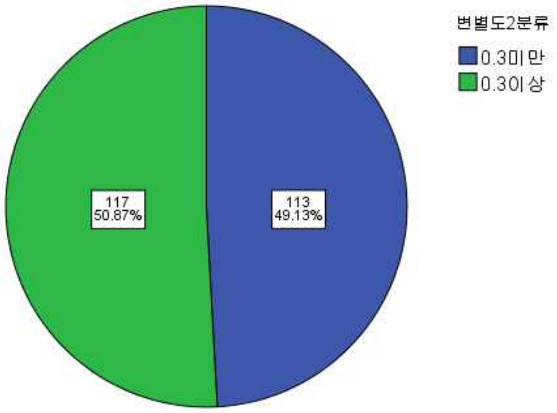

총점	변별도2	표준편차
230	.29	.13

변별도2	문항수	비율(%)
0.3미만	113	49.1
0.3이상	117	50.9
전체	230	100.0

해석

- 난이도 지수가 60 미만인 문항이 92 문항으로 가장 많았으며, 60 이상 80 미만인 문항이 82 문항, 80 이상인 문항이 56 문항인 것으로 나타남
- 변별도 1 지수를 기준으로 분류하였을 때, 0.3 미만인 문항이 102 문항으로 0.3 이상인 문항이 128 문항인 것에 비해 더 적게 나타남
- 변별도 2 지수를 기준으로 분류하였을 때, 0.3 미만인 문항이 113 문항으로 0.3 이상인 문항이 117 문항인 것에 비해 더 적게 나타남

2) 과목별 난이도와 변별도

가) 전회 대비 과목별 난이도와 변별도

(1) 전회 대비 보건의료정보관리학 난이도와 변별도

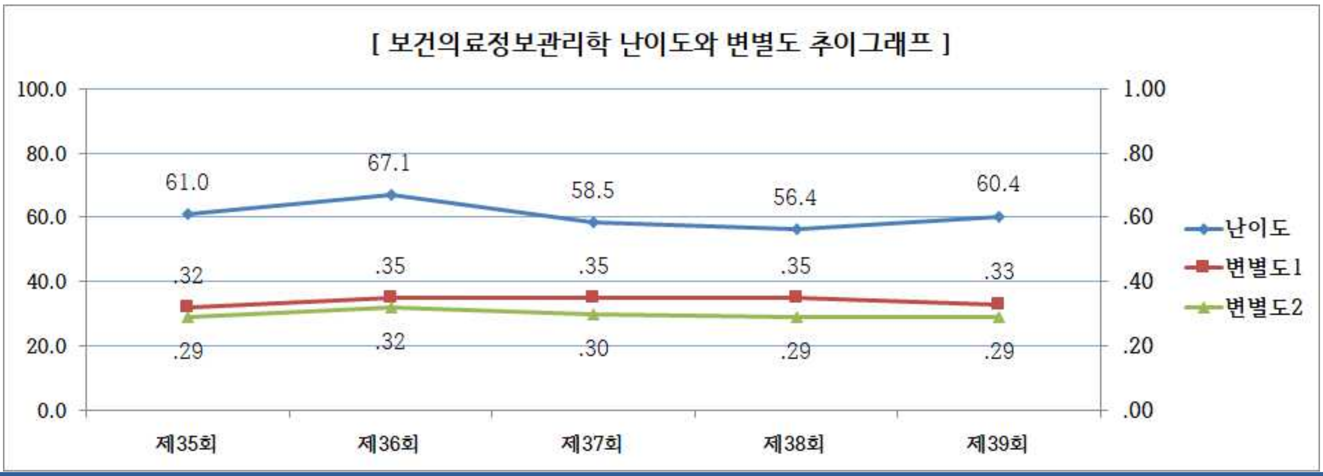

회차	난이도		변별도1		변별도2	
	평균	표준편차	평균	표준편차	평균	표준편차
제35회	61.0	21.8	.32	.15	.29	.11
제36회	67.1	20.3	.35	.18	.32	.13
제37회	58.5	20.6	.35	.18	.30	.13
제38회	56.4	19.1	.35	.18	.29	.14
**제39회**	**60.4**	**22.7**	**.33**	**.18**	**.29**	**.13**

해석

- 전회 대비 보건의료정보관리학 과목의 난이도 지수는 4.0 증가함
- 전회 대비 보건의료정보관리학 과목의 변별도 1 지수는 .02 감소함
- 전회 대비 보건의료정보관리학 과목의 변별도 2 지수는 동일함

(2) 전회 대비 의료관계법규 난이도와 변별도

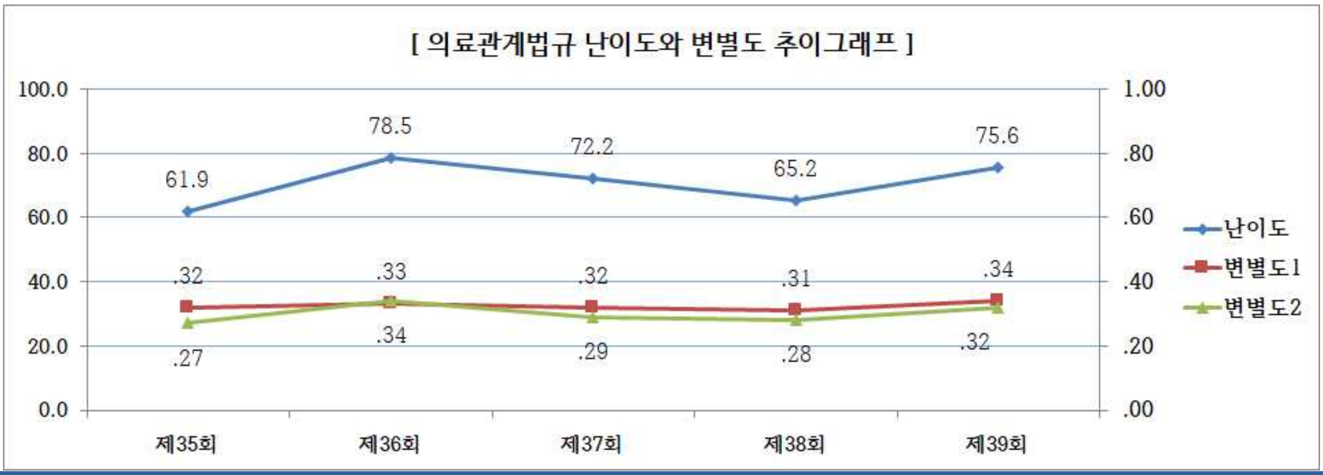

회차	난이도		변별도1		변별도2	
	평균	표준편차	평균	표준편차	평균	표준편차
제35회	61.9	18.8	.32	.16	.27	.12
제36회	78.5	15.4	.33	.18	.34	.11
제37회	72.2	14.0	.32	.13	.29	.10
제38회	65.2	22.9	.31	.16	.28	.11
**제39회**	**75.6**	**13.7**	**.34**	**.15**	**.32**	**.11**

해석

- 전회 대비 의료관계법규 과목의 난이도 지수는 10.4 증가함
- 전회 대비 의료관계법규 과목의 변별도 1 지수는 .03 증가함
- 전회 대비 의료관계법규 과목의 변별도 2 지수는 .04 증가함

(3) 전회 대비 실기시험 난이도와 변별도

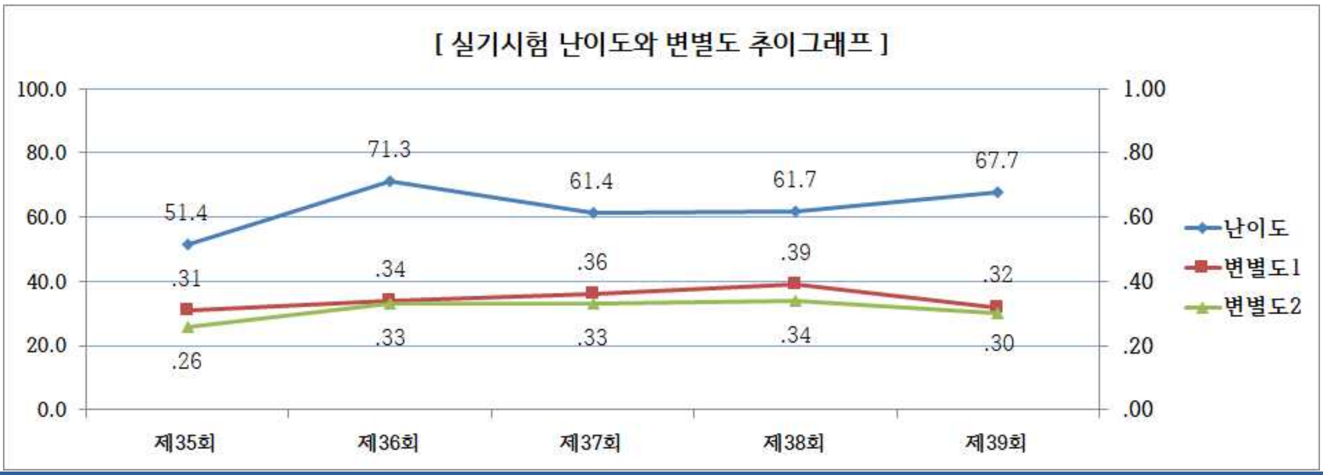

회차	난이도		변별도1		변별도2	
	평균	표준편차	평균	표준편차	평균	표준편차
제35회	51.4	17.3	.31	.15	.26	.13
제36회	71.3	16.3	.34	.16	.33	.14
제37회	61.4	20.6	.36	.15	.33	.12
제38회	61.7	15.8	.39	.12	.34	.09
**제39회**	**67.7**	**18.4**	**.32**	**.15**	**.30**	**.12**

해석

- 전회 대비 실기시험 과목의 난이도 지수는 6.0 증가함
- 전회 대비 실기시험 과목의 변별도 1 지수는 .07 감소함
- 전회 대비 실기시험 과목의 변별도 2 지수는 .04 감소함

나) 과목별 난이도와 변별도 분포도 및 비율분석

(1) 보건의료정보관리학 난이도와 변별도 분석도 및 비율분석

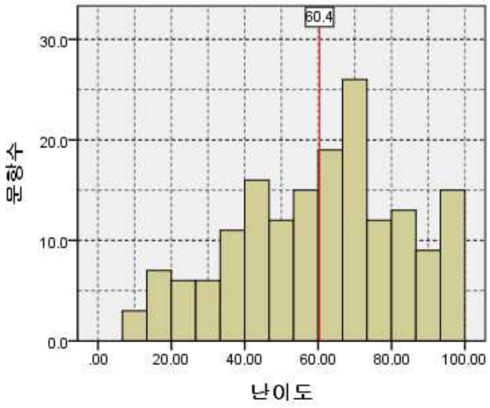

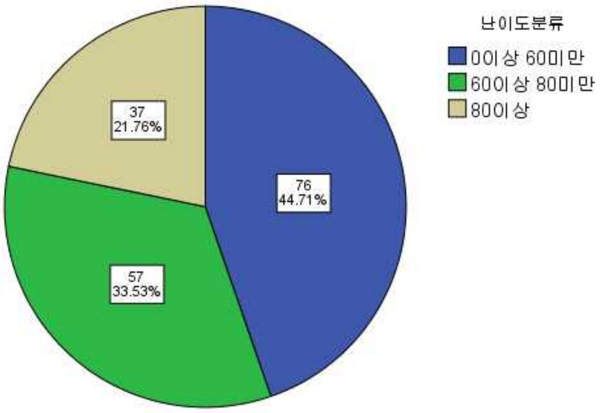

총점	난이도	표준편차
170	60.4	22.7

난이도	문항수	비율(%)
0~60미만	76	44.7
60~80미만	57	33.5
80~100	37	21.8
전체	170	100.0

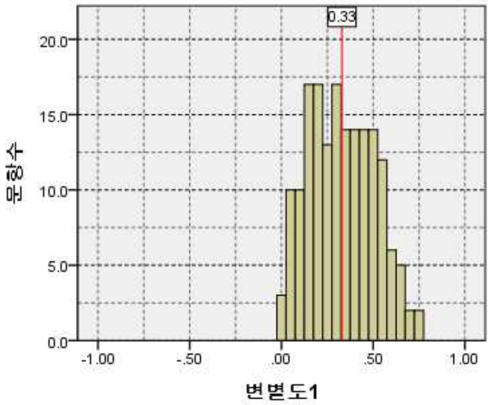

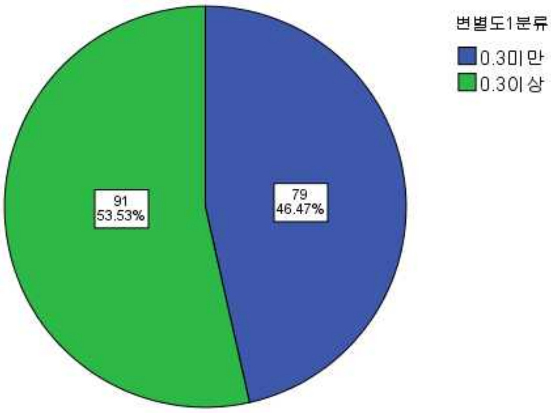

총점	변별도1	표준편차
170	.33	.18

변별도1	문항수	비율(%)
0.3미만	79	46.5
0.3이상	91	53.5
전체	170	100.0

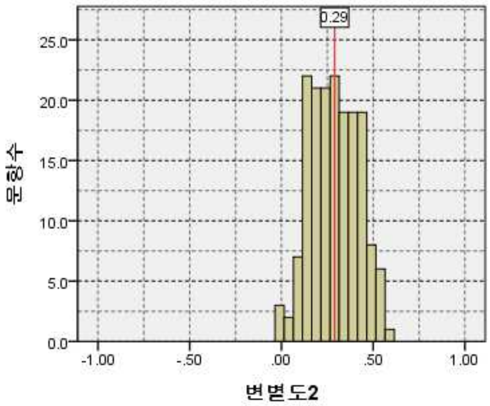

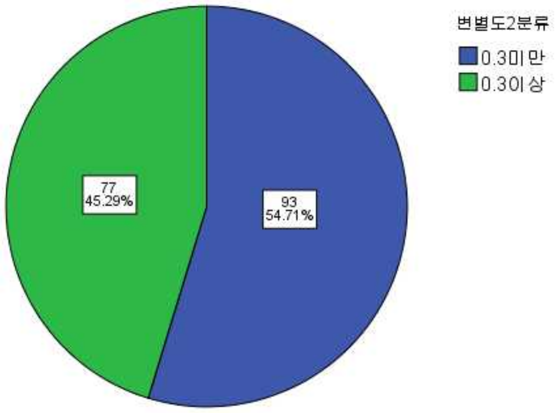

총점	변별도2	표준편차
170	.29	.13

변별도2	문항수	비율(%)
0.3미만	93	54.7
0.3이상	77	45.3
전체	170	100.0

해석

- 보건의료정보관리학 과목에서 난이도 지수가 60 미만인 문항이 76 문항으로 가장 많았으며, 60 이상 80 미만인 문항이 57 문항, 80 이상인 문항이 37 문항으로 나타남
- 변별도 1 지수를 기준으로 분류하였을 때, 0.3 미만인 문항이 79 문항으로 0.3 이상인 문항이 91 문항인 것에 비해 더 적게 나타남
- 변별도 2 지수를 기준으로 분류하였을 때, 0.3 미만인 문항이 93 문항으로 0.3 이상인 문항이 77 문항인 것에 비해 더 많이 나타남

(2) 의료관계법규 난이도와 변별도 분포도 및 비율분석

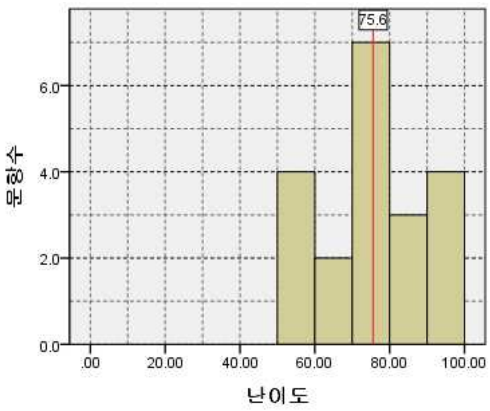

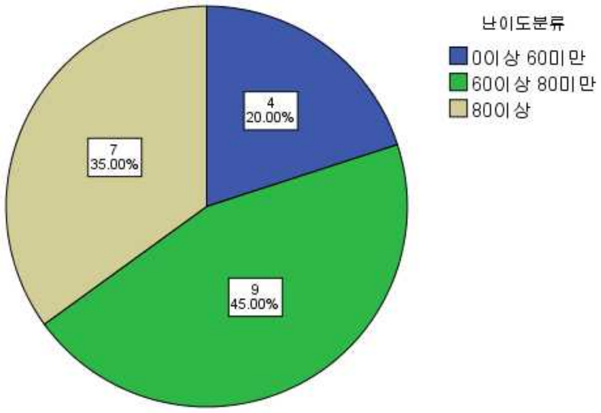

총점	난이도	표준편차
20	75.6	13.7

난이도	문항수	비율(%)
0~60미만	4	20.0
60~80미만	9	45.0
80~100	7	35.0
전체	20	100.0

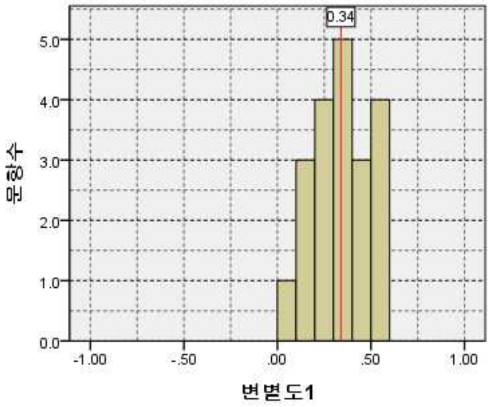

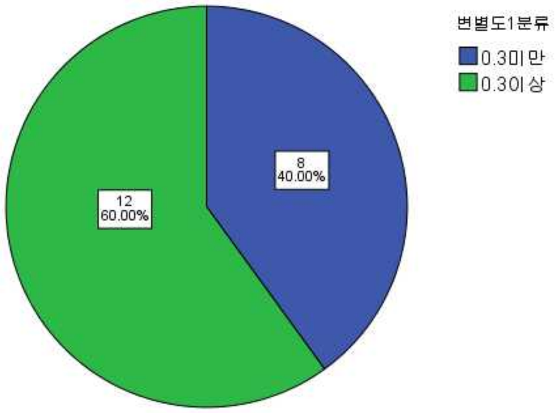

총점	변별도1	표준편차
20	.34	.15

변별도1	문항수	비율(%)
0.3미만	8	40.0
0.3이상	12	60.0
전체	20	100.0

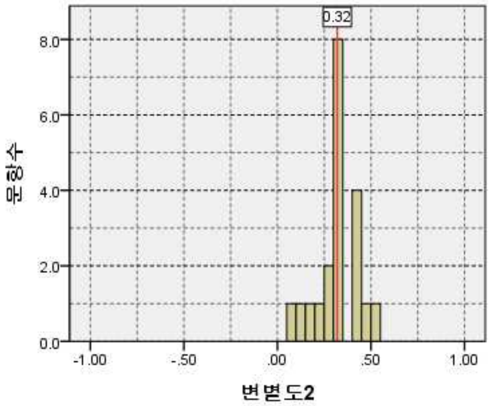

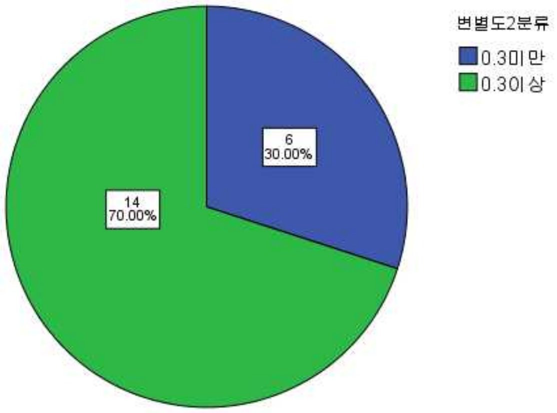

총점	변별도2	표준편차
20	.32	.11

변별도2	문항수	비율(%)
0.3미만	6	30.0
0.3이상	14	70.0
전체	20	100.0

해석

- 의료관계법규 과목에서 난이도 지수가 60 이상 80 미만인 문항이 9 문항으로 가장 많았으며, 80 이상인 문항이 7 문항, 60 미만인 문항이 4 문항으로 나타남
- 변별도 1 지수를 기준으로 분류하였을 때, 0.3 미만인 문항이 8 문항으로 0.3 이상인 문항이 12 문항인 것에 비해 더 적게 나타남
- 변별도 2 지수를 기준으로 분류하였을 때, 0.3 미만인 문항이 6 문항으로 0.3 이상인 문항이 14 문항인 것에 비해 더 적게 나타남

(3) 실기시험 난이도와 변별도 분포도 및 비율분석

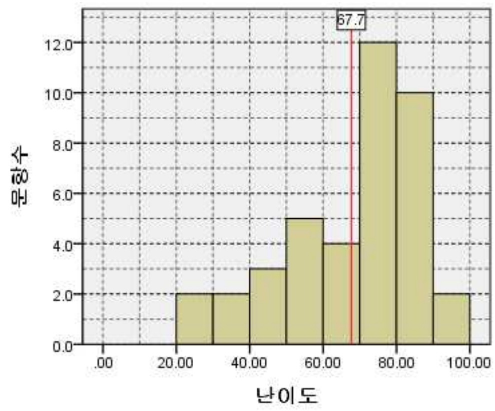

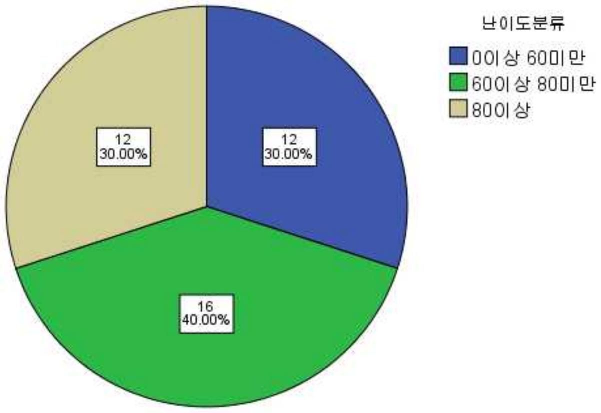

총점	난이도	표준편차
40	67.7	18.4

난이도	문항수	비율(%)
0~60미만	12	30.0
60~80미만	16	40.0
80~100	12	30.0
전체	40	100.0

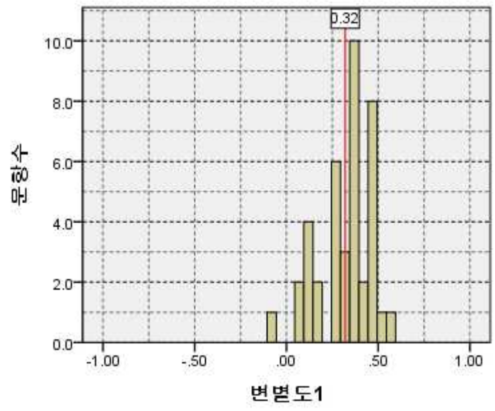

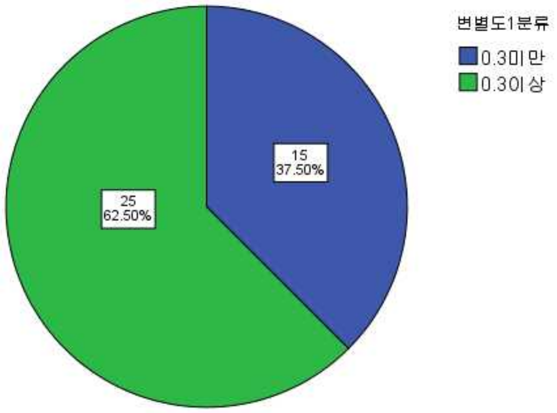

총점	변별도1	표준편차
40	.32	.15

변별도1	문항수	비율(%)
0.3미만	15	37.5
0.3이상	25	62.5
전체	40	100.0

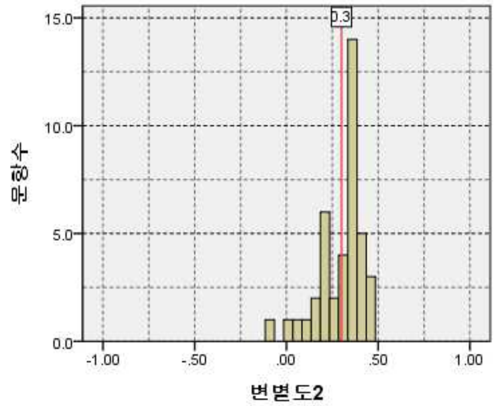

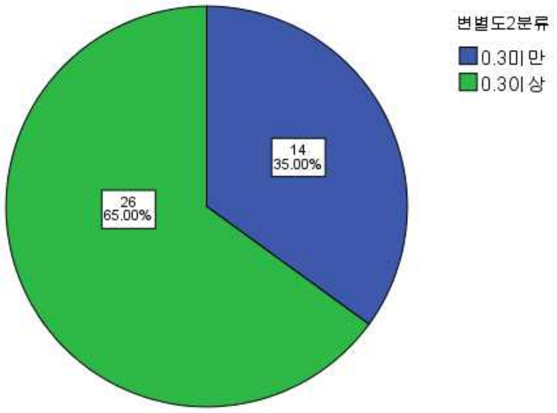

총점	변별도2	표준편차
40	.30	.12

변별도2	문항수	비율(%)
0.3미만	14	35.0
0.3이상	26	65.0
전체	40	100.0

해석

- 실기시험 과목에서 난이도 지수가 60 이상 80 미만인 문항이 16 문항으로 가장 많았으며, 60 미만인 문항과 80 이상인 문항이 각각 12 문항씩 나타남
- 변별도 1 지수를 기준으로 분류하였을 때, 0.3 미만인 문항이 15 문항으로 0.3 이상인 문항이 25 문항인 것에 비해 더 적게 나타남
- 변별도 2 지수를 기준으로 분류하였을 때, 0.3 미만인 문항이 14 문항으로 0.3 이상인 문항이 26 문항인 것에 비해 더 적게 나타남

3) 지식수준별 난이도와 변별도

가) 전회 대비 지식수준별 난이도와 변별도

(1) 전회 대비 암기형 난이도와 변별도

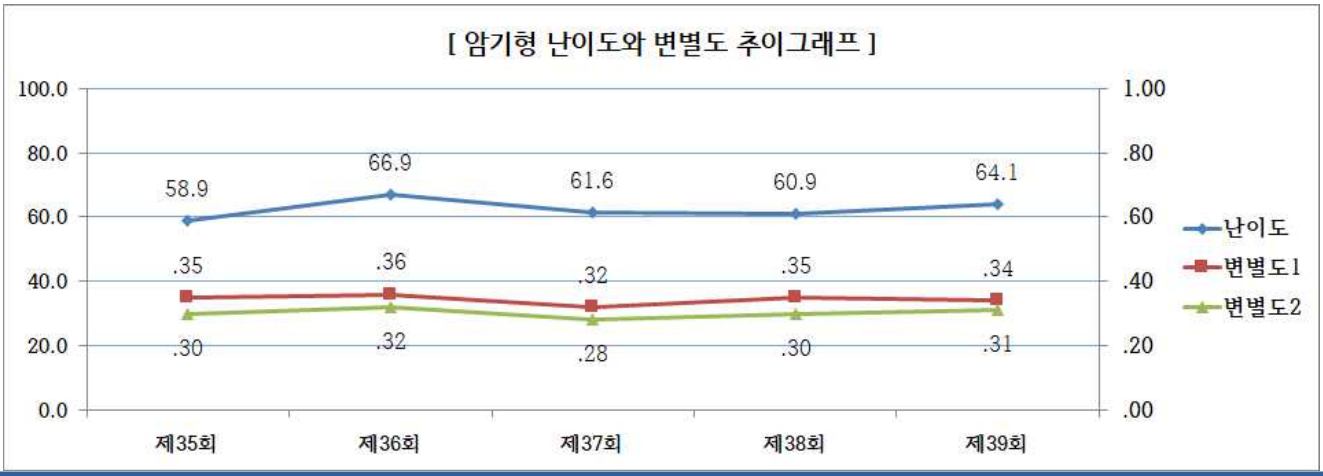

회차	난이도		변별도1		변별도2	
	평균	표준편차	평균	표준편차	평균	표준편차
제35회	58.9	20.7	.35	.15	.30	.12
제36회	66.9	20.4	.36	.18	.32	.13
제37회	61.6	20.9	.32	.16	.28	.12
제38회	60.9	20.1	.35	.18	.30	.13
**제39회**	**64.1**	**22.7**	**.34**	**.17**	**.31**	**.12**

해석

- 전회 대비 암기형 문항의 난이도 지수는 3.2 증가함
- 전회 대비 암기형 문항의 변별도 1 지수는 .01 감소함
- 전회 대비 암기형 문항의 변별도 2 지수는 .01 증가함

(2) 전회 대비 해석형 난이도와 변별도

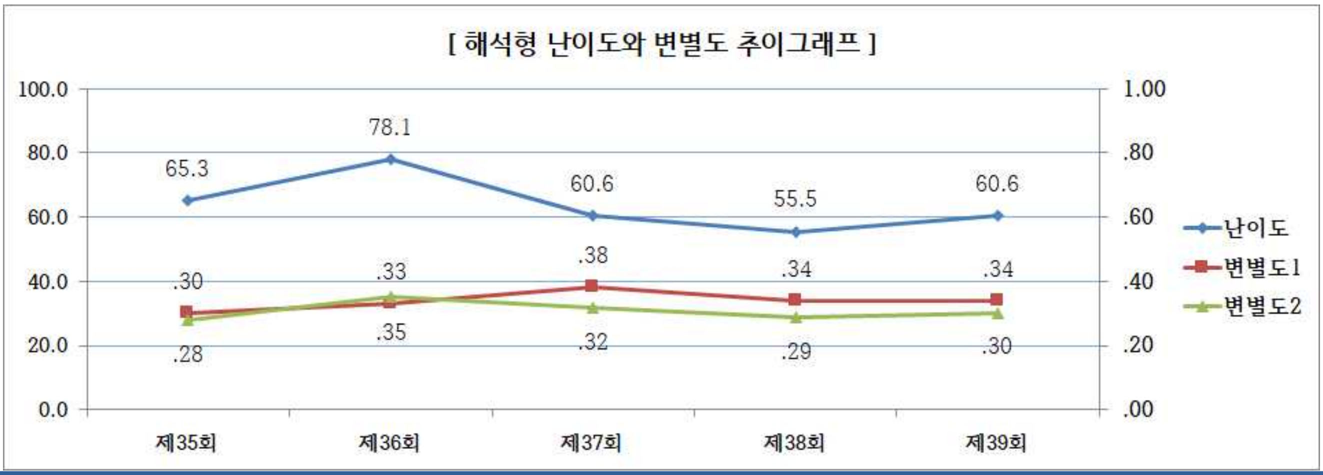

회차	난이도		변별도1		변별도2	
	평균	표준편차	평균	표준편차	평균	표준편차
제35회	65.3	22.0	.30	.15	.28	.11
제36회	78.1	14.7	.33	.18	.35	.12
제37회	60.6	19.5	.38	.19	.32	.14
제38회	55.5	21.0	.34	.18	.29	.14
**제39회**	**60.6**	**22.0**	**.34**	**.18**	**.30**	**.14**

해석

- 전회 대비 해석형 문항의 난이도 지수는 5.1 증가함
- 전회 대비 해석형 문항의 변별도 1 지수는 동일함
- 전회 대비 해석형 문항의 변별도 2 지수는 .01 증가함

(3) 전회 대비 해결형 난이도와 변별도

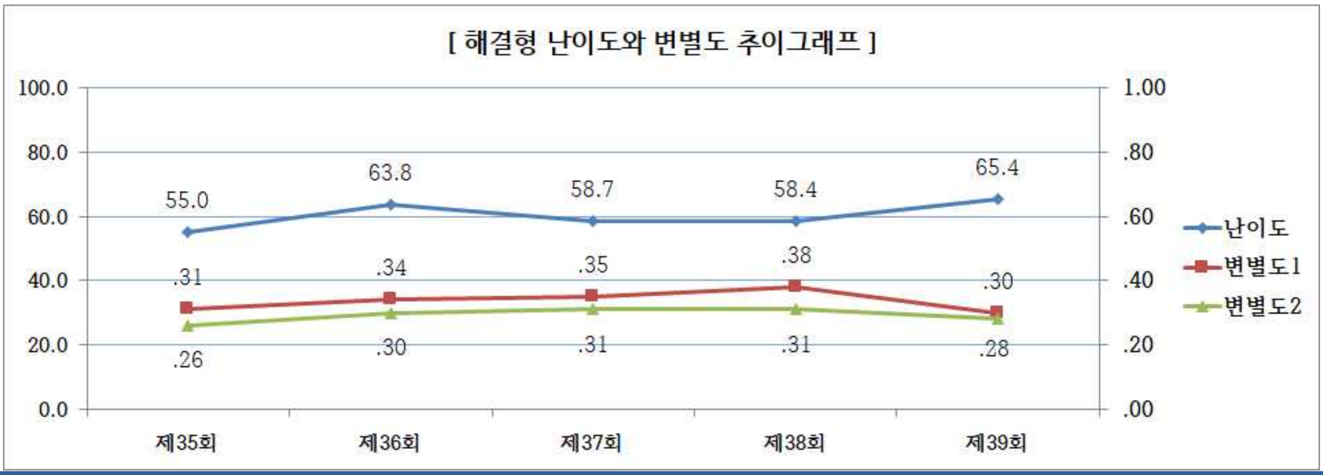

회차	난이도		변별도1		변별도2	
	평균	표준편차	평균	표준편차	평균	표준편차
제35회	55.0	19.7	.31	.14	.26	.12
제36회	63.8	19.3	.34	.16	.30	.14
제37회	58.7	21.0	.35	.16	.31	.12
제38회	58.4	13.9	.38	.14	.31	.11
**제39회**	**65.4**	**20.8**	**.30**	**.16**	**.28**	**.12**

해석

- 전회 대비 해결형 문항의 난이도 지수는 7.0 증가함
- 전회 대비 해결형 문항의 변별도 1 지수는 .08 감소함
- 전회 대비 해결형 문항의 변별도 2 지수는 .03 감소함

나) 지식수준별 난이도와 변별도 분포도 및 비율분석

(1) 암기형 난이도와 변별도 분포도 및 비율분석

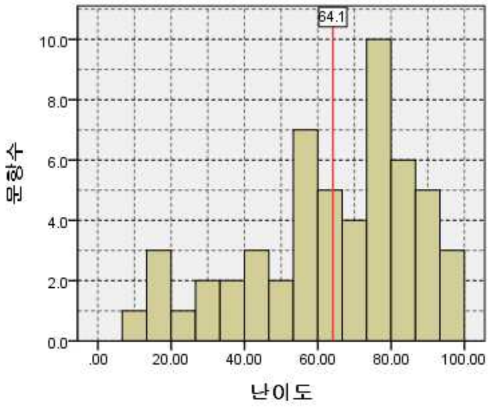

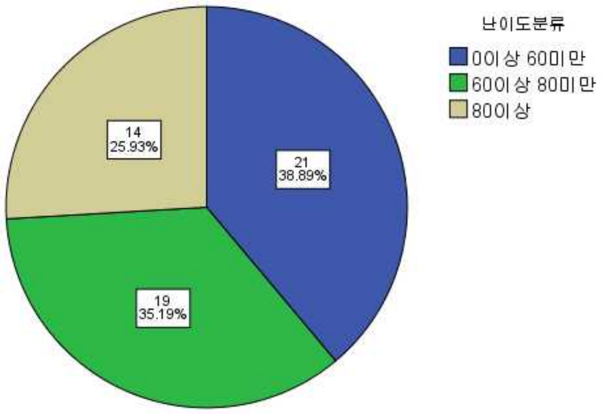

총점	난이도	표준편차
54	64.1	22.7

난이도	문항수	비율(%)
0~60미만	21	38.9
60~80미만	19	35.2
80~100	14	25.9
전체	54	100.0

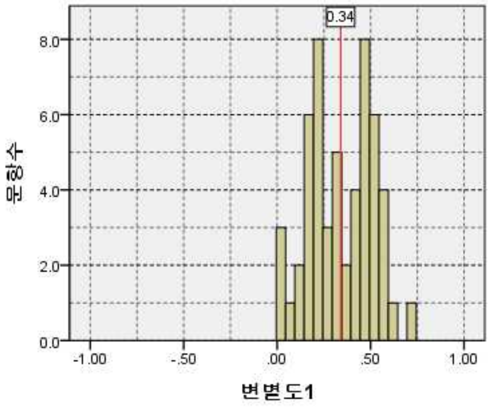

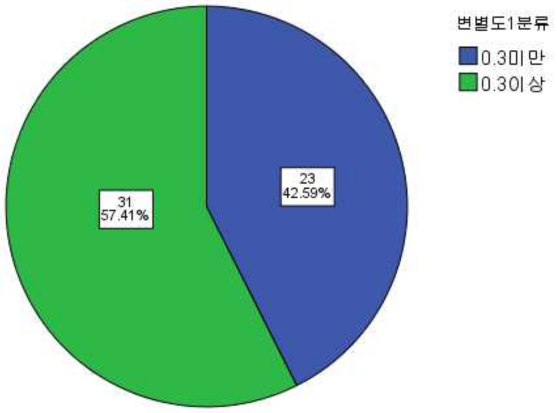

총점	변별도1	표준편차
54	.34	.17

변별도1	문항수	비율(%)
0.3미만	23	42.6
0.3이상	31	57.4
전체	54	100.0

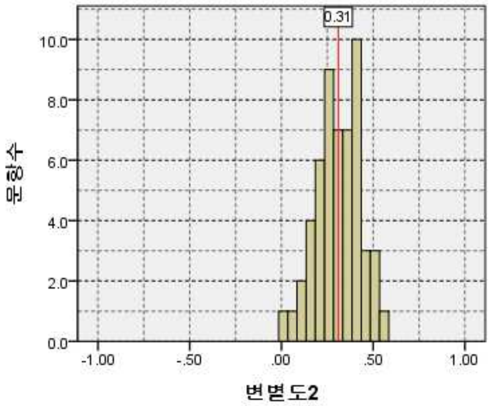

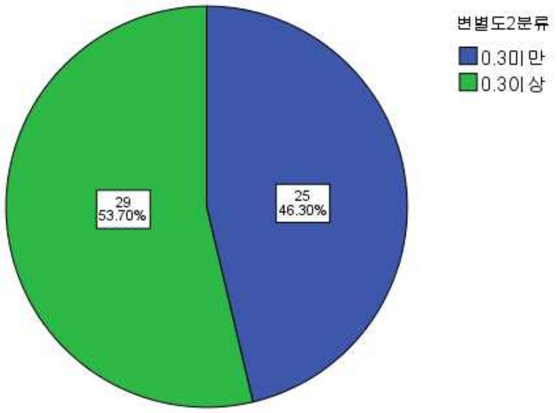

총점	변별도2	표준편차
54	.31	.12

변별도2	문항수	비율(%)
0.3미만	25	46.3
0.3이상	29	53.7
전체	54	100.0

해석

- 암기형 문항에서 난이도 지수가 60 미만인 문항이 21 문항으로 가장 많았으며, 60 이상 80 미만인 문항이 19 문항, 80 이상인 문항이 14 문항으로 나타남
- 변별도 1 지수를 기준으로 분류하였을 때, 0.3 미만인 문항이 23 문항으로 0.3 이상인 문항이 31 문항인 것에 비해 더 적게 나타남
- 변별도 2 지수를 기준으로 분류하였을 때, 0.3 미만인 문항이 25 문항으로 0.3 이상인 문항이 29 문항인 것에 비해 더 적게 나타남

(2) 해석형 난이도와 변별도 분포도 및 비율분석

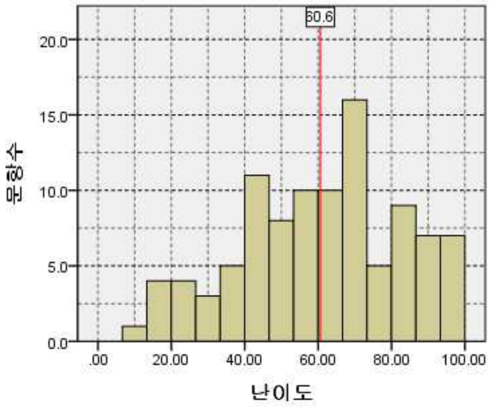

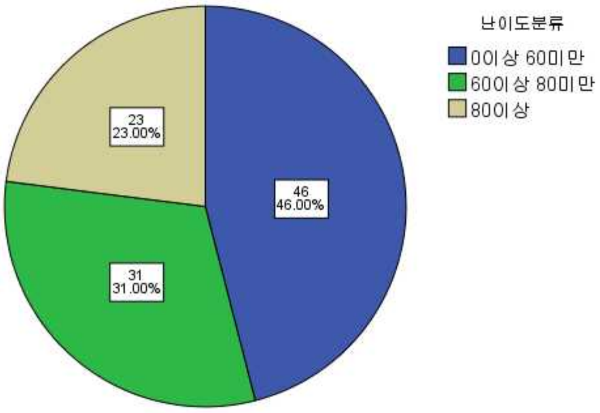

총점	난이도	표준편차
100	60.6	22.0

난이도	문항수	비율(%)
0~60미만	46	46.0
60~80미만	31	31.0
80~100	23	23.0
전체	100	100.0

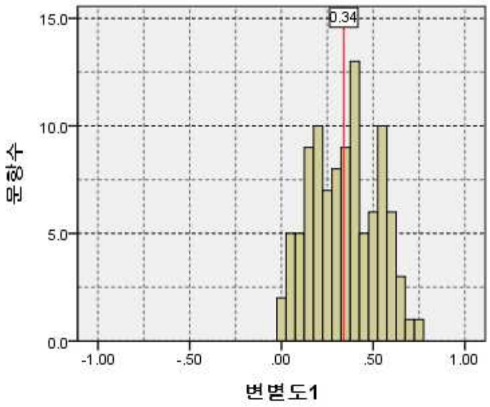

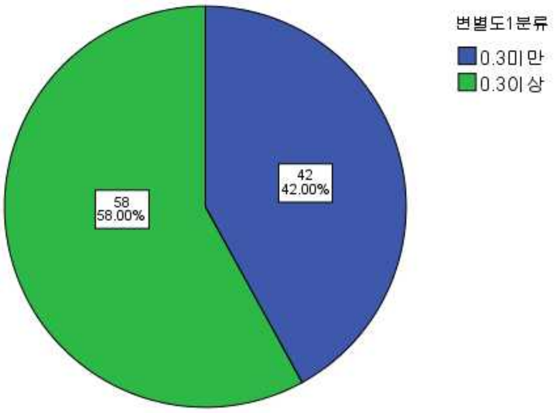

총점	변별도1	표준편차
100	.34	.18

변별도1	문항수	비율(%)
0.3미만	42	42.0
0.3이상	58	58.0
전체	100	100.0

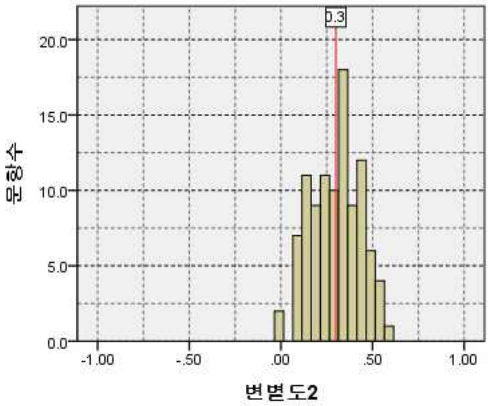

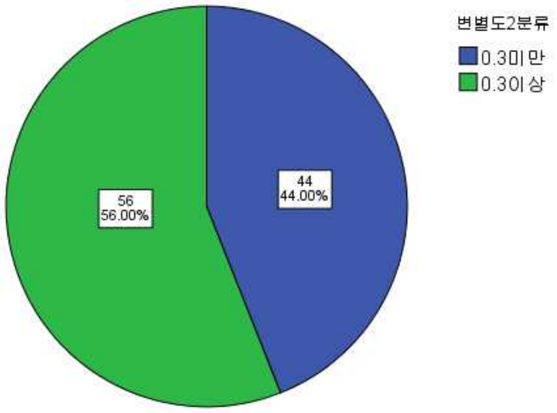

총점	변별도2	표준편차
100	.30	.14

변별도2	문항수	비율(%)
0.3미만	44	44.0
0.3이상	56	56.0
전체	100	100.0

해석

- 해석형 문항에서 난이도 지수가 60 미만인 문항이 46 문항으로 가장 많았으며, 60 이상 80 미만인 문항이 31 문항, 80 이상인 문항이 23 문항으로 나타남
- 변별도 1 지수를 기준으로 분류하였을 때, 0.3 미만인 문항이 42 문항으로 0.3 이상인 문항이 58 문항인 것에 비해 더 적게 나타남
- 변별도 2 지수를 기준으로 분류하였을 때, 0.3 미만인 문항이 44 문항으로 0.3 이상인 문항이 56 문항인 것에 비해 더 적게 나타남

(3) 해결형 난이도와 변별도 분포도 및 비율분석

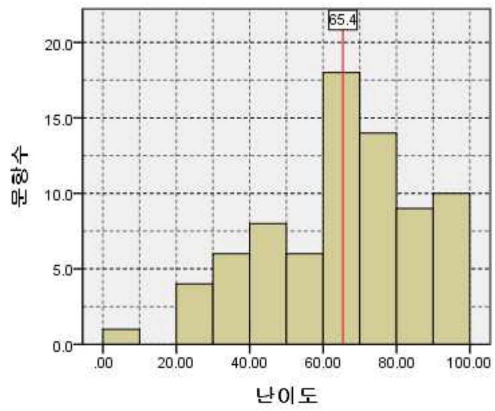

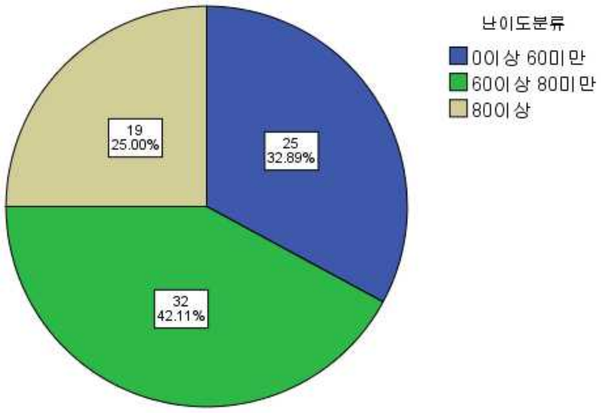

총점	난이도	표준편차
76	65.4	20.8

난이도	문항수	비율(%)
0~60미만	25	32.9
60~80미만	32	42.1
80~100	19	25.0
전체	76	100.0

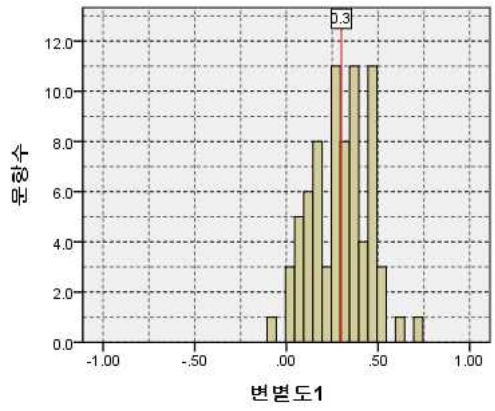

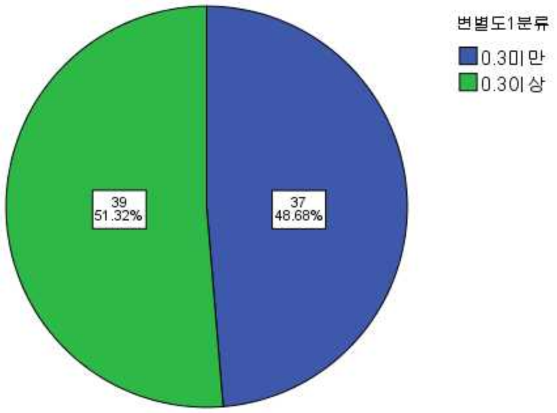

총점	변별도1	표준편차
76	.30	.16

변별도1	문항수	비율(%)
0.3미만	37	48.7
0.3이상	39	51.3
전체	76	100.0

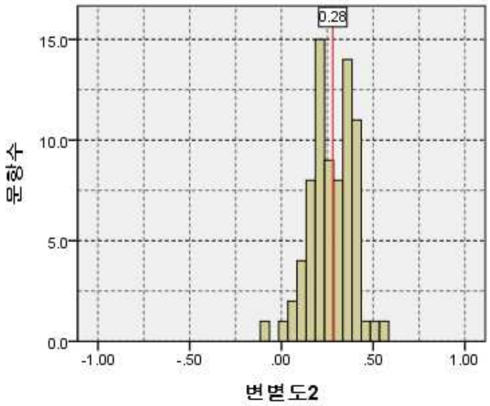

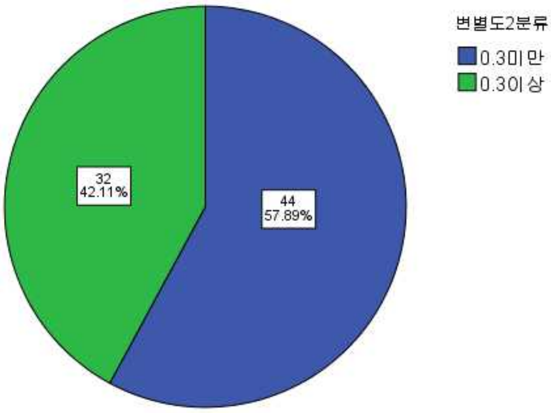

총점	변별도2	표준편차
76	.28	.12

변별도2	문항수	비율(%)
0.3미만	44	57.9
0.3이상	32	42.1
전체	76	100.0

해석

- 해결형 문항에서 난이도 지수가 60 이상 80 미만인 문항이 32 문항으로 가장 많았으며, 60 미만인 문항이 25 문항, 80 이상인 문항이 19 문항으로 나타남
- 변별도 1 지수를 기준으로 분류하였을 때, 0.3 미만인 문항이 37 문항으로 0.3 이상인 문항이 39 문항인 것에 비해 더 적게 나타남
- 변별도 2 지수를 기준으로 분류하였을 때, 0.3 미만인 문항이 44 문항으로 0.3 이상인 문항이 32 문항인 것에 비해 더 많이 나타남

3. 난이도와 변별도 간 산포도

1) 전체 난이도와 변별도 간 산포도

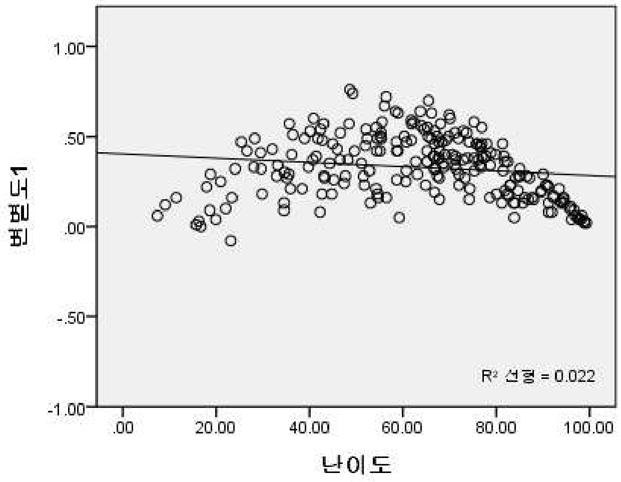

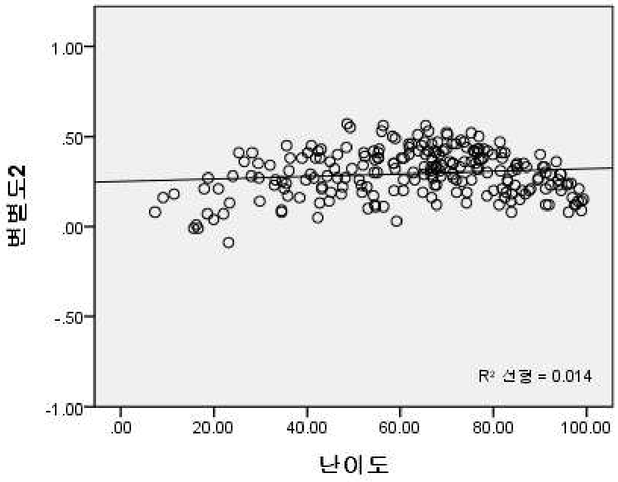

해석

- 전체 문항을 대상으로 한 난이도 지수와 변별도 1 지수 간 상관은 -.150*로 난이도 지수가 높을수록 변별력이 낮아지는 것으로 나타남
- 난이도 지수와 변별도 2 지수 간 상관은 .117 로 관련성이 없는 것으로 나타남

2) 과목별 난이도와 변별도 간 산포도

가) 보건의료정보관리학 난이도와 변별도 간 산포도

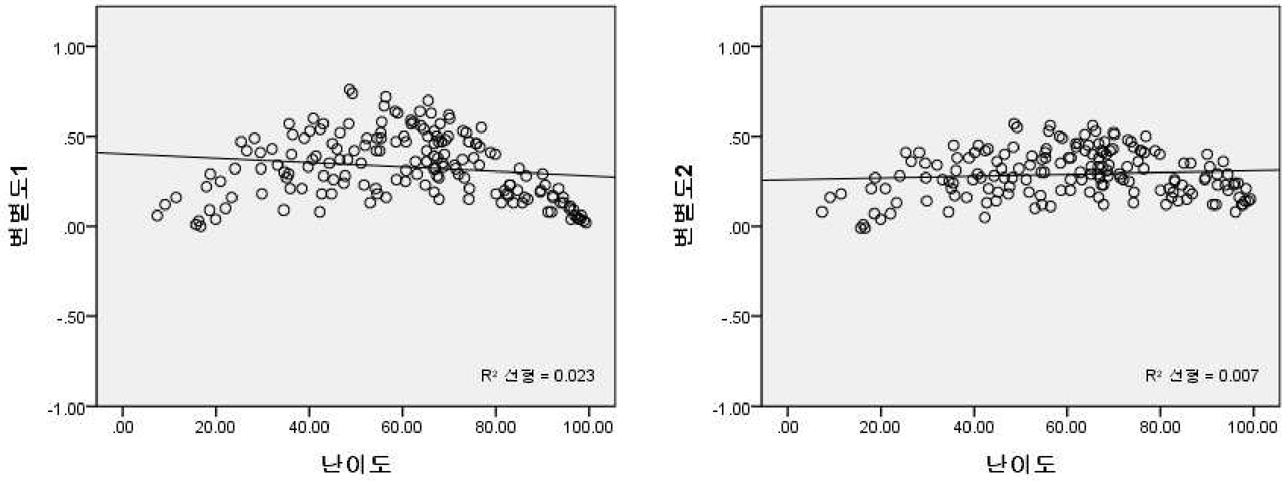

해석

- 보건의료정보관리학 과목 문항을 대상으로 한 난이도 지수와 변별도 1 지수 간 상관은 -.152*로 난이도 지수가 높을수록 변별력이 낮아지는 것으로 나타남
- 난이도 지수와 변별도 2 지수 간 상관은 .086 로 관련성이 없는 것으로 나타남

나) 의료관계법규 난이도와 변별도 간 산포도

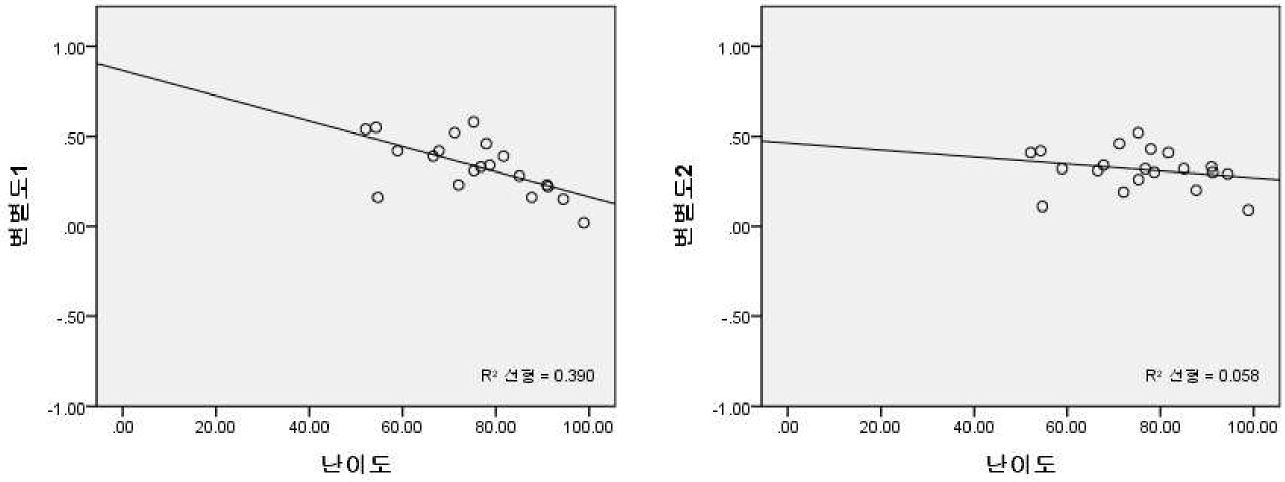

해석

- 의료관계법규 과목 문항을 대상으로 한 난이도 지수와 변별도 1 지수 간 상관은 -.624**로 난이도 지수가 높을수록 변별력이 낮아지는 것으로 나타남
- 난이도 지수와 변별도 2 지수 간 상관은 -.241 로 관련성이 없는 것으로 나타남

다) 실기시험 난이도와 변별도 간 산포도

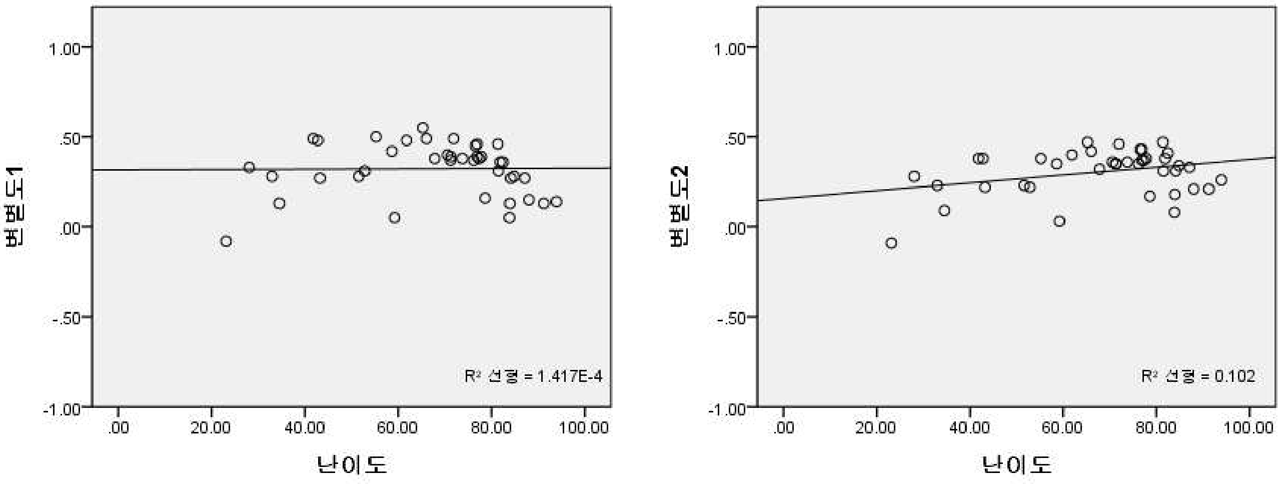

해석

- 실기시험 과목 문항을 대상으로 한 난이도 지수와 변별도 1 지수 간 상관은 .012 으로 관련성이 없는 것으로 나타남
- 난이도 지수와 변별도 2 지수 간 상관은 .320*로 난이도 지수가 높을수록 변별력이 높아지는 것으로 나타남

4. 신뢰도 분석

과목명	문항수	제35회	제36회	제37회	제38회	제39회
전체	230	.954	.966	.961	.961	**0.959**
보건의료정보관리학	170	.942	.954	.947	.945	**0.944**
의료관계법규	20	.687	.760	.711	.685	**0.758**
실기시험	40	.760	.853	.851	.858	**0.825**

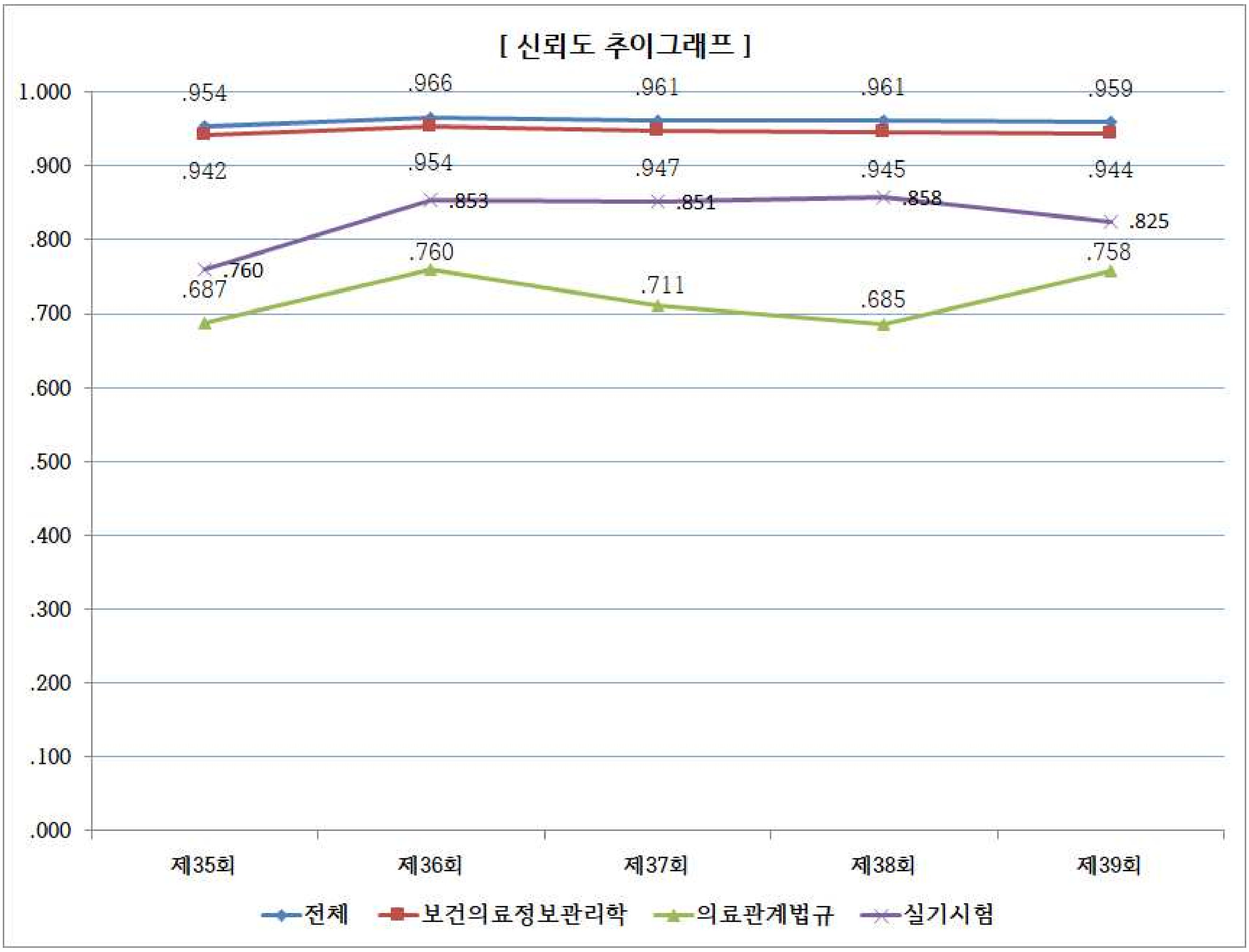

해석

- 전회 대비 전체 문항의 신뢰도는 .002 감소함
- 전회 대비 보건의료정보관리학 과목 문항의 신뢰도는 .001 감소함
- 전회 대비 의료관계법규 과목 문항의 신뢰도는 .073 증가함
- 전회 대비 실기시험 과목 문항의 신뢰도는 .033 감소함

○ 분석결과 관련 문의 : 한국보건의료인국가시험원 연구개발본부 김보현 전임연구원
Tel : 02-2087-8954, FAX : 02-2087-8885
E-mail : kimbohyun@kuksiwon.or.kr
