# Supplementary material for: Item difficulty index, discrimination index, and reliability of the 26 health professions licensing examinations in 2022, Korea: a psychometric study
Source: J Educ Eval Health Prof. 2023 Nov 22;20:31. doi: 10.3352/jeehp.2023.20.31 (PMC11959405; doi:10.3352/jeehp.2023.20.31)
Supplement: Supplementary file 1 — Supplement 1. Item analysis results of 26 health professions licensing examinations administered during late 2022 and early 2023. [file jeehp-20-31_Suppl1.zip › 2022│Γ╡╡ ┴a40╚╕ ┐Σ╛τ║╕╚ú╗τ └┌░▌╜├╟Φ(┐└└n) ║╨╝«░ß░·.pdf]

2022년도 제40회
요양보호사 자격시험(오전)
문항분석 결과

국민이 신뢰하고 감동하는 시험평가기관
한국보건의료인국가시험원
KOREA HEALTH PERSONNEL LICENSING EXAMINATION INSTITUTE

일반 용어 정의

□ **평균**
- 집단에서의 대표적 경향값으로 전체 값을 더하여 총 응시자로 나눈 값

□ **표준편차**
- 평균과 각 점수의 차이인 편차들의 평균으로 점수가 흩어져 분포되어 있는 정도

□ **검사이론**
- 검사와 검사를 구성하고 있는 문항의 양호도를 분석 및 평가하는 방법을 정의한 이론체계
- 대표적으로 고전검사이론과 문항반응이론이 있음

고전검사이론 용어 정의

□ **고전검사이론(Classical Test Theory; CTT)**

- 검사의 질을 분석하는 검사이론 중 한 가지로 19세기 말부터 전개되어 현재까지 주로 사용되고 있는 검사이론임
- 고전검사이론에 의한 문항과 응시자 능력 추정치는 다음과 같음

○ **문항난이도**

· 검사 문항의 쉽고 어려운 정도를 나타내는 지수
· 난이도 지수는 총 반응 수에 대한 정답 반응 수의 비율로 문항의 정답률임
· 문항난이도는 0~100까지의 값을 가짐
· 난이도 값이 큰 경우, 쉬운 문항으로 '난이도가 낮다'라고 해석하며, 난이도 값이 작은 경우, 어려운 문항으로 '난이도가 높다'라고 해석함

○ **문항변별도**

· 각 문항이 응시자의 능력 수준을 변별할 수 있는 정도를 나타내는 지수
· 문항변별도는 -1~+1까지의 값을 가지며, 1에 가까울수록 변별력 크다고 해석함
· 일반적으로 문항변별도가 0.3 이상이면 우수한 문항으로 평가함
· 구하는 방식에는 '상하위집단 구분법', '문항-총점 상관계수' 등이 있음
  1) 변별도 1(상하위구분법): 상위 27%와 하위 27% 집단의 난이도 차이를 구하는 방식
  2) 변별도 2(상관계수법): 문항-총점과의 상관계수로 구하는 방식

○ **신뢰도**

· 시험이 평가하고자 하는 것을 일관성 있게 측정하는가로 시험이 오차없이 정확하게 측정한 정도를 의미함
· 국시원에서는 문항의 내적일관성(Cronbach α)으로 신뢰도를 추정하며 1에 가까울수록 신뢰도가 높다고 해석함

목 차

I. 시행 결과 ···································· 5
1. 시험 현황 ···································· 6
1) 시험명 ···································· 6
2) 시험시행일 ···································· 6
3) 응시현황 ···································· 6
4) 과목별 문항 수, 배점 및 과락 점수 ···································· 6
2. 합격률과 평균성적 ···································· 6
1) 합격 및 불합격 현황 ···································· 6
2) 과목별 과락자수 내역 ···································· 6
3) 전회 대비 합격률과 평균성적 ···································· 7

II. 문항분석 결과 ···································· 9
1. 성적 ···································· 10
1) 전체 성적분포도 ···································· 10
2) 과목별 성적분포도 ···································· 11
2. 난이도와 변별도 ···································· 12
1) 전체 난이도와 변별도 ···································· 12
2) 과목별 난이도와 변별도 ···································· 17
3) 지식수준별 난이도와 변별도 ···································· 24
4) 자료유형별 난이도와 변별도 ···································· 34
3. 난이도와 변별도 간 산포도 ···································· 41
1) 전체 난이도와 변별도 간 산포도 ···································· 41
2) 과목별 난이도와 변별도 간 산포도 ···································· 41
4. 신뢰도 분석 ···································· 43

I. 시행 결과

1. 시험 현황

1) 시험명: 2022년도 제40회 요양보호사 자격시험(오전)※

※2022년도 제40회 요양보호사 자격시험은 오전과 오후 각 1회씩 시행됨

2) 시험시행일: 2022년 8월 6일

3) 응시현황

응시대상자수	결시자수	부정행위자수	응시자 준수사항 위반자 수		응시자수 (%)
			전자기기 등 소지	신분증 미지참	
48,455	2,102	0	0	0	46,328※ (95.6)

※ 46,328명은 응시자대상자(48,455명)에서 결시자와 채점보류자(25명)을 제외한 수치임

4) 과목별 문항 수, 배점 및 과락 점수

교 시	과 목 명	문제 수	배점	총점	합격자 점수기준	
					과목별 합격기준	총점 합격기준
1교시	요양보호론(필기시험)	35	1	35	21점 이상	-
2교시	실기시험	45	1	45	27점 이상	
계		80		80		

2. 합격률과 평균성적

1) 합격 및 불합격 현황

합격자수 (%)	불합격자수(%)				채점보류자수
	평락	과락	기권	계	
**41,244 (89.0)**	0 (0.0)	5,084 (11.0)	0 (0.0)	5,084 (11.0)	25

2) 과목별 과락자수 내역

과목명 / 과락자수	요양보호론(필기시험)	실기시험
과목별 과락자 수	686	2,127
전과목 과락자 수	2,271	

3) 전회 대비 합격률과 평균성적

회차	년도	합격률(%)	평균성적	표준편차	백분율 환산점수
제36회※	2021.08.	90.4	65.4	10.3	81.8
제37회※	2021.11.	90.3	65.7	10.5	82.2
제38회※	2022.02.	91.4	65.6	9.8	82.0
제39회 (오전)	2022.05.	91.7	67.1	10.2	83.9
제39회 (오후)	2022.05.	90.4	65.8	10.5	82.3
**제40회 (오전)**	**2022.08.**	**89.0**	**65.1**	**10.6**	**81.4**

※2021년도 제36회~제38회 요양보호사 자격시험은 오전과 오후 각 1회씩 시행되었으며 이를 평균하여 통합값을 산출함(난이도와 변별도, 신뢰도 분석에도 동일하게 적용함)

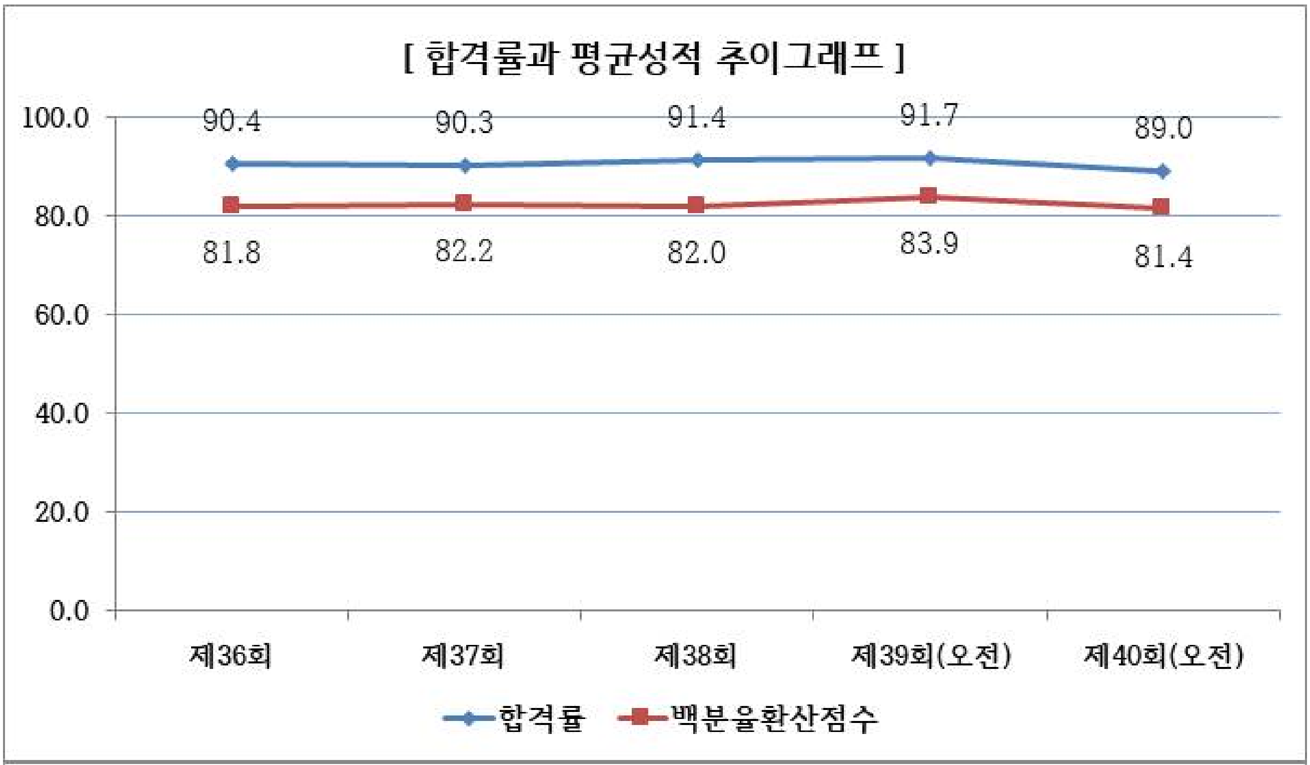

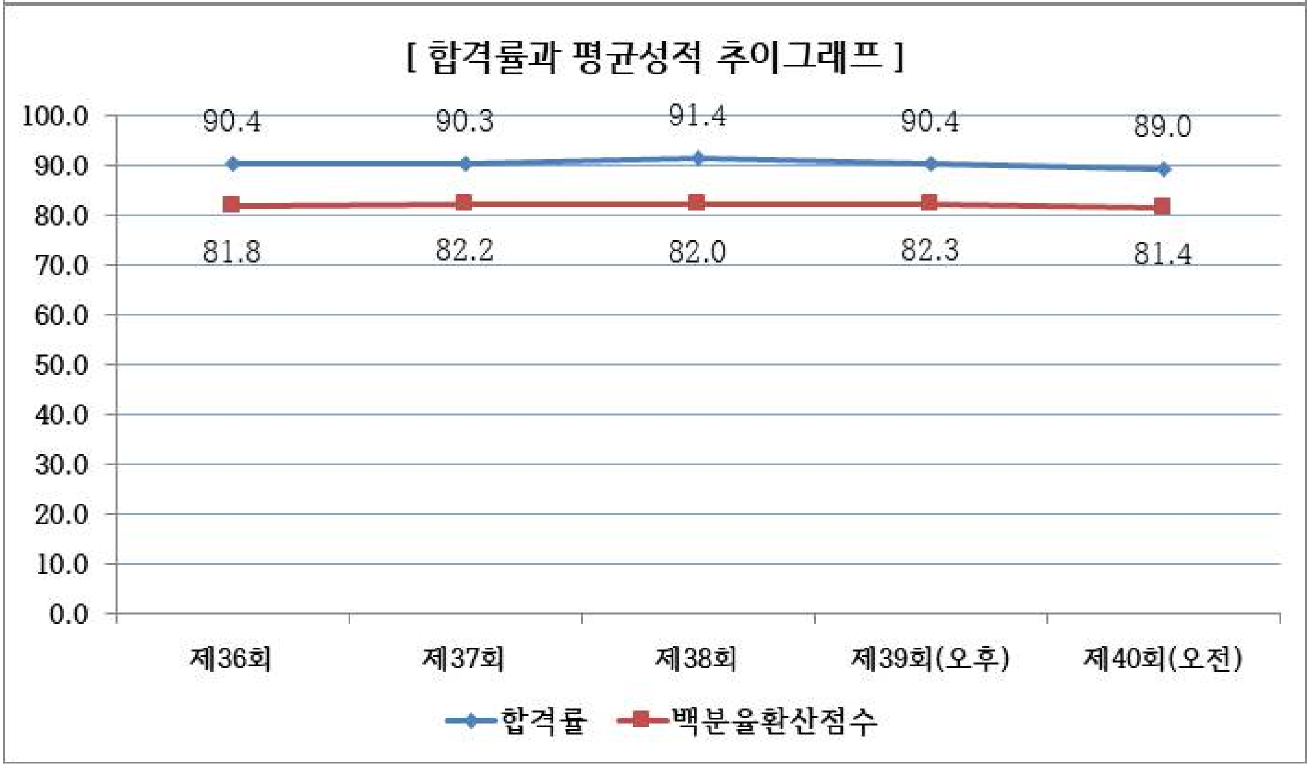

해석

- 전회(오전) 대비 합격률은 2.7%, 백분율 환산점수는 2.5 점 감소함
- 표준편차는 0.4 점 증가함
- 전회(오후) 대비 합격률은 1.4%, 백분율 환산점수는 0.9 점 감소함
- 표준편차는 0.1 점 증가함

Ⅱ. 문항분석 결과

1. 성적(※ 2022.9.30.을 기준으로 한 자료임)

1) 전체 성적분포도

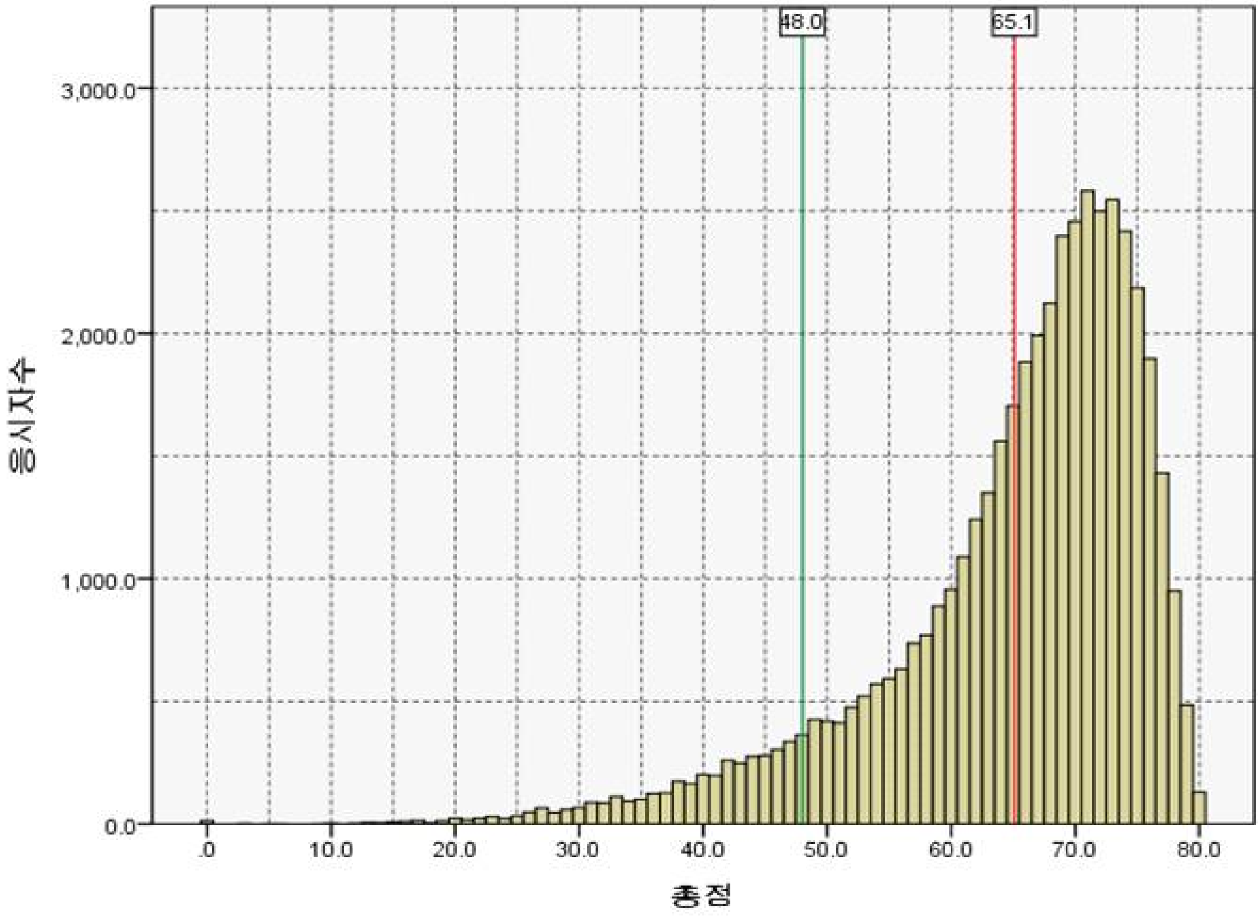

응시자	총점	합격선	평균성적	표준편차
46,353※	80.0	48.0	65.1	10.7

※ 46,353명은 전체응시자(46,328명)에서 채점보류자(25명)를 포함한 수치임

2) 과목별 성적분포도

가) 요양보호론 성적분포도

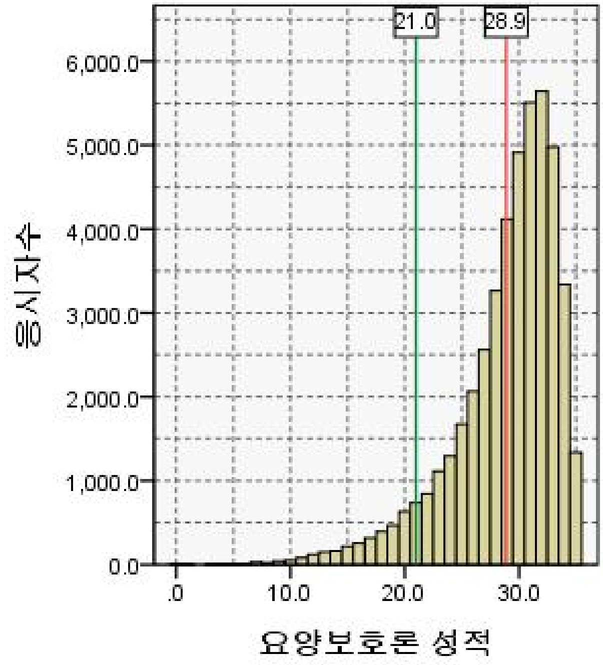

총점	과락선	평균성적	표준편차
35.0	21.0	28.9	4.7

나) 실기시험 성적분포도

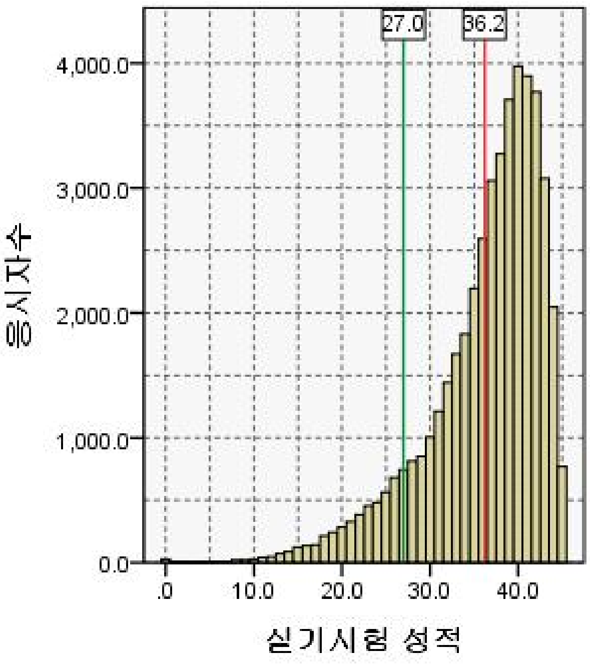

총점	과락선	평균성적	표준편차
45.0	27.0	36.2	6.6

2. 난이도와 변별도

1) 전체 난이도와 변별도

가) 전회 대비 전체 난이도와 변별도

회차	난이도		변별도1		변별도2	
	평균	표준편차	평균	표준편차	평균	표준편차
제36회	81.8	13.4	.28	.13	.35	.10
제37회	82.1	13.8	.28	.15	.36	.10
제38회	82.0	14.4	.27	.13	.33	.08
제39회 (오전)	83.9	13.0	.27	.14	.36	.08
제39회 (오후)	82.3	14.0	.29	.14	.35	.10
**제40회 (오전)**	**81.4**	**14.0**	**.30**	**.14**	**.35**	**.09**

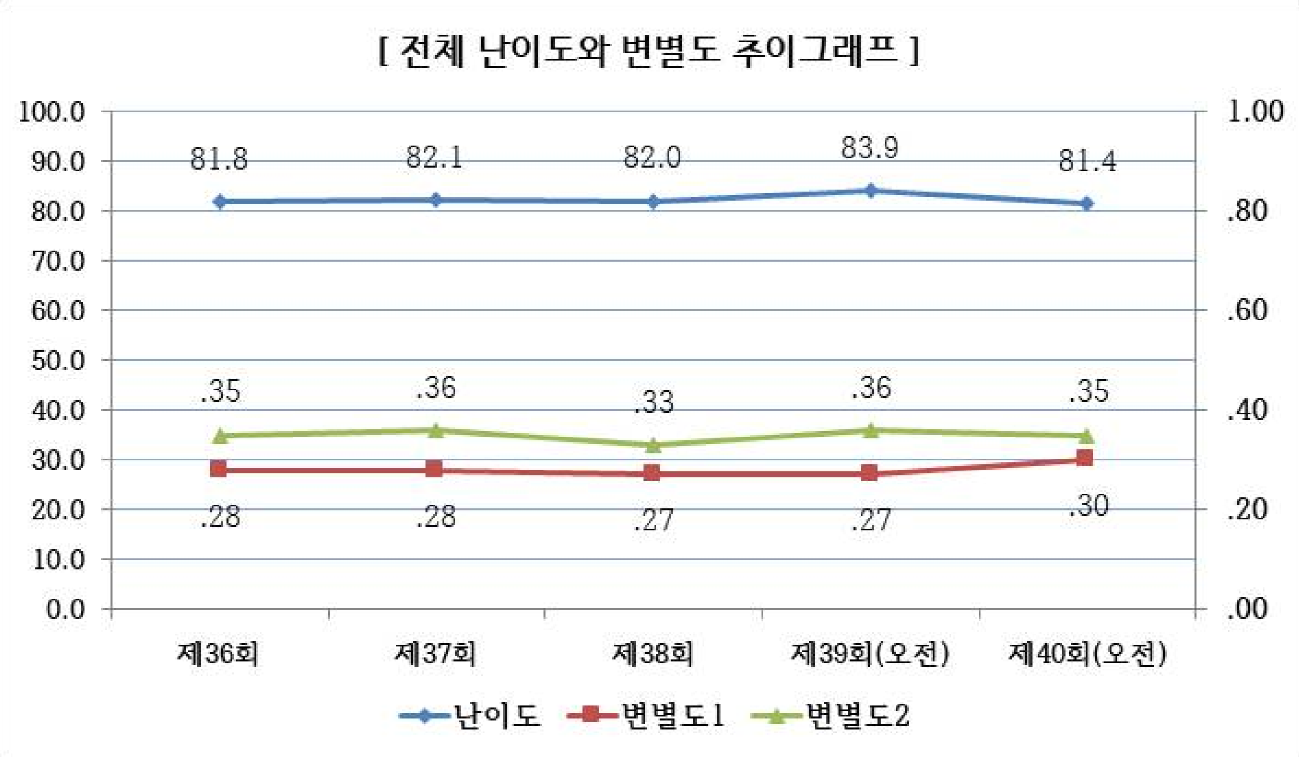
[전체 난이도와 변별도 추이그래프]
100.0
90.0
80.0
70.0
60.0
50.0
40.0
30.0
20.0
10.0
0.0
1.00
.80
.60
.40
.20
.00
81.8
82.1
82.0
83.9
81.4
.35
.36
.33
.36
.35
.28
.28
.27
.27
.30
제36회
제37회
제38회
제39회(오전)
제40회(오전)
난이도
변별도1
변별도2

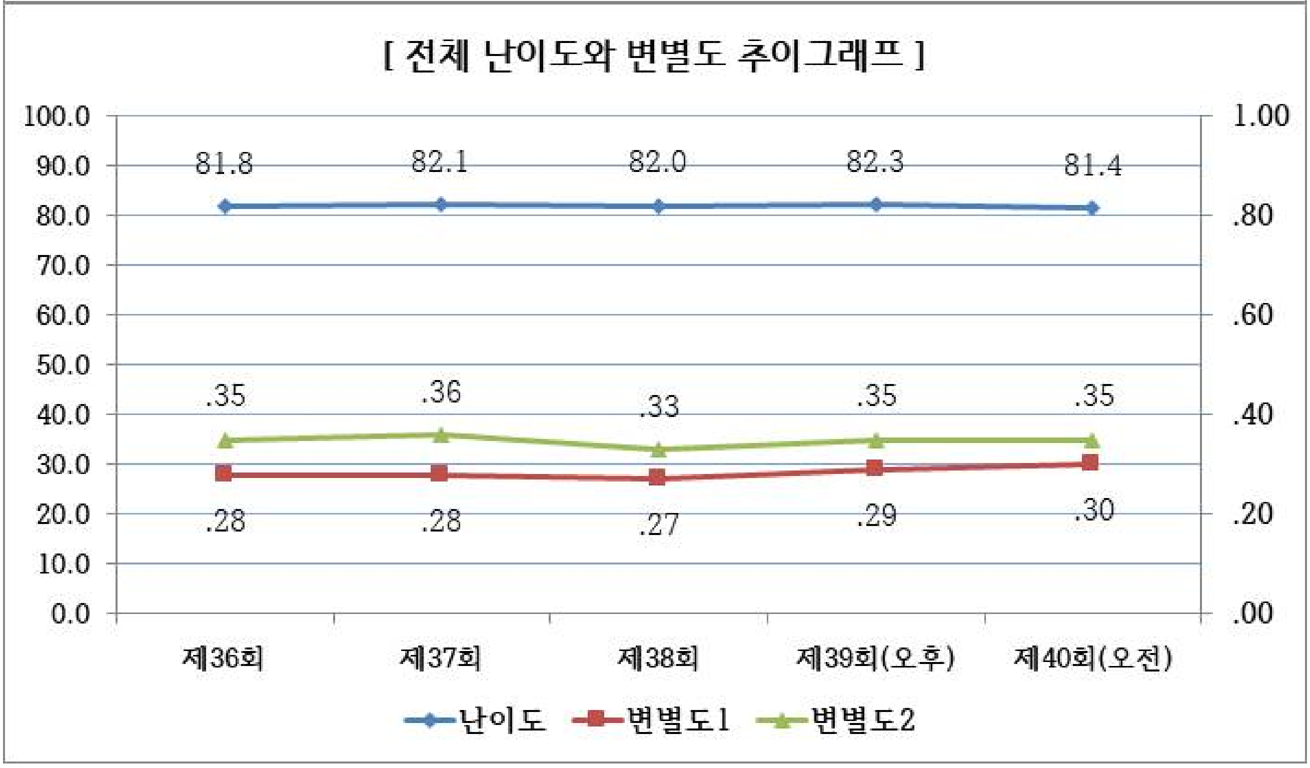
[전체 난이도와 변별도 추이그래프]
100.0
90.0
80.0
70.0
60.0
50.0
40.0
30.0
20.0
10.0
0.0
1.00
.80
.60
.40
.20
.00
81.8
82.1
82.0
82.3
81.4
.35
.36
.33
.35
.35
.28
.28
.27
.29
.30
제36회
제37회
제38회
제39회(오후)
제40회(오전)
난이도
변별도1
변별도2

해석

- 전회(오전) 대비 난이도 지수는 2.5 감소함
- 전회(오전) 대비 변별도1 지수는 0.03 증가함
- 전회(오전) 대비 변별도2 지수는 0.01 감소함
- 전회(오후) 대비 난이도 지수는 0.9 감소함
- 전회(오후) 대비 변별도1 지수는 0.01 증가함
- 전회(오후) 대비 변별도2 지수는 동일함

나) 전체 난이도와 변별도 분포도 및 비율분석

(1) 전체 난이도 분포도 및 비율분석

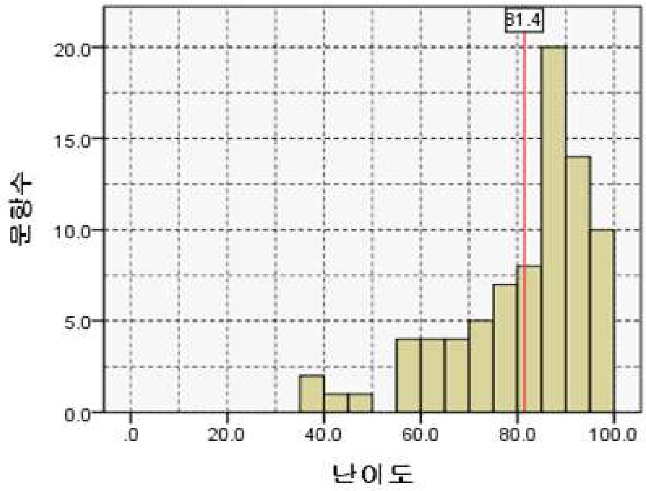

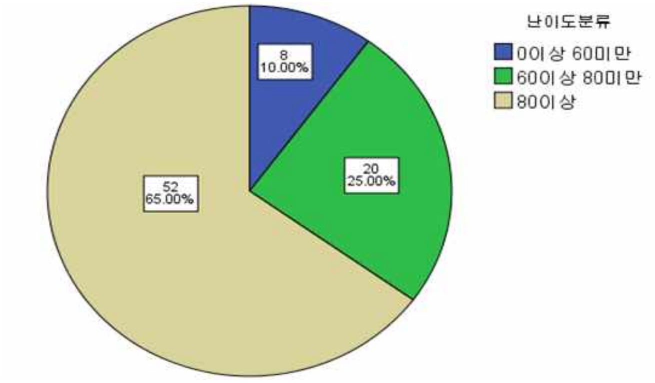

총점	난이도	표준편차
80	81.4	14.0

난이도	문항수	비율(%)
0~60미만	8	10.0
60~80미만	20	25.0
80~100	52	65.0
전체	80	100.0

(2) 전체 변별도1 분포도 및 비율분석

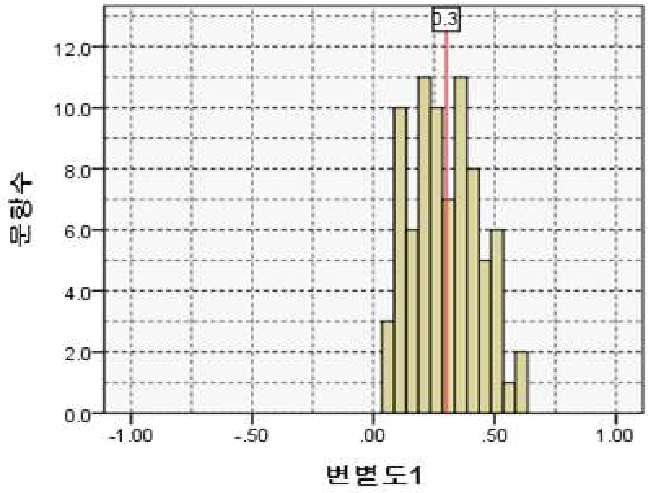

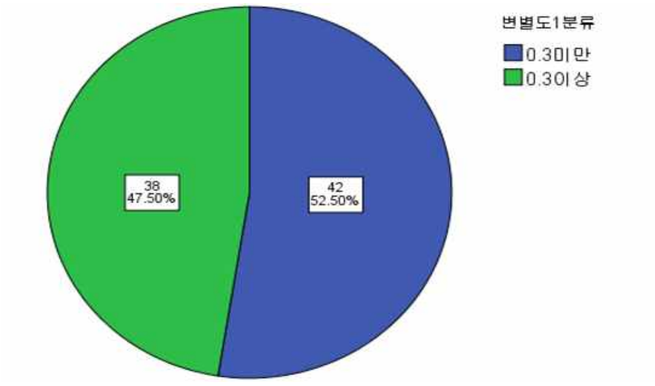

총점	변별도1	표준편차
80	.30	.14

변별도1	문항수	비율(%)
0.3미만	42	52.5
0.3이상	38	47.5
전체	80	100.0

(3) 전체 변별도2 분포도 및 비율분석

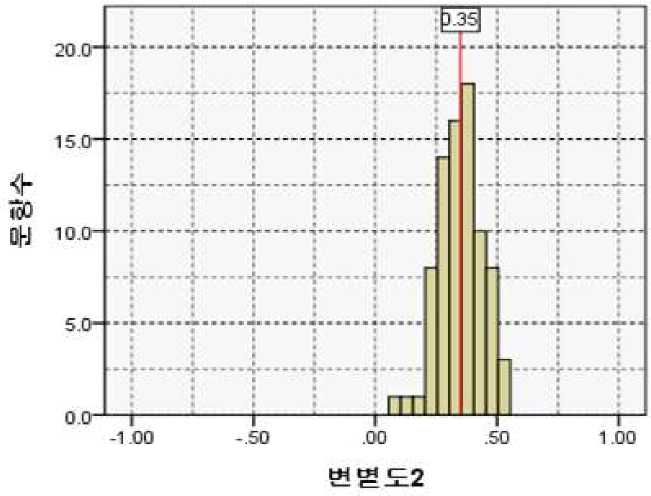

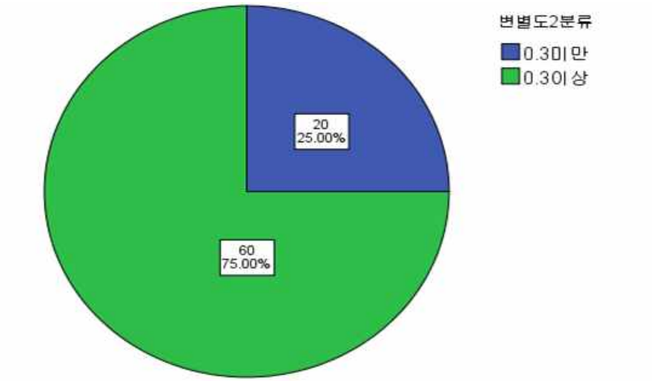

총점	변별도2	표준편차
80	.35	.09

변별도2	문항수	비율(%)
0.3미만	20	25.0
0.3이상	60	75.0
전체	80	100.0

해석

- 난이도 지수가 80에서 100 사이인 문항이 전체 80문항 중 52문항으로 가장 많았으며, 차례로 60이상 80미만인 문항이 20문항, 60미만인 문항이 8문항인 것으로 나타남
- 변별도1 지수를 기준으로 분류하였을 때, 0.3미만인 문항이 42문항으로 0.3이상인 문항이 38문항인 것에 비해 더 많이 나타남
- 변별도2 지수를 기준으로 분류하였을 때, 0.3미만인 문항이 20문항으로 0.3이상인 문항이 60문항인 것에 비해 더 적게 나타남

2) 과목별 난이도와 변별도

가) 전회 대비 과목별 난이도와 변별도

(1) 전회 대비 요양보호론 난이도와 변별도

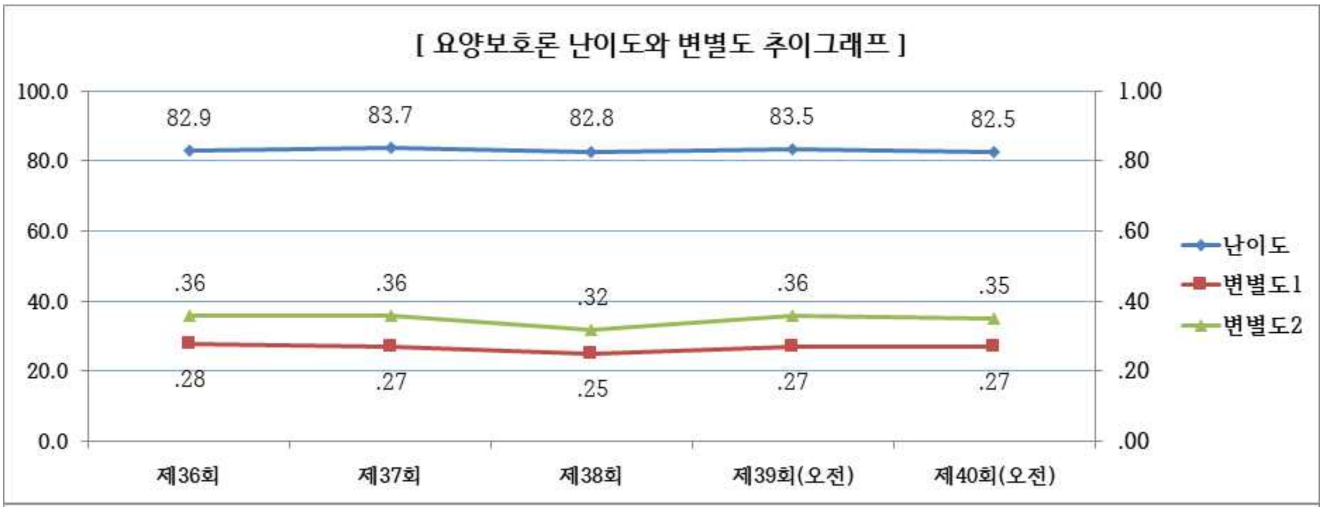

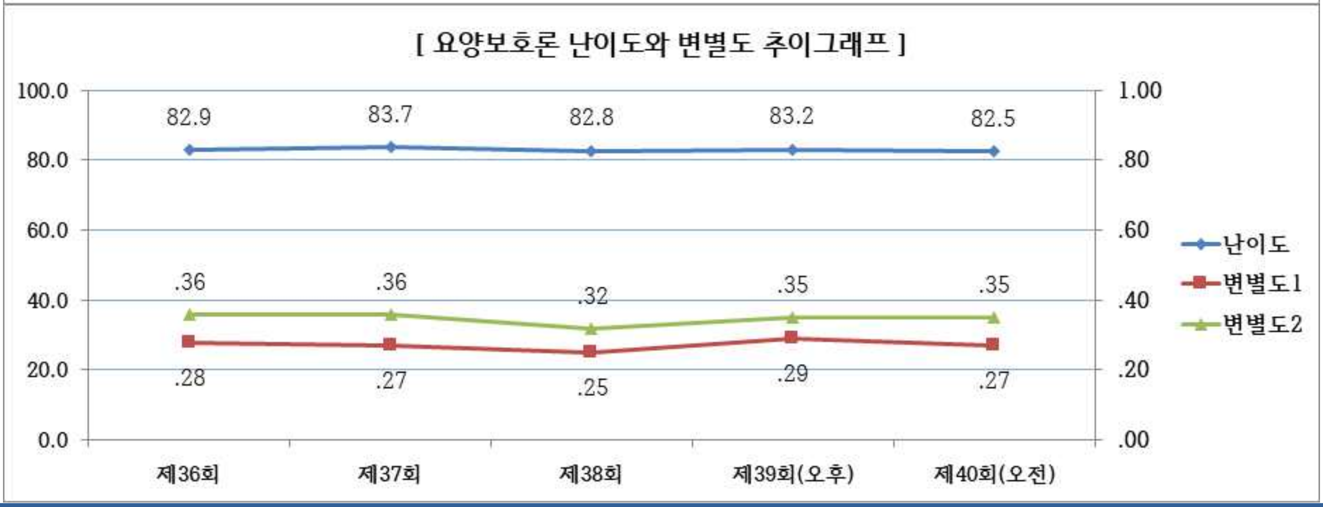

회차	난이도		변별도1		변별도2	
	평균	표준편차	평균	표준편차	평균	표준편차
제36회	82.9	13.0	.28	.14	.36	.11
제37회	83.7	13.4	.27	.16	.36	.11
제38회	82.8	15.3	.25	.13	.32	.09
제39회(오전)	83.5	13.8	.27	.14	.36	.07
제39회(오후)	83.2	12.3	.29	.14	.35	.10
**제40회(오전)**	82.5	16.3	.27	.15	.35	.09

해석
- 전회(오전) 대비 요양보호론 과목의 난이도 지수는 1.0 감소함<br>- 전회(오전) 대비 요양보호론 과목의 변별도1 지수는 동일함<br>- 전회(오전) 대비 요양보호론 과목의 변별도2 지수는 0.01 감소함<br>- 전회(오후) 대비 요양보호론 과목의 난이도 지수는 0.7 감소함<br>- 전회(오후) 대비 요양보호론 과목의 변별도1 지수는 0.02 감소함<br>- 전회(오후) 대비 요양보호론 과목의 변별도2 지수는 동일함

(2) 전회 대비 실기시험 난이도와 변별도

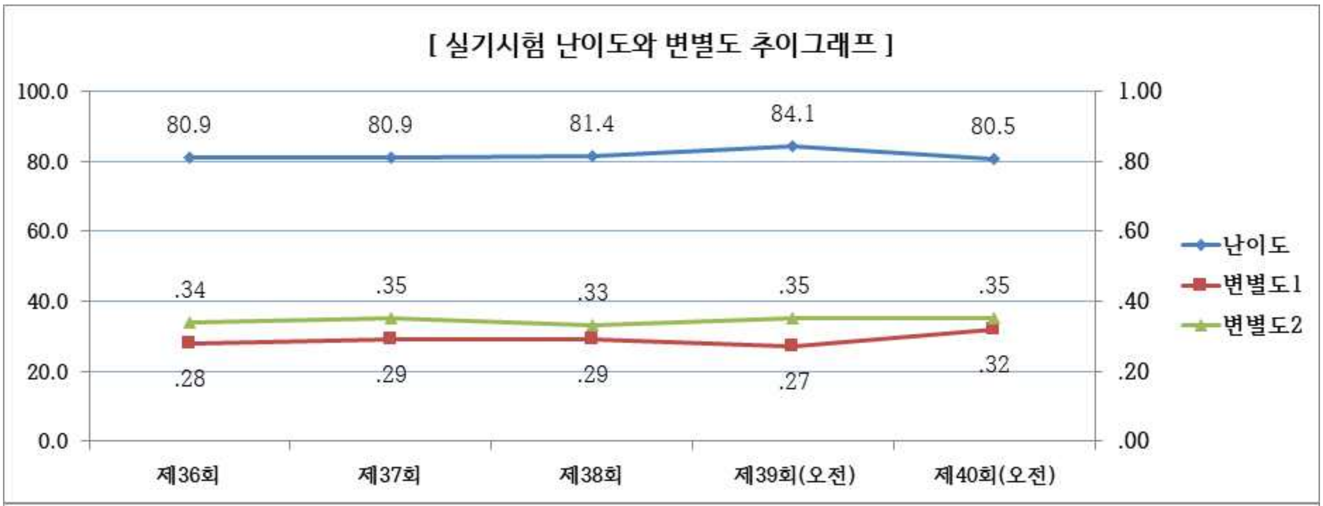

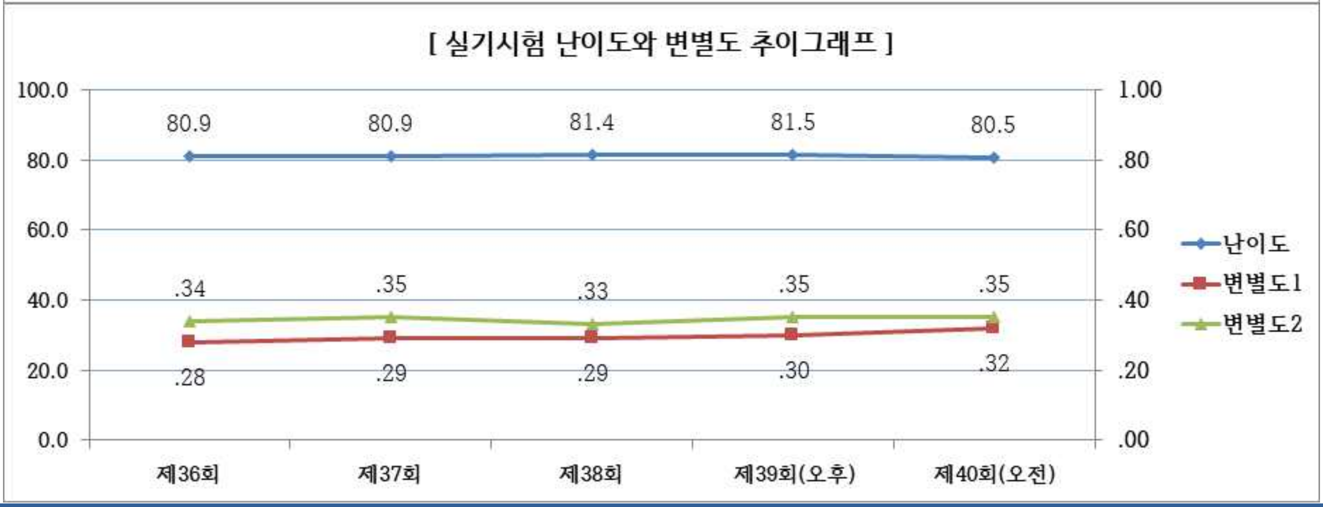

회차	난이도		변별도1		변별도2	
	평균	표준편차	평균	표준편차	평균	표준편차
제36회	80.9	13.7	.28	.12	.34	.10
제37회	80.9	14.0	.29	.14	.35	.09
제38회	81.4	13.7	.29	.13	.33	.08
제39회 (오전)	84.1	12.6	.27	.13	.35	.08
제39회 (오후)	81.5	15.2	.30	.15	.35	.09
**제40회 (오전)**	80.5	12.0	.32	.12	.35	.08

해석

- 전회(오전) 대비 실기시험 과목의 난이도 지수는 3.6 감소함
- 전회(오전) 대비 실기시험 과목의 변별도 1 지수는 0.05 증가함
- 전회(오전) 대비 실기시험 과목의 변별도 2 지수는 동일함
- 전회(오후) 대비 실기시험 과목의 난이도 지수는 1.0 감소함
- 전회(오후) 대비 실기시험 과목의 변별도 1 지수는 0.02 증가함
- 전회(오후) 대비 실기시험 과목의 변별도 2 지수는 동일함

나) 과목별 난이도와 변별도 분포도 및 비율분석

(1) 요양보호론 난이도와 변별도 분포도 및 비율분석

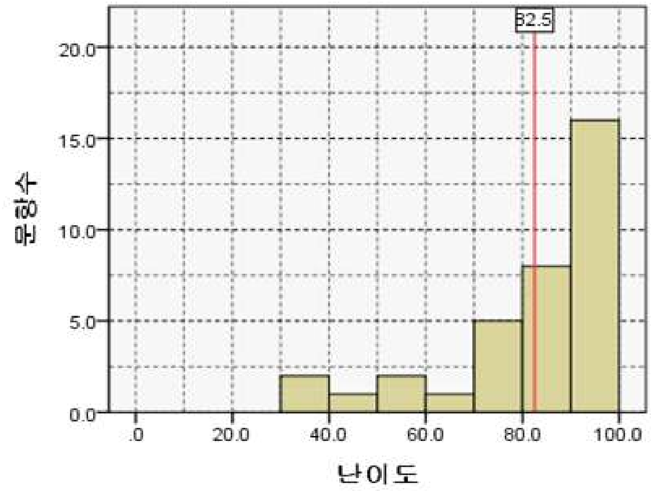

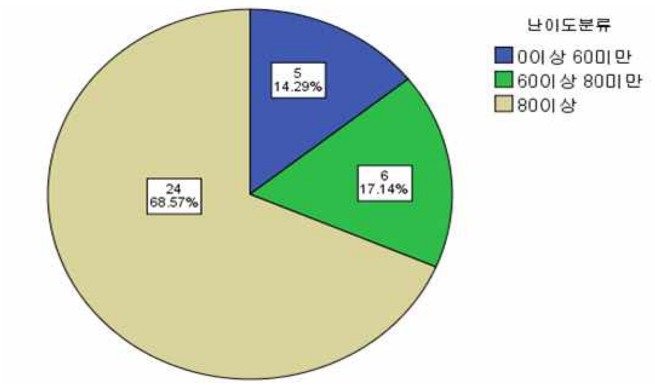

총점	난이도	표준편차	난이도	문항수	비율(%)
35	82.5	16.3	0~60미만	5	14.3
			60~80미만	6	17.1
			80~100	24	68.6
			전체	35	100.0

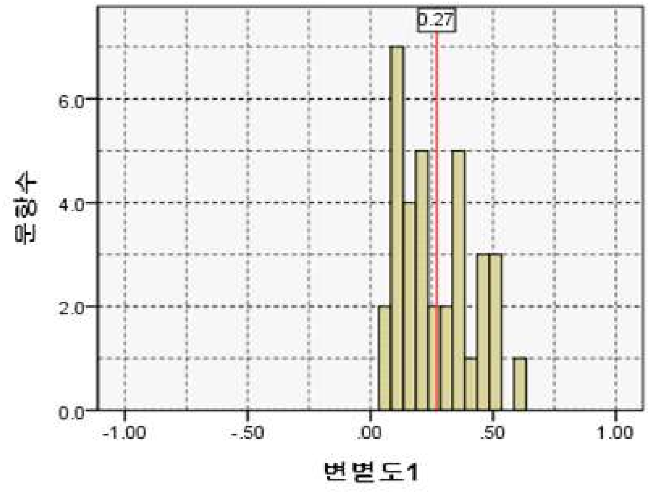

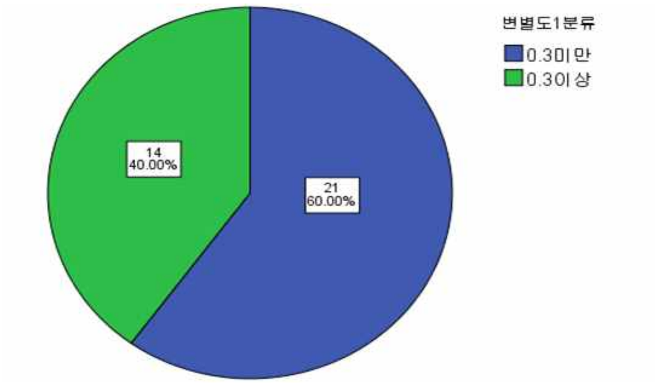

총점	변별도1	표준편차	변별도1	문항수	비율(%)
35	.27	.15	0.3미만	21	60.0
			0.3이상	14	40.0
			전체	35	100.0

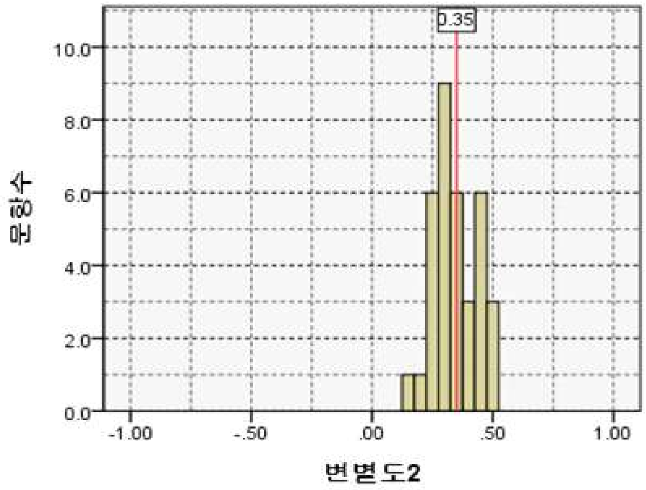

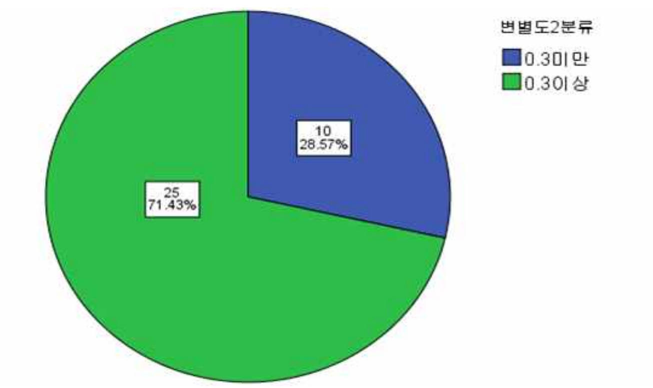

총점	변별도2	표준편차
35	.35	.09

변별도2	문항수	비율(%)
0.3미만	10	28.6
0.3이상	25	71.4
전체	35	100.0

해석

- 요양보호론 과목에서 난이도 지수가 80 에서 100 사이인 문항이 전체 35 문항 중 24 문항으로 가장 많았으며, 차례로 60 이상 80 미만인 문항이 6 문항, 60 미만인 문항이 5 문항인 것으로 나타남
- 변별도 1 지수를 기준으로 분류하였을 때, 0.3 미만인 문항이 21 문항으로 0.3 이상인 문항이 14 문항인 것에 비해 더 많이 나타남
- 변별도 2 지수를 기준으로 분류하였을 때, 0.3 미만인 문항이 10 문항으로 0.3 이상인 문항이 25 문항인 것에 비해 더 적게 나타남

(2) 실기시험 난이도와 변별도 분포도 및 비율분석

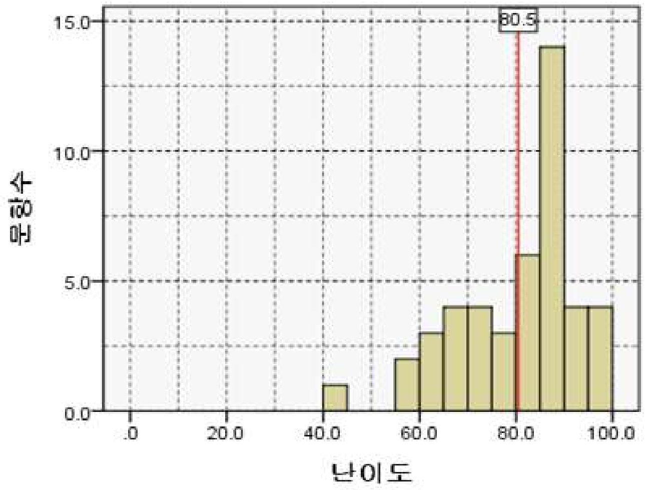

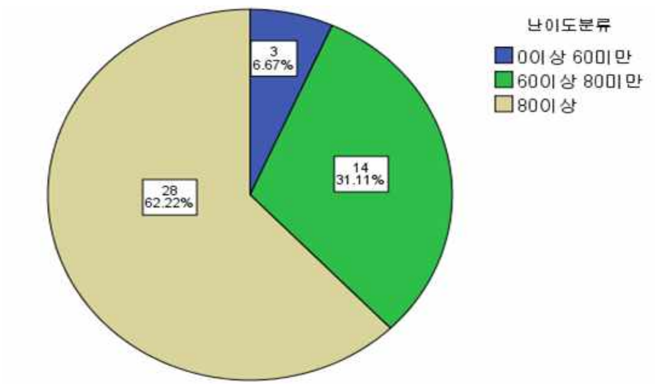

총점	난이도	표준편차
45	80.5	12.0

난이도	문항수	비율(%)
0~60미만	3	6.7
60~80미만	14	31.1
80~100	28	62.2
전체	45	100.0

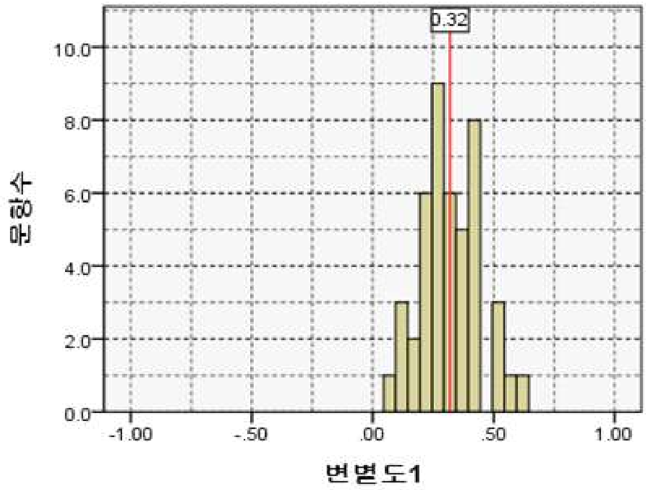

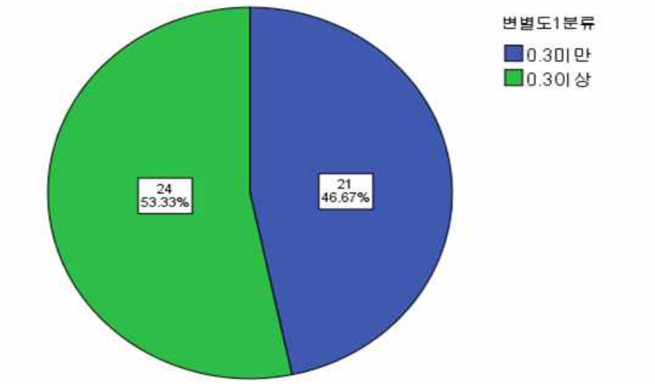

총점	변별도1	표준편차
45	.32	.12

변별도1	문항수	비율(%)
0.3미만	21	46.7
0.3이상	24	53.3
전체	45	100.0

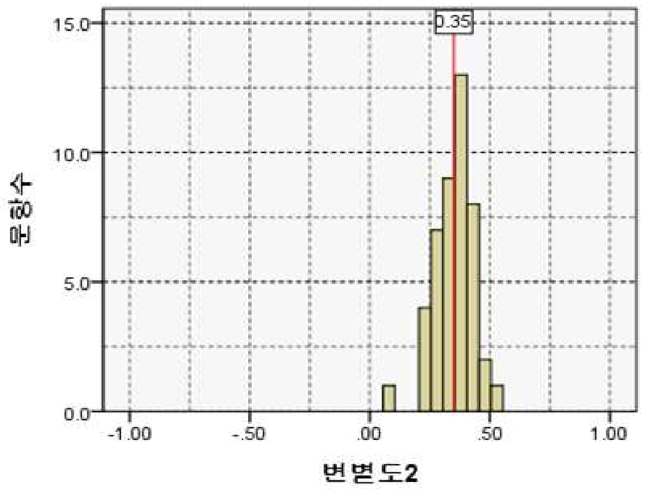

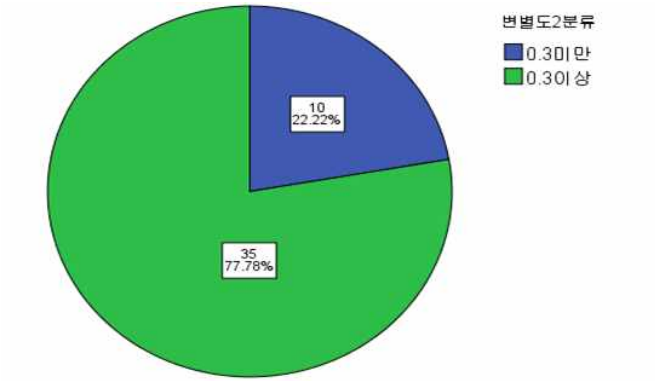

총점	변별도2	표준편차	변별도2	문항수	비율(%)
45	.35	.08	0.3미만	10	22.2
			0.3이상	35	77.8
			전체	45	100.0

해석

- 실기시험 과목에서 난이도 지수가 80 에서 100 사이인 문항이 전체 45 문항 중 28 문항으로 가장 많았으며, 다음으로 60 이상 80 미만인 문항이 14 문항, 60 미만인 문항이 3 문항인 것으로 나타남
- 변별도 1 지수를 기준으로 분류하였을 때, 0.3 미만인 문항이 21 문항으로 0.3 이상인 문항이 24 문항인 것에 비해 더 적게 나타남
- 변별도 2 지수를 기준으로 분류하였을 때, 0.3 미만인 문항이 10 문항으로 0.3 이상인 문항이 35 문항인 것에 비해 더 적게 나타남

3) 지식수준별 난이도와 변별도

가) 전회 대비 지식수준별 난이도와 변별도

(1) 전회 대비 암기형 난이도와 변별도

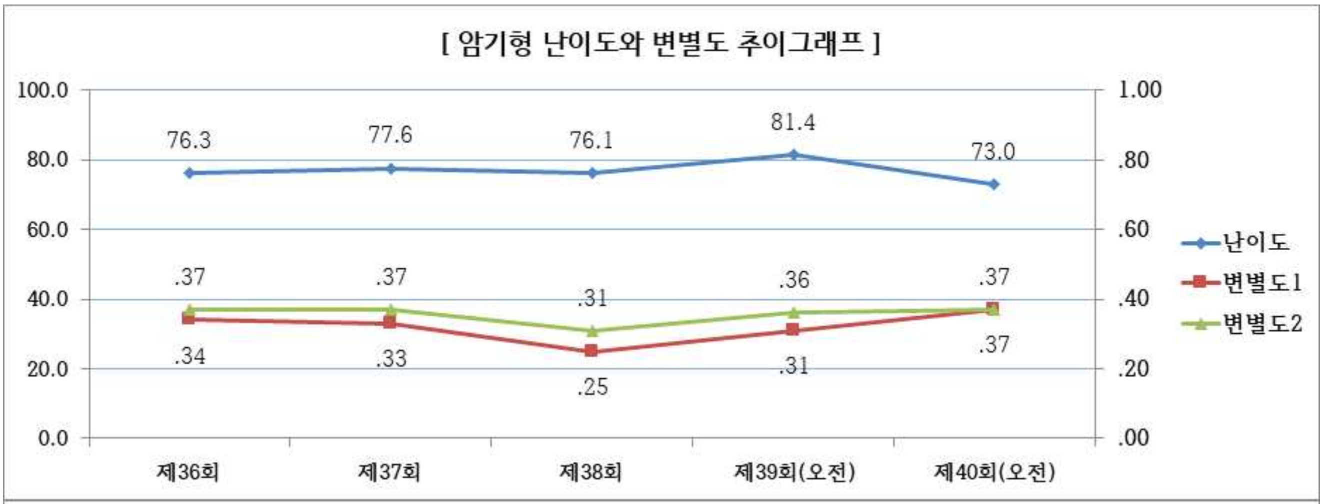

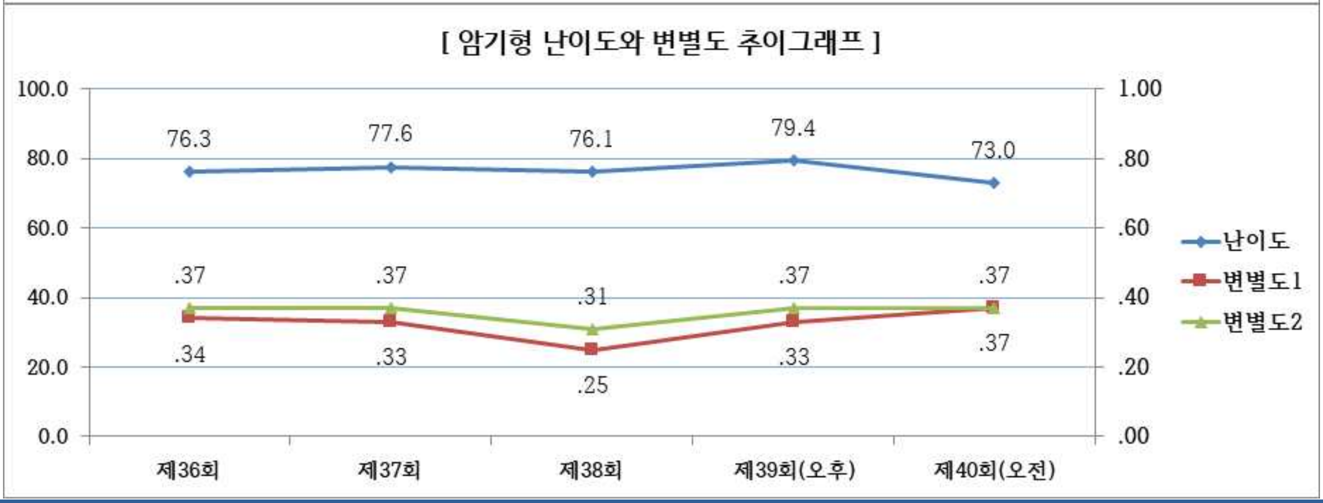

회차	난이도		변별도1		변별도2	
	평균	표준편차	평균	표준편차	평균	표준편차
제36회	76.3	14.4	.34	.12	.37	.12
제37회	77.6	16.7	.33	.15	.37	.09
제38회	76.1	26.5	.25	.11	.31	.10
제39회 (오전)	81.4	12.9	.31	.12	.36	.06
제39회 (오후)	79.4	14.5	.33	.14	.37	.12
**제40회 (오전)**	73.0	19.4	.37	.12	.37	.10

해석

- 전회(오전) 대비 암기형 문항의 난이도 지수는 8.4 감소함
- 전회(오전) 대비 암기형 문항의 변별도1 지수는 0.06 증가함
- 전회(오전) 대비 암기형 문항의 변별도2 지수는 0.01 증가함
- 전회(오후) 대비 암기형 문항의 난이도 지수는 6.4 감소함
- 전회(오후) 대비 암기형 문항의 변별도1 지수는 0.04 증가함
- 전회(오후) 대비 암기형 문항의 변별도2 지수는 동일함

(2) 전회 대비 해석형 난이도와 변별도

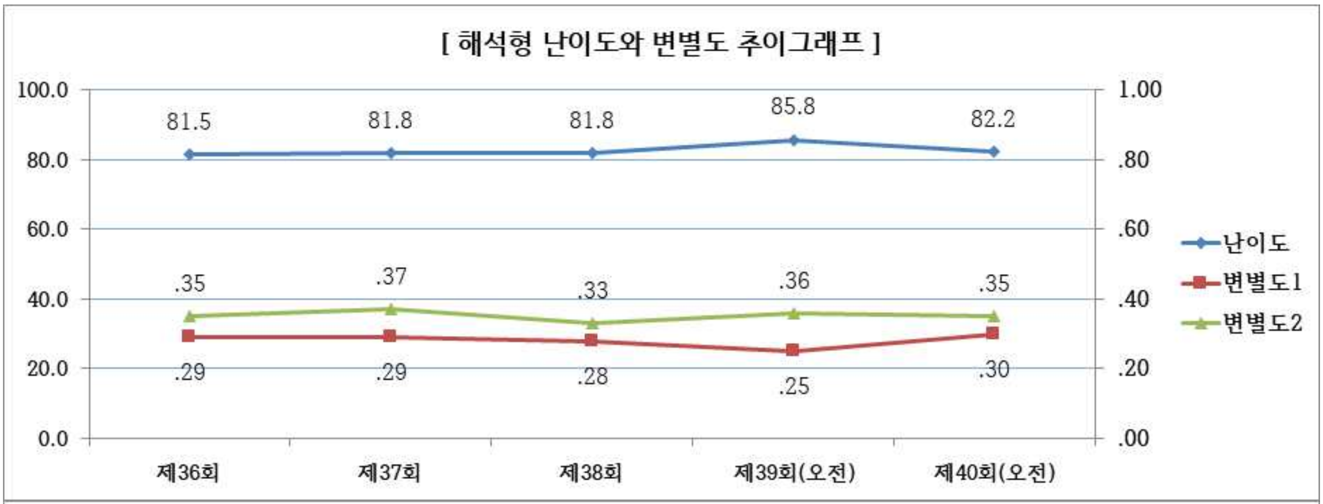

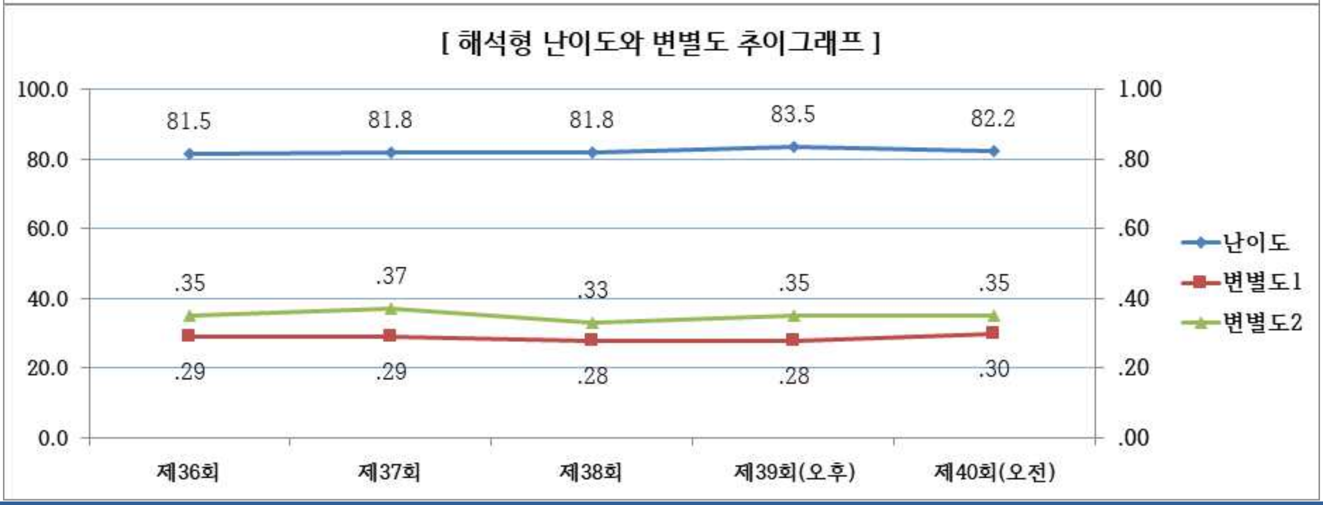

회차	난이도		변별도1		변별도2	
	평균	표준편차	평균	표준편차	평균	표준편차
제36회	81.5	12.7	.29	.13	.35	.10
제37회	81.8	14.2	.29	.15	.37	.10
제38회	81.8	14.1	.28	.13	.33	.07
제39회 (오전)	85.8	11.8	.25	.13	.36	.08
제39회 (오후)	83.5	13.5	.28	.14	.35	.08
**제40회 (오전)**	**82.2**	**12.5**	**.30**	**.14**	**.35**	**.08**

해석

- 전회(오전) 대비 해석형 문항의 난이도 지수는 3.6 감소함
- 전회(오전) 대비 해석형 문항의 변별도 1 지수는 0.05 증가함
- 전회(오전) 대비 해석형 문항의 변별도 2 지수는 0.01 감소함
- 전회(오후) 대비 해석형 문항의 난이도 지수는 1.3 감소함
- 전회(오후) 대비 해석형 문항의 변별도 1 지수는 0.02 증가함
- 전회(오후) 대비 해석형 문항의 변별도 2 지수는 동일함

(3) 전회 대비 해결형 난이도와 변별도

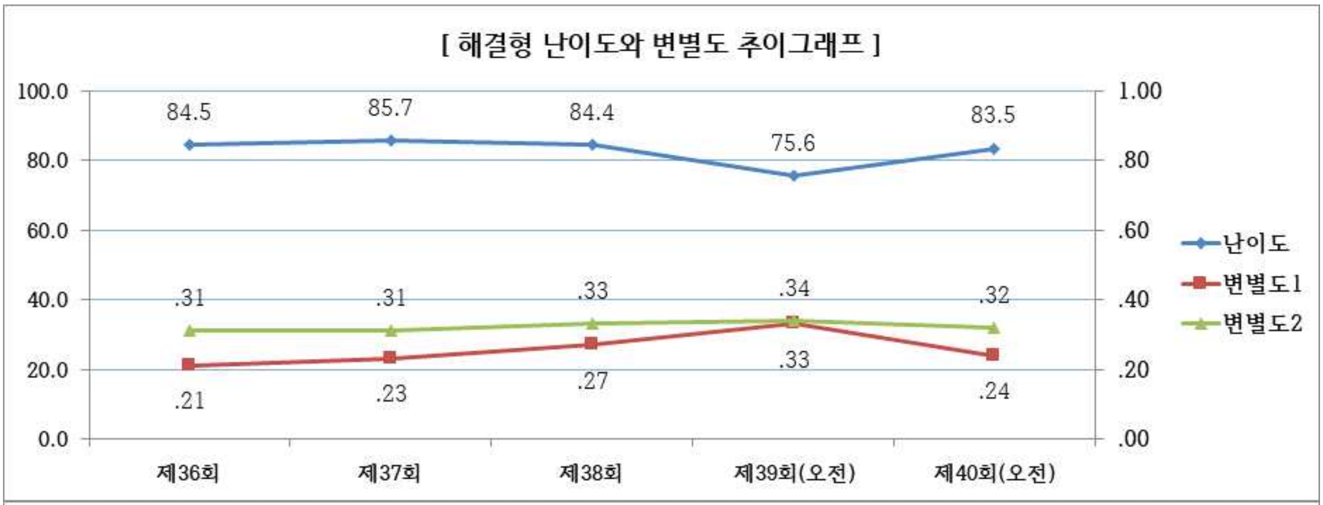

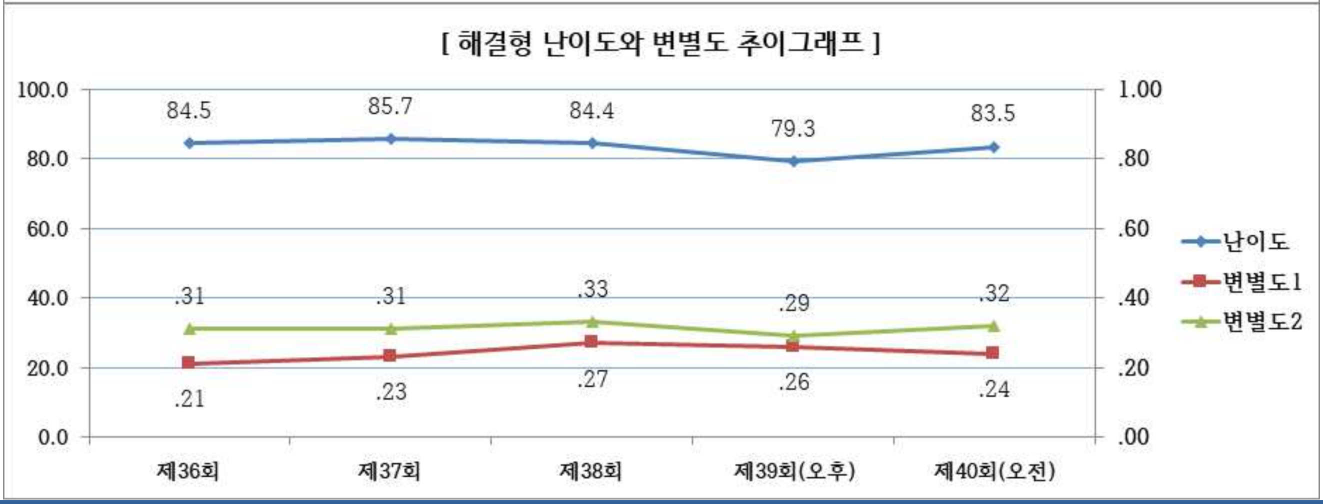

회차	난이도		변별도1		변별도2	
	평균	표준편차	평균	표준편차	평균	표준편차
제36회	84.5	14.6	.21	.09	.31	.10
제37회	85.7	9.7	.23	.14	.31	.10
제38회	84.4	9.7	.27	.13	.33	.08
제39회 (오전)	75.6	19.0	.33	.21	.34	.06
제39회 (오후)	79.3	16.6	.26	.15	.29	.12
**제40회 (오전)**	**83.5**	**15.3**	**.24**	**.10**	**.32**	**.10**

해석

- 전회(오전) 대비 해결형 문항의 난이도 지수는 7.9 증가함
- 전회(오전) 대비 해결형 문항의 변별도 1 지수는 0.09 감소함
- 전회(오전) 대비 해결형 문항의 변별도 2 지수는 0.02 감소함
- 전회(오후) 대비 해결형 문항의 난이도 지수는 4.2 증가함
- 전회(오후) 대비 해결형 문항의 변별도 1 지수는 0.02 감소함
- 전회(오후) 대비 해결형 문항의 변별도 2 지수는 0.03 증가함

나) 지식수준별 난이도와 변별도 분포도 및 비율분석

(1) 암기형 난이도와 변별도 분포도 및 비율분석

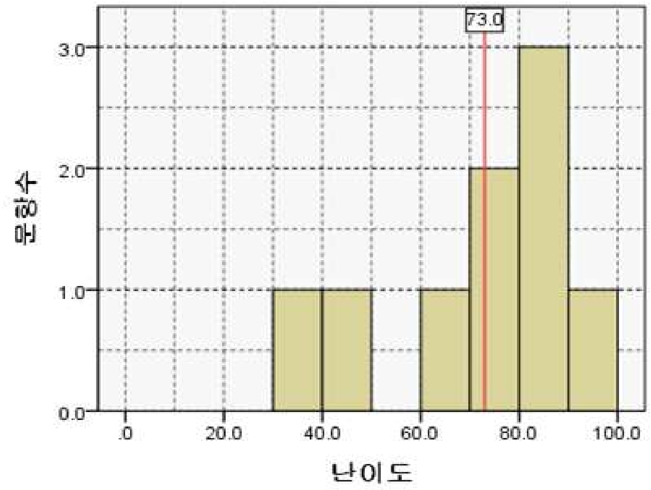

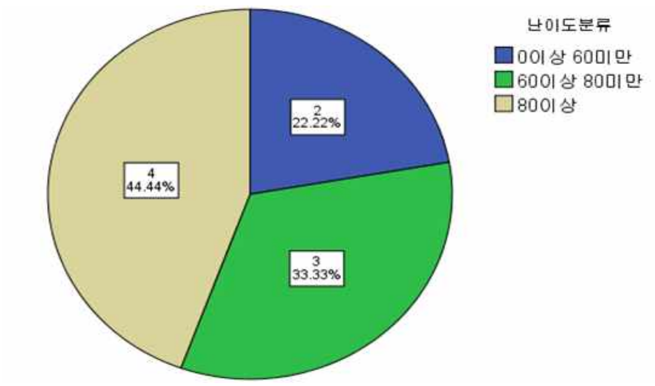

총점	난이도	표준편차
9	73.0	19.4

난이도	문항수	비율(%)
0~60미만	2	22.2
60~80미만	3	33.3
80~100	4	44.4
전체	9	100.0

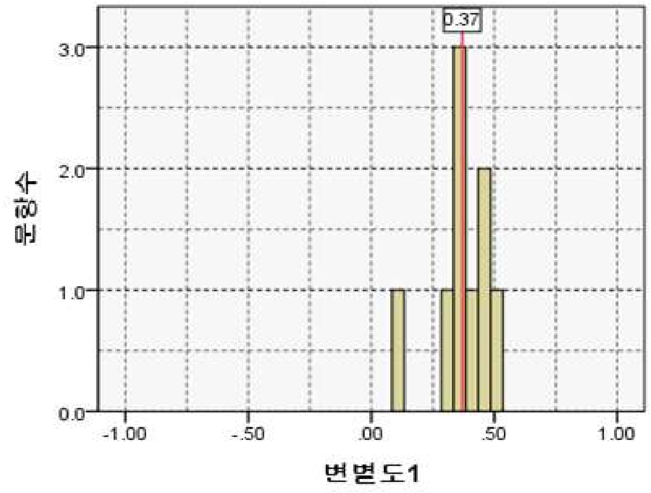

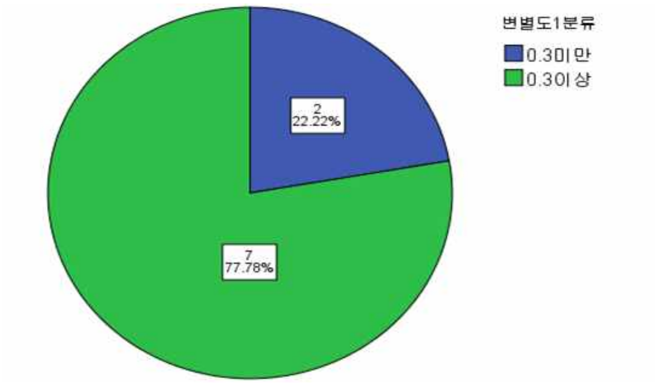

총점	변별도1	표준편차
9	.37	.12

변별도1	문항수	비율(%)
0.3미만	2	22.2
0.3이상	7	77.8
전체	9	100.0

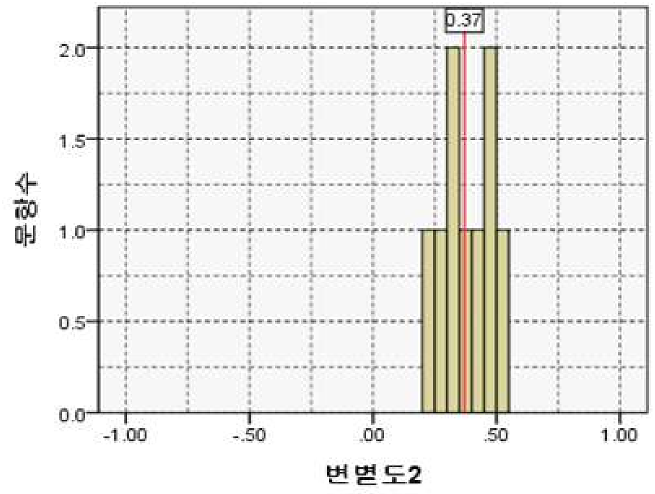

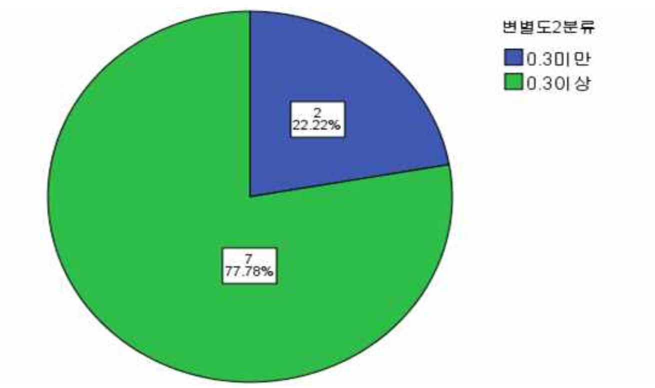

총점	변별도2	표준편차
9	.37	.10

변별도2	문항수	비율(%)
0.3미만	2	22.2
0.3이상	7	77.8
전체	9	100.0

해석

- 암기형 문항에서 난이도 지수가 80 에서 100 사이인 문항이 전체 9 문항 중 4 문항으로 가장 많았으며, 다음으로 60 이상 80 미만인 문항이 3 문항, 60 미만인 문항이 2 문항인 것으로 나타남
- 변별도 1 지수를 기준으로 분류하였을 때, 0.3 미만인 문항이 2 문항으로 0.3 이상인 문항이 7 문항인 것에 비해 더 적게 나타남
- 변별도 2 지수를 기준으로 분류하였을 때, 0.3 미만인 문항이 2 문항으로 0.3 이상인 문항이 7 문항인 것에 비해 더 적게 나타남

(2) 해석형 난이도와 변별도 분포도 및 비율분석

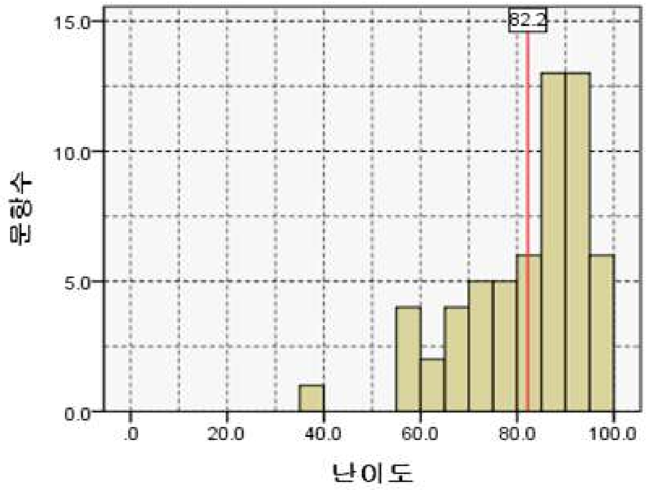

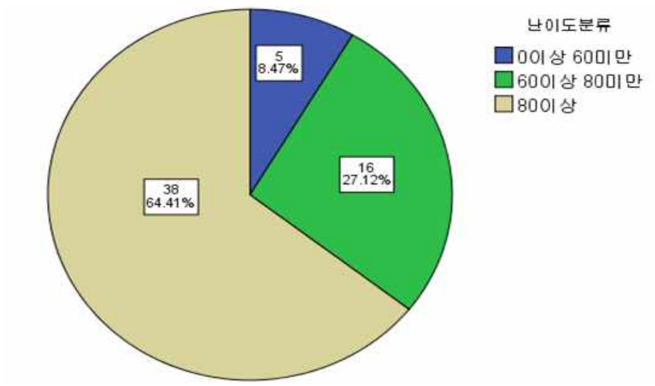

총점	난이도	표준편차	난이도	문항수	비율(%)
59	82.2	12.5	0~60미만	5	8.5
			60~80미만	16	27.1
			80~100	38	64.4
			전체	59	100.0

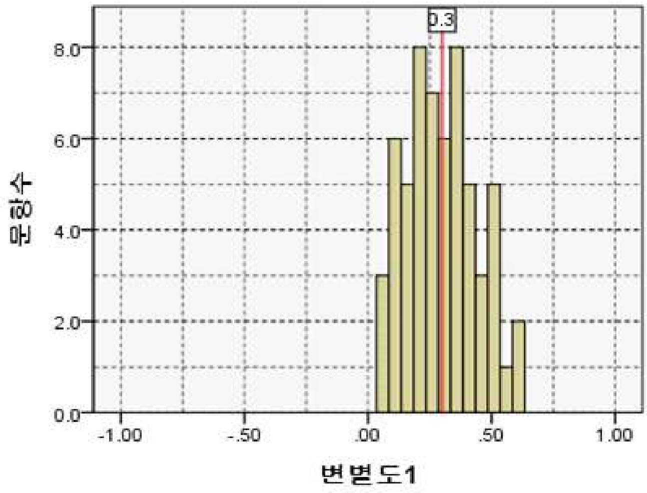

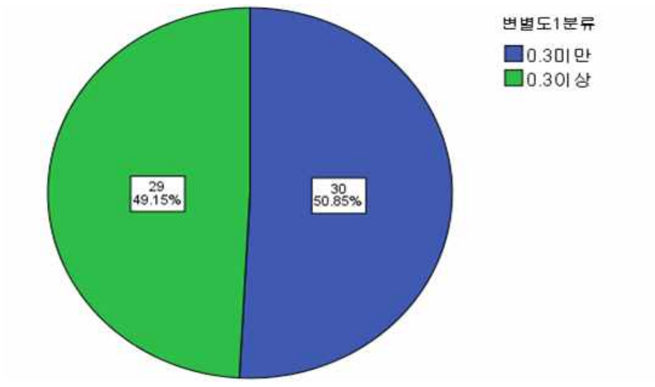

총점	변별도1	표준편차	변별도1	문항수	비율(%)
59	.30	.14	0.3미만	30	50.8
			0.3이상	29	49.2
			전체	59	100.0

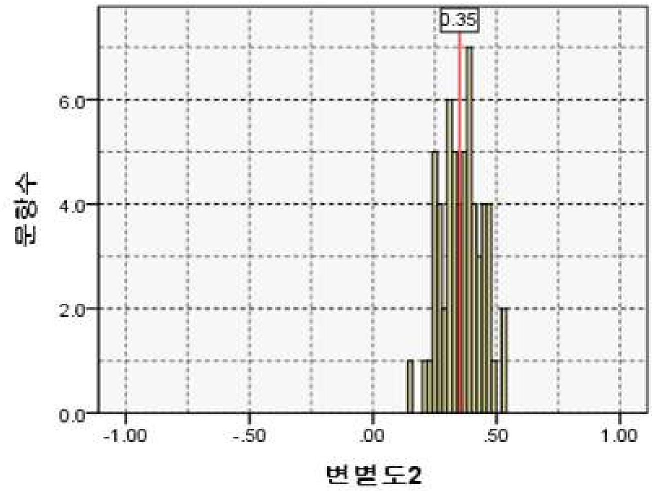

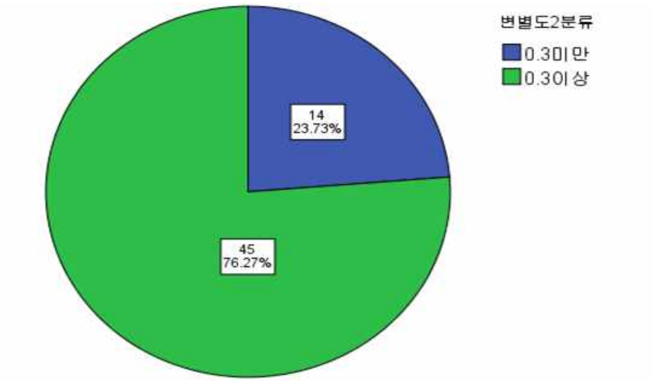

총점	변별도2	표준편차
59	.35	.08

변별도2	문항수	비율(%)
0.3미만	14	23.7
0.3이상	45	76.3
전체	59	100.0

해석

- 해석형 문항에서 난이도 지수가 80에서 100 사이인 문항이 전체 59문항 중 38문항으로 가장 많았으며, 다음으로 60이상 80미만인 문항이 16문항, 60미만인 문항이 5문항인 것으로 나타남
- 변별도1 지수를 기준으로 분류하였을 때, 0.3미만인 문항이 30문항으로 0.3이상인 문항이 29문항인 것에 비해 더 많게 나타남
- 변별도2 지수를 기준으로 분류하였을 때, 0.3미만인 문항이 14문항으로 0.3이상인 문항이 45문항인 것에 비해 더 적게 나타남

(3) 해결형 난이도와 변별도 분포도 및 비율분석

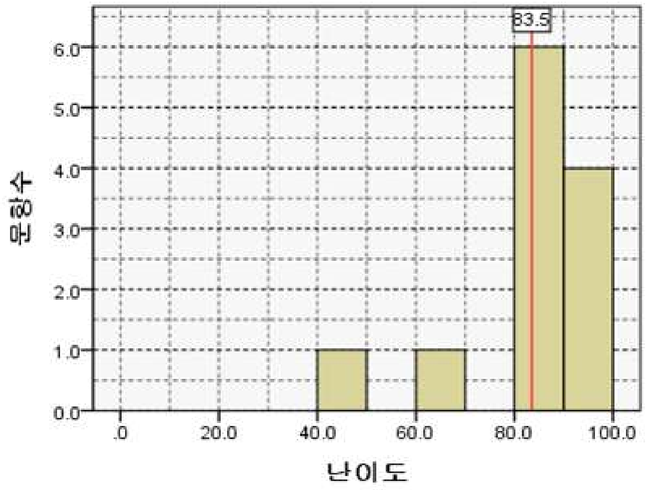

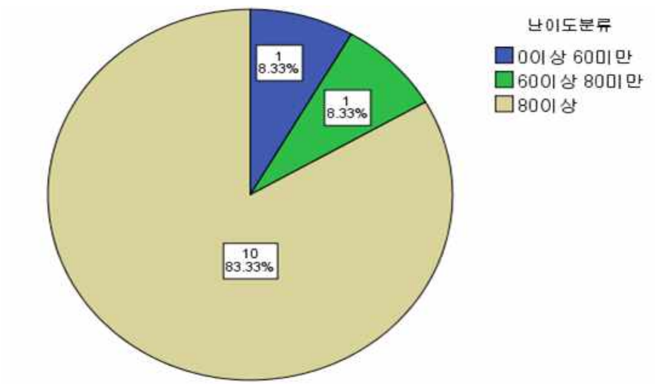

총점	난이도	표준편차
12	83.5	15.3

난이도	문항수	비율(%)
0~60미만	1	8.3
60~80미만	1	8.3
80~100	10	83.3
전체	12	100.0

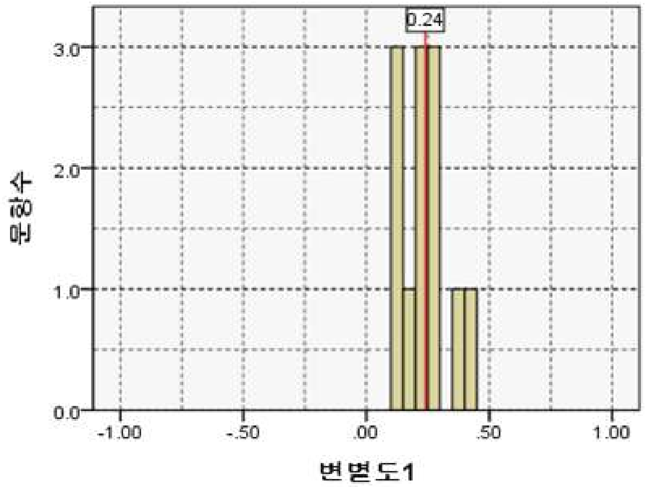

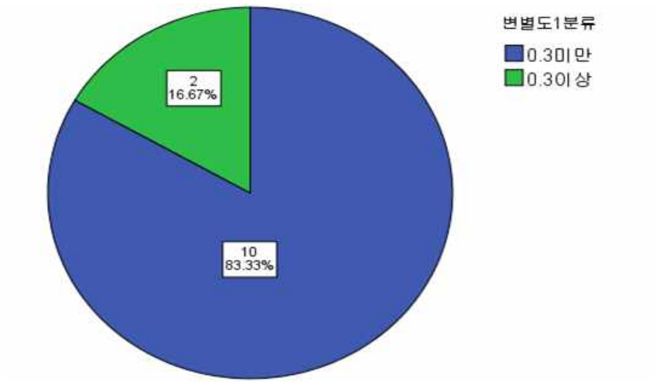

총점	변별도1	표준편차
12	.24	.10

변별도1	문항수	비율(%)
0.3미만	10	83.3
0.3이상	2	16.7
전체	12	100.0

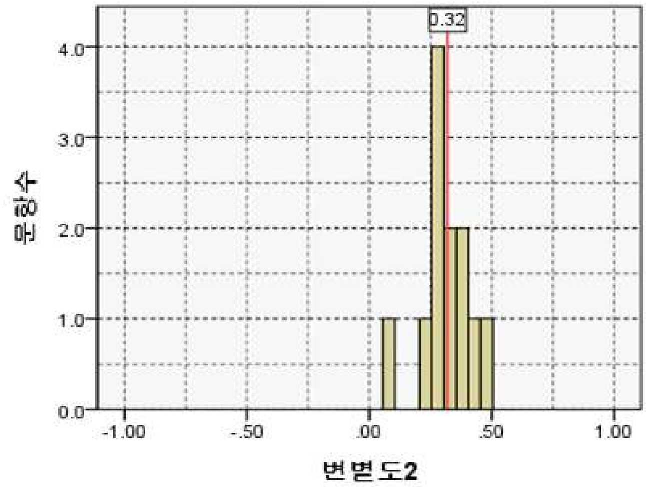

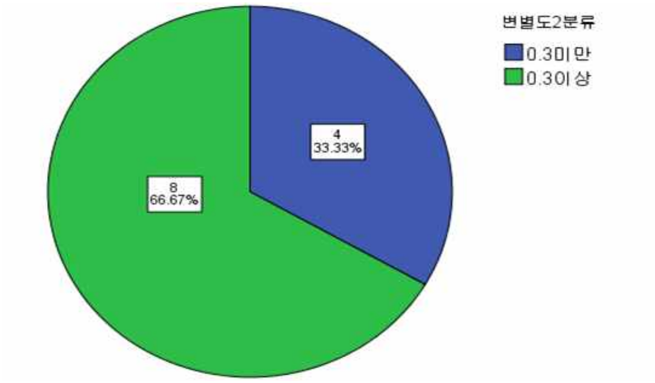

총점	변별도2	표준편차
12	.32	.10

변별도2	문항수	비율(%)
0.3미만	4	33.3
0.3이상	8	66.7
전체	12	100.0

해석

- 해결형 문항에서 난이도 지수가 80에서 100 사이인 문항이 전체 12문항 중 10문항으로 가장 많았으며, 다음으로 60이상 80미만인 문항이 1문항, 60미만인 문항이 1문항인 것으로 나타남
- 변별도1 지수를 기준으로 분류하였을 때, 0.3미만인 문항이 10문항으로 0.3이상인 문항이 2문항인 것에 비해 더 많게 나타남
- 변별도2 지수를 기준으로 분류하였을 때, 0.3미만인 문항이 4문항으로 0.3이상인 문항이 8문항인 것에 비해 더 적게 나타남

4) 자료유형별 난이도와 변별도

가) 전회 대비 자료유형별 난이도와 변별도

(1) 전회 대비 텍스트형 난이도와 변별도

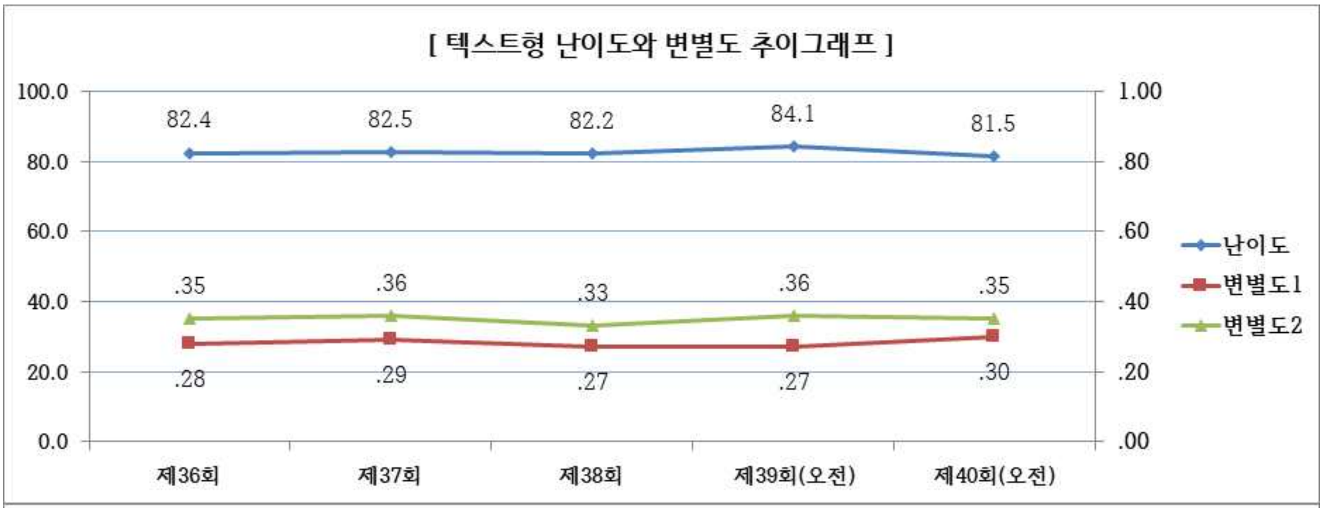

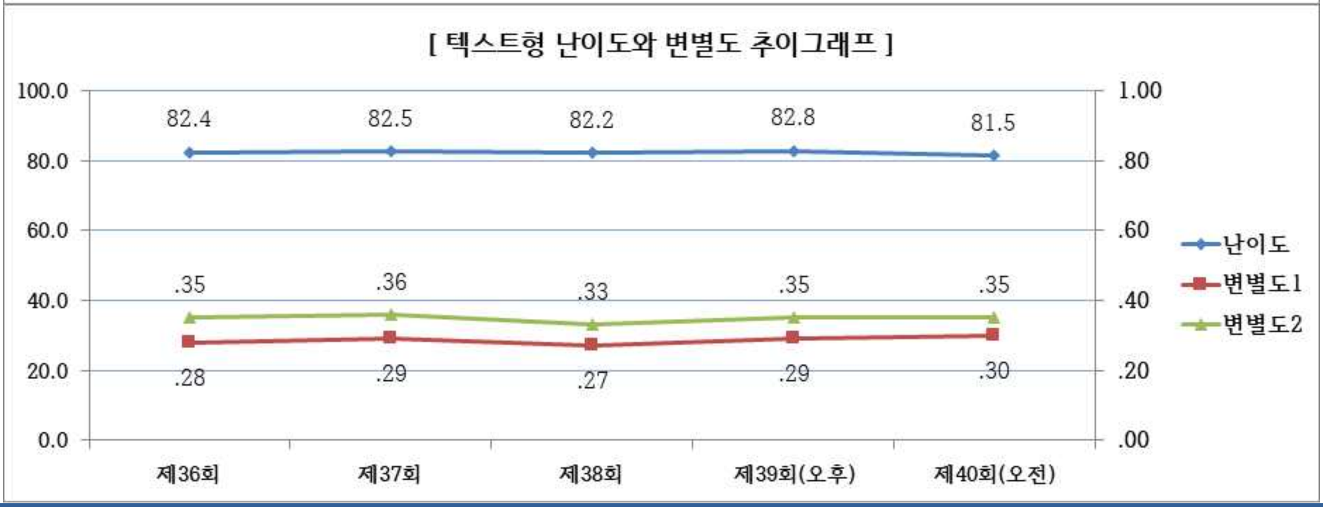

회차	난이도		변별도1		변별도2	
	평균	표준편차	평균	표준편차	평균	표준편차
제36회	82.4	12.8	.28	.13	.35	.11
제37회	82.5	13.2	.29	.15	.36	.10
제38회	82.2	14.4	.27	.13	.33	.08
제39회 (오전)	84.1	13.2	.27	.14	.36	.08
제39회 (오후)	82.8	12.8	.29	.14	.35	.10
**제40회 (오전)**	81.5	14.3	.30	.14	.35	.09

해석

- 전회(오전) 대비 텍스트형 문항의 난이도 지수는 2.6 감소함
- 전회(오전) 대비 텍스트형 문항의 변별도1 지수는 0.03 증가함
- 전회(오전) 대비 텍스트형 문항의 변별도2 지수는 0.01 감소함
- 전회(오후) 대비 텍스트형 문항의 난이도 지수는 1.3 감소함
- 전회(오후) 대비 텍스트형 문항의 변별도1 지수는 0.01 증가함
- 전회(오후) 대비 텍스트형 문항의 변별도2 지수는 동일함

(2) 전회 대비 자료제시형 난이도와 변별도

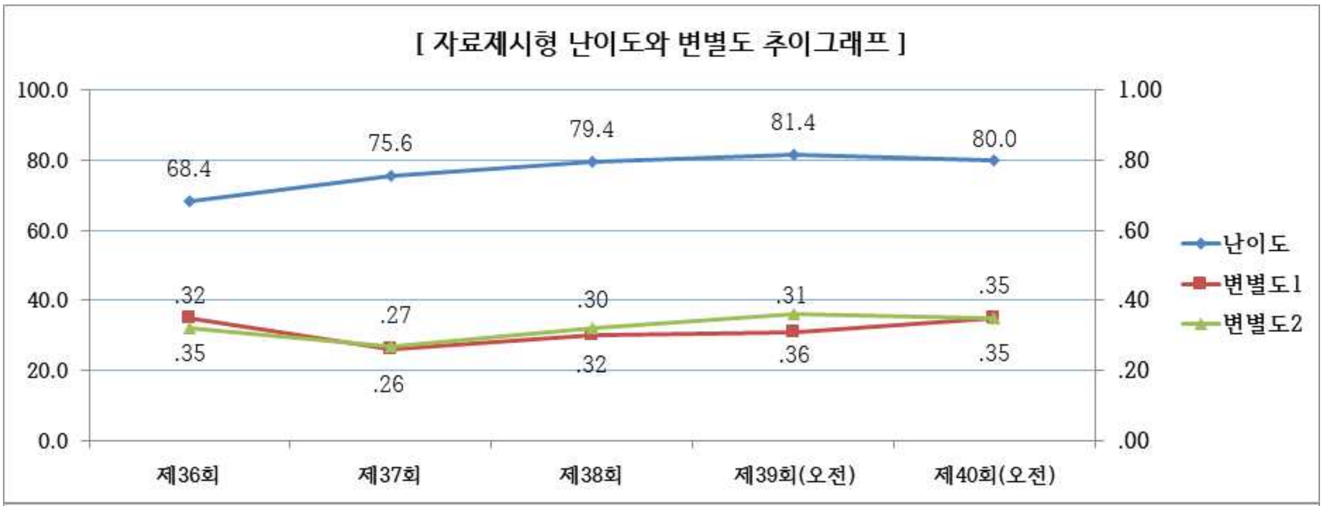

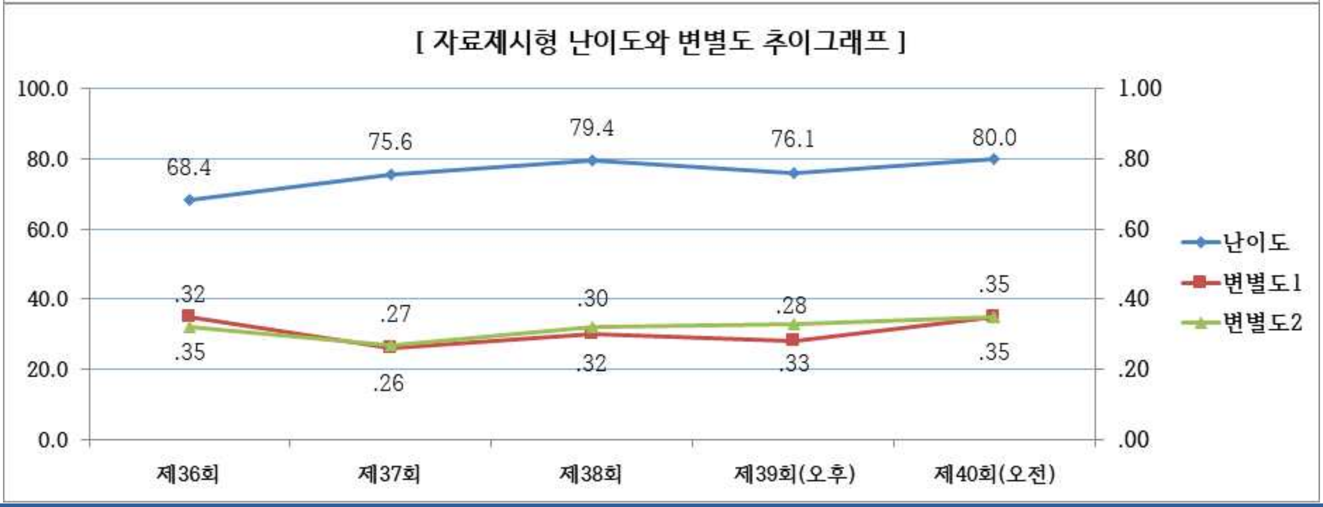

회차	난이도		변별도1		변별도2	
	평균	표준편차	평균	표준편차	평균	표준편차
제36회	68.4	17.1	.35	.10	.32	.13
제37회	75.6	23.3	.26	.13	.27	.11
제38회	79.4	15.4	.30	.12	.32	.06
제39회 (오전)	81.4	11.9	.31	.12	.36	.05
제39회 (오후)	76.1	25.2	.28	.15	.33	.09
**제40회 (오전)**	80.0	10.1	.35	.10	.35	.06

해석

- 전회(오전) 대비 자료제시형 문항의 난이도 지수는 1.4 감소함
- 전회(오전) 대비 자료제시형 문항의 변별도 1 지수는 0.04 증가함
- 전회(오전) 대비 자료제시형 문항의 변별도 2 지수는 0.01 감소함
- 전회(오후) 대비 자료제시형 문항의 난이도 지수는 3.9 증가함
- 전회(오후) 대비 자료제시형 문항의 변별도 1 지수는 0.07 증가함
- 전회(오후) 대비 자료제시형 문항의 변별도 2 지수는 0.02 증가함

나) 자료유형별 난이도와 변별도 분포도 및 비율분석

(1) 텍스트형 난이도와 변별도 분포도 및 비율분석

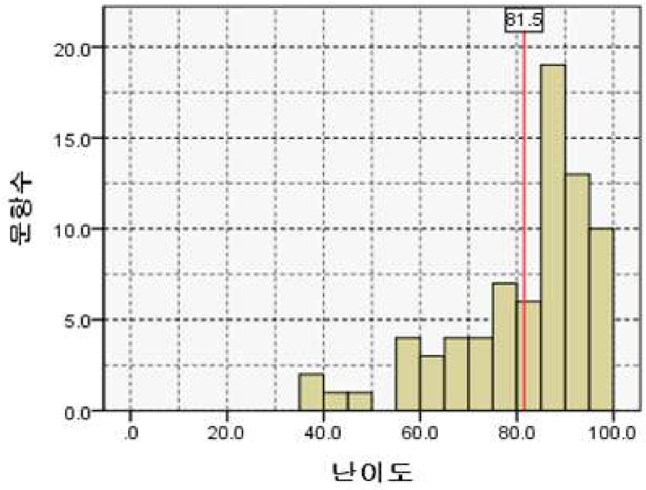

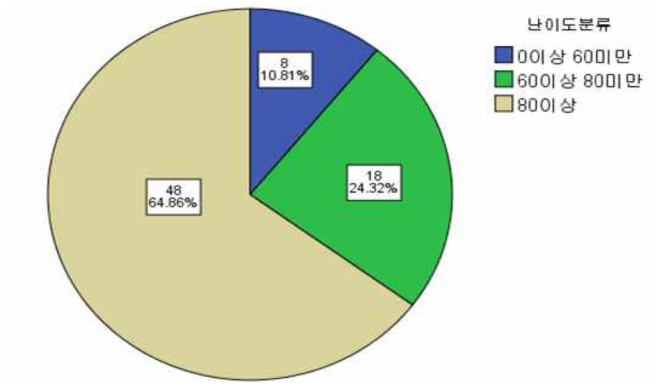

총점	난이도	표준편차
74	81.5	14.3

난이도	문항수	비율(%)
0~60미만	8	10.8
60~80미만	18	24.3
80~100	48	64.9
전체	74	100.0

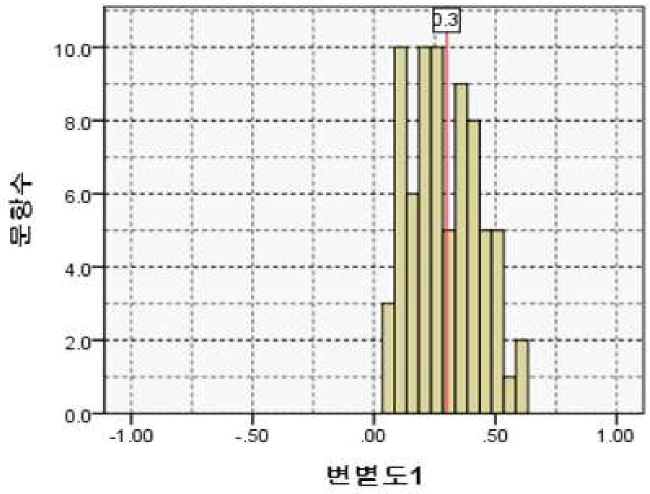

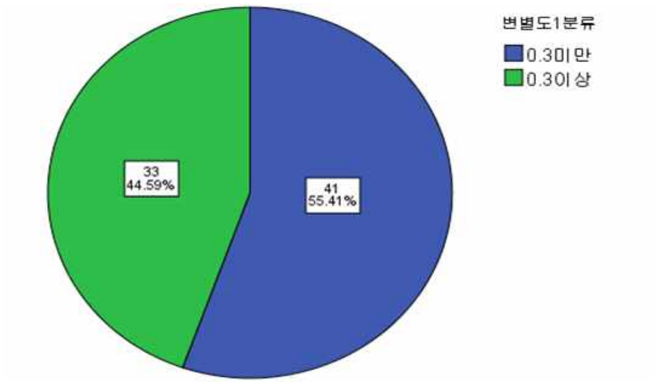

총점	변별도1	표준편차
74	.30	.14

변별도1	문항수	비율(%)
0.3미만	41	55.4
0.3이상	33	44.6
전체	74	100.0

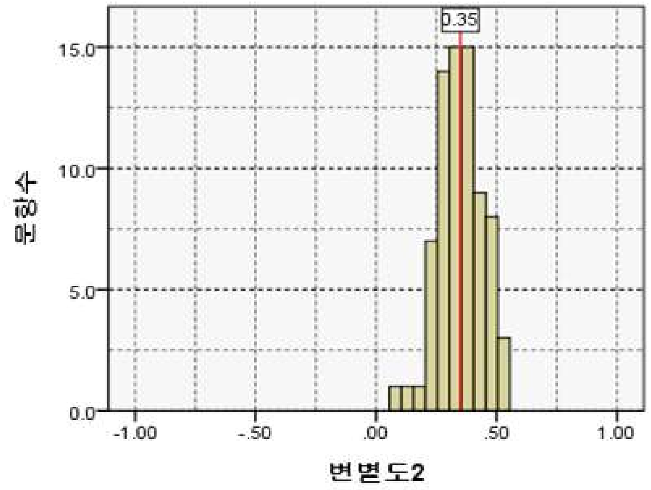

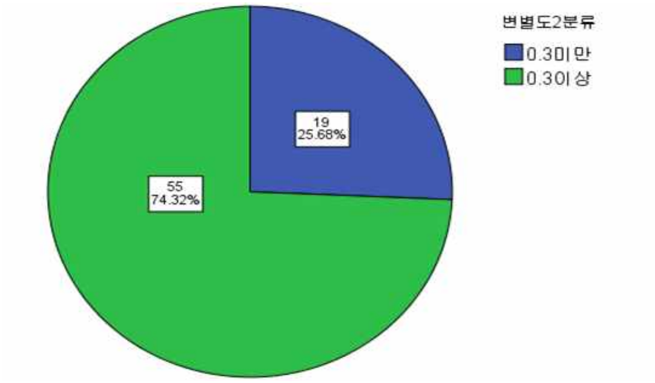

총점	변별도2	표준편차
74	.35	.09

변별도2	문항수	비율(%)
0.3미만	19	25.7
0.3이상	55	74.3
전체	74	100.0

해석

- 텍스트형 문항에서 난이도 지수가 80 에서 100 사이인 문항이 전체 74 문항 중 48 문항으로 가장 많았으며, 다음으로 60 이상 80 미만인 문항이 18 문항, 60 미만인 문항이 8 문항인 것으로 나타남
- 변별도 1 지수를 기준으로 분류하였을 때, 0.3 미만인 문항이 41 문항으로 0.3 이상인 문항이 33 문항인 것에 비해 더 많게 나타남
- 변별도 2 지수를 기준으로 분류하였을 때, 0.3 미만인 문항이 19 문항으로 0.3 이상인 문항이 55 문항인 것에 비해 더 적게 나타남

(2) 자료제시형 난이도와 변별도 분포도 및 비율분석

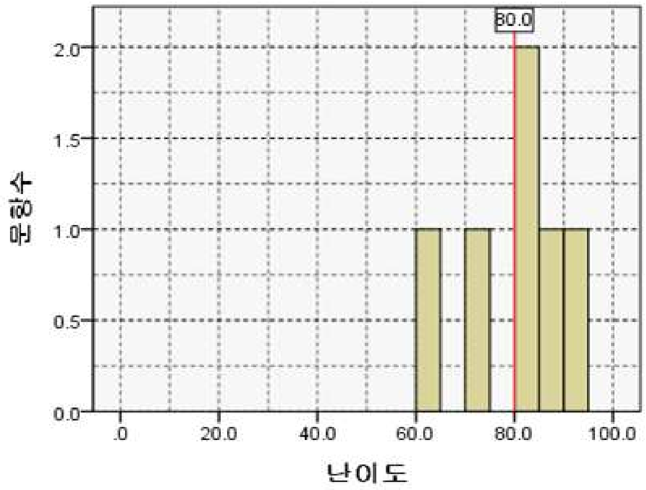

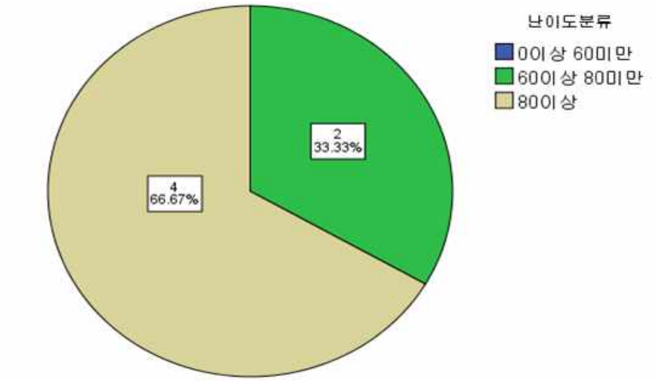

총점	난이도	표준편차	난이도	문항수	비율(%)
6	80.0	10.1	0~60미만	0	0.0
			60~80미만	2	33.3
			80~100	4	66.7
			전체	6	100.0

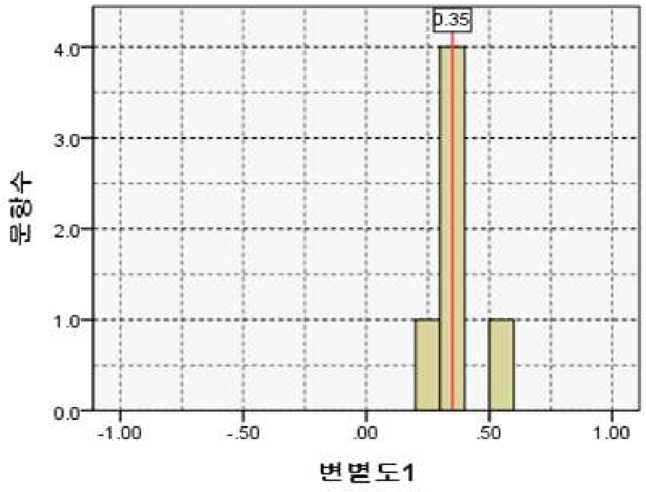

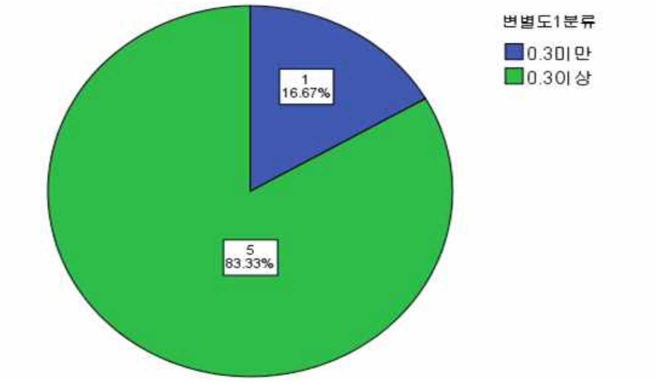

총점	변별도1	표준편차	변별도1	문항수	비율(%)
6	.35	.10	0.3미만	1	16.7
			0.3이상	5	83.3
			전체	6	100.0

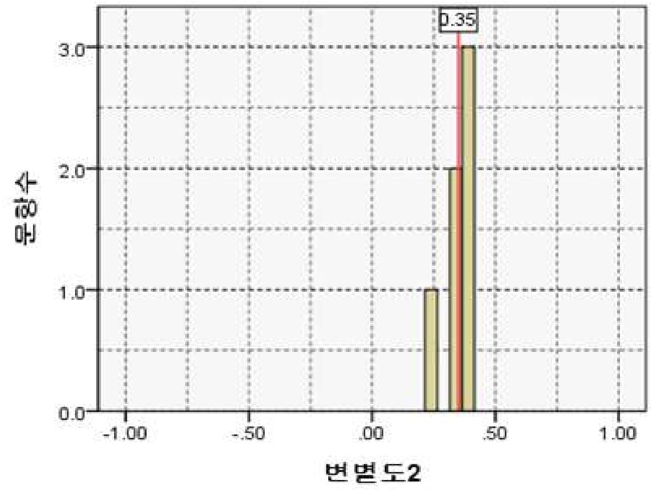

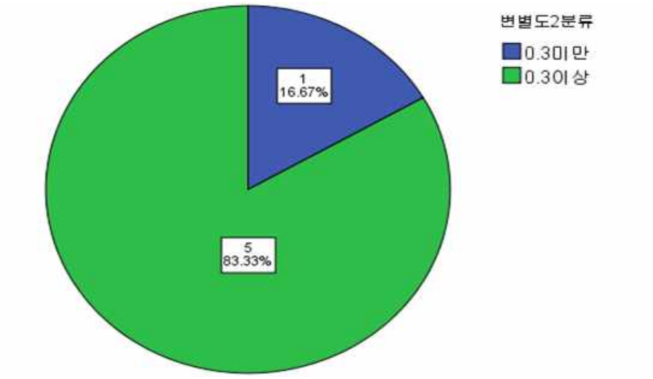

총점	변별도2	표준편차
6	.35	.06

변별도2	문항수	비율(%)
0.3미만	1	16.7
0.3이상	5	83.3
전체	6	100.0

해석

- 자료제시형 문항에서 난이도 지수가 80 에서 100 사이인 문항이 전체 6 문항 중 4 문항으로 가장 많았으며, 다음으로 60 이상 80 미만인 문항이 2 문항, 60 미만인 문항이 0 문항인 것으로 나타남
- 변별도 1 지수를 기준으로 분류하였을 때, 0.3 미만인 문항이 1 문항으로 0.3 이상인 문항이 5 문항인 것에 비해 더 적게 나타남
- 변별도 2 지수를 기준으로 분류하였을 때, 0.3 미만인 문항이 1 문항으로 0.3 이상인 문항이 5 문항인 것에 비해 더 적게 나타남

3. 난이도와 변별도 간 산포도

1) 전체 난이도와 변별도 간 산포도

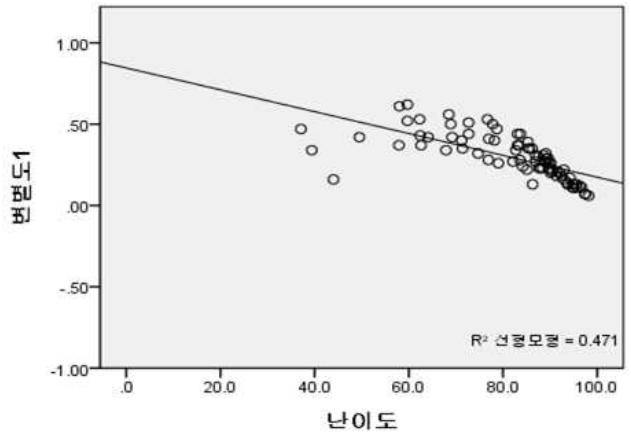

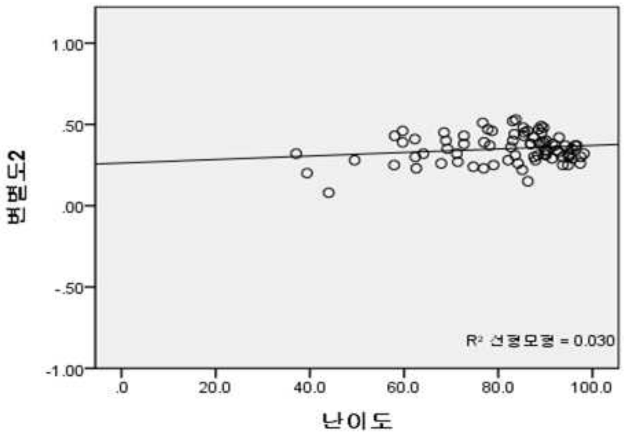

해석

- 전체 문항을 대상으로 난이도와 변별도 1 지수 간 상관은 -.686*으로 문항 난이도가 쉬울수록 변별력이 낮아지는 것으로 나타남
- 난이도와 변별도 2 지수 간 상관은 .173 으로 문항 난이도와 변별력 간 관련성이 없는 것으로 나타남

2) 과목별 난이도와 변별도 간 산포도

가) 요양보호론 난이도와 변별도 간 산포도

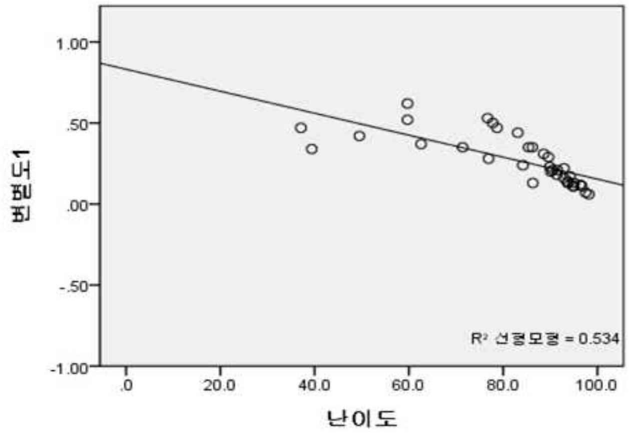

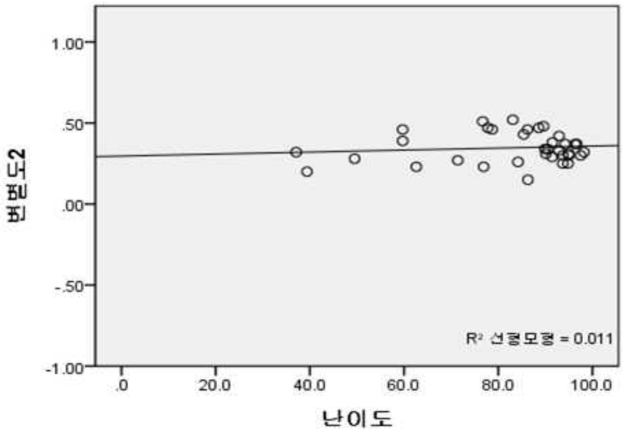

해석

- 요양보호론 과목 문항을 대상으로 난이도와 변별도 1 지수 간 상관은 -.731* 로 문항 난이도가 쉬울수록 변별력이 낮아지는 것으로 나타남
- 난이도와 변별도 2 지수 간 상관은 .107 로 문항 난이도와 변별력 간 관련성이 없는 것으로 나타남

나) 실기시험 난이도와 변별도 간 산포도

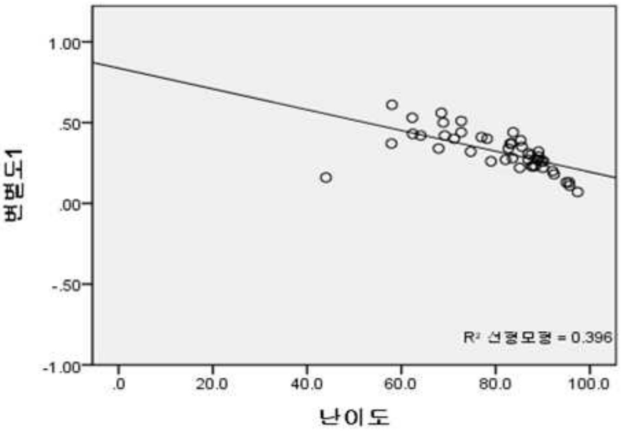

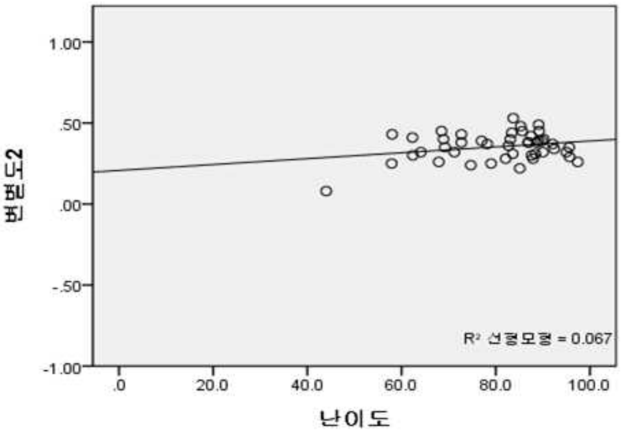

해석

- 실기시험 과목 문항을 대상으로 난이도와 변별도 1 지수 간 상관은 -.629*로 문항 난이도가 쉬울수록 변별력이 낮아지는 것으로 나타남
- 난이도와 변별도 2 지수 간 상관은 .260 으로 문항 난이도와 변별력 간 관련성이 없는 것으로 나타남

4. 신뢰도 분석

과목명	문항수	제36회	제37회	제38회	제39회(오전)	제39회(오후)	제40회(오전)
전체	80	.912	.919	.905	.920	.919	**.919**
요양보호론	35	.836	.838	.801	.838	.834	**.833**
실기시험	45	.846	.863	.851	.869	.867	**.872**

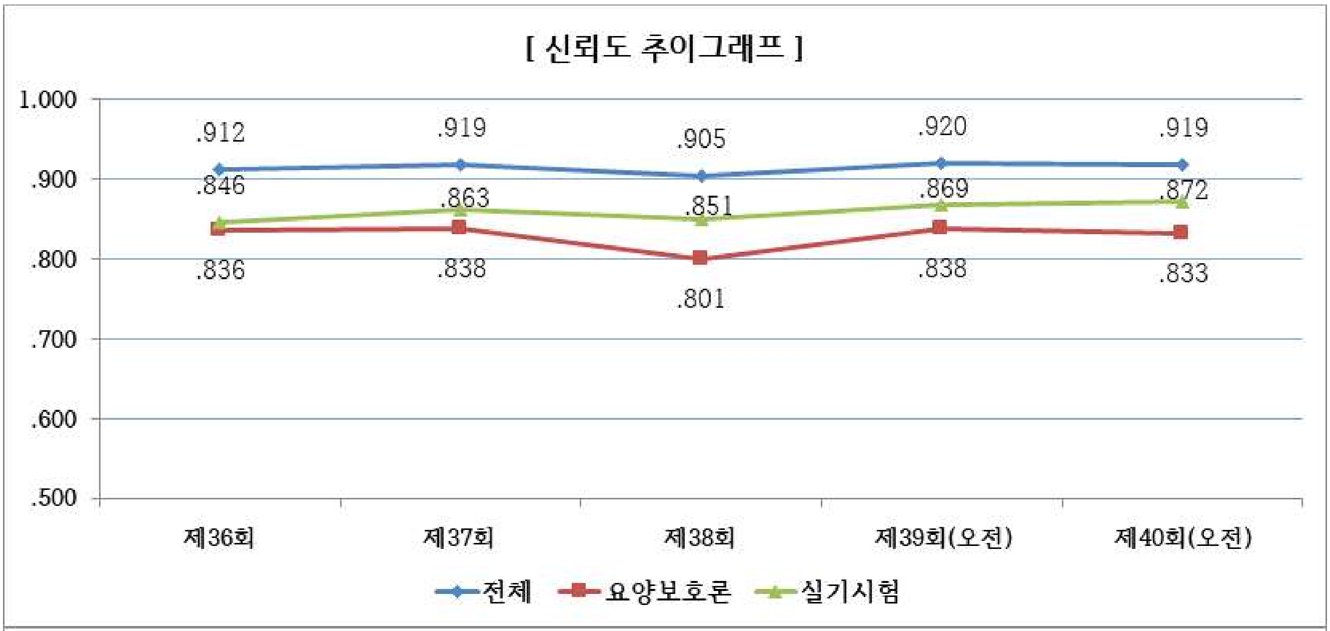

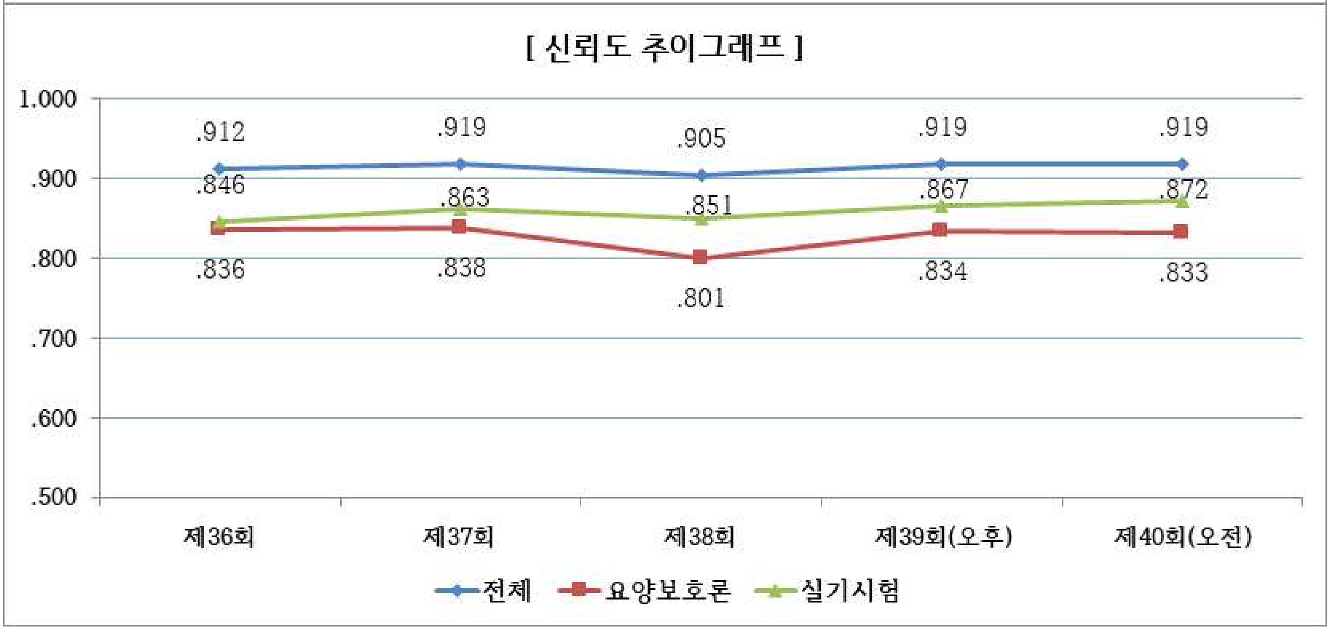

해석

- 요양보호사 자격시험 전체, 요양보호론 과목, 실기시험 과목의 문항 신뢰도가 각각 .919, .833, .872 로 모두 일관되게 해당 영역을 측정하고 있는 것으로 나타남
- 전회(오전) 대비 신뢰도는 자격시험 전체, 요양보호론 과목은 각각 .001, .005 감소하였으며, 실기시험 과목은 .003 증가함
- 전회(오후) 대비 신뢰도는 자격시험 전체는 동일하고, 요양보호론 과목은 .001 감소하였으며, 실기시험 과목 .005 증가함

○ 분석결과 관련 문의 : 한국보건의료인국가시험원 연구개발본부 정보경 전임연구원
Tel : 02-2087-8957, FAX : 02-2087-8885
E-mail : luckys1004@kuksiwon.or.kr
