# Supplementary material for: Item difficulty index, discrimination index, and reliability of the 26 health professions licensing examinations in 2022, Korea: a psychometric study
Source: J Educ Eval Health Prof. 2023 Nov 22;20:31. doi: 10.3352/jeehp.2023.20.31 (PMC11959405; doi:10.3352/jeehp.2023.20.31)
Supplement: Supplementary file 1 — Supplement 1. Item analysis results of 26 health professions licensing examinations administered during late 2022 and early 2023. [file jeehp-20-31_Suppl1.zip › 2022│Γ╡╡ ┴a44╚╕ └o╗2╗τ ▒╣░í╜├╟Φ ║╨╝«░ß░·.pdf]

2022년도 제44회
위생사 국가시험
문항분석 결과

국민이 신뢰하고 감동하는 시험평가기관
한국보건의료인국가시험원
KOREA HEALTH PERSONNEL LICENSING EXAMINATION INSTITUTE

일반 용어 정의

□ **평균**

- 집단에서의 대표적 경향값으로 전체 값을 더하여 총 응시자로 나눈 값

□ **표준편차**

- 평균과 각 점수의 차이인 편차들의 평균으로 점수가 흩어져 분포되어 있는 정도

□ **추정난이도**

- 문항개발자가 예측한 정답률

□ **검사이론**

- 검사와 검사를 구성하고 있는 문항의 양호도를 분석 및 평가하는 방법을 정의한 이론체계
- 대표적으로 고전검사이론과 문항반응이론이 있음

고전검사이론 용어 정의

□ **고전검사이론(Classical Test Theory; CTT)**

- 검사의 질을 분석하는 검사이론 중 한 가지로 19세기 말부터 전개되어 현재까지 주로 사용되고 있는 검사이론임
- 고전검사이론에 의한 문항과 응시자 능력 추정치는 다음과 같음

○ **문항난이도**

· 검사 문항의 쉽고 어려운 정도를 나타내는 지수
· 난이도 지수는 총 반응 수에 대한 정답 반응 수의 비율로 문항의 정답률임
· 문항난이도는 0~100까지의 값을 가짐
· 난이도 값이 큰 경우, 쉬운 문항으로 '난이도가 낮다'라고 해석하며, 난이도 값이 작은 경우, 어려운 문항으로 '난이도가 높다'라고 해석함

○ **문항변별도**

· 각 문항이 응시자의 능력 수준을 변별할 수 있는 정도를 나타내는 지수
· 문항변별도는 -1~+1까지의 값을 가지며, 1에 가까울수록 변별력 크다고 해석함
· 일반적으로 문항변별도가 0.3 이상이면 우수한 문항으로 평가함
· 구하는 방식에는 '상하위집단 구분법', '문항-총점 상관계수' 등이 있음

1) **변별도 1(상하위구분법):** 상위 27%와 하위 27% 집단의 난이도 차이를 구하는 방식
2) **변별도 2(상관계수법):** 문항-총점과의 상관계수로 구하는 방식

○ **신뢰도**

· 시험이 평가하고자 하는 것을 일관성 있게 측정하는가로 시험이 오차없이 정확하게 측정한 정도를 의미함
· 국시원에서는 문항의 내적일관성(Cronbach α)으로 신뢰도를 추정하며 1에 가까울수록 신뢰도가 높다고 해석함

목 차

I. 시행 결과 ···· 6

1. 시험 현황 ···· 7
  1) 시험명 ···· 7
  2) 시험시행일 ···· 7
  3) 응시현황 ···· 7
  4) 과목별 문항 수, 배점 및 과락 점수 ···· 7
2. 합격률과 평균성적 ···· 7
  1) 합격 및 불합격 현황 ···· 7
  2) 과목별 과락자수 내역 ···· 8
  3) 전회 대비 합격률과 평균성적 ···· 8

II. 문항분석 결과 ···· 10

1. 성적 ···· 11
  1) 전체 성적분포도 ···· 11
  2) 과목별 성적분포도(※ 필기형 실기 포함) ···· 12
2. 난이도와 변별도 ···· 14
  1) 전체 난이도와 변별도 ···· 14
  2) 과목별 난이도와 변별도 ···· 17
  3) 지식수준별 난이도와 변별도 ···· 33
3. 난이도와 변별도 간 산포도 ···· 41
  1) 전체 난이도와 변별도 간 산포도 ···· 41
  2) 과목별 난이도와 변별도 간 산포도 ···· 41
4. 신뢰도 분석 ···· 45

I. 시행 결과

1. 시험 현황

1) 시험명: 2022년도 제44회 위생사 국가시험

2) 시험시행일: 2022년 11월 19일

3) 응시현황

응시대상자수	결시자수	부정행위자수	응시자 준수사항 위반자 수		응시자수 (%)
			휴대폰 소지	신분증 미지참	
9,260	1,033	0	0	0	8,221(88.8)

※ 8,221명은 응시대상자(9,260명)에서 결시자수(1,033명) 및 채점보류자수(6명)를 제외한 수치임

4) 과목별 문항 수, 배점 및 과락 점수

교 시	과 목 명	문제 수	배점	총점	합격자 점수기준	
					과목 과락기준	총점 합격기준
1교시	위생 관계 법령	25	1	25	10점 미만	108점 이상 (필기 총점 180점)
	환경위생학	50	1	50	20점 미만	
	위생곤충학	30	1	30	12점 미만	
2교시	공중보건학	35	1	35	14점 미만	
	식품위생학	40	1	40	16점 미만	
3교시	실기시험	40	1	40	24점 미만	24점 이상 (실기 총점 40점)
계		220	1	220		

2. 합격률과 평균성적

1) 합격 및 불합격 현황

합격자수 (%)	불합격자수(%)					채점보류자수
	평락	과락	실기탈락	기권	계	
5,019 (61.1)	2,853 (34.7)	62 (0.75)	275 (3.35)	12 (0.15)	3,202 (38.9)	6

2) 과목별 과락자수 내역

과목명 / 과락자수	공중보건학	환경위생학	위생 관계 법령	위생곤충학
과목별 과락자 수	1	-	13	47
2과목 과락자 수	위생 관계 법령, 위생곤충학: 1			
전과목 과락자 수	-			

3) 전회 대비 합격률과 평균성적

회차	년도	합격률(%)	평균성적	표준편차	백분율 환산점수
제40회	2018	33.5	125.4	32.0	57.0
제41회	2019	58.5	135.0	35.7	64.4
제42회	2020	44.2	122.8	36.4	55.8
제43회	2021	49.6	128.0	35.1	58.2
**제44회**	**2022**	**61.1**	**135.2**	**36.6**	**61.4**

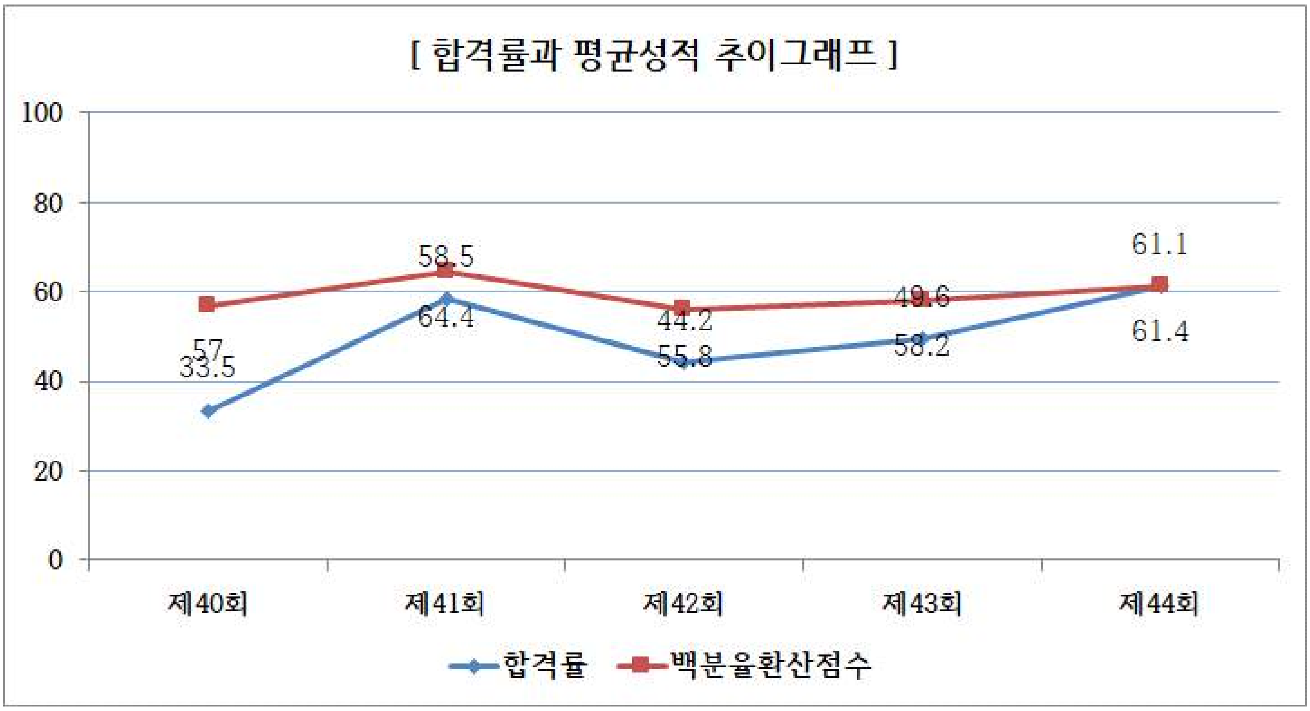

4) 전체 성적분포도

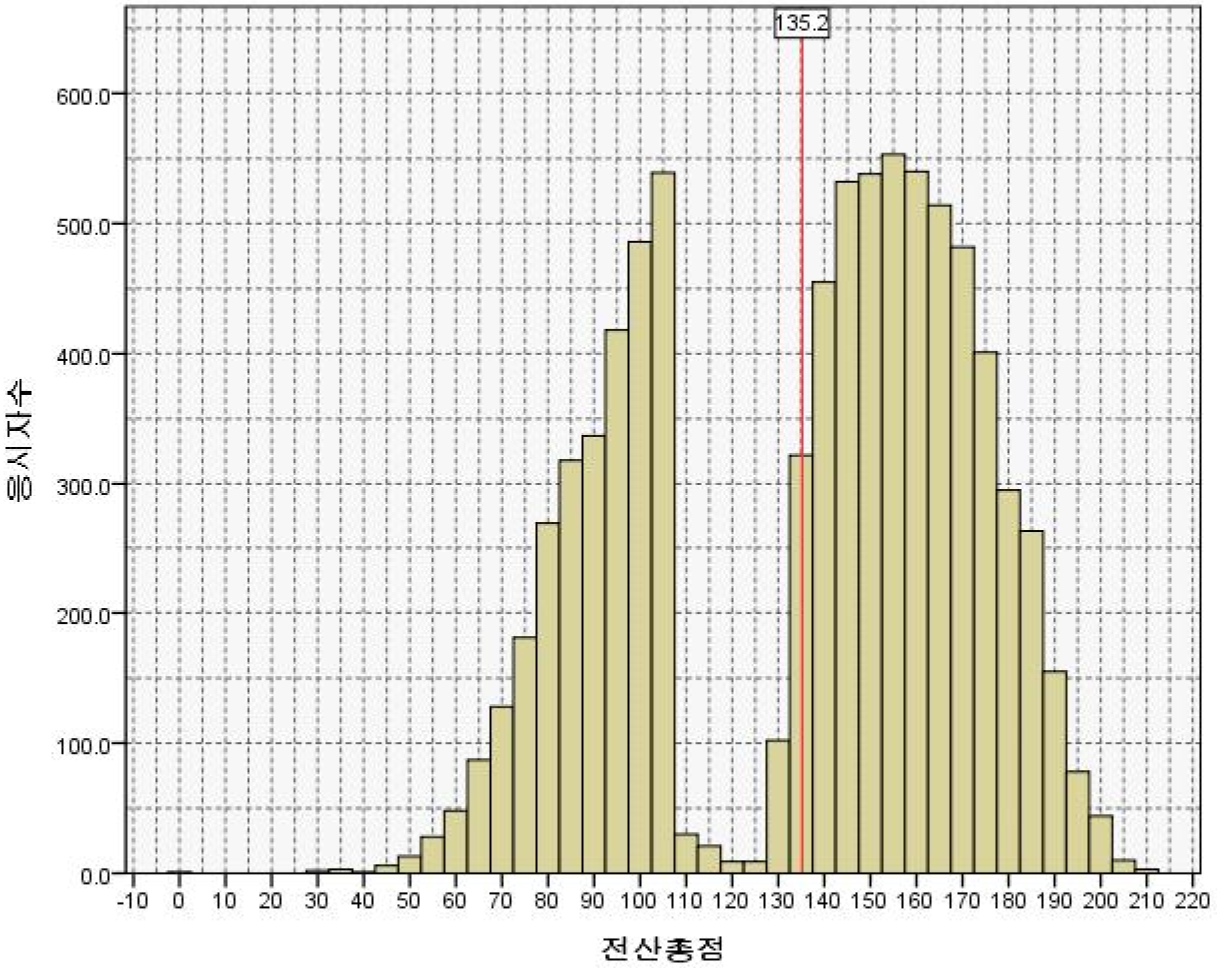

※ 필기시험 불합격자의 실기성적을 포함하지 않음

해석

- 전년 대비 합격률은 11.5% 증가, 백분율 환산 점수는 3.2 점 증가함

II. 문항분석 결과

1. 성적

1) 전체 성적분포도

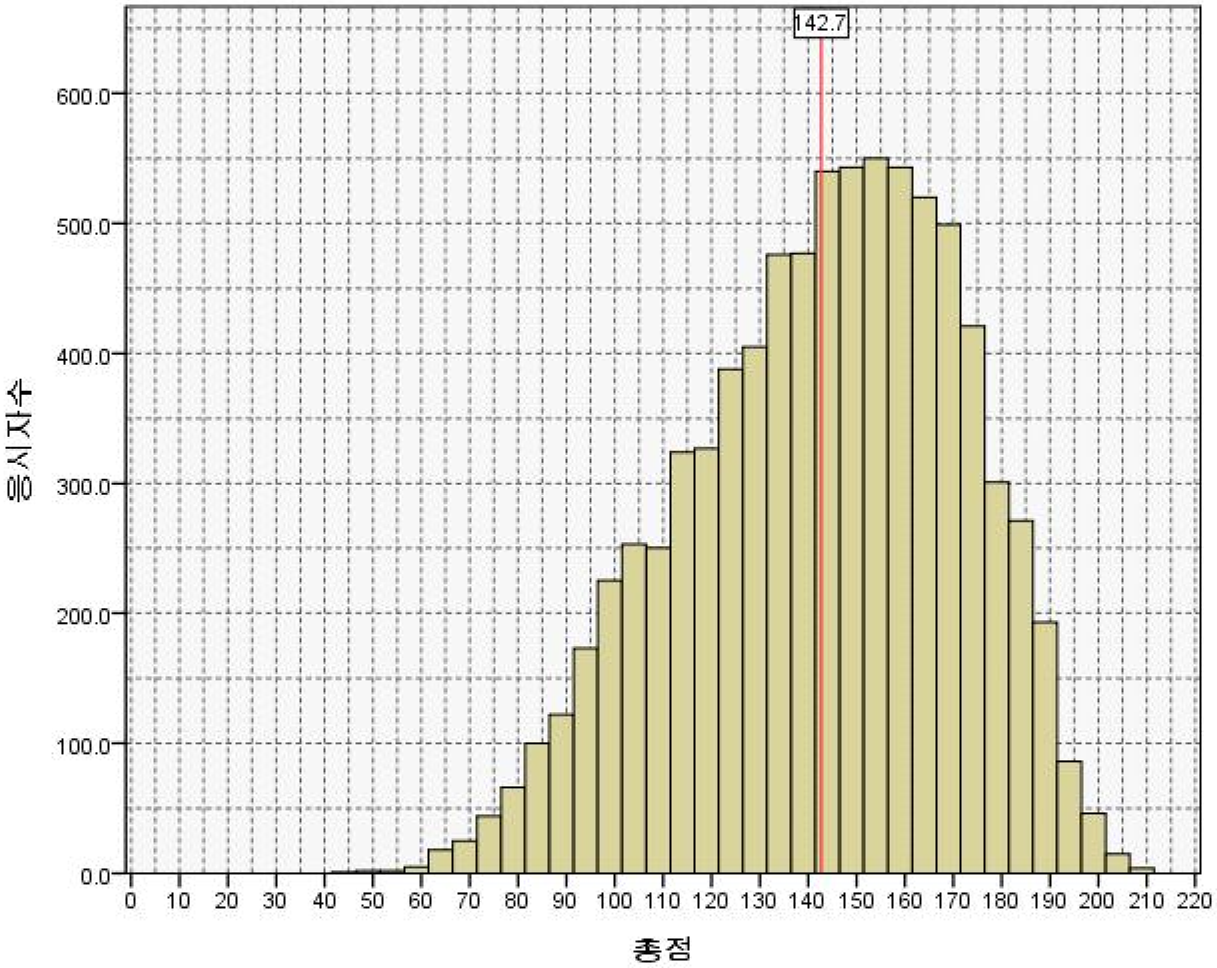

응시자	총점	합격선	평균성적	표준편차
8,215	220	132	142.7	28.4

※ 8,215명은 전체 응시자(8,227명)에서 기권자(12명)를 제외한 수치임

※ 필기시험 불합격자의 실기성적을 포함함

2) 과목별 성적분포도(※ 필기형 실기 포함)

가) 위생 관계 법령

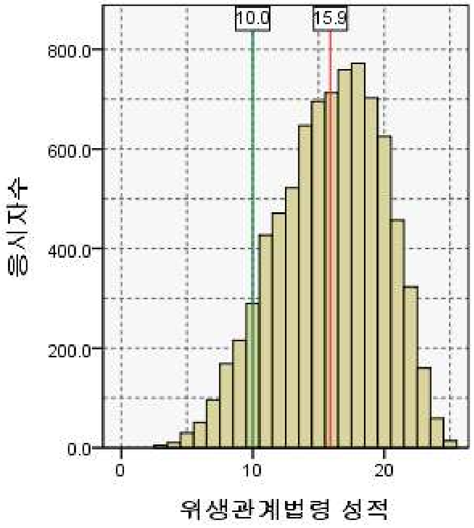

총점	과락선	평균성적	표준편차
25	10	15.9	4.0

나) 환경위생학

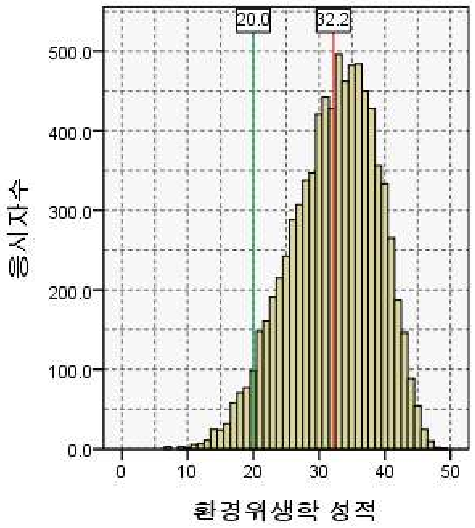

총점	과락선	평균성적	표준편차
50	20	32.2	6.6

다) 위생곤충학

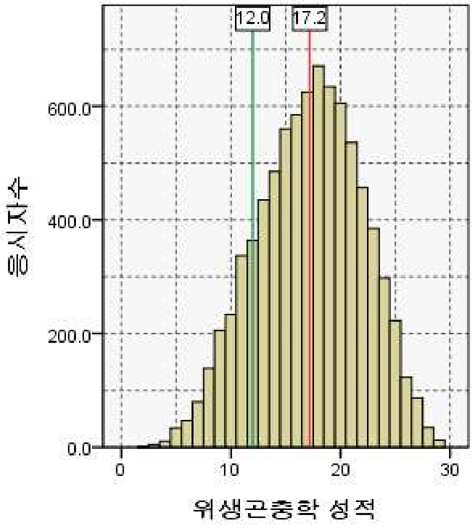

총점	과락선	평균성적	표준편차
30	12	17.2	4.8

라) 공중보건학

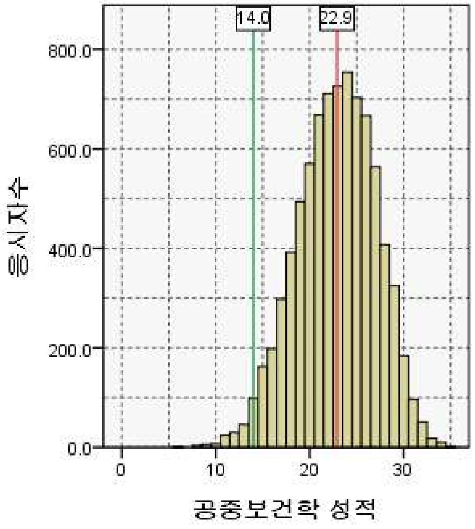

총점	과락선	평균성적	표준편차
35	14	22.9	4.2

마) 식품위생학

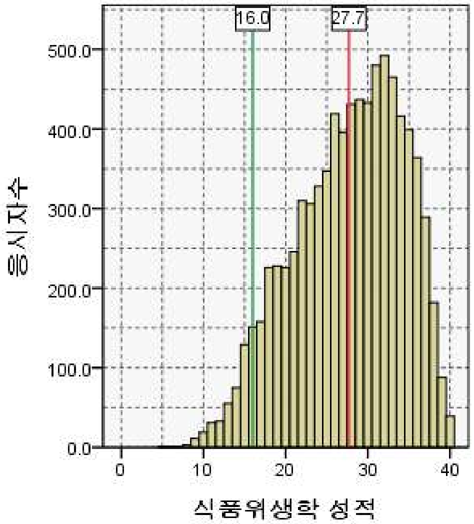

총점	합격선	평균성적	표준편차
40	16	27.7	6.6

바) 실기시험

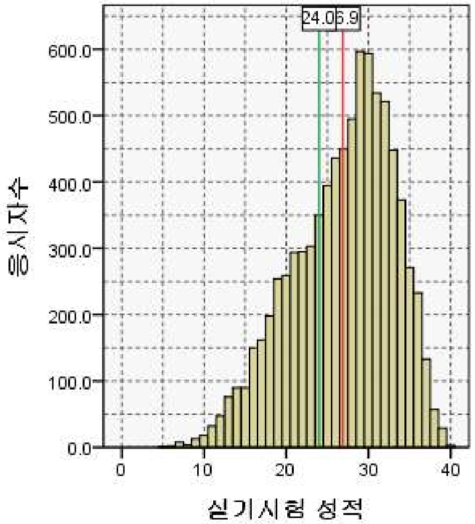

총점	합격선	평균성적	표준편차
40	24	26.9	6.1

2. 난이도와 변별도

1) 전체 난이도와 변별도

가) 전회 대비 전체 난이도와 변별도

회차	난이도		변별도1		변별도2	
	평균	표준편차	평균	표준편차	평균	표준편차
제40회	60.2	23.6	.28	.17	.25	.14
제41회	64.6	20.7	.31	.17	.28	.13
제42회	60.5	21.9	.33	.18	.29	.15
제43회	61.7	22.2	.31	.17	.28	.14
**제44회**	**64.9**	**20.6**	**.32**	**.17**	**.29**	**.13**

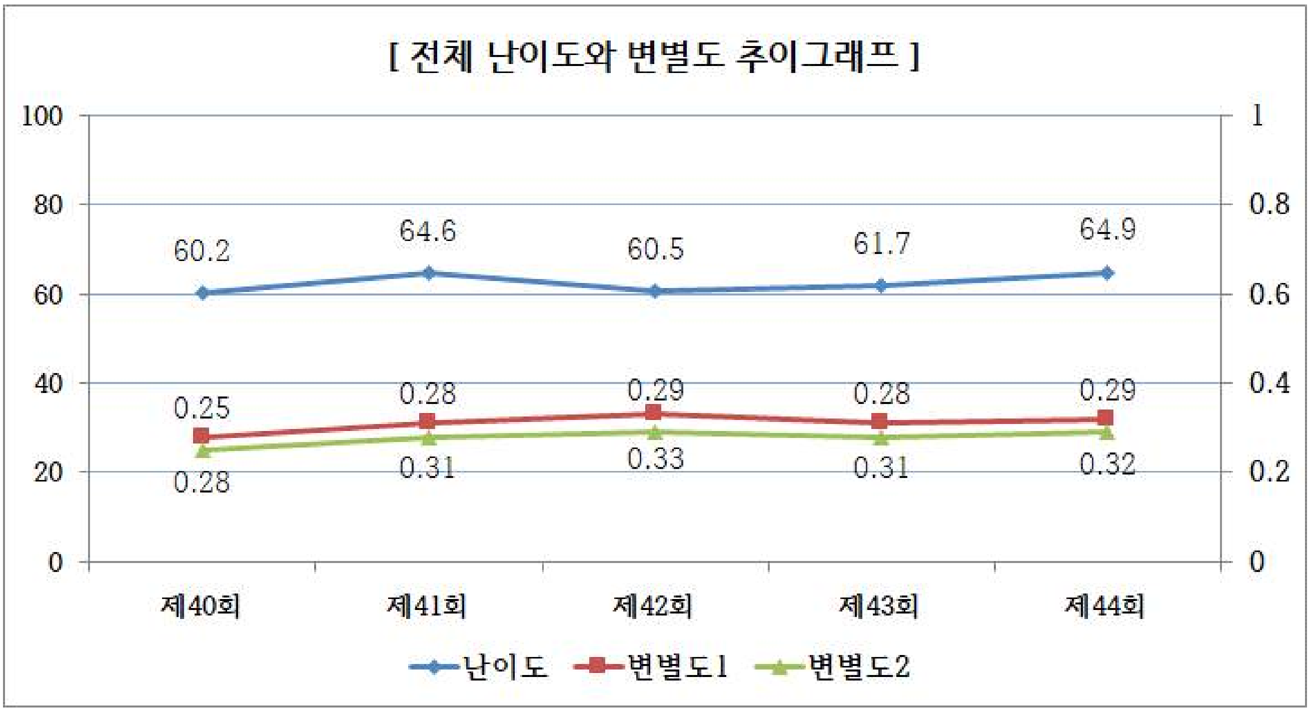

해석

- 전회대비 난이도 지수는 3.2 증가함
- 변별도 1 과 변별도 2 의 값은 각각 .01 씩 증가함

나) 전체 난이도와 변별도 분포도 및 비율분석

(1) 전체 난이도 분포도 및 비율분석

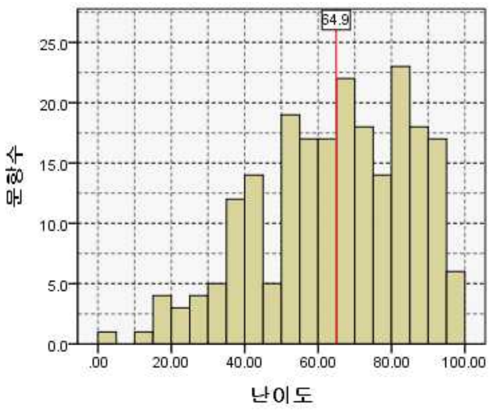

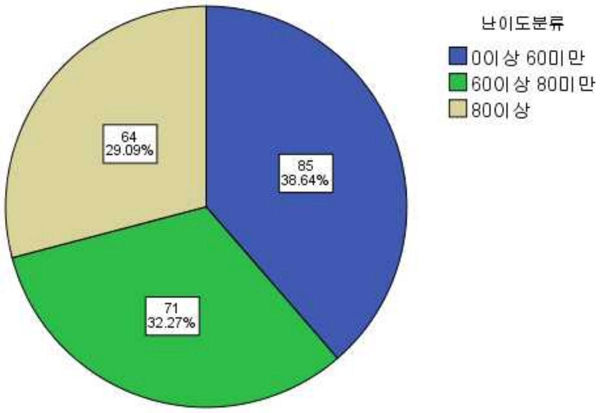

총점	난이도	표준편차
220	64.9	20.6

난이도	문항수	비율(%)
0~60미만	85	38.6
60~80미만	71	32.3
80~100	64	29.1
전체	220	100.0

(2) 전체 변별도1 분포도 및 비율분석

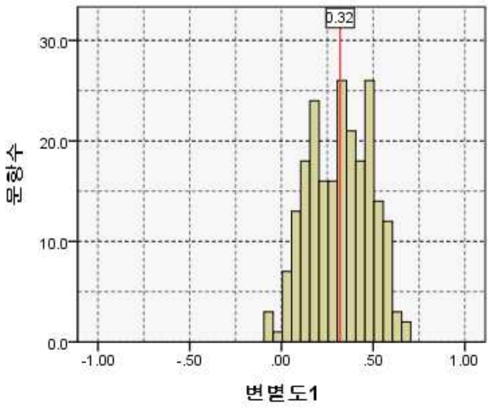

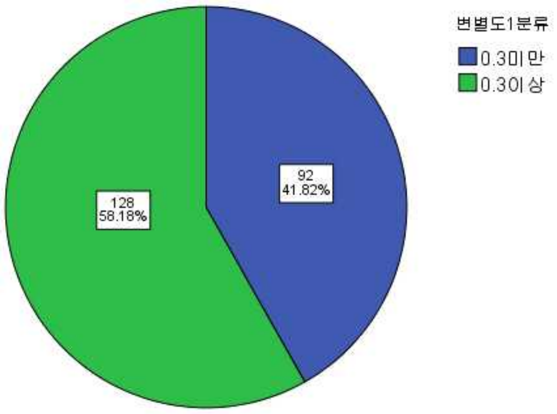

총점	변별도1	표준편차
220	.32	.17

변별도1	문항수	비율(%)
0.3미만	92	41.8
0.3이상	128	58.2
전체	220	100.0

(3) 전체 변별도2 분포도 및 비율분석

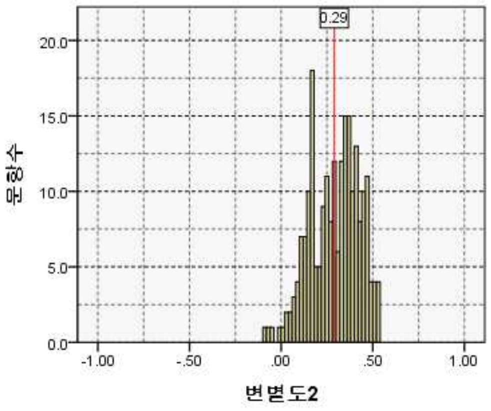

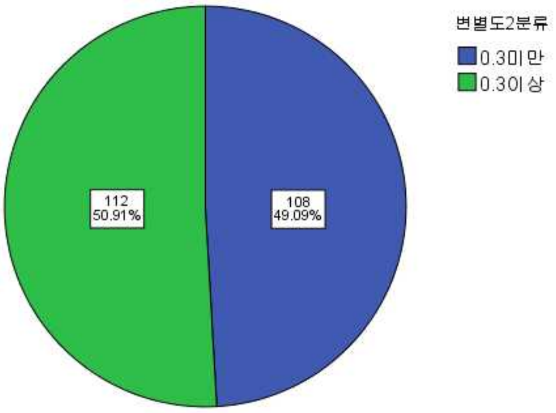

총점	변별도2	표준편차
220	.29	.13

변별도2	문항수	비율(%)
0.3미만	108	49.1
0.3이상	112	50.9
전체	220	100.0

해석

- 난이도 지수가 60 미만인 문항이 85 문항으로 가장 많았으며, 60 이상 80 미만인 문항이 71 문항, 80 이상인 문항이 64 문항인 것으로 나타남
- 변별도 1 지수를 기준으로 분류하였을 때, 0.3 미만인 문항이 92 문항으로 0.3 이상인 문항이 128 문항인 것에 비해 더 적게 나타남
- 변별도 2 지수를 기준으로 분류하였을 때, 0.3 미만인 문항이 108 문항으로 0.3 이상인 문항이 112 문항인 것에 비해 더 적게 나타남

2) 과목별 난이도와 변별도

가) 전회 대비 과목별 난이도와 변별도

(1) 전회 대비 위생관계법령 난이도와 변별도

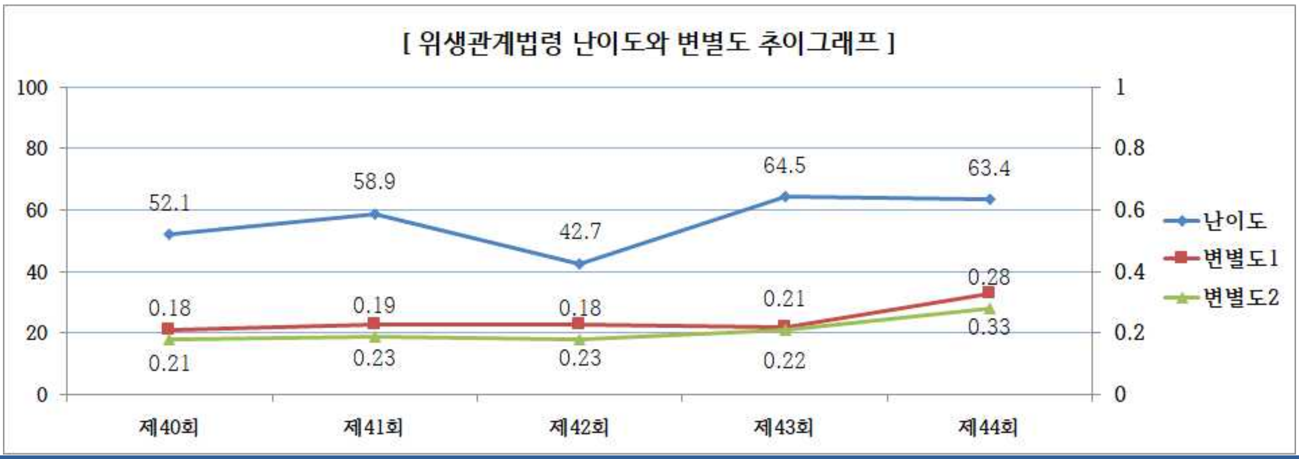

회차	난이도		변별도1		변별도2	
	평균	표준편차	평균	표준편차	평균	표준편차
제40회	52.1	25.4	.21	.16	.18	.14
제41회	58.9	18.0	.23	.17	.19	.14
제42회	42.7	21.1	.23	.18	.18	.15
제43회	64.5	25.9	.22	.13	.21	.10
**제44회**	**63.4**	**17.4**	**.33**	**.19**	**.28**	**.14**

(2) 전회 대비 환경위생학 난이도와 변별도

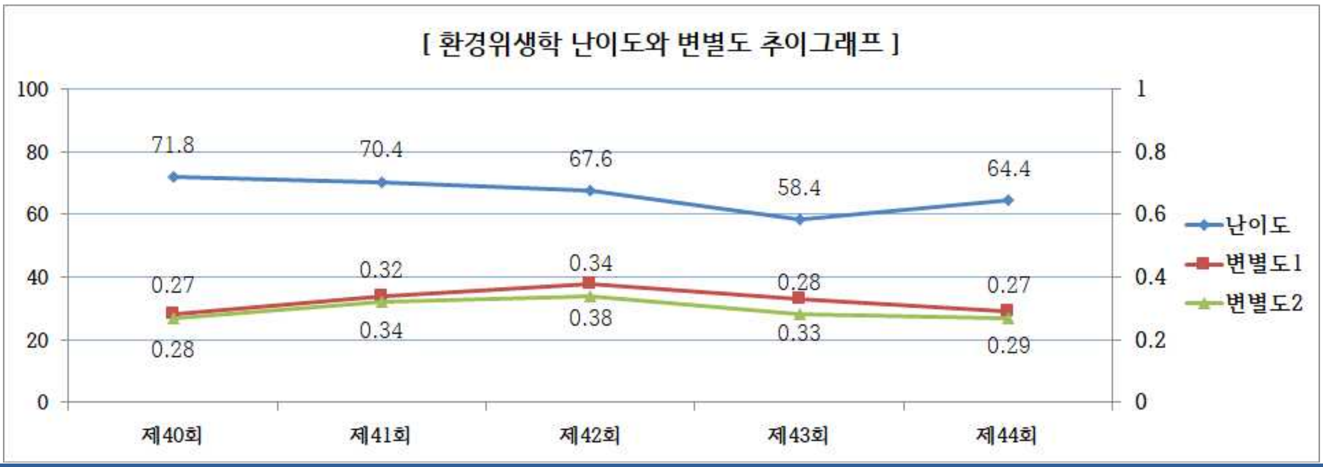

회차	난이도		변별도1		변별도2	
	평균	표준편차	평균	표준편차	평균	표준편차
제40회	71.8	17.9	.28	.15	.27	.12
제41회	70.4	19.1	.34	.15	.32	.11
제42회	67.6	18.3	.38	.19	.34	.14
제43회	58.4	20.4	.33	.15	.28	.12
**제44회**	**64.4**	**22.6**	**.29**	**.16**	**.27**	**.14**

(3) 전회 대비 위생곤충학 난이도와 변별도

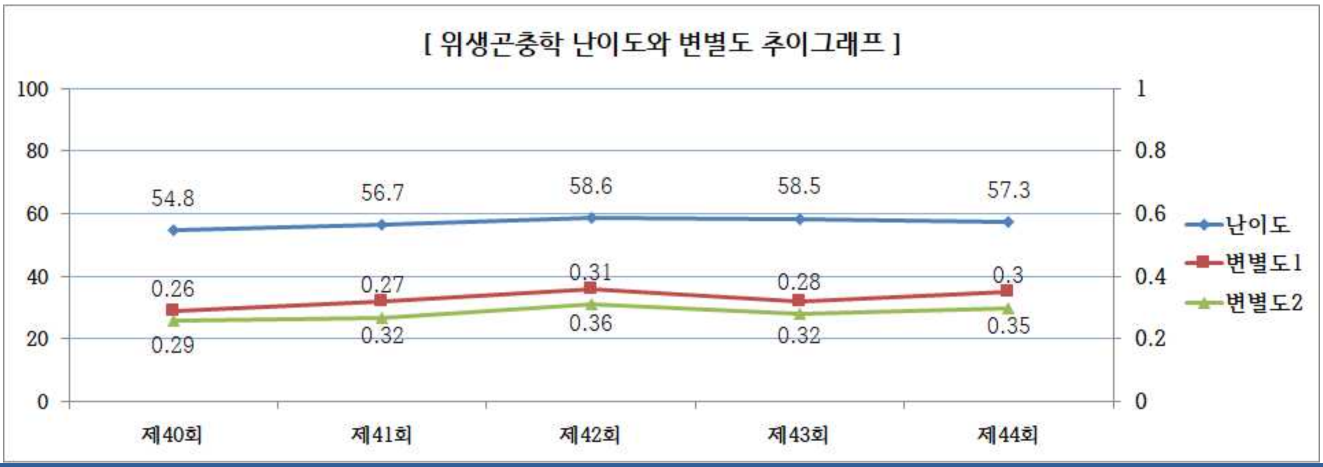

회차	난이도		변별도1		변별도2	
	평균	표준편차	평균	표준편차	평균	표준편차
제40회	54.8	24.5	.29	.17	.26	.14
제41회	56.7	20.4	.32	.15	.27	.12
제42회	58.6	19.3	.36	.16	.31	.13
제43회	58.5	21.4	.32	.17	.28	.14
**제44회**	**57.3**	**20.1**	**.35**	**.14**	**.30**	**.13**

(4) 전회 대비 공중보건학 난이도와 변별도

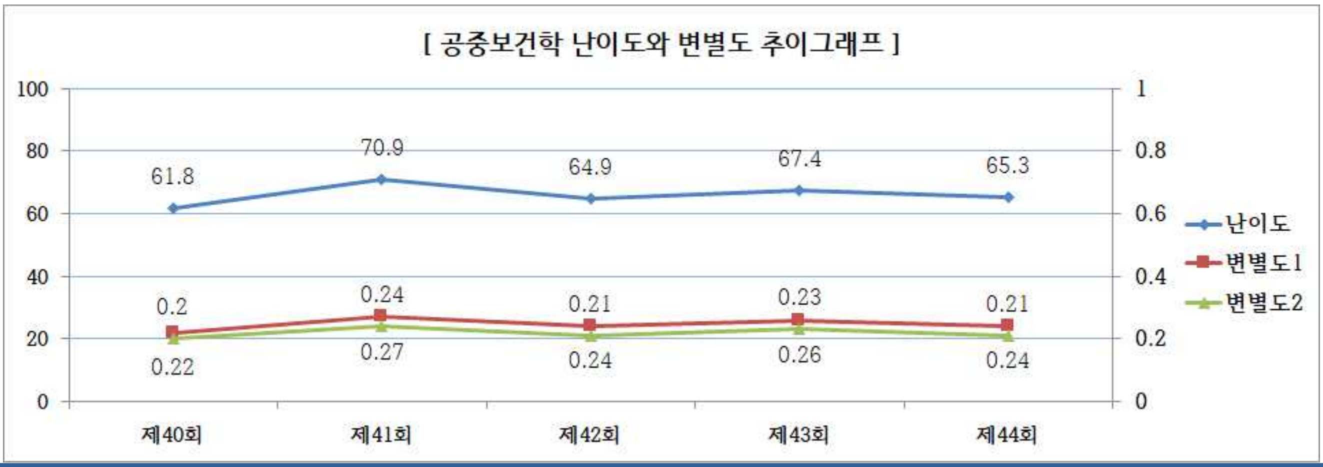

회차	난이도		변별도1		변별도2	
	평균	표준편차	평균	표준편차	평균	표준편차
제40회	61.8	23.1	.22	.15	.20	.12
제41회	70.9	20.4	.27	.17	.24	.13
제42회	64.9	20.3	.24	.14	.21	.11
제43회	67.4	21.2	.26	.19	.23	.13
**제44회**	**65.3**	**22.3**	**.24**	**.15**	**.21**	**.12**

(5) 전회 대비 식품위생학 난이도와 변별도

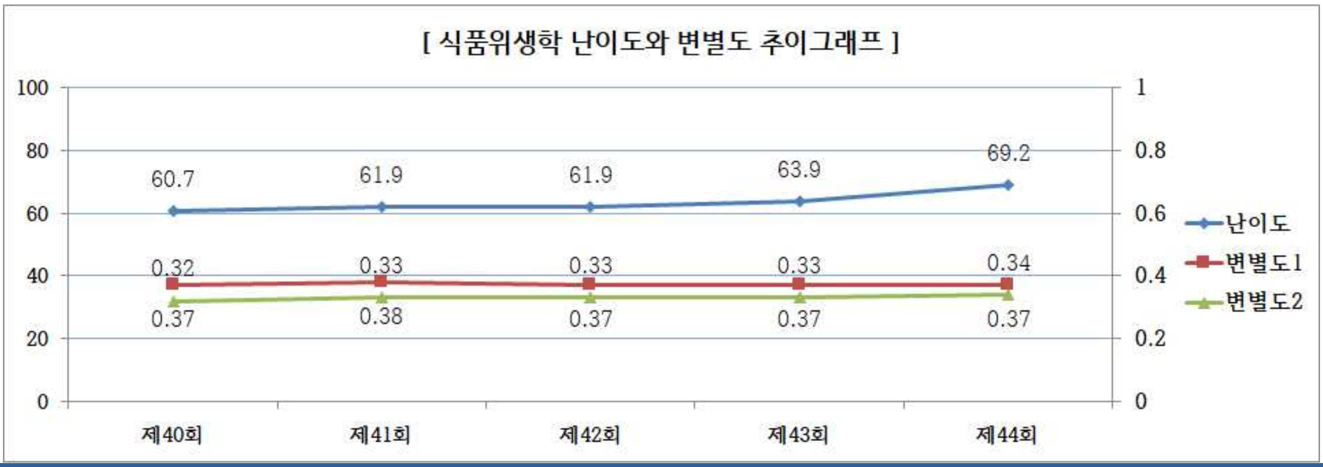

회차	난이도		변별도1		변별도2	
	평균	표준편차	평균	표준편차	평균	표준편차
제40회	60.7	20.3	.37	.17	.32	.14
제41회	61.9	20.3	.38	.15	.33	.11
제42회	61.9	21.7	.37	.18	.33	.14
제43회	63.9	21.9	.37	.18	.33	.13
**제44회**	**69.2**	**20.1**	**.37**	**.18**	**.34**	**.12**

(6) 전회 대비 실기시험 난이도와 변별도

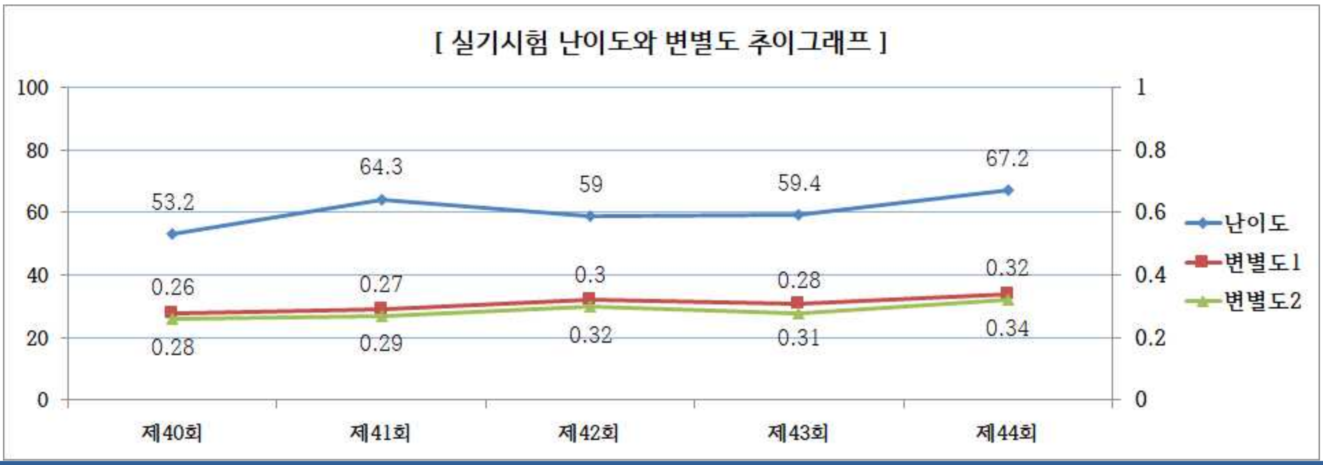

회차	난이도		변별도1		변별도2	
	평균	표준편차	평균	표준편차	평균	표준편차
제40회	53.2	25.3	.28	.17	.26	.15
제41회	64.3	21.7	.29	.17	.27	.14
제42회	59.0	23.2	.32	.17	.30	.16
제43회	59.4	23.7	.31	.17	.28	.16
**제44회**	**67.2**	**18.8**	**.34**	**.14**	**.32**	**.12**

해석

- 전회 대비 위생관계법령 과목의 난이도 지수는 1.1 감소, 변별도 1 지수 및 변별도 2 지수는 각각 .11, .07 증가함
- 환경위생학 과목의 난이도 지수는 6.0 증가, 변별도 1 지수 및 변별도 2 지수는 각각 .04, .01 감소함
- 위생곤충학 과목의 난이도 지수는 1.2 감소, 변별도 1 지수 및 변별도 2 지수는 각각 .03, .02 증가함
- 공중보건학 과목의 난이도 지수는 2.1 감소, 변별도 1 지수 및 변별도 2 지수는 각각 .02 씩 감소함
- 식품위생학 과목의 난이도 지수는 5.3 증가, 변별도 1 지수는 전년과 동일하고 변별도 2 지수는 .01 증가함
- 실기시험 과목의 난이도 지수는 7.8 증가, 변별도 1 지수 및 변별도 2 지수는 각각 .03, .04 증가함

나) 과목별 난이도와 변별도 분포도 및 비율분석

(1) 위생관계법령 난이도와 변별도 분포도 및 비율분석

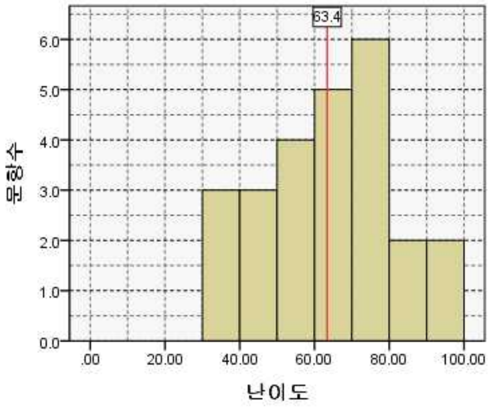

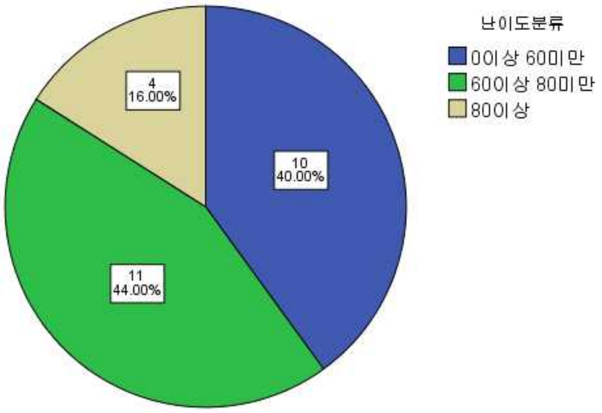

총점	난이도	표준편차
25	63.4	17.4

난이도	문항수	비율(%)
0~60미만	10	40.0
60~80미만	11	44.0
80~100	4	16.0
전체	25	100.0

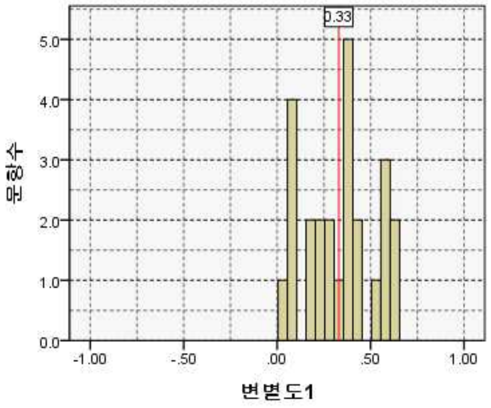

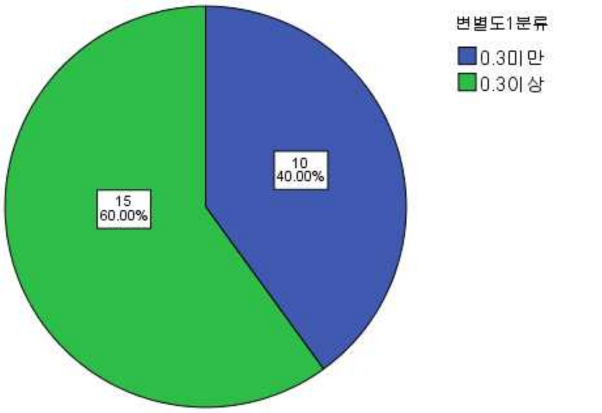

총점	변별도1	표준편차
25	.33	.19

변별도1	문항수	비율(%)
0.3미만	10	40.0
0.3이상	15	60.0
전체	25	100.0

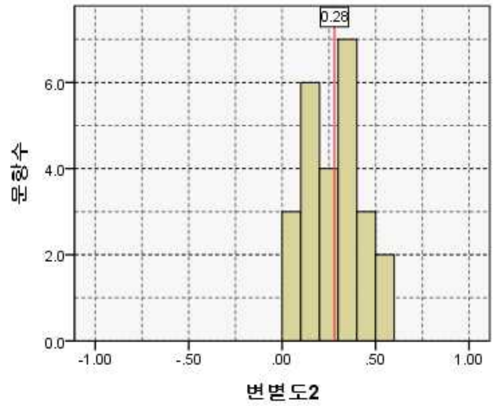

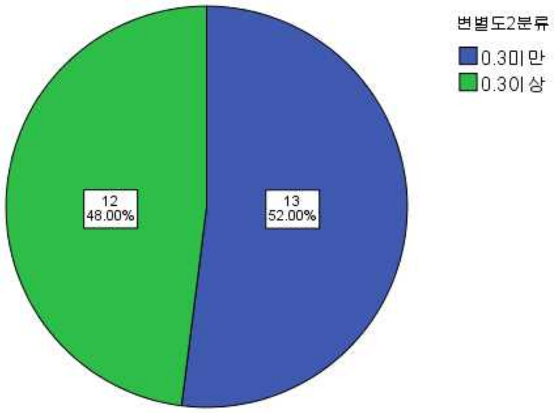

총점	변별도2	표준편차
25	.28	.14

변별도2	문항수	비율(%)
0.3미만	13	52.0
0.3이상	12	48.0
전체	25	100.0

해석

- 위생관계법령 과목에서 난이도 지수가 60 이상 80 미만인 문항이 11 문항으로 가장 많았으며, 60 미만인 문항이 10 문항, 80 이상인 문항이 4 문항으로 나타남
- 변별도 1 지수를 기준으로 분류하였을 때, 0.3 미만인 문항이 10 문항으로 0.3 이상인 문항이 15 문항인 것에 비해 더 적게 나타남
- 변별도 2 지수를 기준으로 분류하였을 때, 0.3 미만인 문항이 13 문항으로 0.3 이상인 문항이 12 문항인 것에 비해 더 많이 나타남

(2) 환경위생학 난이도와 변별도 분포도 및 비율분석

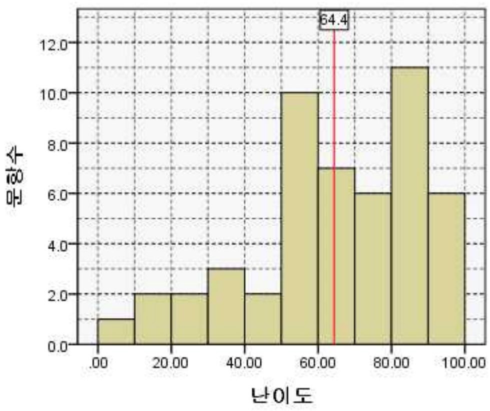

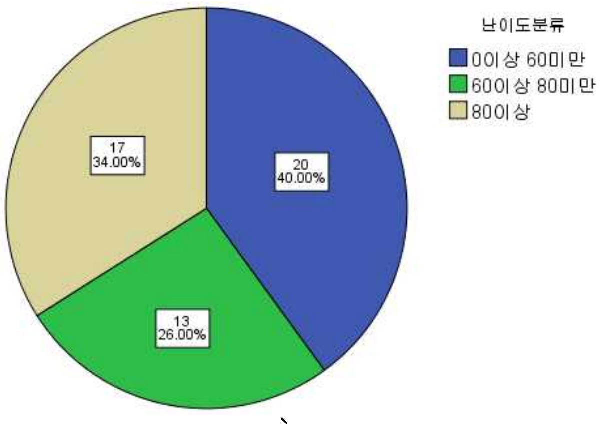

총점	난이도	표준편차
50	64.4	22.6

난이도	문항수	비율(%)
0~60미만	20	40.0
60~80미만	13	26.0
80~100	7	34.0
전체	50	100.0

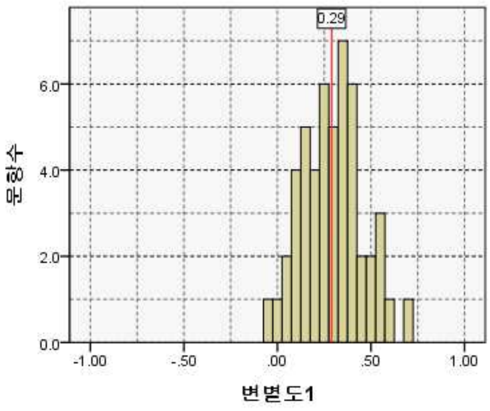

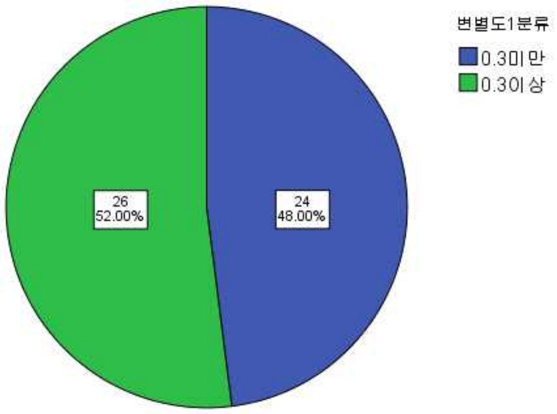

총점	변별도1	표준편차
50	.29	.16

변별도1	문항수	비율(%)
0.3미만	24	48.0
0.3이상	26	52.0
전체	50	100.0

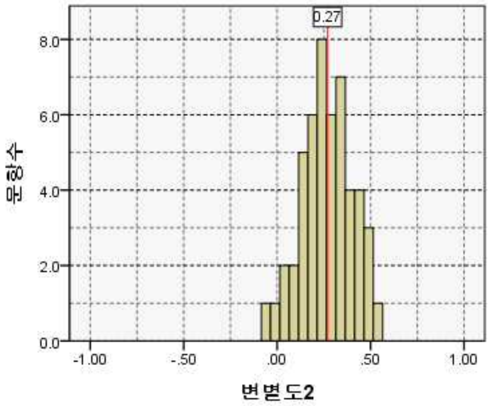

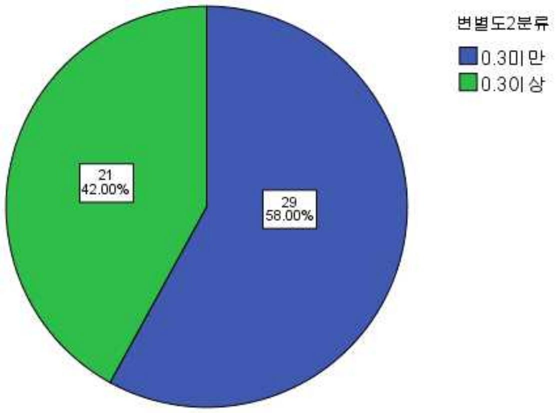

총점	변별도2	표준편차
50	.27	.14

변별도2	문항수	비율(%)
0.3미만	29	58.0
0.3이상	21	42.0
전체	50	100.0

해석

- 환경위생학 과목에서 난이도 지수가 60 미만인 문항이 20 문항으로 가장 많았으며, 60 이상 80 미만인 문항이 13 문항, 80 이상인 문항이 7 문항으로 나타남
- 변별도 1 지수를 기준으로 분류하였을 때, 0.3 미만인 문항이 24 문항으로 0.3 이상인 문항이 26 문항인 것에 비해 더 적게 나타남
- 변별도 2 지수를 기준으로 분류하였을 때, 0.3 미만인 문항이 29 문항으로 0.3 이상인 문항이 21 문항인 것에 비해 더 많이 나타남

(3) 위생곤충학 난이도와 변별도 분포도 및 비율분석

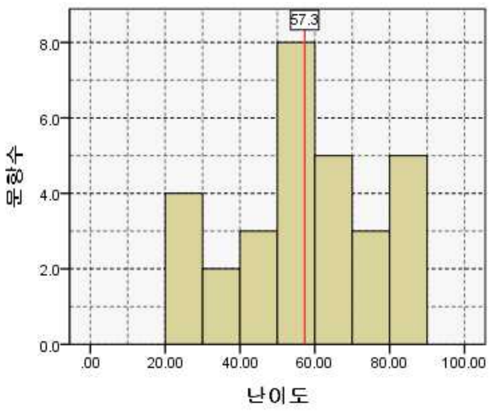

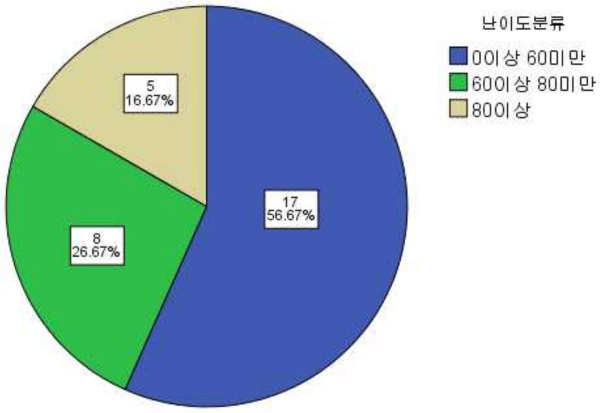

총점	난이도	표준편차
30	57.3	20.1

난이도	문항수	비율(%)
0~60미만	17	56.7
60~80미만	8	26.7
80~100	5	16.7
전체	30	100.0

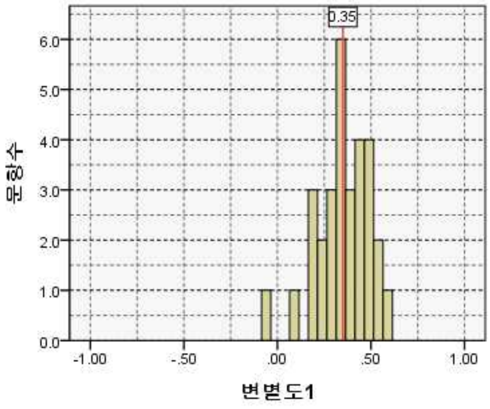

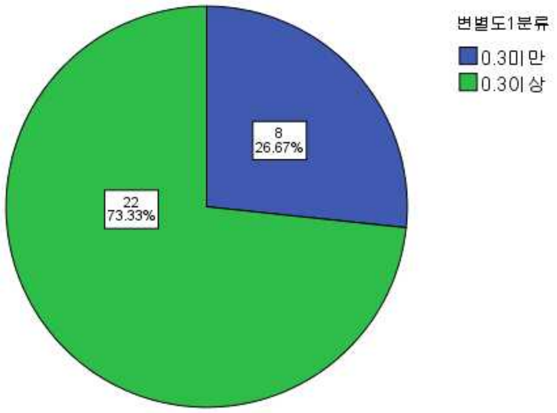

총점	변별도1	표준편차
30	.35	.14

변별도1	문항수	비율(%)
0.3미만	8	26.7
0.3이상	22	73.3
전체	30	100.0

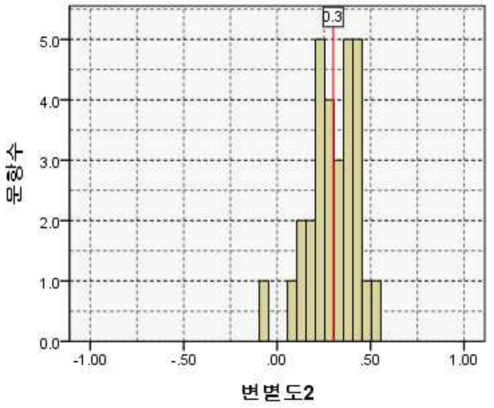

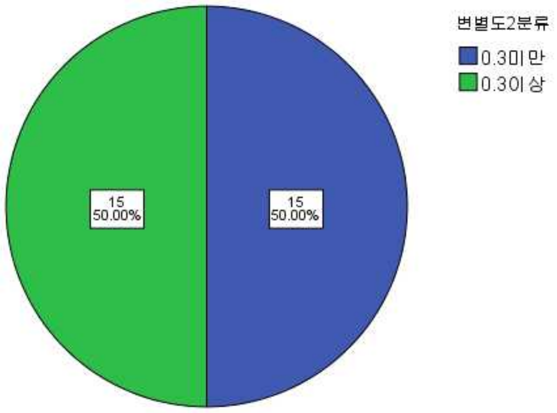

총점	변별도2	표준편차
30	.30	.13

변별도2	문항수	비율(%)
0.3미만	15	50.0
0.3이상	15	50.0
전체	30	100.0

해석

- 위생곤충학 과목에서 난이도 지수가 60 미만인 문항이 17 문항으로 가장 많았으며, 60 이상 80 미만인 문항이 8 문항, 80 이상인 문항이 5 문항으로 나타남
- 변별도 1 지수를 기준으로 분류하였을 때, 0.3 미만인 문항이 8 문항으로 0.3 이상인 문항이 22 문항인 것에 비해 더 적게 나타남
- 변별도 2 지수를 기준으로 분류하였을 때, 0.3 미만인 문항과 0.3 이상인 문항이 각각 15 문항씩 나타남

(4) 공중보건학 난이도와 변별도 분포도 및 비율분석

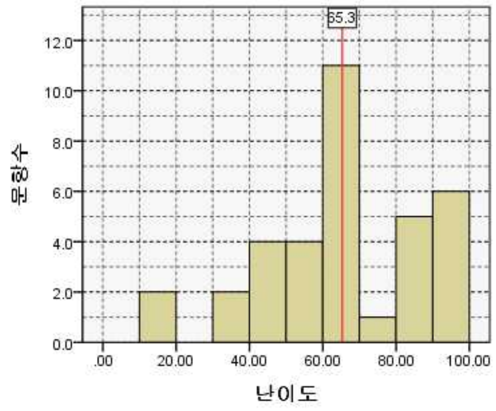

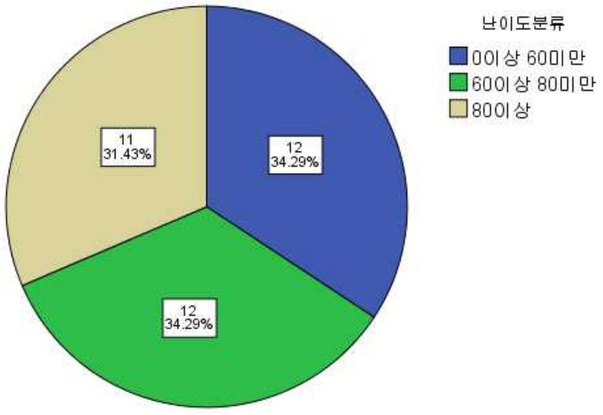

총점	난이도	표준편차
35	65.3	22.3

난이도	문항수	비율(%)
0~60미만	12	34.3
60~80미만	12	34.3
80~100	11	31.4
전체	35	100.0

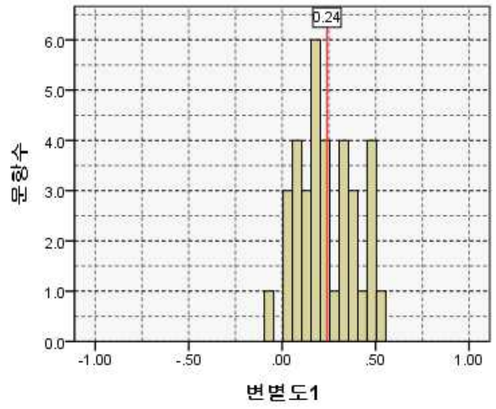

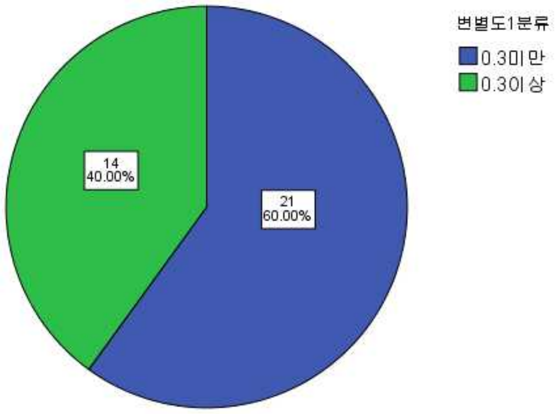

총점	변별도1	표준편차
35	.24	.15

변별도1	문항수	비율(%)
0.3미만	21	60.0
0.3이상	14	40.0
전체	35	100.0

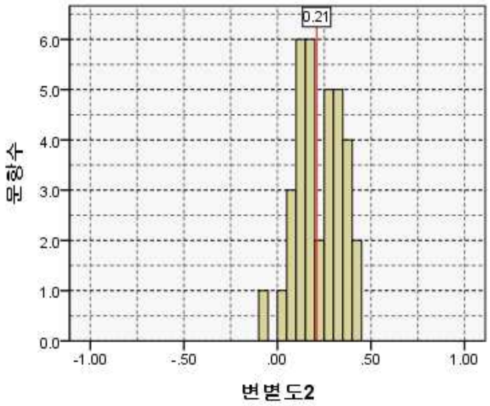

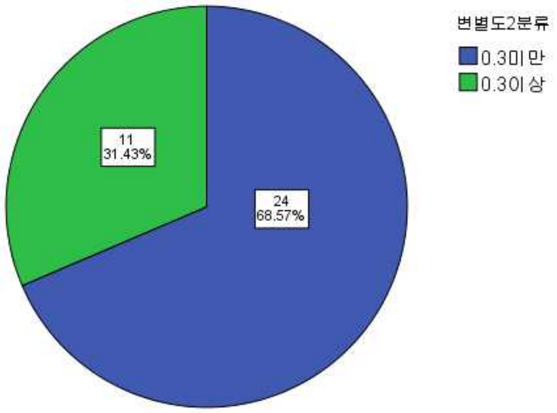

총점	변별도2	표준편차
35	.21	.12

변별도2	문항수	비율(%)
0.3미만	24	68.6
0.3이상	11	31.4
전체	35	100.0

해석

- 공중보건학 과목에서 난이도 지수가 60 미만인 문항과 60 이상 80 미만인 문항이 각각 12 문항으로 가장 많았으며, 80 이상인 문항이 11 문항으로 나타남
- 변별도 1 지수를 기준으로 분류하였을 때, 0.3 미만인 문항이 21 문항으로 0.3 이상인 문항이 14 문항인 것에 비해 더 많이 나타남
- 변별도 2 지수를 기준으로 분류하였을 때, 0.3 미만인 문항이 24 문항으로 0.3 이상인 문항이 11 문항인 것에 비해 더 많이 나타남

(5) 식품위생학 난이도와 변별도 분포도 및 비율분석

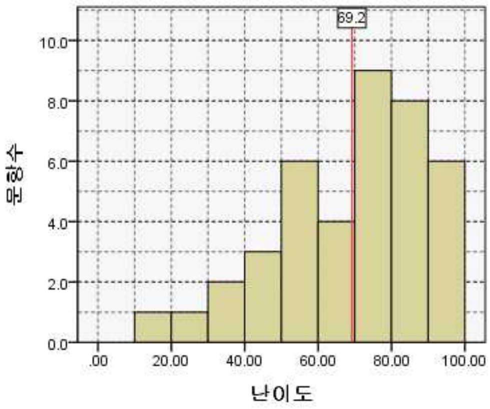

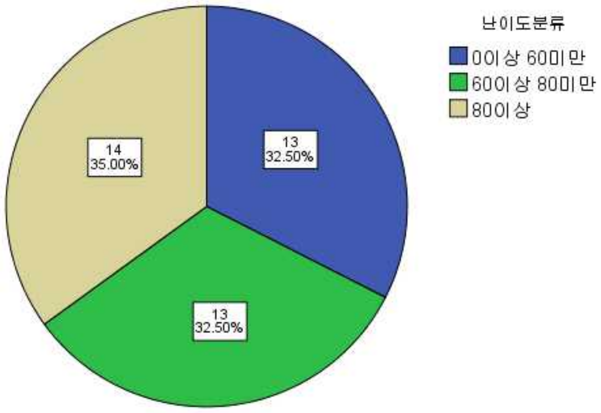

총점	난이도	표준편차
40	69.2	20.1

난이도	문항수	비율(%)
0~60미만	13	32.5
60~80미만	13	32.5
80~100	14	35.0
전체	40	100.0

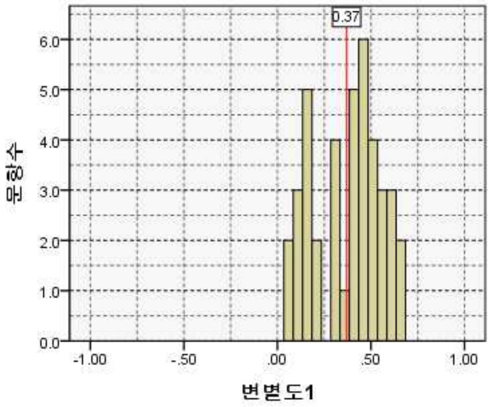

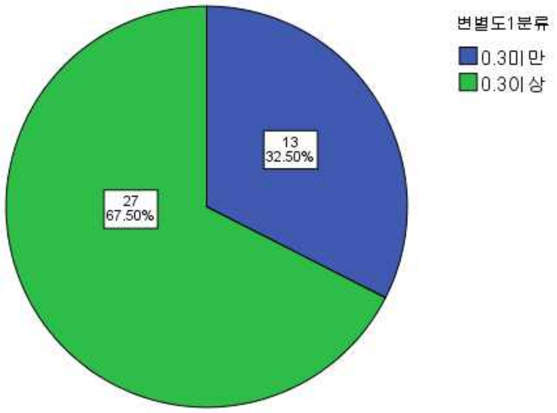

총점	변별도1	표준편차
40	.37	.18

변별도1	문항수	비율(%)
0.3미만	13	32.5
0.3이상	27	67.5
전체	40	100.0

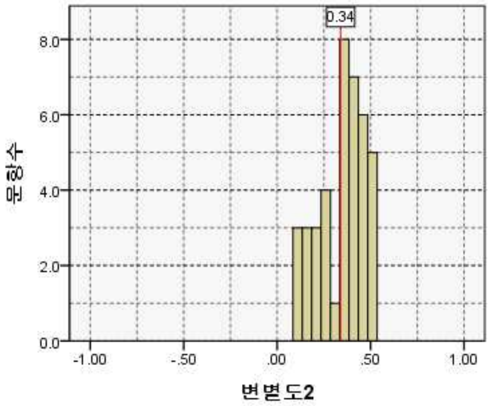

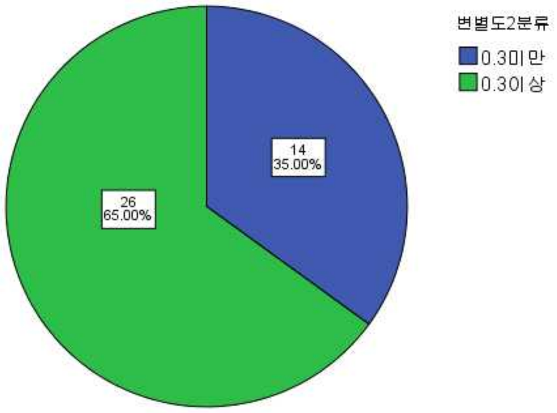

총점	변별도2	표준편차
40	.34	.12

변별도2	문항수	비율(%)
0.3미만	14	35.0
0.3이상	26	65.0
전체	40	100.0

해석

- 식품위생학 과목에서 난이도 지수가 80 이상인 문항이 14 문항으로 가장 많았으며, 60 미만인 문항과 60 이상 80 미만인 문항이 각각 13 문항씩 나타남
- 변별도 1 지수를 기준으로 분류하였을 때, 0.3 미만인 문항이 13 문항으로 0.3 이상인 문항이 27 문항인 것에 비해 더 적게 나타남
- 변별도 2 지수를 기준으로 분류하였을 때, 0.3 미만인 문항이 14 문항으로 0.3 이상인 문항이 26 문항인 것에 비해 더 적게 나타남

(6) 실기시험 난이도와 변별도 분포도 및 비율분석

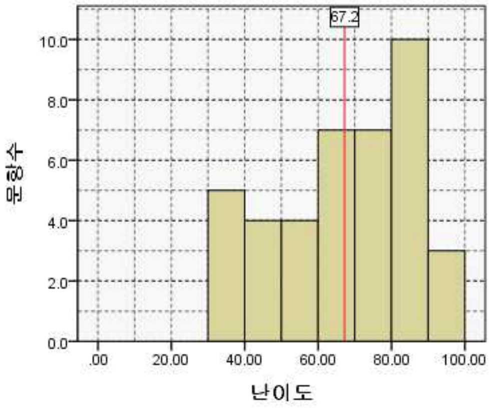

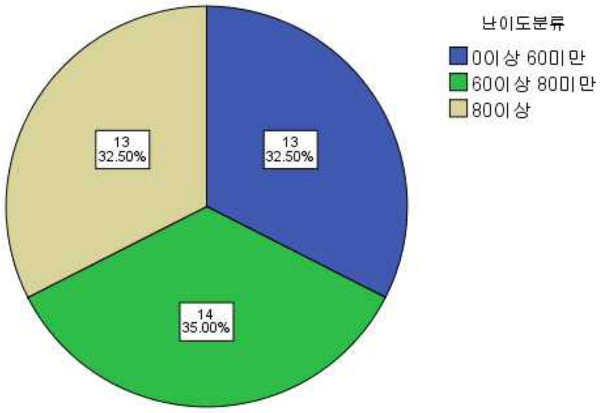

총점	난이도	표준편차
40	67.2	18.8

난이도	문항수	비율(%)
0~60미만	13	32.5
60~80미만	14	35.0
80~100	13	32.5
전체	40	100.0

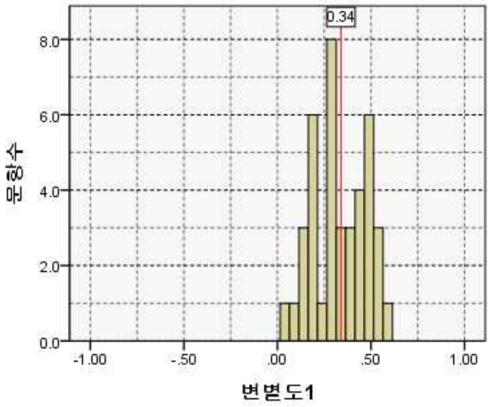

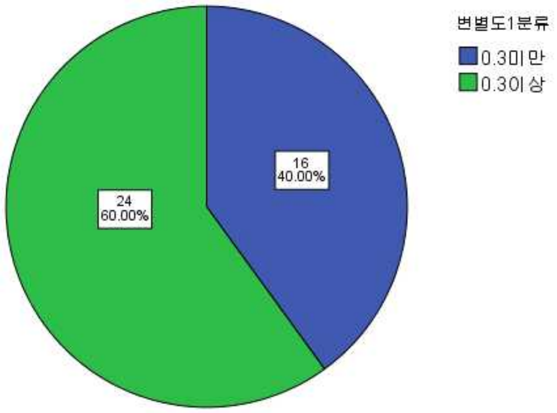

총점	변별도1	표준편차
40	.34	.14

변별도1	문항수	비율(%)
0.3미만	16	40.0
0.3이상	24	60.0
전체	40	100.0

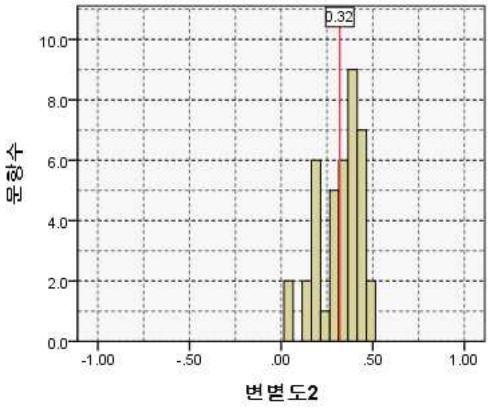

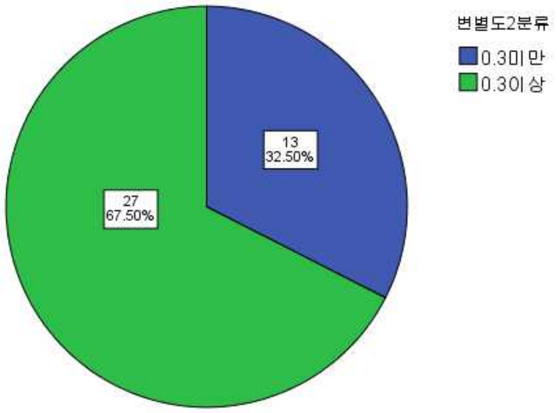

총점	변별도2	표준편차
40	.32	.12

변별도2	문항수	비율(%)
0.3미만	13	32.5
0.3이상	27	67.5
전체	40	100.0

해석

- 실기시험 과목에서 난이도 지수가 60 이상 80 미만인 문항이 14 문항으로 가장 많았으며, 60 미만인 문항과 80 이상인 문항이 각각 13 문항씩 나타남
- 변별도 1 지수를 기준으로 분류하였을 때, 0.3 미만인 문항이 16 문항으로 0.3 이상인 문항이 24 문항인 것에 비해 더 적게 나타남
- 변별도 2 지수를 기준으로 분류하였을 때, 0.3 미만인 문항이 13 문항으로 0.3 이상인 문항이 27 문항인 것에 비해 더 적게 나타남

3) 지식수준별 난이도와 변별도

가) 전회 대비 지식수준별 난이도와 변별도

(1) 전회 대비 암기형 난이도와 변별도

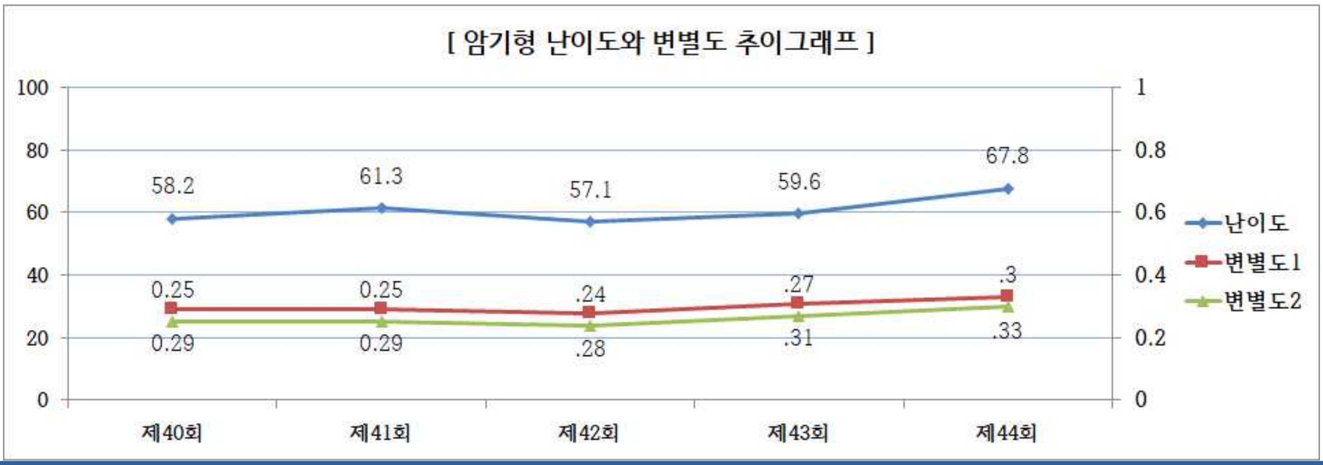

회차	난이도		변별도1		변별도2	
	평균	표준편차	평균	표준편차	평균	표준편차
제40회	58.2	23.2	.29	.18	.25	.14
제41회	61.3	20.6	.29	.17	.25	.14
제42회	57.1	22.2	.28	.17	.24	.15
제43회	59.6	21.8	.31	.17	.27	.14
**제44회**	**67.8**	**18.8**	**.33**	**.18**	**.30**	**.14**

(2) 전회 대비 해석형 난이도와 변별도

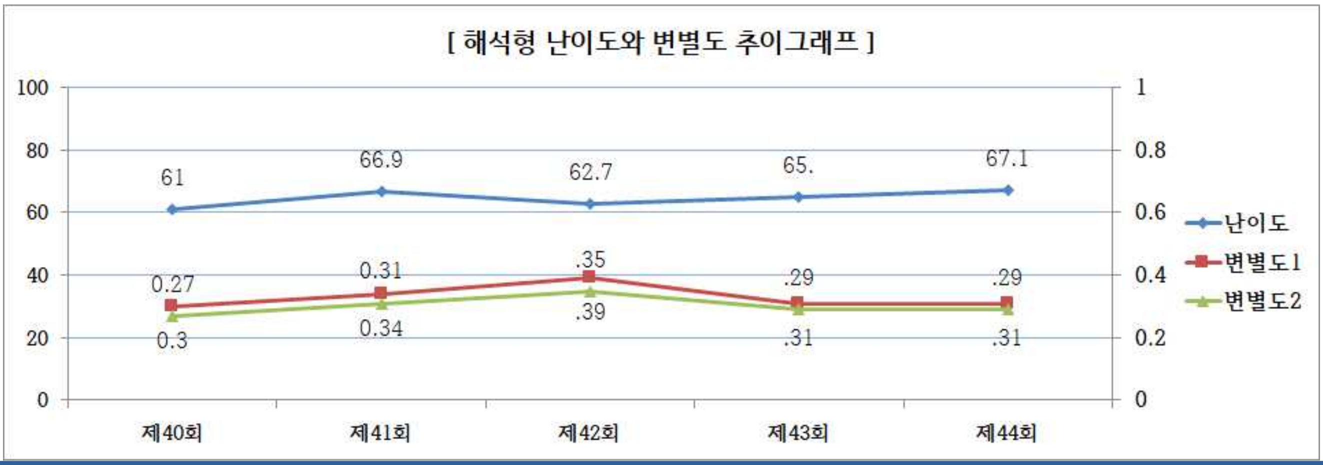

회차	난이도		변별도1		변별도2	
	평균	표준편차	평균	표준편차	평균	표준편차
제40회	61.0	21.7	.30	.18	.27	.15
제41회	66.9	20.1	.34	.14	.31	.11
제42회	62.7	20.0	.39	.17	.35	.14
제43회	65.0	22.4	.31	.17	.29	.13
**제44회**	**67.1**	**20.4**	**.31**	**.16**	**.29**	**.13**

(3) 전회 대비 해결형 난이도와 변별도

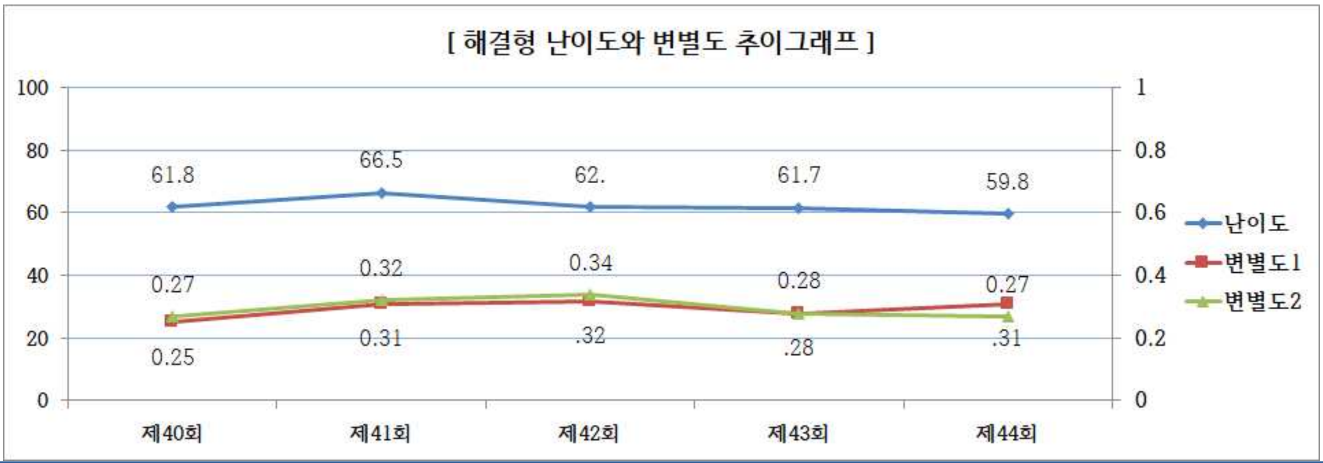

회차	난이도		변별도1		변별도2	
	평균	표준편차	평균	표준편차	평균	표준편차
제40회	61.8	25.4	.25	.15	.23	.13
제41회	66.5	20.9	.31	.18	.28	.14
제42회	62.0	22.7	.32	.18	.29	.14
제43회	61.7	23.4	.28	.18	.26	.16
**제44회**	**59.8**	**21.8**	**.31**	**.17**	**.27**	**.13**

해석

- 전회 대비 암기형 문항, 해석형 문항의 난이도 지수는 각각 8.2, 2.1 증가, 해결형 문항에서는 1.9 감소함
- 변별도 1 지수의 경우 암기형 문항, 해결형 문항에서는 각각 .02, .03 증가하였으며, 해석형 문항에서는 작년과 동일함
- 변별도 2 지수의 경우 암기형 문항, 해결형 문항에서는 각각 .03, .01 증가하였으며, 해석형 문항에서는 작년과 동일함

나) 지식수준별 난이도와 변별도 분포도 및 비율분석

(1) 암기형 난이도와 변별도 분포도 및 비율분석

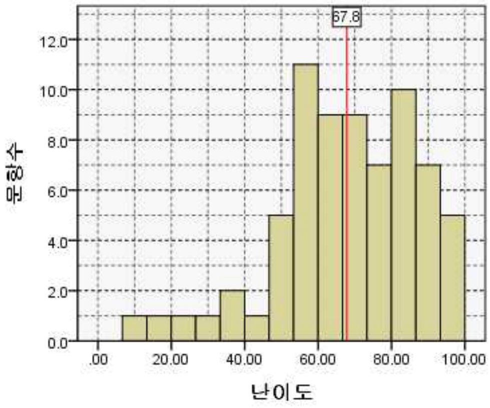

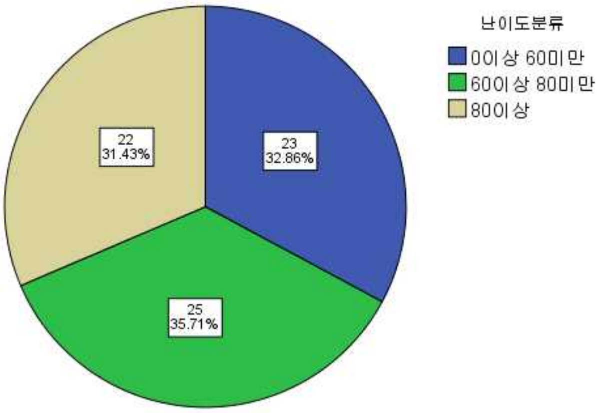

총점	난이도	표준편차
70	67.8	18.8

난이도	문항수	비율(%)
0~60미만	23	32.9
60~80미만	25	35.7
80~100	22	31.4
전체	70	100.0

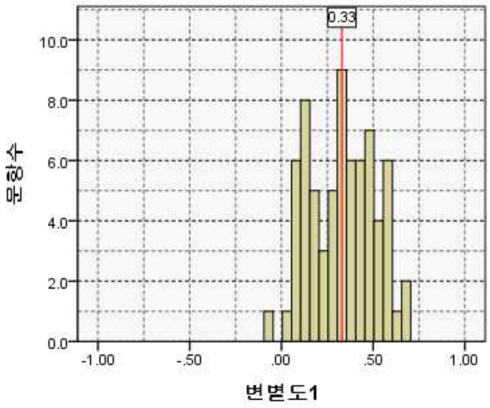

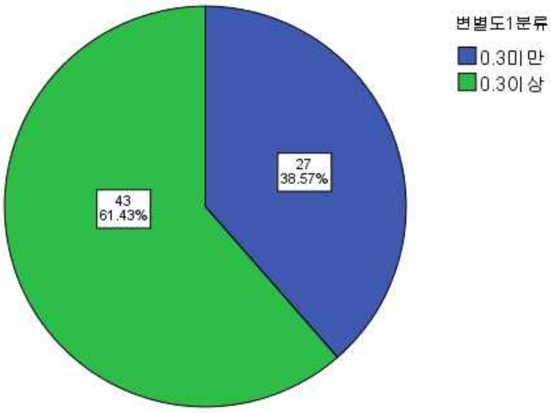

총점	변별도1	표준편차
70	.33	.18

변별도1	문항수	비율(%)
0.3미만	27	38.6
0.3이상	43	61.4
전체	70	100.0

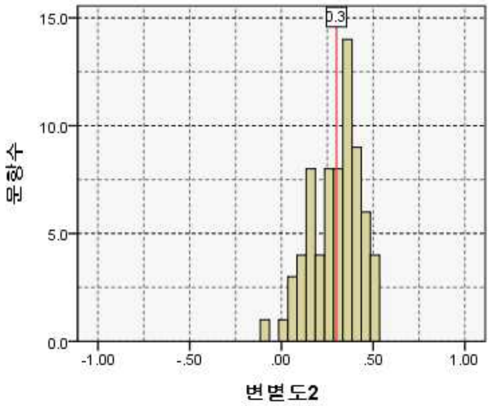

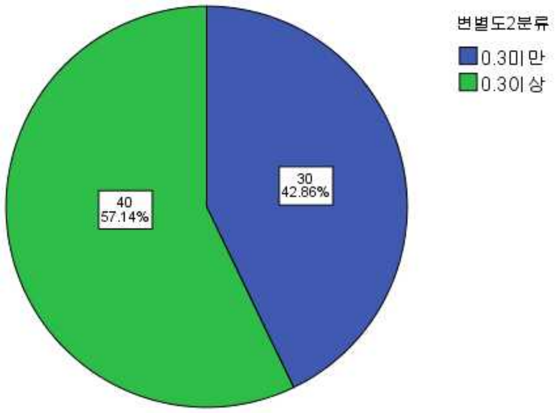

총점	변별도2	표준편차
70	.30	.14

변별도2	문항수	비율(%)
0.3미만	30	42.9
0.3이상	40	57.1
전체	70	100.0

해석

- 암기형 문항에서 난이도 지수가 60 이상 80 미만인 문항이 25 문항으로 가장 많았으며, 60 미만인 문항이 23 문항, 80 이상인 문항이 22 문항으로 나타남
- 변별도 1 지수를 기준으로 분류하였을 때, 0.3 미만인 문항이 27 문항으로 0.3 이상인 문항이 43 문항인 것에 비해 더 적게 나타남
- 변별도 2 지수를 기준으로 분류하였을 때, 0.3 미만인 문항이 30 문항으로 0.3 이상인 문항이 40 문항인 것에 비해 더 적게 나타남

(2) 해석형 난이도와 변별도 분포도 및 비율분석

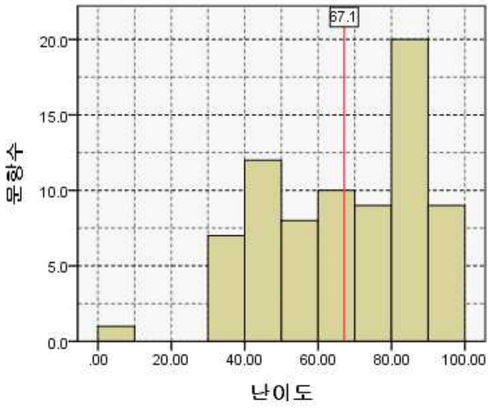

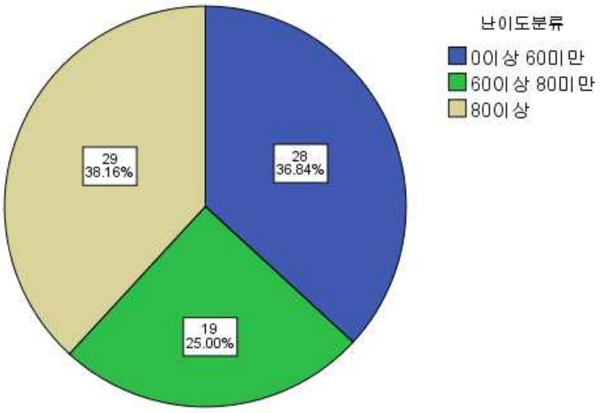

총점	난이도	표준편차
76	67.1	20.4

난이도	문항수	비율(%)
0~60미만	28	36.8
60~80미만	19	25.0
80~100	29	38.2
전체	76	100.0

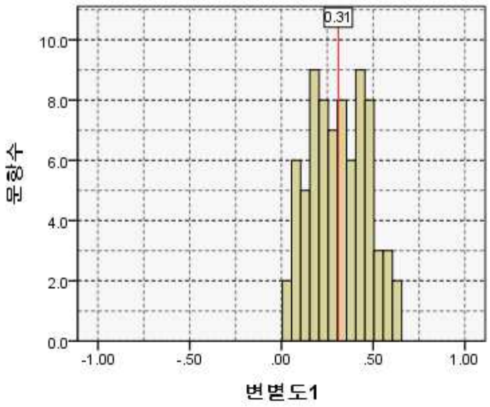

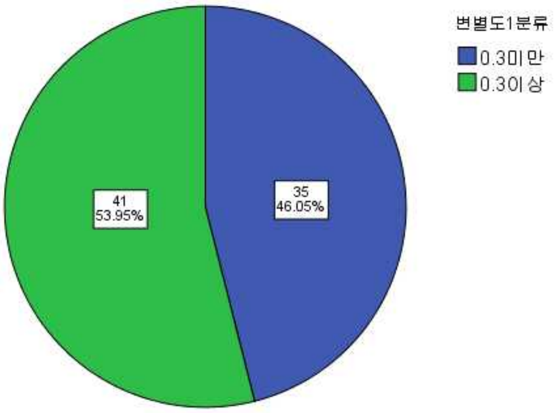

총점	변별도1	표준편차
76	.31	.16

변별도1	문항수	비율(%)
0.3미만	35	46.1
0.3이상	41	53.9
전체	76	100.0

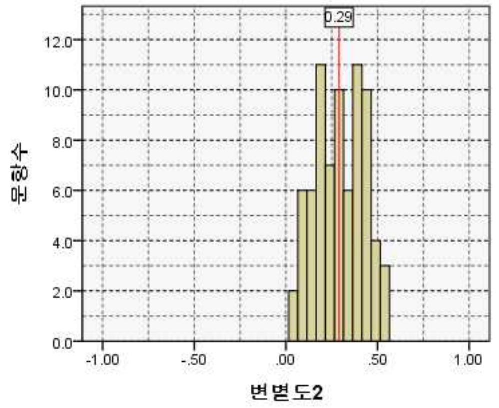

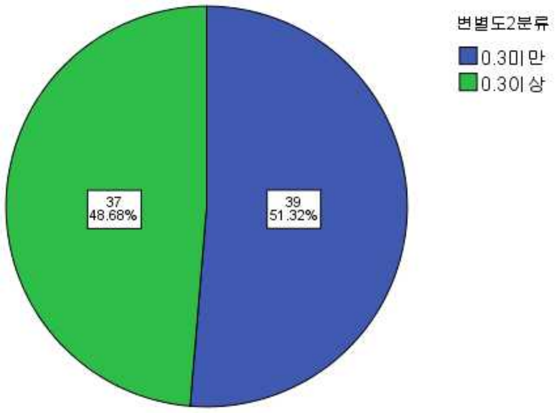

총점	변별도2	표준편차
76	.29	.13

변별도2	문항수	비율(%)
0.3미만	39	51.3
0.3이상	37	48.7
전체	76	100.0

해석

- 해석형 문항에서 난이도 지수가 80 이상인 문항이 29 문항으로 가장 많았으며, 60 미만인 문항이 28 문항, 60 이상 80 미만인 문항이 19 문항으로 나타남
- 변별도 1 지수를 기준으로 분류하였을 때, 0.3 미만인 문항이 35 문항으로 0.3 이상인 문항이 41 문항인 것에 비해 더 적게 나타남
- 변별도 2 지수를 기준으로 분류하였을 때, 0.3 미만인 문항이 39 문항으로 0.3 이상인 문항이 37 문항인 것에 비해 더 많이 나타남

(3) 해결형 난이도와 변별도 분포도 및 비율분석

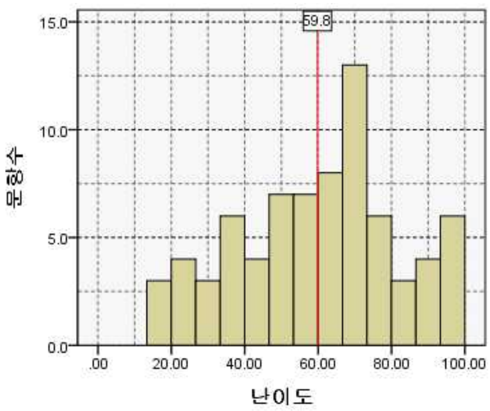

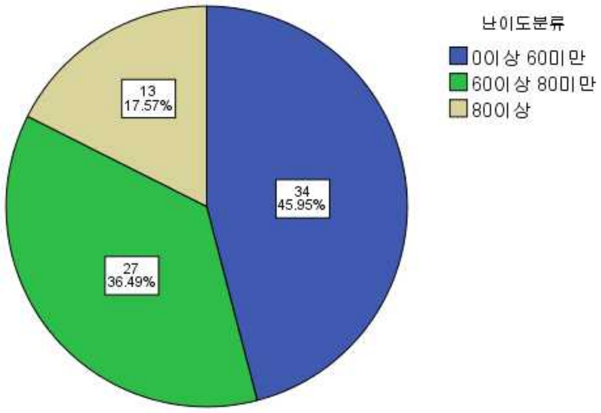

총점	난이도	표준편차
74	59.8	21.8

난이도	문항수	비율(%)
0~60미만	34	45.9
60~80미만	27	36.5
80~100	13	17.6
전체	74	100.0

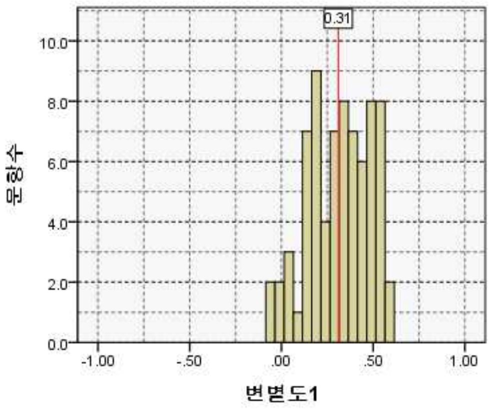

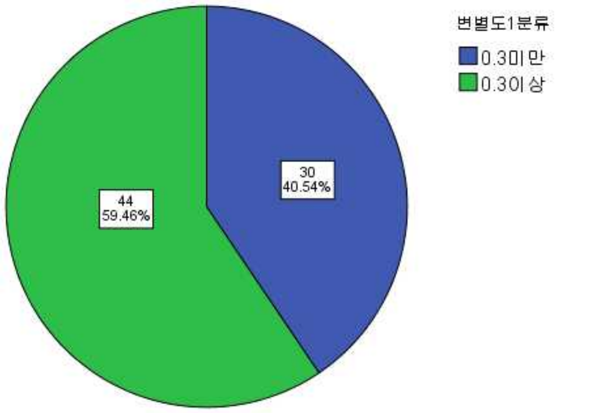

총점	변별도1	표준편차
74	.31	.17

변별도1	문항수	비율(%)
0.3미만	30	40.5
0.3이상	44	59.5
전체	74	100.0

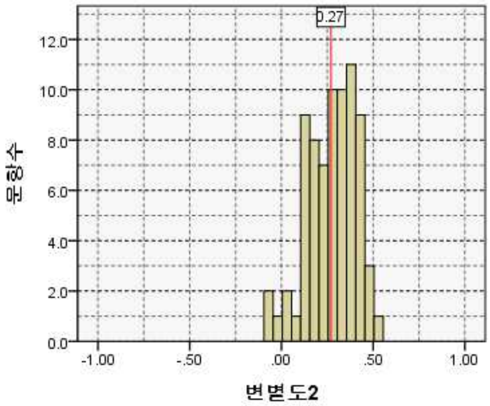

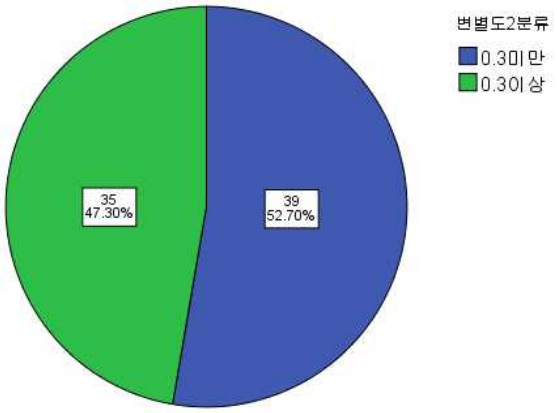

총점	변별도2	표준편차
74	.27	.13

변별도2	문항수	비율(%)
0.3미만	39	52.7
0.3이상	35	47.3
전체	74	100.0

해석

- 해결형 문항에서 난이도 지수가 60 미만인 문항이 34 문항으로 가장 많았으며, 60 이상 80 미만인 문항이 27 문항, 80 이상인 문항이 13 문항으로 나타남
- 변별도 1 지수를 기준으로 분류하였을 때, 0.3 미만인 문항이 30 문항으로 0.3 이상인 문항이 44 문항인 것에 비해 더 적게 나타남
- 변별도 2 지수를 기준으로 분류하였을 때, 0.3 미만인 문항이 39 문항으로 0.3 이상인 문항이 35 문항인 것에 비해 더 많이 나타남

3. 난이도와 변별도 간 산포도

1) 전체 난이도와 변별도 간 산포도

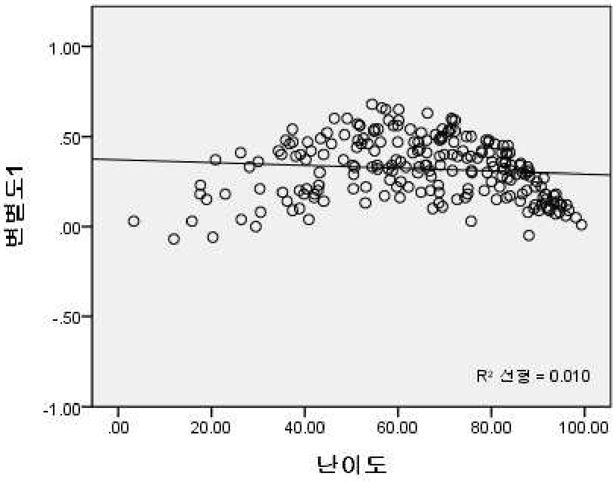

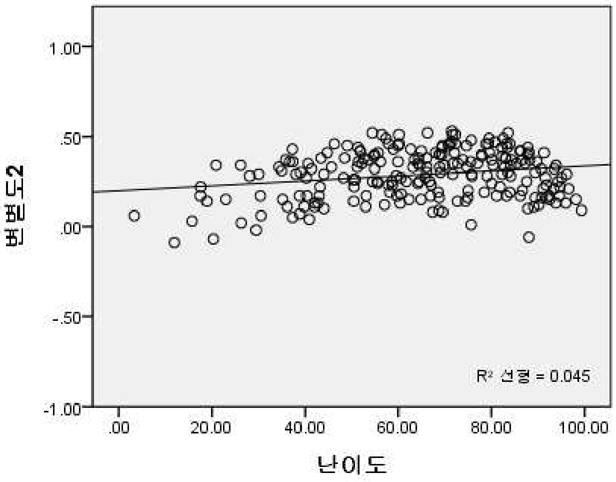

해석

- 전체 문항을 대상으로 난이도 지수와 변별도 1 지수 간 상관은 -.098 로 난이도 지수와 변별도 지수 간 관련성이 적은 것으로 나타남
- 난이도 지수와 변별도 2 지수 간 상관은 .212**으로 난이도 지수가 높을수록 변별력이 높아지는 것으로 나타남

2) 과목별 난이도와 변별도 간 산포도

가) 위생관계법령 난이도와 변별도 간 산포도

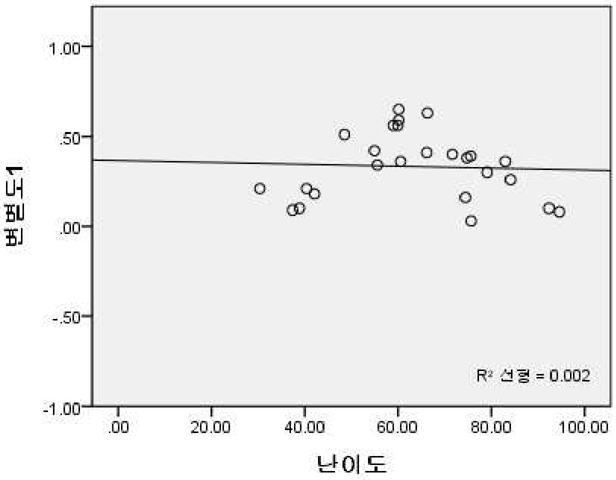

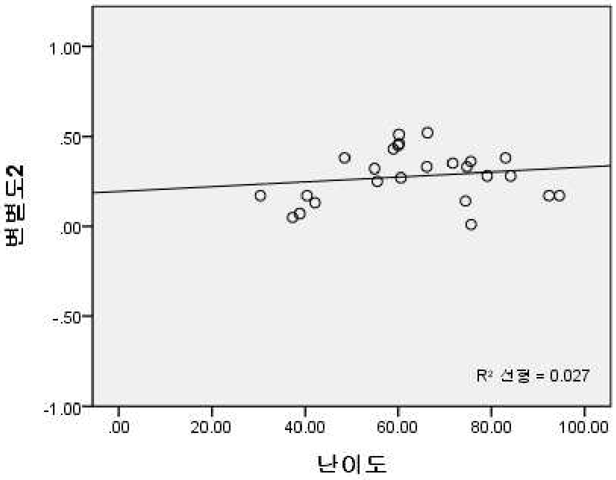

해석
- 위생관계법령 난이도 지수와 변별도 1 지수 간 상관은 -.050 로 난이도 지수와 변별도 지수 간 관련성이 적은 것으로 나타남<br>- 난이도 지수와 변별도 2 지수 간 상관은 .164 로 난이도 지수와 변별도 지수 간 관련성이 적은 것으로 나타남

나) 환경위생학 난이도와 변별도 간 산포도

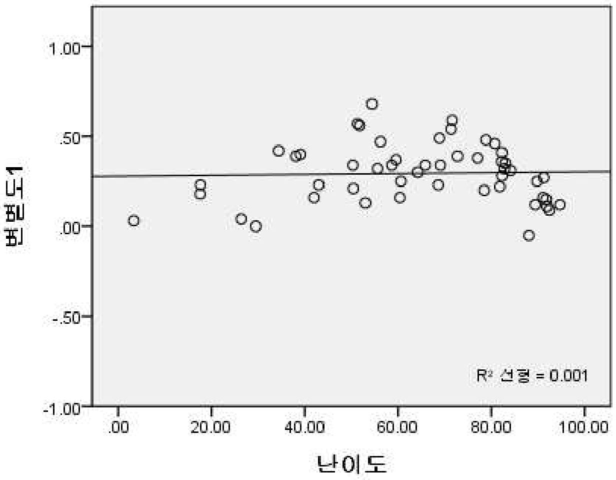

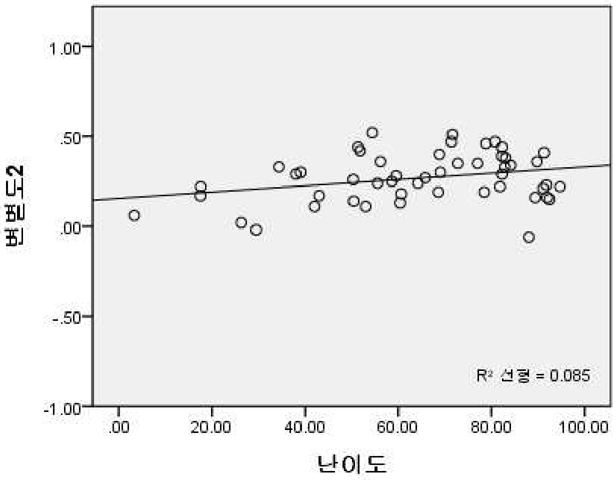

해석
- 환경위생학 과목 문항을 대상으로 난이도 지수와 변별도 1 지수 간 상관은 .034 로 문항 난이도 지수와 변별도 간 관련성이 적은 것으로 나타남<br>- 난이도 지수와 변별도 2 지수 간 상관은 .292*으로 난이도 지수가 높을수록 변별력이 높아지는 것으로 나타남

다) 위생곤충학 난이도와 변별도 간 산포도

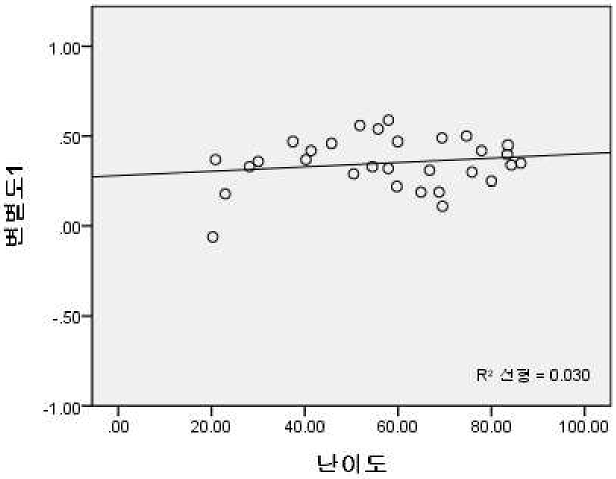

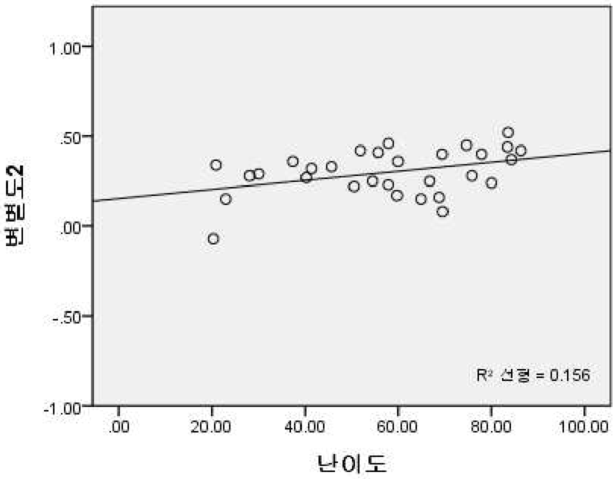

해석

- 위생곤충학 과목 문항을 대상으로 난이도 지수와 변별도 1 지수 간 상관은 .174 로 문항 난이도 지수와 변별도 간 관련성이 적은 것으로 나타남
- 난이도 지수와 변별도 2 지수 간 상관은 .395*으로 난이도 지수가 높을수록 변별력이 높아지는 것으로 나타남

라) 공중보건학 난이도와 변별도 간 산포도

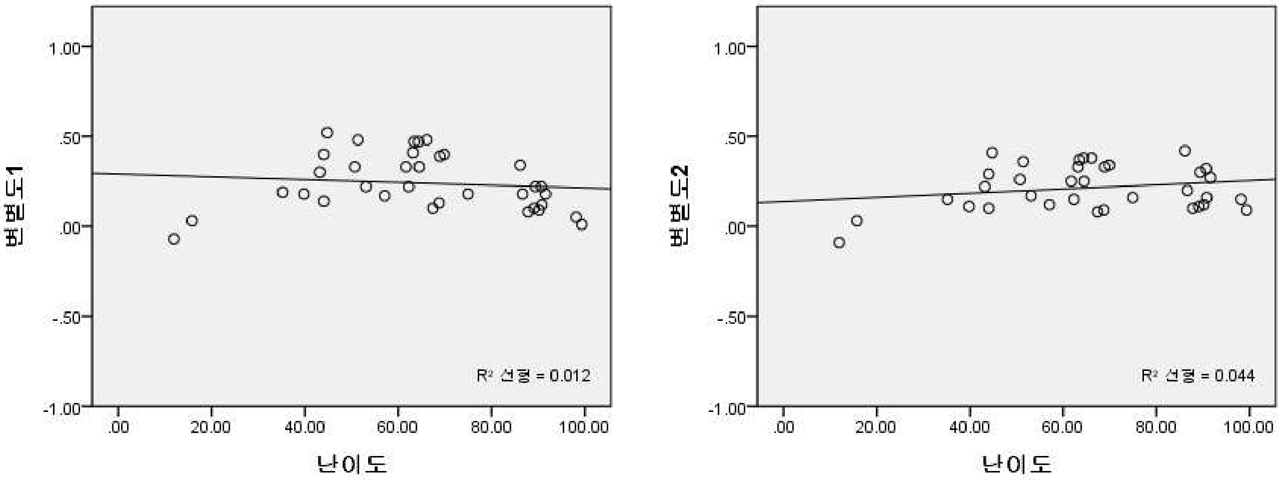

해석

- 공중보건학 과목 문항을 대상으로 -.110 로 난이도 지수와 변별도 지수 간 관련성이 없는 것으로 나타남
- 난이도 지수와 변별도 2 지수 간 상관은 .209 로 난이도 지수와 변별도 지수 간 관련성이 없는 것으로 나타남

마) 식품위생학 난이도와 변별도 간 산포도

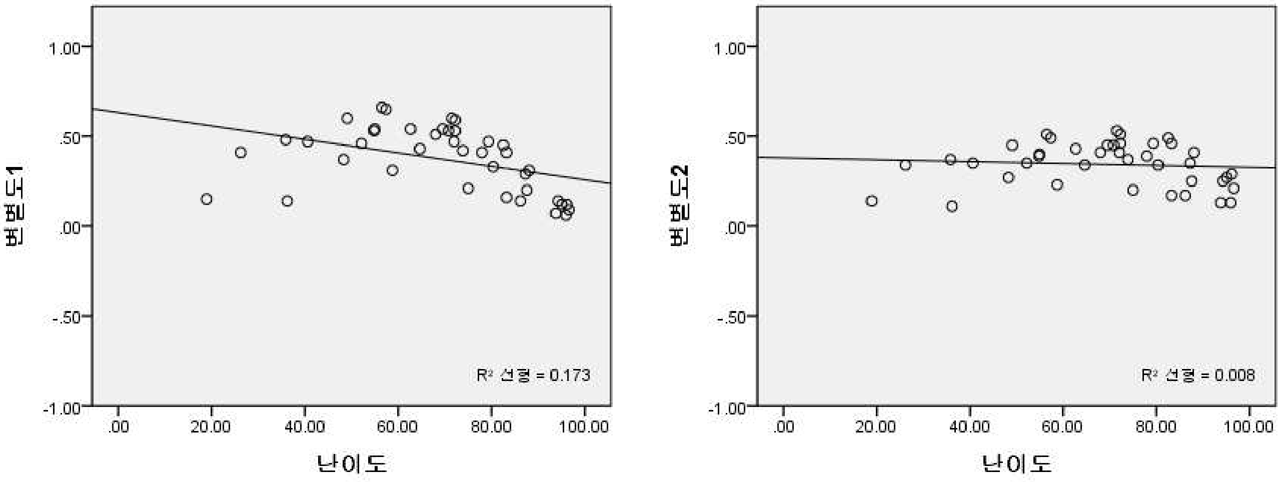

해석

- 식품위생학 과목 문항을 대상으로 난이도 지수와 변별도 1 지수 간 상관은 -.416**으로 난이도 지수가 높을수록 변별력이 낮아지는 것으로 나타남
- 난이도 지수와 변별도 2 지수 간 상관은 -.088 로 문항 난이도 지수와 변별도 지수 간 관련성이 적은 것으로 나타남

바) 실기시험 난이도와 변별도 간 산포도

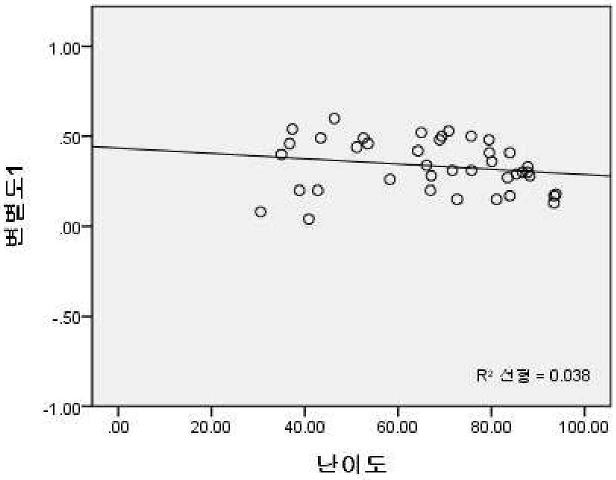

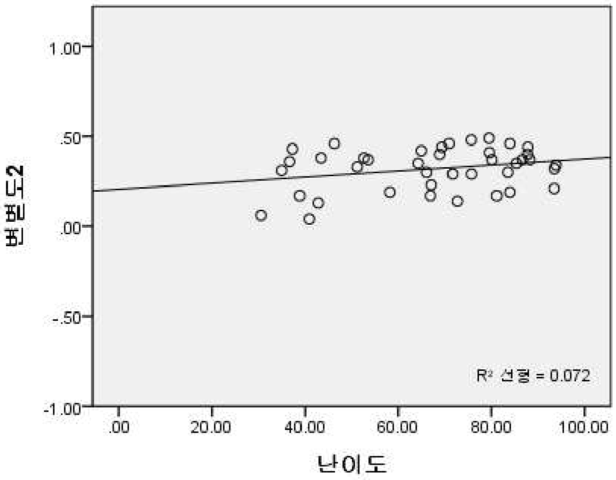

해석

- 실기시험 과목 문항을 대상으로 난이도 지수와 변별도 1 지수 간 상관은 -.195 로 난이도 지수와 변별도 지수 간 관련성이 적은 것으로 나타남
- 난이도 지수와 변별도 2 지수 간 상관은 .268 로 난이도 지수와 변별도 지수 간 관련성이 적은 것으로 나타남

4. 신뢰도 분석

과목명	문항수	제40회	제41회	제42회	제43회	제44회
전체	220	.941	.951	.955	.950	**.954**
위생관계법령	25	.525	.533	.507	.590	**.715**
환경위생학	50	.815	.865	.879	.827	**.813**
위생곤충학	30	.712	.733	.781	.732	**.757**
공중보건학	35	.638	.745	.682	.715	**.660**
식품위생학	40	.845	.851	.852	.848	**.862**
실기시험	40	.756	.776	.812	.789	**.824**

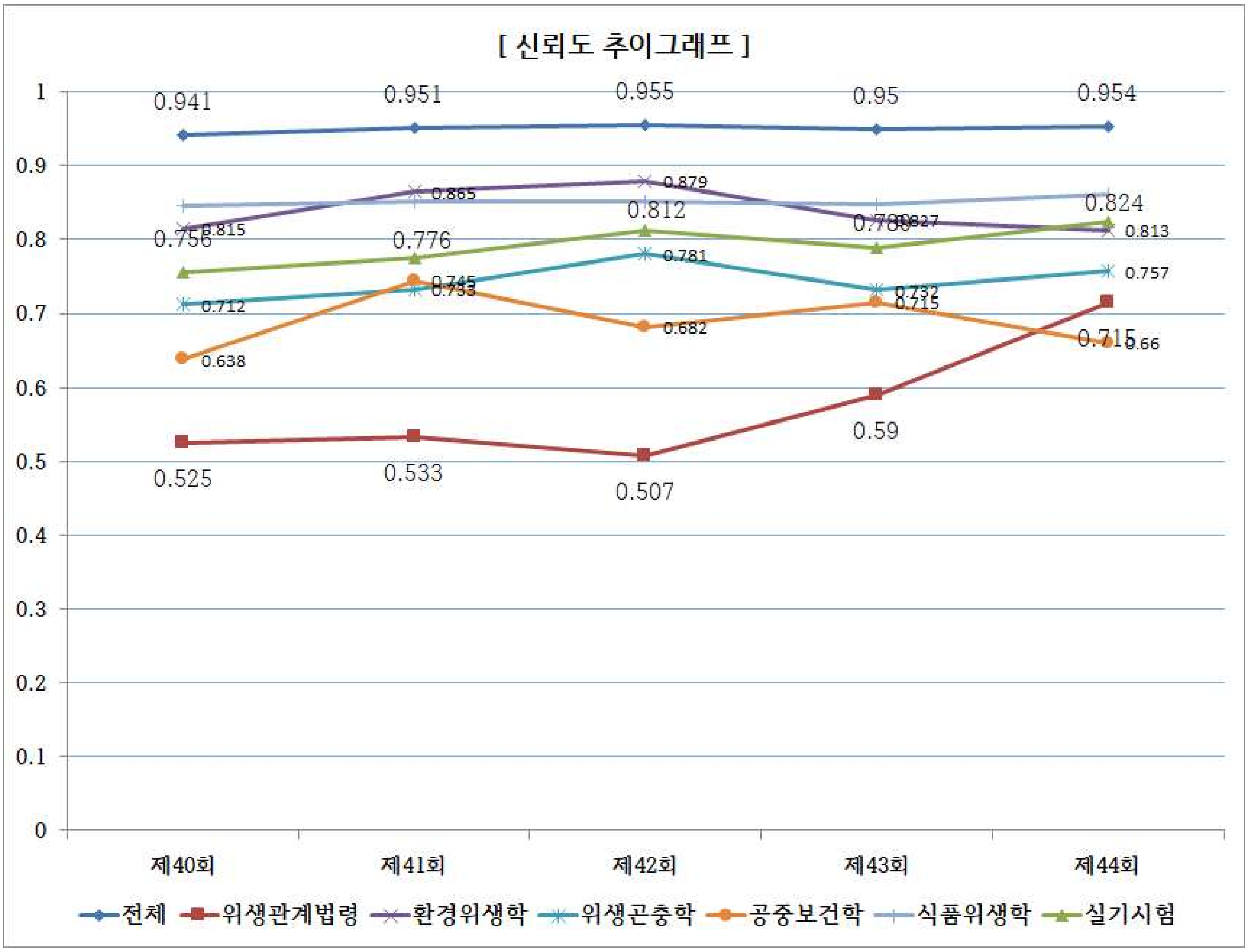

해석

- 위생사 국가시험 전체와 각 과목의 문항 모두 일관되게 해당 영역을 측정하고 있는 것으로 나타남
- 전회 대비 신뢰도는 전체문항을 대상으로 했을 시 0.004 증가함
- 각 과목별 문항을 대상으로 했을 시 위생관계법령은 .125 증가, 환경위생학은 .014 감소, 위생곤충학은 .025 증가, 공중보건학은 .055 감소, 식품위생학은 .014 증가, 실기시험은 .035 증가함

○ 분석결과 관련 문의 : 한국보건의료인국가시험원 연구개발본부 김보현 전임연구원
Tel : 02-2087-8954, FAX : 02-2087-8885
E-mail : kimbohyun@kuksiwon.or.kr
