# Supplementary material for: Item difficulty index, discrimination index, and reliability of the 26 health professions licensing examinations in 2022, Korea: a psychometric study
Source: J Educ Eval Health Prof. 2023 Nov 22;20:31. doi: 10.3352/jeehp.2023.20.31 (PMC11959405; doi:10.3352/jeehp.2023.20.31)
Supplement: Supplementary file 1 — Supplement 1. Item analysis results of 26 health professions licensing examinations administered during late 2022 and early 2023. [file jeehp-20-31_Suppl1.zip › 2022│Γ╡╡ ┴a46╚╕ ┐╡╛τ╗τ ▒╣░í╜├╟Φ ║╨╝«░ß░·.pdf]

2022년도 제46회 영양사 국가시험 문항분석 결과

국민이 신뢰하고 감동하는 시험평가기관
한국보건의료인국가시험원
KOREA HEALTH PERSONNEL LICENSING EXAMINATION INSTITUTE

일반 용어 정의

□ **평균**
- 집단에서의 대표적 경향값으로 전체 값을 더하여 총 응시자로 나눈 값

□ **표준편차**
- 평균과 각 점수의 차이인 편차들의 평균으로 점수가 흩어져 분포되어 있는 정도

□ **추정난이도**
- 문항개발자가 예측한 정답률

□ **검사이론**
- 검사와 검사를 구성하고 있는 문항의 양호도를 분석 및 평가하는 방법을 정의한 이론체계
- 대표적으로 고전검사이론과 문항반응이론이 있음

고전검사이론 용어 정의

□ **고전검사이론(Classical Test Theory; CTT)**

- 검사의 질을 분석하는 검사이론 중 한 가지로 19세기 말부터 전개되어 현재까지 주로 사용되고 있는 검사이론임
- 고전검사이론에 의한 문항과 응시자 능력 추정치는 다음과 같음

○ **문항난이도**

· 검사 문항의 쉽고 어려운 정도를 나타내는 지수
· 난이도 지수는 총 반응 수에 대한 정답 반응 수의 비율로 문항의 정답률임
· 문항난이도는 0~100까지의 값을 가짐
· 난이도 값이 큰 경우, 쉬운 문항으로 '난이도가 낮다'라고 해석하며, 난이도 값이 작은 경우, 어려운 문항으로 '난이도가 높다'라고 해석함

○ **문항변별도**

· 각 문항이 응시자의 능력 수준을 변별할 수 있는 정도를 나타내는 지수
· 문항변별도는 −1~+1까지의 값을 가지며, 1에 가까울수록 변별력 크다고 해석함
· 일반적으로 문항변별도가 0.3 이상이면 우수한 문항으로 평가함
· 구하는 방식에는 '상하위집단 구분법', '문항-총점 상관계수' 등이 있음
  1) 변별도 1(상하위구분법): 상위 27%와 하위 27% 집단의 난이도 차이를 구하는 방식
  2) 변별도 2(상관계수법): 문항-총점과의 상관계수로 구하는 방식

○ **신뢰도**

· 시험이 평가하고자 하는 것을 일관성 있게 측정하는가로 시험이 오차없이 정확하게 측정한 정도를 의미함
· 국시원에서는 문항의 내적일관성(Cronbach α)으로 신뢰도를 추정하며 1에 가까울수록 신뢰도가 높다고 해석함

목 차

I. 시행 결과 ······ 6

1. 시험 현황 ······ **7**

1) 시험명 ······ 7

2) 시험시행일 ······ 7

3) 응시현황 ······ 7

4) 과목별 문항 수, 배점 및 과락 점수 ······ 7

2. 합격률과 평균성적 ······ **7**

1) 합격 및 불합격 현황 ······ 7

2) 과목별 과락자수 내역 ······ 7

3) 전회 대비 합격률과 평균성적 ······ 8

II. 문항분석 결과 ······ 9

1. 성적 ······ **10**

1) 전체 성적분포도 ······ 10

2) 과목별 성적분포도 ······ 11

2. 난이도와 변별도 ······ **12**

1) 전체 난이도와 변별도 ······ 12

2) 과목별 난이도와 변별도 ······ 15

3) 지식수준별 난이도와 변별도 ······ 26

3. 난이도와 변별도 간 산포도 ······ **34**

1) 전체 난이도와 변별도 간 산포도 ······ 34

2) 과목별 난이도와 변별도 간 산포도 ······ 34

4. 신뢰도 분석 ······ **37**

I. 시행 결과

1. 시험 현황

1) 시험명: 2022년도 제46회 영양사 국가시험

2) 시험시행일: 2022년 12월 17일

3) 응시현황

응시대상자수	결시자수	부정행위자수	응시자 준수사항 위반자 수		응시자수 (%)
			휴대폰 소지	신분증 미지참	
5,899	498	0	0	0	5,398 (91.5)

※ 5,398명은 응시대상자(5,899명)에서 결시자수(498명) 및 채점보류자수(3명)를 제외한 수치임

4) 과목별 문항 수, 배점 및 과락 점수

교 시	과 목 명	문제 수	배점	총점	합격자 점수기준	
					과목 과락기준	총점 합격기준
1교시	영양학 및 생화학	60	1	60	24점 미만	132점 이상
	영양교육, 식사요법 및 생리학	60	1	60	24점 미만	
2교시	식품학 및 조리원리	40	1	40	16점 미만	
	급식, 위생 및 관계법규	60	1	60	24점 미만	
계		220		220		

2. 합격률과 평균성적

1) 합격 및 불합격 현황

합격자수 (%)	불합격자수(%)				채점보류자수
	평락	과락	기권	계	
3,629 (67.2)	1,762 (32.64)	3 (0.06)	4 (0.07)	1,769 (32.8)	3

2) 과목별 과락자수 내역

과목명 / 과락자수	영양학 및 생화학	영양교육, 식사요법 및 생리학	식품학 및 조리원리	급식, 위생 및 관계법규
과목별 과락자 수	-	-	1	2
전과목 과락자 수	-			

3) 전회 대비 합격률과 평균성적

회차	년도	합격률(%)	평균성적	표준편차	백분율 환산점수
제42회	2018	69.8	148.6	29.8	67.5
제43회	2019	54.9	134.3	31.2	61.1
제44회	2020	70.2	147.6	29.6	67.1
제45회	2021	74.9	152.7	29.0	69.4
**제46회**	**2022**	**67.2**	**147.5**	**33.0**	**67.0**

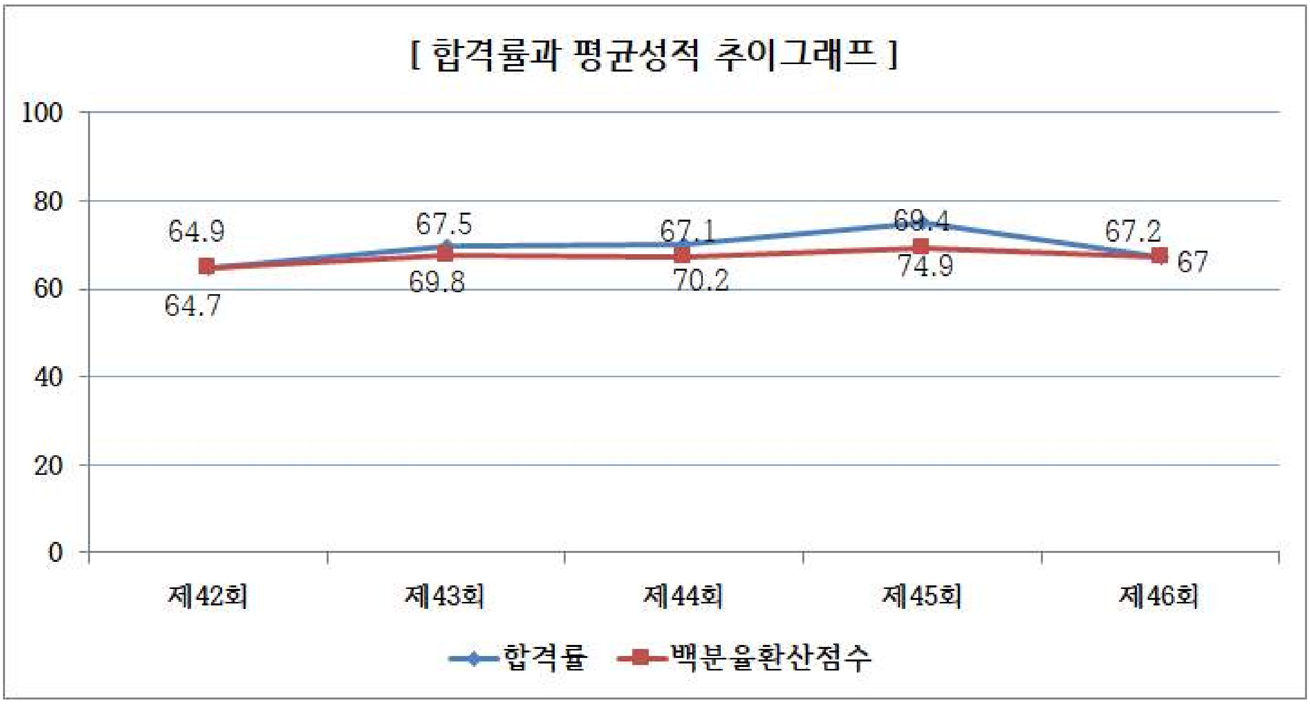

해석

- 전년 대비 합격률은 7.7% 감소하였으며, 백분율 환산점수는 2.4 점 감소함

II. 문항분석 결과

1. 성적

1) 전체 성적분포도

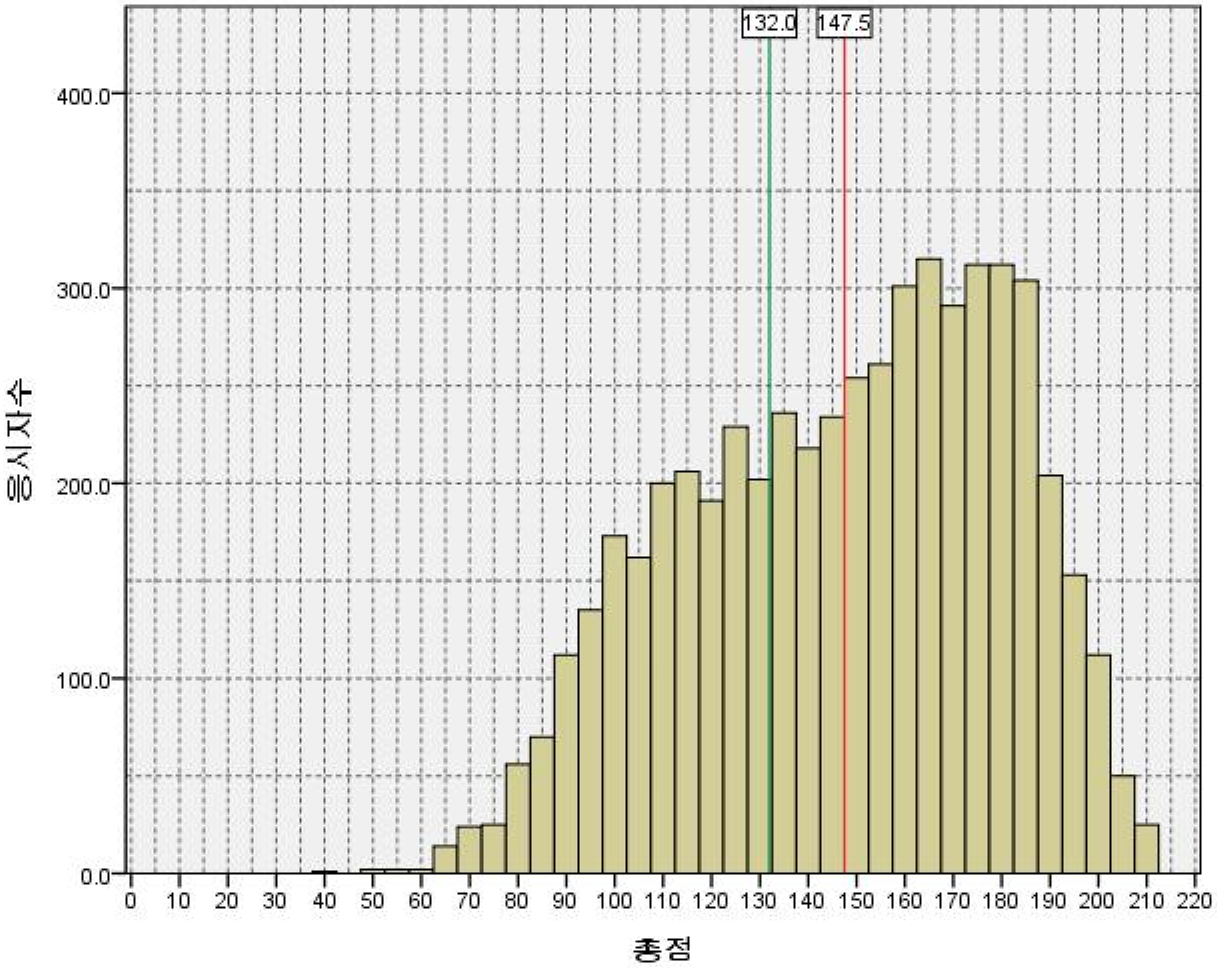

응시자	총점	합격선	평균성적	표준편차
5,397	220	132	147.5	33.0

※ 5,397명은 전체응시자(5,398명)에서 채점보류자(3명)를 더하고 기권자(4명)를 제외한 수치임

2) 과목별 성적분포도

가) 영양학 및 생화학

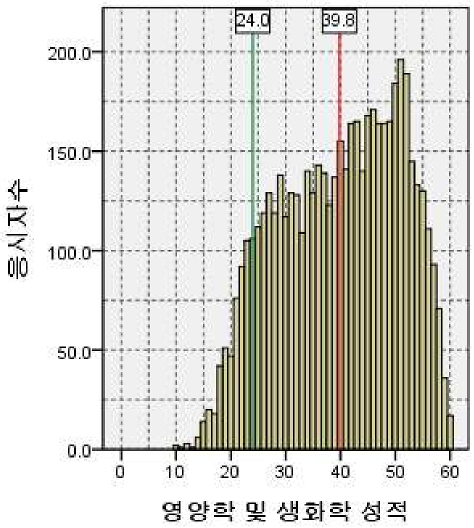

총점	과락선	평균성적	표준편차
60	24	39.8	11.1

나) 영양교육, 식사요법 및 생리학

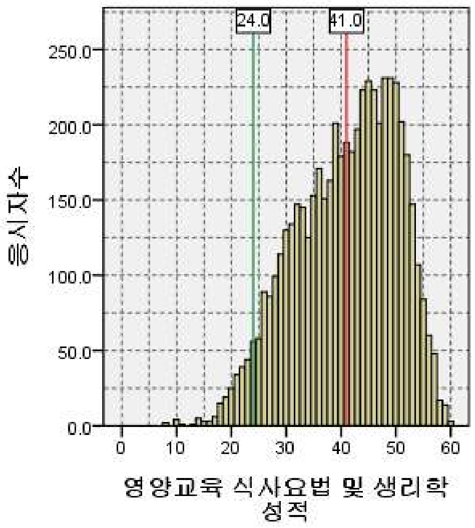

총점	과락선	평균성적	표준편차
60	24	41.0	9.2

다) 식품학 및 조리원리

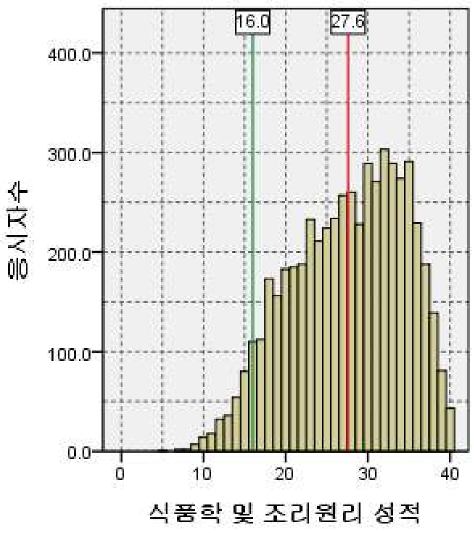

총점	과락선	평균성적	표준편차
40	16	27.6	6.8

라) 급식, 위생 및 관계법규

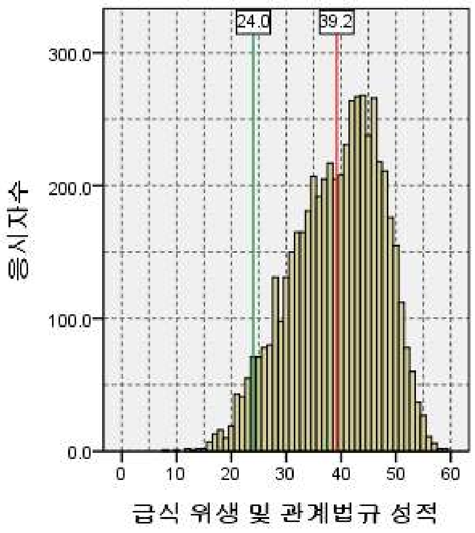

총점	과락선	평균성적	표준편차
60	24	39.2	8.2

2. 난이도와 변별도

1) 전체 난이도와 변별도

가) 전회 대비 전체 난이도와 변별도

회차	난이도		변별도1		변별도2	
	평균	표준편차	평균	표준편차	평균	표준편차
제42회	67.6	19.6	.34	.17	.30	.13
제43회	61.1	20.1	.35	.17	.30	.13
제44회	67.1	20.3	.33	.17	.30	.12
제45회	69.4	19.2	.32	.18	.30	.13
**제46회**	**67.0**	**17.9**	**.37**	**.17**	**.33**	**.12**

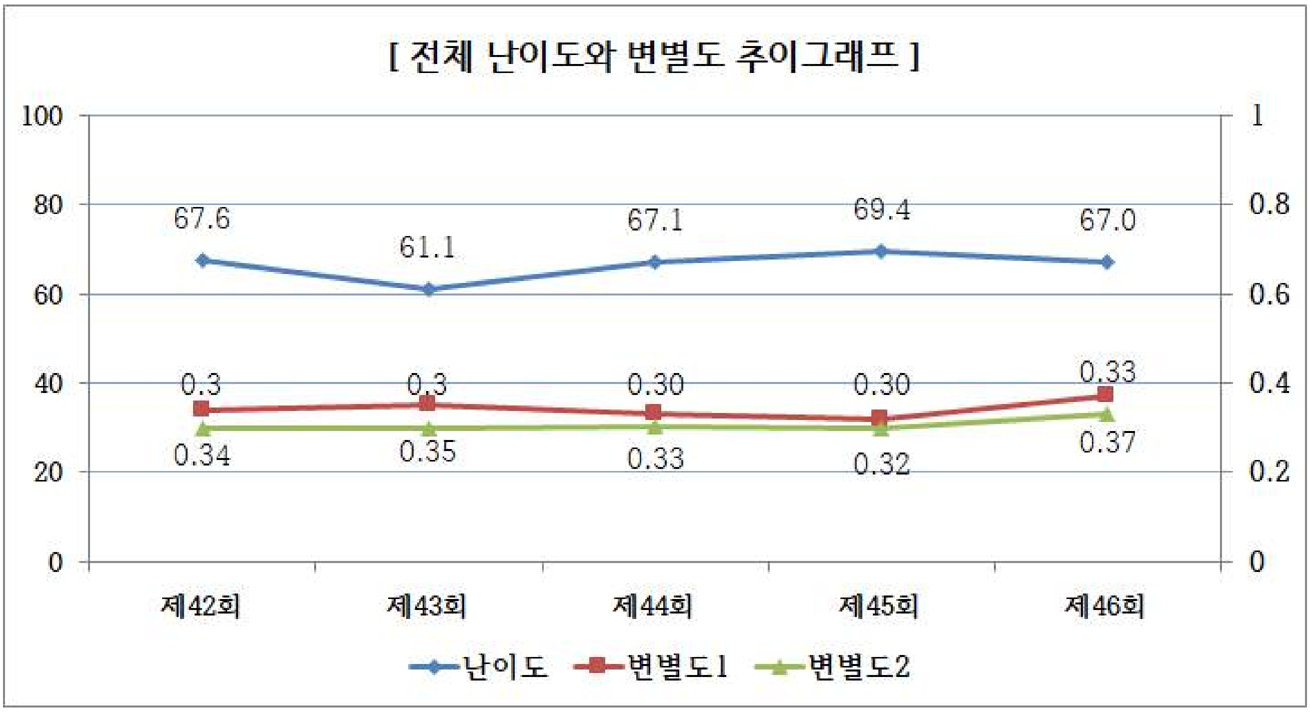

해석

- 전년 대비 난이도 지수는 2.4 감소함
- 변별도 1 지수는 .05 증가, 변별도 2 지수는 .03 증가함

나) 전체 난이도와 변별도 분포도 및 비율분석

(1) 전체 난이도 분포도 및 비율분석

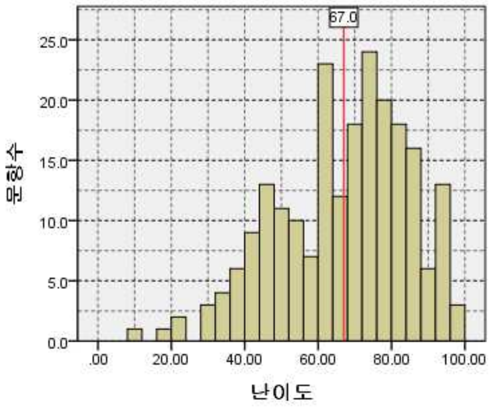

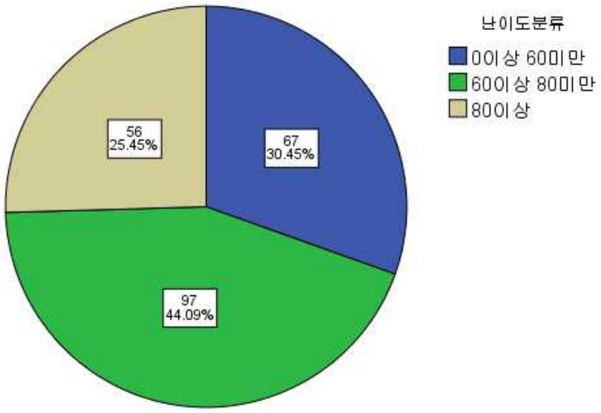

총점	난이도	표준편차
220	67.0	17.9

난이도	문항수	비율(%)
0~60미만	67	30.5
60~80미만	97	44.1
80~100	56	25.5
전체	220	100.0

(2) 전체 변별도1 분포도 및 비율분석

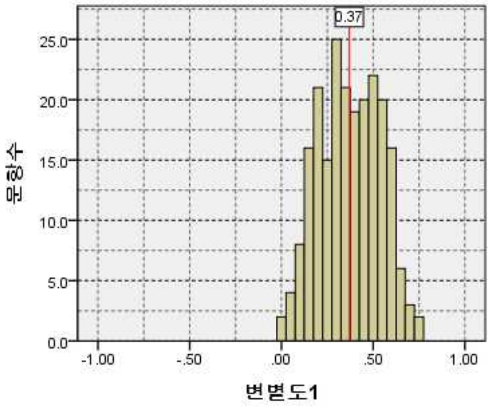

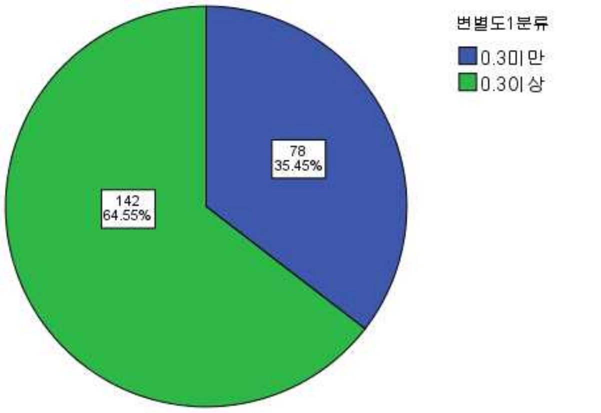

총점	변별도1	표준편차
220	.37	.17

변별도1	문항수	비율(%)
0.3미만	78	35.5
0.3이상	142	64.5
전체	220	100.0

(3) 전체 변별도2 분포도 및 비율분석

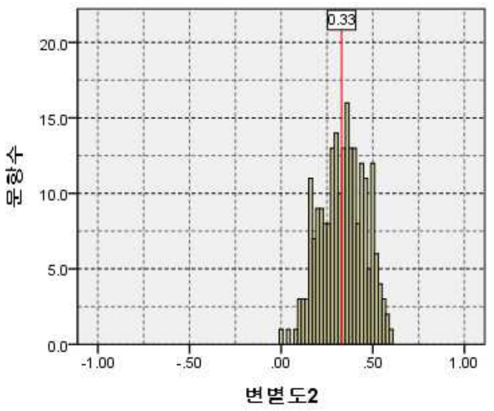

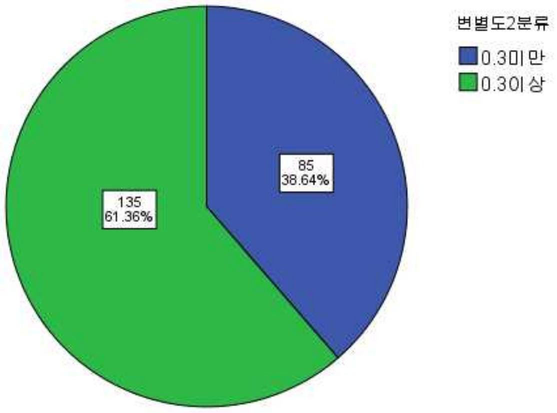

총점	변별도2	표준편차
220	.33	.12

변별도2	문항수	비율(%)
0.3미만	85	38.6
0.3이상	135	61.4
전체	220	100.0

해석

- 난이도 지수가 60 이상 80 미만인 문항이 97 문항으로 가장 많았으며, 60 미만인 문항이 67 문항, 80 이상인 문항이 56 문항으로 나타남
- 변별도 1 지수를 기준으로 분류하였을 때, 0.3 미만인 문항이 78 문항으로 0.3 이상인 문항이 142 문항인 것에 비해 더 적게 나타남
- 변별도 2 지수를 기준으로 분류하였을 때, 0.3 미만인 문항이 85 문항으로 0.3 이상인 문항이 135 문항인 것에 비해 더 적게 나타남

2) 과목별 난이도와 변별도

가) 전회 대비 과목별 난이도와 변별도

(1) 전회 대비 영양학 및 생화학 난이도와 변별도

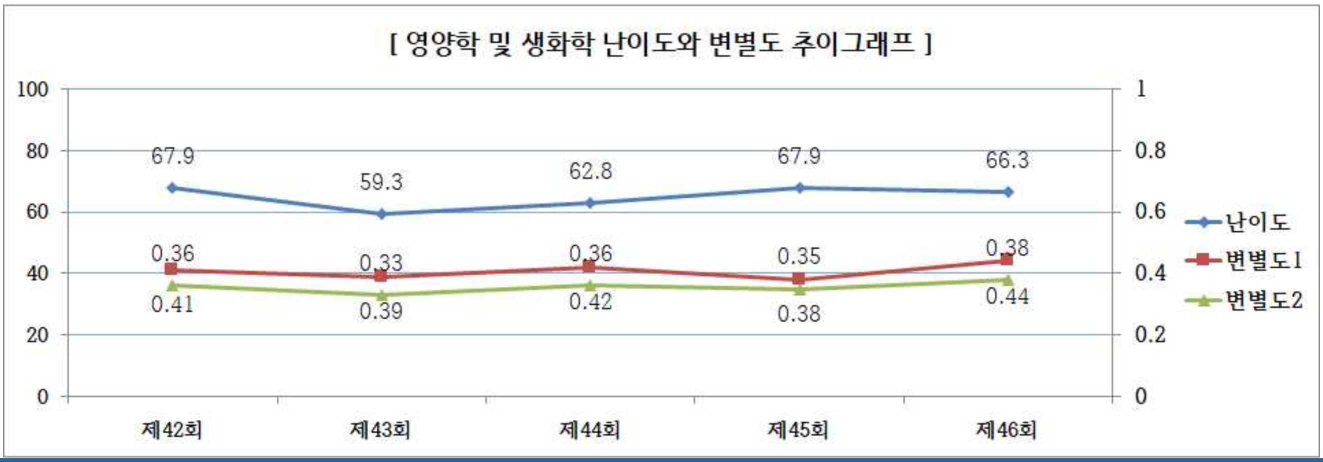

회차	난이도		변별도1		변별도2	
	평균	표준편차	평균	표준편차	평균	표준편차
제42회	67.9	19.2	.41	.19	.36	.13
제43회	59.3	19.1	.39	.17	.33	.14
제44회	62.8	18.3	.42	.16	.36	.12
제45회	67.9	18.3	.38	.15	.35	.11
**제46회**	**66.3**	**14.6**	**.44**	**.16**	**.38**	**.12**

(2) 전회 대비 영양교육, 식사요법 및 생리학 난이도와 변별도

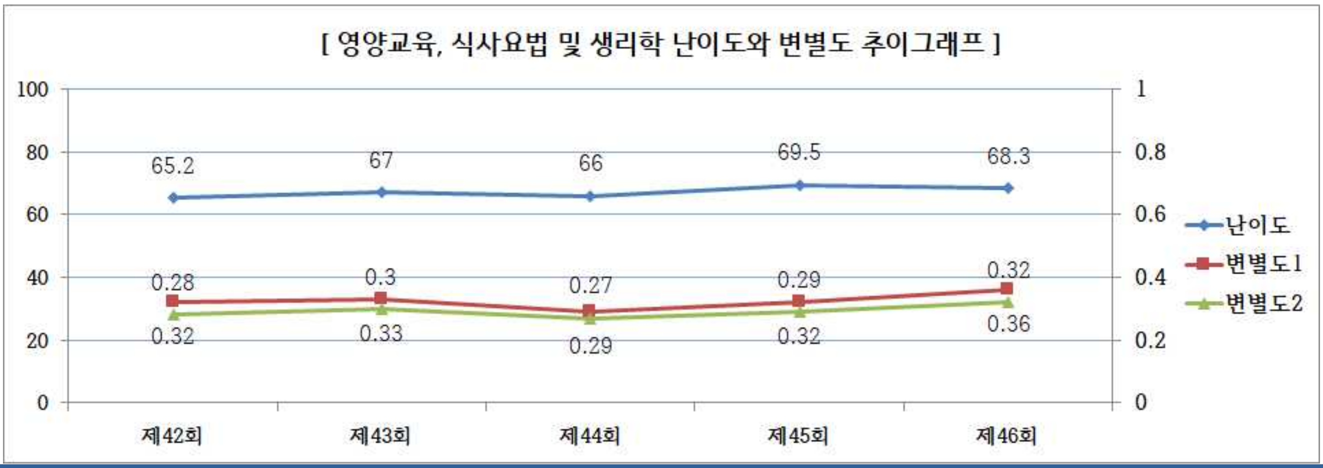

회차	난이도		변별도1		변별도2	
	평균	표준편차	평균	표준편차	평균	표준편차
제42회	65.2	21.3	.32	.17	.28	.12
제43회	67.0	18.2	.33	.15	.30	.12
제44회	66.0	21.5	.29	.16	.27	.12
제45회	69.5	18.2	.32	.19	.29	.14
**제46회**	**68.3**	**18.5**	**.36**	**.17**	**.32**	**.12**

(3) 전회 대비 식품학 및 조리원리 난이도와 변별도

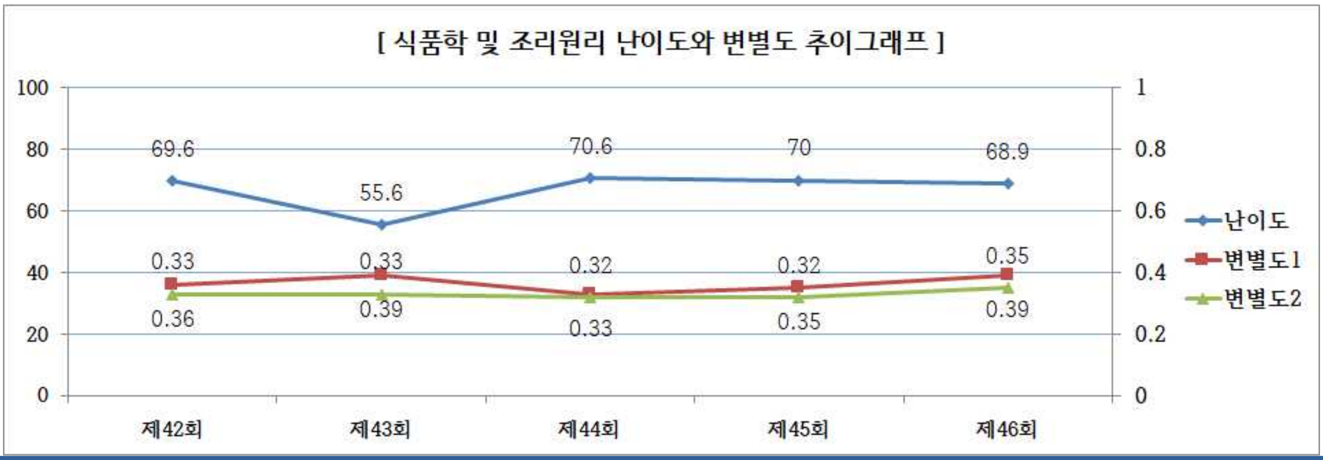

회차	난이도		변별도1		변별도2	
	평균	표준편차	평균	표준편차	평균	표준편차
제42회	69.6	18.8	.36	.16	.33	.11
제43회	55.6	20.0	.39	.16	.33	.12
제44회	70.6	18.1	.33	.14	.32	.10
제45회	70.0	17.1	.35	.17	.32	.12
**제46회**	**68.9**	**17.0**	**.39**	**.15**	**.35**	**.11**

(4) 전회 대비 급식, 위생 및 관계법규 난이도와 변별도

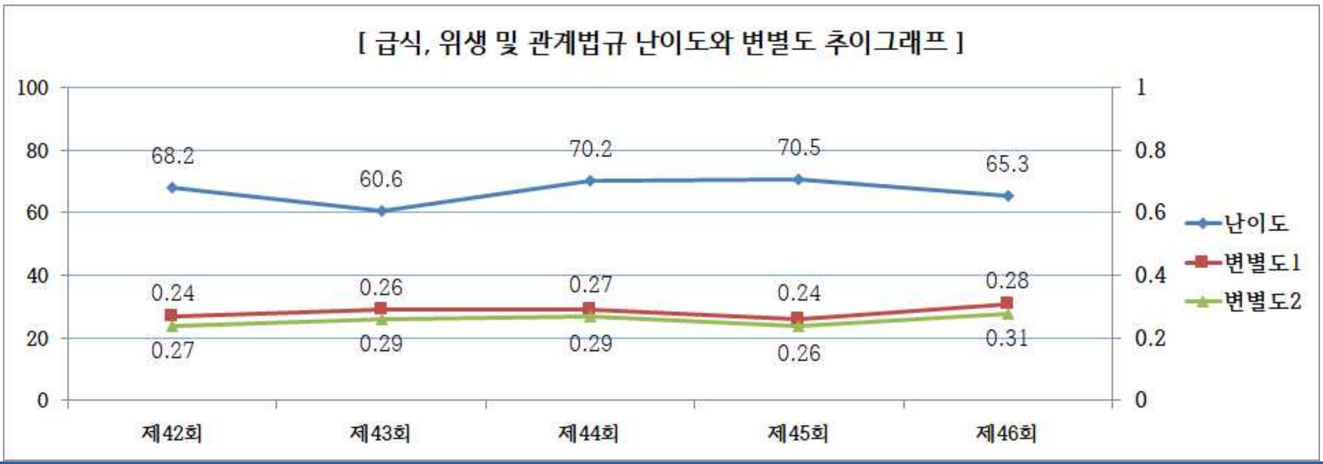

회차	난이도		변별도1		변별도2	
	평균	표준편차	평균	표준편차	평균	표준편차
제42회	68.2	18.4	.27	.13	.24	.10
제43회	60.6	21.4	.29	.16	.26	.13
제44회	70.2	21.3	.29	.17	.27	.12
제45회	70.5	22.4	.26	.17	.24	.13
**제46회**	**65.3**	**20.9**	**.31**	**.15**	**.28**	**.11**

해석

- 전회 대비 각 과목의 난이도 지수는 각각 1.6, 1.2, 1.1, 5.2 감소함
- 영양학 및 생화학 과목의 변별도1 지수와 변별도2 지수는 각각 .06, .03 증가함
- 영양교육, 식사요법 및 생리학 과목의 변별도1 지수와 변별도2 지수는 각각 .04, .03 증가함
- 식품학 및 조리원리 과목의 변별도1 지수와 변별도2 지수는 각각 .04, .03 증가함
- 급식, 위생 및 관계법규 과목의 변별도1 지수와 변별도2 지수는 각각 .05, .04 증가함

나) 과목별 난이도와 변별도 분포도 및 비율분석

(1) 영양학 및 생화학 난이도와 변별도 분포도 및 비율분석

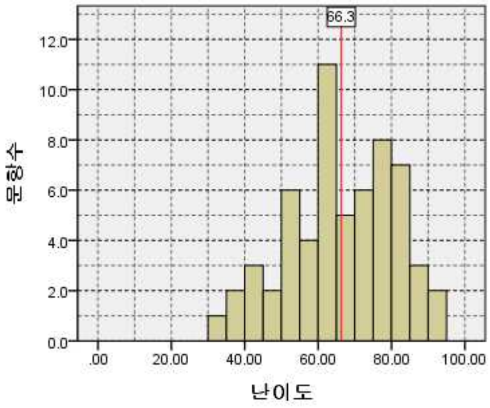

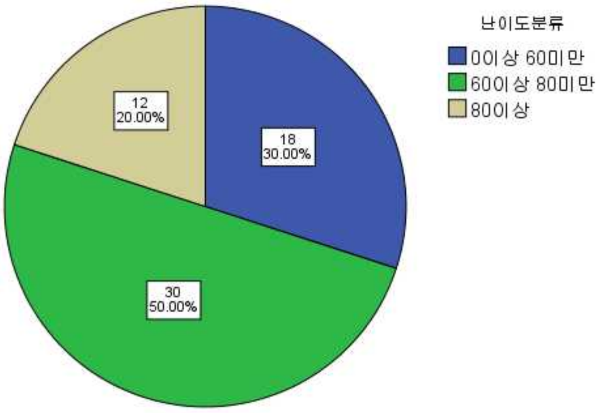

총점	난이도	표준편차
60	66.3	14.6

난이도	문항수	비율(%)
0~60미만	18	30.0
60~80미만	30	50.0
80~100	12	20.0
전체	60	100.0

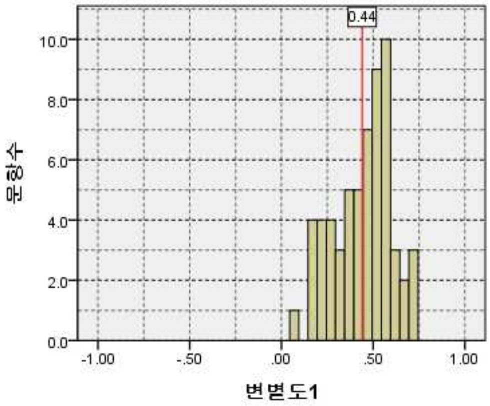

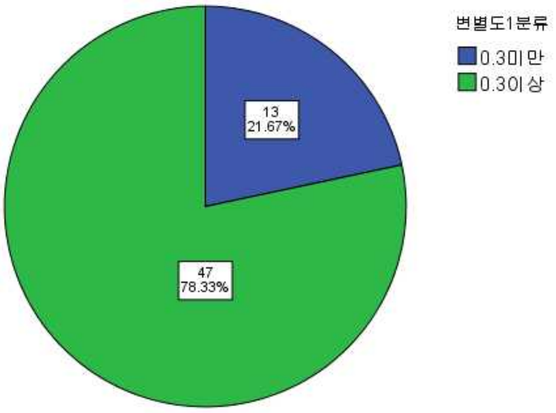

총점	변별도1	표준편차
60	.44	.16

변별도1	문항수	비율(%)
0.3미만	13	21.7
0.3이상	47	78.3
전체	60	100.0

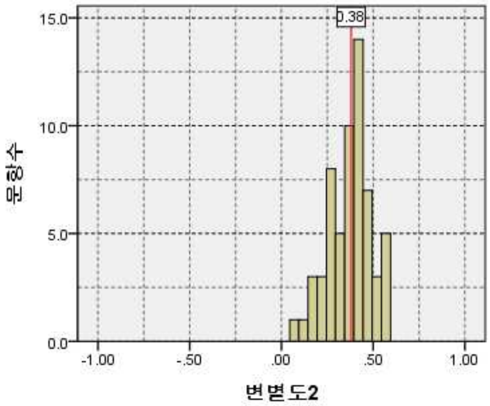

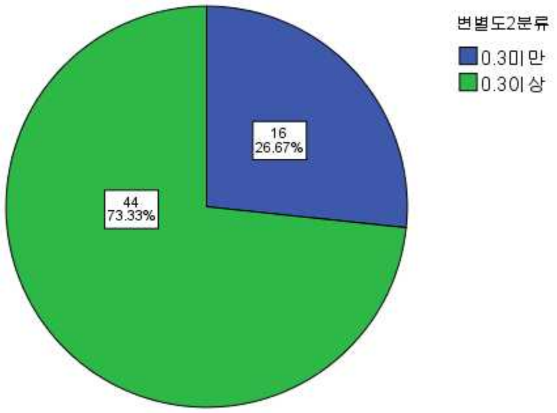

총점	변별도2	표준편차	변별도2	문항수	비율(%)
60	.38	.12	0.3미만	16	26.7
			0.3이상	44	73.3
			전체	60	100.0

해석

- 영양학 및 생화학 과목에서 난이도 지수가 60 이상 80 미만인 문항이 30 문항으로 가장 많았으며, 60 미만인 문항이 18 문항, 80 이상인 문항이 12 문항으로 나타남
- 변별도 1 지수를 기준으로 분류하였을 때, 0.3 미만인 문항이 13 문항으로 0.3 이상인 문항이 47 문항인 것에 비해 더 적게 나타남
- 변별도 2 지수를 기준으로 분류하였을 때, 0.3 미만인 문항이 16 문항으로 0.3 이상인 문항이 44 문항인 것에 비해 더 적게 나타남

(2) 영양교육, 식사요법 및 생리학 난이도와 변별도 분포도 및 비율분석

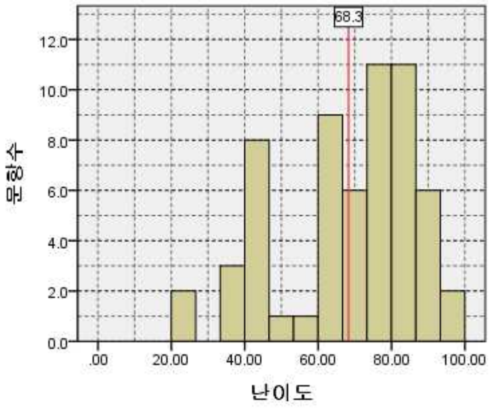

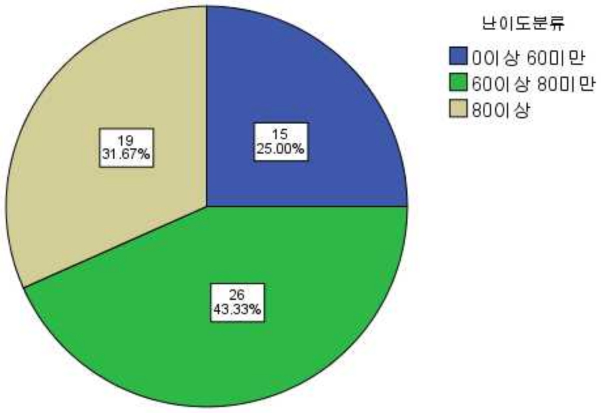

총점	난이도	표준편차
60	68.3	18.5

난이도	문항수	비율(%)
0~60미만	15	25.0
60~80미만	26	43.3
80~100	19	31.7
전체	60	100.0

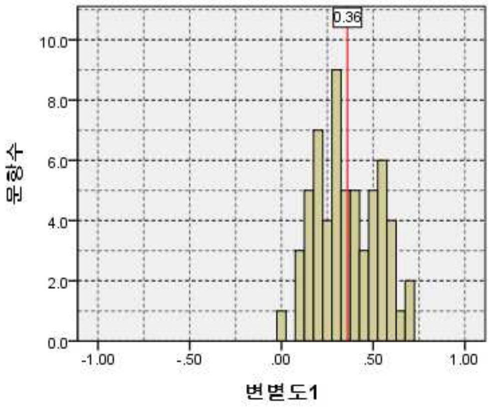

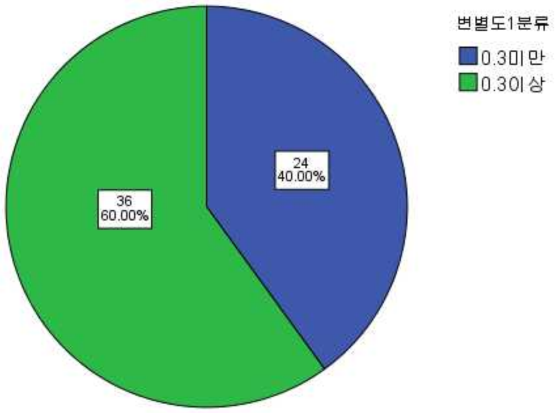

총점	변별도1	표준편차
60	.36	.17

변별도1	문항수	비율(%)
0.3미만	24	40.0
0.3이상	36	60.0
전체	60	100.0

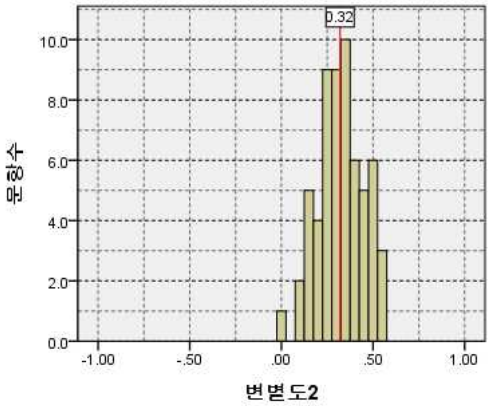

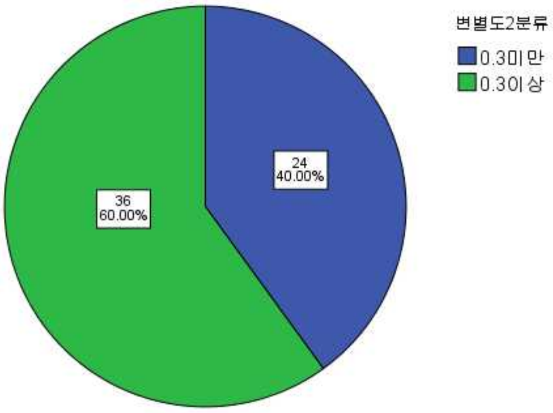

총점	변별도2	표준편차
60	.32	.12

변별도2	문항수	비율(%)
0.3미만	24	40.0
0.3이상	36	60.0
전체	60	100.0

해석

- 영양교육, 식사요법 및 생리학 과목에서 난이도 지수가 60 이상 80 미만인 문항이 26 문항으로 가장 많았으며, 80 이상인 문항이 19 문항, 60 미만인 문항이 15 문항으로 나타남
- 변별도 1 지수를 기준으로 분류하였을 때, 0.3 미만인 문항이 24 문항으로 0.3 이상인 문항이 36 문항인 것에 비해 더 적게 나타남
- 변별도 2 지수를 기준으로 분류하였을 때, 0.3 미만인 문항이 24 문항으로 0.3 이상인 문항이 36 문항인 것에 비해 더 적게 나타남

(3) 식품학 및 조리원리 난이도와 변별도 분포도 및 비율분석

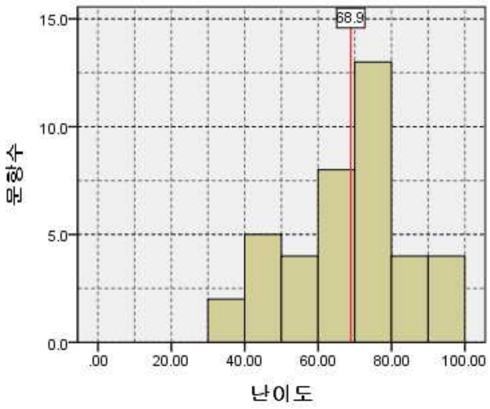

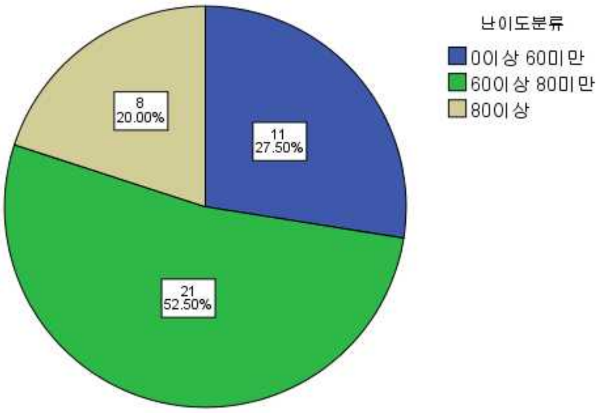

총점	난이도	표준편차	난이도	문항수	비율(%)
40	68.9	17.0	0~60미만	11	27.5
			60~80미만	21	52.5
			80~100	8	20.0
			전체	40	100.0

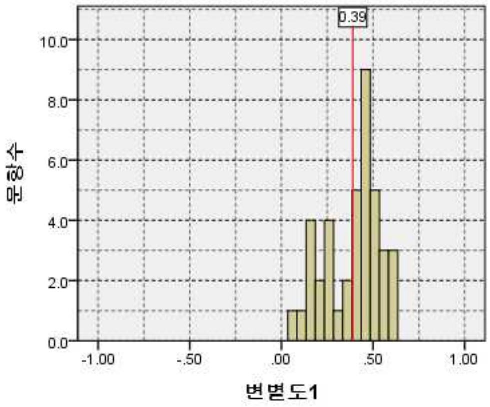

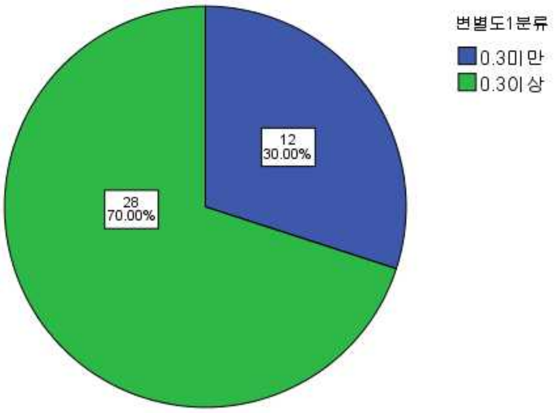

총점	변별도1	표준편차	변별도1	문항수	비율(%)
40	.39	.15	0.3미만	12	30.0
			0.3이상	28	70.0
			전체	40	100.0

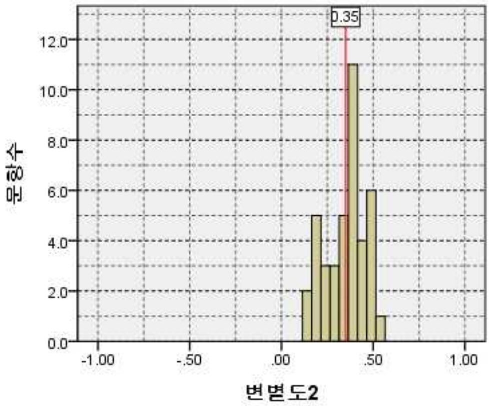

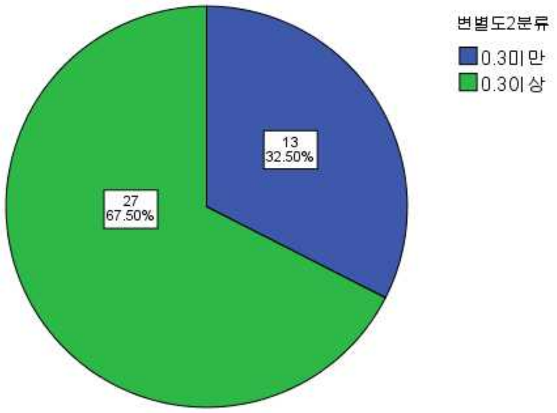

총점	변별도2	표준편차
40	.35	.11

변별도2	문항수	비율(%)
0.3미만	13	32.5
0.3이상	27	67.5
전체	40	100.0

해석

- 식품학 및 조리원리 과목에서 난이도 지수가 60 이상 80 미만인 문항이 21 문항으로 가장 많았으며, 60 미만인 문항이 11 문항, 80 이상인 문항이 8 문항으로 나타남
- 변별도 1 지수를 기준으로 분류하였을 때, 0.3 미만인 문항이 12 문항으로 0.3 이상인 문항이 28 문항인 것에 비해 더 적게 나타남
- 변별도 2 지수를 기준으로 분류하였을 때, 0.3 미만인 문항이 13 문항으로 0.3 이상인 문항이 27 문항인 것에 비해 더 적게 나타남

(4) 급식, 위생 및 관계법규 난이도와 변별도 분포도 및 비율분석

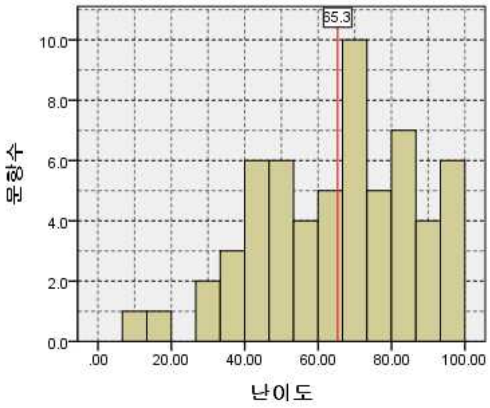

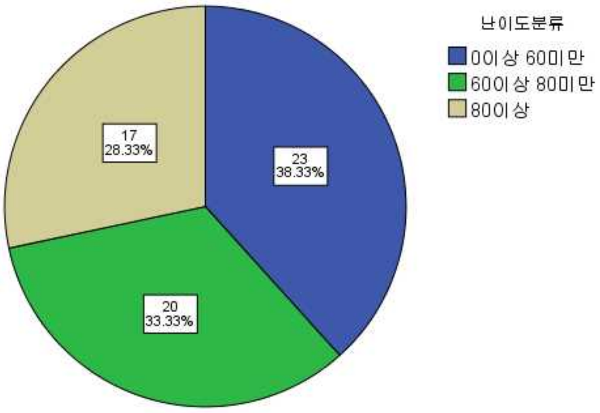

총점	난이도	표준편차
60	65.3	20.9

난이도	문항수	비율(%)
0~60미만	23	38.3
60~80미만	20	33.3
80~100	17	28.3
전체	60	100.0

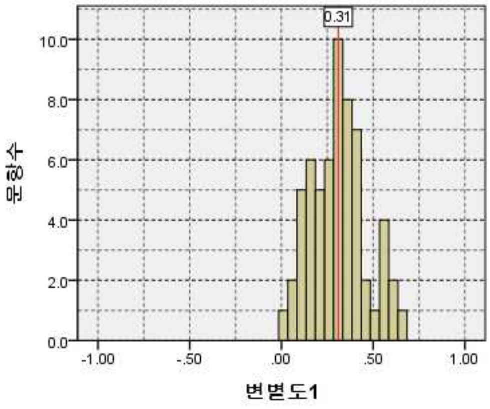

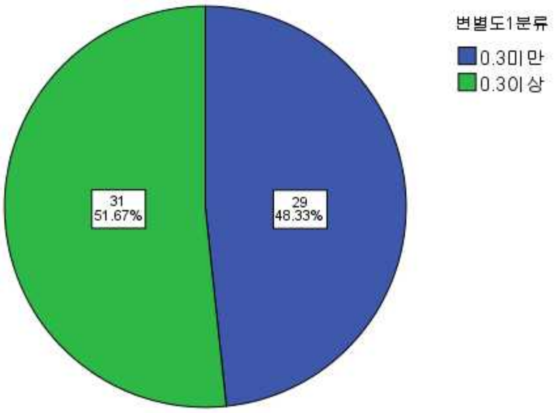

총점	변별도1	표준편차
60	.31	.15

변별도1	문항수	비율(%)
0.3미만	29	48.3
0.3이상	31	51.7
전체	60	100.0

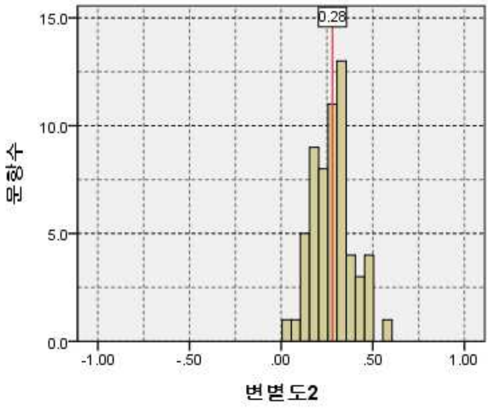

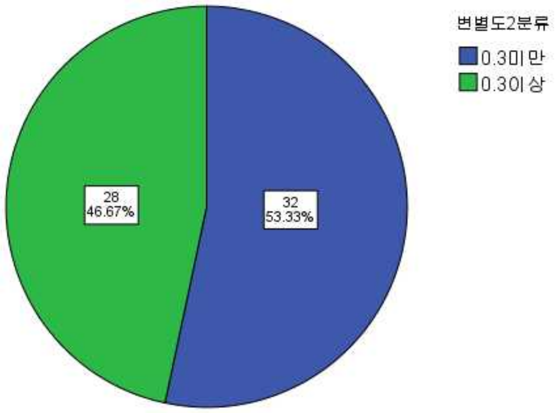

총점	변별도2	표준편차
60	.28	.11

변별도2	문항수	비율(%)
0.3미만	32	53.3
0.3이상	28	46.7
전체	60	100.0

해석

- 급식, 위생 및 관계법규 과목에서 난이도 지수가 60 미만인 문항이 23 문항으로 가장 많았으며, 60 이상 80 미만인 문항이 20 문항, 80 이상인 문항이 17 문항으로 나타남
- 변별도 1 지수를 기준으로 분류하였을 때, 0.3 미만인 문항이 29 문항으로 0.3 이상인 문항이 31 문항인 것에 비해 더 적게 나타남
- 변별도 2 지수를 기준으로 분류하였을 때, 0.3 미만인 문항이 32 문항으로 0.3 이상인 문항이 28 문항인 것에 비해 더 많이 나타남

3) 지식수준별 난이도와 변별도

가) 전회 대비 지식수준별 난이도와 변별도

(1) 전회 대비 암기형 난이도와 변별도

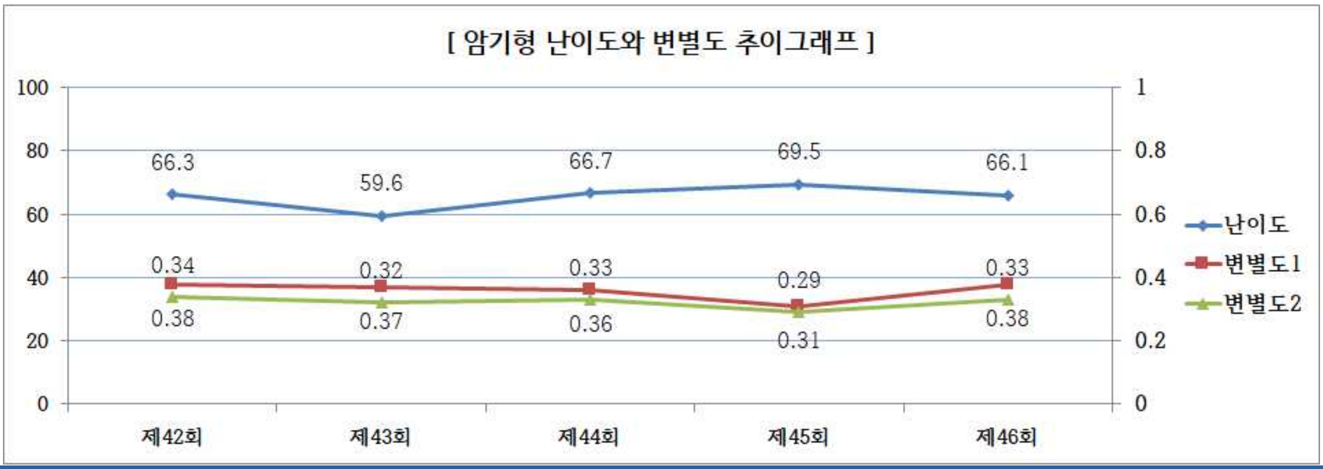

회차	난이도		변별도1		변별도2	
	평균	표준편차	평균	표준편차	평균	표준편차
제42회	66.3	18.3	.38	.18	.34	.14
제43회	59.6	19.6	.37	.17	.32	.14
제44회	66.7	18.7	.36	.18	.33	.13
제45회	69.5	20.2	.31	.16	.29	.12
**제46회**	**66.1**	**18.4**	**.38**	**.17**	**.33**	**.13**

(2) 전회 대비 해석형 난이도와 변별도

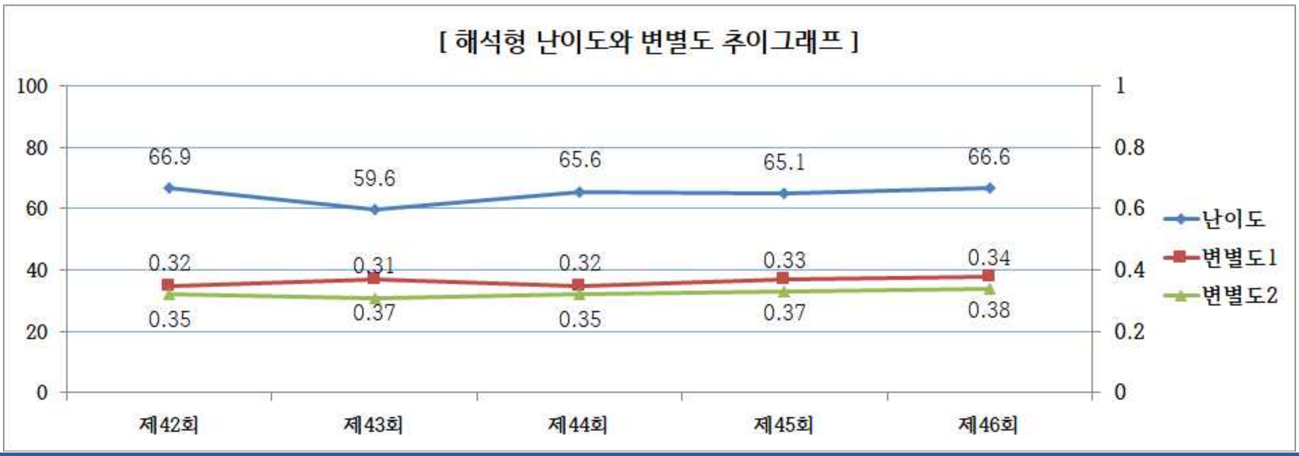

회차	난이도		변별도1		변별도2	
	평균	표준편차	평균	표준편차	평균	표준편차
제42회	66.9	21.1	.35	.17	.32	.12
제43회	59.6	18.7	.37	.17	.31	.13
제44회	65.6	21.7	.35	.16	.32	.11
제45회	65.1	19.6	.37	.18	.33	.13
**제46회**	**66.6**	**18.2**	**.38**	**.18**	**.34**	**.13**

(3) 전회 대비 해결형 난이도와 변별도

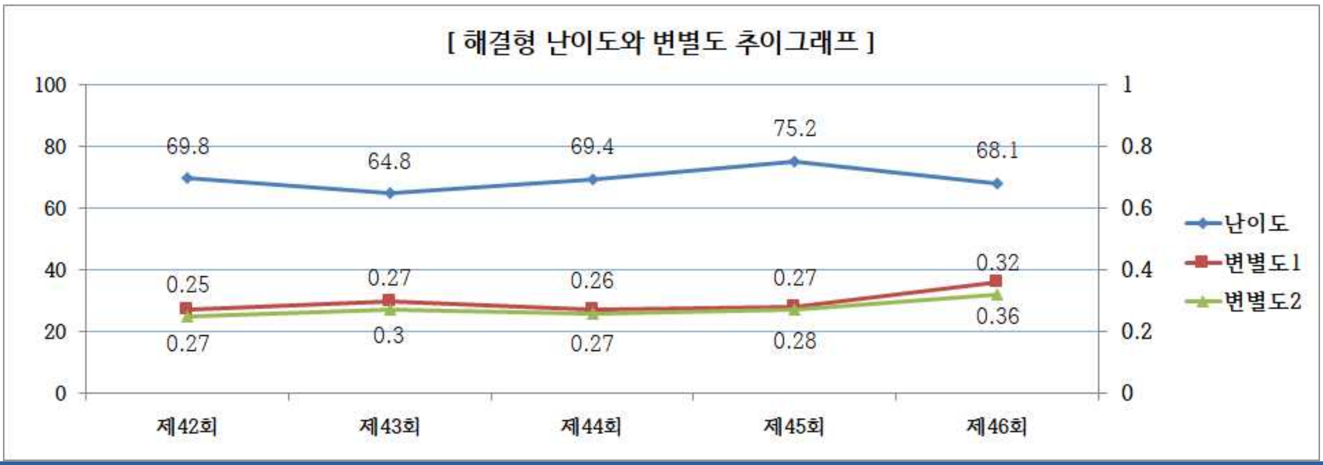

회차	난이도		변별도1		변별도2	
	평균	표준편차	평균	표준편차	평균	표준편차
제42회	69.8	18.8	.27	.14	.25	.10
제43회	64.8	21.9	.30	.14	.27	.11
제44회	69.4	19.9	.27	.17	.26	.12
제45회	75.2	15.9	.28	.17	.27	.14
**제46회**	**68.1**	**17.4**	**.36**	**.15**	**.32**	**.11**

해석

- 전회 대비 암기형과 해결형 문항의 난이도 지수는 각각 3.4, 7.1 감소였으며, 해석형 문항의 난이도 지수는 1.5 증가함
- 암기형 문항의 변별도 1 지수와 변별도 2 지수는 각각 .07, .04 증가함
- 해석형 문항의 변별도 1 지수와 변별도 2 지수는 각각 .01 씩 증가함
- 해결형 문항의 변별도 1 지수와 변별도 2 지수는 각각 .08, .05 증가함

나) 지식수준별 난이도와 변별도 분포도 및 비율분석

(1) 암기형 난이도와 변별도 분포도 및 비율분석

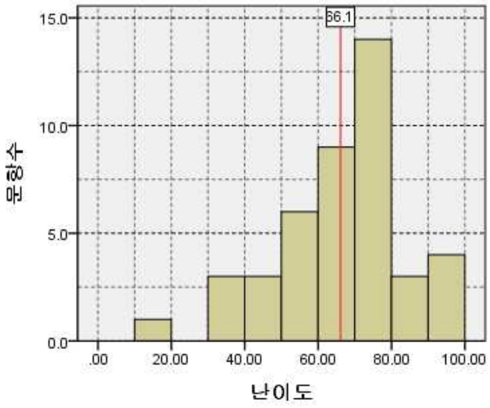

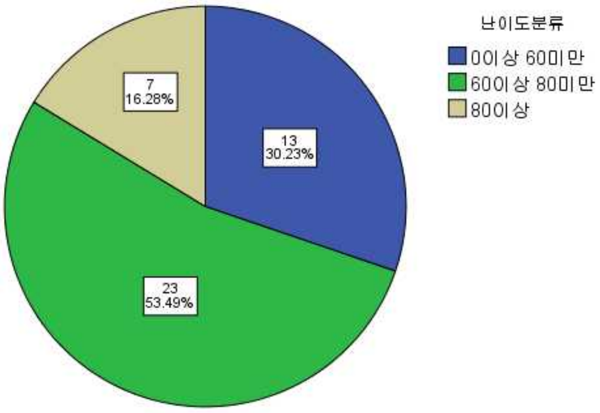

총점	난이도	표준편차
43	66.1	18.4

난이도	문항수	비율(%)
0~60미만	13	30.2
60~80미만	23	53.5
80~100	7	16.3
전체	43	100.0

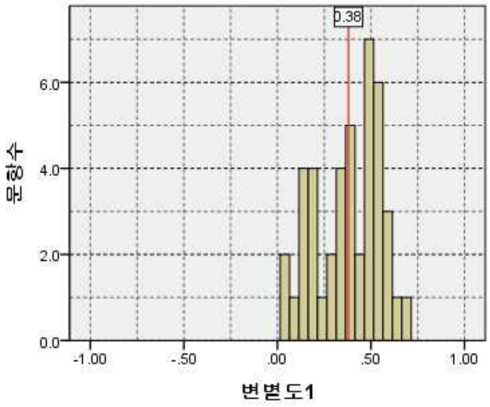

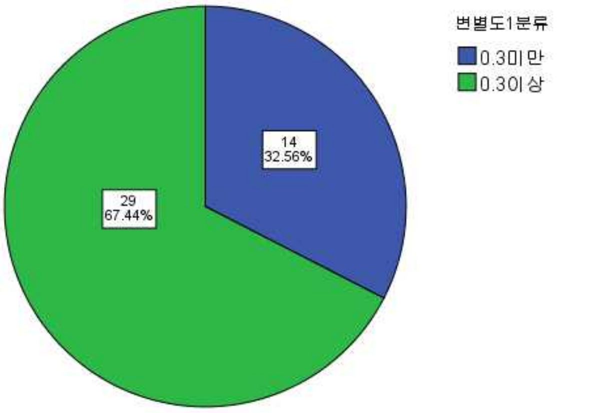

총점	변별도1	표준편차
43	.38	.17

변별도1	문항수	비율(%)
0.3미만	14	32.6
0.3이상	29	67.4
전체	43	100.0

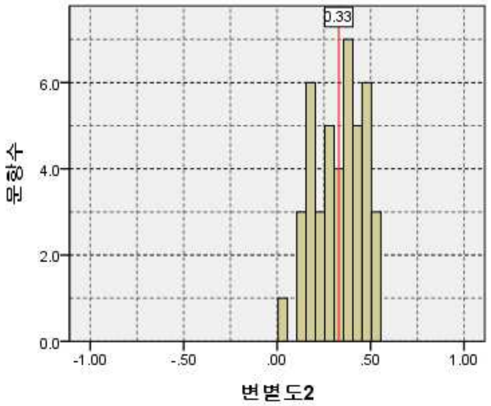

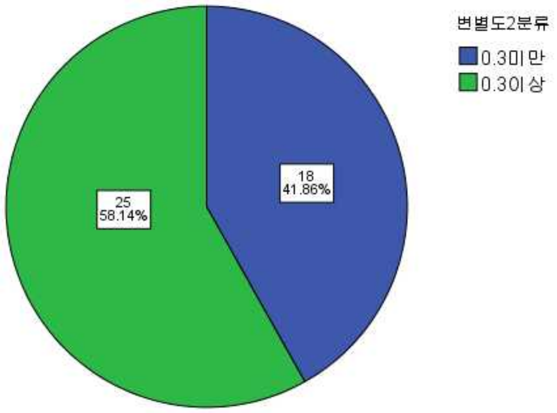

총점	변별도2	표준편차
43	.33	.13

변별도2	문항수	비율(%)
0.3미만	18	41.9
0.3이상	25	58.1
전체	43	100.0

해석

- 암기형 문항에서 난이도 지수가 60 이상 80 미만인 문항이 23 문항으로 가장 많았으며, 60 미만인 문항이 13 문항, 80 이상인 문항이 7 문항으로 나타남
- 변별도 1 지수를 기준으로 분류하였을 때, 0.3 미만인 문항이 14 문항으로 0.3 이상인 문항이 29 문항인 것에 비해 더 적게 나타남
- 변별도 2 지수를 기준으로 분류하였을 때, 0.3 미만인 문항이 18 문항으로 0.3 이상인 문항이 25 문항인 것에 비해 더 적게 나타남

(2) 해석형 난이도와 변별도 분포도 및 비율분석

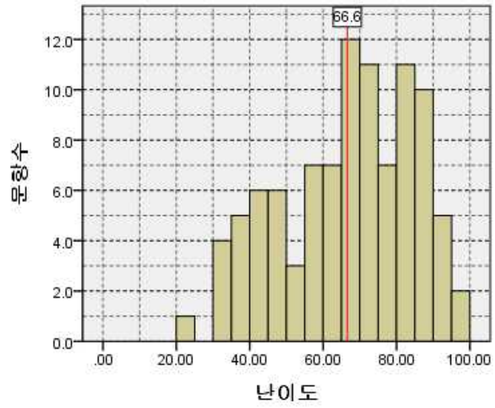

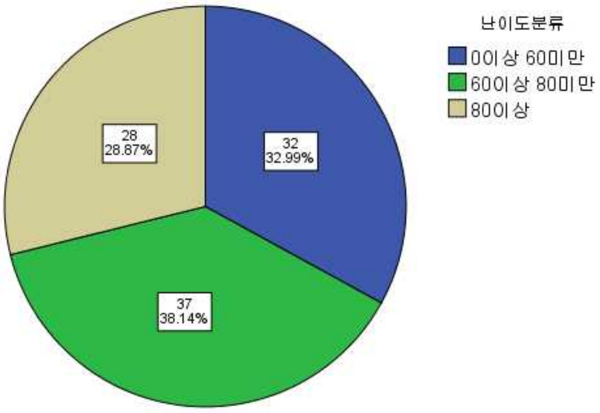

총점	난이도	표준편차
97	66.6	18.2

난이도	문항수	비율(%)
0~60미만	32	33.0
60~80미만	37	38.1
80~100	28	28.9
전체	97	100.0

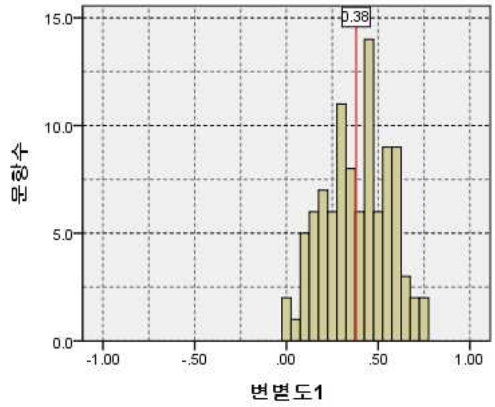

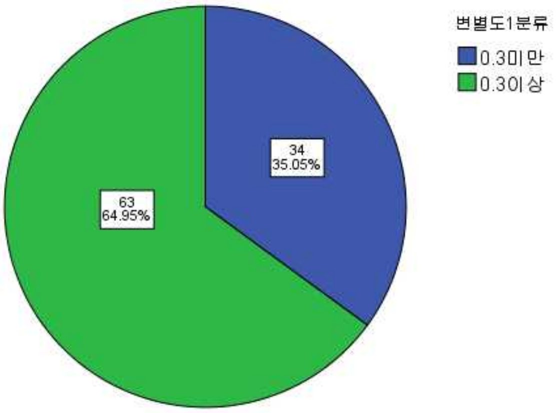

총점	변별도1	표준편차
97	.38	.18

변별도1	문항수	비율(%)
0.3미만	34	35.1
0.3이상	63	64.9
전체	97	100.0

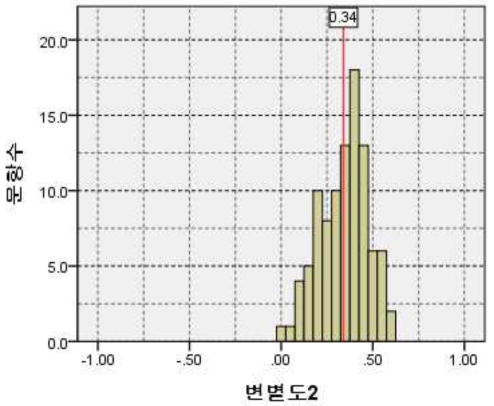

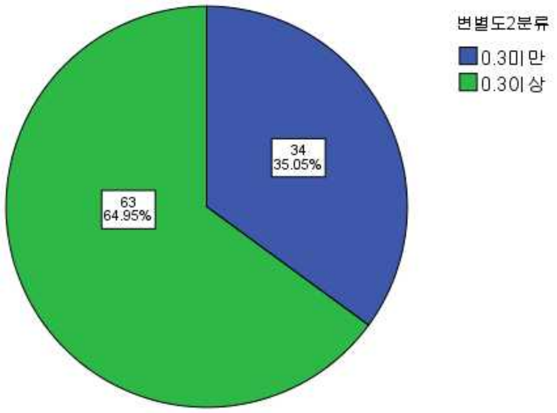

총점	변별도2	표준편차	변별도2	문항수	비율(%)
97	.34	.13	0.3미만	34	35.1
			0.3이상	63	64.9
			전체	97	100.0

해석

- 해석형 문항에서 난이도 지수가 60 이상 80 미만인 문항이 37 문항으로 가장 많았으며, 60 미만인 문항이 32 문항, 80 이상인 문항이 28 문항으로 나타남
- 변별도 1 지수를 기준으로 분류하였을 때, 0.3 미만인 문항이 34 문항으로 0.3 이상인 문항이 63 문항인 것에 비해 더 적게 나타남
- 변별도 2 지수를 기준으로 분류하였을 때, 0.3 미만인 문항이 34 문항으로 0.3 이상인 문항이 63 문항인 것에 비해 더 적게 나타남

(3) 해결형 난이도와 변별도 분포도 및 비율분석

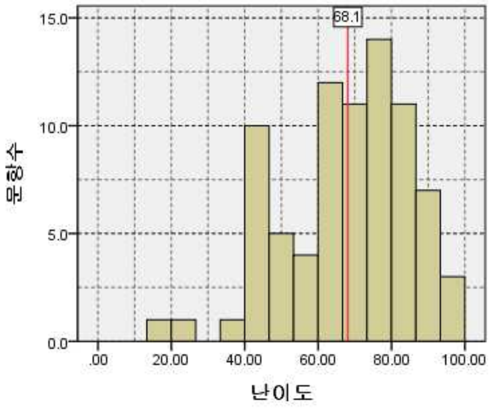

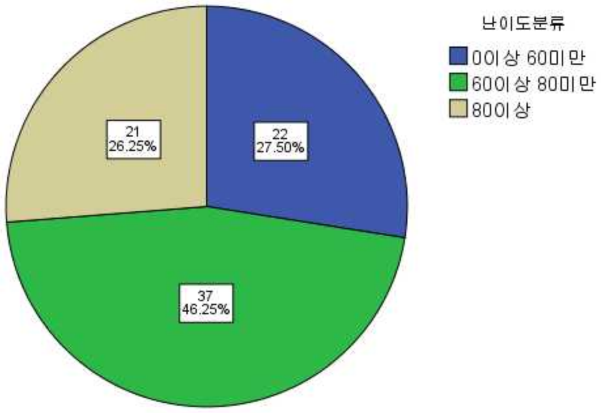

총점	난이도	표준편차
80	68.1	17.4

난이도	문항수	비율(%)
0~60미만	22	27.5
60~80미만	37	46.3
80~100	21	26.3
전체	80	100.0

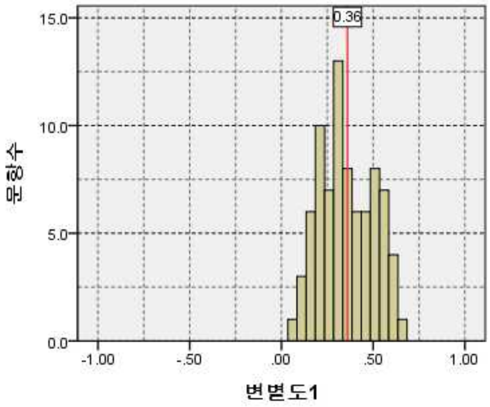

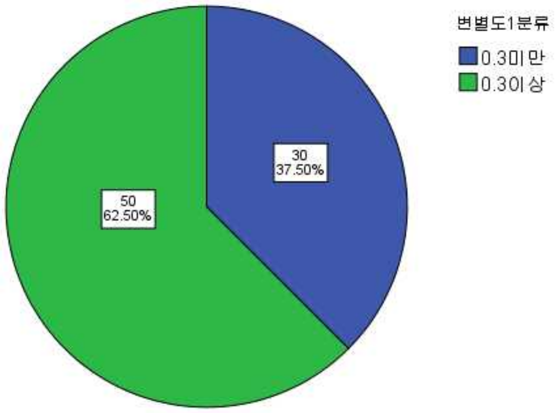

총점	변별도1	표준편차
80	.36	.15

변별도1	문항수	비율(%)
0.3미만	30	37.5
0.3이상	50	62.5
전체	80	100.0

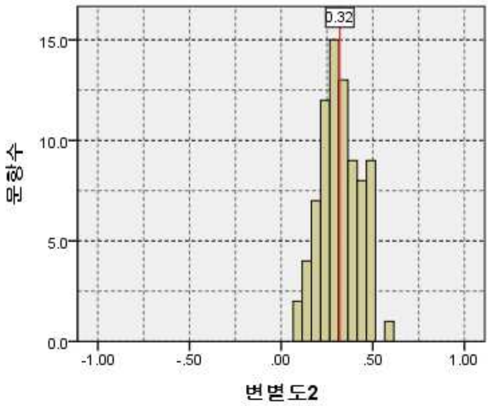

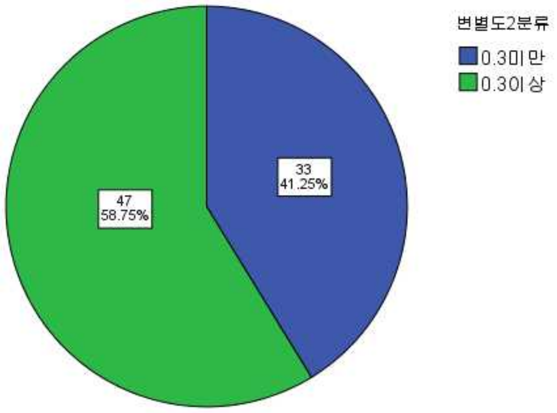

총점	변별도2	표준편차
80	.32	.11

변별도2	문항수	비율(%)
0.3미만	33	41.3
0.3이상	47	58.8
전체	80	100.0

해석

- 해결형 문항에서 난이도 지수가 60 이상 80 미만인 문항이 37 문항으로 가장 많았으며, 60 미만인 문항이 22 문항, 80 이상인 문항이 21 문항으로 나타남
- 변별도 1 지수를 기준으로 분류하였을 때, 0.3 미만인 문항이 30 문항으로 0.3 이상인 문항이 50 문항인 것에 비해 더 적게 나타남
- 변별도 2 지수를 기준으로 분류하였을 때, 0.3 미만인 문항이 33 문항으로 0.3 이상인 문항이 47 문항인 것에 비해 더 적게 나타남

3. 난이도와 변별도 간 산포도

1) 전체 난이도와 변별도 간 산포도

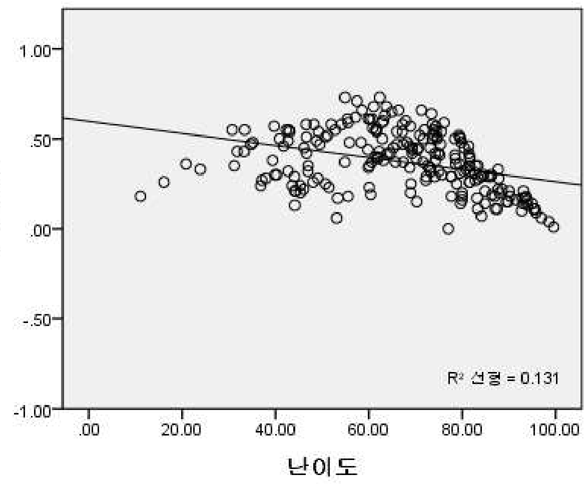

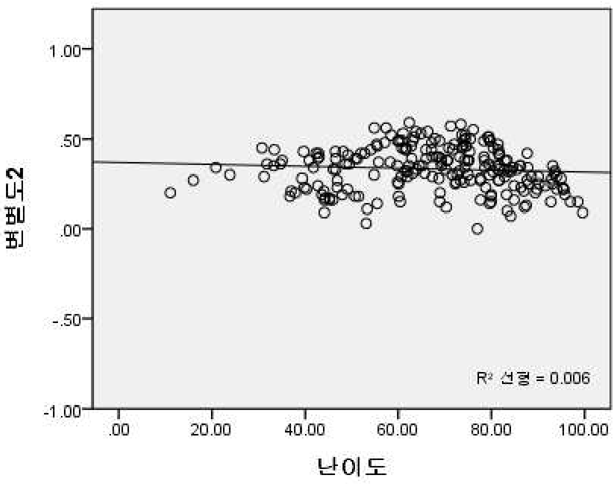

해석

- 전체 문항을 대상으로 난이도와 변별도 1 지수 간 상관은 -.362**로 난이도 지수가 높을수록 변별력이 낮아지는 것으로 나타남
- 난이도와 변별도 2 지수간 상관은 -.079 로 문항 난이도와 변별도 간 관련성이 없는 것으로 나타남

2) 과목별 난이도와 변별도 간 산포도

가) 영양학 및 생화학 난이도와 변별도 간 산포도

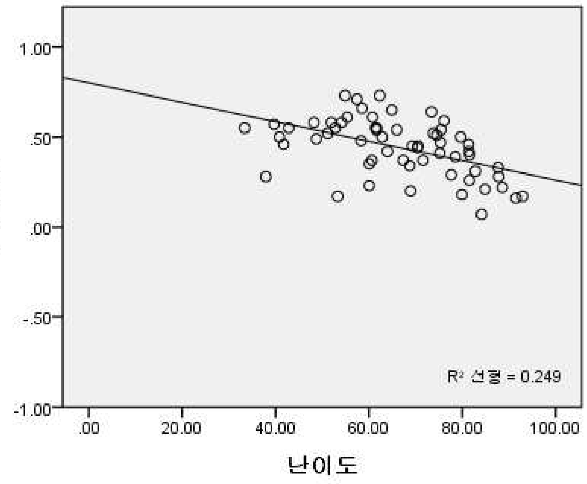

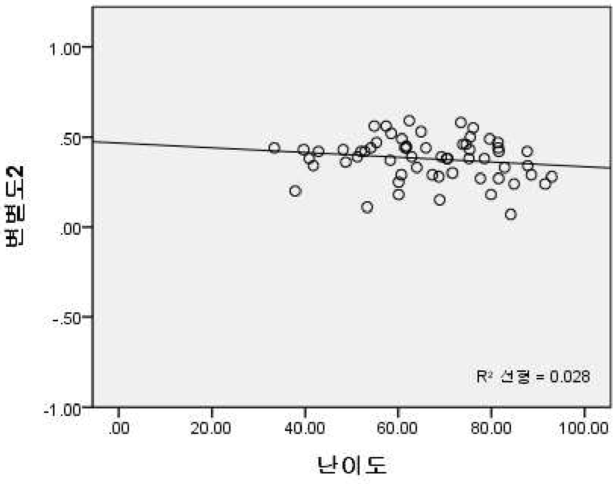

해석

- 영양학 및 생화학 과목을 대상으로 난이도와 변별도 1 지수 간 상관은 -.499**로 난이도 지수가 높을수록 변별력이 낮아지는 것으로 나타남
- 난이도와 변별도 2 지수 간 상관은 -.166 로 문항 난이도와 변별도 간 관련성이 없는 것으로 나타남

나) 영양교육, 식사요법 및 생리학 난이도와 변별도 간 산포도

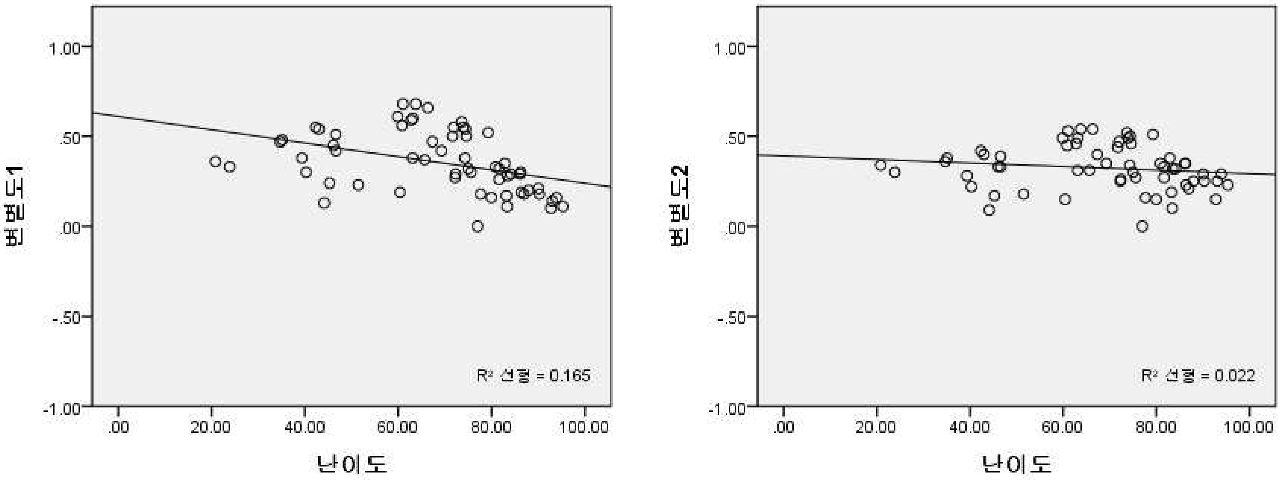

해석

- 영양교육, 식사요법 및 생리학 과목을 대상으로 난이도와 변별도 1 지수 간 상관은 -.407**로 난이도 지수가 높을수록 변별력이 낮아지는 것으로 나타남
- 난이도와 변별도 2 지수 간 상관은 -.148 으로 문항 난이도와 변별도 간 관련성이 없는 것으로 나타남

다) 식품학 및 조리원리 난이도와 변별도 간 산포도

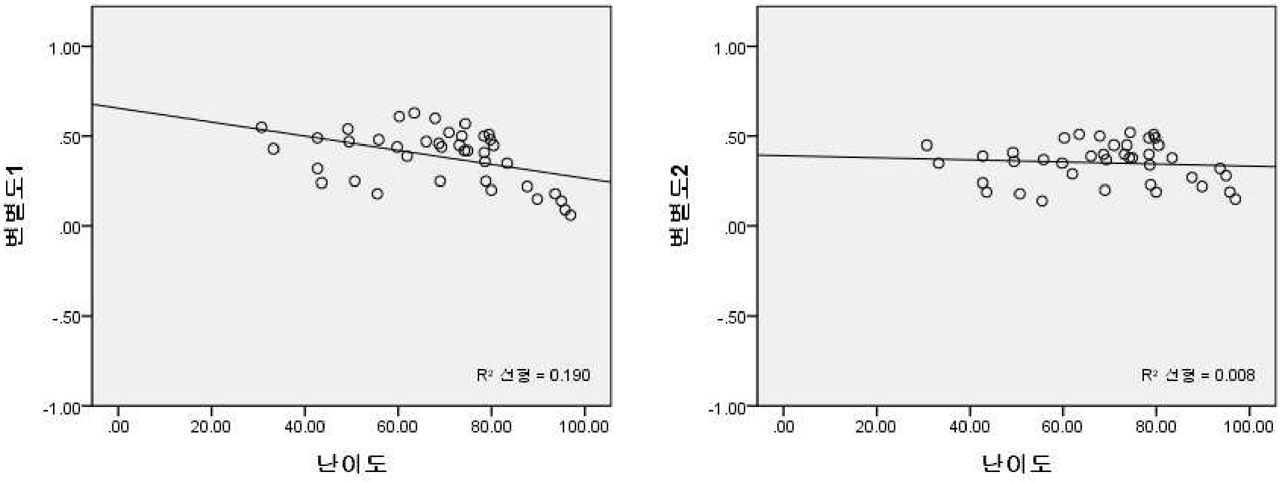

해석

- 식품학 및 조리원리 과목을 대상으로 난이도와 변별도 1 지수 간 상관은 -.436**로 난이도 지수가 높을수록 변별력이 낮아지는 것으로 나타남
- 난이도와 변별도 2 지수 간 상관은 -.089 로 문항 난이도와 변별력 간 관련성이 적은 것으로 나타남

라) 급식, 위생 및 관계법규 난이도와 변별도 간 산포도

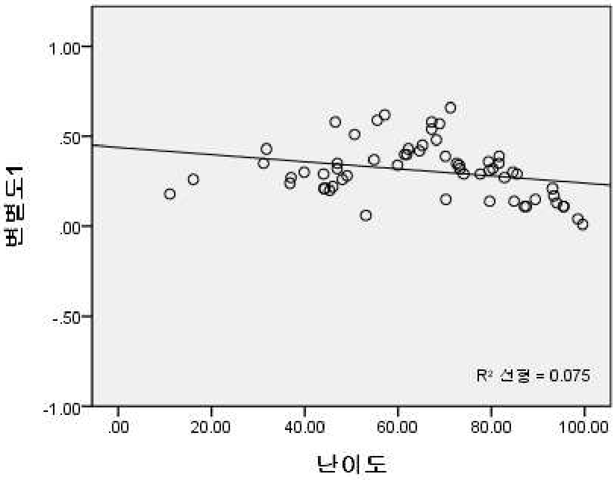

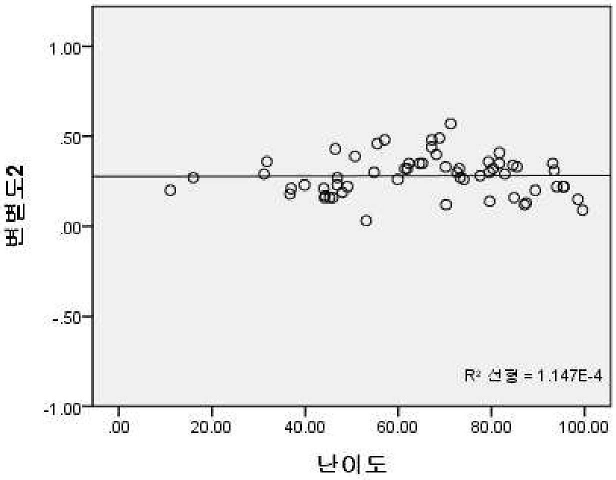

해석

- 급식, 위생 및 관계법규 과목을 대상으로 난이도와 변별도 1 지수 간 상관은 -.275*로 난이도 지수가 높을수록 변별력이 낮아지는 것으로 나타남
- 난이도와 변별도 2 지수 간 상관은 .011 로 문항 난이도와 변별력 간 관련성이 적은 것으로 나타남

4. 신뢰도 분석

과목명	문항수	제42회	제43회	제44회	제45회	제46회
전체	220	.959	.959	.959	.958	.966
영양학 및 생화학	60	.913	.892	.906	.899	.916
영양교육, 식사요법 및 생리학	60	.853	.868	.842	.860	.886
식품학 및 조리원리	40	.848	.850	.833	.832	.863
급식, 위생 및 관계법규	60	.806	.832	.847	.816	.851

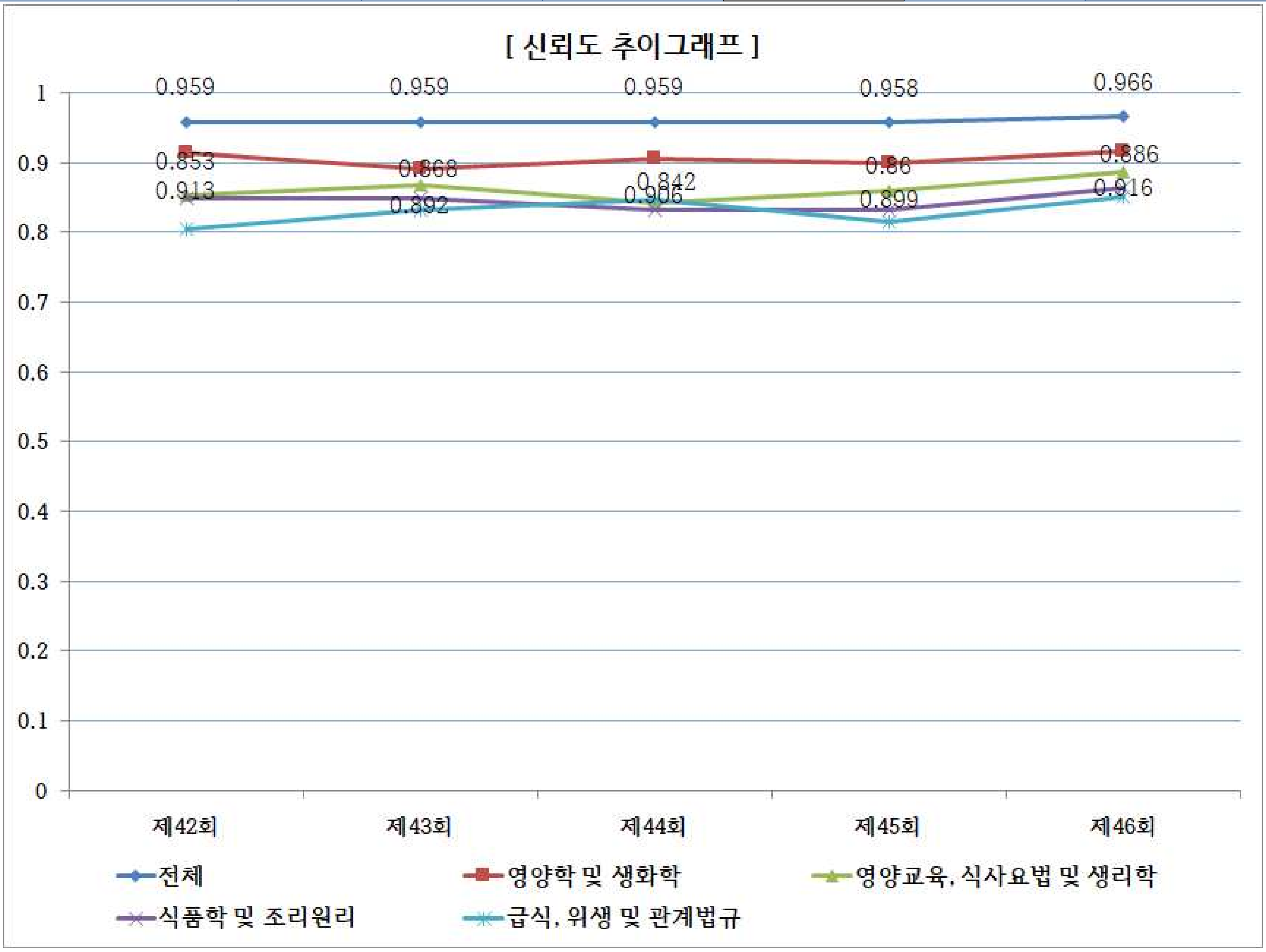

해석

- 영양사 국가시험 전체 및 각 과목의 문항 모두 일관되게 해당 영역을 측정하고 있는 것으로 나타남
- 전회 대비 신뢰도를 보았을 때 전체 문항에 대한 신뢰도는 .008 증가하였음
- 영양학 및 생화학 과목은 .017, 영양교육, 식사요법 및 생리학 과목은 .026, 식품학 및 조리원리 과목은 .031, 급식, 위생 및 관계법규 과목은 .035 증가함

○ 분석결과 관련 문의 : 한국보건의료인국가시험원 연구개발본부 김보현 전임연구원
Tel : 02-2087-8954, FAX : 02-2087-8885
E-mail : kimbohyun@kuksiwon.or.kr
