# Supplementary material for: Item difficulty index, discrimination index, and reliability of the 26 health professions licensing examinations in 2022, Korea: a psychometric study
Source: J Educ Eval Health Prof. 2023 Nov 22;20:31. doi: 10.3352/jeehp.2023.20.31 (PMC11959405; doi:10.3352/jeehp.2023.20.31)
Supplement: Supplementary file 1 — Supplement 1. Item analysis results of 26 health professions licensing examinations administered during late 2022 and early 2023. [file jeehp-20-31_Suppl1.zip › 2022│Γ╡╡ ┴a4╚╕ ║╕┴╢░°╟╨╗τ ▒╣░í╜├╟Φ ║╨╝«░ß░·.pdf]

2022년도 제4회
보조공학사 국가시험
문항분석 결과

국민이 신뢰하고 감동하는 시험평가기관
한국보건의료인국가시험원
KOREA HEALTH PERSONNEL LICENSING EXAMINATION INSTITUTE

일반 용어 정의

□ **평균**
- 집단에서의 대표적 경향값으로 전체 값을 더하여 총 응시자로 나눈 값

□ **표준편차**
- 평균과 각 점수의 차이인 편차들의 평균으로 점수가 흩어져 분포되어 있는 정도

□ **추정난이도**
- 문항개발자가 예측한 정답률

□ **검사이론**
- 검사와 검사를 구성하고 있는 문항의 양호도를 분석 및 평가하는 방법을 정의한 이론체계
- 대표적으로 고전검사이론과 문항반응이론이 있음

고전검사이론 용어 정의

□ **고전검사이론(Classical Test Theory; CTT)**

- 검사의 질을 분석하는 검사이론 중 한 가지로 19세기 말부터 전개되어 현재까지 주로 사용되고 있는 검사이론임
- 고전검사이론에 의한 문항과 응시자 능력 추정치는 다음과 같음

○ **문항난이도**

· 검사 문항의 쉽고 어려운 정도를 나타내는 지수
· 난이도 지수는 총 반응 수에 대한 정답 반응 수의 비율로 문항의 정답률임
· 문항난이도는 0~100까지의 값을 가짐
· 난이도 값이 큰 경우, 쉬운 문항으로 '난이도가 낮다'라고 해석하며, 난이도 값이 작은 경우, 어려운 문항으로 '난이도가 높다'라고 해석함

○ **문항변별도**

· 각 문항이 응시자의 능력 수준을 변별할 수 있는 정도를 나타내는 지수
· 문항변별도는 -1~+1까지의 값을 가지며, 1에 가까울수록 변별력 크다고 해석함
· 일반적으로 문항변별도가 0.3 이상이면 우수한 문항으로 평가함
· 구하는 방식에는 '상하위집단 구분법', '문항-총점 상관계수' 등이 있음
  1) **변별도 1(상하위구분법):** 상위 27%와 하위 27% 집단의 난이도 차이를 구하는 방식
  2) **변별도 2(상관계수법):** 문항-총점과의 상관계수로 구하는 방식

○ **신뢰도**

· 시험이 평가하고자 하는 것을 일관성 있게 측정하는가로 시험이 오차없이 정확하게 측정한 정도를 의미함
· 국시원에서는 문항의 내적일관성(Cronbach α)으로 신뢰도를 추정하며 1에 가까울수록 신뢰도가 높다고 해석함

목 차

I. 시행 결과 ······ 5
1. 시험 현황 ······ 6
1) 시험명 ······ 6
2) 시험시행일 ······ 6
3) 응시현황 ······ 6
4) 과목별 문항 수, 배점 및 과락 점수 ······ 6
2. 합격률과 평균성적 ······ 6
1) 합격 및 불합격 현황 ······ 6
2) 과목별 과락자수 내역 ······ 6
3) 전회 대비 합격률과 평균성적 ······ 7

II. 문항분석 결과 ······ 8
1. 성적 ······ 9
1) 전체 성적분포도 ······ 9
2) 과목별 성적분포도 ······ 10
2. 난이도와 변별도 ······ 11
1) 전체 난이도와 변별도 ······ 11
2) 과목별 난이도와 변별도 ······ 14
3) 지식수준별 난이도와 변별도 ······ 20
4) 자료유형별 난이도와 변별도 ······ 29
3. 난이도와 변별도 간 산포도 ······ 35
1) 전체 난이도와 변별도 간 산포도 ······ 35
2) 과목별 난이도와 변별도 간 산포도 ······ 35
4. 신뢰도 분석 ······ 37

I. 시행 결과

1. 시험 현황(※ 합격자 발표일을 기준으로 한 자료임)

1) 시험명: 2022년도 제4회 보조공학사 국가시험

2) 시험시행일: 2022년 2월 12일

3) 응시현황

응시대상자수	결시자수	부정행위자수	응시자 준수사항 위반자 수		응시자수 (%)
			휴대폰 소지	신분증 미지참	
265	17	0	0	0	248 (94.6)

4) 과목별 문항 수, 배점 및 과락 점수

교 시	과 목 명	문제 수	배점	총점	합격자 점수기준	
					과목 과락기준	총점 합격기준
1교시	보조공학사 기초	70	1	70	28점 미만	102점 이상
2교시	보조공학사 응용·실기	100	1	100	40점 미만	
계		170	-	170	-	-

2. 합격률과 평균성적

1) 합격 및 불합격 현황

합격자수 (%)	불합격자수(%)				채점보류자수
	평락	과락	기권	계	
167 (67.3)	81 (32.7)	0 (0.0)	0 (0.0)	81 (32.7)	0

2) 과목별 과락자수 내역

과목명 / 과락자수	보조공학사 기초	보조공학사 응용실기
과목별 과락자 수	0	0
전과목 과락자 수	0	

3) 전회 대비 합격률과 평균성적

회차	년도	합격률(%)	평균성적	표준편차	백분율 환산점수
제1회	2019.07	95.9	125.8	13.3	74.0
제2회	2020.02	68.1	106.9	12.9	62.9
제3회	2021.01	81.3	114.7	15.9	67.5
**제4회**	**2022.02**	**67.3**	**109.5**	**16.1**	**64.4**

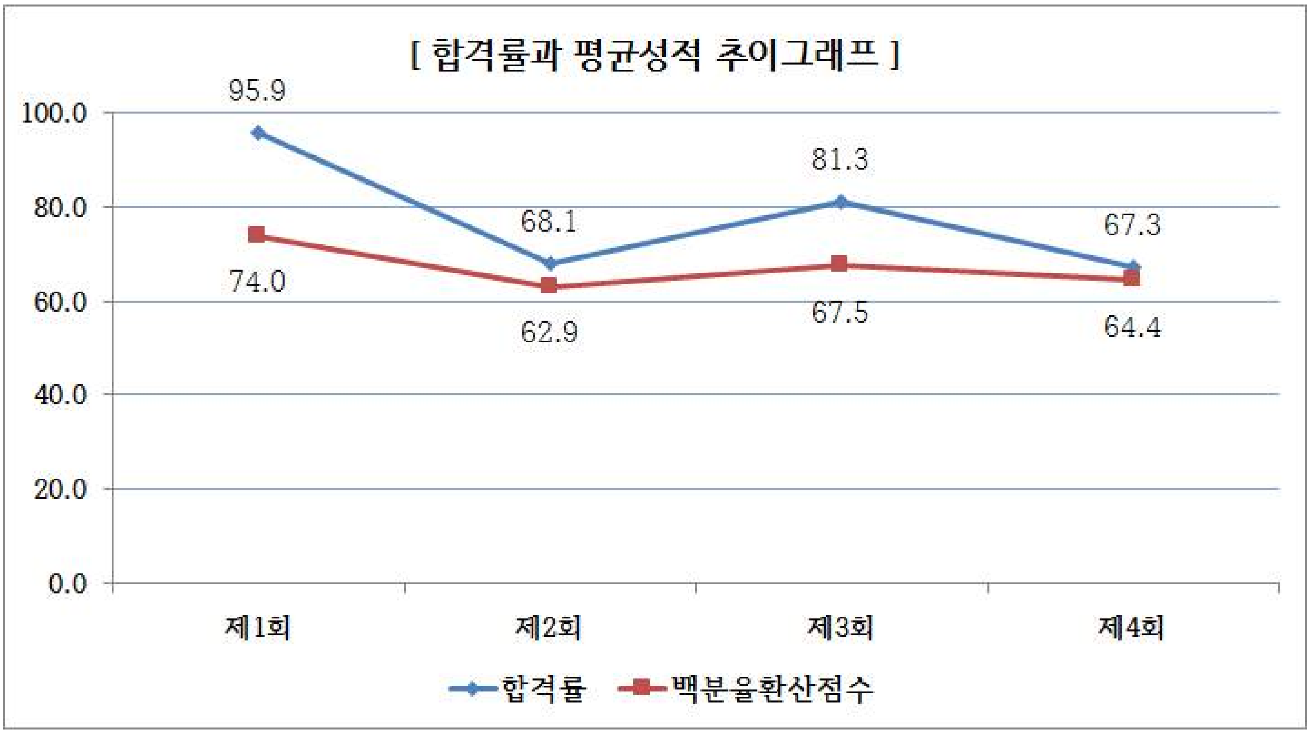

해석

- 전년 대비 합격률은 14.0% 감소했으며, 백분율 환산점수는 3.1 감소함

II. 문항분석 결과

1. 성적

1) 전체 성적분포도

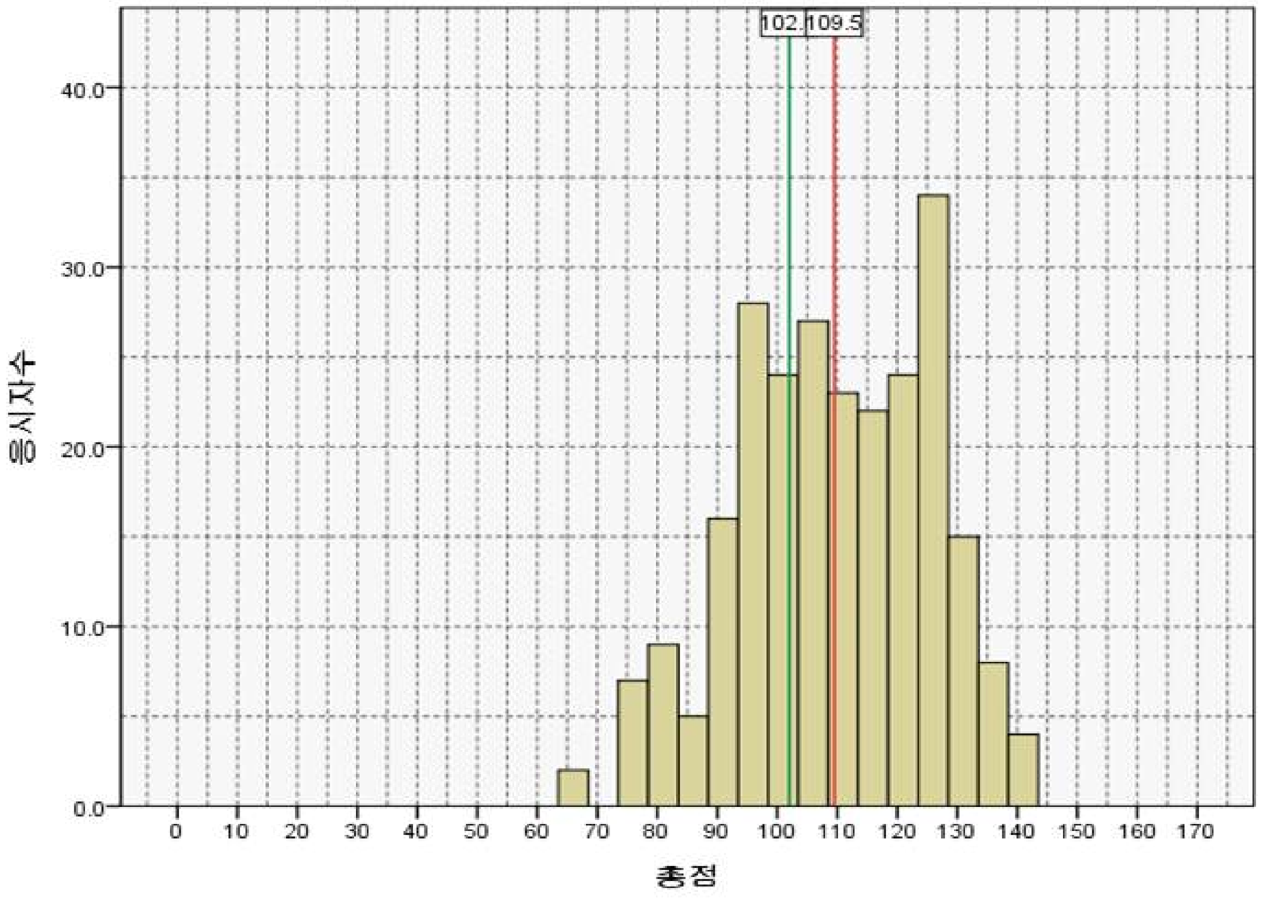

응시자	총점	합격선	평균성적	표준편차
248	170.0	102.0	109.5	16.1

2) 과목별 성적분포도

가) 보조공학사 기초

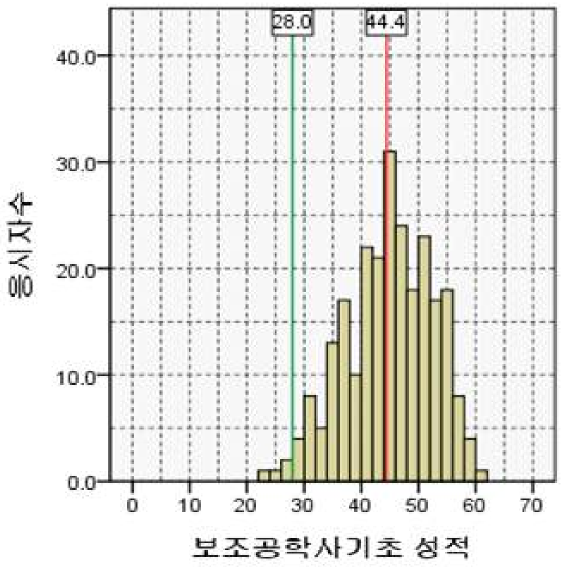

나) 보조공학사 응용실기

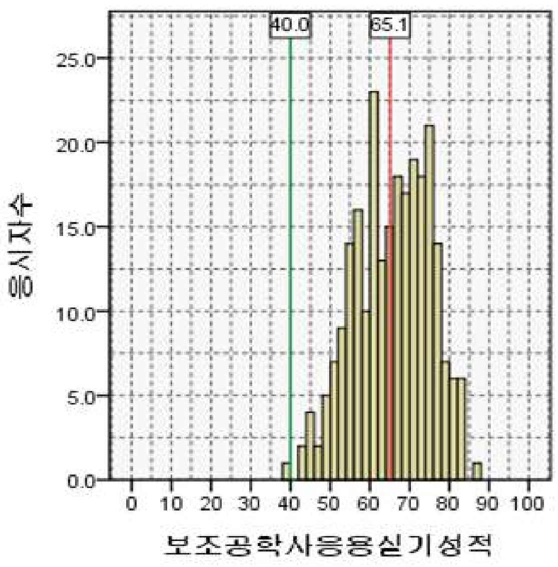

총점	과락선	평균성적	표준편차
70.0	28.0	44.4	7.6

총점	과락선	평균성적	표준편차
100.0	40.0	65.1	9.5

2. 난이도와 변별도

1) 전체 난이도와 변별도

가) 전회 대비 전체 난이도와 변별도

회차	난이도		변별도1		변별도2	
	평균	표준편차	평균	표준편차	평균	표준편차
제1회	74.0	23.2	.19	.15	.19	.12
제2회	62.9	25.1	.18	.15	.16	.15
제3회	67.5	23.1	.23	.16	.20	.12
**제4회**	**64.4**	**24.3**	**.23**	**.15**	**.20**	**.12**

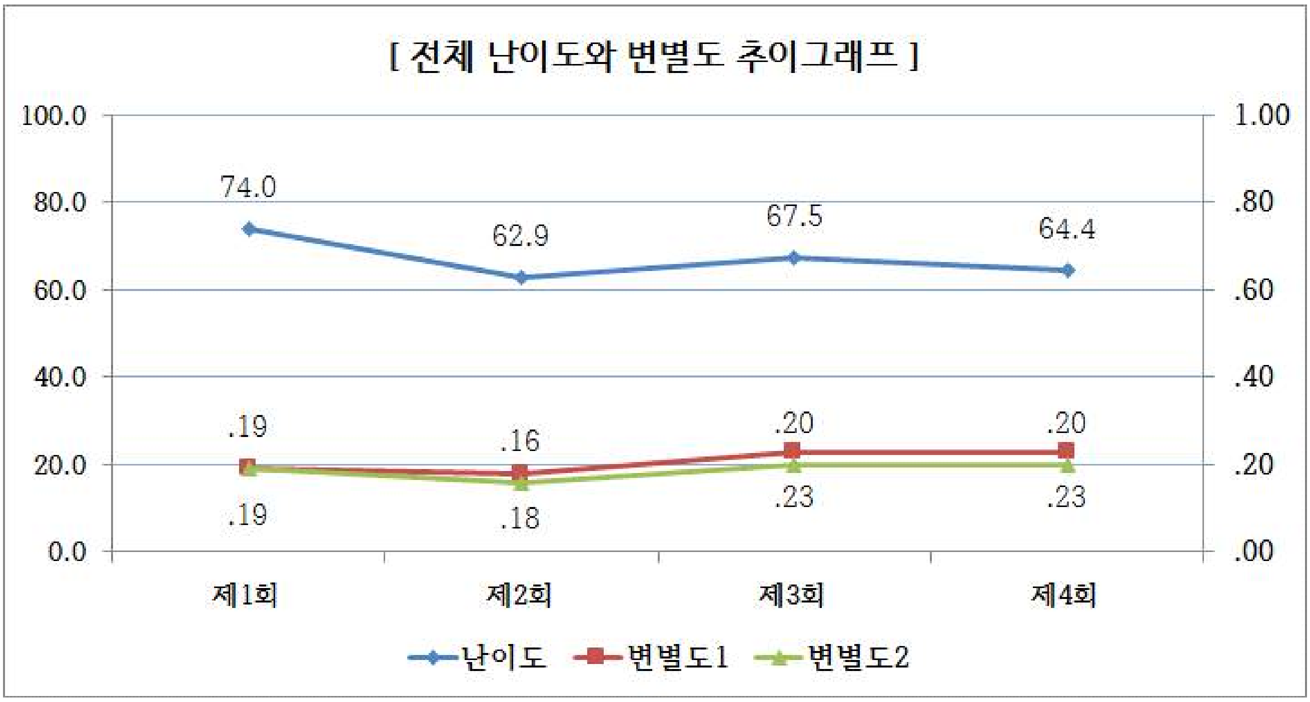

해석

- 전년 대비 난이도 지수는 3.1 감소함
- 전년 대비 변별도 1 지수는 동일함
- 전년 대비 변별도 2 지수는 동일함

나) 전체 난이도와 변별도 분포도 및 비율분석

(1) 전체 난이도 분포도 및 비율분석

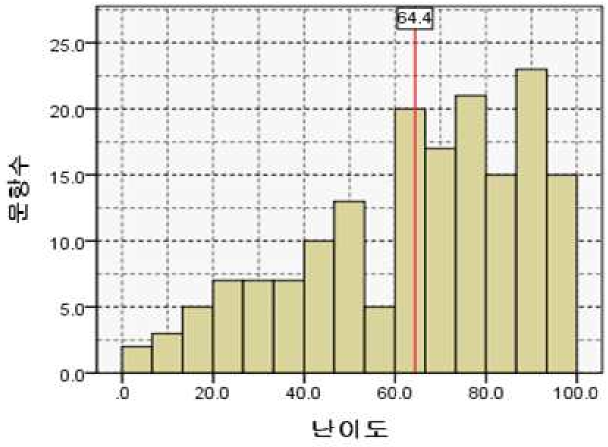

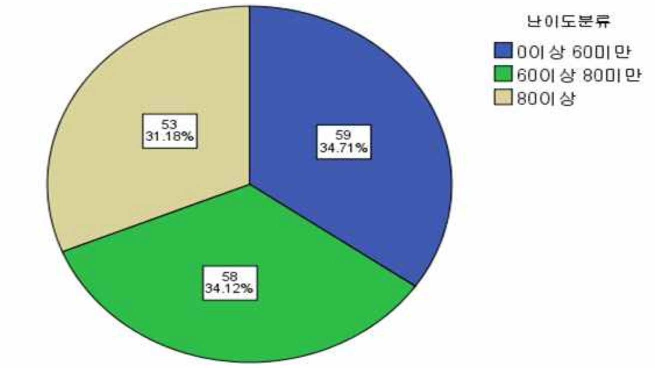

총점	난이도	표준편차
170	64.4	24.3

난이도	문항수	비율(%)
0~60미만	59	34.7
60~80미만	58	34.1
80~100	53	31.2
전체	170	100.0

(2) 전체 변별도1 분포도 및 비율분석

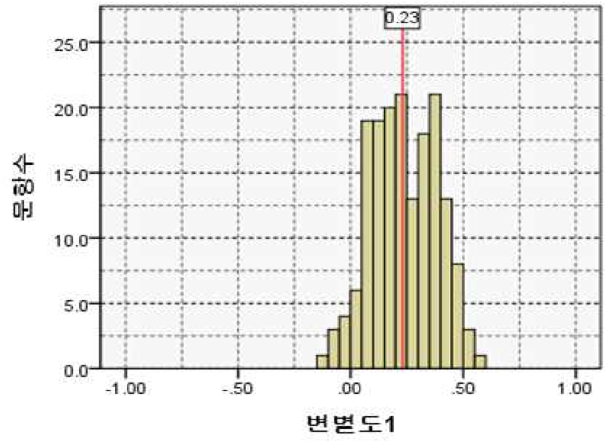

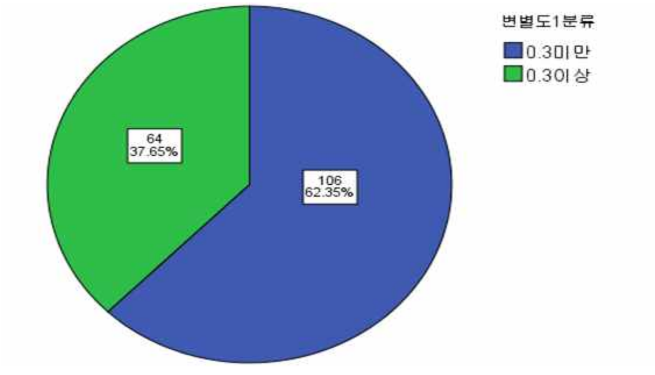

총점	변별도1	표준편차
170	.23	.15

변별도1	문항수	비율(%)
0.3미만	106	62.4
0.3이상	64	37.6
전체	170	100.0

(3) 전체 변별도2 분포도 및 비율분석

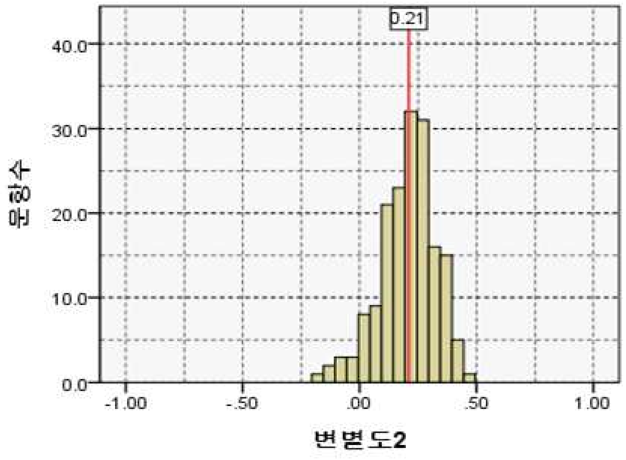

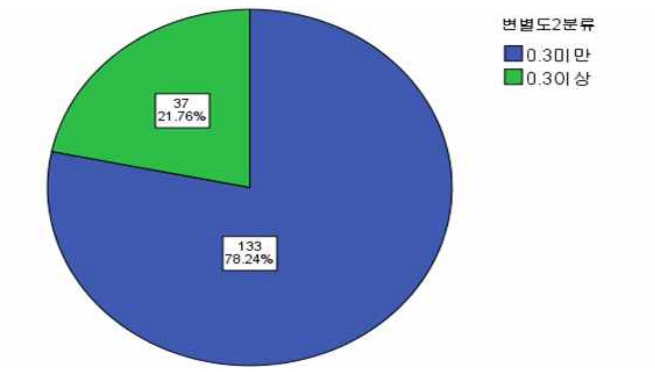

총점	변별도2	표준편차
170	.21	.12

변별도2	문항수	비율(%)
0.3미만	133	78.2
0.3이상	37	21.8
전체	170	100.0

해석

- 난이도 지수가 60 미만인 문항이 전체 170 문항 중 59 문항으로 가장 많았으며, 차례로 60 이상 80 미만인 문항이 58 문항, 60 미만인 문항이 53 문항인 것으로 나타남
- 변별도 1 지수를 기준으로 분류하였을 때, 0.3 미만인 문항이 106 문항으로 0.3 이상인 문항이 64 문항인 것에 비해 더 많이 나타남
- 변별도 2 지수를 기준으로 분류하였을 때, 0.3 미만인 문항이 133 문항으로 0.3 이상인 문항이 37 문항인 것에 비해 더 많이 나타남

2) 과목별 난이도와 변별도

가) 전회 대비 과목별 난이도와 변별도

(1) 전회 대비 보조공학사 기초 난이도와 변별도

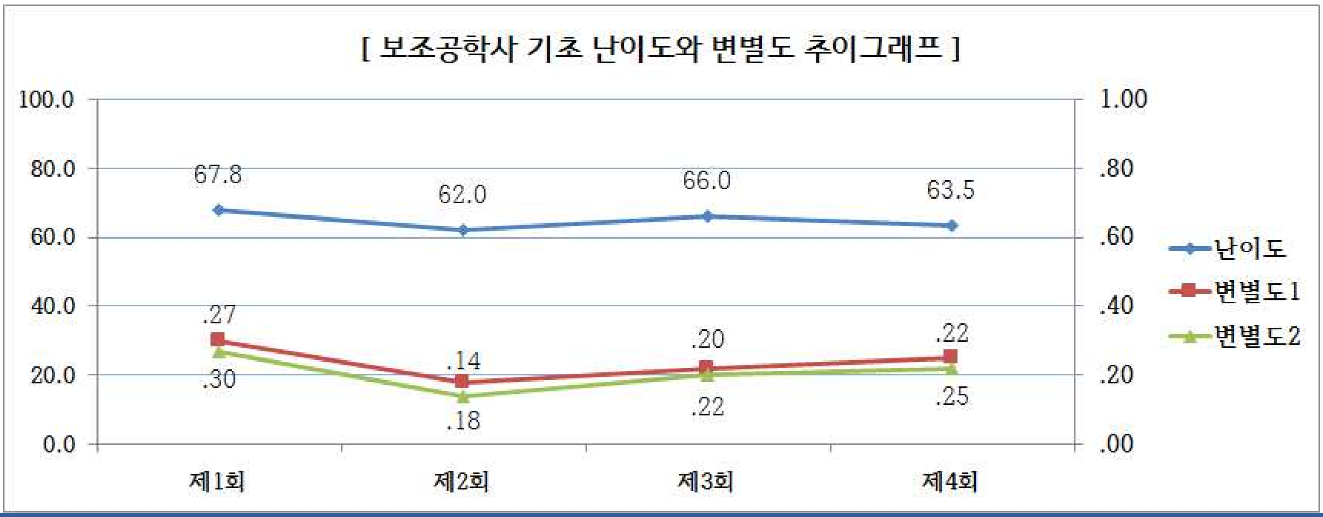

회차	난이도		변별도1		변별도2	
	평균	표준편차	평균	표준편차	평균	표준편차
제1회	67.8	19.2	.30	.13	.27	.11
제2회	62.0	22.8	.18	.16	.14	.14
제3회	66.0	23.0	.22	.16	.20	.13
**제4회**	**63.5**	**25.8**	**.25**	**.14**	**.22**	**.12**

해석

- 전회 대비 보조공학사 기초 과목의 난이도 지수는 2.5 감소함
- 전회 대비 보조공학사 기초 과목의 변별도 1 지수는 0.03 증가함
- 전회 대비 보조공학사 기초 과목의 변별도 2 지수는 0.02 증가함

(2) 전회 대비 보조공학사 응용·실기 난이도와 변별도

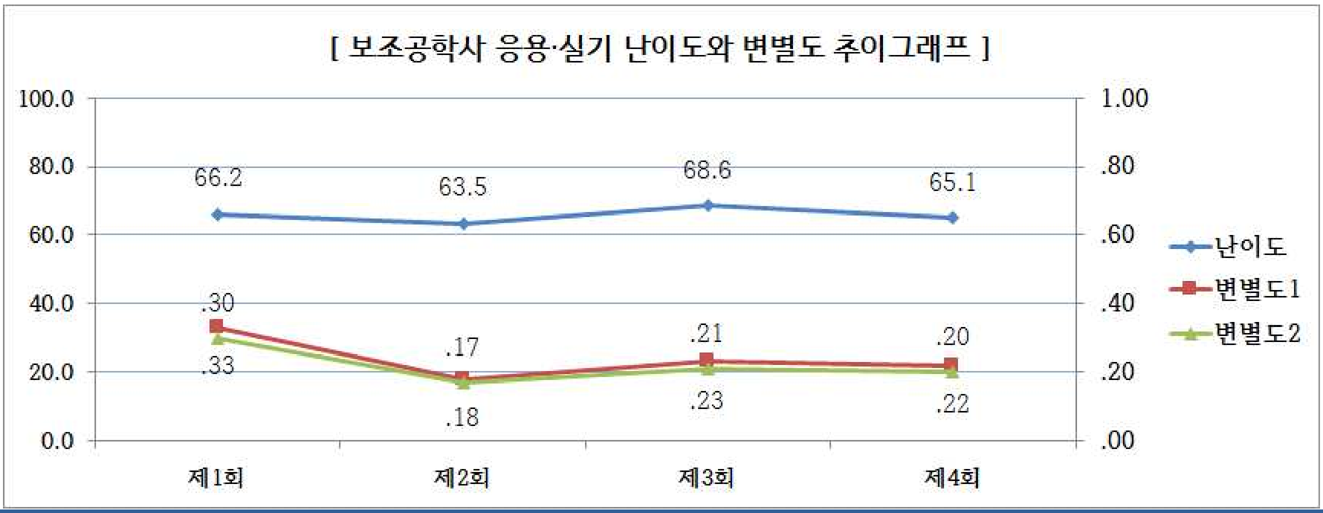

회차	난이도		변별도1		변별도2	
	평균	표준편차	평균	표준편차	평균	표준편차
제1회	66.2	16.9	.33	.10	.30	.10
제2회	63.5	26.7	.18	.15	.17	.15
제3회	68.6	23.2	.23	.15	.21	.12
**제4회**	**65.1**	**23.3**	**.22**	**.15**	**.20**	**.12**

해석

- 전회 대비 보조공학사 응용·실기 과목의 난이도 지수는 3.5 감소함
- 전회 대비 보조공학사 응용·실기 과목의 변별도 1 지수는 0.01 감소함
- 전회 대비 보조공학사 응용·실기 과목의 변별도 2 지수는 0.01 감소함

나) 과목별 난이도와 변별도 분포도 및 비율분석

(1) 보조공학사 기초 난이도와 변별도 분포도 및 비율분석

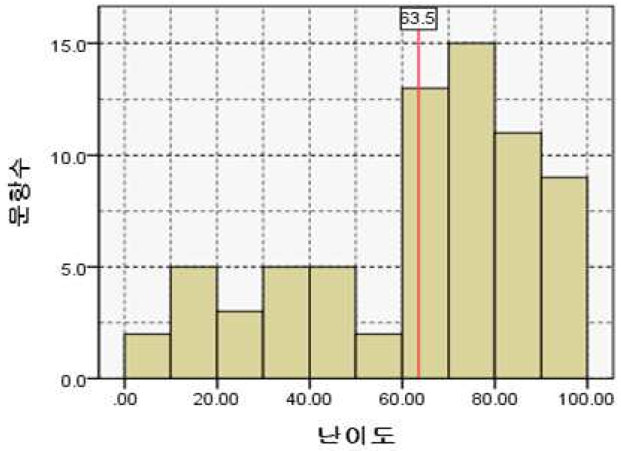

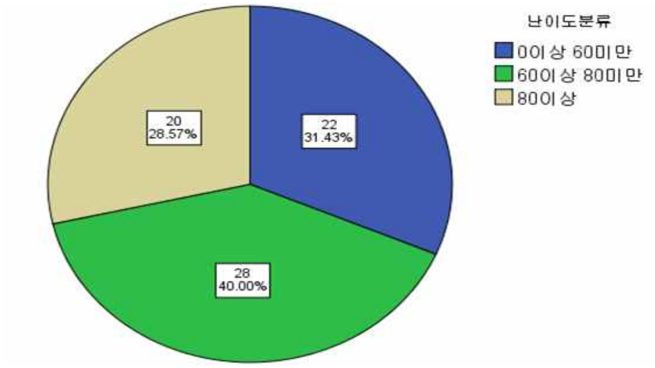

총점	난이도	표준편차
70	63.5	25.8

난이도	문항수	비율(%)
0~60미만	22	31.4
60~80미만	28	40.0
80~100	20	28.6
전체	70	100.0

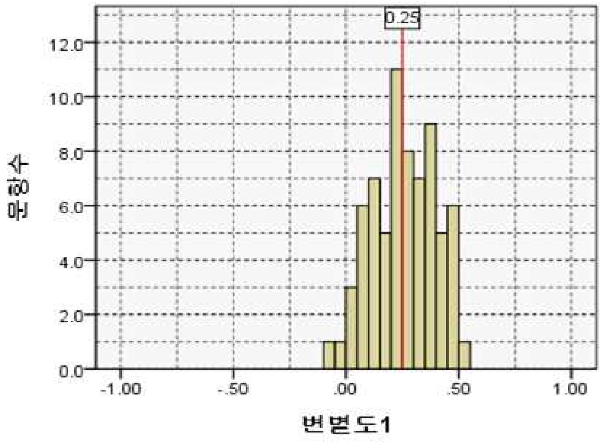

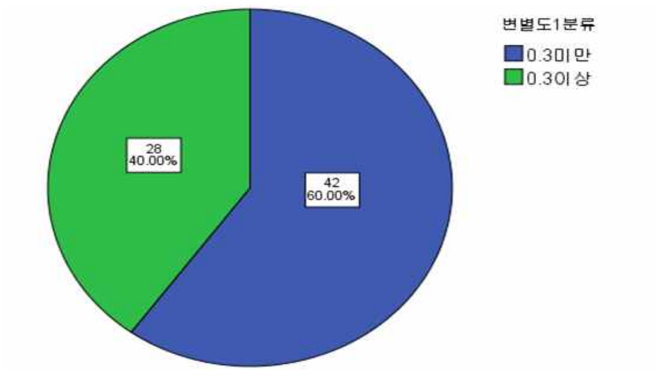

총점	변별도1	표준편차
70	.25	.14

변별도1	문항수	비율(%)
0.3미만	42	60.0
0.3이상	28	40.0
전체	70	100.0

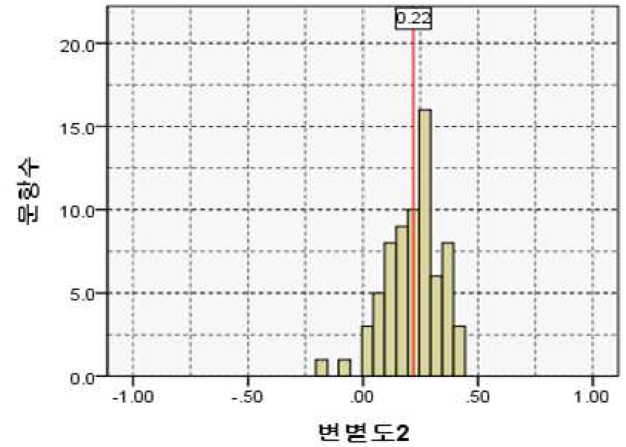

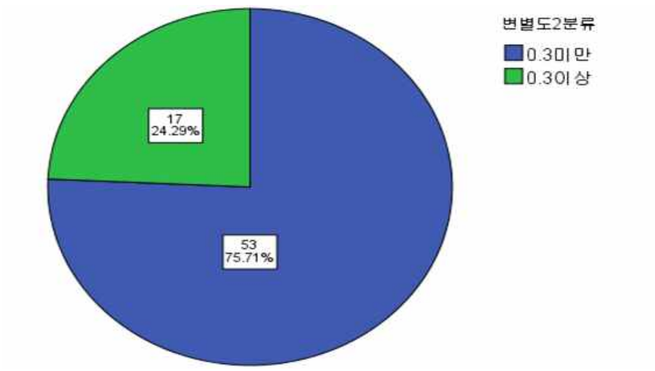

총점	변별도2	표준편차
70	.22	.12

변별도2	문항수	비율(%)
0.3미만	53	75.7
0.3이상	17	24.3
전체	70	100.0

해석

- 보조공학사 기초 과목에서 난이도 지수가 60 이상 80 미만인 문항이 전체 70 문항 중 28 문항으로 가장 많았으며, 차례로 60 미만인 문항이 22 문항, 80 에서 100 사이인 문항이 20 문항인 것으로 나타남
- 변별도 1 지수를 기준으로 분류하였을 때, 0.3 미만인 문항이 42 문항으로 0.3 이상인 문항이 28 문항인 것에 비해 더 많이 나타남
- 변별도 2 지수를 기준으로 분류하였을 때, 0.3 미만인 문항이 53 문항으로 0.3 이상인 문항이 17 문항인 것에 비해 더 많이 나타남

(2) 보조공학사 응용·실기 난이도와 변별도 분포도 및 비율분석

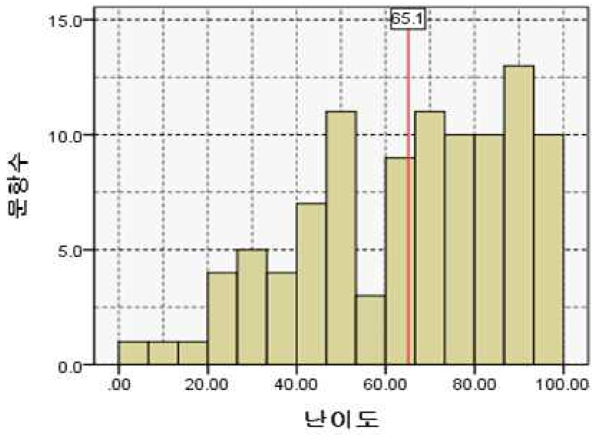

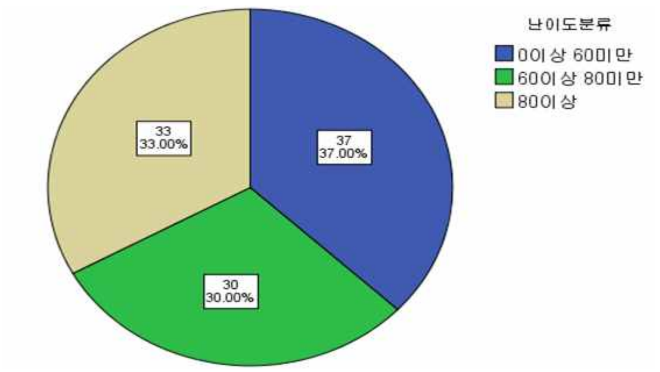

총점	난이도	표준편차	난이도	문항수	비율(%)
100	65.1	23.3	0~60미만	37	37.0
			60~80미만	30	30.0
			80~100	33	33.0
			전체	100	100.0

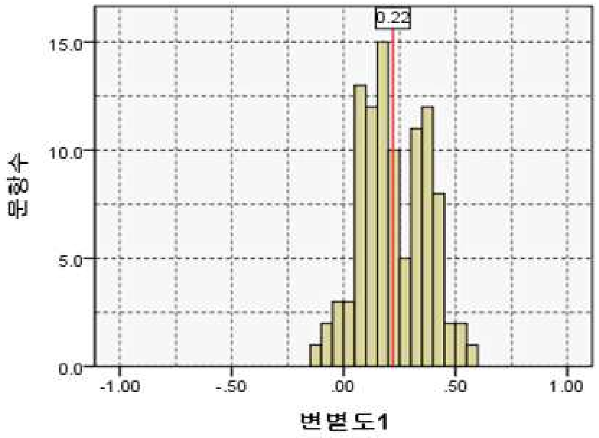

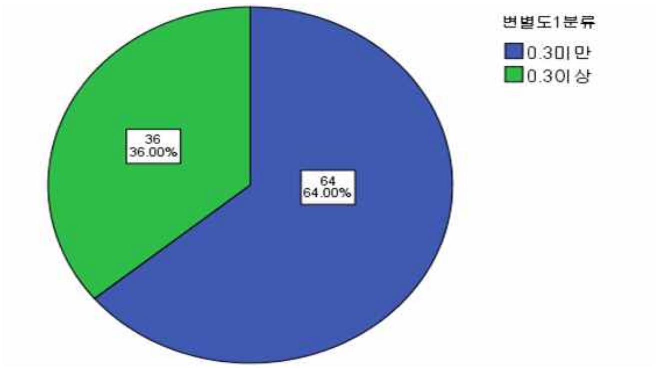

총점	변별도1	표준편차	변별도1	문항수	비율(%)
100	.22	.15	0.3미만	64	64.0
			0.3이상	36	36.0
			전체	100	100.0

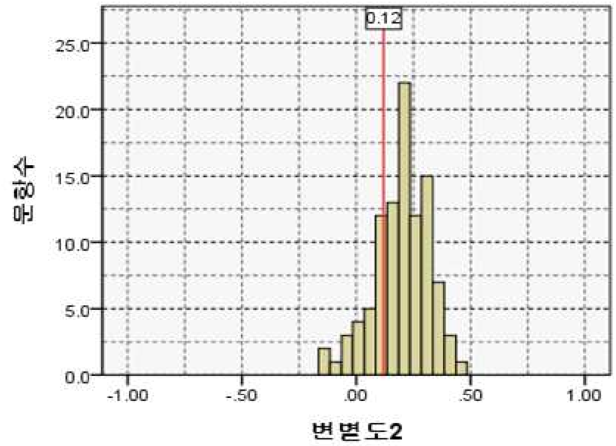

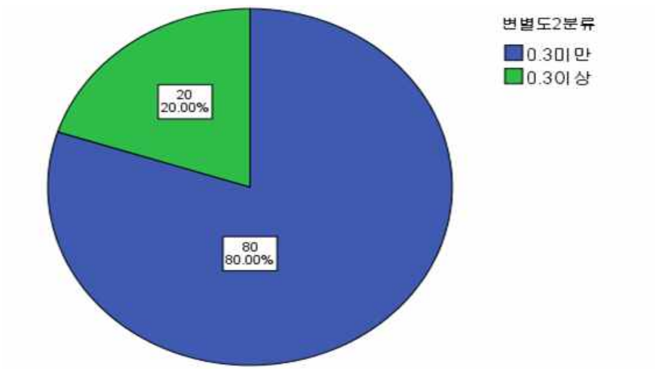

총점	변별도2	표준편차
100	.20	.12

변별도2	문항수	비율(%)
0.3미만	80	80.0
0.3이상	20	20.0
전체	100	100.0

해석

- 보조공학사 응용·실기 과목에서 난이도 지수가 60 미만인 문항이 전체 100 문항 중 37 문항으로 가장 많았으며, 차례로 80 에서 100 사이인 문항이 33 문항, 60 이상 80 미만인 문항이 30 문항인 것으로 나타남
- 변별도 1 지수를 기준으로 분류하였을 때, 0.3 미만인 문항이 64 문항으로 0.3 이상인 문항이 36 문항인 것에 비해 더 많이 나타남
- 변별도 2 지수를 기준으로 분류하였을 때, 0.3 미만인 문항이 80 문항으로 0.3 이상인 문항이 20 문항인 것에 비해 더 많이 나타남

3) 지식수준별 난이도와 변별도

가) 전회 대비 지식수준별 난이도와 변별도

(1) 전회 대비 암기형 난이도와 변별도

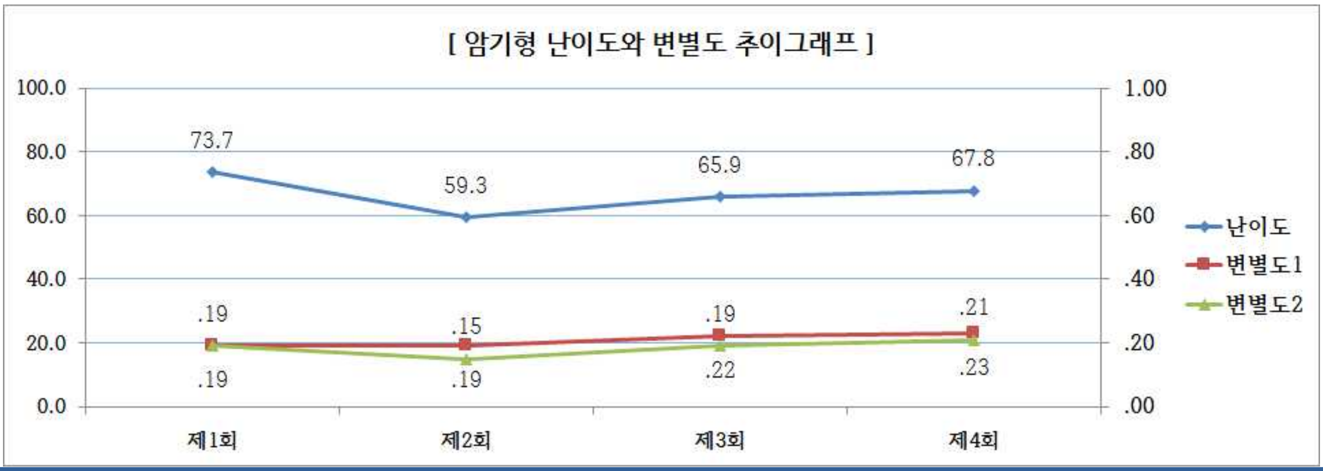

회차	난이도		변별도1		변별도2	
	평균	표준편차	평균	표준편차	평균	표준편차
제1회	73.7	23.7	.19	.15	.19	.12
제2회	59.3	23.8	.19	.15	.15	.15
제3회	65.9	24.5	.22	.16	.19	.13
**제4회**	**67.8**	**23.2**	**.23**	**.15**	**.21**	**.11**

해석

- 전회 대비 암기형 문항의 난이도 지수는 1.9 증가함
- 전회 대비 암기형 문항의 변별도 1 지수는 0.01 증가함
- 전회 대비 암기형 문항의 변별도 2 지수는 0.02 증가함

(2) 전회 대비 해석형 난이도와 변별도

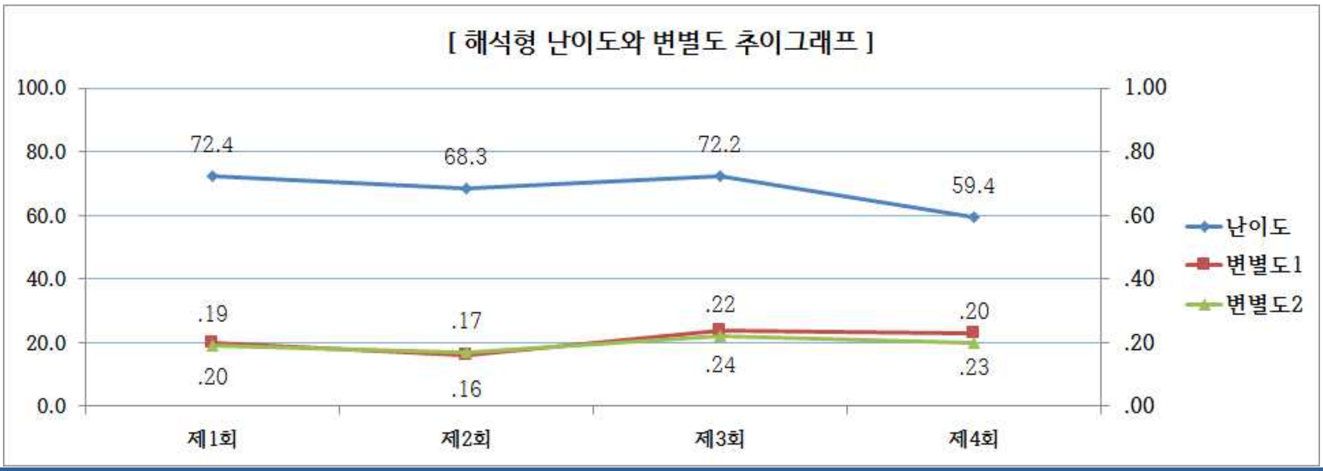

회차	난이도		변별도1		변별도2	
	평균	표준편차	평균	표준편차	평균	표준편차
제1회	72.4	22.7	.20	.14	.19	.11
제2회	68.3	26.2	.16	.14	.17	.14
제3회	72.2	19.8	.24	.15	.22	.11
**제4회**	**59.4**	**26.2**	**.23**	**.14**	**.20**	**.12**

해석

- 전회 대비 해석형 문항의 난이도 지수는 12.8 감소함
- 전회 대비 해석형 문항의 변별도 1 지수는 0.01 감소함
- 전회 대비 해석형 문항의 변별도 2 지수는 0.02 감소함

(3) 전회 대비 해결형 난이도와 변별도

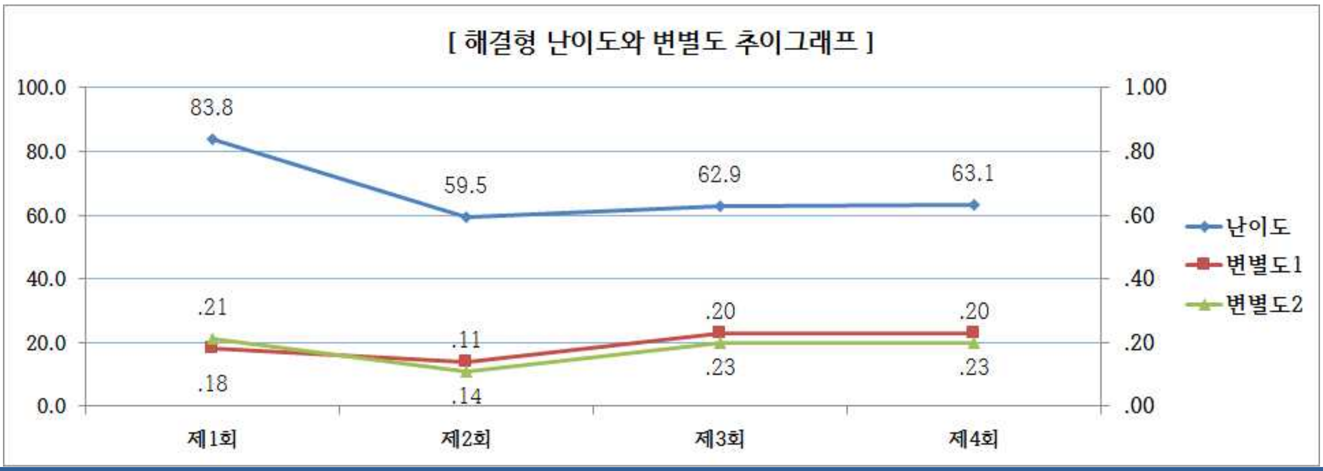

회차	난이도		변별도1		변별도2	
	평균	표준편차	평균	표준편차	평균	표준편차
제1회	83.8	15.5	.18	.14	.21	.08
제2회	59.5	25.8	.14	.19	.11	.15
제3회	62.9	24.0	.23	.15	.20	.12
**제4회**	**63.1**	**21.7**	**.23**	**.18**	**.20**	**.15**

해석

- 전회 대비 해결형 문항의 난이도 지수는 0.2 감소함
- 전회 대비 해결형 문항의 변별도1 지수는 동일함
- 전회 대비 해결형 문항의 변별도2 지수는 동일함

나) 지식수준별 난이도와 변별도 분포도 및 비율분석

(1) 암기형 난이도와 변별도 분포도 및 비율분석

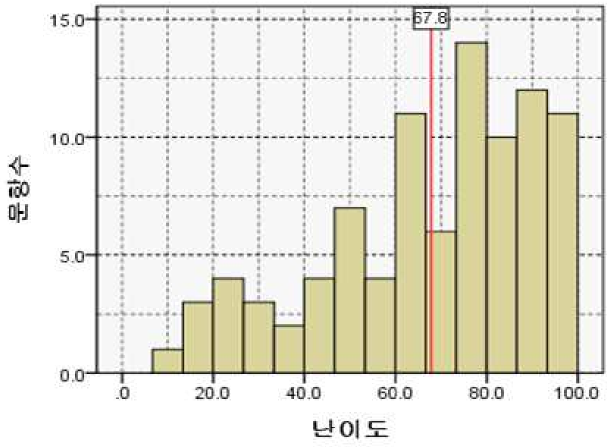

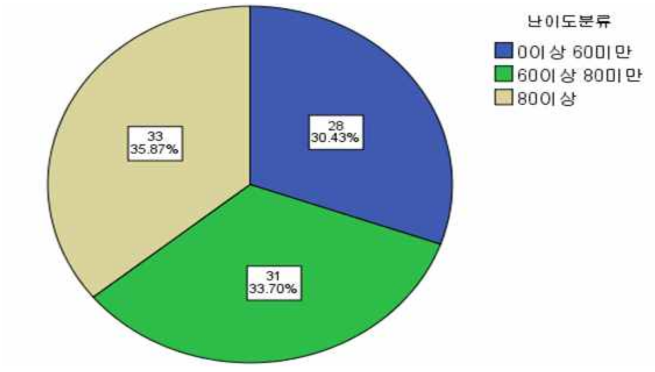

총점	난이도	표준편차
92	67.8	23.2

난이도	문항수	비율(%)
0~60미만	28	30.4
60~80미만	31	33.7
80~100	33	35.9
전체	92	100.0

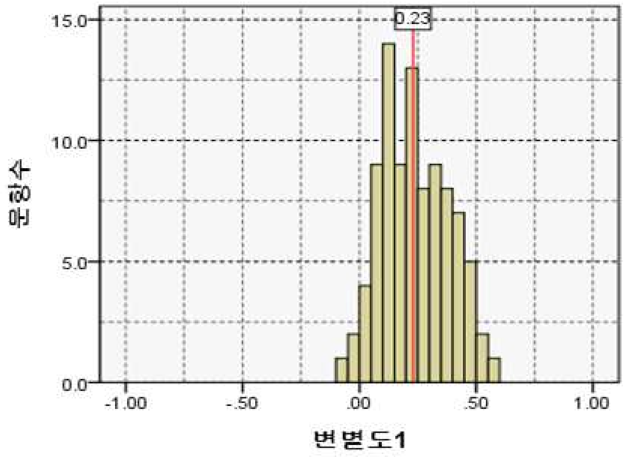

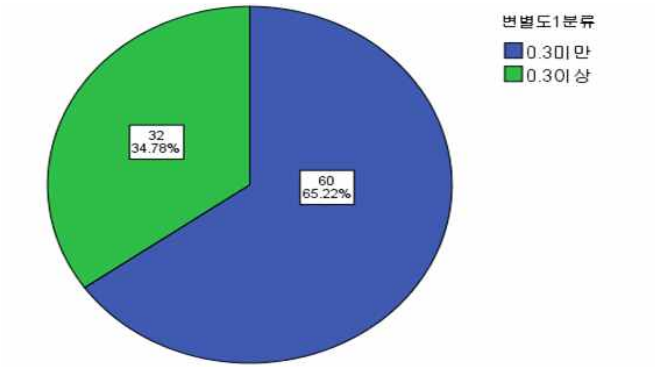

총점	변별도1	표준편차
92	.23	.15

변별도1	문항수	비율(%)
0.3미만	60	65.2
0.3이상	32	34.8
전체	92	100.0

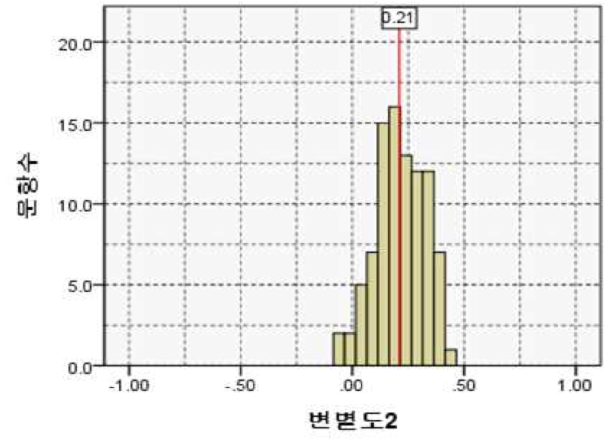

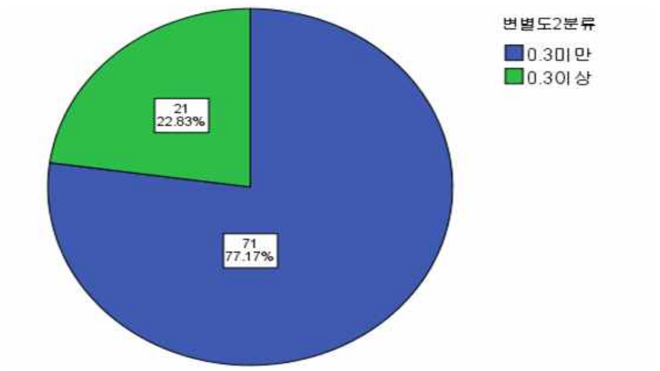

총점	변별도2	표준편차
92	.21	.11

변별도2	문항수	비율(%)
0.3미만	71	77.2
0.3이상	21	22.8
전체	92	100.0

해석

- 암기형 문항에서 난이도 지수가 80에서 100사이인 문항이 전체 92문항 중 33문항으로 가장 많았으며, 차례로 60이상 80미만인 문항이 31문항, 60미만인 문항이 28문항인 것으로 나타남
- 변별도1 지수를 기준으로 분류하였을 때, 0.3미만인 문항이 60문항으로 0.3이상인 문항이 32문항인 것에 비해 더 많이 나타남
- 변별도2 지수를 기준으로 분류하였을 때, 0.3미만인 문항이 71문항으로 0.3이상인 문항이 21문항인 것에 비해 더 많이 나타남

(2) 해석형 난이도와 변별도 분포도 및 비율분석

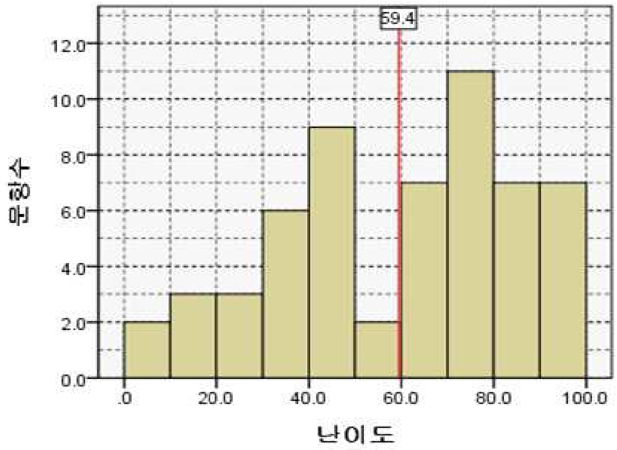

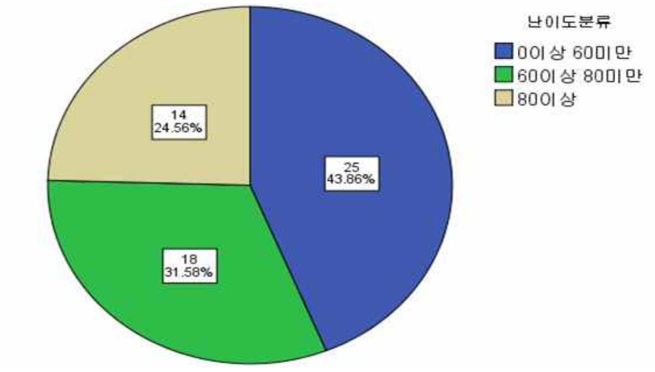

총점	난이도	표준편차
57	59.4	26.2

난이도	문항수	비율(%)
0~60미만	25	43.9
60~80미만	18	31.6
80~100	14	24.6
전체	57	100.0

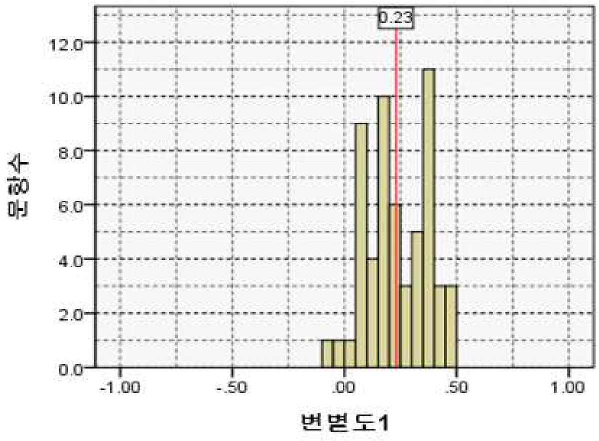

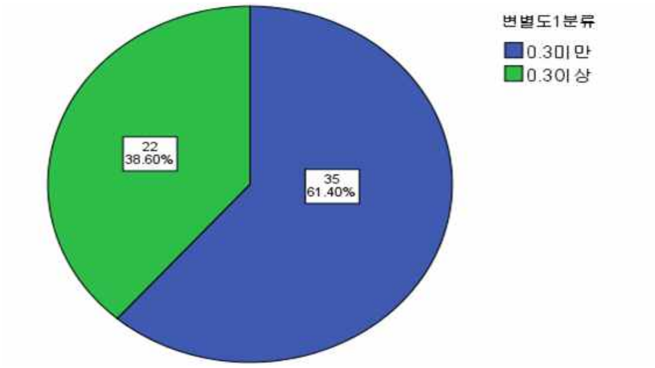

총점	변별도1	표준편차
57	.23	.14

변별도1	문항수	비율(%)
0.3미만	35	61.4
0.3이상	22	38.6
전체	57	100.0

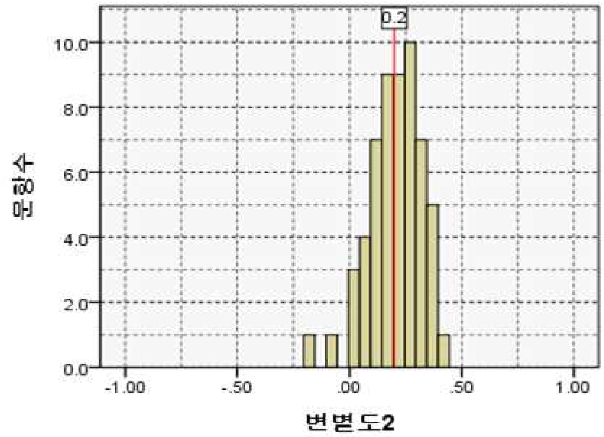

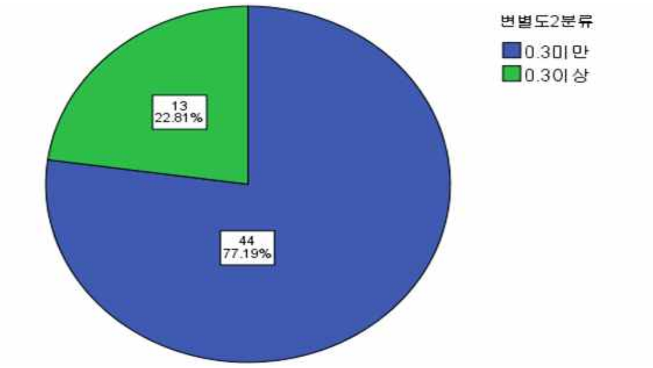

총점	변별도2	표준편차	변별도2	문항수	비율(%)
57	.20	.12	0.3미만	44	77.2
			0.3이상	13	22.8
			전체	57	100.0

해석

- 해석형 문항에서 난이도 지수가 60 미만인 문항이 전체 57 문항 중 25 문항으로 가장 많았으며, 차례로 60 이상 80 미만인 문항이 18 문항, 80 에서 100 사이인 문항이 14 문항인 것으로 나타남
- 변별도 1 지수를 기준으로 분류하였을 때, 0.3 미만인 문항이 35 문항으로 0.3 이상인 문항이 22 문항인 것에 비해 더 많이 나타남
- 변별도 2 지수를 기준으로 분류하였을 때, 0.3 미만인 문항이 44 문항으로 0.3 이상인 문항이 13 문항인 것에 비해 더 많이 나타남

(3) 해결형 난이도와 변별도 분포도 및 비율분석

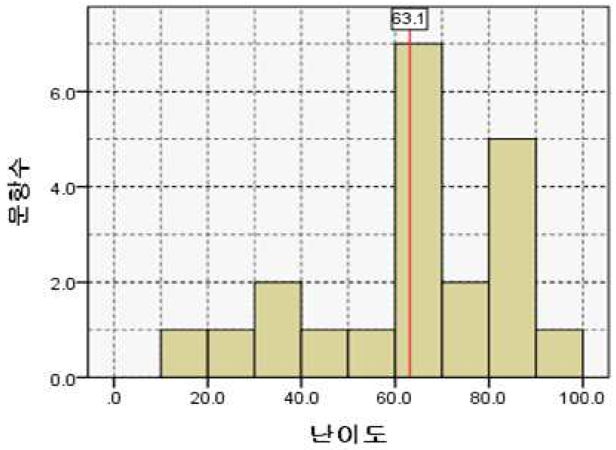

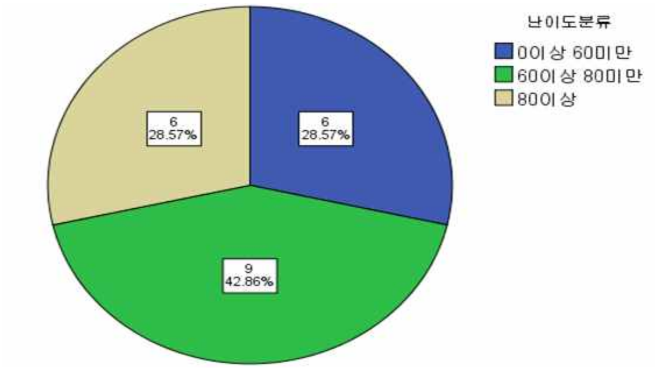

총점	난이도	표준편차
21	63.1	21.7

난이도	문항수	비율(%)
0~60미만	6	44.0
60~80미만	9	24.0
80~100	6	32.0
전체	21	100.0

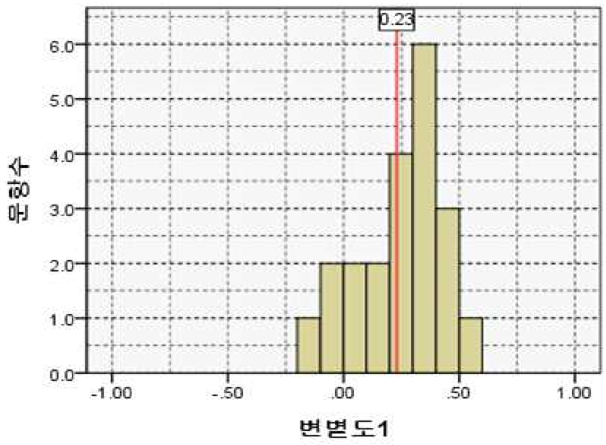

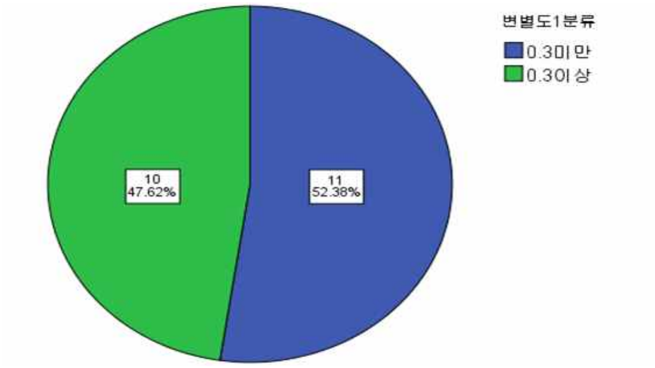

총점	변별도1	표준편차
21	.23	.18

변별도1	문항수	비율(%)
0.3미만	11	52.4
0.3이상	10	47.6
전체	21	100.0

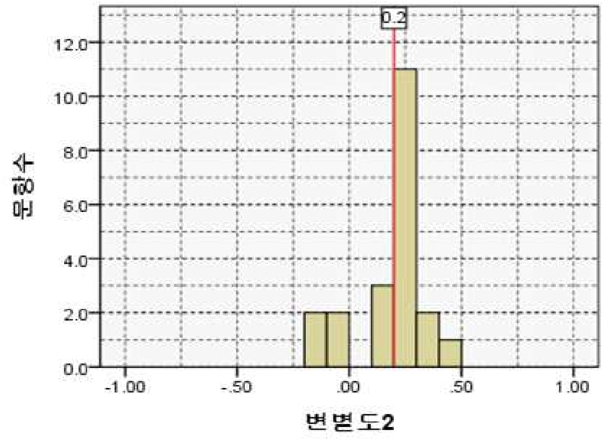

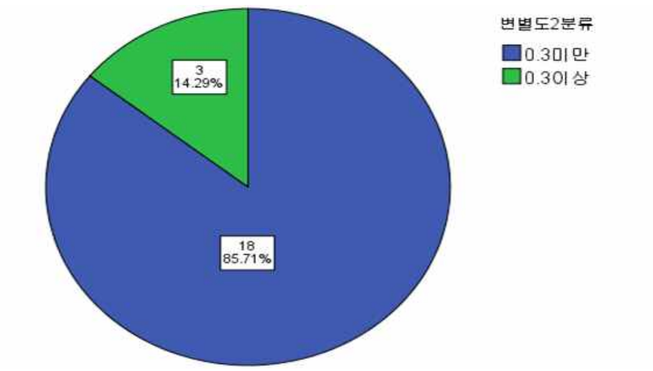

총점	변별도2	표준편차
21	.20	.15

변별도2	문항수	비율(%)
0.3미만	18	85.7
0.3이상	3	14.3
전체	21	100.0

해석

- 해결형 문항에서 난이도 지수가 60 이상 80 미만인 문항이 전체 21 문항 중 9 문항으로 가장 많았으며, 차례로 60 미만인 문항과 80 에서 100 사이인 문항이 6 문항인 것으로 나타남
- 변별도 1 지수를 기준으로 분류하였을 때, 0.3 미만인 문항이 11 문항으로 0.3 이상인 문항이 10 문항인 것에 비해 더 많이 나타남
- 변별도 2 지수를 기준으로 분류하였을 때, 0.3 미만인 문항이 18 문항으로 0.3 이상인 문항이 3 문항인 것에 비해 더 많이 나타남

4) 자료유형별 난이도와 변별도

가) 전회 대비 자료유형별 난이도와 변별도

(1) 전회 대비 텍스트형 난이도와 변별도

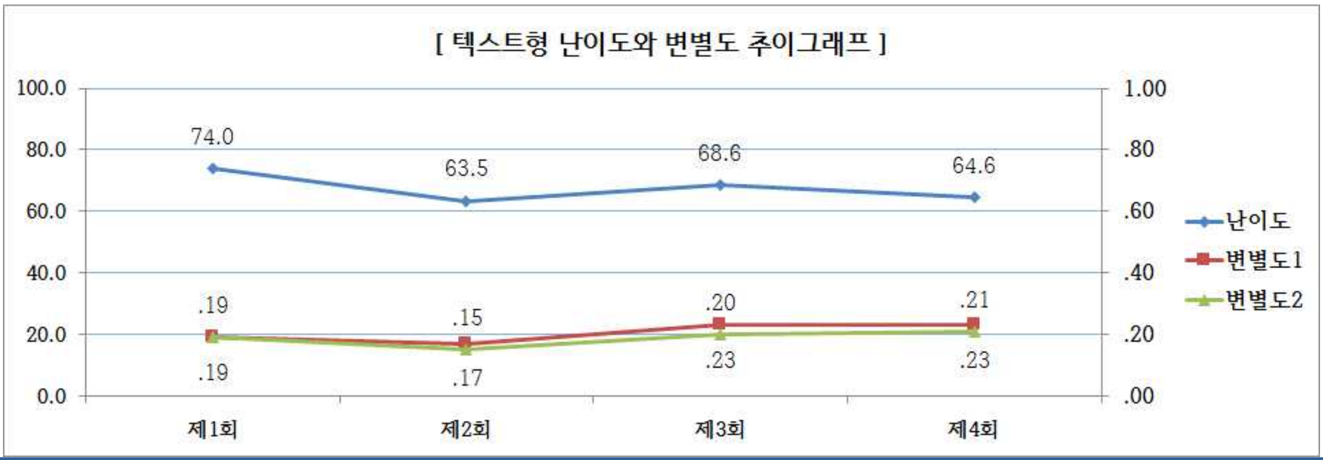

회차	난이도		변별도1		변별도2	
	평균	표준편차	평균	표준편차	평균	표준편차
제1회	74.0	22.9	.19	.15	.19	.12
제2회	63.5	25.0	.17	.15	.15	.15
제3회	68.6	22.8	.23	.16	.20	.12
**제4회**	**64.6**	**24.4**	**.23**	**.15**	**.21**	**.12**

해석

- 전회 대비 텍스트형 문항의 난이도 지수는 4.0 감소함
- 전회 대비 텍스트형 문항의 변별도 1 지수는 동일함
- 전회 대비 텍스트형 문항의 변별도 2 지수는 0.01 감소함

(2) 전회 대비 자료제시형 난이도와 변별도

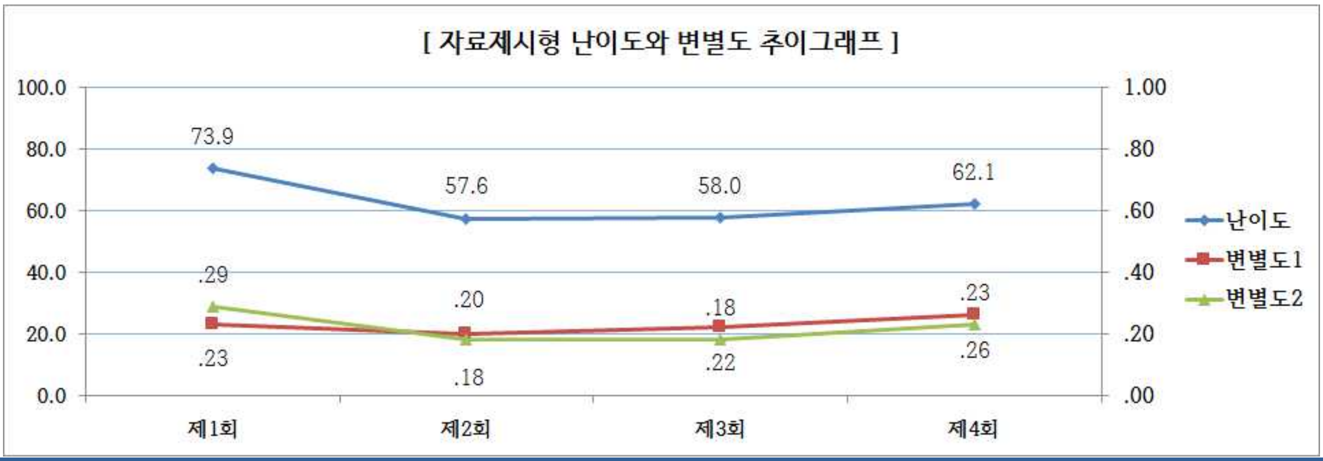

회차	난이도		변별도1		변별도2	
	평균	표준편차	평균	표준편차	평균	표준편차
제1회	73.9	27.6	.23	.08	.29	.14
제2회	57.6	26.6	.20	.13	.18	.12
제3회	58.0	24.1	.22	.17	.18	.13
**제4회**	**62.1**	**23.9**	**.26**	**.15**	**.23**	**.10**

해석

- 전회 대비 자료제시형 문항의 난이도 지수는 4.1 감소함
- 전회 대비 자료제시형 문항의 변별도 1 지수는 0.04 증가함
- 전회 대비 자료제시형 문항의 변별도 2 지수는 0.05 증가함

나) 자료유형별 난이도와 변별도 분포도 및 비율분석

(1) 텍스트형 난이도와 변별도 분포도 및 비율분석

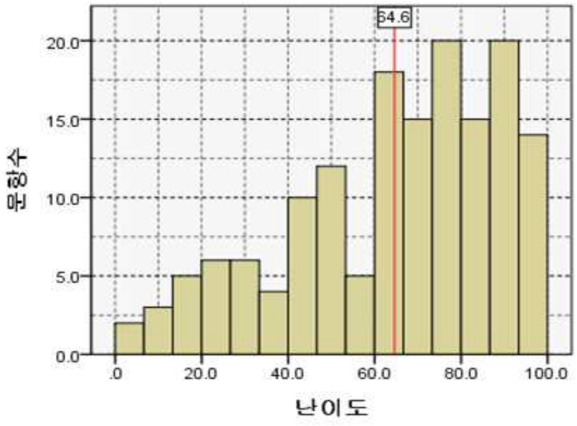

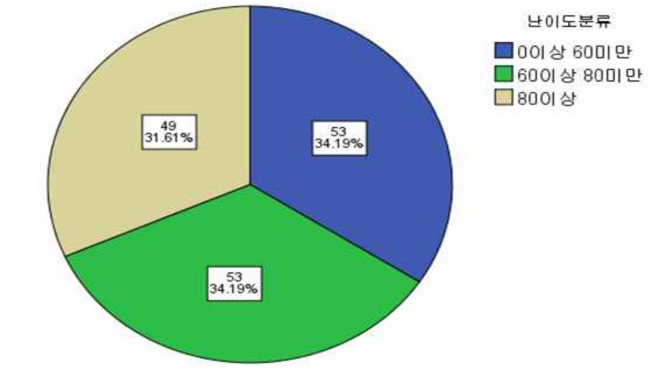

총점	난이도	표준편차
155	64.6	24.4

난이도	문항수	비율(%)
0~60미만	53	34.2
60~80미만	53	34.2
80~100	49	31.6
전체	155	100.0

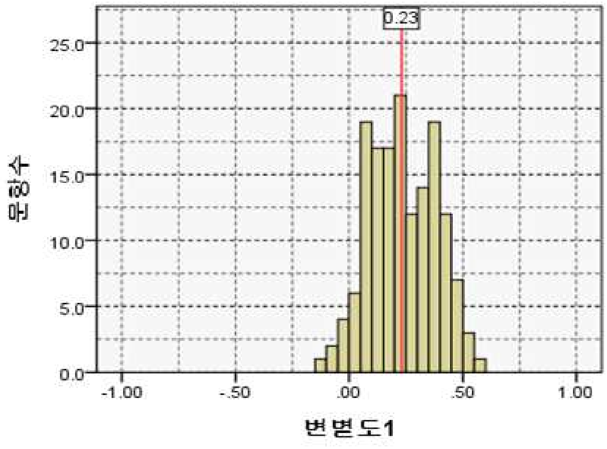

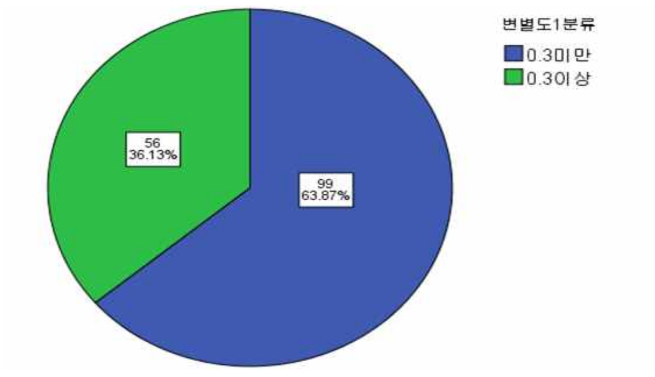

총점	변별도1	표준편차
155	.23	.15

변별도1	문항수	비율(%)
0.3미만	99	63.9
0.3이상	56	36.1
전체	155	100.0

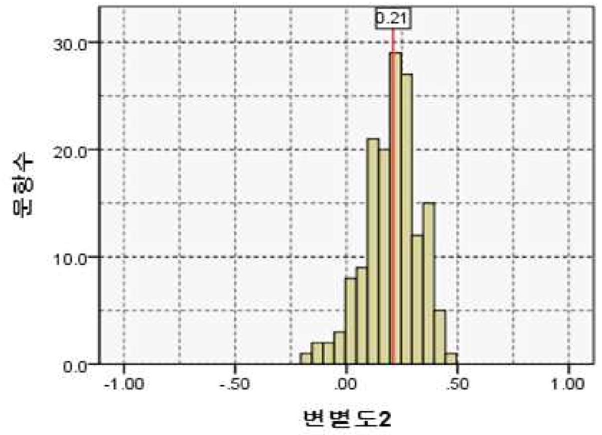

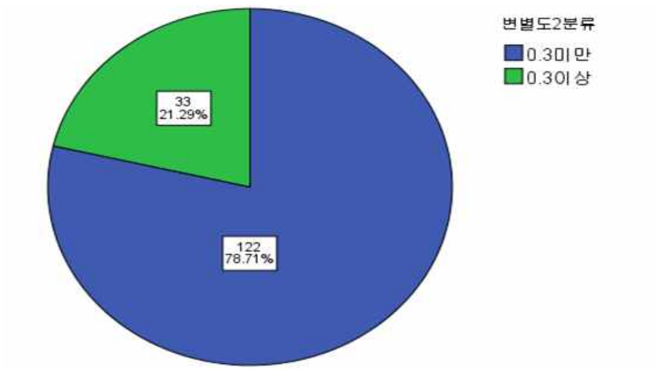

총점	변별도2	표준편차	변별도2	문항수	비율(%)
155	.21	.12	0.3미만	122	78.7
			0.3이상	33	21.3
			전체	155	100.0

해석

- 텍스트형 문항에서 난이도 지수가 60 미만인 문항과 60 이상 80 미만인 문항이 전체 155 문항 중 각각 53 문항으로 가장 많았으며, 80 에서 100 사이 문항은 49 문항인 것으로 나타남
- 변별도 1 지수를 기준으로 분류하였을 때, 0.3 미만인 문항이 99 문항으로 0.3 이상인 문항이 56 문항인 것에 비해 더 많이 나타남
- 변별도 2 지수를 기준으로 분류하였을 때, 0.3 미만인 문항이 122 문항으로 0.3 이상인 문항이 33 문항인 것에 비해 더 많이 나타남

(2) 자료제시형 난이도와 변별도 분포도 및 비율분석

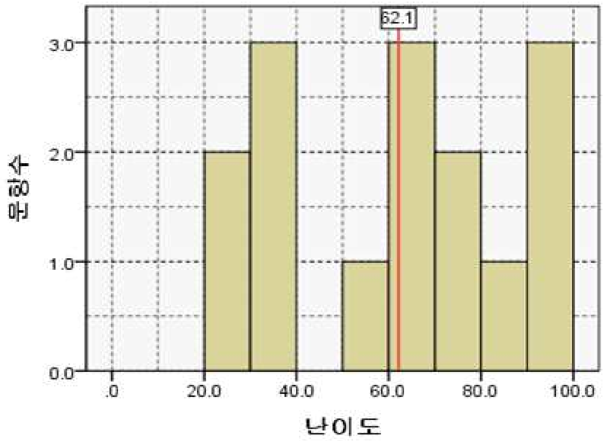

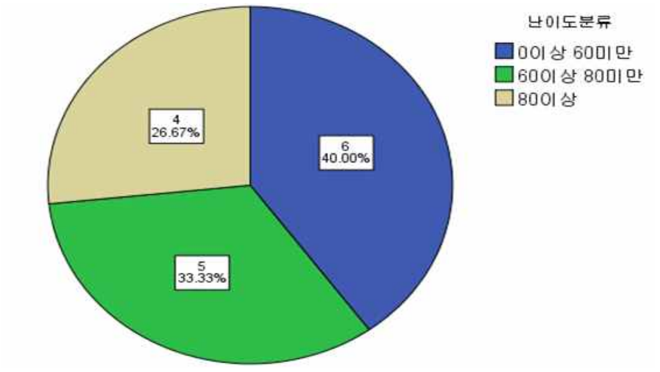

총점	난이도	표준편차
15	62.1	23.9

난이도	문항수	비율(%)
0~60미만	6	40.0
60~80미만	5	33.3
80~100	4	26.7
전체	15	100.0

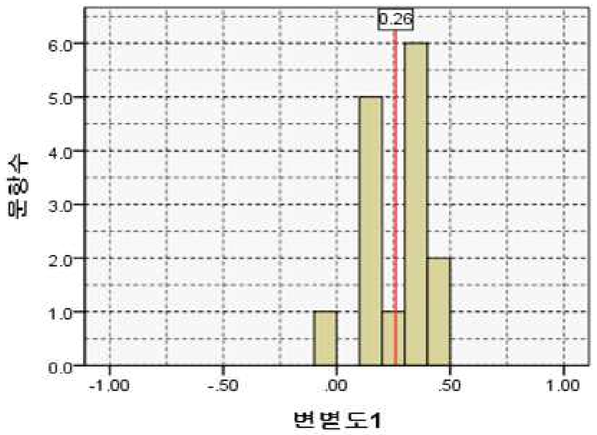

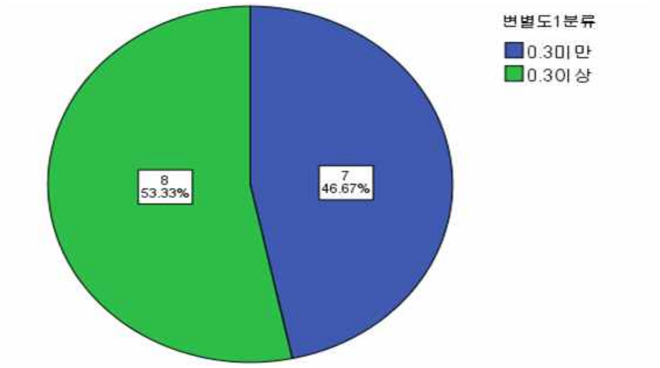

총점	변별도1	표준편차
15	.26	.15

변별도1	문항수	비율(%)
0.3미만	7	46.7
0.3이상	8	53.3
전체	15	100.0

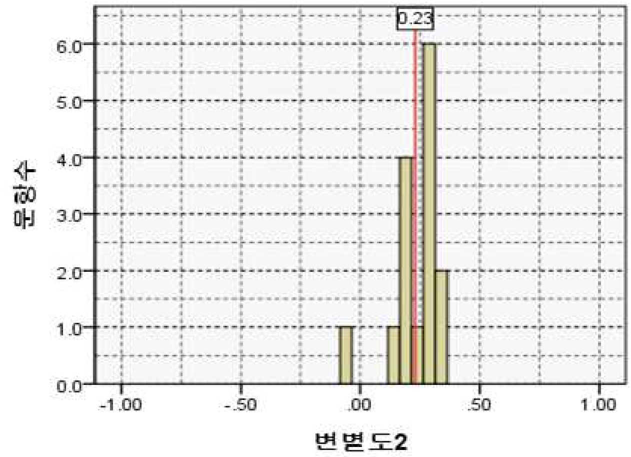

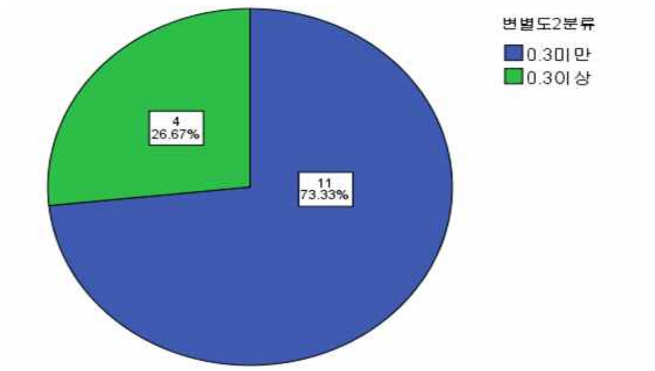

총점	변별도2	표준편차
15	.23	.10

변별도2	문항수	비율(%)
0.3미만	11	73.3
0.3이상	4	26.7
전체	15	100.0

해석

- 자료제시형 문항에서 난이도 지수가 60 미만인 문항이 전체 15 문항 중 6 문항으로 가장 많았으며, 차례로 60 이상 80 미만인 문항이 5 문항, 80 에서 100 사이인 문항이 4 문항인 것으로 나타남
- 변별도 1 지수를 기준으로 분류하였을 때, 0.3 미만인 문항이 7 문항으로 0.3 이상인 문항이 8 문항인 것에 비해 더 적게 나타남
- 변별도 2 지수를 기준으로 분류하였을 때, 0.3 미만인 문항이 11 문항으로 0.3 이상인 문항이 4 문항인 것에 비해 더 많이 나타남

3. 난이도와 변별도 간 산포도

1) 전체 난이도와 변별도 간 산포도

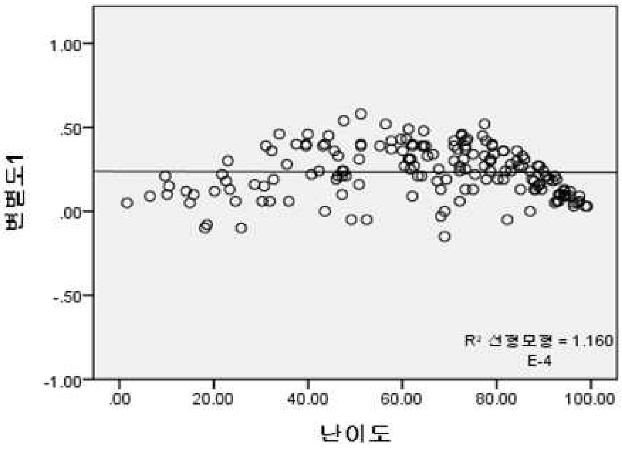

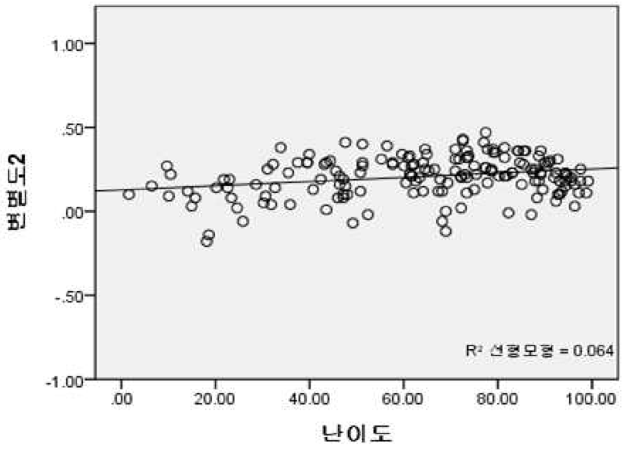

해석

- 전체 문항을 대상으로 난이도와 변별도 1 지수 간 상관은 -.011 로 관련성이 없는 것으로 나타남
- 난이도와 변별도 2 지수 간 상관은 .253**으로 난이도 지수가 높을수록 변별력이 높아지는 것으로 나타남

2) 과목별 난이도와 변별도 간 산포도

가) 보조공학사 기초 난이도와 변별도 간 산포도

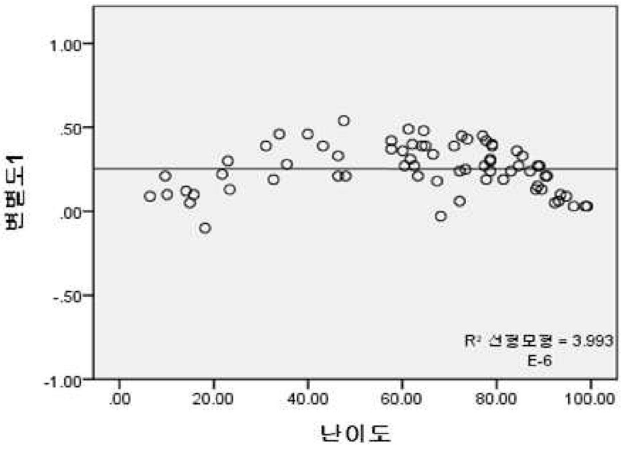

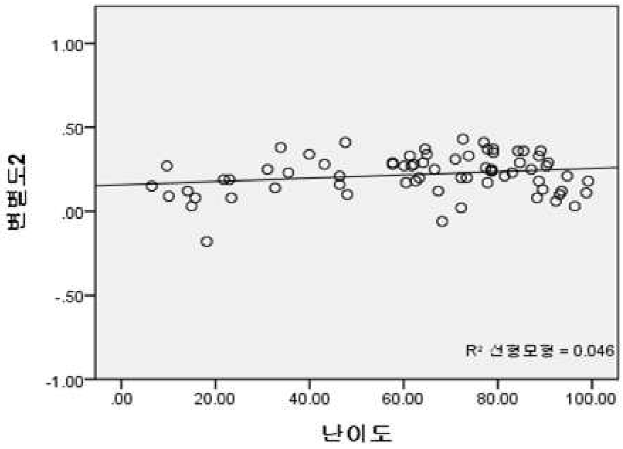

해석
- 보조공학사 기초 과목 문항을 대상으로 난이도와 변별도 1 지수 간 상관은 -.002 로 문항 난이도와 변별력 간 관련성이 없는 것으로 나타남<br>- 난이도와 변별도 2 지수 간 상관은 .213 으로 문항 난이도와 변별력 간 관련성이 없는 것으로 나타남

나) 보조공학사 응용·실기 난이도와 변별도 간 산포도

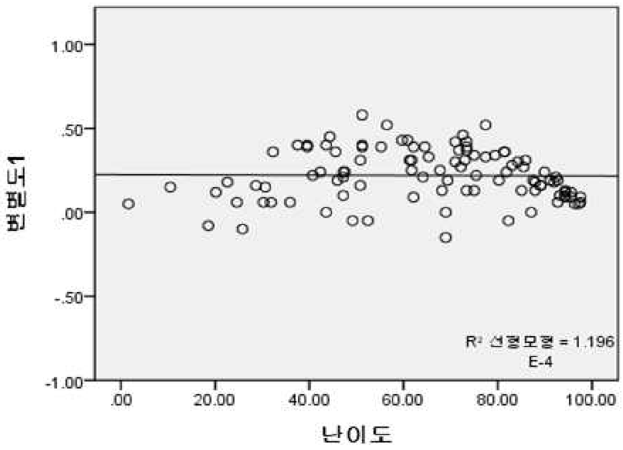

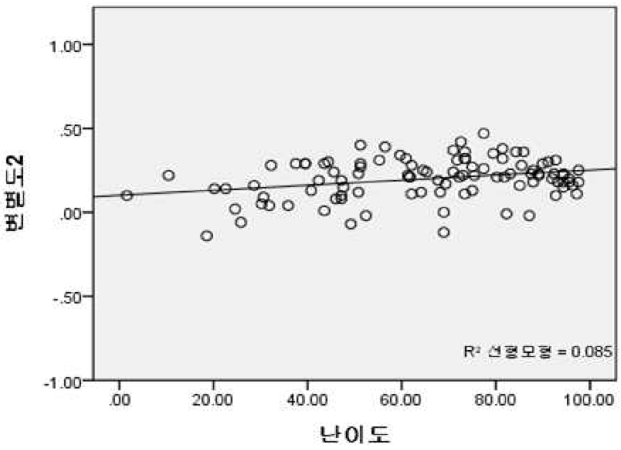

해석
- 보조공학사 응용·실기 과목 문항을 대상으로 난이도와 변별도 1 지수 간 상관은 -.011 로 문항 난이도와 변별력 간 관련성이 없는 것으로 나타남<br>- 난이도와 변별도 2 지수 간 상관은 .292**로 난이도 지수가 높을수록 변별력이 높아지는 것으로 나타남

4. 신뢰도 분석

과목명	문항수	제1회	제2회	제3회	제4회
전체	170	.871	.829	.893	**.893**
보조공학사 기초	70	.754	.609	.767	**.809**
보조공학사 응용·실기	100	.800	.769	.840	**.817**

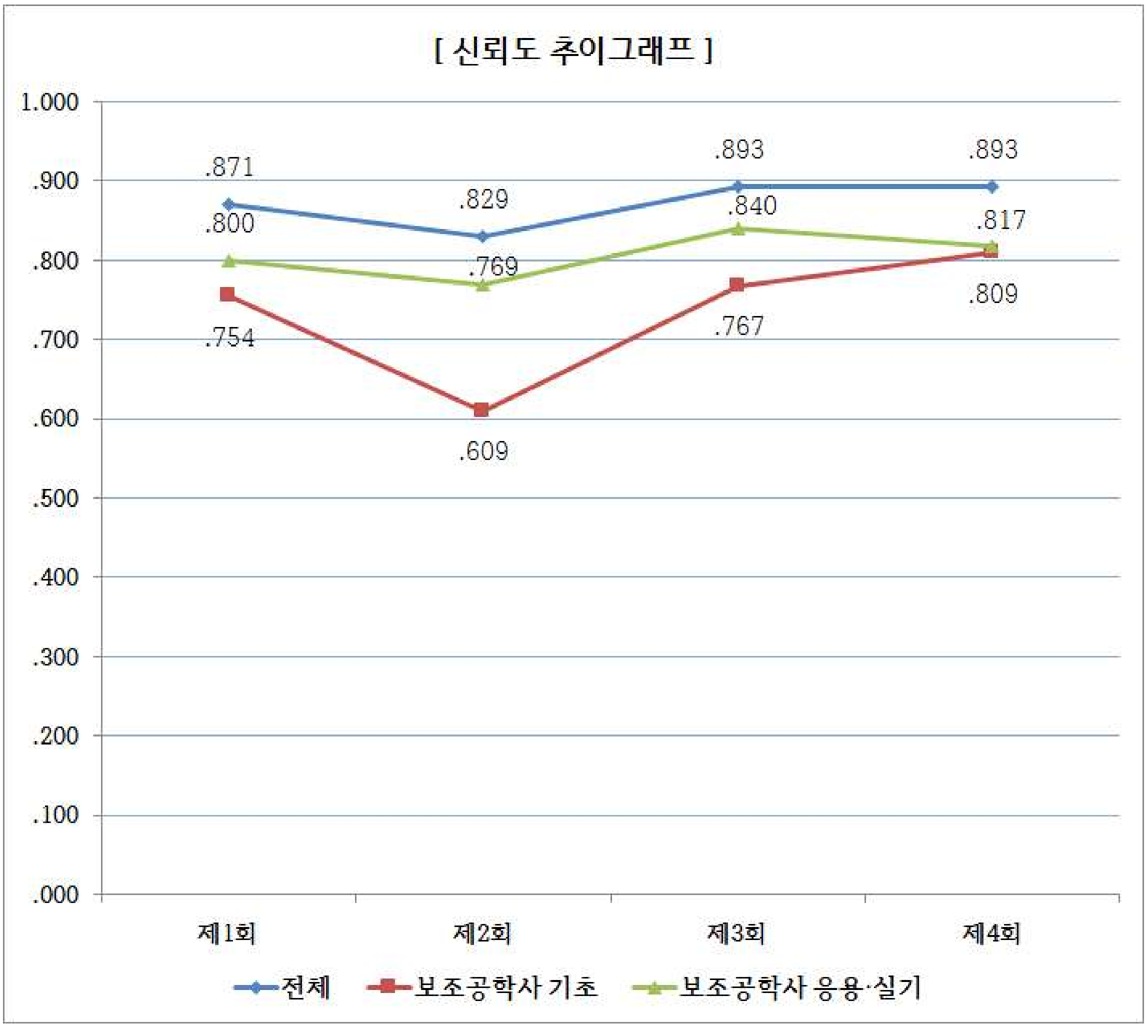

해석

- 보조공학사 국가시험 전체, 보조공학사 기초 과목, 보조공학사 응용·실기 과목의 문항 신뢰도가 각각 .893, .809, .817 로 모두 일관되게 해당 영역을 측정하고 있는 것으로 나타남

○ 분석결과 관련 문의 : 한국보건의료인국가시험원 연구개발본부 박경은 주임
Tel : 02-2087-8953, FAX : 02-2087-8885
E-mail : kyung0511@kuksiwon.or.kr
