# Supplementary material for: Item difficulty index, discrimination index, and reliability of the 26 health professions licensing examinations in 2022, Korea: a psychometric study
Source: J Educ Eval Health Prof. 2023 Nov 22;20:31. doi: 10.3352/jeehp.2023.20.31 (PMC11959405; doi:10.3352/jeehp.2023.20.31)
Supplement: Supplementary file 1 — Supplement 1. Item analysis results of 26 health professions licensing examinations administered during late 2022 and early 2023. [file jeehp-20-31_Suppl1.zip › 2022│Γ╡╡ ┴a50╚╕ ╣░╕«─í╖ß╗τ ▒╣░í╜├╟Φ ║╨╝«░ß░·.pdf]

# 2022년도 제50회 물리치료사 국가시험 문항분석 결과

국민이 신뢰하고 감동하는 시험평가기관
한국보건의료인국가시험원
KOREA HEALTH PERSONNEL LICENSING EXAMINATION INSTITUTE

# 일반 용어 정의

□ **평균**

- 집단에서의 대표적 경향값으로 전체 값을 더하여 총 응시자로 나눈 값

□ **표준편차**

- 평균과 각 점수의 차이인 편차들의 평균으로 점수가 흩어져 분포되어 있는 정도

□ **추정난이도**

- 문항개발자가 예측한 정답률

□ **검사이론**

- 검사와 검사를 구성하고 있는 문항의 양호도를 분석 및 평가하는 방법을 정의한 이론체계
- 대표적으로 고전검사이론과 문항반응이론이 있음

## 고전검사이론 용어 정의

□ **고전검사이론(Classical Test Theory; CTT)**

- 검사의 질을 분석하는 검사이론 중 한 가지로 19세기 말부터 전개되어 현재까지 주로 사용되고 있는 검사이론임
- 고전검사이론에 의한 문항과 응시자 능력 추정치는 다음과 같음

○ **문항난이도**

· 검사 문항의 쉽고 어려운 정도를 나타내는 지수
· 난이도 지수는 총 반응 수에 대한 정답 반응 수의 비율로 문항의 정답률임
· 문항난이도는 0~100까지의 값을 가짐
· 난이도 값이 큰 경우, 쉬운 문항으로 '난이도가 낮다'라고 해석하며, 난이도 값이 작은 경우, 어려운 문항으로 '난이도가 높다'라고 해석함

○ **문항변별도**

· 각 문항이 응시자의 능력 수준을 변별할 수 있는 정도를 나타내는 지수
· 문항변별도는 -1~+1까지의 값을 가지며, 1에 가까울수록 변별력 크다고 해석함
· 일반적으로 문항변별도가 0.3 이상이면 우수한 문항으로 평가함
· 구하는 방식에는 '상하위집단 구분법', '문항-총점 상관계수' 등이 있음
  1) **변별도 1(상하위구분법):** 상위 27%와 하위 27% 집단의 난이도 차이를 구하는 방식
  2) **변별도 2(상관계수법):** 문항-총점과의 상관계수로 구하는 방식

○ **신뢰도**

· 시험이 평가하고자 하는 것을 일관성 있게 측정하는가로 시험이 오차없이 정확하게 측정한 정도를 의미함
· 국시원에서는 문항의 내적일관성(Cronbach $\alpha$)으로 신뢰도를 추정하며 1에 가까울수록 신뢰도가 높다고 해석함

# 목 차


I. 시행 결과 ········ 6

**1. 시험 현황** ········ **7**

1) 시험명 ········ 7

2) 시험시행일 ········ 7

3) 응시현황 ········ 7

4) 과목별 문항 수, 배점 및 과락 점수 ········ 7

**2. 합격률과 평균성적** ········ **7**

1) 합격 및 불합격 현황 ········ 7

2) 과목별 과락자수 내역 ········ 7

3) 전회 대비 합격률과 평균성적 ········ 8

II. 문항분석 결과 ········ 10

**1. 성적** ········ **11**

1) 전체 성적분포도 ········ 11

2) 과목별 성적분포도 ········ 12

**2. 난이도와 변별도** ········ **14**

1) 전체 난이도와 변별도 ········ 14

2) 과목별 난이도와 변별도 ········ 17

3) 지식수준별 난이도와 변별도 ········ 32

**3. 난이도와 변별도 간 산포도** ········ **40**

1) 전체 난이도와 변별도 간 산포도 ········ 40

2) 과목별 난이도와 변별도 간 산포도 ········ 40

**4. 신뢰도 분석** ········ **44**

# I. 시행 결과

## 1. 시험 현황

1) 시험명: 2022년도 제50회 물리치료사 국가시험

2) 시험시행일: 2022년 12월 11일

3) 응시현황

| 응시대상자수 | 결시자수 | 부정행위자수 | 응시자 준수사항 위반자 수 | | 응시자수 (%) |
|---|---|---|---|---|---|
| | | | 휴대폰 소지 | 신분증 미지참 | |
| 5,542 | 111 | 0 | 0 | 0 | 5,430※ (98.0) |

※ 응시대상자수(5,542)에서 결시자수(111) 및 채점보류자수(1)를 제외한 수치임

4) 과목별 문항 수, 배점 및 과락 점수

| 교 시 | 과 목 명 | 문제 수 | 배점 | 총점 | 합격자 점수기준 | |
|---|---|---|---|---|---|---|
| | | | | | 과목 과락기준 | ※총점 합격기준 |
| 1교시 | 물리치료 기초 | 60 | 1 | 60 | 24점 미만 | 114점 이상 |
| 1교시 | 물리치료 진단평가 | 45 | 1 | 45 | 18점 미만 | |
| 2교시 | 물리치료 중재 | 65 | 1 | 65 | 26점 미만 | |
| 2교시 | 의료관계법규 | 20 | 1 | 20 | 8점 미만 | |
| 3교시 | 실기시험 | 70 | 1 | 60 | 42점 미만 | 42점 이상 |
| 계 | | 260 | | | | |

※ 필기시험에서 각 과목 만점의 40% 이상, 전 과목 총점의 60% 이상 득점. 실기시험에서 만점의 60% 이상 득점.

## 2. 합격률과 평균성적

1) 합격 및 불합격 현황

| 합격자수 (%) | 불합격자수(%) | | | | | 채점보류자수 |
|---|---|---|---|---|---|---|
| | 평락 | 과락 | 실기탈락 | 기권 | 계 | |
| 4,677 (86.1) | 676 (12.5) | 19 (0.4) | 56 (1.0) | 2 (0.0) | 753 (13.9) | 1 (0.0) |

2) 과목별 과락자수 내역

| 과목명 / 과락자수 | 물리치료 기초 | 물리치료 진단평가 | 물리치료 중재 | 의료관계법규 | 실기시험 |
|---|---|---|---|---|---|
| 과목별 과락자 수 | 0 | 0 | 0 | 19 | 0 |
| 전과목 과락자 수 | 19 | | | | |

3) 전회 대비 합격률과 평균성적

| 회차 | 년도 | 합격률(%) | 평균성적 | 표준편차 | 백분율 환산점수 |
|---|---|---|---|---|---|
| 제46회 | 2019 | 89.4 | 194.6 | 33.8 | 74.8 |
| 제47회 | 2019 | 85.8 | 184.2 | 35.7 | 70.9 |
| 제48회 | 2020 | 80.2 | 176.3 | 43.0 | 67.8 |
| 제49회 | 2021 | 83.7 | 183.8 | 43.3 | 70.7 |
| 제50회 | 2022 | 86.1 | 193.1 | 42.1 | 74.3 |

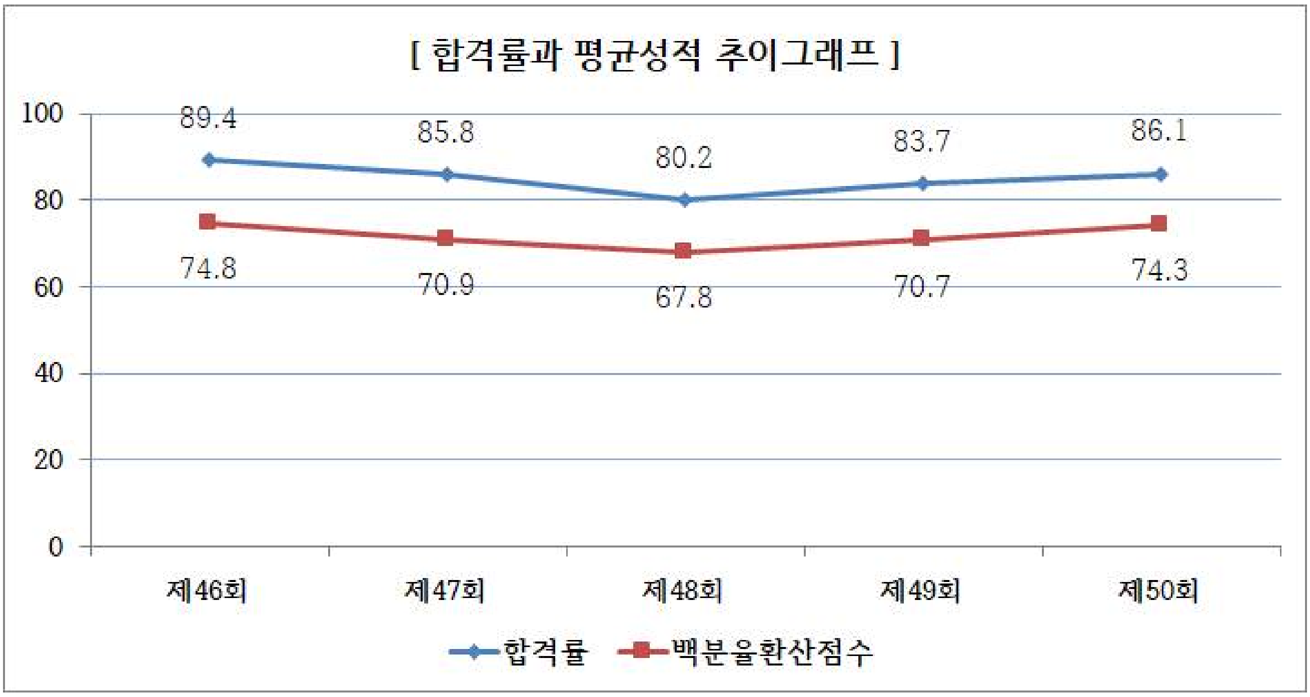


**해석**

- 전년 대비 합격률은 2.4% 증가하고, 백분율 환산점수는 3.6 점 증가함

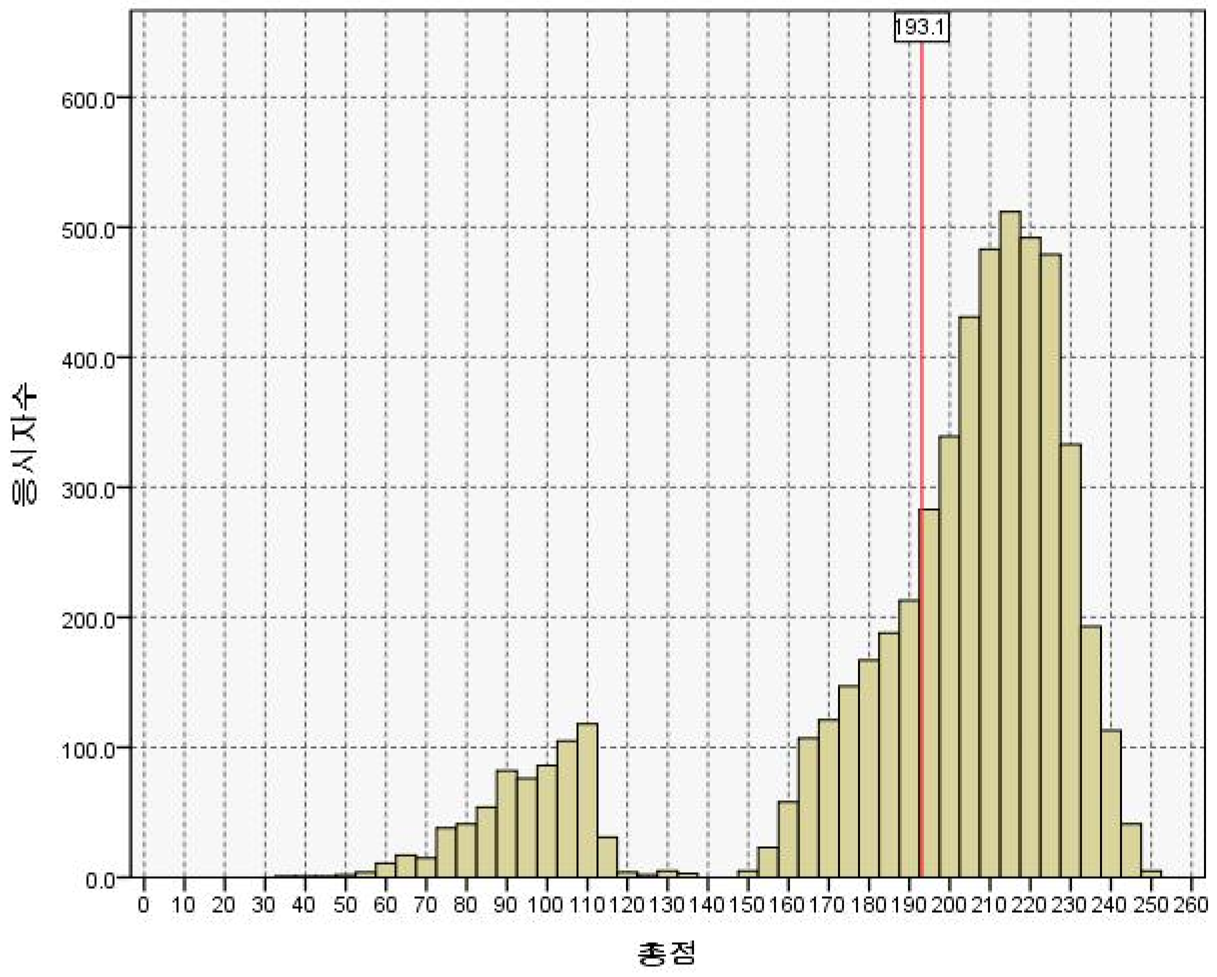


| 응시자 | 총점 | 합격선 | 평균성적 | 표준편차 |
|---|---|---|---|---|
| 5,431 | 260 | | 193.1 | 42.1 |

※ 필기시험 불합격자의 실기성적을 포함하지 않음

※ 채점보류자 1명을 포함함

# Ⅱ. 문항분석 결과

# 1. 성적

1) 전체 성적분포도

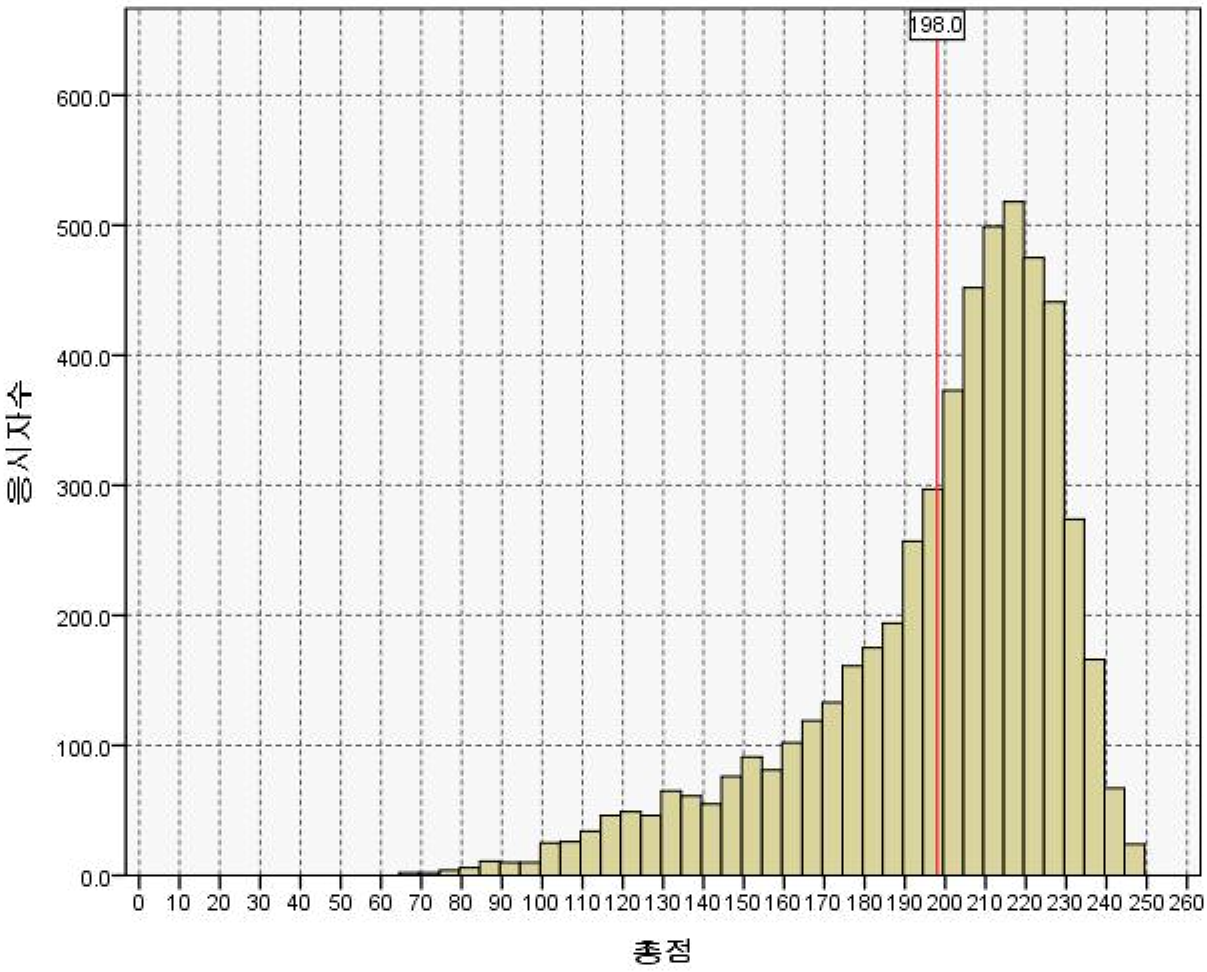


| 응시자 | 총점 | 합격선 | 평균성적 | 표준편차 |
|---|---|---|---|---|
| 5,429 | 260 | | 198.0 | 31.8 |

※ 필기시험 불합격자의 실기성적을 포함함

※ 기권자 2명을 제외함

2) 과목별 성적분포도

가) 물리치료 기초

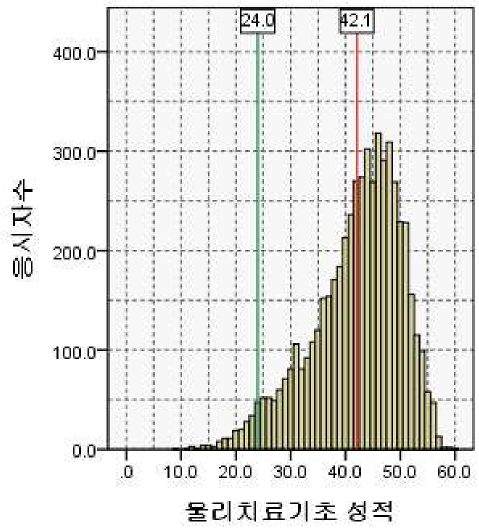


| 총점 | 과락선 | 평균성적 | 표준편차 |
|---|---|---|---|
| 60 | 24 | 42.1 | 8.1 |

나) 물리치료 진단평가

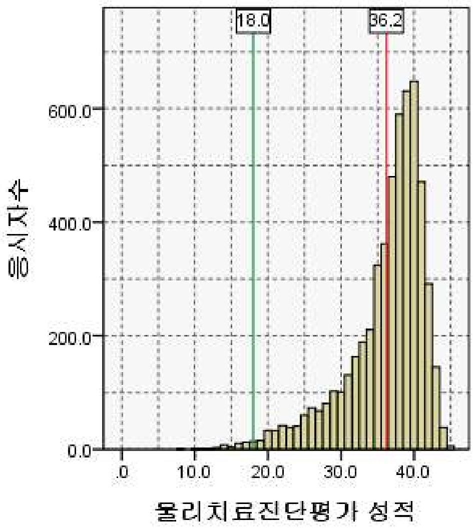


| 총점 | 과락선 | 평균성적 | 표준편차 |
|---|---|---|---|
| 45 | 18 | 36.2 | 5.4 |

다) 물리치료 중재

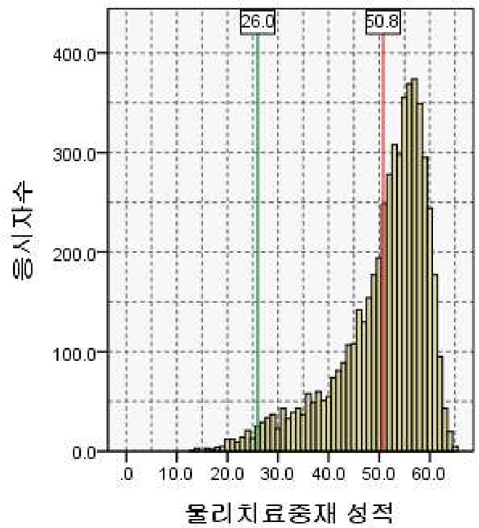


| 총점 | 과락선 | 평균성적 | 표준편차 |
|---|---|---|---|
| 65 | 26 | 50.8 | 9.0 |

라) 의료관계법규

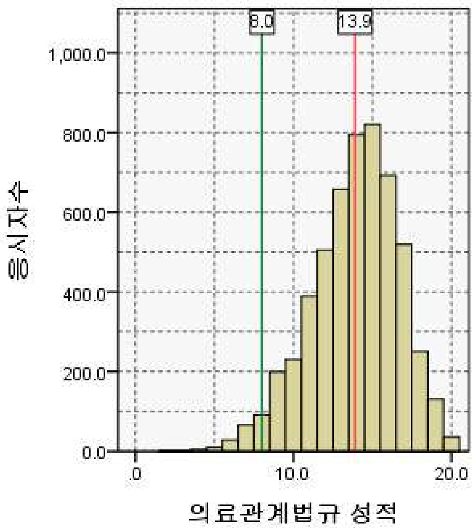


| 총점 | 과락선 | 평균성적 | 표준편차 |
|---|---|---|---|
| 20 | 8 | 13.9 | 2.8 |

마) 실기시험

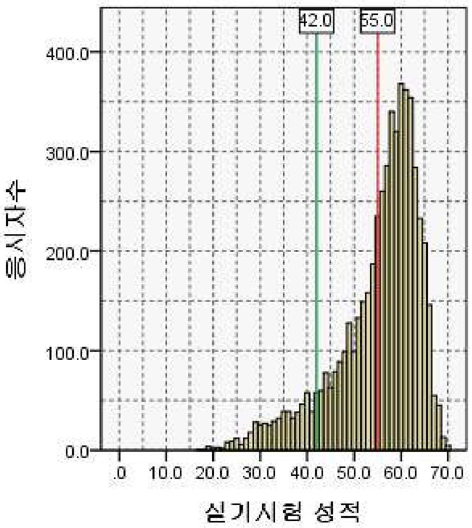


| 총점 | 과락선 | 평균성적 | 표준편차 |
|---|---|---|---|
| 70 | 42 | 55.0 | 9.1 |

## 2. 난이도와 변별도

### 1) 전체 난이도와 변별도

#### 가) 전회 대비 전체 난이도와 변별도

| 회차 | 난이도 | | 변별도1 | | 변별도2 | |
|---|---|---|---|---|---|---|
| | 평균 | 표준편차 | 평균 | 표준편차 | 평균 | 표준편차 |
| 제46회 | 75.9 | 27.0 | .24 | .13 | .27 | .10 |
| 제47회 | 72.5 | 19.7 | .24 | .12 | .26 | .11 |
| 제48회 | 71.0 | 20.4 | .27 | .13 | .28 | .11 |
| 제49회 | 73.3 | 20.3 | .27 | .14 | .30 | .12 |
| 제50회 | 76.1 | 18.1 | .28 | .14 | .32 | .14 |

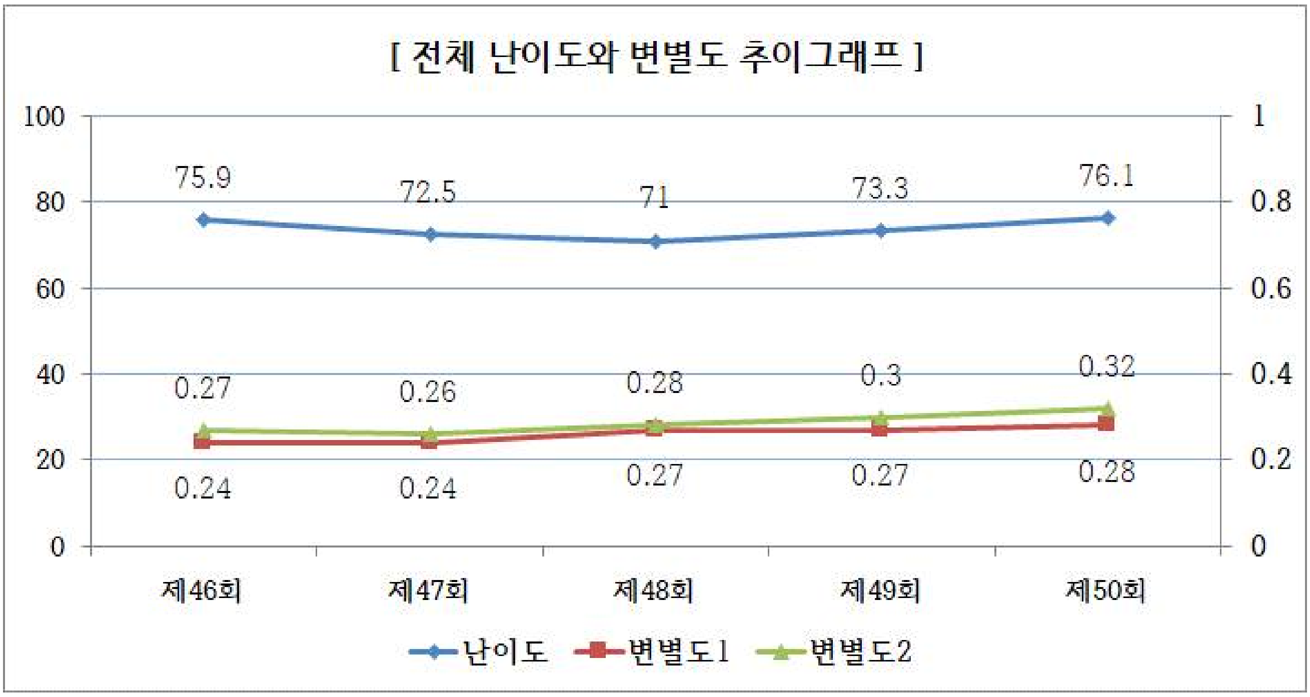


**해석**

- 전년 대비 난이도 지수는 2.8 증가함
- 변별도 1 지수는 .01 증가함
- 변별도 2 지수는 .02 증가함

## 나) 전체 난이도와 변별도 분포도 및 비율분석

### (1) 전체 난이도 분포도 및 비율분석

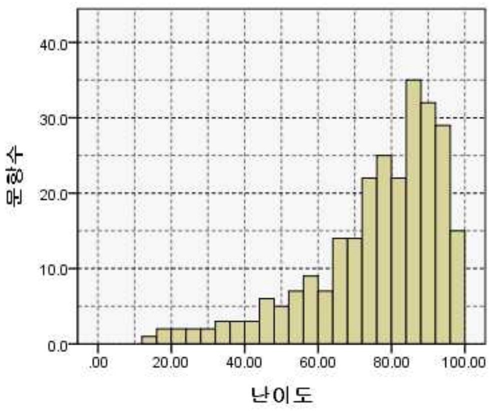


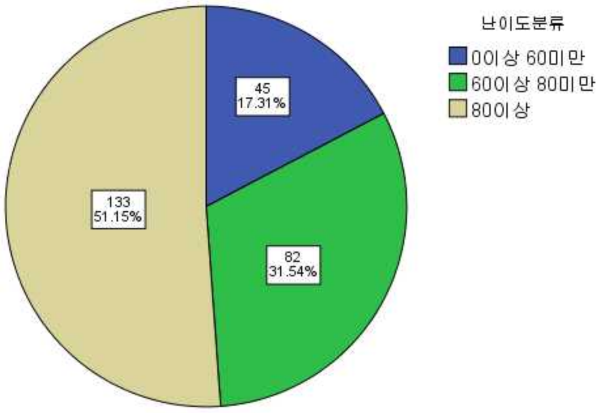


| 총점 | 난이도 | 표준편차 |
|---|---|---|
| 260 | 76.1 | 18.1 |

| 난이도 | 문항수 | 비율(%) |
|---|---|---|
| 0~60미만 | 45 | 17.3 |
| 60~80미만 | 82 | 31.5 |
| 80~100 | 133 | 51.2 |
| 전체 | 260 | 100.0 |

### (2) 전체 변별도1 분포도 및 비율분석

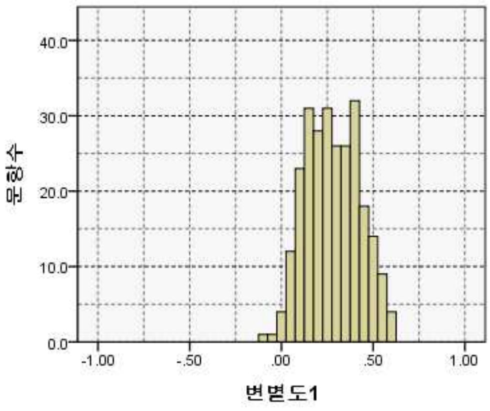


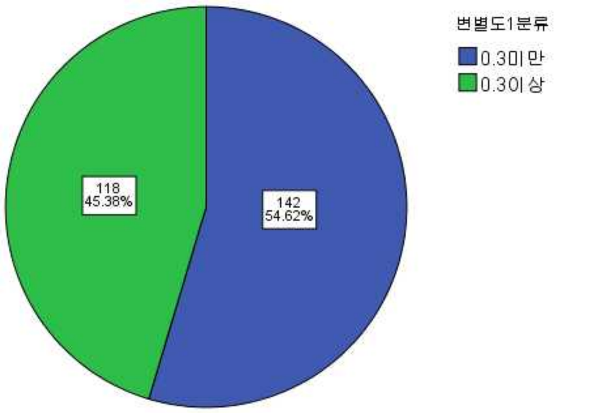


| 총점 | 변별도1 | 표준편차 |
|---|---|---|
| 260 | .28 | .14 |

| 변별도1 | 문항수 | 비율(%) |
|---|---|---|
| 0.3미만 | 142 | 54.6 |
| 0.3이상 | 118 | 45.4 |
| 전체 | 260 | 100.0 |

(3) 전체 변별도2 분포도 및 비율분석

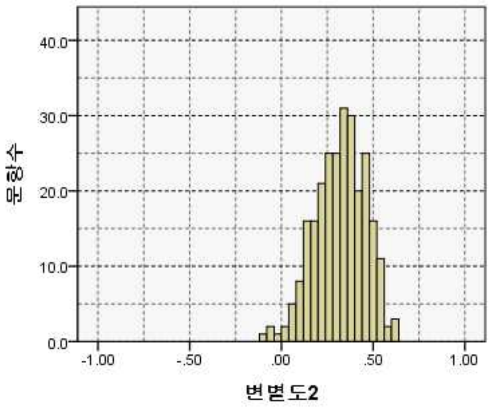


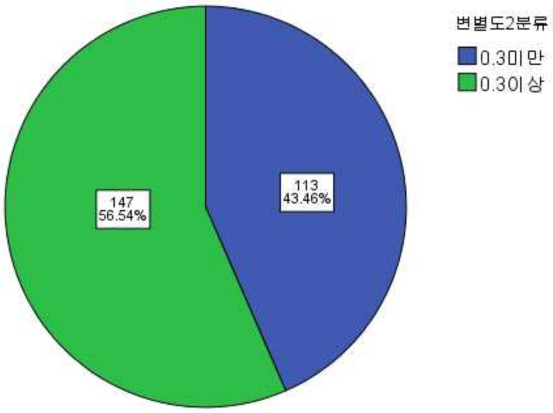


| 총점 | 변별도2 | 표준편차 | 변별도2 | 문항수 | 비율(%) |
|---|---|---|---|---|---|
| 260 | .32 | .14 | 0.3미만 | 113 | 43.5 |
| | | | 0.3이상 | 147 | 56.5 |
| | | | 전체 | 260 | 100.0 |

**해석**

- 난이도 지수가 80에서 100사이인 문항이 전체 260문항 중 133문항이었으며, 60이상 80미만인 문항이 82문항, 60미만인 문항이 45문항인 것으로 나타남
- 변별도1 지수를 기준으로 분류하였을 때, 0.3 미만인 문항이 142문항으로 0.3 이상인 문항이 118문항인 것에 비해 더 많이 나타남
- 변별도2 지수를 기준으로 분류하였을 때, 0.3 미만인 문항이 113문항으로 0.3 이상인 문항이 147문항인 것에 비해 더 적게 나타남

## 2) 과목별 난이도와 변별도

### 가) 전회 대비 과목별 난이도와 변별도

#### (1) 전회 대비 물리치료 기초 난이도와 변별도

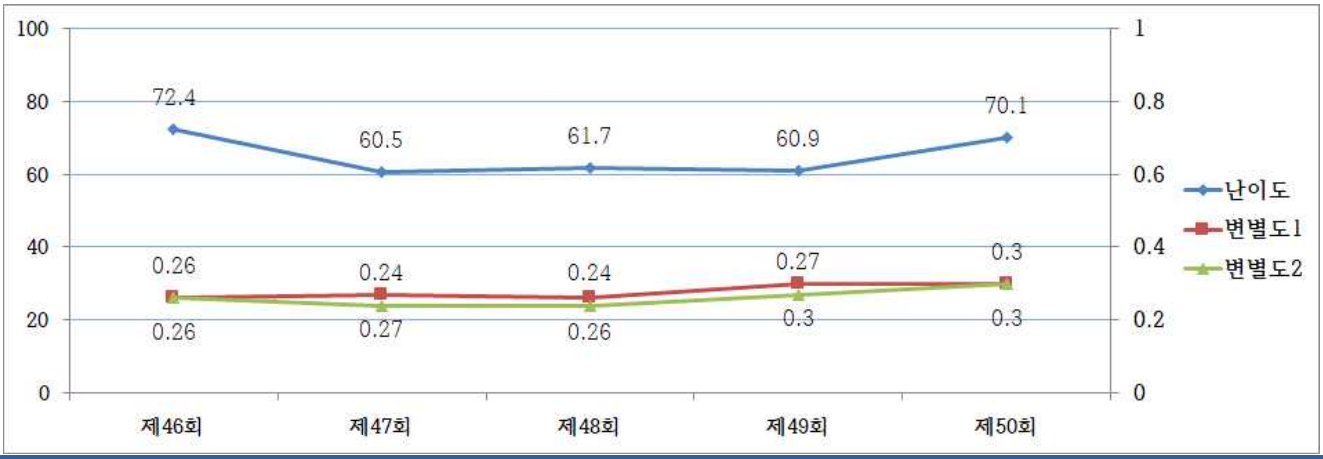


| 회차 | 난이도 | | 변별도1 | | 변별도2 | |
|---|---|---|---|---|---|---|
| | 평균 | 표준편차 | 평균 | 표준편차 | 평균 | 표준편차 |
| 제46회 | 72.4 | 18.8 | .26 | .12 | .26 | .10 |
| 제47회 | 60.5 | 20.7 | .27 | .12 | .24 | .10 |
| 제48회 | 61.7 | 23.2 | .26 | .12 | .24 | .11 |
| 제49회 | 60.9 | 21.9 | .30 | .14 | .27 | .12 |
| 제50회 | 70.1 | 20.0 | .30 | .15 | .30 | .13 |

**해석**

- 전회 대비 물리치료 기초 과목의 난이도 지수는 9.2 증가함
- 전회 대비 물리치료 기초 과목의 변별도 1 지수는 변하지 않음
- 전회 대비 물리치료 기초 과목의 변별도 2 지수는 .03 증가함

(2) 전회 대비 물리치료 진단평가 난이도와 변별도

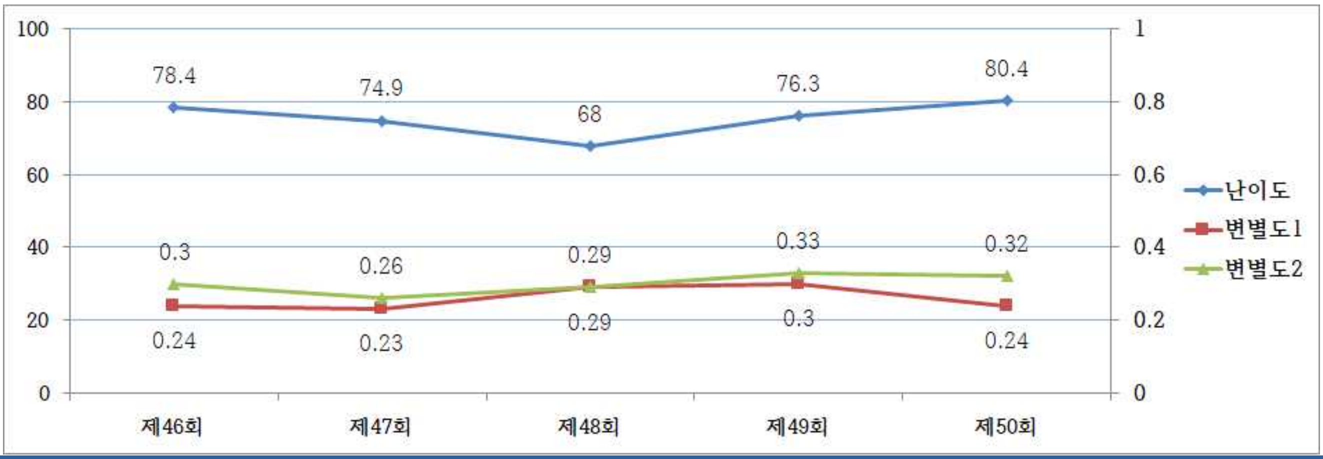


| 회차 | 난이도 | | 변별도1 | | 변별도2 | |
|---|---|---|---|---|---|---|
| | 평균 | 표준편차 | 평균 | 표준편차 | 평균 | 표준편차 |
| 제46회 | 78.4 | 18.0 | .24 | .13 | .30 | .08 |
| 제47회 | 74.9 | 17.8 | .23 | .11 | .26 | .11 |
| 제48회 | 68.0 | 19.5 | .29 | .13 | .29 | .11 |
| 제49회 | 76.3 | 16.8 | .30 | .15 | .33 | .12 |
| 제50회 | 80.4 | 16.8 | .24 | .15 | .32 | .16 |

**해석**

- 전회 대비 물리치료 진단평가 과목의 난이도 지수는 4.1 증가함
- 전회 대비 물리치료 진단평가 과목의 변별도 1 지수는 .06 감소함
- 전회 대비 물리치료 진단평가 과목의 변별도 2 지수는 .01 감소함

(3) 전회 대비 물리치료 중재 난이도와 변별도

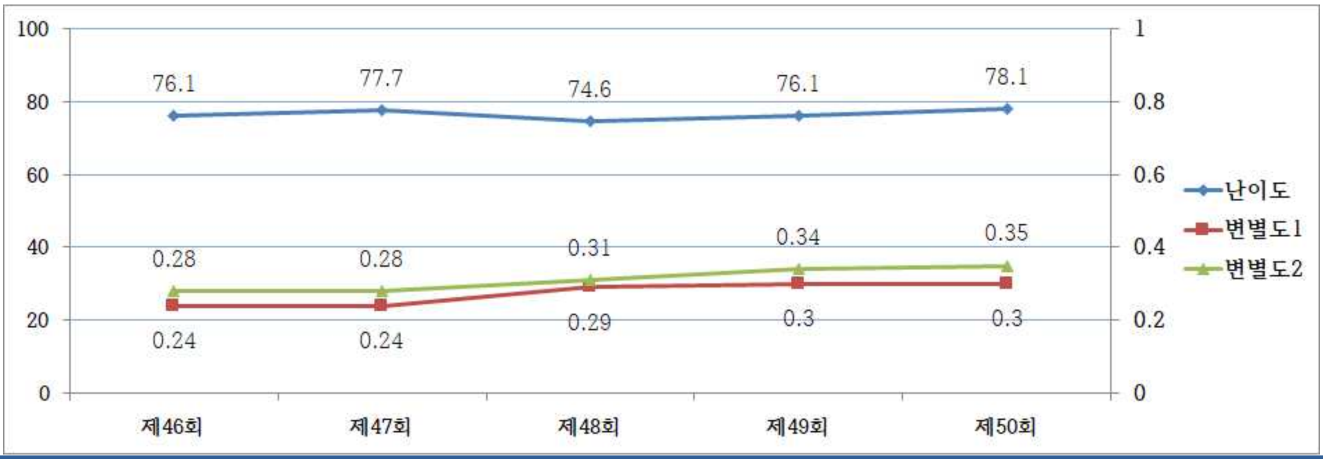


| 회차 | 난이도 | | 변별도1 | | 변별도2 | |
|---|---|---|---|---|---|---|
| | 평균 | 표준편차 | 평균 | 표준편차 | 평균 | 표준편차 |
| 제46회 | 76.1 | 19.3 | .24 | .13 | .28 | .09 |
| 제47회 | 77.7 | 17.4 | .24 | .13 | .28 | .10 |
| 제48회 | 74.6 | 17.0 | .29 | .14 | .31 | .11 |
| 제49회 | 76.1 | 16.9 | .30 | .13 | .34 | .11 |
| 제50회 | 78.1 | 15.9 | .30 | .13 | .35 | .12 |

**해석**

- 전회 대비 물리치료 중재 과목의 난이도 지수는 2.0 증가함
- 전회 대비 물리치료 중재 과목의 변별도 1 지수는 변하지 않음
- 전회 대비 물리치료 중재 과목의 변별도 2 지수는 .01 증가함

(4) 전회 대비 의료관계법규 난이도와 변별도

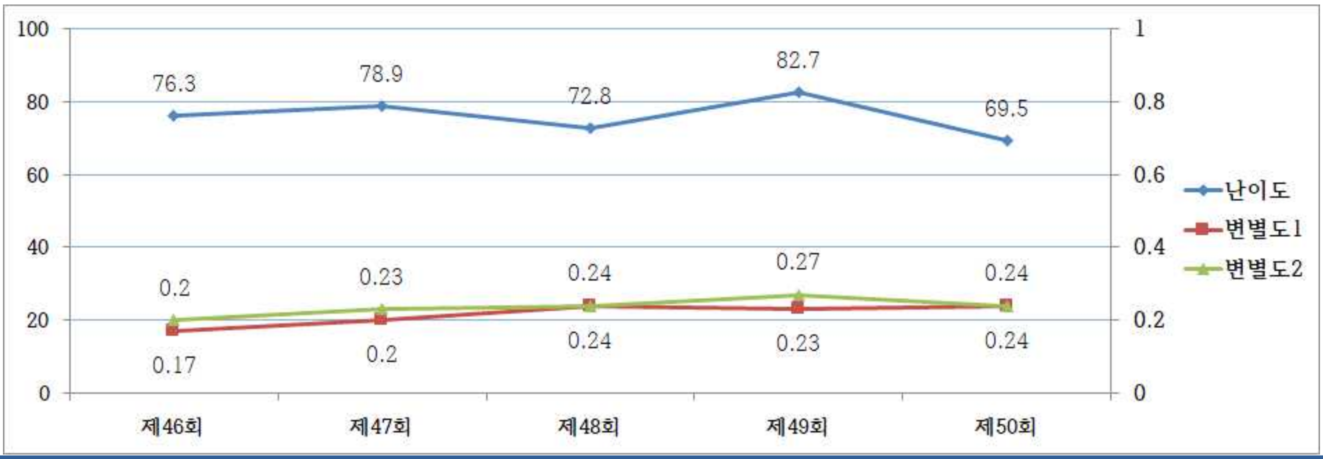


| 회차 | 난이도 | | 변별도1 | | 변별도2 | |
|---|---|---|---|---|---|---|
| | 평균 | 표준편차 | 평균 | 표준편차 | 평균 | 표준편차 |
| 제46회 | 76.3 | 19.1 | .17 | .10 | .20 | .10 |
| 제47회 | 78.9 | 15.8 | .20 | .11 | .23 | .11 |
| 제48회 | 72.8 | 20.9 | .24 | .11 | .24 | .08 |
| 제49회 | 82.7 | 14.3 | .23 | .12 | .27 | .07 |
| 제50회 | 69.5 | 23.6 | .24 | .15 | .24 | .12 |

**해석**

- 전회 대비 의료관계법규 과목의 난이도 지수는 13.2 감소함
- 전회 대비 의료관계법규 과목의 변별도 1 지수는 .01 증가함
- 전회 대비 의료관계법규 과목의 변별도 2 지수는 .03 감소함

(5) 전회 대비 실기시험 난이도와 변별도

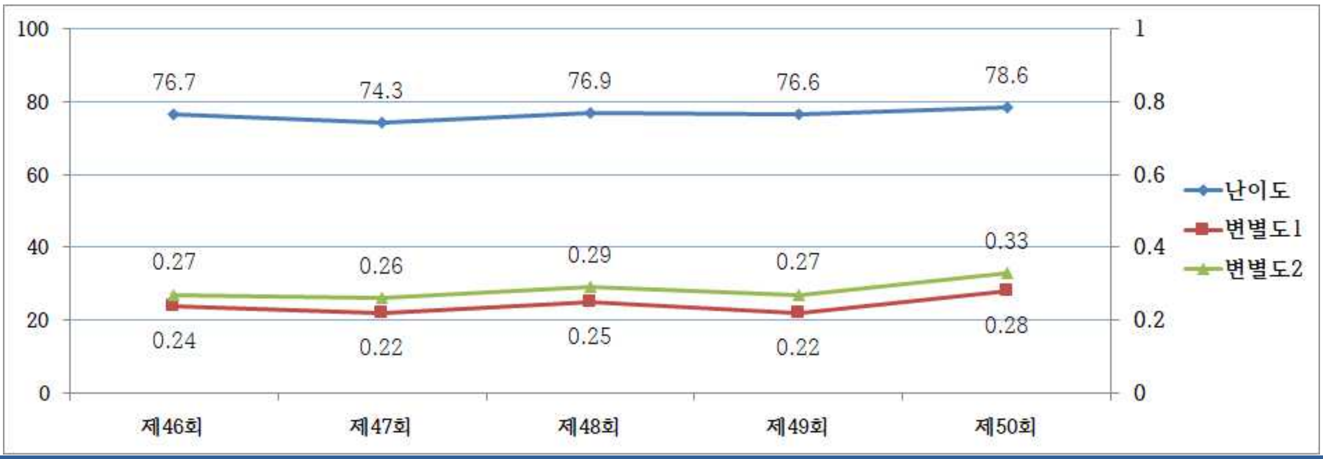


| 회차 | 난이도 | | 변별도1 | | 변별도2 | |
|---|---|---|---|---|---|---|
| | 평균 | 표준편차 | 평균 | 표준편차 | 평균 | 표준편차 |
| 제46회 | 76.7 | 17.4 | .24 | .14 | .27 | .11 |
| 제47회 | 74.3 | 19.7 | .22 | .13 | .26 | .11 |
| 제48회 | 76.9 | 18.1 | .25 | .14 | .29 | .11 |
| 제49회 | 76.6 | 21.3 | .22 | .12 | .27 | .12 |
| 제50회 | 78.6 | 16.0 | .28 | .14 | .33 | .14 |

**해석**

- 전회 대비 실기시험 과목의 난이도 지수는 2.0 증가함
- 전회 대비 실기시험 과목의 변별도 1 지수는 .06 증가함
- 전회 대비 실기시험 과목의 변별도 2 지수는 .06 증가함

## 나) 과목별 난이도와 변별도 분포도 및 비율분석

### (1) 물리치료 기초 난이도와 변별도 분포도 및 비율분석

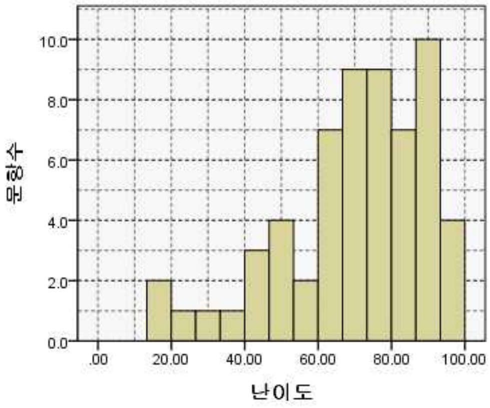


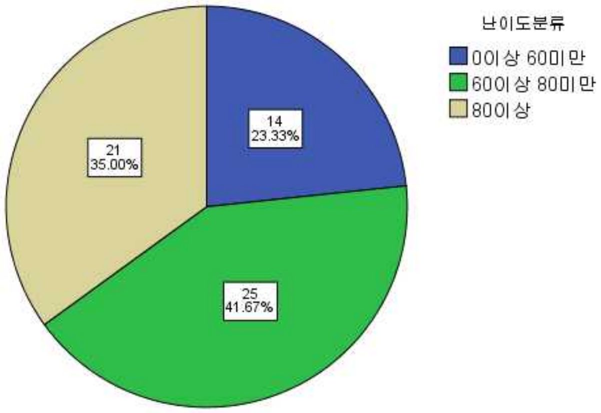


| 총점 | 난이도 | 표준편차 |
|---|---|---|
| 60 | 70.1 | 20.0 |

| 난이도 | 문항수 | 비율(%) |
|---|---|---|
| 0~60미만 | 14 | 23.3 |
| 60~80미만 | 25 | 41.7 |
| 80~100 | 21 | 35.0 |
| 전체 | 60 | 100.0 |

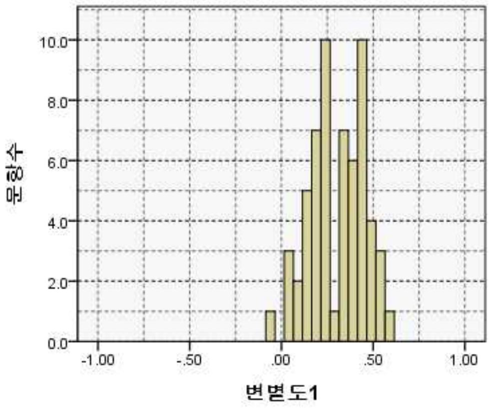


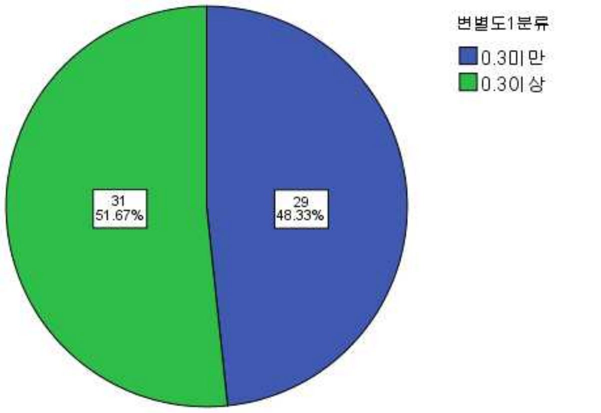


| 총점 | 변별도1 | 표준편차 |
|---|---|---|
| 60 | .30 | .15 |

| 변별도1 | 문항수 | 비율(%) |
|---|---|---|
| 0.3미만 | 29 | 48.3 |
| 0.3이상 | 31 | 51.7 |
| 전체 | 60 | 100.0 |

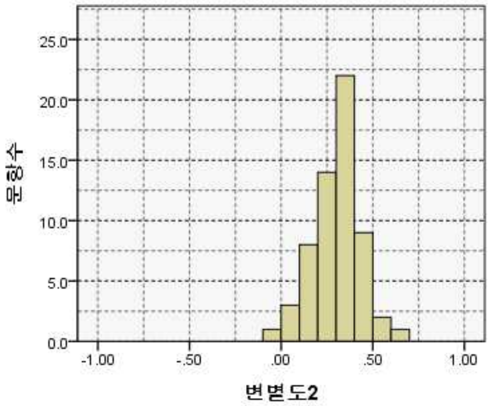


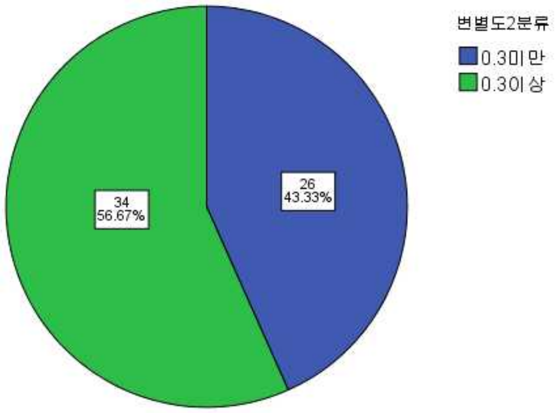


| 총점 | 변별도2 | 표준편차 |
|---|---|---|
| 60 | .30 | .13 |

| 변별도2 | 문항수 | 비율(%) |
|---|---|---|
| 0.3미만 | 26 | 43.3 |
| 0.3이상 | 34 | 56.7 |
| 전체 | 60 | 100.0 |

**해석**

- 물리치료 기초 과목에서 난이도 지수가 80에서 100 사이인 문항이 전체 60문항 중 21문항이었으며, 60이상 80미만인 문항이 25문항, 60 미만인 문항이 14문항으로 나타남
- 변별도1 지수를 기준으로 분류하였을 때, 0.3미만인 문항이 29문항으로 0.3 이상인 문항이 31문항인 것에 비해 더 적게 나타남
- 변별도2 지수를 기준으로 분류하였을 때, 0.3미만인 문항이 26문항으로 0.3 이상인 문항이 34문항인 것에 비해 더 적게 나타남

(2) 물리치료 진단평가 난이도와 변별도 분포도 및 비율분석

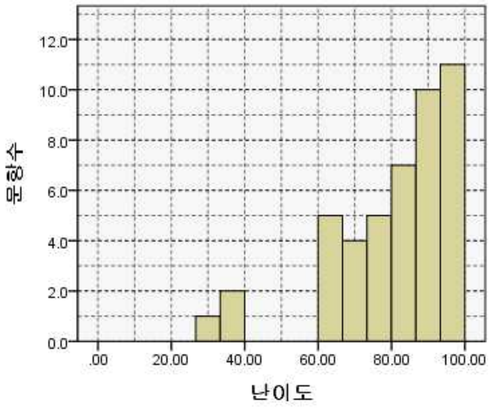


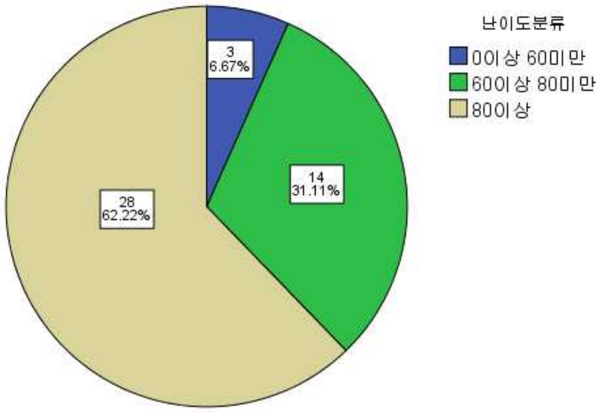


| 총점 | 난이도 | 표준편차 |
|---|---|---|
| 45 | 80.4 | 16.8 |

| 난이도 | 문항수 | 비율(%) |
|---|---|---|
| 0~60미만 | 3 | 6.7 |
| 60~80미만 | 14 | 31.1 |
| 80~100 | 28 | 62.2 |
| 전체 | 45 | 100.0 |

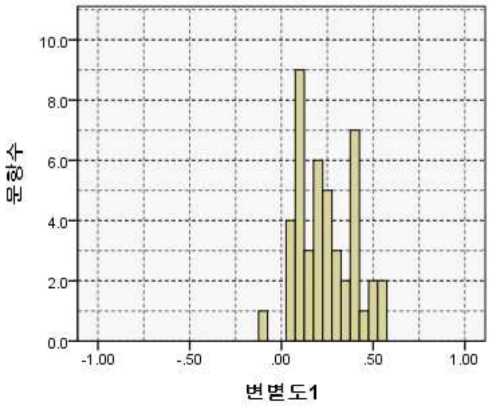


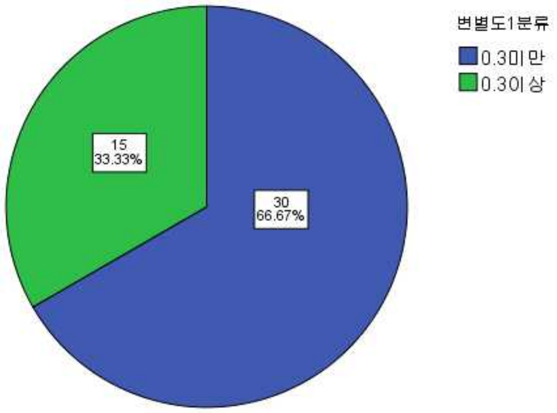


| 총점 | 변별도1 | 표준편차 |
|---|---|---|
| 45 | .24 | .15 |

| 변별도1 | 문항수 | 비율(%) |
|---|---|---|
| 0.3미만 | 30 | 66.7 |
| 0.3이상 | 15 | 33.3 |
| 전체 | 45 | 100.0 |

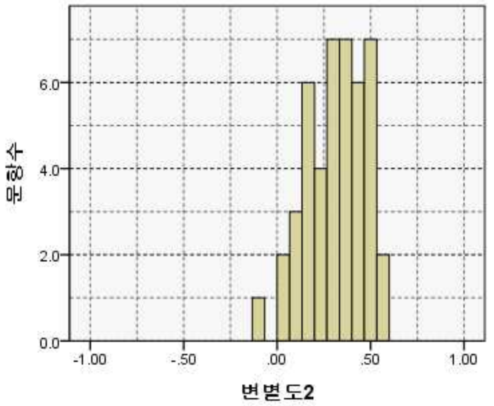


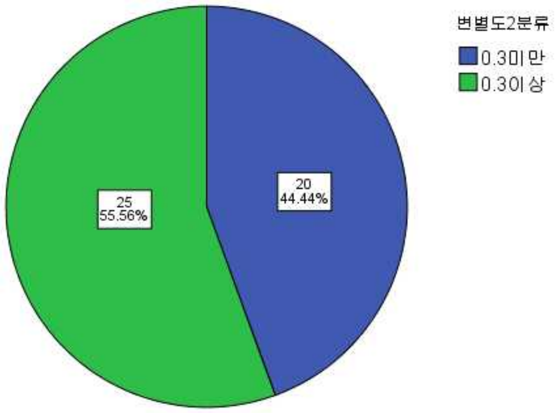


| 총점 | 변별도2 | 표준편차 | 변별도2 | 문항수 | 비율(%) |
|---|---|---|---|---|---|
| 45 | .32 | .16 | 0.3미만 | 20 | 44.4 |
| | | | 0.3이상 | 25 | 55.6 |
| | | | 전체 | 45 | 100.0 |

**해석**

- 물리치료 진단평가 과목에서 난이도 지수가 80 에서 100 사이인 문항이 전체 45 문항 중 28 문항이었으며, 60 이상 80 미만인 문항이 14 문항, 60 미만인 문항이 3 문항으로 나타남
- 변별도 1 지수를 기준으로 분류하였을 때, 0.3 미만인 문항이 30 문항으로 0.3 이상인 문항이 15 문항인 것에 비해 더 많이 나타남
- 변별도 2 지수를 기준으로 분류하였을 때, 0.3 미만인 문항이 20 문항으로 0.3 이상인 문항이 25 문항인 것에 비해 더 적게 나타남

(3) 물리치료 중재 난이도와 변별도 분포도 및 비율분석

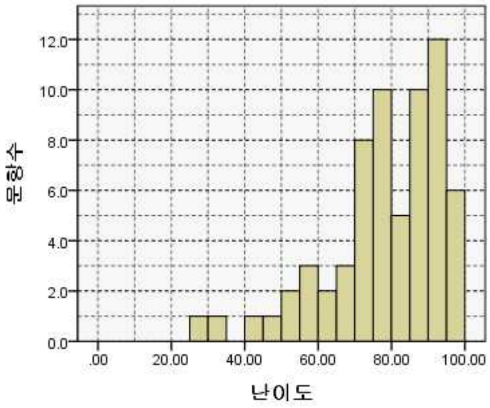


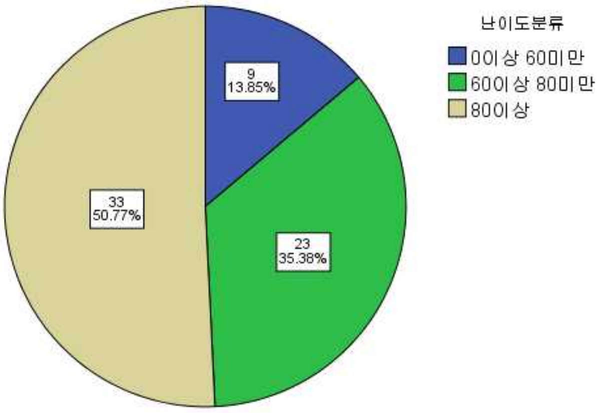


| 총점 | 난이도 | 표준편차 |
|---|---|---|
| 65 | 78.1 | 15.9 |

| 난이도 | 문항수 | 비율(%) |
|---|---|---|
| 0~60미만 | 9 | 13.8 |
| 60~80미만 | 23 | 35.4 |
| 80~100 | 33 | 50.8 |
| 전체 | 65 | 100.0 |

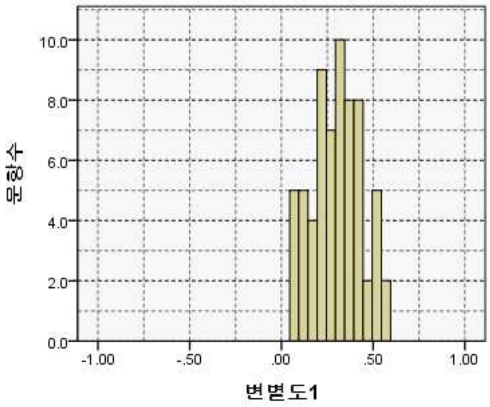


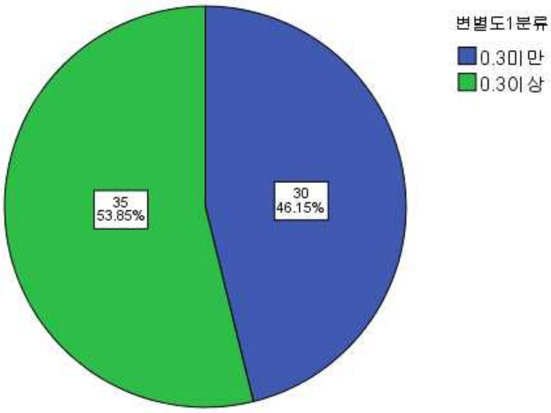


| 총점 | 변별도1 | 표준편차 |
|---|---|---|
| 65 | .30 | .13 |

| 변별도1 | 문항수 | 비율(%) |
|---|---|---|
| 0.3미만 | 30 | 46.2 |
| 0.3이상 | 35 | 53.8 |
| 전체 | 65 | 100.0 |

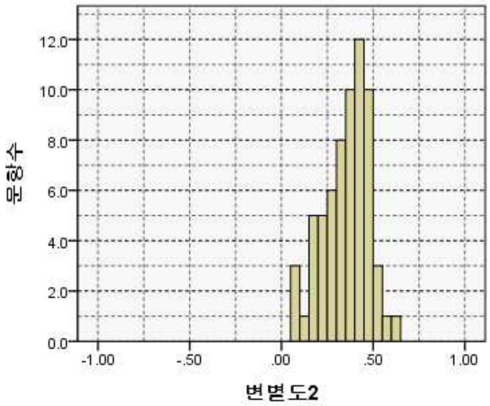


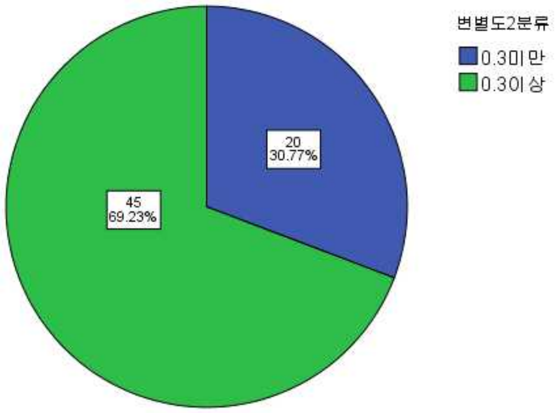


| 총점 | 변별도2 | 표준편차 |
|---|---|---|
| 65 | .35 | .12 |

| 변별도2 | 문항수 | 비율(%) |
|---|---|---|
| 0.3미만 | 20 | 30.8 |
| 0.3이상 | 45 | 69.2 |
| 전체 | 65 | 100.0 |

**해석**

- 물리치료 중재 과목에서 난이도 지수가 80에서 100 사이인 문항이 전체 65문항 중 33문항이었으며, 60이상 80미만인 문항이 23문항, 60 미만인 문항이 9문항으로 나타남
- 변별도1 지수를 기준으로 분류하였을 때, 0.3미만인 문항이 30문항으로 0.3 이상인 문항이 35문항인 것에 비해 더 적게 나타남
- 변별도2 지수를 기준으로 분류하였을 때, 0.3미만인 문항이 20문항으로 0.3 이상인 문항이 45문항인 것에 비해 더 적게 나타남

(4) 의료관계법규 난이도와 변별도 분포도 및 비율분석

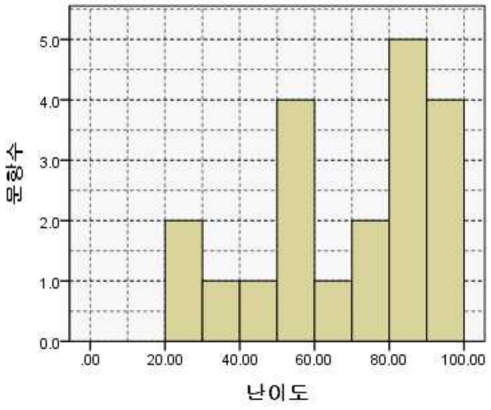


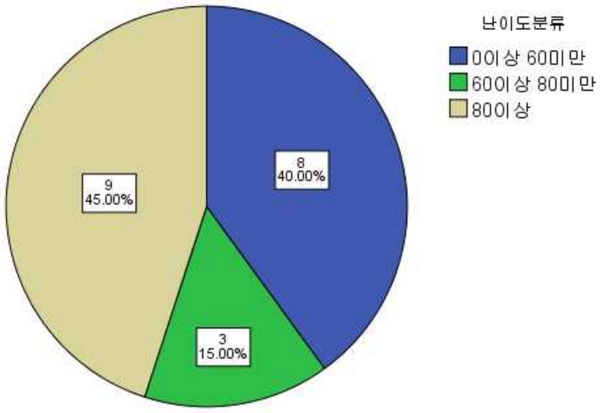


| 총점 | 난이도 | 표준편차 |
|---|---|---|
| 20 | 69.5 | 23.6 |

| 난이도 | 문항수 | 비율(%) |
|---|---|---|
| 0~60미만 | 8 | 40.0 |
| 60~80미만 | 3 | 15.0 |
| 80~100 | 9 | 45.0 |
| 전체 | 20 | 100.0 |

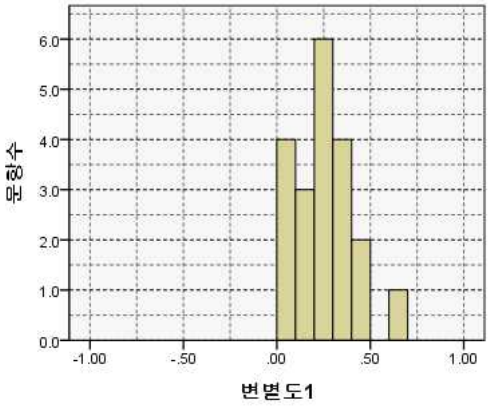


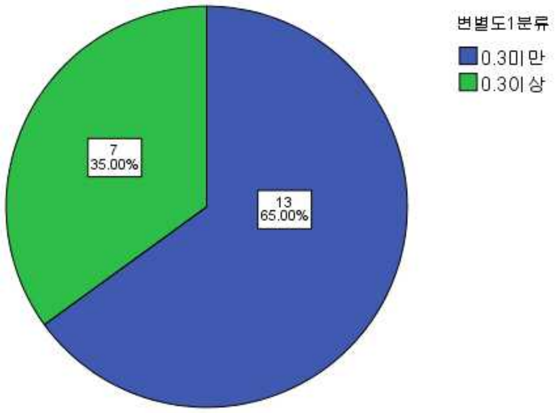


| 총점 | 변별도1 | 표준편차 |
|---|---|---|
| 20 | .24 | .15 |

| 변별도1 | 문항수 | 비율(%) |
|---|---|---|
| 0.3미만 | 13 | 65.0 |
| 0.3이상 | 7 | 35.0 |
| 전체 | 20 | 100.0 |

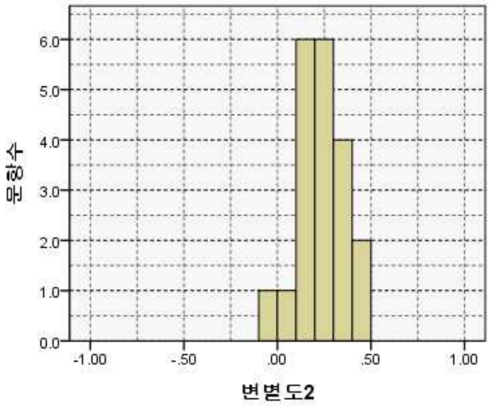


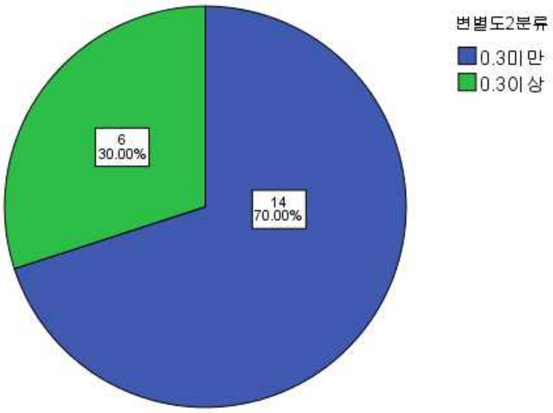


| 총점 | 변별도2 | 표준편차 |
|---|---|---|
| 20 | .24 | .12 |

| 변별도2 | 문항수 | 비율(%) |
|---|---|---|
| 0.3미만 | 14 | 70.0 |
| 0.3이상 | 6 | 30.0 |
| 전체 | 20 | 100.0 |

**해석**

- 의료관계법규 과목에서 난이도 지수가 80 에서 100 사이인 문항이 전체 20 문항 중 9 문항이었으며, 60 이상 80 미만인 문항이 3 문항, 60 미만인 문항이 8 문항으로 나타남
- 변별도 1 지수를 기준으로 분류하였을 때, 0.3 미만인 문항이 13 문항으로 0.3 이상인 문항이 7 문항인 것에 비해 더 많이 나타남
- 변별도 2 지수를 기준으로 분류하였을 때, 0.3 미만인 문항이 14 문항으로 0.3 이상인 문항이 6 문항인 것에 비해 더 많이 나타남

(5) 실기시험 난이도와 변별도 분포도 및 비율분석

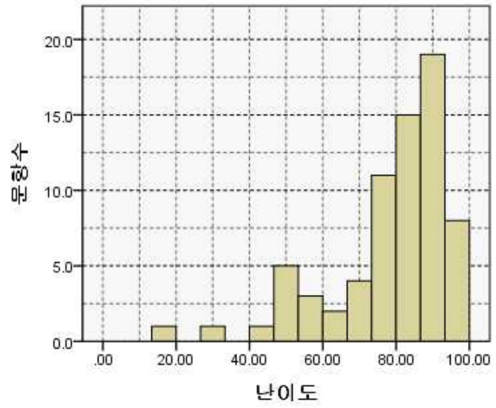


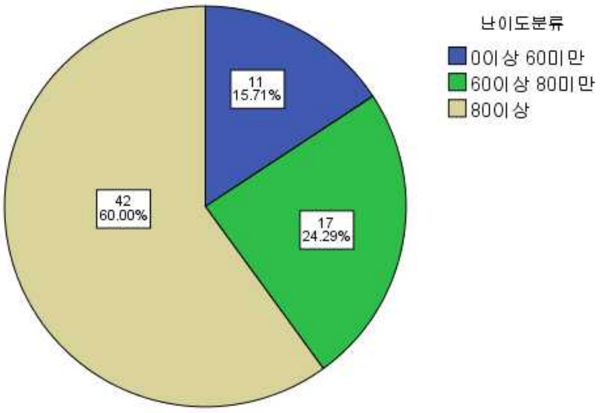


| 총점 | 난이도 | 표준편차 |
|---|---|---|
| 70 | 78.6 | 16.0 |

| 난이도 | 문항수 | 비율(%) |
|---|---|---|
| 0~60미만 | 11 | 15.7 |
| 60~80미만 | 17 | 24.3 |
| 80~100 | 42 | 60.0 |
| 전체 | 70 | 100.0 |

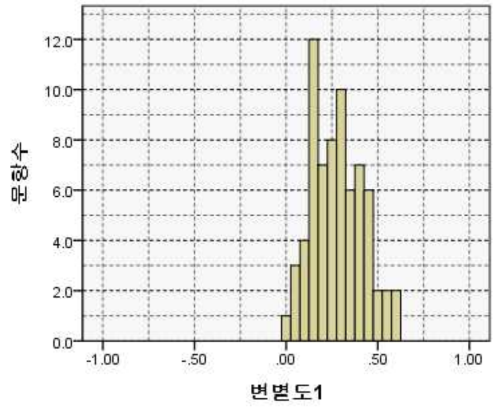


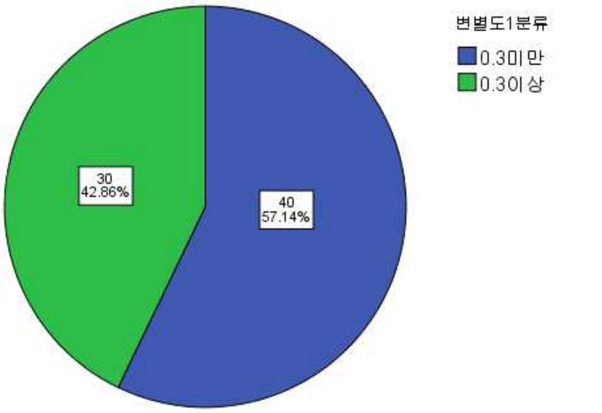


| 총점 | 변별도1 | 표준편차 |
|---|---|---|
| 70 | .28 | .14 |

| 변별도1 | 문항수 | 비율(%) |
|---|---|---|
| 0.3미만 | 40 | 57.1 |
| 0.3이상 | 30 | 42.9 |
| 전체 | 70 | 100.0 |

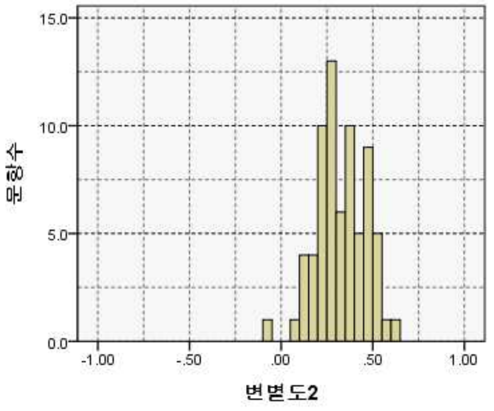


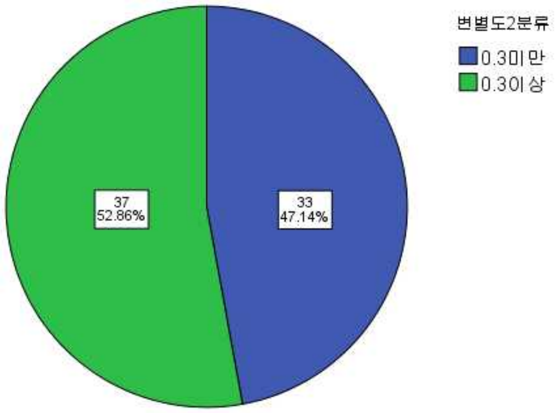


| 총점 | 변별도2 | 표준편차 |
|---|---|---|
| 70 | .33 | .14 |

| 변별도2 | 문항수 | 비율(%) |
|---|---|---|
| 0.3미만 | 33 | 47.1 |
| 0.3이상 | 37 | 52.9 |
| 전체 | 70 | 100.0 |

**해석**

- 실기시험 과목에서 난이도 지수가 80 에서 100 사이인 문항이 전체 70 문항 중 42 문항이었으며, 60 이상 80 미만인 문항이 17 문항, 60 미만인 문항이 11 문항으로 나타남
- 변별도 1 지수를 기준으로 분류하였을 때, 0.3 미만인 문항이 40 문항으로 0.3 이상인 문항이 30 문항인 것에 비해 더 많이 나타남
- 변별도 2 지수를 기준으로 분류하였을 때, 0.3 미만인 문항이 33 문항으로 0.3 이상인 문항이 37 문항인 것에 비해 더 많이 나타남

3) 지식수준별 난이도와 변별도

가) 전회 대비 지식수준별 난이도와 변별도

(1) 전회 대비 암기형 난이도와 변별도

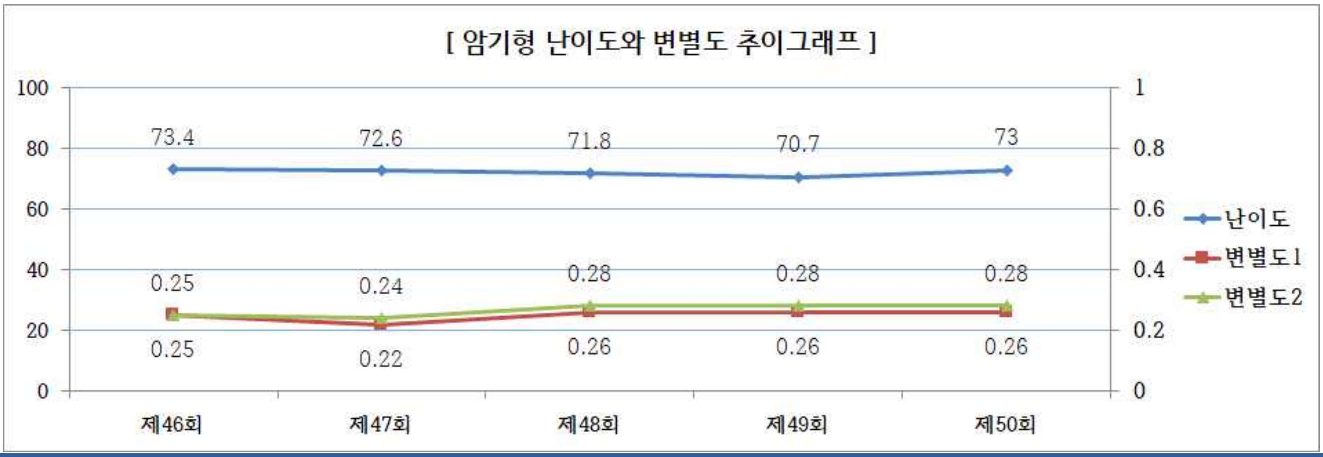


| 회차 | 난이도 | | 변별도1 | | 변별도2 | |
|---|---|---|---|---|---|---|
| | 평균 | 표준편차 | 평균 | 표준편차 | 평균 | 표준편차 |
| 제46회 | 73.4 | 19.1 | .25 | .13 | .25 | .10 |
| 제47회 | 72.6 | 21.4 | .22 | .12 | .24 | .10 |
| 제48회 | 71.8 | 21.4 | .26 | .12 | .28 | .10 |
| 제49회 | 70.7 | 23.1 | .26 | .14 | .28 | .12 |
| 제50회 | 73.0 | 21.4 | .26 | .14 | .28 | .13 |

(2) 전회 대비 해석형 난이도와 변별도

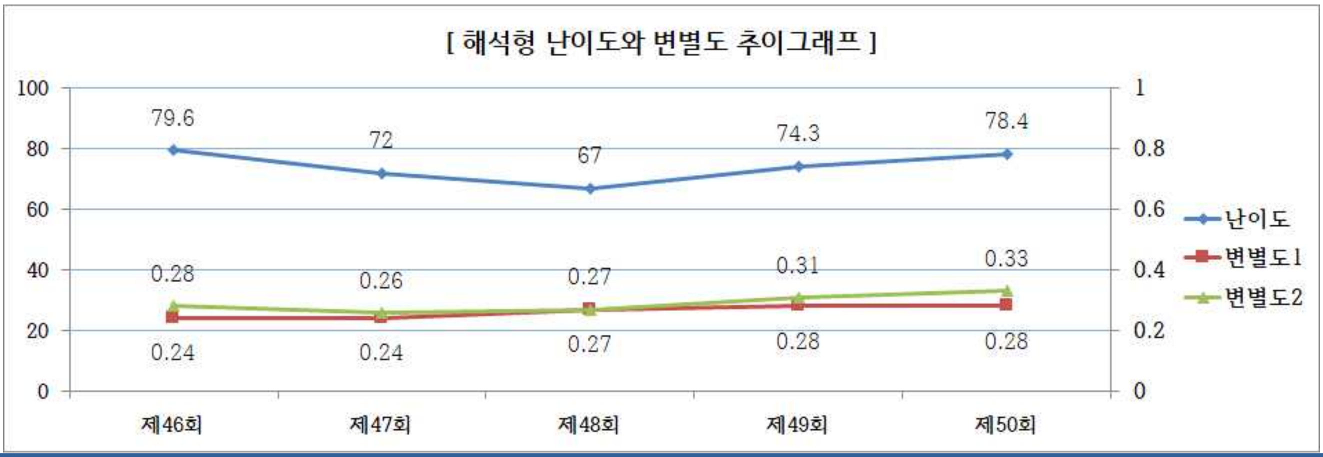


| 회차 | 난이도 | | 변별도1 | | 변별도2 | |
|---|---|---|---|---|---|---|
| | 평균 | 표준편차 | 평균 | 표준편차 | 평균 | 표준편차 |
| 제46회 | 79.6 | 16.3 | .24 | .12 | .28 | .09 |
| 제47회 | 72.0 | 19.3 | .24 | .12 | .26 | .10 |
| 제48회 | 67.0 | 21.7 | .27 | .14 | .27 | .12 |
| 제49회 | 74.3 | 18.9 | .28 | .13 | .31 | .12 |
| 제50회 | 78.4 | 16.2 | .28 | .15 | .33 | .14 |

(3) 전회 대비 해결형 난이도와 변별도

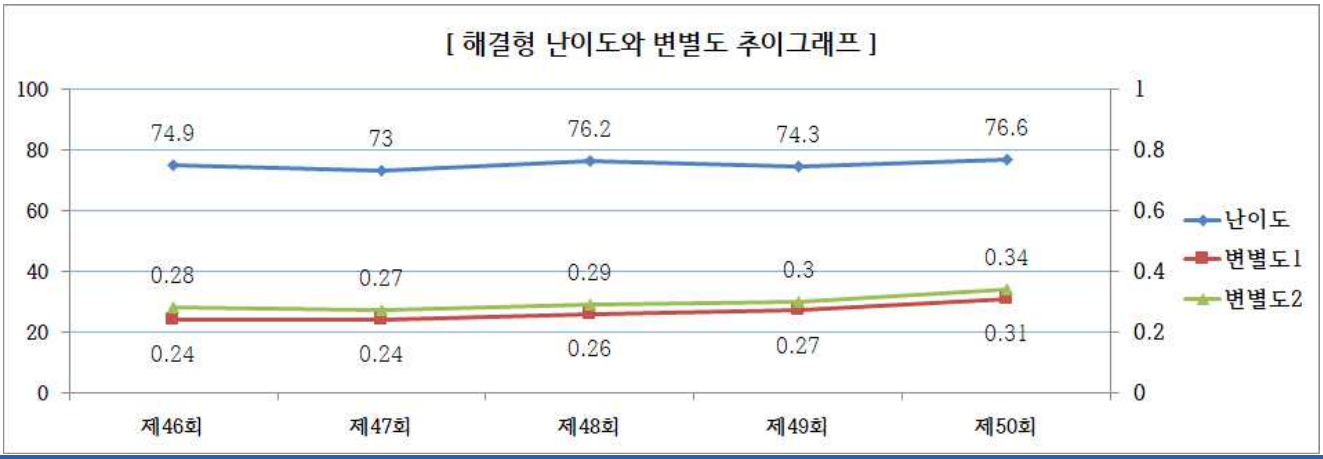


| 회차 | 난이도 | | 변별도1 | | 변별도2 | |
|---|---|---|---|---|---|---|
| | 평균 | 표준편차 | 평균 | 표준편차 | 평균 | 표준편차 |
| 제46회 | 74.9 | 19.4 | .24 | .13 | .28 | .11 |
| 제47회 | 73.0 | 19.1 | .24 | .13 | .27 | .12 |
| 제48회 | 76.2 | 15.5 | .26 | .13 | .29 | .10 |
| 제49회 | 74.3 | 19.6 | .27 | .14 | .30 | .13 |
| 제50회 | 76.6 | 16.5 | .31 | .14 | .34 | .13 |

**해석**

- 전회 대비 암기형 문항의 난이도 지수는 2.3 증가하였고, 해석형 문항의 난이도 지수는 4.1 증가하였으며 해결형 문항의 난이도 지수는 2.3 증가함
- 변별도 1 지수는 암기형 문항에서 동일하였고 해석형 문항에서 동일하였으며 해결형 문항에서는 .04 증가함
- 변별도 2 지수는 암기형 문항에서 동일하였고 해석형 문항에서 .02 증가하였으며 해결형 문항에서는 .04 증가함

## 나) 지식수준별 난이도와 변별도 분포도 및 비율분석

### (1) 암기형 난이도와 변별도 분문도 및 비율분석

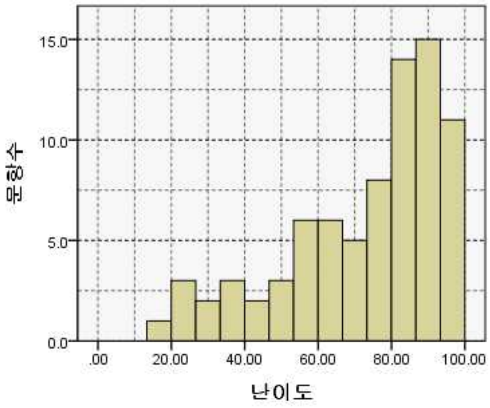


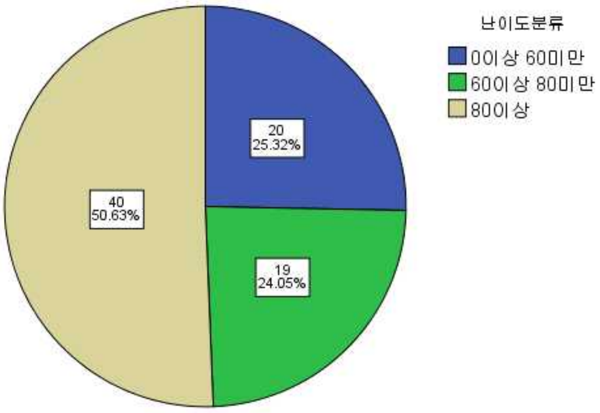


| 총점 | 난이도 | 표준편차 |
|---|---|---|
| 79 | 73.0 | 21.4 |

| 난이도 | 문항수 | 비율(%) |
|---|---|---|
| 0~60미만 | 20 | 25.3 |
| 60~80미만 | 19 | 24.1 |
| 80~100 | 40 | 50.6 |
| 전체 | 79 | 100.0 |

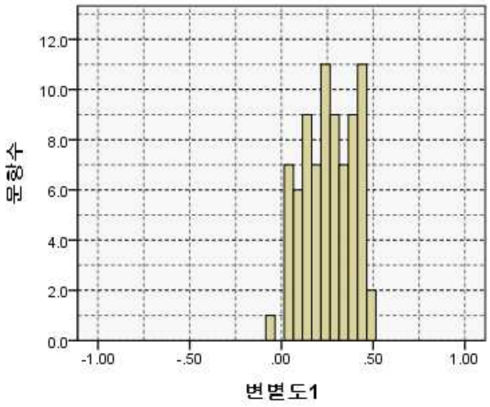


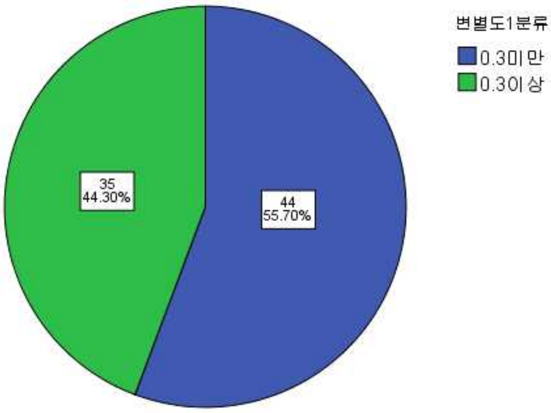


| 총점 | 변별도1 | 표준편차 |
|---|---|---|
| 79 | .26 | .14 |

| 변별도1 | 문항수 | 비율(%) |
|---|---|---|
| 0.3미만 | 44 | 55.7 |
| 0.3이상 | 35 | 44.3 |
| 전체 | 79 | 100.0 |

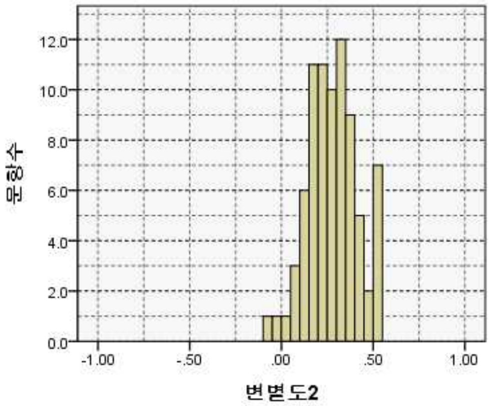


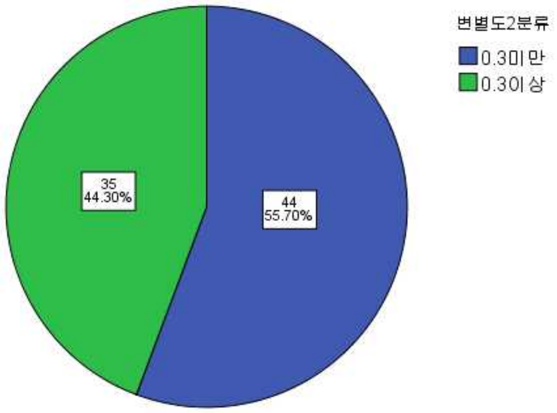


| 총점 | 변별도2 | 표준편차 |
|---|---|---|
| 79 | .28 | .13 |

| 변별도2 | 문항수 | 비율(%) |
|---|---|---|
| 0.3미만 | 44 | 55.7 |
| 0.3이상 | 35 | 44.3 |
| 전체 | 79 | 100.0 |

**해석**

- 암기형 문항에서 난이도 지수가 80 에서 100 사이인 문항이 전체 79 문항 중 40 문항이었으며, 60 이상 80 미만인 문항이 19 문항, 60 미만인 문항이 20 문항인 것으로 나타남
- 변별도 1 지수를 기준으로 하였을 때, 0.3 미만인 문항이 44 문항으로 0.3 이상인 문항이 35 문항인 것에 비해 더 많이 나타남
- 변별도 2 지수를 기준으로 분류하였을 때, 0.3 미만인 문항이 44 문항으로 0.3 이상인 문항이 35 문항인 것에 비해 더 많이 나타남

(2) 해석형 난이도와 변별도 분포도 및 비율분석

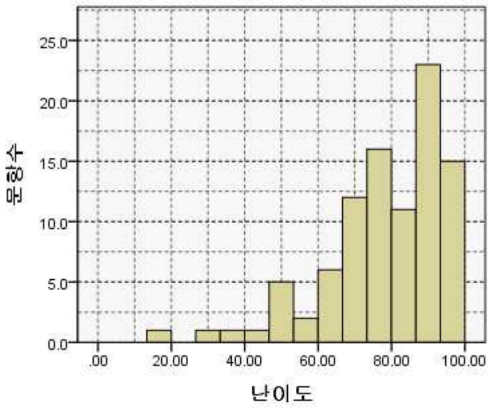


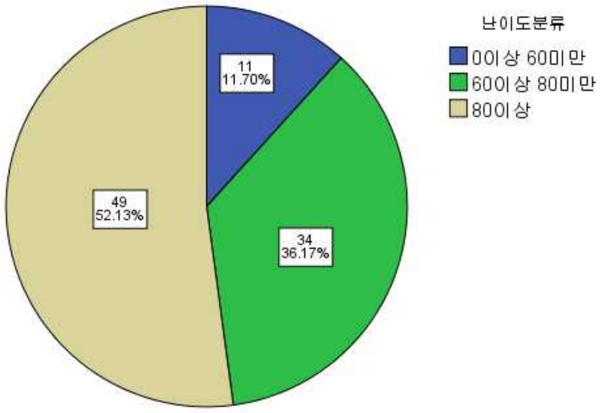


| 총점 | 난이도 | 표준편차 |
|---|---|---|
| 94 | 78.4 | 16.2 |

| 난이도 | 문항수 | 비율(%) |
|---|---|---|
| 0~60미만 | 11 | 11.7 |
| 60~80미만 | 34 | 36.2 |
| 80~100 | 49 | 52.1 |
| 전체 | 94 | 100.0 |

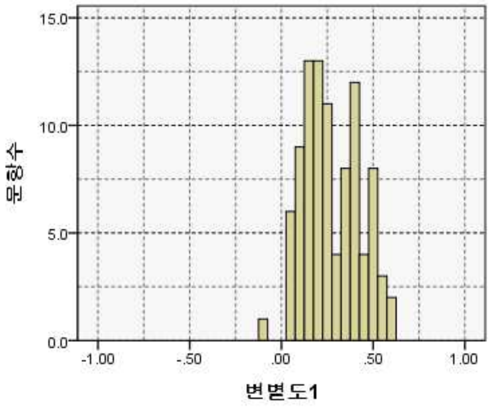


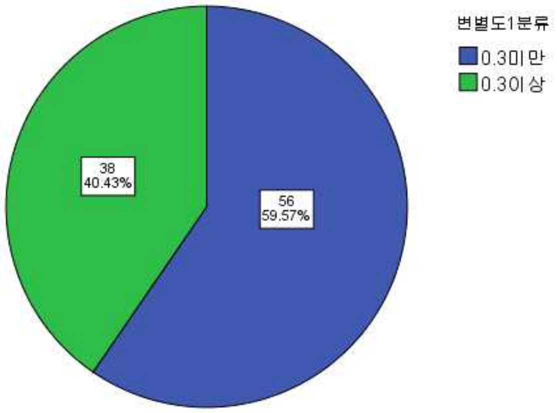


| 총점 | 변별도1 | 표준편차 |
|---|---|---|
| 94 | .28 | .15 |

| 변별도1 | 문항수 | 비율(%) |
|---|---|---|
| 0.3미만 | 56 | 59.6 |
| 0.3이상 | 38 | 40.4 |
| 전체 | 94 | 100.0 |

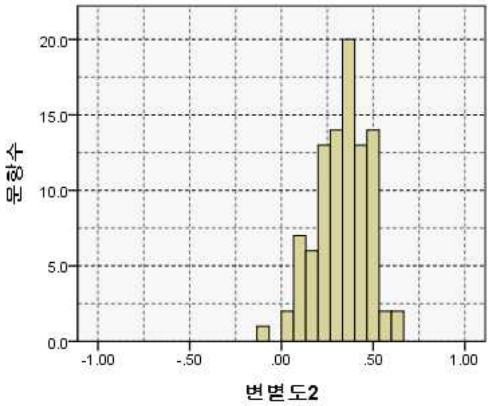


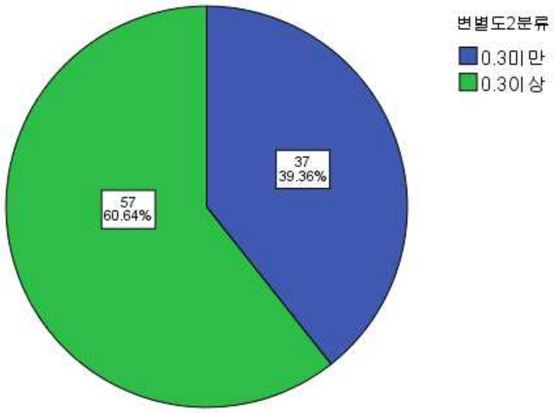


| 총점 | 변별도2 | 표준편차 |
|---|---|---|
| 94 | .33 | .14 |

| 변별도2 | 문항수 | 비율(%) |
|---|---|---|
| 0.3미만 | 37 | 39.4 |
| 0.3이상 | 57 | 60.6 |
| 전체 | 94 | 100.0 |

**해석**

- 해석형 문항에서 난이도 지수가 80에서 100 사이인 문항이 전체 94문항 중 49문항이었으며, 60 이상 80 미만인 문항이 34문항, 60 미만인 문항이 11문항인 것으로 나타남
- 변별도1 지수를 기준으로 하였을 때, 0.3 미만인 문항이 56문항으로 0.3 이상인 문항이 38문항인 것에 비해 더 많이 나타남
- 변별도2 지수를 기준으로 분류하였을 때, 0.3 미만인 문항이 37문항으로 0.3 이상인 문항이 57문항인 것에 비해 더 적게 나타남

(3) 해결형 난이도와 변별도 분포도 및 비율분석

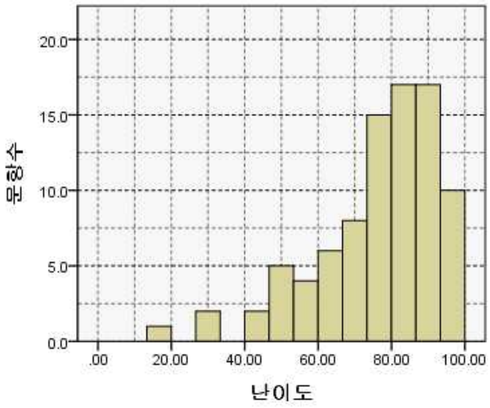


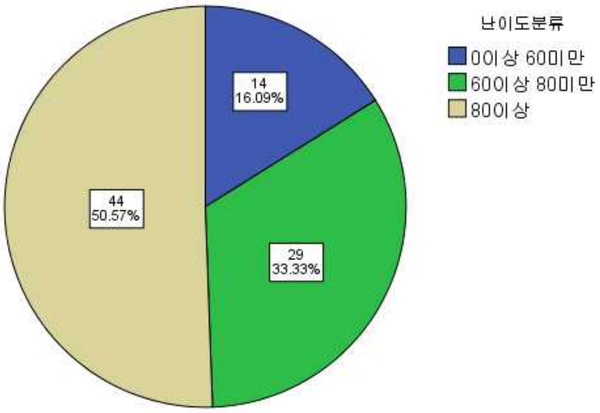


| 총점 | 난이도 | 표준편차 |
|---|---|---|
| 87 | 76.6 | 16.5 |

| 난이도 | 문항수 | 비율(%) |
|---|---|---|
| 0~60미만 | 14 | 16.1 |
| 60~80미만 | 29 | 33.3 |
| 80~100 | 44 | 50.6 |
| 전체 | 87 | 100.0 |

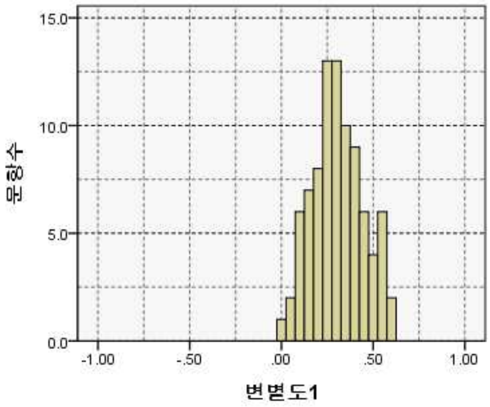


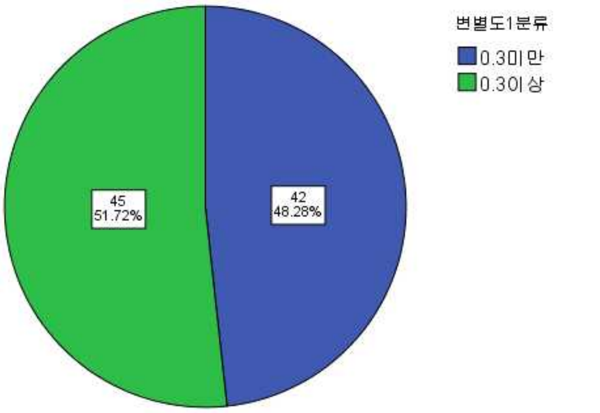


| 총점 | 변별도1 | 표준편차 |
|---|---|---|
| 87 | .31 | .14 |

| 변별도1 | 문항수 | 비율(%) |
|---|---|---|
| 0.3미만 | 42 | 48.3 |
| 0.3이상 | 45 | 51.7 |
| 전체 | 87 | 100.0 |

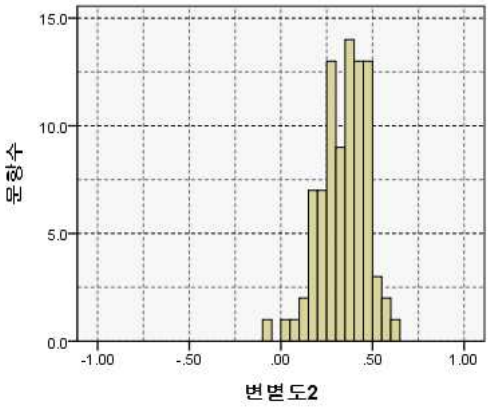


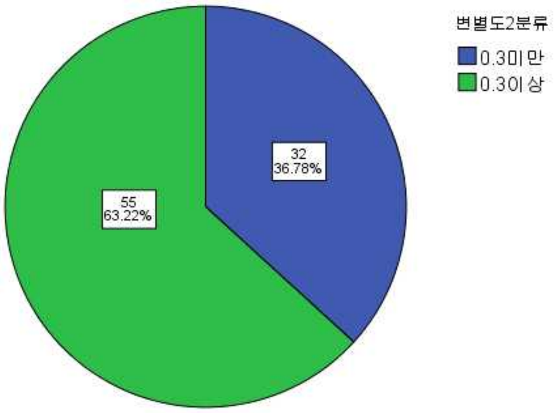


| 총점 | 변별도2 | 표준편차 | 변별도2 | 문항수 | 비율(%) |
|---|---|---|---|---|---|
| 87 | .34 | .13 | 0.3미만 | 32 | 36.8 |
| | | | 0.3이상 | 55 | 63.2 |
| | | | 전체 | 87 | 100.0 |

**해석**

- 해결형 문항에서 난이도 지수가 80 에서 100 사이인 문항이 전체 87 문항 중 44 문항이었으며, 60 이상 80 미만인 문항이 29 문항, 60 미만인 문항이 14 문항인 것으로 나타남
- 변별도 1 지수를 기준으로 하였을 때, 0.3 미만인 문항이 42 문항으로 0.3 이상인 문항이 45 문항인 것에 비해 더 적게 나타남
- 변별도 2 지수를 기준으로 분류하였을 때, 0.3 미만인 문항이 32 문항으로 0.3 이상인 문항이 55 문항인 것에 비해 더 적게 나타남

## 3. 난이도와 변별도 간 산포도

### 1) 전체 난이도와 변별도 간 산포도

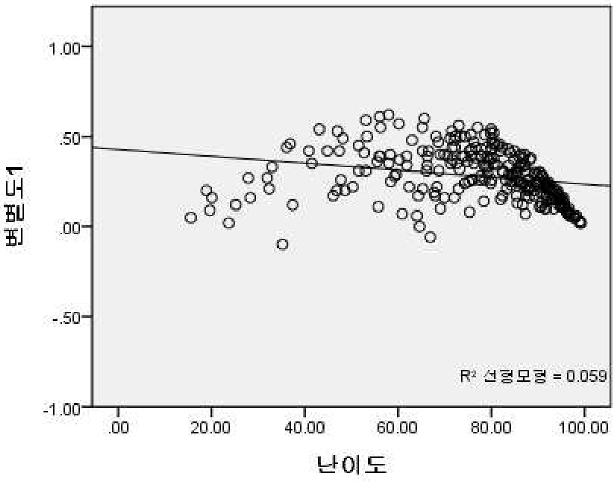


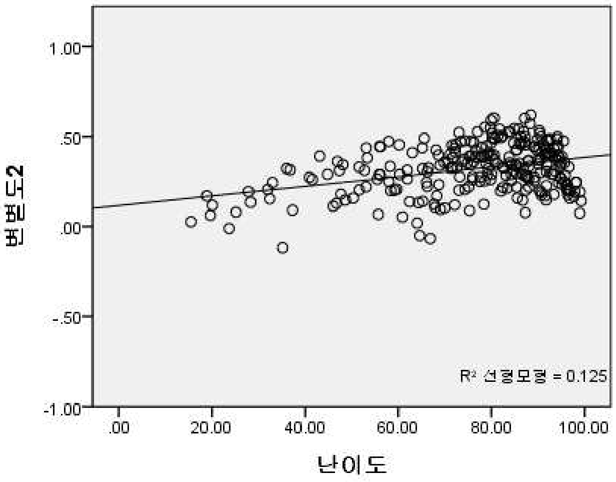


**해석**

- 난이도 지수와 변별도 1 지수 간 상관은 -.243*으로 난이도 지수가 높을수록 변별력이 낮아지는 것으로 나타남
- 난이도 지수와 변별도 2 지수 간 상관은 .353*으로 난이도 지수가 높을수록 변별력이 높아지는 것으로 나타남

### 2) 과목별 난이도와 변별도 간 산포도

#### 가) 물리치료 기초 난이도와 변별도 간 산포도

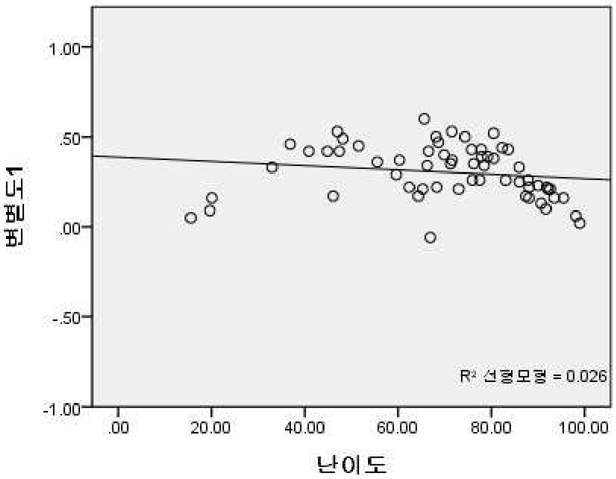


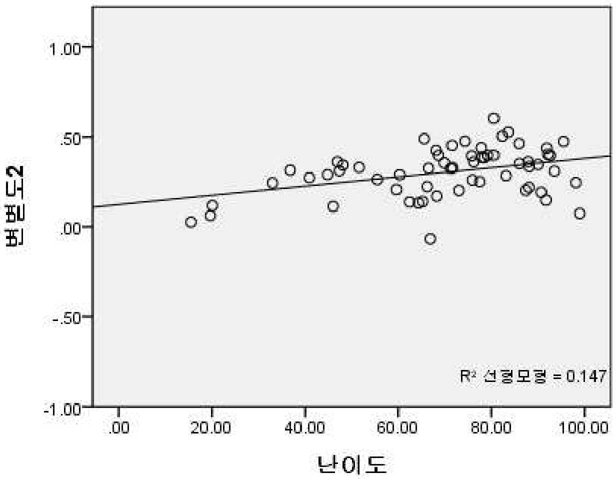

**해석**

- 난이도 지수와 변별도1 지수 간 상관은 -.162로 관련성이 없는 것으로 나타남
- 난이도 지수와 변별도2 지수 간 상관은 .383*으로 난이도 지수가 높을수록 변별력이 높아지는 것으로 나타남

나) 물리치료 진단평가 난이도와 변별도 간 산포도

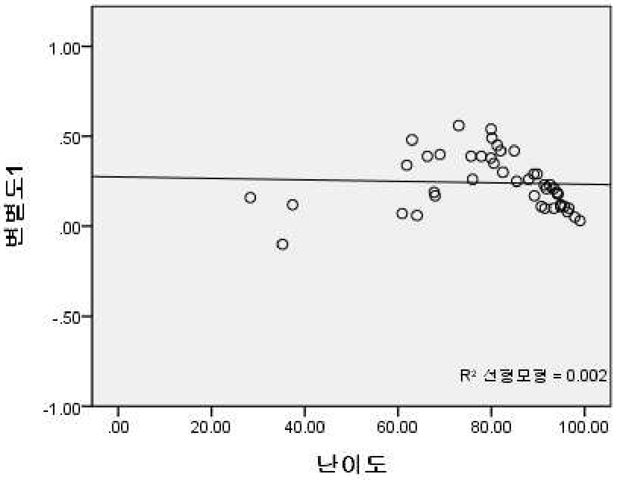


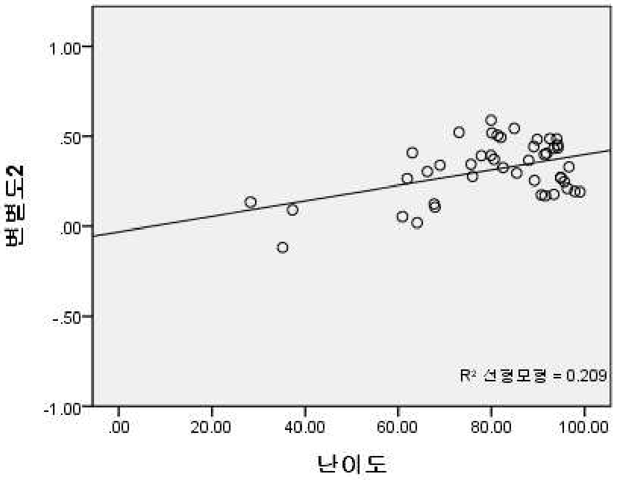


**해석**

- 난이도 지수와 변별도1 지수 간 상관은 -.045로 관련성이 없는 것으로 나타남
- 난이도 지수와 변별도2 지수 간 상관은 .457*로 난이도 지수가 높을수록 변별력이 높아지는 것으로 나타남

다) 물리치료 중재 난이도와 변별도 간 산포도

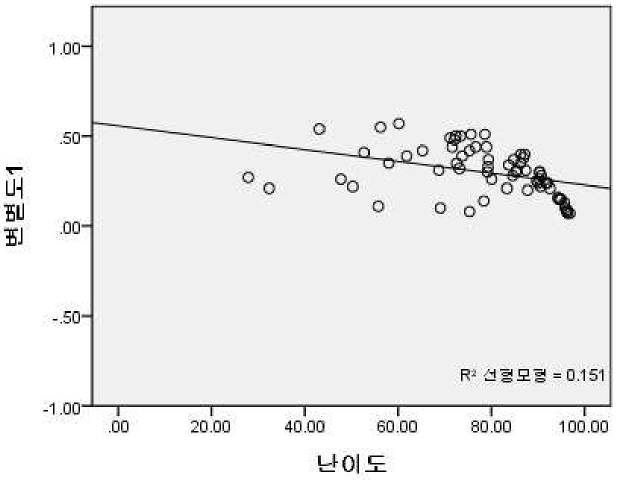


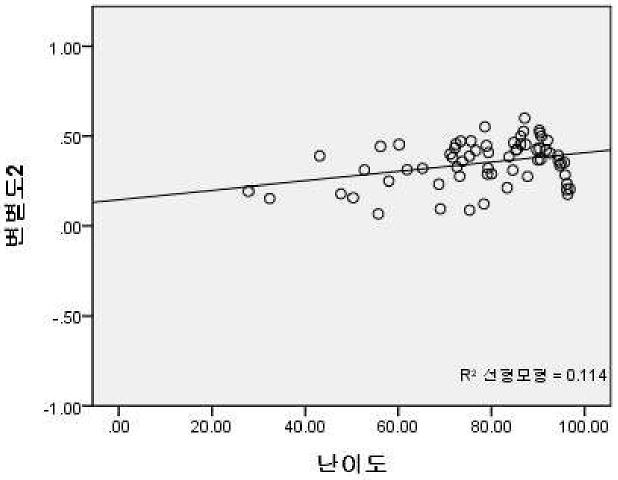

해석

- 난이도 지수와 변별도 1 지수 간 상관은 -.388*로 난이도 지수가 높을수록 변별력이 낮아지는 것으로 나타남
- 난이도 지수와 변별도 2 지수 간 상관은 .338*로 난이도 지수가 높을수록 변별력이 높아지는 것으로 나타남

라) 의료관계법규 난이도와 변별도 간 산포도

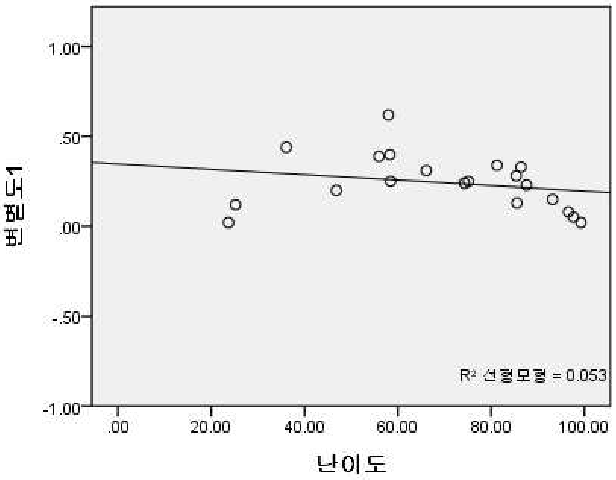


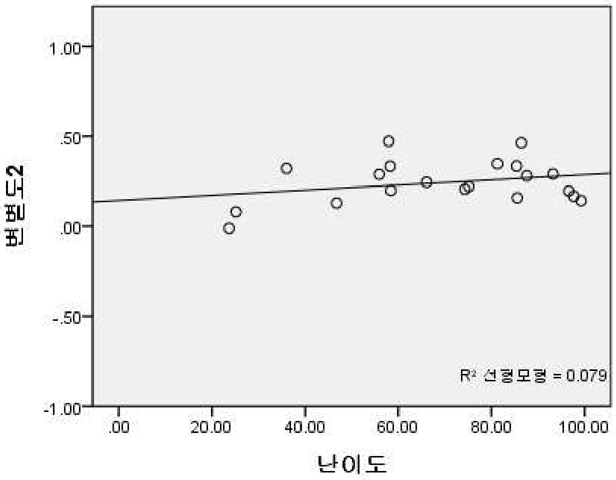


해석

- 난이도 지수와 변별도 1 지수 간 상관은 -.230 으로 관련성이 없는 것으로 나타남
- 난이도 지수와 변별도 2 지수 간 상관은 .281 로 관련성이 없는 것으로 나타남

마) 실기시험 난이도와 변별도 간 산포도

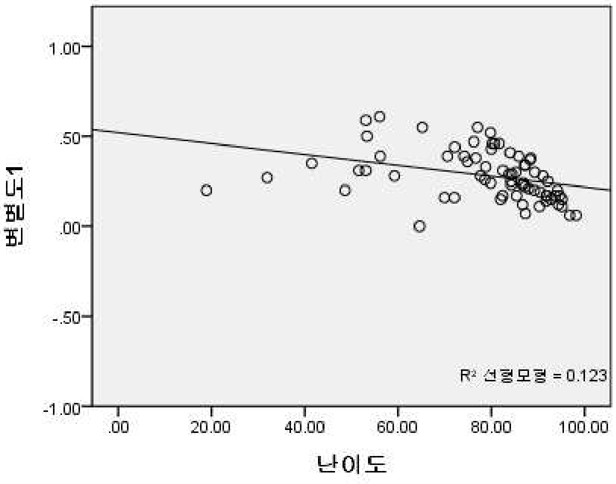


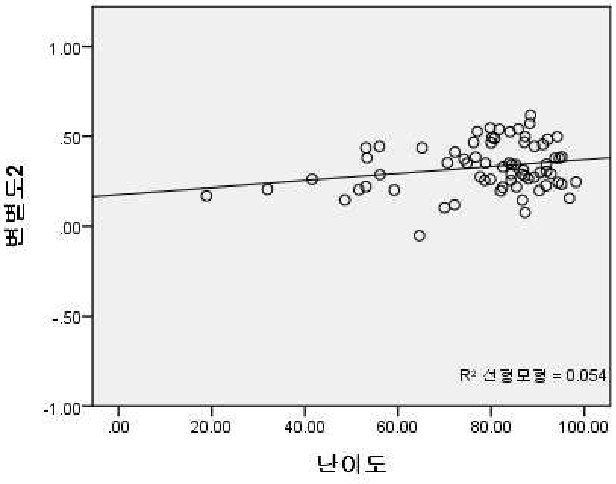

**해석**

- 난이도 지수와 변별도1 지수 간 상관은 -.351*로 난이도 지수가 높을수록 변별력이 낮아지는 것으로 나타남
- 난이도 지수와 변별도2 지수 간 상관은 .231로 관련성이 없는 것으로 나타남

## 4. 신뢰도 분석

| 과목명 | 문항수 | 제46회 | 제47회 | 제48회 | 제49회 | 제50회 |
|---|---|---|---|---|---|---|
| 전체 | 260 | .951 | .946 | .954 | .964 | .965 |
| 물리치료 기초 | 60 | .821 | .798 | .791 | .838 | .860 |
| 물리치료 진단평가 | 45 | .819 | .776 | .801 | .860 | .818 |
| 물리치료 중재 | 65 | .849 | .853 | .869 | .900 | .896 |
| 의료관계법규 | 20 | .480 | .570 | .616 | .680 | .615 |
| 실기시험 | 70 | .827 | .796 | .859 | .855 | .893 |

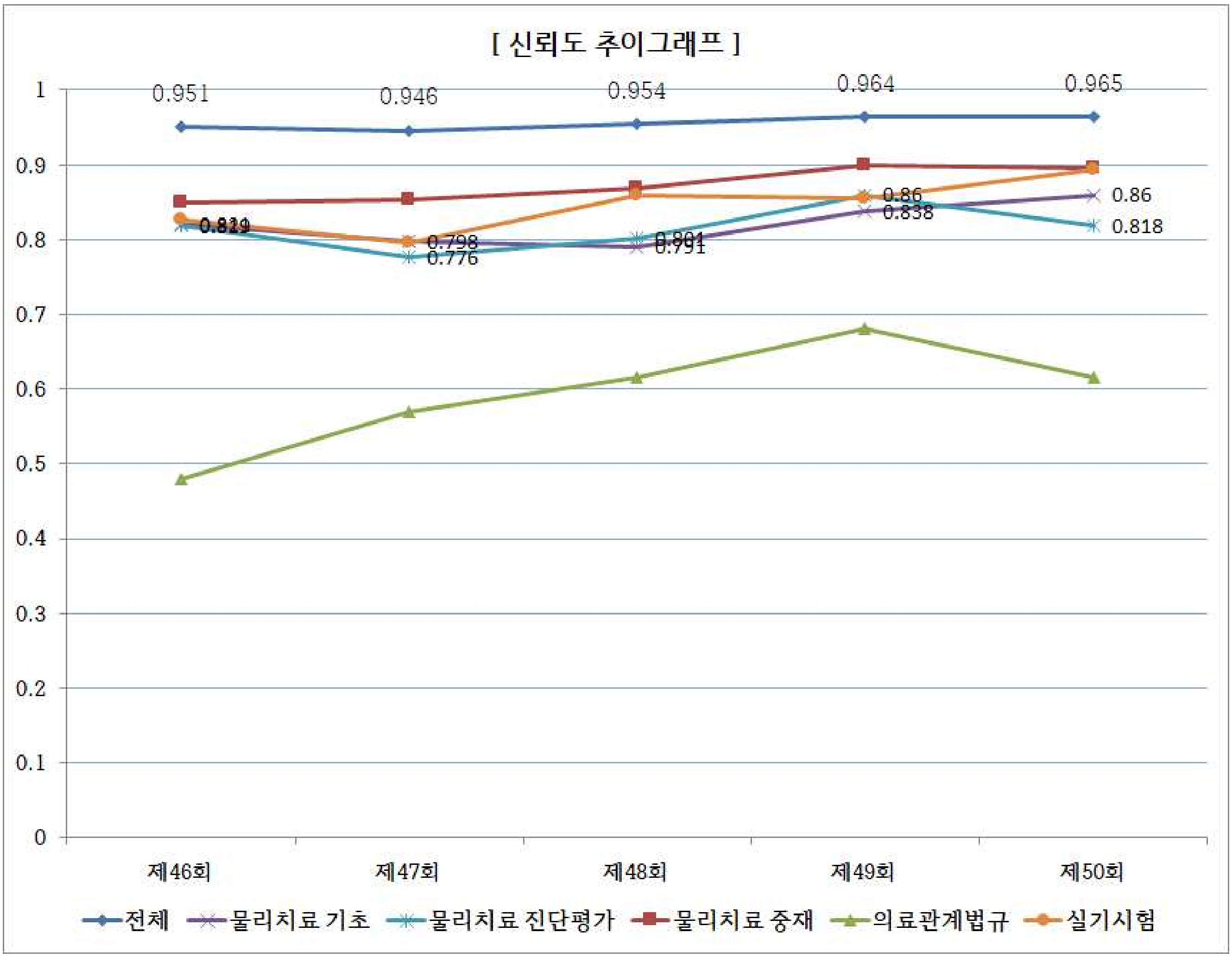

**해석**

- 전회 대비 전체 문항의 신뢰도는 .001 증가함
- 전회 대비 물리치료 기초 과목 문항의 신뢰도는 .022 증가함
- 전회 대비 물리치료 진단평가 과목 문항의 신뢰도는 .042 감소함
- 전회 대비 물리치료 중재 과목 문항의 신뢰도는 .004 감소함
- 전회 대비 의료관계법규 과목 문항의 신뢰도는 .065 감소함
- 전회 대비 실기시험 과목 문항의 신뢰도는 .038 증가함

○ 분석결과 관련 문의 : 한국보건의료인국가시험원 연구개발본부 김준기 전임연구원
Tel : 02-2087-8956, FAX : 02-2087-8885
E-mail : tontates@kuksiwon.or.kr
