# Supplementary material for: Item difficulty index, discrimination index, and reliability of the 26 health professions licensing examinations in 2022, Korea: a psychometric study
Source: J Educ Eval Health Prof. 2023 Nov 22;20:31. doi: 10.3352/jeehp.2023.20.31 (PMC11959405; doi:10.3352/jeehp.2023.20.31)
Supplement: Supplementary file 1 — Supplement 1. Item analysis results of 26 health professions licensing examinations administered during late 2022 and early 2023. [file jeehp-20-31_Suppl1.zip › 2022│Γ╡╡ ┴a50╚╕ ╣μ╗τ╝▒╗τ ▒╣░í╜├╟Φ ║╨╝«░ß░·.pdf]

2022년도 제50회
방사선사 국가시험
문항분석 결과

국민이 신뢰하고 감동하는 시험평가기관
한국보건의료인국가시험원
KOREA HEALTH PERSONNEL LICENSING EXAMINATION INSTITUTE

일반 용어 정의

□ **평균**

- 집단에서의 대표적 경향값으로 전체 값을 더하여 총 응시자로 나눈 값

□ **표준편차**

- 평균과 각 점수의 차이인 편차들의 평균으로 점수가 흩어져 분포되어 있는 정도

□ **추정난이도**

- 문항개발자가 예측한 정답률

□ **검사이론**

- 검사와 검사를 구성하고 있는 문항의 양호도를 분석 및 평가하는 방법을 정의한 이론체계
- 대표적으로 고전검사이론과 문항반응이론이 있음

고전검사이론 용어 정의

□ **고전검사이론(Classical Test Theory; CTT)**

- 검사의 질을 분석하는 검사이론 중 한 가지로 19세기 말부터 전개되어 현재까지 주로 사용되고 있는 검사이론임
- 고전검사이론에 의한 문항과 응시자 능력 추정치는 다음과 같음

○ **문항난이도**

· 검사 문항의 쉽고 어려운 정도를 나타내는 지수
· 난이도 지수는 총 반응 수에 대한 정답 반응 수의 비율로 문항의 정답률임
· 문항난이도는 0~100까지의 값을 가짐
· 난이도 값이 큰 경우, 쉬운 문항으로 '난이도가 낮다'라고 해석하며, 난이도 값이 작은 경우, 어려운 문항으로 '난이도가 높다'라고 해석함

○ **문항변별도**

· 각 문항이 응시자의 능력 수준을 변별할 수 있는 정도를 나타내는 지수
· 문항변별도는 -1~+1까지의 값을 가지며, 1에 가까울수록 변별력 크다고 해석함
· 일반적으로 문항변별도가 0.3 이상이면 우수한 문항으로 평가함
· 구하는 방식에는 '상하위집단 구분법', '문항-총점 상관계수' 등이 있음
  1) **변별도 1(상하위구분법):** 상위 27%와 하위 27% 집단의 난이도 차이를 구하는 방식
  2) **변별도 2(상관계수법):** 문항-총점과의 상관계수로 구하는 방식

○ **신뢰도**

· 시험이 평가하고자 하는 것을 일관성 있게 측정하는가로 시험이 오차없이 정확하게 측정한 정도를 의미함
· 국시원에서는 문항의 내적일관성(Cronbach α)으로 신뢰도를 추정하며 1에 가까울수록 신뢰도가 높다고 해석함

목 차

I. 시행 결과 ······ 6
1. 시험 현황 ······ 7
1) 시험명 ······ 7
2) 시험시행일 ······ 7
3) 응시현황 ······ 7
4) 과목별 문항 수, 배점 및 과락 점수 ······ 7
2. 합격률과 평균성적 ······ 7
1) 합격 및 불합격 현황 ······ 7
2) 과목별 과락자수 내역 ······ 7
3) 전회 대비 합격률과 평균성적 ······ 8

II. 문항분석 결과 ······ 10
1. 성적 ······ 11
1) 전체 성적분포도 ······ 11
2) 과목별 성적분포도 ······ 12
2. 난이도와 변별도 ······ 13
1) 전체 난이도와 변별도 ······ 13
2) 과목별 난이도와 변별도 ······ 16
3) 지식수준별 난이도와 변별도 ······ 28
3. 난이도와 변별도 간 산포도 ······ 37
1) 전체 난이도와 변별도 간 산포도 ······ 37
2) 과목별 난이도와 변별도 간 산포도 ······ 38
4. 신뢰도 분석 ······ 41

I. 시행 결과

1. 시험 현황

1) 시험명: 2022년도 제50회 방사선사 국가시험

2) 시험시행일: 2022년 12월 17일

3) 응시현황

응시대상자 수	결시자 수	부정행위자 수	응시자 준수사항 위반자 수		응시자 수 (%)
			휴대폰 소지	신분증 미지참	
2,715	105	0	0	0	2,609 (96.1)

4) 과목별 문항 수, 배점 및 과락 점수

교 시	과 목 명	문제 수	배점	총점	합격자 점수기준	
					과목 과락기준	총점 합격기준
1교시	방사선이론	90	1	90	36점 미만	120점 이상
2교시	의료관계법규	20	1	20	8점 미만	
2교시	방사선응용	90	1	90	36점 미만	
3교시	실기시험	50	1	50	30점 미만	
계		250		250		

2. 합격률과 평균성적

1) 합격 및 불합격 현황

합격자 수 (%)	불합격자 수(%)					채점보류자 수
	평락	과락	실기탈락	기권	계	
1,958 (75.0)	505 (19.36)	0 (0.00)	145 (5.56)	1 (0.04)	651 (25.00)	1

2) 과목별 과락자수 내역

과목명 / 과락자 수	방사선이론	의료관계법규	방사선응용	실기시험
과목별 과락자 수	0	0	0	0
전과목 과락자 수	0			

3) 전회 대비 합격률과 평균성적

회차	년도	합격률(%)	평균성적	표준편차	백분율 환산점수
제46회	2018	79.7	188.3	39.4	75.3
제47회	2019	77.1	174.3	42.8	69.7
제48회	2020	69.3	176.4	41.8	70.6
제49회	2021	79.9	182.1	41.7	72.8
**제50회**	**2022**	**75.0**	**177.9**	**45.7**	**71.1**

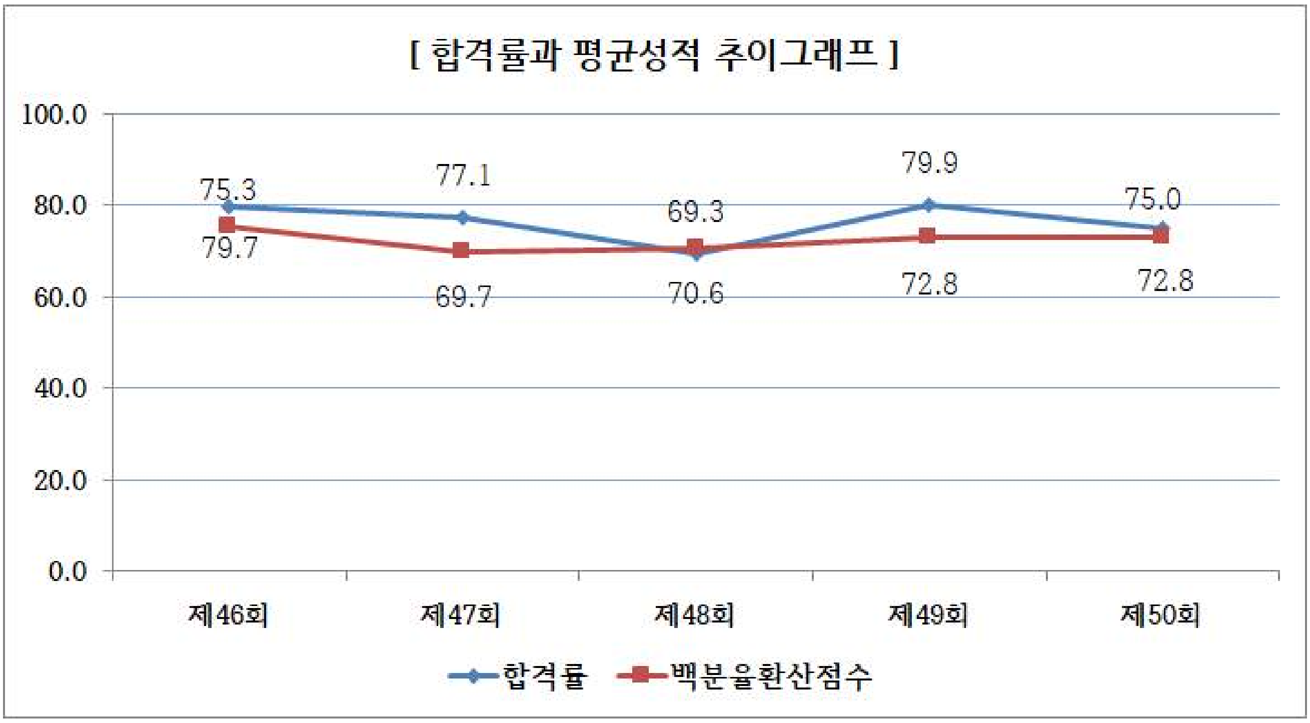

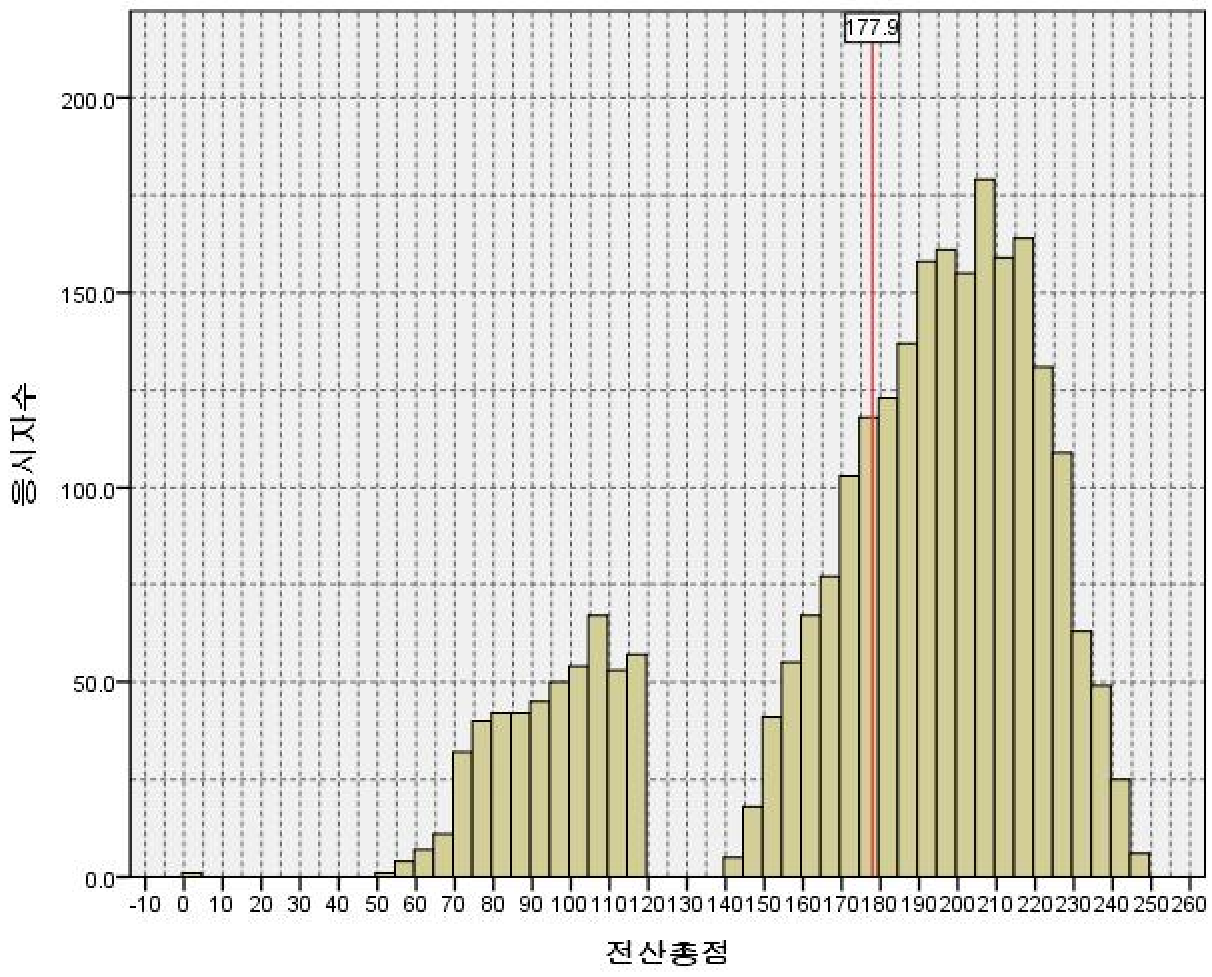

응시자	총점	합격선	평균성적	표준편차
2,609	250		177.9	45.7

※ 필기시험 불합격자의 실기성적을 포함하지 않음

해석

- 전년대비 합격률은 4.9% 감소하고, 백분율 환산점수는 1.7 점 감소함

II. 문항분석 결과

1. 성적

1) 전체 성적분포도

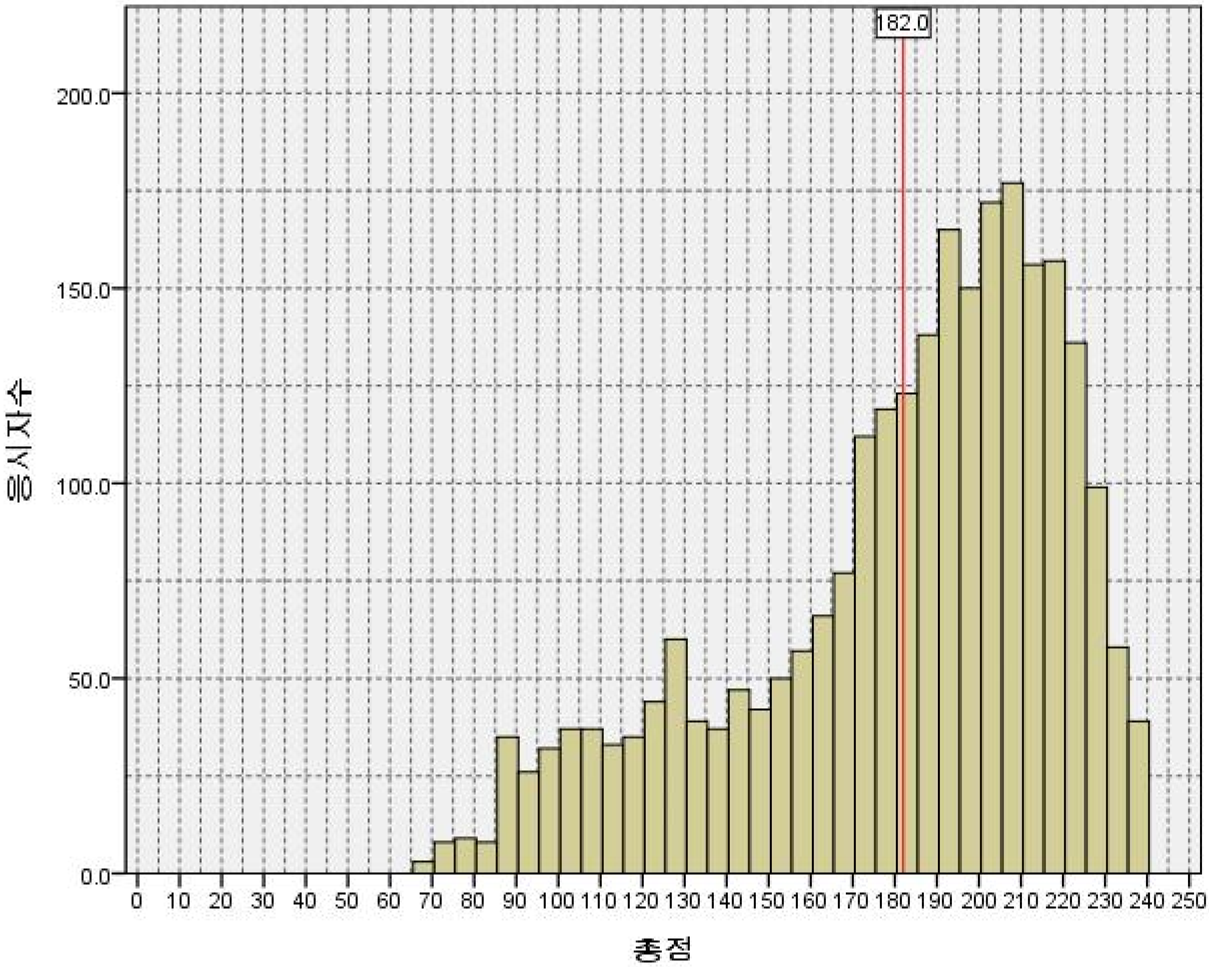

응시자	총점	합격선	평균성적	표준편차
2,609	250		182.0	38.8

※ 2,609명은 기권자 1명을 제외한 수치임

※ 필기시험 불합격자의 실기성적을 포함함

2) 과목별 성적분포도(※ 필기형 실기 포함)

가) 방사선이론

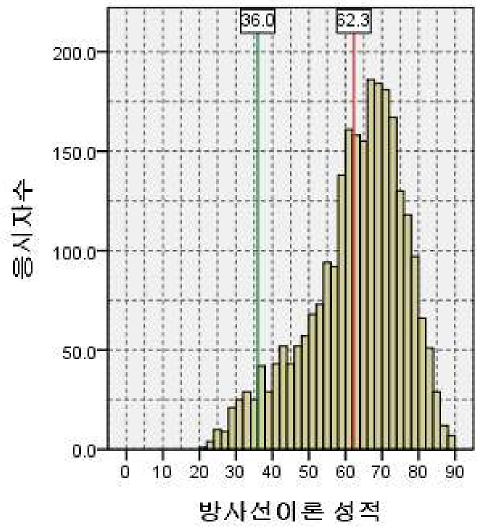

총점	과락선	평균성적	표준편차
90	36	62.3	13.2

나) 의료관계법규

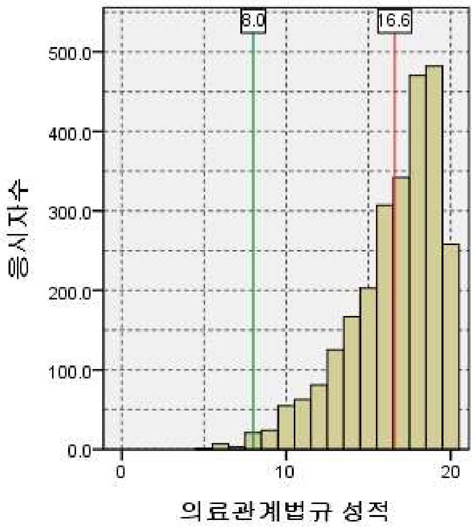

총점	과락선	평균성적	표준편차
20	8	16.6	2.8

다) 방사선응용

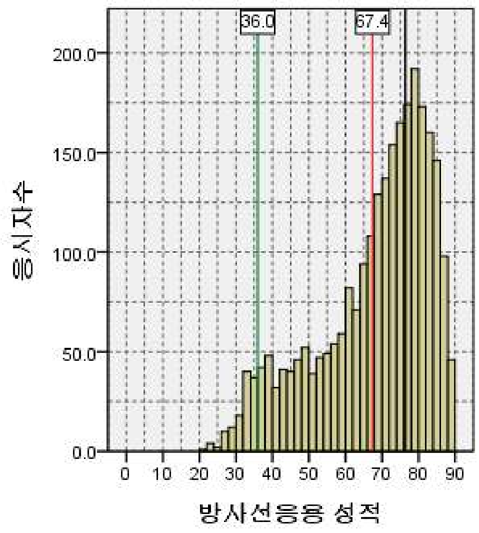

총점	과락선	평균성적	표준편차
90	36	67.4	15.4

라) 실기시험

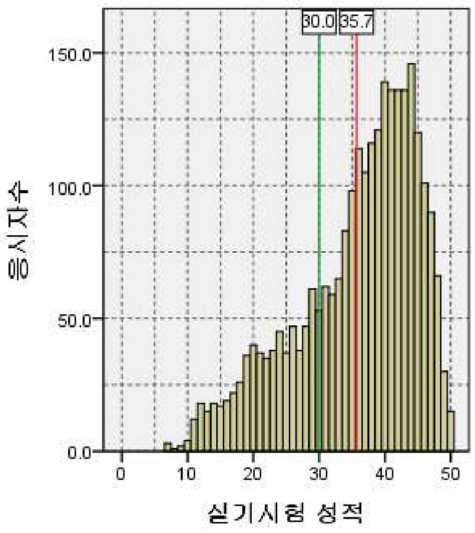

총점	과락선	평균성적	표준편차
50	30	35.7	9.2

2. 난이도와 변별도

1) 전체 난이도와 변별도

가) 전회 대비 전체 난이도와 변별도

회차	난이도		변별도1		변별도2	
	평균	표준편차	평균	표준편차	평균	표준편차
제46회	76.4	15.9	.32	.14	.34	.11
제47회	71.7	17.6	.33	.14	.33	.12
제48회	72.2	18.4	.33	.15	.33	.12
제49회	74.3	16.7	.34	.16	.34	.13
**제50회**	**72.8**	**17.4**	**.38**	**.16**	**.37**	**.13**

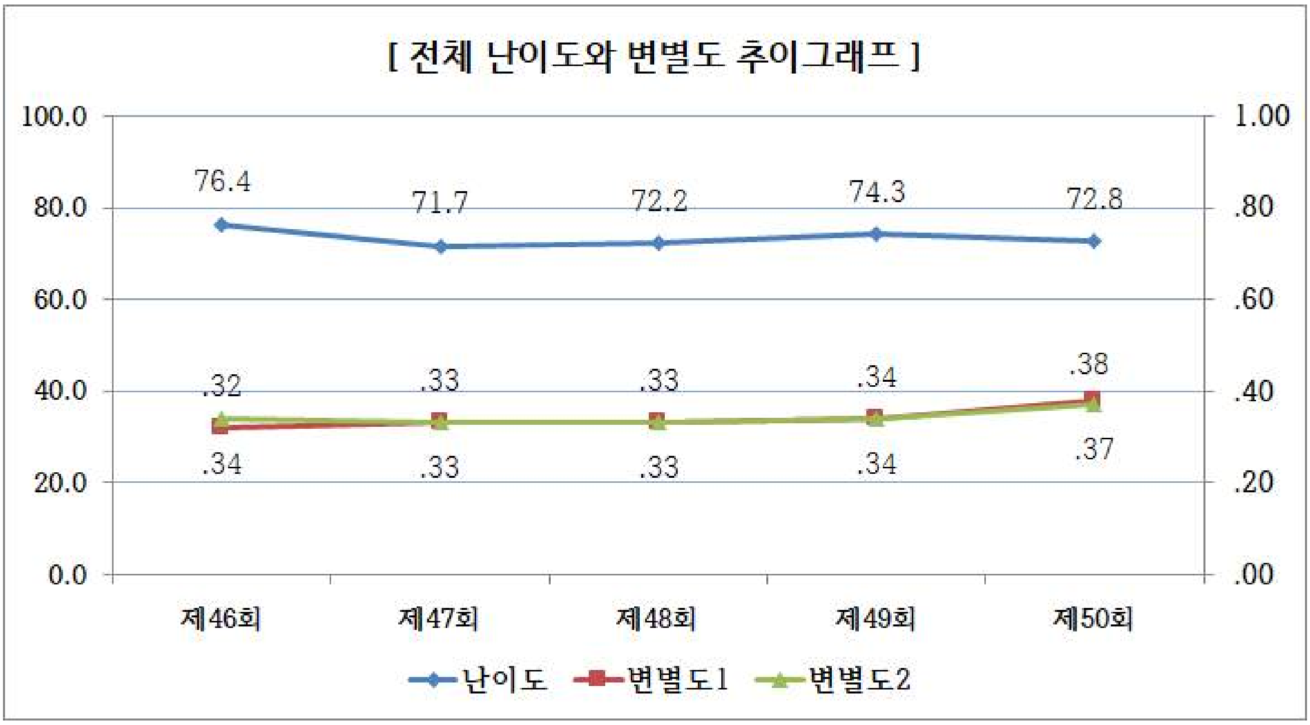

해석

- 전회 대비 난이도 지수는 1.5 감소함
- 변별도 1 지수는 .04 증가하였고, 변별도 2 지수는 .03 증가함

나) 전체 난이도와 변별도 분포도 및 비율분석

(1) 전체 난이도 분포도 및 비율분석

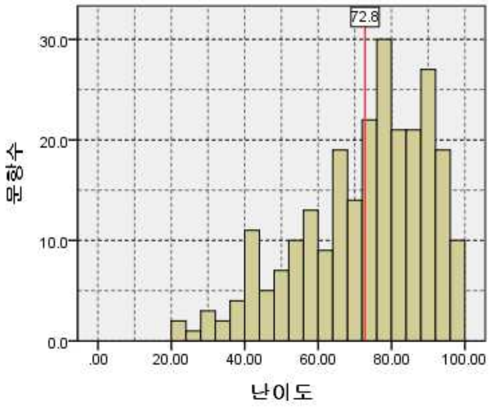

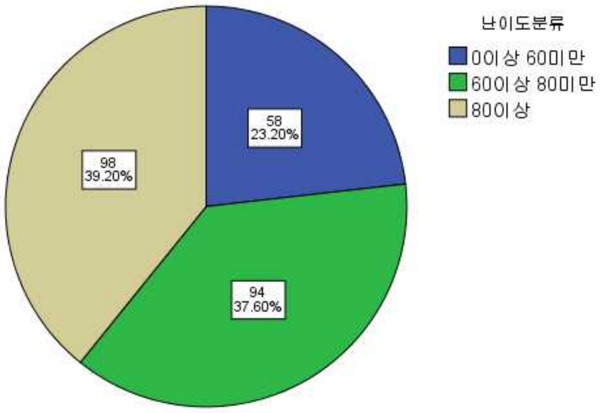

총점	난이도	표준편차
250	72.8	17.4

난이도	문항수	비율(%)
0~60미만	58	23.2
60~80미만	94	37.6
80~100	98	39.2
전체	250	100.0

(2) 전체 변별도1 분포도 및 비율분석

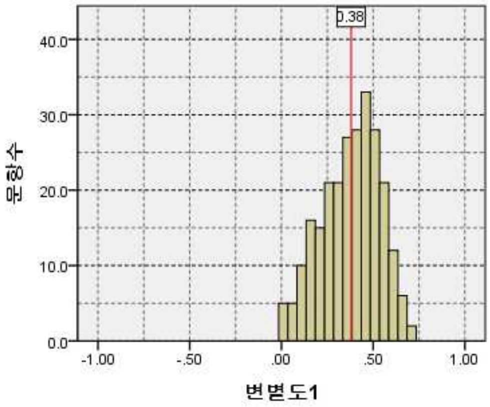

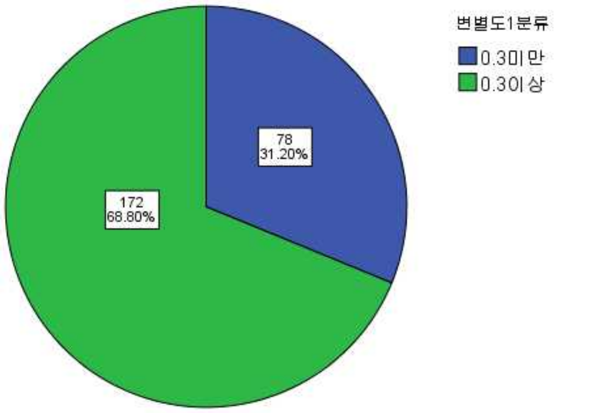

총점	변별도1	표준편차
250	.38	.16

변별도1	문항수	비율(%)
0.3미만	78	31.2
0.3이상	172	68.8
전체	250	100.0

(3) 전체 변별도2 분포도 및 비율분석

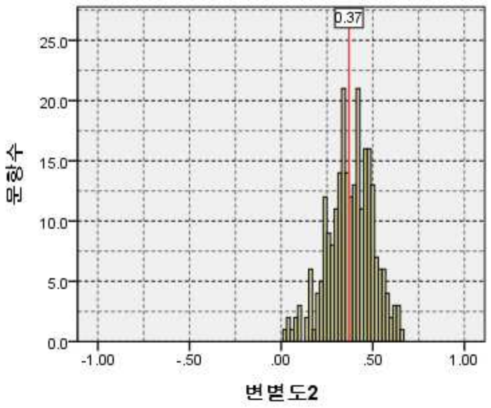

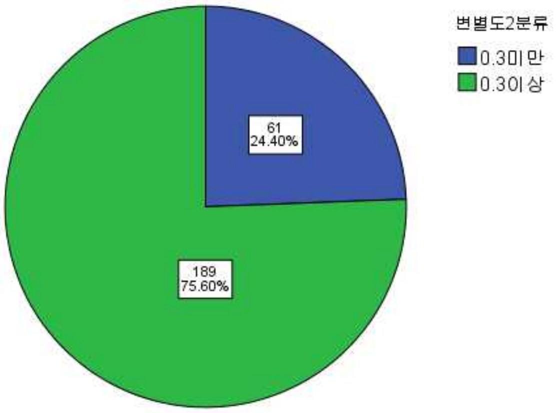

총점	변별도2	표준편차
250	.37	.13

변별도2	문항수	비율(%)
0.3미만	61	24.4
0.3이상	189	75.6
전체	250	100.0

해석

- 난이도 지수가 80 이상인 문항이 98 문항으로 가장 많았으며, 60 이상 80 미만인 문항이 94 문항, 60 미만인 문항이 58 문항으로 나타남
- 변별도 1 지수를 기준으로 분류하였을 때, 0.3 미만인 문항이 78 문항으로 0.3 이상인 문항이 172 문항인 것에 비해 더 적게 나타남
- 변별도 2 지수를 기준으로 분류하였을 때, 0.3 미만인 문항이 61 문항으로 0.3 이상인 문항이 189 문항인 것에 비해 더 적게 나타남

2) 과목별 난이도와 변별도

가) 전회 대비 과목별 난이도와 변별도

(1) 전회 대비 방사선이론 난이도와 변별도

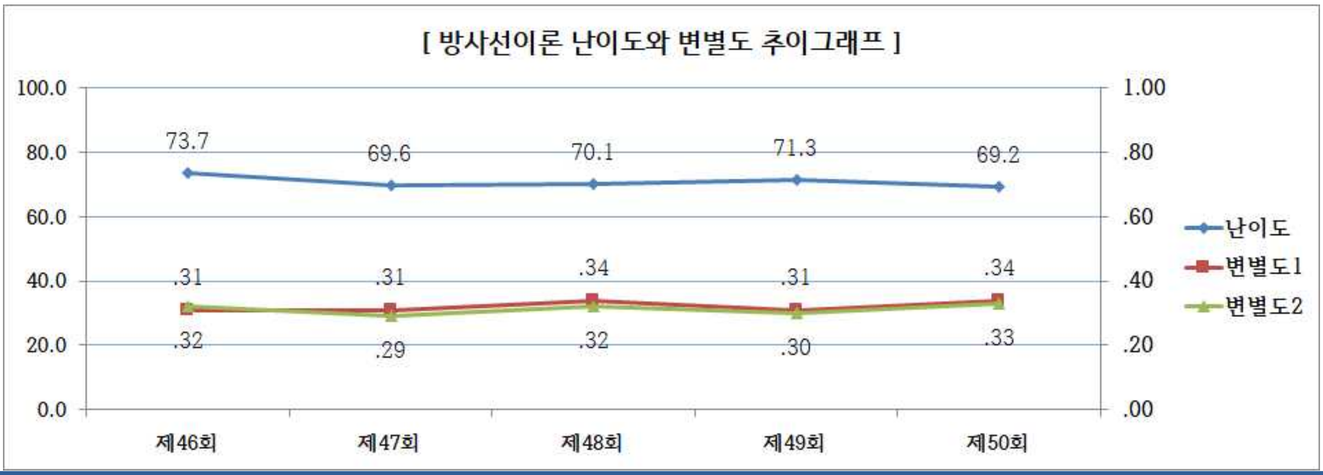

회차	난이도		변별도1		변별도2	
	평균	표준편차	평균	표준편차	평균	표준편차
제46회	73.7	16.8	.31	.12	.32	.11
제47회	69.6	18.2	.31	.14	.29	.12
제48회	70.1	18.1	.34	.14	.32	.12
제49회	71.3	17.2	.31	.15	.30	.13
**제50회**	**69.2**	**19.6**	**.34**	**.14**	**.33**	**.12**

해석

- 전회 대비 방사선이론 과목의 난이도 지수는 2.1 감소함
- 전회 대비 방사선이론 과목의 변별도 1 지수는 .03 증가함
- 전회 대비 방사선이론 과목의 변별도 2 지수는 .03 증가함

(2) 전회 대비 의료관계법규 난이도와 변별도

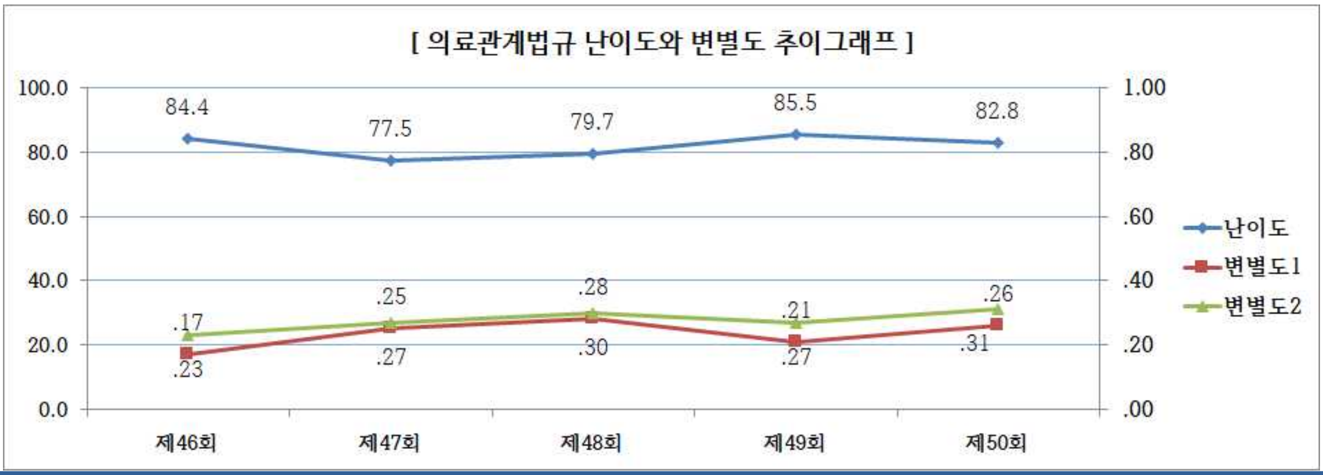

회차	난이도		변별도1		변별도2	
	평균	표준편차	평균	표준편차	평균	표준편차
제46회	84.4	12.8	.17	.09	.23	.11
제47회	77.5	19.7	.25	.12	.27	.09
제48회	79.7	17.1	.28	.16	.30	.12
제49회	85.5	11.0	.21	.15	.27	.13
**제50회**	**82.8**	**13.7**	**.26**	**.15**	**.31**	**.12**

해석

- 전회 대비 의료관계법규 과목의 난이도 지수는 2.7 감소함
- 전회 대비 의료관계법규 과목의 변별도 1 지수는 .05 증가함
- 전회 대비 의료관계법규 과목의 변별도 2 지수는 .04 증가함

(3) 전회 대비 방사선응용 난이도와 변별도

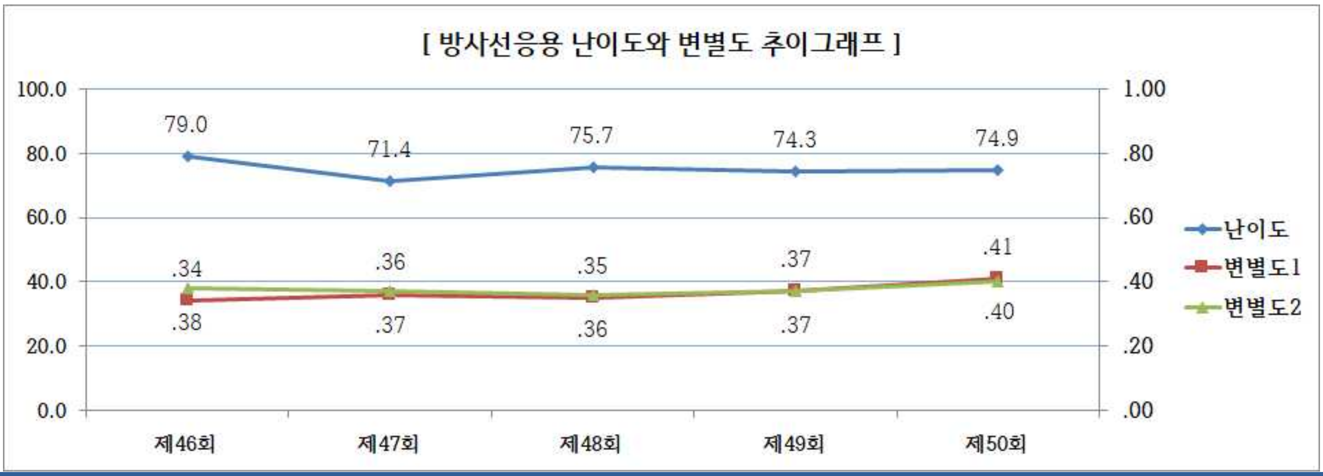

회차	난이도		변별도1		변별도2	
	평균	표준편차	평균	표준편차	평균	표준편차
제46회	79.0	13.3	.34	.14	.38	.10
제47회	71.4	17.4	.36	.14	.37	.12
제48회	75.7	15.7	.35	.15	.36	.11
제49회	74.3	16.5	.37	.14	.37	.13
**제50회**	**74.9**	**15.2**	**.41**	**.17**	**.40**	**.12**

해석

- 전회 대비 방사선응용 과목의 난이도 지수는 0.6 증가함
- 전회 대비 방사선응용 과목의 변별도 1 지수는 .04 증가함
- 전회 대비 방사선응용 과목의 변별도 2 지수는 .03 증가함

(4) 전회 대비 실기시험 난이도와 변별도

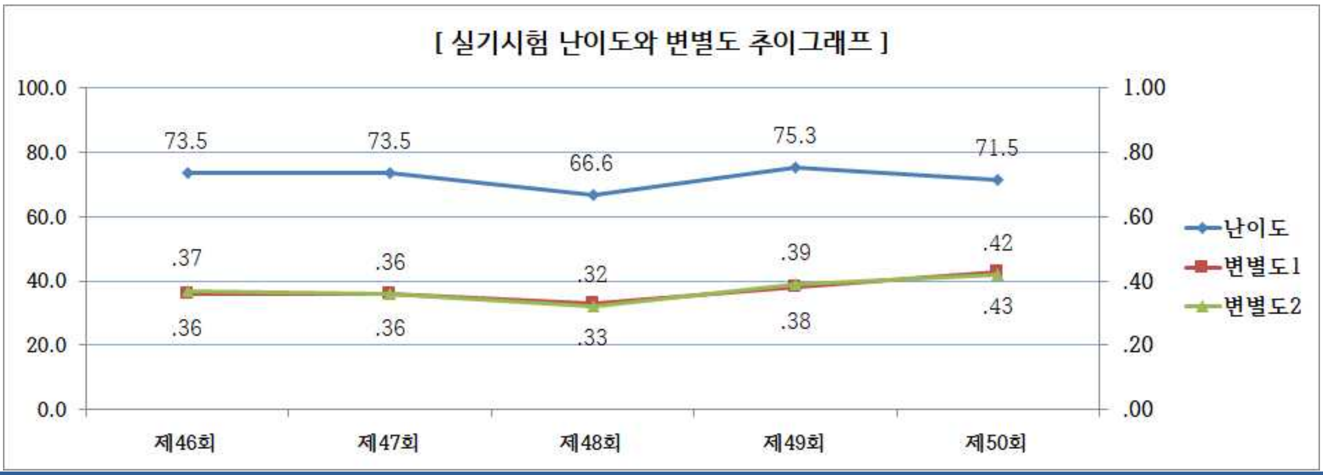

회차	난이도		변별도1		변별도2	
	평균	표준편차	평균	표준편차	평균	표준편차
제46회	73.5	18.0	.36	.15	.37	.10
제47회	73.5	15.8	.36	.15	.36	.12
제48회	66.6	22.0	.33	.18	.32	.13
제49회	75.3	16.6	.38	.17	.39	.12
**제50회**	**71.5**	**16.6**	**.43**	**.15**	**.42**	**.11**

해석

- 전회 대비 실기시험 과목의 난이도 지수는 3.8 감소함
- 전회 대비 실기시험 과목의 변별도 1 지수는 .05 증가함
- 전회 대비 실기시험 과목의 변별도 2 지수는 .03 증가함

나) 과목별 난이도와 변별도 분포도 및 비율분석

(1) 방사선기초 난이도와 변별도 분포도 및 비율분석

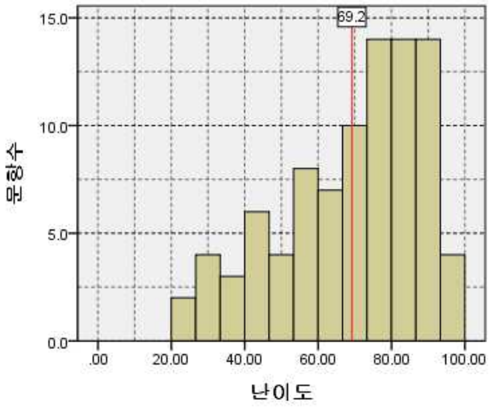

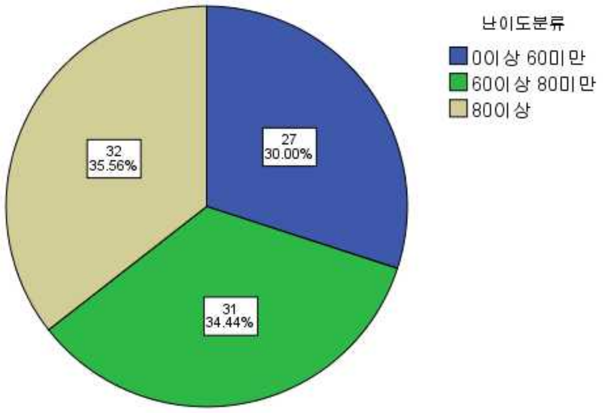

총점	난이도	표준편차
90	69.2	19.6

난이도	문항수	비율(%)
0~60미만	27	30.0
60~80미만	31	34.4
80~100	32	35.6
전체	90	100.0

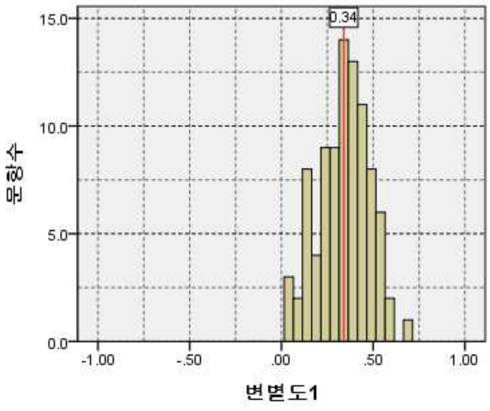

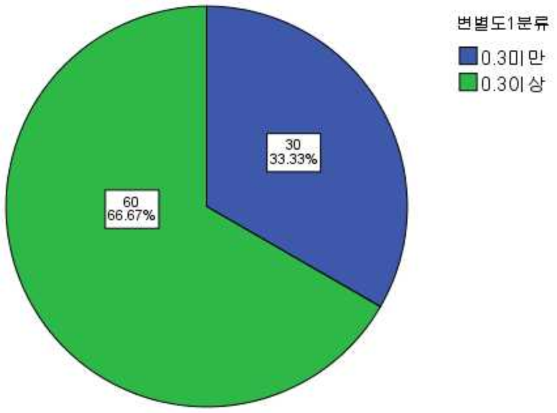

총점	변별도1	표준편차
90	.34	.14

변별도1	문항수	비율(%)
0.3미만	30	33.3
0.3이상	60	66.7
전체	90	100.0

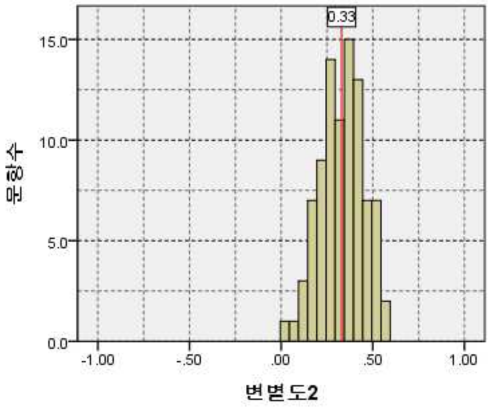

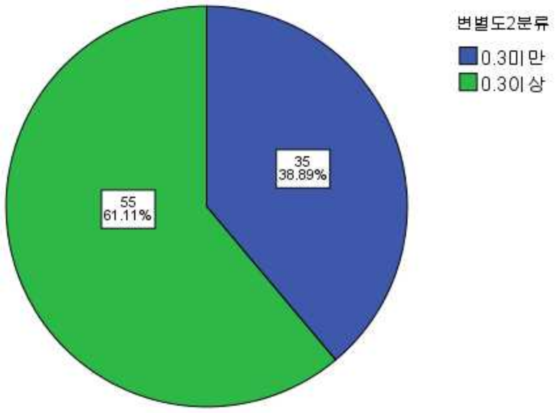

총점	변별도2	표준편차
90	.33	.12

변별도2	문항수	비율(%)
0.3미만	35	38.9
0.3이상	55	61.1
전체	90	100.0

해석

- 방사선기초 과목에서 난이도 지수가 80 이상인 문항이 32 문항으로 가장 많았으며, 60 이상 80 미만인 문항이 31 문항, 60 미만인 문항이 27 문항으로 나타남
- 변별도 1 지수를 기준으로 분류하였을 때, 0.3 미만인 문항이 30 문항으로 0.3 이상인 문항이 60 문항인 것에 비해 더 적게 나타남
- 변별도 2 지수를 기준으로 분류하였을 때, 0.3 미만인 문항이 35 문항으로 0.3 이상인 문항이 55 문항인 것에 비해 더 적게 나타남

(2) 의료관계법규 난이도와 변별도 분포도 및 비율분석

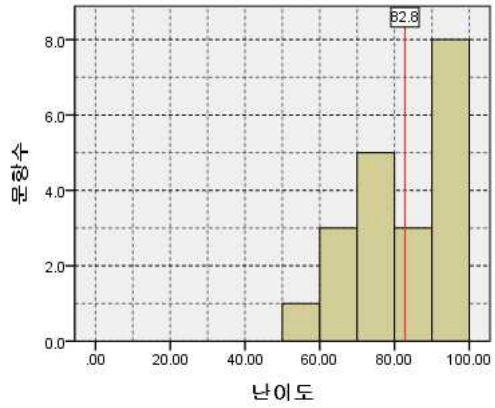

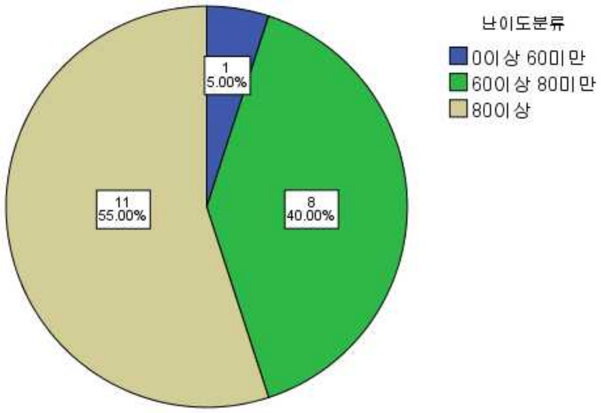

총점	난이도	표준편차
20	82.8	13.7

난이도	문항수	비율(%)
0~60미만	1	5.0
60~80미만	8	40.0
80~100	11	55.0
전체	20	100.0

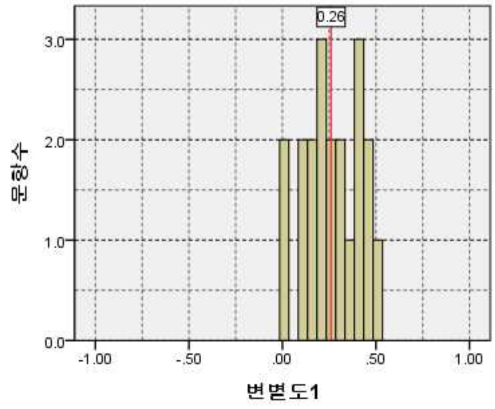

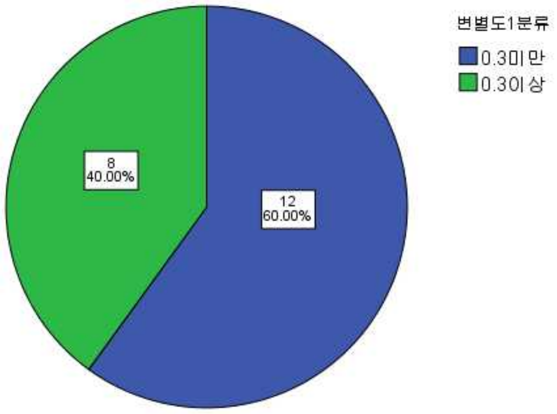

총점	변별도1	표준편차
20	.26	.15

변별도1	문항수	비율(%)
0.3미만	12	60.0
0.3이상	8	40.0
전체	20	100.0

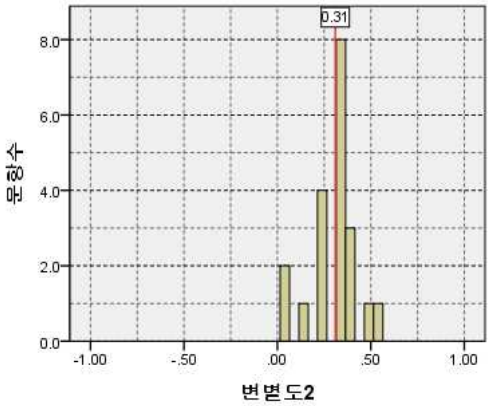

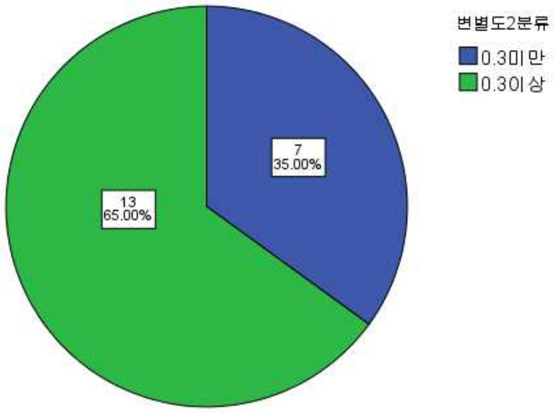

총점	변별도2	표준편차
20	.31	.12

변별도2	문항수	비율(%)
0.3미만	7	35.0
0.3이상	13	65.0
전체	20	100.0

해석

- 의료관계법규 과목에서 난이도 지수가 80 이상인 문항이 14 문항으로 가장 많았으며, 60 이상 80 미만인 문항이 8 문항, 60 미만인 문항이 1 문항으로 나타남
- 변별도 1 지수를 기준으로 분류하였을 때, 0.3 미만인 문항이 12 문항으로 0.3 이상인 문항이 8 문항인 것에 비해 더 많이 나타남
- 변별도 2 지수를 기준으로 분류하였을 때, 0.3 미만인 문항이 7 문항으로 0.3 이상인 문항이 13 문항인 것에 비해 더 적게 나타남

(3) 방사선응용 난이도와 변별도 분포도 및 비율분석

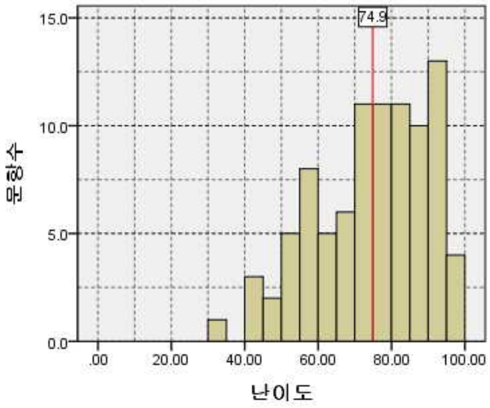

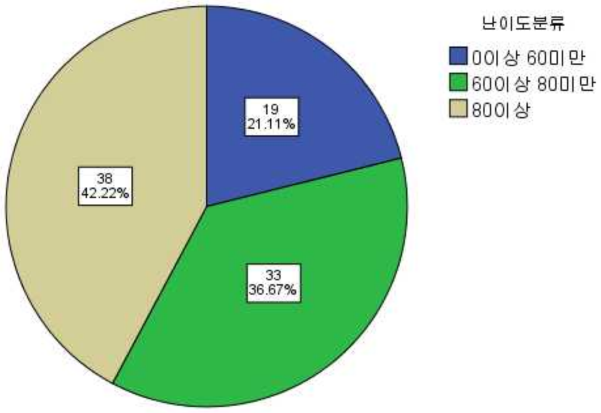

총점	난이도	표준편차
90	74.9	15.2

난이도	문항수	비율(%)
0~60미만	19	21.1
60~80미만	33	36.7
80~100	38	42.2
전체	90	100.0

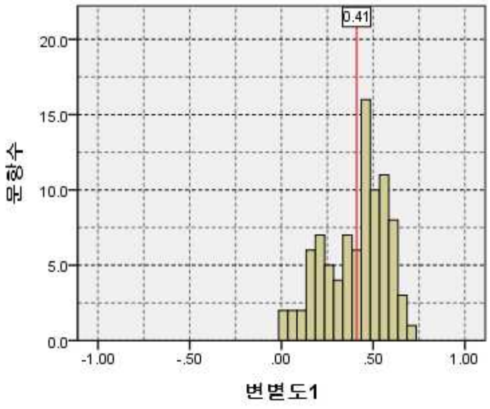

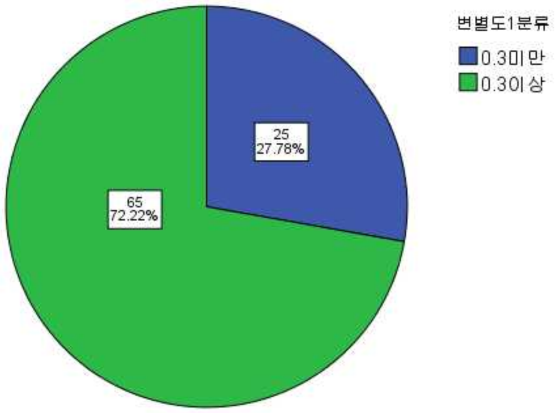

총점	변별도1	표준편차
90	.41	.17

변별도1	문항수	비율(%)
0.3미만	25	27.8
0.3이상	65	72.2
전체	90	100.0

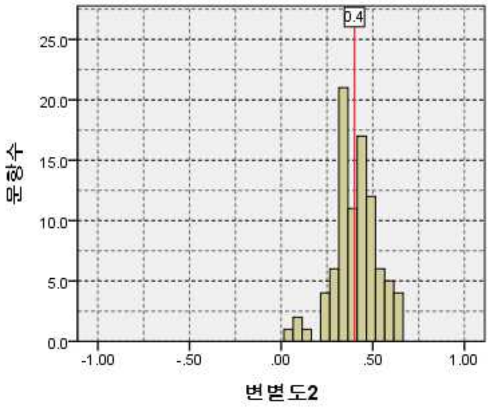

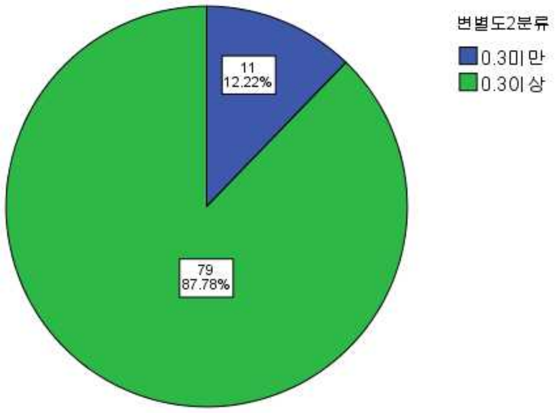

총점	변별도2	표준편차
90	.40	.12

변별도2	문항수	비율(%)
0.3미만	11	12.2
0.3이상	79	87.8
전체	90	100.0

해석

- 방사선응용 과목에서 난이도 지수가 80 이상인 문항이 38 문항으로 가장 많았으며, 60 이상 80 미만인 문항이 33 문항, 60 미만인 문항이 19 문항으로 나타남
- 변별도 1 지수를 기준으로 분류하였을 때, 0.3 미만인 문항이 25 문항으로 0.3 이상인 문항이 65 문항인 것에 비해 더 적게 나타남
- 변별도 2 지수를 기준으로 분류하였을 때, 0.3 미만인 문항이 11 문항으로 0.3 이상인 문항이 79 문항인 것에 비해 더 적게 나타남

(4) 실기시험 난이도와 변별도 분포도 및 비율분석

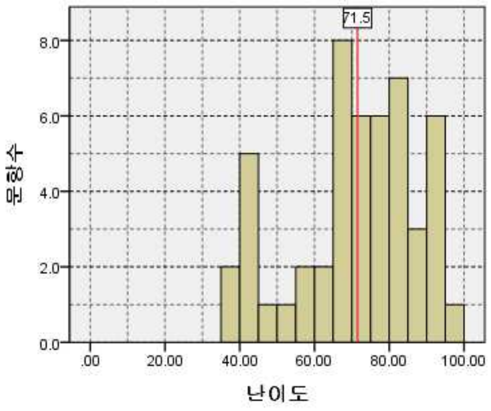

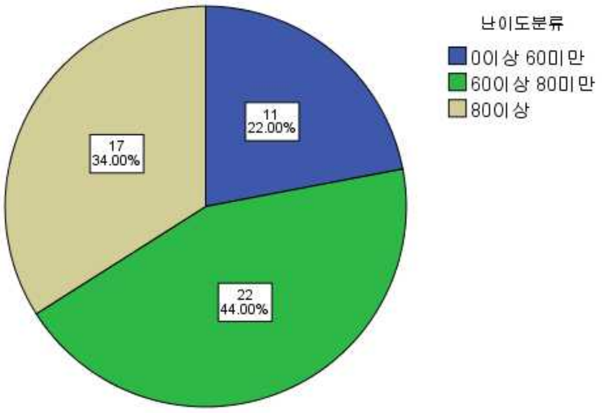

총점	난이도	표준편차
50	71.5	16.6

난이도	문항수	비율(%)
0~60미만	11	22.0
60~80미만	22	44.0
80~100	17	34.0
전체	50	100.0

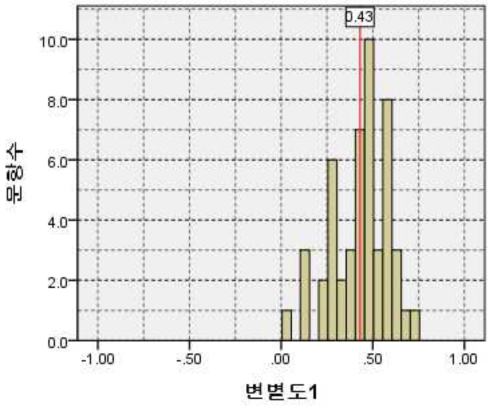

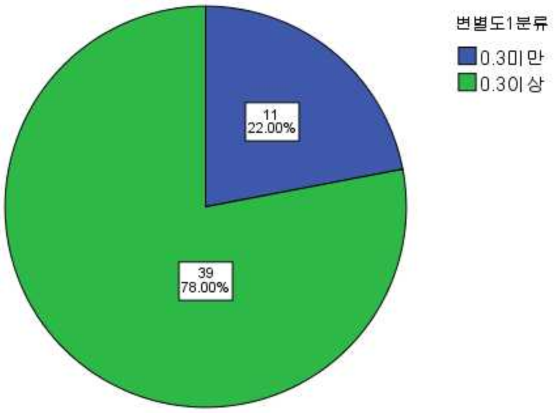

총점	변별도1	표준편차
50	.43	.15

변별도1	문항수	비율(%)
0.3미만	11	22.0
0.3이상	39	78.0
전체	50	100.0

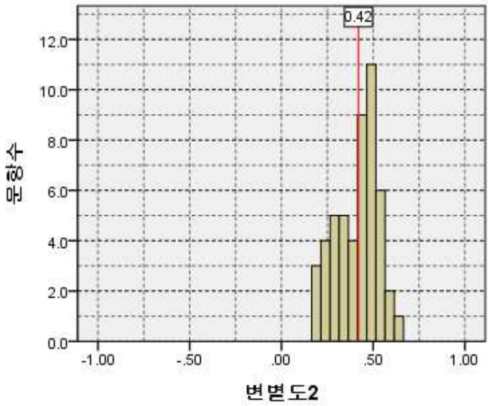

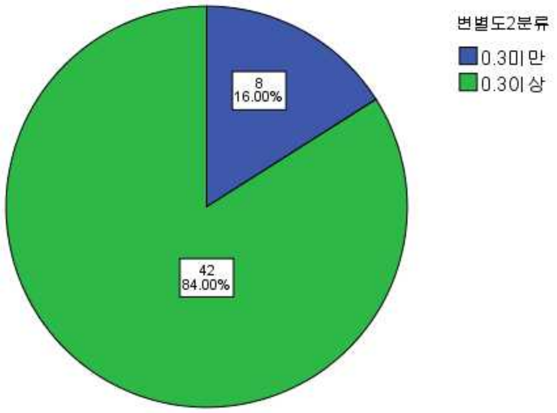

총점	변별도2	표준편차
50	.42	.11

변별도2	문항수	비율(%)
0.3미만	8	16.0
0.3이상	42	84.0
전체	50	100.0

해석

- 실기시험 과목에서 난이도 지수가 60 이상 80 미만인 문항이 22 문항으로 가장 많았으며, 80 이상인 문항이 17 문항, 60 미만인 문항이 11 문항으로 나타남
- 변별도 1 지수를 기준으로 분류하였을 때, 0.3 미만인 문항이 11 문항으로 0.3 이상인 문항이 39 문항인 것에 비해 더 적게 나타남
- 변별도 2 지수를 기준으로 분류하였을 때, 0.3 미만인 문항이 8 문항으로 0.3 이상인 문항이 42 문항인 것에 비해 더 적게 나타남

3) 지식수준별 난이도와 변별도

가) 전회 대비 지식수준별 난이도와 변별도

(1) 전회 대비 암기형 난이도와 변별도

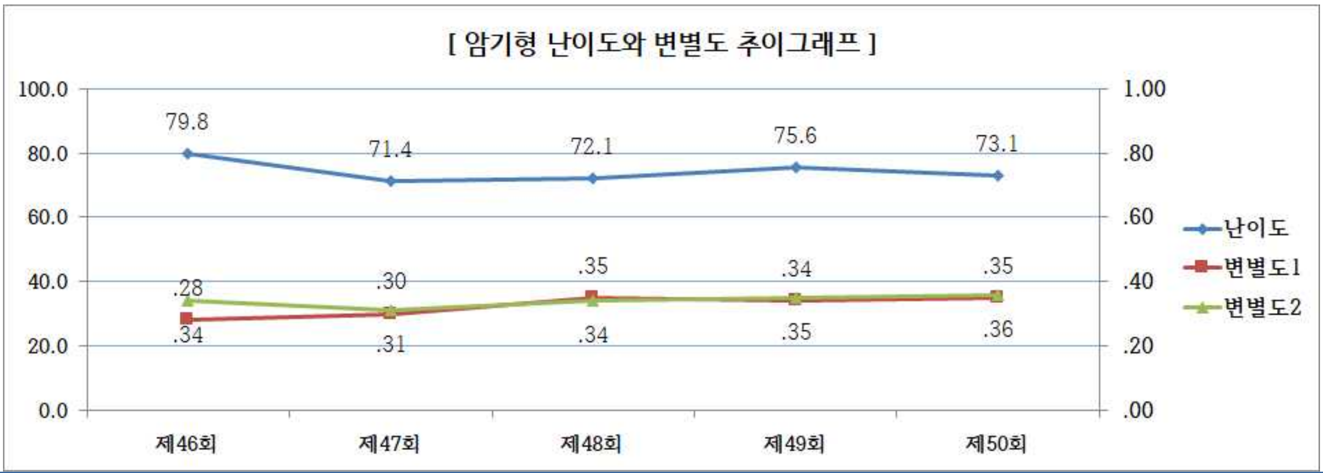

회차	난이도		변별도1		변별도2	
	평균	표준편차	평균	표준편차	평균	표준편차
제46회	79.8	16.0	.28	.12	.34	.11
제47회	71.4	18.6	.30	.14	.31	.13
제48회	72.1	18.7	.35	.15	.34	.12
제49회	75.6	13.5	.34	.14	.35	.13
**제50회**	**73.1**	**18.2**	**.35**	**.15**	**.36**	**.13**

해석

- 전회 대비 암기형 문항의 난이도 지수는 2.5 감소함
- 전회 대비 암기형 문항의 변별도 1 지수는 .01 증가함
- 전회 대비 암기형 문항의 변별도 2 지수는 .01 증가함

(2) 전회 대비 해석형 난이도와 변별도

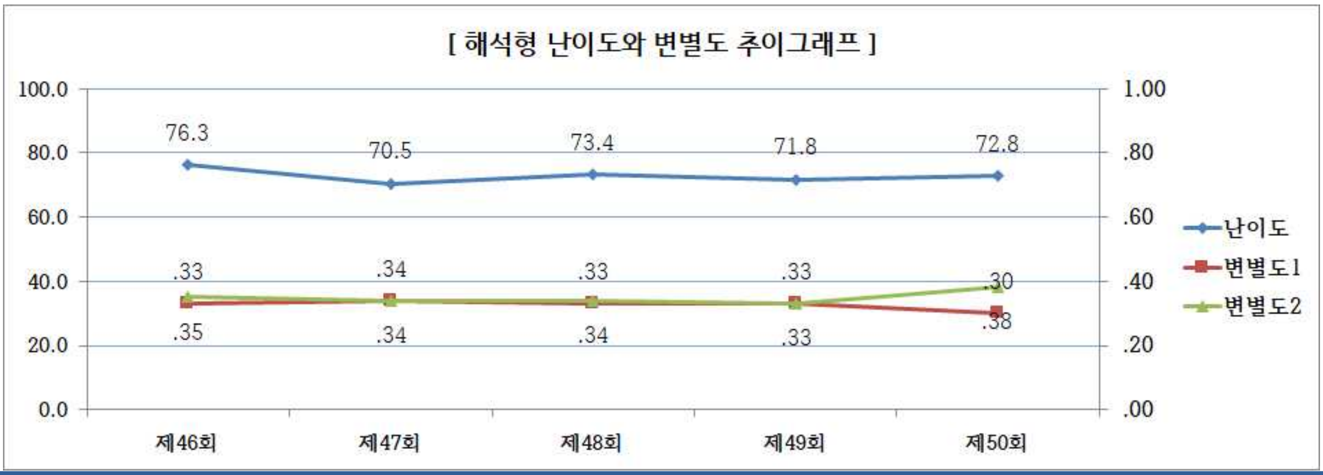

회차	난이도		변별도1		변별도2	
	평균	표준편차	평균	표준편차	평균	표준편차
제46회	76.3	15.3	.33	.13	.35	.11
제47회	70.5	17.8	.34	.14	.34	.13
제48회	73.4	16.4	.33	.14	.34	.12
제49회	71.8	18.9	.33	.16	.33	.13
**제50회**	**72.8**	**17.8**	**.30**	**.16**	**.38**	**.13**

해석

- 전회 대비 해석형 문항의 난이도 지수는 1.0 증가함
- 전회 대비 해석형 문항의 변별도 1 지수는 .03 감소함
- 전회 대비 해석형 문항의 변별도 2 지수는 .05 증가함

(3) 전회 대비 해결형 난이도와 변별도

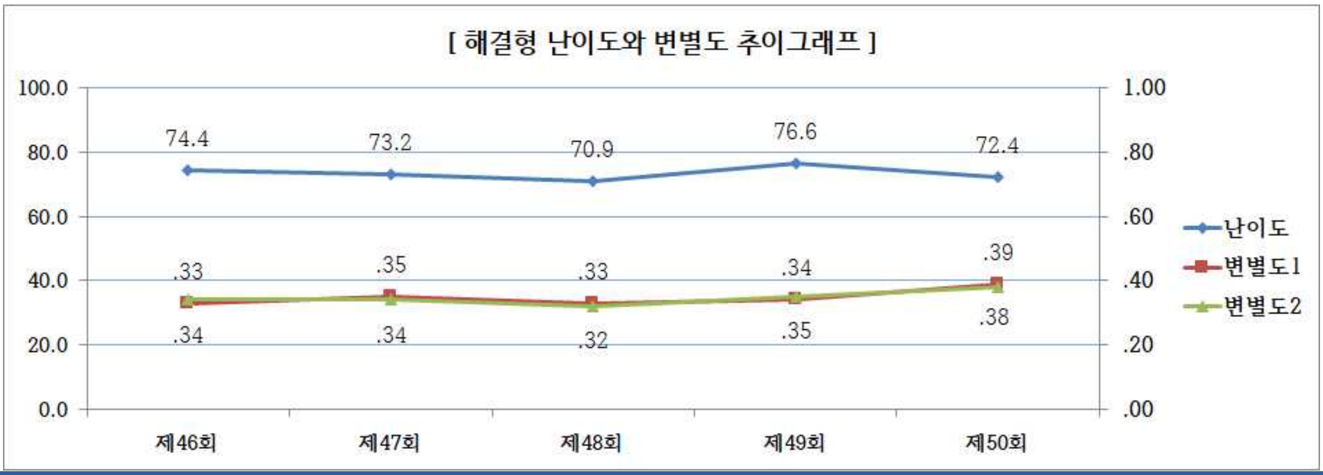

회차	난이도		변별도1		변별도2	
	평균	표준편차	평균	표준편차	평균	표준편차
제46회	74.4	16.5	.33	.15	.34	.12
제47회	73.2	16.6	.35	.15	.34	.11
제48회	70.9	20.4	.33	.17	.32	.12
제49회	76.6	16.4	.34	.17	.35	.14
**제50회**	**72.4**	**15.7**	**.39**	**.15**	**.38**	**.12**

해석

- 전회 대비 해결형 문항의 난이도 지수는 4.2 감소함
- 전회 대비 해결형 문항의 변별도 1 지수는 .05 증가함
- 전회 대비 해결형 문항의 변별도 2 지수는 .03 증가함

나) 지식수준별 난이도와 변별도 분포도 및 비율분석

(1) 암기형 난이도와 변별도 분포도 및 비율분석

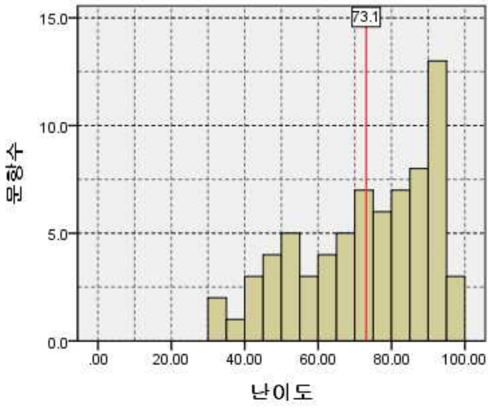

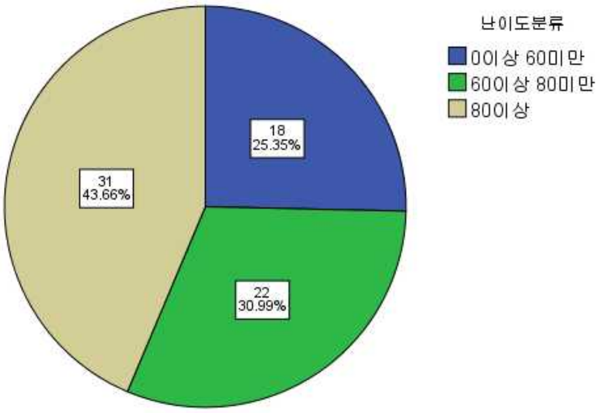

총점	난이도	표준편차
71	73.1	18.2

난이도	문항수	비율(%)
0~60미만	18	25.4
60~80미만	22	31.0
80~100	31	43.7
전체	71	100.0

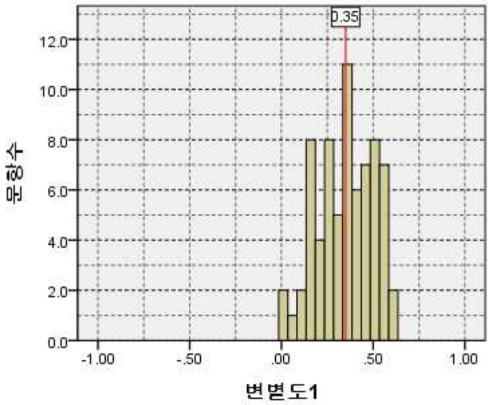

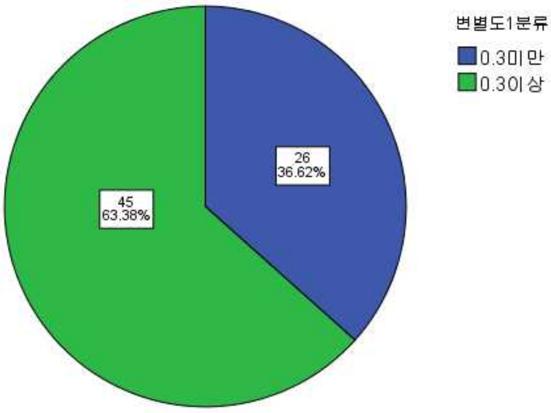

총점	변별도1	표준편차
71	.35	.15

변별도1	문항수	비율(%)
0.3미만	26	36.6
0.3이상	45	63.4
전체	71	100.0

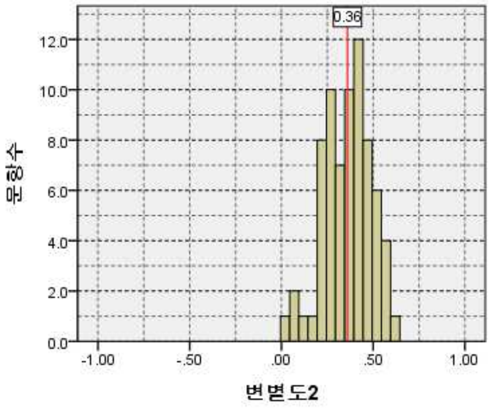

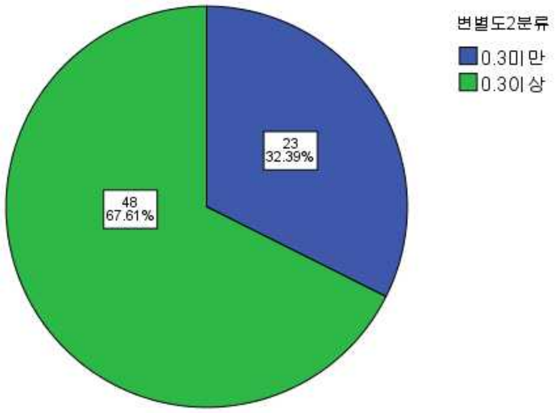

총점	변별도2	표준편차
71	.36	.13

변별도2	문항수	비율(%)
0.3미만	23	32.4
0.3이상	48	67.6
전체	71	100.0

해석

- 암기형 문항에서 난이도 지수가 80 이상인 문항이 31 문항으로 가장 많았으며, 60 이상 80 미만인 문항이 22 문항, 60 미만인 문항이 18 문항으로 나타남
- 변별도 1 지수를 기준으로 분류하였을 때, 0.3 미만인 문항이 26 문항으로 0.3 이상인 문항이 45 문항인 것에 비해 더 적게 나타남
- 변별도 2 지수를 기준으로 분류하였을 때, 0.3 미만인 문항이 23 문항으로 0.3 이상인 문항이 48 문항인 것에 비해 더 적게 나타남

(2) 해석형 난이도와 변별도 분포도 및 비율분석

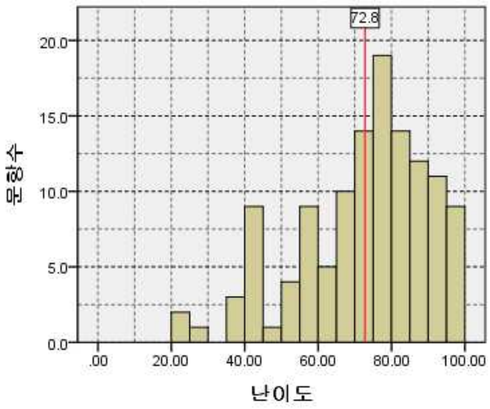

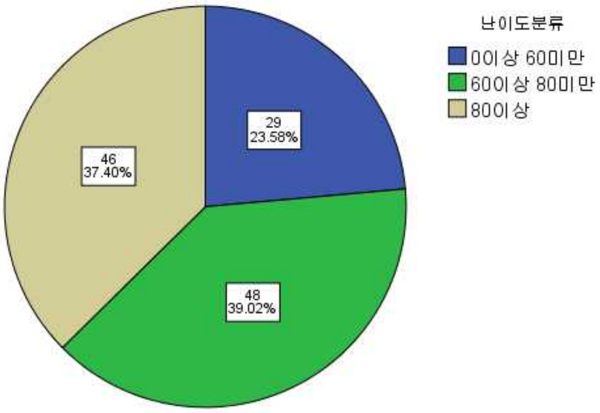

총점	난이도	표준편차	난이도	문항수	비율(%)
123	72.8	17.8	0~60미만	29	23.6
			60~80미만	48	39.0
			80~100	46	37.4
			전체	123	100.0

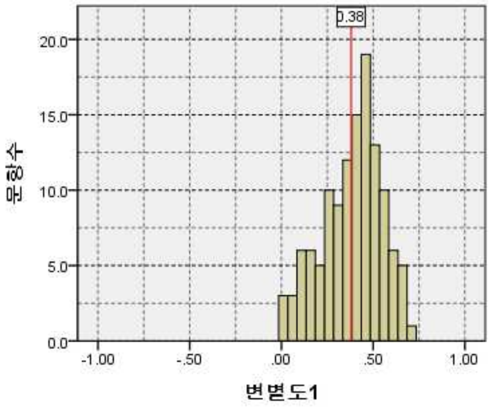

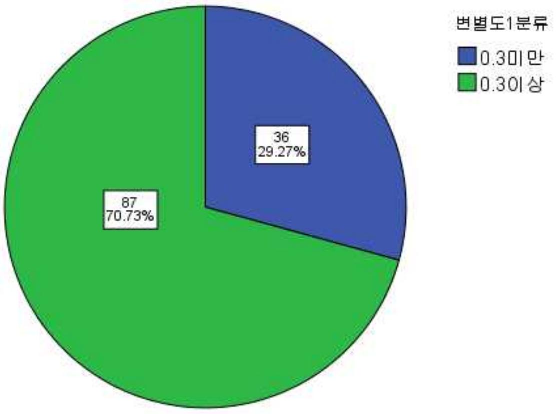

총점	변별도1	표준편차	변별도1	문항수	비율(%)
123	.38	.16	0.3미만	36	29.3
			0.3이상	87	70.7
			전체	123	100.0

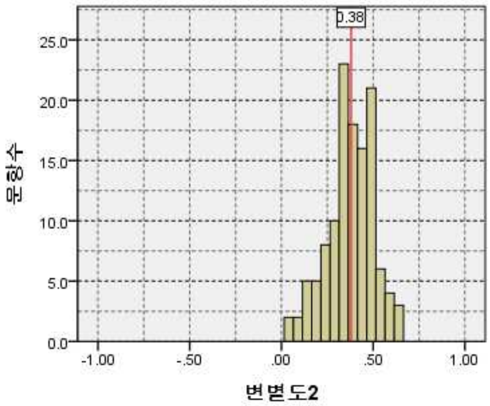

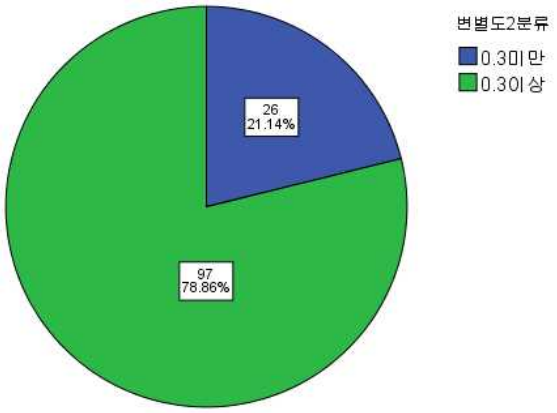

총점	변별도2	표준편차
123	.38	.13

변별도2	문항수	비율(%)
0.3미만	26	21.1
0.3이상	97	78.9
전체	123	100.0

해석

- 해석형 문항에서 난이도 지수가 60 이상 80 미만인 문항이 48 문항으로 가장 많았으며, 80 이상인 문항이 46 문항, 60 미만인 문항이 29 문항으로 나타남
- 변별도 1 지수를 기준으로 분류하였을 때, 0.3 미만인 문항이 36 문항으로 0.3 이상인 문항이 87 문항인 것에 비해 더 적게 나타남
- 변별도 2 지수를 기준으로 분류하였을 때, 0.3 미만인 문항이 26 문항으로 0.3 이상인 문항이 97 문항인 것에 비해 더 적게 나타남

(3) 해결형 난이도와 변별도 분포도 및 비율분석

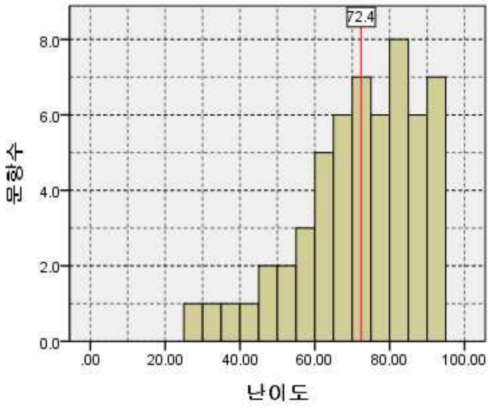

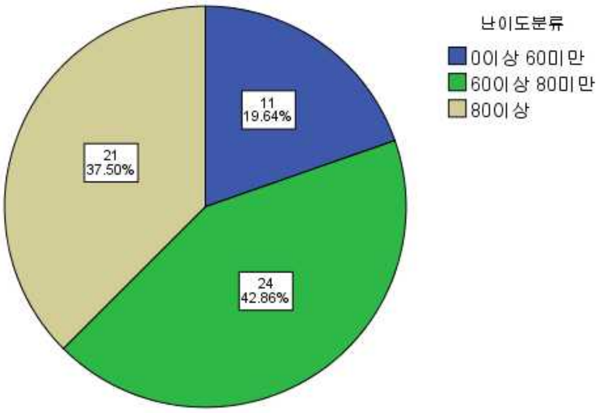

총점	난이도	표준편차
56	72.4	15.7

난이도	문항수	비율(%)
0~60미만	11	19.6
60~80미만	24	42.9
80~100	21	37.5
전체	56	100.0

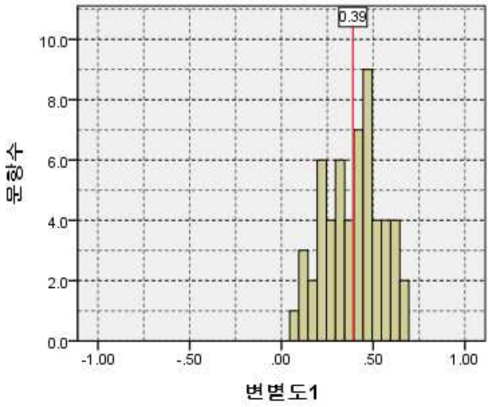

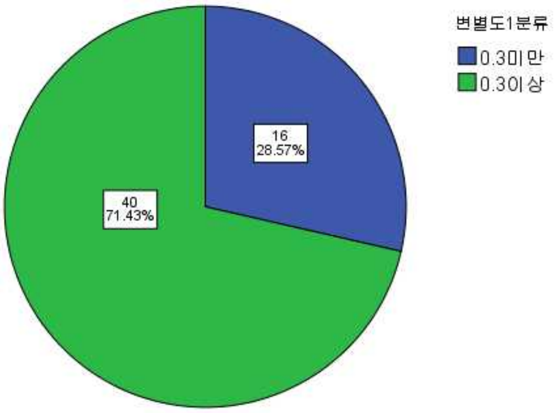

총점	변별도1	표준편차
56	.39	.15

변별도1	문항수	비율(%)
0.3미만	16	28.6
0.3이상	40	71.4
전체	56	100.0

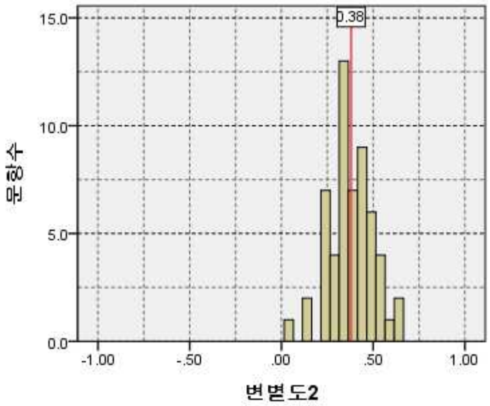

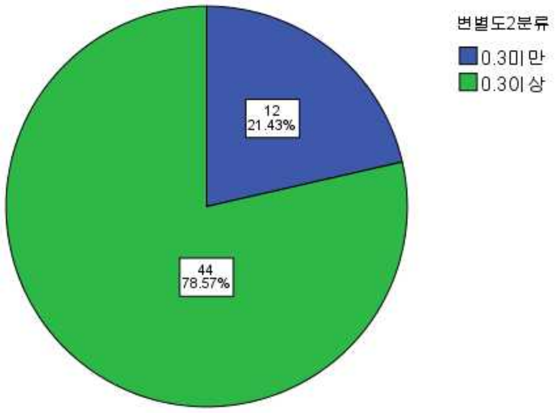

총점	변별도2	표준편차	변별도2	문항수	비율(%)
56	.38	.12	0.3미만	12	21.4
			0.3이상	44	78.6
			전체	56	100.0

해석

- 해결형 문항에서 난이도 지수가 60 이상 80 미만인 문항이 24 문항으로 가장 많았으며, 80 이상인 문항이 21 문항, 60 미만인 문항이 11 문항으로 나타남
- 변별도 1 지수를 기준으로 분류하였을 때, 0.3 미만인 문항이 16 문항으로 0.3 이상인 문항이 40 문항인 것에 비해 더 적게 나타남
- 변별도 2 지수를 기준으로 분류하였을 때, 0.3 미만인 문항이 12 문항으로 0.3 이상인 문항이 44 문항인 것에 비해 더 적게 나타남

3. 난이도와 변별도 간 산포도

1) 전체 난이도와 변별도 간 산포도

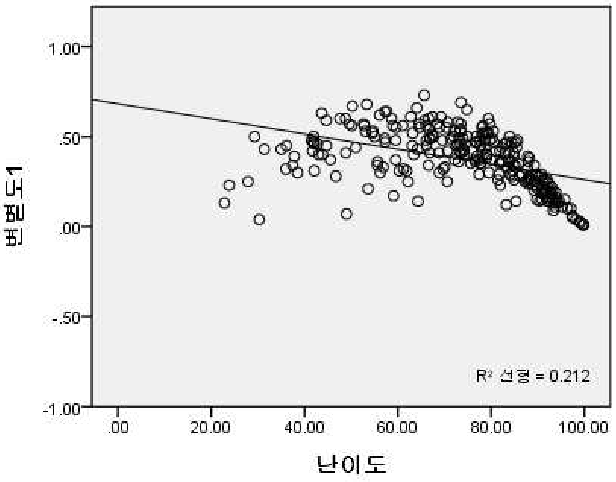

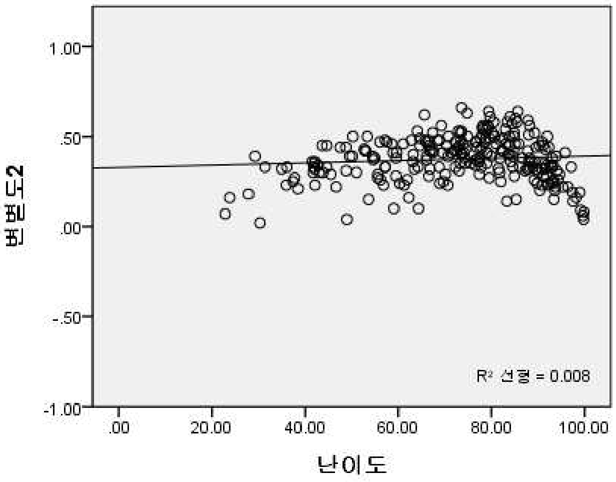

해석

- 전체 문항을 대상으로 한 난이도 지수와 변별도 1 지수 간 상관은 -.461**로 난이도 지수가 높을수록 변별력이 낮아지는 것으로 나타남
- 난이도 지수와 변별도 2 지수 간 상관은 .088 로 난이도 지수와 변별력 간에 관련성이 없는 것으로 나타남

2) 과목별 난이도와 변별도 간 산포도

가) 방사선이론 난이도와 변별도 간 산포도

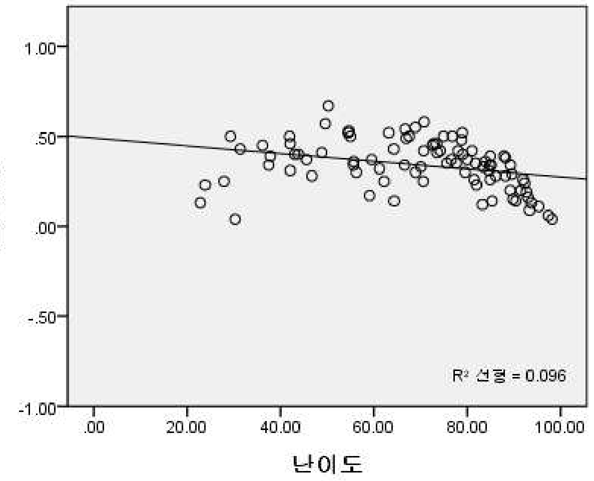

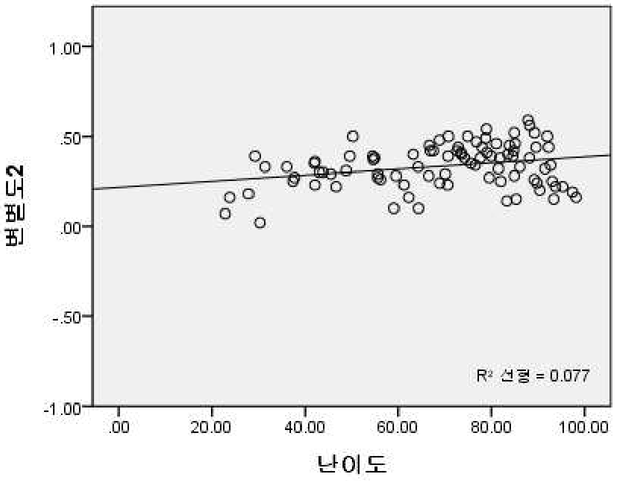

해석

- 방사선이론 과목 문항을 대상으로 한 난이도 지수와 변별도 1 지수 간 상관은 -.310**로 난이도 지수가 높을수록 변별력이 낮아지는 것으로 나타남
- 난이도 지수와 변별도 2 지수 간 상관은 277**로 난이도 지수가 높을수록 변별력이 높아지는 것으로 나타남

나) 의료관계법규 난이도와 변별도 간 산포도

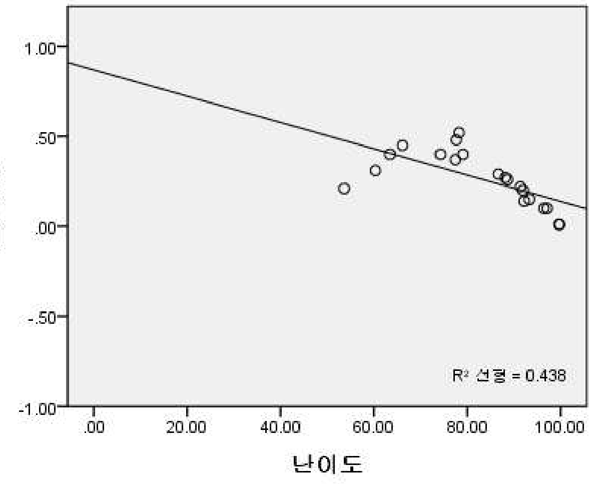

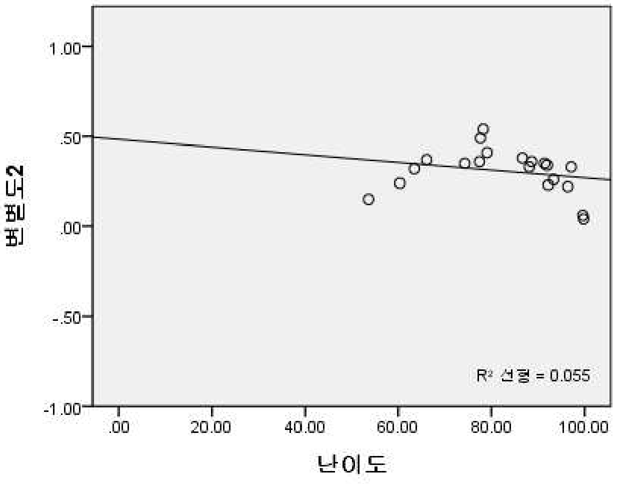

해석

- 의료관계법규 과목 문항을 대상으로 한 난이도 지수와 변별도 1 지수 간 상관은 -.661**로 난이도 지수가 높을수록 변별력이 낮아지는 것으로 나타남
- 난이도 지수와 변별도 2 지수 간 상관은 -.235 로 난이도 지수와 변별력 간에 관련성이 없는 것으로 나타남

다) 방사선응용 난이도와 변별도 간 산포도

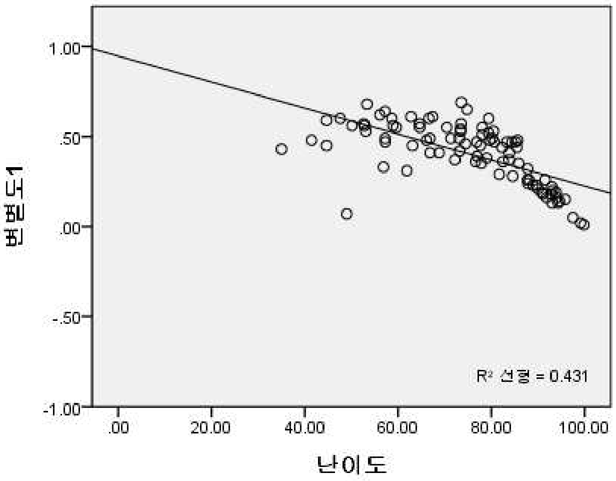

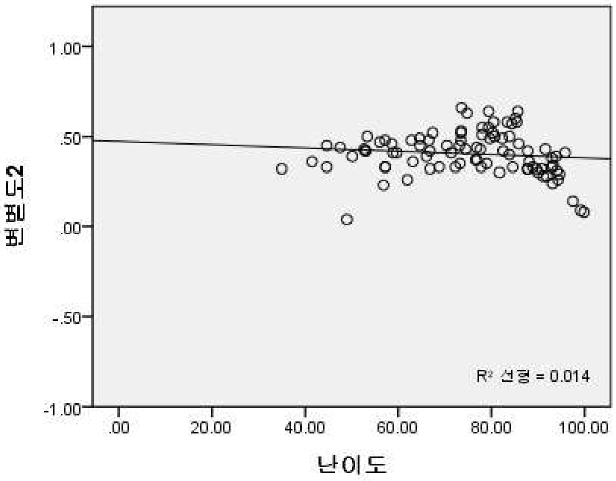

해석

- 방사선응용 과목 문항을 대상으로 한 난이도 지수와 변별도 1 지수 간 상관은 -.656**으로 난이도 지수가 높을수록 변별력이 낮아지는 것으로 나타남
- 난이도 지수와 변별도 2 지수 간 상관은 -.117 로 난이도 지수와 변별력 간에 관련성이 없는 것으로 나타남

라) 실기시험 난이도와 변별도 간 산포도

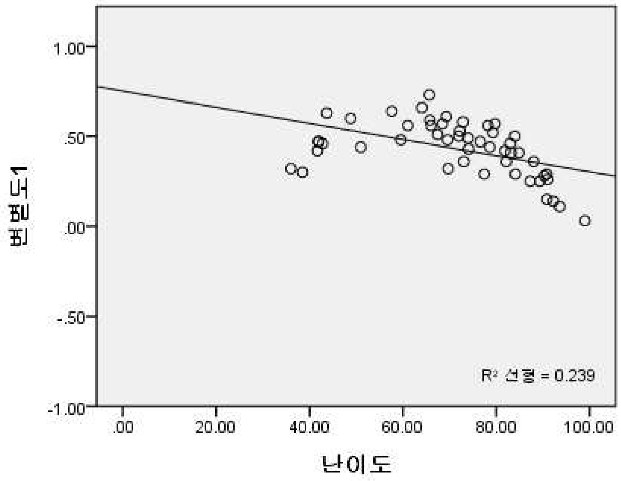

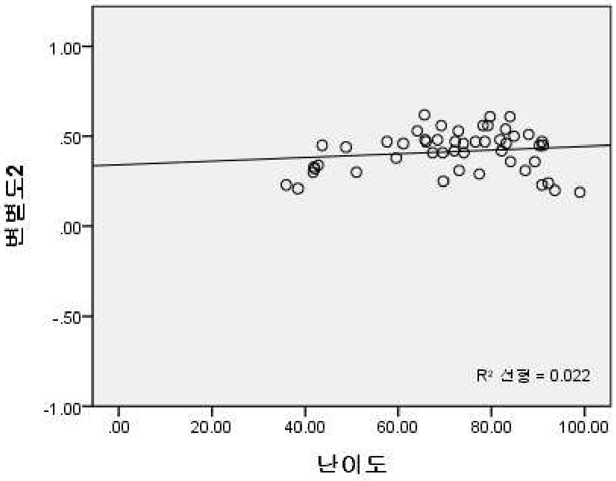

해석

- 실기시험 과목 문항을 대상으로 한 난이도 지수와 변별도1 지수 간 상관은 -.489**으로 난이도 지수가 높을수록 변별력이 낮아지는 것으로 나타남
- 난이도 지수와 변별도2 지수 간 상관은 .149로 난이도 지수와 변별력 간에 관련성이 없는 것으로 나타남

4. 신뢰도 분석

과목명	문항수	제46회	제47회	제48회	제49회	제50회
전체	250	.970	.970	.969	.971	**.976**
방사선이론	90	.911	.903	.916	.904	**.920**
의료관계법규	20	.557	.674	.729	.656	**.715**
방사선응용	90	.937	.937	.933	.935	**.948**
실기시험	50	.889	.892	.853	.904	**.914**

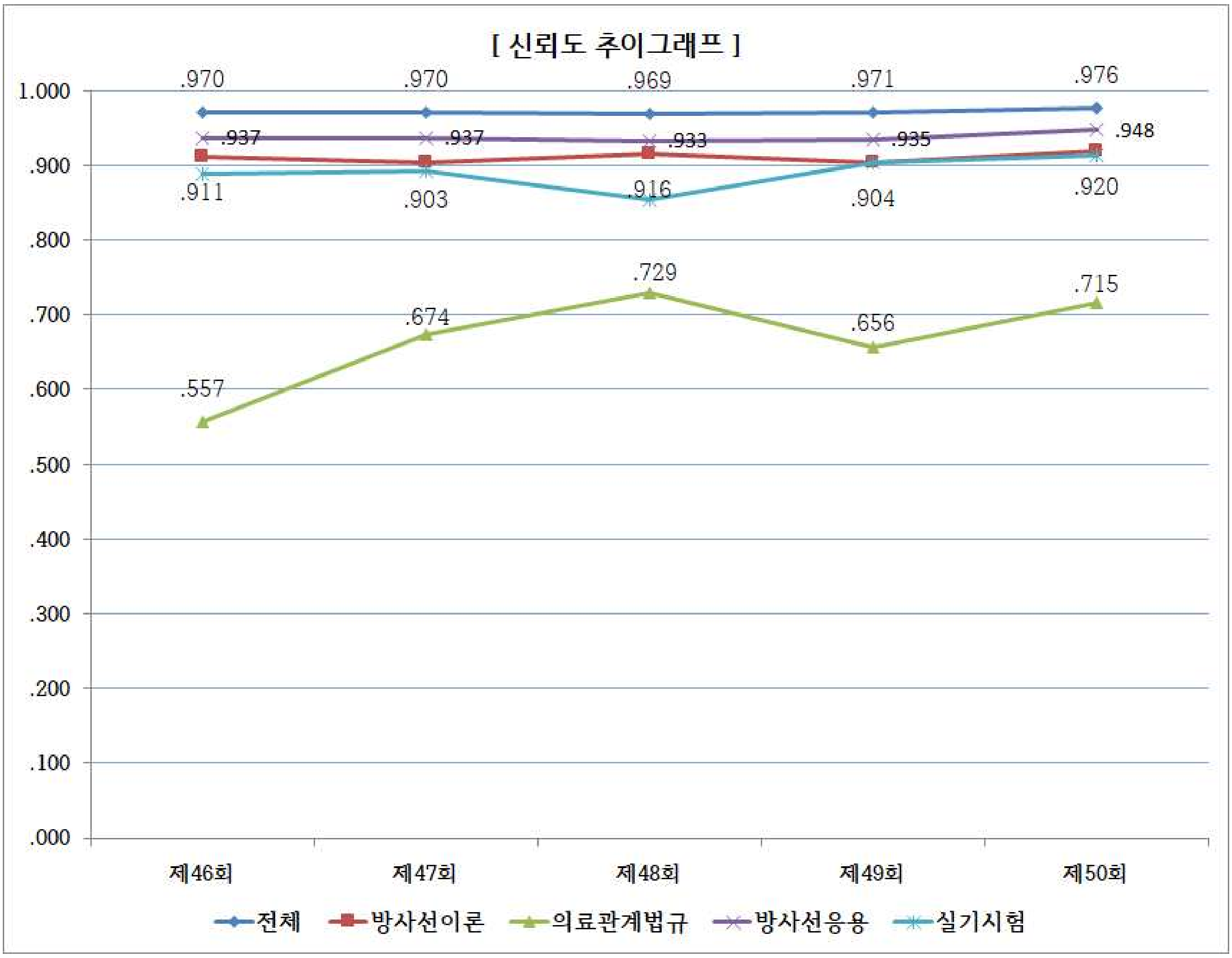

해석

- 전회 대비 전체 문항의 신뢰도는 .005 증가함
- 전회 대비 방사선이론 과목 문항의 신뢰도는 .016 증가함
- 전회 대비 의료관계법규 과목 문항의 신뢰도는 .059 증가함
- 전회 대비 방사선응용 과목 문항의 신뢰도는 .013 증가함
- 전회 대비 실기시험 과목 문항의 신뢰도는 .010 증가함

○ 분석결과 관련 문의 : 한국보건의료인국가시험원 연구개발본부 김보현 전임연구원
Tel : 02-2087-8954, FAX : 02-2087-8885
E-mail : kimbohyun@kuksiwon.or.kr
