# Supplementary material for: Item difficulty index, discrimination index, and reliability of the 26 health professions licensing examinations in 2022, Korea: a psychometric study
Source: J Educ Eval Health Prof. 2023 Nov 22;20:31. doi: 10.3352/jeehp.2023.20.31 (PMC11959405; doi:10.3352/jeehp.2023.20.31)
Supplement: Supplementary file 1 — Supplement 1. Item analysis results of 26 health professions licensing examinations administered during late 2022 and early 2023. [file jeehp-20-31_Suppl1.zip › 2022│Γ╡╡ ┴a50╚╕ └╙╗≤║┤╕«╗τ ▒╣░í╜├╟Φ ║╨╝«░ß░·.pdf]

2022년도 제50회 임상병리사 국가시험 문항분석 결과

국민이 신뢰하고 감동하는 시험평가기관
한국보건의료인국가시험원
KOREA HEALTH PERSONNEL LICENSING EXAMINATION INSTITUTE

일반 용어 정의

□ **평균**

- 집단에서의 대표적 경향값으로 전체 값을 더하여 총 응시자로 나눈 값

□ **표준편차**

- 평균과 각 점수의 차이인 편차들의 평균으로 점수가 흩어져 분포되어 있는 정도

□ **검사이론**

- 검사와 검사를 구성하고 있는 문항의 양호도를 분석 및 평가하는 방법을 정의한 이론체계
- 대표적으로 고전검사이론과 문항반응이론이 있음

고전검사이론 용어 정의

□ **고전검사이론(Classical Test Theory; CTT)**

- 검사의 질을 분석하는 검사이론 중 한 가지로 19세기 말부터 전개되어 현재까지 주로 사용되고 있는 검사이론임
- 고전검사이론에 의한 문항과 응시자 능력 추정치는 다음과 같음

○ **문항난이도**

· 검사 문항의 쉽고 어려운 정도를 나타내는 지수
· 난이도 지수는 총 반응 수에 대한 정답 반응 수의 비율로 문항의 정답률임
· 문항난이도는 0~100까지의 값을 가짐
· 난이도 값이 큰 경우, 쉬운 문항으로 '난이도가 낮다'라고 해석하며, 난이도 값이 작은 경우, 어려운 문항으로 '난이도가 높다'라고 해석함

○ **문항변별도**

· 각 문항이 응시자의 능력 수준을 변별할 수 있는 정도를 나타내는 지수
· 문항변별도는 −1~+1까지의 값을 가지며, 1에 가까울수록 변별력 크다고 해석함
· 일반적으로 문항변별도가 0.3 이상이면 우수한 문항으로 평가함
· 구하는 방식에는 '상하위집단 구분법', '문항-총점 상관계수' 등이 있음
  1) **변별도 1(상하위구분법):** 상위 27%와 하위 27% 집단의 난이도 차이를 구하는 방식
  2) **변별도 2(상관계수법):** 문항-총점과의 상관계수로 구하는 방식

○ **신뢰도**

· 시험이 평가하고자 하는 것을 일관성 있게 측정하는가로 시험이 오차없이 정확하게 측정한 정도를 의미함
· 국시원에서는 문항의 내적일관성(Cronbach α)으로 신뢰도를 추정하며 1에 가까울수록 신뢰도가 높다고 해석함

목 차

I. 시행 결과 ········· 5

1. 시험 현황 ········· 6

1) 시험명: 2022년도 제50회 임상병리사 국가시험 ········· 6

2) 시험시행일: 2022년 12월 11일 ········· 6

3) 응시현황 ········· 6

4) 과목별 문항 수, 배점 및 과락 점수 ········· 6

2. 합격률과 평균성적 ········· 6

1) 합격 및 불합격 현황 ········· 6

2) 과목별 과락자수 내역 ········· 6

3) 전회 대비 합격률과 평균성적 ········· 7

II. 문항분석 결과 ········· 9

1. 성적 ········· 10

1) 전체 성적분포도 ········· 10

2) 과목별 성적분포도(※ 필기형 실기 포함) ········· 11

2. 난이도와 변별도 ········· 12

1) 전체 난이도와 변별도 ········· 12

2) 과목별 난이도와 변별도 ········· 15

3) 지식수준별 난이도와 변별도 ········· 26

4) 자료유형별 난이도와 변별도 ········· 34

3. 난이도와 변별도 간 산포도 ········· 40

1) 전체 난이도와 변별도 간 산포도 ········· 40

2) 과목별 난이도와 변별도 간 산포도 ········· 40

4. 신뢰도 분석 ········· 43

I. 시행 결과

1. 시험 현황

1) 시험명: 2022년도 제50회 임상병리사 국가시험

2) 시험시행일: 2022년 12월 11일

3) 응시현황

응시대상자수	결시자수	부정행위자수	응시자 준수사항 위반자 수		응시자수<br>(%)
			휴대폰 소지	신분증 미지참	
3,024	106	0	0	0	2,917※<br>(96.3)

4) 과목별 문항 수, 배점 및 과락 점수

교 시	과 목 명	문제 수	배점	총점	합격자 점수기준	
					과목 과락기준	총점 합격기준
1교시	의료관계법규	20	1	20	8점 미만	129점 이상
	임상검사이론 I	80	1	80	32점 미만	
2교시	임상검사이론 II	115	1	115	46점 미만	
3교시	실기시험	65	1	65	39점 미만	39점 이상
계		280		280		

2. 합격률과 평균성적

1) 합격 및 불합격 현황

합격자수<br>(%)	불합격자수(%)					채점보류자수
	평락	과락	실기탈락	기권	계	
**2,561<br>(87.8)**	307<br>(10.6)	0<br>(0.0)	49<br>(1.7)	0<br>(0.0)	356<br>(12.2)	1<br>(0.0)

2) 과목별 과락자수 내역

과목명 / 과락자수	의료관계법규	임상검사이론 I	임상검사이론 II	실기시험
과목별 과락자 수	0	0	0	-
전과목 과락자 수	0			

3) 전회 대비 합격률과 평균성적

회차	년도	합격률(%)	평균성적	표준편차	백분율 환산점수
제46회	2019	64.2	188.6	50.3	67.4
제47회	2019	86.7	218.8	46.0	78.2
제48회	2020	80.7	210.6	51.7	75.2
제49회	2021	84.8	212.5	50.6	75.9
**제50회**	**2022**	**87.8**	**222.2**	**47.1**	**79.4**

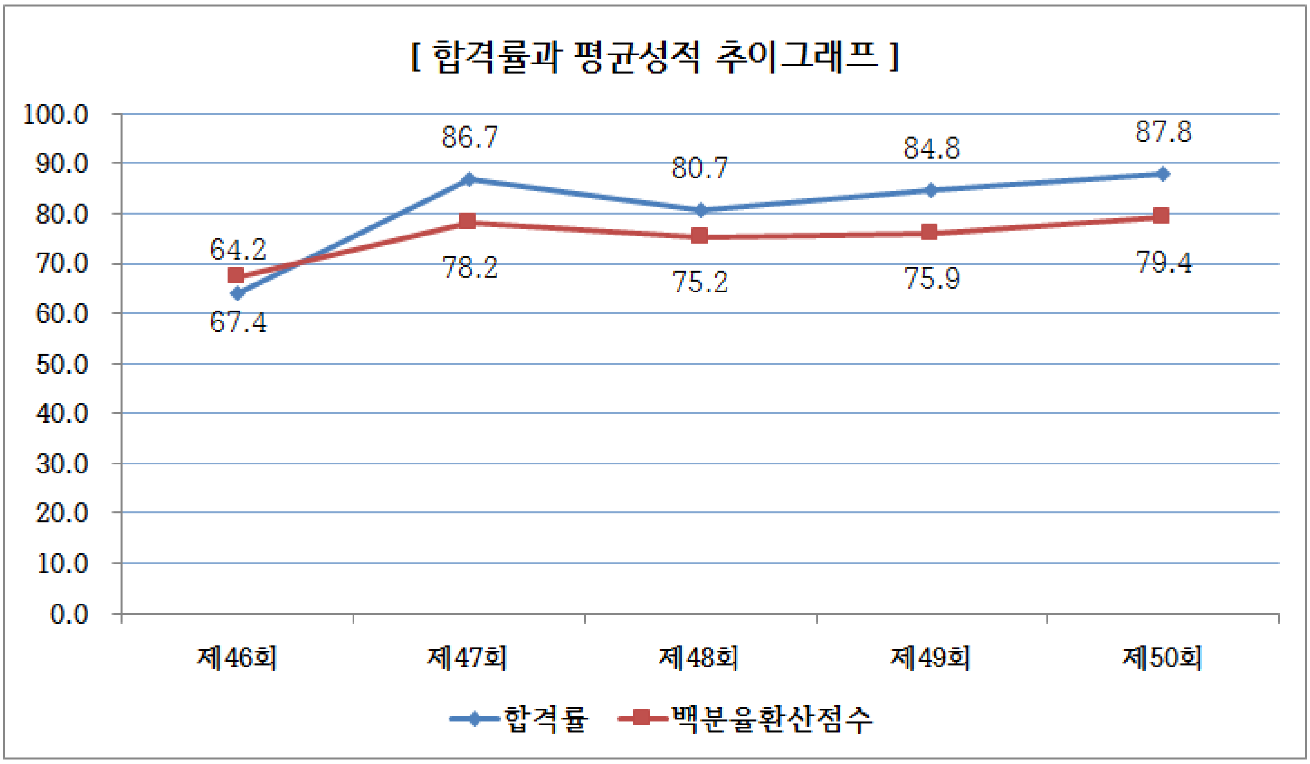

4) 전체 성적분포도

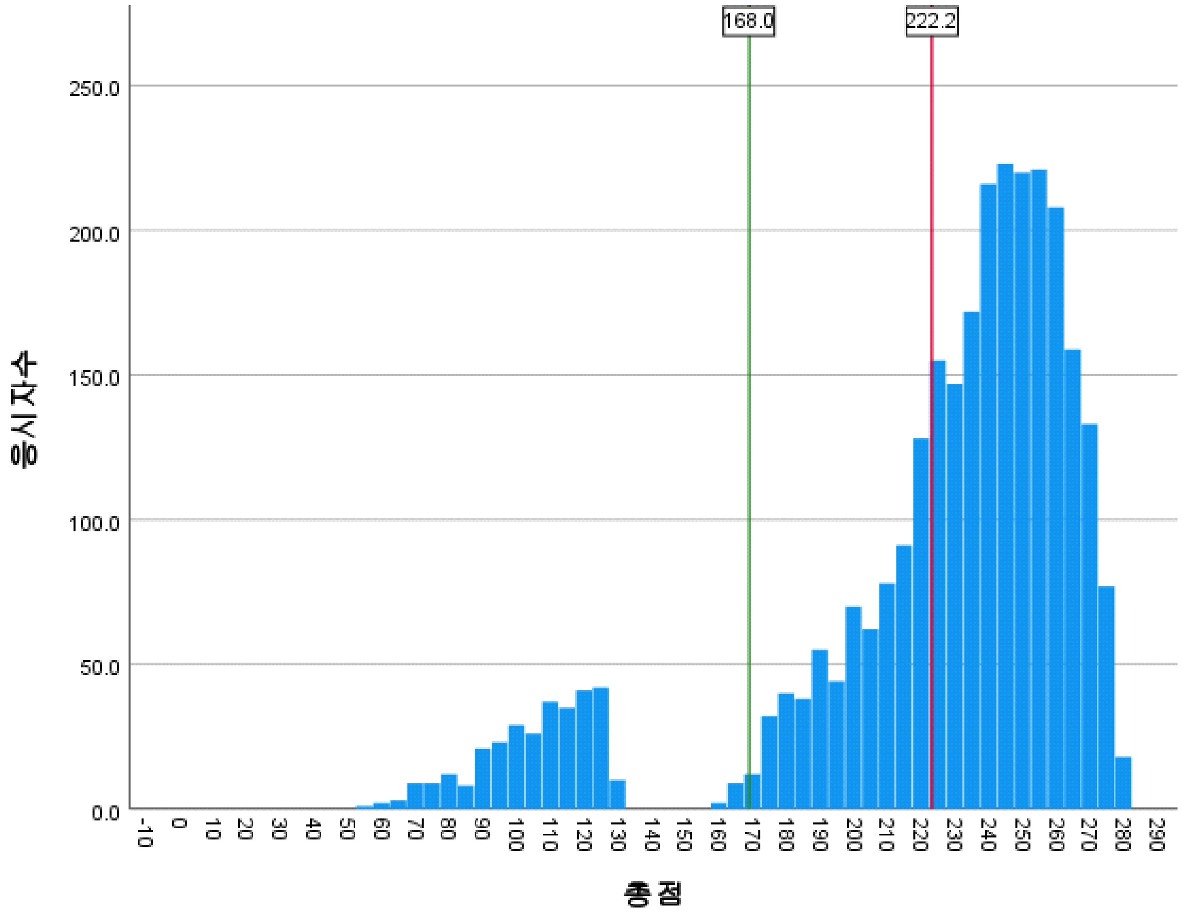

※ 필기 불합격자의 실기성적 미포함

해석

- 전년 대비 합격률은 3.0%, 백분율 환산점수는 3.5 점 증가함

Ⅱ. 문항분석 결과

1. 성적

1) 전체 성적분포도

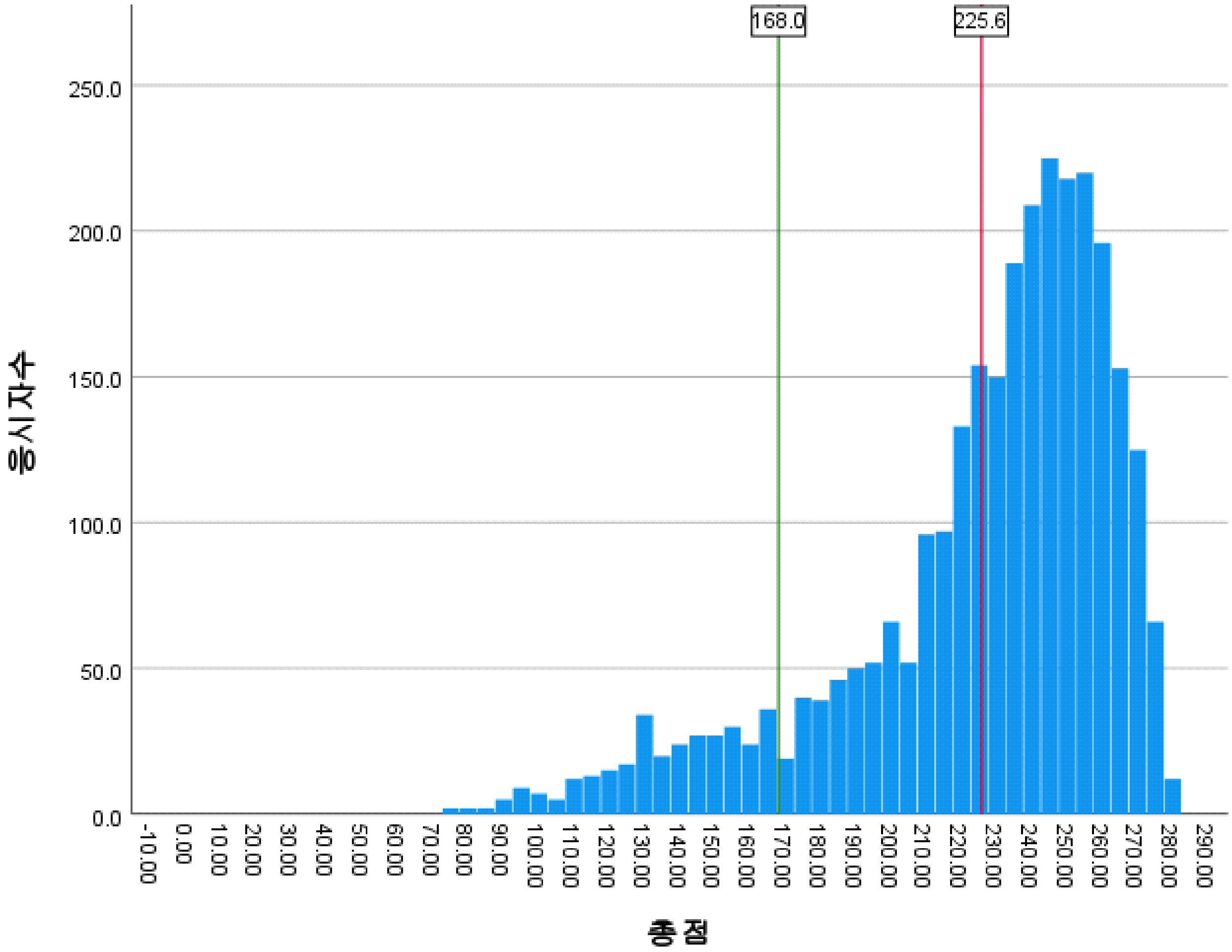

응시자	총점	합격선	평균성적	표준편차
2,918	280	168	225.6	39.3

※ 필기 불합격자의 실기성적 포함

※ 2,918명은 전체응시자(2,917명)에서 채점보류자수(1명)을 포함한 수치임

2) 과목별 성적분포도(※ 필기형 실기 포함)

가) 의료관계법규

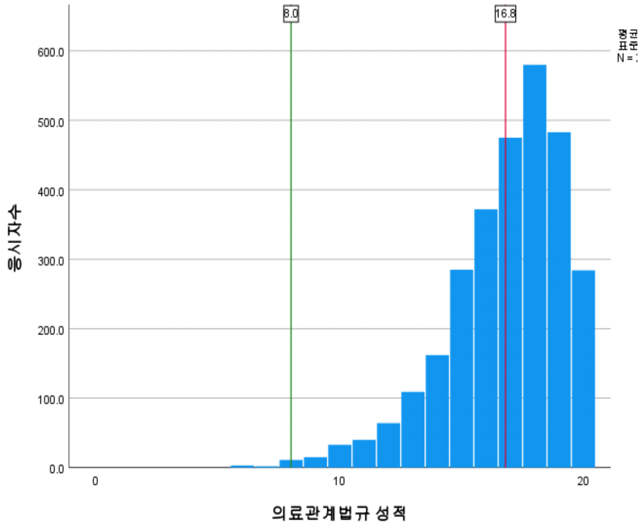

총점	과락선	평균성적	표준편차
20	8	16.8	2.4

나) 임상검사이론 I

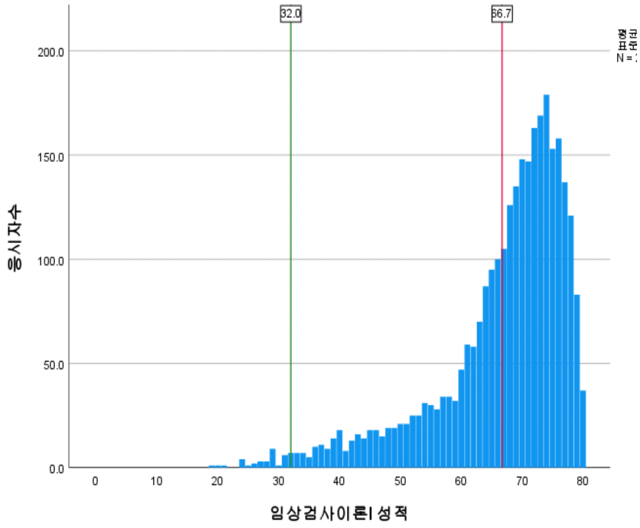

총점	과락선	평균성적	표준편차
80	32	66.7	10.8

다) 임상검사이론 II

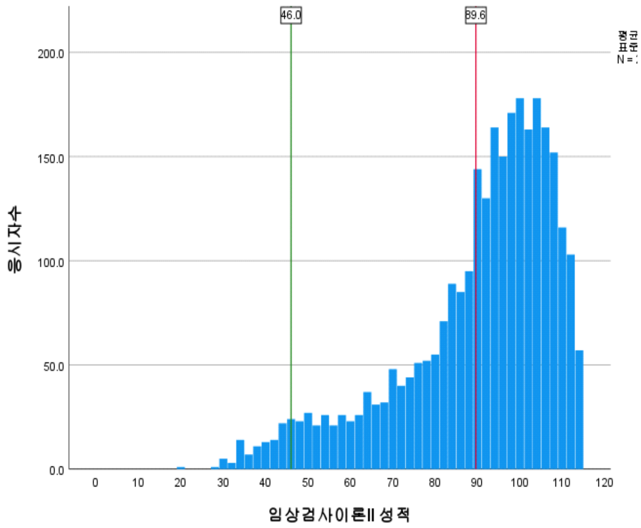

총점	과락선	평균성적	표준편차
115	46	89.6	18.4

라) 실기시험

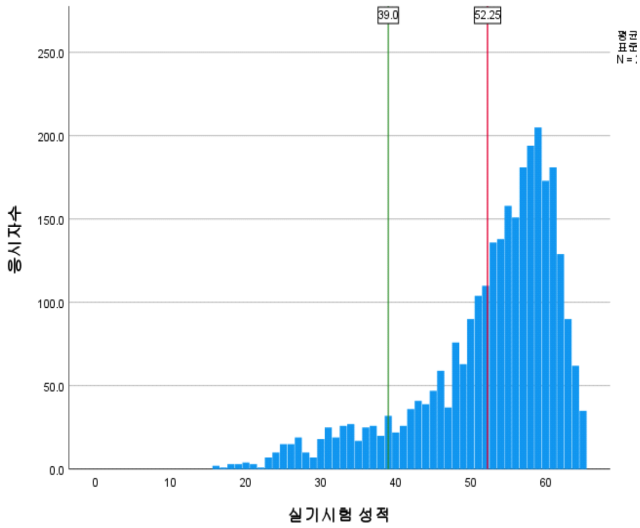

총점	과락선	평균성적	표준편차
65	39	52.5	9.4

2. 난이도와 변별도

1) 전체 난이도와 변별도

가) 전회 대비 전체 난이도와 변별도

회차	난이도		변별도1		변별도2	
	평균	표준편차	평균	표준편차	평균	표준편차
제46회	69.4	17.9	.36	.13	.34	.11
제47회	79.3	14.2	.32	.13	.37	.12
제48회	76.8	13.9	.36	.14	.39	.12
제49회	77.4	16.1	.35	.15	.39	.12
**제50회**	**80.6**	**13.2**	**.32**	**.15**	**.37**	**.12**

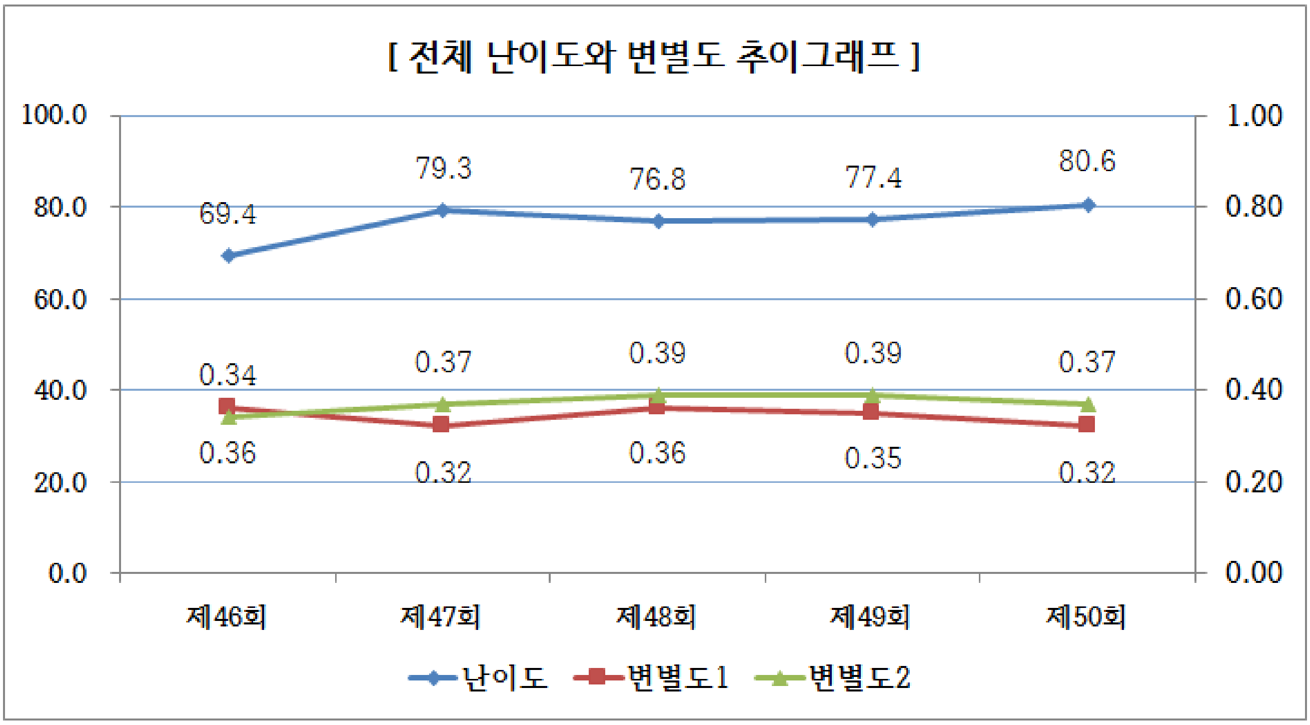

해석

- 전년 대비 난이도 지수는 3.2 증가함
- 변별도 1 지수는 .03, 변별도 2 지수는 .02 감소함

나) 전체 난이도와 변별도 분포도 및 비율분석

(1) 전체 난이도 분포도 및 비율분석

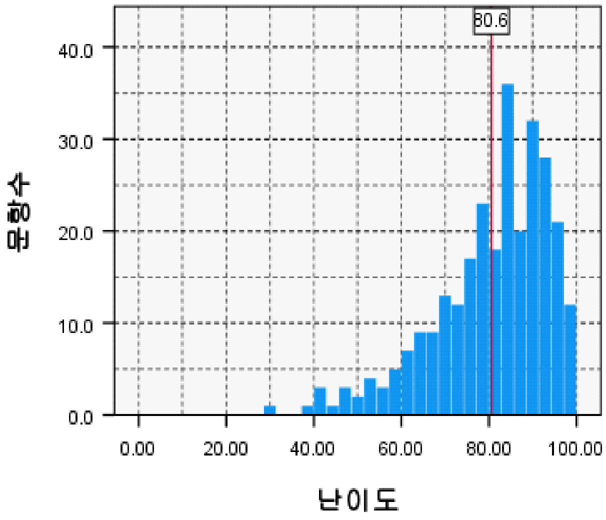

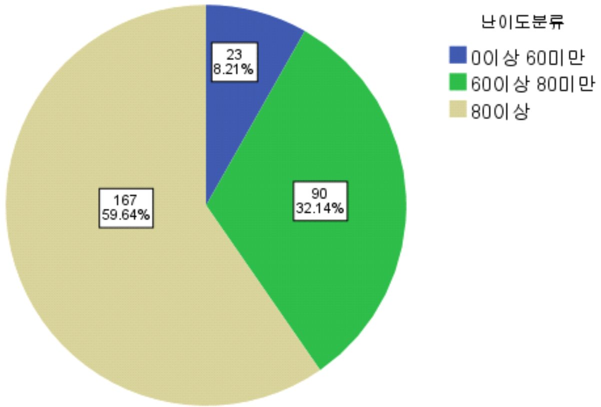

총점	난이도	표준편차
280	80.6	13.2

난이도	문항수	비율(%)
0~60미만	23	8.2
60~80미만	90	32.1
80~100	167	59.6
전체	280	100.0

(2) 전체 변별도1 분포도 및 비율분석

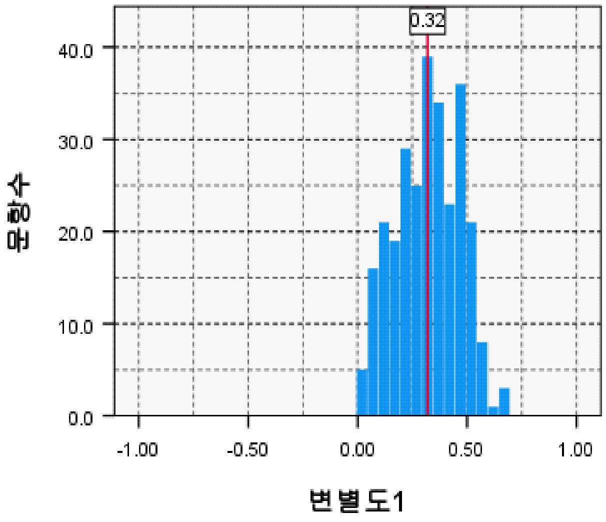

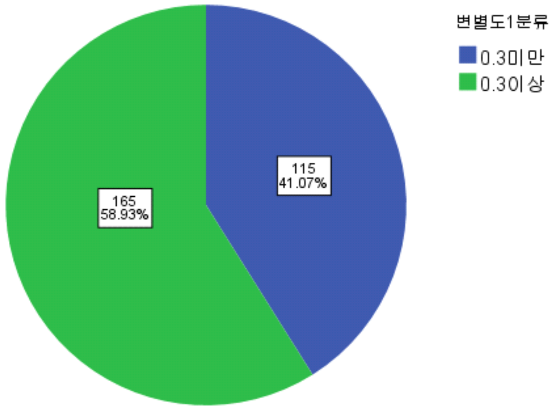

총점	변별도1	표준편차
280	.32	.15

변별도1	문항수	비율(%)
0.3미만	115	41.1
0.3이상	165	58.9
전체	280	100.0

(3) 전체 변별도2 분포도 및 비율분석

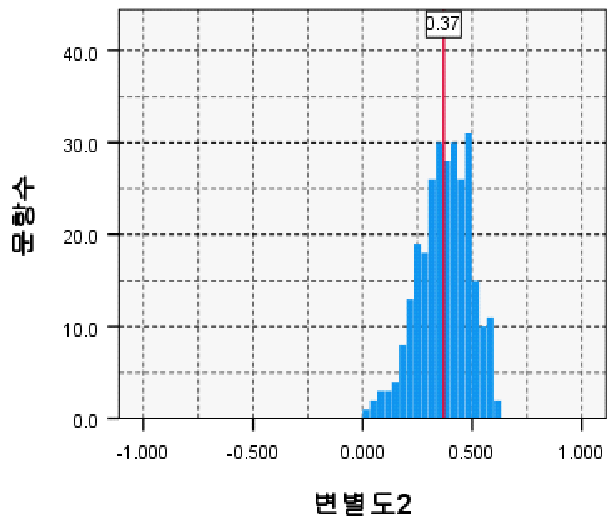

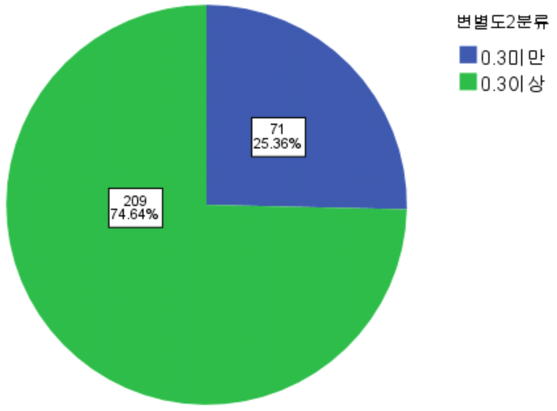

총점	변별도2	표준편차	변별도2	문항수	비율(%)
280	.37	.12	0.3미만	71	25.4
			0.3이상	209	74.6
			전체	280	100.0

해석

- 난이도 지수가 80 에서 100 사이인 문항이 전체 280 문항 중 167 문항으로 가장 많았으며, 차례로 60 이상 80 미만인 문항이 90 문항, 0 에서 60 사이인 문항이 23 문항인 것으로 나타남
- 변별도 1 지수를 기준으로 분류하였을 때, 0.3 미만인 문항이 115 문항으로 0.3 이상인 문항이 165 문항인 것에 비해 더 적게 나타남
- 변별도 2 지수를 기준으로 분류하였을 때, 0.3 미만인 문항이 71 문항으로 0.3 이상인 문항 이 209 문항인 것에 비해 더 적게 나타남

2) 과목별 난이도와 변별도

가) 전회 대비 과목별 난이도와 변별도

(1) 전회 대비 의료관계법규 난이도와 변별도

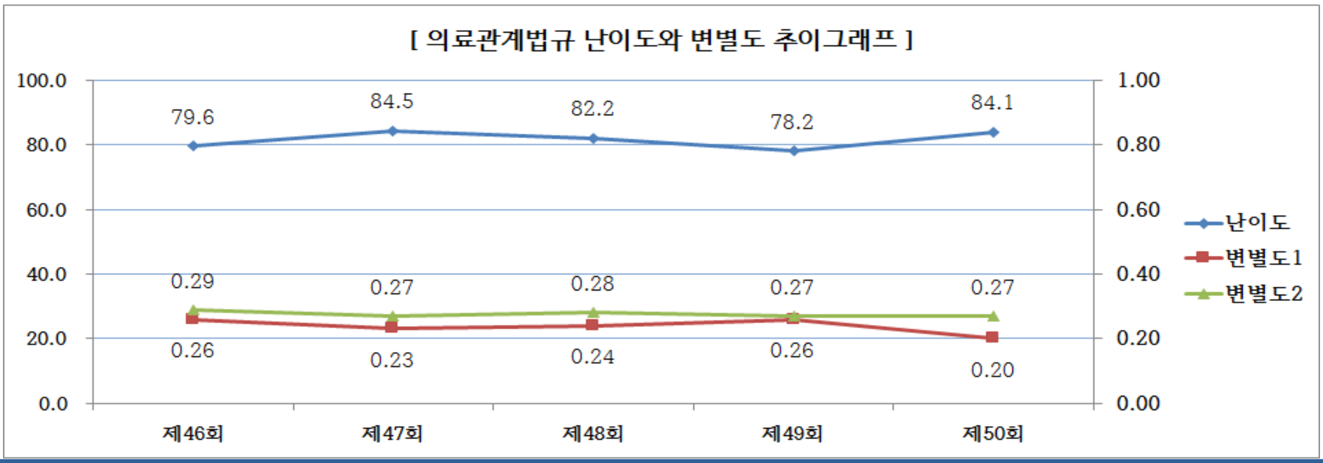

회차	난이도		변별도1		변별도2	
	평균	표준편차	평균	표준편차	평균	표준편차
제46회	79.6	17.4	.26	.13	.29	.10
제47회	84.5	11.8	.23	.14	.27	.10
제48회	82.2	11.9	.24	.14	.28	.11
제49회	78.2	17.4	.26	.16	.27	.11
**제50회**	**84.1**	**14.1**	**.20**	**.12**	**.27**	**.09**

(2) 전회 대비 임상검사이론I 난이도와 변별도

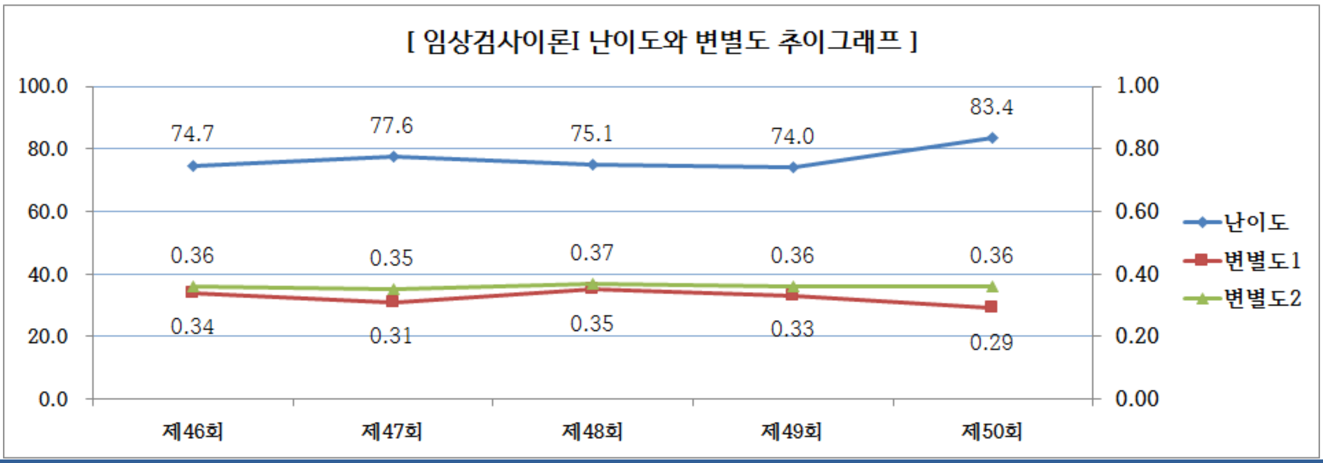

회차	난이도		변별도1		변별도2	
	평균	표준편차	평균	표준편차	평균	표준편차
제46회	74.7	15.9	.34	.11	.36	.09
제47회	77.6	15.5	.31	.13	.35	.12
제48회	75.1	14.7	.35	.13	.37	.13
제49회	74.0	18.9	.33	.13	.36	.13
**제50회**	**83.4**	**11.4**	**.29**	**.13**	**.36**	**.11**

(3) 전회 대비 임상검사이론II 난이도와 변별도

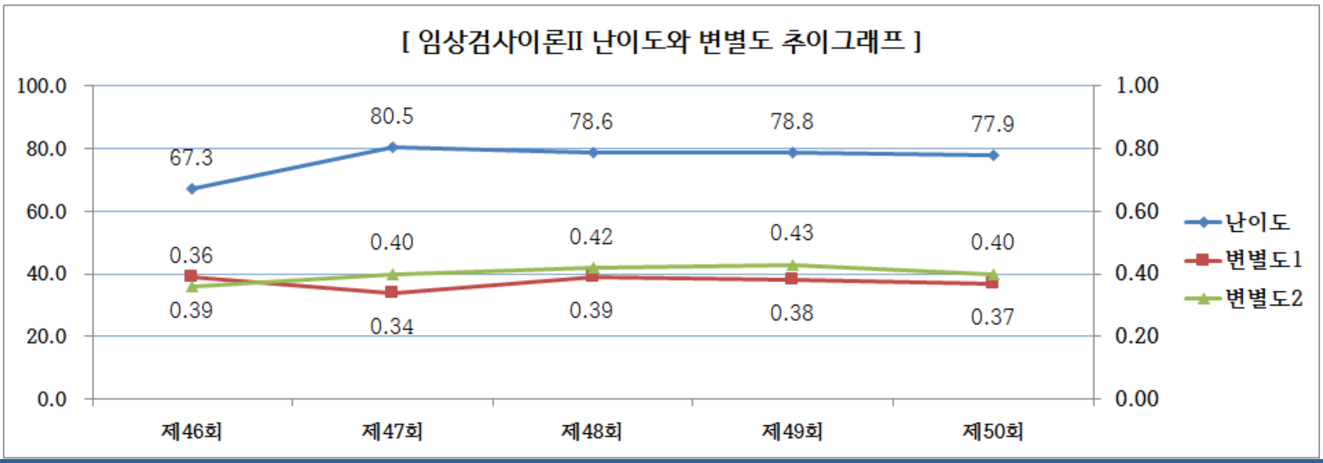

회차	난이도		변별도1		변별도2	
	평균	표준편차	평균	표준편차	평균	표준편차
제46회	67.3	16.5	.39	.12	.36	.10
제47회	80.5	12.2	.34	.13	.40	.11
제48회	78.6	11.1	.39	.13	.42	.10
제49회	78.8	13.5	.38	.15	.43	.10
**제50회**	**77.9**	**13.3**	**.37**	**.14**	**.40**	**.11**

(4) 전회 대비 실기시험 난이도와 변별도

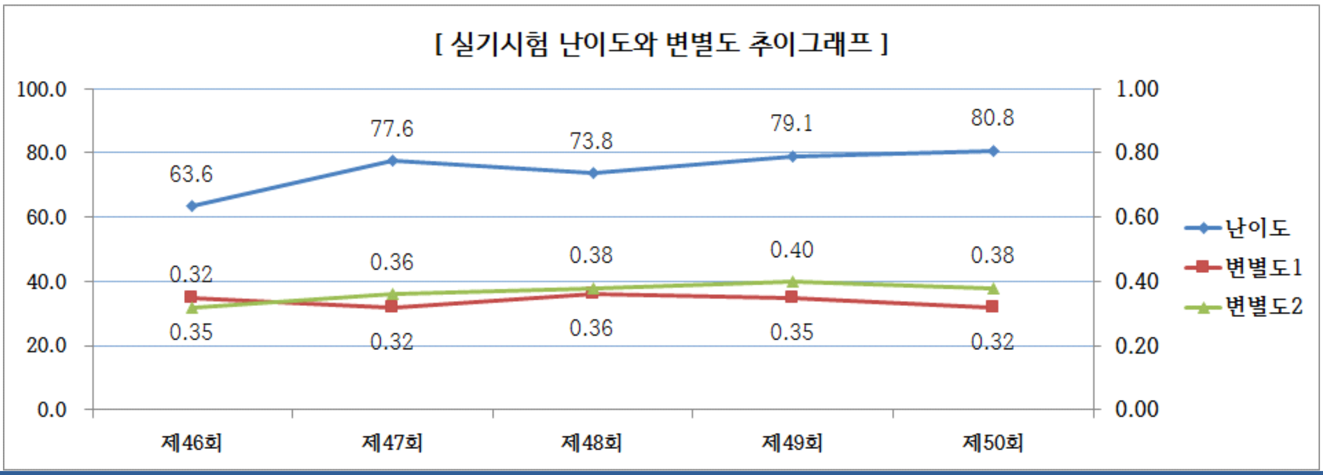

회차	난이도		변별도1		변별도2	
	평균	표준편차	평균	표준편차	평균	표준편차
제46회	63.6	19.9	.35	.16	.32	.13
제47회	77.6	15.7	.32	.13	.36	.12
제48회	73.8	16.7	.36	.13	.38	.13
제49회	79.1	16.1	.35	.14	.40	.12
**제50회**	**80.8**	**13.6**	**.32**	**.14**	**.38**	**.13**

해석

- 전회대비 의료관계법규 과목의 난이도 지수는 5.9 증가하였으며, 변별도 1 지수는 .06 감소, 변별도 2 지수 동일함
- 임상검사이론Ⅰ 과목의 난이도 지수는 9.4 증가하였으며, 변별도 1 지수는 .04 감소, 변별도 2 지수는 동일함
- 임상검사이론Ⅱ 과목의 난이도 지수는 .9 감소하였으며, 변별도 1 지수와 변별도 2 지수는 각각 .01, .03 감소함
- 실기시험 과목의 난이도 지수는 1.7 증가하였으며, 변별도 1 지수와 변별도 2 지수는 각각 .03, .02 감소함

나) 과목별 난이도와 변별도 분포도 및 비율분석

(1) 의료관계법규 난이도와 변별도 분포도 및 비율분석

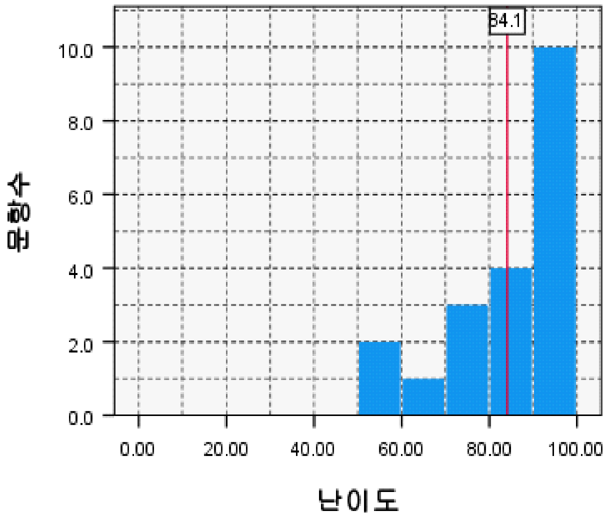

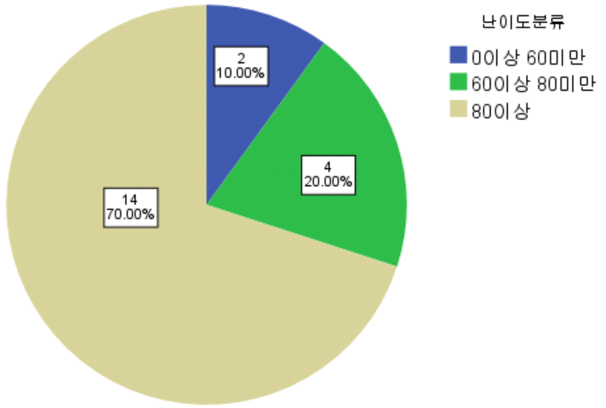

총점	난이도	표준편차
20	84.1	14.5

난이도	문항수	비율(%)
0~60미만	2	10.0
60~80미만	4	20.0
80~100	14	70.0
전체	20	100.0

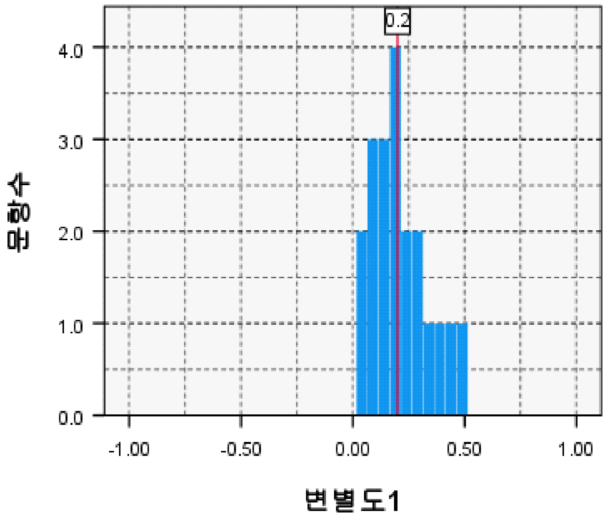

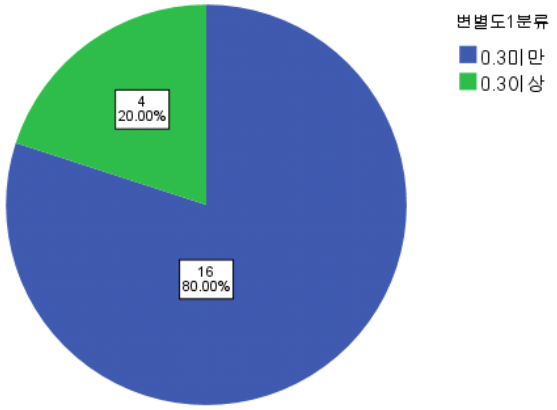

총점	변별도1	표준편차
20	.20	.13

변별도1	문항수	비율(%)
0.3미만	16	80.0
0.3이상	4	20.0
전체	20	100.0

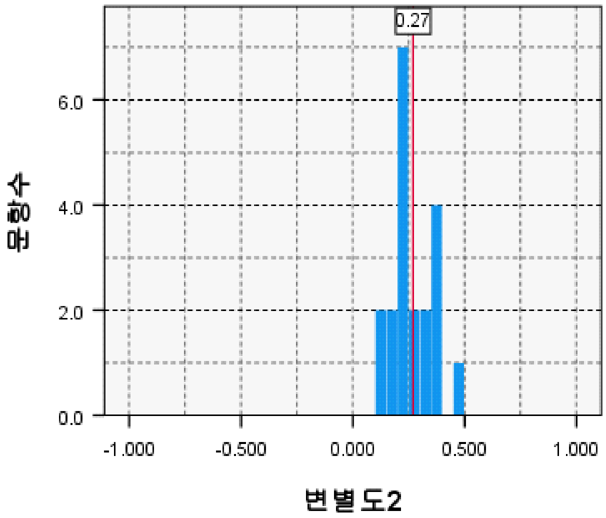

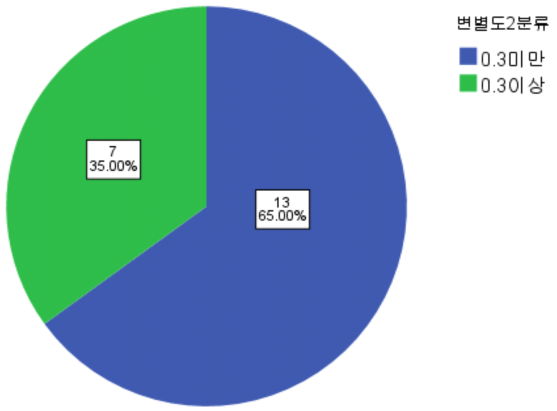

총점	변별도2	표준편차
20	.27	.09

변별도2	문항수	비율(%)
0.3미만	13	65.0
0.3이상	7	35.0
전체	20	100.0

해석

- 의료관계법규 과목에서 난이도 지수가 80 에서 100 사이인 문항이 전체 20 문항 중 14 문항으로 가장 많았으며, 차례로 60 이상 80 미만인 문항이 4 문항, 0 에서 60 사이인 문항이 2 문항으로 나타남
- 변별도 1 지수를 기준으로 분류하였을 때, 0.3 미만인 문항이 16 문항으로 0.3 이상인 문항이 4 문항인 것에 비해 더 많게 나타남
- 변별도 2 지수를 기준으로 분류하였을 때, 0.3 미만인 문항이 13 문항으로 0.3 이상인 문항이 7 문항인 것에 비해 더 많게 나타남

(2) 임상검사이론I 난이도와 변별도 분포도 및 비율분석

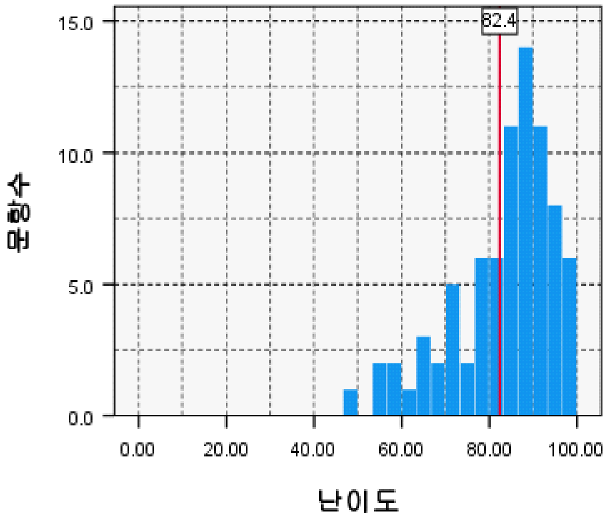

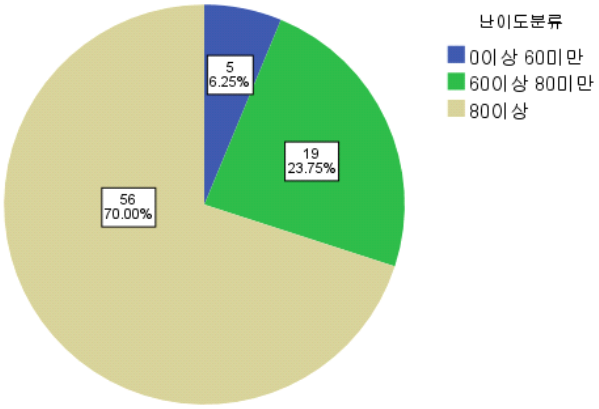

총점	난이도	표준편차	난이도	문항수	비율(%)
80	83.4	11.4	0~60미만	5	6.3
			60~80미만	19	23.8
			80~100	56	70.0
			전체	80	100.0

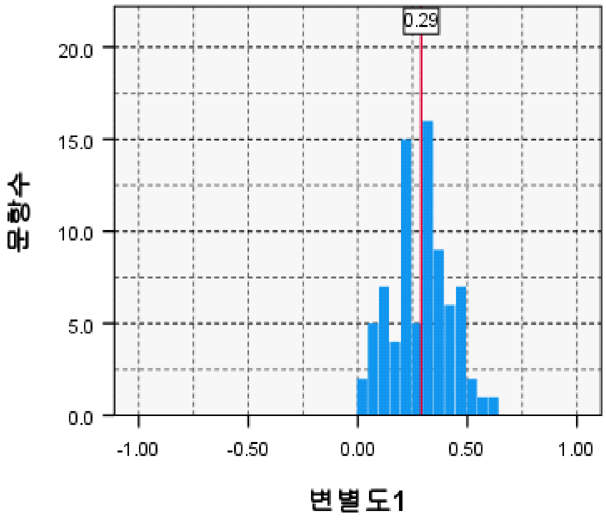

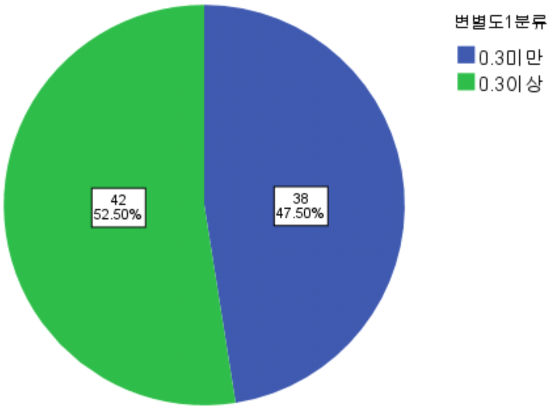

총점	변별도1	표준편차	변별도1	문항수	비율(%)
80	.29	.13	0.3미만	38	47.5
			0.3이상	42	52.5
			전체	80	100.0

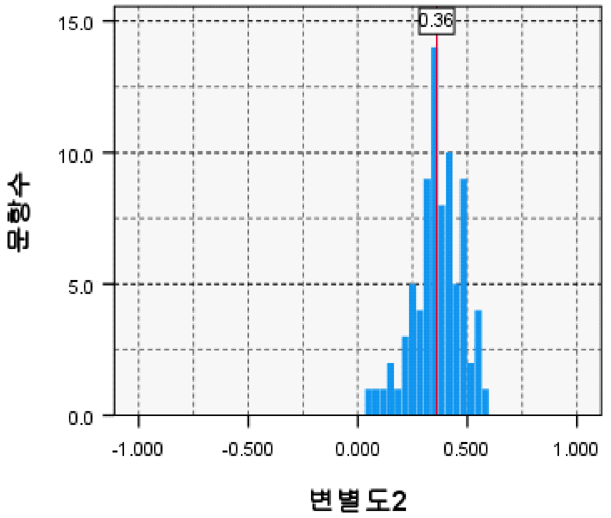

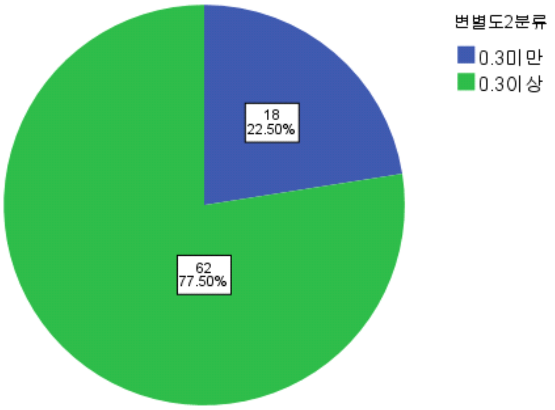

총점	변별도2	표준편차	변별도2	문항수	비율(%)
80	.36	.10	0.3미만	18	22.5
			0.3이상	62	77.5
			전체	80	100.0

해석

- 임상검사이론Ⅰ 과목에서 난이도 지수가 80에서 100 사이인 문항이 전체 80문항 중 56문항으로 가장 많았으며, 차례로 60 이상 80 미만인 문항이 19문항, 0에서 60 사이인 문항이 5 문항으로 나타남
- 변별도1 지수를 기준으로 분류하였을 때, 0.3미만인 문항이 38문항으로 0.3 이상인 문항이 42문항인 것에 비해 더 적게 나타남
- 변별도2 지수를 기준으로 분류하였을 때, 0.3미만인 문항이 18문항으로 0.3 이상인 문항이 62문항인 것에 비해 더 적게 나타남

(3) 임상검사이론II 난이도와 변별도 분포도 및 비율분석

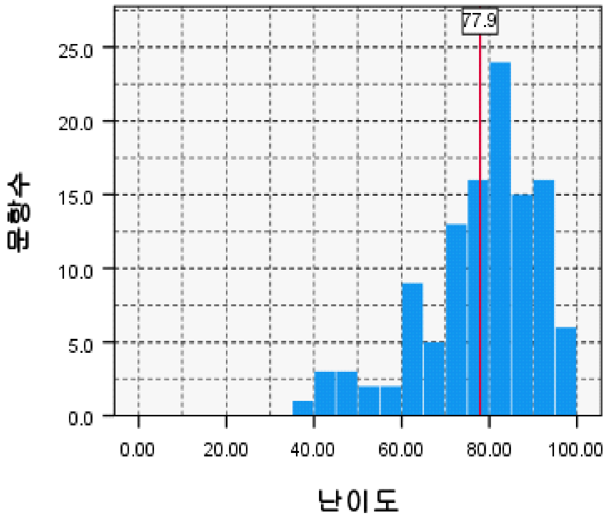

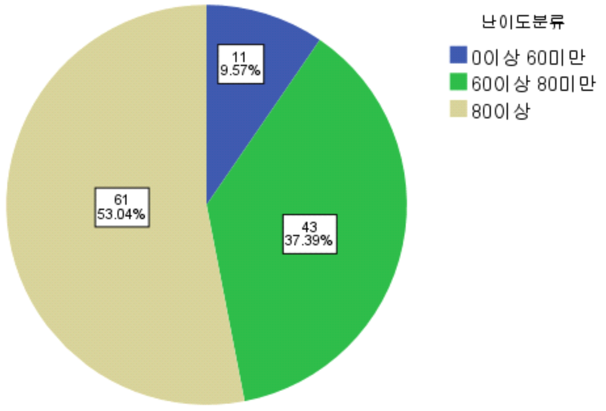

총점	난이도	표준편차
115	77.9	13.4

난이도	문항수	비율(%)
0~60미만	11	9.6
60~80미만	43	37.4
80~100	61	53.0
전체	115	100.0

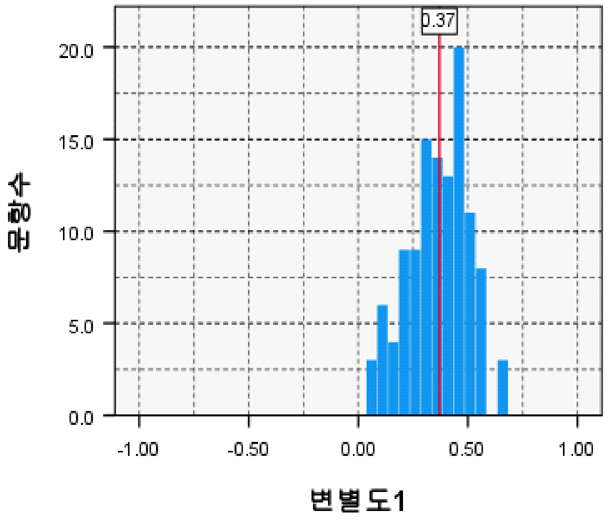

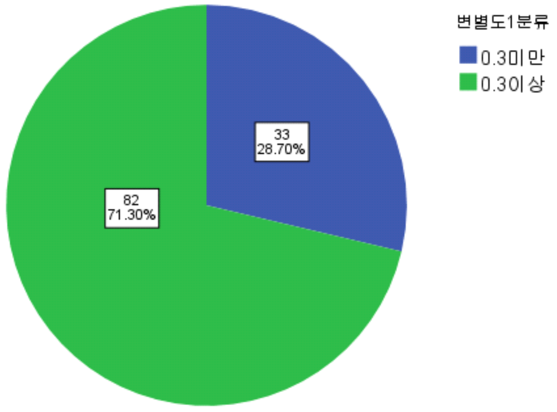

총점	변별도1	표준편차
115	.37	.14

변별도1	문항수	비율(%)
0.3미만	33	28.7
0.3이상	82	71.3
전체	115	100.0

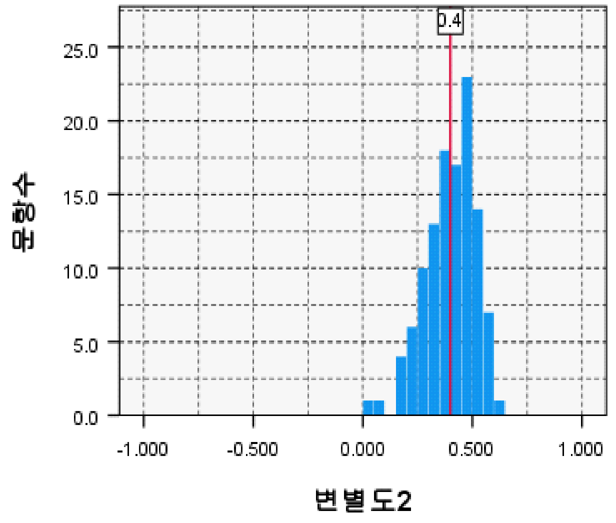

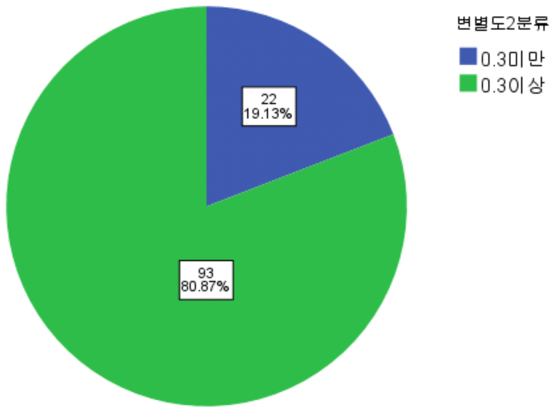

총점	변별도2	표준편차
115	.40	.11

변별도2	문항수	비율(%)
0.3미만	22	19.1
0.3이상	93	80.9
전체	115	100.0

해석

- 임상검사이론 II 과목에서 난이도 지수가 80 에서 100 사이인 문항이 전체 115 문항 중 61 문항으로 가장 많았으며, 차례로 60 이상 80 미만인 문항이 43 문항, 0 에서 60 사이인 문항이 11 문항으로 나타남
- 변별도 1 지수를 기준으로 분류하였을 때, 0.3 미만인 문항이 33 문항으로 0.3 이상인 문항이 82 문항인 것에 비해 더 적게 나타남
- 변별도 2 지수를 기준으로 분류하였을 때, 0.3 미만인 문항이 22 문항으로 0.3 이상인 문항이 93 문항인 것에 비해 더 적게 나타남

(4) 실기시험 난이도와 변별도 분포도 및 비율분석

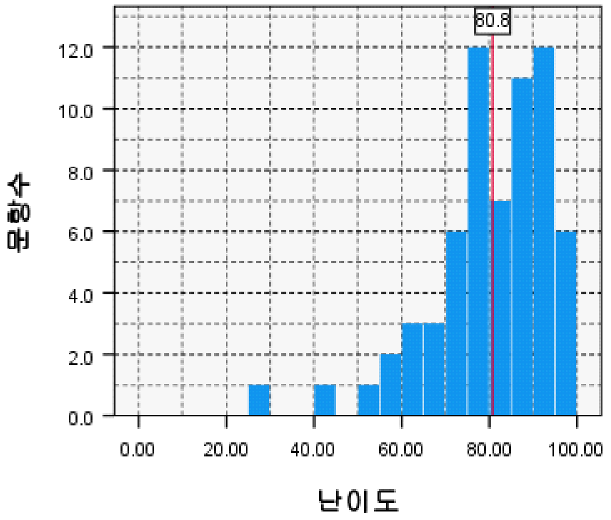

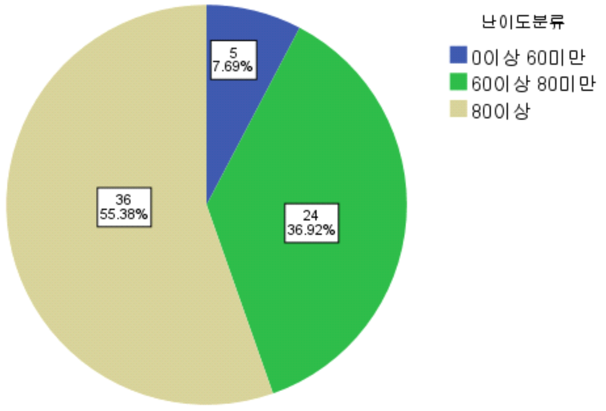

총점	난이도	표준편차
65	80.8	13.8

난이도	문항수	비율(%)
0~60미만	5	7.7
60~80미만	24	36.9
80~100	36	55.4
전체	65	100.0

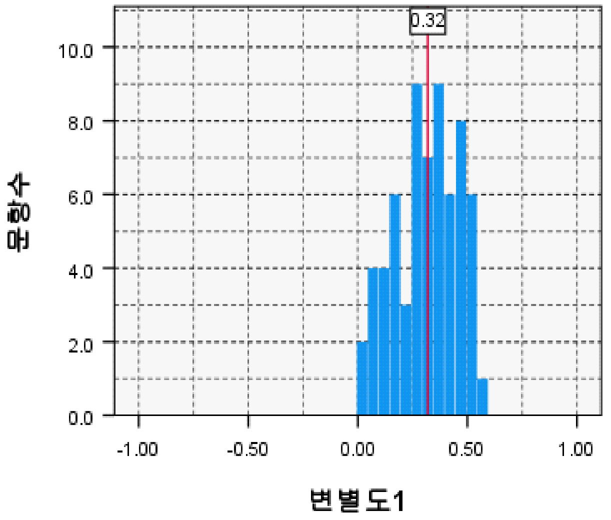

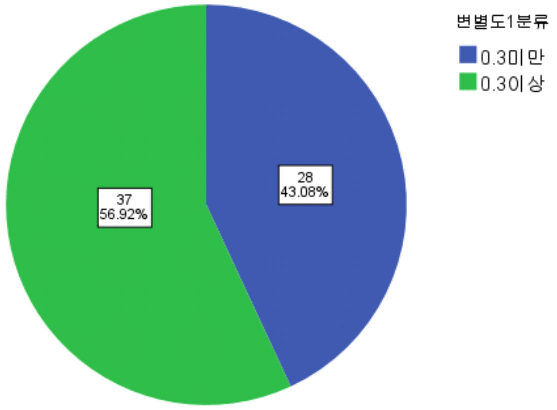

총점	변별도1	표준편차
65	.32	.15

변별도1	문항수	비율(%)
0~60미만	28	43.1
60~80미만	37	56.9
80~100	65	100.0

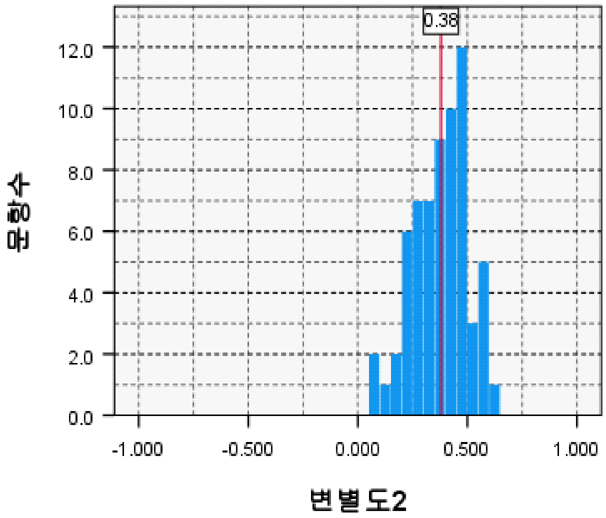

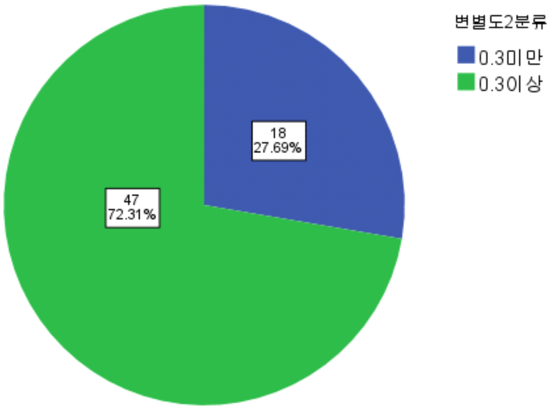

총점	변별도2	표준편차	변별도2	문항수	비율%)
65	.38	.13	0~60미만	18	27.7
			60~80미만	47	72.3
			80~100	65	100.0

해석

- 실기시험 과목에서 난이도 지수가 80 에서 100 사이인 문항이 전체 65 문항 중 36 문항으로 가장 많았으며, 차례로 60 이상 80 미만인 문항이 24 문항, 0 에서 60 사이인 문항이 5 문항으로 나타남
- 변별도 1 지수를 기준으로 분류하였을 때, 0.3 미만인 문항이 28 문항으로 0.3 이상인 문항이 37 문항인 것에 비해 더 적게 나타남
- 변별도 2 지수를 기준으로 분류하였을 때, 0.3 미만인 문항이 18 문항으로 0.3 이상인 문항이 47 문항인 것에 비해 더 적게 나타남

3) 지식수준별 난이도와 변별도

가) 전회 대비 지식수준별 난이도와 변별도

(1) 전회 대비 암기형 난이도와 변별도

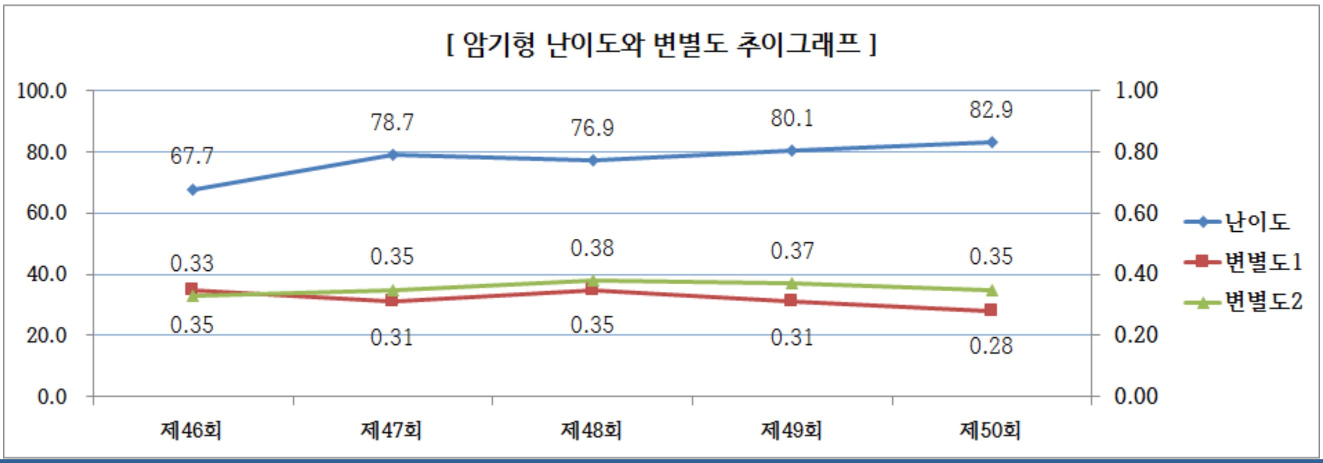

회차	난이도		변별도1		변별도2	
	평균	표준편차	평균	표준편차	평균	표준편차
제46회	67.7	19.3	.35	.13	.33	.11
제47회	78.7	14.1	.31	.14	.35	.12
제48회	76.9	14.5	.35	.13	.38	.13
제49회	80.1	15.1	.31	.15	.37	.13
**제50회**	**82.9**	**13.7**	**.28**	**.14**	**.35**	**.12**

(2) 전회 대비 해석형 난이도와 변별도

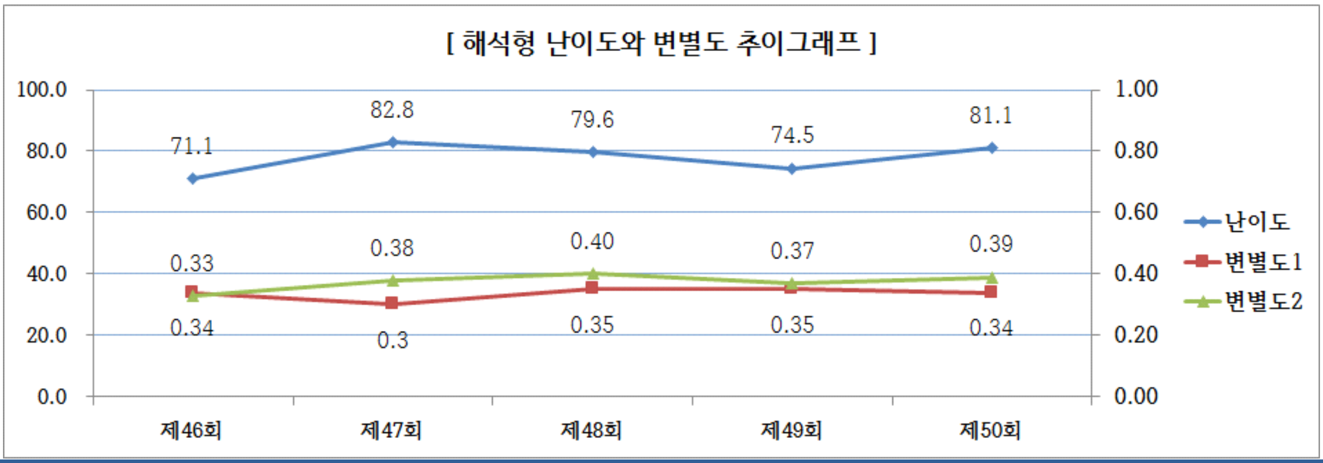

회차	난이도		변별도1		변별도2	
	평균	표준편차	평균	표준편차	평균	표준편차
제46회	71.1	16.7	.34	.13	.33	.11
제47회	82.8	12.1	.30	.13	.38	.11
제48회	79.6	12.1	.35	.13	.40	.11
제49회	74.5	18.3	.35	.15	.37	.13
**제50회**	**81.1**	**12.3**	**.34**	**.14**	**.39**	**.11**

(3) 전회 대비 해결형 난이도와 변별도

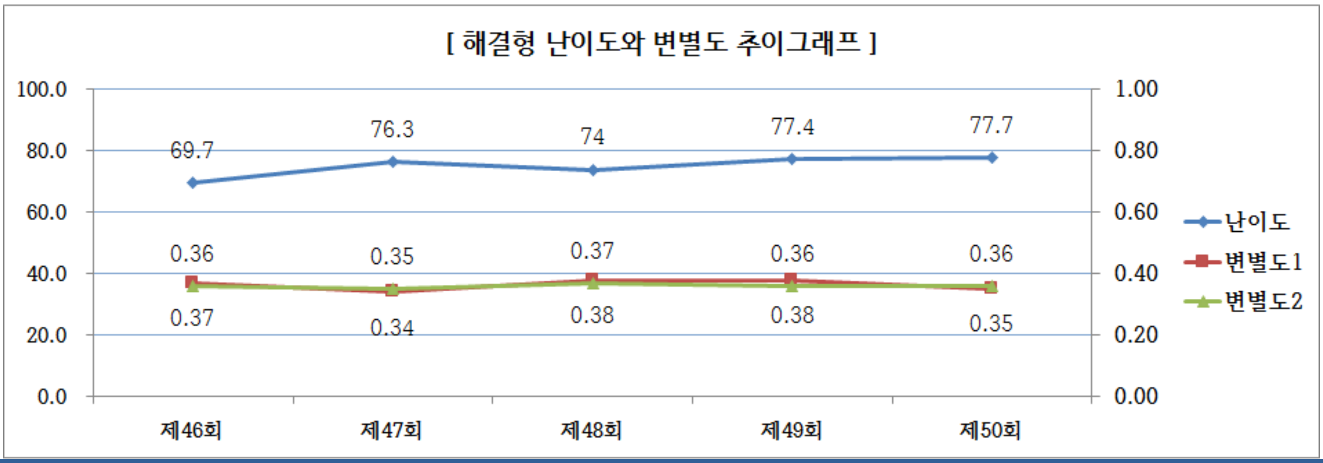

회차	난이도		변별도1		변별도2	
	평균	표준편차	평균	표준편차	평균	표준편차
제46회	69.7	17.6	.37	.14	.36	.11
제47회	76.3	15.4	.34	.14	.37	.12
제48회	74.0	14.3	.38	.14	.38	.13
제49회	77.4	14.7	.38	.13	.42	.10
**제50회**	**77.7**	**12.9**	**.35**	**.15**	**.39**	**.12**

해석

- 전회 대비 암기형 문항의 난이도 지수는 2.8 증가했으며, 변별도 1 지수와 변별도 2 지수는 각각 .03, .02 감소함
- 해석형 문항의 난이도 지수는 6.6 증가했으며, 변별도 1 지수는 .01 감소, 변별도 2 지수는 .02 증가함
- 해결형 문항의 난이도 지수는 0.3 증가했으며, 변별도 1 지수와 변별도 2 지수는 각각 .03, .03 감소함

나) 지식수준별 난이도와 변별도 분포도 및 비율분석

(1) 암기형 난이도와 변별도 분포도 및 비율분석

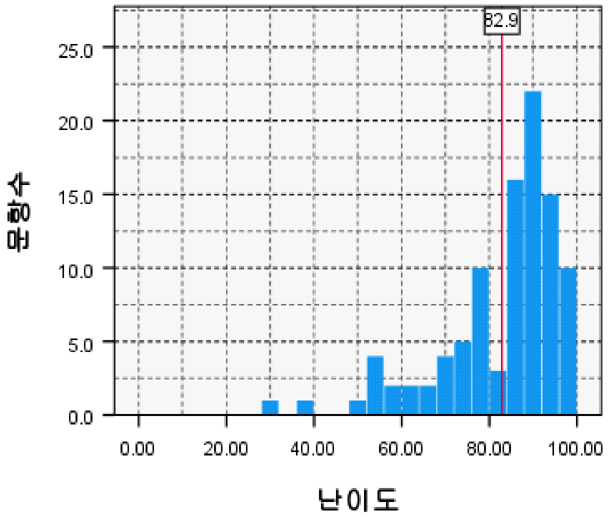

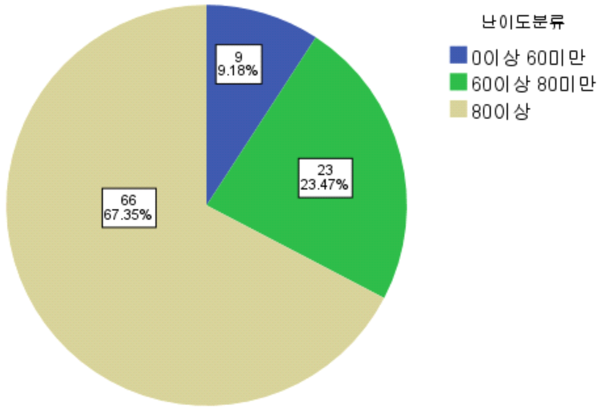

총점	난이도	표준편차	난이도	문항수	비율(%)
98	82.9	13.8	0~60미만	9	9.2
			60~80미만	23	23.5
			80~100	66	67.3
			전체	98	100.0

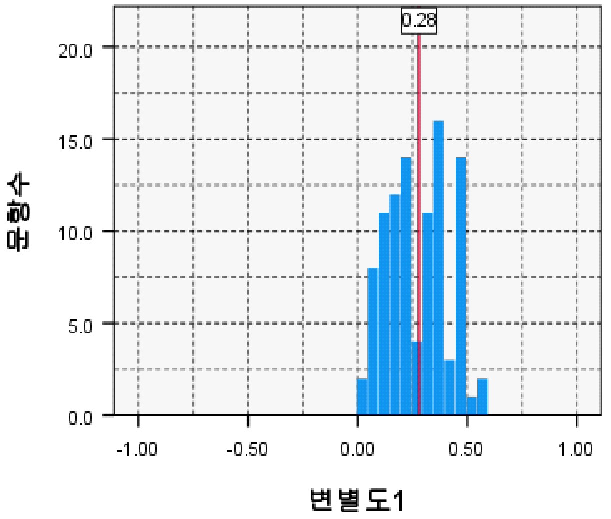

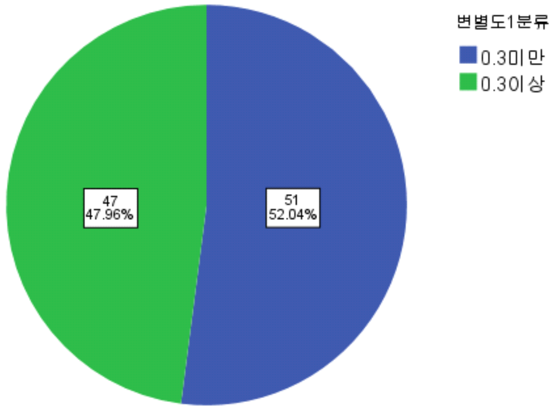

총점	변별도1	표준편차	변별도1	문항수	비율(%)
98	.28	.14	0.3미만	51	52.0
			0.3이상	47	48.0
			전체	98	100.0

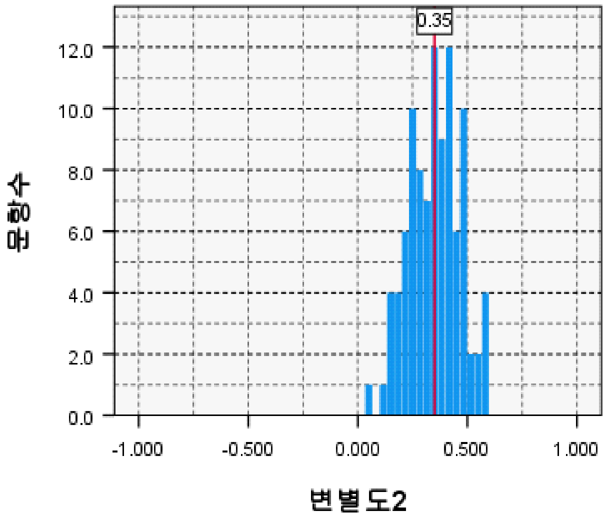

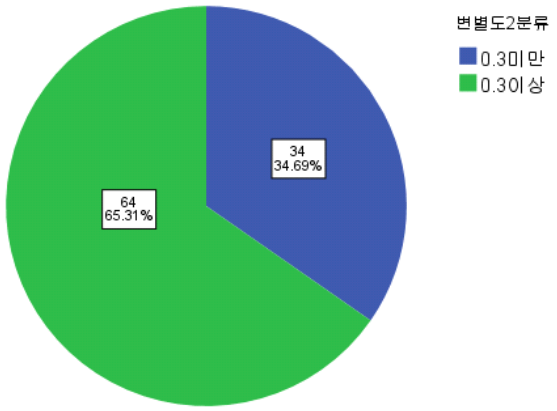

총점	변별도2	표준편차	변별도2	문항수	비율(%)
98	.35	.12	0.3미만	34	34.7
			0.3이상	54	65.3
			전체	98	100.0

해석

- 암기형 문항에서 난이도 지수가 80에서 100 사이인 문항이 전체 98문항 중 66문항으로 가장 많았으며, 60이상 80미만인 문항이 23문항, 0에서 60미만인 문항이 9문항인 것으로 나타남
- 변별도1 지수를 기준으로 분류하였을 때, 0.3미만인 문항이 51문항으로 0.3 이상인 문항이 47문항인 것에 비해 많이 나타남
- 변별도2 지수를 기준으로 분류하였을 때, 0.3미만인 문항이 34문항으로 0.3 이상인 문항이 54문항인 것에 비해 더 적게 나타남

(2) 해석형 난이도와 변별도 분포도 및 비율분석

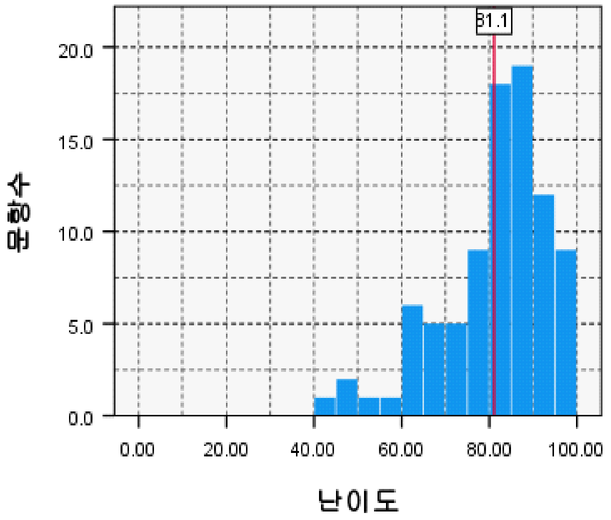

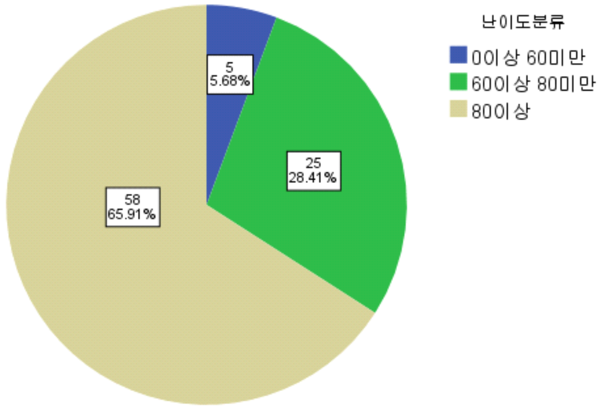

총점	난이도	표준편차
88	81.1	12.3

난이도	문항수	비율(%)
0~60미만	5	5.7
60~80미만	25	28.4
80~100	58	65.9
전체	88	100.0

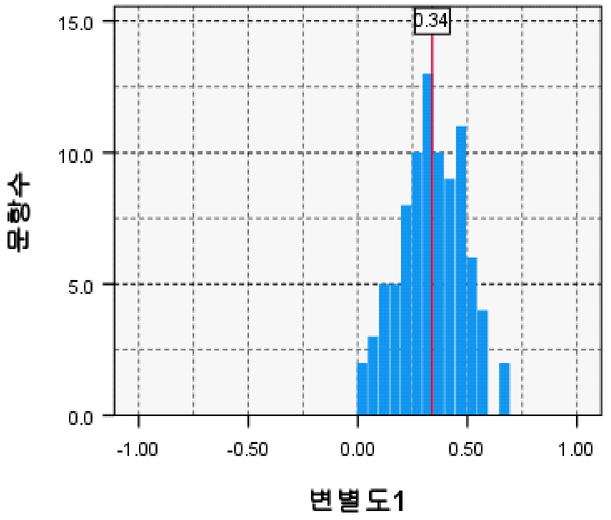

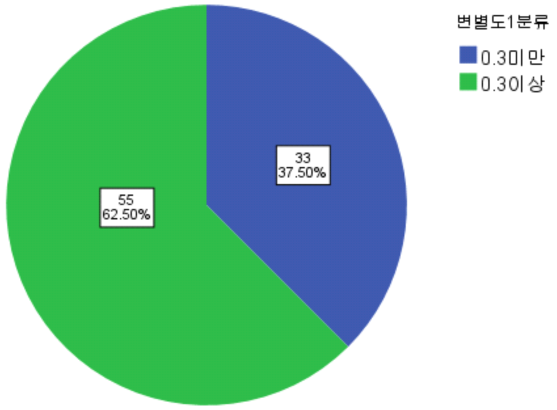

총점	변별도2	표준편차
88	.34	.15

변별도2	문항수	비율(%)
0.3미만	33	37.5
0.3이상	55	62.5
전체	88	100.0

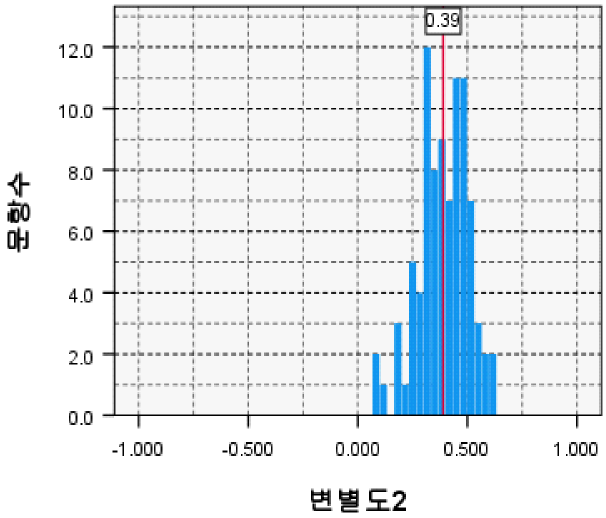

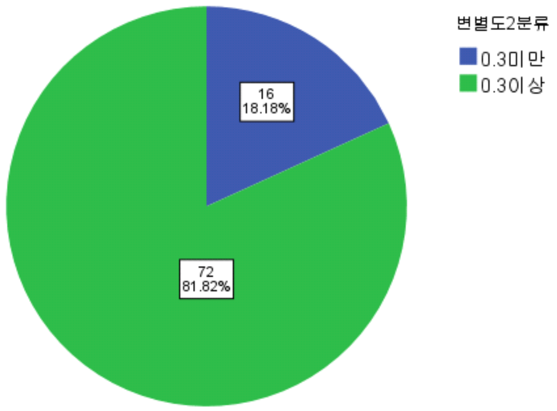

총점	변별도2	표준편차
88	.39	.12

변별도2	문항수	비율(%)
0.3미만	16	18.2
0.3이상	72	81.8
전체	88	100.0

해석

- 해석형 문항에서 난이도 지수가 80 에서 100 사이인 문항이 전체 88 문항 중 58 문항으로 가장 많았으며, 차례로 60 이상 80 미만인 문항이 25 문항, 0 에서 60 미만인 문항이 5 문항인 것으로 나타남
- 변별도 1 지수를 기준으로 분류하였을 때, 0.3 미만인 문항이 33 문항으로 0.3 이상인 문항이 55 문항인 것에 비해 더 적게 나타남
- 변별도 2 지수를 기준으로 분류하였을 때, 0.3 미만인 문항이 16 문항으로 0.3 이상인 문항이 72 문항인 것에 비해 더 적게 나타남

(3) 해결형 난이도와 변별도 분포도 및 비율분석

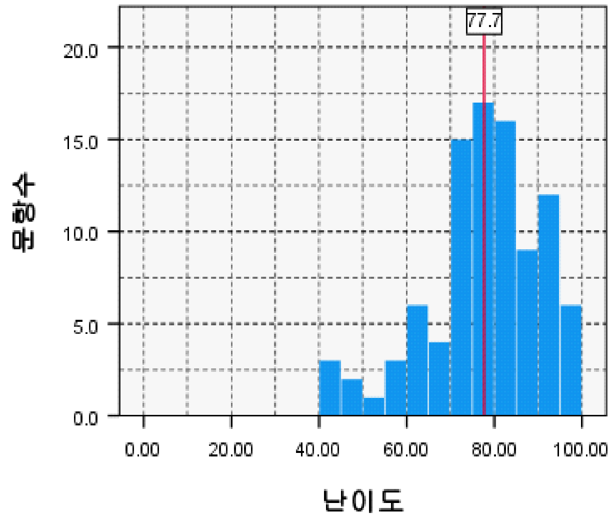

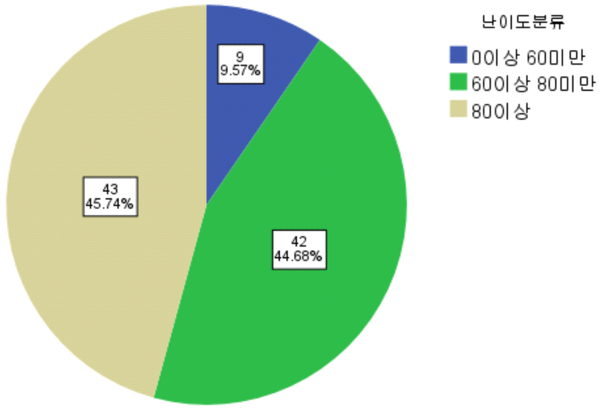

총점	난이도	표준편차
94	77.7	13.0

난이도	문항수	비율(%)
0~60미만	9	9.6
60~80미만	42	44.7
80~100	43	45.7
전체	94	100.0

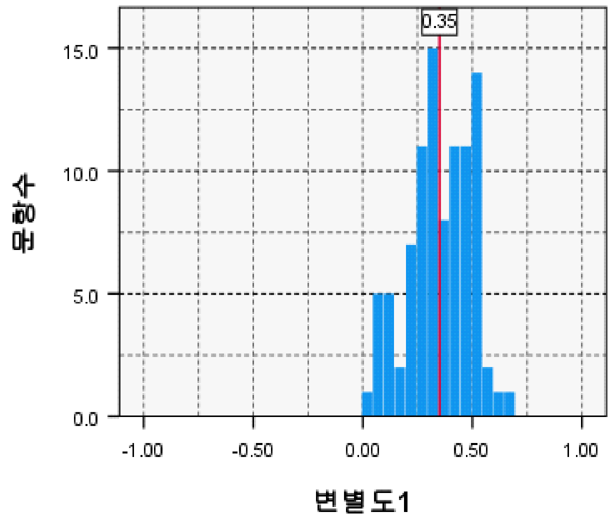

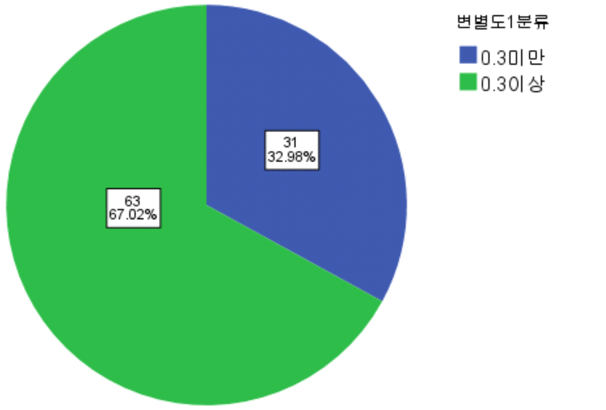

총점	변별도2	표준편차
94	.35	.15

변별도2	문항수	비율(%)
0.3미만	31	33.0
0.3이상	63	67.0
전체	94	100.0

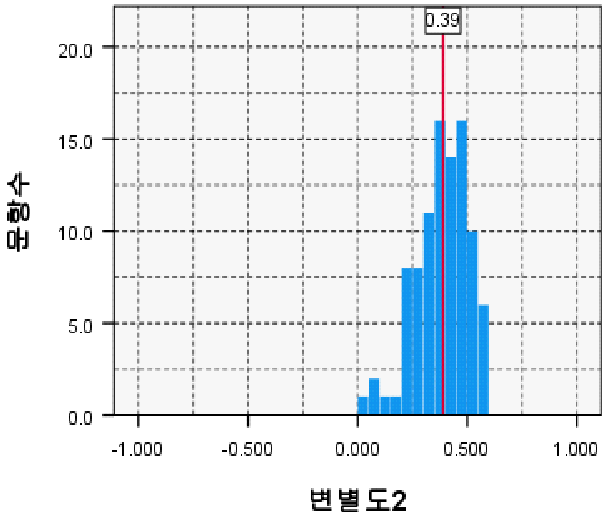

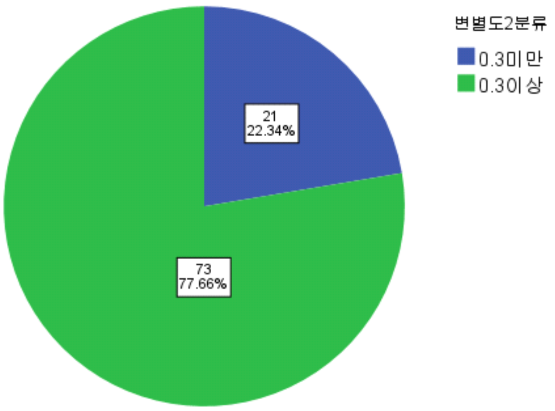

총점	변별도2	표준편차	변별도2	문항수	비율(%)
94	.39	.12	0.3미만	21	22.3
			0.3이상	73	77.7
			전체	94	100.0

해석

- 해결형 문항에서 난이도 지수가 80 에서 100 사이인 문항이 전체 94 문항 중 43 문항으로 가장 많았으며, 차례로 60 이상 80 미만인 문항이 42 문항, 0 에서 60 미만인 문항이 9 문항인 것으로 나타남
- 변별도 1 지수를 기준으로 분류하였을 때, 0.3 미만인 문항이 31 문항으로 0.3 이상인 문항이 63 문항인 것에 비해 더 적게 나타남
- 변별도 2 지수를 기준으로 분류하였을 때, 0.3 미만인 문항이 21 문항으로 0.3 이상인 문항이 73 문항인 것에 비해 더 적게 나타남

4) 자료유형별 난이도와 변별도

가) 전회 대비 자료유형별 난이도와 변별도

(1) 전회 대비 텍스트형 난이도와 변별도

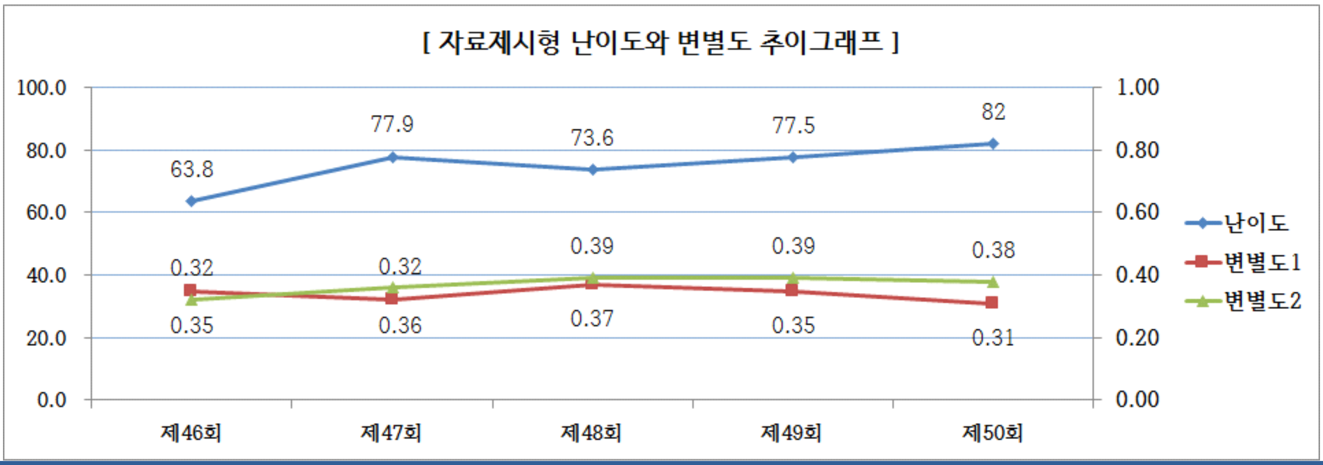

회차	난이도		변별도1		변별도2	
	평균	표준편차	평균	표준편차	평균	표준편차
제46회	71.4	17.0	.36	.13	.35	.10
제47회	79.8	13.8	.32	.13	.37	.12
제48회	77.9	12.9	.36	.14	.39	.13
제49회	77.4	15.8	.35	.15	.39	.13
**제50회**	**80.0**	**13.2**	**.33**	**.15**	**.37**	**.12**

(2) 전회 대비 자료제시형 난이도와 변별도

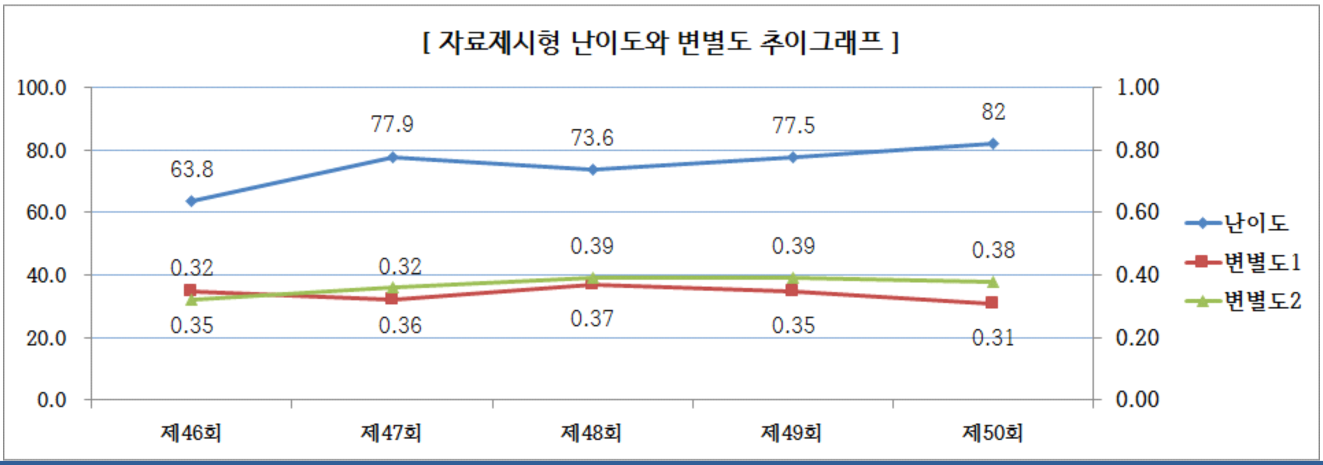

회차	난이도		변별도1		변별도2	
	평균	표준편차	평균	표준편차	평균	표준편차
제46회	63.8	19.6	.35	.15	.32	.13
제47회	77.9	15.2	.32	.13	.36	.12
제48회	73.6	16.1	.37	.13	.39	.12
제49회	77.5	17.3	.35	.14	.39	.12
**제50회**	**82.0**	**13.0**	**.31**	**.14**	**.38**	**.12**

해석

- 전회 대비 텍스트형 문항의 난이도 지수는 2.6 증가하였고 변별도 1 지수와 변별도 2 지수는 각각 .02, .02 감소함
- 자료제시형 문항의 난이도 지수는 4.5 증가하였고 변별도 1 지수와 변별도 2 지수는 각각 .04, .01 감소함

나) 자료유형별 난이도와 변별도 분포도 및 비율분석

(1) 텍스트형 난이도와 변별도 분포도 및 비율분석

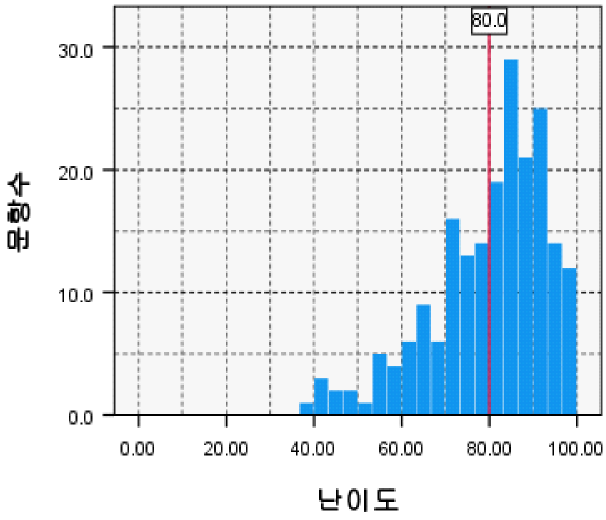

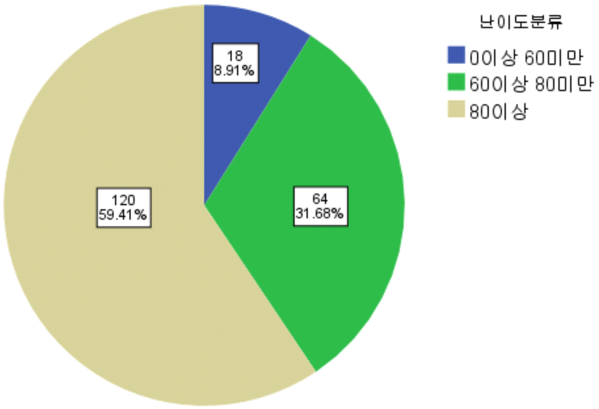

총점	난이도	표준편차
202	80.0	13.2

난이도	문항수	비율(%)
0~60미만	18	8.9
60~80미만	64	31.7
80~100	120	59.4
전체	202	100.0

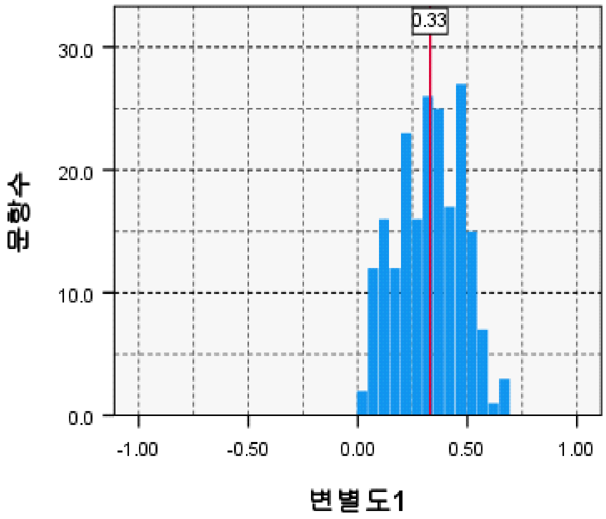

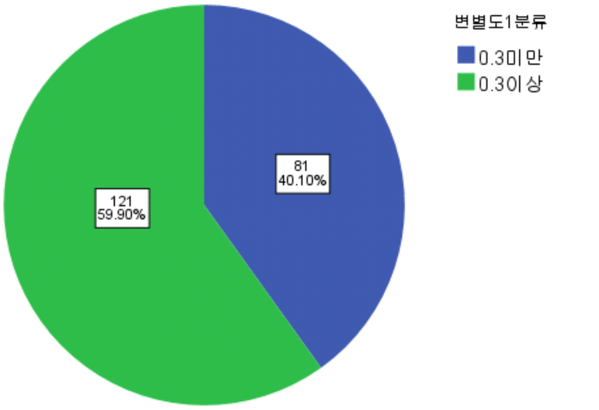

총점	변별도2	표준편차
202	.33	.15

변별도2	문항수	비율(%)
0.3미만	81	40.1
0.3이상	121	59.9
전체	202	100.0

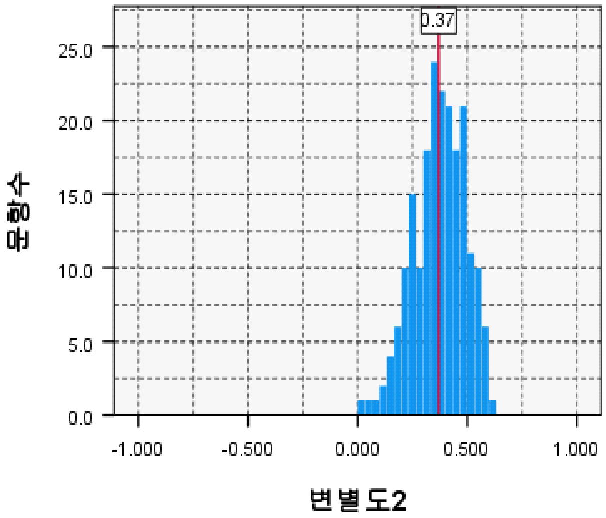

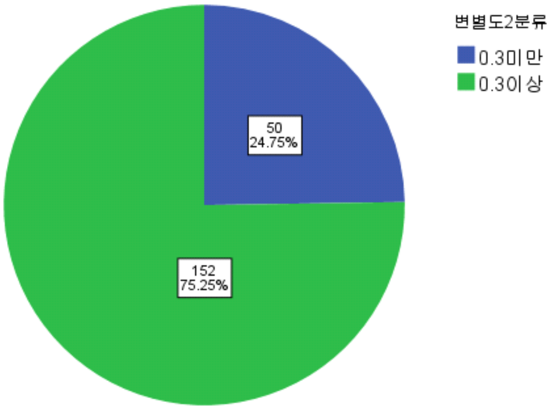

총점	변별도2	표준편차
202	.37	.12

변별도2	문항수	비율(%)
0.3미만	50	24.8
0.3이상	152	75.2
전체	202	100.0

해석

- 텍스트형 문항에서 난이도 지수가 80에서 100 사이인 문항이 전체 202문항 중 120문항으로 가장 많았으며, 차례로 60이상 80미만인 문항이 64문항, 0에서 60미만인 문항이 18문항인 것으로 나타남
- 변별도1 지수를 기준으로 분류하였을 때, 0.3미만인 문항이 81문항으로 0.3 이상인 문항이 121문항인 것에 비해 더 적게 나타남
- 변별도2 지수를 기준으로 분류하였을 때, 0.3미만인 문항이 50문항으로 0.3 이상인 문항이 152문항인 것에 비해 더 적게 나타남

(2) 자료제시형 난이도와 변별도 분포도 및 비율분석

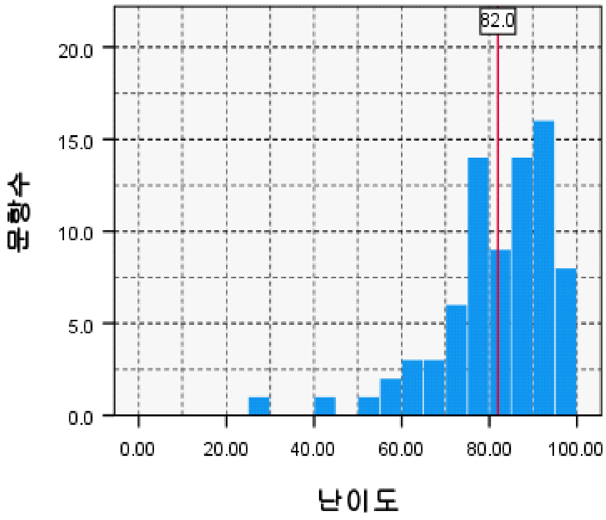

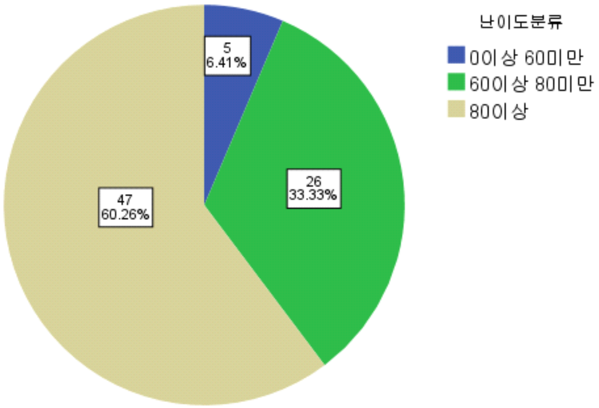

총점	난이도	표준편차	난이도	문항수	비율(%)
78	82.0	13.1	0~60미만	5	6.4
			60~80미만	26	33.3
			80~100	47	60.3
			전체	78	100.0

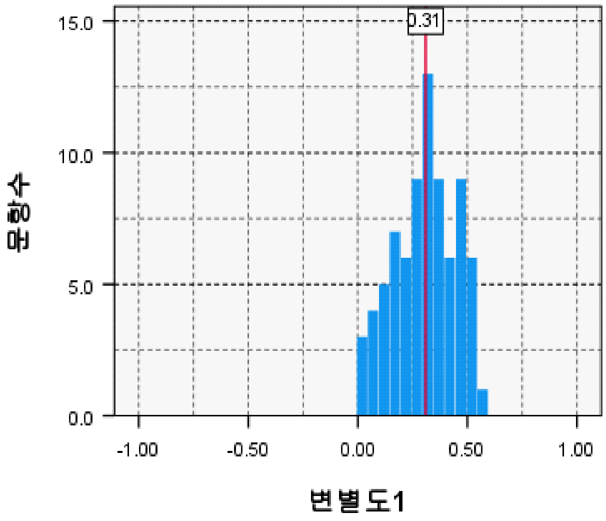

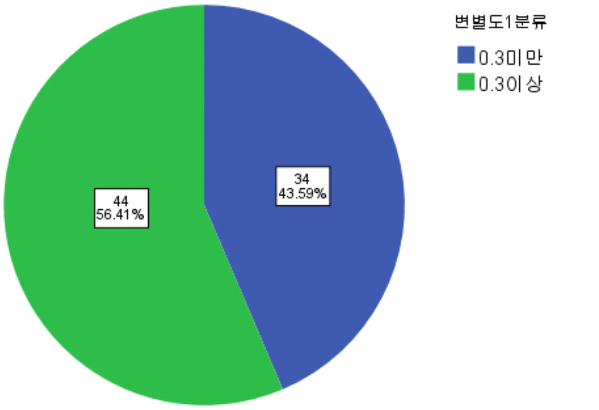

총점	변별도2	표준편차	변별도2	문항수	비율(%)
78	.31	.14	0.3미만	34	43.6
			0.3이상	44	56.4
			전체	78	100.0

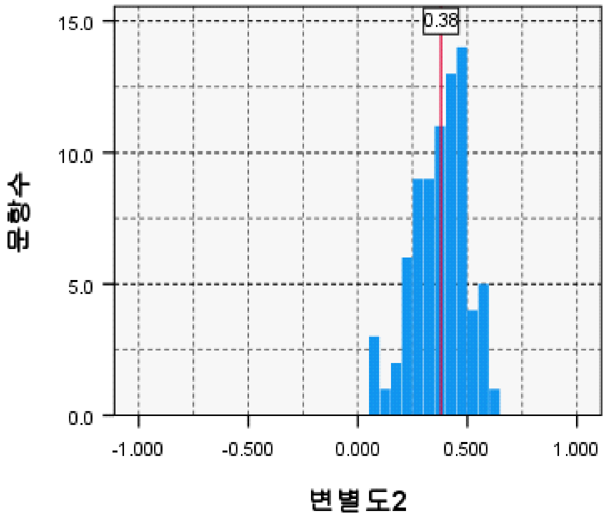

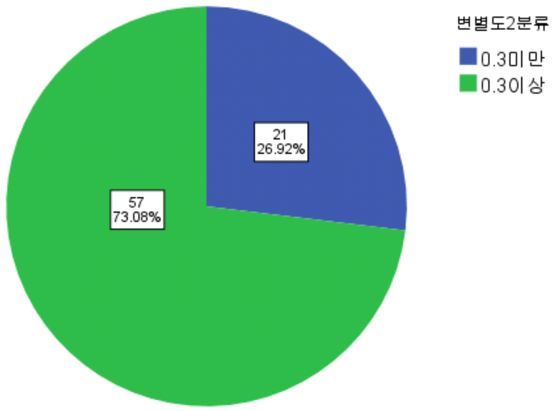

총점	변별도2	표준편차
78	.38	.12

변별도2	문항수	비율(%)
0.3미만	21	26.9
0.3이상	57	73.1
전체	78	100.0

해석

- 자료제시형 문항에서 난이도 지수가 80 에서 100 사이인 문항이 전체 78 문항 중 47 문항으로 가장 많았으며, 차례로 60 이상 80 미만인 문항이 26 문항, 0 에서 60 미만인 문항이 5 문항인 것으로 나타남
- 변별도 1 지수를 기준으로 분류하였을 때, 0.3 미만인 문항이 34 문항으로 0.3 이상인 문항이 44 문항인 것에 비해 더 적게 나타남
- 변별도 2 지수를 기준으로 분류하였을 때, 0.3 미만인 문항이 21 문항으로 0.3 이상인 문항이 57 문항인 것에 비해 더 적게 나타남

3. 난이도와 변별도 간 산포도

1) 전체 난이도와 변별도 간 산포도

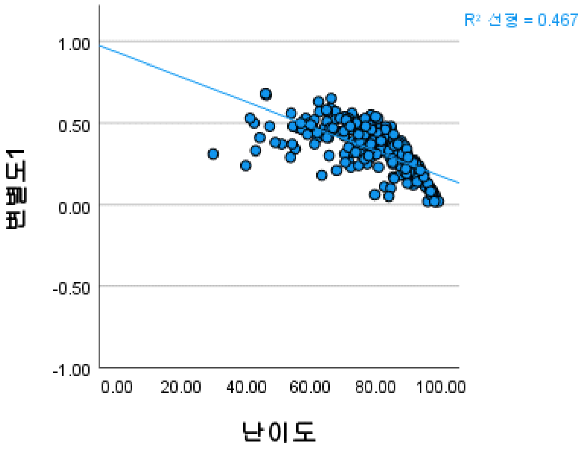

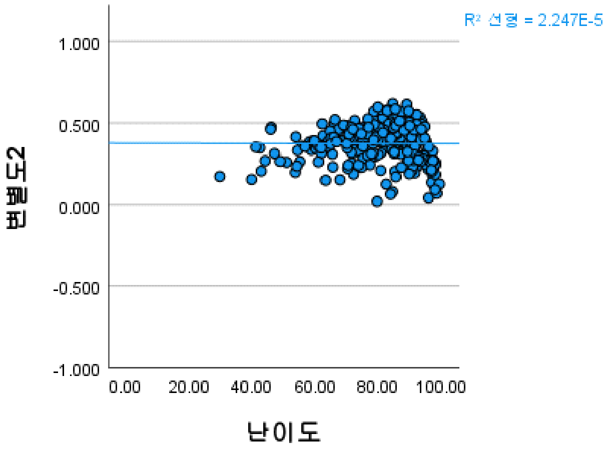

해석

- 전체 문항을 대상으로 난이도와 변별도 1 지수 간 상관은 -.683**으로 난이도 지수가 낮을수록 변별력이 높아지는 것으로 나타남
- 난이도와 변별도 2 지수 간 상관은 -.005 로 난이도와 변별력 간 관련성이 없는 것으로 나타남

2) 과목별 난이도와 변별도 간 산포도

가) 의료관계법규 난이도와 변별도 간 산포도

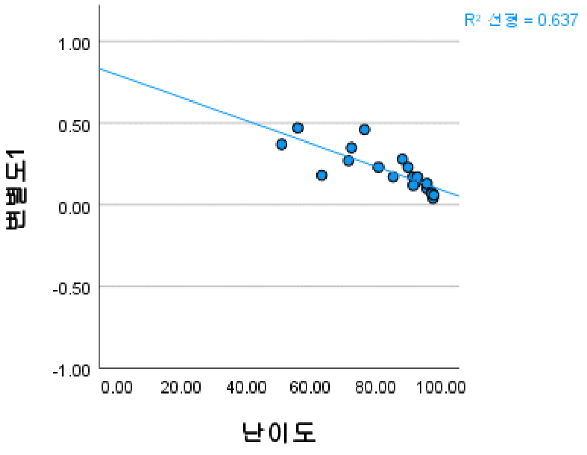

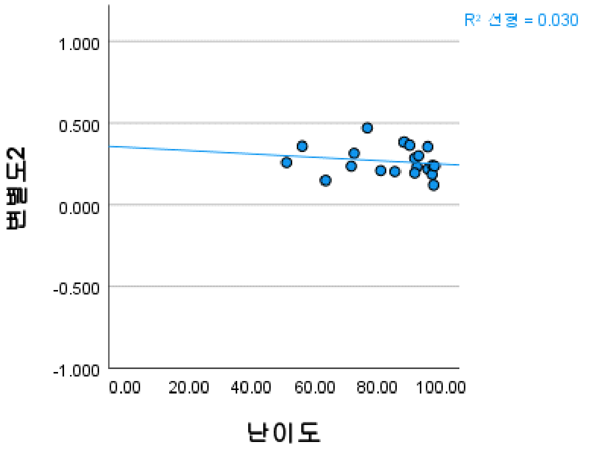

해석

- 의료관계법규 문항을 대상으로 난이도와 변별도1 지수 간 상관은 -.798**로 난이도 지수가 낮을수록 변별력이 높아지는 것으로 나타남
- 난이도와 변별도2 지수 간 상관은 -.172으로 문항 난이도와 변별력 간 관련성이 없는 것으로 나타남

나) 임상검사이론 I 난이도와 변별도 간 산포도

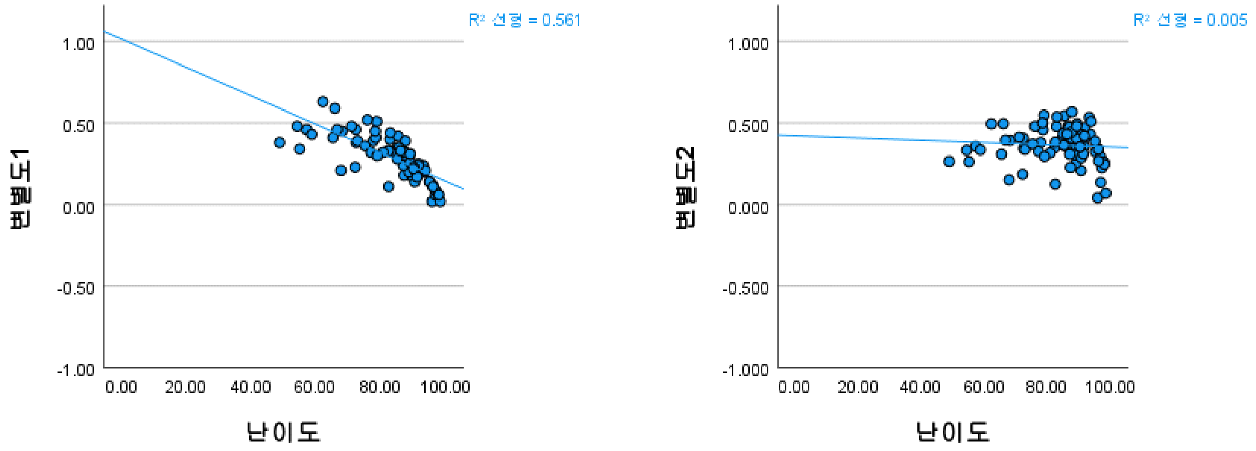

해석

- 임상검사이론I 문항을 대상으로 난이도와 변별도1 지수 간 상관은 -.749** 로 난이도 지수가 낮을수록 변별력이 높아지는 것으로 나타남
- 난이도와 변별도2 지수 간 상관은 .072로 문항 난이도와 변별력 간 관련성이 없는 것으로 나타남

다) 임상검사이론 II 난이도와 변별도 간 산포도

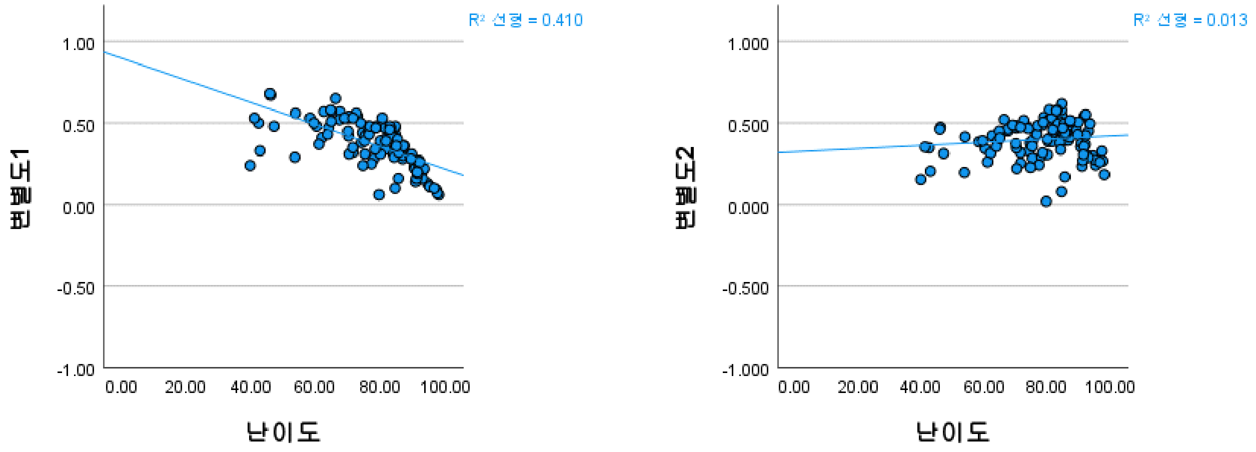

해석

- 임상검사이론Ⅱ 문항을 대상으로 난이도와 변별도1 지수 간 상관은 -.640** 로 난이도 지수가 낮을수록 변별력이 높아지는 것으로 나타남
- 난이도와 변별도2 지수 간 상관은 .114로 문항 난이도와 변별력 간 관련성이 없는 것으로 나타남

라) 실기시험 난이도와 변별도 간 산포도

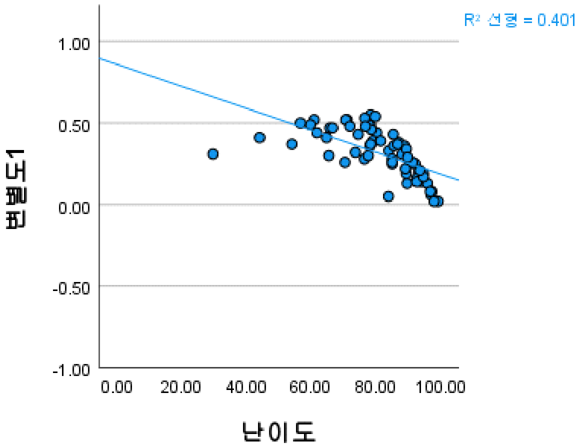

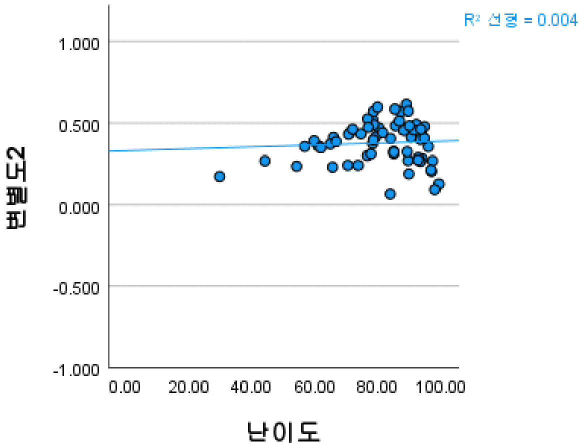

해석

- 실기시험 문항을 대상으로 난이도와 변별도1 지수 간 상관은 -.633**로 난이도 지수가 낮을수록 변별력이 높아지는 것으로 나타남
- 난이도와 변별도2 지수 간 상관은 .063으로 문항 난이도와 변별력 간 관련성이 없는 것으로 나타남

4. 신뢰도 분석

과목명	문항수	제46회	제47회	제48회	제49회	제50회
전체	280	.974	.977	.980	0.980	**.978**
의료관계법규	20	.713	.677	.662	0.661	**.635**
임상검사이론 I	80	.921	.916	.926	0.917	**.926**
임상검사이론 II	115	.944	.955	.962	0.962	**.956**
실기시험	65	.883	.906	.915	0.926	**.914**

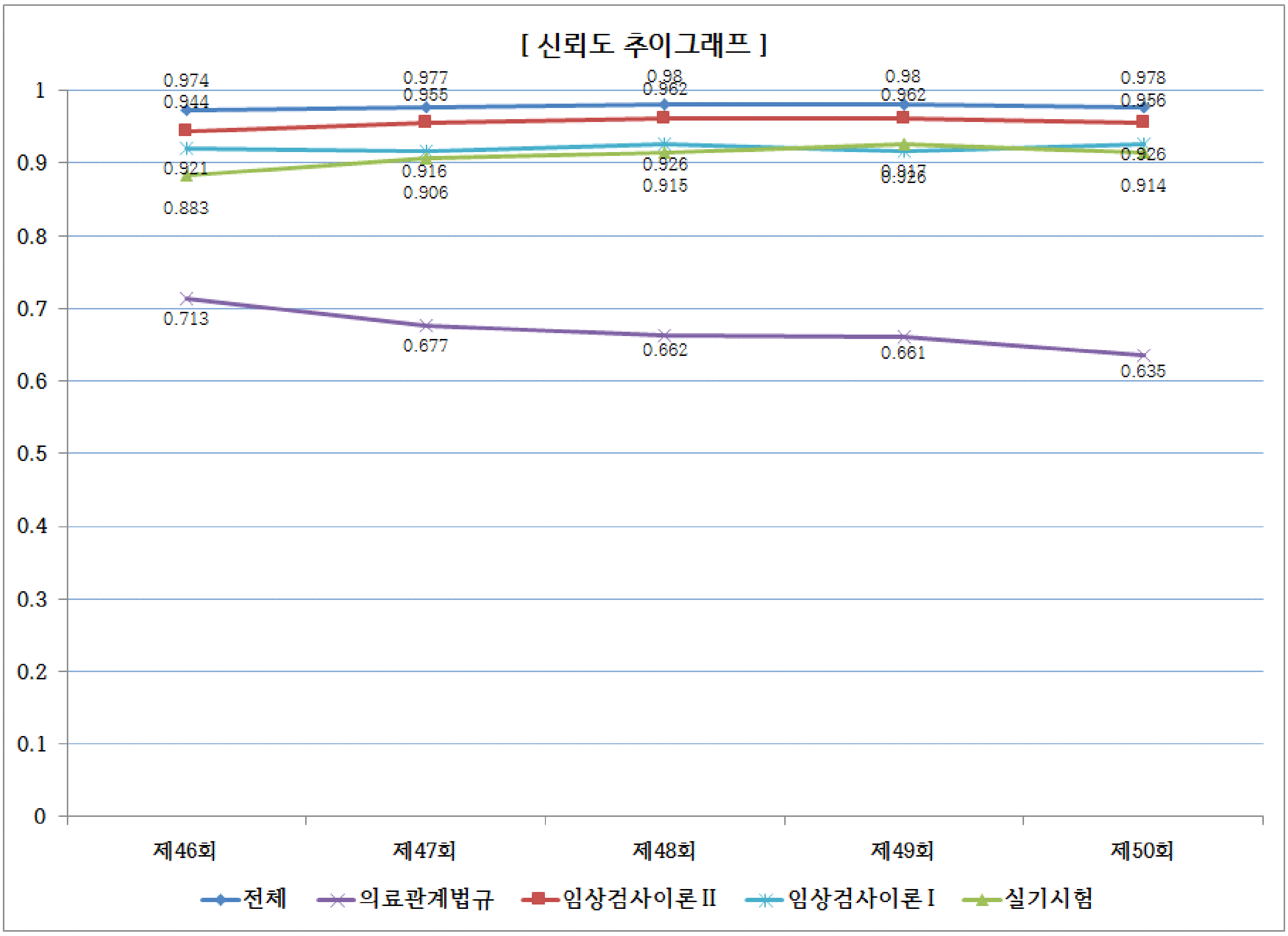

해석

- 임상병리사 전체 및 각 과목의 문항 모두 일관되게 해당 영역을 측정하고 있는 것으로 나타남
- 전회 대비 전체문항과 의료관계법규, 임상검사이론Ⅱ, 실기시험 과목 문항을 대상으로 했을 시 각각 .002, .026, .006, .012 감소함
- 임상검사이론Ⅰ 과목을 대상으로 했을 시 .009 증가함

○ 분석결과 관련 문의 : 한국보건의료인국가시험원 연구개발본부 배상영 책임연구원
Tel : 02-2087-8955, FAX : 02-2087-8885
E-mail : bsy0601@kuksiwon.or.kr
