# Supplementary material for: Item difficulty index, discrimination index, and reliability of the 26 health professions licensing examinations in 2022, Korea: a psychometric study
Source: J Educ Eval Health Prof. 2023 Nov 22;20:31. doi: 10.3352/jeehp.2023.20.31 (PMC11959405; doi:10.3352/jeehp.2023.20.31)
Supplement: Supplementary file 1 — Supplement 1. Item analysis results of 26 health professions licensing examinations administered during late 2022 and early 2023. [file jeehp-20-31_Suppl1.zip › 2022│Γ╡╡ ┴a50╚╕ └█╛≈─í╖ß╗τ ▒╣░í╜├╟Φ ║╨╝«░ß░·.pdf]

2022년도 제50회
작업치료사 국가시험
문항분석 결과

국민이 신뢰하고 감동하는 시험평가기관
한국보건의료인국가시험원
KOREA HEALTH PERSONNEL LICENSING EXAMINATION INSTITUTE

일반 용어 정의

□ **평균**

- 집단에서의 대표적 경향값으로 전체 값을 더하여 총 응시자로 나눈 값

□ **표준편차**

- 평균과 각 점수의 차이인 편차들의 평균으로 점수가 흩어져 분포되어 있는 정도

□ **검사이론**

- 검사와 검사를 구성하고 있는 문항의 양호도를 분석 및 평가하는 방법을 정의한 이론체계
- 대표적으로 고전검사이론과 문항반응이론이 있음

고전검사이론 용어 정의

□ **고전검사이론(Classical Test Theory; CTT)**

- 검사의 질을 분석하는 검사이론 중 한 가지로 19세기 말부터 전개되어 현재까지 주로 사용되고 있는 검사이론임
- 고전검사이론에 의한 문항과 응시자 능력 추정치는 다음과 같음

○ **문항난이도**

· 검사 문항의 쉽고 어려운 정도를 나타내는 지수
· 난이도 지수는 총 반응 수에 대한 정답 반응 수의 비율로 문항의 정답률임
· 문항난이도는 0~100까지의 값을 가짐
· 난이도 값이 큰 경우, 쉬운 문항으로 '난이도가 낮다'라고 해석하며, 난이도 값이 작은 경우, 어려운 문항으로 '난이도가 높다'라고 해석함

○ **문항변별도**

· 각 문항이 응시자의 능력 수준을 변별할 수 있는 정도를 나타내는 지수
· 문항변별도는 -1~+1까지의 값을 가지며, 1에 가까울수록 변별력 크다고 해석함
· 일반적으로 문항변별도가 0.3 이상이면 우수한 문항으로 평가함
· 구하는 방식에는 '상하위집단 구분법', '문항-총점 상관계수' 등이 있음
  1) 변별도 1(상하위구분법): 상위 27%와 하위 27% 집단의 난이도 차이를 구하는 방식
  2) 변별도 2(상관계수법): 문항-총점과의 상관계수로 구하는 방식

○ **신뢰도**

· 시험이 평가하고자 하는 것을 일관성 있게 측정하는가로 시험이 오차없이 정확하게 측정한 정도를 의미함
· 국시원에서는 문항의 내적일관성(Cronbach α)으로 신뢰도를 추정하며 1에 가까울수록 신뢰도가 높다고 해석함

목 차

I. 시행 결과 ······ 5

1. 시험 현황 ······ 6

1) 시험명 ······ 6

2) 시험시행일 ······ 6

3) 응시현황 ······ 6

4) 과목별 문항 수, 배점 및 과락 점수 ······ 6

2. 합격률과 평균성적 ······ 6

1) 합격 및 불합격 현황 ······ 61

2) 과목별 과락자수 내역 ······ 6

3) 전회 대비 합격률과 평균성적 ······ 7

II. 문항분석 결과 ······ 9

1. 성적 ······ 10

1) 전체 성적분포도 ······ 10

2) 과목별 성적분포도 ······ 11

2. 난이도와 변별도 ······ 12

1) 전체 난이도와 변별도 ······ 12

2) 과목별 난이도와 변별도 ······ 15

3) 지식수준별 난이도와 변별도 ······ 26

4) 자료유형별 난이도와 변별도 ······ 35

5) 문항형태별 난이도와 변별도 ······ 41

3. 난이도와 변별도 간 산포도 ······ 47

1) 전체 난이도와 변별도 간 산포도 ······ 47

2) 과목별 난이도와 변별도 간 산포도 ······ 47

4. 신뢰도 분석 ······ 50

I. 시행 결과

1. 시험 현황(※ 합격자 발표일을 기준으로 한 자료임)

1) 시험명: 2022년도 제50회 작업치료사 국가시험

2) 시험시행일: 2022년 12월 3일

3) 응시현황

응시대상자수	결시자수	부정행위자수	응시자 준수사항 위반자 수		응시자수 (%)
			휴대폰 소지	신분증 미지참	
2,013	18	0	0	0	1,995 (99.1)

4) 과목별 문항 수, 배점 및 과락 점수

교 시	과 목 명	문제 수	배점	총점	합격자 점수기준	
					과목 과락기준	총점 합격기준
1교시	작업치료학 기초	70	1	70	28점 미만	114점 이상
1교시	의료관계법규	20	1	20	8점 미만	
2교시	작업치료학	100	1	100	40점 미만	
2교시	실기시험	50	1	50	30점 미만	-
계		240	-	240	-	-

2. 합격률과 평균성적

1) 합격 및 불합격 현황

합격자수 (%)	불합격자수(%)					채점보류자수
	평락	과락	실기탈락	기권	계	
1,577 (79.0)	271 (13.6)	128 (6.4)	19 (1.0)	0 (0.0)	418 (21.0)	0

2) 과목별 과락자수 내역

과목명 / 과락자수	작업치료학 기초	의료관계법규	작업치료학	실기시험
과목별 과락자 수	0	128	0	0
전과목 과락자 수	0			

3) 전회 대비 합격률과 평균성적

회차	년도	합격률(%)	평균성적	표준편차	백분율 환산점수
제46회	2018.12	88.0	180.4	33.2	75.2
제47회	2019.12	91.1	181.4	28.5	75.6
제48회	2020.12	93.3	186.5	28.7	77.7
제49회	2021.12	91.0	181.6	30.1	75.7
**제50회**	**2022.12**	**79.0**	**173.3**	**38.2**	**72.2**

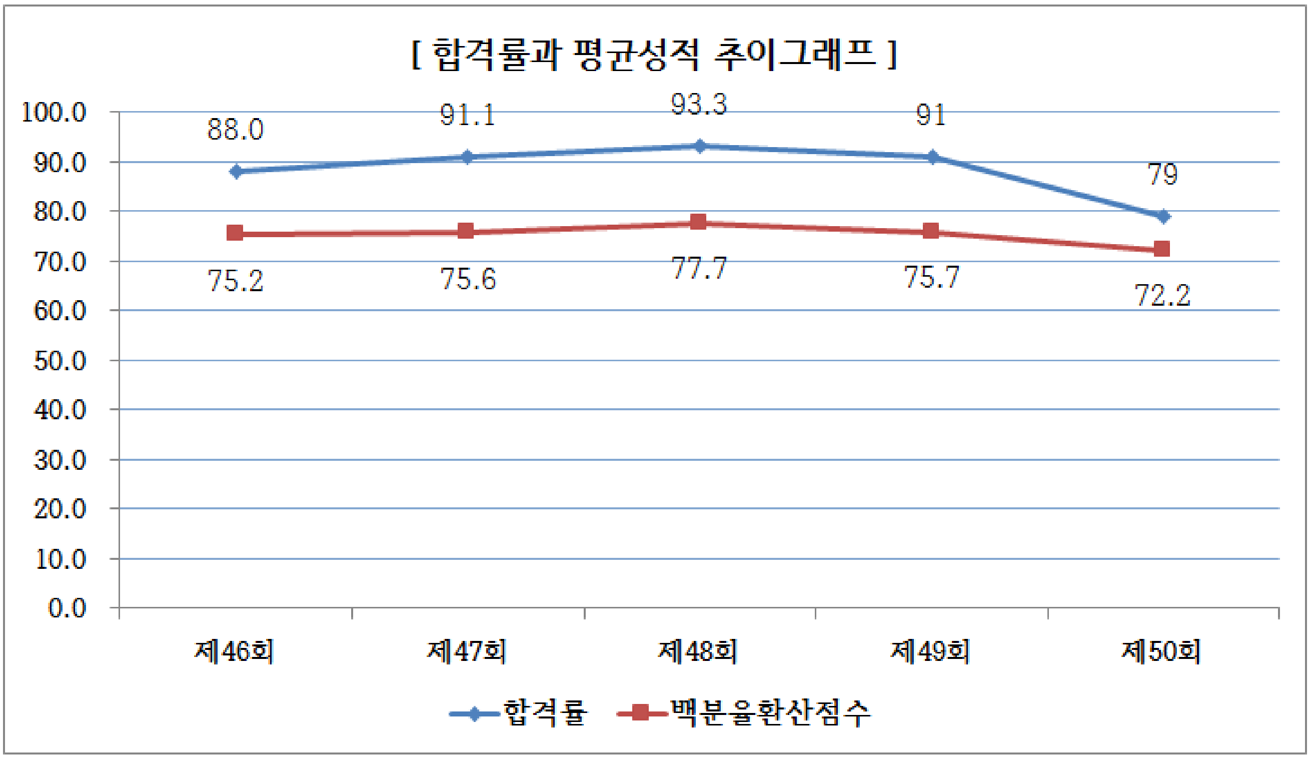

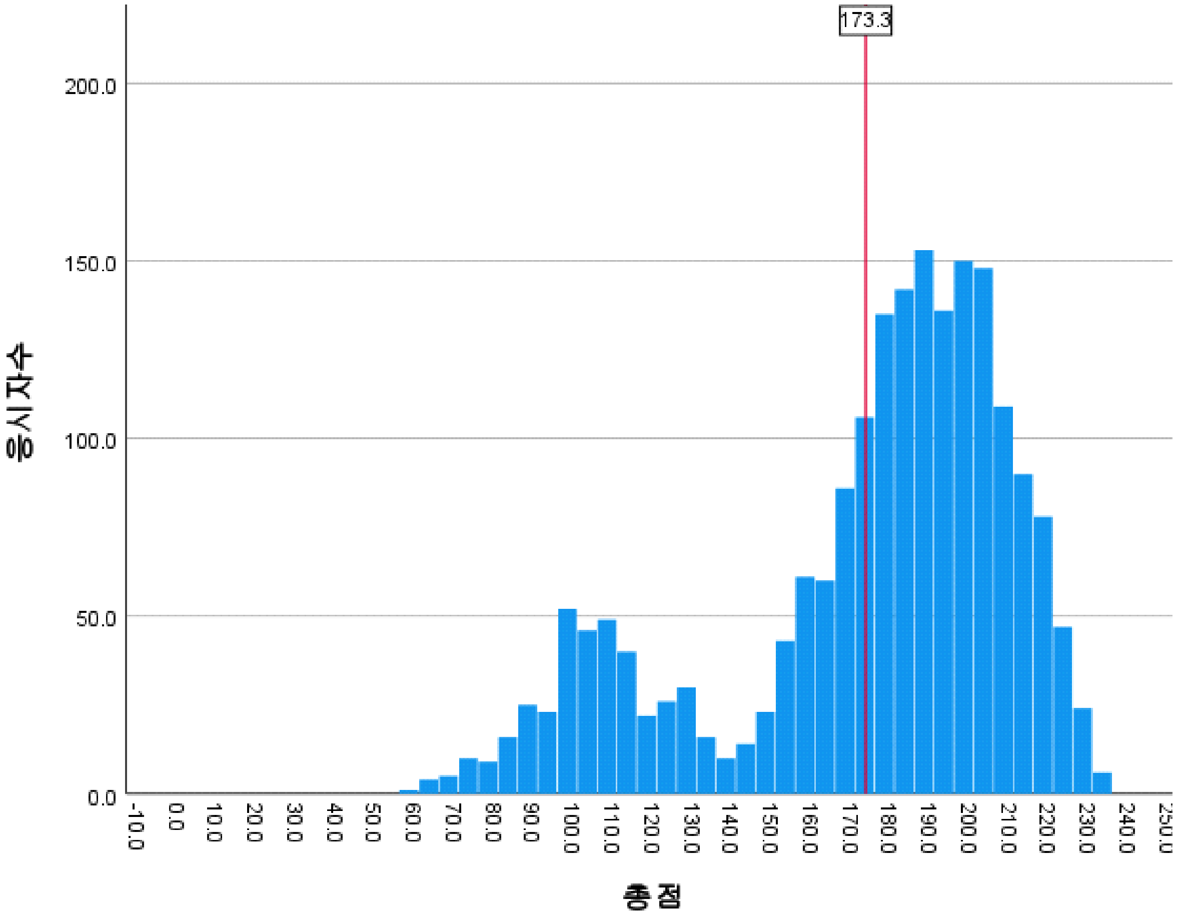

응시자	총점	합격선	평균성적	표준편차
1,995	240.0		173.3	38.2

※ 필기시험 불합격자의 실기성적을 포함하지 않음

해석

- 전년 대비 합격률은 12.0%, 백분율 환산점수는 3.5 감소함

II. 문항분석 결과

1. 성적

1) 전체 성적분포도

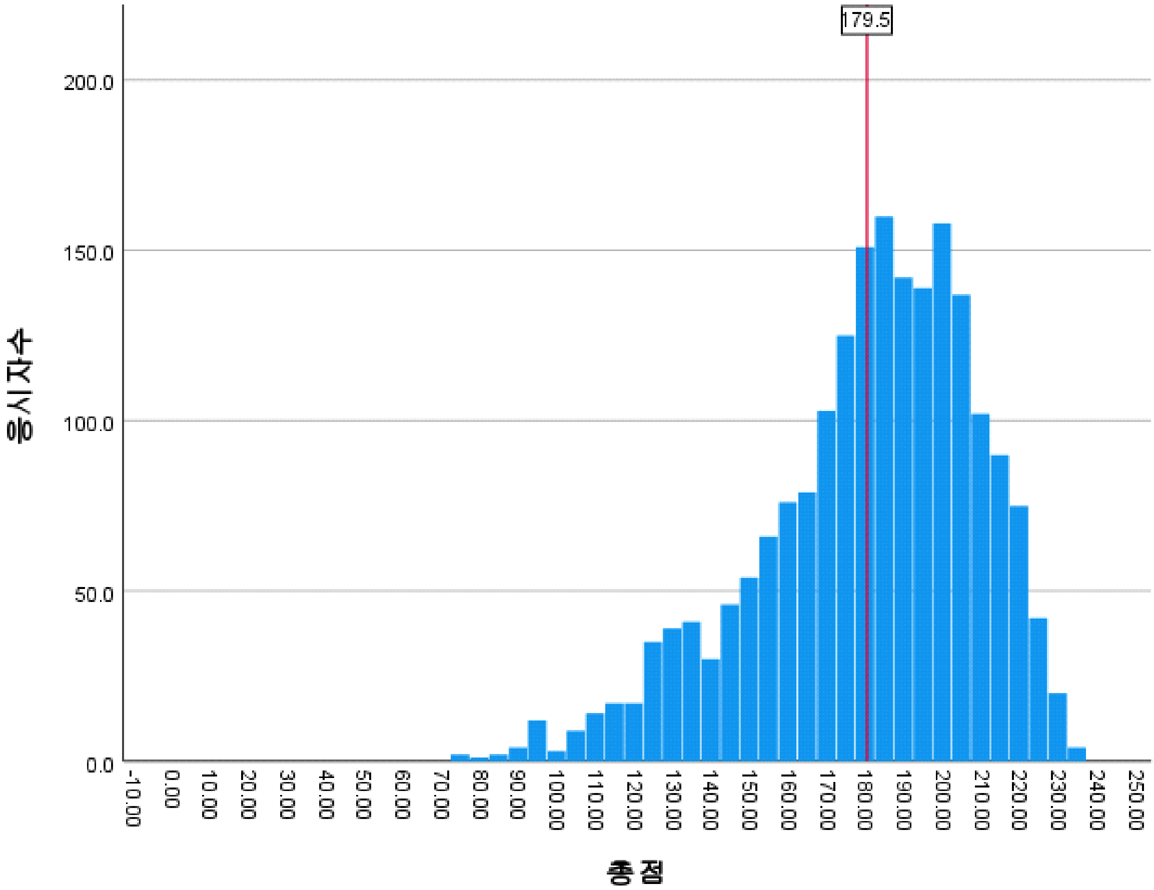

응시자	총점	합격선	평균성적	표준편차
1,995	240.0		179.5	28.7

※ 필기시험 불합격자의 실기성적을 포함함

2) 과목별 성적분포도

가) 작업치료학 기초

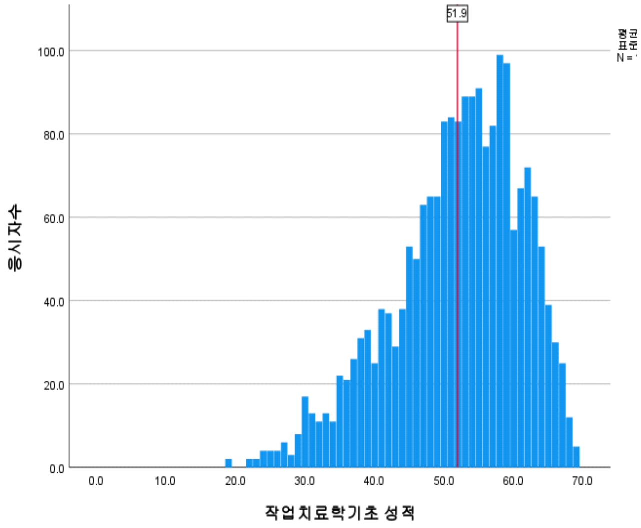

총점	과락선	평균성적	표준편차
70.0	28.0	51.9	9.3

나) 의료관계법규

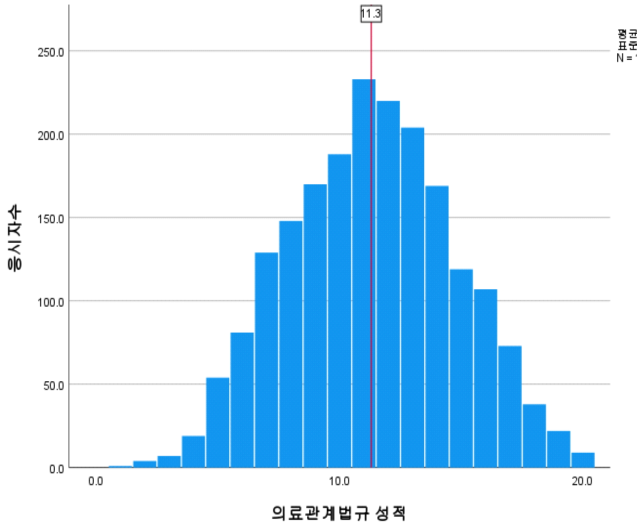

총점	과락선	평균성적	표준편차
20.0	8.0	11.3	3.4

다) 작업치료학

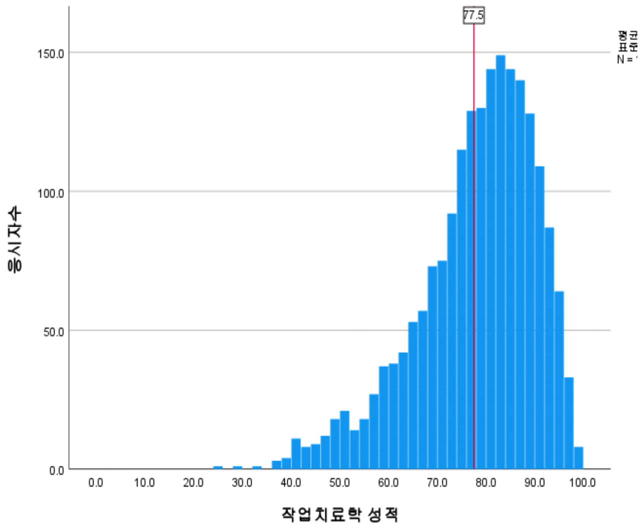

총점	과락선	평균성적	표준편차
100.0	40.0	77.5	12.2

라) 실기시험

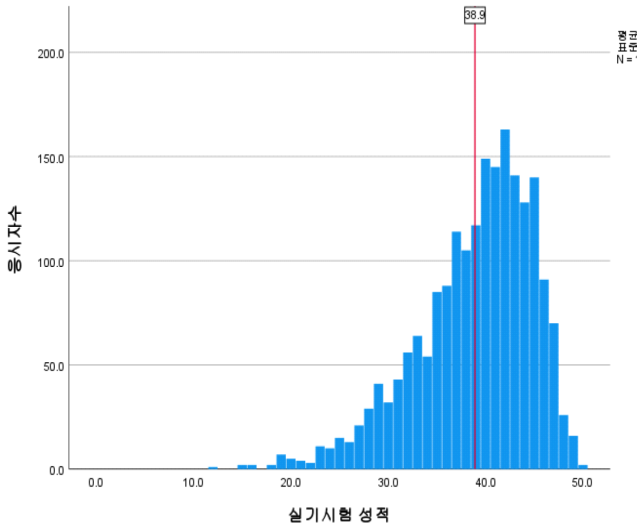

총점	과락선	평균성적	표준편차
50.0	30.0	38.9	6.0

2. 난이도와 변별도

1) 전체 난이도와 변별도

가) 전회 대비 전체 난이도와 변별도

회차	난이도		변별도1		변별도2	
	평균	표준편차	평균	표준편차	평균	표준편차
제46회	76.6	18.0	.25	.14	.27	.11
제47회	76.5	20.0	.22	.13	.25	.11
제48회	78.4	18.0	.23	.13	.26	.10
제49회	76.6	19.5	.24	.14	.27	.11
**제50회**	**74.8**	**18.0**	**.28**	**.15**	**.29**	**.11**

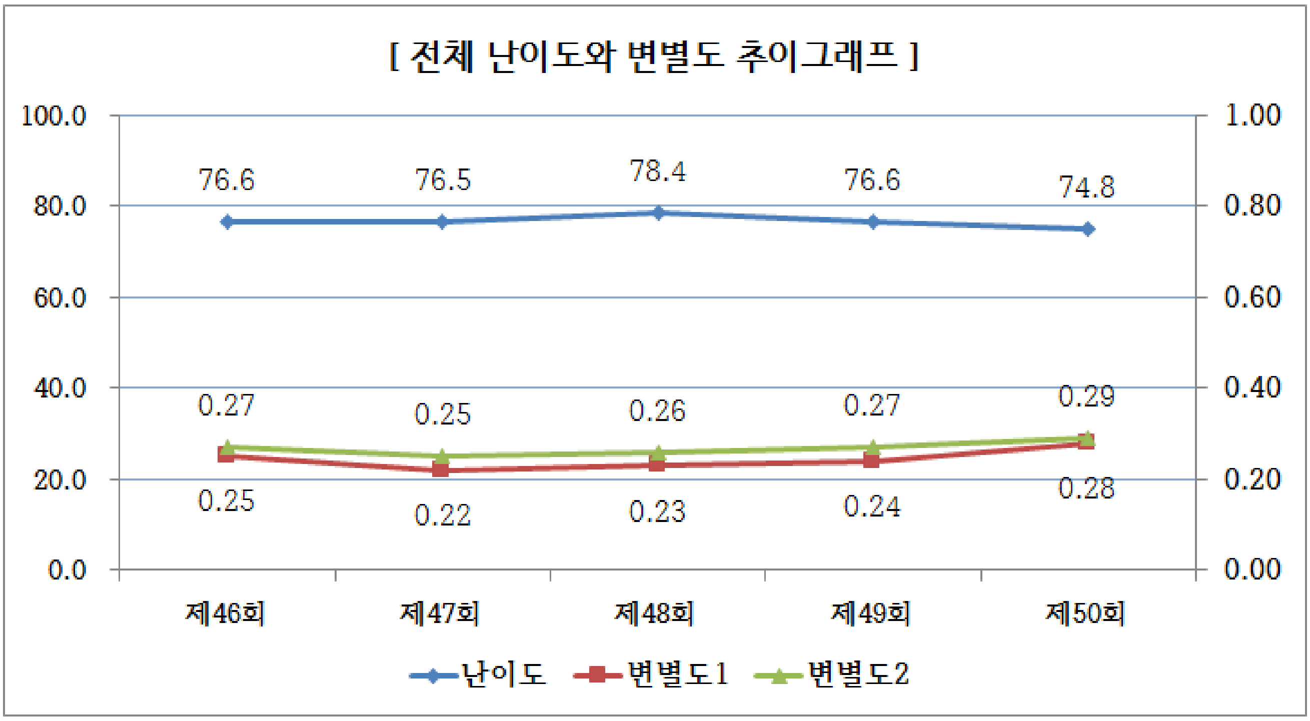

해석

- 전년 대비 난이도 지수는 1.8 감소함
- 변별도 1 지수는 .04 증가함
- 변별도 2 지수는 .02 증가함

나) 전체 난이도와 변별도 분포도 및 비율분석

(1) 전체 난이도 분포도 및 비율분석

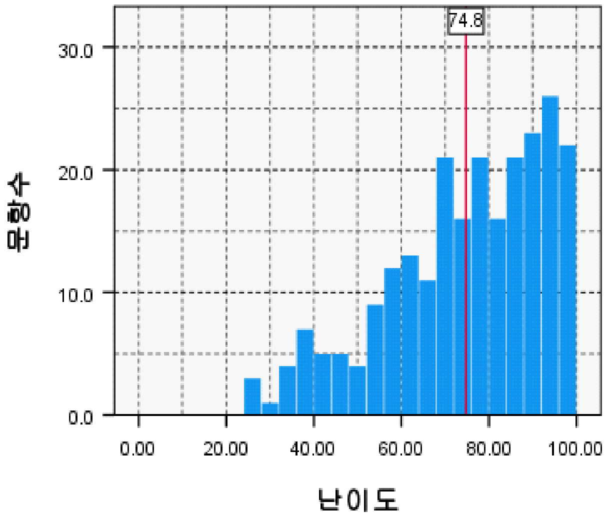

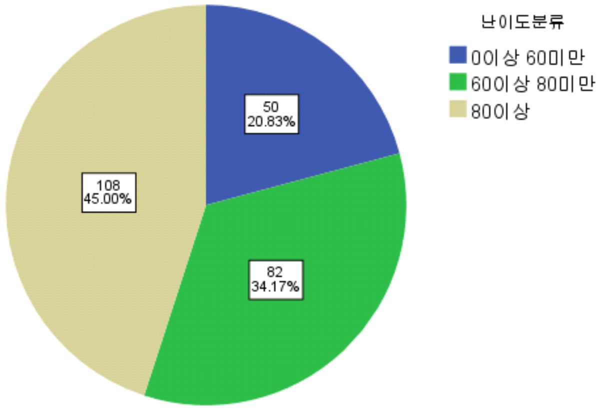

총점	난이도	표준편차
240	74.8	18.1

난이도	문항수	비율(%)
0~60미만	50	20.8
60~80미만	82	34.2
80~100	108	45.0
전체	240	100.0

(2) 전체 변별도1 분포도 및 비율분석

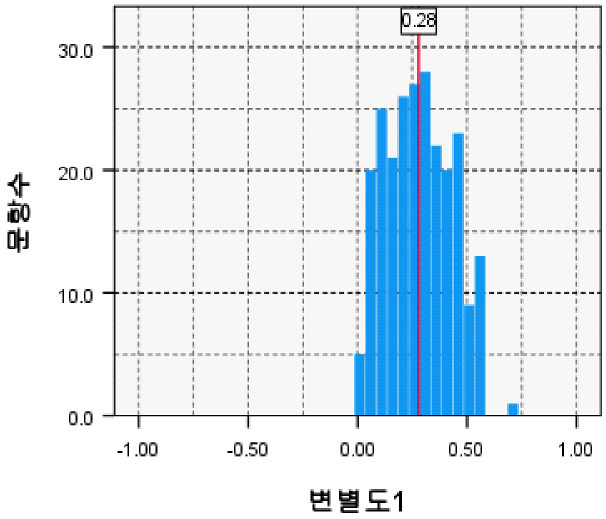

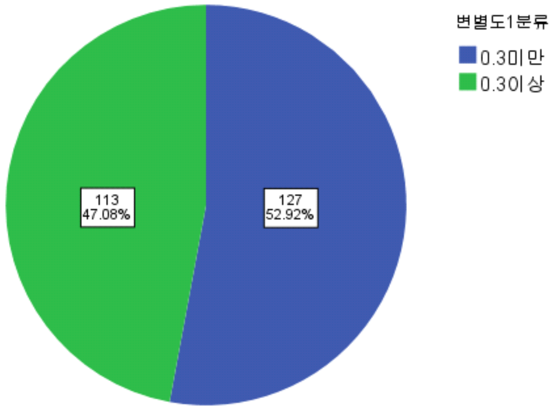

총점	변별도1	표준편차
240	.28	.15

변별도1	문항수	비율(%)
0.3미만	127	52.9
0.3이상	113	47.1
전체	240	100.0

(3) 전체 변별도2 분포도 및 비율분석

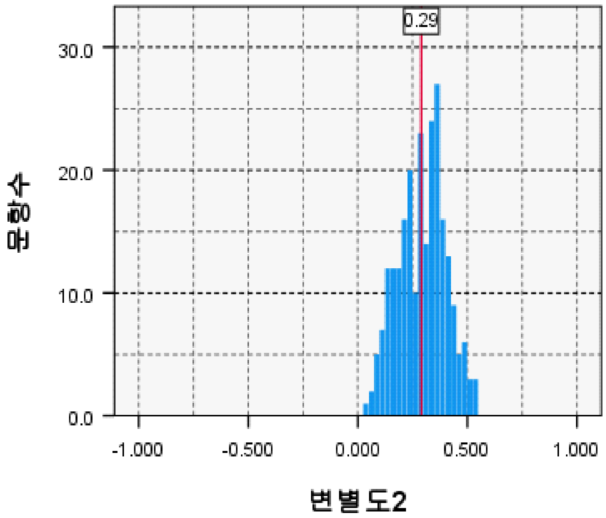

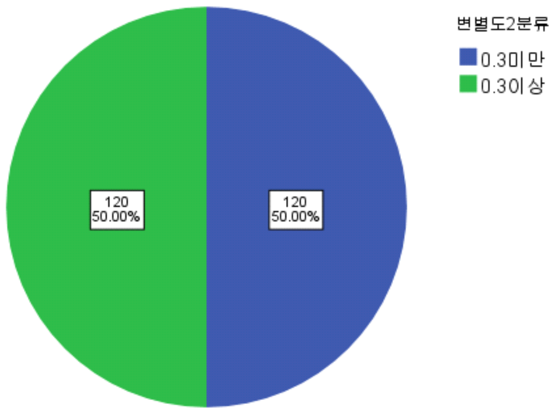

총점	변별도2	표준편차
240	.29	.11

변별도2	문항수	비율(%)
0.3미만	120	50.0
0.3이상	120	50.0
전체	240	100.0

해석

- 난이도 지수가 80 에서 100 사이인 문항이 전체 240 문항 중 108 문항으로 가장 많았으며, 차례로 60 이상 80 미만인 문항이 82 문항, 60 미만인 문항이 50 문항인 것으로 나타남
- 변별도 1 지수를 기준으로 분류하였을 때, 0.3 미만인 문항이 127 문항으로 0.3 이상인 문항이 113 문항인 것에 비해 더 많이 나타남
- 변별도 2 지수를 기준으로 분류하였을 때, 0.3 미만인 문항이 120 문항으로 0.3 이상인 문항이 120 문항인 것에 비해 같게 나타남

2) 과목별 난이도와 변별도

가) 전회 대비 과목별 난이도와 변별도

(1) 전회 대비 작업치료학 기초 난이도와 변별도

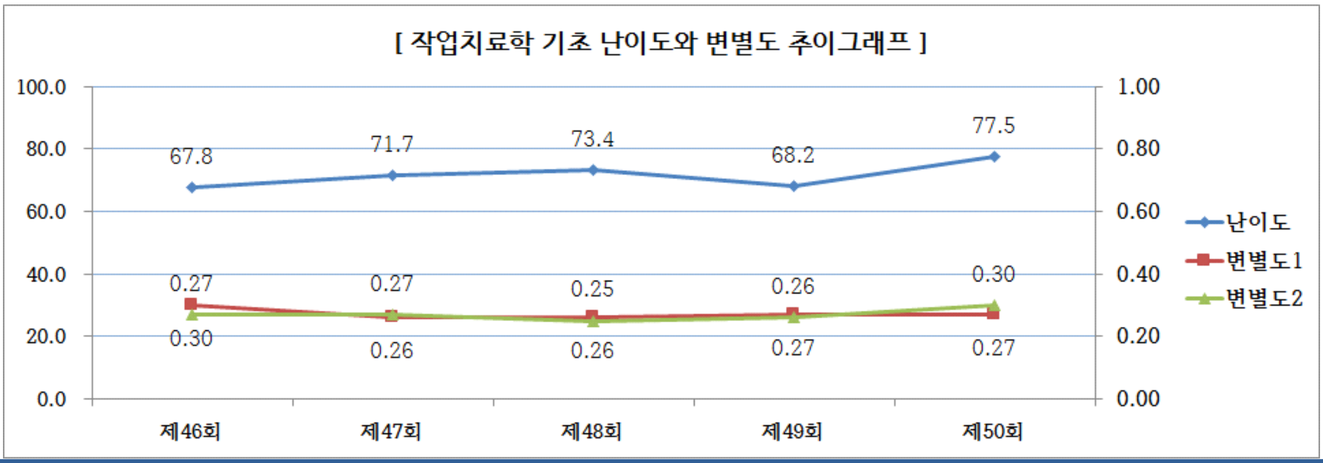

회차	난이도		변별도1		변별도2	
	평균	표준편차	평균	표준편차	평균	표준편차
제46회	67.8	19.2	.30	.13	.27	.11
제47회	71.7	20.6	.26	.13	.27	.10
제48회	73.4	19.2	.26	.14	.25	.09
제49회	68.2	22.5	.27	.14	.26	.10
**제50회**	**77.5**	**16.3**	**.27**	**.16**	**.30**	**.12**

해석

- 전회 대비 작업치료학 기초 과목의 난이도 지수는 9.3 증가함
- 전회 대비 작업치료학 기초 과목의 변별도 1 지수는 동일함
- 전회 대비 작업치료학 기초 과목의 변별도 2 지수는 .04 증가함

(2) 전회 대비 의료관계법규 난이도와 변별도

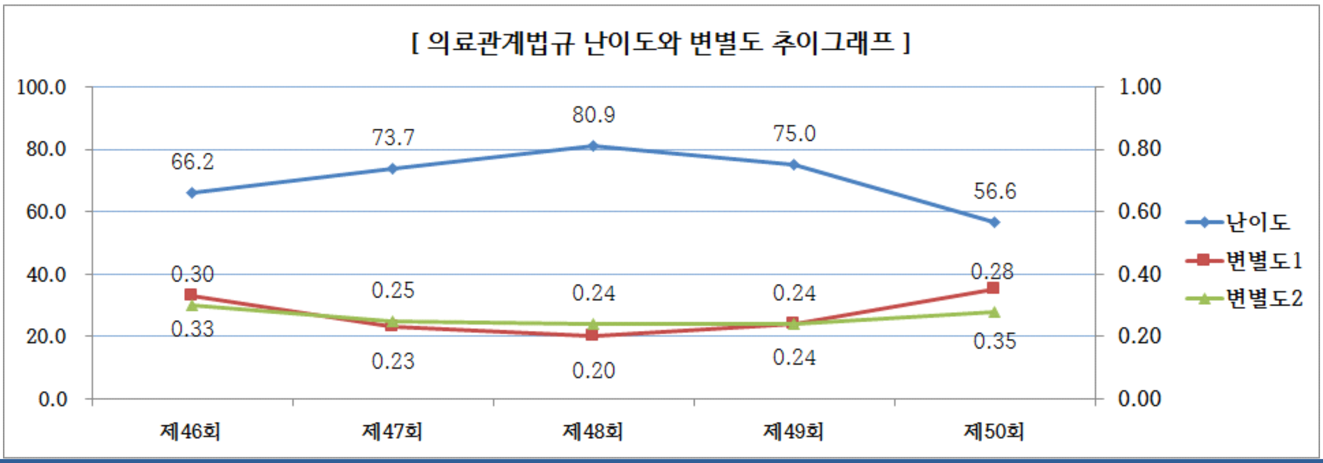

회차	난이도		변별도1		변별도2	
	평균	표준편차	평균	표준편차	평균	표준편차
제46회	66.2	16.9	.33	.10	.30	.10
제47회	73.7	22.3	.23	.10	.25	.09
제48회	80.9	18.1	.20	.13	.24	.09
제49회	75.0	19.3	.24	.11	.24	.08
**제50회**	56.6	21.2	.35	.12	.28	.09

해석

- 전회 대비 의료관계법규 과목의 난이도 지수는 18.4 감소함
- 전회 대비 의료관계법규 과목의 변별도 1 지수는 .11 증가함
- 전회 대비 의료관계법규 과목의 변별도 2 지수는 .04 증가함

(3) 전회 대비 작업치료학 난이도와 변별도

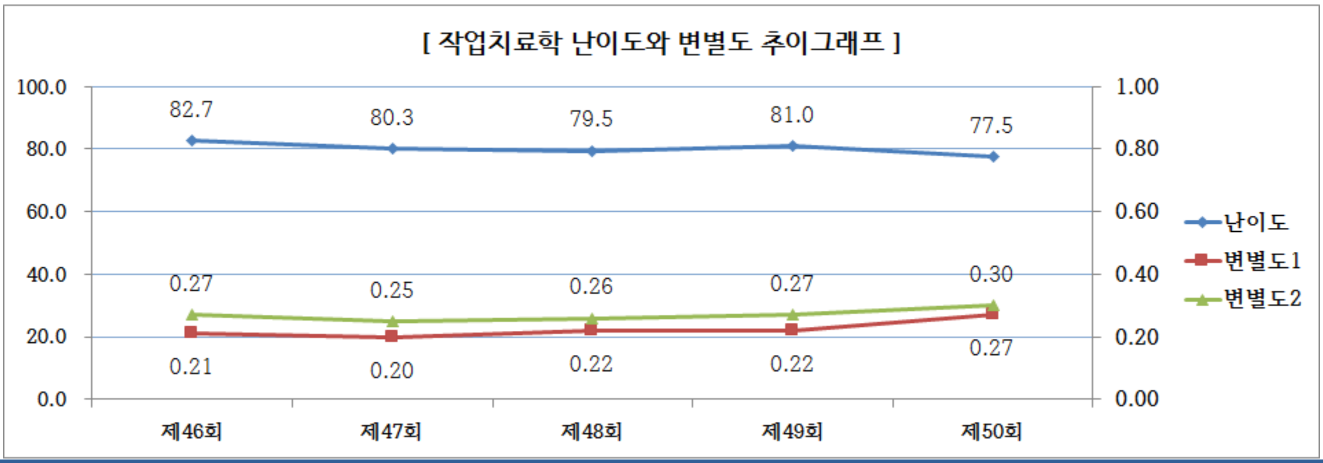

회차	난이도		변별도1		변별도2	
	평균	표준편차	평균	표준편차	평균	표준편차
제46회	82.7	15.5	.21	.13	.27	.11
제47회	80.3	17.8	.20	.12	.25	.10
제48회	79.5	17.9	.22	.14	.26	.11
제49회	81.0	17.4	.22	.14	.27	.11
**제50회**	**77.5**	**16.3**	**.27**	**.16**	**.30**	**.12**

해석

- 전회 대비 작업치료학 과목의 난이도 지수는 3.5 감소함
- 전회 대비 작업치료학 과목의 변별도 1 지수는 .05 증가함
- 전회 대비 작업치료학 과목의 변별도 2 지수는 .03 증가함

(4) 전회 대비 실기시험 난이도와 변별도

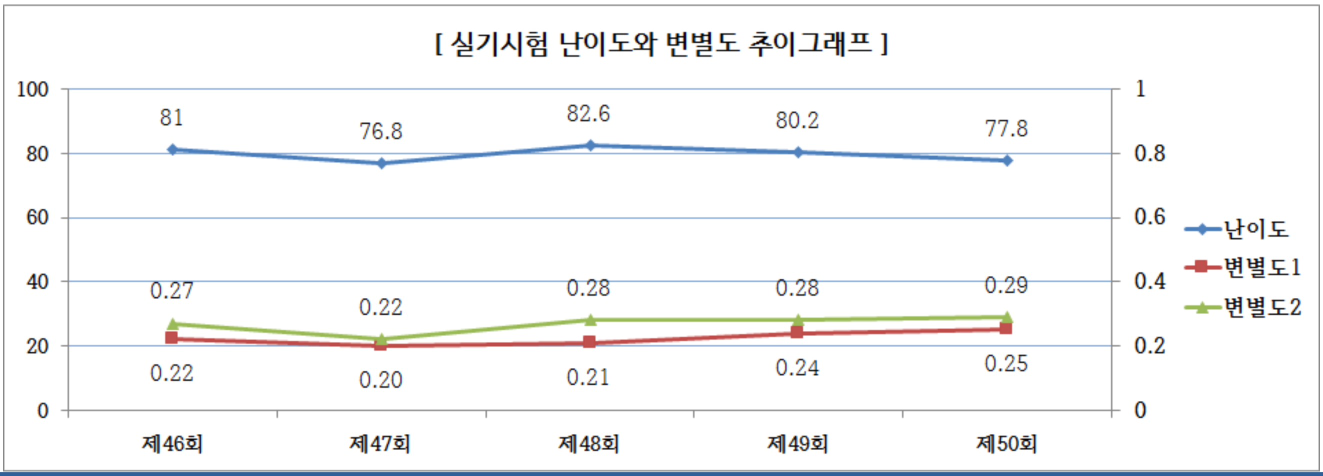

회차	난이도		변별도1		변별도2	
	평균	표준편차	평균	표준편차	평균	표준편차
제46회	81.0	14.4	.22	.13	.27	.12
제47회	76.8	21.1	.20	.15	.22	.13
제48회	82.6	15.2	.21	.12	.28	.10
제49회	80.2	15.6	.24	.13	.28	.11
**제50회**	**77.8**	**17.8**	**.25**	**.15**	**.29**	**.11**

해석

- 전회 대비 실기시험 과목의 난이도 지수는 2.4 감소함
- 전회 대비 실기시험 과목의 변별도 1 지수는 .01 증가함
- 전회 대비 실기시험 과목의 변별도 2 지수는 .01 증가함

나) 과목별 난이도와 변별도 분포도 및 비율분석

(1) 작업치료학 기초 난이도와 변별도 분포도 및 비율분석

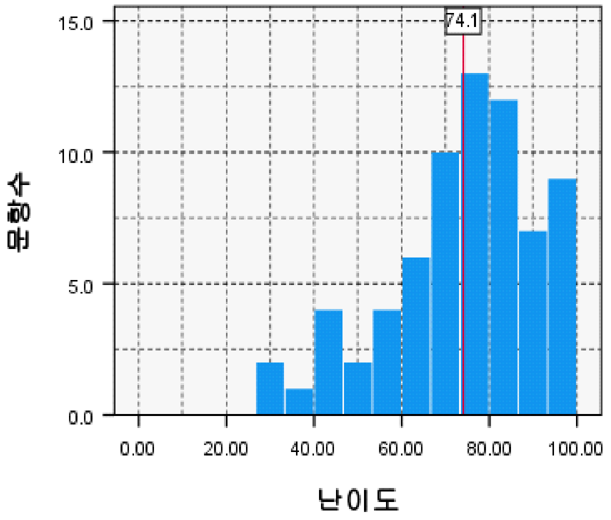

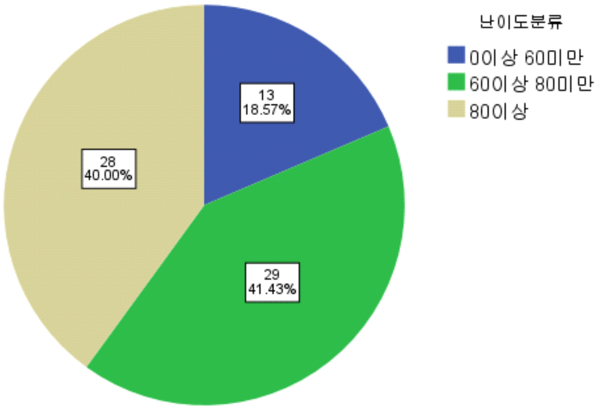

총점	난이도	표준편차
70	74.1	16.5

난이도	문항수	비율(%)
0~60미만	13	18.6
60~80미만	29	41.4
80~100	28	40.0
전체	70	100.0

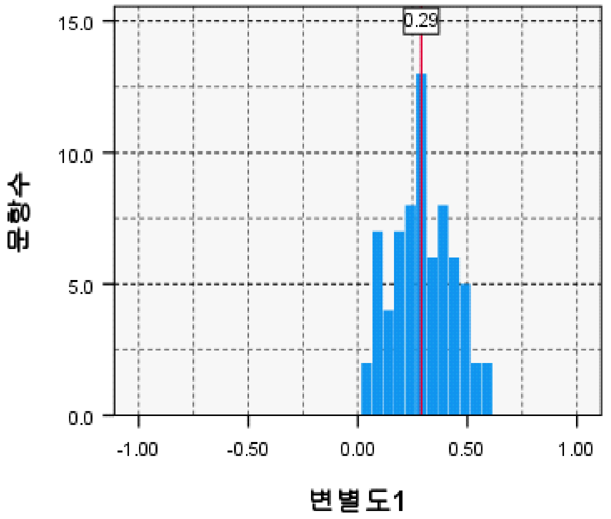

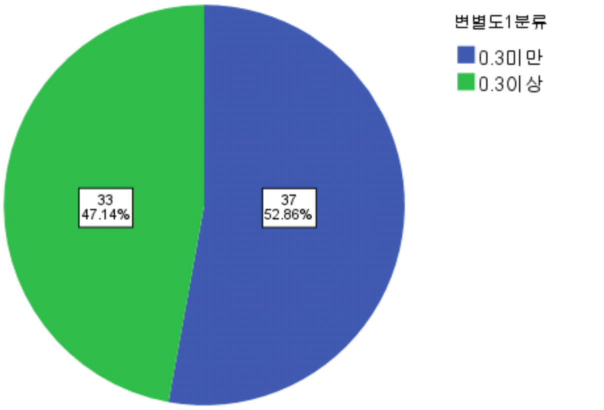

총점	변별도1	표준편차
70	.29	.14

변별도1	문항수	비율(%)
0.3미만	37	52.9
0.3이상	33	47.1
전체	70	100.0

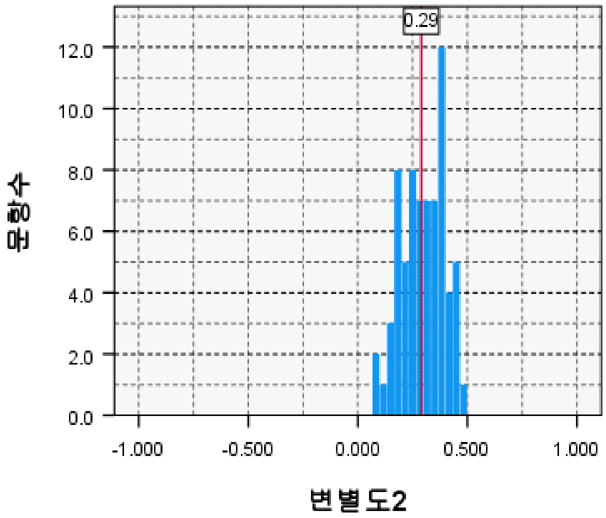

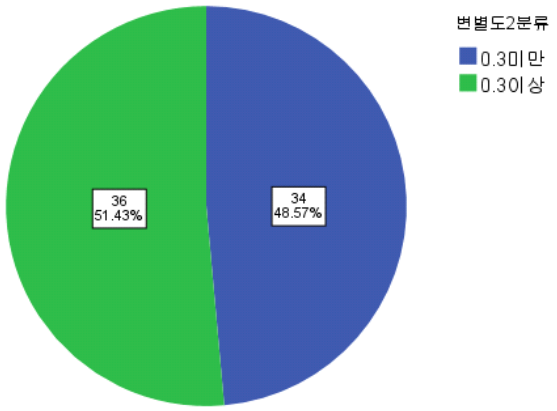

총점	변별도2	표준편차
70	.29	.10

변별도2	문항수	비율(%)
0.3미만	34	48.6
0.3이상	36	51.4
전체	70	100.0

해석

- 작업치료학 기초 과목에서 난이도 지수가 60 이상 80 미만인 문항이 전체 70 문항 중 29 문항으로 가장 많았으며, 차례로 80 에서 100 사이인 문항이 28 문항, 60 미만인 문항이 13 문항인 것으로 나타남
- 변별도 1 지수를 기준으로 분류하였을 때, 0.3 미만인 문항이 37 문항으로 0.3 이상인 문항이 33 문항인 것에 비해 더 많이 나타남
- 변별도 2 지수를 기준으로 분류하였을 때, 0.3 미만인 문항이 34 문항으로 0.3 이상인 문항이 36 문항인 것에 비해 더 적게 나타남

(2) 의료관계법규 난이도와 변별도 분포도 및 비율분석

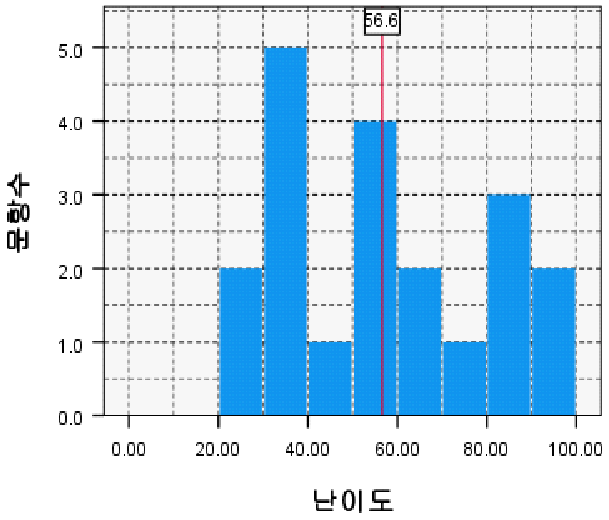

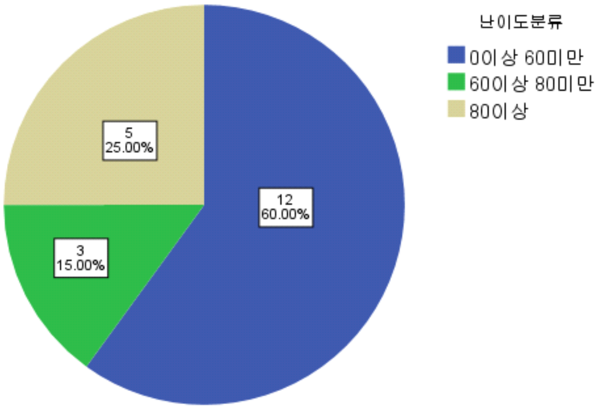

총점	난이도	표준편차
20	56.6	21.8

난이도	문항수	비율(%)
0~60미만	12	60.0
60~80미만	3	15.0
80~100	5	25.0
전체	20	100.0

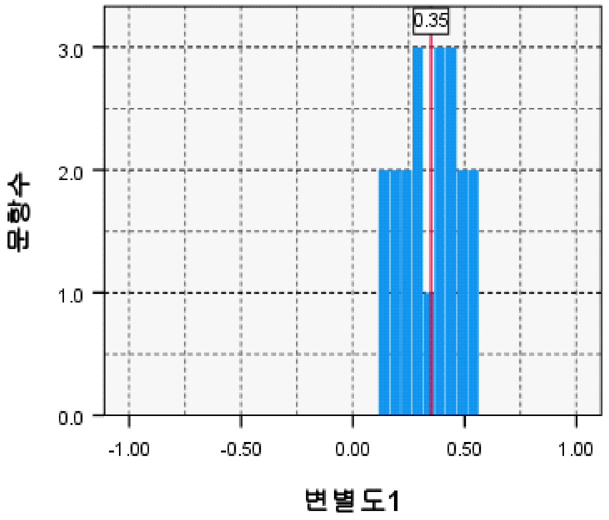

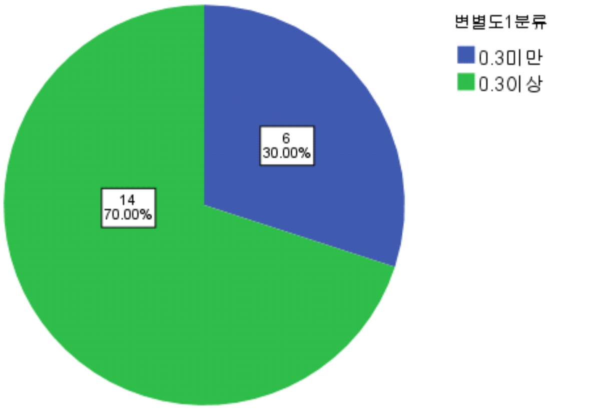

총점	변별도1	표준편차
20	.35	.12

변별도1	문항수	비율(%)
0.3미만	6	30.0
0.3이상	14	70.0
전체	20	100.0

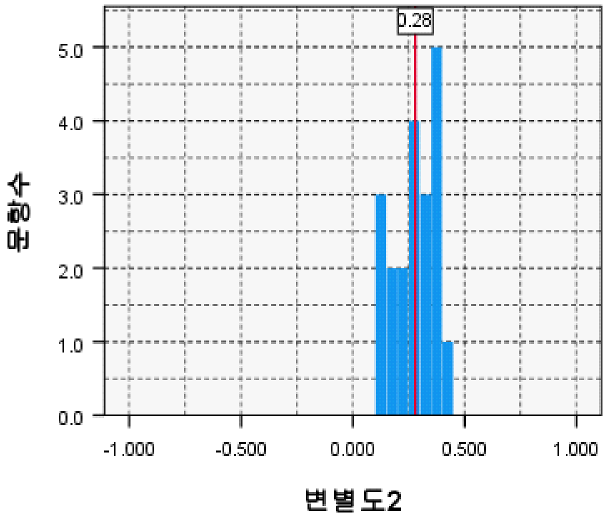

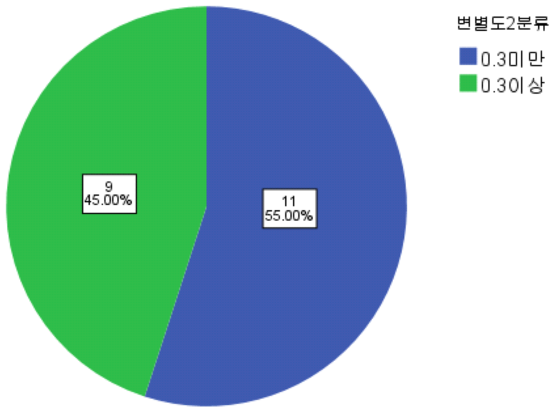

총점	변별도2	표준편차	변별도2	문항수	비율(%)
20	.28	.09	0.3미만	11	55.0
			0.3이상	9	45.0
			전체	20	100.0

해석

- 의료관계법규 과목에서 난이도 지수가 60 미만인 문항이 전체 20 문항 중 12 문항으로 가장 많았으며, 차례로 80 에서 100 사이인 문항이 5 문항, 60 이상 80 미만인 문항이 3 문항인 것으로 나타남
- 변별도 1 지수를 기준으로 분류하였을 때, 0.3 미만인 문항이 6 문항으로 0.3 이상인 문항이 14 문항인 것에 비해 더 적게 나타남
- 변별도 2 지수를 기준으로 분류하였을 때, 0.3 미만인 문항이 11 문항으로 0.3 이상인 문항이 9 문항인 것에 비해 더 많이 나타남

(3) 작업치료학 난이도와 변별도 분포도 및 비율분석

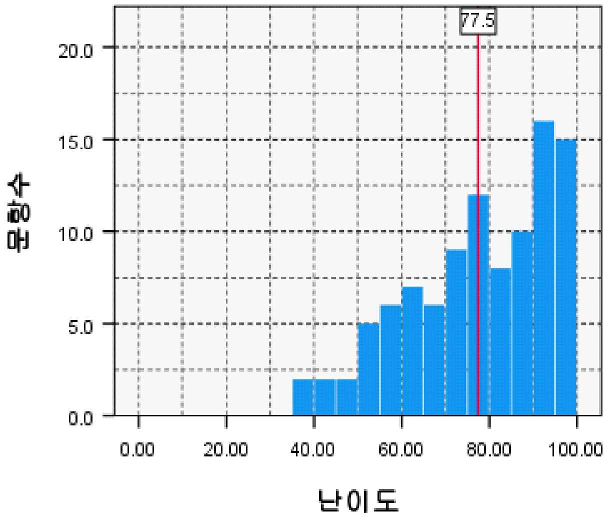

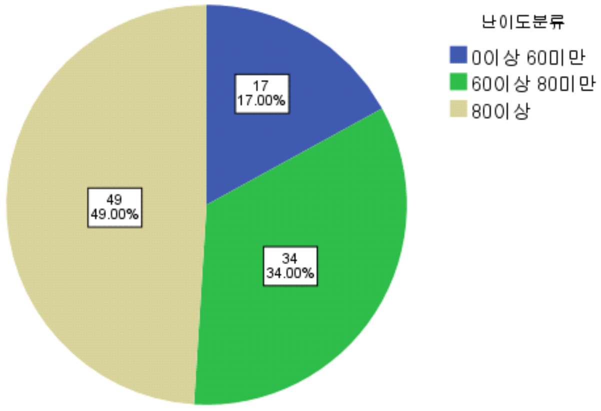

총점	난이도	표준편차
100	77.5	16.4

난이도	문항수	비율(%)
0~60미만	17	17.0
60~80미만	34	34.0
80~100	49	49.0
전체	100	100.0

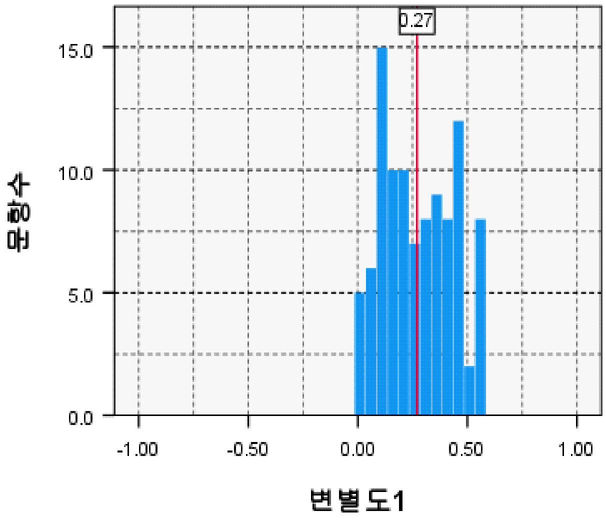

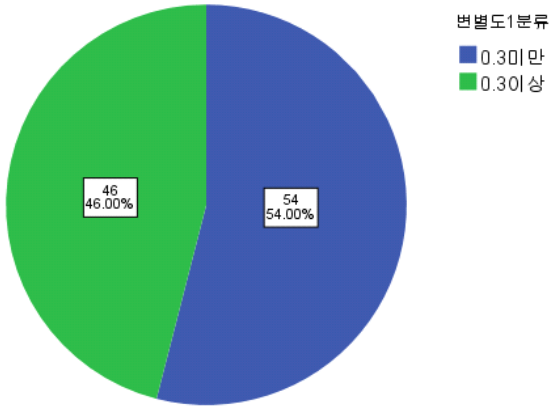

총점	변별도1	표준편차
100	.27	.16

변별도1	문항수	비율(%)
0.3미만	54	54.0
0.3이상	46	46.0
전체	100	100.0

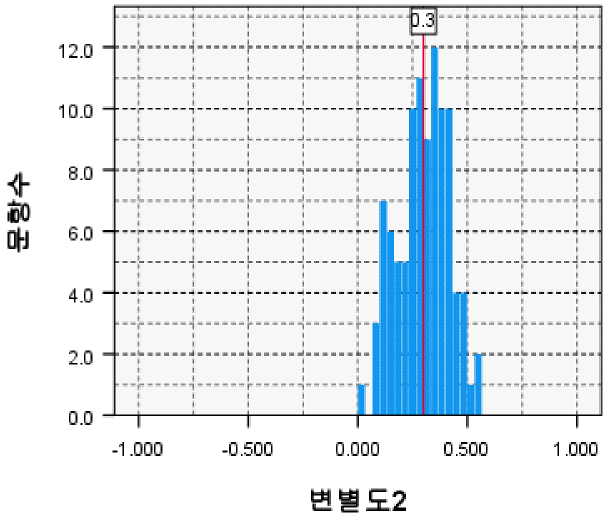

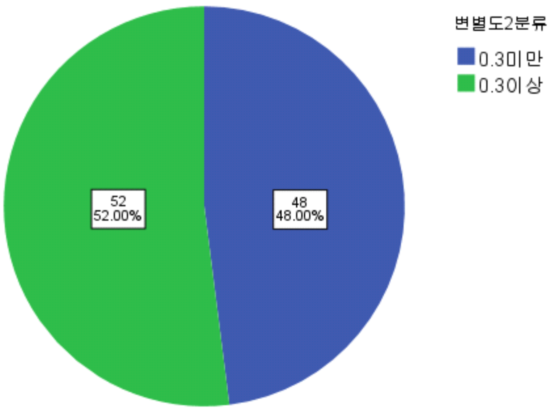

총점	변별도2	표준편차
100	.30	.12

변별도2	문항수	비율(%)
0.3미만	48	48.0
0.3이상	52	52.0
전체	100	100.0

해석

- 작업치료학 과목에서 난이도 지수가 80 에서 100 사이인 문항이 전체 100 문항 중 49 문항으로 가장 많았으며, 다음으로 60 이상 80 미만인 문항이 34 문항, 60 미만인 문항이 17 문항으로 나타남
- 변별도 1 지수를 기준으로 분류하였을 때, 0.3 미만인 문항이 54 문항으로 0.3 이상인 문항이 46 문항인 것에 비해 더 많이 나타남
- 변별도 2 지수를 기준으로 분류하였을 때, 0.3 미만인 문항이 48 문항으로 0.3 이상인 문항이 52 문항인 것에 비해 더 적게 나타남

(4) 실기시험 난이도와 변별도 분포도 및 비율분석

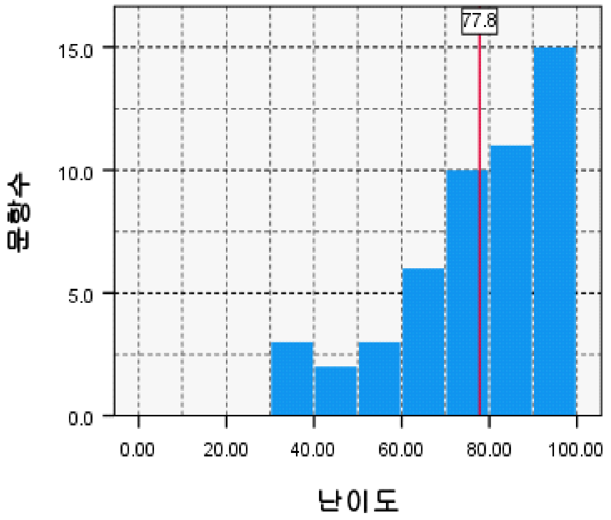

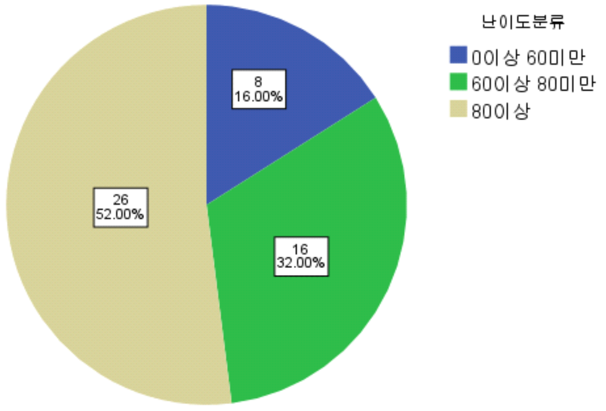

총점	난이도	표준편차	난이도	문항수	비율(%)
50	77.8	18.0	0~60미만	8	16.0
			60~80미만	16	32.0
			80~100	26	52.0
			전체	50	100.0

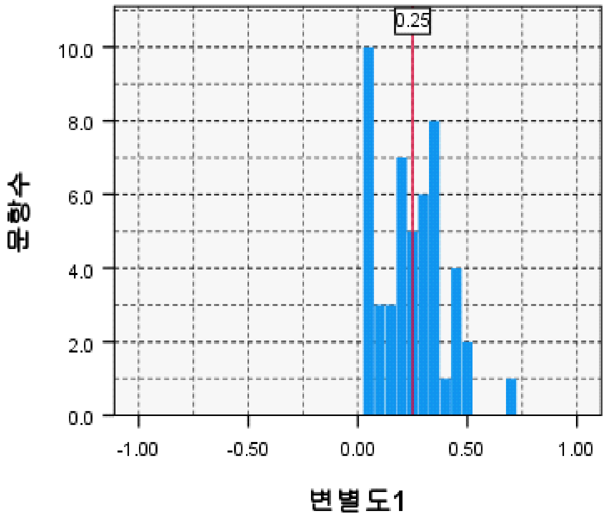

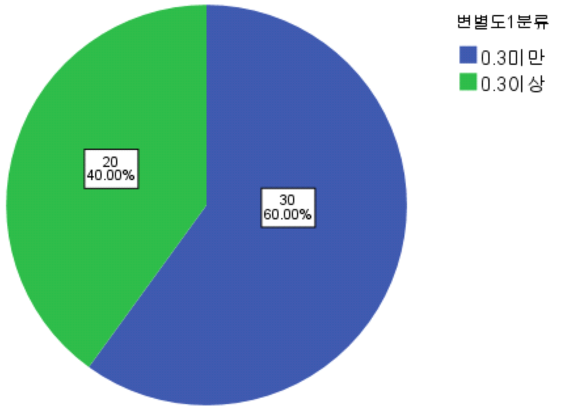

총점	변별도1	표준편차	변별도1	문항수	비율(%)
50	.25	.15	0.3미만	30	60.0
			0.3이상	20	40.0
			전체	50	100.0

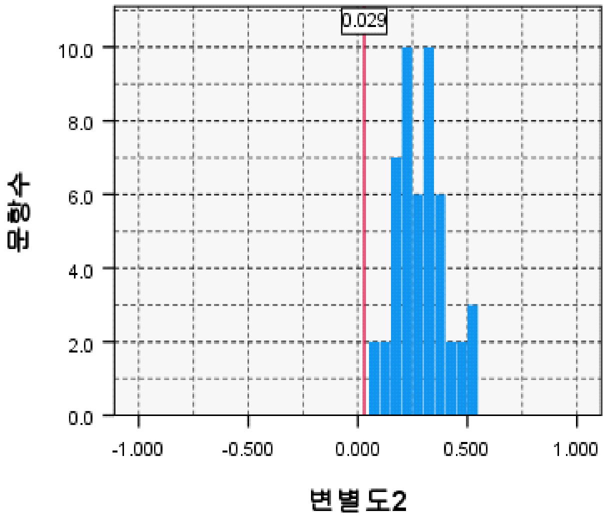

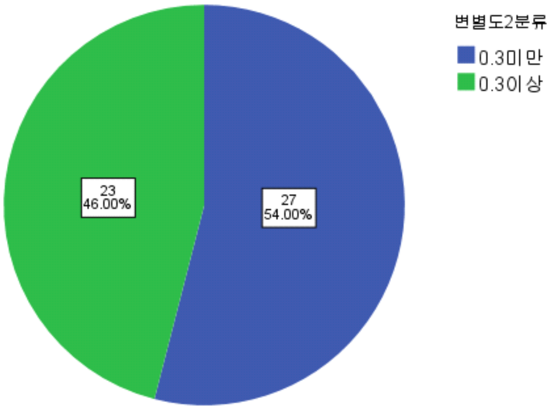

총점	변별도2	표준편차
50	.29	.11

변별도2	문항수	비율(%)
0.3미만	27	54.0
0.3이상	23	46.0
전체	50	100.0

해석

- 실기시험 과목에서 난이도 지수가 80에서 100 사이인 문항이 전체 50문항 중 26문항으로 가장 많았으며, 다음으로 60이상 80미만인 문항이 16문항, 60 미만인 문항이 8문항으로 나타남
- 변별도1 지수를 기준으로 분류하였을 때, 0.3미만인 문항이 30문항으로 0.3 이상인 문항이 20문항인 것에 비해 더 많이 나타남
- 변별도2 지수를 기준으로 분류하였을 때, 0.3미만인 문항이 27문항으로 0.3 이상인 문항이 23문항인 것에 비해 더 많이 나타남

3) 지식수준별 난이도와 변별도

가) 전회 대비 지식수준별 난이도와 변별도

(1) 전회 대비 암기형 난이도와 변별도

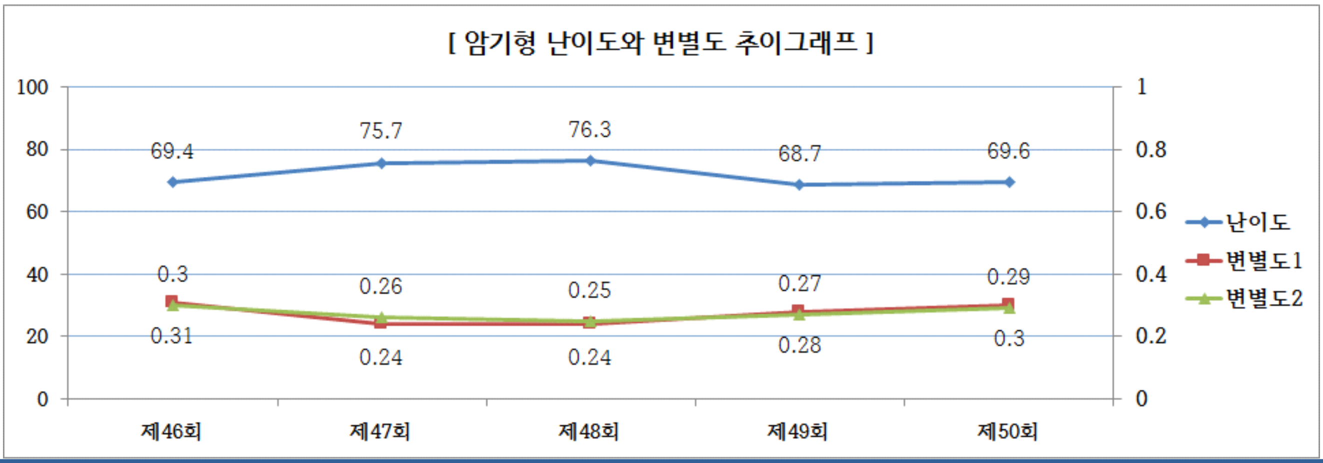

회차	난이도		변별도1		변별도2	
	평균	표준편차	평균	표준편차	평균	표준편차
제46회	69.4	19.2	.31	.12	.30	.10
제47회	75.7	17.9	.24	.12	.26	.10
제48회	76.3	18.9	.24	.13	.25	.09
제49회	68.7	21.0	.28	.13	.27	.09
**제50회**	**69.6**	**19.5**	**.30**	**.13**	**.29**	**.10**

해석

- 전회 대비 암기형 문항의 난이도 지수는 0.9 증가함
- 전회 대비 암기형 문항의 변별도 1 지수는 .02 증가함
- 전회 대비 암기형 문항의 변별도 2 지수는 .02 증가함

(2) 전회 대비 해석형 난이도와 변별도

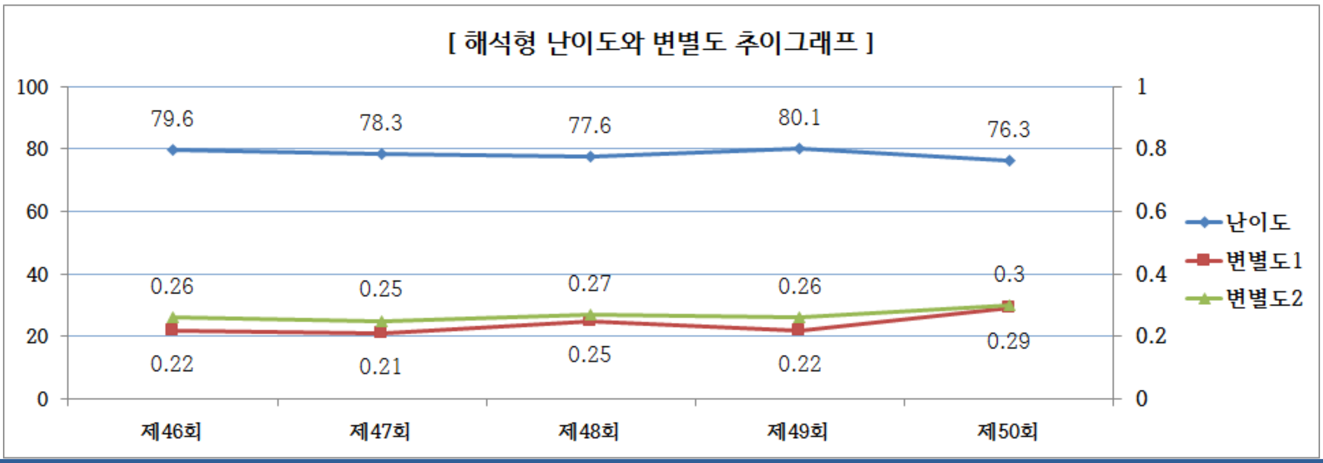

회차	난이도		변별도1		변별도2	
	평균	표준편차	평균	표준편차	평균	표준편차
제46회	79.6	16.3	.22	.13	.26	.11
제47회	78.3	20.0	.21	.13	.25	.11
제48회	77.6	16.7	.25	.13	.27	.10
제49회	80.1	18.7	.22	.15	.26	.12
**제50회**	**76.3**	**16.9**	**.29**	**.16**	**.30**	**.11**

해석

- 전회 대비 해석형 문항의 난이도 지수는 3.8 감소함
- 전회 대비 해석형 문항의 변별도 1 지수는 .07 증가함
- 전회 대비 해석형 문항의 변별도 2 지수는 .04 증가함

(3) 전회 대비 해결형 난이도와 변별도

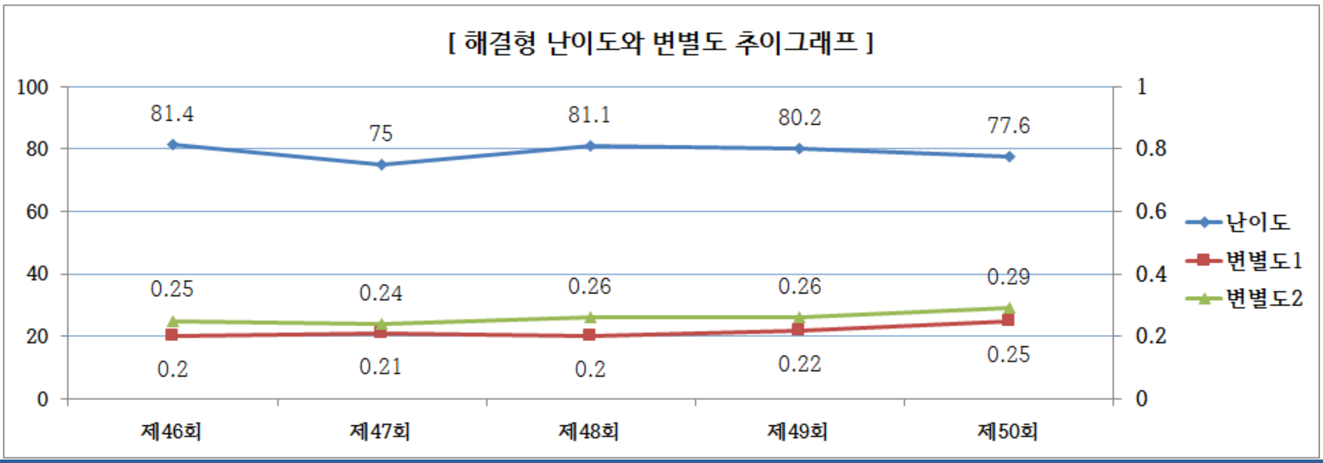

회차	난이도		변별도1		변별도2	
	평균	표준편차	평균	표준편차	평균	표준편차
제46회	81.4	15.7	.20	.13	.25	.12
제47회	75.0	21.6	.21	.13	.24	.11
제48회	81.1	18.2	.20	.13	.26	.11
제49회	80.2	16.9	.22	.13	.26	.10
**제50회**	**77.6**	**17.3**	**.25**	**.15**	**.29**	**.11**

해석

- 전회 대비 해결형 문항의 난이도 지수는 2.6 감소함
- 전회 대비 해결형 문항의 변별도1 지수는 .03 증가함
- 전회 대비 해결형 문항의 변별도2 지수는 .03 증가함

나) 지식수준별 난이도와 변별도 분포도 및 비율분석

(1) 암기형 난이도와 변별도 분포도 및 비율분석

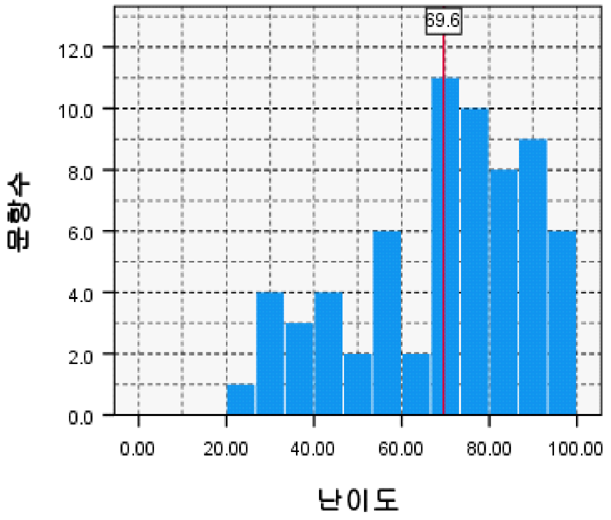

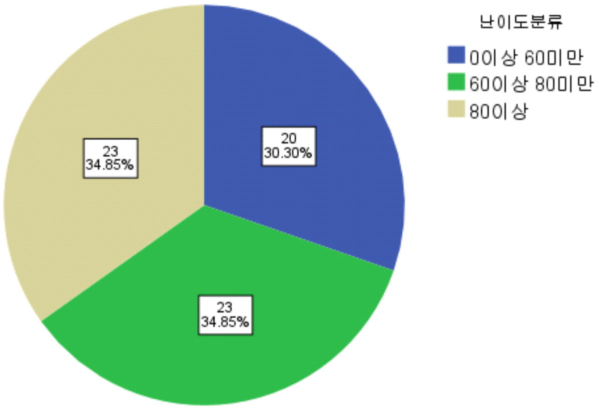

총점	난이도	표준편차	난이도	문항수	비율(%)
66	69.6	19.6	0~60미만	20	30.3
			60~80미만	23	34.8
			80~100	23	34.8
			전체	66	100.0

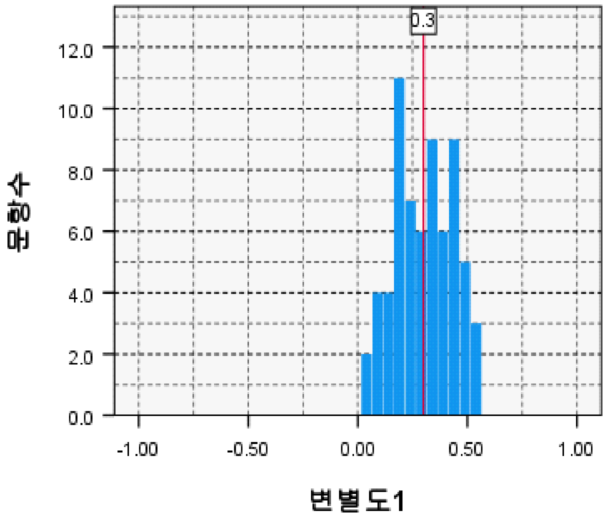

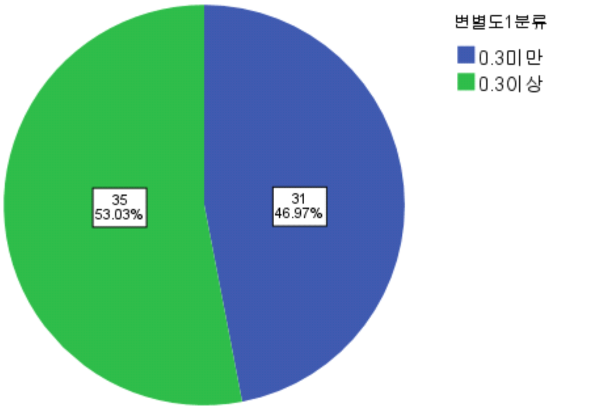

총점	변별도1	표준편차	변별도1	문항수	비율(%)
66	.30	.13	0.3미만	31	47.0
			0.3이상	35	53.0
			전체	66	100.0

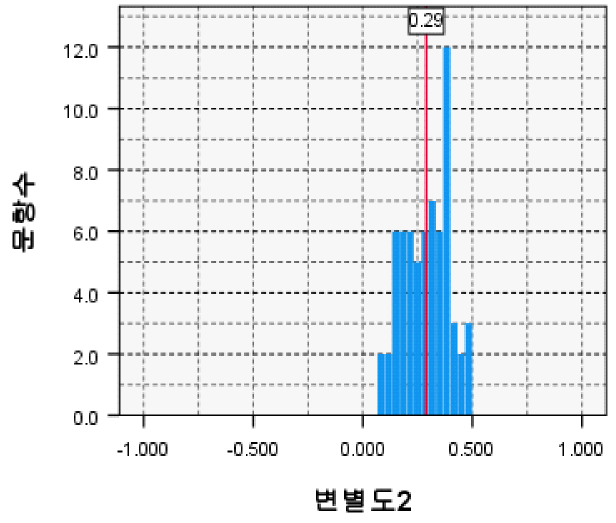

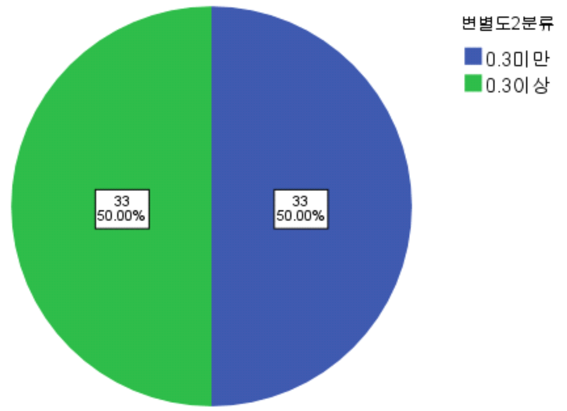

총점	변별도2	표준편차
66	.29	.11

변별도2	문항수	비율(%)
0.3미만	33	50.0
0.3이상	33	50.0
전체	66	100.0

해석

- 암기형 문항에서 난이도 지수가 80에서 100 사이인 문항이 전체 66문항 중 23문항, 60 이상 80 미만인 문항이 23문항으로 가장 많았으며, 다음으로, 60 미만인 문항이 20문항인 것으로 나타남
- 변별도 1 지수를 기준으로 분류하였을 때, 0.3 미만인 문항이 31문항으로 0.3 이상인 문항이 35문항인 것에 비해 더 적게 나타남
- 변별도 2 지수를 기준으로 분류하였을 때, 0.3 미만인 문항이 33문항으로 0.3 이상인 문항이 33문항인 것에 비해 같게 나타남

(2) 해석형 난이도와 변별도 분포도 및 비율분석

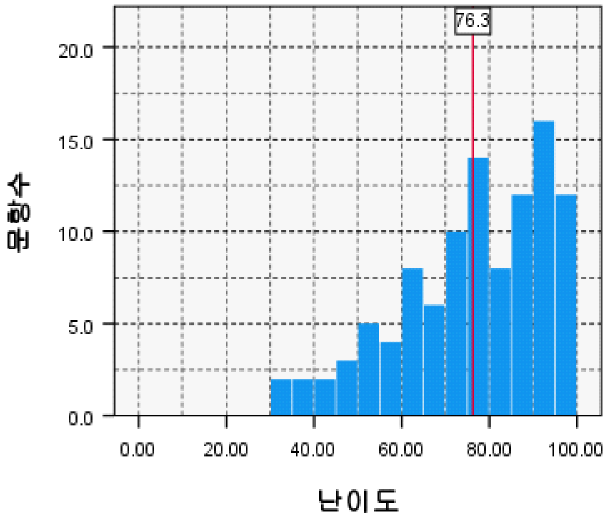

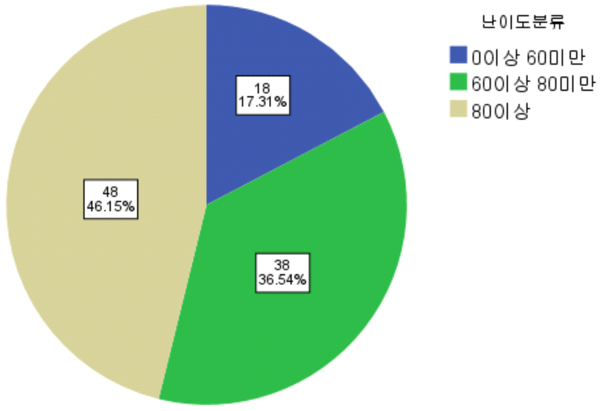

총점	난이도	표준편차
104	76.3	17.0

난이도	문항수	비율(%)
0~60미만	18	17.3
60~80미만	38	36.5
80~100	48	46.2
전체	104	100.0

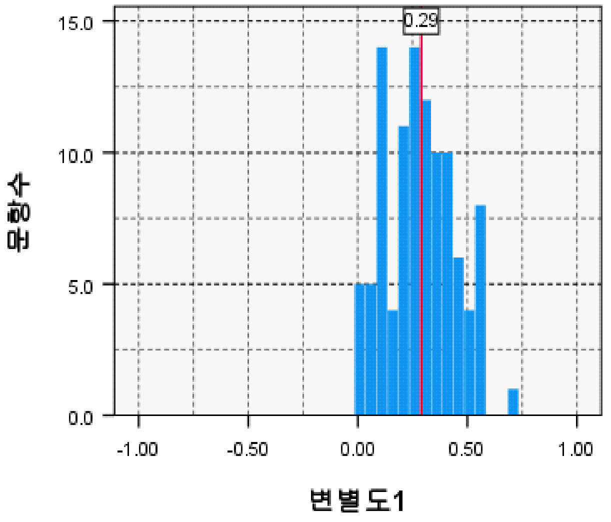

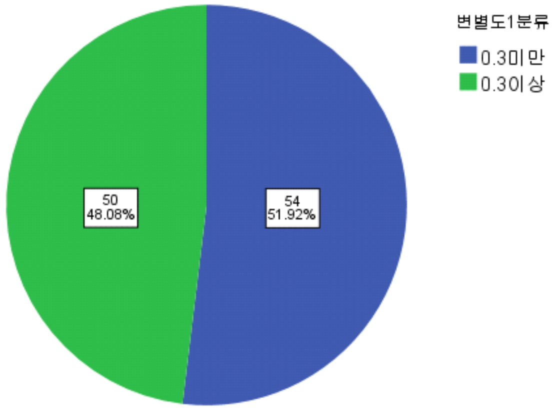

총점	변별도1	표준편차
104	.29	.16

변별도1	문항수	비율(%)
0.3미만	54	51.9
0.3이상	50	48.1
전체	104	100.0

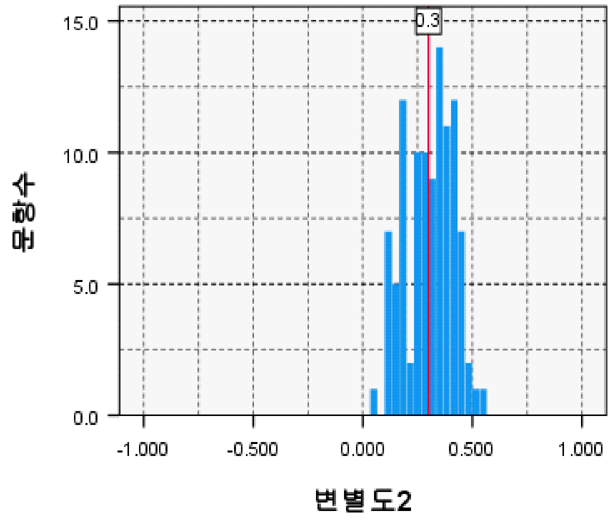

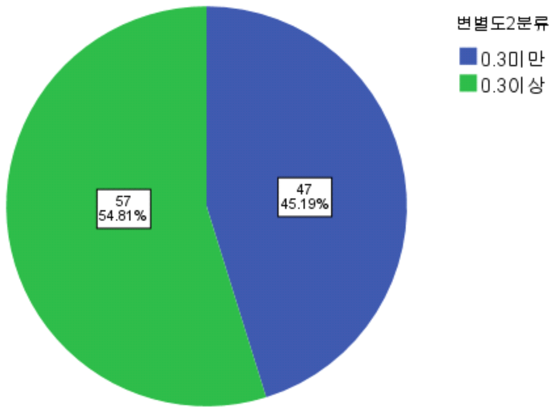

총점	변별도2	표준편차
104	.30	.11

변별도2	문항수	비율(%)
0.3미만	47	45.2
0.3이상	57	54.8
전체	104	100.0

해석

- 해석형 문항에서 난이도 지수가 80에서 100 사이인 문항이 전체 104문항 중 48문항으로 가장 많았으며, 다음으로 60이상 80미만인 문항이 38문항, 60미만인 문항이 18문항인 것으로 나타남
- 변별도1 지수를 기준으로 분류하였을 때, 0.3미만인 문항이 54문항으로 0.3이상인 문항이 50문항인 것에 비해 더 많이 나타남
- 변별도2 지수를 기준으로 분류하였을 때, 0.3미만인 문항이 47문항으로 0.3이상인 문항이 57문항인 것에 비해 더 적게 나타남

(3) 해결형 난이도와 변별도 분포도 및 비율분석

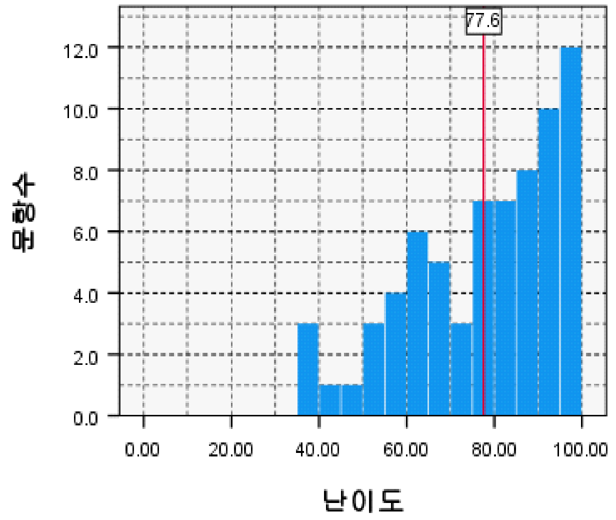

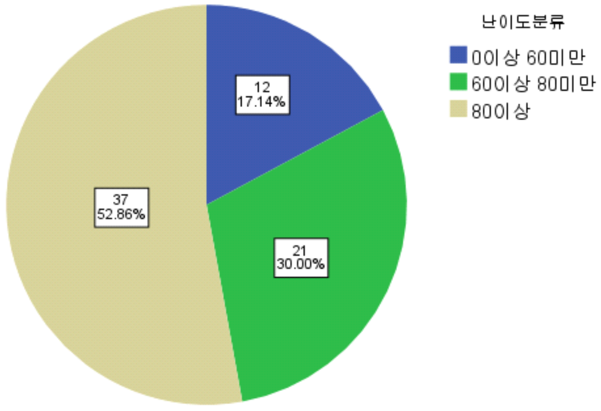

총점	난이도	표준편차
70	77.6	17.4

난이도	문항수	비율(%)
0~60미만	12	17.1
60~80미만	21	30.0
80~100	37	52.9
전체	70	100.0

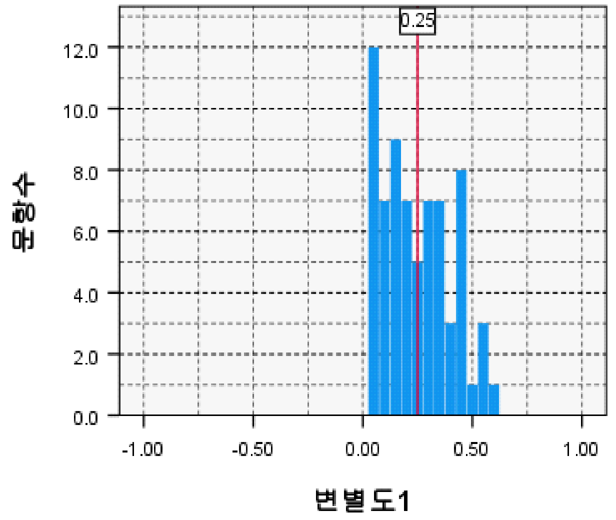

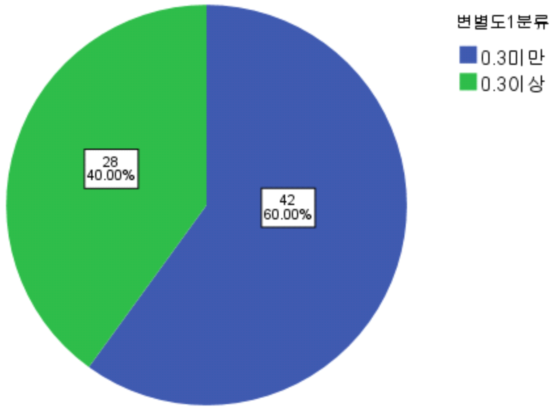

총점	변별도1	표준편차
70	.25	.15

변별도1	문항수	비율(%)
0.3미만	42	60.0
0.3이상	28	40.0
전체	70	100.0

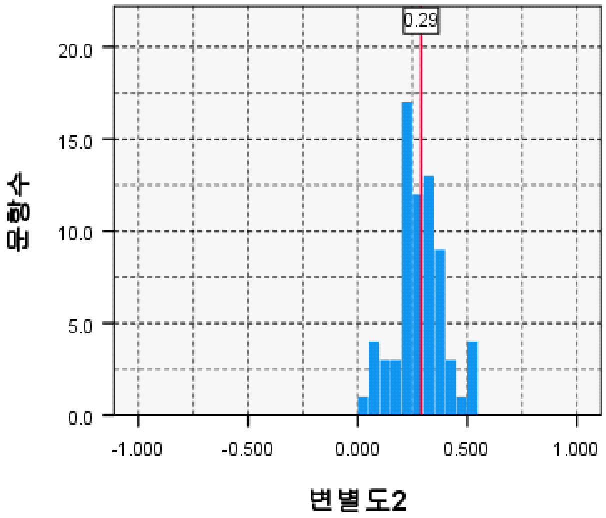

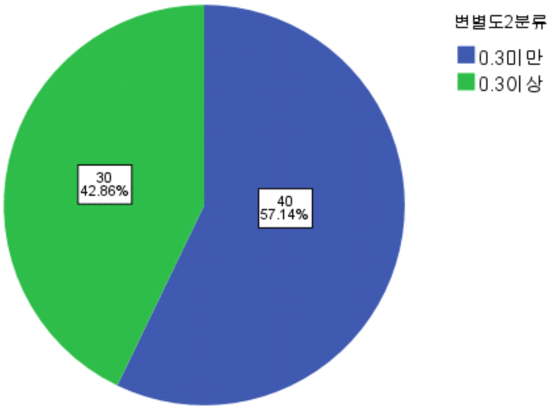

총점	변별도2	표준편차
70	.29	.11

변별도2	문항수	비율(%)
0.3미만	40	57.1
0.3이상	30	42.9
전체	70	100.0

해석

- 해결형 문항에서 난이도 지수가 80 에서 100 사이인 문항이 전체 70 문항 중 37 문항으로 가장 많았으며, 다음으로 60 이상 80 미만인 문항이 21 문항, 60 미만인 문항이 12 문항인 것으로 나타남
- 변별도 1 지수를 기준으로 하였을 때, 0.3 미만인 문항이 42 문항으로 0.3 이상인 문항이 28 문항인 것에 비해 더 많이 나타남
- 변별도 2 지수를 기준으로 분류하였을 때, 0.3 미만인 문항이 40 문항으로 0.3 이상인 문항이 30 문항인 것에 비해 더 많이 나타남

4) 자료유형별 난이도와 변별도

가) 전회 대비 자료유형별 난이도와 변별도

(1) 전회 대비 텍스트형 난이도와 변별도

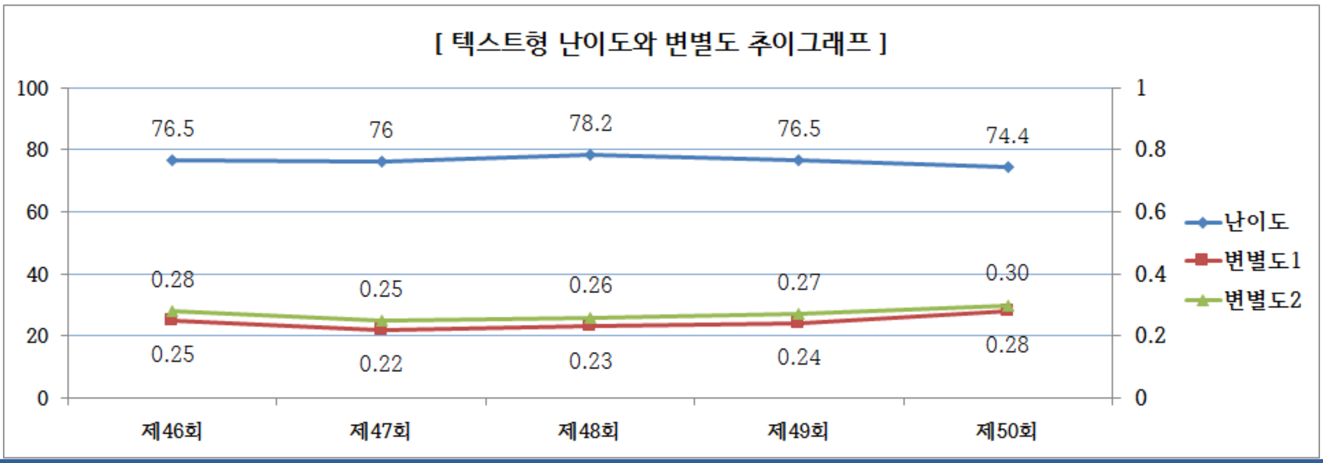

회차	난이도		변별도1		변별도2	
	평균	표준편차	평균	표준편차	평균	표준편차
제46회	76.5	18.0	.25	.14	.28	.11
제47회	76.0	20.1	.22	.13	.25	.11
제48회	78.2	18.2	.23	.14	.26	.10
제49회	76.5	19.7	.24	.14	.27	.11
**제50회**	**74.4**	**18.2**	**.28**	**.15**	**.30**	**.11**

해석

- 전회 대비 텍스트형 문항의 난이도 지수는 2.1 감소함
- 전회 대비 텍스트형 문항의 변별도 1 지수는 .04 증가함
- 전회 대비 텍스트형 문항의 변별도 2 지수는 .03 증가함

(2) 전회 대비 자료제시형 난이도와 변별도

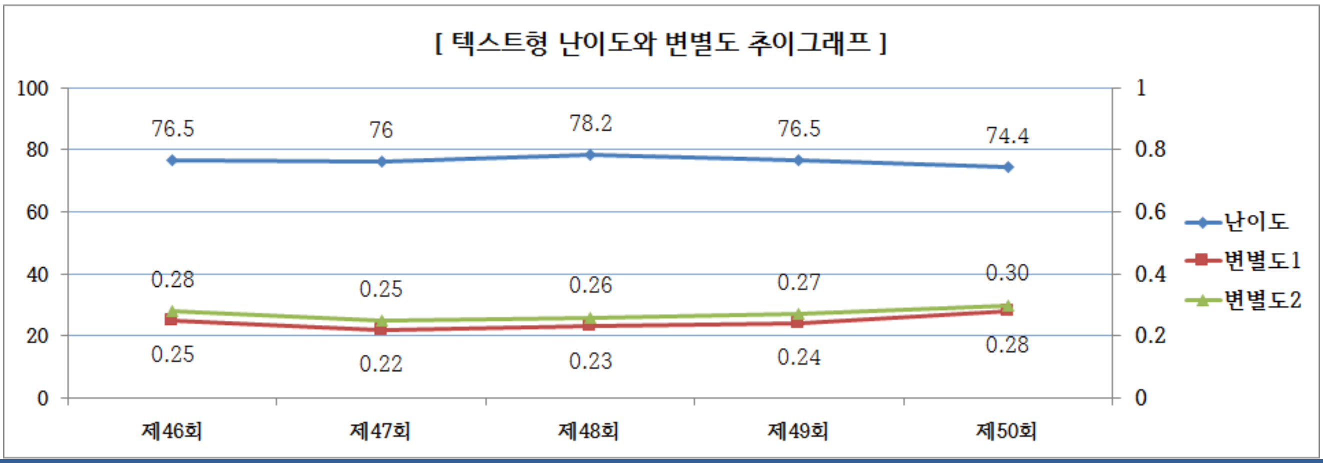

회차	난이도		변별도1		변별도2	
	평균	표준편차	평균	표준편차	평균	표준편차
제46회	78.9	16.9	.21	.14	.23	.10
제47회	87.1	13.9	.16	.13	.22	.12
제48회	83.2	13.6	.24	.10	.32	.10
제49회	79.2	14.9	.25	.13	.29	.12
**제50회**	**84.4**	**10.0**	**.23**	**.11**	**.28**	**.09**

해석

- 전회 대비 자료제시형 문항의 난이도 지수는 5.2 증가함
- 전회 대비 자료제시형 문항의 변별도 1 지수는 .02 감소함
- 전회 대비 자료제시형 문항의 변별도 2 지수는 .01 감소함

나) 자료유형별 난이도와 변별도 분포도 및 비율분석

(1) 텍스트형 난이도와 변별도 분포도 및 비율분석

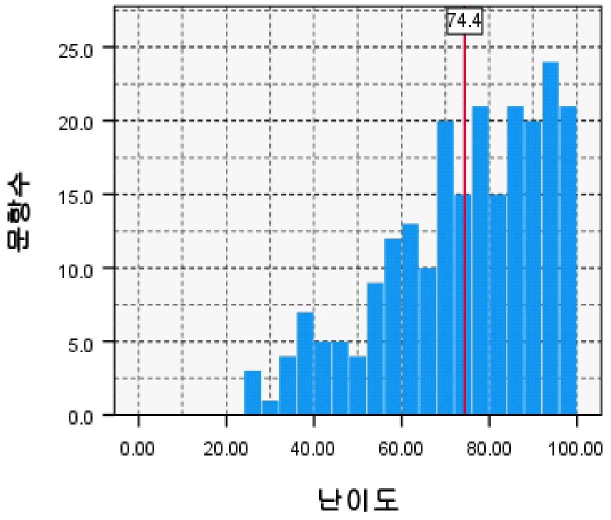

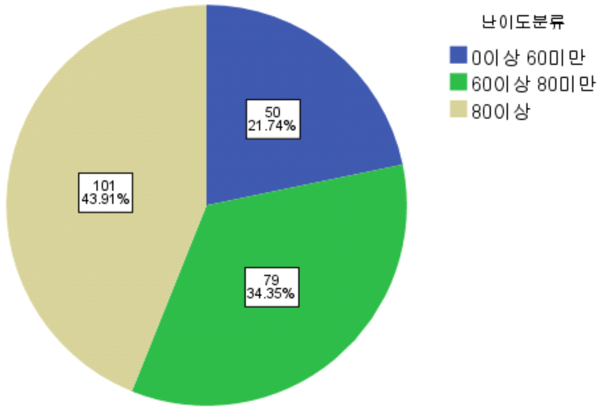

총점	난이도	표준편차
230	74.4	18.2

난이도	문항수	비율(%)
0~60미만	50	21.7
60~80미만	79	34.3
80~100	101	43.9
전체	230	100.0

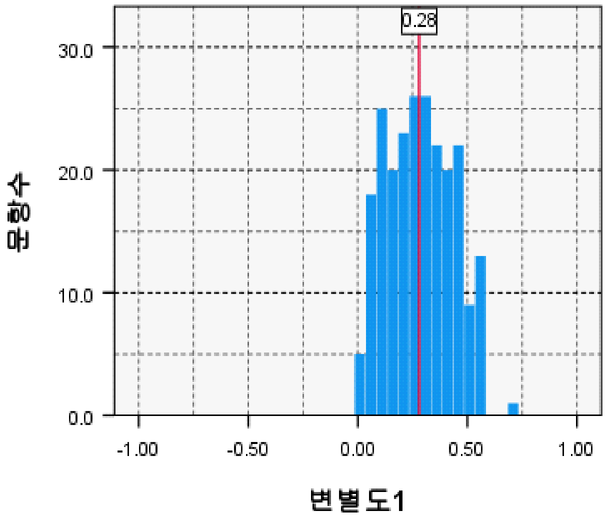

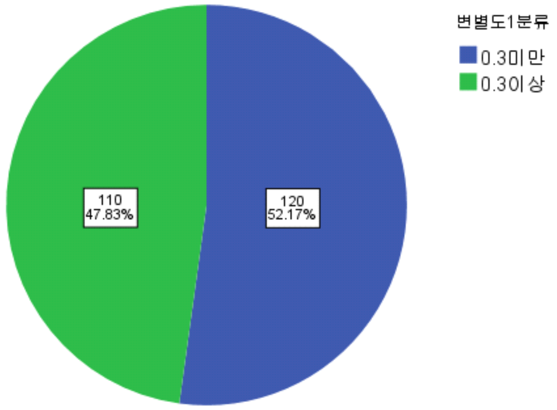

총점	변별도1	표준편차
230	.28	.15

변별도1	문항수	비율(%)
0.3미만	120	52.2
0.3이상	110	47.8
전체	230	100.0

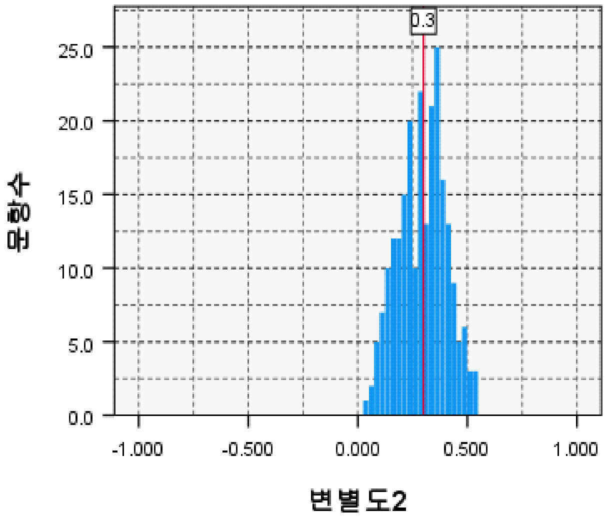

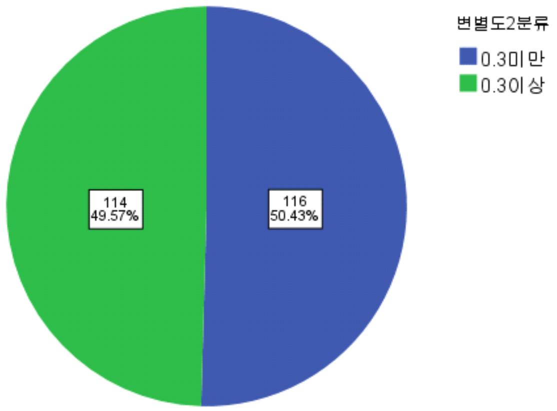

총점	변별도2	표준편차
230	.30	.11

변별도2	문항수	비율(%)
0.3미만	116	50.4
0.3이상	114	49.6
전체	230	100.0

해석

- 텍스트형 문항에서 난이도 지수가 80에서 100 사이인 문항이 전체 230문항 중 101문항으로 가장 많았으며, 다음으로 60 이상 80 미만인 문항이 79문항, 60 미만인 문항이 50문항인 것으로 나타남
- 변별도 1 지수를 기준으로 분류하였을 때, 0.3 미만인 문항이 120문항으로 0.3 이상인 문항이 110문항인 것에 비해 더 많이 나타남
- 변별도 2 지수를 기준으로 분류하였을 때, 0.3 미만인 문항이 116문항으로 0.3 이상인 문항이 114문항인 것에 비해 더 많이 나타남

(2) 자료제시형 난이도와 변별도 분포도 및 비율분석

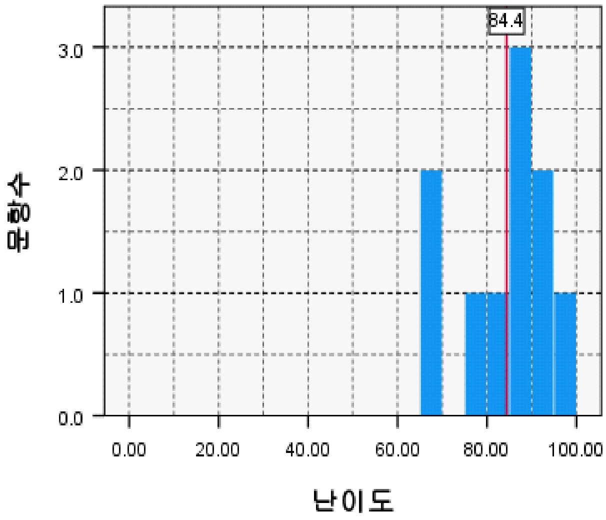

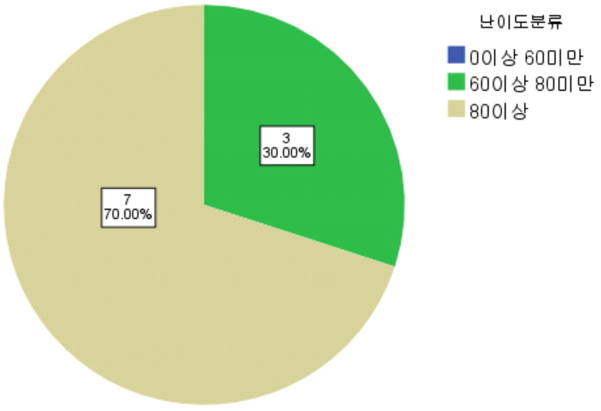

총점	난이도	표준편차
10	84.4	10.6

난이도	문항수	비율(%)
0~60미만	-	-
60~80미만	3	30.0
80~100	7	70.0
전체	10	100.0

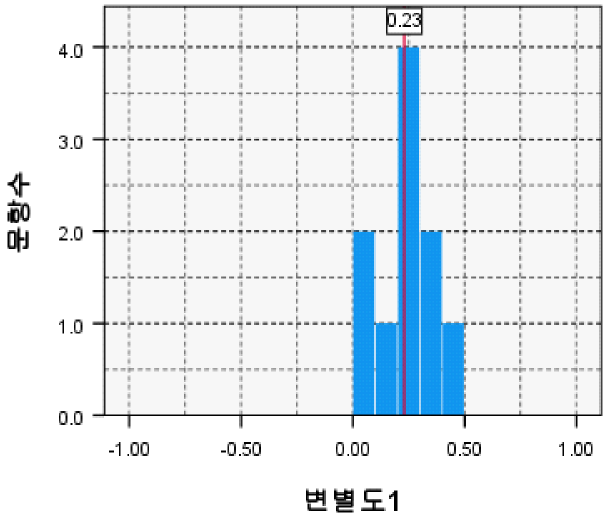

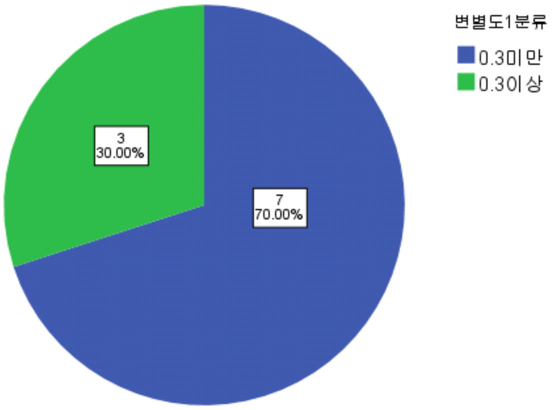

총점	변별도1	표준편차
10	.23	.12

변별도1	문항수	비율(%)
0.3미만	7	70.0
0.3이상	3	30.0
전체	10	100.0

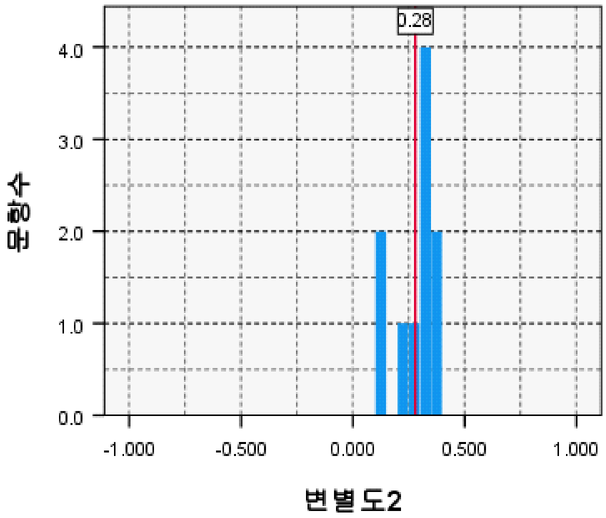

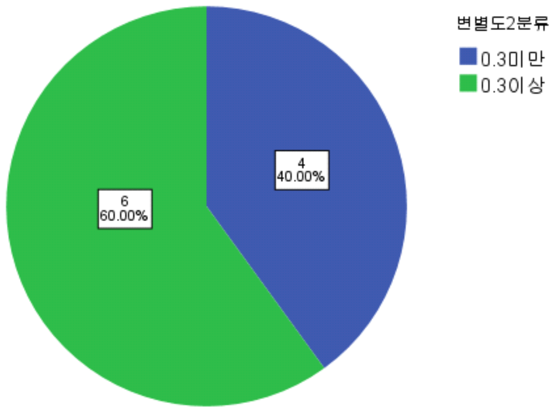

총점	변별도2	표준편차
10	.28	.10

변별도2	문항수	비율(%)
0.3미만	4	40.0
0.3이상	6	60.0
전체	10	100.0

해석

- 자료제시형 문항에서 난이도 지수가 80 에서 100 사이인 문항이 전체 10 문항 중 7 문항으로 가장 많았으며, 다음으로 60 이상 80 미만인 문항이 3 문항, 60 미만인 문항이 0 문항인 것으로 나타남
- 변별도 1 지수를 기준으로 분류하였을 때, 0.3 미만인 문항이 7 문항으로 0.3 이상인 문항이 3 문항인 것에 비해 더 많이 나타남
- 변별도 2 지수를 기준으로 분류하였을 때, 0.3 미만인 문항이 4 문항으로 0.3 이상인 문항이 6 문항인 것에 비해 더 적게 나타남

5) 문항형태별 난이도와 변별도

가) 전회 대비 문항형태별 난이도와 변별도

(1) 전회 대비 A형 난이도와 변별도

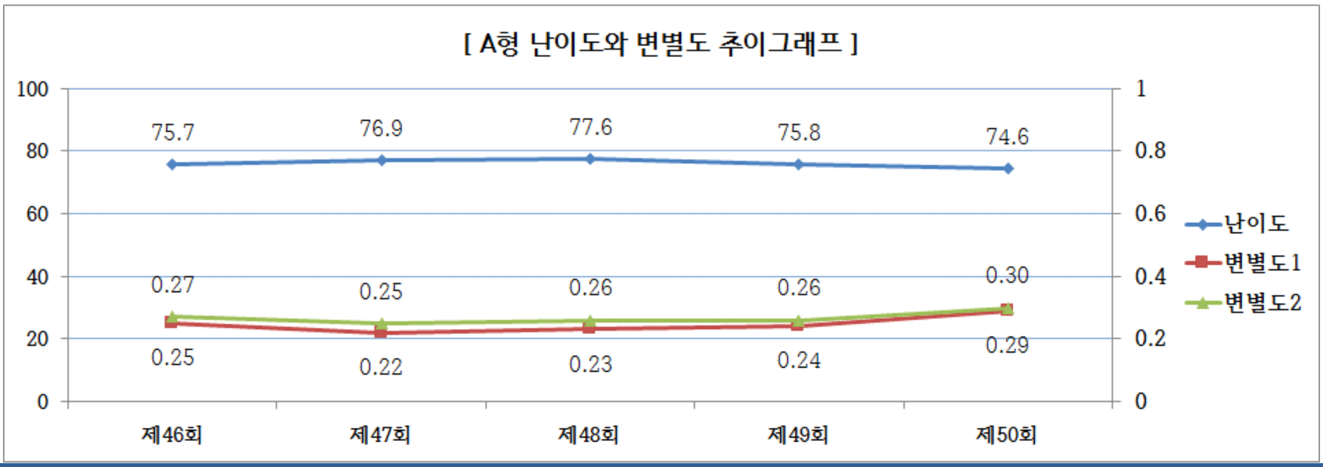

회차	난이도		변별도1		변별도2	
	평균	표준편차	평균	표준편차	평균	표준편차
제46회	75.7	18.5	.25	.14	.27	.11
제47회	76.9	19.6	.22	.12	.25	.10
제48회	77.6	18.4	.23	.14	.26	.10
제49회	75.8	20.1	.24	.14	.26	.11
**제50회**	**74.6**	**17.9**	**.29**	**.15**	**.30**	**.11**

해석

- 전회 대비 A 형 문항의 난이도 지수는 1.2 감소함
- 전회 대비 A 형 문항의 변별도 1 지수는 .05 증가함
- 전회 대비 A 형 문항의 변별도 2 지수는 .04 증가함

(2) 전회 대비 사례형 난이도와 변별도

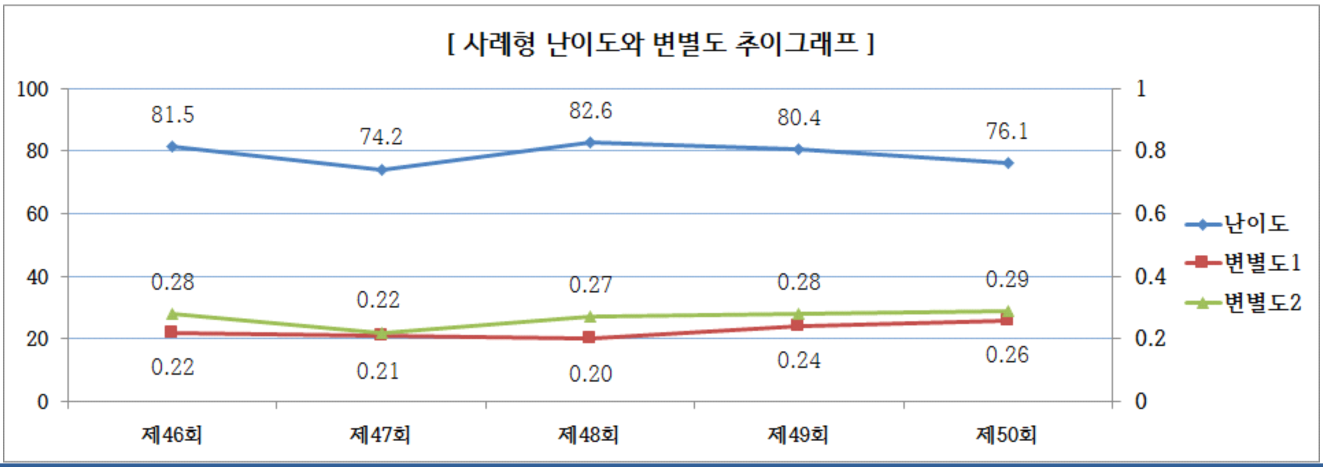

회차	난이도		변별도1		변별도2	
	평균	표준편차	평균	표준편차	평균	표준편차
제46회	81.5	13.9	.22	.13	.28	.12
제47회	74.2	21.9	.21	.15	.22	.13
제48회	82.6	15.5	.20	.12	.27	.10
제49회	80.4	15.9	.24	.14	.28	.11
**제50회**	**76.1**	**18.9**	**.26**	**.15**	**.29**	**.12**

해석

- 전회 대비 사례형 문항의 난이도 지수는 4.3 감소함
- 전회 대비 사례형 문항의 변별도 1 지수는 .02 증가함
- 전회 대비 사례형 문항의 변별도 2 지수는 .01 증가함

나) 문항형태별 난이도와 변별도 분포도 및 비율분석

(1) A형 난이도와 변별도 분포도 및 비율분석

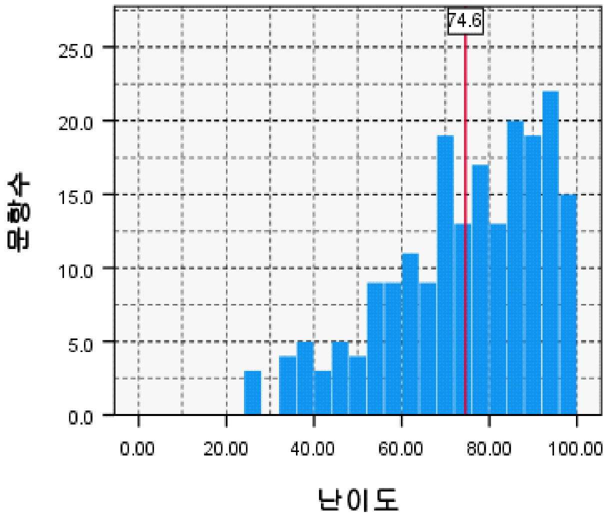

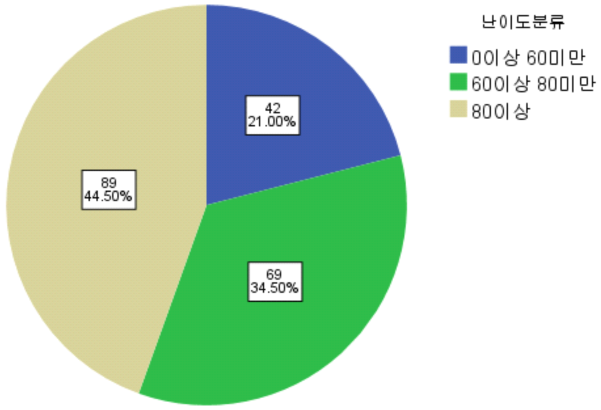

총점	난이도	표준편차
200	74.6	17.9

난이도	문항수	비율(%)
0~60미만	42	21.0
60~80미만	69	34.5
80~100	89	44.5
전체	200	100.0

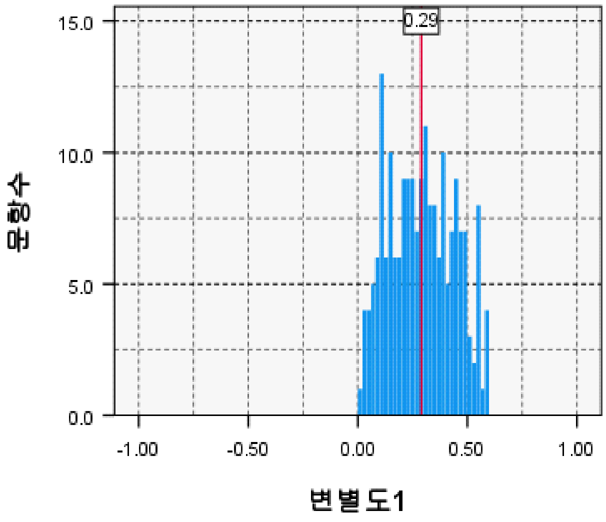

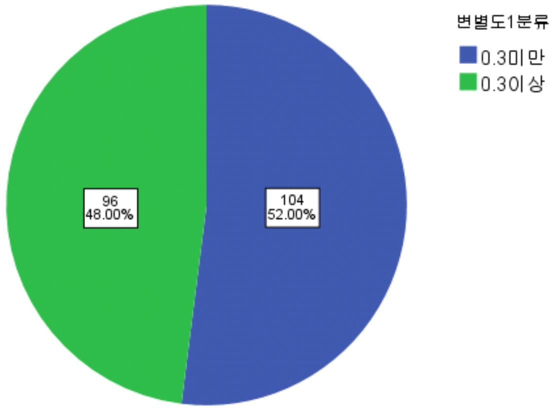

총점	변별도1	표준편차
200	.29	.15

변별도1	문항수	비율(%)
0.3미만	104	52.0
0.3이상	96	48.0
전체	200	100.0

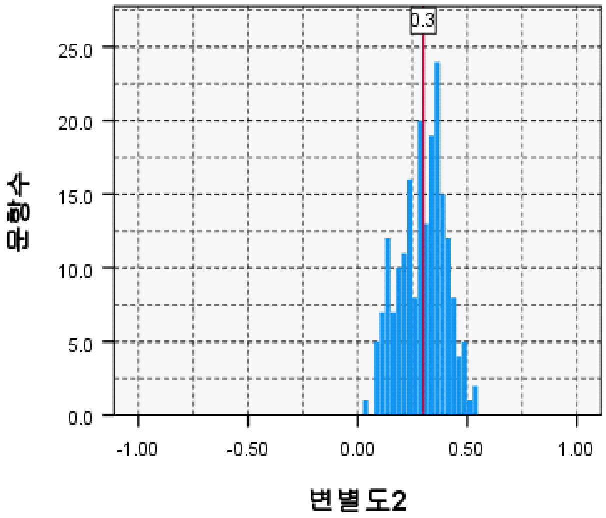

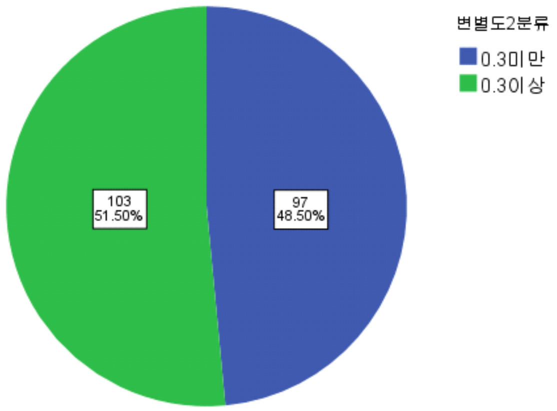

총점	변별도2	표준편차
200	.30	.11

변별도2	문항수	비율(%)
0.3미만	97	48.5
0.3이상	103	51.5
전체	200	100.0

해석

- A형 문항에서 난이도 지수가 80에서 100 사이인 문항이 전체 200문항 중 89문항으로 가장 많았으며, 다음으로 60이상 80미만인 문항이 69문항, 60미만인 문항이 42문항인 것으로 나타남
- 변별도1 지수를 기준으로 분류하였을 때, 0.3 미만인 문항이 104문항으로 0.3 이상인 문항이 96문항인 것에 비해 더 많이 나타남
- 변별도2 지수를 기준으로 분류하였을 때, 0.3미만인 문항이 97문항으로 0.3 이상인 문항이 103문항인 것에 비해 더 적게 나타남

(2) 사례형 난이도와 변별도 분포도 및 비율분석

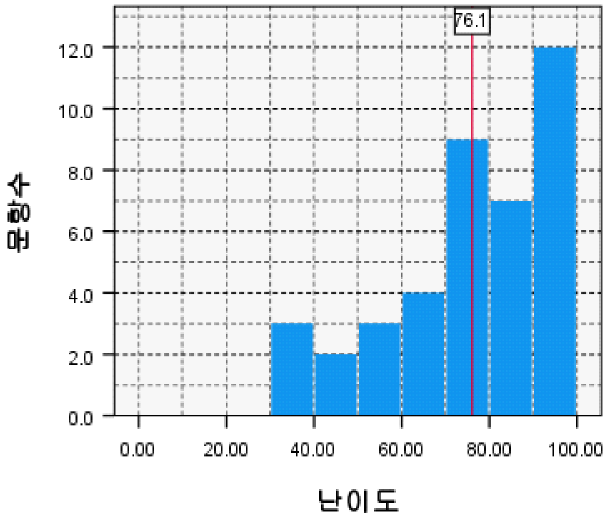

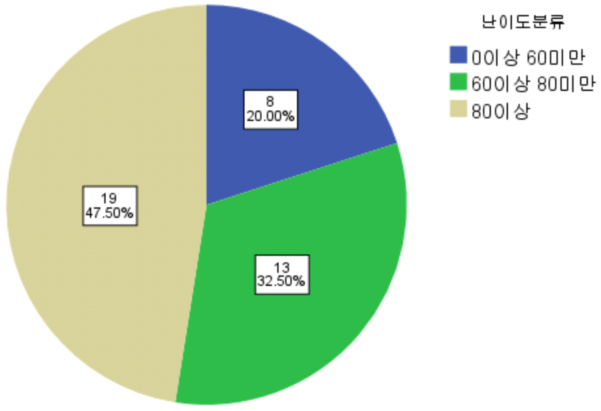

총점	난이도	표준편차
40	76.1	19.2

난이도	문항수	비율(%)
0~60미만	8	20.0
60~80미만	13	32.5
80~100	19	47.5
전체	40	100.0

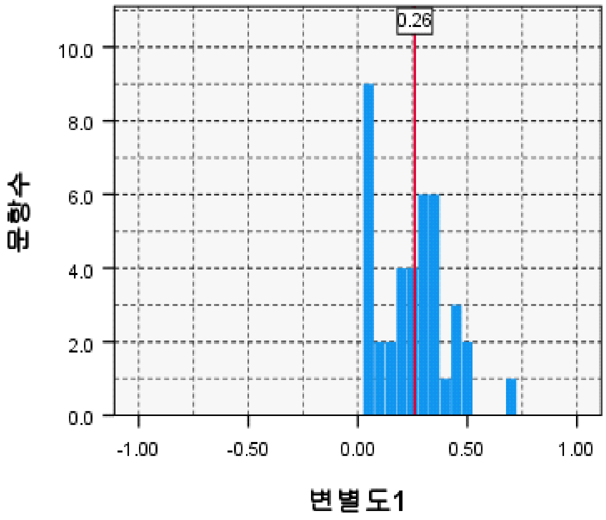

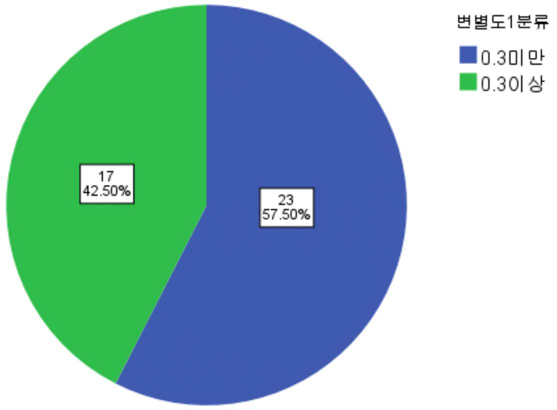

총점	변별도1	표준편차
40	.26	.16

변별도1	문항수	비율(%)
0.3미만	23	57.5
0.3이상	17	42.5
전체	40	100.0

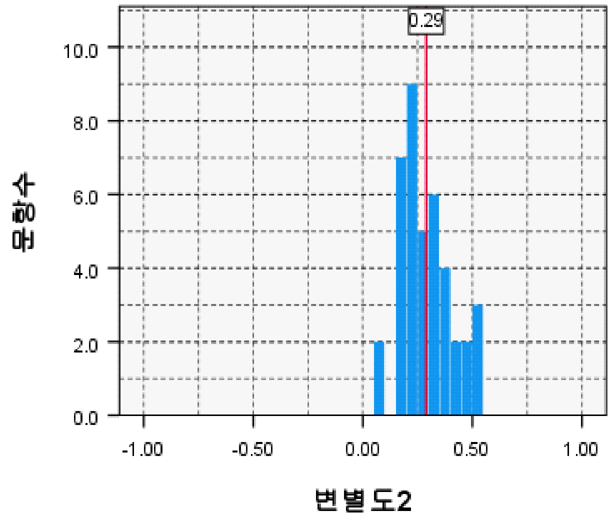

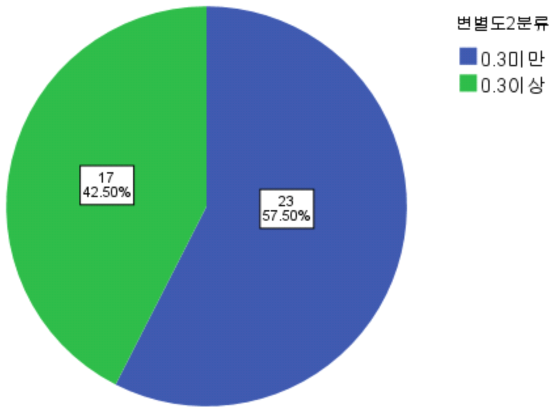

총점	변별도2	표준편차
40	.29	.12

변별도2	문항수	비율(%)
0.3미만	23	57.5
0.3이상	17	42.5
전체	40	100.0

해석

- 사례형 문항에서 난이도 지수가 80에서 100 사이인 문항이 전체 40문항 중 19문항으로 가장 많았으며, 다음으로 60이상 80미만인 문항이 13문항, 60미만인 문항이 8문항인 것으로 나타남
- 변별도1 지수를 기준으로 분류하였을 때, 0.3 미만인 문항이 23문항으로 0.3 이상인 문항이 17문항인 것에 비해 더 많이 나타남
- 변별도2 지수를 기준으로 분류하였을 때, 0.3 미만인 문항이 23문항으로 0.3 이상인 문항이 17문항인 것에 비해 더 많이 나타남

3. 난이도와 변별도 간 산포도

1) 전체 난이도와 변별도 간 산포도

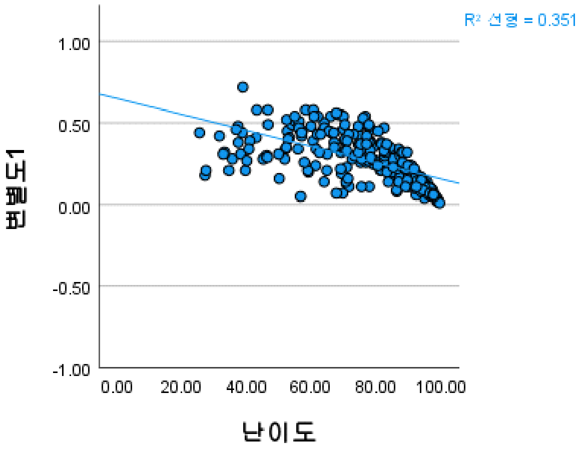

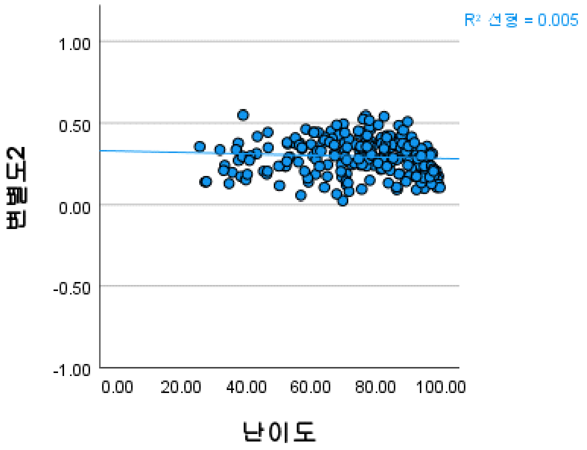

해석

- 전체 문항을 대상으로 난이도와 변별도1 지수 간 상관은 -.593*로 문항 난이도가 낮을수록 변별력이 낮아지는 것으로 나타남
- 난이도와 변별도2 지수 간 상관은 -.071으로 문항 난이도와 변별력 간 관련성이 없는 것으로 나타남

2) 과목별 난이도와 변별도 간 산포도

가) 작업치료학 기초 난이도와 변별도 간 산포도

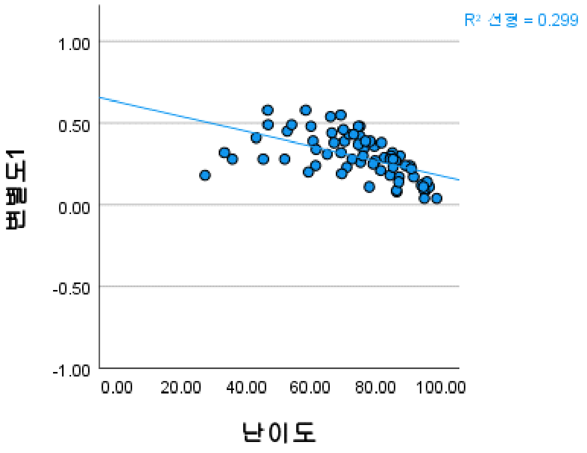

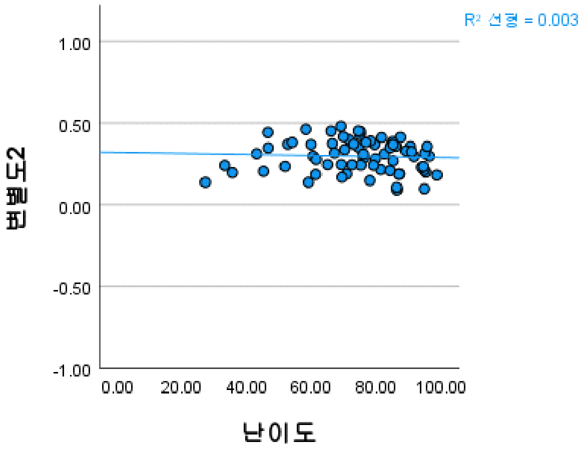

해석
- 작업치료학 기초 과목 문항을 대상으로 난이도와 변별도 1 지수 간 상관은 -.547*로 문항 난이도가 낮을수록 변별력이 낮아지는 것으로 나타남<br>- 난이도와 변별도 2 지수 간 상관은 -.053 으로 문항 난이도와 변별력 간 관련성이 없는 것으로 나타남

나) 의료관계법규 난이도와 변별도 간 산포도

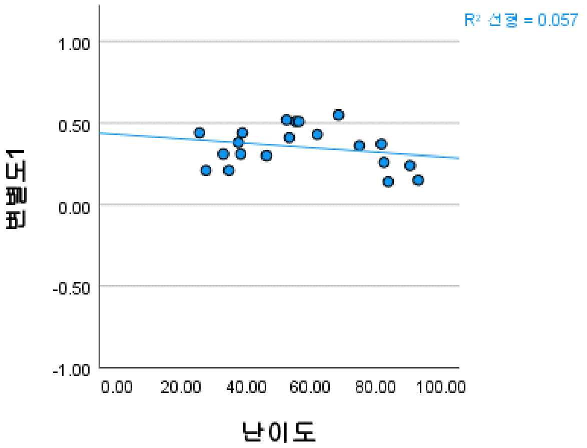

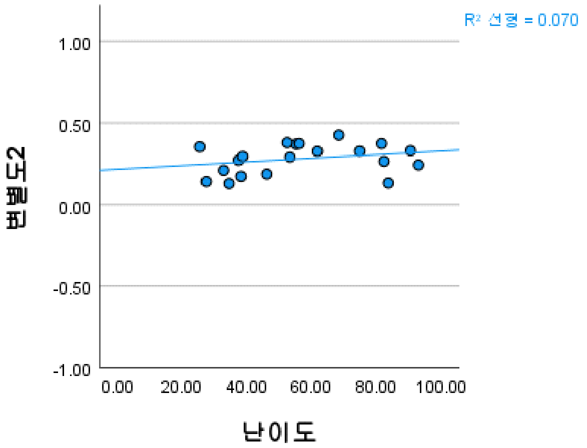

해석
- 의료관계법규 과목 문항을 대상으로 난이도와 변별도 1 지수 간 상관은 -.240*으로 문항 난이도와 변별력 간 관련성이 낮은 것으로 나타남<br>- 난이도와 변별도 2 지수 간 상관은 .265 으로 문항 난이도와 변별력 간 관련성이 낮은 것으로 나타남

다) 작업치료학 난이도와 변별도 간 산포도

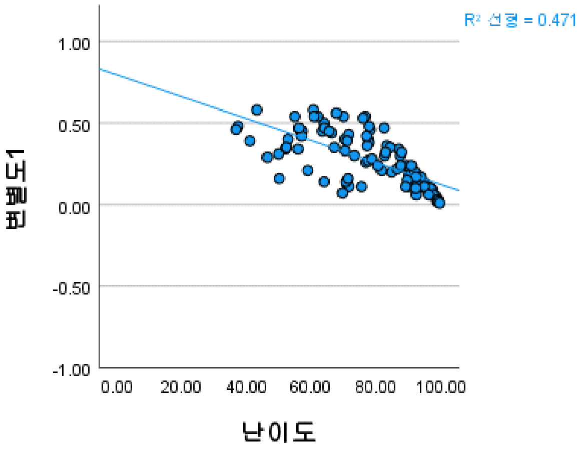

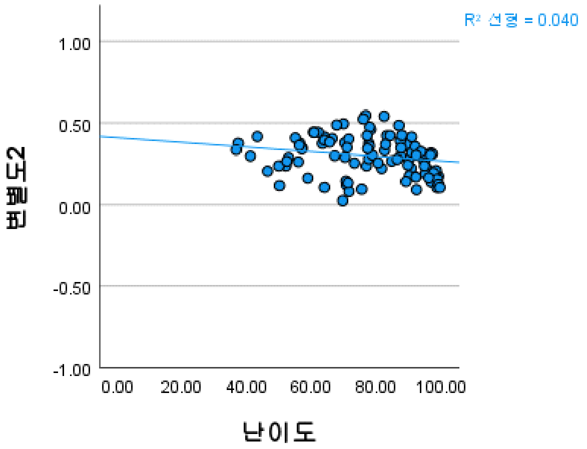

해석

- 작업치료학 과목 문항을 대상으로 난이도와 변별도 1 지수 간 상관은 -.686* 로 문항 난이도가 낮을수록 변별력이 낮아지는 것으로 나타남
- 난이도와 변별도 2 지수 간 상관은 -.200 로 문항 난이도와 변별력 간 관련성이 낮은 것으로 나타남

라) 실기시험 난이도와 변별도 간 산포도

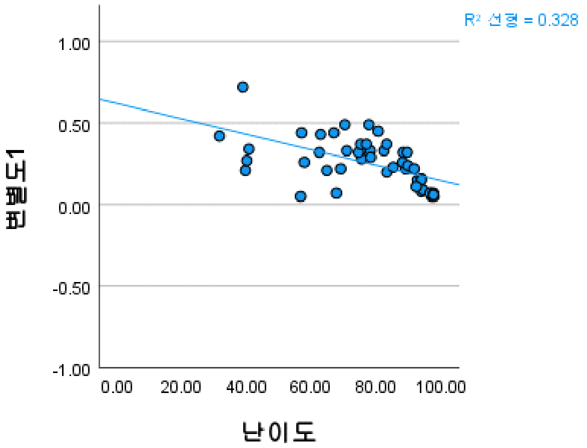

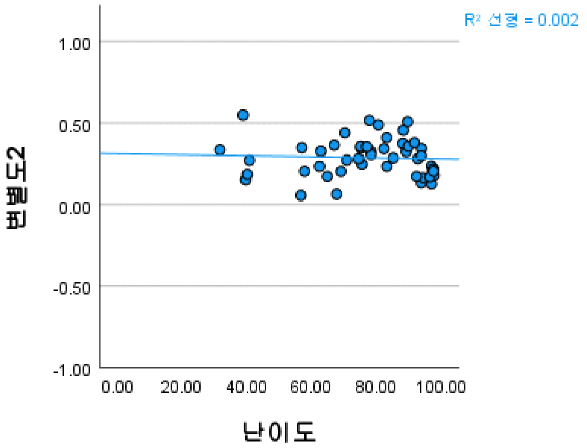

해석

- 실기시험 과목 문항을 대상으로 난이도와 변별도 1 지수 간 상관은 -.573*으로 문항 난이도가 낮을수록 변별력이 낮아지는 것으로 나타남
- 난이도와 변별도 2 지수 간 상관은 .050 으로 문항 난이도와 변별력 간 관련성이 없는 것으로 나타남

4. 신뢰도 분석

과목명	문항수	제46회	제47회	제48회	제49회	제50회
전체	240	.950	.940	.945	.949	**.959**
작업치료학 기초	70	.859	.850	.839	.848	**.878**
의료관계법규	20	.704	.632	.576	.609	**.696**
작업치료학	100	.888	.864	.883	.888	**.910**
실기시험	50	.791	.734	.798	.816	**.819**

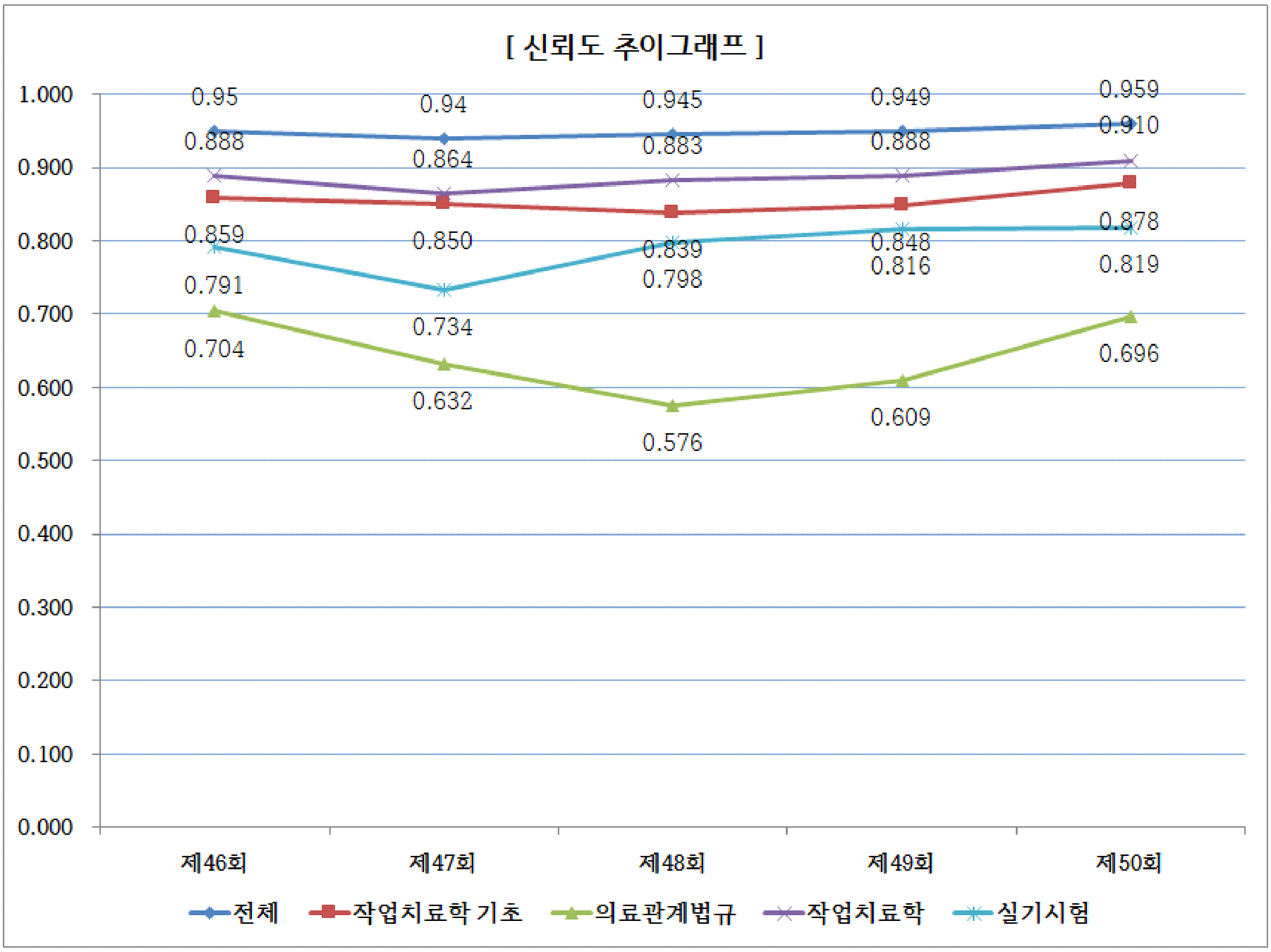

해석

- 전회 대비 전체문항의 신뢰도는 .010 증가함
- 전회 대비 작업치료학 기초 과목 문항의 신뢰도는 .030 증가함
- 전회 대비 의료관계법규 과목 문항의 신뢰도는 .087 증가함
- 전회 대비 작업치료학 과목 문항의 신뢰도는 .022 증가함
- 전회 대비 실기시험 과목 문항의 신뢰도는 .003 증가함

○ 분석결과 관련 문의 : 한국보건의료인국가시험원 연구개발본부 배상영 책임연구원
Tel : 02-2087-8955, FAX : 02-2087-8885
E-mail : bsy0601@kuksiwon.or.kr
