# Supplementary material for: Item difficulty index, discrimination index, and reliability of the 26 health professions licensing examinations in 2022, Korea: a psychometric study
Source: J Educ Eval Health Prof. 2023 Nov 22;20:31. doi: 10.3352/jeehp.2023.20.31 (PMC11959405; doi:10.3352/jeehp.2023.20.31)
Supplement: Supplementary file 1 — Supplement 1. Item analysis results of 26 health professions licensing examinations administered during late 2022 and early 2023. [file jeehp-20-31_Suppl1.zip › 2022│Γ╡╡ ┴a50╚╕ ─í░·▒Γ░°╗τ ▒╣░í╜├╟Φ ║╨╝«░ß░·.pdf]

2022년도 제50회 치과기공사 국가시험 문항분석 결과

국민이 신뢰하고 감동하는 시험평가기관
한국보건의료인국가시험원
KOREA HEALTH PERSONNEL LICENSING EXAMINATION INSTITUTE

일반 용어 정의

□ **평균**

- 집단에서의 대표적 경향값으로 전체 값을 더하여 총 응시자로 나눈 값

□ **표준편차**

- 평균과 각 점수의 차이인 편차들의 평균으로 점수가 흩어져 분포되어 있는 정도

□ **검사이론**

- 검사와 검사를 구성하고 있는 문항의 양호도를 분석 및 평가하는 방법을 정의한 이론체계
- 대표적으로 고전검사이론과 문항반응이론이 있음

고전검사이론 용어 정의

□ 고전검사이론(Classical Test Theory; CTT)

- 검사의 질을 분석하는 검사이론 중 한 가지로 19세기 말부터 전개되어 현재까지 주로 사용되고 있는 검사이론임
- 고전검사이론에 의한 문항과 응시자 능력 추정치는 다음과 같음

○ **문항난이도**

· 검사 문항의 쉽고 어려운 정도를 나타내는 지수
· 난이도 지수는 총 반응 수에 대한 정답 반응 수의 비율로 문항의 정답률임
· 문항난이도는 0~100까지의 값을 가짐
· 난이도 값이 큰 경우, 쉬운 문항으로 '난이도가 낮다'라고 해석하며, 난이도 값이 작은 경우, 어려운 문항으로 '난이도가 높다'라고 해석함

○ **문항변별도**

· 각 문항이 응시자의 능력 수준을 변별할 수 있는 정도를 나타내는 지수
· 문항변별도는 -1~+1까지의 값을 가지며, 1에 가까울수록 변별력 크다고 해석함
· 일반적으로 문항변별도가 0.3 이상이면 우수한 문항으로 평가함
· 구하는 방식에는 '상하위집단 구분법', '문항-총점 상관계수' 등이 있음

1) **변별도 1(상하위구분법):** 상위 27%와 하위 27% 집단의 난이도 차이를 구하는 방식
2) **변별도 2(상관계수법):** 문항-총점과의 상관계수로 구하는 방식

○ **신뢰도**

· 시험이 평가하고자 하는 것을 일관성 있게 측정하는가로 시험이 오차없이 정확하게 측정한 정도를 의미함
· 국시원에서는 문항의 내적일관성(Cronbach α)으로 신뢰도를 추정하며 1에 가까울수록 신뢰도가 높다고 해석함

목 차

I. 시행 결과 ······ 5

1. 시험 현황 ······ 6

1) 시험명 ······ 6

2) 시험시행일 ······ 6

3) 응시현황 ······ 6

4) 과목별 문항 수, 배점 및 과락 점수 ······ 6

2. 합격률과 평균성적 ······ 6

1) 합격 및 불합격 현황 ······ 6

2) 과목별 과락자수 내역 ······ 6

3) 전회 대비 합격률과 평균성적 ······ 7

II. 문항분석 결과 ······ 9

1. 성적 ······ 10

1) 전체 성적분포도 ······ 10

2) 과목별 성적분포도 ······ 12

2. 난이도와 변별도 ······ 13

1) 전체 난이도와 변별도 ······ 13

2) 과목별 난이도와 변별도 ······ 16

3) 지식수준별 난이도와 변별도 ······ 25

3. 난이도와 변별도 간 산포도 ······ 34

1) 전체 난이도와 변별도 간 산포도 ······ 34

2) 과목별 난이도와 변별도 간 산포도 ······ 34

4. 신뢰도 분석 ······ 37

I. 시행 결과

1. 시험 현황(※ 합격자 발표일을 기준으로 한 자료임)

1) 시험명: 2022년도 제50회 치과기공사 국가시험

2) 시험시행일: 2022년 11월 26일

3) 응시현황

응시대상자수	결시자수	부정행위자수	응시자 준수사항 위반자 수		응시자수 (%)
			휴대폰 소지	신분증 미지참	
1,089	31	0	0	0	1,157 (97.1)

4) 과목별 문항 수, 배점 및 과락 점수

교 시	과 목 명	문제 수	배점	총점	합격자 점수기준	
					과목 과락기준	총점 합격기준
1교시	치과기공학 기초	75	1	75	30점 미만	123점 이상
1교시	의료관계법규	20	1	20	8점 미만	
2교시	치과기공학	110	1	110	44점 미만	
2교시	실기시험	1	1	100	60점 미만	-
계		206	-	305	-	-

2. 합격률과 평균성적

1) 합격 및 불합격 현황

합격자수 (%)	불합격자수(%)					채점보류자수
	평락	과락	실기탈락	기권	계	
867 (82.0)	183 (17.3)	0 (0.0)	5 (0.5)	2 (0.2)	190 (18.0)	1

2) 과목별 과락자수 내역

과목명 / 과락자수	치과기공학 기초	의료관계법규	치과기공학	실기시험
과목별 과락자 수	0	0	0	5
전과목 과락자 수	0			

3) 전회 대비 합격률과 평균성적

회차	년도	합격률(%)	평균성적	표준편차	백분율 환산점수
제46회	2018.12.	83.5	219.3	52.4	71.9
제47회	2019.11.	79.1	212.7	56.3	69.7
제48회	2020.11.	83.4	218.2	52.7	71.5
제49회	2021.11.	79.8	221.8	62.5	72.7
**제50회**	**2022.11.**	**82.0**	**219.7**	**58.1**	**72.0**

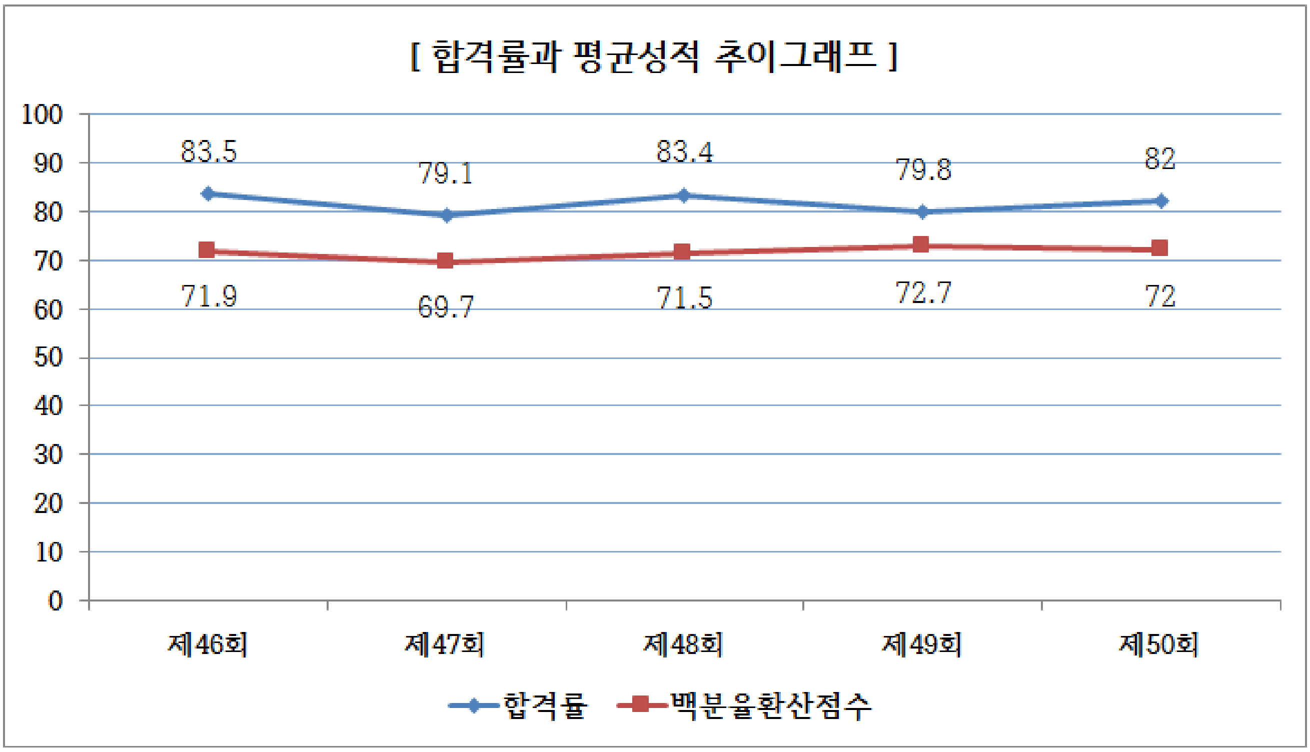

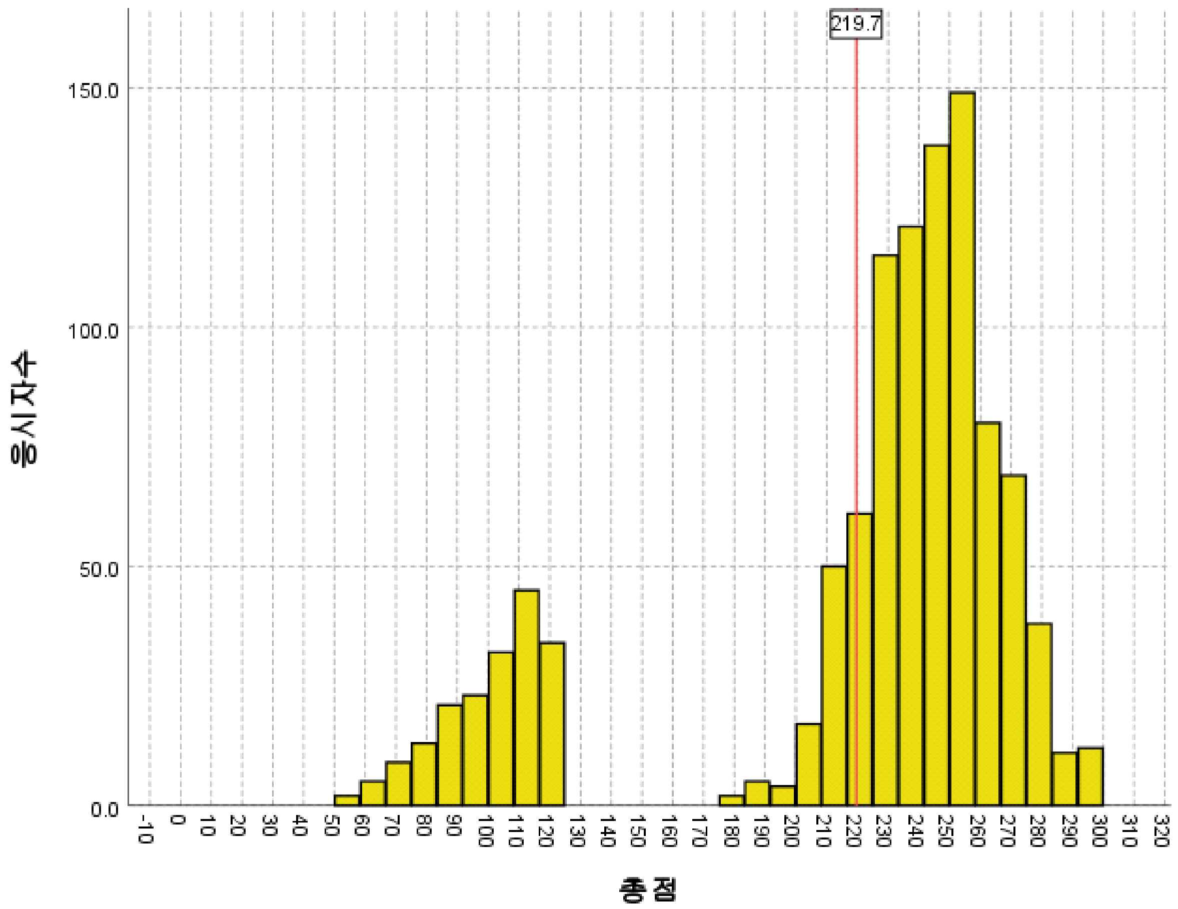

응시자	총점	합격선	평균성적	표준편차
1,056	305.0		219.7	58.1

※ 필기시험 불합격자의 실기성적을 포함하지 않음

해석

- 전년 대비 합격률은 2.2% 증가하고, 백분율 환산점수는 0.7 점 감소함

Ⅱ. 문항분석 결과

1. 성적(※ 2022.2.4.을 기준으로 한 자료임)

1) 전체 성적분포도

○ 직접실기성적 포함 전체 성적분포도

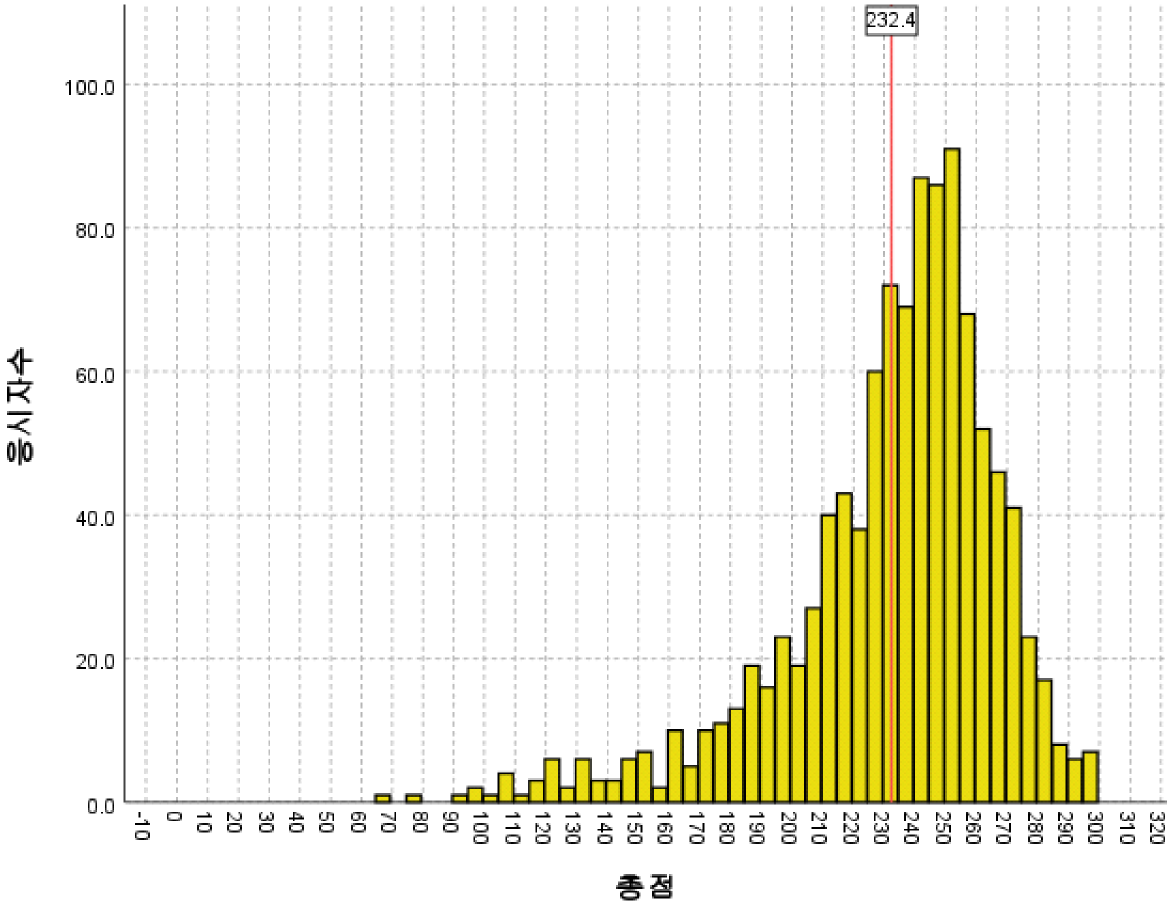

응시자	총점	합격선	평균성적	표준편차
1,056※	305.0		232.4	35.3

※ 1,056명은 전체응시자(1,058명)에 기권자(2명)을 제외한 수치임

○ 필기시험 성적분포도

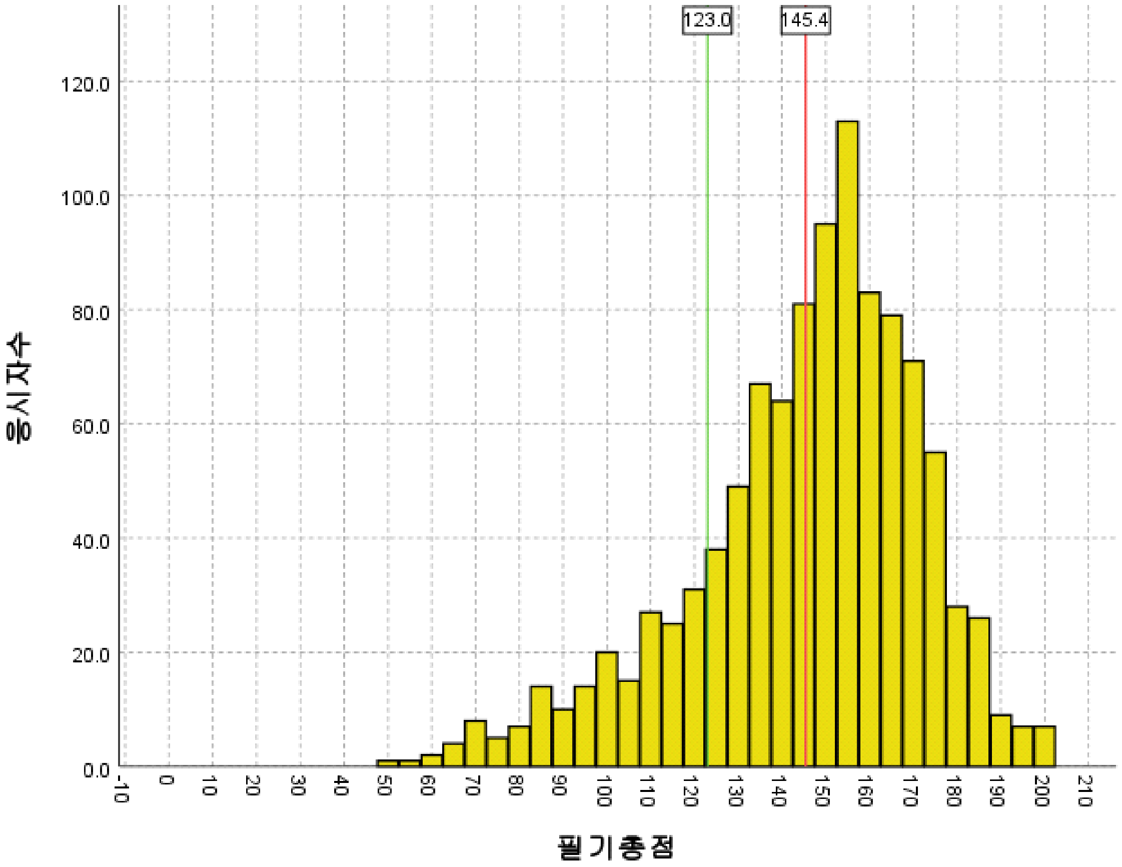

응시자	총점	합격선	평균성적	표준편차
1,056※	205.0	123.0	145.4	26.4

※ 1,056명은 전체응시자(1,058명)에 기권자(2명)을 제외한 수치임

※ 필기시험 성적을 분석대상으로 하며, 직접실기시험의 성적을 제외함

2) 과목별 성적분포도

가) 치과기공학 기초

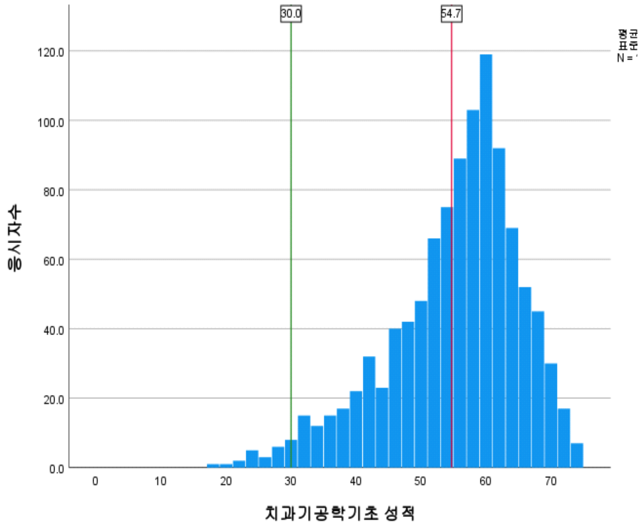

총점	과락선	평균성적	표준편차
75.0	30.0	74.7	10.1

나) 의료관계법규

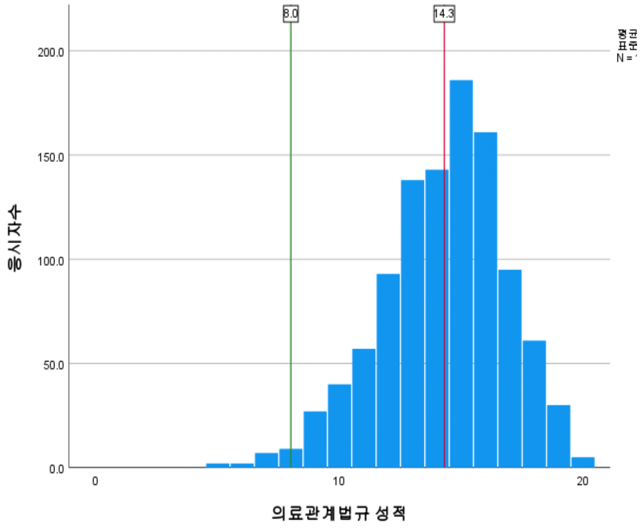

총점	과락선	평균성적	표준편차
20.0	8.0	14.3	2.5

다) 치과기공학

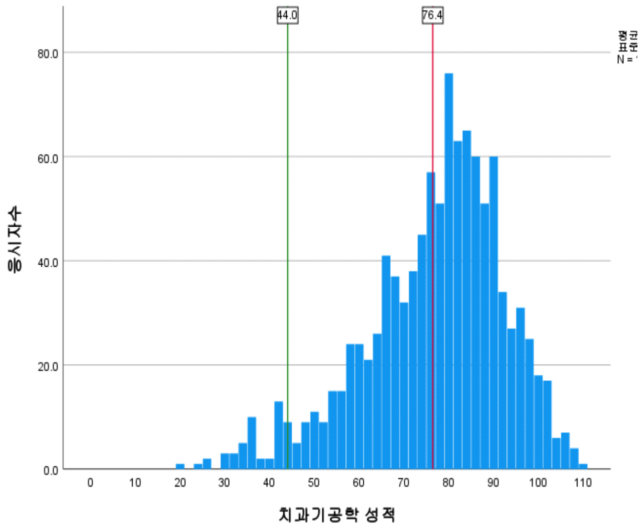

총점	과락선	평균성적	표준편차
110.0	44.0	76.4	15.6

2. 난이도와 변별도

1) 전체 난이도와 변별도

가) 전회 대비 전체 난이도와 변별도

회차	난이도		변별도1		변별도2	
	평균	표준편차	평균	표준편차	평균	표준편차
제46회	70.8	20.3	.25	.12	.27	.10
제47회	70.6	17.7	.29	.12	.28	.08
제48회	72.6	17.6	.29	.13	.30	.10
제49회	71.3	19.1	.31	.14	.32	.11
**제50회**	**70.8**	**19.7**	**.30**	**.13**	**.31**	**.11**

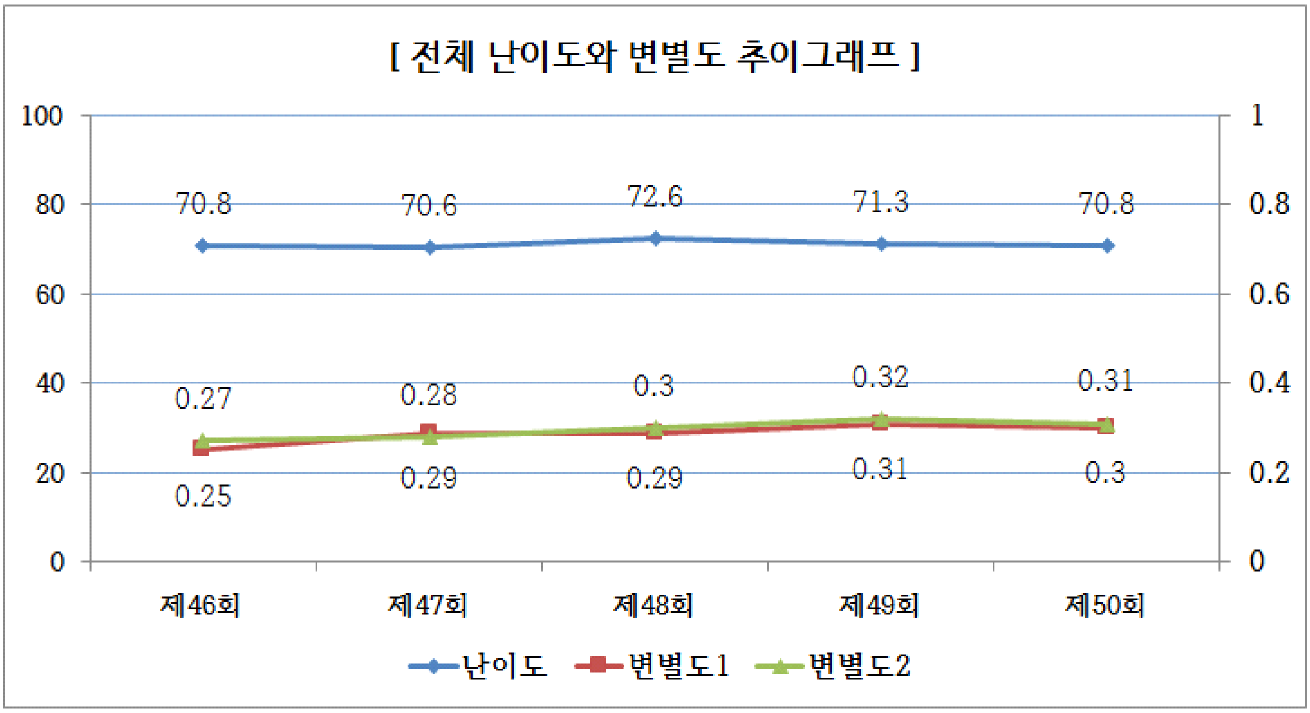

해석

- 전년 대비 난이도 지수는 0.5 감소함
- 변별도 1 지수는 0.01 감소함
- 변별도 2 지수는 0.01 감소함

나) 전체 난이도와 변별도 분포도 및 비율분석

(1) 전체 난이도 분포도 및 비율분석

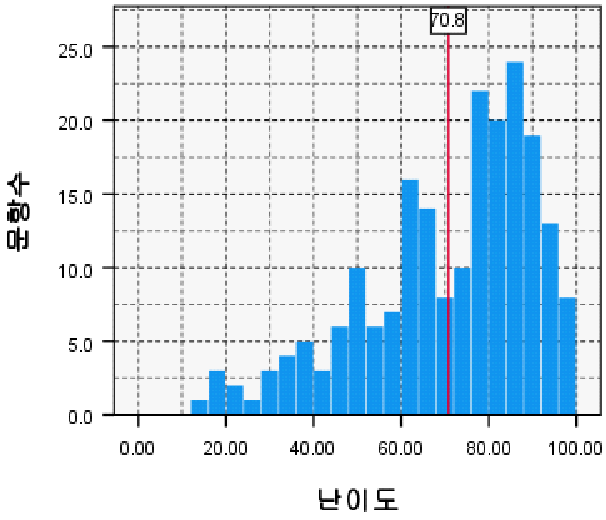

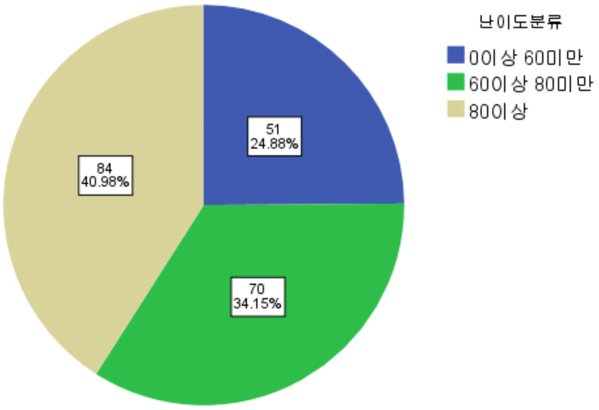

총점	난이도	표준편차
205	70.8	19.7

난이도	문항수	비율(%)
0~60미만	51	24.9
60~80미만	70	34.1
80~100	84	41.0
전체	205	100.0

(2) 전체 변별도1 분포도 및 비율분석

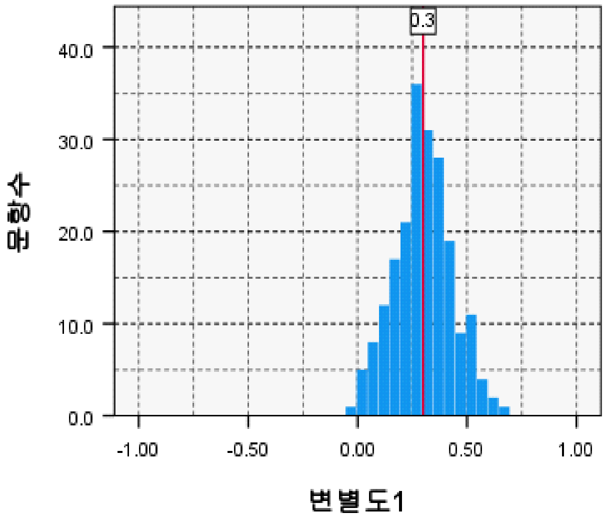

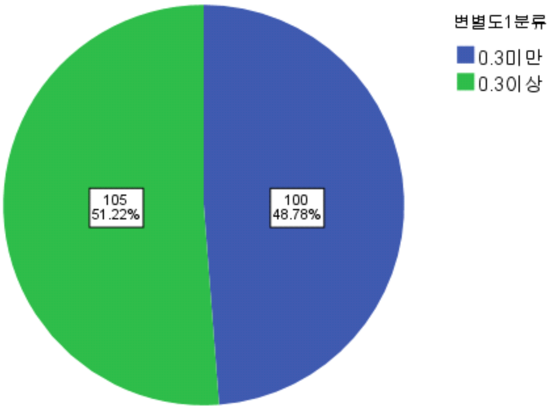

총점	변별도1	표준편차
205	.30	.13

변별도1	문항수	비율(%)
0.3미만	100	48.8
0.3이상	105	51.2
전체	205	100.0

(3) 전체 변별도2 분포도 및 비율분석

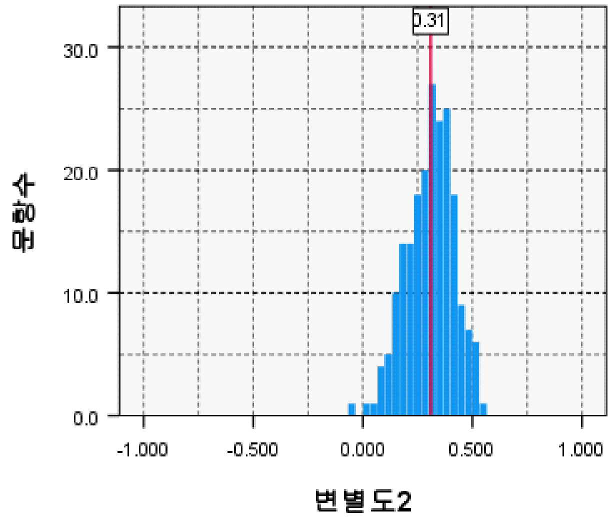

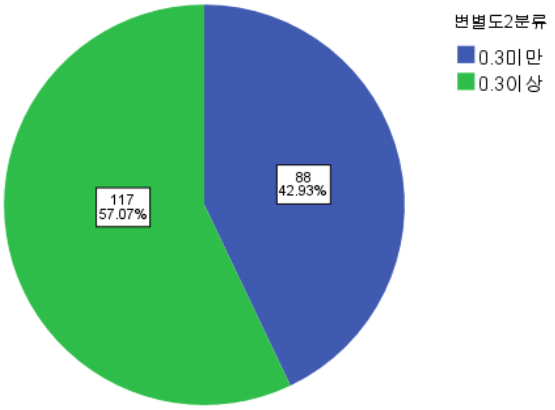

총점	변별도2	표준편차
205	.31	.11

변별도2	문항수	비율(%)
0.3미만	88	42.9
0.3이상	117	57.1
전체	205	100.0

해석

- 난이도 지수가 80에서 100사이인 문항이 전체 205문항 중 84문항으로 가장 많았으며, 차례로 60이상 80미만인 문항이 70문항, 60미만인 문항이 51문항인 것으로 나타남
- 변별도1 지수를 기준으로 분류하였을 때, 0.3 미만인 문항이 100문항으로 0.3 이상인 문항이 105문항인 것에 비해 더 적게 나타남
- 변별도2 지수를 기준으로 분류하였을 때, 0.3 미만인 문항이 88문항으로 0.3 이상인 문항이 117문항인 것에 비해 더 적게 나타남

2) 과목별 난이도와 변별도

가) 전회 대비 과목별 난이도와 변별도

(1) 전회 대비 치과기공학 기초 난이도와 변별도

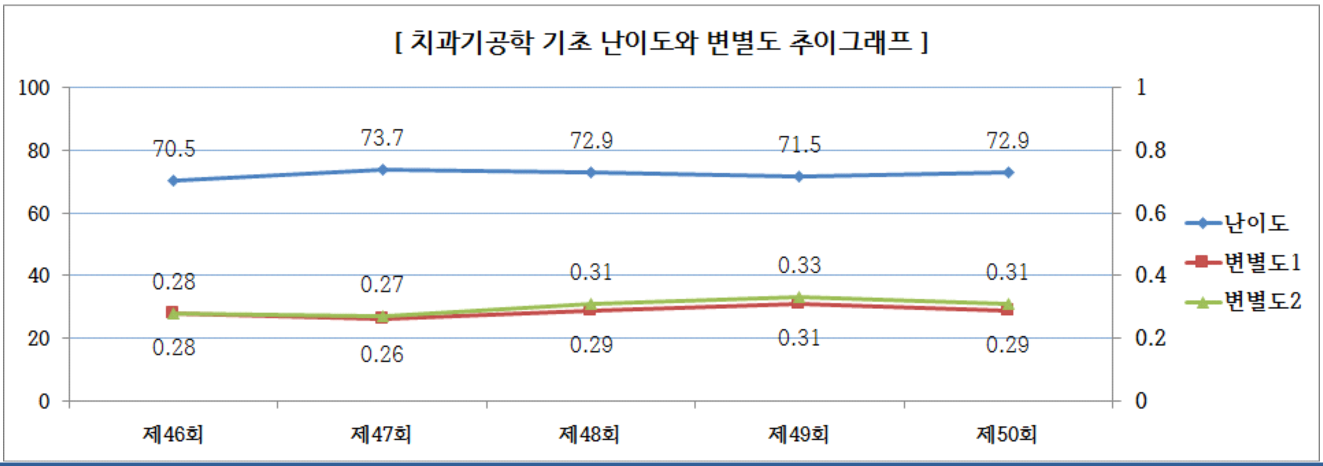

회차	난이도		변별도1		변별도2	
	평균	표준편차	평균	표준편차	평균	표준편차
제46회	70.5	17.6	.28	.10	.28	.08
제47회	73.7	18.1	.26	.10	.27	.07
제48회	72.9	17.5	.29	.12	.31	.10
제49회	71.5	17.4	.31	.13	.33	.10
**제50회**	**72.9**	**18.0**	**.29**	**.11**	**.31**	**.10**

해석

- 전회 대비 치과기공학 기초 과목의 난이도 지수는 1.4 증가함
- 전회 대비 치과기공학 기초 과목의 변별도 1 지수는 0.02 감소함
- 전회 대비 치과기공학 기초 과목의 변별도 2 지수는 0.02 감소함

(2) 전회 대비 의료관계법규 난이도와 변별도

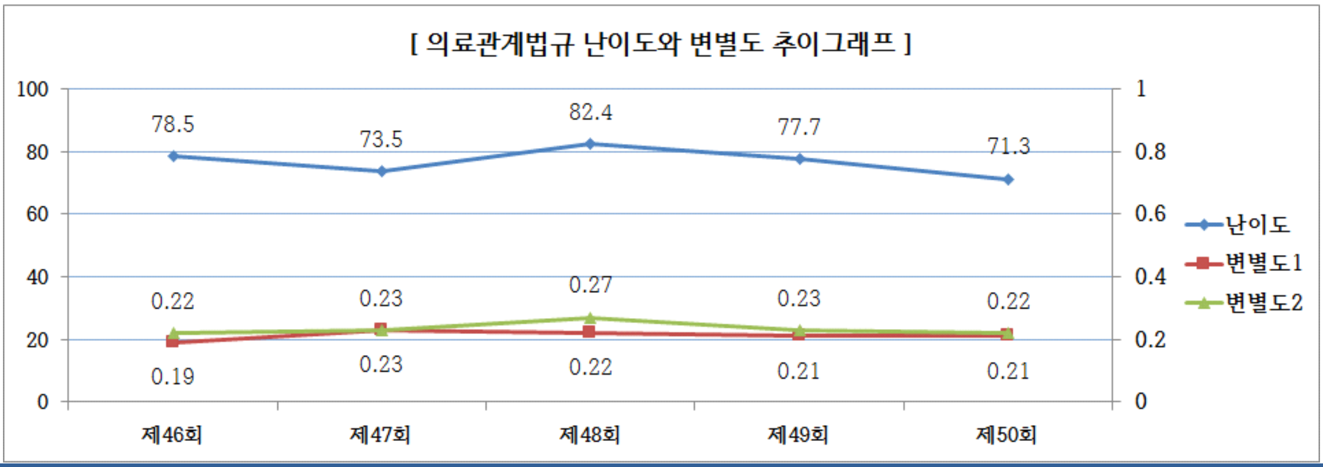

회차	난이도		변별도1		변별도2	
	평균	표준편차	평균	표준편차	평균	표준편차
제46회	78.5	19.5	.19	.12	.22	.09
제47회	73.5	17.9	.23	.13	.23	.08
제48회	82.4	14.8	.22	.16	.27	.10
제49회	77.7	16.0	.21	.14	.23	.11
**제50회**	**71.3**	**20.0**	**.21**	**.13**	**.22**	**.12**

해석

- 전회 대비 의료관계법규 과목의 난이도 지수는 6.4 감소함
- 전회 대비 의료관계법규 과목의 변별도 1 지수는 동일함
- 전회 대비 의료관계법규 과목의 변별도 2 지수는 0.01 감소함

(3) 전회 대비 치과기공학 난이도와 변별도

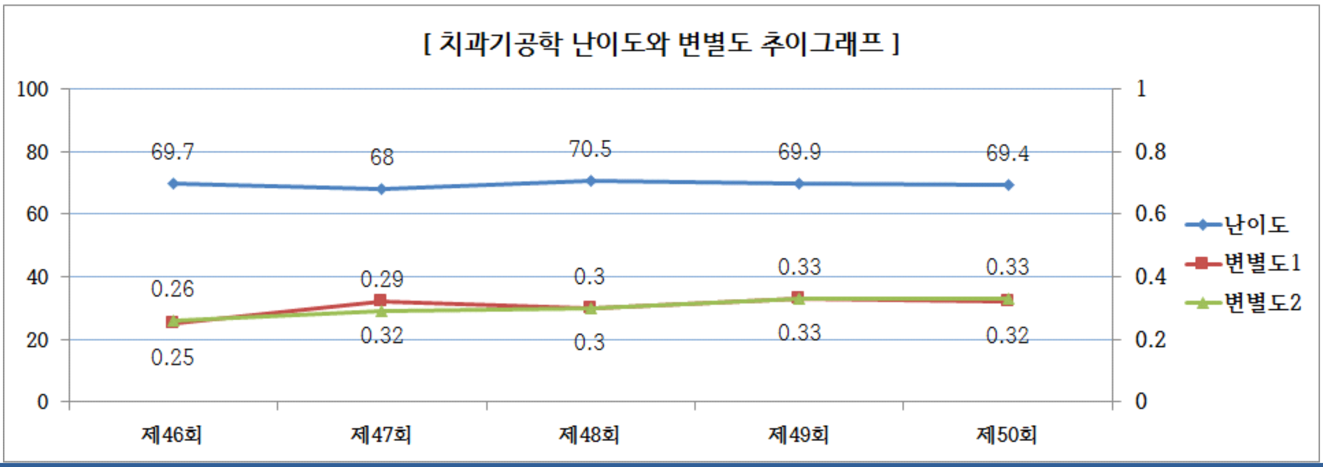

회차	난이도		변별도1		변별도2	
	평균	표준편차	평균	표준편차	평균	표준편차
제46회	69.7	22.0	.25	.13	.26	.12
제47회	68.0	17.2	.32	.12	.29	.09
제48회	70.5	17.7	.30	.13	.30	.11
제49회	69.9	20.6	.33	.13	.33	.10
**제50회**	**69.4**	**20.5**	**.32**	**.14**	**.33**	**.11**

해석

- 전회 대비 치과기공학 과목의 난이도 지수는 0.5 감소함
- 전회 대비 치과기공학 과목의 변별도 1 지수는 0.01 감소함
- 전회 대비 치과기공학 과목의 변별도 2 지수는 동일함

나) 과목별 난이도와 변별도 분포도 및 비율분석

(1) 치과기공학 기초 난이도와 변별도 분포도 및 비율분석

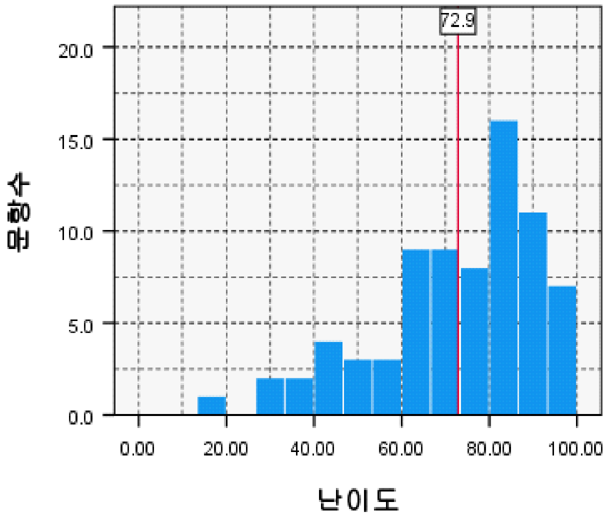

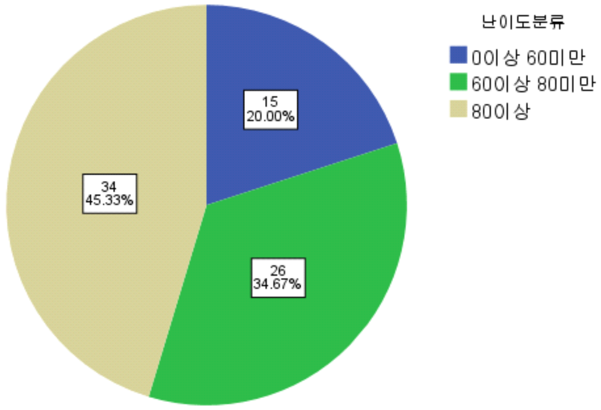

총점	난이도	표준편차	난이도	문항수	비율(%)
75	72.9	18.2	0~60미만	15	20.0
			60~80미만	26	34.7
			80~100	34	45.3
			전체	75	100.0

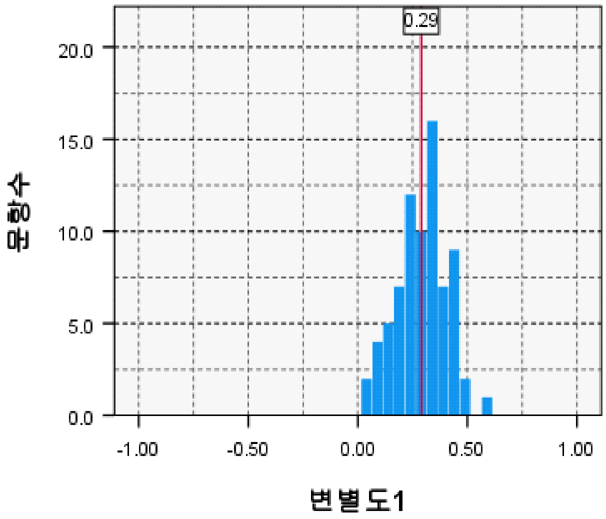

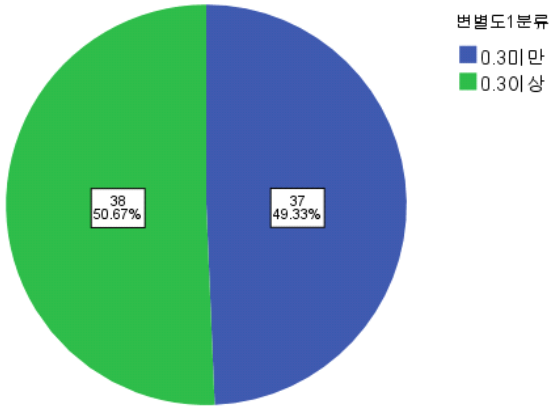

총점	변별도1	표준편차	변별도1	문항수	비율(%)
75	.29	.11	0.3미만	37	49.3
			0.3이상	38	50.7
			전체	75	100.0

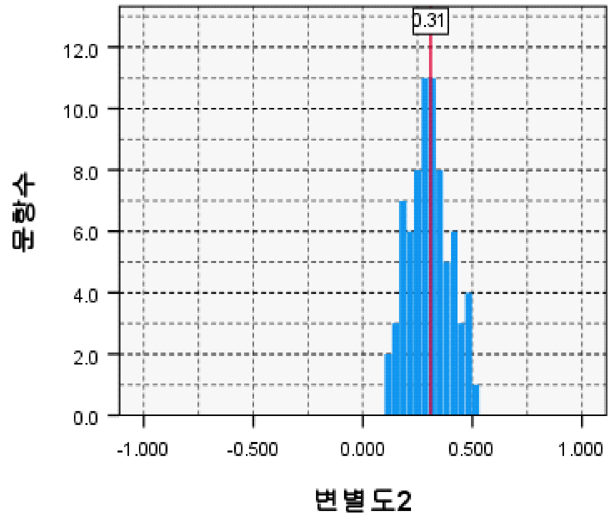

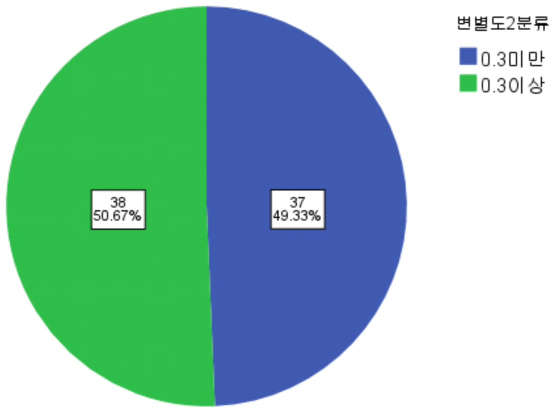

총점	변별도2	표준편차
75	.31	.10

변별도2	문항수	비율(%)
0.3미만	37	49.3
0.3이상	38	50.7
전체	75	100.0

해석

- 난이도 지수가 80 에서 100 사이인 문항이 전체 75 문항 중 34 문항으로 가장 많았으며, 차례로 60 이상 80 미만인 문항이 26 문항, 60 미만인 문항이 15 문항인 것으로 나타남
- 변별도 1 지수를 기준으로 분류하였을 때, 0.3 미만인 문항이 37 문항으로 0.3 이상인 문항이 38 문항인 것에 비해 더 적게 나타남
- 변별도 2 지수를 기준으로 분류하였을 때, 0.3 미만인 문항이 37 문항으로 0.3 이상인 문항이 38 문항인 것에 비해 더 적게 나타남

(2) 의료관계법규 난이도와 변별도 분포도 및 비율분석

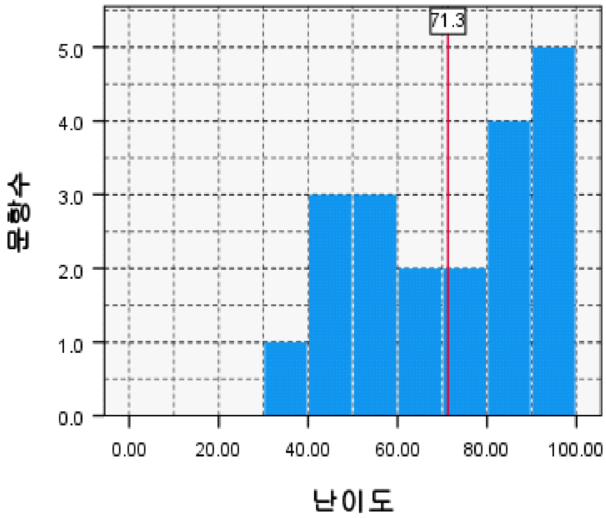

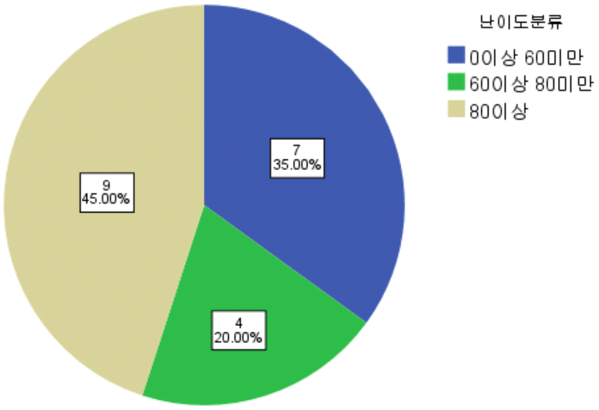

총점	난이도	표준편차
20	71.3	20.5

난이도	문항수	비율(%)
0~60미만	7	35.0
60~80미만	4	20.0
80~100	9	45.0
전체	20	100.0

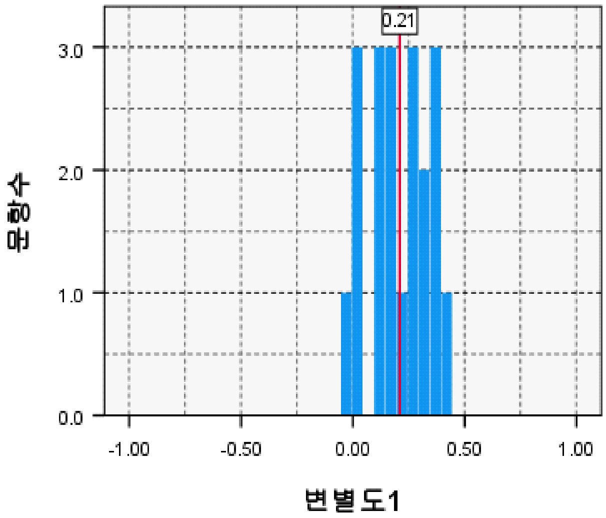

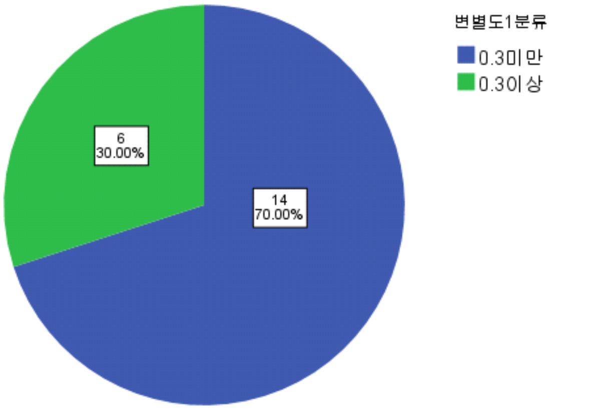

총점	변별도1	표준편차
20	.21	.14

변별도1	문항수	비율(%)
0.3미만	14	70.0
0.3이상	6	30.0
전체	20	100.0

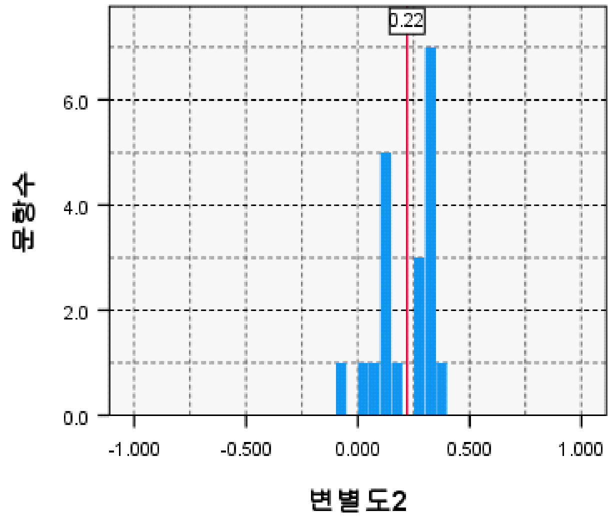

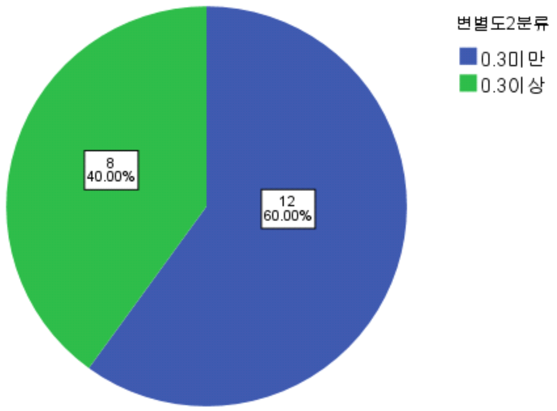

총점	변별도2	표준편차	변별도2	문항수	비율(%)
20	.22	.12	0.3미만	12	60.0
			0.3이상	8	40.0
			전체	20	100.0

해석

- 난이도 지수가 80에서 100사이인 문항이 전체 20문항 중 11문항으로 가장 많았으며, 차례로 60미만인 문항이 7문항, 60이상 80미만인 문항이 4문항인 것으로 나타남
- 변별도1 지수를 기준으로 분류하였을 때, 0.3 미만인 문항이 14문항으로 0.3 이상인 문항이 6문항인 것에 비해 더 많이 나타남
- 변별도2 지수를 기준으로 분류하였을 때, 0.3 미만인 문항이 12문항으로 0.3 이상인 문항이 8문항인 것에 비해 더 많이 나타남

(3) 치과기공학 난이도와 변별도 분포도 및 비율분석

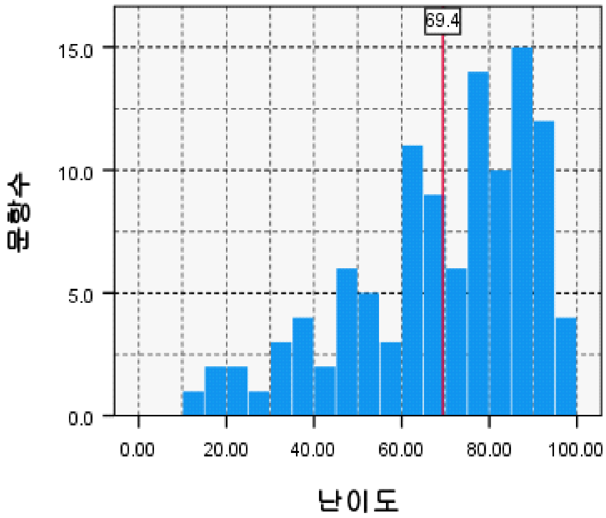

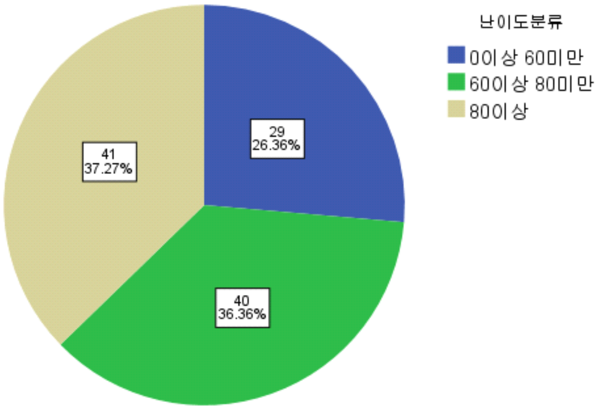

총점	난이도	표준편차
110	69.4	20.6

난이도	문항수	비율(%)
0~60미만	29	26.4
60~80미만	40	36.4
80~100	41	37.3
전체	110	100.0

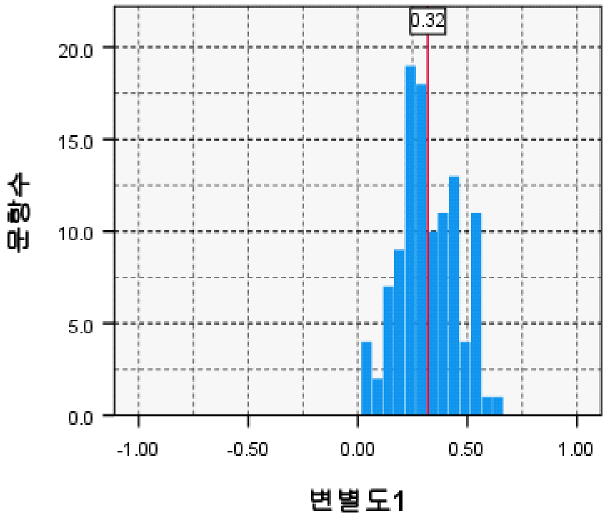

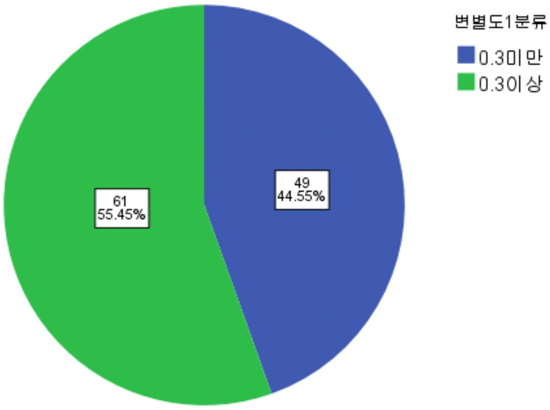

총점	변별도1	표준편차
110	.32	.14

변별도1	문항수	비율(%)
0.3미만	49	44.5
0.3이상	61	55.5
전체	110	100.0

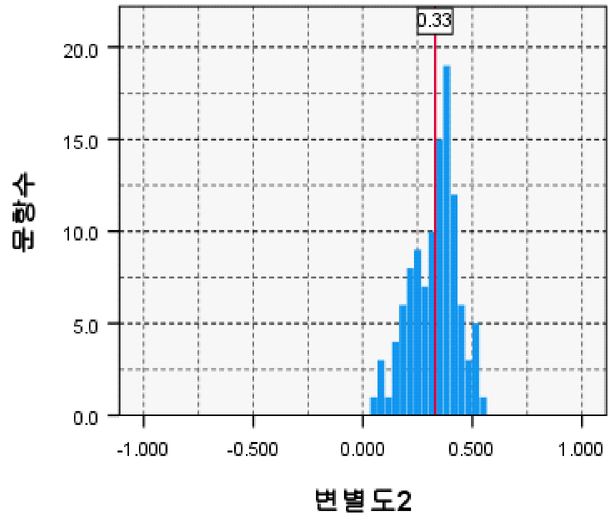

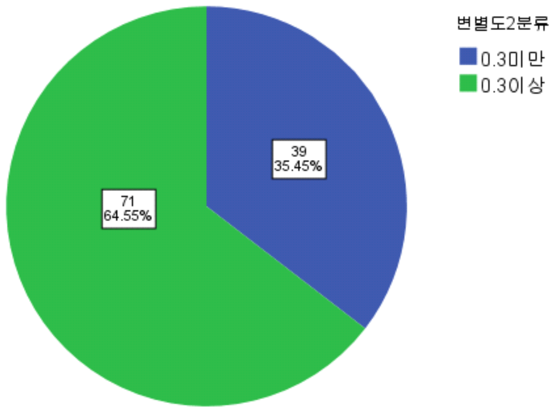

총점	변별도2	표준편차
110	.33	.11

변별도2	문항수	비율(%)
0.3미만	39	35.5
0.3이상	71	64.5
전체	110	100.0

해석

- 난이도 지수가 80에서 100사이인 문항이 전체 110문항 중 41문항으로 가장 많았으며, 차례로 60이상 80미만인 문항이 40문항, 60미만인 문항이 29문항인 것으로 나타남
- 변별도1 지수를 기준으로 분류하였을 때, 0.3 미만인 문항이 49문항으로 0.3 이상인 문항이 61문항인 것에 비해 더 적게 나타남
- 변별도2 지수를 기준으로 분류하였을 때, 0.3 미만인 문항이 39문항으로 0.3 이상인 문항이 71문항인 것에 비해 더 적게 나타남

3) 지식수준별 난이도와 변별도

가) 전회 대비 지식수준별 난이도와 변별도

(1) 전회 대비 암기형 난이도와 변별도

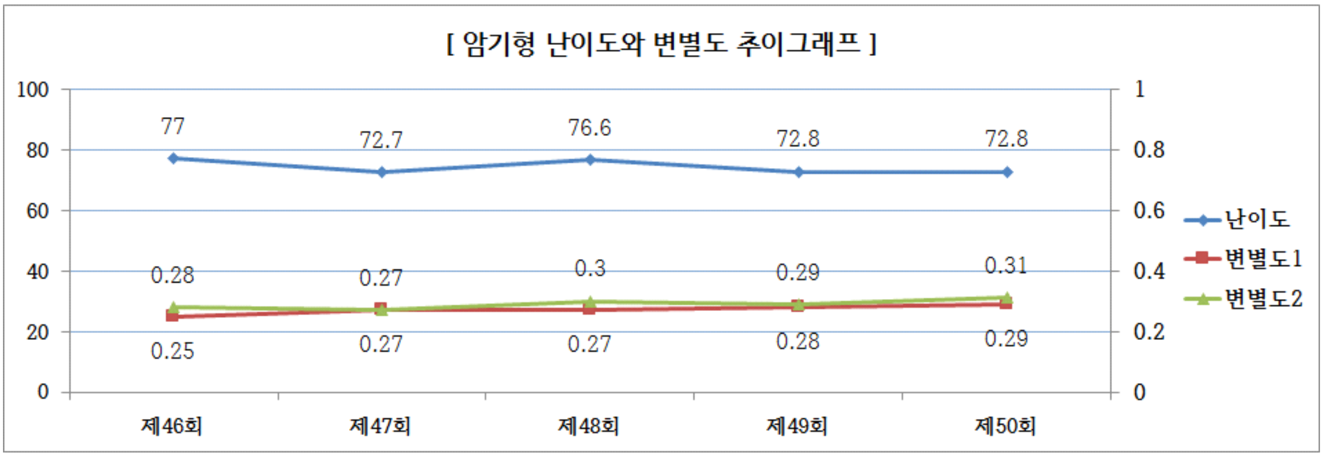

회차	난이도		변별도1		변별도2	
	평균	표준편차	평균	표준편차	평균	표준편차
제46회	77.0	17.9	.25	.12	.28	.09
제47회	72.7	17.3	.27	.13	.27	.09
제48회	76.6	15.9	.27	.11	.30	.09
제49회	72.8	18.2	.28	.13	.29	.11
**제50회**	**72.8**	**19.0**	**.29**	**.11**	**.31**	**.10**

해석

- 전회 대비 암기형 문항의 난이도 지수는 동일함
- 전회 대비 암기형 문항의 변별도 1 지수는 0.01 증가함
- 전회 대비 암기형 문항의 변별도 2 지수는 0.02 증가함

(2) 전회 대비 해석형 난이도와 변별도

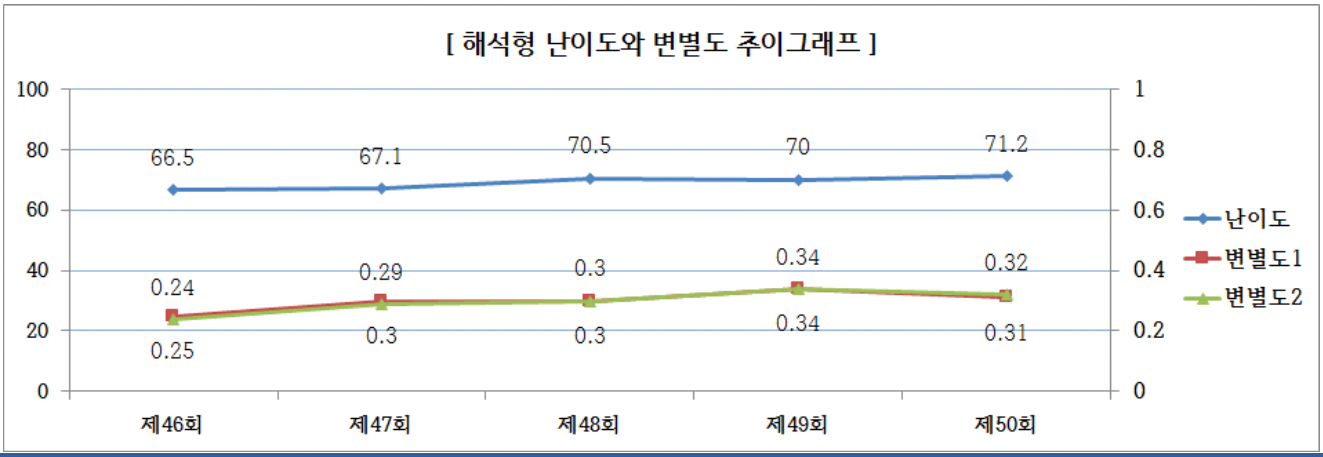

회차	난이도		변별도1		변별도2	
	평균	표준편차	평균	표준편차	평균	표준편차
제46회	66.5	20.1	.25	.12	.24	.11
제47회	67.1	18.4	.30	.11	.29	.08
제48회	70.5	17.8	.30	.13	.30	.10
제49회	70.0	19.9	.34	.13	.34	.09
**제50회**	**71.2**	**18.4**	**.31**	**.14**	**.32**	**.11**

해석

- 전회 대비 해석형 문항의 난이도 지수는 1.2 증가함
- 전회 대비 해석형 문항의 변별도 1 지수는 0.03 감소함
- 전회 대비 해석형 문항의 변별도 2 지수는 0.02 감소함

(3) 전회 대비 해결형 난이도와 변별도

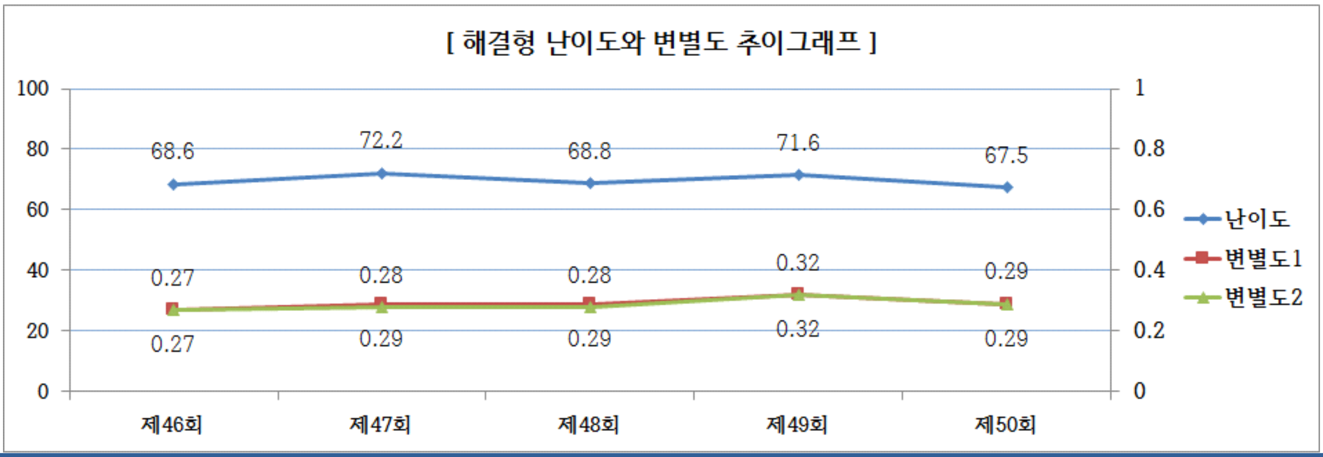

회차	난이도		변별도1		변별도2	
	평균	표준편차	평균	표준편차	평균	표준편차
제46회	68.6	21.8	.27	.12	.27	.11
제47회	72.2	17.1	.29	.12	.28	.08
제48회	68.8	19.4	.29	.15	.28	.13
제49회	71.6	19.0	.32	.15	.32	.12
**제50회**	**67.5**	**23.0**	**.29**	**.14**	**.29**	**.10**

해석

- 전회 대비 해결형 문항의 난이도 지수는 4.1 감소함
- 전회 대비 해결형 문항의 변별도 1 지수는 0.03 감소함
- 전회 대비 해결형 문항의 변별도 2 지수는 0.03 감소함

나) 지식수준별 난이도와 변별도 분포도 및 비율분석

(1) 암기형 난이도와 변별도 분문도 및 비율분석

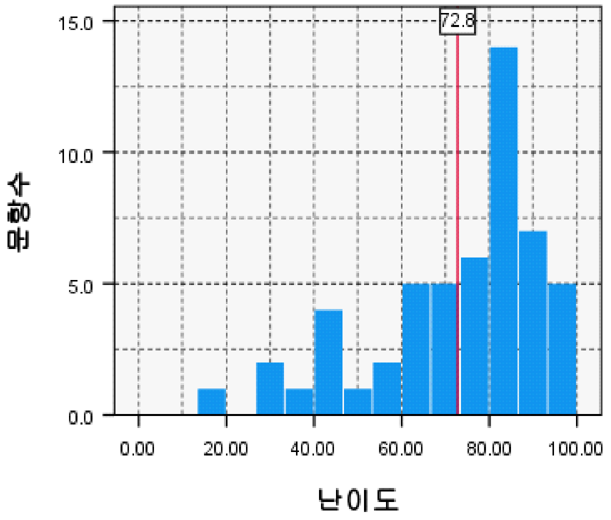

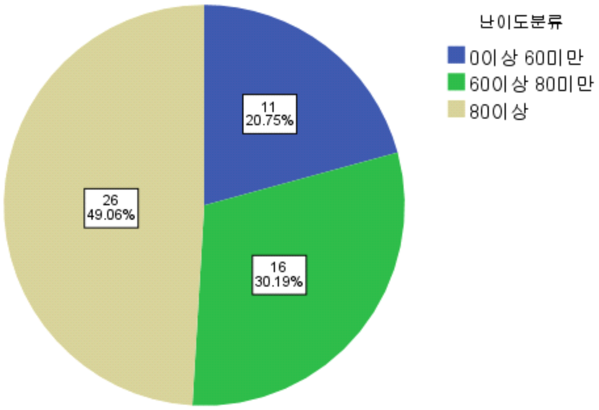

총점	난이도	표준편차
53	72.8	19.2

난이도	문항수	비율(%)
0~60미만	11	20.8
60~80미만	16	30.2
80~100	26	49.0
전체	53	100.0

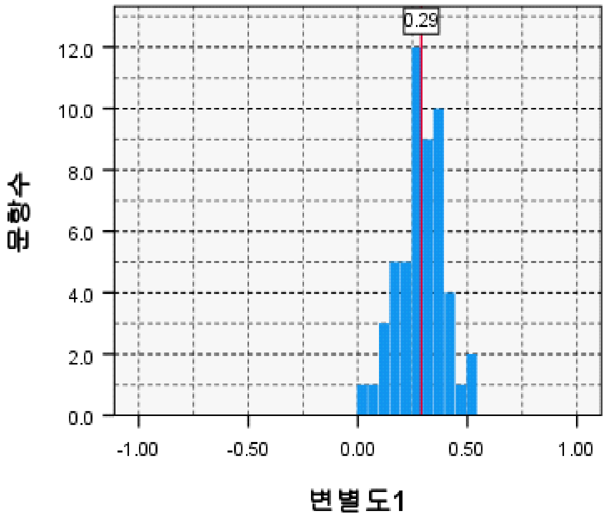

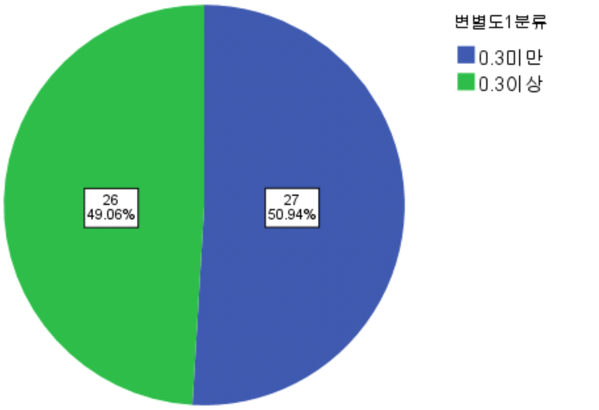

총점	변별도1	표준편차
53	.29	.11

변별도1	문항수	비율(%)
0.3미만	27	50.9
0.3이상	26	49.1
전체	53	100.0

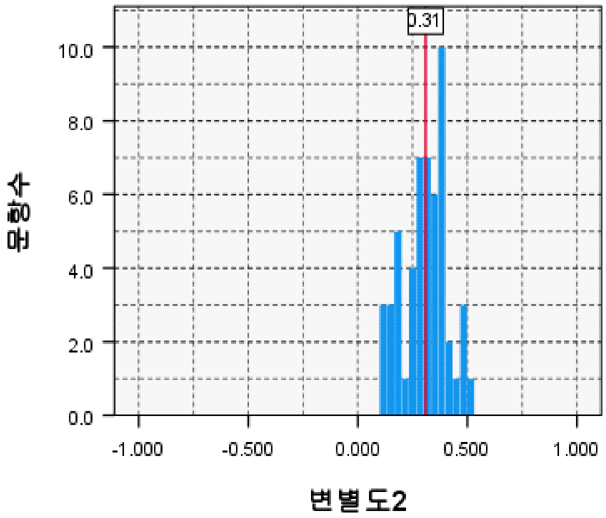

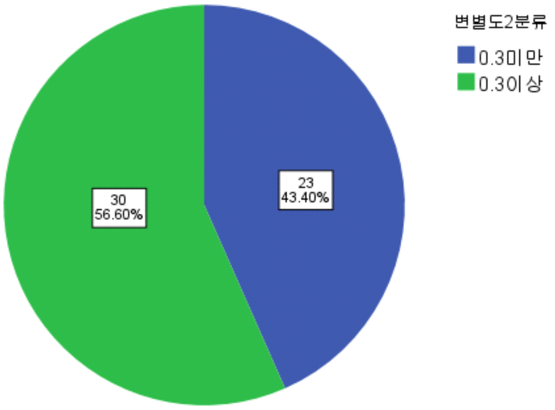

총점	변별도2	표준편차	변별도2	문항수	비율(%)
53	.31	.10	0.3미만	23	43.4
			0.3이상	30	56.6
			전체	53	100.0

해석

- 난이도 지수가 80 에서 100 사이인 문항이 전체 53 문항 중 26 문항으로 가장 많았으며, 차례로 60 이상 80 미만인 문항이 16 문항, 60 미만인 문항이 11 문항인 것으로 나타남
- 변별도 1 지수를 기준으로 분류하였을 때, 0.3 미만인 문항이 27 문항으로 0.3 이상인 문항이 26 문항인 것에 비해 더 적게 나타남
- 변별도 2 지수를 기준으로 분류하였을 때, 0.3 미만인 문항이 23 문항으로 0.3 이상인 문항이 30 문항인 것에 비해 더 적게 나타남

(2) 해석형 난이도와 변별도 분포도 및 비율분석

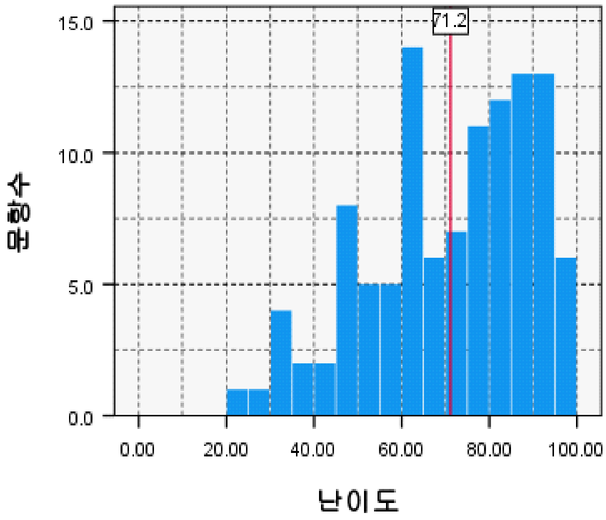

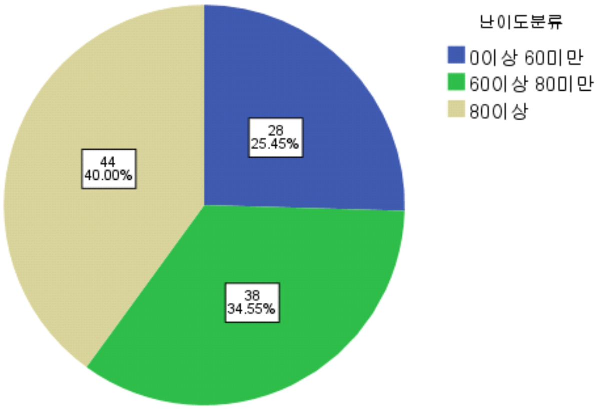

총점	난이도	표준편차
110	71.2	18.5

난이도	문항수	비율(%)
0~60미만	28	25.5
60~80미만	38	34.5
80~100	44	40.0
전체	110	100.0

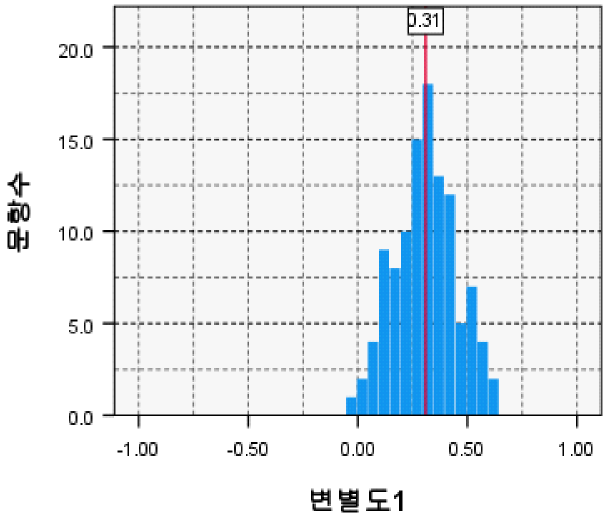

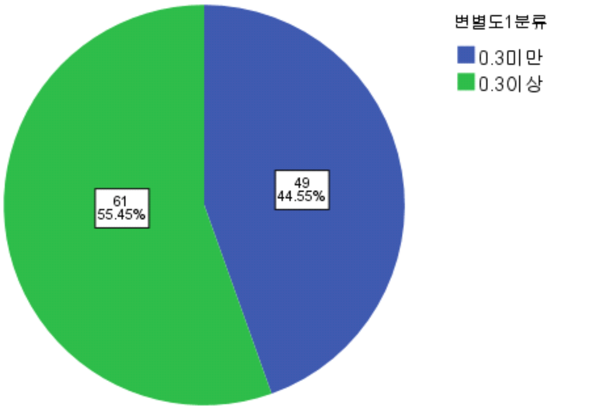

총점	변별도1	표준편차
110	.31	.14

변별도1	문항수	비율(%)
0.3미만	49	44.5
0.3이상	61	55.5
전체	110	100.0

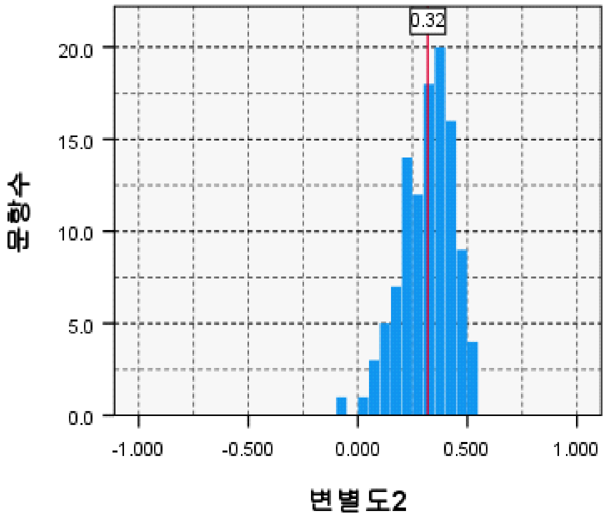

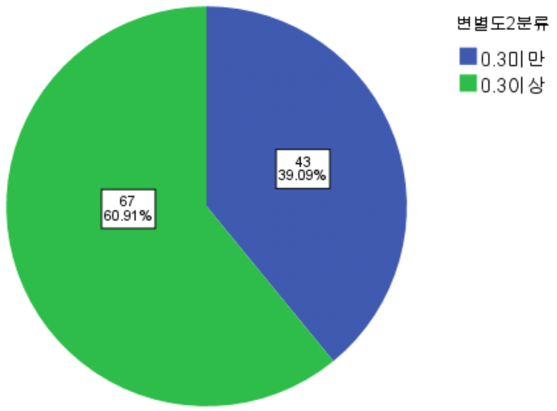

총점	변별도2	표준편차
110	.32	.11

변별도2	문항수	비율(%)
0.3미만	43	39.1
0.3이상	57	60.9
전체	110	100.0

해석

- 난이도 지수가 80에서 100사이인 문항이 전체 110문항 중 44문항으로 가장 많았으며, 차례로 60미만인 문항이 38문항, 60이상 80미만인 문항이 28문항인 것으로 나타남
- 변별도1 지수를 기준으로 분류하였을 때, 0.3 미만인 문항이 49문항으로 0.3 이상인 문항이 61문항인 것에 비해 더 적게 나타남
- 변별도2 지수를 기준으로 분류하였을 때, 0.3 미만인 문항이 43문항으로 0.3 이상인 문항이 57문항인 것에 비해 더 적게 나타남

(3) 해결형 난이도와 변별도 분포도 및 비율분석

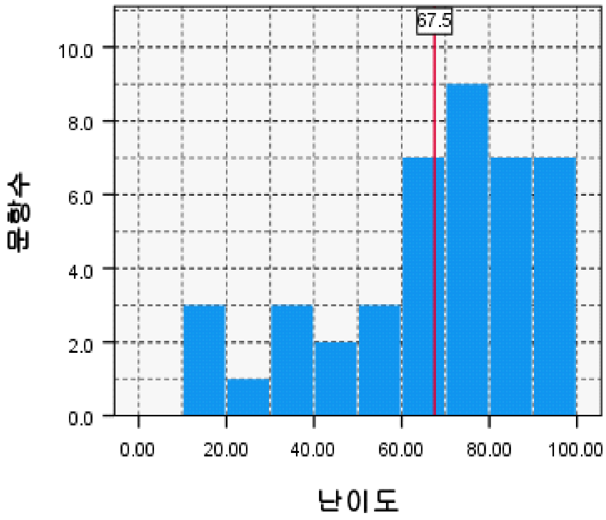

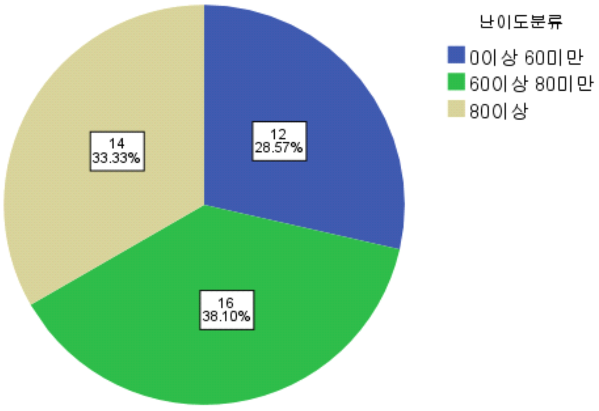

총점	난이도	표준편차	난이도	문항수	비율(%)
42	67.5	23.3	0~60미만	12	28.6
			60~80미만	16	38.1
			80~100	14	33.3
			전체	42	100.0

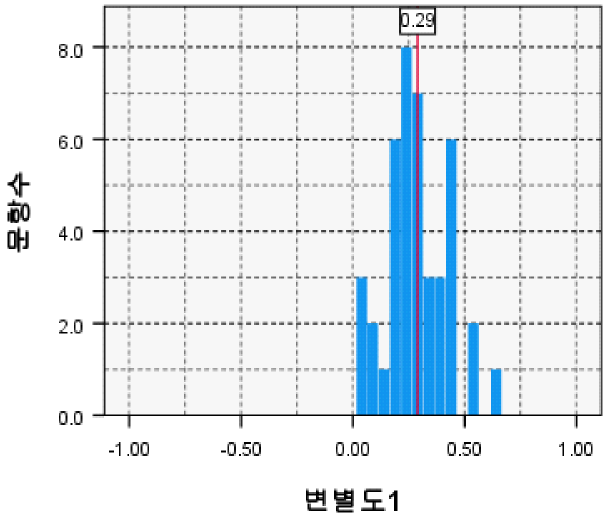

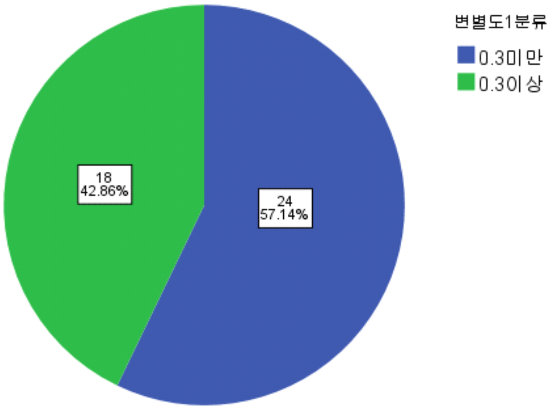

총점	변별도1	표준편차	변별도1	문항수	비율(%)
42	.29	.14	0.3미만	24	57.1
			0.3이상	18	42.9
			전체	42	100.0

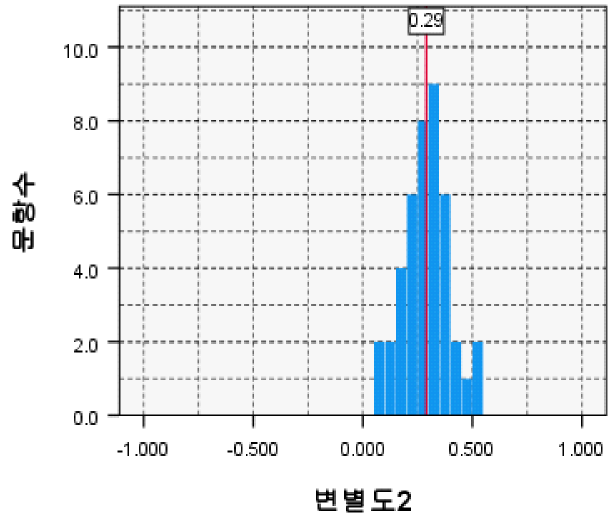

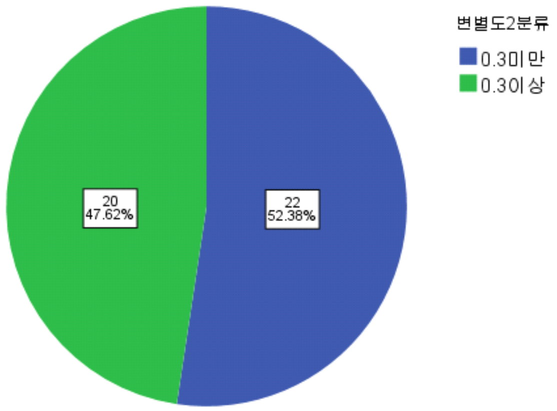

총점	변별도2	표준편차
42	.29	.11

변별도2	문항수	비율(%)
0.3미만	22	52.4
0.3이상	20	47.6
전체	42	100.0

해석

- 난이도 지수가 60 이상 80 미만인 문항이 전체 42 문항 중 16 문항으로 가장 많았으며, 차례로 80 에서 100 사이인 문항이 14 문항, 60 미만인 문항이 12 문항인 것으로 나타남
- 변별도 1 지수를 기준으로 분류하였을 때, 0.3 미만인 문항이 24 문항으로 0.3 이상인 문항이 18 문항인 것에 비해 더 많이 나타남
- 변별도 2 지수를 기준으로 분류하였을 때, 0.3 미만인 문항이 22 문항으로 0.3 이상인 문항이 20 문항인 것에 비해 더 많이 나타남

3. 난이도와 변별도 간 산포도

1) 전체 난이도와 변별도 간 산포도

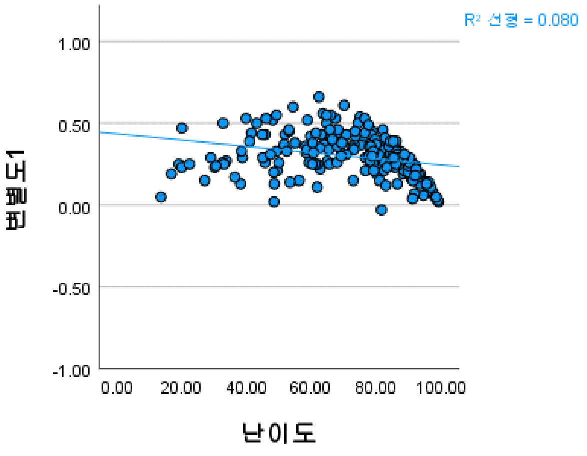

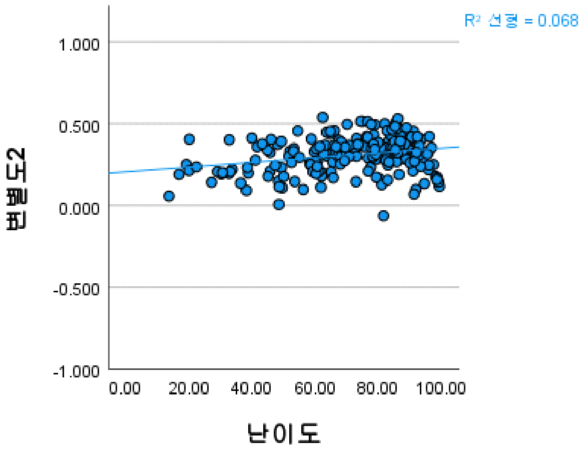

해석

- 전체 문항을 대상으로 난이도와 변별도1 지수 간 상관은 -.283*으로 문항 난이도와 변별력 간 관련성이 낮은 것으로 나타남
- 난이도와 변별도2 지수 간 상관은 .261로 문항 난이도와 변별력 간 관련성이 낮은 것으로 나타남

2) 과목별 난이도와 변별도 간 산포도

가) 치과기공학 기초 난이도와 변별도 간 산포도

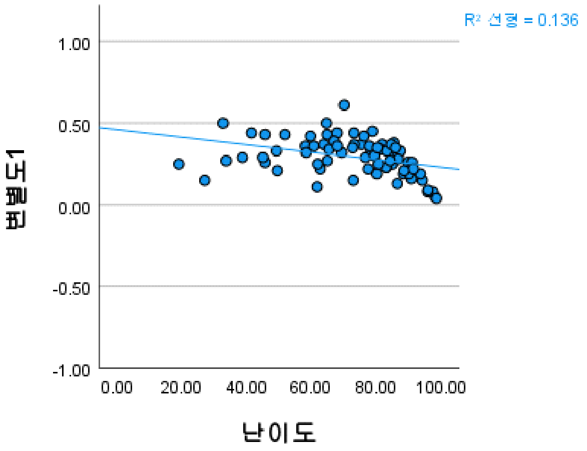

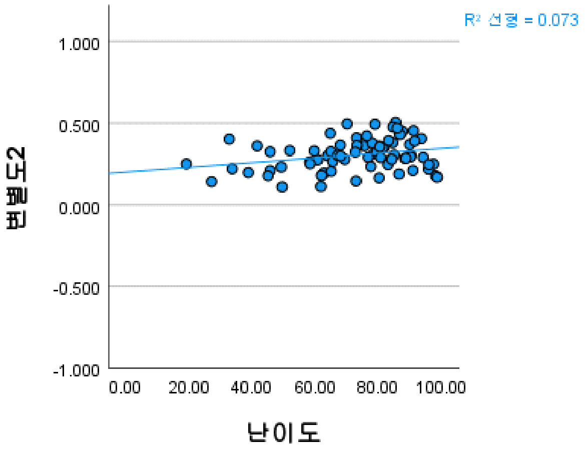

해석

- 치과기공학 기초 과목 문항을 대상으로 난이도와 변별도1 지수 간 상관은 $-.368^{**}$으로 문항 난이도가 쉬울수록 변별력이 낮아지는 것으로 나타남
- 난이도와 변별도2 지수 간 상관은 $.270^{*}$으로 문항 난이도와 변별력 간 관련성이 낮은 것으로 나타남

나) 의료관계법규 난이도와 변별도 간 산포도

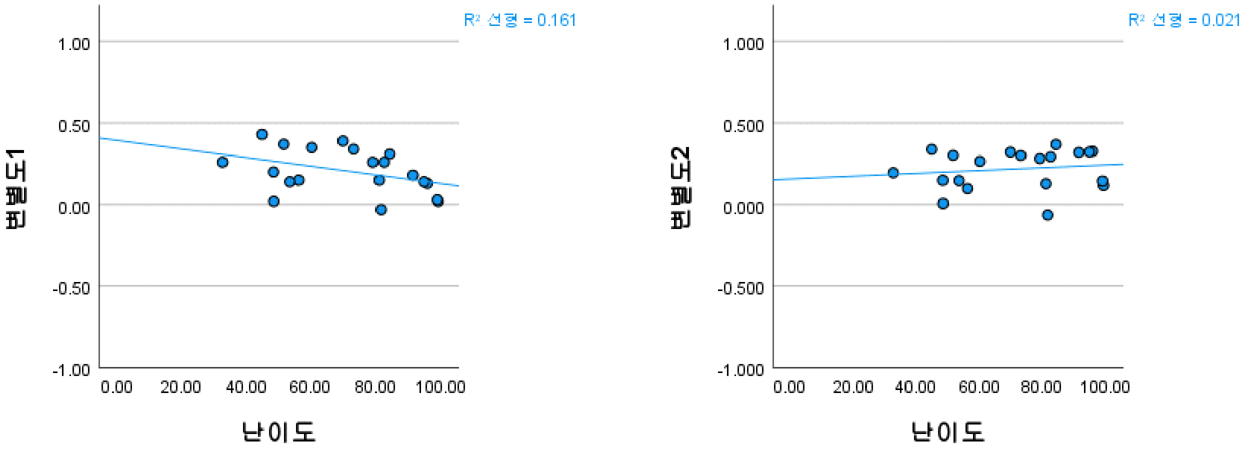

해석

- 의료관계법규 과목 문항을 대상으로 난이도와 변별도1 지수 간 상관은 $-.401^{*}$로 문항 난이도가 쉬울수록 변별력이 낮아지는 것으로 나타남
- 난이도와 변별도2 지수 간 상관은 .146로 문항 난이도와 변별력 간 관련성이 없는 것으로 나타남

다) 치과기공학 난이도와 변별도 간 산포도

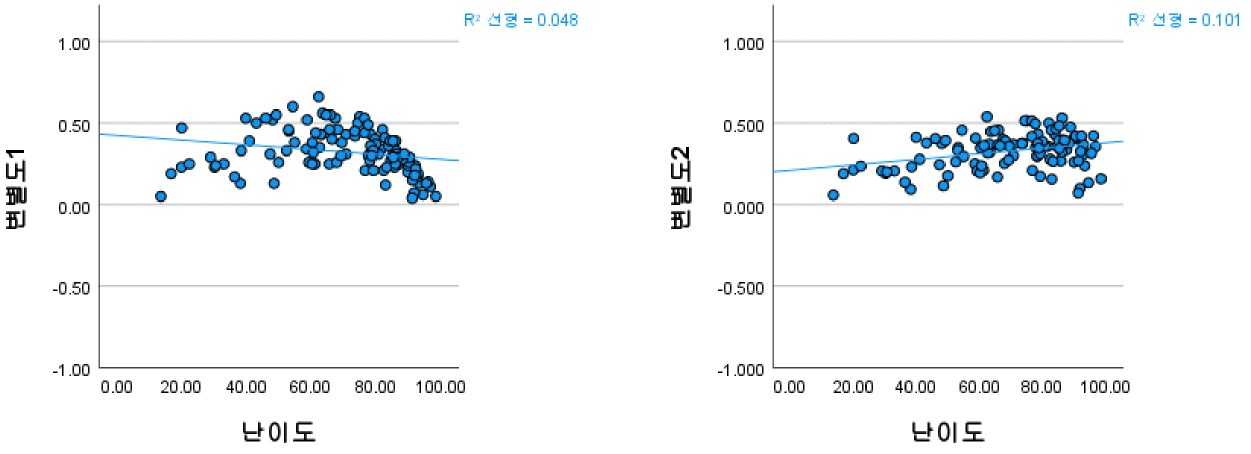

해석

- 치과기공학 과목 문항을 대상으로 난이도와 변별도1 지수 간 상관은 -.220* 으로 문항 난이도와 변별력 간 관련성이 낮은 것으로 나타남
- 난이도와 변별도2 지수 간 상관은 .318*로 문항 난이도가 쉬울수록 변별력이 높아지는 것으로 나타남

4. 신뢰도 분석

과목명	문항수	제46회	제47회	제48회	제49회	제50회
전체	205	.939	.947	.951	.959	**.955**
치과기공학 기초	75	.872	.866	.887	.900	**.890**
의료관계법규	20	.570	.623	.687	.617	**.516**
치과기공학	110	.893	.917	.913	.934	**.932**

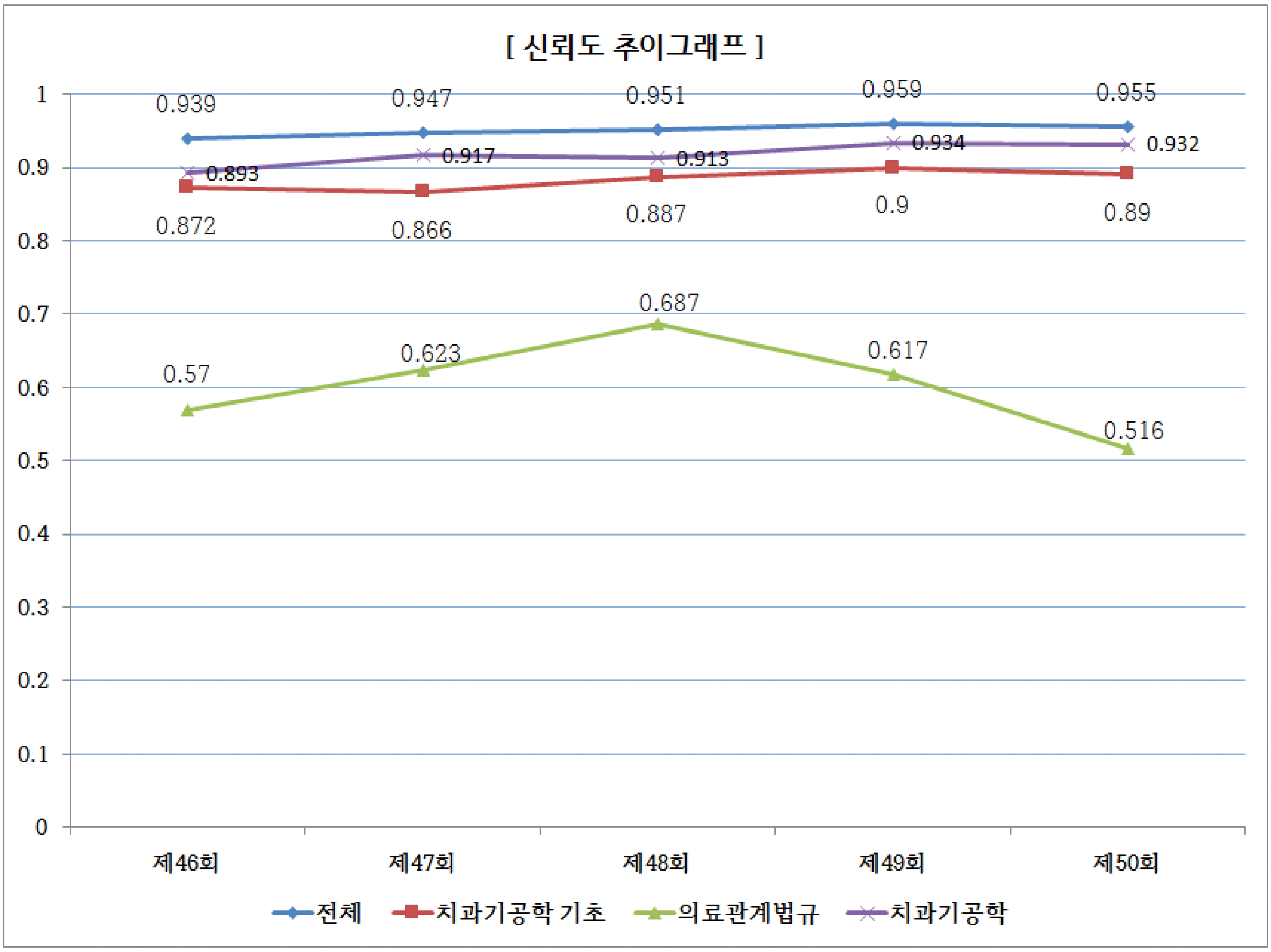

해석

- 의료관계법규 외 치과기공사 국가시험 전체와 나머지 과목은 문항 신뢰도가 각각 .955, .890, .932 로 모두 일관되게 해당 영역을 측정하고 있는 것으로 나타남
- 의료관계법규 과목의 경우 신뢰도 .516 로 문항 간 일관성이 다소 낮은 것으로 나타남
- 치과기공사 국가시험 전체의 경우 전회 대비 신뢰도가 .004 감소하였으며, 치과기공학의 기초, 의료관계법규, 치과기공학 과목의 경우 차례로 .010, .101, .002 순으로 신뢰도가 감소하였음

○ 분석결과 관련 문의 : 한국보건의료인국가시험원 연구개발본부 배상영 책임연구원
Tel : 02-2087-8955, FAX : 02-2087-8885
E-mail : bsy0601@kuksiwon.or.kr
