# Supplementary material for: Item difficulty index, discrimination index, and reliability of the 26 health professions licensing examinations in 2022, Korea: a psychometric study
Source: J Educ Eval Health Prof. 2023 Nov 22;20:31. doi: 10.3352/jeehp.2023.20.31 (PMC11959405; doi:10.3352/jeehp.2023.20.31)
Supplement: Supplementary file 1 — Supplement 1. Item analysis results of 26 health professions licensing examinations administered during late 2022 and early 2023. [file jeehp-20-31_Suppl1.zip › 2022│Γ╡╡ ┴a50╚╕ ─í░·└o╗2╗τ ▒╣░í╜├╟Φ ║╨╝«░ß░·.pdf]

2022년도 제50회
치과위생사 국가시험
문항분석 결과

국민이 신뢰하고 감동하는 시험평가기관
한국보건의료인국가시험원
KOREA HEALTH PERSONNEL LICENSING EXAMINATION INSTITUTE

일반 용어 정의

□ **평균**

- 집단에서의 대표적 경향값으로 전체 값을 더하여 총 응시자로 나눈 값

□ **표준편차**

- 평균과 각 점수의 차이인 편차들의 평균으로 점수가 흩어져 분포되어 있는 정도

□ **추정난이도**

- 문항개발자가 예측한 정답률

□ **검사이론**

- 검사와 검사를 구성하고 있는 문항의 양호도를 분석 및 평가하는 방법을 정의한 이론체계
- 대표적으로 고전검사이론과 문항반응이론이 있음

고전검사이론 용어 정의

□ **고전검사이론(Classical Test Theory; CTT)**

- 검사의 질을 분석하는 검사이론 중 한 가지로 19세기 말부터 전개되어 현재까지 주로 사용되고 있는 검사이론임
- 고전검사이론에 의한 문항과 응시자 능력 추정치는 다음과 같음

○ **문항난이도**

· 검사 문항의 쉽고 어려운 정도를 나타내는 지수
· 난이도 지수는 총 반응 수에 대한 정답 반응 수의 비율로 문항의 정답률임
· 문항난이도는 0~100까지의 값을 가짐
· 난이도 값이 큰 경우, 쉬운 문항으로 '난이도가 낮다'라고 해석하며, 난이도 값이 작은 경우, 어려운 문항으로 '난이도가 높다'라고 해석함

○ **문항변별도**

· 각 문항이 응시자의 능력 수준을 변별할 수 있는 정도를 나타내는 지수
· 문항변별도는 -1~+1까지의 값을 가지며, 1에 가까울수록 변별력 크다고 해석함
· 일반적으로 문항변별도가 0.3 이상이면 우수한 문항으로 평가함
· 구하는 방식에는 '상하위집단 구분법', '문항-총점 상관계수' 등이 있음

1) **변별도 1(상하위구분법):** 상위 27%와 하위 27% 집단의 난이도 차이를 구하는 방식
2) **변별도 2(상관계수법):** 문항-총점과의 상관계수로 구하는 방식

○ **신뢰도**

· 시험이 평가하고자 하는 것을 일관성 있게 측정하는가로 시험이 오차없이 정확하게 측정한 정도를 의미함
· 국시원에서는 문항의 내적일관성(Cronbach α)으로 신뢰도를 추정하며 1에 가까울수록 신뢰도가 높다고 해석함

목 차

I. 시행 결과 ········ 6

1. 시험 현황 ········ 7

1) 시험명 ········ 7

2) 시험시행일 ········ 7

3) 응시현황 ········ 7

4) 과목별 문항 수, 배점 및 과락 점수 ········ 7

2. 합격률과 평균성적 ········ 7

1) 합격 및 불합격 현황 ········ 7

2) 과목별 과락자수 내역 ········ 8

3) 전회 대비 합격률과 평균성적 ········ 8

II. 문항분석 결과 ········ 10

1. 성적 ········ 11

1) 전체 성적분포도 ········ 11

2) 과목별 성적분포도 ········ 12

2. 난이도와 변별도 ········ 14

1) 전체 난이도와 변별도 ········ 14

2) 과목별 난이도와 변별도 ········ 16

3) 지식수준별 난이도와 변별도 ········ 22

3. 난이도와 변별도 간 산포도 ········ 30

1) 전체 난이도와 변별도 간 산포도 ········ 30

2) 과목별 난이도와 변별도 간 산포도 ········ 30

4. 신뢰도 분석 ········ 32

I. 시행 결과

1. 시험 현황

1) 시험명: 2022년도 제50회 치과위생사 국가시험

2) 시험시행일: 2022년 12월 11일

3) 응시현황

구분	응시대상자수	결시자수	부정행위자수	응시자 준수사항 위반자 수		응시자수 (%)
				휴대폰 소지	신분증 미지참	
실기	5,641	66	0	1	0	5,574 (98.8)
필기	5,641	159	0	0	0	5,481 (97.2)
종합	5,641	*64	0	1	0	**5,575 (98.8)

* 실기시험과 필기시험을 모두 결시

** 실기시험과 필기시험 중 하나라도 응시(채점보류자수 제외)

4) 과목별 문항 수, 배점 및 과락 점수

교 시	과 목 명	문제 수	배점	총점	합격자 점수기준	
					과목 과락기준	**총점 합격기준
1교시	의료관계법규	20	1	20	8점 미만	120점 이상
1교시	*치위생학1	80	1	80	72점 미만	
2교시	*치위생학2	100	1	100		
-	실기시험	1	100	100	60점 미만	60점 이상
계		201		300		

* 1교시 치위생학1, 2교시 치위생학2는 동일과목

** 필기시험에서 각 과목 만점의 40% 이상, 전 과목 총점의 60% 이상 득점. 실기시험에서 만점의 60% 이상 득점.

2. 합격률과 평균성적

1) 합격 및 불합격 현황

구분	합격자수 (%)	불합격자수(%)					채점보류자수
		평락	과락	실기탈락	기권	계	
실기	5494 (98.6)	0 (0.0)	80 (1.4)	0 (0.0)	0 (0.0)	80 (1.4)	0
필기	4,590 (83.7)	890 (16.2)	1 (0.0)	0 (0.0)	0 (0.0)	891 (16.3)	1
종합	4,575 (82.1)	984 (17.7)	1 (0.0)	15 (0.3)	0 (0.0)	1,000 (17.9)	1

2) 과목별 과락자수 내역

과목명 / 과락자수	의료관계법규	치위생학
과목별 과락자 수	1	0
전과목 과락자 수	1	

3) 전회 대비 합격률과 평균성적

회차	년도	합격률(%)	평균성적	표준편차	백분율 환산점수
제46회	2019	80.0	206.9	52.3	69.0
제47회	2019	84.6	217.2	49.6	72.4
제48회	2020	74.1	197.7	57.2	65.9
제49회	2021	80.8	211.2	54.4	70.4
제50회	2022	82.1	214.4	53.8	71.5

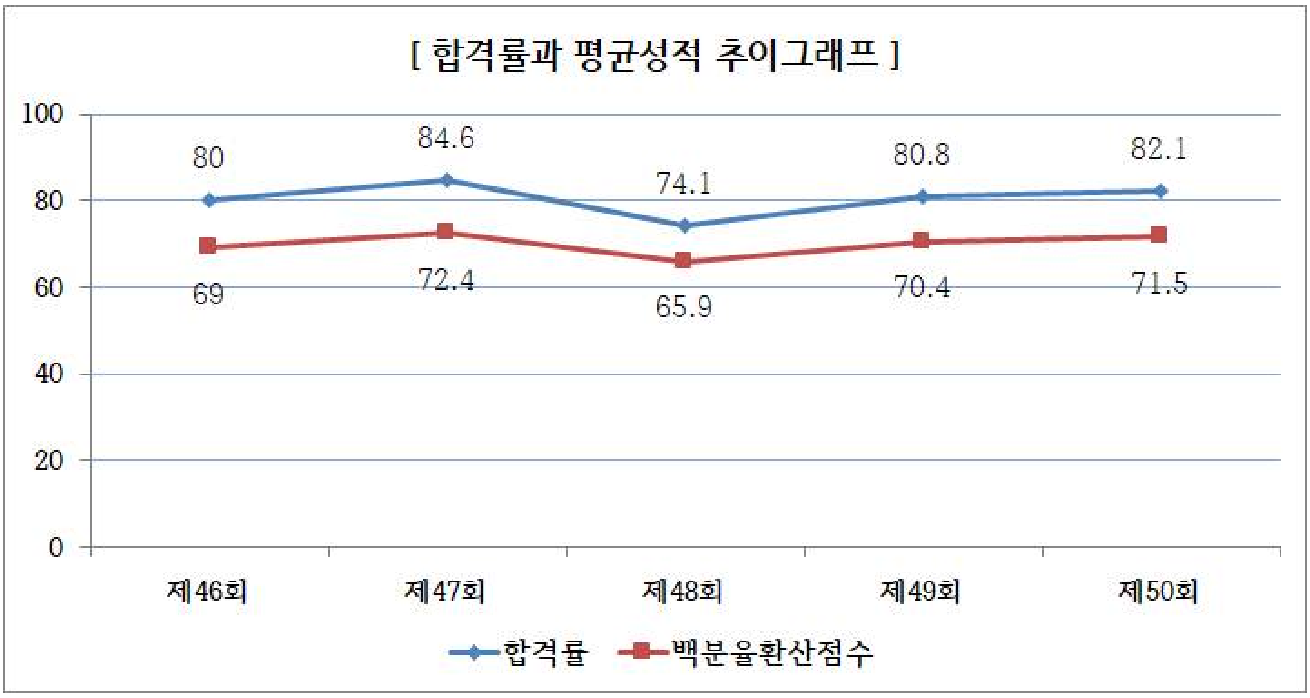

해석

- 전년 대비 합격률은 6.7% 증가하고, 백분율 환산점수는 5.4 점 증가함

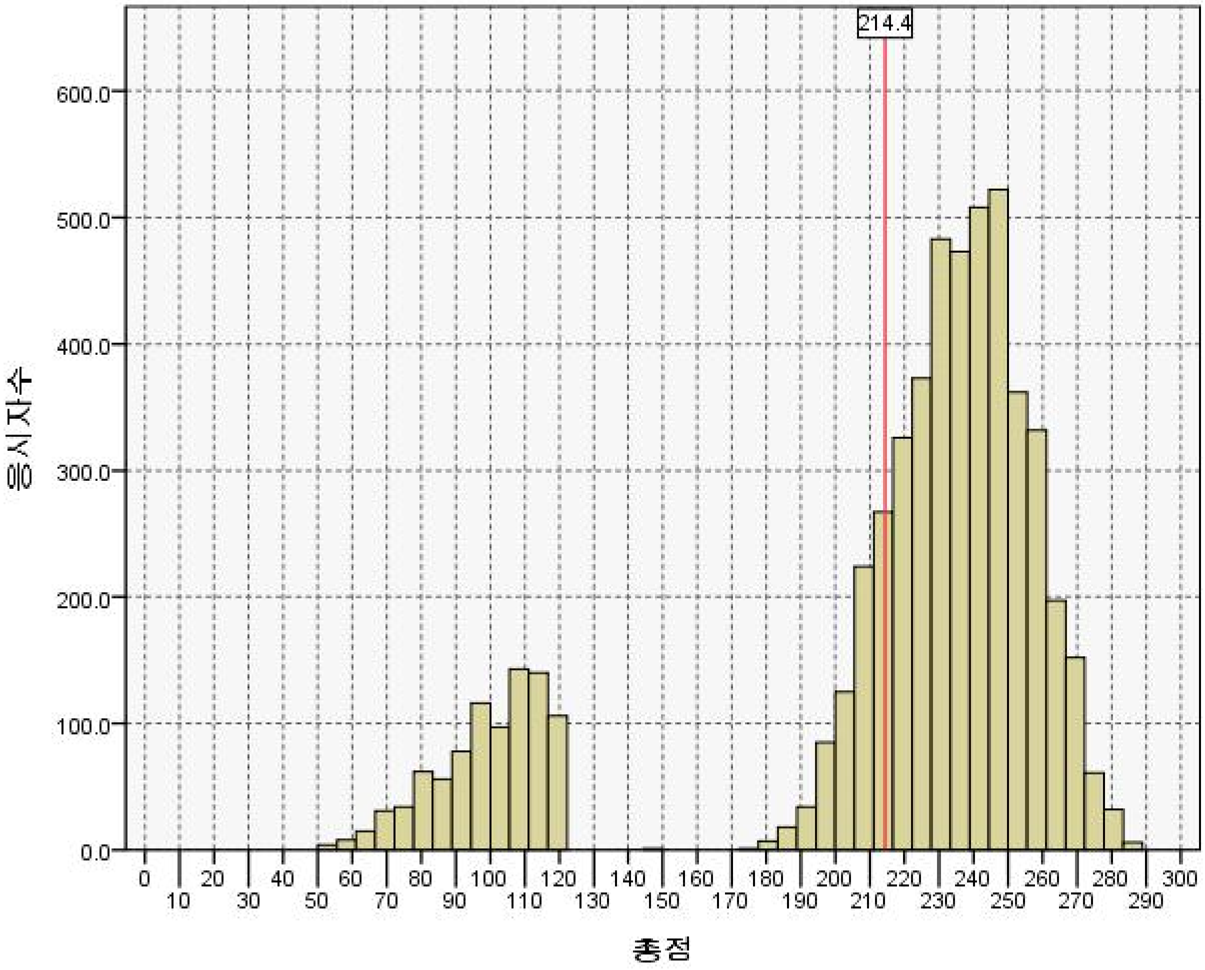

응시자	총점	합격선	평균성적	표준편차
5,481	300		214.4	53.8

※ 필기시험 불합격자의 실기성적을 포함하지 않음

Ⅱ. 문항분석 결과

1. 성적

1) 전체 성적분포도

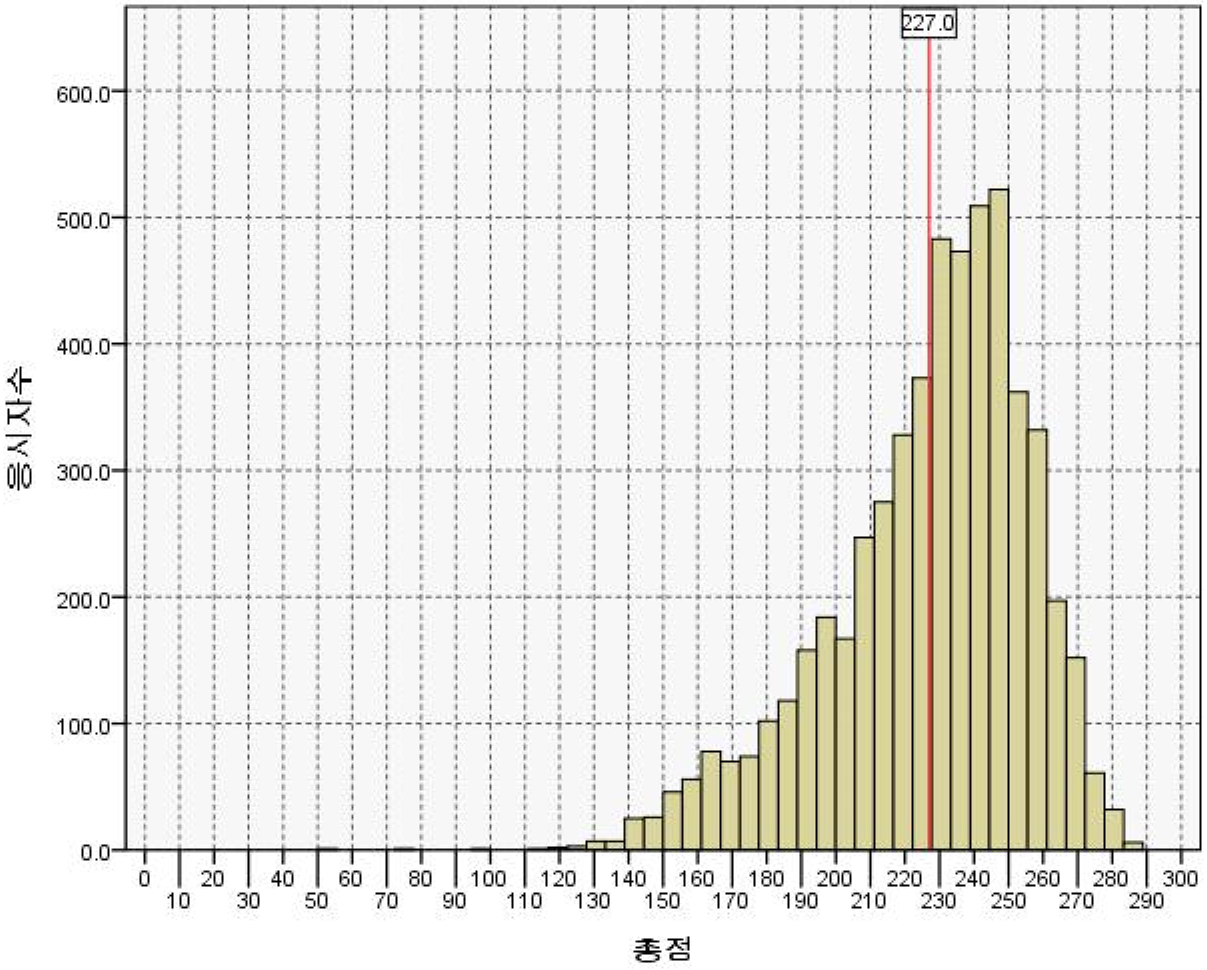

응시자	총점	합격선	평균성적	표준편차
5,481	300		227.0	29.1

※ 필기시험 불합격자의 실기성적을 포함함

2) 과목별 성적분포도

가) 의료관계법규

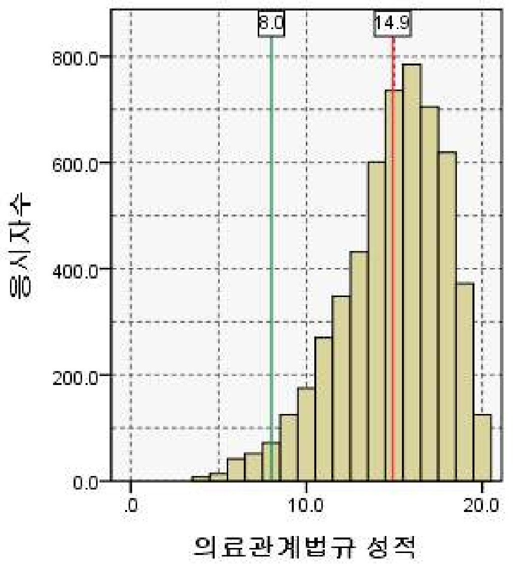

총점	과락선	평균성적	표준편차
20	8	14.9	3.0

나) 치위생학

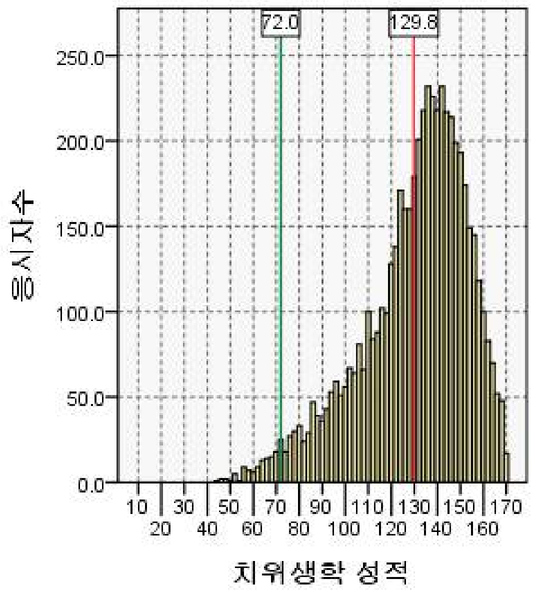

총점	과락선	평균성적	표준편차
180	72	129.8	23.2

2. 난이도와 변별도

1) 전체 난이도와 변별도

가) 전회 대비 전체 난이도와 변별도

회차	난이도		변별도1		변별도2	
	평균	표준편차	평균	표준편차	평균	표준편차
제46회	69.3	19.9	.25	.11	.25	.09
제47회	72.8	17.2	.26	.12	.27	.10
제48회	67.3	19.7	.28	.13	.26	.09
제49회	71.3	17.7	.29	.12	.29	.10
제50회	72.3	17.3	.29	.12	.30	.10

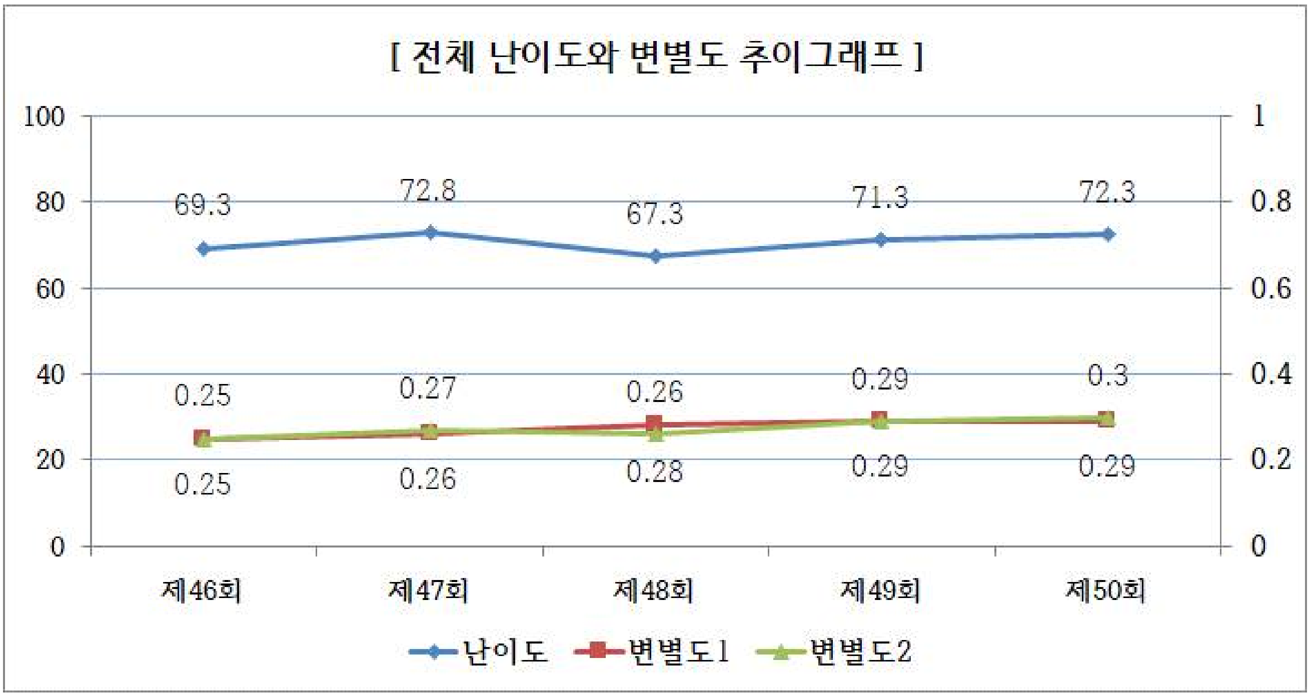

해석
- 전년 대비 난이도 지수는 1.0 증가함<br>- 변별도 1 지수는 변하지 않음<br>- 변별도 2 지수는 .01 증가함

나) 전체 난이도와 변별도 분포도 및 비율분석

(1) 전체 난이도 분포도 및 비율분석

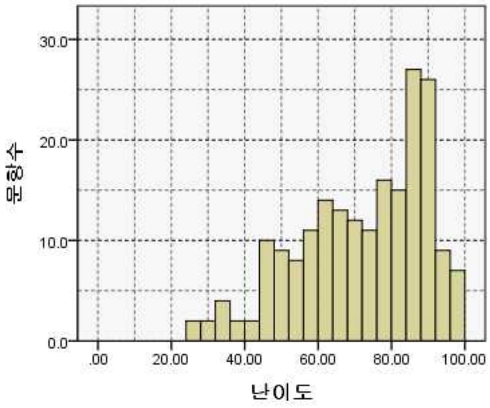

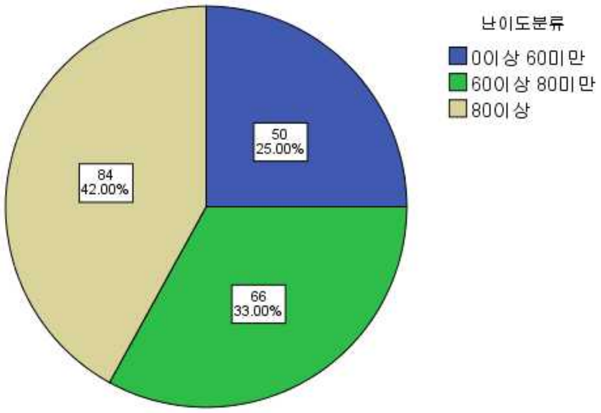

총점	난이도	표준편차
200	72.3	17.3

난이도	문항수	비율(%)
0~60미만	50	25.0
60~80미만	66	33.0
80~100	84	42.0
전체	200	100.0

(2) 전체 변별도1 분포도 및 비율분석

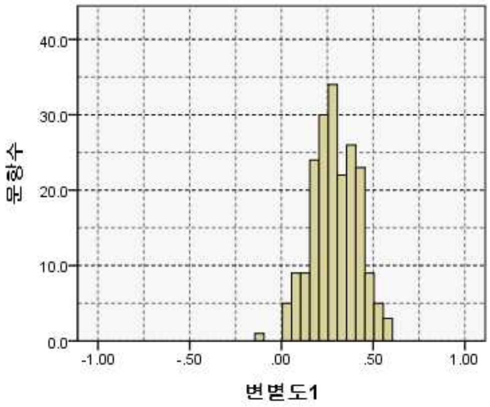

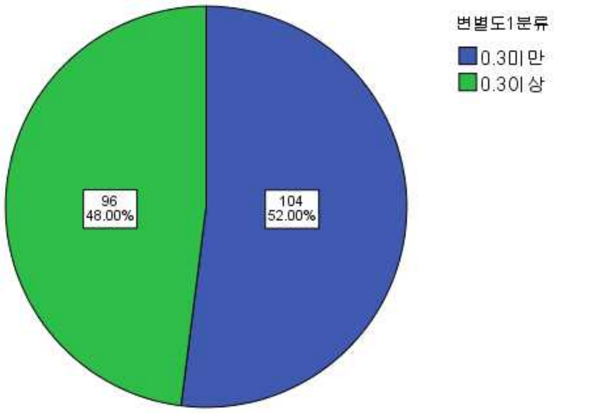

총점	변별도1	표준편차
200	.29	.12

변별도1	문항수	비율(%)
0.3미만	104	52.0
0.3이상	96	48.0
전체	200	100.0

(3) 전체 변별도2 분포도 및 비율분석

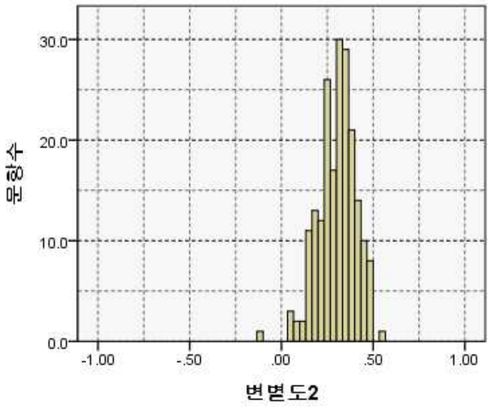

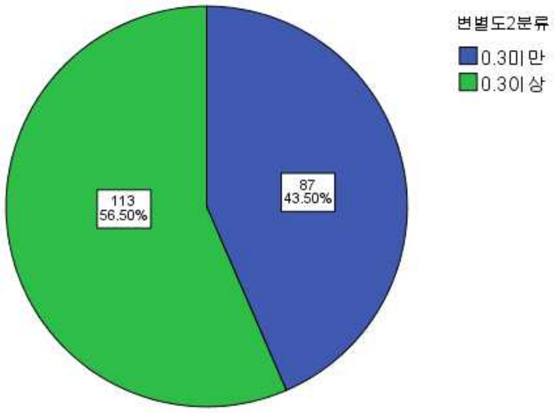

총점	변별도2	표준편차
200	.30	.10

변별도2	문항수	비율(%)
0.3미만	87	43.5
0.3이상	113	56.5
전체	200	100.0

해석

- 난이도 지수가 80 에서 100 사이인 문항이 전체 200 문항 중 84 문항이었으며, 60 이상 80 미만인 문항이 66 문항, 60 미만인 문항이 50 문항인 것으로 나타남
- 변별도 1 지수를 기준으로 분류하였을 때, 0.3 미만인 문항이 104 문항으로 0.3 이상인 문항이 96 문항인 것에 비해 더 많이 나타남
- 변별도 2 지수를 기준으로 분류하였을 때, 0.3 미만인 문항이 87 문항으로 0.3 이상인 문항이 113 문항인 것에 비해 더 적게 나타남

2) 과목별 난이도와 변별도

가) 전회 대비 과목별 난이도와 변별도

(1) 전회 대비 의료관계법규 난이도와 변별도

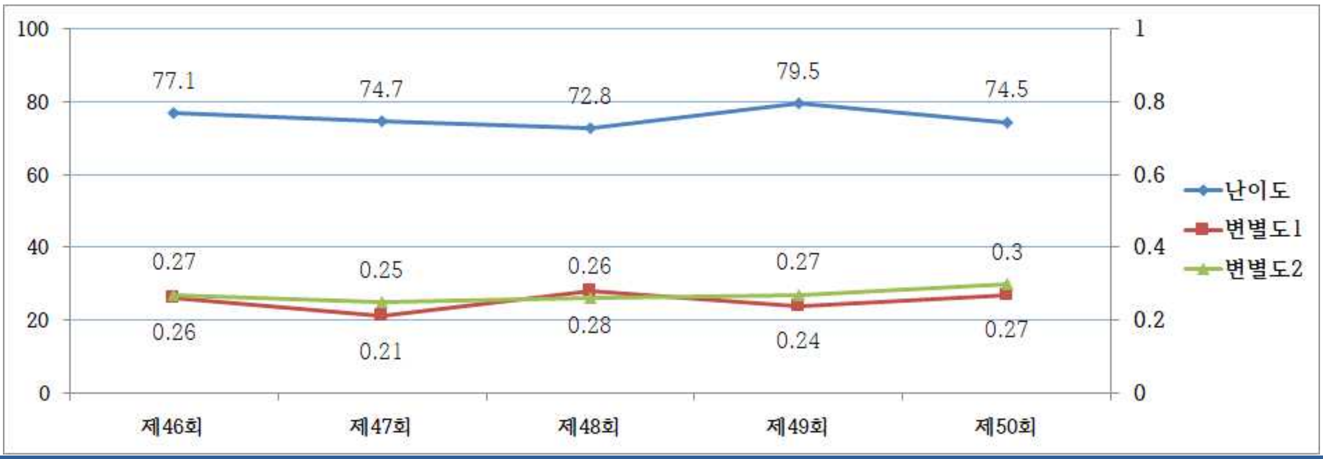

회차	난이도		변별도1		변별도2	
	평균	표준편차	평균	표준편차	평균	표준편차
제46회	77.1	12.8	.26	.12	.27	.09
제47회	74.7	20.3	.21	.10	.25	.11
제48회	72.8	18.0	.28	.16	.26	.11
제49회	79.5	16.0	.24	.12	.27	.09
제50회	74.5	18.9	.27	.12	.30	.09

해석

- 전회 대비 의료관계법규 과목의 난이도 지수는 5.0 감소함
- 전회 대비 의료관계법규 과목의 변별도 1 지수는 .03 증가함
- 전회 대비 의료관계법규 과목의 변별도 2 지수는 .03 증가함

(2) 전회 대비 치위생학 난이도와 변별도

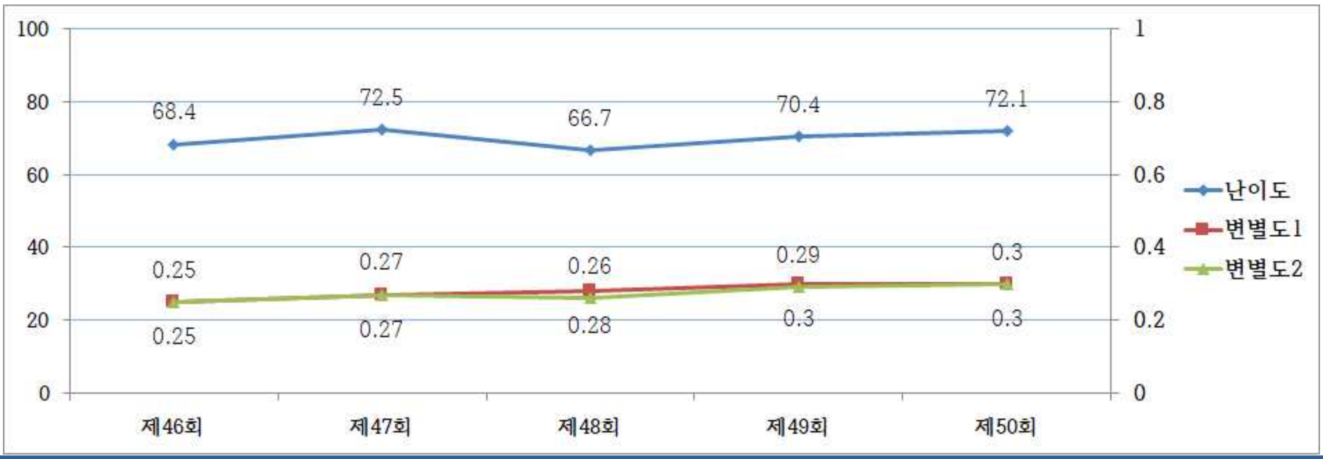

회차	난이도		변별도1		변별도2	
	평균	표준편차	평균	표준편차	평균	표준편차
제46회	68.4	20.4	.25	.11	.25	.09
제47회	72.5	16.8	.27	.12	.27	.10
제48회	66.7	19.8	.28	.12	.26	.09
제49회	70.4	17.7	.30	.12	.29	.10
제50회	72.1	17.2	.30	.12	.30	.10

해석

- 전회 대비 치위생학 과목의 난이도 지수는 1.7 증가함
- 전회 대비 치위생학 과목의 변별도 1 지수는 변하지 않음
- 전회 대비 치위생학 과목의 변별도 2 지수는 .01 증가함

나) 과목별 난이도와 변별도 분포도 및 비율분석

(1) 의료관계법규 난이도와 변별도 분포도 및 비율분석

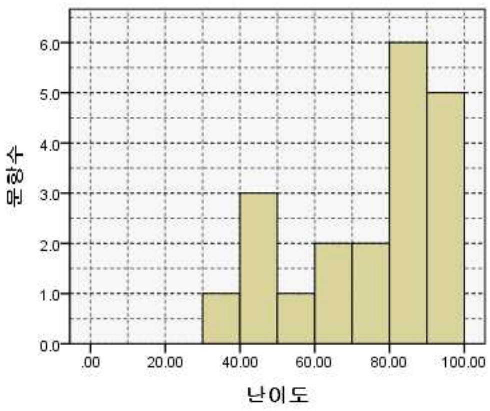

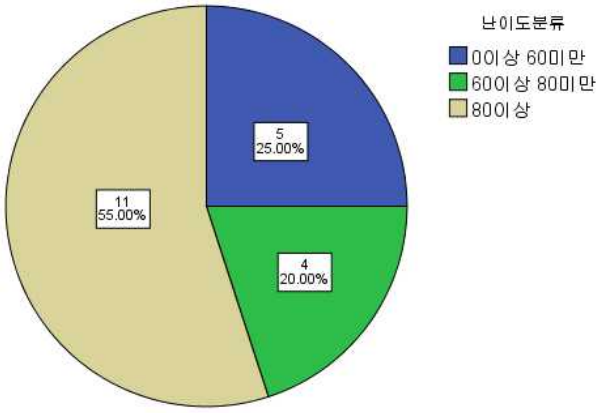

총점	난이도	표준편차	난이도	문항수	비율(%)
20	74.5	18.9	0~60미만	5	25.0
			60~80미만	4	20.0
			80~100	11	55.0
			전체	20	100.0

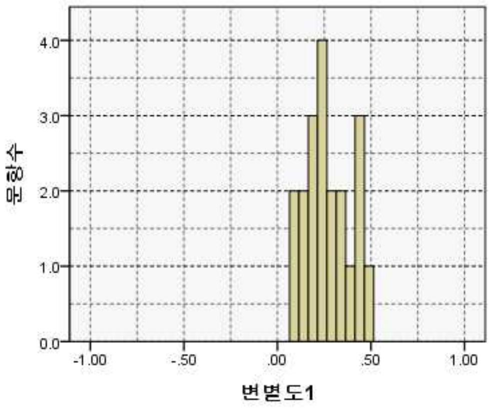

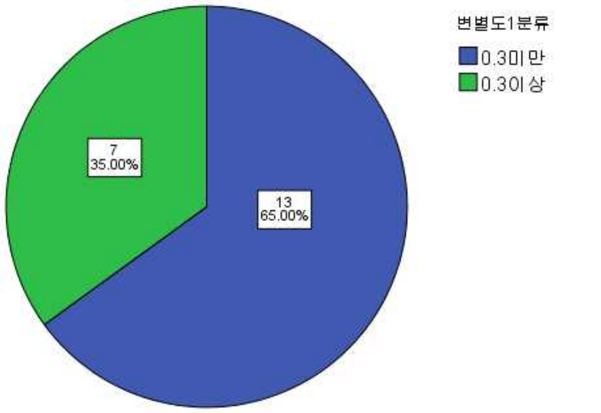

총점	변별도1	표준편차	변별도1	문항수	비율(%)
20	.27	.12	0.3미만	13	65.0
			0.3이상	7	35.0
			전체	20	100.0

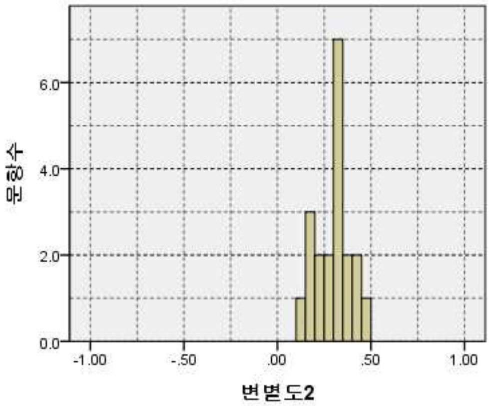

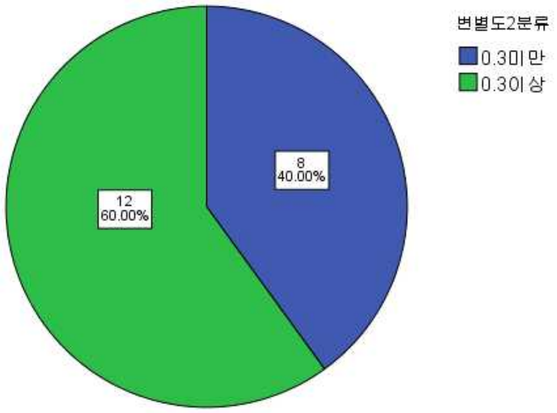

총점	변별도2	표준편차
20	.30	.09

변별도2	문항수	비율(%)
0.3미만	8	40.0
0.3이상	12	60.0
전체	20	100.0

해석

- 의료관계법규 과목에서 난이도 지수가 80에서 100 사이인 문항이 전체 20문항 중 11문항이었으며, 60이상 80미만인 문항이 4문항, 60 미만인 문항이 5문항으로 나타남
- 변별도1 지수를 기준으로 분류하였을 때, 0.3미만인 문항이 13문항으로 0.3 이상인 문항이 7문항인 것에 비해 더 많이 나타남
- 변별도2 지수를 기준으로 분류하였을 때, 0.3미만인 문항이 8문항으로 0.3 이상인 문항이 12문항인 것에 비해 더 적게 나타남

(2) 치위생학 난이도와 변별도 분포도 및 비율분석

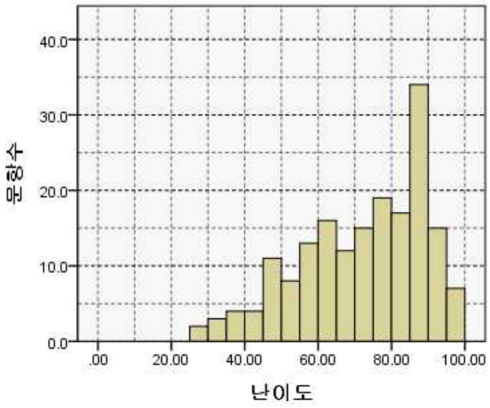

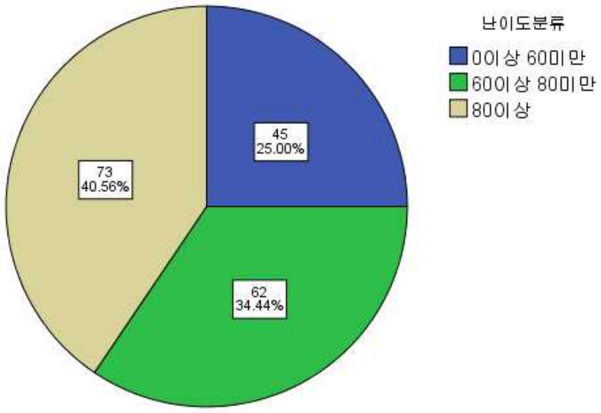

총점	난이도	표준편차
180	72.1	17.2

난이도	문항수	비율(%)
0~60미만	45	25.0
60~80미만	62	34.4
80~100	73	40.6
전체	180	100.0

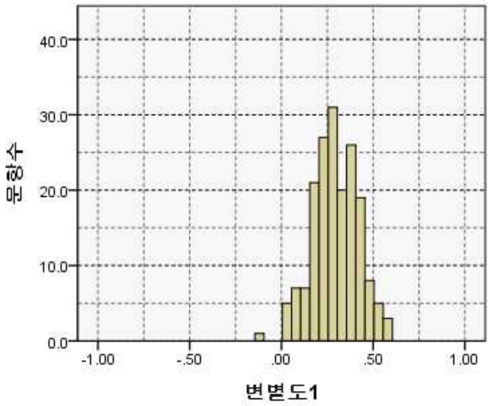

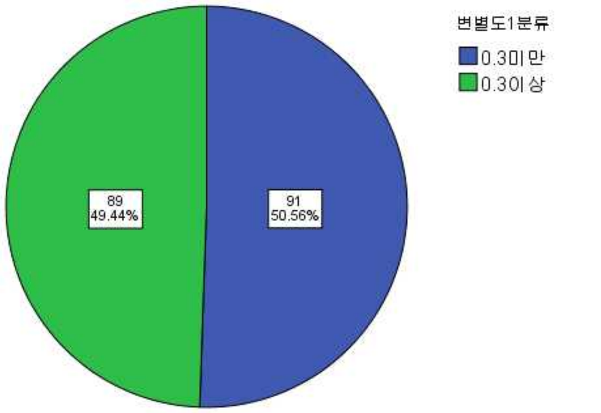

총점	변별도1	표준편차
180	.30	.12

변별도1	문항수	비율(%)
0.3미만	91	50.6
0.3이상	89	49.4
전체	180	100.0

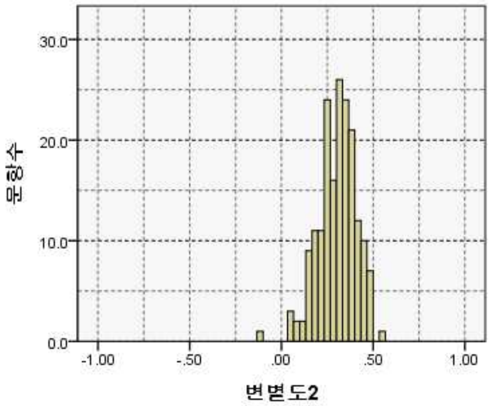

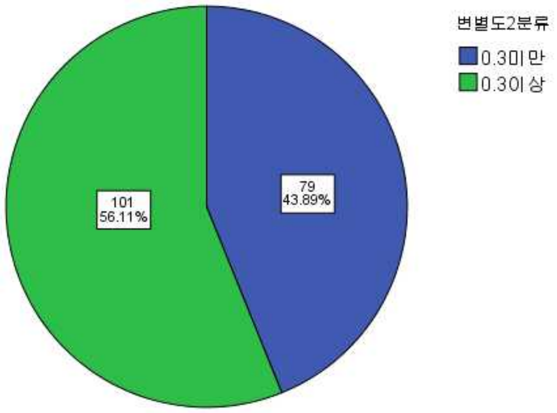

총점	변별도2	표준편차
180	.30	.10

변별도2	문항수	비율(%)
0.3미만	79	43.9
0.3이상	101	56.1
전체	180	100.0

해석

- 치위생학 과목에서 난이도 지수가 80 에서 100 사이인 문항이 전체 180 문항 중 73 문항이었으며, 60 이상 80 미만인 문항이 62 문항, 60 미만인 문항이 45 문항으로 나타남
- 변별도 1 지수를 기준으로 분류하였을 때, 0.3 미만인 문항이 91 문항으로 0.3 이상인 문항이 89 문항인 것에 비해 더 많이 나타남
- 변별도 2 지수를 기준으로 분류하였을 때, 0.3 미만인 문항이 79 문항으로 0.3 이상인 문항이 101 문항인 것에 비해 더 적게 나타남

3) 지식수준별 난이도와 변별도

가) 전회 대비 지식수준별 난이도와 변별도

(1) 전회 대비 암기형 난이도와 변별도

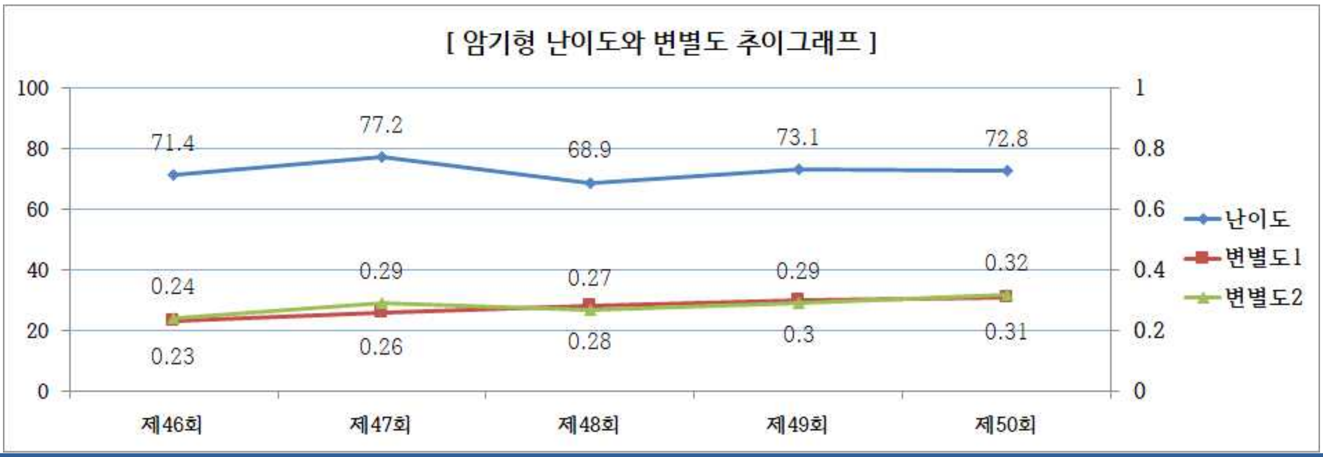

회차	난이도		변별도1		변별도2	
	평균	표준편차	평균	표준편차	평균	표준편차
제46회	71.4	20.2	.23	.11	.24	.09
제47회	77.2	13.2	.26	.12	.29	.10
제48회	68.9	19.6	.28	.13	.27	.10
제49회	73.1	15.4	.30	.11	.29	.09
제50회	72.8	17.4	.31	.13	.32	.10

(2) 전회 대비 해석형 난이도와 변별도

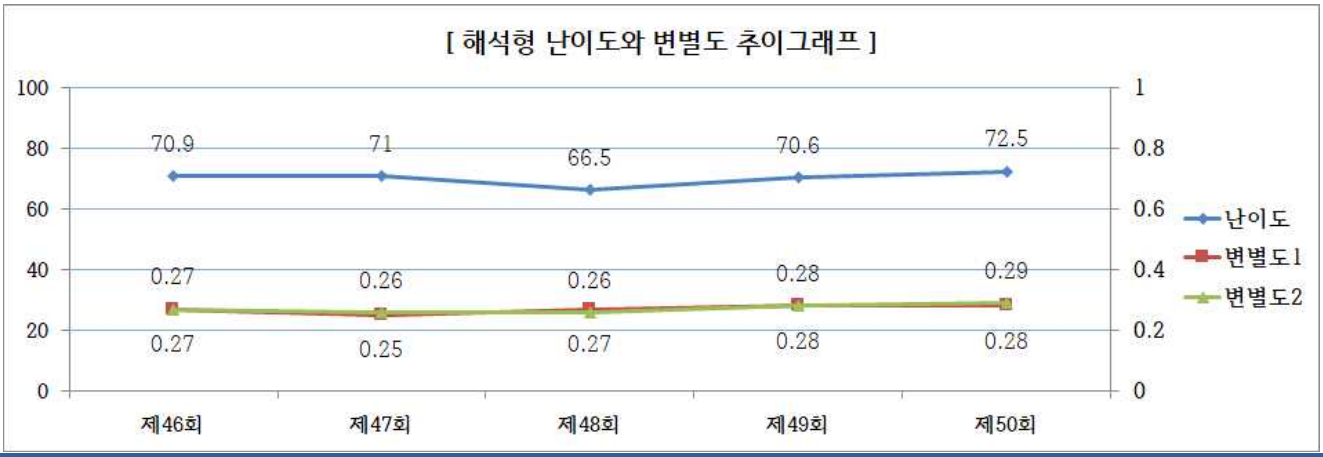

회차	난이도		변별도1		변별도2	
	평균	표준편차	평균	표준편차	평균	표준편차
제46회	70.9	18.0	.27	.11	.27	.09
제47회	71.0	18.8	.25	.12	.26	.10
제48회	66.5	20.0	.27	.13	.26	.10
제49회	70.6	19.1	.28	.13	.28	.10
제50회	72.5	17.3	.28	.12	.29	.10

(3) 전회 대비 해결형 난이도와 변별도

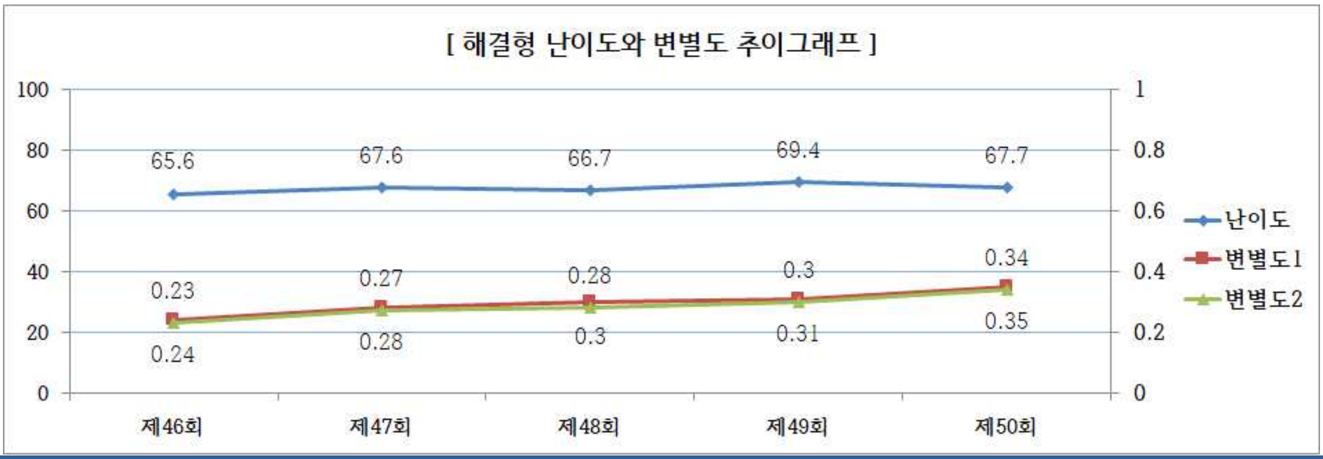

회차	난이도		변별도1		변별도2	
	평균	표준편차	평균	표준편차	평균	표준편차
제46회	65.6	21.5	.24	.12	.23	.09
제47회	67.6	17.2	.28	.10	.27	.10
제48회	66.7	18.8	.30	.11	.28	.08
제49회	69.4	17.1	.31	.12	.30	.09
제50회	67.7	18.4	.35	.13	.34	.12

해석

- 전회 대비 암기형 문항의 난이도 지수는 0.3 감소하였으며, 해석형 문항의 난이도 지수는 1.9 증가하였고, 해결형 문항의 난이도 지수는 1.7 감소함
- 변별도 1 지수는 암기형 문항에서 .01 증가하였으며 해석형 문항에서는 변하지 않았고, 해결형 문항에서는 .04 증가함
- 변별도 2 지수는 암기형 문항에서 .03 증가하였으며 해석형 문항에서는 .01 증가하였고 해결형 문항에서는 .04 증가함

나) 지식수준별 난이도와 변별도 분포도 및 비율분석

(1) 암기형 난이도와 변별도 분포도 및 비율분석

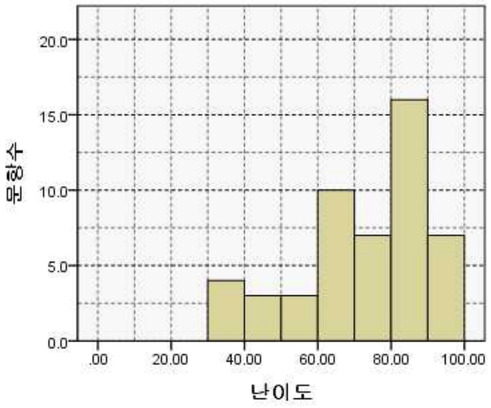

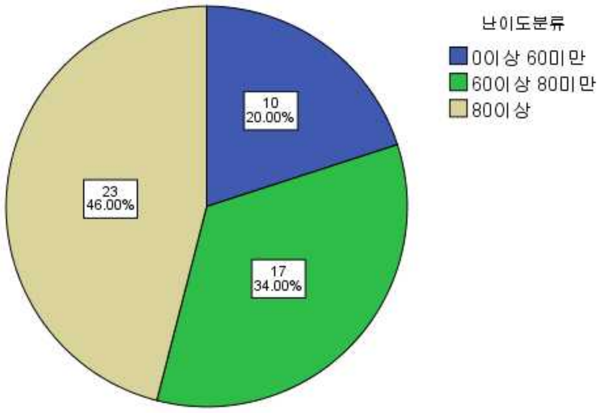

총점	난이도	표준편차
50	72.8	17.4

난이도	문항수	비율(%)
0~60미만	10	20.0
60~80미만	17	34.0
80~100	23	46.0
전체	50	100.0

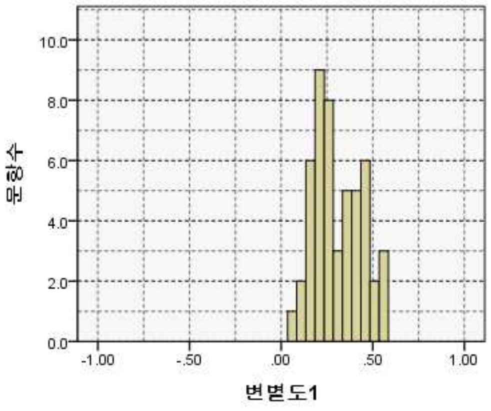

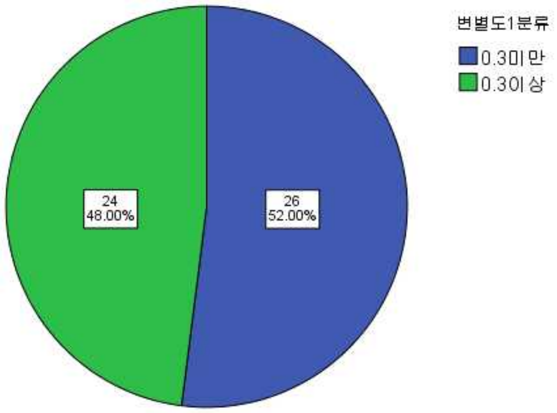

총점	변별도1	표준편차
50	.31	.13

변별도1	문항수	비율(%)
0.3미만	26	52.0
0.3이상	24	48.0
전체	50	100.0

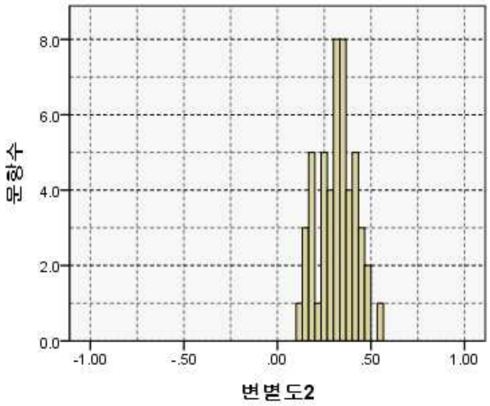

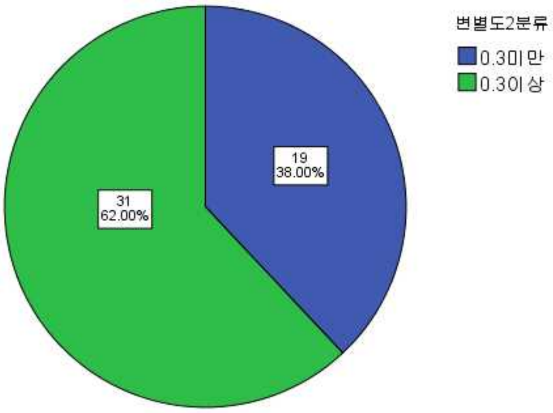

총점	변별도2	표준편차
50	.32	.10

변별도2	문항수	비율(%)
0.3미만	19	38.0
0.3이상	31	62.0
전체	50	100.0

해석

- 암기형 문항에서 난이도 지수가 80 에서 100 사이인 문항이 전체 50 문항 중 23 문항이었으며, 60 이상 80 미만인 문항이 17 문항, 60 미만인 문항이 10 문항인 것으로 나타남
- 변별도 1 지수를 기준으로 분류하였을 때, 0.3 미만인 문항이 26 문항으로 0.3 이상인 문항이 24 문항인 것에 비해 더 많이 나타남
- 변별도 2 지수를 기준으로 분류하였을 때, 0.3 미만인 문항이 19 문항으로 0.3 이상인 문항이 31 문항인 것에 비해 더 적게 나타남

(2) 해석형 난이도와 변별도 분포도 및 비율분석

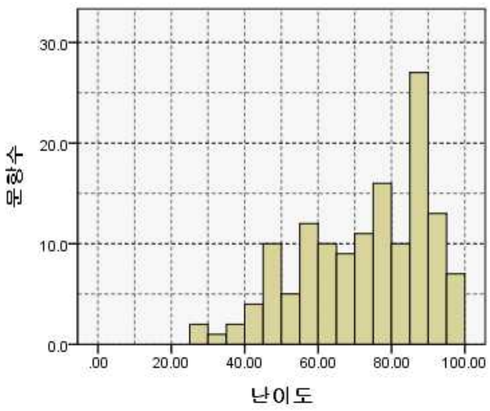

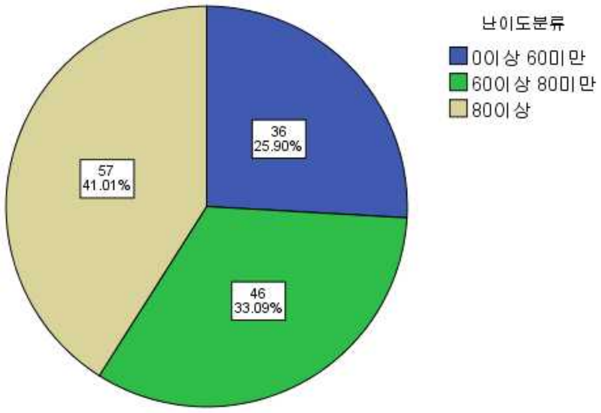

총점	난이도	표준편차
139	72.5	17.3

난이도	문항수	비율(%)
0~60미만	36	25.9
60~80미만	46	33.1
80~100	57	41.0
전체	139	100.0

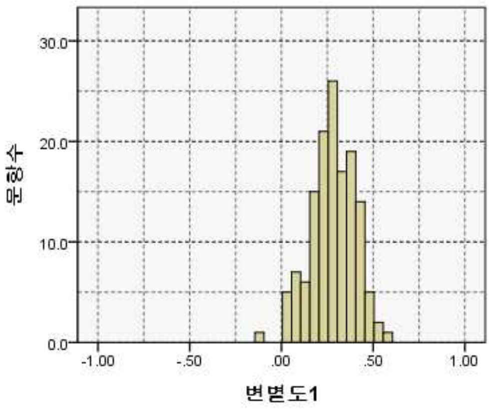

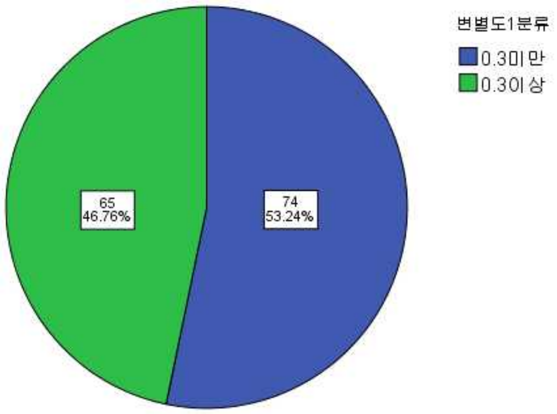

총점	변별도1	표준편차
139	.28	.12

변별도1	문항수	비율(%)
0.3미만	74	53.2
0.3이상	65	46.8
전체	139	100.0

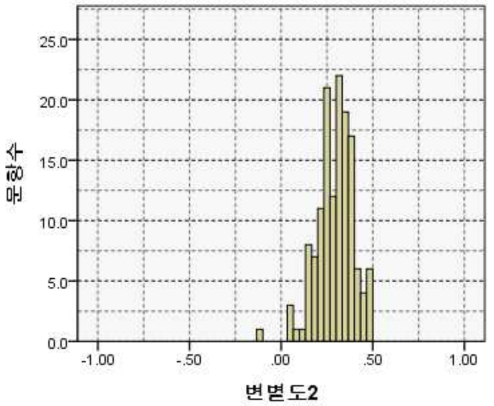

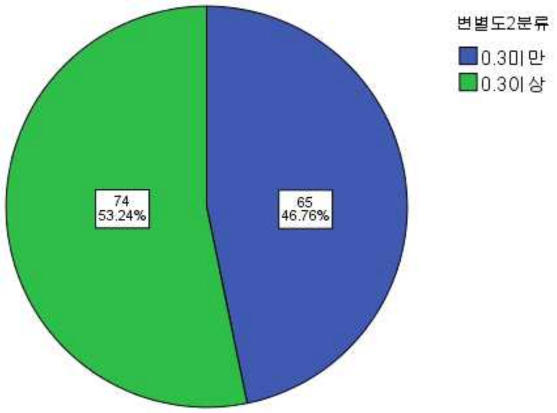

총점	변별도2	표준편차
139	.29	.10

변별도2	문항수	비율(%)
0.3미만	65	46.8
0.3이상	74	53.2
전체	139	100.0

해석

- 해석형 문항에서 난이도 지수가 80 에서 100 사이인 문항이 전체 139 문항 중 57 문항이었으며, 60 이상 80 미만인 문항이 46 문항, 60 미만인 문항이 36 문항인 것으로 나타남
- 변별도 1 지수를 기준으로 분류하였을 때, 0.3 미만인 문항이 74 문항으로 0.3 이상인 문항이 65 문항인 것에 비해 더 많이 나타남
- 변별도 2 지수를 기준으로 분류하였을 때, 0.3 미만인 문항이 65 문항으로 0.3 이상인 문항이 74 문항인 것에 비해 더 적게 나타남

(3) 해결형 난이도와 변별도 분포도 및 비율분석

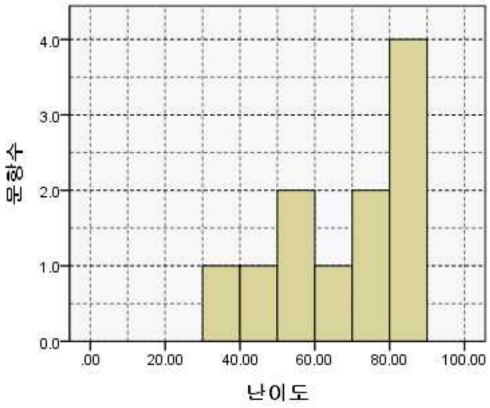

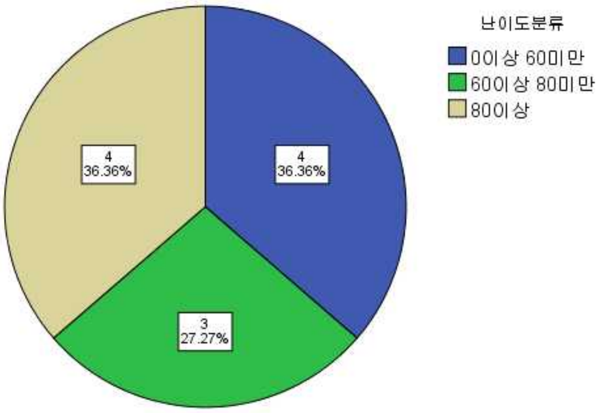

총점	난이도	표준편차
11	67.7	18.4

난이도	문항수	비율(%)
0~60미만	4	36.4
60~80미만	3	27.3
80~100	4	36.4
전체	11	100.0

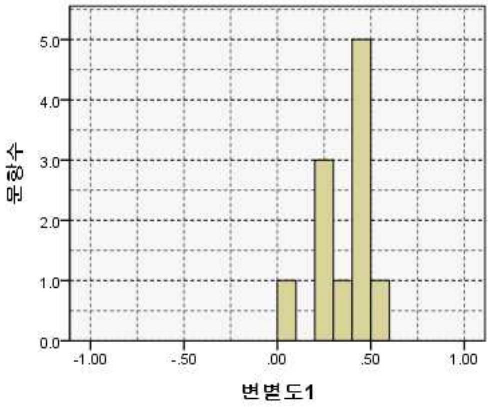

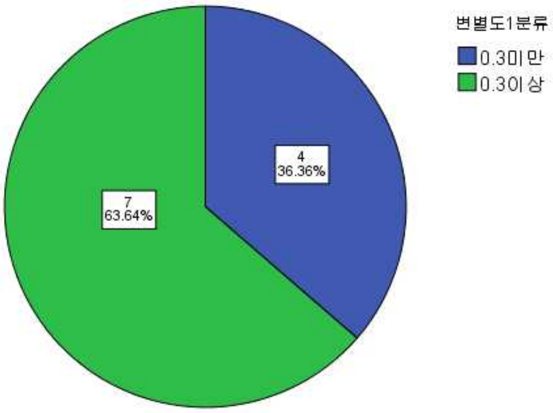

총점	변별도1	표준편차
11	.35	.13

변별도1	문항수	비율(%)
0.3미만	4	36.4
0.3이상	7	63.6
전체	11	100.0

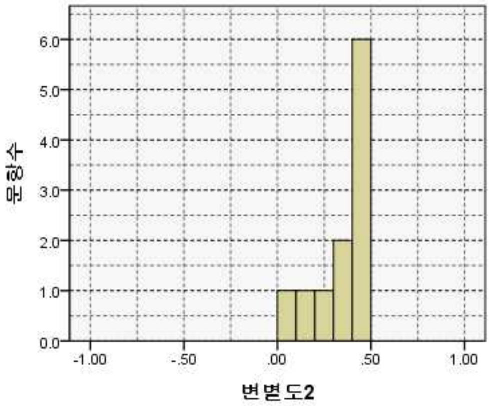

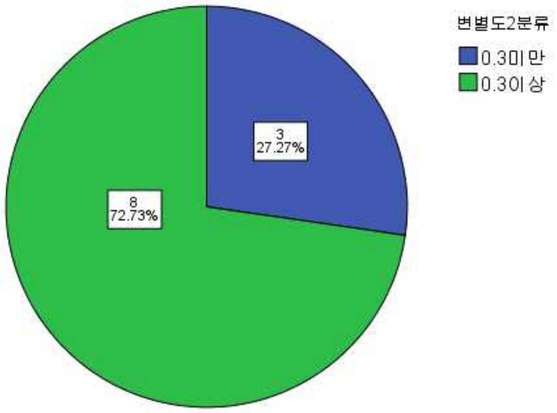

총점	변별도2	표준편차
11	.34	.12

변별도2	문항수	비율(%)
0.3미만	3	27.3
0.3이상	8	72.7
전체	11	100.0

해석

- 해결형 문항에서 난이도 지수가 80 에서 100 사이인 문항이 전체 11 문항 중 4 문항이었으며, 60 이상 80 미만인 문항이 3 문항, 60 미만인 문항이 4 문항인 것으로 나타남
- 변별도 1 지수를 기준으로 하였을 때, 0.3 미만인 문항이 4 문항으로 0.3 이상인 문항이 7 문항인 것에 비해 더 적게 나타남
- 변별도 2 지수를 기준으로 분류하였을 때, 0.3 미만인 문항이 3 문항으로 0.3 이상인 문항이 8 문항인 것에 비해 더 적게 나타남

3. 난이도와 변별도 간 산포도

1) 전체 난이도와 변별도 간 산포도

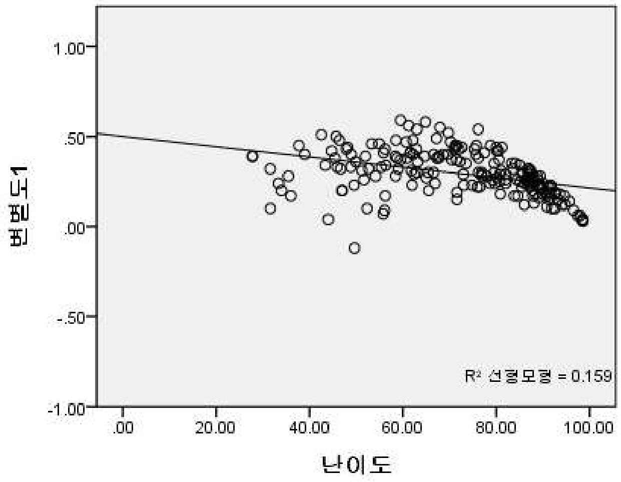

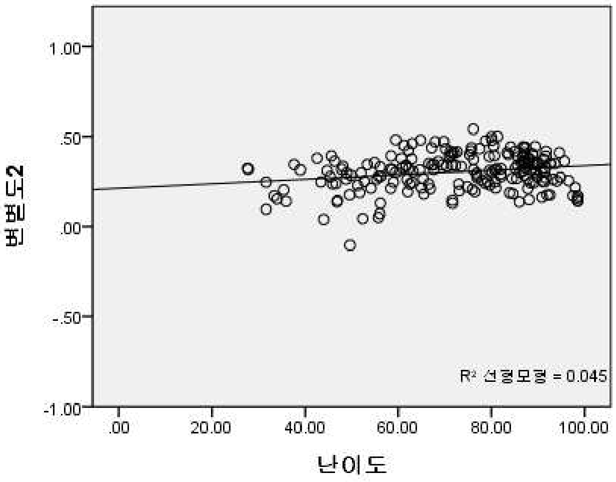

해석

- 난이도 지수와 변별도1 지수 간 상관은 -.399*로 난이도 지수가 높을수록 변별력이 낮아지는 것으로 나타남
- 난이도 지수와 변별도2 지수 간 상관은 .211*로 난이도 지수가 높을수록 변별력이 높아지는 것으로 나타남

2) 과목별 난이도와 변별도 간 산포도

가) 의료관계법규 난이도와 변별도 간 산포도

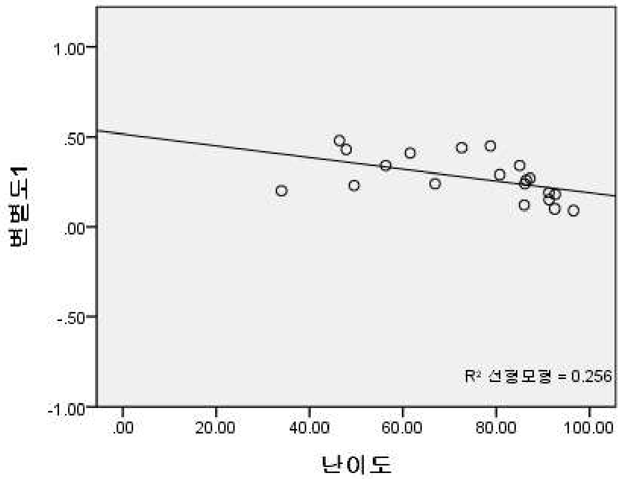

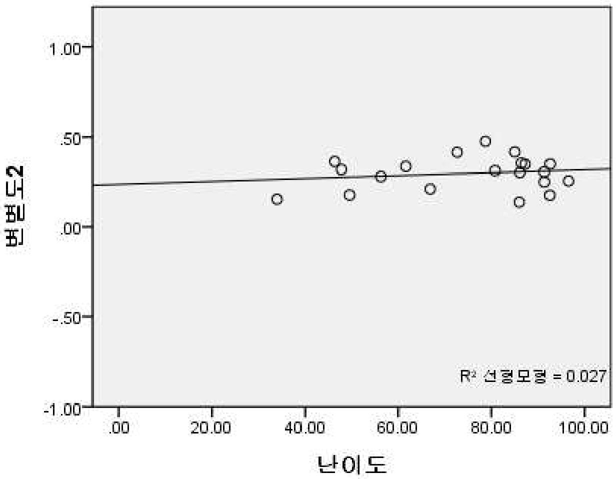

해석

- 난이도 지수와 변별도1 지수 간 상관은 -.506*로 난이도 지수가 높을수록 변별력이 낮아지는 것으로 나타남
- 난이도 지수와 변별도2 지수 간 상관은 -.165로 관련성이 없는 것으로 나타남

나) 치위생학 난이도와 변별도 간 산포도

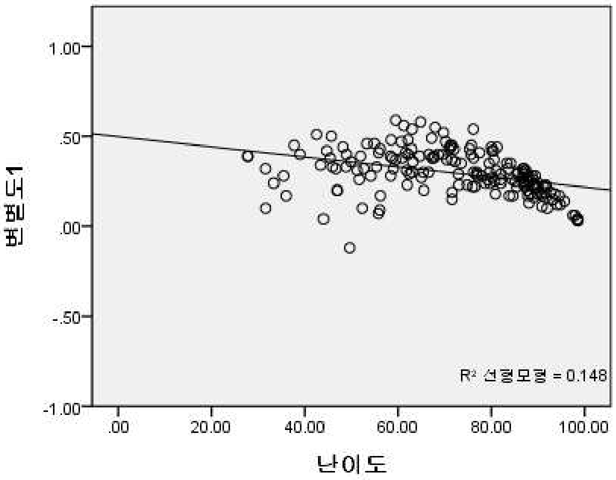

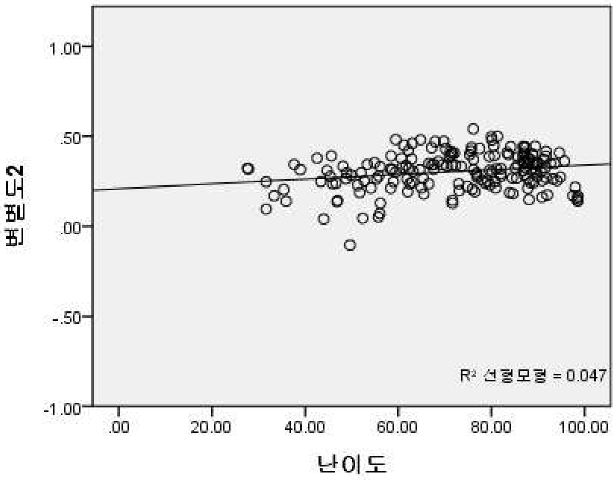

해석

- 난이도 지수와 변별도1 지수 간 상관은 -.385*로 난이도 지수가 높을수록 변별력이 낮아지는 것으로 나타남
- 난이도 지수와 변별도2 지수 간 상관은 -.218*로 난이도 지수가 높을수록 변별력이 높아지는 것으로 나타남

4. 신뢰도 분석

과목명	문항수	제46회	제47회	제48회	제49회	제50회
전체	200	.927	.942	.939	.949	.952
의료관계법규	20	.683	.604	.679	.677	.688
치위생학	180	.927	.938	.932	.944	.948

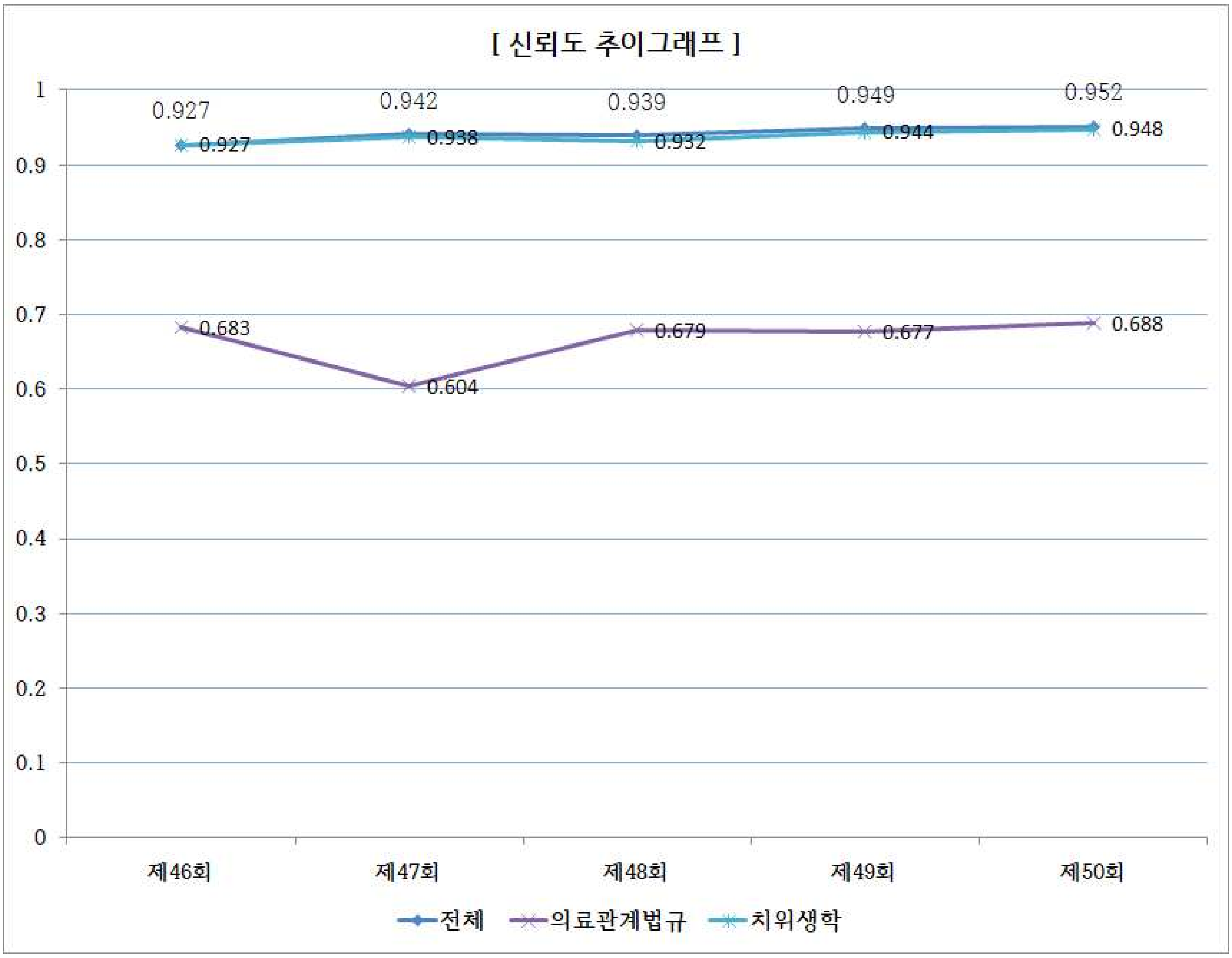

해석

- 전회 대비 전체 문항의 신뢰도는 .003 증가함
- 전회 대비 의료관계법규 과목 문항의 신뢰도는 .011 증가함
- 전회 대비 치위생학 과목 문항의 신뢰도는 .004 증가함

○ 분석결과 관련 문의 : 한국보건의료인국가시험원 연구개발본부 김준기 전임연구원
Tel : 02-2087-8956, FAX : 02-2087-8885
E-mail : tontates@kuksiwon.or.kr
