# Supplementary material for: Item difficulty index, discrimination index, and reliability of the 26 health professions licensing examinations in 2022, Korea: a psychometric study
Source: J Educ Eval Health Prof. 2023 Nov 22;20:31. doi: 10.3352/jeehp.2023.20.31 (PMC11959405; doi:10.3352/jeehp.2023.20.31)
Supplement: Supplementary file 1 — Supplement 1. Item analysis results of 26 health professions licensing examinations administered during late 2022 and early 2023. [file jeehp-20-31_Suppl1.zip › 2022│Γ╡╡ ┴a62╚╕ ░ú╚ú╗τ ▒╣░í╜├╟Φ ║╨╝«░ß░·.pdf]

2022년도 제62회
간호사 국가시험
문항분석 결과

국민이 신뢰하고 감동하는 시험평가기관

한국보건의료인국가시험원

KOREA HEALTH PERSONNEL LICENSING EXAMINATION INSTITUTE

일반 용어 정의

◗ **평균**

- 집단에서의 대표적 경향값으로 전체 값을 더하여 총 응시자로 나눈 값

◗ **표준편차**

- 평균과 각 점수의 차이인 편차들의 평균으로 점수가 흩어져 분포되어 있는 정도

◗ **추정난이도**

- 문항개발자가 예측한 정답률

◗ **검사이론**

- 검사와 검사를 구성하고 있는 문항의 양호도를 분석 및 평가하는 방법을 정의한 이론체계
- 대표적으로 고전검사이론과 문항반응이론이 있음

고전검사이론 용어 정의

◗ **고전검사이론(Classical Test Theory; CTT)**

- 검사의 질을 분석하는 검사이론 중 한 가지로 19세기 말부터 전개되어 현재까지 주로 사용되고 있는 검사이론임
- 고전검사이론에 의한 문항과 응시자 능력 추정치는 다음과 같음

◗ **문항난이도**

- 검사 문항의 쉽고 어려운 정도를 나타내는 지수
- 난이도 지수는 총 반응 수에 대한 정답 반응 수의 비율로 문항의 정답률임
- 문항난이도는 0~100까지의 값을 가짐
- 난이도 값이 큰 경우, 쉬운 문항으로 '난이도가 낮다'라고 해석하며, 난이도 값이 작은 경우, 어려운 문항으로 '난이도가 높다'라고 해석함

◗ **문항변별도**

- 각 문항이 응시자의 능력 수준을 변별할 수 있는 정도를 나타내는 지수
- 문항변별도는 -1~+1까지의 값을 가지며, 1에 가까울수록 변별력 크다고 해석함
- 일반적으로 문항변별도가 0.3이상이면 우수한 문항으로 평가함
- 구하는 방식에는 '상하위집단 구분법', '문항-총점 상관계수' 등이 있음
  1) **변별도 1(상하위구분법)**: 상위 27%와 하위 27% 집단의 난이도 차이를 구하는 방식
  2) **변별도 2(상관계수법)**: 문항-총점과의 상관계수로 구하는 방식

◗ **신뢰도**

- 시험이 평가하고자 하는 것을 일관성 있게 측정하는가로 시험이 오차없이 정확하게 측정한 정도를 의미함
- 국시원에서는 문항의 내적일관성(Cronbach α)으로 신뢰도를 추정하며 1에 가까울수록 신뢰도가 높다고 해석함

목 차

I. 시행 결과 ········ 6

1. 시험 현황 ········ 7

1) 시험명 ········ 7
2) 시험시행일 ········ 7
3) 응시현황 ········ 7
4) 과목별 문항 수, 배점 및 과락 점수 ········ 7

2. 합격률과 평균성적 ········ 7

1) 합격 및 불합격 현황 ········ 7
2) 과목별 과락자수 내역 ········ 8
3) 전회 대비 합격률과 평균성적 ········ 8

II. 문항분석 결과 ········ 9

1. 성적 ········ 10

1) 전체 성적분포도 ········ 10
2) 과목별 성적분포도 ········ 11

2. 난이도와 변별도 ········ 13

1) 전체 난이도와 변별도 ········ 13
2) 과목별 난이도와 변별도 ········ 16
3) 지식수준별 난이도와 변별도 ········ 37
4) 자료유형별 난이도와 변별도 ········ 45

3. 난이도와 변별도 간 산포도 ········ 51

1) 전체 난이도와 변별도 간 산포도 ········ 51
2) 과목별 난이도와 변별도 간 산포도 ········ 51

4. 신뢰도 분석 ········ 56

I. 시행 결과

1. 시험 현황

1) 시험명: 2022년도 제62회 간호사 국가시험

2) 시험시행일: 2022년 1월 21일

3) 응시현황

응시대상자수	결시자수	부정행위자수	응시자 준수사항 위반자 수		응시자수 (%)
			휴대폰 소지	신분증 미지참	
24,367	191	0	0	0	24,175※ (99.2)

※ 전체 응시자수(24,367명)에서 결시자수(191명) 및 채점보류자수(1명)을 제외한 수

4) 과목별 문항 수, 배점 및 과락 점수

교 시	과 목 명	문제 수	배점	총점	합격자 점수기준	
					과목 과락기준	총점 합격기준
1교시	성인간호학	70	1	70	28점 미만	177점 이상
	모성간호학	35	1	35	14점 미만	
2교시	아동간호학	35	1	35	14점 미만	
	지역사회간호학	35	1	35	14점 미만	
	정신간호학	35	1	35	14점 미만	
3교시	간호관리학	35	1	35	14점 미만	
	기본간호학	30	1	30	12점 미만	
	보건의약관계 법규	20	1	20	8점 미만	
계		295		295		

2. 합격률과 평균성적

1) 합격 및 불합격 현황

합격자수 (%)	불합격자수(%)				채점보류자수
	평락	과락	기권	계	
23,363 (96.6)	691 (2.9)	118 (0.5)	3 (0.0)	812 (3.4)	1 (0.0)

2) 과목별 과락자수 내역

과목명 / 과락자수	성인간호학	모성간호학	아동간호학	지역사회 간호학	정신간호학	간호관리학	기본간호학	보건의약 관계 법규
과목별 과락자 수	-	1	-	20	2	1	-	94
전과목 과락자 수	-							

3) 전회 대비 합격률과 평균성적

회차	년도	합격률(%)	평균성적	표준편차	백분율 환산점수
제58회	2018	96.1	228.7	24.0	77.5
제59회	2019	96.4	230.7	23.9	78.2
제60회	2020	96.2	228.4	23.2	77.4
제61회	2021	94.8	228.5	26.3	77.3
**제62회**	2022	96.6	232.8	23.3	78.9

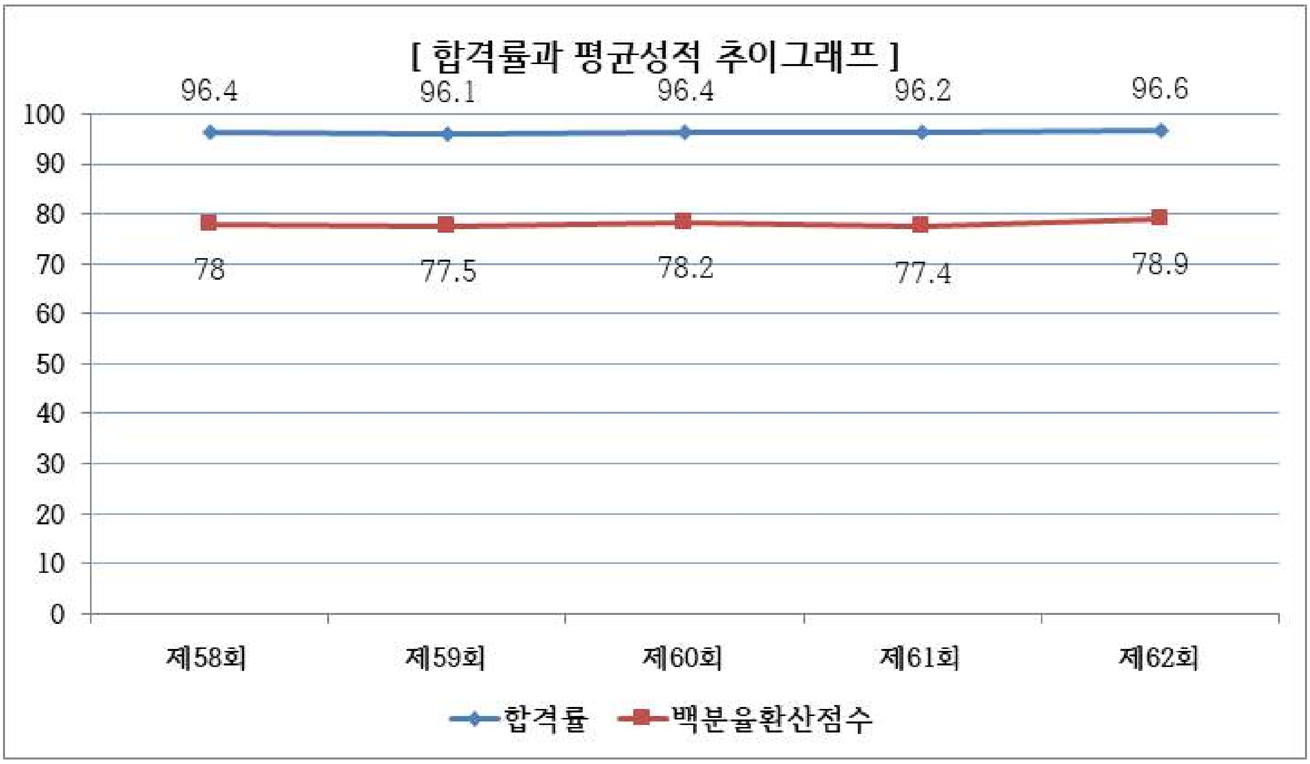

해석

- 전년 대비 합격률은 1.8, 백분율 환산점수는 1.6 점 증가함

II. 문항분석 결과

1. 성적

1) 전체 성적분포도

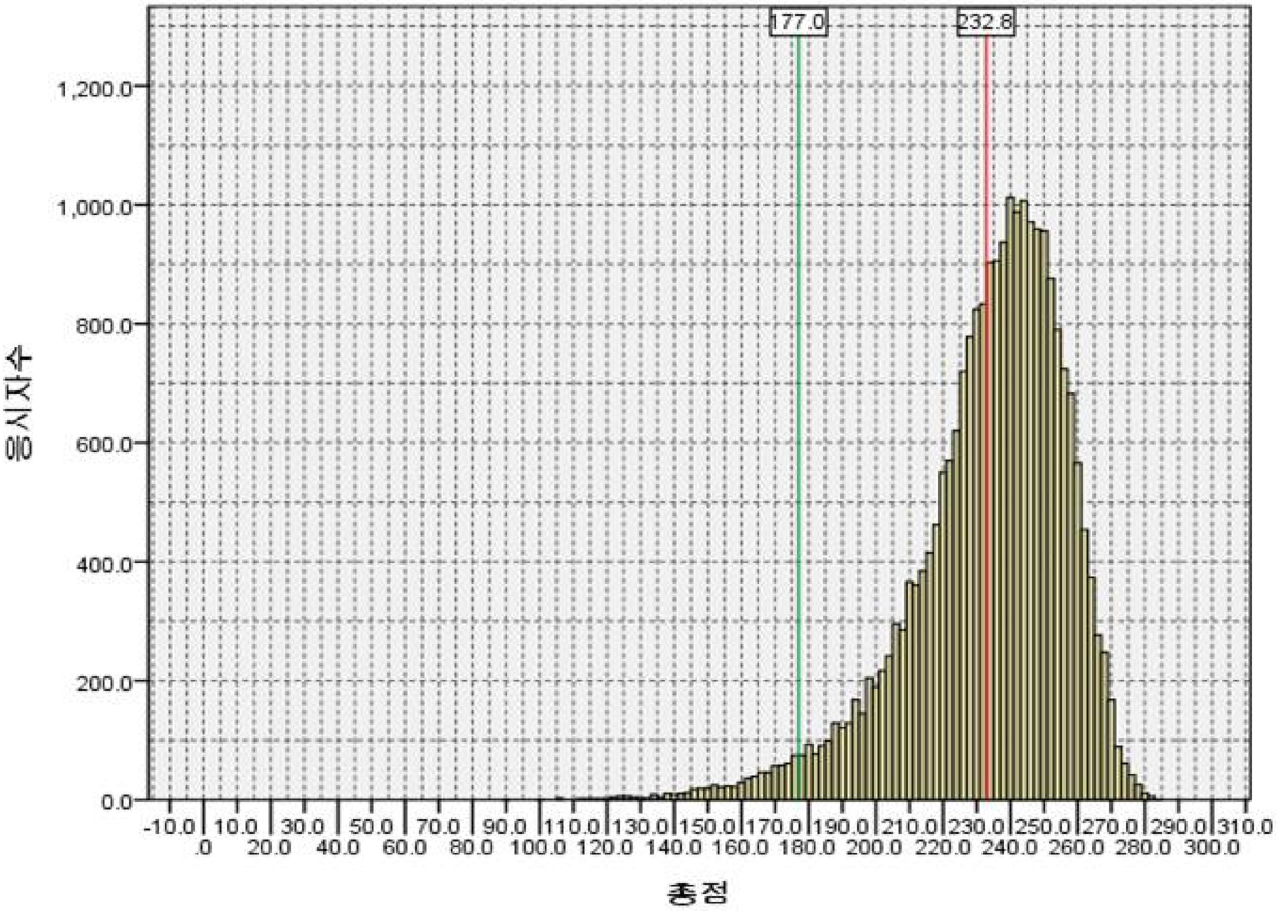

응시자	총점	합격선	평균성적	표준편차
24,173※	295	177	232.8	23.3

※ 24,173명은 전체응시자(24,175명)에서 채점보류자수(1명)을 더하고 기권자(3명)는 제외한 수치임

2) 과목별 성적분포도

가) 성인간호학

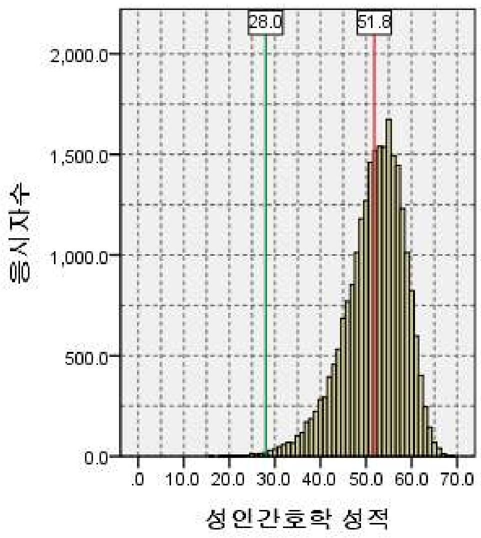

총점	과락선	평균성적	표준편차
70	28	51.8	6.6

나) 모성간호학

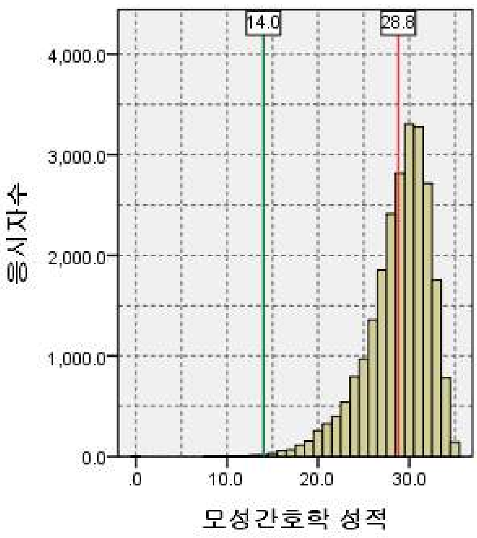

총점	과락선	평균성적	표준편차
35	14	28.8	3.5

다) 아동간호학

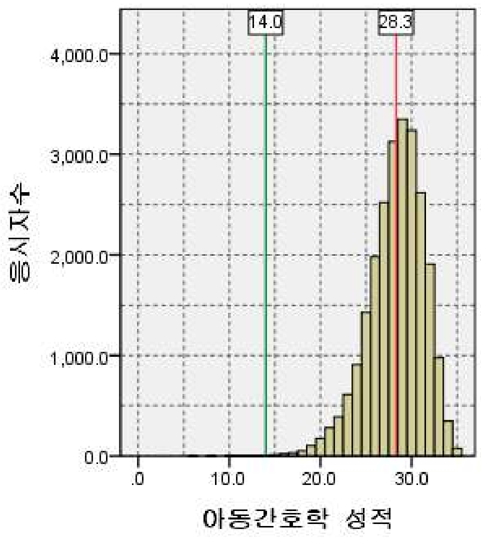

총점	과락선	평균성적	표준편차
35	14	28.3	3.1

라) 지역사회간호학

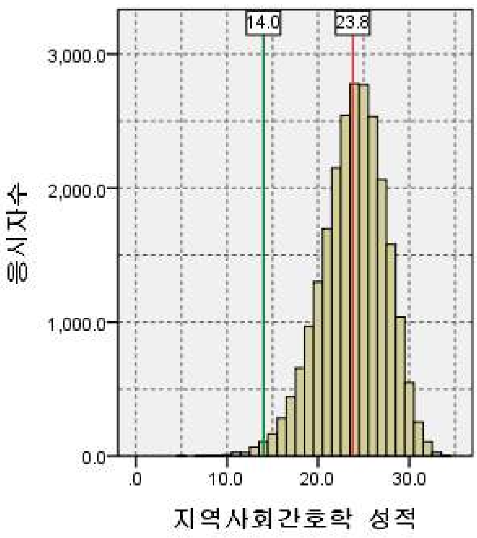

총점	과락선	평균성적	표준편차
35	14	23.8	3.6

마) 정신간호학

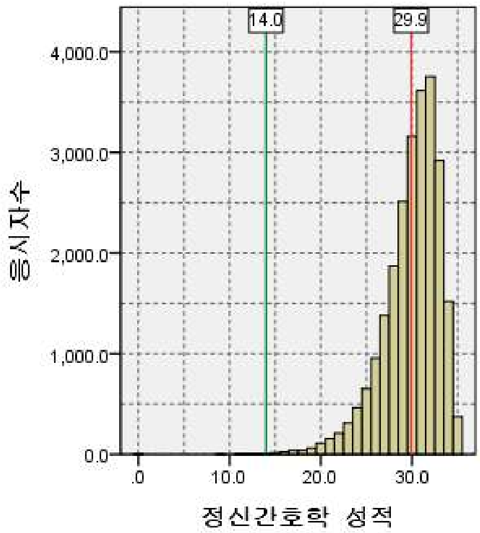

총점	과락선	평균성적	표준편차
35	14	29.9	3.1

바) 간호관리학

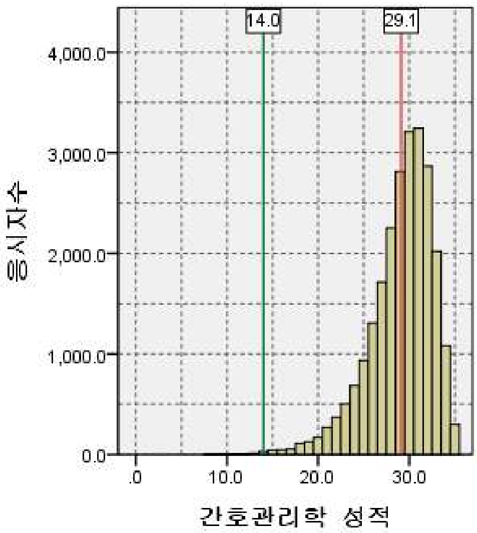

총점	과락선	평균성적	표준편차
35	14	29.1	3.5

사) 기본간호학

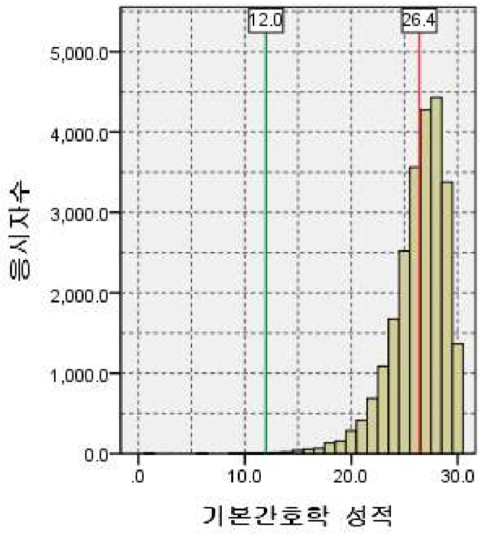

총점	과락선	평균성적	표준편차
30	12	26.36	2.60

아) 보건의약관계법규

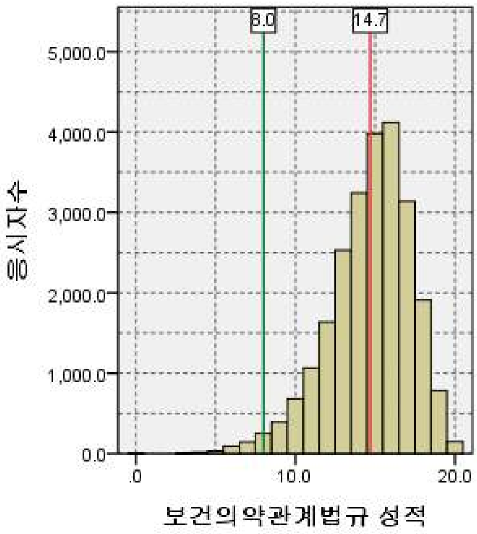

총점	과락선	평균성적	표준편차
20	8	14.7	2.6

2. 난이도와 변별도

1) 전체 난이도와 변별도

가) 전회 대비 전체 난이도와 변별도

회차	난이도		변별도1		변별도2	
	평균	표준편차	평균	표준편차	평균	표준편차
제58회	77.5	19.7	.19	.12	.21	.09
제59회	78.2	18.8	.19	.12	.23	.09
제60회	77.4	20.1	.18	.11	.21	.10
**제61회**	**77.5**	**19.2**	**.21**	**.13**	**.23**	**.09**
**제62회**	**78.9**	**19.9**	.18	.13	.23	.10

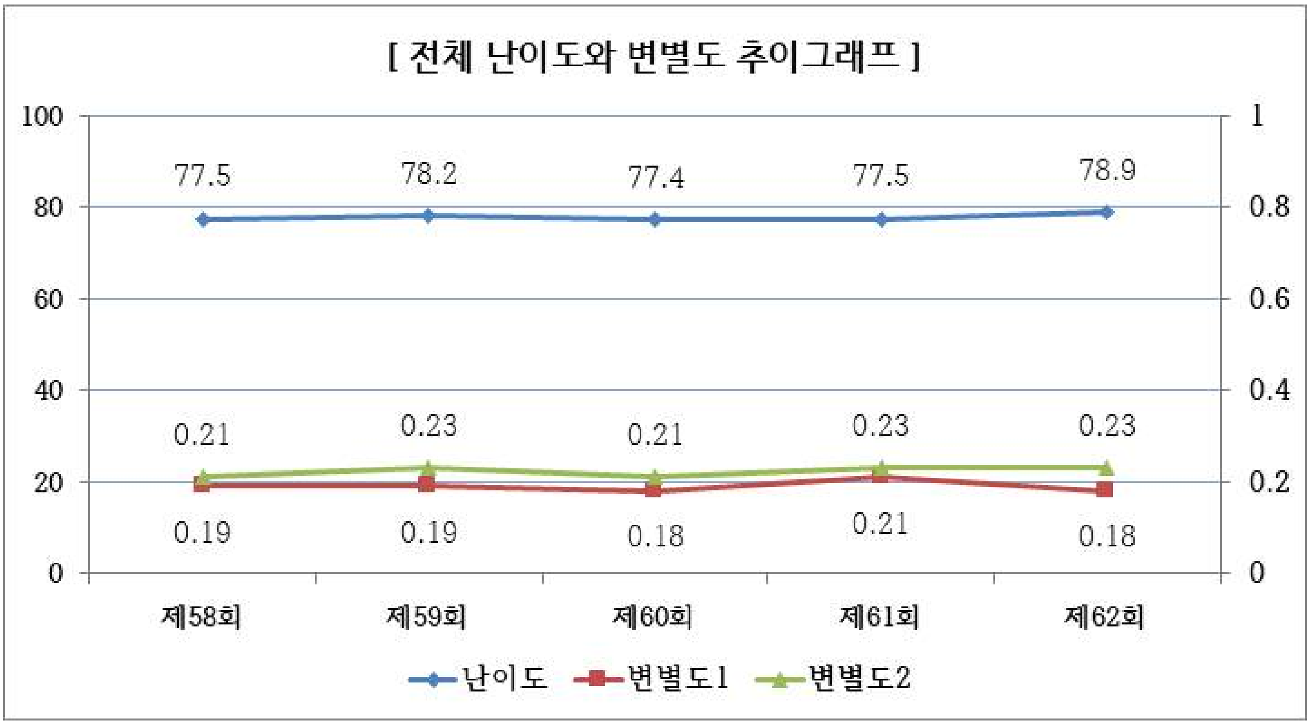

해석

- 전년 대비 난이도 지수는 1.4 증가함
- 변별도 1 지수는 .03 감소하였으며, 변별도 2 지수는 변화없음

나) 전체 난이도와 변별도 분포도 및 비율분석

(1) 전체 난이도 분포도 및 비율분석

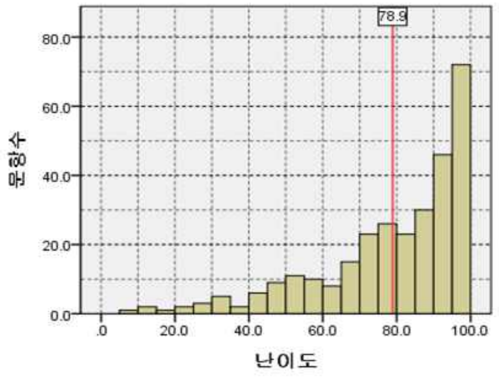

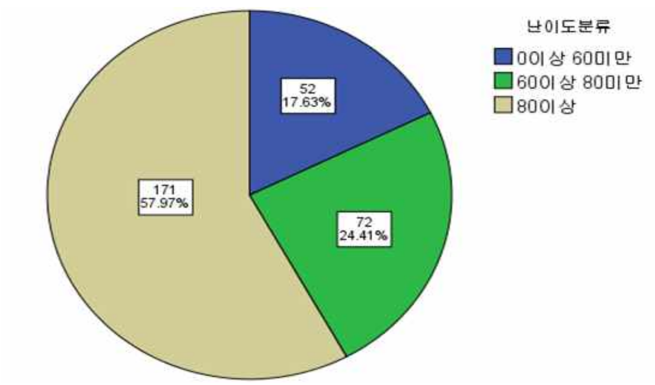

총점	난이도	표준편차
295	78.9	19.9

난이도	문항수	비율(%)
0~60미만	52	17.6
60~80미만	72	24.4
80~100	171	58.0
전체	295	100.0

(2) 전체 변별도1 분포도 및 비율분석

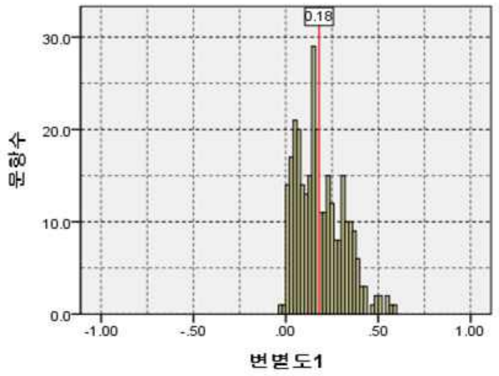

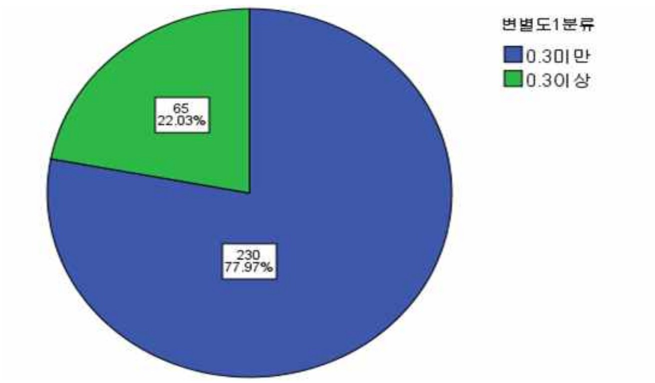

총점	변별도1	표준편차
295	.18	.13

변별도1	문항수	비율(%)
0.3미만	230	78.0
0.3이상	65	22.0
전체	295	100.0

(3) 전체 변별도2 분포도 및 비율분석

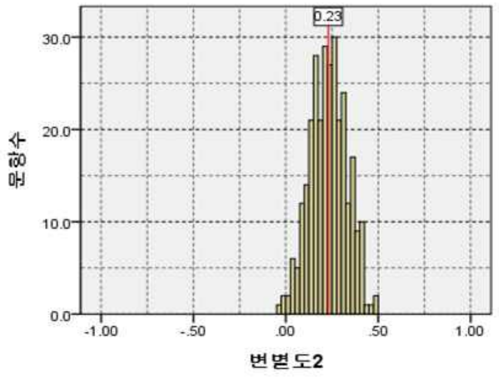

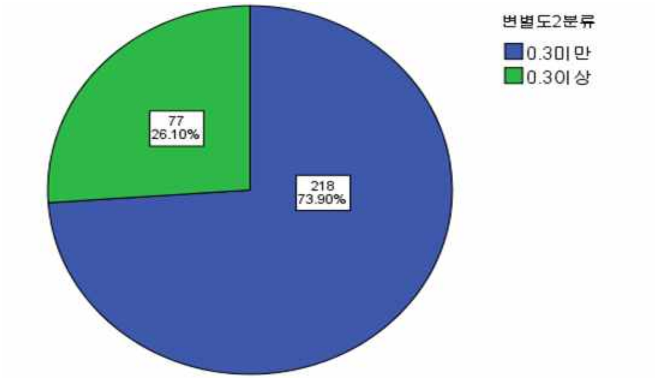

총점	변별도2	표준편차	변별도2	문항수	비율(%)
295	.23	.10	0.3미만	218	73.9
			0.3이상	77	26.1
			전체	295	100.0

해석

- 난이도 지수 80 에서 100 사이인 문항이 전체 295 문항 중 171 문항으로 가장 많았으며, 차례로 60 이상 80 미만인 문항이 72 문항, 0 에서 60 미만인 문항이 52 문항인 것으로 나타남
- 변별도 1 지수를 기준으로 분류하였을 때, 0.3 미만인 문항이 230 문항으로 0.3 이상인 문항이 65 문항인 것에 비해 더 많이 나타남
- 변별도 2 지수를 기준으로 분류하였을 때, 0.3 미만인 문항이 218 문항으로 0.3 이상인 문항이 77 문항인 것에 비해 더 많이 나타남

2) 과목별 난이도와 변별도

가) 전회 대비 과목별 난이도와 변별도

(1) 전회 대비 성인간호학 난이도와 변별도

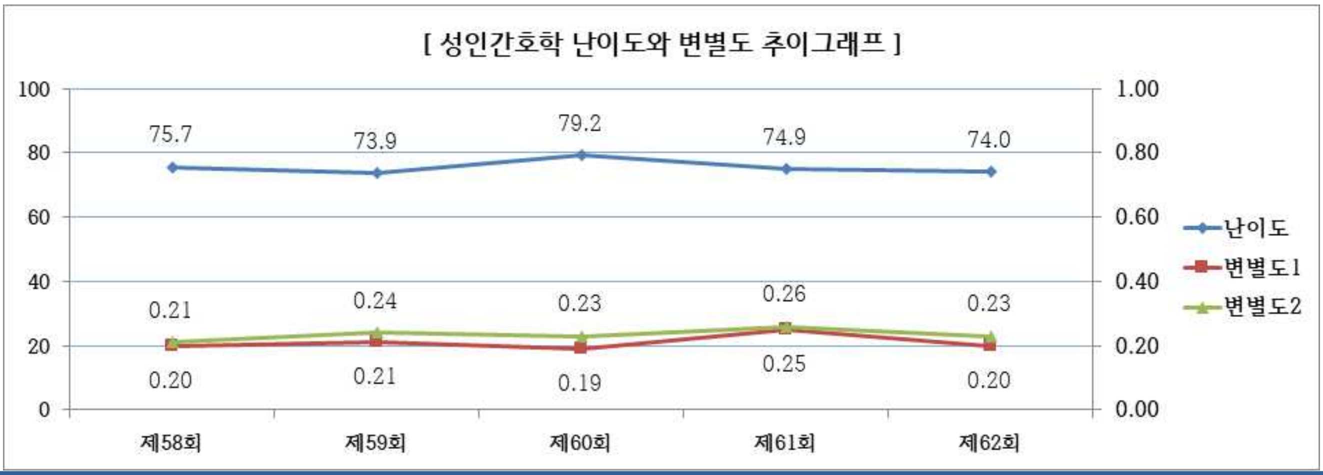

회차	난이도		변별도1		변별도2	
	평균	표준편차	평균	표준편차	평균	표준편차
제58회	75.7	19.3	.20	.12	.21	.10
제59회	73.9	20.1	.21	.13	.24	.11
제60회	79.2	18.3	.19	.12	.23	.10
제61회	74.9	20.1	.25	.13	.26	.09
**제62회**	**74.0**	**20.9**	**.20**	**.13**	**.23**	**.11**

(2) 전회 대비 모성간호학 난이도와 변별도

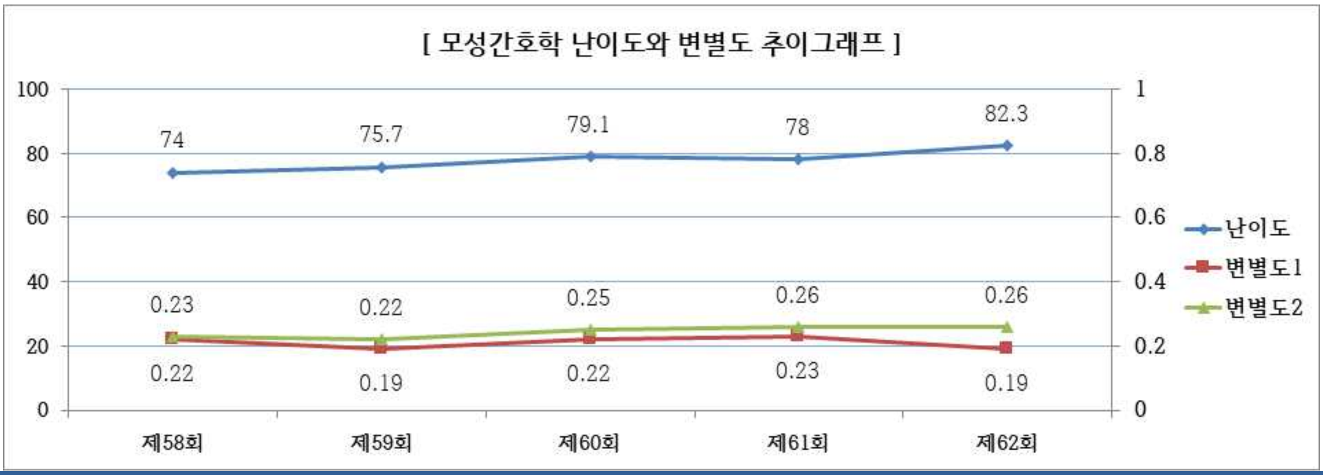

회차	난이도		변별도1		변별도2	
	평균	표준편차	평균	표준편차	평균	표준편차
제58회	74.0	19.7	.22	.14	.23	.11
제59회	75.7	19.6	.19	.11	.22	.10
제60회	79.1	14.0	.22	.11	.25	.07
제61회	78.0	15.4	.23	.11	.26	.10
**제62회**	**82.3**	**17.7**	**.19**	**.13**	**.26**	**.11**

(3) 전회 대비 아동간호학 난이도와 변별도

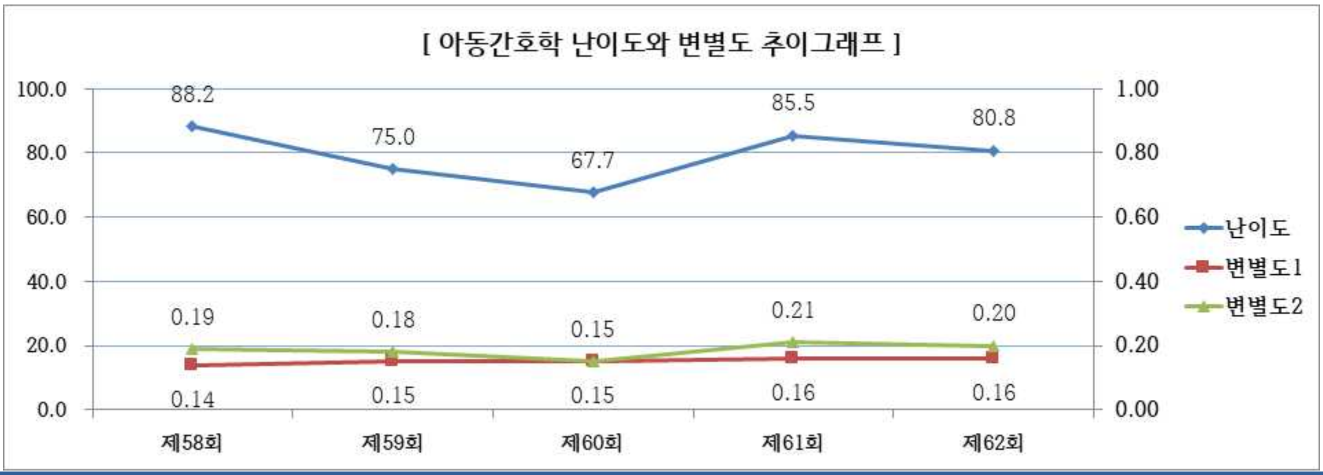

회차	난이도		변별도1		변별도2	
	평균	표준편차	평균	표준편차	평균	표준편차
제58회	88.2	14.1	.14	.13	.19	.09
제59회	75.0	21.4	.15	.11	.18	.01
제60회	67.7	22.3	.15	.10	.15	.11
제61회	85.5	15.4	.16	.13	.21	.08
**제62회**	**80.8**	**18.6**	**.16**	**.13**	**.20**	**.09**

(4) 전회 대비 지역사회간호학 난이도와 변별도

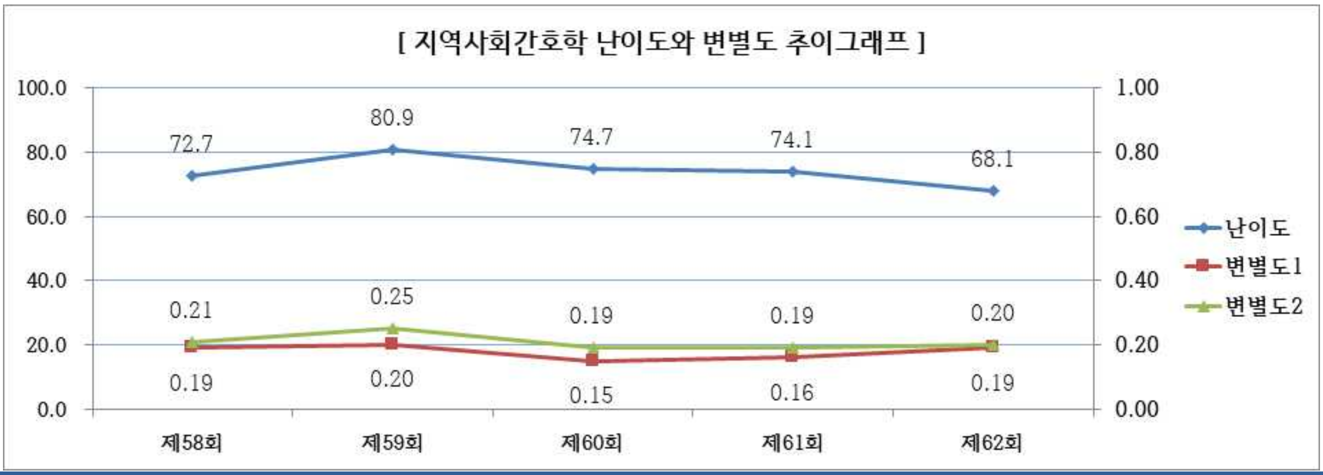

회차	난이도		변별도1		변별도2	
	평균	표준편차	평균	표준편차	평균	표준편차
제58회	72.7	23.1	.19	.10	.21	.08
제59회	80.9	16.3	.20	.13	.25	.08
제60회	74.7	24.7	.15	.10	.19	.09
제61회	74.1	22.0	.16	.10	.19	.10
**제62회**	**68.1**	**23.6**	**.19**	**.12**	**.20**	**.10**

(5) 전회 대비 정신간호학 난이도와 변별도

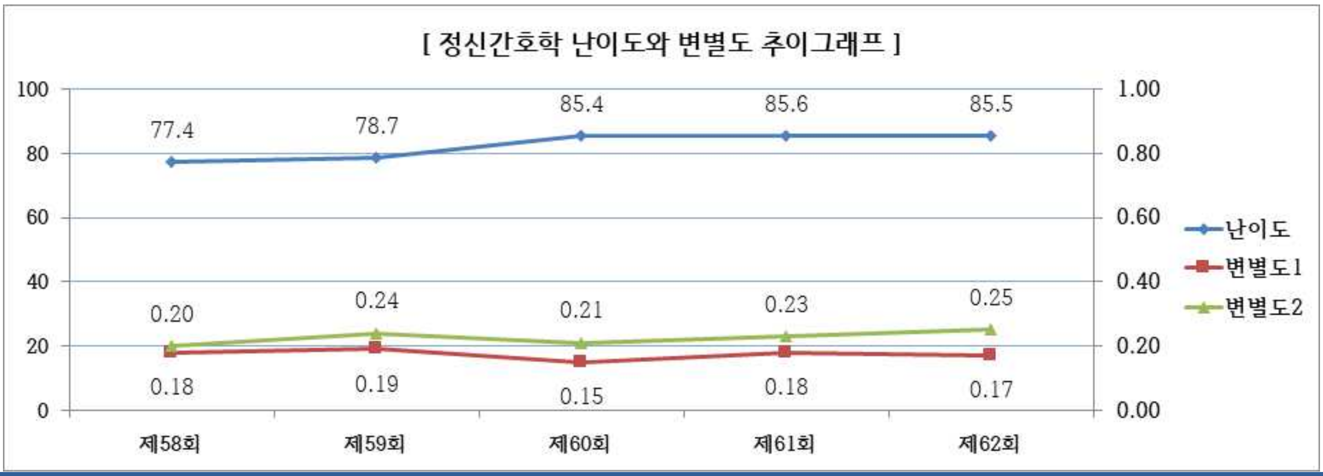

회차	난이도		변별도1		변별도2	
	평균	표준편차	평균	표준편차	평균	표준편차
제58회	77.4	21.6	.18	.12	.20	.07
제59회	78.7	18.5	.19	.12	.24	.08
제60회	85.4	15.5	.15	.09	.21	.08
제61회	85.6	14.2	.18	.12	.23	.09
**제62회**	**85.5**	**19.7**	**.17**	**.15**	**.25**	**.11**

(6) 전회 대비 간호관리학 난이도와 변별도

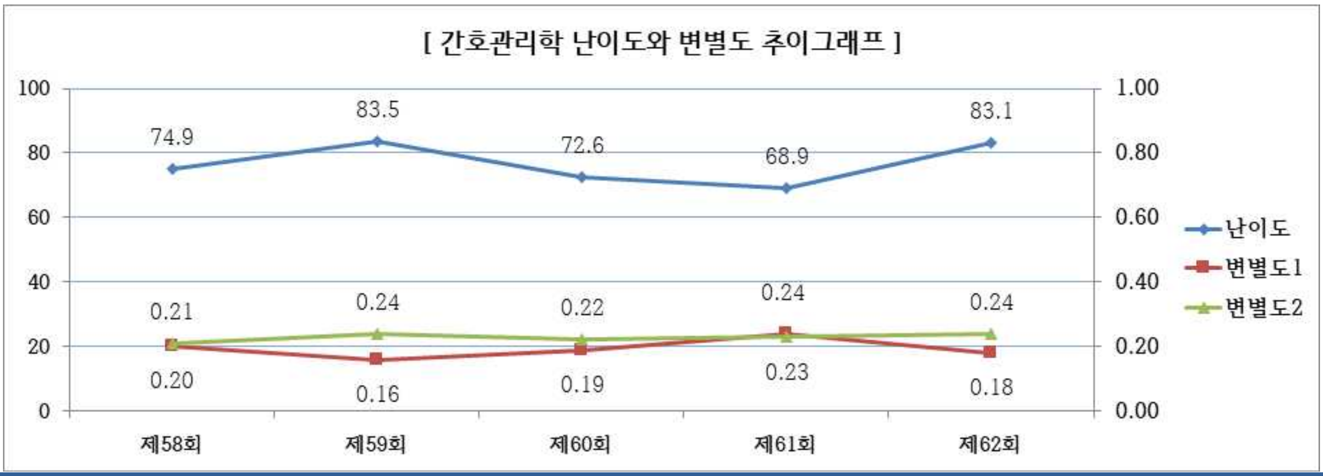

회차	난이도		변별도1		변별도2	
	평균	표준편차	평균	표준편차	평균	표준편차
제58회	74.9	19.6	.20	.13	.21	.10
제59회	83.5	18.2	.16	.10	.24	0.09
제60회	72.6	23.1	.19	.11	.22	.08
제61회	68.9	19.5	.24	.13	.23	.10
**제62회**	**83.1**	**13.9**	**.18**	**.12**	**.24**	**.10**

(7) 전회 대비 기본간호학 난이도와 변별도

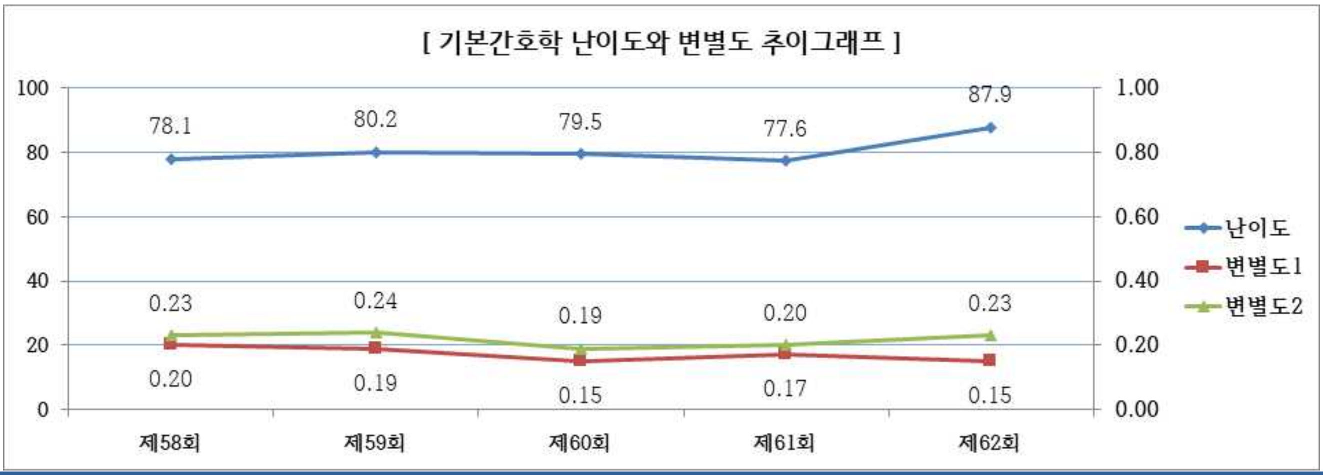

회차	난이도		변별도1		변별도2	
	평균	표준편차	평균	표준편차	평균	표준편차
제58회	78.1	17.5	.20	.10	.23	.09
제59회	80.2	16.7	.19	.10	.24	.08
제60회	79.5	19.6	.15	.11	.19	.10
제61회	77.6	21.1	.17	.12	.20	.08
**제62회**	**87.9**	**12.6**	**.15**	**.11**	**.23**	**.06**

(8) 전회 대비 보건의약관계법규 난이도와 변별도

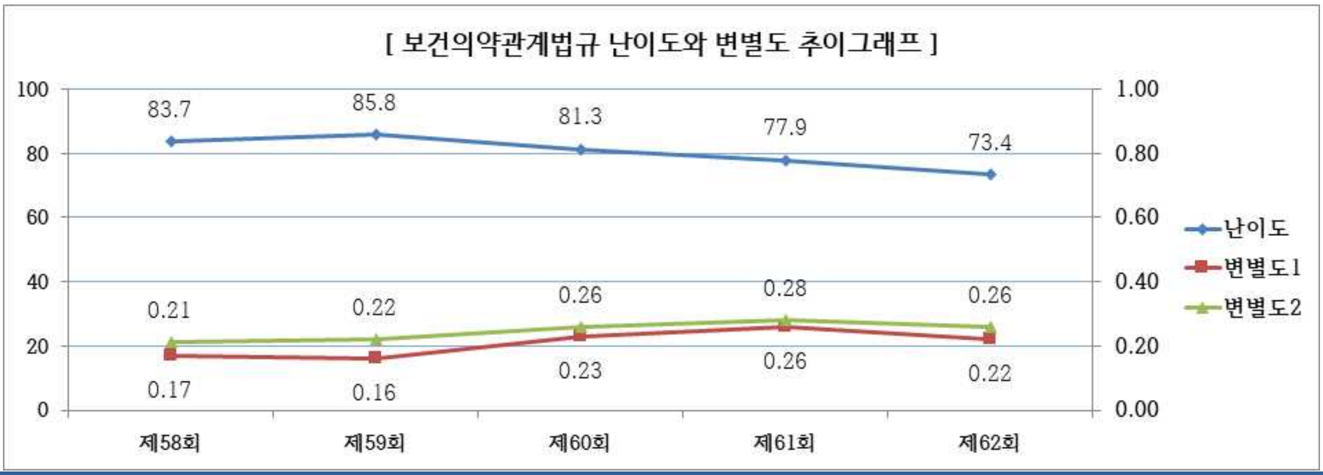

회차	난이도		변별도1		변별도2	
	평균	표준편차	평균	표준편차	평균	표준편차
제58회	83.7	14.1	.17	.09	.21	.07
제59회	85.8	11.7	.16	.13	.22	.10
제60회	81.3	13.3	.23	.14	.26	.10
제61회	77.9	16.8	.26	.11	.28	.07
**제62회**	**73.4**	**22.4**	**.22**	**.09**	**.26**	**.07**

해석

- 전회 대비 성인간호학의 난이도 지수는 0.9 감소, 변별도 1 지수와 변별도 2 지수는 각각 0.05, 0.03 감소함
- 모성간호학 과목의 난이도 지수는 4.3 증가, 변별도 1 지수는 0.04 감소하였으며, 변별도 2 지수는 변화 없음
- 아동간호학 과목의 난이도 지수는 4.7 감소, 변별도 1 지수는 변화 없었으며, 변별도 2 지수는 0.01 감소함
- 지역사회간호학 과목의 난이도 지수는 6.0 감소, 변별도 1 지수와 변별도 2 지수는 각각 0.03, 0.01 증가함
- 정신간호학 과목의 난이도 지수는 0.1 감소, 변별도 1 지수는 0.01 감소하였으며, 변별도 2 지수는 0.02 증가함
- 간호관리학 과목의 난이도 지수는 14.2 증가, 변별도 1 지수는 0.06 감소하였으며, 변별도 2 지수는 0.01 증가함
- 기본간호학 과목의 난이도 지수는 10.3 증가, 변별도 1 지수는 0.02 감소, 변별도 2 지수는 0.03 증가함
- 보건의약관계법규 과목의 난이도 지수는 4.5 감소, 변별도 1 지수와 변별도 2 지수는 각각 0.04, 0.02 감소함

나) 과목별 난이도와 변별도 분포도 및 비율분석

(1) 성인간호학 난이도와 변별도 분포도 및 비율분석

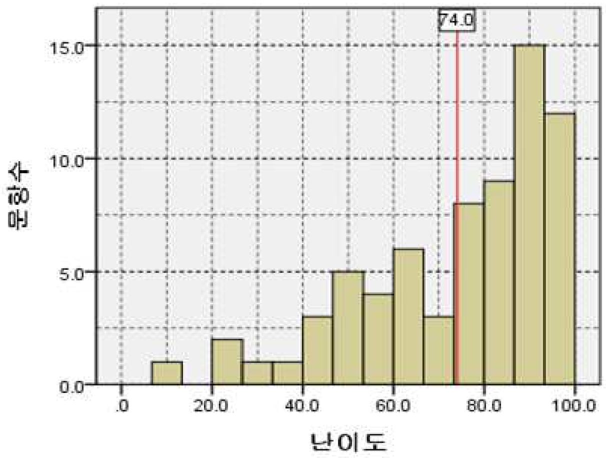

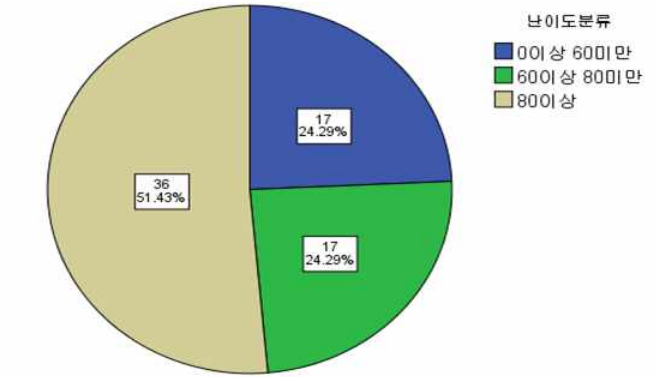

총점	난이도	표준편차	난이도	문항수	비율(%)
70	74.0	20.9	0~60미만	17	24.3
			60~80미만	17	24.3
			80~100	36	51.4
			전체	70	100.0

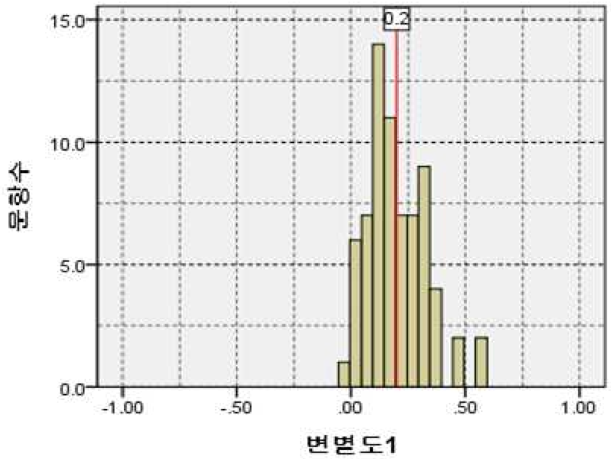

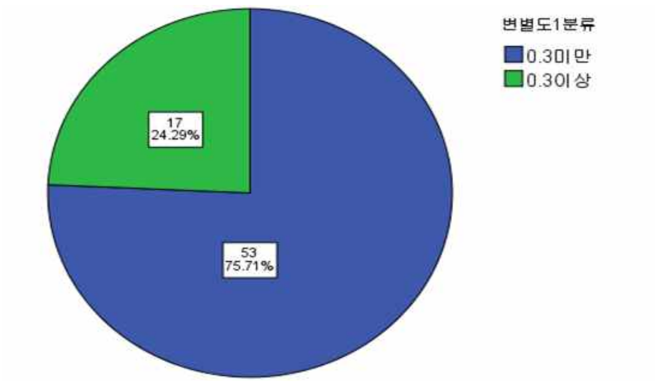

총점	변별도1	표준편차	변별도1	문항수	비율(%)
70	.20	.13	0.3미만	53	75.7
			0.3이상	17	24.3
			전체	70	100.0

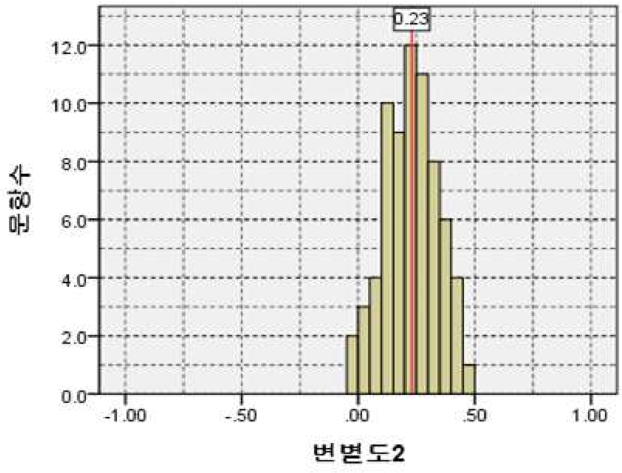

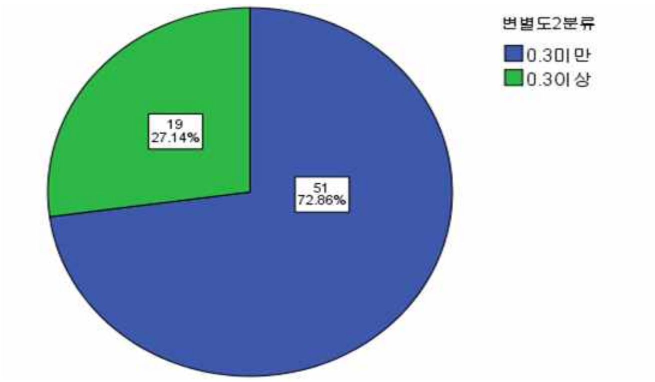

총점	변별도2	표준편차
70	.23	.11

변별도2	문항수	비율(%)
0.3미만	51	72.9
0.3이상	19	27.1
전체	70	100.0

해석

- 성인간호학 과목에서 난이도 지수가 80 에서 100 사이인 문항이 전체 70 문항 중 36 문항으로 가장 많았으며, 차례로 60 이상 80 미만인 문항이 17 문항, 0 에서 60 미만인 문항이 17 문항으로 나타남
- 변별도 1 지수를 기준으로 분류하였을 때, 0.3 미만인 문항이 53 문항으로 0.3 이상인 문항이 17 문항인 것에 비해 더 많이 나타남
- 변별도 2 지수를 기준으로 분류하였을 때, 0.3 미만인 문항이 51 문항으로 0.3 이상인 문항이 19 문항인 것에 비해 더 많이 나타남

(2) 모성간호학 난이도와 변별도 분포도 및 비율분석

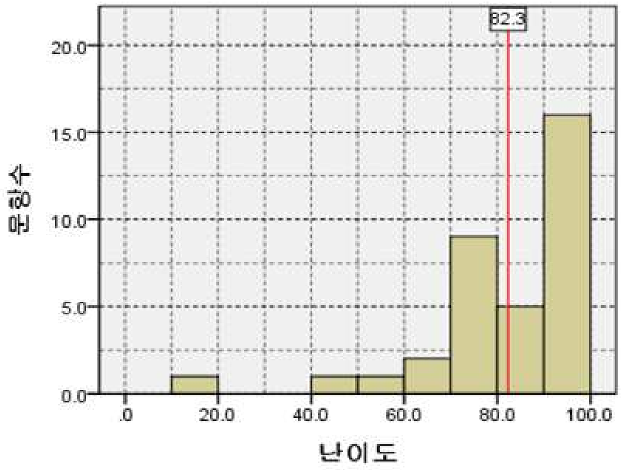

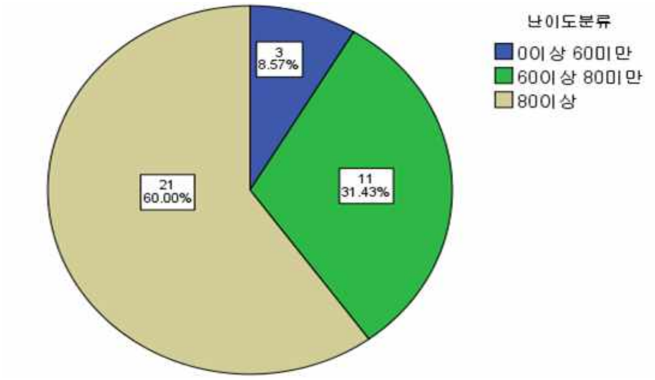

총점	난이도	표준편차	난이도	문항수	비율(%)
35	82.3	17.7	0~60미만	3	8.6
			60~80미만	11	31.4
			80~100	21	60.0
			전체	35	100.0

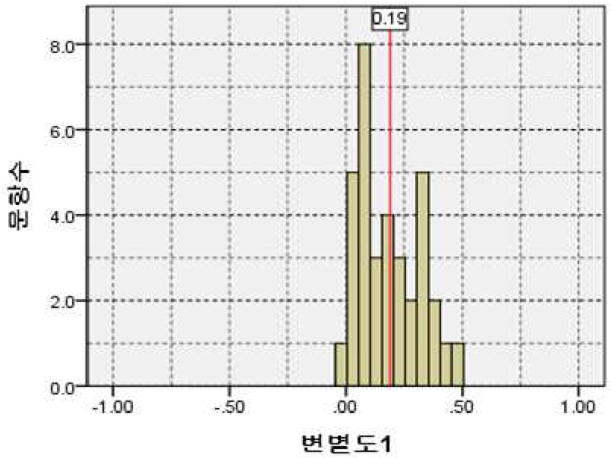

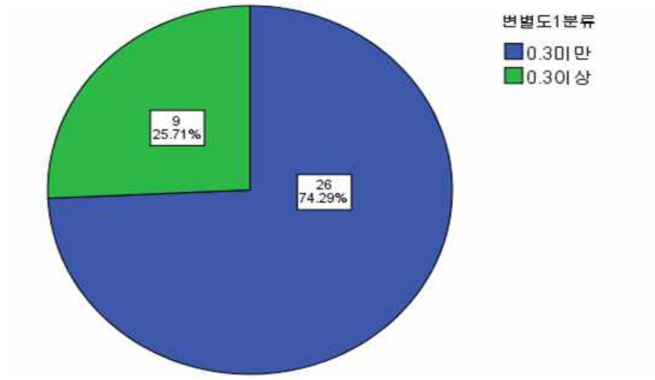

총점	변별도1	표준편차	변별도1	문항수	비율(%)
35	.19	.13	0.3미만	26	74.3
			0.3이상	9	25.7
			전체	35	100.0

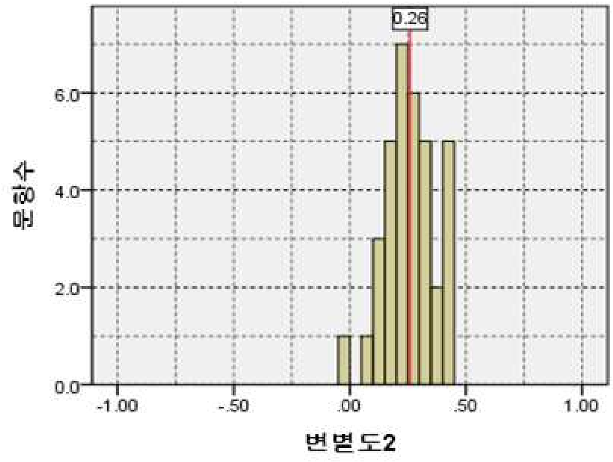

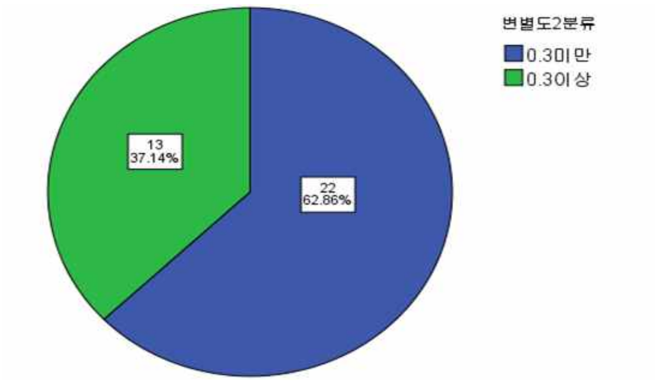

총점	변별도2	표준편차
35	.26	.11

변별도2	문항수	비율(%)
0.3미만	22	62.9
0.3이상	13	37.1
전체	35	100.0

해석

- 모성간호학 과목에서 난이도 지수가 80 이상 100 사이인 문항이 전체 35 문항 중 21 문항으로 가장 많았으며, 차례로 60 이상 80 미만인 문항이 11 문항, 0 에서 60 미만인 문항이 3 문항으로 나타남
- 변별도 1 지수를 기준으로 분류하였을 때, 0.3 미만인 문항이 26 문항으로 0.3 이상인 문항이 9 문항인 것에 비해 더 많이 나타남
- 변별도 2 지수를 기준으로 분류하였을 때, 0.3 미만인 문항이 22 문항으로 0.3 이상인 문항이 13 문항인 것에 비해 더 많이 나타남

(3) 아동간호학 난이도와 변별도 분포도 및 비율분석

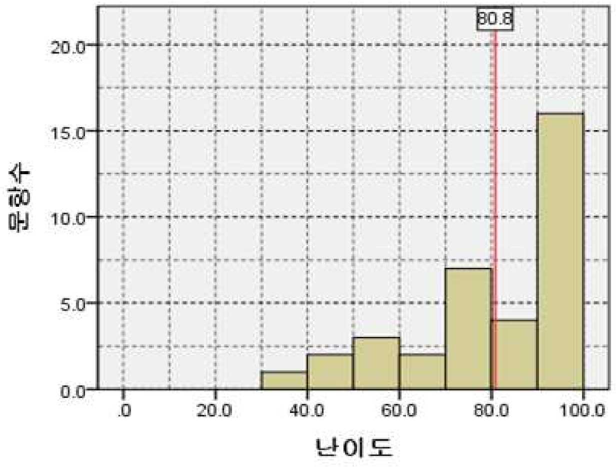

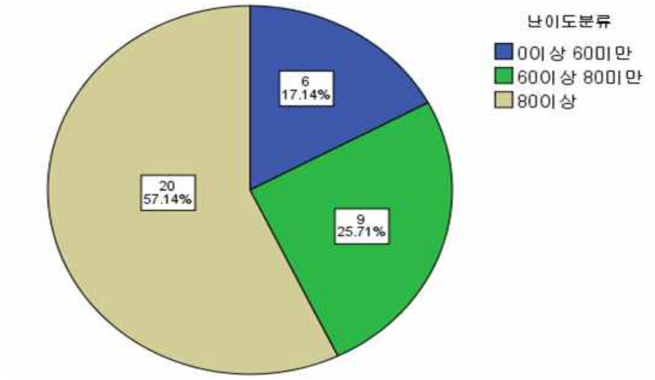

총점	난이도	표준편차
35	80.8	18.6

난이도	문항수	비율(%)
0~60미만	6	17.1
60~80미만	9	25.7
80~100	20	57.1
전체	35	100.0

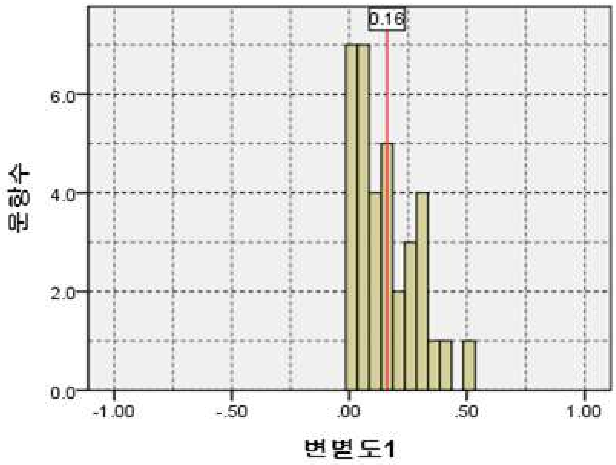

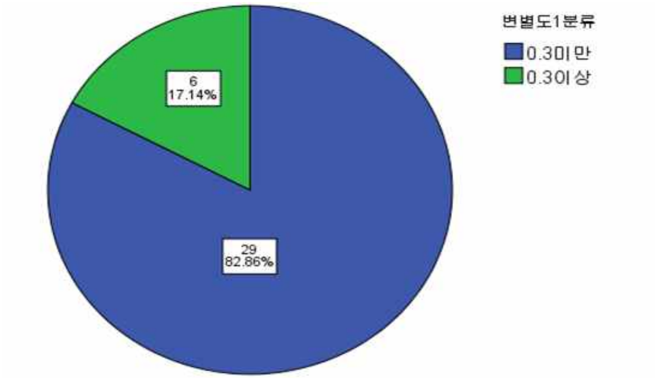

총점	변별도1	표준편차
35	.16	.13

변별도1	문항수	비율(%)
0.3미만	29	82.9
0.3이상	6	17.1
전체	35	100.0

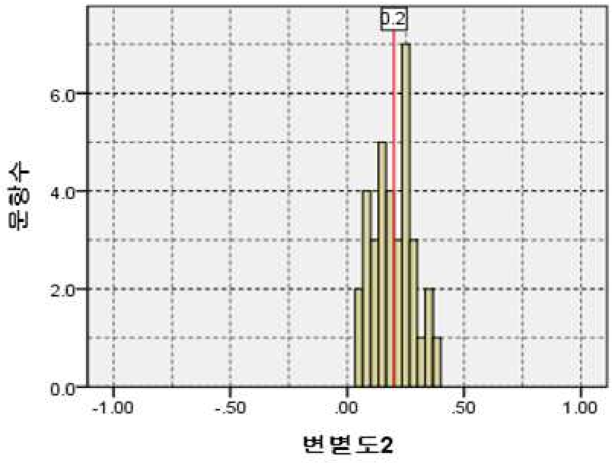

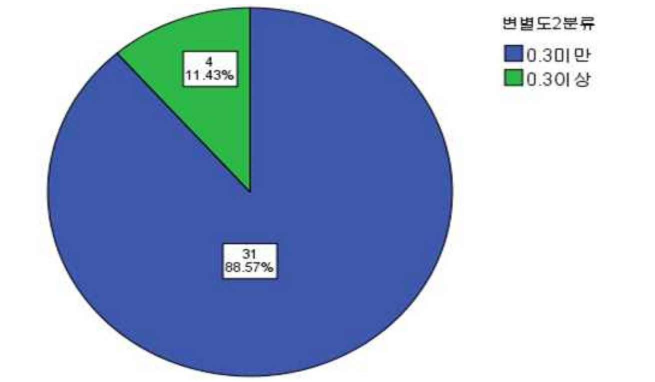

총점	변별도2	표준편차
35	.20	.09

변별도2	문항수	비율(%)
0.3미만	31	88.6
0.3이상	4	11.4
전체	35	100.0

해석

- 아동간호학 과목에서 난이도 지수가 80 이상 100 사이인 문항이 전체 35 문항 중 20 문항으로 가장 많았으며, 차례로 60 이상 80 미만인 문항이 9 문항, 0 에서 60 미만인 문항이 6 문항으로 나타남
- 변별도 1 지수를 기준으로 분류하였을 때, 0.3 미만인 문항이 29 문항으로 0.3 이상인 문항이 6 문항인 것에 비해 더 많이 나타남
- 변별도 2 지수를 기준으로 분류하였을 때, 0.3 미만인 문항이 31 문항으로 0.3 이상인 문항이 4 문항인 것에 비해 더 많이 나타남

(4) 지역사회간호학 난이도와 변별도 분포도 및 비율분석

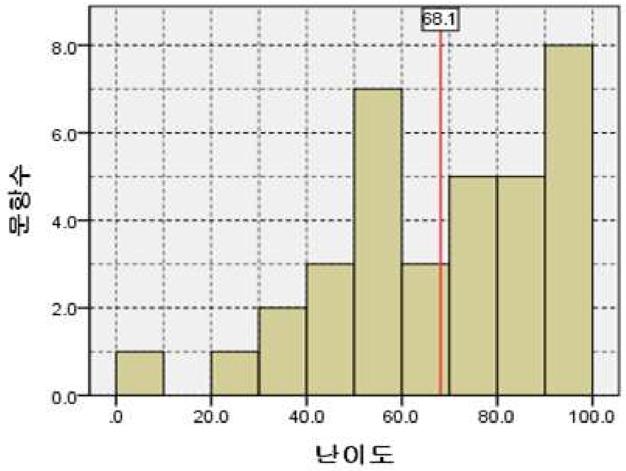

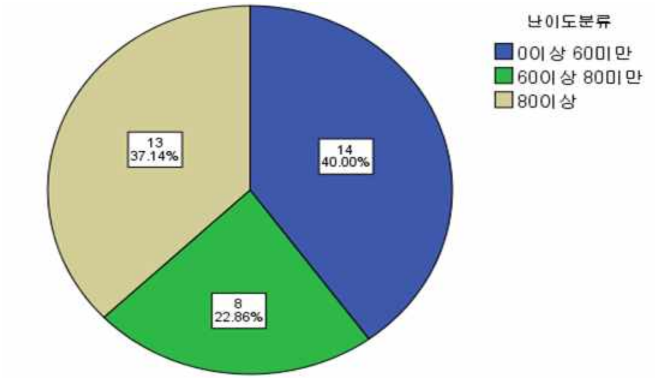

총점	난이도	표준편차
35	68.1	23.6

난이도	문항수	비율(%)
0~60미만	14	40.0
60~80미만	8	22.9
80~100	13	37.1
전체	35	100.0

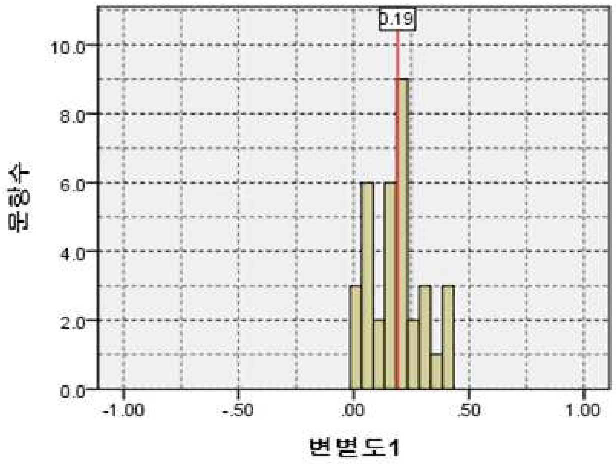

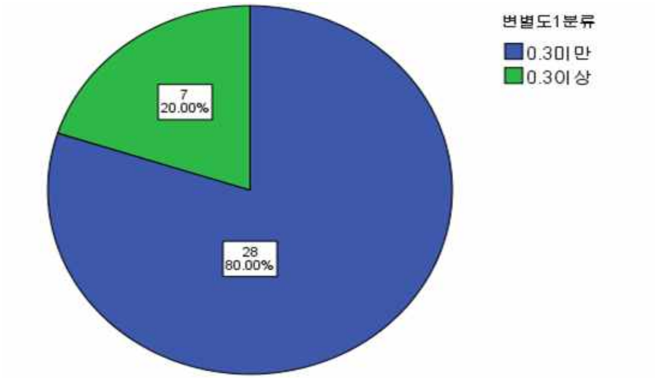

총점	변별도1	표준편차
35	.19	.12

변별도1	문항수	비율(%)
0.3미만	28	80.0
0.3이상	7	20.0
전체	35	100.0

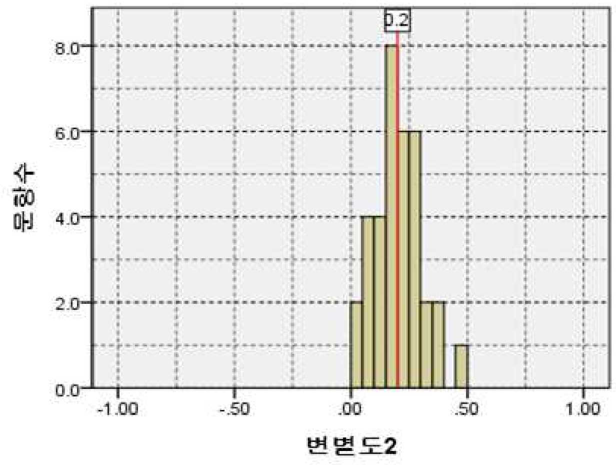

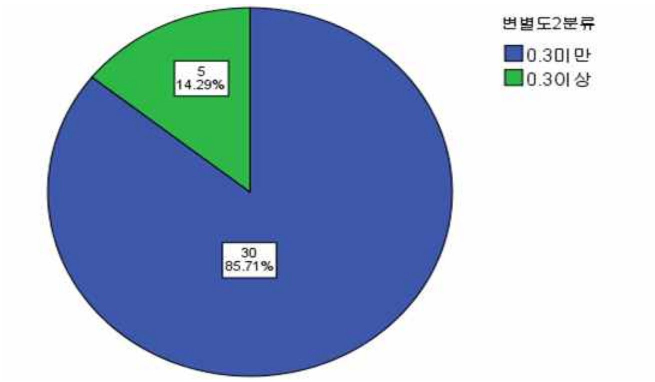

총점	변별도2	표준편차
35	.20	.10

변별도2	문항수	비율(%)
0.3미만	30	85.7
0.3이상	5	14.3
전체	35	100.0

해석

- 지역사회간호학 과목에서 난이도 지수가 80 이상 100 사이인 문항이 전체 35 문항 중 13 문항으로 나타났으며, 60 이상 80 미만인 문항이 8 문항, 0 에서 60 미만인 문항이 14 문항으로 나타남
- 변별도 1 지수를 기준으로 분류하였을 때, 0.3 미만인 문항이 28 문항으로 0.3 이상인 문항이 7 문항인 것에 비해 더 많이 나타남
- 변별도 2 지수를 기준으로 분류하였을 때, 0.3 미만인 문항이 30 문항으로 0.3 이상인 문항이 5 문항인 것에 비해 더 많이 나타남

(5) 정신간호학 난이도와 변별도 분포도 및 비율분석

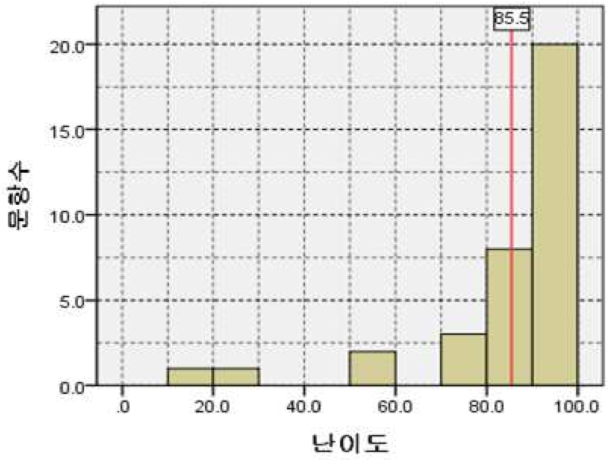

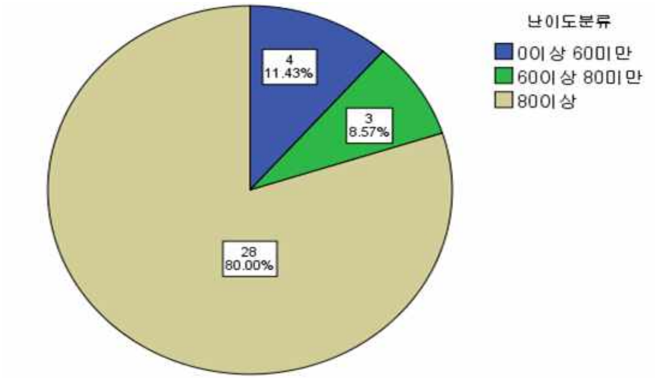

총점	난이도	표준편차
35	85.5	19.7

난이도	문항수	비율(%)
0~60미만	4	11.4
60~80미만	3	8.6
80~100	28	80.0
전체	35	100.0

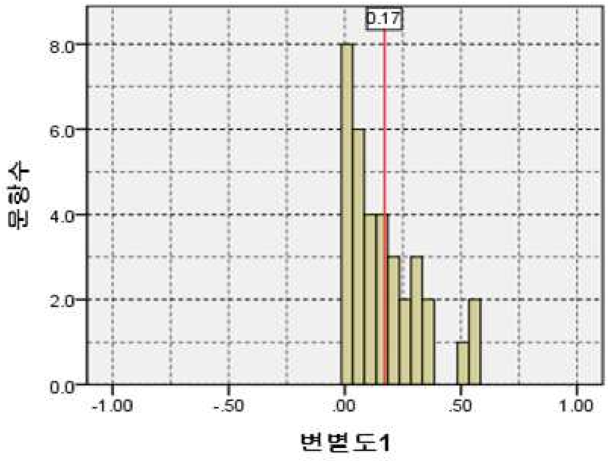

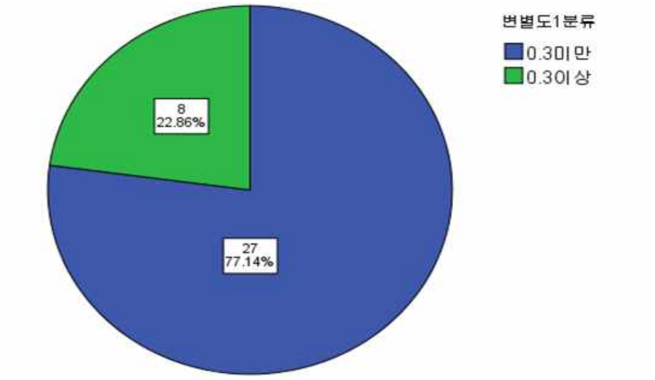

총점	변별도1	표준편차
35	.17	.15

변별도1	문항수	비율(%)
0.3미만	27	77.1
0.3이상	8	22.9
전체	35	100.0

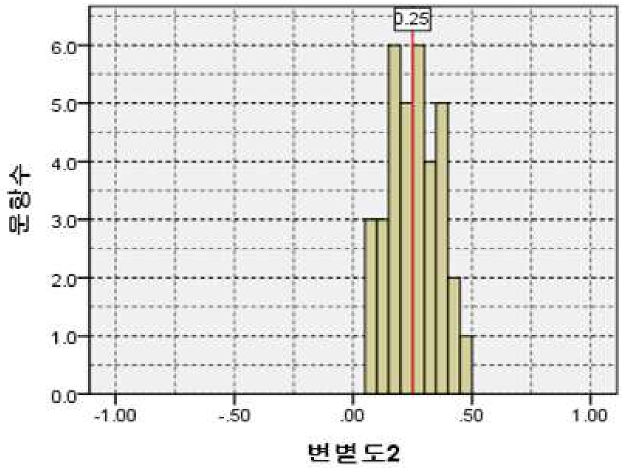

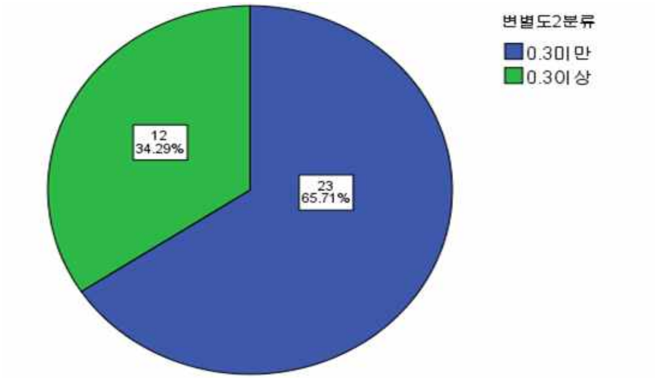

총점	변별도2	표준편차
35	.25	.11

변별도2	문항수	비율(%)
0.3미만	23	65.7
0.3이상	12	34.3
전체	35	100.0

해석

- 정신간호학 과목에서 난이도 지수가 80 이상 100 사이인 문항이 전체 35 문항 중 28 문항으로 가장 많았으며, 차례로 60 이상 80 미만인 문항이 3 문항, 0 에서 60 미만인 문항이 4 문항으로 나타남
- 변별도 1 지수를 기준으로 분류하였을 때, 0.3 미만인 문항이 27 문항으로 0.3 이상인 문항이 8 문항인 것에 비해 더 많이 나타남
- 변별도 2 지수를 기준으로 분류하였을 때, 0.3 미만인 문항이 23 문항으로 0.3 이상인 문항이 12 문항인 것에 비해 더 많이 나타남

(6) 간호관리학 난이도와 변별도 분포도 및 비율분석

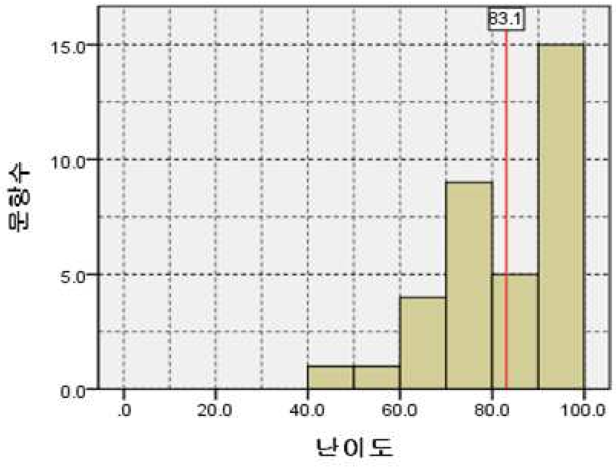

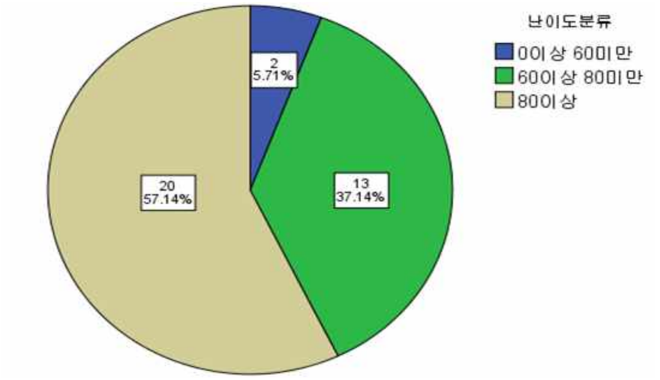

총점	난이도	표준편차
35	83.1	13.9

난이도	문항수	비율(%)
0~60미만	2	5.7
60~80미만	13	37.1
80~100	20	57.1
전체	35	100.0

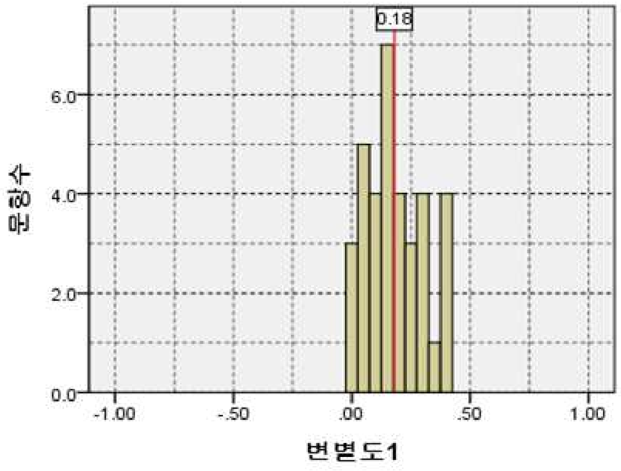

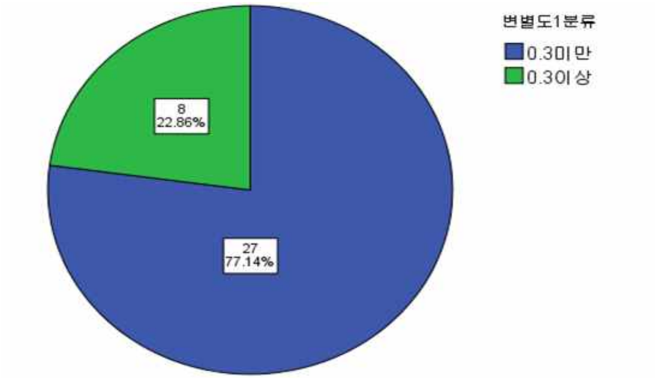

총점	변별도1	표준편차
35	.18	.12

변별도1	문항수	비율(%)
0.3미만	27	77.1
0.3이상	8	22.9
전체	35	100.0

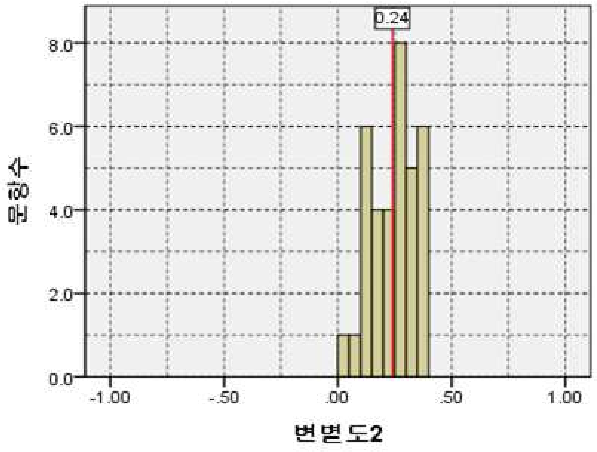

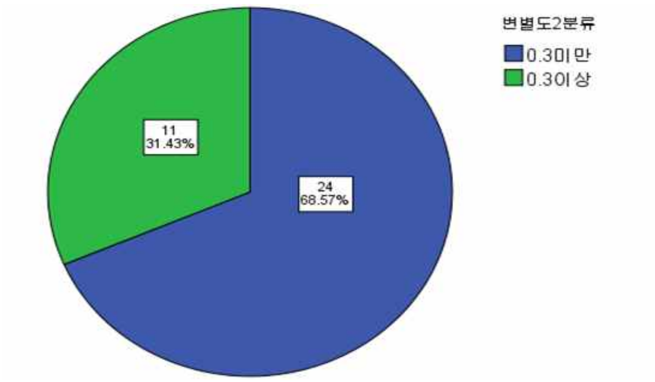

총점	변별도2	표준편차
35	.24	.10

변별도2	문항수	비율(%)
0.3미만	24	68.6
0.3이상	11	31.4
전체	35	100.0

해석

- 간호관리학 과목에서 난이도 지수가 60 이상 80 미만인 문항이 전체 35 문항 중 20 문항으로 가장 많았으며, 차례로 80 이상 100 사이인 문항이 12 문항, 0 에서 60 미만인 문항이 1 문항으로 나타남
- 변별도 1 지수를 기준으로 분류하였을 때, 0.3 미만인 문항이 27 문항으로 0.3 이상인 문항이 8 문항인 것에 비해 더 많이 나타남
- 변별도 2 지수를 기준으로 분류하였을 때, 0.3 미만인 문항이 24 문항으로 0.3 이상인 문항이 11 문항인 것에 비해 더 많이 나타남

(7) 기본간호학 난이도와 변별도 분포도 및 비율분석

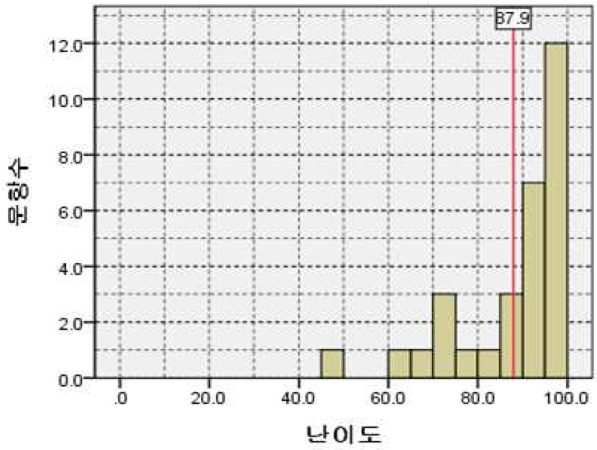

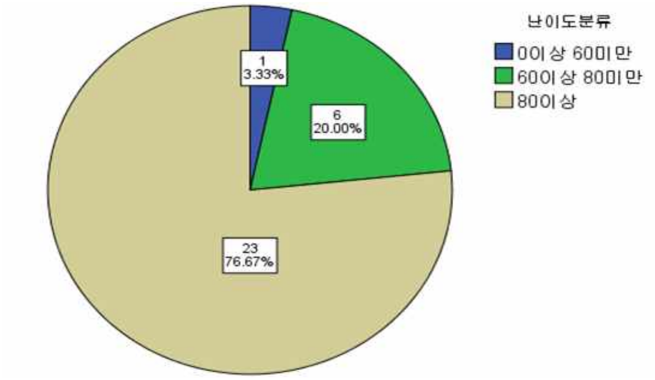

총점	난이도	표준편차
30	87.9	12.6

난이도	문항수	비율(%)
0~60미만	1	3.3
60~80미만	6	20.0
80~100	23	76.7
전체	30	100.0

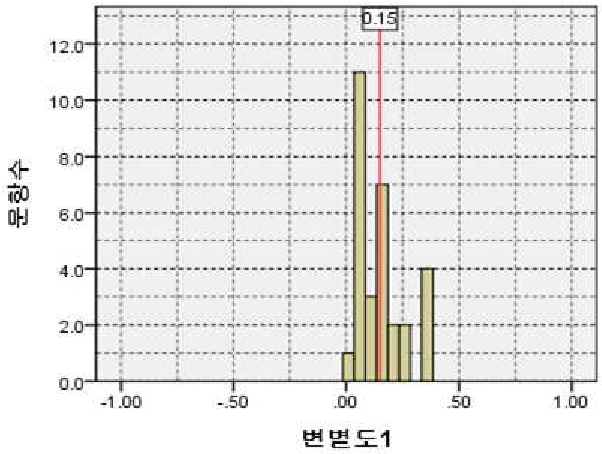

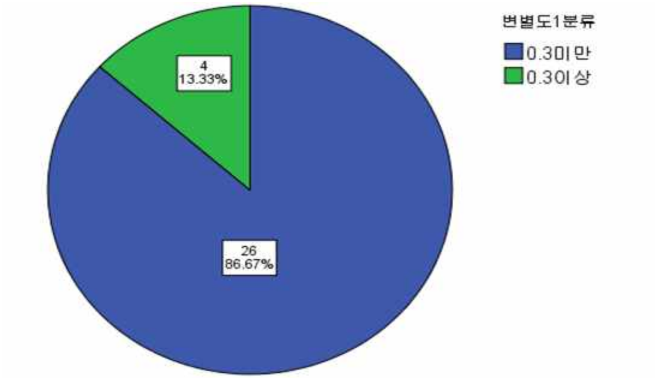

총점	변별도1	표준편차
30	.15	.11

변별도1	문항수	비율(%)
0.3미만	26	86.7
0.3이상	4	13.3
전체	30	100.0

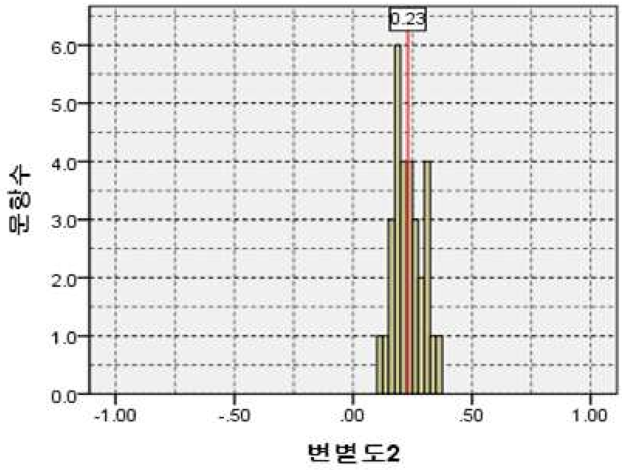

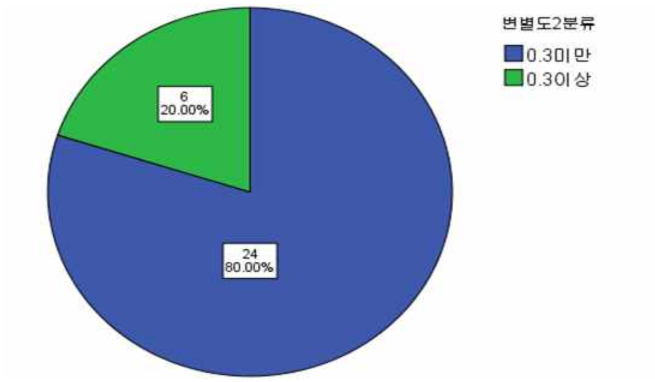

<table>
<tr><th>총점</th><th>변별도2</th><th>표준편차</th><th>변별도2</th><th>문항수</th><th>비율(%)</th></tr>
<tr><td rowspan="3">30</td><td rowspan="3">.23</td><td rowspan="3">.06</td><td>0.3미만</td><td>24</td><td>80.0</td></tr>
<tr><td>0.3이상</td><td>6</td><td>20.0</td></tr>
<tr><td>전체</td><td>30</td><td>100.0</td></tr>
</table>

해석

- 기본간호학 과목에서 난이도 지수가 80 에서 100 사이인 문항이 전체 30 문항 중 23 문항으로 가장 많았으며, 차례로 60 이상 80 미만인 문항이 6 문항, 0 에서 60 미만인 문항이 1 문항으로 나타남
- 변별도 1 지수를 기준으로 분류하였을 때, 0.3 미만인 문항이 26 문항으로 0.3 이상인 문항이 4 문항인 것에 비해 더 많이 나타남
- 변별도 2 지수를 기준으로 분류하였을 때, 0.3 미만인 문항이 24 문항으로 0.3 이상인 문항이 6 문항인 것에 비해 더 많이 나타남

(8) 보건의약관계법규 난이도와 변별도 분포도 및 비율분석

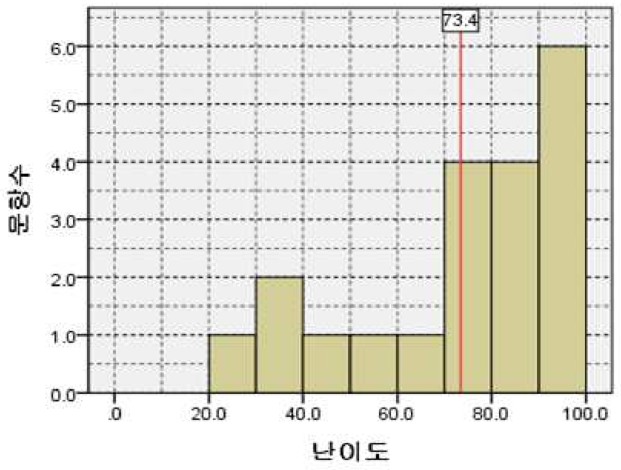

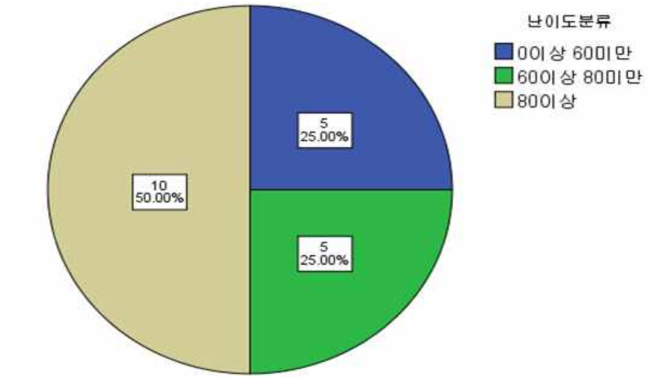

총점	난이도	표준편차
20	73.4	22.4

난이도	문항수	비율(%)
0~60미만	5	25.0
60~80미만	5	25.0
80~100	10	50.0
전체	20	100.0

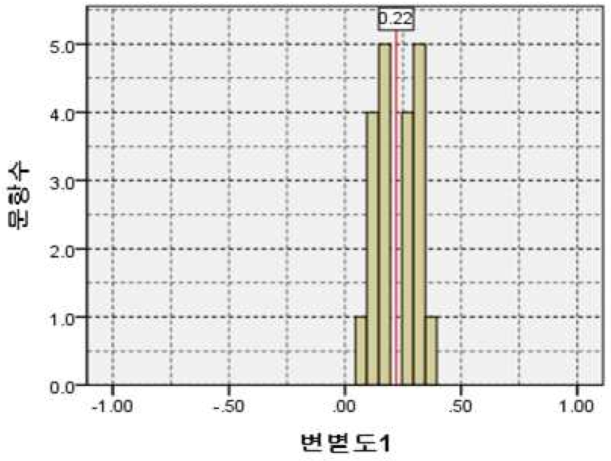

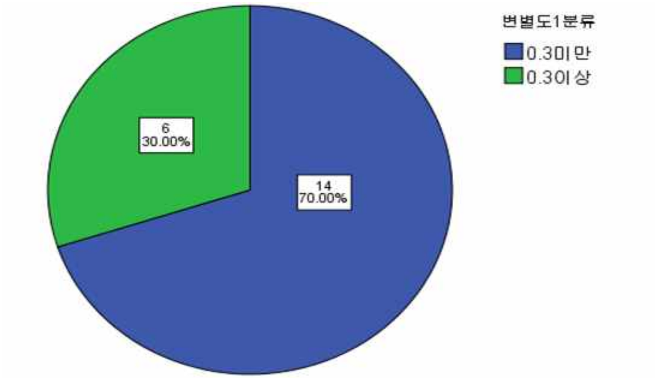

총점	변별도1	표준편차
20	.22	.09

변별도1	문항수	비율(%)
0.3미만	14	70.0
0.3이상	6	30.0
전체	20	100.0

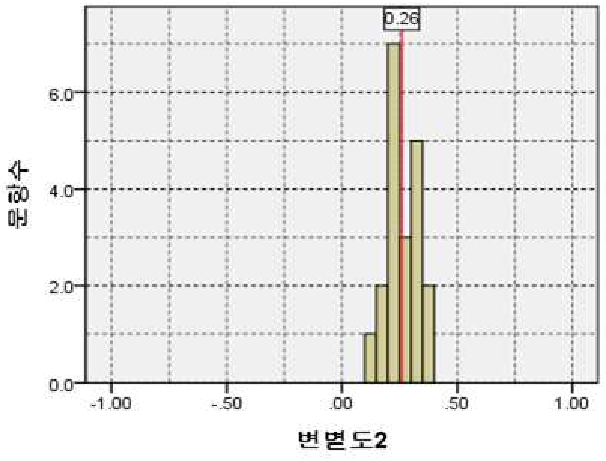

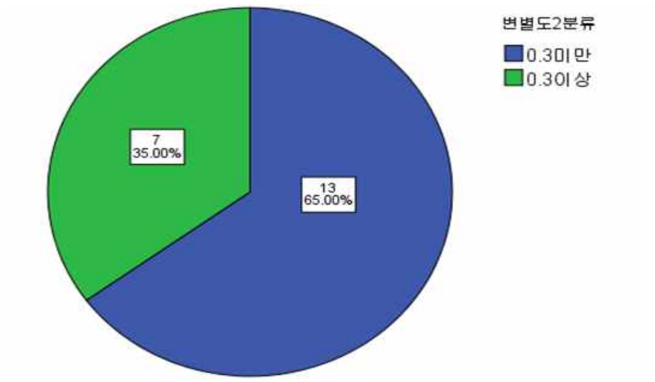

총점	변별도2	표준편차
20	.26	.07

변별도2	문항수	비율(%)
0.3미만	13	65.0
0.3이상	7	35.0
전체	20	100.0

해석

- 보건의약관계법규 과목에서 난이도 지수가 80 이상 100 사이인 문항이 전체 20 문항 중 10 문항으로 가장 많았으며, 60 이상 80 미만인 문항이 5 문항, 0 에서 60 미만인 문항이 5 문항으로 나타남
- 변별도 1 지수를 기준으로 분류하였을 때, 0.3 미만인 문항이 14 문항으로 0.3 이상인 문항이 6 문항인 것에 비해 더 많이 나타남
- 변별도 2 지수를 기준으로 분류하였을 때, 0.3 미만인 문항이 13 문항으로 0.3 이상인 문항이 7 문항인 것에 비해 더 많이 나타남

3) 지식수준별 난이도와 변별도

가) 전회 대비 지식수준별 난이도와 변별도

(1) 전회 대비 암기형 난이도와 변별도

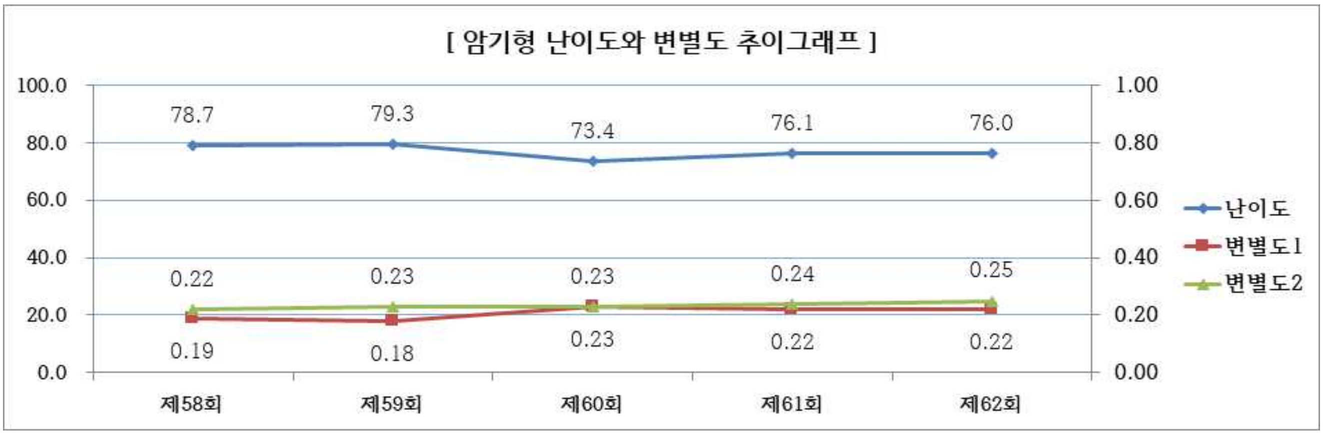

회차	난이도		변별도1		변별도2	
	평균	표준편차	평균	표준편차	평균	표준편차
제58회	78.7	18.4	.19	.11	.22	.07
제59회	79.3	17.1	.18	.12	.23	.10
제60회	73.4	19.9	.23	.12	.23	.10
제61회	76.1	20.1	.22	.12	.24	.09
**제62회**	**76.0**	**19.5**	**.22**	**.12**	**.25**	**.10**

(2) 전회 대비 해석형 난이도와 변별도

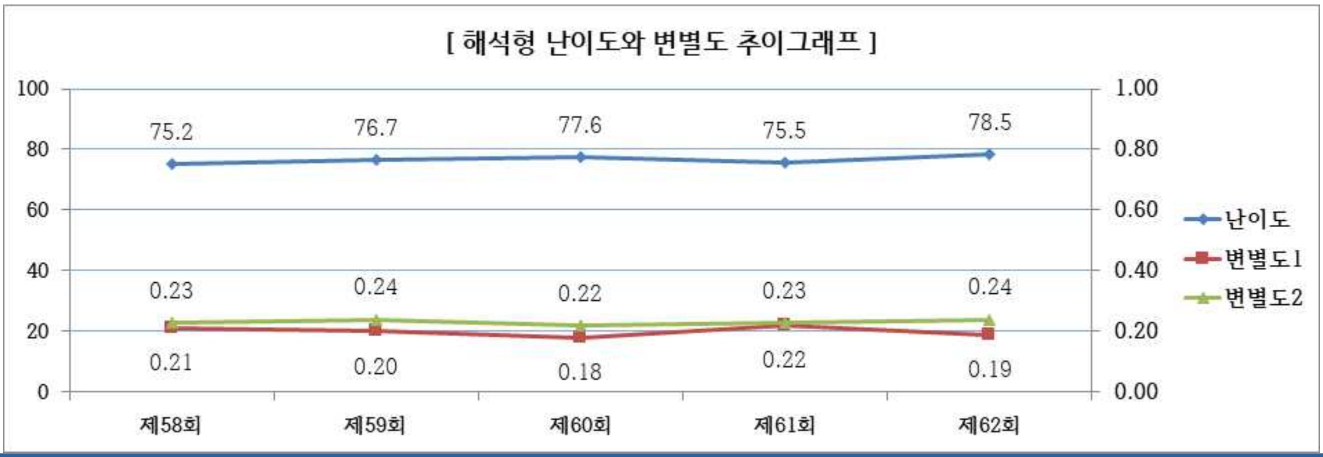

회차	난이도		변별도1		변별도2	
	평균	표준편차	평균	표준편차	평균	표준편차
제58회	75.2	19.8	.21	.12	.23	.09
제59회	76.7	19.5	.20	.13	.24	.09
제60회	77.6	20.2	.18	.12	.22	.10
제61회	75.5	19.5	.22	.13	.23	.10
**제62회**	**78.5**	**20.0**	**.19**	**.13**	**.24**	**.10**

(3) 전회 대비 해결형 난이도와 변별도

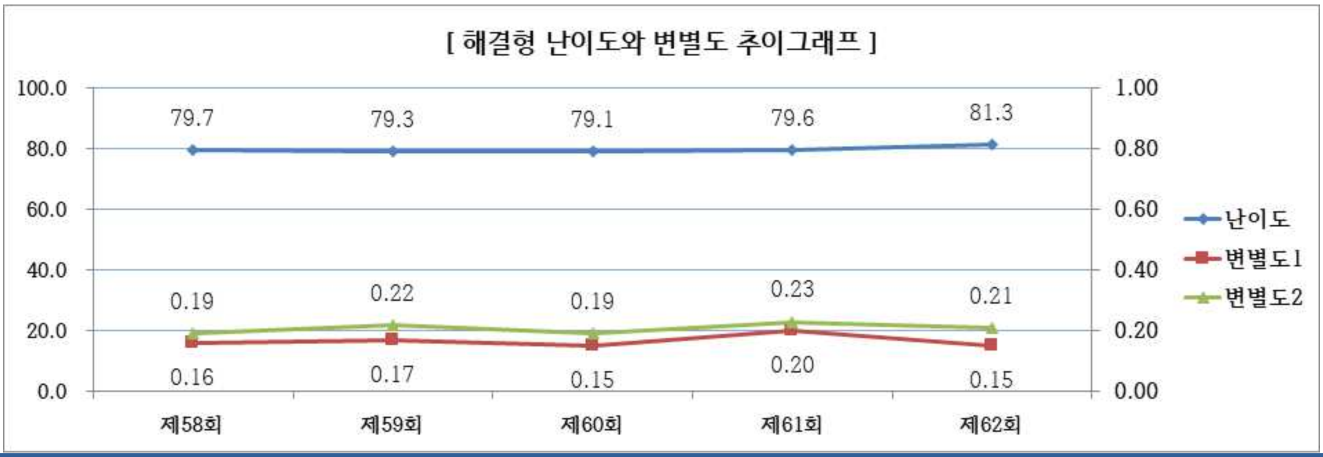

회차	난이도		변별도1		변별도2	
	평균	표준편차	평균	표준편차	평균	표준편차
제58회	79.7	20.0	.16	.12	.19	.10
제59회	79.3	18.8	.17	.11	.22	.09
제60회	79.1	19.7	.15	.10	.19	.09
제61회	79.6	18.4	.20	.13	.23	.10
**제62회**	**81.3**	**20.0**	**.15**	**.12**	**.21**	**.10**

해석

- 전회 대비 암기형 문항의 난이도 지수는 0.1 감소, 해석형 문항의 난이도 지수는 3.0 증가, 해결형 문항의 난이도 지수는 1.7 증가함
- 암기형 문항의 변별도 1 지수는 변화 없으며, 변별도 2 지수는 .01 증가함
- 해석형 문항의 변별도 1 지수는 0.03 감소, 변별도 2 지수는 0.01 증가함
- 해결형 문항의 변별도 1 지수와 변별도 2 지수는 각각 0.05, 0.02 감소함

나) 지식수준별 난이도와 변별도 분포도 및 비율분석

(1) 암기형 난이도와 변별도 분석도 및 비율분석

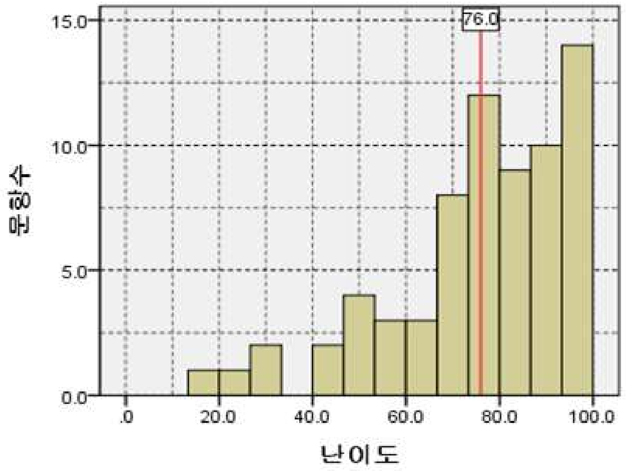

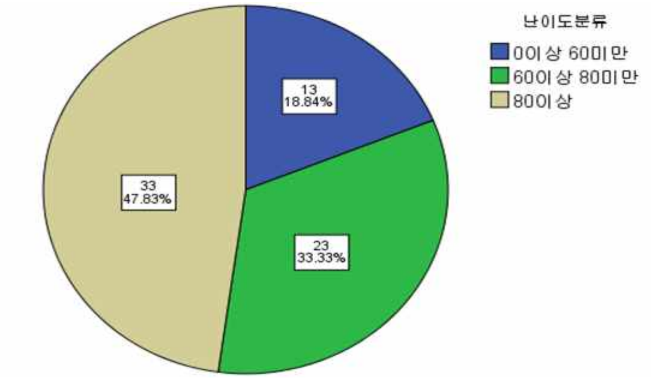

총점	난이도	표준편차	난이도	문항수	비율(%)
69	76.0	19.5	0~60미만	13	18.8
			60~80미만	23	33.3
			80~100	33	47.8
			전체	69	100.0

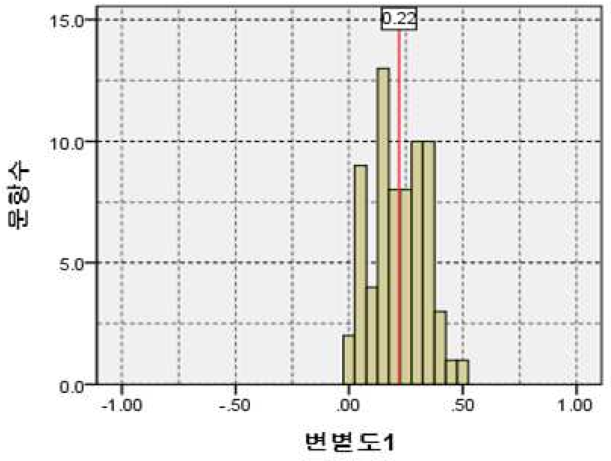

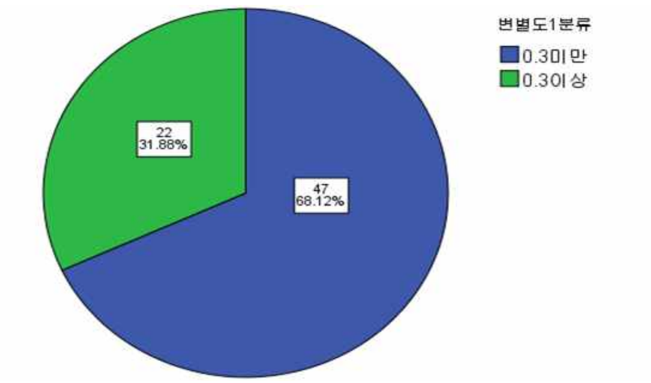

총점	변별도1	표준편차	변별도1	문항수	비율(%)
69	.22	.12	0.3미만	47	68.1
			0.3이상	22	31.9
			전체	69	100.0

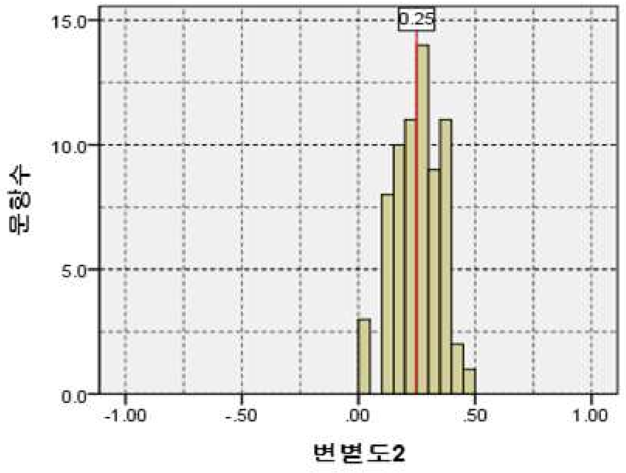

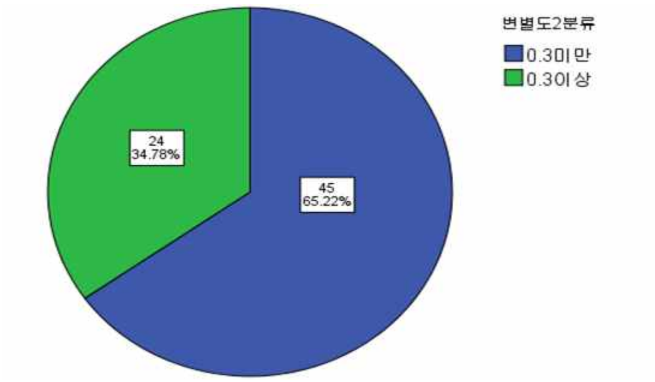

총점	변별도2	표준편차
69	.25	.10

변별도2	문항수	비율(%)
0.3미만	45	65.2
0.3이상	24	34.8
전체	69	100.0

해석

- 암기형 문항에서 난이도 지수가 80 이상 100 사이인 문항이 전체 69 문항 중 33 문항으로 가장 많았으며, 차례로 60 이상 80 미만인 문항이 23 문항, 0 에서 60 미만인 문항이 13 문항으로 나타남
- 변별도 1 지수를 기준으로 분류하였을 때, 0.3 미만인 문항이 47 문항으로 0.3 이상인 문항이 22 문항인 것에 비해 더 많이 나타남
- 변별도 2 지수를 기준으로 분류하였을 때, 0.3 미만인 문항이 45 문항으로 0.3 이상인 문항이 24 문항인 것에 비해 더 많이 나타남

(2) 해석형 난이도와 변별도 분포도 및 비율분석

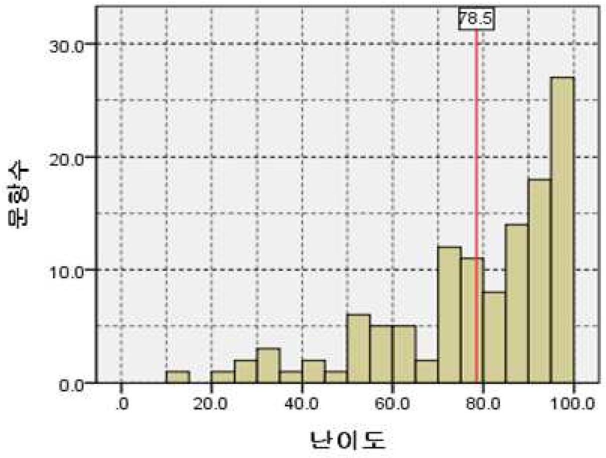

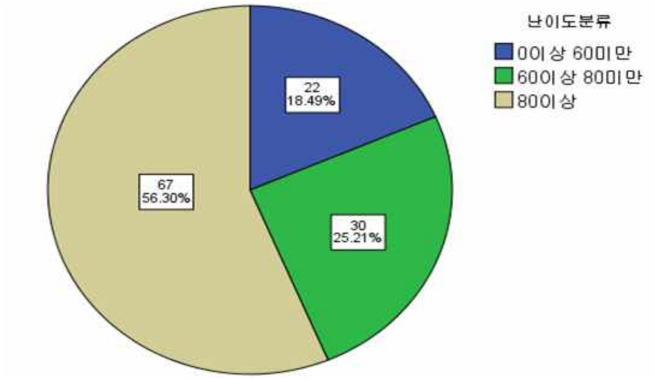

총점	난이도	표준편차
119	78.5	20.0

난이도	문항수	비율(%)
0~60미만	22	18.5
60~80미만	30	25.2
80~100	67	56.3
전체	119	100.0

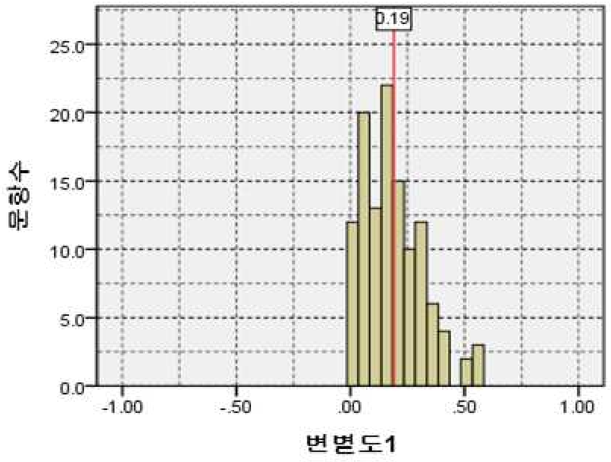

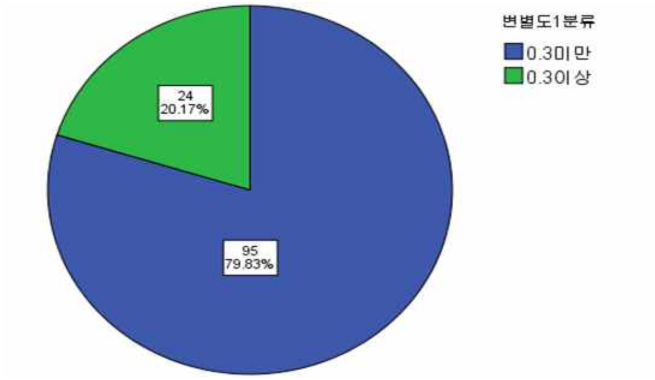

총점	변별도1	표준편차
119	.19	.13

변별도1	문항수	비율(%)
0.3미만	95	79.8
0.3이상	24	20.2
전체	119	100.0

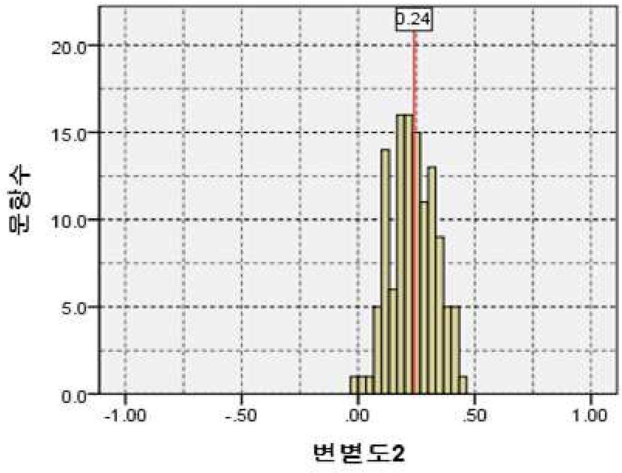

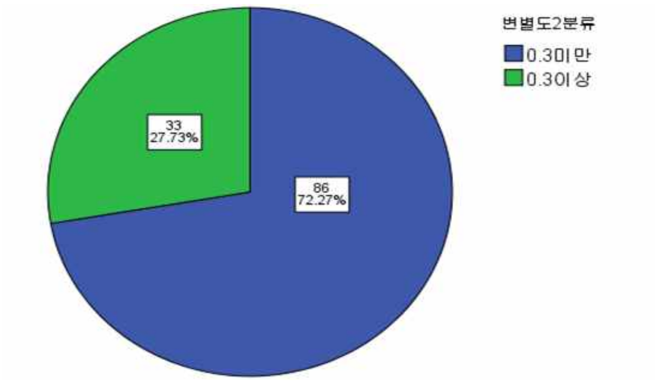

총점	변별도2	표준편차
119	.24	.10

변별도2	문항수	비율(%)
0.3미만	86	72.3
0.3이상	33	27.7
전체	119	100.0

해석

- 해석형 문항에서 난이도 지수가 80 이상 100 사이인 문항이 전체 119 문항 중 67 문항으로 가장 많았으며, 차례로 60 이상 80 미만인 문항이 30 문항, 60 미만인 문항이 22 문항으로 나타남
- 변별도 1 지수를 기준으로 분류하였을 때, 0.3 미만인 문항이 95 문항으로 0.3 이상인 문항이 24 문항인 것에 비해 더 많이 나타남
- 변별도 2 지수를 기준으로 분류하였을 때, 0.3 미만인 문항이 86 문항으로 0.3 이상인 문항이 33 문항인 것에 비해 더 많이 나타남

(3) 해결형 난이도와 변별도 분포도 및 비율분석

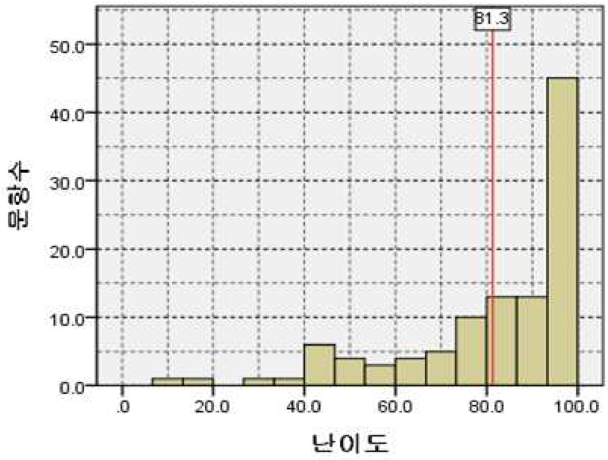

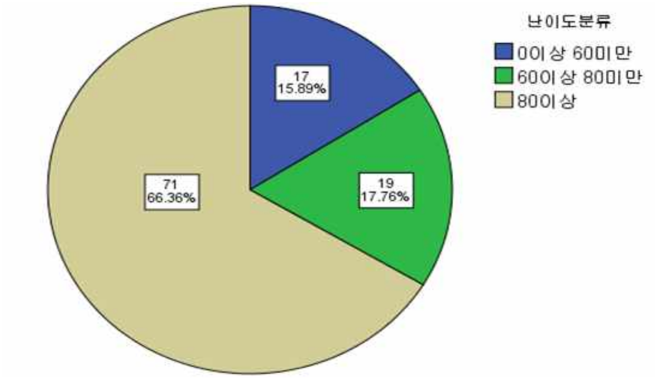

총점	난이도	표준편차
107	81.3	20.0

난이도	문항수	비율(%)
0~60미만	17	15.9
60~80미만	19	17.8
80~100	71	66.4
전체	107	100.0

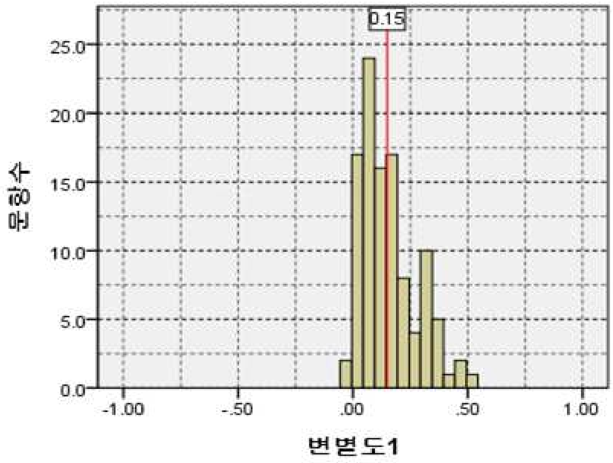

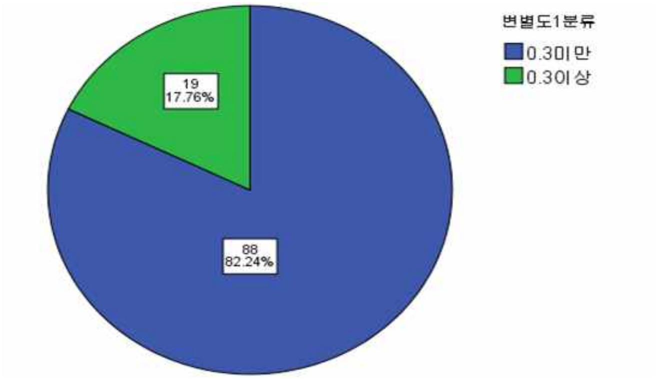

총점	변별도1	표준편차
107	.15	.12

변별도1	문항수	비율(%)
0.3미만	88	82.2
0.3이상	19	17.8
전체	107	100.0

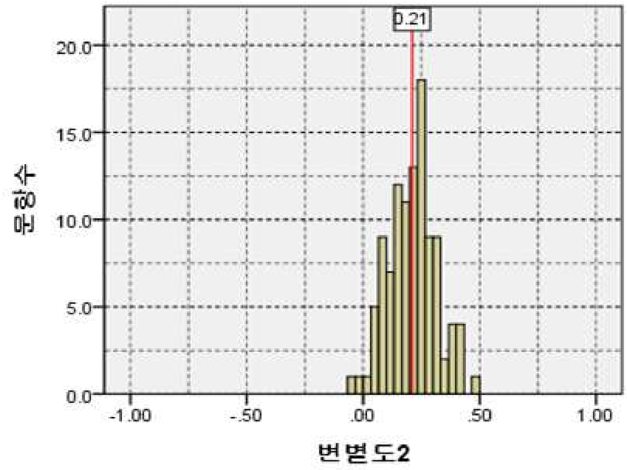

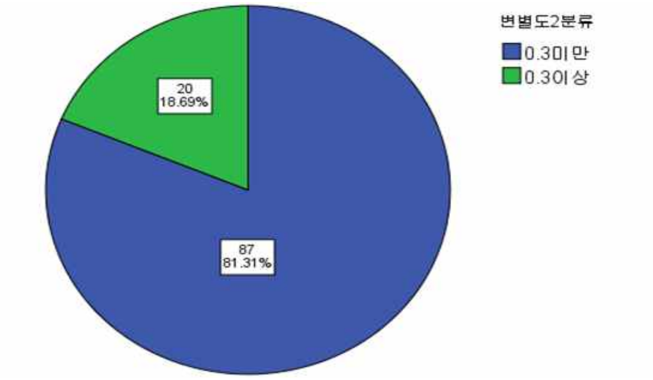

총점	변별도2	표준편차
107	.21	.10

변별도2	문항수	비율(%)
0.3미만	87	81.3
0.3이상	20	18.7
전체	107	100.0

해석

- 해결형 문항에서 난이도 지수가 80 이상 100 사이인 문항이 전체 107 문항 중 71 문항으로 가장 많았으며, 차례로 60 이상 80 미만인 문항이 19 문항, 60 미만인 문항이 17 문항으로 나타남
- 변별도 1 지수를 기준으로 분류하였을 때, 0.3 미만인 문항이 88 문항으로 0.3 이상인 문항이 19 문항인 것에 비해 더 많이 나타남
- 변별도 2 지수를 기준으로 분류하였을 때, 0.3 미만인 문항이 87 문항으로 0.3 이상인 문항이 20 문항인 것에 비해 더 많이 나타남

4) 자료유형별 난이도와 변별도

가) 전회 대비 자료유형별 난이도와 변별도

(1) 전회 대비 텍스트형 난이도와 변별도

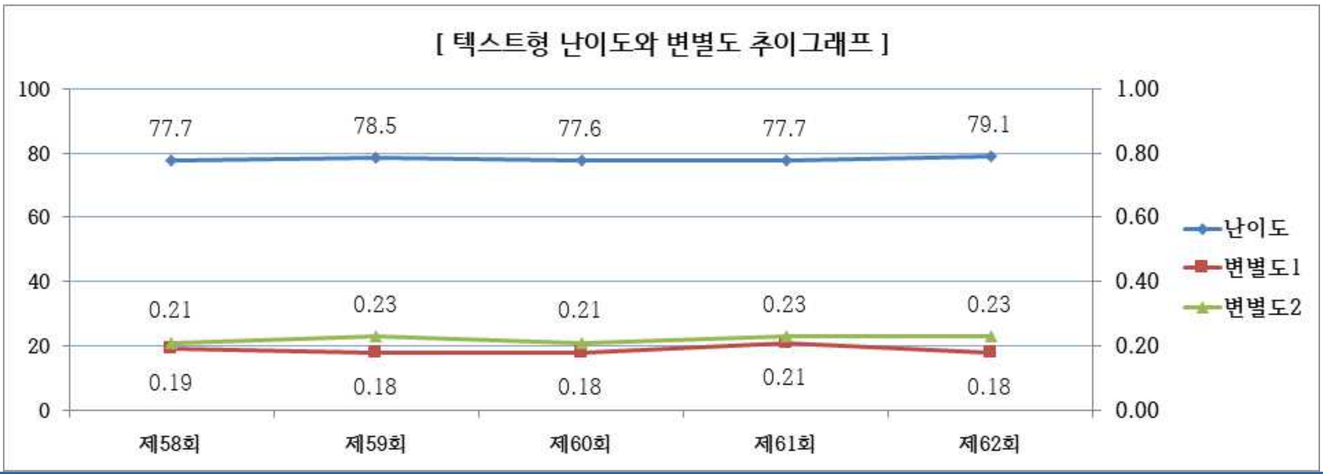

회차	난이도		변별도1		변별도2	
	평균	표준편차	평균	표준편차	평균	표준편차
제58회	77.7	19.7	.19	.12	.21	.09
제59회	78.5	18.5	.18	.12	.23	.09
제60회	77.6	19.9	.18	.11	.21	.10
제61회	77.7	19.1	.21	.13	.23	.09
**제62회**	**79.1**	**19.9**	**.18**	**.13**	**.23**	**.10**

(2) 전회 대비 자료제시형 난이도와 변별도

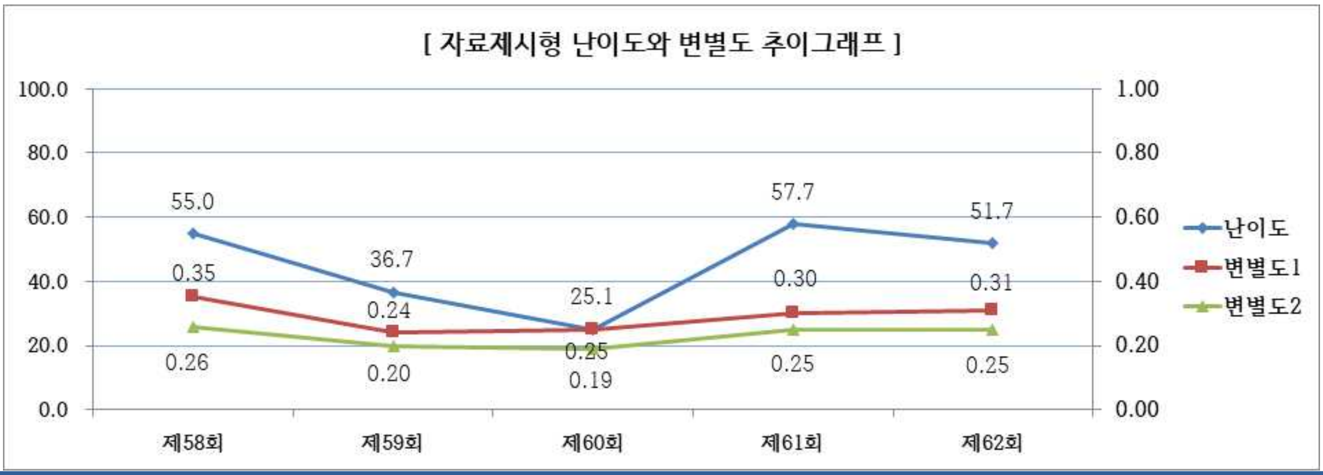

회차	난이도		변별도1		변별도2	
	평균	표준편차	평균	표준편차	평균	표준편차
제58회	55.0	3.7	.35	.10	.26	.09
제59회	36.7	2.7	.24	.01	.20	.02
제60회	25.1	0.0	.25	.00	.19	.00
제61회	57.7	19.0	.30	.17	.25	.15
**제62회**	**51.7**	**7.5**	**.31**	**.06**	**.25**	**.03**

해석

- 전회 대비 텍스트형 문항의 난이도 지수는 1.4 증가하였으나, 자료제시형 문항의 난이도 지수는 6.0 감소함
- 전회 대비 텍스트형 문항의 변별도 1 지수는 0.03 감소, 변별도 2 지수는 변화 없음
- 전회 대비 자료제시형 문항의 변별도 1 지수는 0.01 증가, 변별도 2 지수는 변화 없음

나) 자료유형별 난이도와 변별도 분포도 및 비율분석

(1) 텍스트형 난이도와 변별도 분포도 및 비율분석

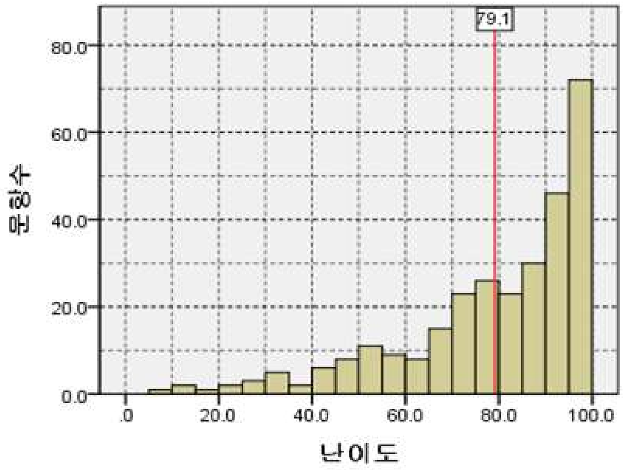

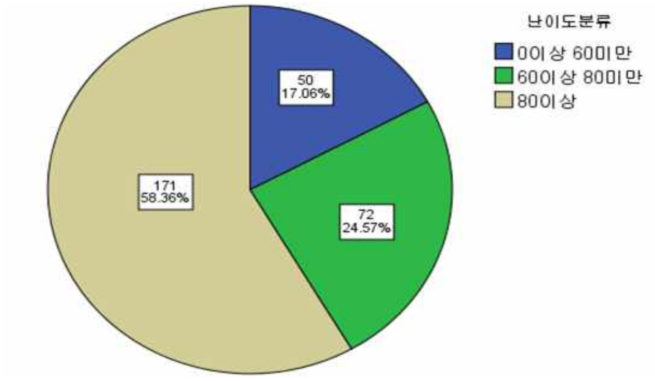

총점	난이도	표준편차
293	79.1	19.9

난이도	문항수	비율(%)
0~60미만	50	17.1
60~80미만	72	24.6
80~100	171	58.4
전체	293	100.0

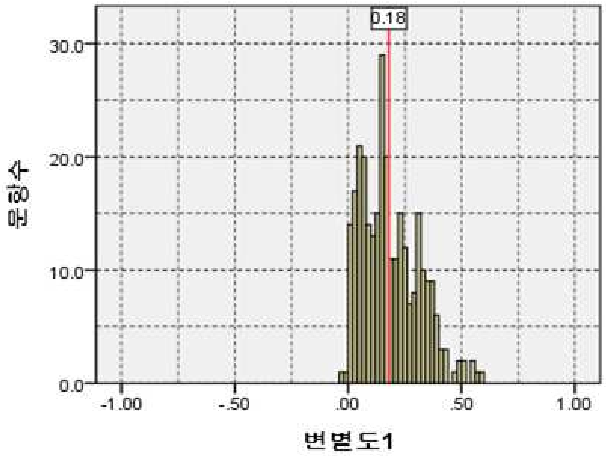

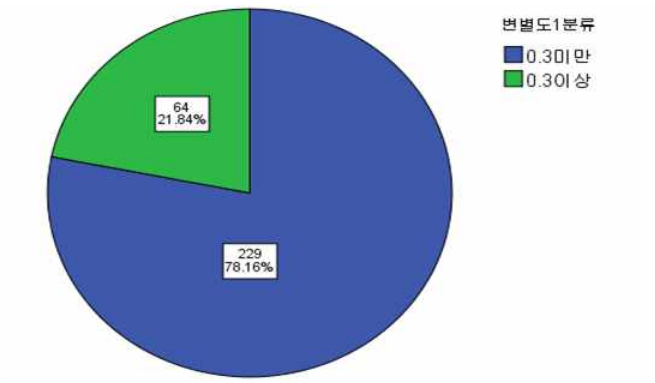

총점	변별도1	표준편차
293	.18	.13

변별도1	문항수	비율(%)
0.3미만	229	78.2
0.3이상	64	21.8
전체	293	100.0

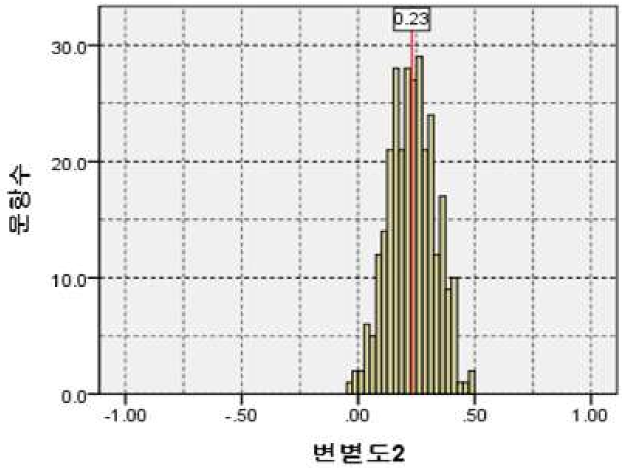

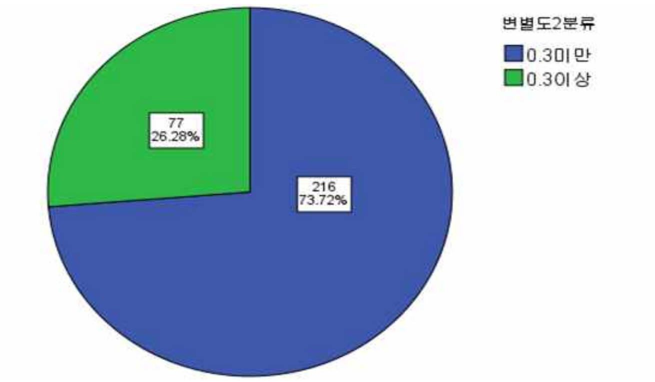

총점	변별도2	표준편차
293	.23	.10

변별도2	문항수	비율(%)
0.3미만	216	73.7
0.3이상	77	26.3
전체	293	100.0

해석

- 텍스트형 문항에서 난이도 지수가 80 에서 100 사이인 문항이 전체 293 문항 중 171 문항으로 가장 많았으며, 차례로 60 이상 80 미만인 문항이 72 문항, 60 미만인 문항이 50 문항인 것으로 나타남
- 변별도 1 지수를 기준으로 분류하였을 때, 0.3 미만인 문항이 229 문항으로 0.3 이상인 문항이 64 문항인 것에 비해 더 많이 나타남
- 변별도 2 지수를 기준으로 분류하였을 때, 0.3 미만인 문항이 216 문항으로 0.3 이상인 문항이 77 문항인 것에 비해 더 많이 나타남

(2) 자료제시형 난이도와 변별도 분포도 및 비율분석

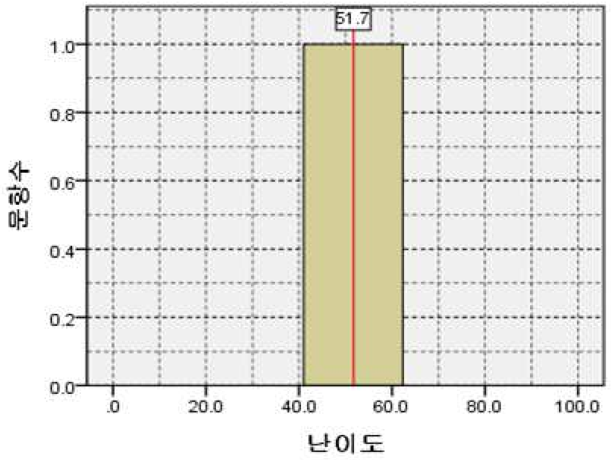

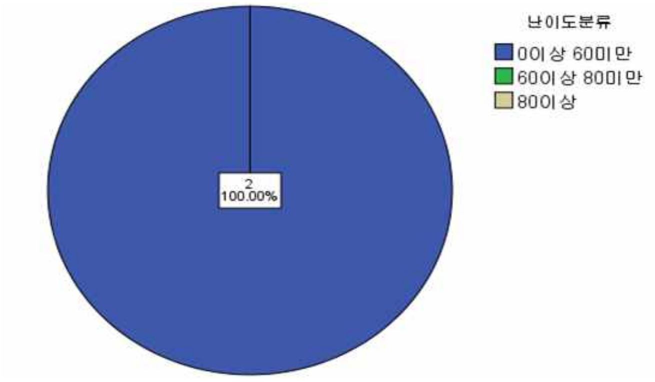

총점	난이도	표준편차
2	51.7	7.5

난이도	문항수	비율(%)
0~60미만	2	100.0
60~80미만	-	0.0
80~100	-	0.0
전체	2	100.0

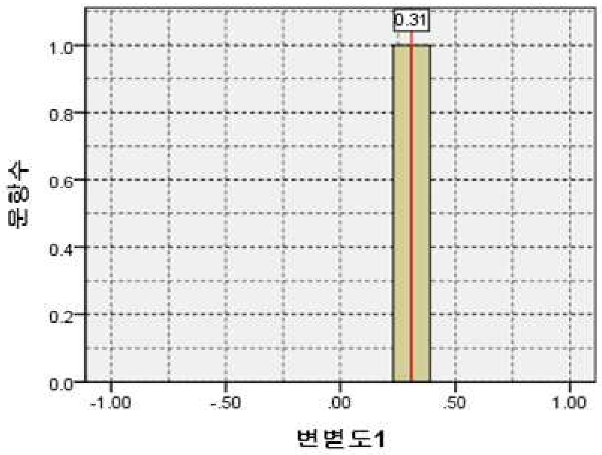

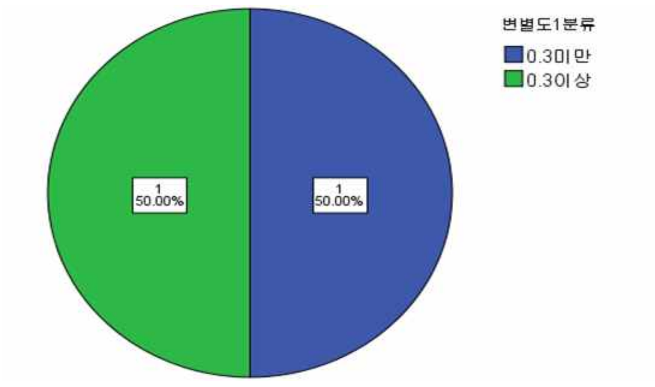

총점	변별도1	표준편차
2	.31	.06

변별도1	문항수	비율(%)
0.3미만	1	50.0
0.3이상	1	50.0
전체	2	100.0

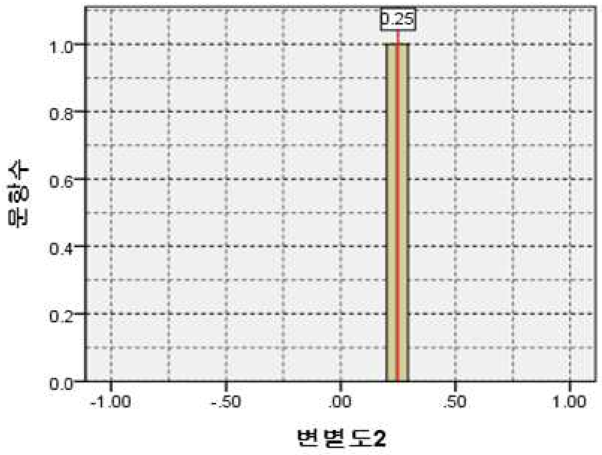

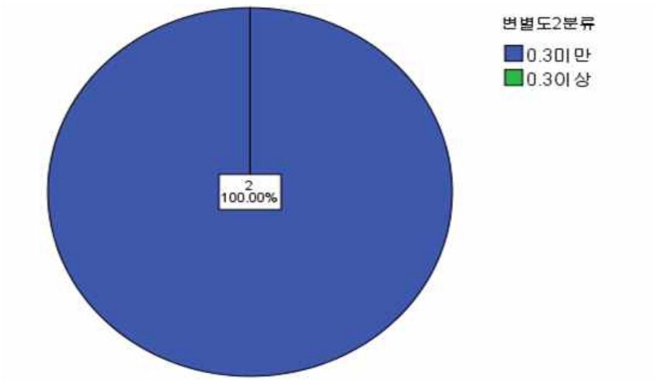

총점	변별도2	표준편차
2	.25	.03

변별도2	문항수	비율(%)
0.3미만	2	100.0
0.3이상	-	0.0
전체	2	100.0

해석

- 자료제시형 문항에서 난이도 지수가 0에서 60미만인 문항이 전체 2문항 중 2문항인 것으로 나타남
- 변별도1 지수를 기준으로 분류하였을 때, 0.3미만인 문항이 1문항으로 0.3이상인 문항이 1문항으로 같게 나타남
- 변별도2 지수를 기준으로 분류하였을 때, 0.3미만인 문항이 2문항으로 0.3이상인 문항이 0문항인 것에 비해 더 많이 나타남

3. 난이도와 변별도 간 산포도

1) 전체 난이도와 변별도 간 산포도

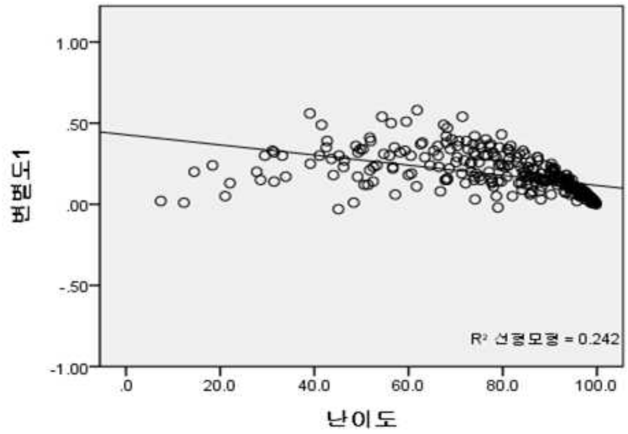

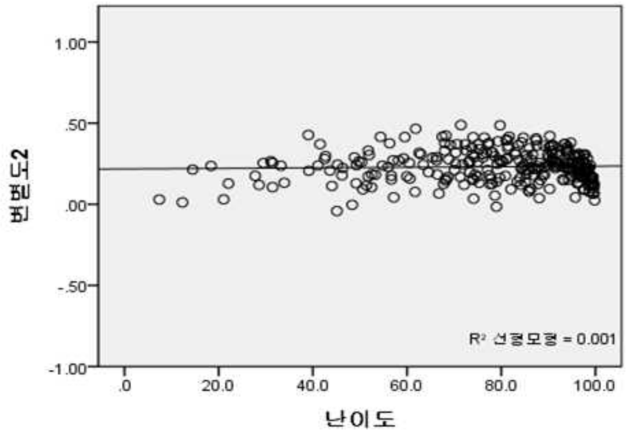

해석

- 전체 문항을 대상으로 난이도와 변별도 1 지수 간 상관은 -.492**로 난이도 지수가 높을수록 변별력이 낮아지는 것으로 나타남
- 난이도와 변별도 2 지수 간 상관은 .034 로 문항 난이도와 변별도 간 관련성이 없는 것으로 나타남

2) 과목별 난이도와 변별도 간 산포도

가) 성인간호학 난이도와 변별도 간 산포도

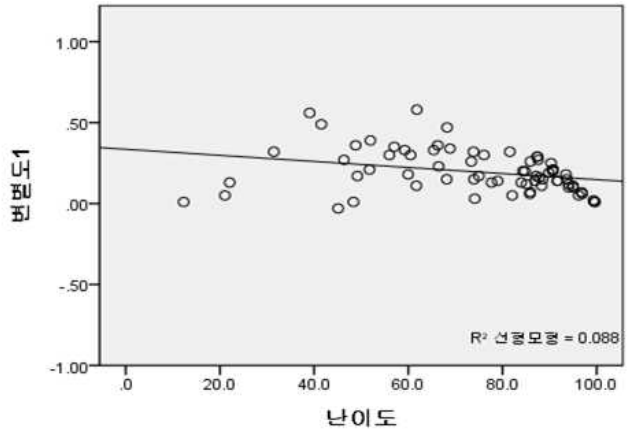

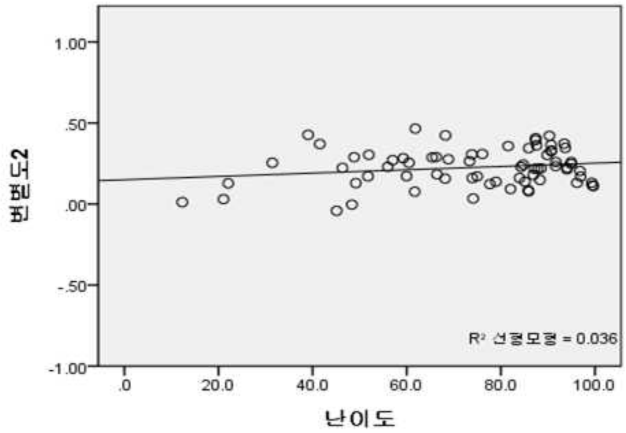

해석

- 성인간호학 과목을 대상으로 난이도와 변별도 1 지수 간 상관은 -.296*으로 난이도 지수가 높을수록 변별력이 낮아지는 것으로 나타남
- 난이도와 변별도 2 지수 간 상관은 .189 로 문항 난이도와 변별도 간 관련성이 낮은 것으로 나타남

나) 모성간호학 난이도와 변별도 간 산포도

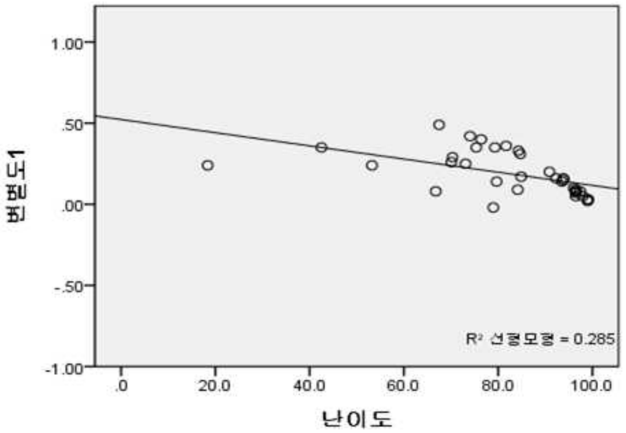

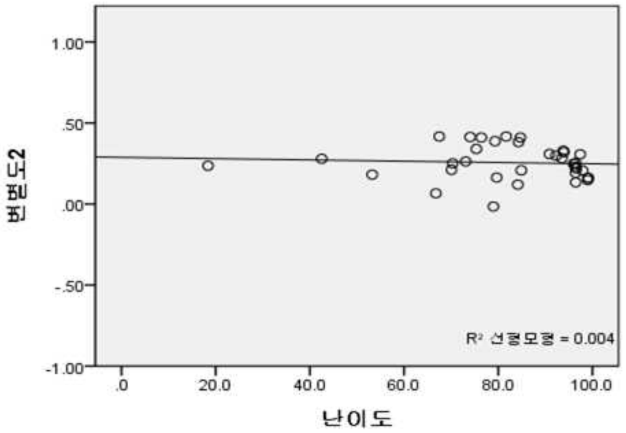

해석

- 모성간호학 과목을 대상으로 난이도와 변별도 1 지수 간 상관은 -.534*로 난이도 지수가 높을수록 변별력이 낮아지는 것으로 나타남
- 난이도와 변별도 2 지수 간 상관은 -.066 으로 문항 난이도와 변별도 간 관련성이 없는 것으로 나타남

다) 아동간호학 난이도와 변별도 간 산포도

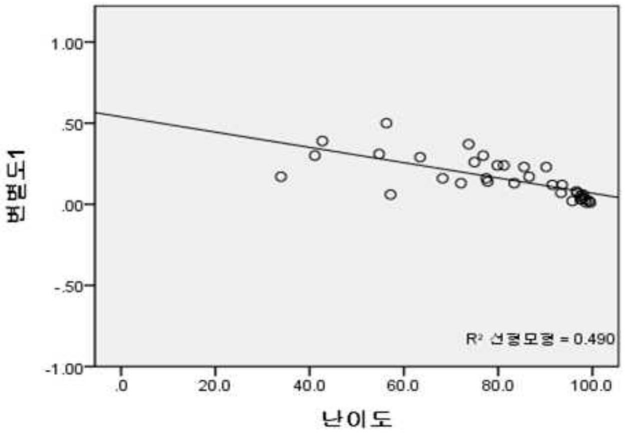

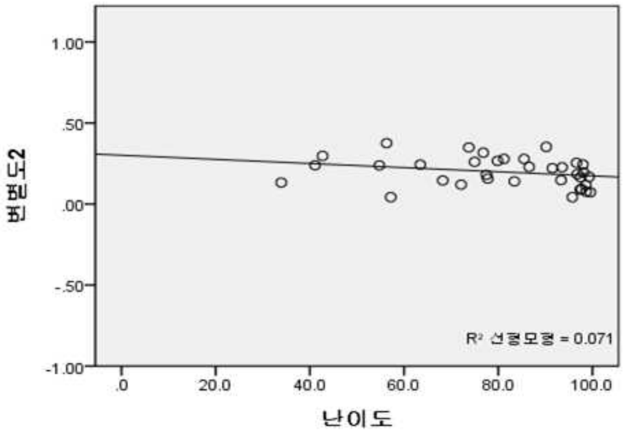

해석

- 아동간호학 과목을 대상으로 난이도와 변별도 1 지수 간 상관은 -.700**으로 난이도 지수가 높을수록 변별력이 낮아지는 것으로 나타남
- 난이도와 변별도 2 지수 간 상관은 -.267 로 문항 난이도와 변별도 간 관련성이 낮은 것으로 나타남

라) 지역사회간호학 난이도와 변별도 간 산포도

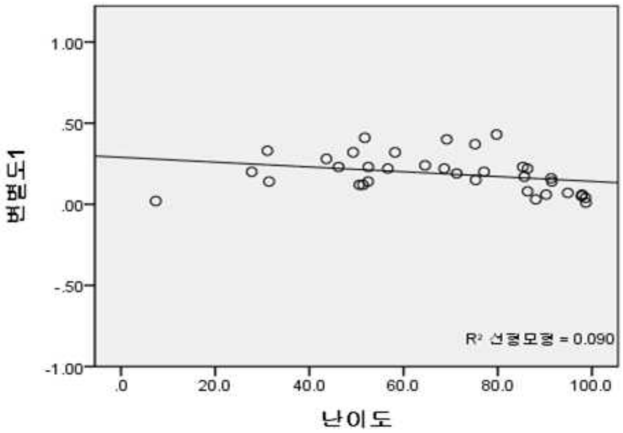

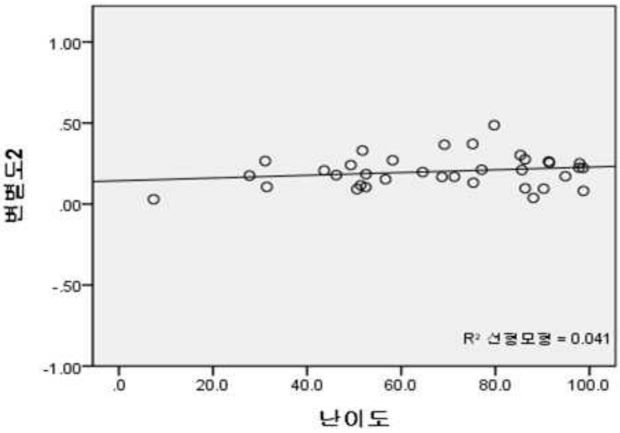

해석

- 지역사회간호학 과목을 대상으로 난이도와 변별도 1 지수 간 상관은 -.300 으로 문항 난이도와 변별도 간 관련성이 낮은 것으로 나타남
- 난이도와 변별도 2 지수 간 상관은 .201 로 문항 난이도와 변별도 간 관련성이 낮은 것으로 나타남

마) 정신간호학 난이도와 변별도 간 산포도

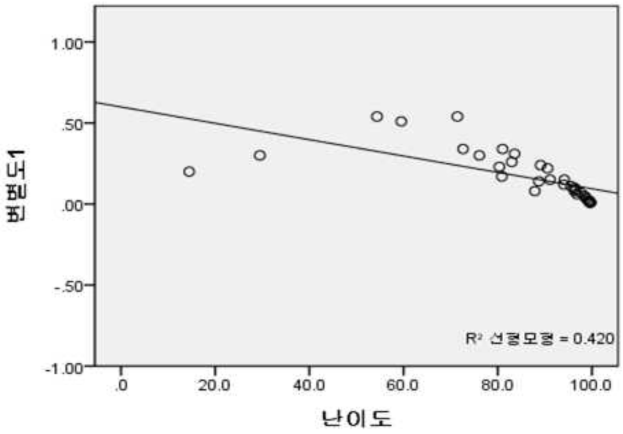

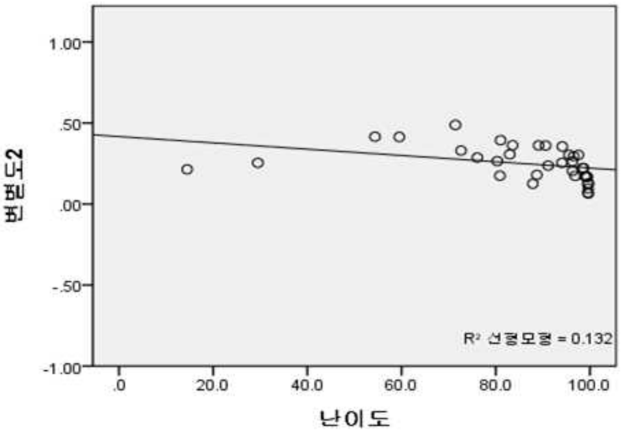

해석

- 정신간호학 과목을 대상으로 난이도와 변별도 1 지수 간 상관은 -.648**로 난이도 지수가 높을수록 변별력이 낮아지는 것으로 나타남
- 난이도와 변별도 2 지수 간 상관은 -.364*로 난이도 지수가 높을수록 변별력이 낮아지는 것으로 나타남

바) 간호관리학 난이도와 변별도 간 산포도

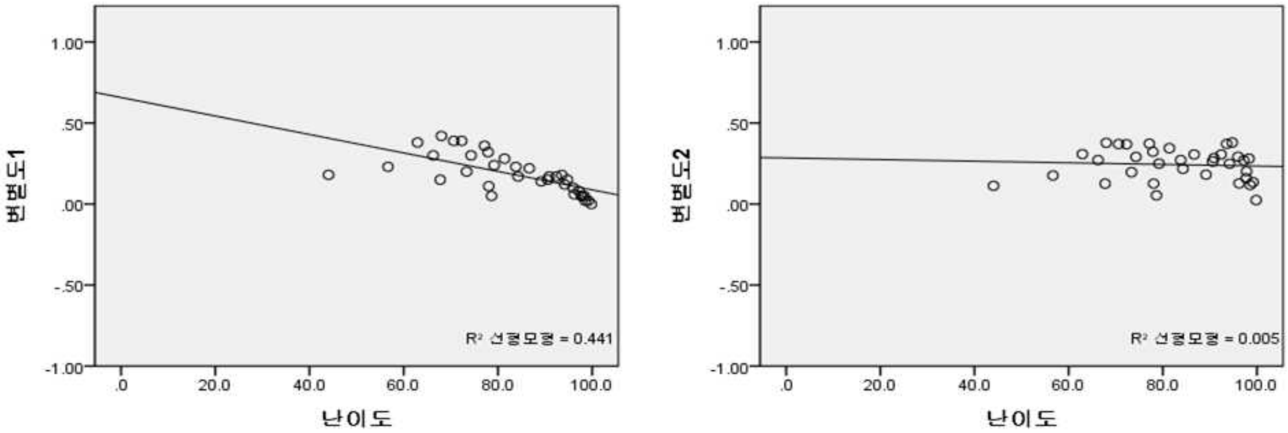

해석

- 간호관리학 과목을 대상으로 난이도와 변별도 1 지수 간 상관은 -.664*로 난이도 지수가 높을수록 변별력이 낮아지는 것으로 나타남
- 난이도와 변별도 2 지수 간 상관은 -.070 으로 문항 난이도와 변별도 간 관련성이 없는 것으로 나타남

사) 기본간호학 난이도와 변별도 간 산포도

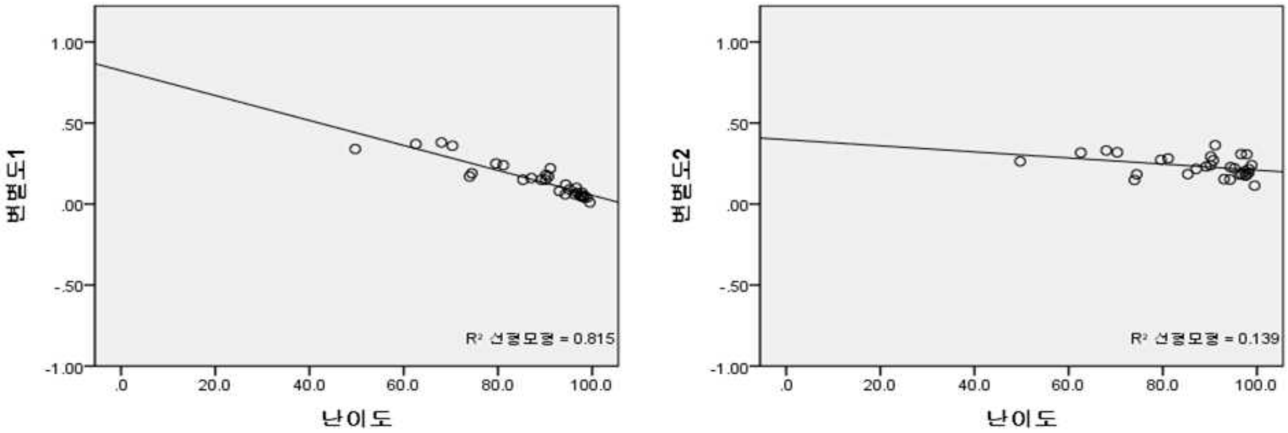

해석

- 기본간호학 과목을 대상으로 난이도와 변별도1 지수 간 상관은 -.903**으로 난이도 지수가 높을수록 변별력이 낮아지는 것으로 나타남
- 난이도와 변별도2 지수 간 상관은 -.373*으로 난이도 지수가 높을수록 변별력이 낮아지는 것으로 나타남

아) 보건의약관계법규 난이도와 변별도 간 산포도

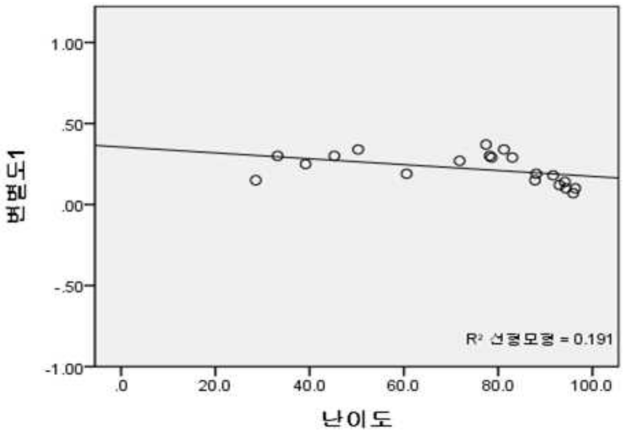

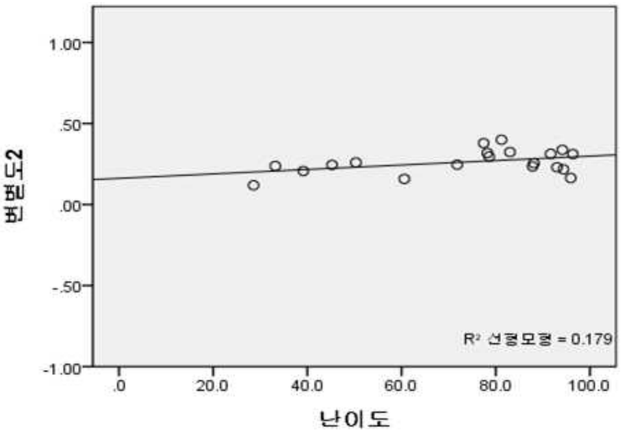

해석

- 보건의약관계법규 과목을 대상으로 난이도와 변별도1 지수 간 상관은 -.437로 문항 난이도와 변별도 간 관련성이 낮은 것으로 나타남
- 난이도와 변별도2 지수 간 상관은 .423로 문항 난이도와 변별도 간 관련성이 낮은 것으로 나타남

4. 신뢰도 분석

과목명	문항수	제58회	제59회	제60회	제61회	제62회
전체	295	.934	.934	.929	.944	**.934**
성인간호학	70	.775	.796	.792	.849	**.769**
모성간호학	35	.688	.625	.710	.710	**.693**
아동간호학	35	.616	.492	.451	.629	**.567**
지역사회간호학	35	.623	.695	.562	.550	**.564**
정신간호학	35	.621	.669	.635	.691	**.713**
간호관리학	35	.654	.662	.651	.694	**.673**
기본간호학	30	.641	.647	.530	.564	**.616**
보건의약관계법규	20	.533	.519	.651	.663	**.582**

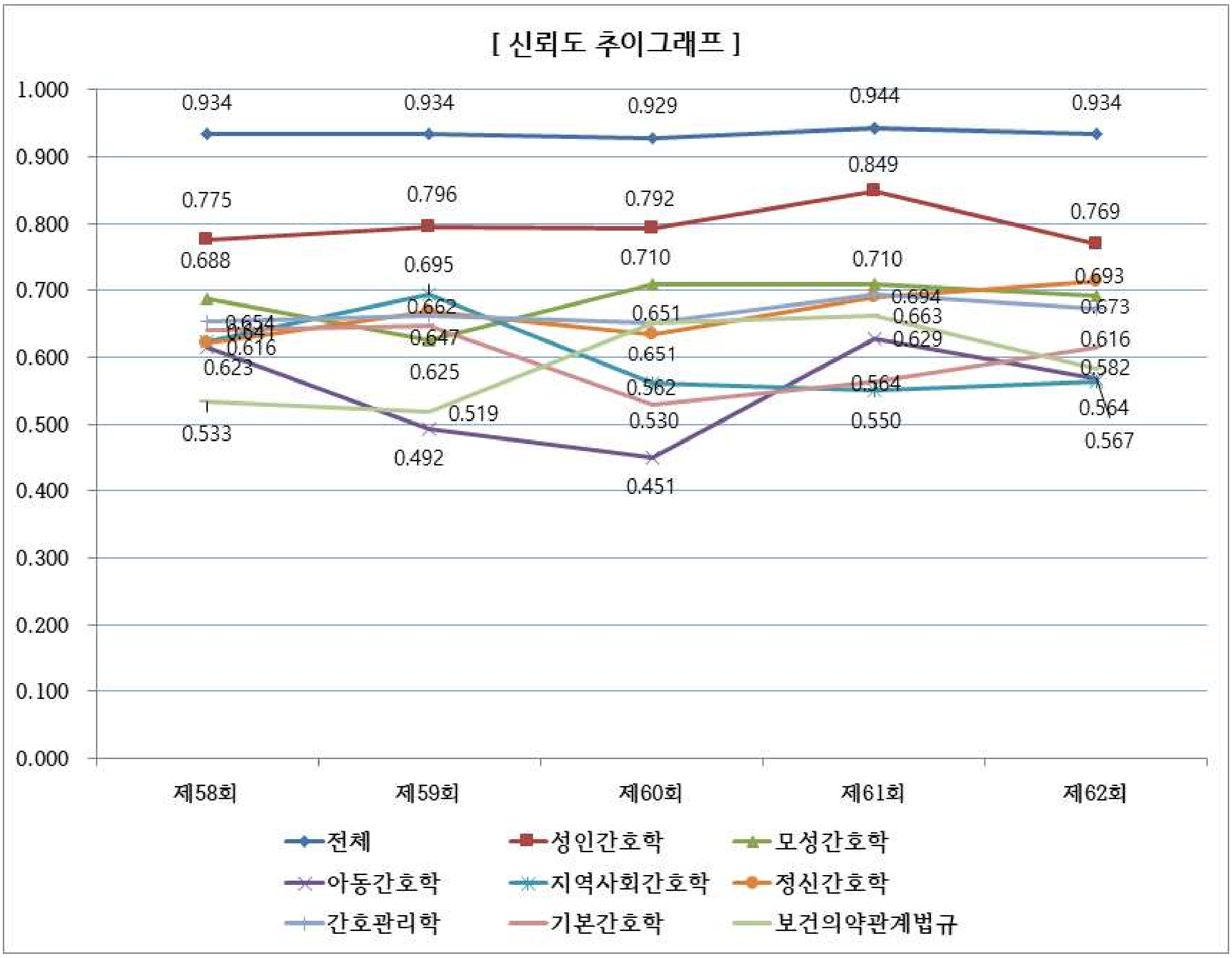

해석

- 간호사 국가시험 전체 문항에서 일관되게 해당 영역을 측정하고 있는 것으로 나타남
- 전회 대비 신뢰도는 전체문항, 모성간호학, 성인간호학, 아동간호학, 간호관리학, 보건의약관계법규 과목을 대상으로 했을 시 0.010, 0.080, 0.017, 0.062, 0.021 감소함
- 지역사회간호학, 정신간호학. 기본간호학 과목의 경우 0.014, 0.022, 0.052 증가함
