# Supplementary material for: Item difficulty index, discrimination index, and reliability of the 26 health professions licensing examinations in 2022, Korea: a psychometric study
Source: J Educ Eval Health Prof. 2023 Nov 22;20:31. doi: 10.3352/jeehp.2023.20.31 (PMC11959405; doi:10.3352/jeehp.2023.20.31)
Supplement: Supplementary file 1 — Supplement 1. Item analysis results of 26 health professions licensing examinations administered during late 2022 and early 2023. [file jeehp-20-31_Suppl1.zip › 2022│Γ╡╡ ┴a6╚╕ 1▒▐ └σ╛╓└╬└τ╚░╗≤┤π╗τ ▒╣░í╜├╟Φ ║╨╝«░ß░·.pdf]

2022년도 제6회
1급 장애인재활상담사
국가시험 문항분석 결과

국민이 신뢰하고 감동하는 시험평가기관
한국보건의료인국가시험원
KOREA HEALTH PERSONNEL LICENSING EXAMINATION INSTITUTE

일반 용어 정의

□ **평균**
- 집단에서의 대표적 경향 값으로 전체 값을 더하여 총 응시자로 나눈 값

□ **표준편차**
- 평균과 각 점수의 차이인 편차들의 평균으로 점수가 흩어져 분포되어 있는 정도

□ **추정난이도**
- 문항개발자가 예측한 정답률

□ **검사이론**
- 검사와 검사를 구성하고 있는 문항의 양호도를 분석 및 평가하는 방법을 정의한 이론체계
- 대표적으로 고전검사이론과 문항반응이론이 있음

고전검사이론 용어 정의

□ 고전검사이론(Classical Test Theory; CTT)

- 검사의 질을 분석하는 검사이론 중 한 가지로 19세기 말부터 전개되어 현재까지 주로 사용되고 있는 검사이론임
- 고전검사이론에 의한 문항과 응시자 능력 추정치는 다음과 같음

○ **문항난이도**

· 검사 문항의 쉽고 어려운 정도를 나타내는 지수

· 난이도 지수는 총 반응 수에 대한 정답 반응 수의 비율로 문항의 정답률임

· 문항난이도는 0~100까지의 값을 가짐

· 난이도 값이 큰 경우, 쉬운 문항으로 '난이도가 낮다'라고 해석하며, 난이도 값이 작은 경우, 어려운 문항으로 '난이도가 높다'라고 해석함

○ **문항변별도**

· 각 문항이 응시자의 능력 수준을 변별할 수 있는 정도를 나타내는 지수

· 문항변별도는 -1~+1까지의 값을 가지며, 1에 가까울수록 변별력 크다고 해석함

· 일반적으로 문항변별도가 0.3 이상이면 우수한 문항으로 평가함

· 구하는 방식에는 '상하위집단 구분법', '문항-총점 상관계수' 등이 있음

1) **변별도 1(상하위구분법):** 상위 27%와 하위 27% 집단의 난이도 차이를 구하는 방식

2) **변별도 2(상관계수법):** 문항-총점과의 상관계수로 구하는 방식

○ **신뢰도**

· 시험이 평가하고자 하는 것을 일관성 있게 측정하는가로 시험이 오차없이 정확하게 측정한 정도를 의미함

· 국시원에서는 문항의 내적일관성(Cronbach α)으로 신뢰도를 추정하며 1에 가까울수록 신뢰도가 높다고 해석함

목 차

I. 시행 결과 ······ 6
1. 시험 현황 ······ 7
1) 시험명 ······ 7
2) 시험시행일 ······ 7
3) 응시현황 ······ 7
4) 과목별 문항 수, 배점 및 과락 점수 ······ 7
2. 합격률과 평균성적 ······ 7
1) 합격 및 불합격 현황 ······ 7
2) 과목별 과락자수 내역 ······ 7
3) 전회 대비 합격률과 평균성적 ······ 8

II. 문항분석 결과 ······ 9
1. 성적 ······ 10
1) 전체 성적분포도 ······ 10
2) 과목별 성적분포도 ······ 11
2. 난이도와 변별도 ······ 13
1) 전체 난이도와 변별도 ······ 13
2) 과목별 난이도와 변별도 ······ 16
3) 지식수준별 난이도와 변별도 ······ 34
3. 난이도와 변별도 간 산포도 ······ 42
1) 전체 난이도와 변별도 간 산포도 ······ 42
2) 과목별 난이도와 변별도 간 산포도 ······ 42
4. 신뢰도 분석 ······ 47

I. 시행 결과

1. 시험 현황

1) 시험명: 2022년도 제6회 1급 장애인재활상담사 국가시험

2) 시험시행일: 2022년 2월 25일

3) 응시현황

응시대상자수	결시자수	부정행위자수	응시자 준수사항 위반자 수		응시자수 (%)
			휴대폰 소지	신분증 미지참	
459	40	-	-	-	412(90.9)

※ 채점보류자 1인 제외

4) 과목별 문항 수, 배점 및 과락 점수

교 시	과 목 명	문제 수	배점	총점	합격자 점수기준	
					과목 과락기준	총점 합격기준
1교시	직업재활개론	22	1	22	8.8	72 이상
	재활상담	22	1	22	8.8	
	재활사례관리	22	1	22	8.8	
2교시	직업평가	12	1	12	4.8	
	직무개발과 배치	17	1	17	6.8	
	재활행정	10	1	10	4.0	
	재활정책	15	1	15	6.0	
계		120		120		

2. 합격률과 평균성적

1) 합격 및 불합격 현황

합격자수 (%)	불합격자수(%)				채점보류자수
	평락	과락	기권	계	
376 (91.3)	27 (6.6)	9 (2.2)	0 (0.0)	36 (8.7)	1

2) 과목별 과락자수 내역

과목명 / 과락자수	직업재활 개론	재활상담	재활사례 관리	직업평가	직무개발 과 배치	재활행정	재활정책
과목별 과락자 수	1	2	-	2	-	1	3
2과목 과락자 수	-						

3) 전회 대비 합격률과 평균성적

회차	년도	합격률(%)	평균성적	표준편차	백분율 환산점수
제2회	2018	40.1	69.7	10.2	58.1
제3회	2019	59.7	76.0	10.5	63.3
제4회	2020	79.2	82.2	10.7	68.5
**제5회**	**2021**	**69.3**	**80.1**	**12.9**	**66.9**
**제6회**	**2022**	**91.3**	**90.7**	**12.1**	**75.5**

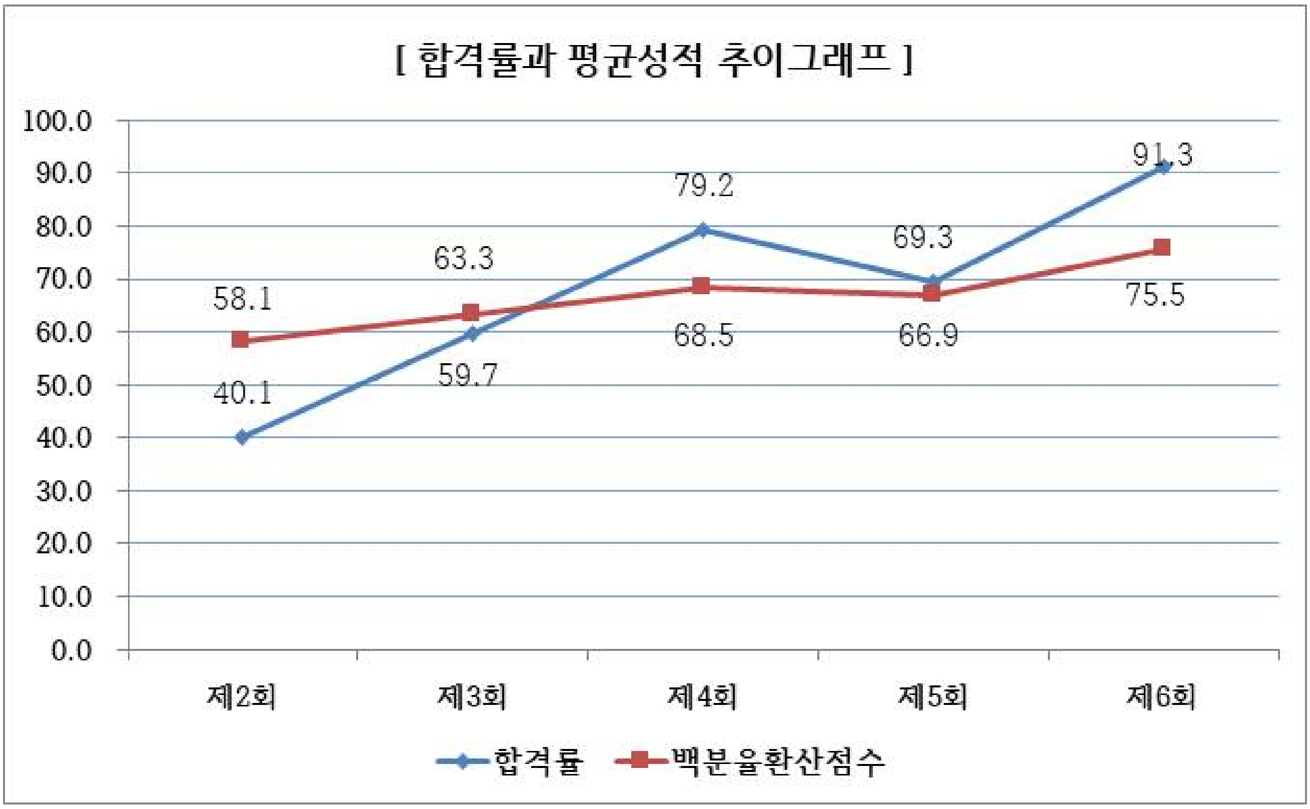

해석

- 전년 대비 합격률은 22.0%, 백분율 환산점수는 8.6 점 증가함
- 표준편차는 0.8 점 감소함

Ⅱ. 문항분석 결과

1. 성적

1) 전체 성적분포도

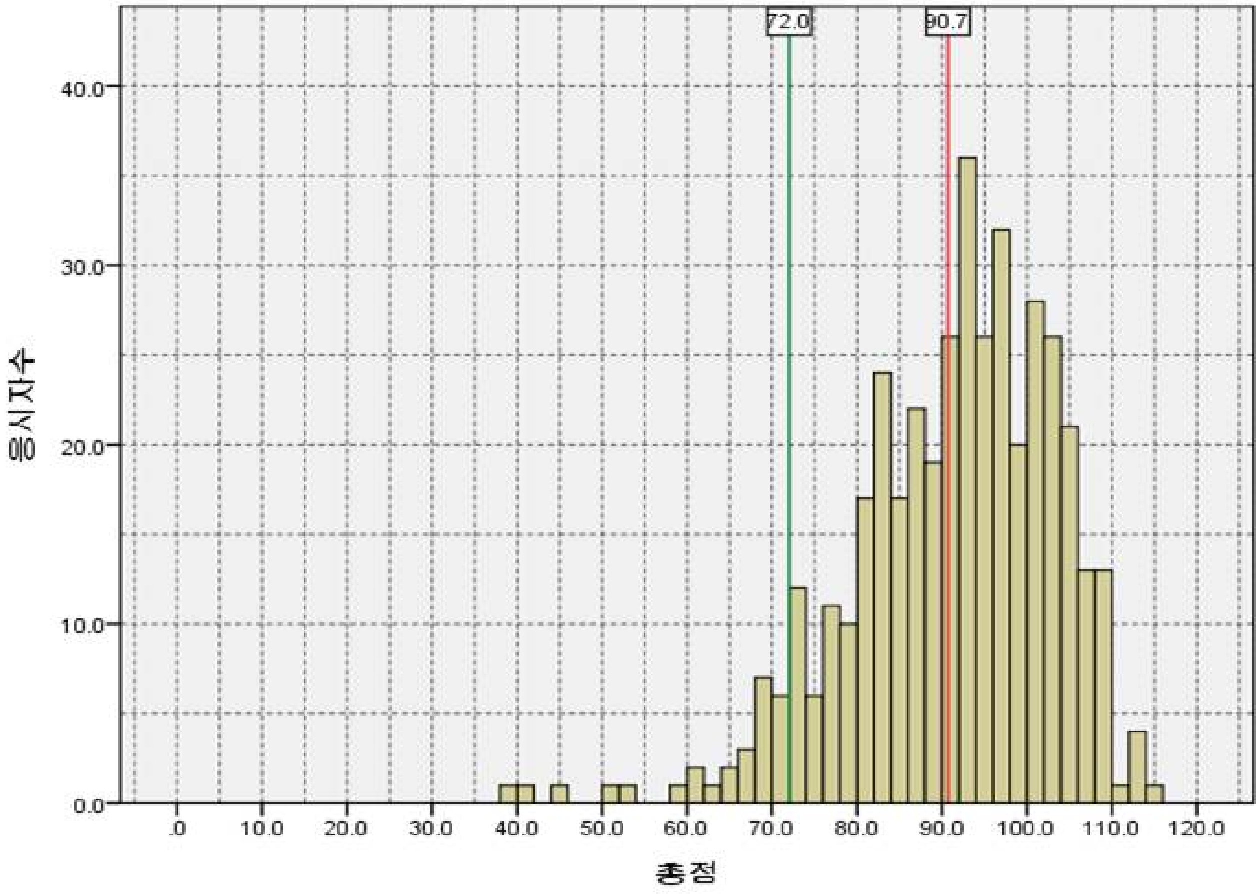

응시자	총점	합격선	평균성적	표준편차
412	120	72	90.7	12.1

2) 과목별 성적분포도

가) 직업재활개론

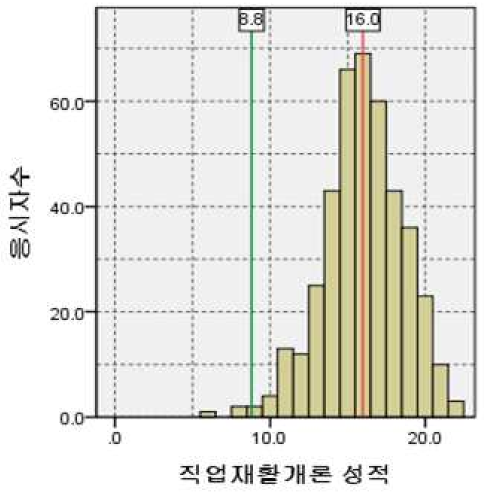

총점	과락선	평균성적	표준편차
22	8.8	16.0	2.6

나) 재활상담

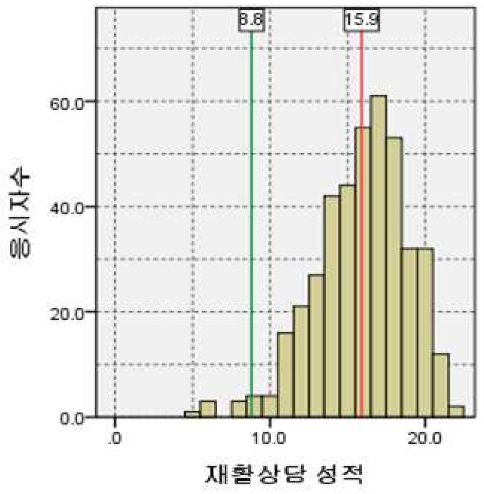

총점	과락선	평균성적	표준편차
22	8.8	15.9	3.0

다) 재활사례관리

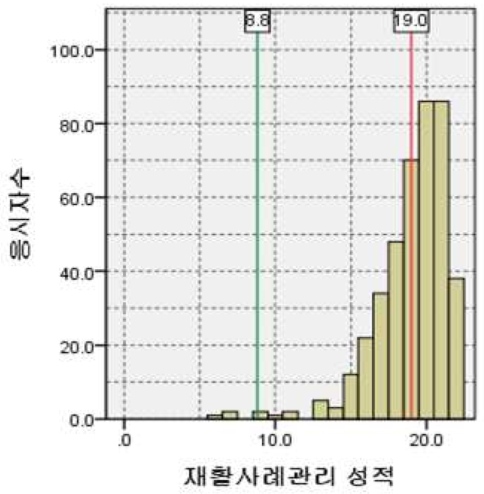

총점	과락선	평균성적	표준편차
22	8.8	19.0	2.4

직업평가

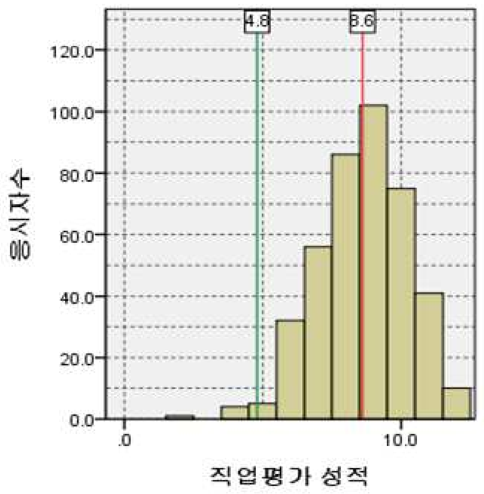

총점	과락선	평균성적	표준편차
12	4.8	8.6	1.6

라) 직무개발과 배치

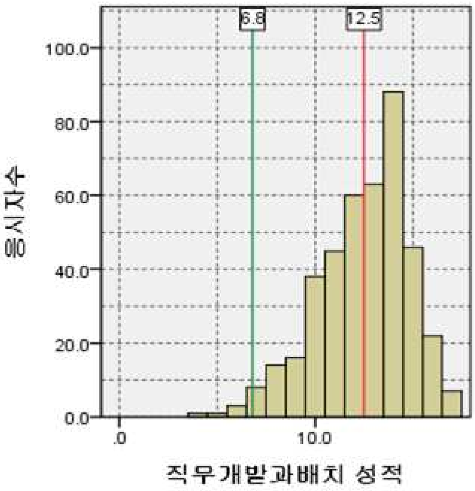

총점	과락선	평균성적	표준편차
17	6.8	12.5	2.3

마) 재활행정

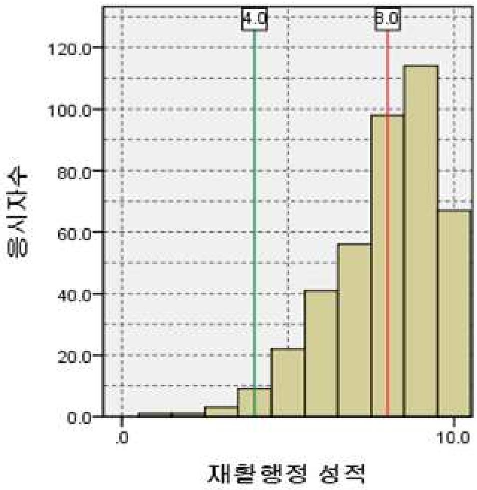

총점	과락선	평균성적	표준편차
10	4.0	8.0	1.6

바) 재활정책

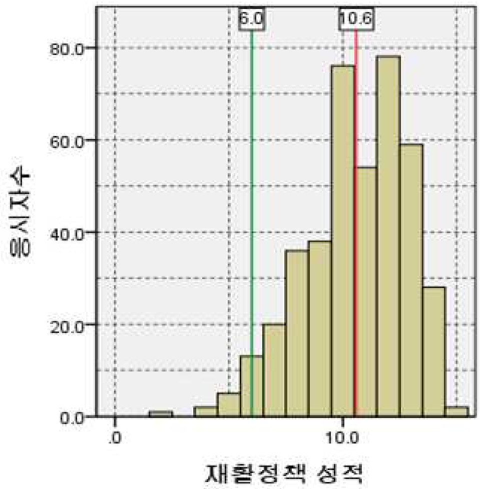

총점	과락선	평균성적	표준편차
15	6.0	10.6	2.3

2. 난이도와 변별도

1) 전체 난이도와 변별도

가) 전회 대비 전체 난이도와 변별도

회차	난이도		변별도1		변별도2	
	평균	표준편차	평균	표준편차	평균	표준편차
제2회	58.0	24.4	0.20	0.14	0.16	0.11
제3회	63.3	23.4	0.21	0.13	0.21	0.11
제4회	68.5	24.5	0.21	0.14	0.23	0.11
제5회	67.0	24.1	0.24	0.14	0.28	0.11
**제6회**	**75.4**	**19.1**	**0.24**	**0.16**	**0.27**	**0.11**

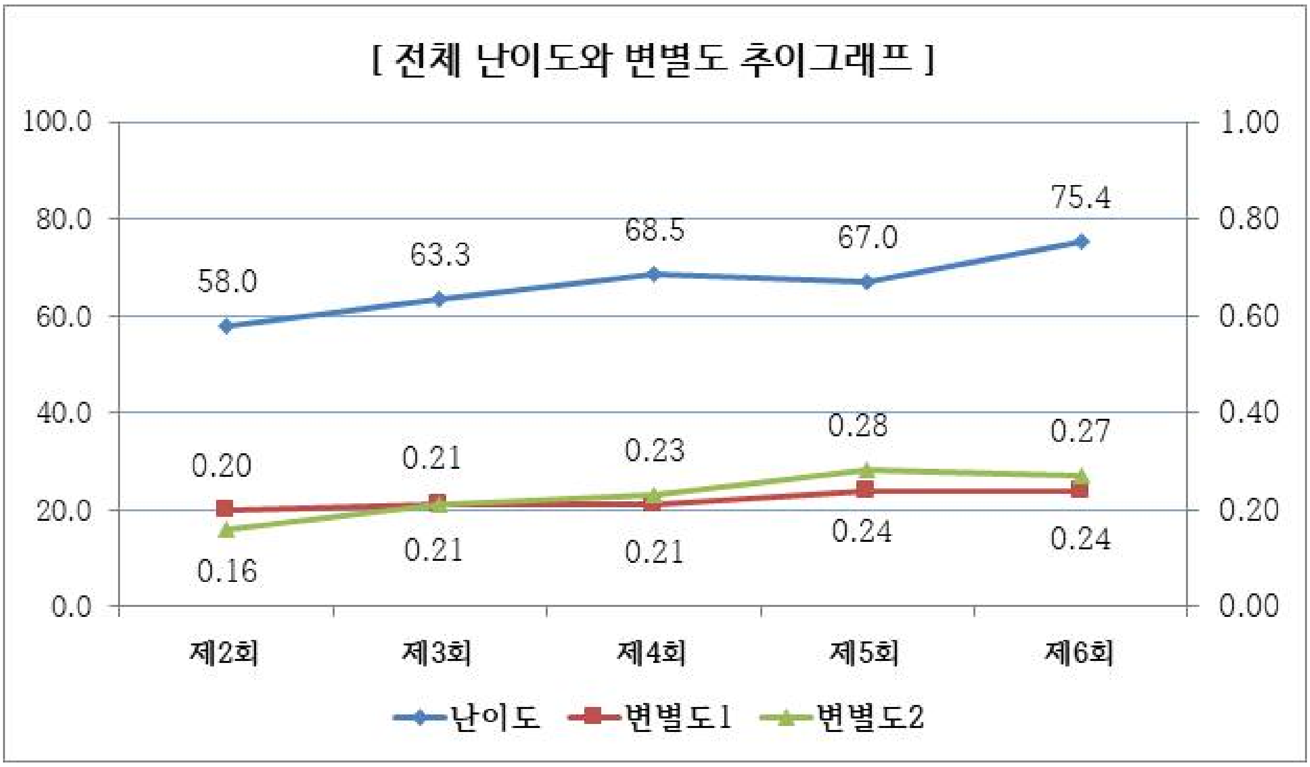

해석

- 전년 대비 난이도 지수는 8.4 증가함
- 변별도 1 지수는 변화 없으며, 변별도 2 지수는 0.01 감소함

나) 전회 대비 전체 난이도와 변별도

(1) 전체 난이도 분포도 및 비율분석

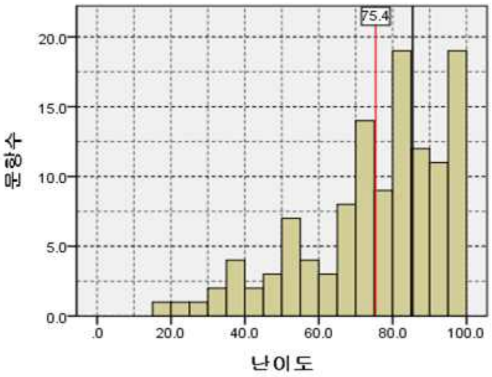

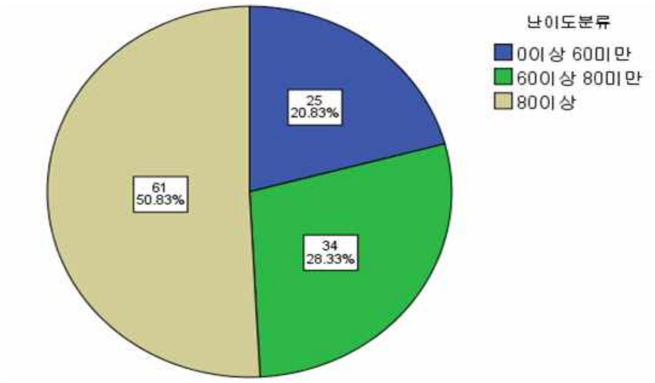

총점	난이도	표준편차
120	75.4	19.1

난이도	문항수	비율(%)
0~60미만	25	20.8
60~80미만	34	28.3
80~100	61	50.8
전체	120	100.0

(2) 전체 변별도1 분포도 및 비율분석

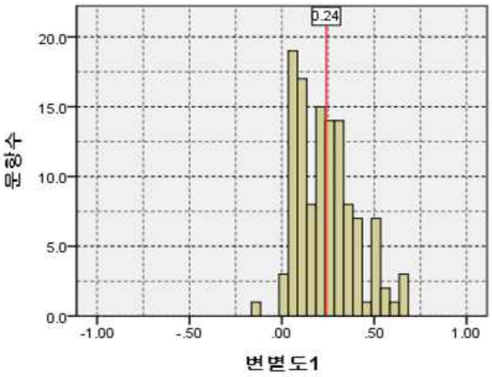

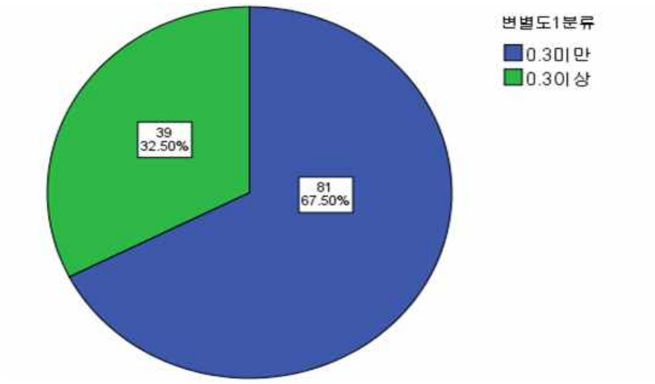

총점	변별도1	표준편차
120	.24	.16

변별도1	문항수	비율(%)
0.3미만	81	67.5
0.3이상	39	32.5
전체	120	100.0

(3) 전체 변별도2 분포도 및 비율분석

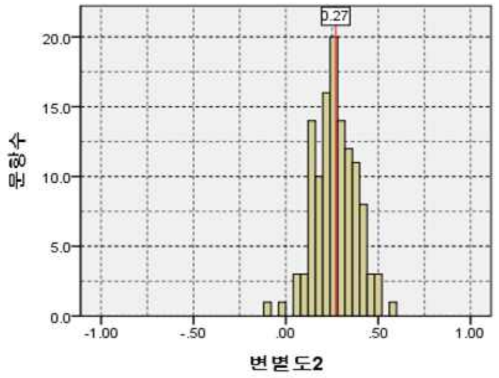

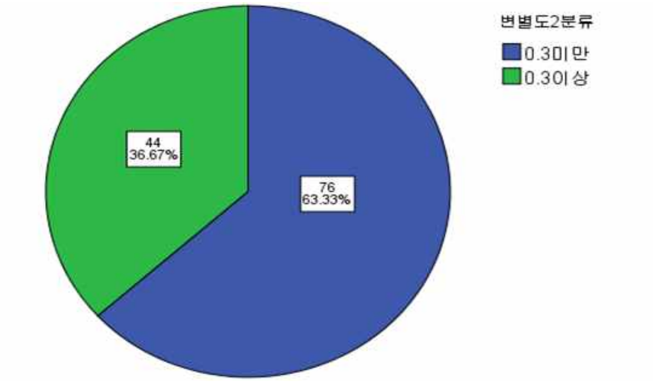

총점	변별도2	표준편차
120	.27	.11

변별도2	문항수	비율(%)
0.3미만	76	63.3
0.3이상	44	36.7
전체	120	100.0

해석

- 난이도 지수가 80에서 100 사이인 문항이 전체 120문항 중 61문항으로 가장 많았으며, 60이상 80미만인 문항이 34문항, 60미만인 문항이 25문항인 것으로 나타남
- 변별도1 지수를 기준으로 분류하였을 때, 0.3미만인 문항이 81문항으로 0.3 이상인 문항이 39문항인 것에 비해 더 많게 나타남
- 변별도2 지수를 기준으로 분류하였을 때, 0.3미만인 문항이 76문항으로 0.3 이상인 문항이 44문항인 것에 비해 더 많게 나타남

2) 과목별 난이도와 변별도

가) 과목별 난이도와 변별도 분포도 및 비율분석

(1) 전회 대비 직업재활개론 및 평가 난이도와 변별도

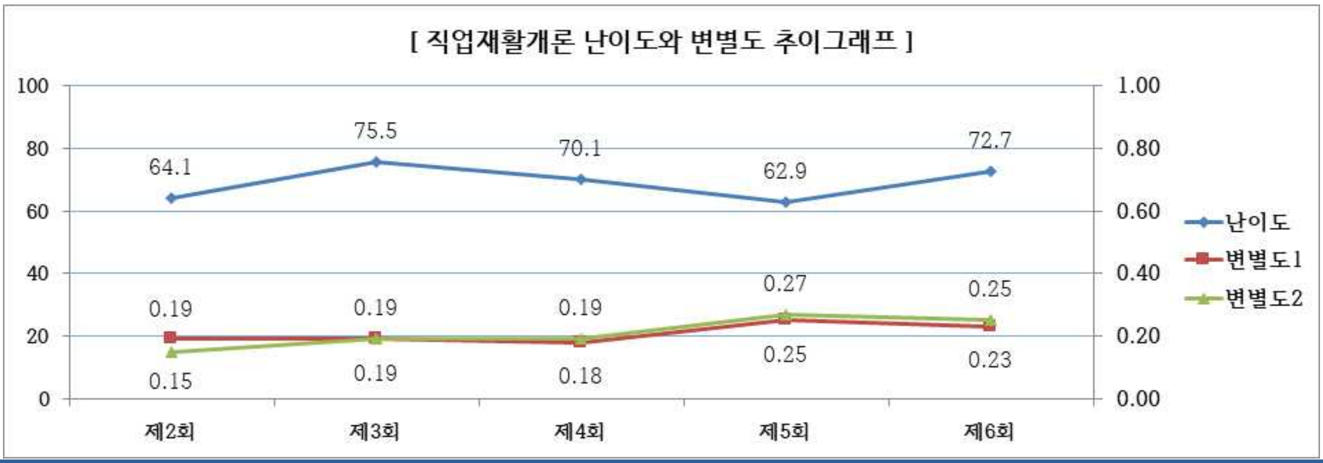

회차	난이도		변별도1		변별도2	
	평균	표준편차	평균	표준편차	평균	표준편차
제2회	64.1	25.5	0.19	0.12	0.15	0.08
제3회	75.5	21.2	0.19	0.15	0.19	0.15
제4회	70.1	25.4	0.18	0.11	0.19	0.10
제5회	62.9	26.4	0.25	0.15	0.27	0.12
**제6회**	**72.7**	**22.1**	**0.23**	**0.16**	**0.25**	**0.12**

(2) 전회 대비 재활상담 난이도와 변별도

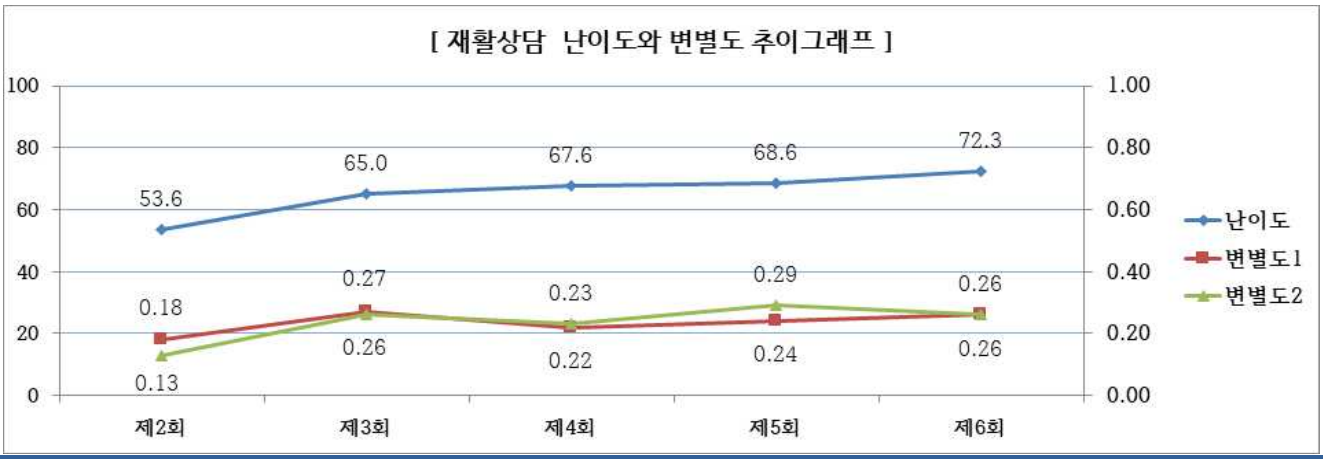

회차	난이도		변별도1		변별도2	
	평균	표준편차	평균	표준편차	평균	표준편차
제2회	53.6	24.6	0.18	0.15	0.13	0.14
제3회	65.0	22.0	0.27	0.12	0.26	0.08
제4회	67.6	25.7	0.22	0.12	0.23	0.09
제5회	68.6	22.3	0.24	0.13	0.29	0.10
**제6회**	**72.3**	**14.8**	**0.26**	**0.18**	**0.26**	**0.14**

(3) 전회 대비 재활사례관리 난이도와 변별도

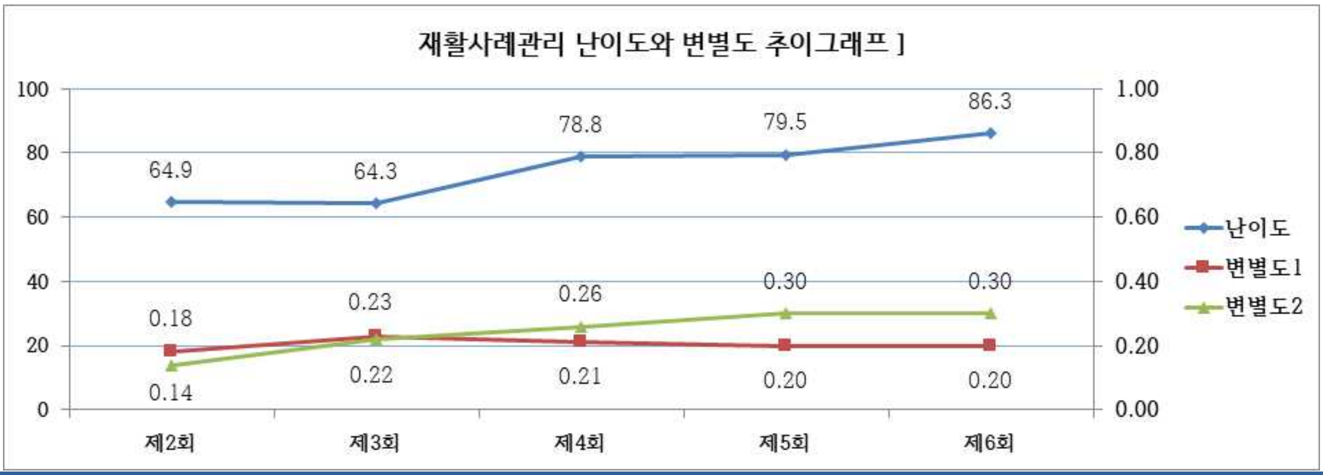

회차	난이도		변별도1		변별도2	
	평균	표준편차	평균	표준편차	평균	표준편차
제2회	64.9	21.1	0.18	0.11	0.14	0.09
제3회	64.3	21.1	0.23	0.14	0.22	0.11
제4회	78.8	19.6	0.21	0.16	0.26	0.11
제5회	79.5	19.2	0.20	0.15	0.30	0.10
**제6회**	**86.3**	**14.3**	**0.20**	**0.13**	**0.30**	**0.09**

(4) 전회 대비 직업평가 난이도와 변별도

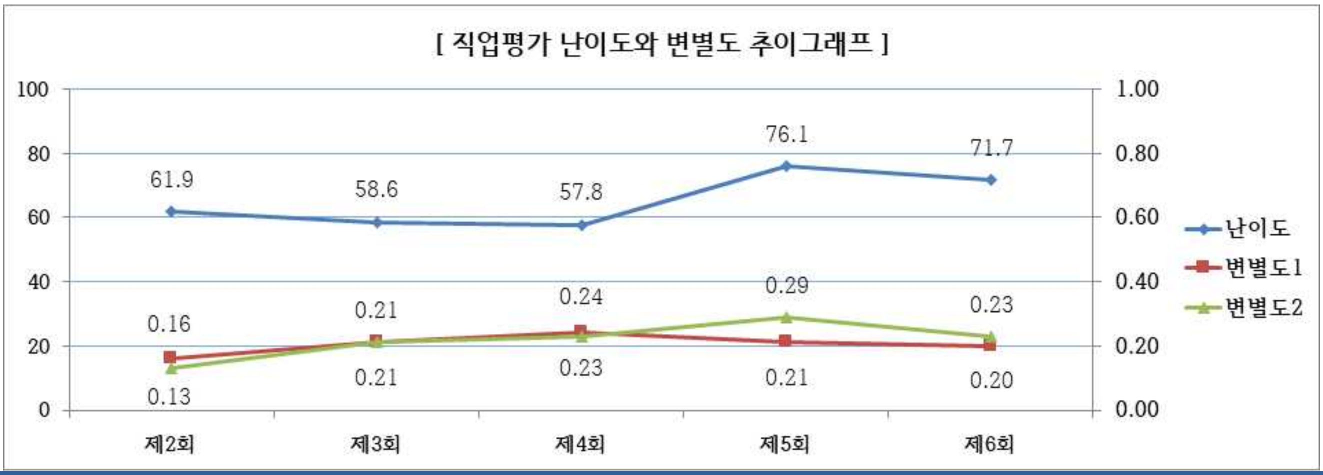

회차	난이도		변별도1		변별도2	
	평균	표준편차	평균	표준편차	평균	표준편차
제2회	61.9	29.8	0.16	0.10	0.13	0.10
제3회	58.6	25.0	0.21	0.11	0.21	0.08
제4회	57.8	24.1	0.24	0.18	0.23	0.14
제5회	76.1	22.9	0.21	0.15	0.29	0.10
**제6회**	**71.7**	**24.4**	**0.20**	**0.13**	**0.23**	**0.09**

(5) 전회 대비 직무개발과 배치 난이도와 변별도

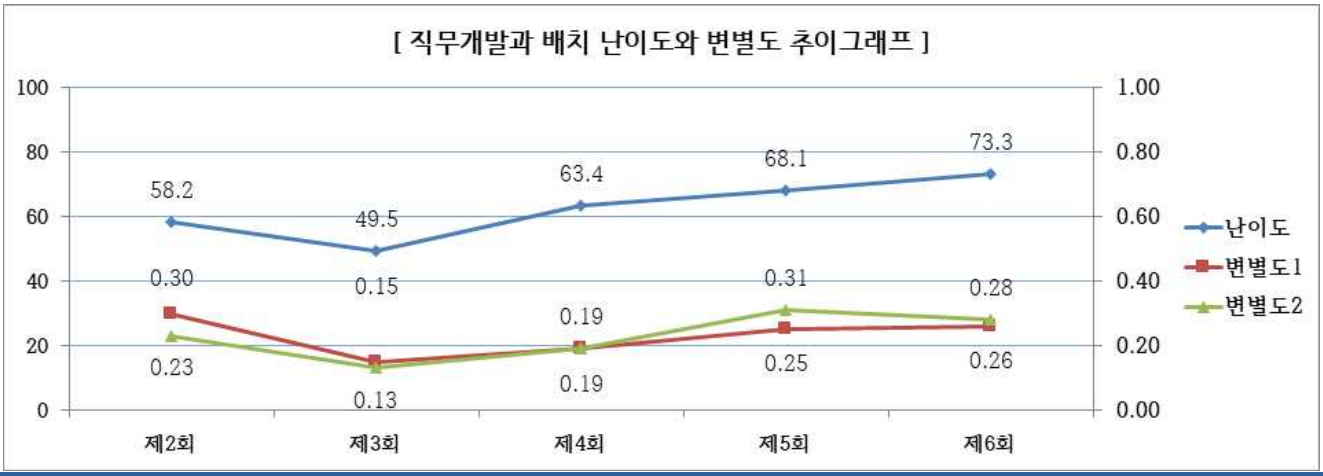

회차	난이도		변별도1		변별도2	
	평균	표준편차	평균	표준편차	평균	표준편차
제2회	58.2	21.9	0.30	0.14	0.23	0.11
제3회	49.5	24.2	0.15	0.15	0.13	0.14
제4회	63.4	23.7	0.19	0.16	0.19	0.14
제5회	68.1	24.2	0.25	0.11	0.31	0.10
**제6회**	**73.3**	**20.2**	**0.26**	**0.14**	**0.28**	**0.10**

(6) 전회 대비 재활행정 난이도와 변별도

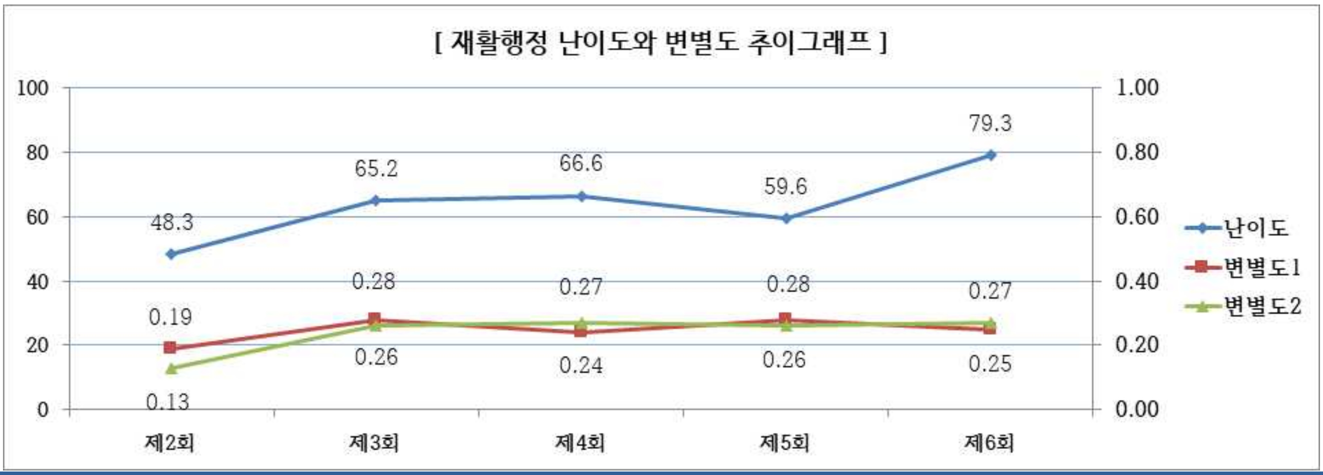

회차	난이도		변별도1		변별도2	
	평균	표준편차	평균	표준편차	평균	표준편차
제2회	48.3	24.7	0.19	0.19	0.13	0.14
제3회	65.2	16.7	0.28	0.11	0.26	0.08
제4회	66.6	26.2	0.24	0.16	0.27	0.12
제5회	59.6	15.9	0.28	0.19	0.26	0.14
**제6회**	**79.3**	**12.4**	**0.25**	**0.17**	**0.27**	**0.11**

(7) 전회 대비 재활정책 난이도와 변별도

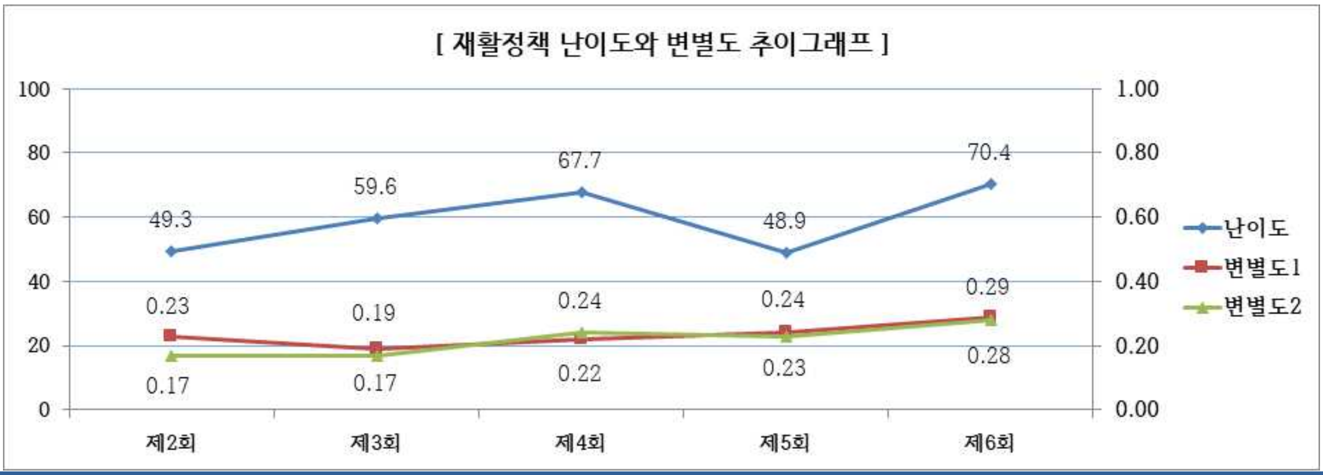

회차	난이도		변별도1		변별도2	
	평균	표준편차	평균	표준편차	평균	표준편차
제2회	49.3	23.5	0.23	0.12	0.17	0.10
제3회	59.6	27.0	0.19	0.13	0.17	0.11
제4회	67.7	27.6	0.22	0.17	0.24	0.11
제5회	48.9	24.8	0.24	0.12	0.23	0.12
**제6회**	**70.4**	**21.0**	**0.29**	**0.20**	**0.28**	**0.13**

해석

- 전회 대비 직업재활개론, 재활상담, 재활사례관리, 직무개발과 배치, 재활행정, 재활정책 과목의 난이도 지수는 각각 9.8, 3.7, 6.8, 5.2, 19.7, 21.5 증가하였고, 직업평가재활정책 과목의 난이도 지수는 4.4 감소함
- 재활상담, 직무개발과 배치, 재활정책 과목의 변별도 1 지수는 각각 0.02, 0.01, 0.05 증가하였으며, 직업재활개론, 직업평가, 재활행정 과목의 변별도 1 지수는 각각 0.02, 0.01, 0.03 감소함
- 재활사례관리 과목의 변별도 1 지수는 변화 없음
- 재활상담, 재활행정, 재활정책 과목의 변별도 2 지수는 0.08, 0.06, 0.05 증가하였으며, 직업재활개론, 직업평가, 직무개발과 배치 과목의 변별도 2 지수는 각각 0.02, 0.06, 0.03, 감소함
- 재활사례관리 과목의 변별도 2 지수는 변화 없음

나) 과목별 난이도와 변별도 분포도 및 비율분석

(1) 직업재활개론 난이도와 변별도 분포도 및 비율분석

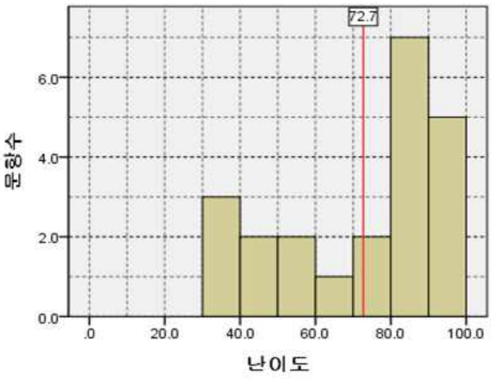

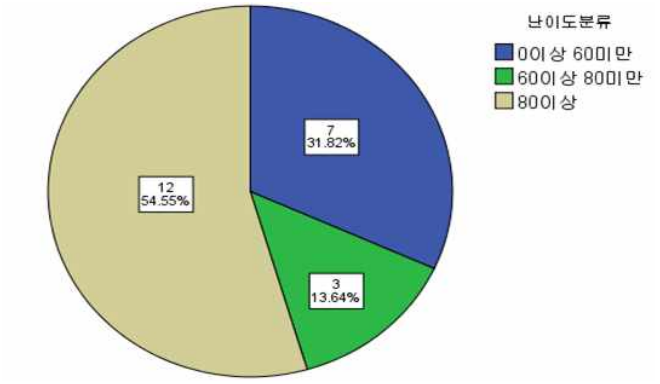

총점	난이도	표준편차
22	72.7	22.1

난이도	문항수	비율(%)
0~60미만	7	31.8
60~80미만	3	13.6
80~100	12	54.5
전체	22	100.0

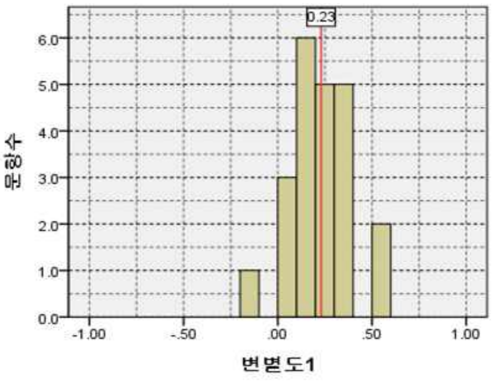

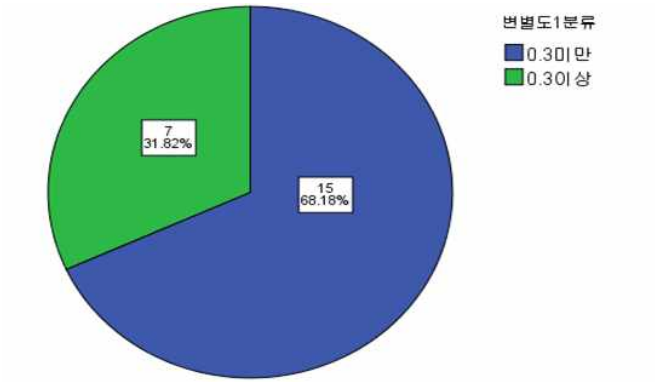

총점	변별도1	표준편차
22	.23	.16

변별도1	문항수	비율(%)
0.3미만	15	68.2
0.3이상	7	31.8
전체	22	100.0

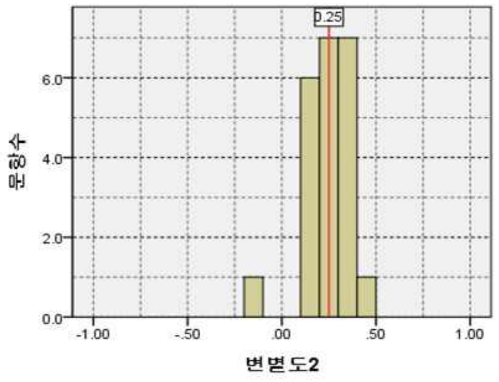

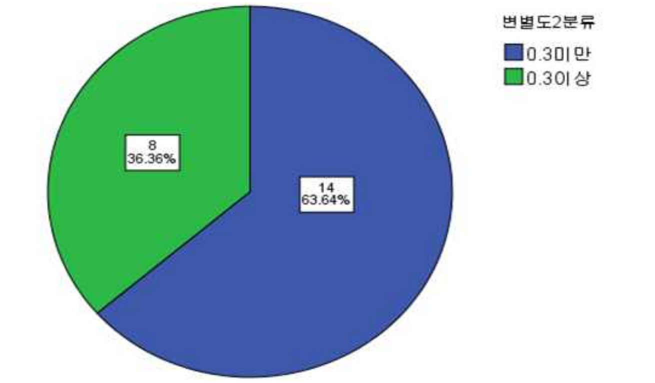

총점	변별도2	표준편차
22	.25	.12

변별도2	문항수	비율(%)
0.3미만	14	63.6
0.3이상	8	36.4
전체	22	100.0

해석

- 직업재활개론 과목에서 난이도 지수가 80 에서 100 사이인 문항이 전체 22 문항 중 12 문항이며, 60 이상 80 미만인 문항이 3 문항, 60 미만인 문항이 7 문항인 것으로 나타남
- 변별도 1 지수를 기준으로 분류하였을 때, 0.3 미만인 문항이 15 문항으로 0.3 이상인 문항이 7 문항인 것에 비해 더 많게 나타남
- 변별도 2 지수를 기준으로 분류하였을 때, 0.3 미만인 문항이 14 문항으로 0.3 이상인 문항이 8 문항인 것에 비해 더 많게 나타남

(2) 재활상담 난이도와 변별도 분포도 및 비율분석

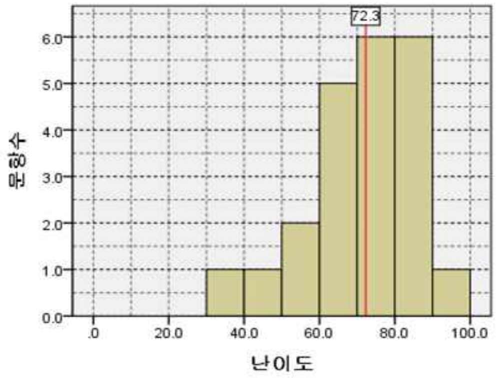

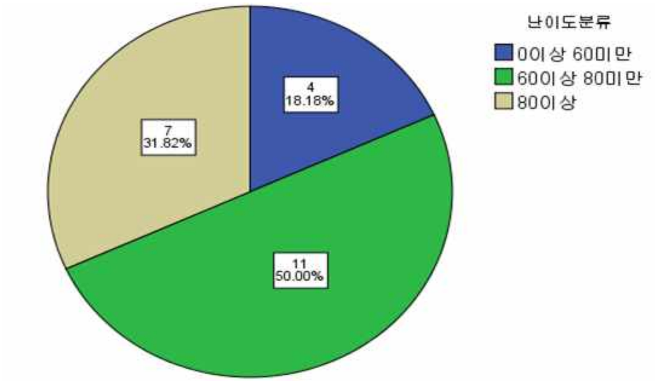

총점	난이도	표준편차
22	72.3	14.8

난이도	문항수	비율(%)
0~60미만	4	18.2
60~80미만	11	50.0
80~100	7	31.8
전체	22	100.0

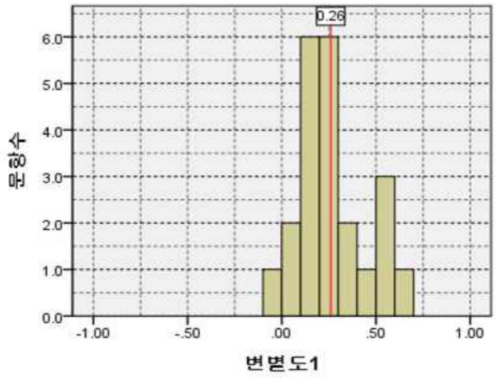

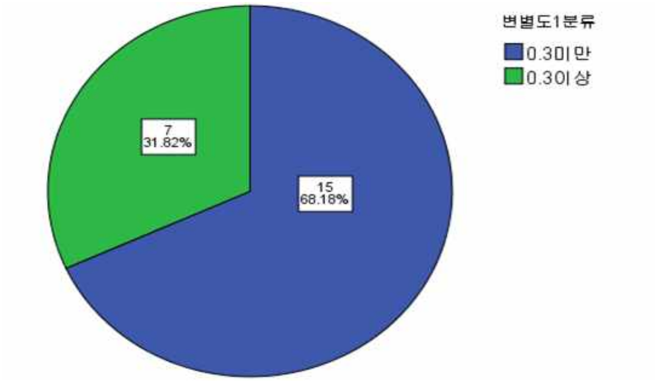

총점	변별도1	표준편차
22	.26	.18

변별도1	문항수	비율(%)
0.3미만	15	68.2
0.3이상	7	31.8
전체	22	100.0

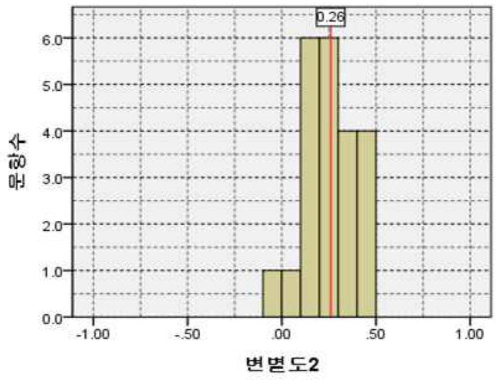

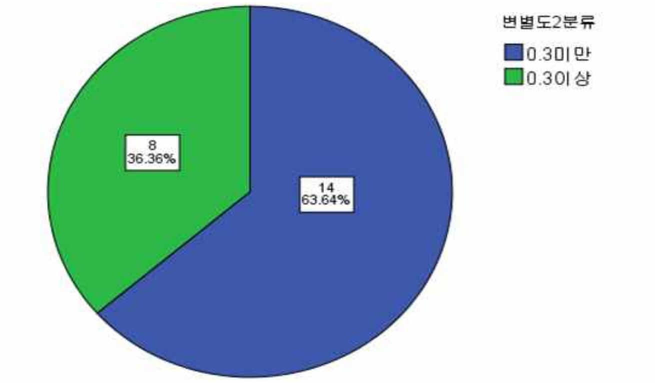

총점	변별도2	표준편차
22	.26	.14

변별도2	문항수	비율(%)
0.3미만	14	63.6
0.3이상	8	36.4
전체	22	100.0

해석

- 재활상담 과목에서 난이도 지수가 80에서 100 사이인 문항이 전체 22문항 중 7문항 이였으며, 60이상 80미만인 문항이 11문항, 60미만인 문항이 4문항인 것으로 나타남
- 변별도1 지수를 기준으로 분류하였을 때, 0.3미만인 문항이 15문항으로 0.3 이상인 문항이 7문항인 것에 비해 더 많게 나타남
- 변별도2 지수를 기준으로 분류하였을 때, 0.3미만인 문항이 14문항으로 0.3 이상인 문항이 8문항인 것에 비해 더 많게 나타남

(3) 재활사례관리 난이도와 변별도 분포도 및 비율분석

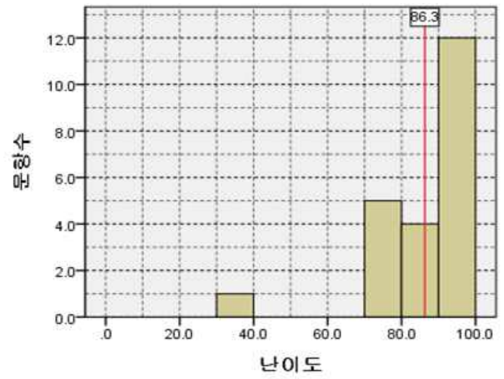

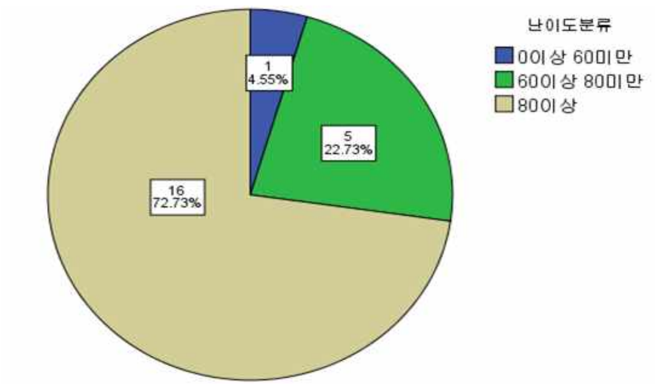

총점	난이도	표준편차
22	86.3	14.3

난이도	문항수	비율(%)
0~60미만	1	4.5
60~80미만	5	22.7
80~100	16	72.7
전체	22	100.0

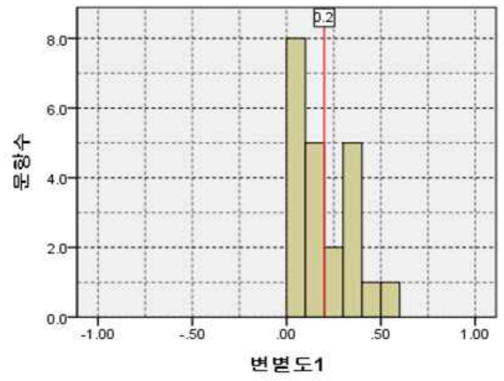

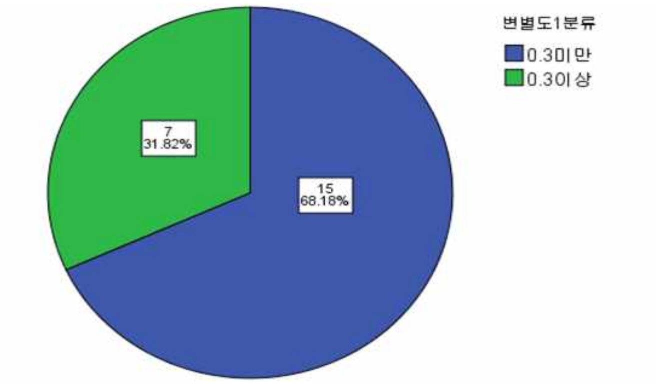

총점	변별도1	표준편차
22	.20	.13

변별도1	문항수	비율(%)
0.3미만	15	68.2
0.3이상	7	31.8
전체	22	100.0

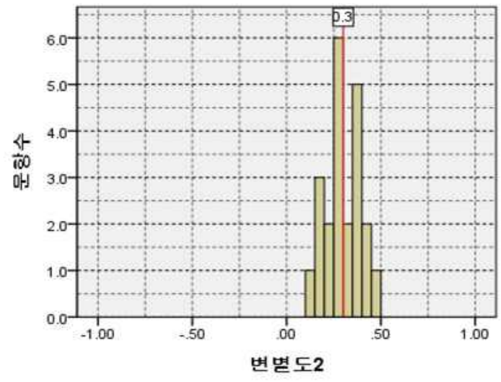

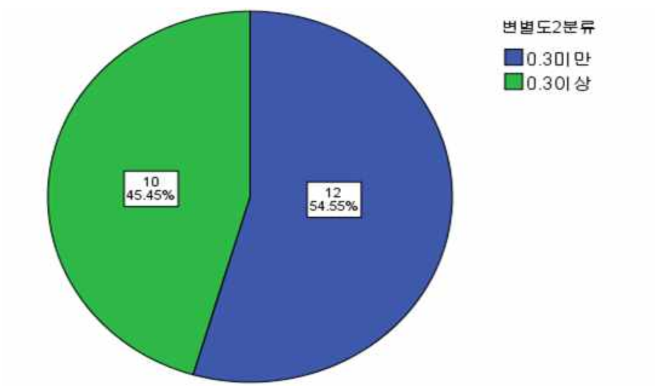

총점	변별도2	표준편차
22	.30	.09

변별도2	문항수	비율(%)
0.3미만	12	54.5
0.3이상	10	45.5
전체	22	100.0

해석

- 재활사례관리 과목에서 난이도 지수가 80 에서 100 사이인 문항이 전체 22 문항 중 16 문항으로 가장 많았으며, 다음으로 60 이상 80 미만인 문항이 5 문항, 60 미만인 문항이 1 문항 인 것으로 나타남
- 변별도 1 지수를 기준으로 분류하였을 때, 0.3 미만인 문항이 15 문항으로 0.3 이상인 문항이 7 문항인 것에 비해 더 많게 나타남
- 변별도 2 지수를 기준으로 분류하였을 때, 0.3 미만인 문항이 12 문항으로 0.3 이상인 문항이 10 문항인 것에 비해 더 많게 나타남

(4) 직업평가 난이도와 변별도 분포도 및 비율분석

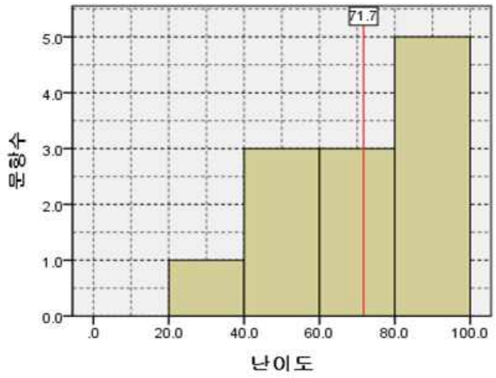

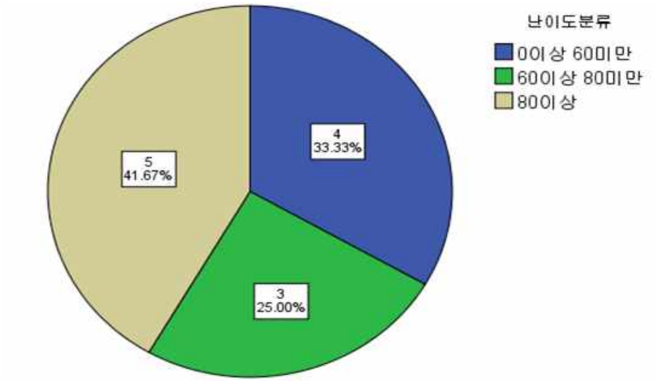

총점	난이도	표준편차	난이도	문항수	비율(%)
12	71.7	24.4	0~60미만	4	33.3
			60~80미만	3	25.0
			80~100	5	41.7
			전체	12	100.0

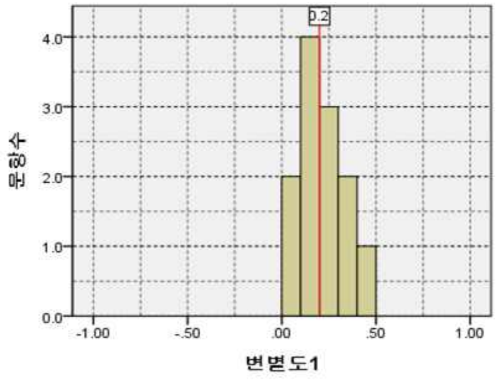

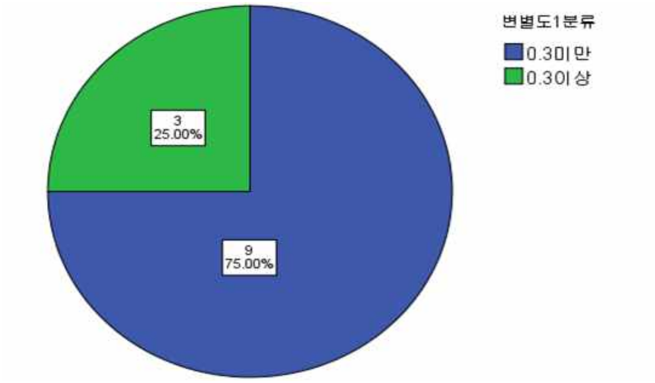

총점	변별도1	표준편차	변별도1	문항수	비율(%)
12	.20	.13	0.3미만	9	75.0
			0.3이상	3	25.0
			전체	12	100.0

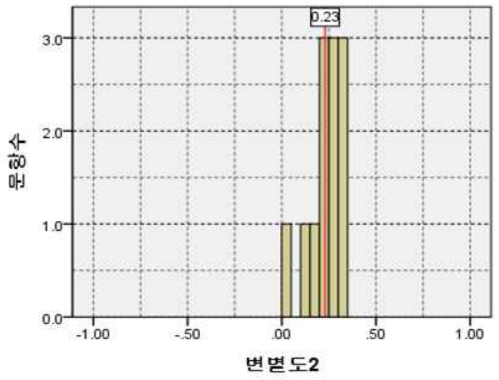

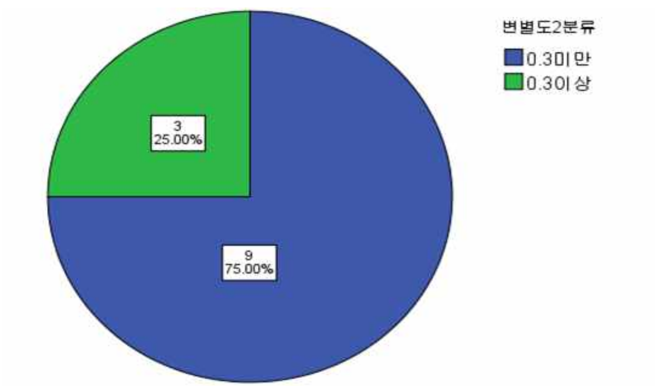

총점	변별도2	표준편차
12	.23	.09

변별도2	문항수	비율(%)
0.3미만	9	75.0
0.3이상	3	25.0
전체	12	100.0

해석

- 직업평가 과목에서 난이도 지수가 80 에서 100 사이인 문항이 전체 12 문항 중 5 문항으로 나타났으며, 다음으로 60 이상 80 미만인 문항이 3 문항, 60 미만인 문항이 4 문항 인 것으로 나타남
- 변별도 1 지수를 기준으로 분류하였을 때, 0.3 미만인 문항이 9 문항으로 0.3 이상인 문항이 3 문항인 것에 비해 더 많이 나타남
- 변별도 2 지수를 기준으로 분류하였을 때, 0.3 미만인 문항이 9 문항, 0.3 이상인 문항이 3 문항으로 같게 나타남

(5) 직무개발과 배치 난이도와 변별도 분포도 및 비율분석

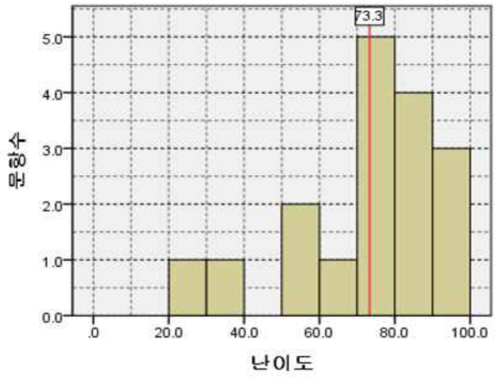

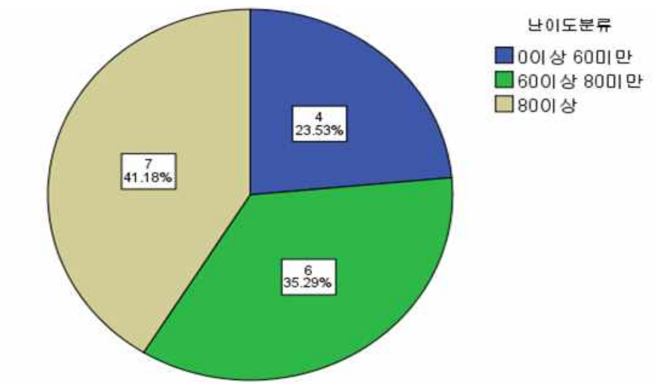

총점	난이도	표준편차
17	73.3	20.2

난이도	문항수	비율(%)
0~60미만	4	23.5
60~80미만	6	35.3
80~100	7	41.2
전체	17	100.0

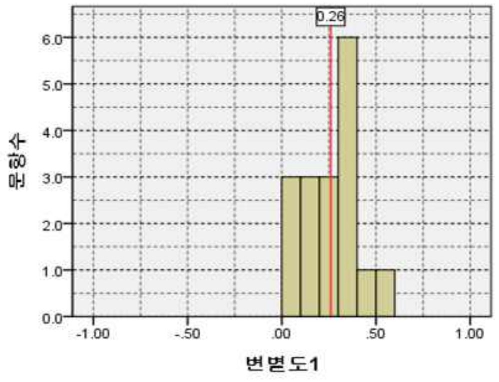

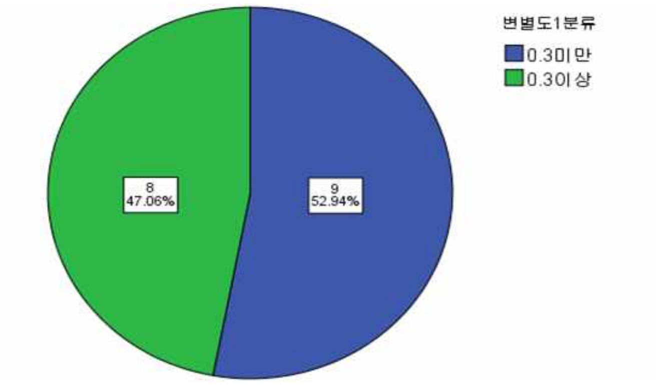

총점	변별도1	표준편차
17	.26	.14

변별도1	문항수	비율(%)
0.3미만	9	52.9
0.3이상	8	47.1
전체	17	100.0

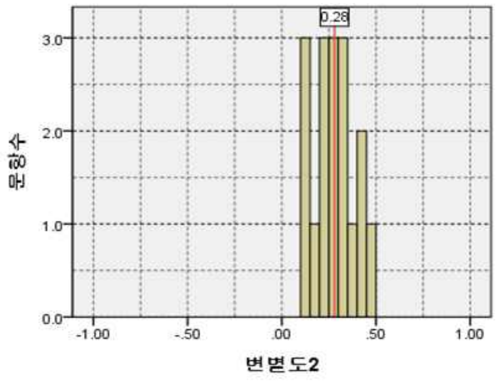

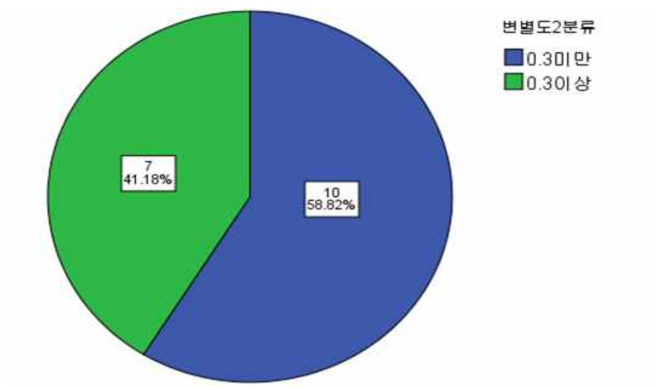

총점	변별도2	표준편차
17	.28	.10

변별도2	문항수	비율(%)
0.3미만	10	58.8
0.3이상	7	41.2
전체	17	100.0

해석

- 직무개발과 배치 과목에서 난이도 지수가 80 에서 100 사이인 문항이 전체 17 문항 중 7 문항으로 나타났으며, 60 이상 80 미만인 문항이 6 문항, 60 미만인 문항이 4 문항 인 것으로 나타남
- 변별도 1 지수를 기준으로 분류하였을 때, 0.3 미만인 문항이 9 문항으로 0.3 이상인 문항이 8 문항인 것에 비해 더 많게 나타남
- 변별도 2 지수를 기준으로 분류하였을 때, 0.3 미만인 문항이 10 문항으로 0.3 이상인 문항이 7 문항인 것에 비해 더 많게 나타남

(6) 재활행정 난이도와 변별도 분포도 및 비율분석

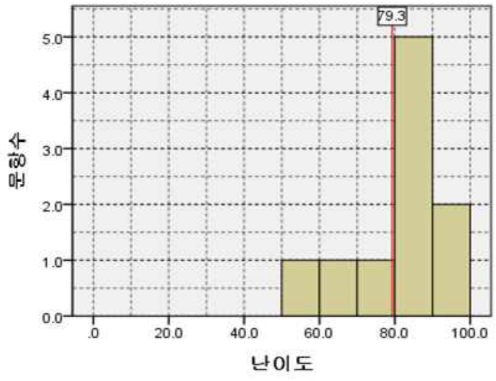

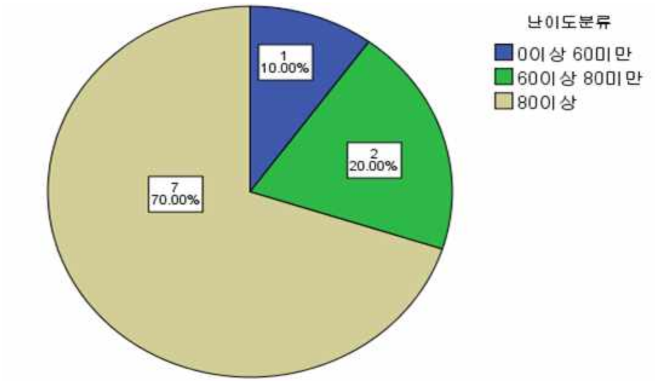

총점	난이도	표준편차
10	79.3	12.4

난이도	문항수	비율(%)
0~60미만	1	10.0
60~80미만	2	20.0
80~100	7	70.0
전체	10	100.0

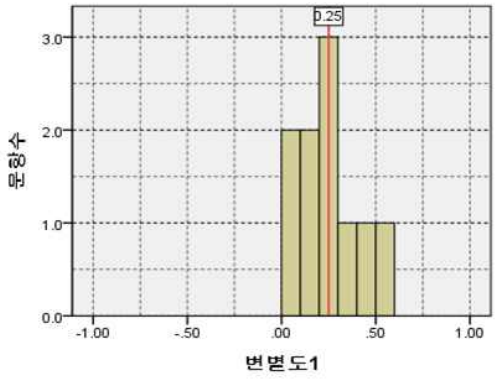

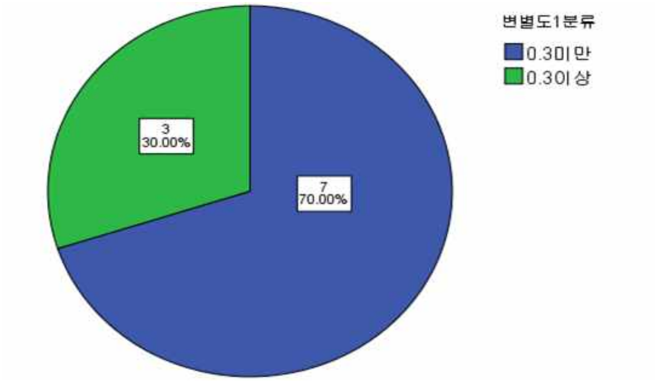

총점	변별도1	표준편차
10	.25	.17

변별도1	문항수	비율(%)
0.3미만	7	70.0
0.3이상	3	30.0
전체	10	100.0

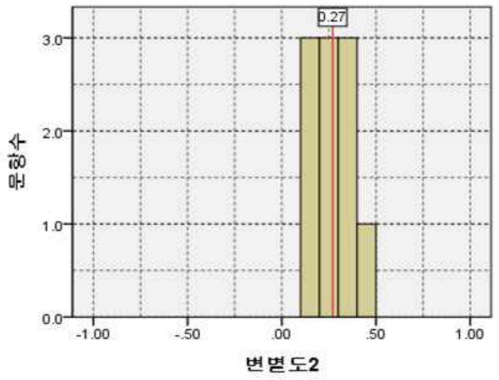

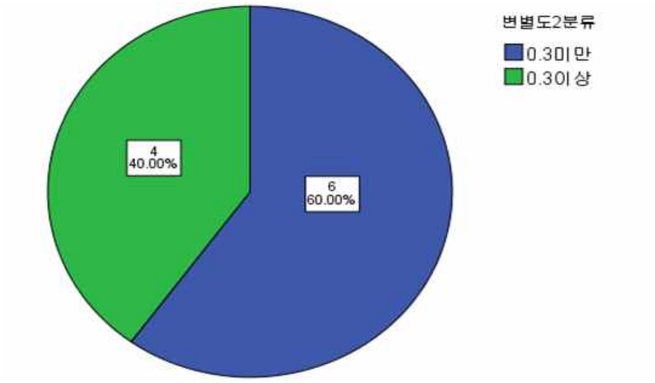

총점	변별도2	표준편차
10	.27	.11

변별도2	문항수	비율(%)
0.3미만	6	60.0
0.3이상	4	40.0
전체	10	100.0

해석

- 재활행정 과목에서 난이도 지수가 80 에서 100 사이인 문항이 전체 10 문항 중 7 문항, 60 이상 80 미만인 문항이 2 문항, 60 미만인 문항이 1 문항 인 것으로 나타남
- 변별도 1 지수를 기준으로 분류하였을 때, 0.3 미만인 문항이 7 문항으로 0.3 이상인 문항이 3 문항인 것에 비해 더 많게 나타남
- 변별도 2 지수를 기준으로 분류하였을 때, 0.3 미만인 문항이 6 문항으로 0.3 이상인 문항이 4 문항인 것에 비해 더 많게 나타남

(7) 재활정책 난이도와 변별도 분포도 및 비율분석

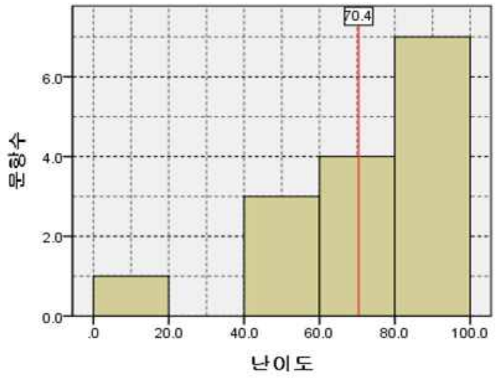

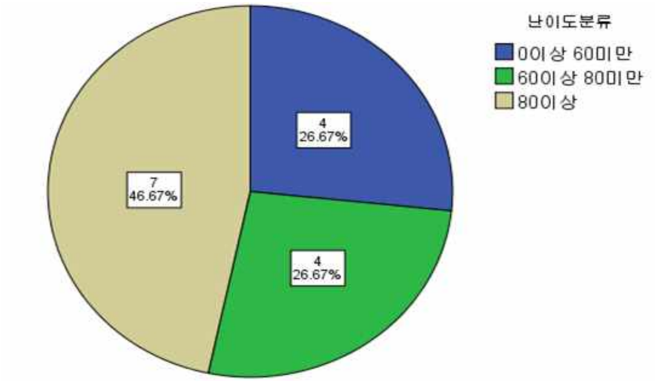

총점	난이도	표준편차	난이도	문항수	비율(%)
15	70.4	21.0	0~60미만	4	26.7
			60~80미만	4	26.7
			80~100	7	46.7
			전체	15	100.0

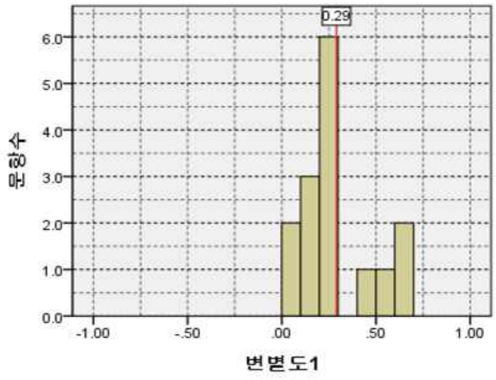

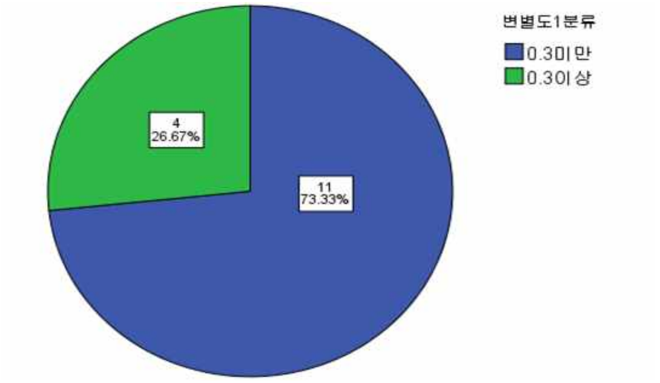

총점	변별도1	표준편차	변별도1	문항수	비율(%)
15	.29	.20	0.3미만	11	73.3
			0.3이상	4	26.7
			전체	15	100.0

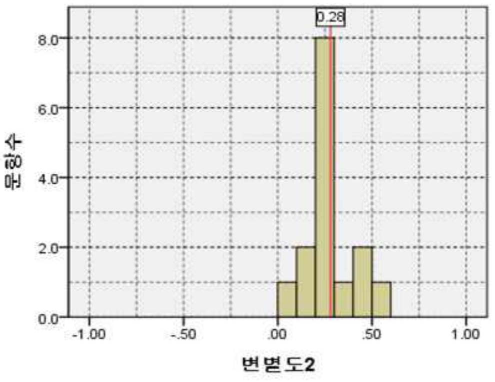

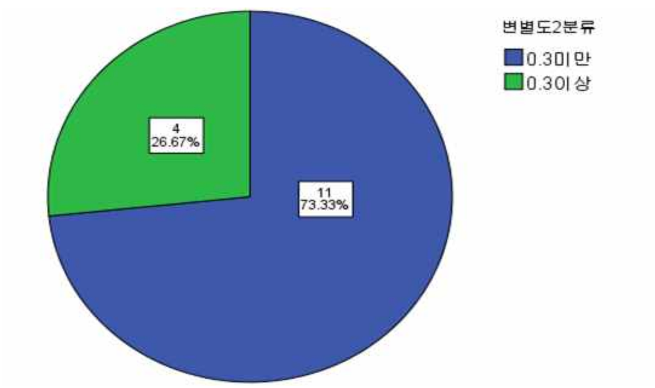

총점	변별도2	표준편차
15	.28	.13

변별도2	문항수	비율(%)
0.3미만	11	73.3
0.3이상	4	26.7
전체	15	100.0

해석

- 재활정책 과목에서 난이도 지수가 80 에서 100 사이인 문항이 전체 15 문항 중 4 문항 이였으며, 60 이상 80 미만인 문항이 4 문항, 60 미만인 문항이 4 문항인 것으로 나타남
- 변별도 1 지수를 기준으로 분류하였을 때, 0.3 미만인 문항이 11 문항으로 0.3 이상인 문항이 4 문항인 것에 비해 더 많게 나타남
- 변별도 2 지수를 기준으로 분류하였을 때, 0.3 미만인 문항이 11 문항으로 0.3 이상인 문항이 4 문항인 것에 비해 더 많게 나타남

3) 지식수준별 난이도와 변별도

가) 전회 대비 지식수준별 난이도와 변별도

(1) 전회 대비 암기형 난이도와 변별도

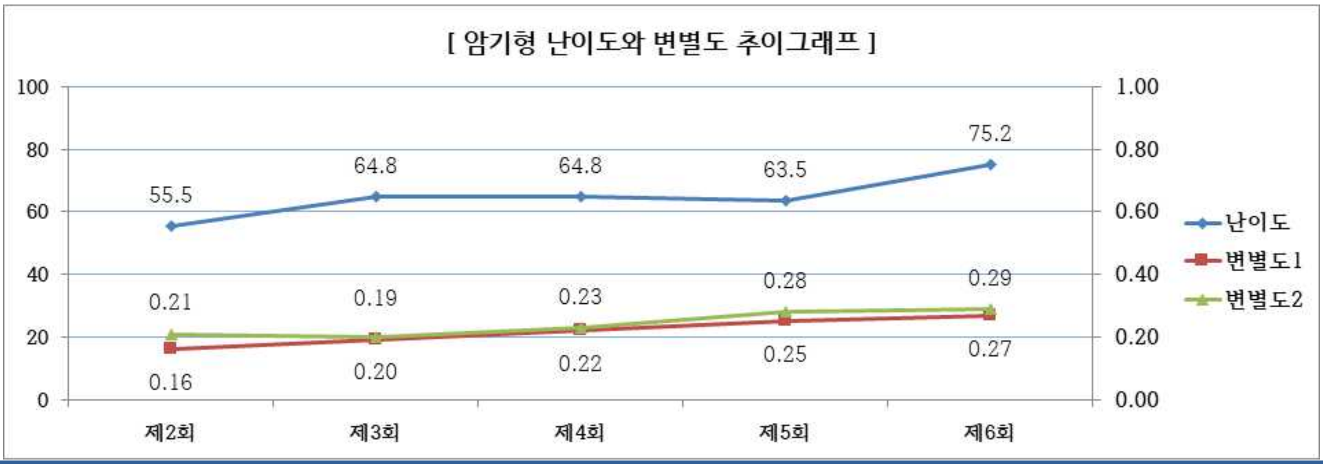

회차	난이도		변별도1		변별도2	
	평균	표준편차	평균	표준편차	평균	표준편차
제2회	55.5	23.8	0.16	0.11	0.21	0.14
제3회	64.8	22.8	0.19	0.11	0.20	0.13
제4회	64.8	24.8	0.22	0.13	0.23	0.11
제5회	63.5	25.1	0.25	0.14	0.28	0.12
**제6회**	**75.2**	**20.0**	**0.27**	**0.17**	**0.29**	**0.11**

(2) 전회 대비 해석형 난이도와 변별도

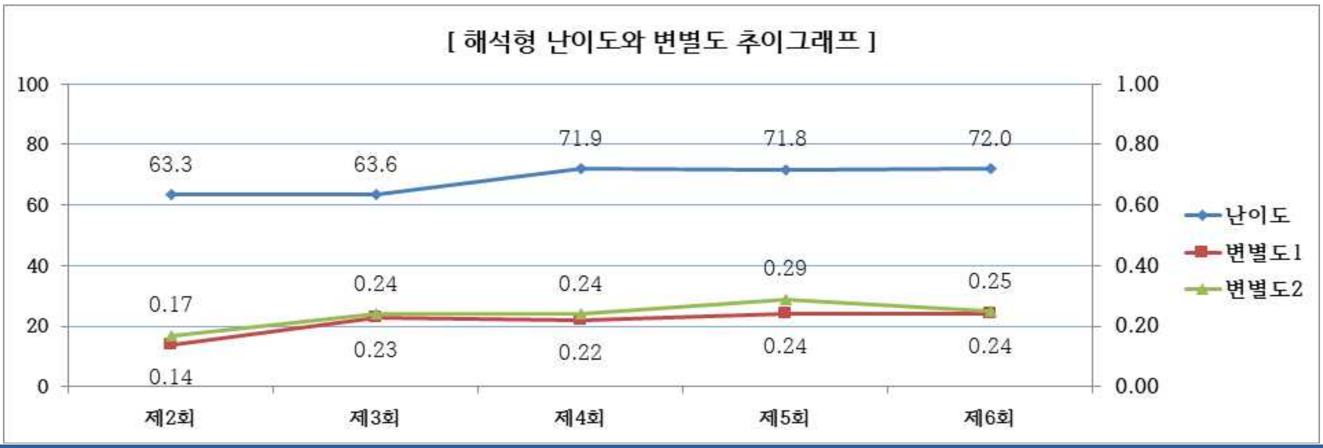

회차	난이도		변별도1		변별도2	
	평균	표준편차	평균	표준편차	평균	표준편차
제2회	63.3	26.2	0.14	0.12	0.17	0.13
제3회	63.6	24.6	0.23	0.10	0.24	0.14
제4회	71.9	22.8	0.22	0.16	0.24	0.11
제5회	71.8	21.4	0.24	0.14	0.29	0.10
**제6회**	72.0	18.7	0.24	0.15	0.25	0.12

(3) 전회 대비 해결형 난이도와 변별도

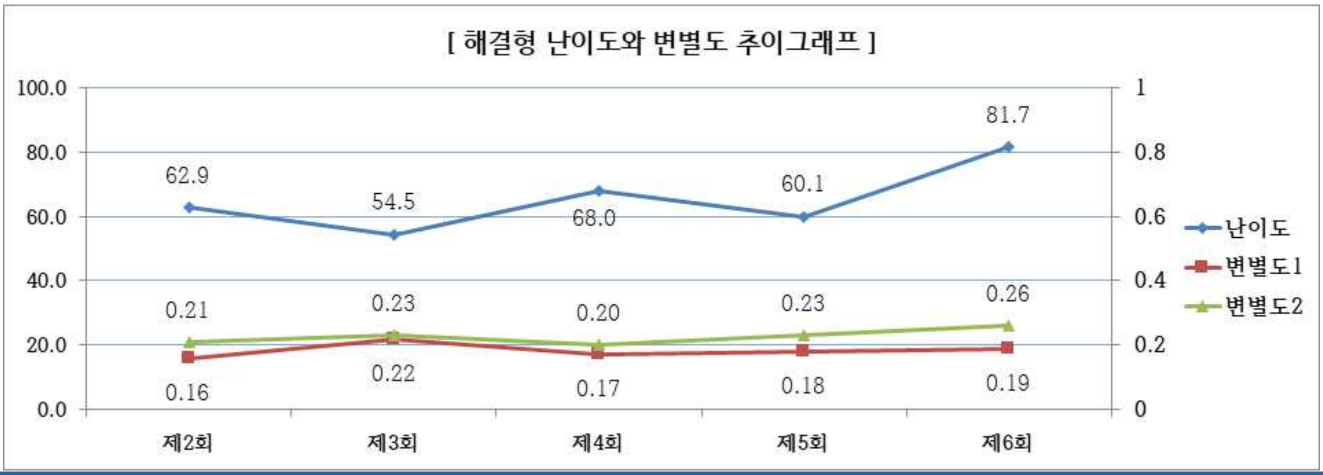

회차	난이도		변별도1		변별도2	
	평균	표준편차	평균	표준편차	평균	표준편차
제2회	62.9	21.3	0.16	0.10	0.21	0.11
제3회	54.5	22.7	0.22	0.14	0.23	0.17
제4회	68.0	27.8	0.17	0.15	0.20	0.13
제5회	60.1	28.4	0.18	0.15	0.23	0.10
**제6회**	**81.7**	**17.1**	**0.19**	**0.14**	**0.26**	**0.11**

해석

- 전회 대비 암기형, 해석형, 해결형 문항의 난이도 지수는 각각 11.7, 0.2, 21.6 증가함
- 암기형 문항의 변별도 1 지수는 변화 없었으며, 변별도 2 지수는 0.04 감소함
- 해석형 문항의 변별도 1 지수와 변별도 2 지수는 각각 0.02, 0.05 증가함
- 해결형 문항의 변별도 1 지수와 변별도 2 지수는 각각 0.01, 0.03 증가함

나) 지식수준별 난이도와 변별도 분포도 및 비율분석

(1) 암기형 난이도와 변별도 분포도 및 비율분석

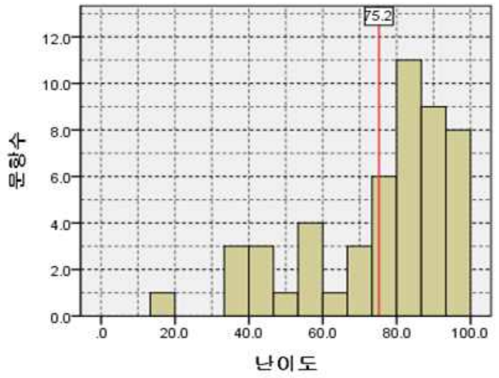

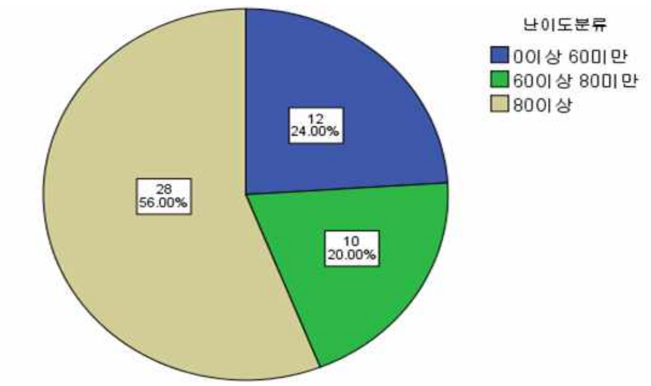

총점	난이도	표준편차	난이도	문항수	비율(%)
50	75.2	20.0	0~60미만	12	24.0
			60~80미만	10	20.0
			80~100	28	56.0
			전체	50	100.0

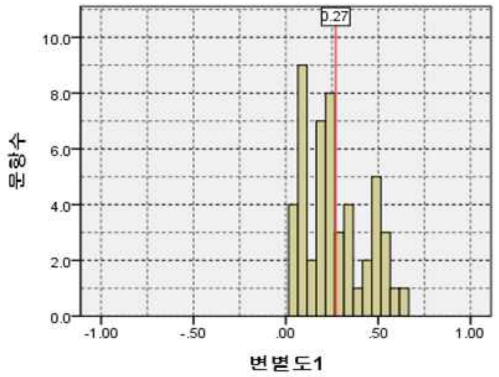

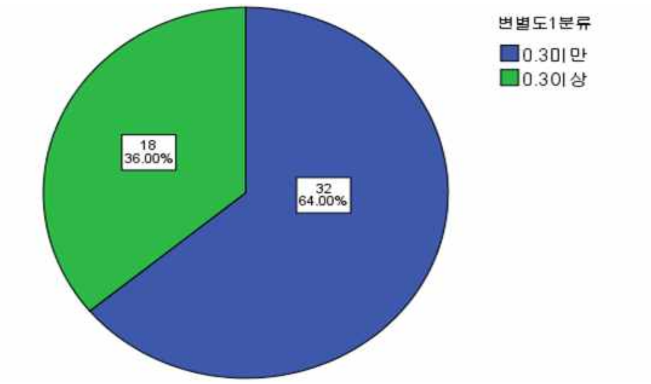

총점	변별도1	표준편차	변별도1	문항수	비율(%)
50	.27	.17	0.3미만	32	64.0
			0.3이상	18	36.0
			전체	50	100.0

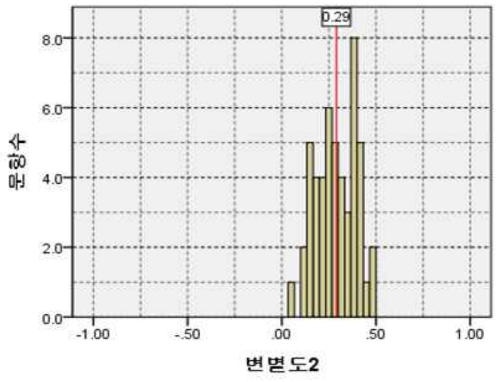

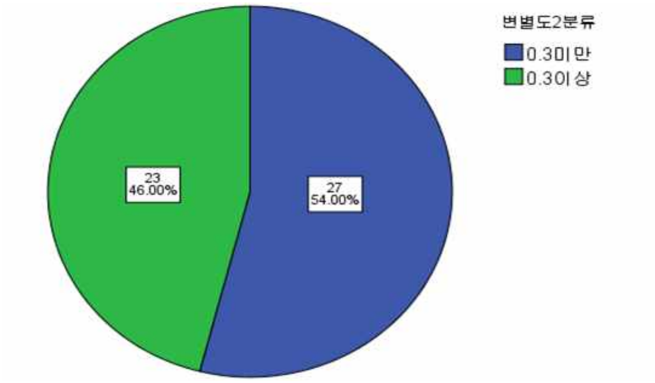

총점	변별도2	표준편차	변별도2	문항수	비율(%)
50	.29	.11	0.3미만	27	54.0
			0.3이상	23	46.0
			전체	50	100.0

해석

- 난이도 지수가 80에서 100사이인 문항이 전체 50문항 중 28문항 이였으며, 60이상 80미만인 문항이 10문항, 60미만인 문항이 12문항인 것으로 나타남
- 변별도1 지수를 기준으로 분류하였을 때, 0.3 미만인 문항이 32문항으로 0.3 이상인 문항이 18문항인 것에 비해 더 많게 나타남
- 변별도2 지수를 기준으로 분류하였을 때, 0.3 미만인 문항이 27문항으로 0.3 이상인 문항이 23문항인 것에 비해 더 많게 나타남

(2) 해석형 난이도와 변별도 분포도 및 비율분석

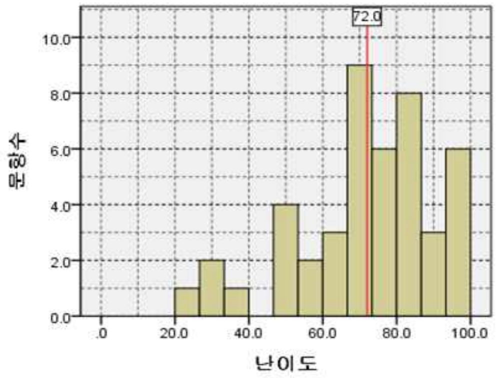

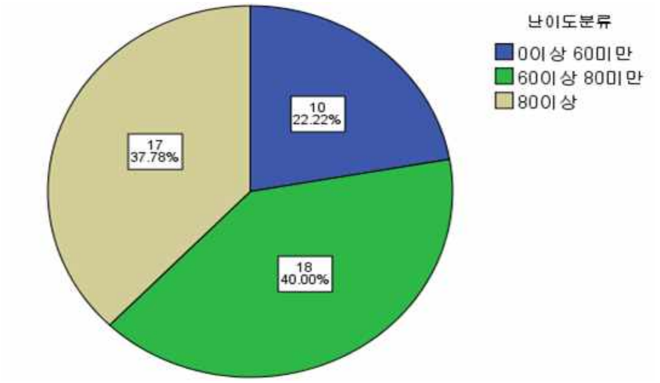

총점	난이도	표준편차
45	72.0	18.7

난이도	문항수	비율(%)
0~60미만	10	22.2
60~80미만	18	40.0
80~100	17	37.8
전체	45	100.0

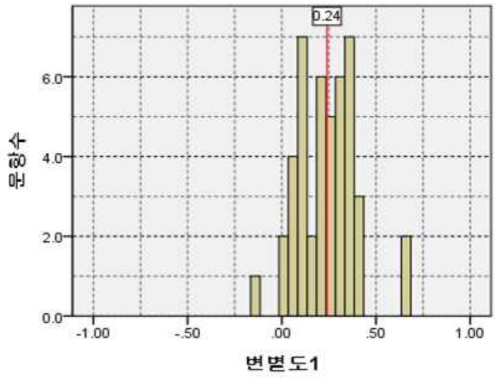

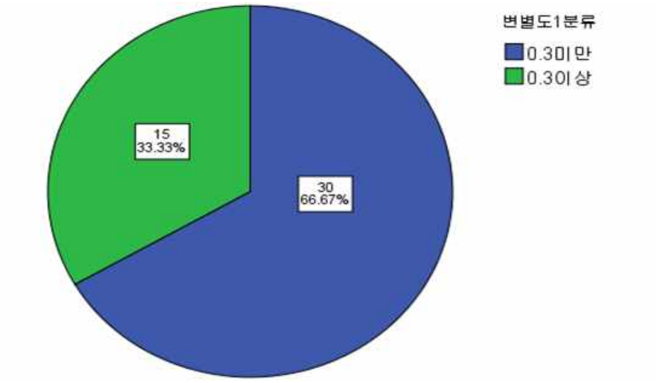

총점	변별도1	표준편차
45	.24	.15

변별도1	문항수	비율(%)
0.3미만	30	66.7
0.3이상	15	33.3
전체	45	100.0

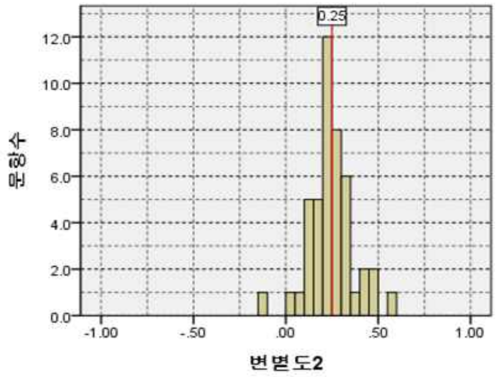

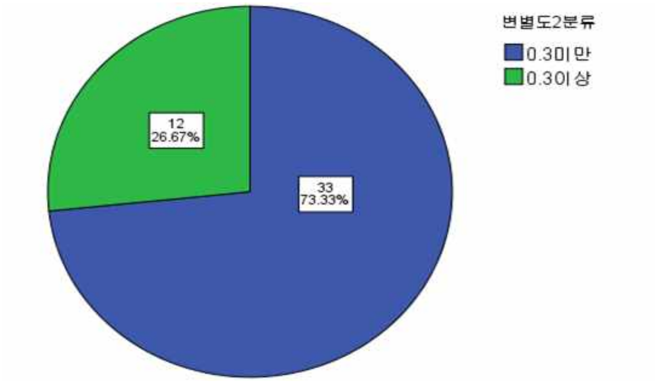

총점	변별도2	표준편차	변별도2	문항수	비율(%)
45	.25	.12	0.3미만	33	73.3
			0.3이상	12	26.7
			전체	45	100.0

해석

- 난이도 지수가 80에서 100사이인 문항이 전체 45문항 중 17문항으로 나타났으며, 다음으로 60이상 80미만인 문항이 18문항, 60미만인 문항이 10문항인 것으로 나타남
- 변별도1 지수를 기준으로 분류하였을 때, 0.3 미만인 문항이 30문항으로 0.3 이상인 문항이 15문항인 것에 비해 더 많이 나타남
- 변별도2 지수를 기준으로 분류하였을 때, 0.3 미만인 문항이 33문항으로 0.3 이상인 문항이 12문항인 것에 비해 더 많게 나타남

(3) 해결형 난이도와 변별도 분포도 및 비율분석

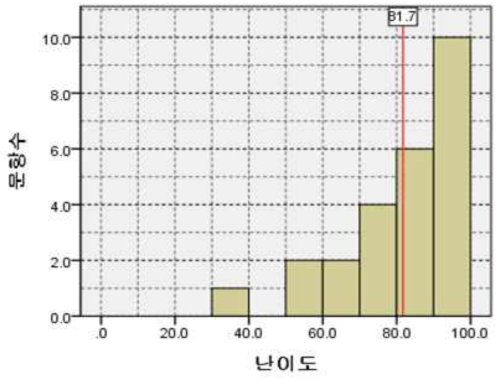

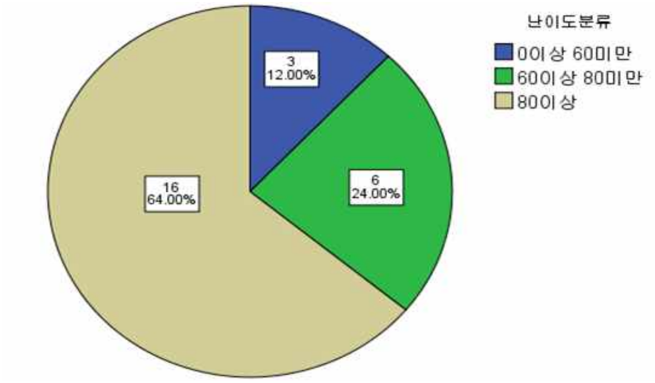

총점	난이도	표준편차
25	81.7	17.1

난이도	문항수	비율(%)
0~60미만	3	12.0
60~80미만	6	24.0
80~100	16	64.0
전체	25	100.0

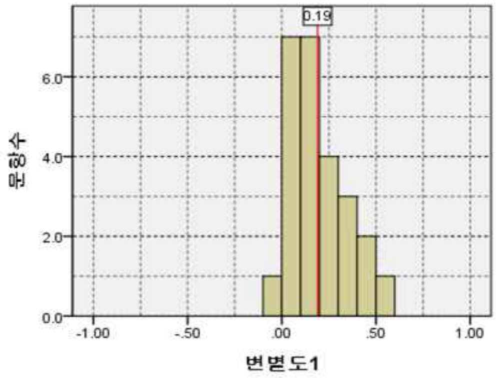

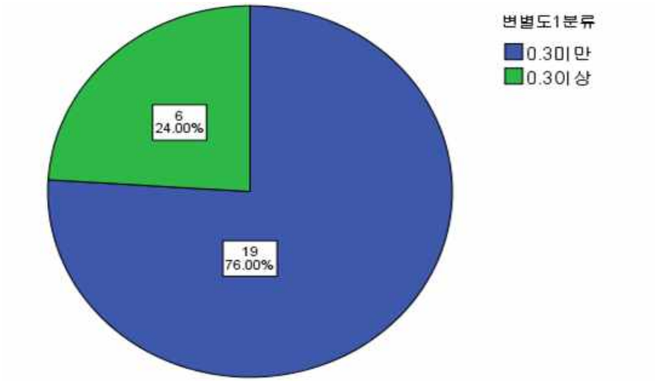

총점	변별도1	표준편차
25	.19	.14

변별도1	문항수	비율(%)
0.3미만	19	76.0
0.3이상	6	24.0
전체	25	100.0

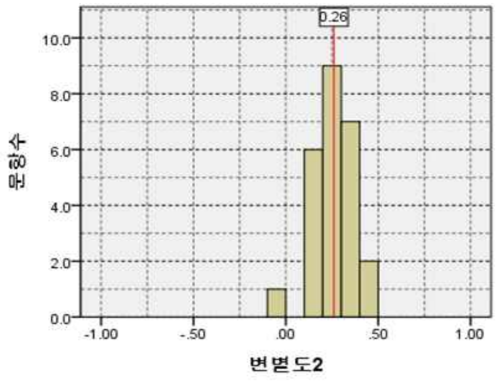

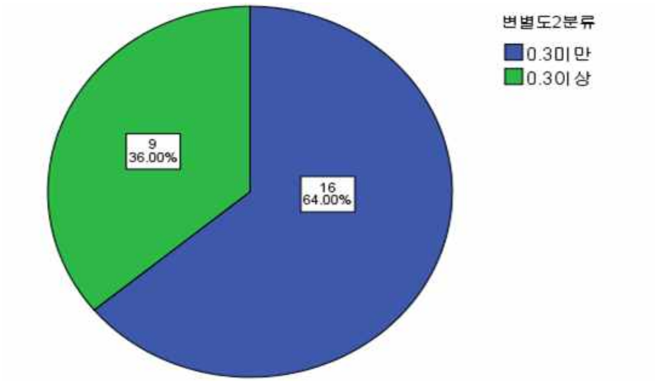

총점	변별도2	표준편차	변별도2	문항수	비율(%)
25	.26	.11	0.3미만	16	64.0
			0.3이상	9	36.0
			전체	25	100.0

해석

- 난이도 지수가 80에서 100사이인 문항이 전체 25문항 중 16문항으로 나타났으며, 다음으로 60이상 80미만인 문항이 6문항, 60미만인 문항이 3문항인 것으로 나타남
- 변별도1 지수를 기준으로 분류하였을 때, 0.3 미만인 문항이 19문항으로 0.3 이상인 문항이 6문항인 것에 비해 더 많게 나타남
- 변별도2 지수를 기준으로 분류하였을 때, 0.3 미만인 문항이 16문항으로 0.3 이상인 문항이 9문항인 것에 비해 더 많게 나타남

3. 난이도와 변별도 간 산포도

1) 전체 난이도와 변별도 간 산포도

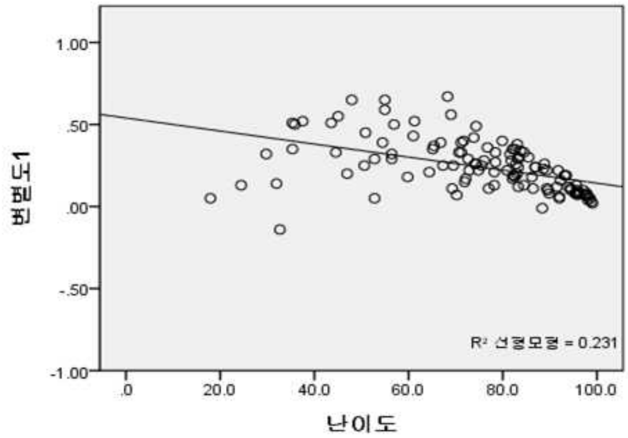

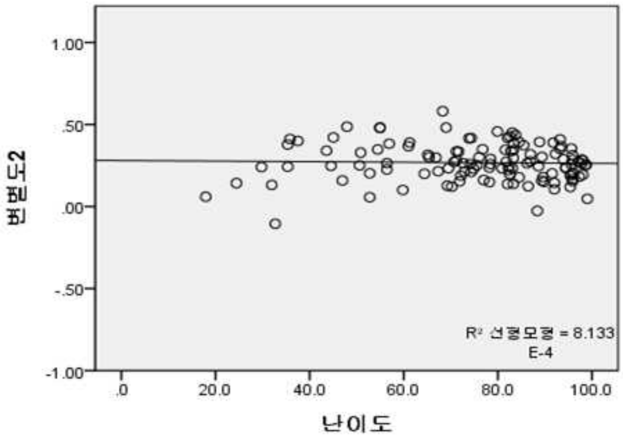

해석

- 전체 문항을 대상으로 난이도와 변별도1 지수 간 상관은 -.481*로 문항 난이도 지수가 높을수록 변별력이 낮아지는 것으로 나타남
- 난이도와 변별도2 지수 간 상관은 -.029로 문항 난이도와 변별력 간 관련성은 없는 것으로 나타남

2) 과목별 난이도와 변별도 간 산포도

가) 직업재활개론 난이도와 변별도 간 산포도

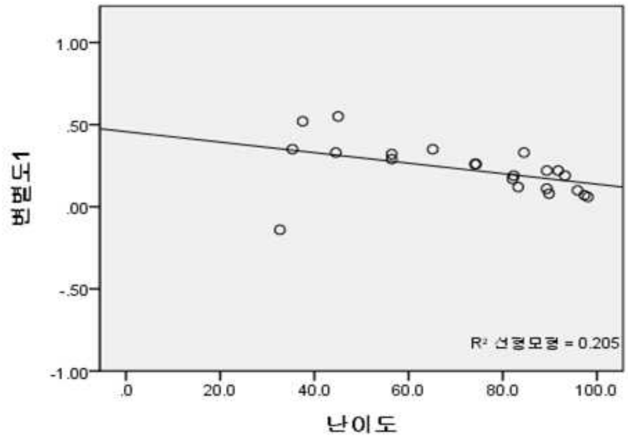

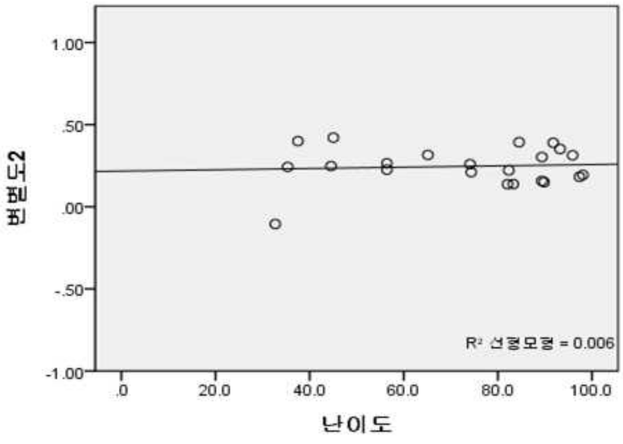

해석
- 직업재활개론 과목 문항을 대상으로 난이도와 변별도 1 지수 간 상관은 -.452*로 문항 난이도 지수가 높을수록 변별력이 낮아지는 것으로 나타남<br>- 난이도와 변별도 2 지수 간 상관은 .074 로 문항 난이도와 변별력 간 관련성은 없는 것으로 나타남

나) 재활상담난이도와 변별도 간 산포도

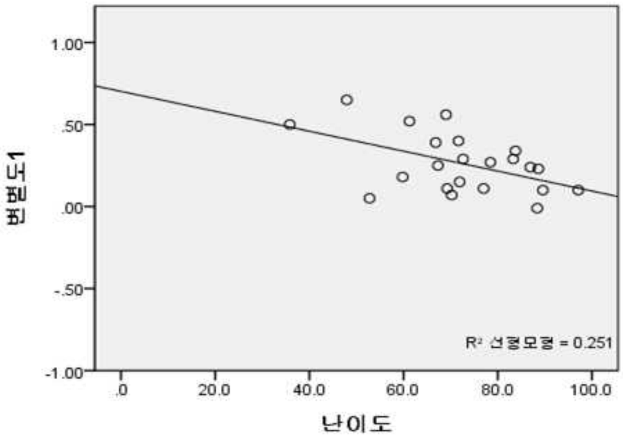

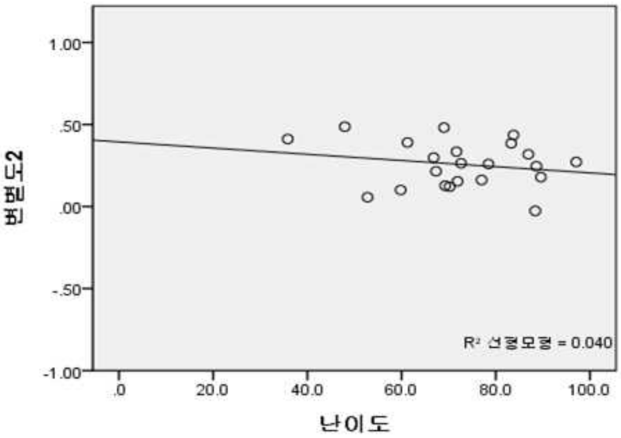

해석
- 재활상담 과목 문항을 대상으로 난이도와 변별도 1 지수 간 상관은 -.501*로 문항 난이도 지수가 높을수록 변별력이 낮아지는 것으로 나타남<br>- 난이도와 변별도 2 지수 간 상관은 -.200 로 문항 난이도와 변별력 간 관련성이 낮은 것으로 나타남

다) 재활사례관리 난이도와 변별도 간 산포도

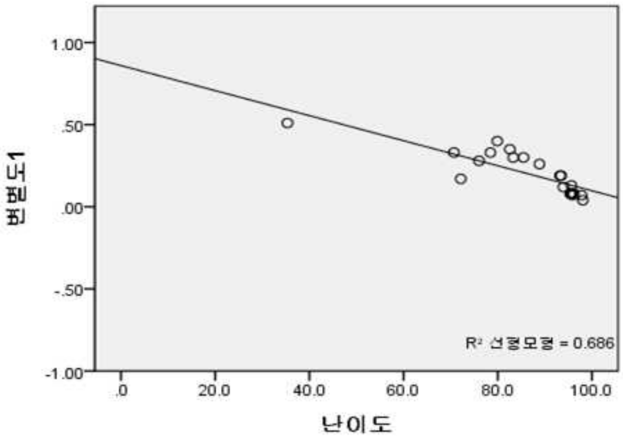

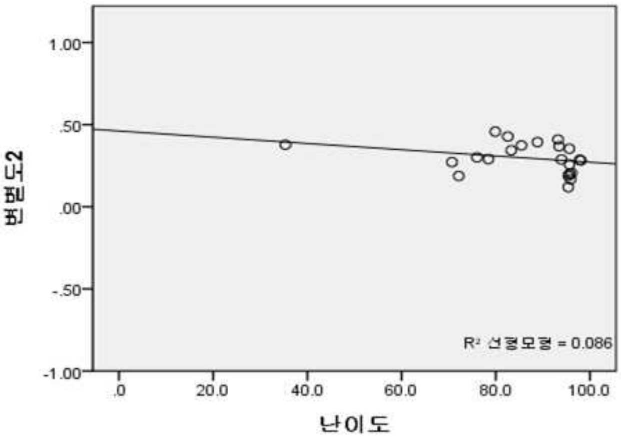

해석

- 재활사례관리 과목 문항을 대상으로 난이도와 변별도 1 지수 간 상관은 -.828*로 문항 난이도 지수가 높을수록 변별력이 낮아지는 것으로 나타남
- 난이도와 변별도 2 지수 간 상관은 -.294 로 문항 난이도와 변별력 간 관련성이 낮은 것으로 나타남

라) 직업평가 난이도와 변별도 간 산포도

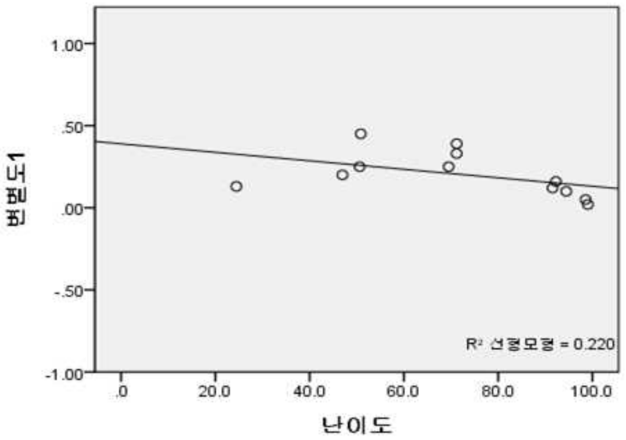

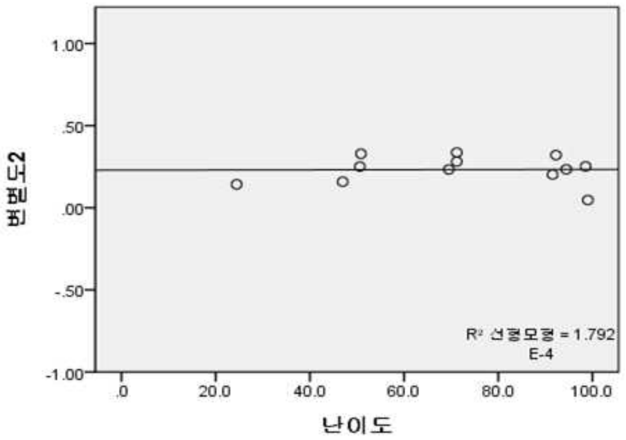

해석

- 직업평가 과목 문항을 대상으로 난이도와 변별도 1 지수 간 상관은 -.469 로 문항 난이도와 변별력 간 관련성이 낮은 것으로 나타남
- 난이도와 변별도 2 지수 간 상관은 .013 으로 문항 난이도와 변별력 간 관련성이 없는 것으로 나타남

마) 직무개발과 배치 난이도와 변별도 간 산포도

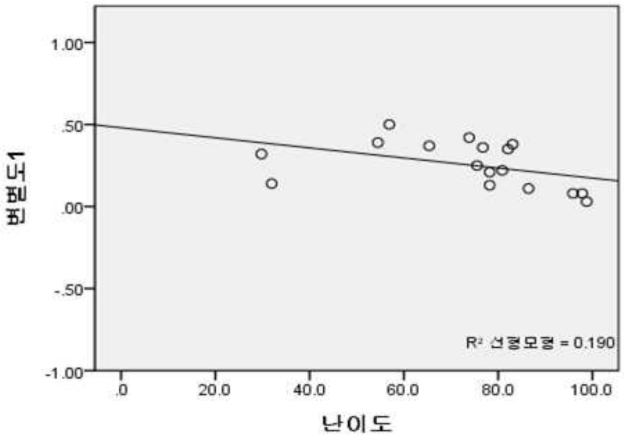

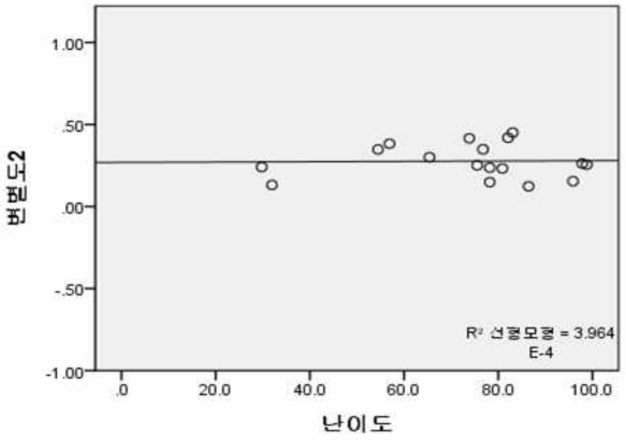

해석

- 직무개발과 배치 과목 문항을 대상으로 난이도와 변별도1 지수 간 상관은 -.436로 문항 난이도와 변별력 간 관련성이 낮은 것으로 나타남
- 난이도와 변별도2 지수 간 상관은 .020로 문항 난이도와 변별력 간 관련성이 없는 것으로 나타남

바) 재활행정 난이도와 변별도 간 산포도

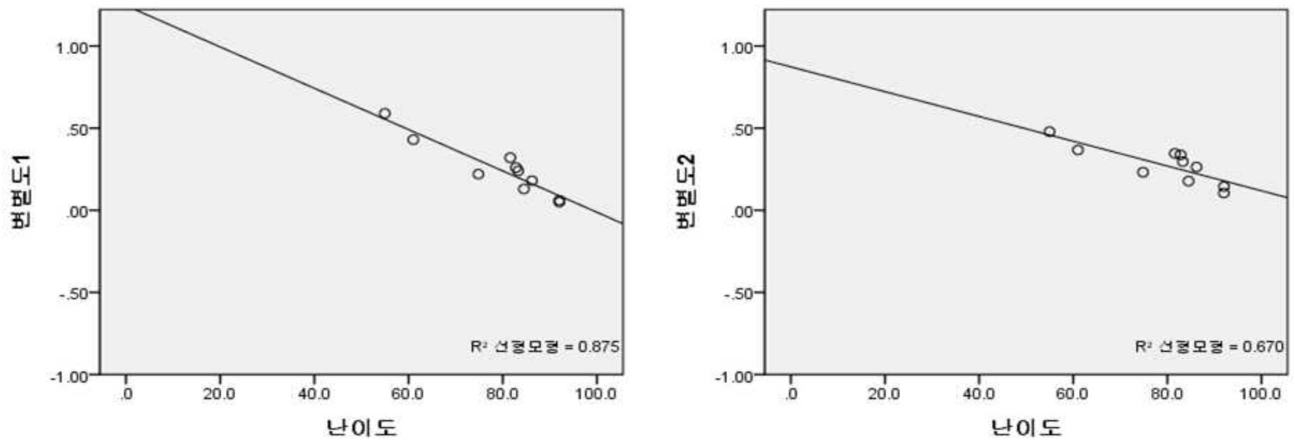

해석

- 재활행정 과목 문항을 대상으로 난이도와 변별도1 지수 간 상관은 -.935*으로 문항 난이도 지수가 높을수록 변별력이 낮아지는 것으로 나타남
- 난이도와 변별도2 지수 간 상관은 -.818*로 문항 난이도 지수가 높을수록 변별력이 낮아지는 것으로 나타남

사) 재활정책 난이도와 변별도 간 산포도

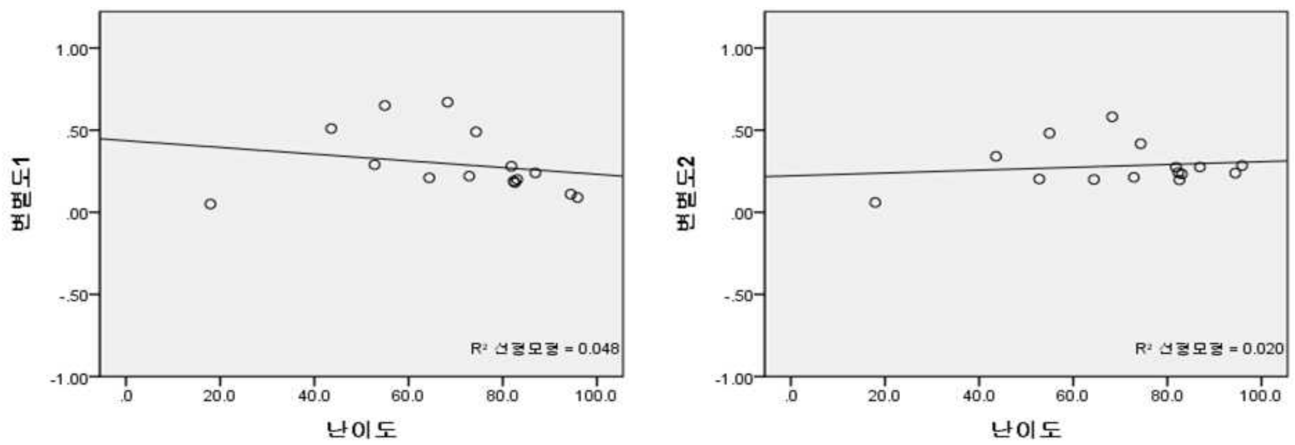

해석

- 재활정책 과목 문항을 대상으로 난이도와 변별도1 지수 간 상관은 -.219로 문항 난이도와 변별력 간 관련성이 낮은 것으로 나타남
- 난이도와 변별도2 지수 간 상관은 .140로 문항 난이도와 변별력 간 관련성이 낮은 것으로 나타남

4. 신뢰도 분석

과목명	문항수	제2회	제3회	제4회	제5회	제6회
전체	120	0.797	0.814	.843	.888	**.886**
직업재활개론	22	0.414	0.433	.353	.574	**.522**
재활상담	22	0.431	0.587	.496	.641	**.575**
재활사례관리	22	0.407	0.534	.602	.661	**.675**
직업평가	12	0.126	0.362	.444	.462	**.367**
직무개발과 배치	17	0.578	0.190	.388	.600	**.544**
재활행정	10	0.149	0.396	.365	.404	**.493**
재활정책	15	0.406	0.305	.472	.417	**.543**

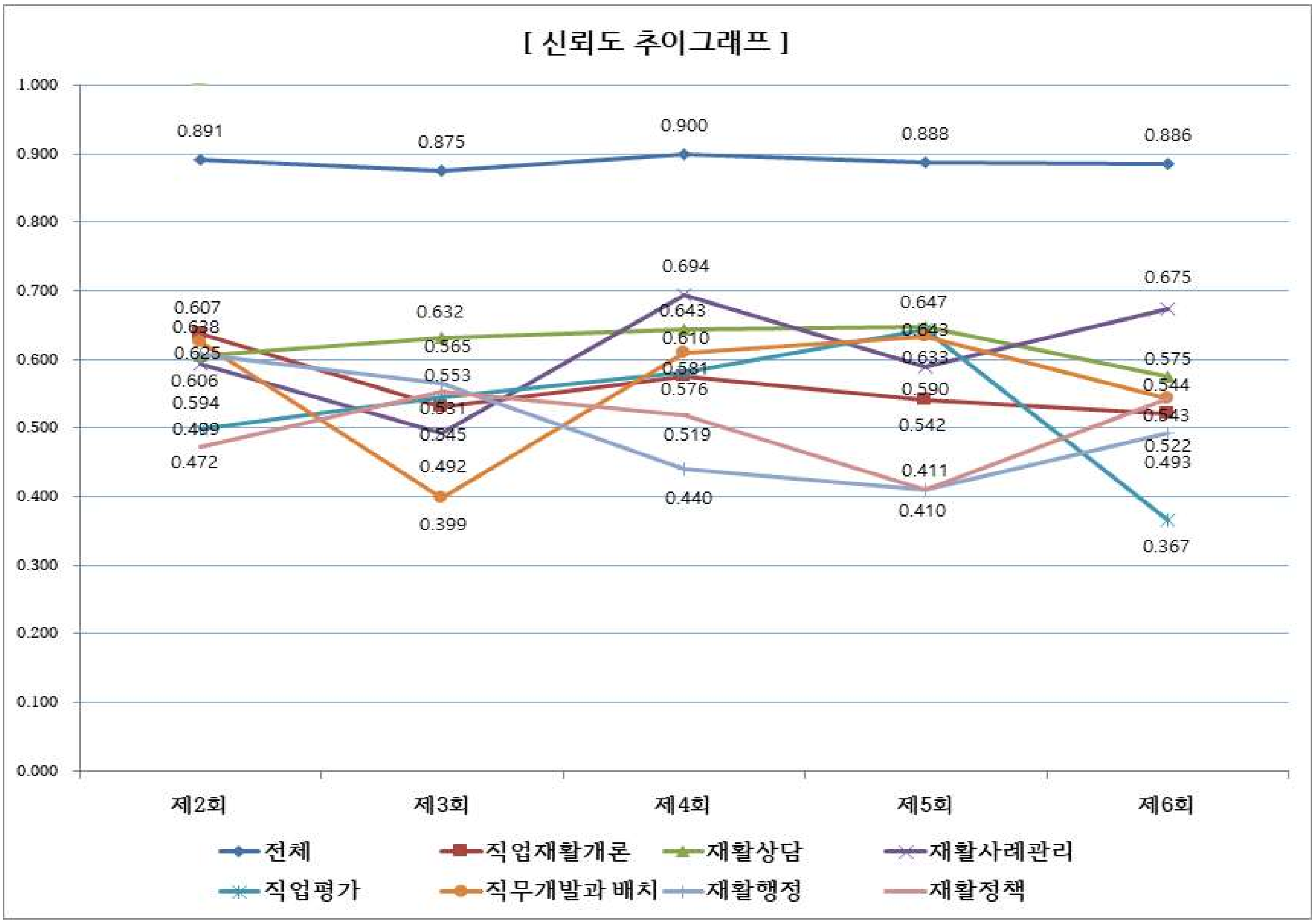

해석

- 전회 대비 전체문항의 신뢰도는 .002 감소함
- 전회 대비 재활사례관리, 재활행정, 재활정책 과목의 신뢰도는 각각 .085, .083, .132 증가함
- 전회 대비 직업재활개론, 재활상담, 직업평가, 직무개발과 배치 과목의 신뢰도는 각각 .020, .072, .276, .089 감소함
