# Supplementary material for: Item difficulty index, discrimination index, and reliability of the 26 health professions licensing examinations in 2022, Korea: a psychometric study
Source: J Educ Eval Health Prof. 2023 Nov 22;20:31. doi: 10.3352/jeehp.2023.20.31 (PMC11959405; doi:10.3352/jeehp.2023.20.31)
Supplement: Supplementary file 1 — Supplement 1. Item analysis results of 26 health professions licensing examinations administered during late 2022 and early 2023. [file jeehp-20-31_Suppl1.zip › 2022│Γ╡╡ ┴a73╚╕ ╛α╗τ ▒╣░í╜├╟Φ ║╨╝«░ß░·.pdf]

# 2022년도 제73회
# 약사 국가시험
# 문항분석 결과

국민이 신뢰하고 감동하는 시험평가기관
한국보건의료인국가시험원
KOREA HEALTH PERSONNEL LICENSING EXAMINATION INSTITUTE

# 일반 용어 정의

□ **평균**
- 집단에서의 대표적 경향값으로 전체 값을 더하여 총 응시자로 나눈 값

□ **표준편차**
- 평균과 각 점수의 차이인 편차들의 평균으로 점수가 흩어져 분포되어 있는 정도

□ **추정난이도**
- 문항개발자가 예측한 정답률

□ **검사이론**
- 검사와 검사를 구성하고 있는 문항의 양호도를 분석 및 평가하는 방법을 정의한 이론체계
- 대표적으로 고전검사이론과 문항반응이론이 있음

## 고전검사이론 용어 정의

□ 고전검사이론(Classical Test Theory; CTT)

- 검사의 질을 분석하는 검사이론 중 한 가지로 19세기 말부터 전개되어 현재까지 주로 사용되고 있는 검사이론임
- 고전검사이론에 의한 문항과 응시자 능력 추정치는 다음과 같음

○ **문항난이도**

· 검사 문항의 쉽고 어려운 정도를 나타내는 지수
· 난이도 지수는 총 반응 수에 대한 정답 반응 수의 비율로 문항의 정답률임
· 문항난이도는 0~100까지의 값을 가짐
· 난이도 값이 큰 경우, 쉬운 문항으로 '난이도가 낮다'라고 해석하며, 난이도 값이 작은 경우, 어려운 문항으로 '난이도가 높다'라고 해석함

○ **문항변별도**

· 각 문항이 응시자의 능력 수준을 변별할 수 있는 정도를 나타내는 지수
· 문항변별도는 −1~+1까지의 값을 가지며, 1에 가까울수록 변별력 크다고 해석함
· 일반적으로 문항변별도가 0.3 이상이면 우수한 문항으로 평가함
· 구하는 방식에는 '상하위집단 구분법', '문항-총점 상관계수' 등이 있음
  1) **변별도 1(상하위구분법):** 상위 27%와 하위 27% 집단의 난이도 차이를 구하는 방식
  2) **변별도 2(상관계수법):** 문항-총점과의 상관계수로 구하는 방식

○ **신뢰도**

· 시험이 평가하고자 하는 것을 일관성 있게 측정하는가로 시험이 오차없이 정확하게 측정한 정도를 의미함
· 국시원에서는 문항의 내적일관성(Cronbach $\alpha$)으로 신뢰도를 추정하며 1에 가까울수록 신뢰도가 높다고 해석함

## 목 차


**I. 시행 결과 ···································· 5**

**1. 시험 현황 ···································· 6**

1) 시험명 ···································· 6

2) 시험시행일 ···································· 6

3) 응시현황 ···································· 6

4) 과목별 문항 수, 배점 및 과락 점수 ···································· 6

**2. 합격률과 평균성적 ···································· 6**

1) 합격 및 불합격 현황 ···································· 6

2) 과목별 과락자수 내역 ···································· 6

3) 전회 대비 합격률과 평균성적 ···································· 7

**II. 문항분석 결과 ···································· 8**

**1. 성적 ···································· 9**

1) 전체 성적분포도 ···································· 9

2) 과목별 성적분포도 ···································· 10

**2. 난이도와 변별도 ···································· 11**

1) 전체 난이도와 변별도 ···································· 11

2) 과목별 난이도와 변별도 ···································· 14

3) 지식수준별 난이도와 변별도 ···································· 26

4) 문항형태별 난이도와 변별도 ···································· 35

**3. 난이도와 변별도 간 산포도 ···································· 41**

1) 전체 난이도와 변별도 간 산포도 ···································· 41

2) 과목별 난이도와 변별도 간 산포도 ···································· 42

**4. 신뢰도 분석 ···································· 44**

# I. 시행 결과

## 1. 시험 현황

1) 시험명: 2022년도 제73회 약사 국가시험

2) 시험시행일: 2022년 1월 21일

3) 응시현황

| 응시대상자 수 | 결시자 수 | 부정행위자 수 | 응시자 준수사항 위반자 수 | | 응시자 수 (%) |
|---|---|---|---|---|---|
| | | | 휴대폰 소지 | 신분증 미지참 | |
| 2,008 | 15 | 0 | 0 | 0 | 1,993 (99.3) |

4) 과목별 문항 수, 배점 및 과락 점수

| 교 시 | 과 목 명 | 문제 수 | 배점 | 총점 | 합격자 점수기준 | |
|---|---|---|---|---|---|---|
| | | | | | 과목 과락기준 | 총점 합격기준 |
| 1교시 | 생명약학 | 100 | 1 | 100 | 40점 미만 | 210점 이상 |
| 2교시 | 산업약학 | 90 | 1 | 90 | 36점 미만 | |
| 3교시 | 임상 · 실무약학1 | 77 | 1 | 77 | 56점 미만 | |
| 4교시 | 임상 · 실무약학2 | 63 | 1 | 63 | | |
| 4교시 | 보건 · 의약관계법규 | 20 | 1 | 20 | 8점 미만 | |
| 계 | | 350 | | 350 | | |

※ 3교시 임상 · 실무약학1과 4교시 임상 · 실무약학2는 동일과목임

## 2. 합격률과 평균성적

1) 합격 및 불합격 현황

| 합격자 수 (%) | 불합격자 수(%) | | | | | 채점보류자 수 |
|---|---|---|---|---|---|---|
| | 평락 | 과락 | 실기탈락 | 기권 | 계 | |
| 1,840 (92.3) | 153 (7.7) | 0 (0.0) | - - | 0 (0.0) | 153 (7.7) | 0 |

2) 과목별 과락자수 내역

| 과목명 / 과락자 수 | 생명약학 | 산업약학 | 임상·실무약학 | 보건·의약관계법규 |
|---|---|---|---|---|
| 과목별 과락자 수 | 0 | 0 | 0 | 0 |
| 전과목 과락자 수 | 0 | | | |

3) 전회 대비 합격률과 평균성적

| 회차 | 년도 | 합격률(%) | 평균성적 | 표준편차 | 백분율 환산점수 |
|---|---|---|---|---|---|
| 제69회 | 2018 | 91.2 | 263.1 | 41.7 | 75.1 |
| 제70회 | 2019 | 90.0 | 263.5 | 42.0 | 75.3 |
| 제71회 | 2020 | 91.1 | 260.7 | 38.2 | 74.5 |
| 제72회 | 2021 | 91.0 | 246.0 | 31.1 | 70.3 |
| **제73회** | **2022** | **92.3** | **248.0** | **31.2** | **70.9** |

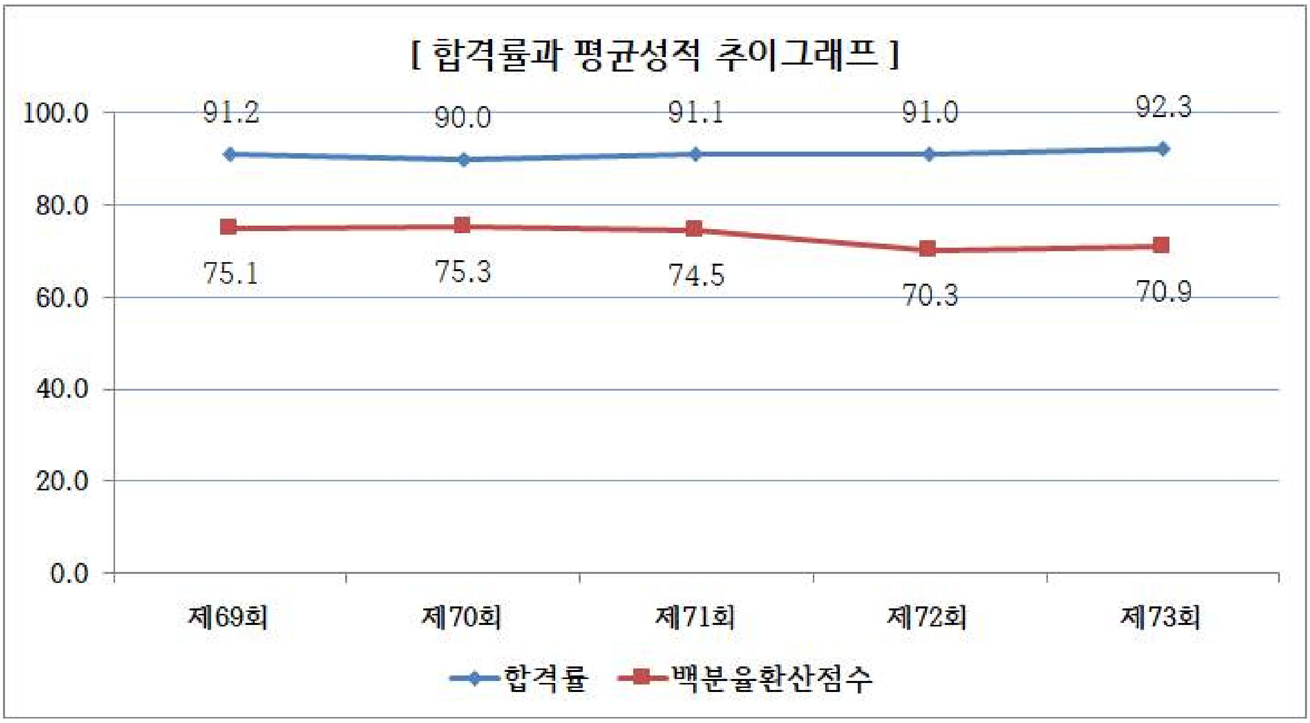


**해석**

- 전년 대비 합격률은 1.3% 증가하고, 백분율 환산점수는 0.6 점 증가함

# II. 문항분석 결과

# 1. 성적

## 1) 전체 성적분포도

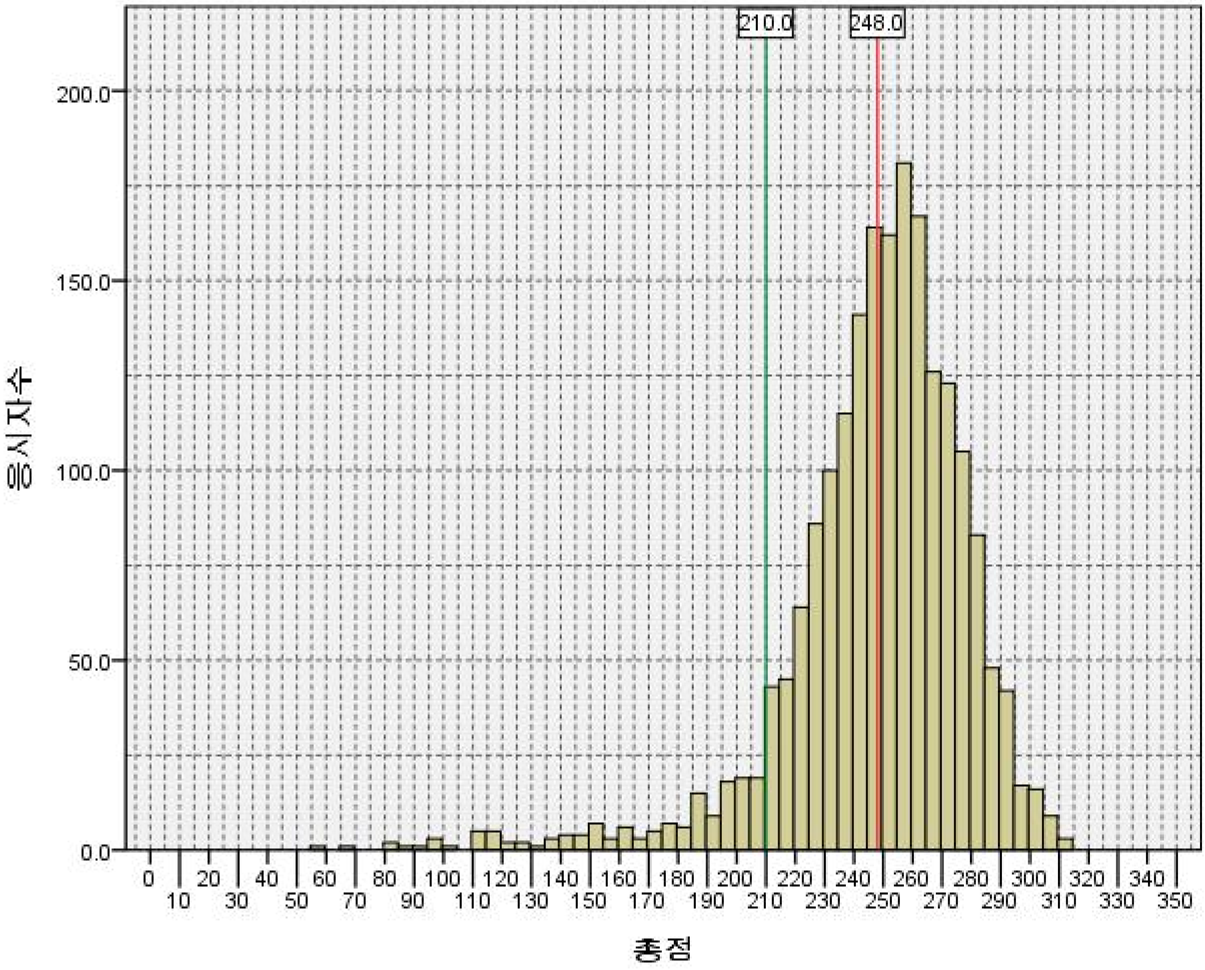


| 응시자 | 총점 | 합격선 | 평균성적 | 표준편차 |
|---|---|---|---|---|
| 1,993 | 350 | 210 | 248.0 | 31.2 |

※ 1.993은 전체응시자(2,008명)에서 기권자(0명)를 제외한 수치임

2) 과목별 성적분포도

가) 생명약학

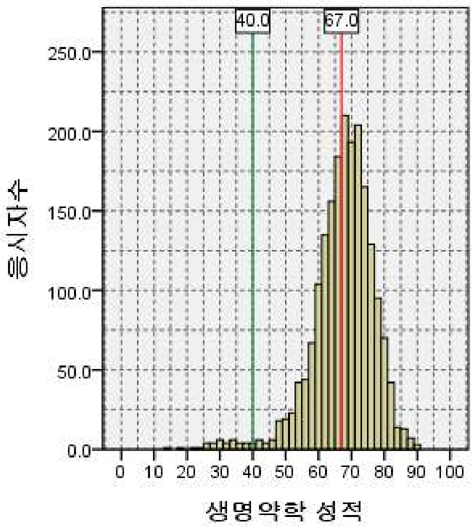


| 총점 | 과락선 | 평균성적 | 표준편차 |
|---|---|---|---|
| 100 | 40 | 67.0 | 9.4 |

나) 산업약학

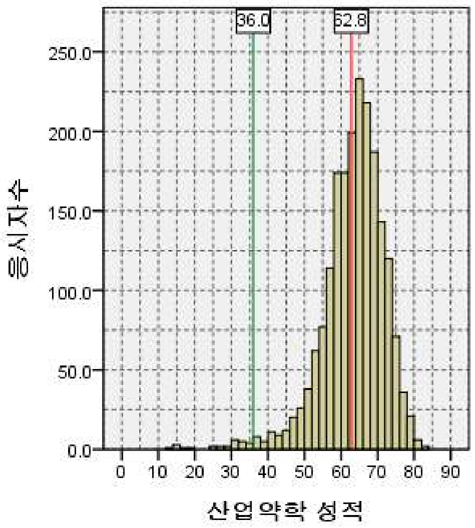


| 총점 | 과락선 | 평균성적 | 표준편차 |
|---|---|---|---|
| 90 | 36 | 62.8 | 8.6 |

다) 임상·실무약학

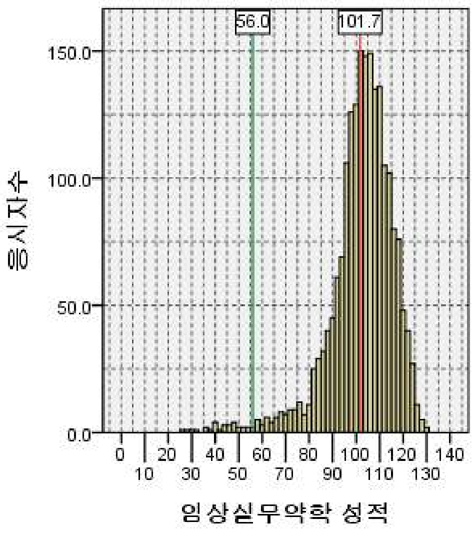


| 총점 | 과락선 | 평균성적 | 표준편차 |
|---|---|---|---|
| 140 | 56 | 101.7 | 13.8 |

라) 보건·의약관계법규

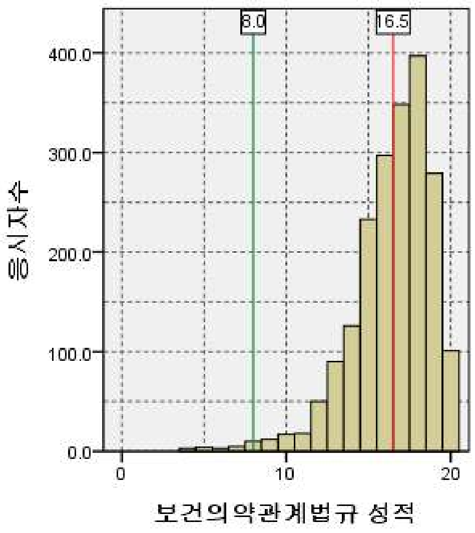


| 총점 | 과락선 | 평균성적 | 표준편차 |
|---|---|---|---|
| 20 | 8 | 16.5 | 2.4 |

## 2. 난이도와 변별도

### 1) 전체 난이도와 변별도

가) 전회 대비 전체 난이도와 변별도

| 회차 | 난이도 | | 변별도1 | | 변별도2 | |
|---|---|---|---|---|---|---|
| | 평균 | 표준편차 | 평균 | 표준편차 | 평균 | 표준편차 |
| 제69회 | 75.2 | 19.3 | .25 | .11 | .33 | .14 |
| 제70회 | 75.3 | 19.6 | .25 | .12 | .33 | .15 |
| 제71회 | 74.5 | 20.7 | .24 | .13 | .30 | .13 |
| 제72회 | 70.3 | 22.7 | .20 | .11 | .23 | .11 |
| **제73회** | **70.9** | **23.3** | **.19** | **.11** | **.24** | **.11** |

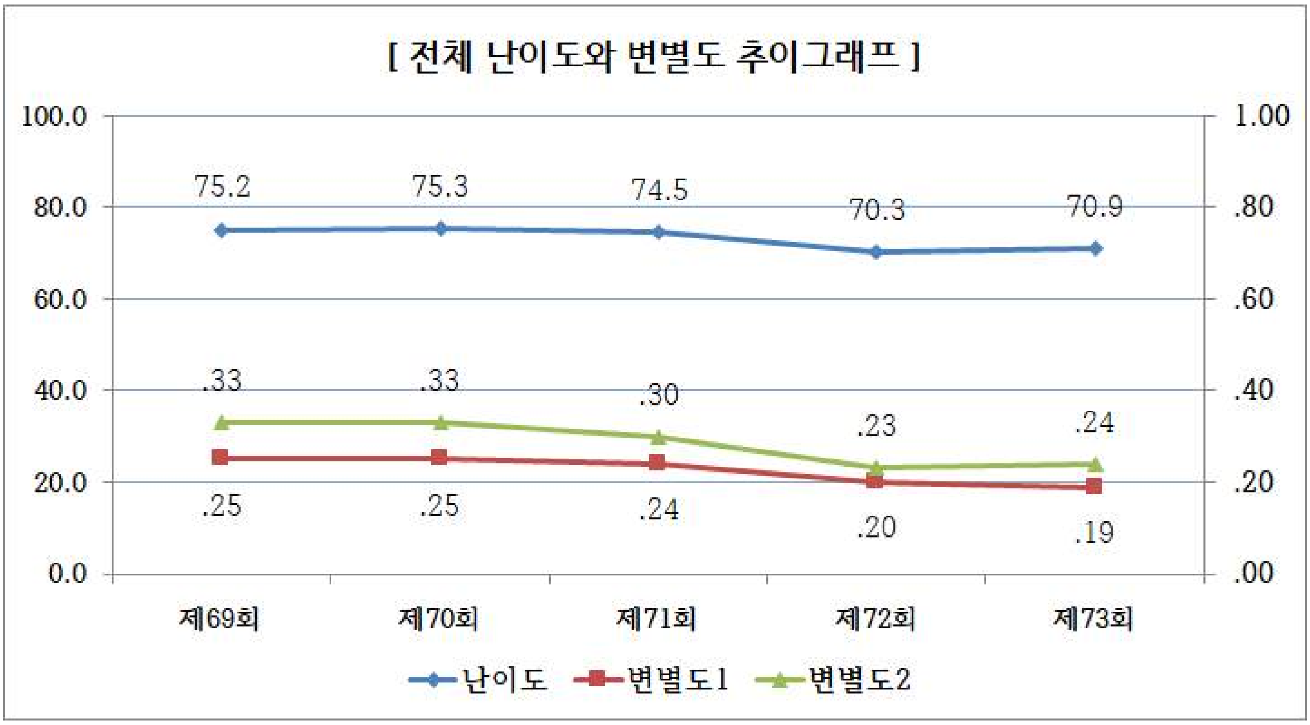


**해석**

- 전회 대비 난이도 지수는 0.6 증가함
- 전회 대비 변별도 1 지수는 .01 감소함
- 전회 대비 변별도 2 지수는 .01 증가함

## 나) 전체 난이도와 변별도 분포도 및 비율분석

### (1) 전체 난이도 분포도 및 비율분석

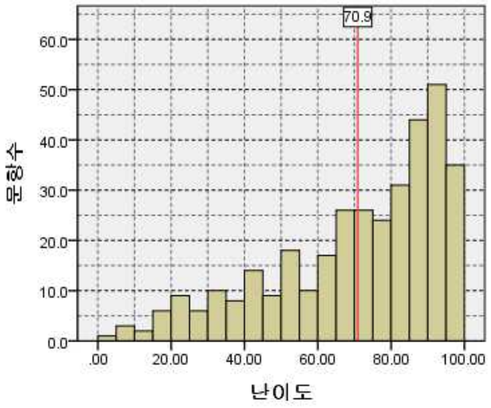


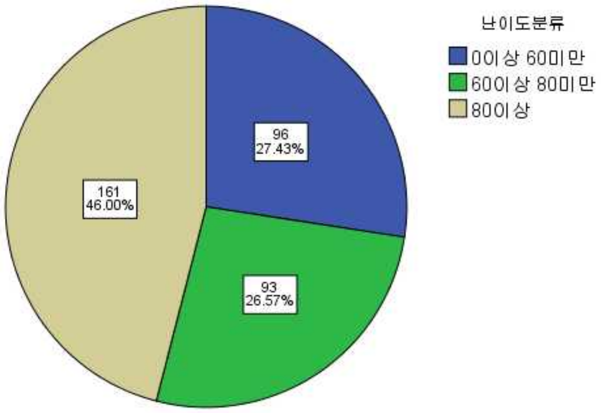


| 총점 | 난이도 | 표준편차 |
|---|---|---|
| 350 | 70.9 | 23.3 |

| 난이도 | 문항수 | 비율(%) |
|---|---|---|
| 0~60미만 | 96 | 27.4 |
| 60~80미만 | 93 | 26.6 |
| 80~100 | 161 | 46.0 |
| 전체 | 350 | 100.0 |

### (2) 전체 변별도1 분포도 및 비율분석

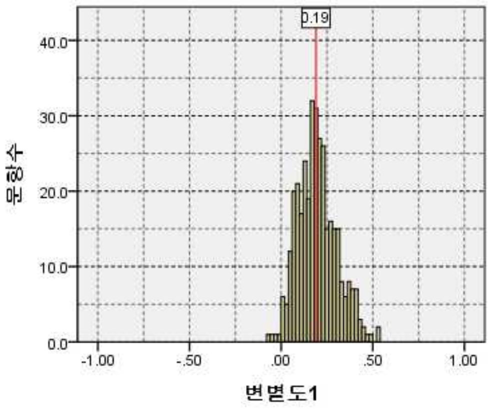


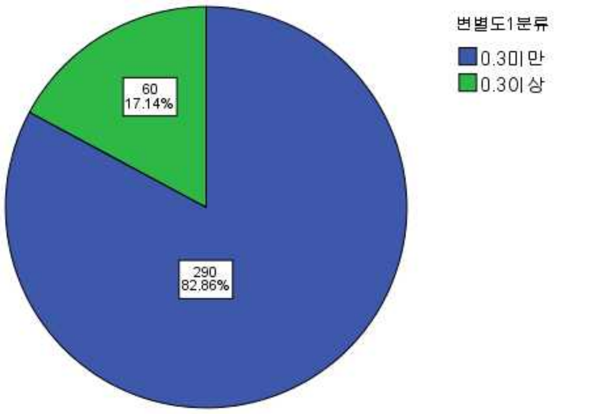


| 총점 | 변별도1 | 표준편차 |
|---|---|---|
| 350 | .19 | .11 |

| 변별도1 | 문항수 | 비율(%) |
|---|---|---|
| 0.3미만 | 290 | 82.9 |
| 0.3이상 | 60 | 17.1 |
| 전체 | 350 | 100.0 |

(3) 전체 변별도2 분포도 및 비율분석

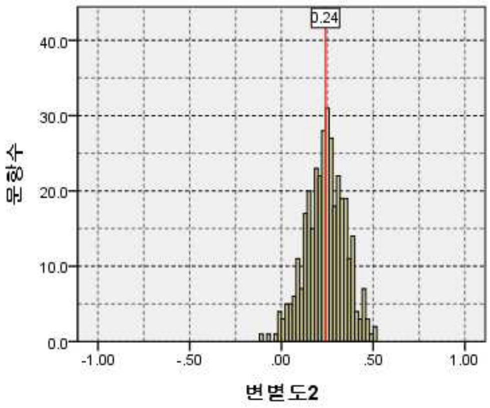


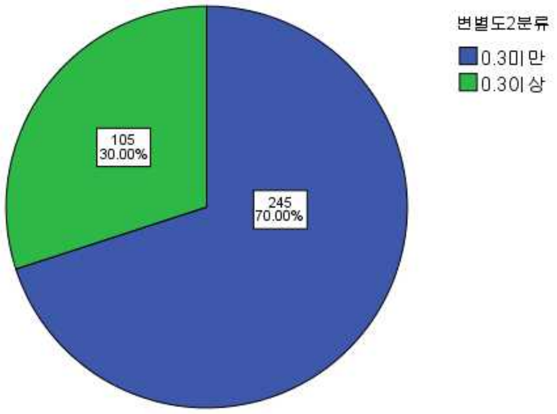


| 총점 | 변별도2 | 표준편차 |
|---|---|---|
| 350 | .24 | .11 |

| 변별도2 | 문항수 | 비율(%) |
|---|---|---|
| 0.3미만 | 245 | 70.0 |
| 0.3이상 | 105 | 30.0 |
| 전체 | 350 | 100.0 |

**해석**

- 난이도 지수가 80 이상인 문항이 161 문항으로 가장 많았으며, 60 미만인 문항이 96 문항, 60 이상 80 미만인 문항이 93 문항으로 나타남
- 변별도 1 지수를 기준으로 분류하였을 때, 0.3 미만인 문항이 290 문항으로 0.3 이상인 문항이 60 문항인 것에 비해 더 많이 나타남
- 변별도 2 지수를 기준으로 분류하였을 때, 0.3 미만인 문항이 245 문항으로 0.3 이상인 문항이 105 문항인 것에 비해 더 많이 나타남

2) 과목별 난이도와 변별도

가) 전회 대비 과목별 난이도와 변별도

(1) 전회 대비 생명약학 난이도와 변별도

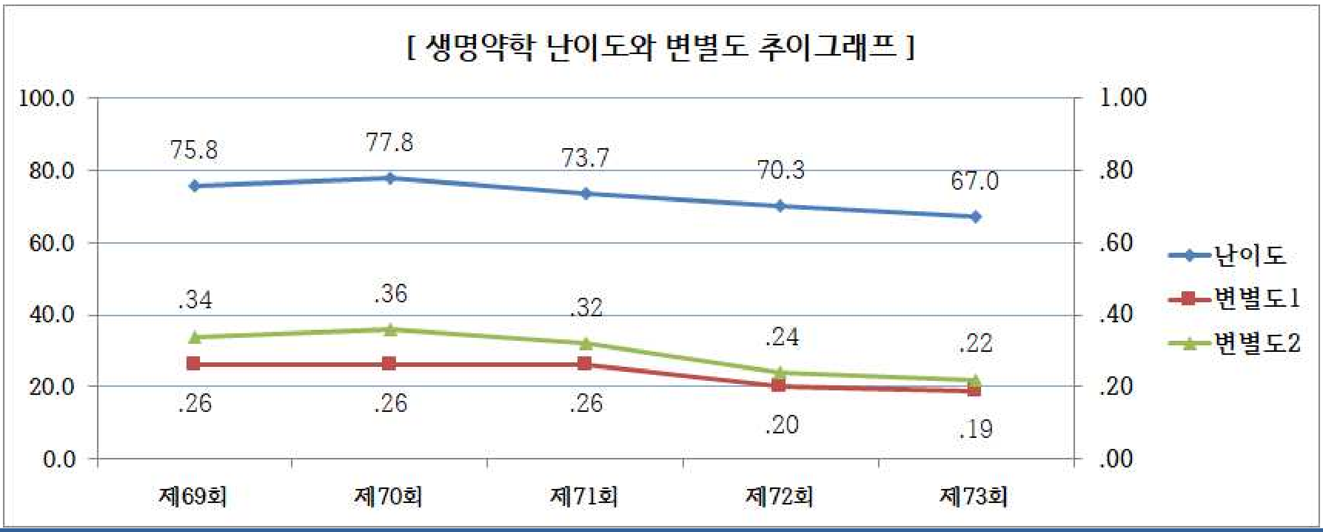


| 회차 | 난이도 | | 변별도1 | | 변별도2 | |
|---|---|---|---|---|---|---|
| | 평균 | 표준편차 | 평균 | 표준편차 | 평균 | 표준편차 |
| 제69회 | 75.8 | 18.9 | .26 | .11 | .34 | .13 |
| 제70회 | 77.8 | 18.7 | .26 | .12 | .36 | .15 |
| 제71회 | 73.7 | 20.8 | .26 | .13 | .32 | .12 |
| 제72회 | 70.3 | 24.2 | .20 | .12 | .24 | .11 |
| **제73회** | **67.0** | **25.6** | **.19** | **.10** | **.22** | **.12** |

**해석**

- 전회 대비 생명약학 과목의 난이도는 3.3 감소함
- 전회 대비 생명약학 과목의 변별도 1 지수는 .01 감소함
- 전회 대비 생명약학 과목의 변별도 2 지수는 .02 감소함

(2) 전회 대비 산업약학 난이도와 변별도

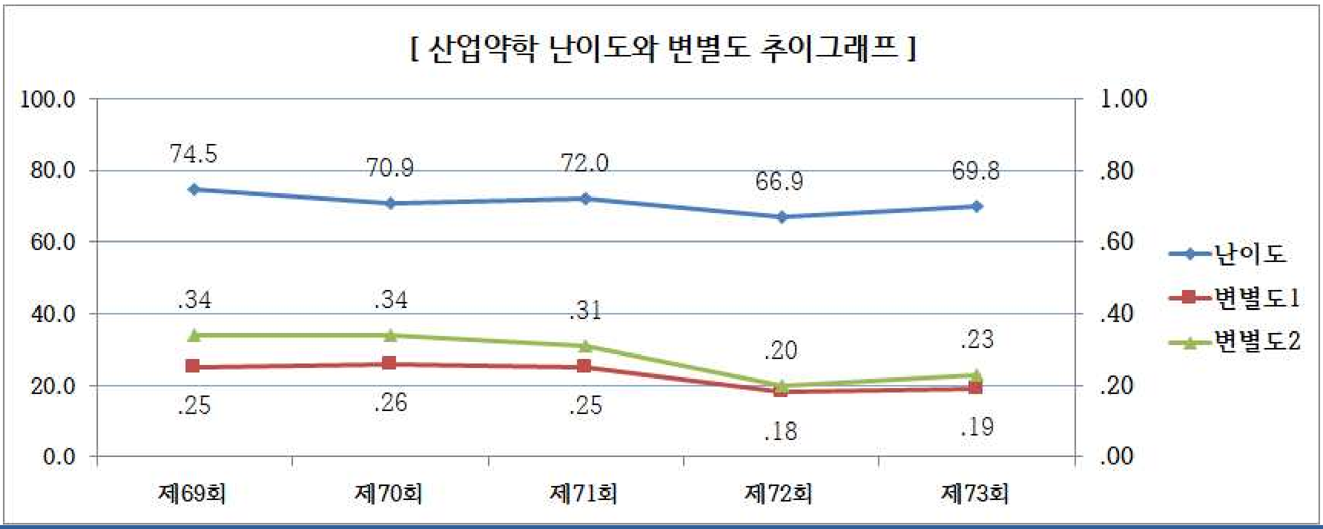


| 회차 | 난이도 | | 변별도1 | | 변별도2 | |
|---|---|---|---|---|---|---|
| | 평균 | 표준편차 | 평균 | 표준편차 | 평균 | 표준편차 |
| 제69회 | 74.5 | 19.7 | .25 | .11 | .34 | .14 |
| 제70회 | 70.9 | 22.8 | .26 | .12 | .34 | .16 |
| 제71회 | 72.0 | 22.2 | .25 | .15 | .31 | .15 |
| 제72회 | 66.9 | 24.0 | .18 | .11 | .20 | .12 |
| **제73회** | **69.8** | **23.5** | **.19** | **.10** | **.23** | **.10** |

**해석**

- 전회 대비 산업약학 과목의 난이도는 2.9 증가함
- 전회 대비 산업약학 과목의 변별도 1 지수는 .01 증가함
- 전회 대비 산업약학 과목의 변별도 2 지수는 .03 증가함

(3) 전회 대비 임상·실무약학 난이도와 변별도

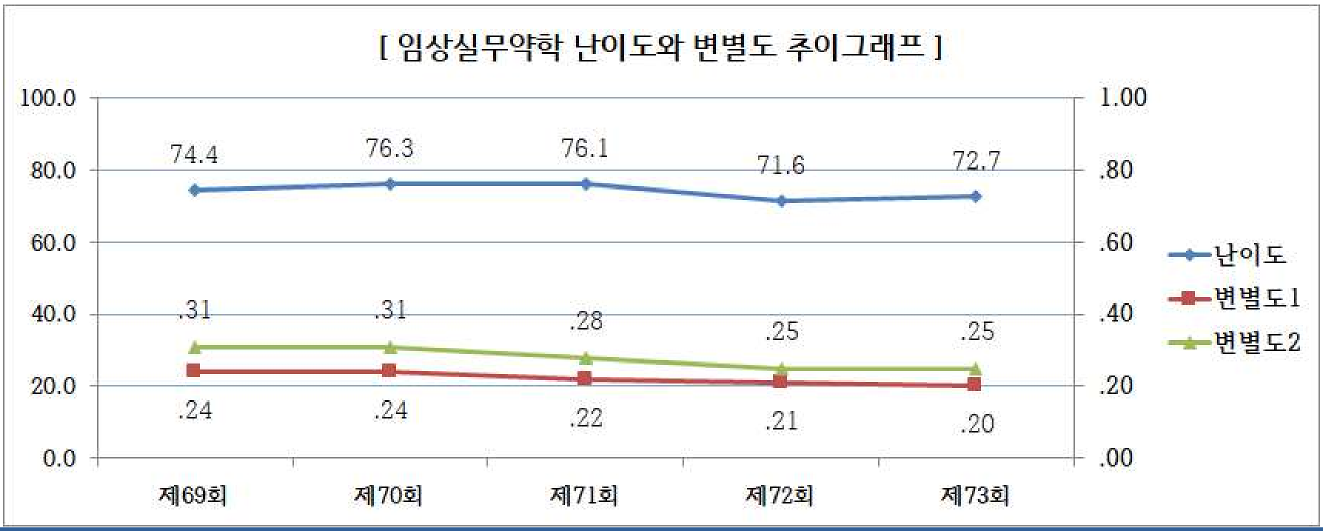


| 회차 | 난이도 | | 변별도1 | | 변별도2 | |
|---|---|---|---|---|---|---|
| | 평균 | 표준편차 | 평균 | 표준편차 | 평균 | 표준편차 |
| 제69회 | 74.4 | 19.9 | .24 | .11 | .31 | .14 |
| 제70회 | 76.3 | 17.8 | .24 | .13 | .31 | .14 |
| 제71회 | 76.1 | 19.3 | .22 | .10 | .28 | .12 |
| 제72회 | 71.6 | 20.9 | .21 | .11 | .25 | .11 |
| **제73회** | **72.7** | **21.9** | **.20** | **.11** | **.25** | **.11** |

**해석**

- 전회 대비 임상·실무약학 과목의 난이도는 1.1 증가함
- 전회 대비 임상·실무약학 과목의 변별도 1 지수는 .01 감소함
- 전회 대비 임상·실무약학 과목의 변별도 2 지수는 동일함

(4) 전회 대비 보건·의약관계법규 난이도와 변별도

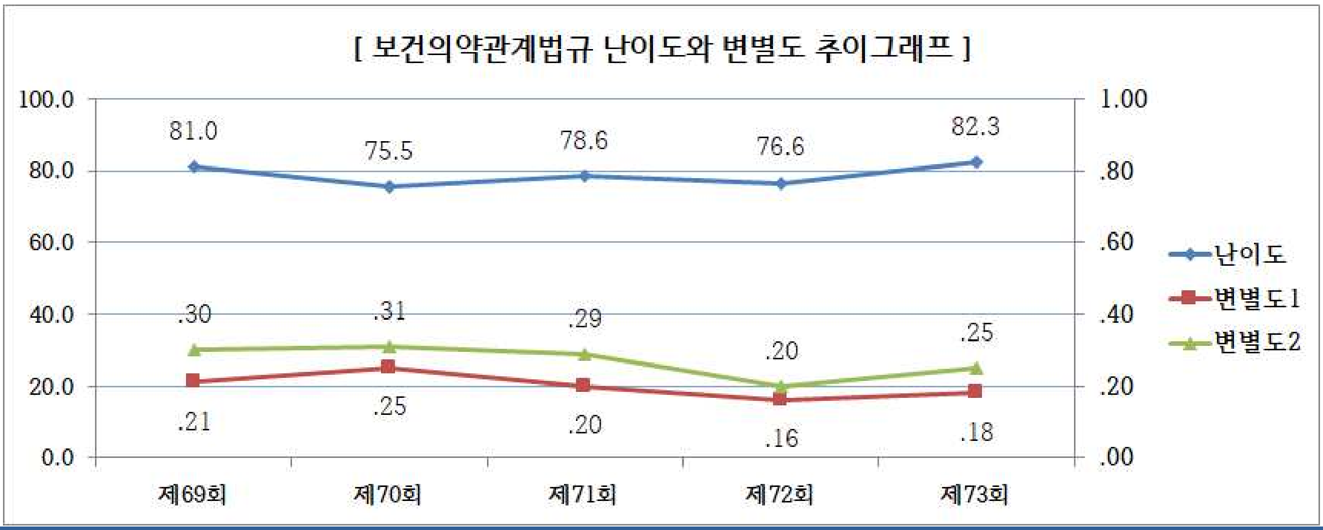


| 회차 | 난이도 | | 변별도1 | | 변별도2 | |
|---|---|---|---|---|---|---|
| | 평균 | 표준편차 | 평균 | 표준편차 | 평균 | 표준편차 |
| 제69회 | 81.0 | 14.8 | .21 | .10 | .30 | .14 |
| 제70회 | 75.5 | 18.2 | .25 | .12 | .31 | .11 |
| 제71회 | 78.6 | 22.2 | .20 | .12 | .29 | .13 |
| 제72회 | 76.6 | 21.3 | .16 | .10 | .20 | .08 |
| **제73회** | **82.3** | **15.7** | **.18** | **.11** | **.25** | **.09** |

**해석**

- 전회 대비 보건·의약관계법규 과목의 난이도는 5.7 증가함
- 전회 대비 보건·의약관계법규 과목의 변별도 1 지수는 .02 증가함
- 전회 대비 보건·의약관계법규 과목의 변별도 2 지수는 .05 증가함

## 나) 과목별 난이도와 변별도 분포도 및 비율분석

### (1) 생명약학 난이도와 변별도 분포도 및 비율분석

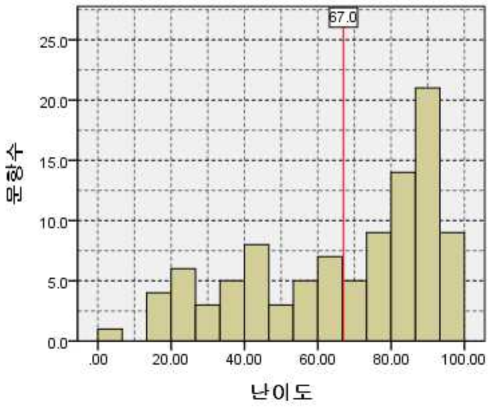


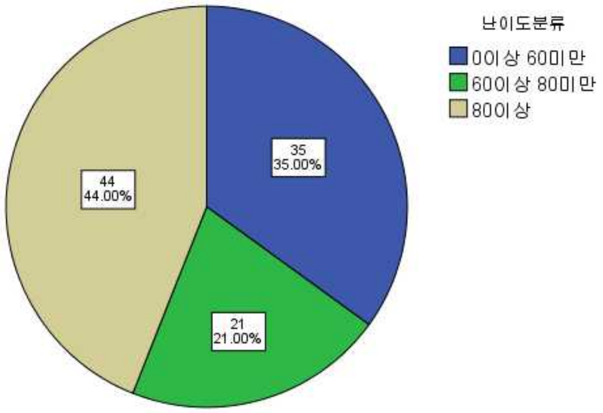


| 총점 | 난이도 | 표준편차 |
|---|---|---|
| 100 | 67.0 | 25.6 |

| 난이도 | 문항수 | 비율(%) |
|---|---|---|
| 0~60미만 | 35 | 35.0 |
| 60~80미만 | 21 | 21.0 |
| 80~100 | 44 | 44.0 |
| 전체 | 100 | 100.0 |

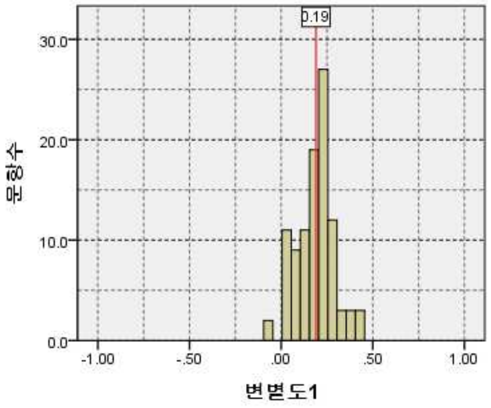


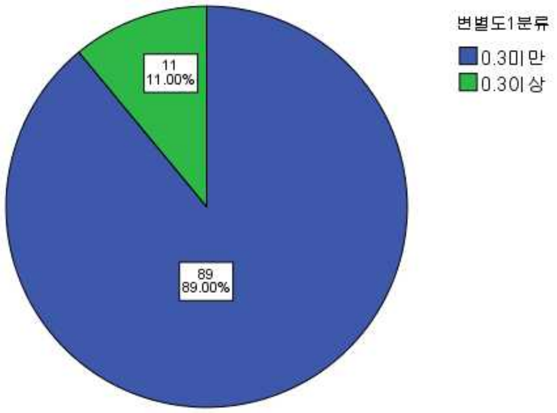


| 총점 | 변별도1 | 표준편차 |
|---|---|---|
| 100 | .19 | .10 |

| 변별도1 | 문항수 | 비율(%) |
|---|---|---|
| 0.3미만 | 89 | 89.0 |
| 0.3이상 | 11 | 11.0 |
| 전체 | 100 | 100.0 |

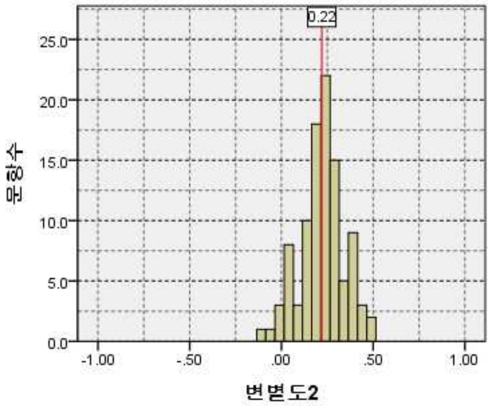


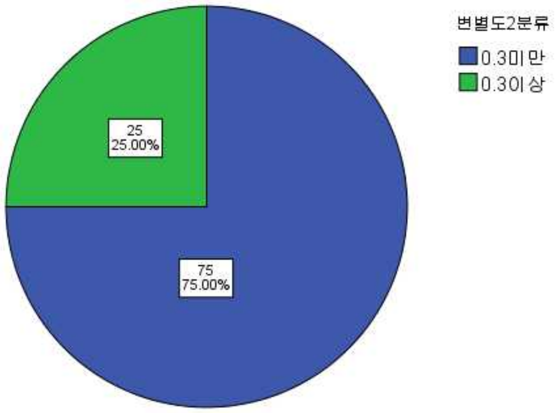


| 총점 | 변별도2 | 표준편차 |
|---|---|---|
| 100 | .22 | .12 |

| 변별도2 | 문항수 | 비율(%) |
|---|---|---|
| 0.3미만 | 75 | 75.0 |
| 0.3이상 | 25 | 25.0 |
| 전체 | 100 | 100.0 |

**해석**

- 생명약학 과목에서 난이도 지수가 80 이상인 문항이 44 문항으로 가장 많았으며, 60 미만인 문항이 35 문항, 60 이상 80 미만인 문항이 21 문항으로 나타남
- 변별도 1 지수를 기준으로 분류하였을 때, 0.3 미만인 문항이 89 문항으로 0.3 이상인 문항이 11 문항인 것에 비해 더 많이 나타남
- 변별도 2 지수를 기준으로 분류하였을 때, 0.3 미만인 문항이 75 문항으로 0.3 이상인 문항이 25 문항인 것에 비해 더 많이 나타남

(2) 산업약학 난이도와 변별도 분포도 및 비율분석

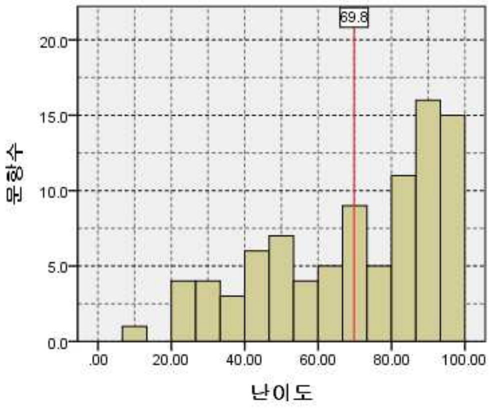


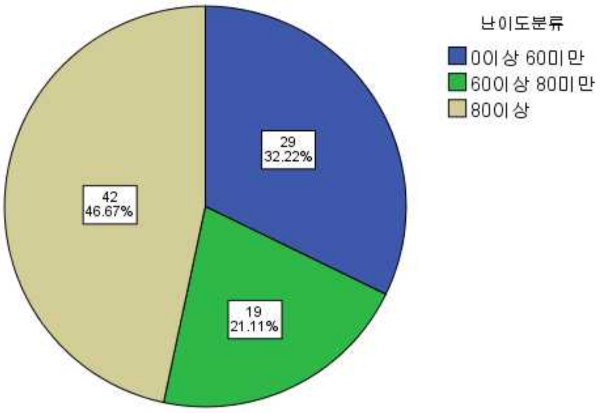


| 총점 | 난이도 | 표준편차 |
|---|---|---|
| 90 | 69.8 | 23.5 |

| 난이도 | 문항수 | 비율(%) |
|---|---|---|
| 0~60미만 | 29 | 32.2 |
| 60~80미만 | 19 | 21.1 |
| 80~100 | 42 | 46.7 |
| 전체 | 90 | 100.0 |

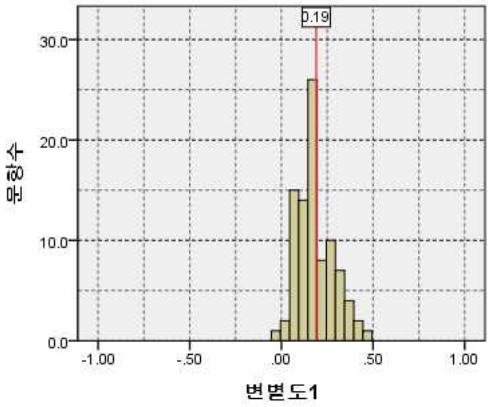


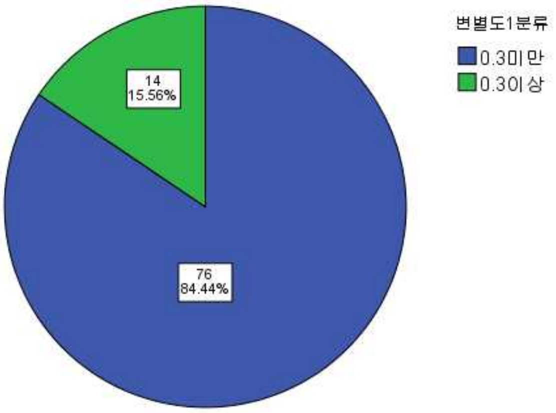


| 총점 | 변별도1 | 표준편차 |
|---|---|---|
| 90 | .19 | .10 |

| 변별도1 | 문항수 | 비율(%) |
|---|---|---|
| 0.3미만 | 76 | 84.4 |
| 0.3이상 | 14 | 15.6 |
| 전체 | 90 | 100.0 |

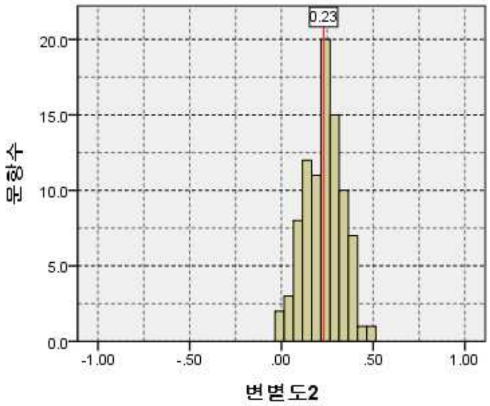


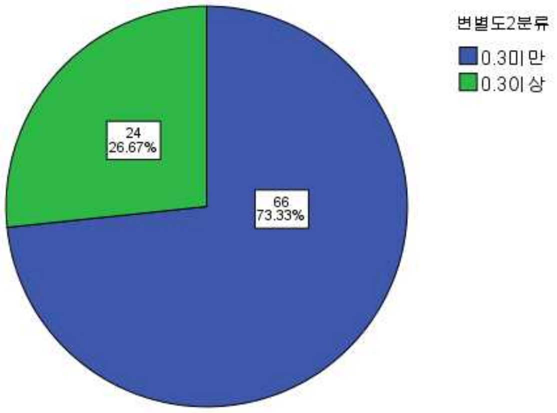


| 총점 | 변별도2 | 표준편차 |
|---|---|---|
| 90 | .23 | .10 |

| 변별도2 | 문항수 | 비율(%) |
|---|---|---|
| 0.3미만 | 66 | 73.3 |
| 0.3이상 | 24 | 26.7 |
| 전체 | 90 | 100.0 |

**해석**

- 산업약학 과목에서 난이도 지수가 80 이상인 문항이 42 문항으로 가장 많았으며, 60 미만인 문항이 29 문항, 60 이상 80 미만인 문항이 19 문항으로 나타남
- 변별도 1 지수를 기준으로 분류하였을 때, 0.3 미만인 문항이 76 문항으로 0.3 이상인 문항이 14 문항인 것에 비해 더 많이 나타남
- 변별도 2 지수를 기준으로 분류하였을 때, 0.3 미만인 문항이 66 문항으로 0.3 이상인 문항이 24 문항인 것에 비해 더 많이 나타남

(3) 임상·실무약학 난이도와 변별도 분포도 및 비율분석

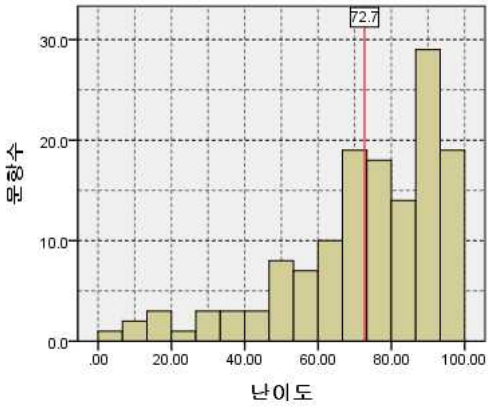


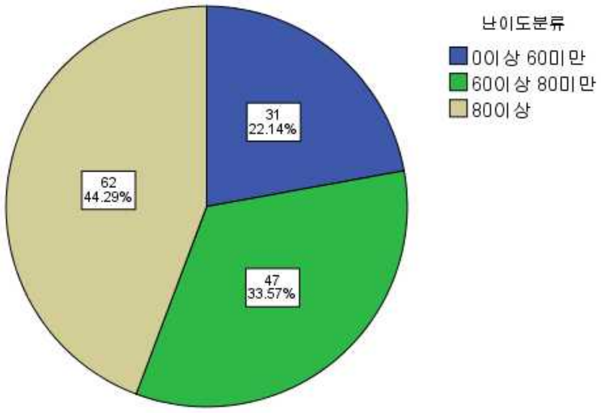


| 총점 | 난이도 | 표준편차 |
|---|---|---|
| 140 | 72.7 | 21.9 |

| 난이도 | 문항수 | 비율(%) |
|---|---|---|
| 0~60미만 | 31 | 22.1 |
| 60~80미만 | 47 | 33.6 |
| 80~100 | 62 | 44.3 |
| 전체 | 140 | 100.0 |

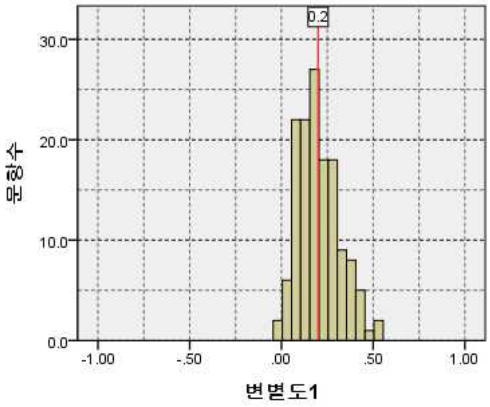


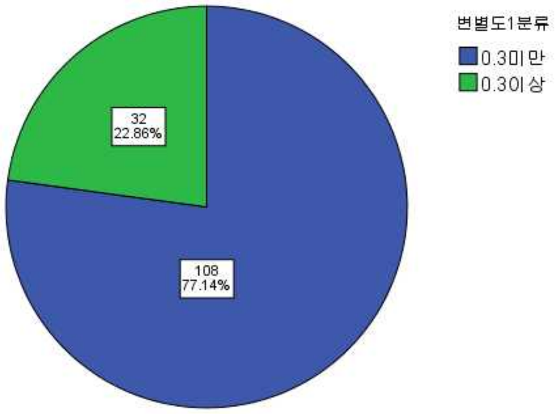


| 총점 | 변별도1 | 표준편차 |
|---|---|---|
| 140 | .20 | .11 |

| 변별도1 | 문항수 | 비율(%) |
|---|---|---|
| 0.3미만 | 108 | 77.1 |
| 0.3이상 | 32 | 22.9 |
| 전체 | 140 | 100.0 |

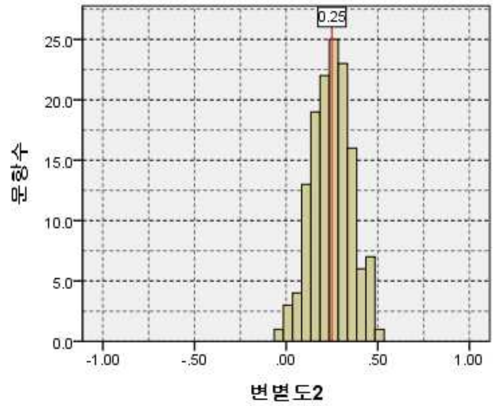


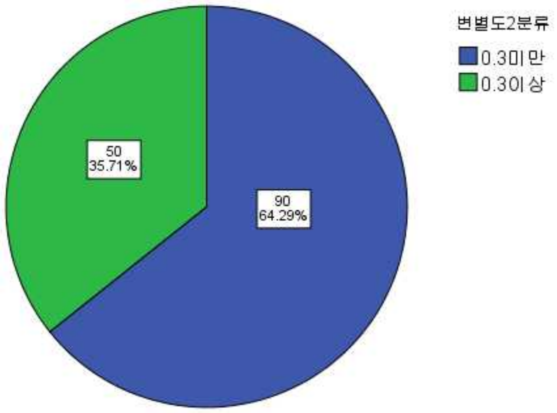


| 총점 | 변별도2 | 표준편차 |
|---|---|---|
| 140 | .25 | .11 |

| 변별도2 | 문항수 | 비율(%) |
|---|---|---|
| 0.3미만 | 90 | 64.3 |
| 0.3이상 | 50 | 35.7 |
| 전체 | 140 | 100.0 |

### 해석

- 임상·실무약학 과목에서 난이도 지수가 80 이상인 문항이 62 문항으로 가장 많았으며, 60 이상 80 미만인 문항이 47 문항, 60 미만인 문항이 31 문항으로 나타남
- 변별도 1 지수를 기준으로 분류하였을 때, 0.3 미만인 문항이 108 문항으로 0.3 이상인 문항이 32 문항인 것에 비해 더 많이 나타남
- 변별도 2 지수를 기준으로 분류하였을 때, 0.3 미만인 문항이 90 문항으로 0.3 이상인 문항이 50 문항인 것에 비해 더 많이 나타남

(4) 보건·의약관계법규 난이도와 변별도 분포도 및 비율분석

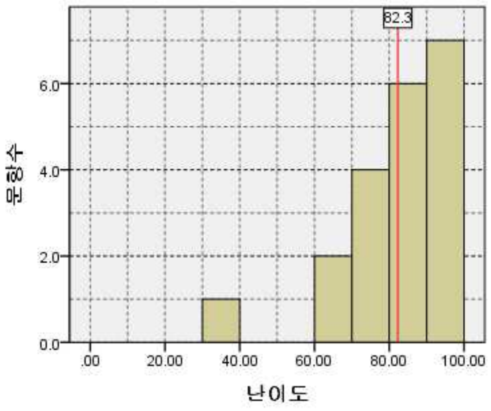


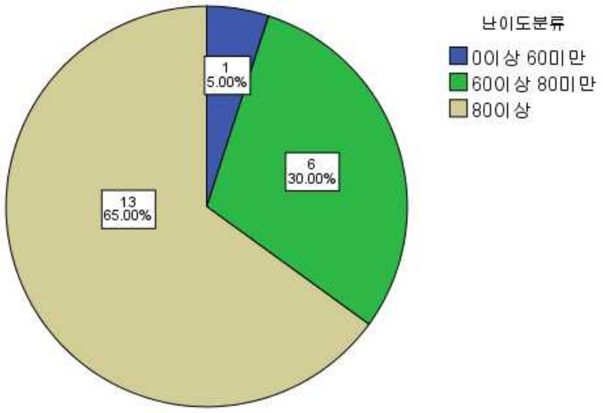


| 총점 | 난이도 | 표준편차 |
|---|---|---|
| 20 | 82.3 | 15.7 |

| 난이도 | 문항수 | 비율(%) |
|---|---|---|
| 0~60미만 | 1 | 5.0 |
| 60~80미만 | 6 | 30.0 |
| 80~100 | 13 | 65.0 |
| 전체 | 20 | 100.0 |

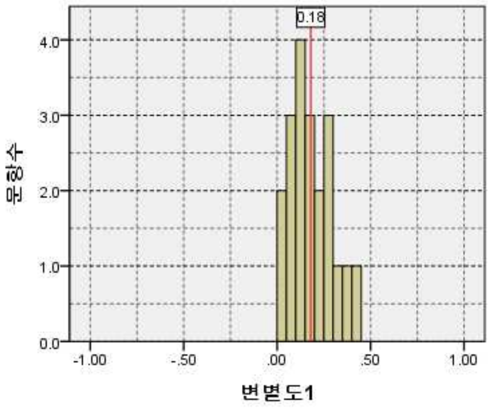


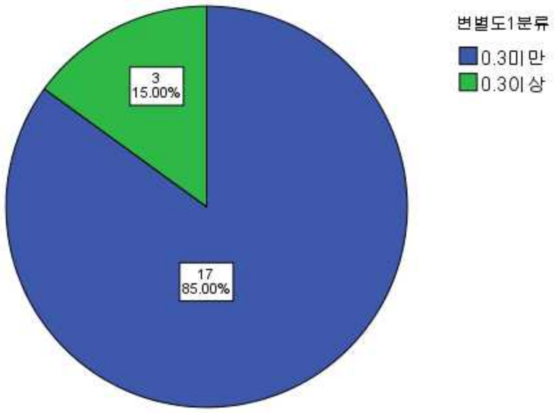


| 총점 | 변별도1 | 표준편차 |
|---|---|---|
| 20 | .18 | .11 |

| 변별도1 | 문항수 | 비율(%) |
|---|---|---|
| 0.3미만 | 17 | 85.0 |
| 0.3이상 | 3 | 15.0 |
| 전체 | 20 | 100.0 |

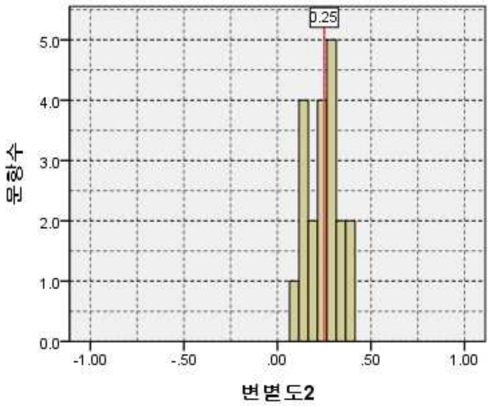


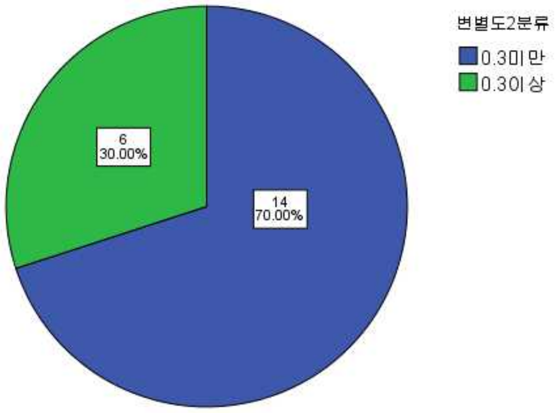


| 총점 | 변별도2 | 표준편차 |
|---|---|---|
| 20 | .25 | .09 |

| 변별도2 | 문항수 | 비율(%) |
|---|---|---|
| 0.3미만 | 14 | 70.0 |
| 0.3이상 | 6 | 30.0 |
| 전체 | 20 | 100.0 |

**해석**

- 보건·의약관계법규 과목에서 난이도 지수가 80 이상인 문항이 13 문항으로 가장 많았으며, 60 이상 80 미만인 문항이 6 문항, 60 미만인 문항이 1 문항으로 나타남
- 변별도 1 지수를 기준으로 분류하였을 때, 0.3 미만인 문항이 17 문항으로 0.3 이상인 문항이 3 문항인 것에 비해 더 많이 나타남
- 변별도 2 지수를 기준으로 분류하였을 때, 0.3 미만인 문항이 14 문항으로 0.3 이상인 문항이 6 문항인 것에 비해 더 많이 나타남

### 3) 지식수준별 난이도와 변별도

#### 가) 전회 대비 지식수준별 난이도와 변별도

##### (1) 전회 대비 암기형 난이도와 변별도

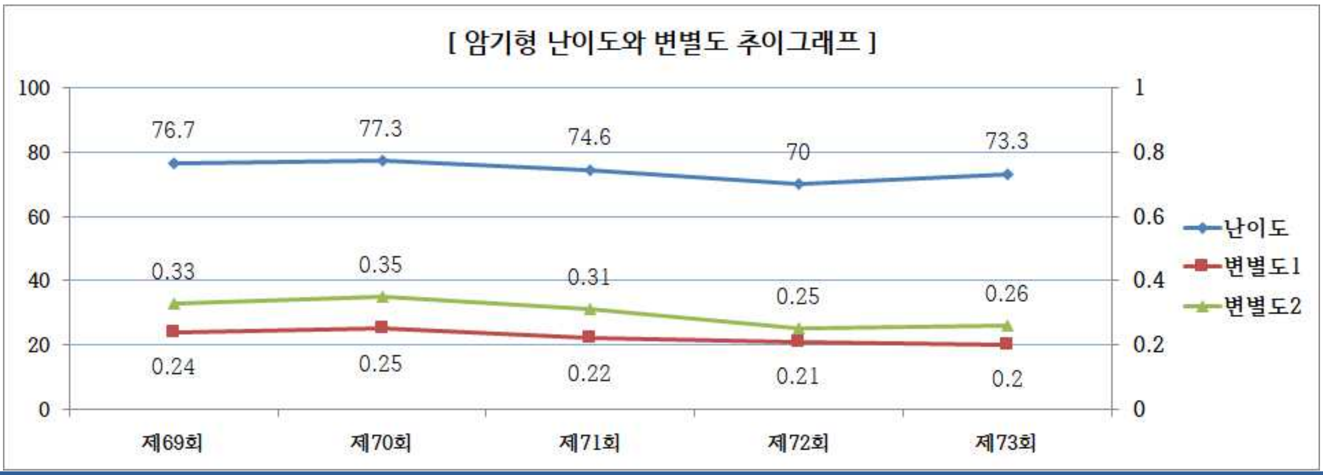


| 회차 | 난이도 | | 변별도1 | | 변별도2 | |
|---|---|---|---|---|---|---|
| | 평균 | 표준편차 | 평균 | 표준편차 | 평균 | 표준편차 |
| 제69회 | 76.7 | 18.8 | .24 | .12 | .33 | .15 |
| 제70회 | 77.3 | 20.7 | .25 | .11 | .35 | .14 |
| 제71회 | 74.6 | 23.7 | .22 | .12 | .31 | .13 |
| 제72회 | 70.0 | 23.9 | .21 | .12 | .25 | .12 |
| **제73회** | **73.3** | **23.1** | **.20** | **.10** | **.26** | **.09** |

**해석**

- 전회 대비 암기형 문항의 난이도 지수는 3.3 증가함
- 전회 대비 암기형 문항의 변별도 1 지수는 .01 감소함
- 전회 대비 암기형 문항의 변별도 2 지수는 .01 증가함

(2) 전회 대비 해석형 난이도와 변별도

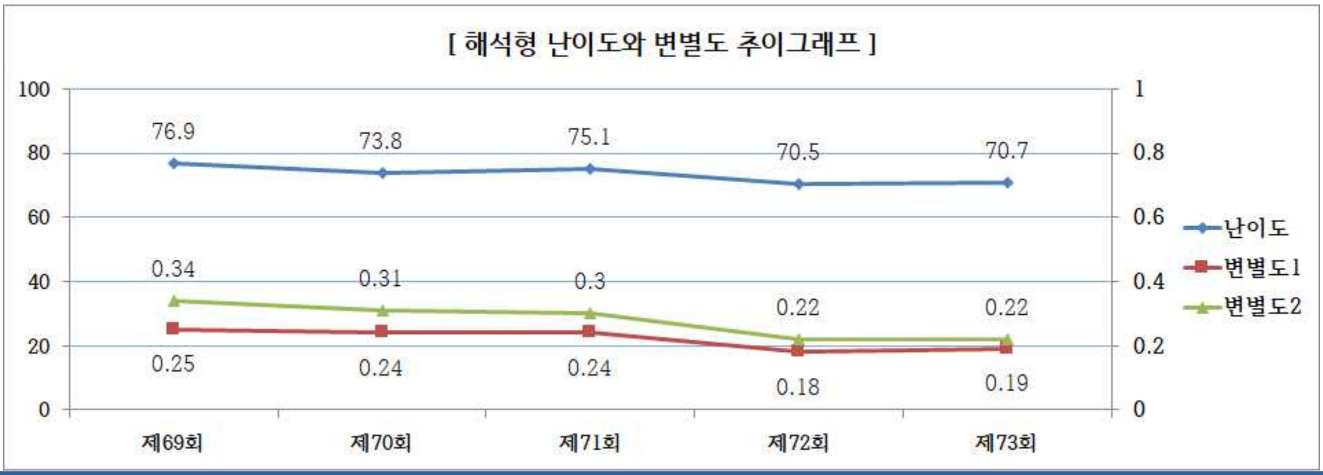


| 회차 | 난이도 | | 변별도1 | | 변별도2 | |
|---|---|---|---|---|---|---|
| | 평균 | 표준편차 | 평균 | 표준편차 | 평균 | 표준편차 |
| 제69회 | 76.9 | 18.6 | .25 | .11 | .34 | .13 |
| 제70회 | 73.8 | 19.8 | .24 | .13 | .31 | .15 |
| 제71회 | 75.1 | 20.6 | .24 | .13 | .30 | .12 |
| 제72회 | 70.5 | 23.6 | .18 | .11 | .22 | .11 |
| **제73회** | **70.7** | **23.5** | **.19** | **.11** | **.22** | **.12** |

**해석**

- 전회 대비 해석형 문항의 난이도 지수는 0.2 증가함
- 전회 대비 해석형 문항의 변별도 1 지수는 .01 증가함
- 전회 대비 해석형 문항의 변별도 2 지수는 동일함

(3) 전회 대비 해결형 난이도와 변별도

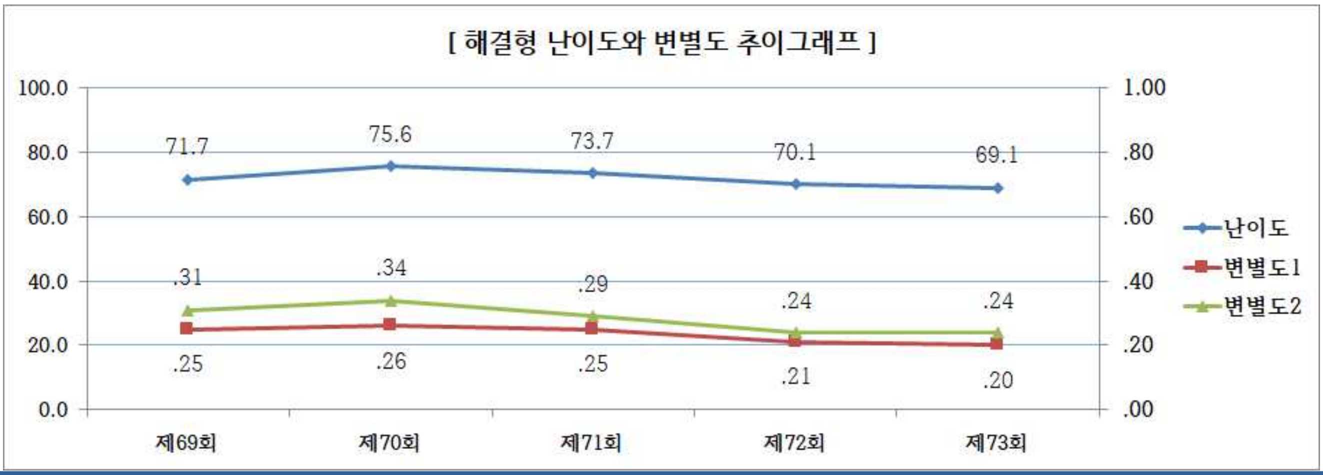


| 회차 | 난이도 | | 변별도1 | | 변별도2 | |
|---|---|---|---|---|---|---|
| | 평균 | 표준편차 | 평균 | 표준편차 | 평균 | 표준편차 |
| 제69회 | 71.7 | 20.2 | .25 | .11 | .31 | .13 |
| 제70회 | 75.6 | 18.6 | .26 | .13 | .34 | .14 |
| 제71회 | 73.7 | 18.6 | .25 | .13 | .29 | .14 |
| 제72회 | 70.1 | 20.9 | .21 | .11 | .24 | .11 |
| **제73회** | **69.1** | **23.4** | **.20** | **.11** | **.24** | **.11** |

**해석**

- 전회 대비 해결형 문항의 난이도 지수는 1.0 감소함
- 전회 대비 해결형 문항의 변별도 1 지수는 .01 감소함
- 전회 대비 해결형 문항의 변별도 2 지수는 동일함

## 나) 지식수준별 난이도와 변별도 분포도 및 비율분석

### (1) 암기형 난이도와 변별도 분포도 및 비율분석

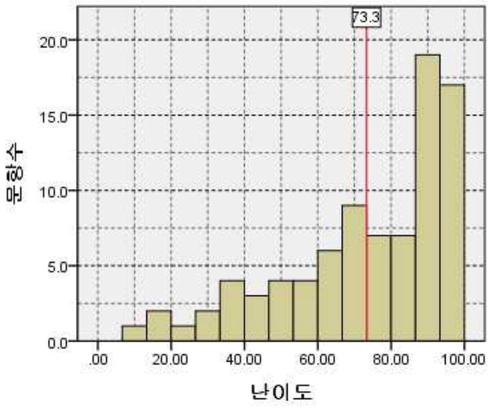


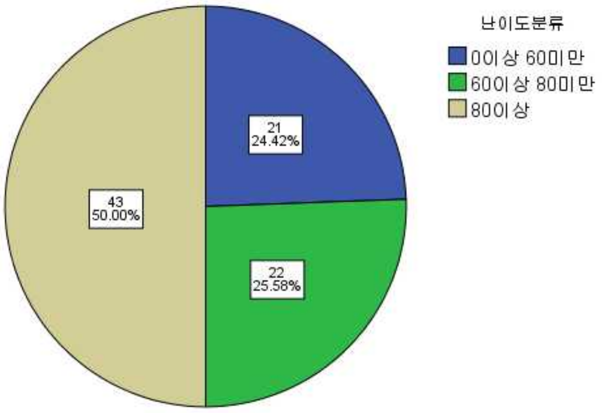


| 총점 | 난이도 | 표준편차 |
|---|---|---|
| 86 | 73.3 | 23.1 |

| 난이도 | 문항수 | 비율(%) |
|---|---|---|
| 0~60미만 | 21 | 24.4 |
| 60~80미만 | 22 | 25.6 |
| 80~100 | 43 | 50.0 |
| 전체 | 86 | 100.0 |

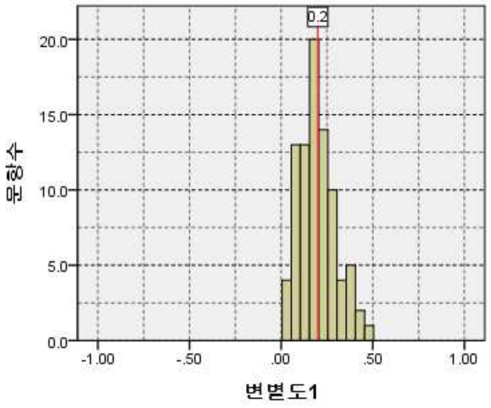


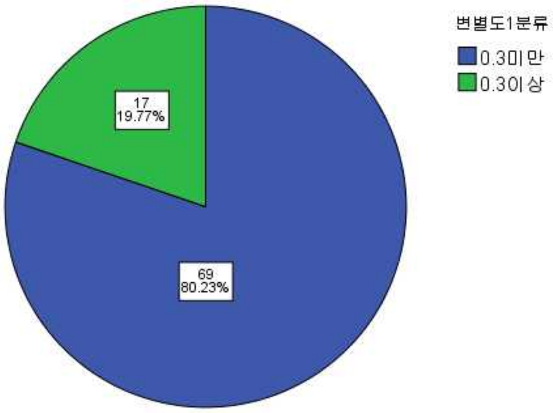


| 총점 | 변별도1 | 표준편차 |
|---|---|---|
| 86 | .20 | .10 |

| 변별도1 | 문항수 | 비율(%) |
|---|---|---|
| 0.3미만 | 69 | 80.2 |
| 0.3이상 | 17 | 19.8 |
| 전체 | 86 | 100.0 |

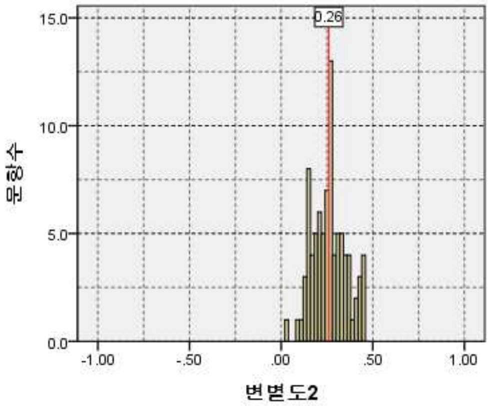


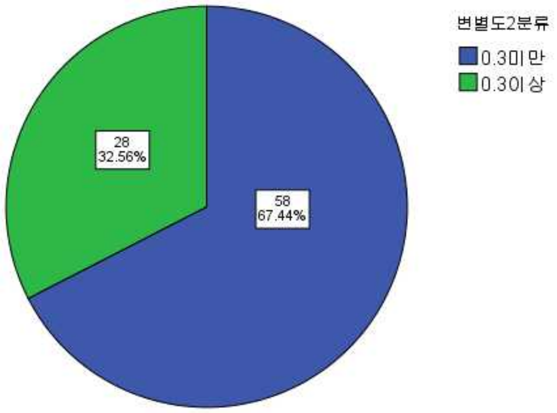


| 총점 | 변별도2 | 표준편차 |
|---|---|---|
| 86 | .26 | .09 |

| 변별도2 | 문항수 | 비율(%) |
|---|---|---|
| 0.3미만 | 58 | 67.4 |
| 0.3이상 | 28 | 32.6 |
| 전체 | 86 | 100.0 |

**해석**

- 암기형 문항에서 난이도 지수가 80 이상인 문항이 43 문항으로 가장 많았으며, 60 이상 80 미만인 문항이 22 문항, 60 미만인 문항이 21 문항으로 나타남
- 변별도 1 지수를 기준으로 분류하였을 때, 0.3 미만인 문항이 69 문항으로 0.3 이상인 문항이 17 문항인 것에 비해 더 많이 나타남
- 변별도 2 지수를 기준으로 분류하였을 때, 0.3 미만인 문항이 58 문항으로 0.3 이상인 문항이 28 문항인 것에 비해 더 많이 나타남

(2) 해석형 난이도와 변별도 분포도 및 비율분석

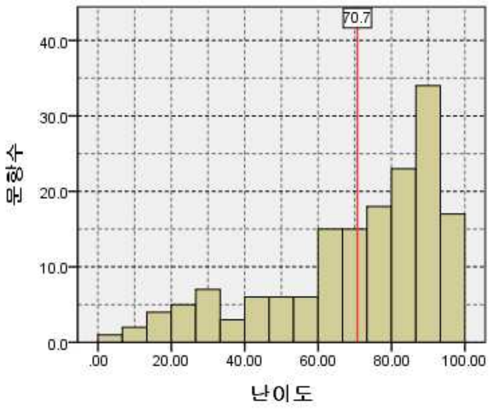


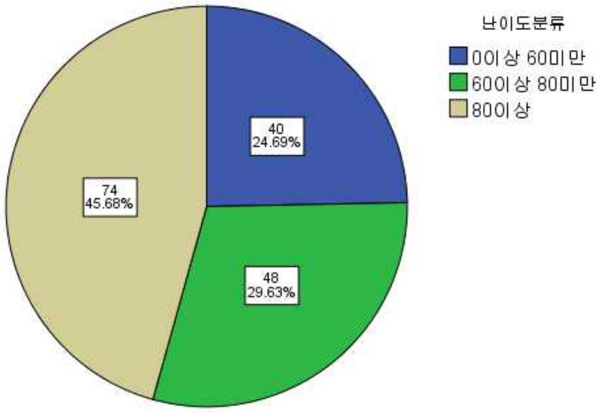


| 총점 | 난이도 | 표준편차 | 난이도 | 문항수 | 비율(%) |
|---|---|---|---|---|---|
| 162 | 70.7 | 23.5 | 0~60미만 | 40 | 24.7 |
| | | | 60~80미만 | 48 | 29.6 |
| | | | 80~100 | 74 | 45.7 |
| | | | 전체 | 162 | 100.0 |

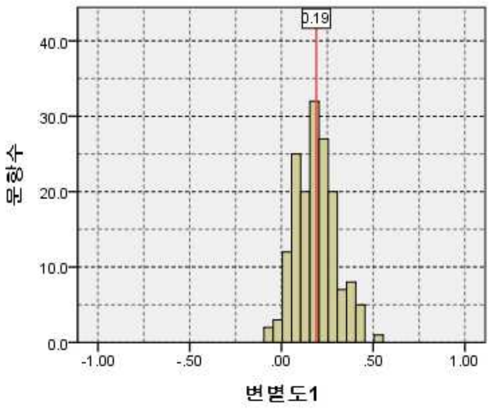


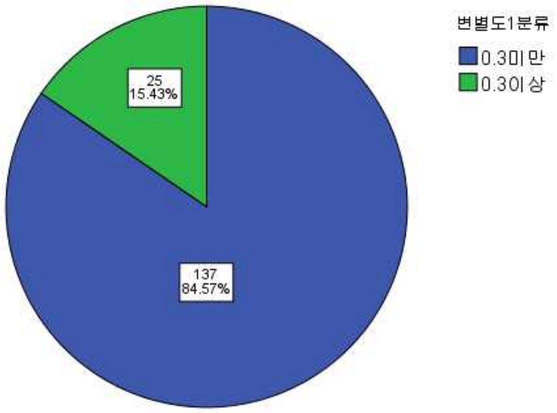


| 총점 | 변별도1 | 표준편차 | 변별도1 | 문항수 | 비율(%) |
|---|---|---|---|---|---|
| 162 | .19 | .11 | 0.3미만 | 137 | 84.6 |
| | | | 0.3이상 | 25 | 15.4 |
| | | | 전체 | 162 | 100.0 |

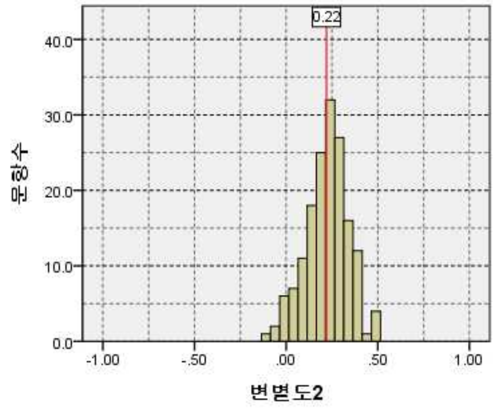


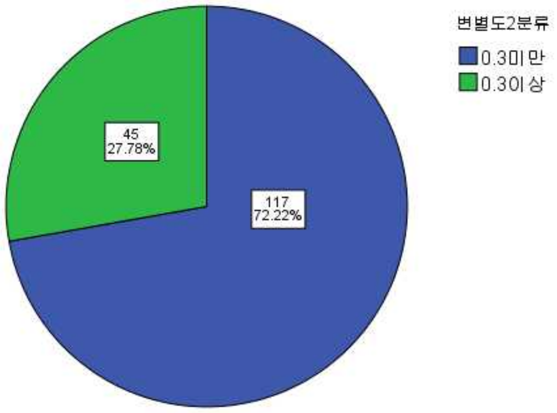


| 총점 | 변별도2 | 표준편차 |
|---|---|---|
| 162 | .22 | .12 |

| 변별도2 | 문항수 | 비율(%) |
|---|---|---|
| 0.3미만 | 117 | 72.2 |
| 0.3이상 | 45 | 27.8 |
| 전체 | 162 | 100.0 |

**해석**

- 해석형 문항에서 난이도 지수가 80 이상인 문항이 74 문항으로 가장 많았으며, 60 이상 80 미만인 문항이 48 문항, 60 미만인 문항이 40 문항으로 나타남
- 변별도 1 지수를 기준으로 분류하였을 때, 0.3 미만인 문항이 137 문항으로 0.3 이상인 문항이 25 문항인 것에 비해 더 많이 나타남
- 변별도 2 지수를 기준으로 분류하였을 때, 0.3 미만인 문항이 117 문항으로 0.3 이상인 문항이 45 문항인 것에 비해 더 많이 나타남

(3) 해결형 난이도와 변별도 분포도 및 비율분석

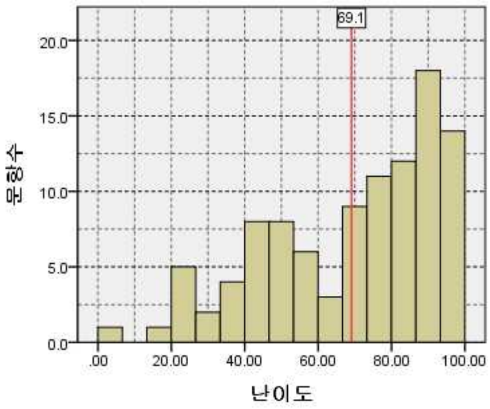


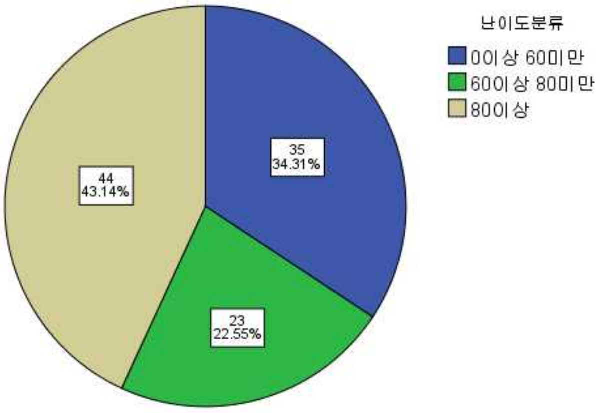


| 총점 | 난이도 | 표준편차 | 난이도 | 문항수 | 비율(%) |
|---|---|---|---|---|---|
| 102 | 69.1 | 23.4 | 0~60미만 | 35 | 34.3 |
| | | | 60~80미만 | 23 | 22.5 |
| | | | 80~100 | 44 | 43.1 |
| | | | 전체 | 102 | 100.0 |

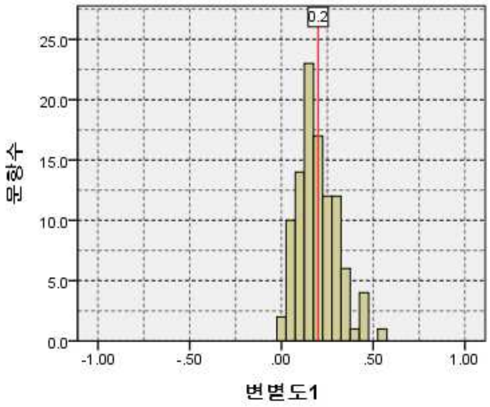


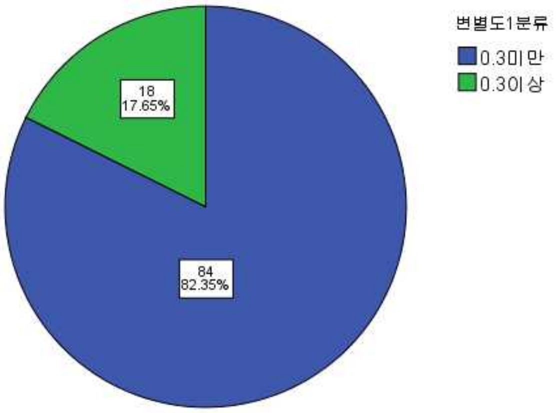


| 총점 | 변별도1 | 표준편차 | 변별도1 | 문항수 | 비율(%) |
|---|---|---|---|---|---|
| 102 | .20 | .11 | 0.3미만 | 84 | 82.4 |
| | | | 0.3이상 | 18 | 17.6 |
| | | | 전체 | 102 | 100.0 |

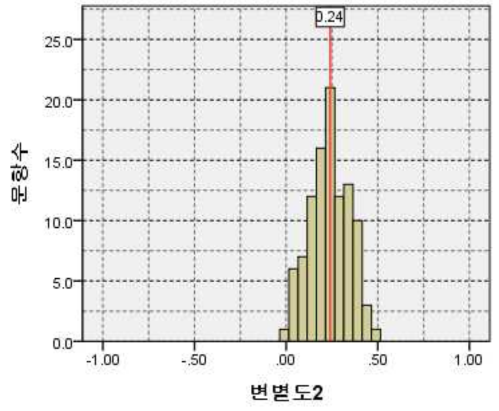


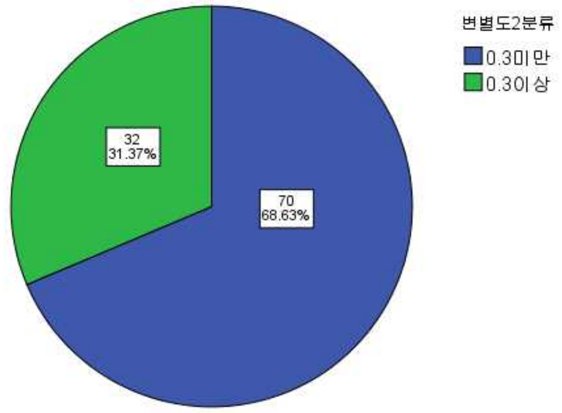


| 총점 | 변별도2 | 표준편차 |
|---|---|---|
| 102 | .24 | .11 |

| 변별도2 | 문항수 | 비율(%) |
|---|---|---|
| 0.3미만 | 70 | 68.6 |
| 0.3이상 | 32 | 31.4 |
| 전체 | 102 | 100.0 |

**해석**

- 해결형 문항에서 난이도 지수가 80 이상인 문항이 44 문항으로 가장 많았으며, 60 미만인 문항이 35 문항, 60 이상 80 미만인 문항이 23 문항으로 나타남
- 변별도 1 지수를 기준으로 분류하였을 때, 0.3 미만인 문항이 84 문항으로 0.3 이상인 문항이 18 문항인 것에 비해 더 많이 나타남
- 변별도 2 지수를 기준으로 분류하였을 때, 0.3 미만인 문항이 70 문항으로 0.3 이상인 문항이 32 문항인 것에 비해 더 많이 나타남

## 4) 문항형태별 난이도와 변별도

### 가) 전회 대비 문항형태별 난이도와 변별도

#### (1) 전회 대비 A형 난이도와 변별도

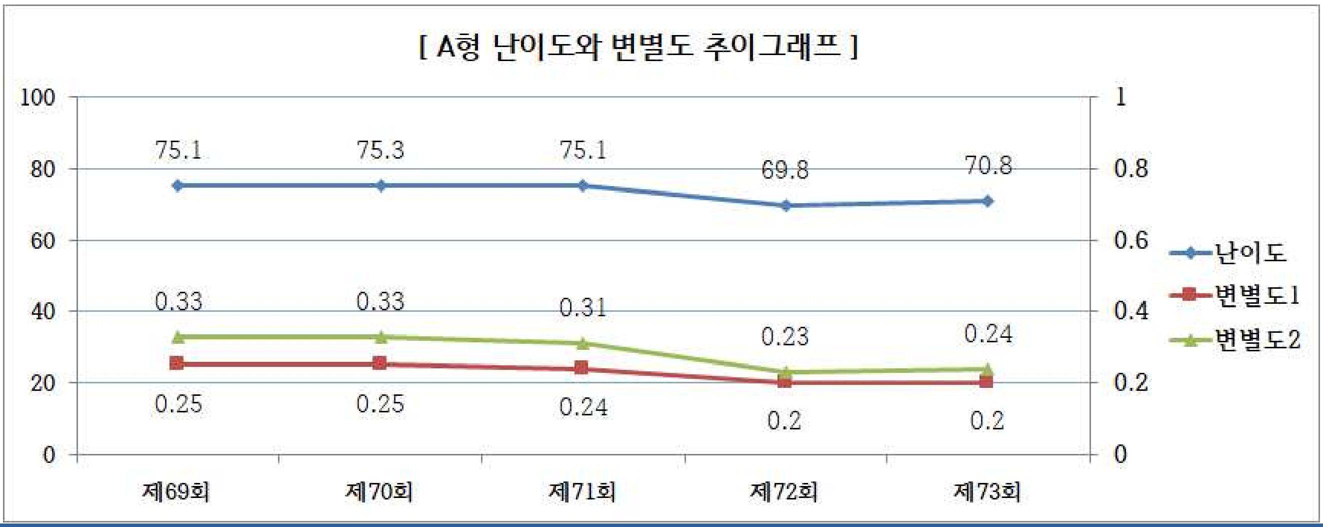


| 회차 | 난이도 | | 변별도1 | | 변별도2 | |
|---|---|---|---|---|---|---|
| | 평균 | 표준편차 | 평균 | 표준편차 | 평균 | 표준편차 |
| 제69회 | 75.1 | 19.2 | .25 | .11 | .33 | .13 |
| 제70회 | 75.3 | 19.7 | .25 | .13 | .33 | .15 |
| 제71회 | 75.1 | 20.5 | .24 | .12 | .31 | .12 |
| 제72회 | 69.8 | 23.1 | .20 | .12 | .23 | .11 |
| **제73회** | **70.8** | **23.0** | **.20** | **.11** | **.24** | **.11** |

**해석**

- 전회 대비 A 형 문항의 난이도 지수는 1.0 증가함
- 전회 대비 A 형 문항의 변별도 1 지수는 동일함
- 전회 대비 A 형 문항의 변별도 2 지수는 .01 증가함

(2) 전회 대비 사례형 난이도와 변별도

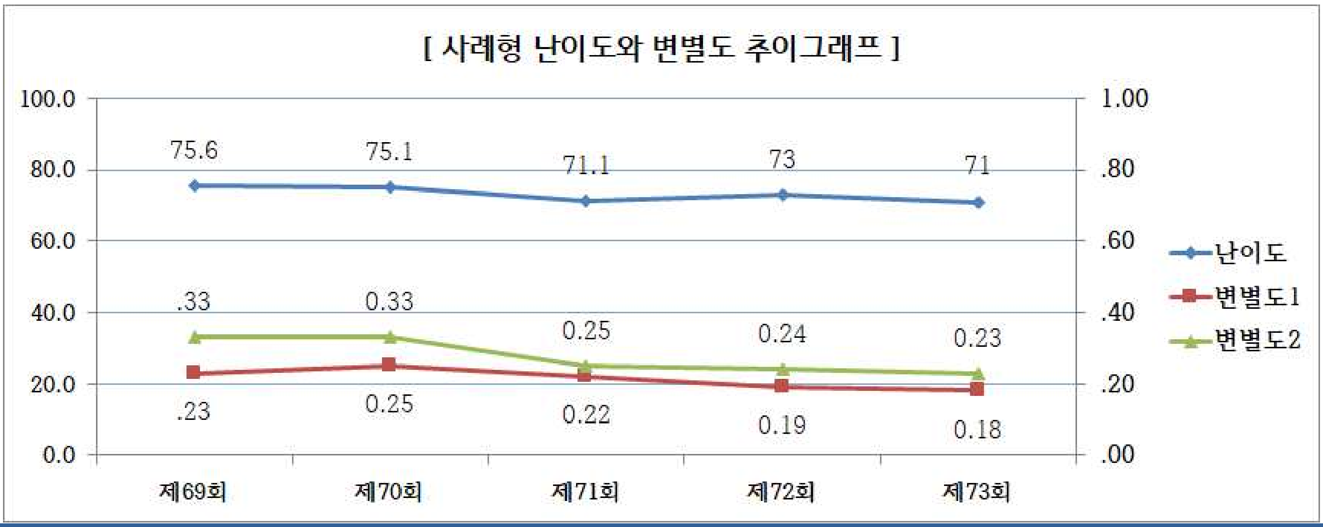


| 회차 | 난이도 | | 변별도1 | | 변별도2 | |
|---|---|---|---|---|---|---|
| | 평균 | 표준편차 | 평균 | 표준편차 | 평균 | 표준편차 |
| 제69회 | 75.6 | 19.9 | .23 | .11 | .33 | .14 |
| 제70회 | 75.1 | 19.1 | .25 | .12 | .33 | .14 |
| 제71회 | 71.1 | 21.3 | .22 | .14 | .25 | .15 |
| 제72회 | 73.0 | 20.2 | .19 | .10 | .24 | .10 |
| **제73회** | **71.0** | **25.3** | **.18** | **.11** | **.23** | **.10** |

**해석**

- 전회 대비 사례형 문항의 난이도 지수는 2.0 감소함
- 전회 대비 사례형 문항의 변별도 1 지수는 .01 감소함
- 전회 대비 사례형 문항의 변별도 2 지수는 .01 감소함

## 나) 문항형태별 난이도와 변별도 분포도 및 비율분석

### (1) A형 난이도와 변별도 분포분석

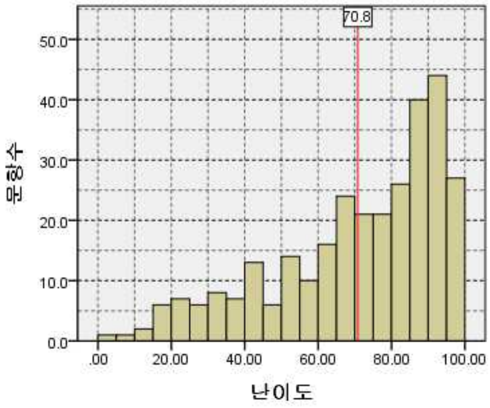


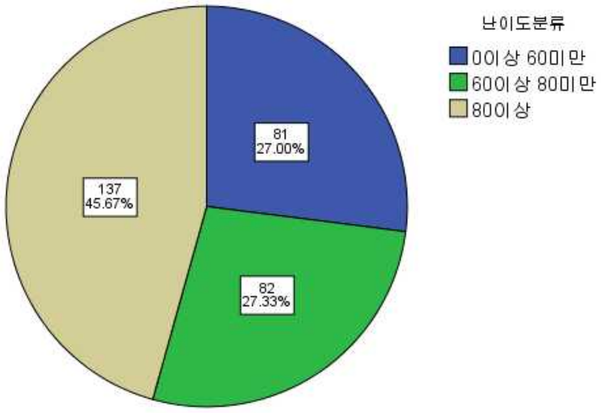


| 총점 | 난이도 | 표준편차 |
|---|---|---|
| 300 | 70.8 | 23.0 |

| 난이도 | 문항수 | 비율(%) |
|---|---|---|
| 0~60미만 | 81 | 27.0 |
| 60~80미만 | 82 | 27.3 |
| 80~100 | 137 | 45.7 |
| 전체 | 300 | 100.0 |

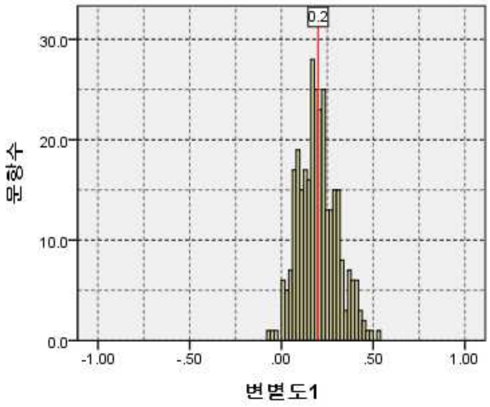


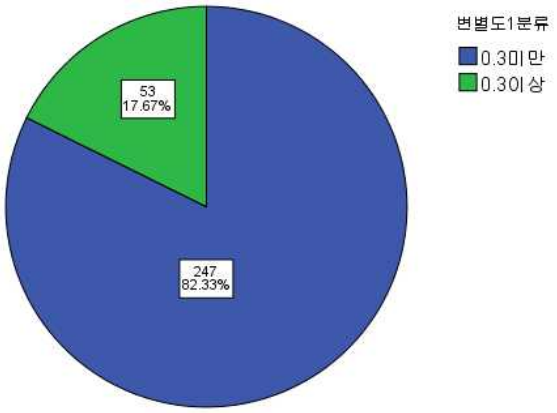


| 총점 | 변별도1 | 표준편차 |
|---|---|---|
| 300 | .20 | .11 |

| 변별도1 | 문항수 | 비율(%) |
|---|---|---|
| 0.3미만 | 247 | 82.3 |
| 0.3이상 | 53 | 17.7 |
| 전체 | 300 | 100.0 |

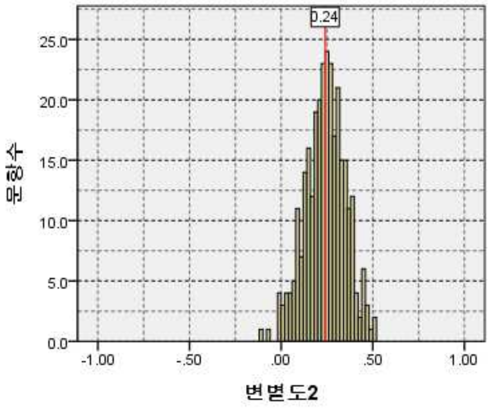


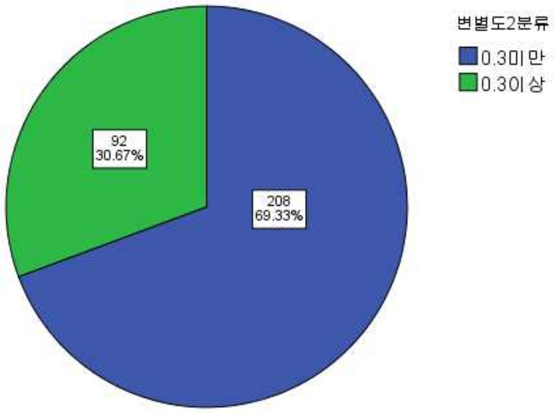


| 총점 | 변별도2 | 표준편차 |
|---|---|---|
| 300 | .24 | .11 |

| 변별도2 | 문항수 | 비율(%) |
|---|---|---|
| 0.3미만 | 208 | 69.3 |
| 0.3이상 | 92 | 30.7 |
| 전체 | 300 | 100.0 |

**해석**

- A형 문항에서 난이도 지수가 80이상인 문항이 137문항으로 가장 많았으며, 60이상 80미만인 문항이 82문항, 60미만인 문항이 81문항으로 나타남
- 변별도1 지수를 기준으로 분류하였을 때, 0.3미만인 문항이 247문항으로 0.3이상인 문항이 53문항인 것에 비해 더 많이 나타남
- 변별도2 지수를 기준으로 분류하였을 때, 0.3미만인 문항이 208문항으로 0.3이상인 문항이 92문항인 것에 비해 더 많이 나타남

(2) 사례형 난이도와 변별도 분포도 및 비율분석

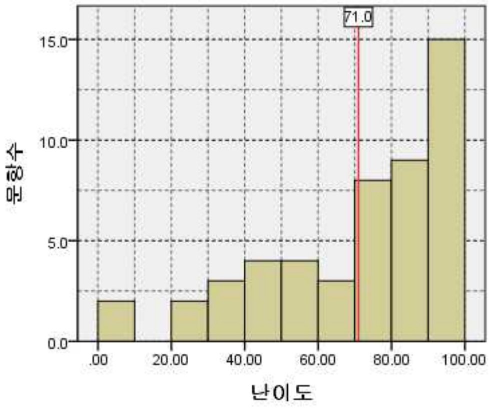


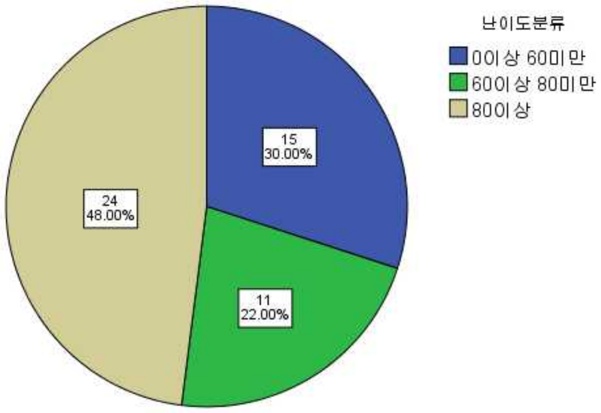


| 총점 | 난이도 | 표준편차 |
|---|---|---|
| 50 | 71.0 | 25.3 |

| 난이도 | 문항수 | 비율(%) |
|---|---|---|
| 0~60미만 | 15 | 30.0 |
| 60~80미만 | 11 | 22.0 |
| 80~100 | 24 | 48.0 |
| 전체 | 50 | 100.0 |

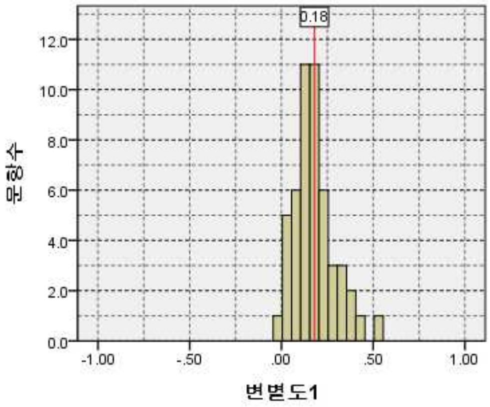


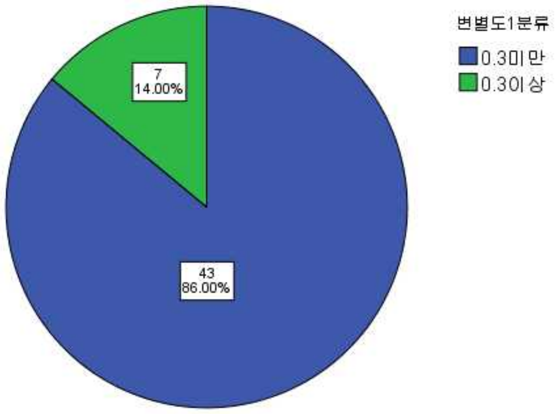


| 총점 | 변별도1 | 표준편차 |
|---|---|---|
| 50 | .18 | .11 |

| 변별도1 | 문항수 | 비율(%) |
|---|---|---|
| 0.3미만 | 43 | 86.0 |
| 0.3이상 | 7 | 14.0 |
| 전체 | 50 | 100.0 |

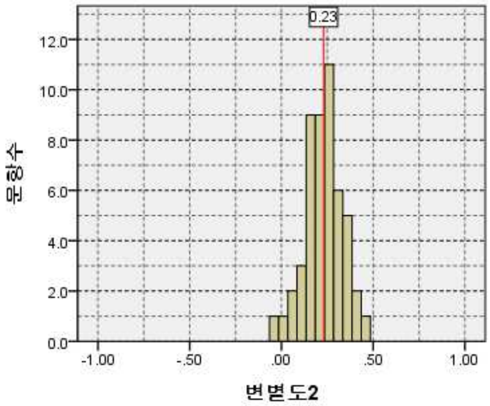


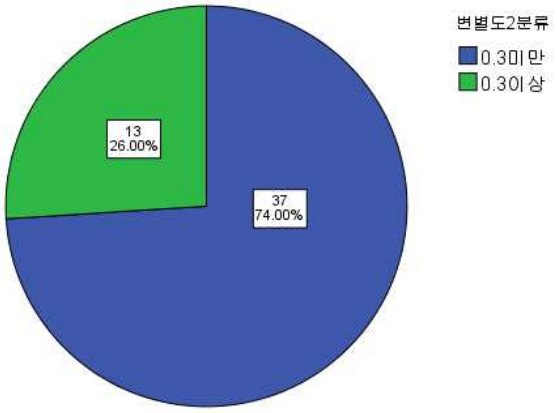


| 총점 | 변별도2 | 표준편차 |
|---|---|---|
| 50 | .23 | .10 |

| 변별도2 | 문항수 | 비율(%) |
|---|---|---|
| 0.3미만 | 37 | 74.0 |
| 0.3이상 | 13 | 26.0 |
| 전체 | 50 | 100.0 |

**해석**

- 사례형 문항에서 난이도 지수가 80 이상인 문항이 24 문항으로 가장 많았으며, 60 미만인 문항이 15 문항, 60 이상 80 미만인 문항이 11 문항으로 나타남
- 변별도 1 지수를 기준으로 분류하였을 때, 0.3 미만인 문항이 43 문항으로 0.3 이상인 문항이 7 문항인 것에 비해 더 많이 나타남
- 변별도 2 지수를 기준으로 분류하였을 때, 0.3 미만인 문항이 37 문항으로 0.3 이상인 문항이 13 문항인 것에 비해 더 많이 나타남

## 3. 난이도와 변별도 간 산포도

1) 전체 난이도와 변별도 간 산포도

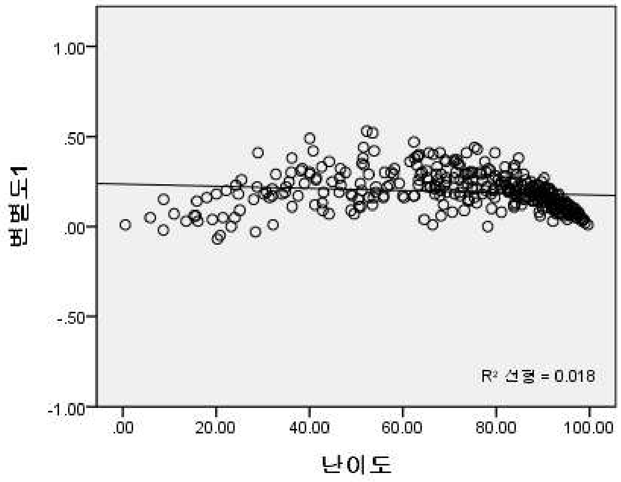


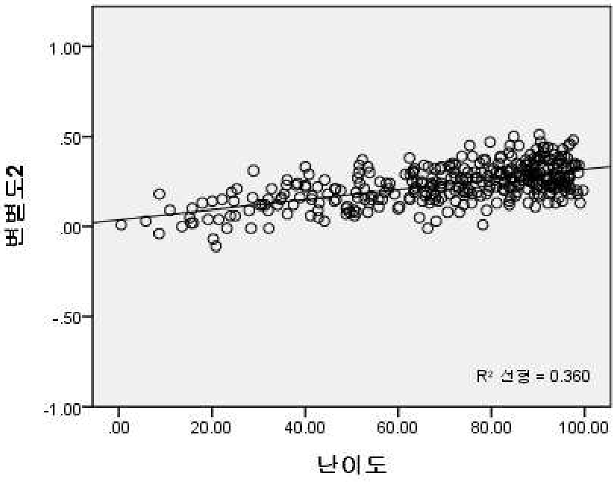


**해석**

- 난이도 지수와 변별도1 지수 간 상관은 -.133**로 난이도 지수가 높을수록 변별력이 낮아지는 것으로 나타남
- 난이도 지수와 변별도2 지수 간 상관은 .600**로 문항 난이도 지수가 높을수록 변별력이 높아지는 것으로 나타남

2) 과목별 난이도와 변별도 간 산포도

가) 생명약학 난이도와 변별도 간 산포도

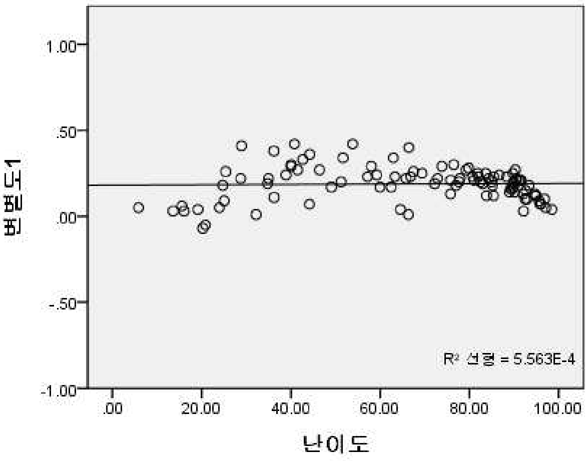


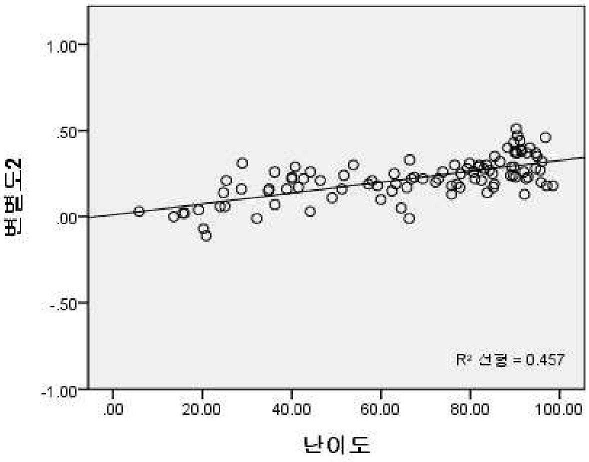


**해석**

- 난이도 지수와 변별도 1 지수 간 상관은 -.024 으로 문항 난이도 지수와 변별력 간 관련성이 없는 것으로 나타남
- 난이도 지수와 변별도 2 지수 간 상관은 .676**로 문항 난이도 지수가 높을수록 변별력이 높아지는 것으로 나타남

나) 산업약학 난이도와 변별도 간 산포도

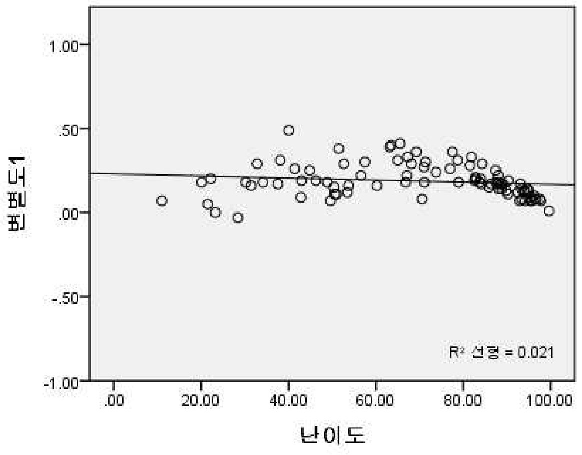


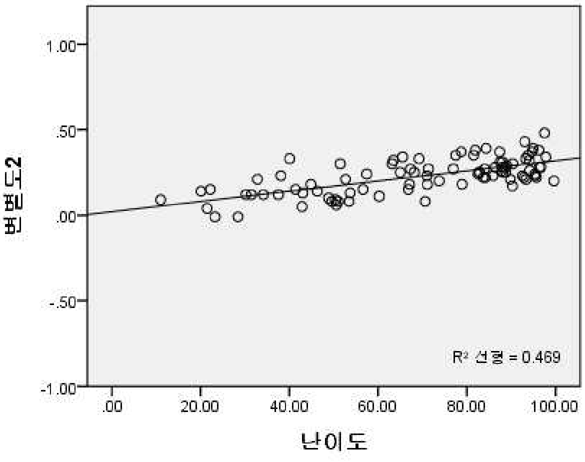


**해석**

- 난이도 지수와 변별도 1 지수 간 상관은 -.145 로 문항 난이도 지수와 변별력 간 관련성이 없는 것으로 나타남
- 난이도 지수와 변별도 2 지수 간 상관은 .685**으로 문항 난이도 지수가 높을수록 변별력이 높아지는 것으로 나타남

다) 임상·실무약학 난이도와 변별도 간 산포도

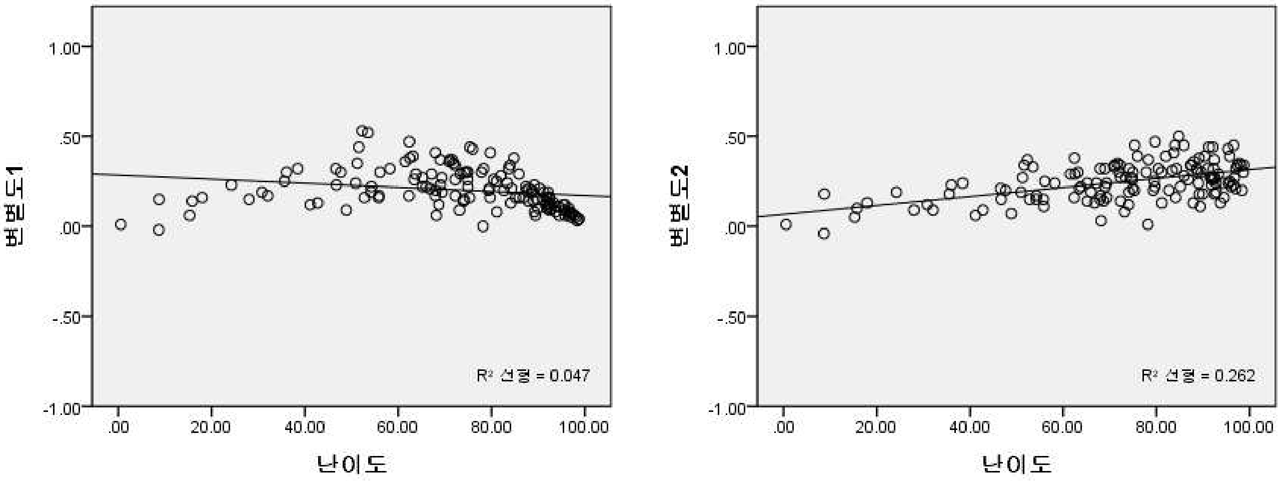


| 해석 |
|---|
| - 난이도 지수와 변별도 1 지수 간 상관은 -.216**로 문항 난이도 지수가 높을수록 변별력이 낮아지는 것으로 나타남<br>- 난이도 지수와 변별도 2 지수 간 상관은 .511**로 문항 난이도 지수가 높을수록 변별력이 높아지는 것으로 나타남 |

라) 보건·의약관계법규 난이도와 변별도 간 산포도

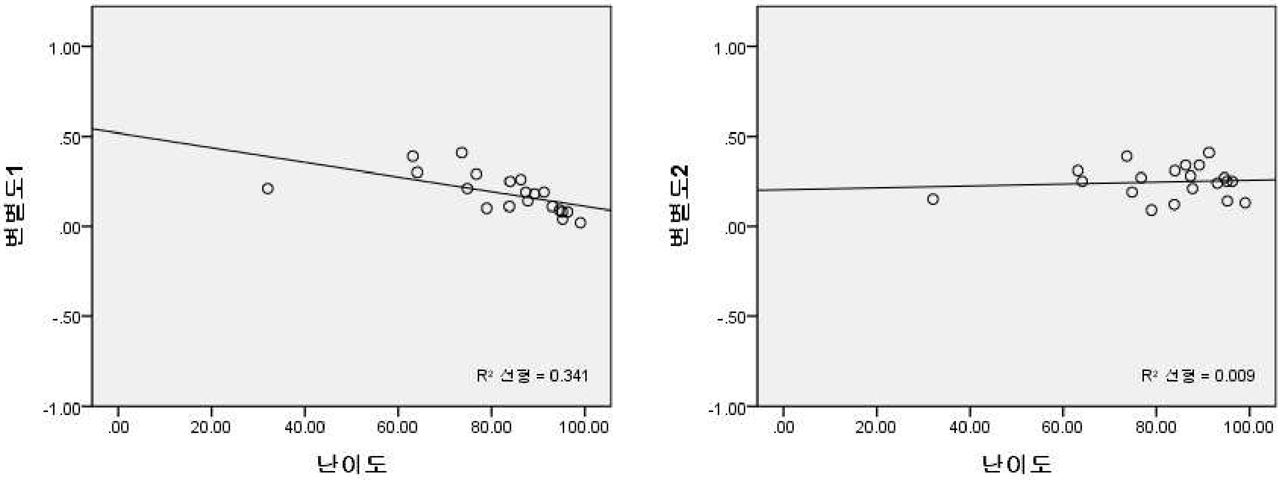


| 해석 |
|---|
| - 난이도 지수와 변별도 1 지수 간 상관은 -584**로 문항 난이도 지수가 높을수록 변별력이 낮아지는 것으로 나타남<br>- 난이도 지수와 변별도 2 지수 간 상관은 .093 으로 문항 난이도 지수와 변별력 간 관련성이 없는 것으로 나타남 |

## 4. 신뢰도 분석

| 과목명 | 문항수 | 제69회 | 제70회 | 제71회 | 제72회 | 제73회 |
|---|---|---|---|---|---|---|
| 전체 | 350 | .973 | .973 | .967 | .946 | **.948** |
| 생명약학 | 100 | .922 | .930 | .911 | .850 | **.831** |
| 산업약학 | 90 | .913 | .913 | .898 | .796 | **.819** |
| 임상·실무약학 | 140 | .930 | .929 | .916 | .896 | **.896** |
| 보건·의약관계법규 | 20 | .672 | .670 | .651 | .500 | **.621** |

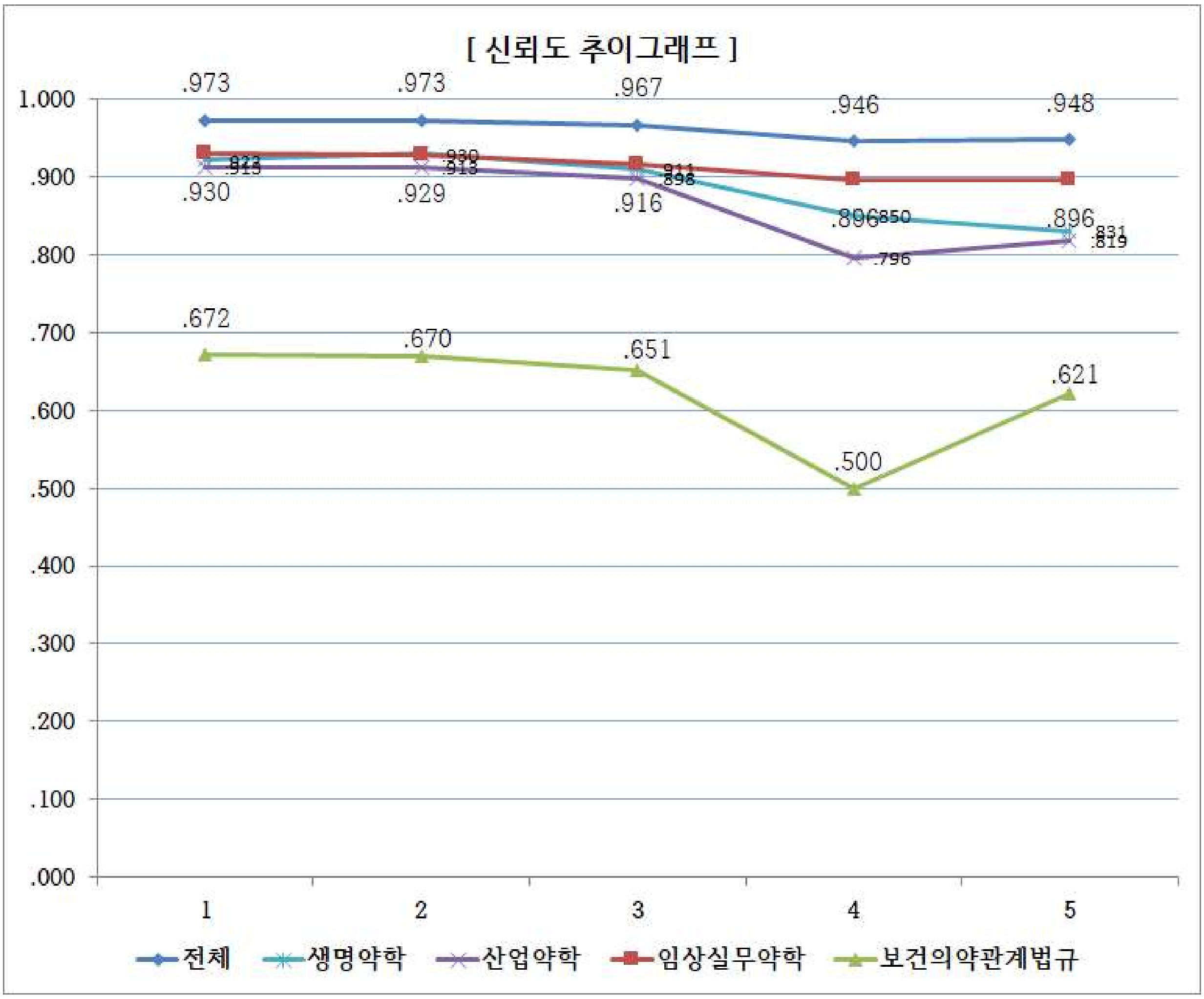


**해석**

- 전회 대비 전체 문항의 신뢰도는 .002 증가함
- 전회 대비 생명약학 과목 문항의 신뢰도는 .019 감소함
- 전회 대비 산업약학 과목 문항의 신뢰도는 .023 증가함
- 전회 대비 임상·실무약학 과목 문항의 신뢰도는 동일함
- 전회 대비 보건·의약관계법규 과목 문항의 신뢰도는 .121 증가함

○ 분석결과 관련 문의 : 한국보건의료인국가시험원 연구개발본부 김보현 전임연구원
Tel : 02-2087-8954, FAX : 02-2087-8885
E-mail : kimbohyun@kuksiwon.or.kr
