# Supplementary material for: Item difficulty index, discrimination index, and reliability of the 26 health professions licensing examinations in 2022, Korea: a psychometric study
Source: J Educ Eval Health Prof. 2023 Nov 22;20:31. doi: 10.3352/jeehp.2023.20.31 (PMC11959405; doi:10.3352/jeehp.2023.20.31)
Supplement: Supplementary file 1 — Supplement 1. Item analysis results of 26 health professions licensing examinations administered during late 2022 and early 2023. [file jeehp-20-31_Suppl1.zip › 2022│Γ╡╡ ┴a74╚╕ ─í░·└╟╗τ ▒╣░í╜├╟Φ ║╨╝«░ß░·.pdf]

2022년도 제74회 치과의사 국가시험 문항분석 결과

일반 용어 정의

□ **평균**

- 집단에서의 대표적 경향값으로 전체 값을 더하여 총 응시자로 나눈 값

□ **표준편차**

- 평균과 각 점수의 차이인 편차들의 평균으로 점수가 흩어져 분포되어 있는 정도

□ **추정난이도**

- 문항개발자가 예측한 정답률

□ **검사이론**

- 검사와 검사를 구성하고 있는 문항의 양호도를 분석 및 평가하는 방법을 정의한 이론체계
- 대표적으로 고전검사이론과 문항반응이론이 있음

고전검사이론 용어 정의

□ **고전검사이론(Classical Test Theory; CTT)**

- 검사의 질을 분석하는 검사이론 중 한 가지로 19세기 말부터 전개되어 현재까지 주로 사용되고 있는 검사이론임
- 고전검사이론에 의한 문항과 응시자 능력 추정치는 다음과 같음

○ **문항난이도**

· 검사 문항의 쉽고 어려운 정도를 나타내는 지수
· 난이도 지수는 총 반응 수에 대한 정답 반응 수의 비율로 문항의 정답률임
· 문항난이도는 0~100까지의 값을 가짐
· 난이도 값이 큰 경우, 쉬운 문항으로 '난이도가 낮다'라고 해석하며, 난이도 값이 작은 경우, 어려운 문항으로 '난이도가 높다'라고 해석함

○ **문항변별도**

· 각 문항이 응시자의 능력 수준을 변별할 수 있는 정도를 나타내는 지수
· 문항변별도는 -1~+1까지의 값을 가지며, 1에 가까울수록 변별력 크다고 해석함
· 일반적으로 문항변별도가 0.3 이상이면 우수한 문항으로 평가함
· 구하는 방식에는 '상하위집단 구분법', '문항-총점 상관계수' 등이 있음
  1) **변별도 1(상하위구분법):** 상위 27%와 하위 27% 집단의 난이도 차이를 구하는 방식
  2) **변별도 2(상관계수법):** 문항-총점과의 상관계수로 구하는 방식

○ **신뢰도**

· 시험이 평가하고자 하는 것을 일관성 있게 측정하는가로 시험이 오차없이 정확하게 측정한 정도를 의미함
· 국시원에서는 문항의 내적일관성(Cronbach α)으로 신뢰도를 추정하며 1에 가까울수록 신뢰도가 높다고 해석함

목 차

I. 시행 결과 ······ 6
1. 시험 현황 ······ 7
1) 시험명 ······ 7
2) 시험시행일 ······ 7
3) 응시현황 ······ 7
4) 과목별 문항 수, 배점 및 과락 점수 ······ 7
2. 합격률과 평균성적 ······ 8
1) 합격 및 불합격 현황 ······ 8
2) 과목별 과락자수 내역 ······ 8
3) 전회 대비 합격률과 평균성적 ······ 9

II. 문항분석 결과 ······ 10
1. 성적 ······ 11
1) 전체 성적분포도 ······ 11
2) 과목별 성적분포도 ······ 12
2. 난이도와 변별도 ······ 16
1) 전체 난이도와 변별도 ······ 16
2) 과목별 난이도와 변별도 ······ 19
3) 지식수준별 난이도와 변별도 ······ 58
3. 난이도와 변별도 간 산포도 ······ 67
1) 전체 난이도와 변별도 간 산포도 ······ 67
2) 과목별 난이도와 변별도 간 산포도 ······ 67
4. 신뢰도 분석 ······ 75

I. 시행 결과

1. 시험 현황

1) 시험명: 2022년도 제74회 치과의사 국가시험

2) 시험시행일: 2021년 1월 14일

3) 응시현황

응시대상자수	결시자수	부정행위자수	응시자 준수사항 위반자 수		응시자수 (%)
			휴대폰 소지	신분증 미지참	
764	0	0	0	0	762 (99.7)

4) 과목별 문항 수, 배점 및 과락 점수

교 시	과 목 명	문제 수	배점	총점	합격자 점수기준	
					과목 과락기준	총점 합격기준
1교시	구강내과학	15	1	15	세부기준 참조	204점 이상
	치과보철학	40	1	40	16점 미만	
	소아치과학	26	1	26	세부기준 참조	
2교시	치과교정학	33	1	33	세부기준 참조	
	구강병리학	15	1	15	세부기준 참조	
	구강생물학	48	0.5	24	세부기준 참조	
3교시	영상치의학	26	1	26	세부기준 참조	
	치주과학	26	1	26	세부기준 참조	
	구강악안면외과학	40	1	40	16점 미만	
4교시	치과보존학	40	1	40	16점 미만	
	구강보건학	20	1	20	세부기준 참조	
	치과재료학	15	1	15	세부기준 참조	
	보건의약관계 법규	20	1	20	8점 미만	
계		364		340		

* 과락 세부기준

과 목 명	문제 수	배점	총점	합격자 점수기준	
				과목 과락기준	총점 합격기준
구강내과학	15	1	56	22.4점 미만	204점 이상
구강병리학	15	1			
영상치의학	26	1			
치과보철학	40	1	40	16점 미만	
소아치의학	26	1	59	23.6점 미만	
치과교정학	33	1			
구강생물학	48	0.5	39	15.6점 미만	
치과재료학	15	1			
치주과학	26	1	46	18.4점 미만	
구강보건학	20	1			
구강악안면외과학	40	1	40	16점 미만	
치과보존학	40	1	40	16점 미만	
보건의약관계법규	20	1	20	8점 미만	
계	364		340		

※ 치과의사의 경우 구강악안면외과학, 치과보존학, 치과보철학, 보건의약관계법규 과목을 제외한 과목의 경우 개별 합격점수가 아닌 과목통합 합격선이 있음.

2. 합격률과 평균성적

1) 합격 및 불합격 현황

합격자수 (%)	불합격자수(%)				채점보류자수
	평락	과락	기권	계	
745 (94.1)	15 (2.6)	2 (0.1)	0 (0.0)	17 (2.7)	0 (0.0)

2) 과목별 과락자수 내역

과목명 / 과락자수	구강내과학·구강병리학·영상치의학	치과보철학	소아치의학·치과교정학	구강생물학·치과재료학	치주과학·구강보건학	구강악안면외과학	치과보존학	보건의약관계법규
과목별 과락자 수	0	0	0	0	0	0	0	2
전과목 과락자 수	2							

3) 전회 대비 합격률과 평균성적

회차	년도	합격률(%)	평균성적	표준편차	백분율 환산점수
제70회	2018	94.9	250.4	27.1	73.6
제71회	2019	97.3	268.2	27.4	78.9
제72회	2020	97.3	261.1	29.0	76.8
제73회	2021	97.3	266.8	27.8	78.5
제74회	2022	92.3	268.7	27.6	79.0

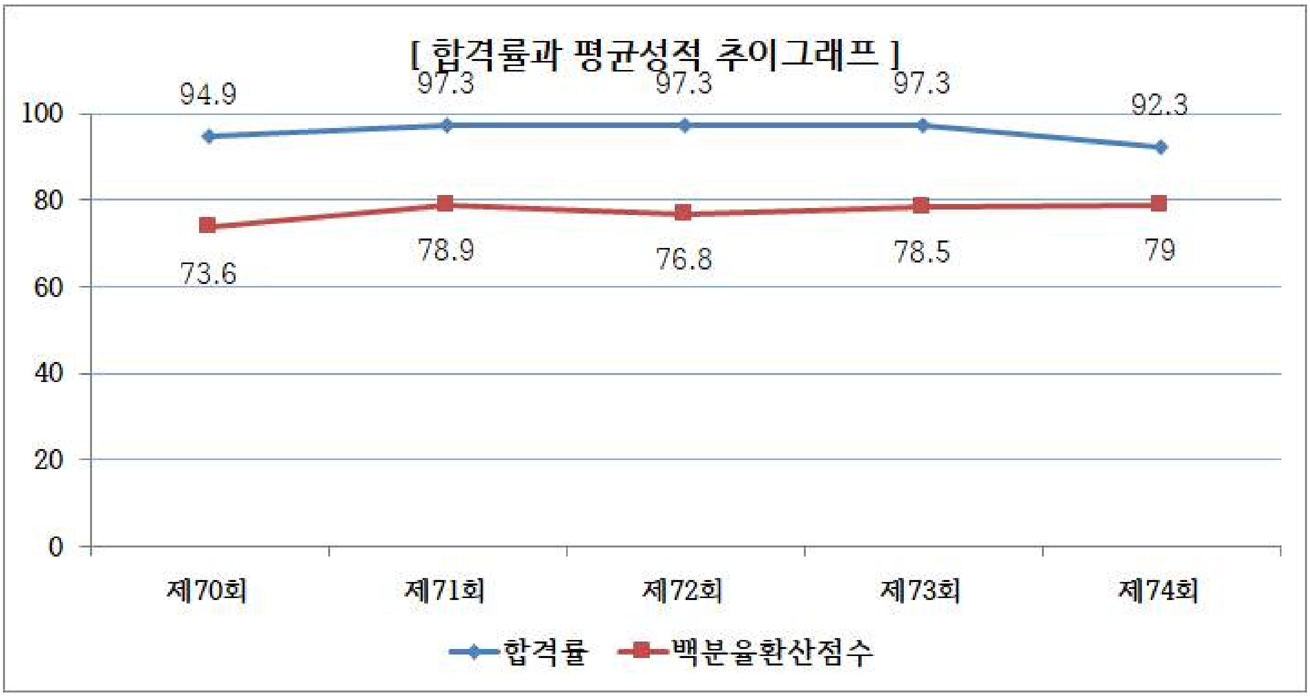

해석

- 전년 대비 합격률은 5.0 감소하였으며, 백분율 환산점수는 0.5 점 증가함

Ⅱ. 문항분석 결과

1. 성적

1) 전체 성적분포도

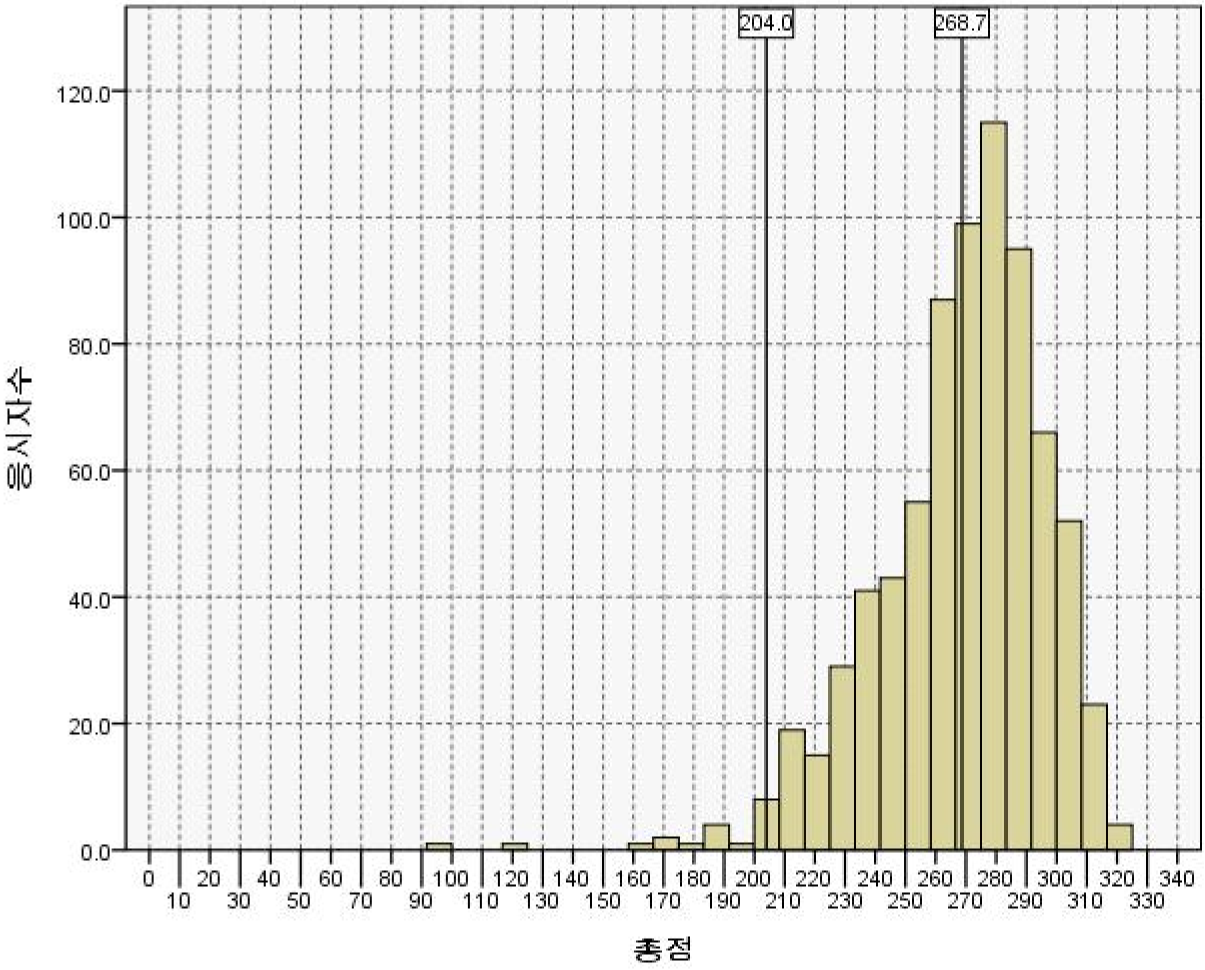

응시자	총점	합격선	평균성적	표준편차
762	340	204	268.7	27.6

2) 과목별 성적분포도

가) 구강악안면외과학

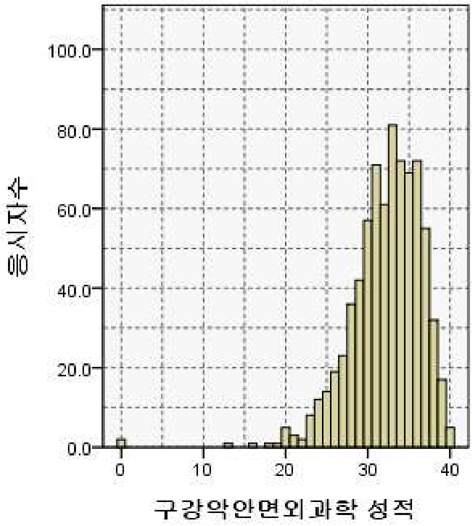

총점	과락선	평균성적	표준편차
40	16	32.2	4.4

나) 치과보존학

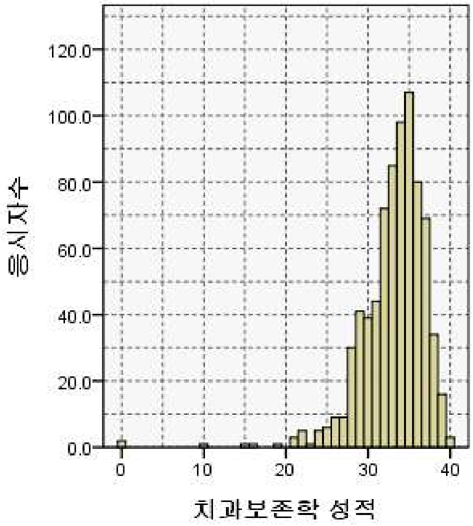

총점	과락선	평균성적	표준편차
40	16	33.1	4.0

다) 치과보철학

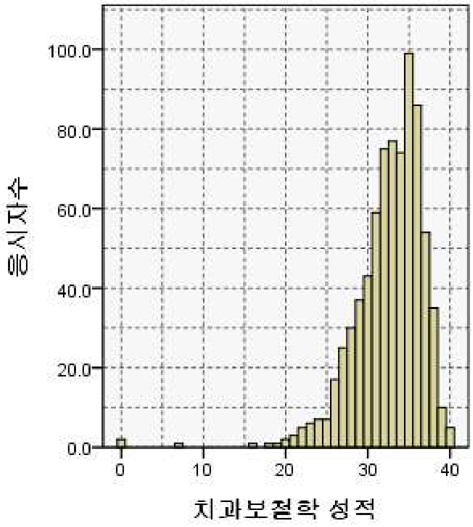

총점	과락선	평균성적	표준편차
40	16	32.6	4.3

라) 소아치과학

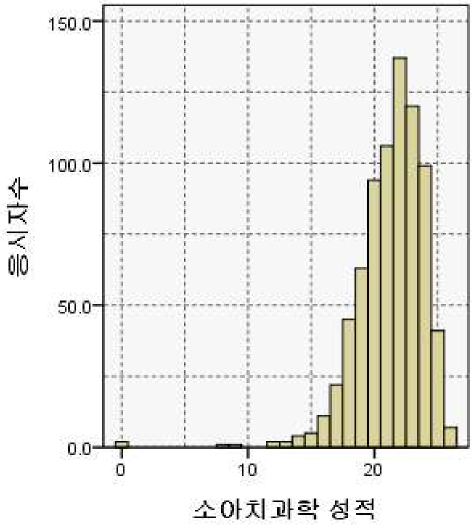

총점	과락선	평균성적	표준편차
26	-	21.3	2.7

마) 영상치의학

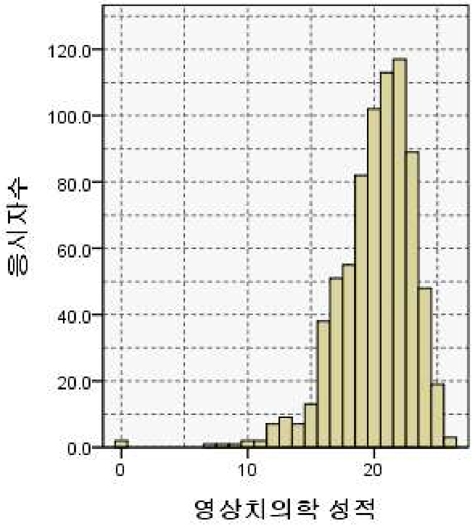

총점	과락선	평균성적	표준편차
26	-	20.1	3.1

바) 치주과학

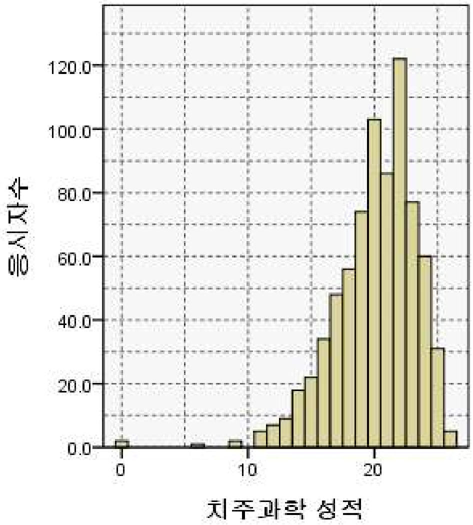

총점	과락선	평균성적	표준편차
26	-	20.1	3.3

사) 구강내과학

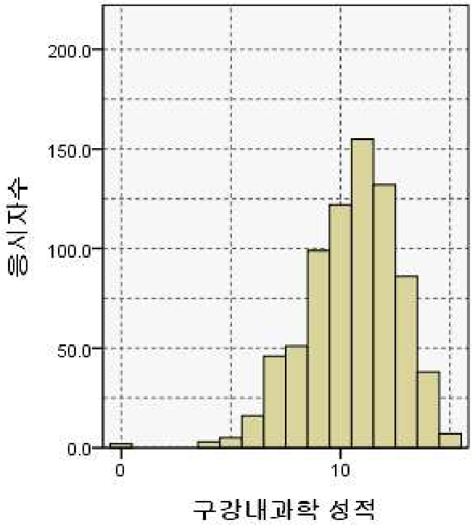

총점	과락선	평균성적	표준편차
15	-	10.5	2.1

아) 치과재료학

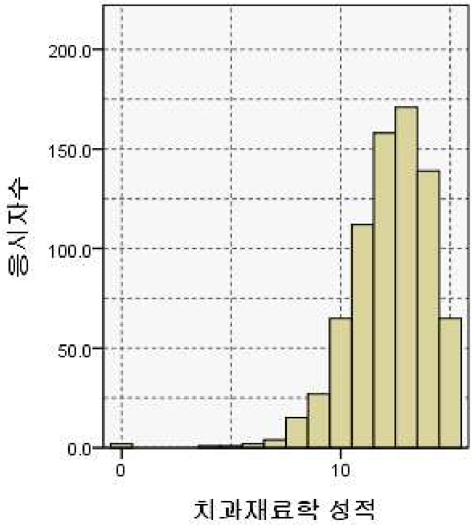

총점	과락선	평균성적	표준편차
15	-	12.3	1.9

자) 치과교정학

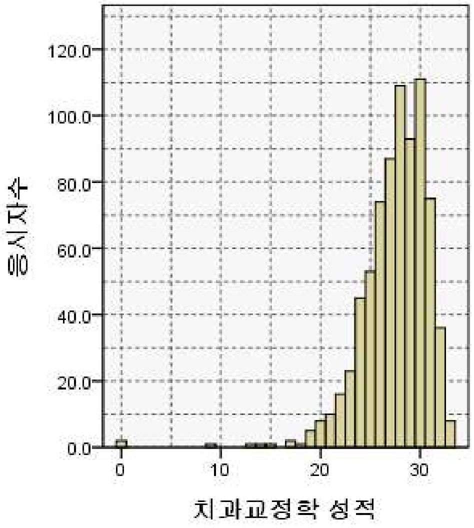

총점	과락선	평균성적	표준편차
33	-	27.5	3.4

차) 구강병리학

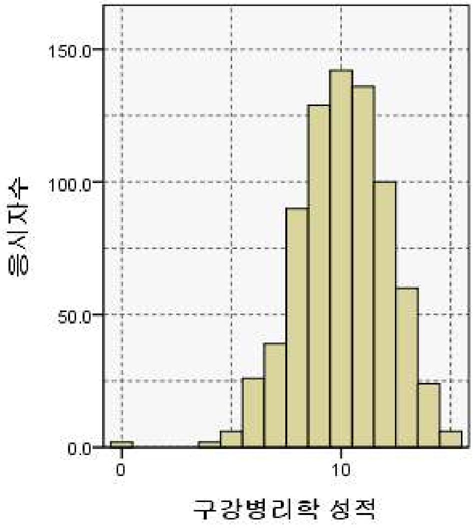

총점	과락선	평균성적	표준편차
15	-	10.1	2.1

카) 구강보건학

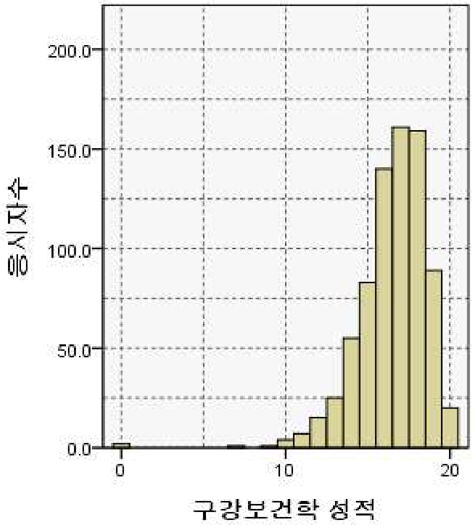

총점	과락선	평균성적	표준편차
20	-	16.5	2.1

타) 구강생물학

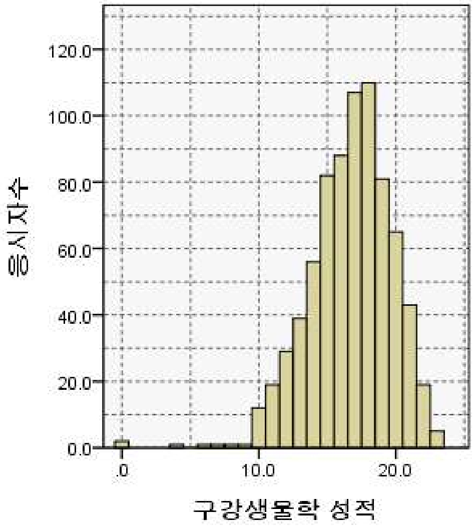

총점	과락선	평균성적	표준편차
24	-	16.4	3.0

파) 보건의약관계법규

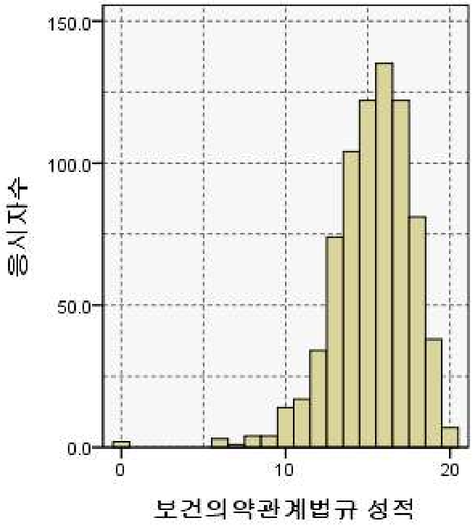

총점	과락선	평균성적	표준편차
20	8	15.3	2.4

2. 난이도와 변별도

1) 전체 난이도와 변별도

가) 전회 대비 전체 난이도와 변별도

회차	난이도		변별도1		변별도2	
	평균	표준편차	평균	표준편차	평균	표준편차
제70회	73.3	22.1	.19	.12	.21	.09
제71회	78.3	19.5	.18	.12	.22	.09
제72회	76.0	20.3	.20	.12	.23	.09
제73회	77.8	20.2	.19	.13	.23	.10
제74회	78.3	19.1	.19	.12	.22	.09

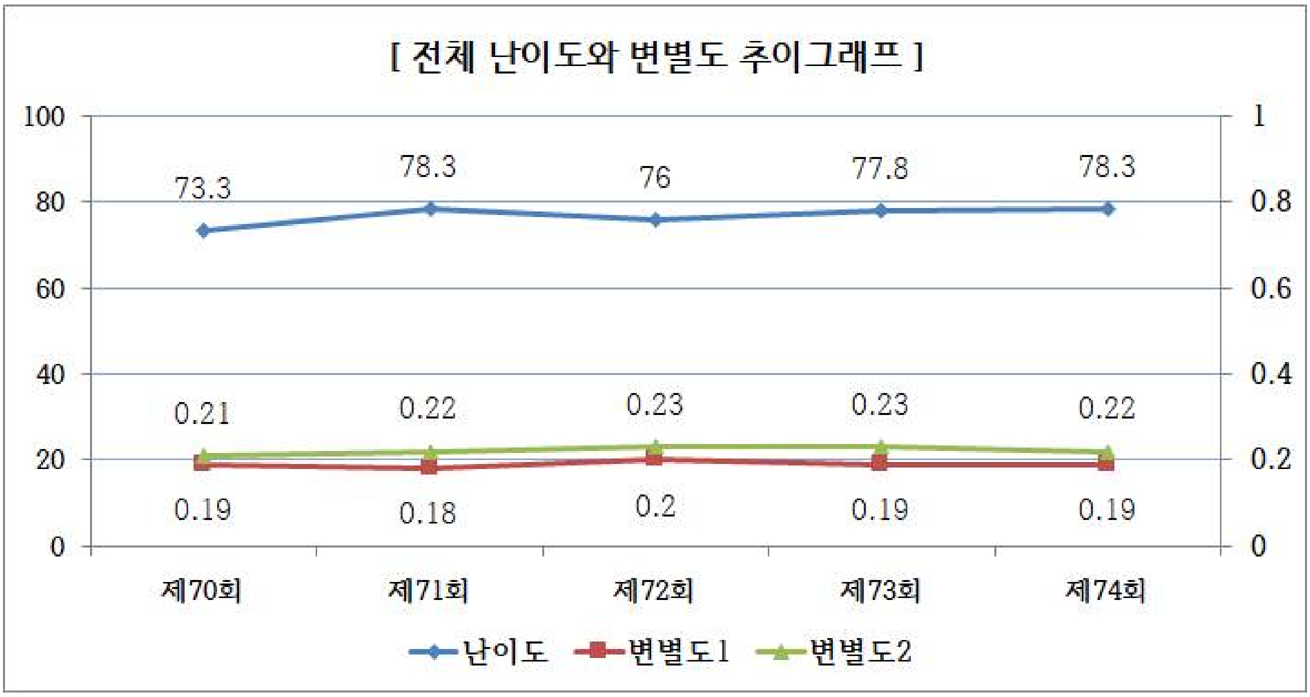

해석

- 전년 대비 난이도 지수는 0.5 증가함
- 변별도 1 지수는 변하지 않음
- 변별도 2 지수는 .01 감소함

나) 전체 난이도와 변별도 분포도 및 비율분석

(1) 전체 난이도 분포도 및 비율분석

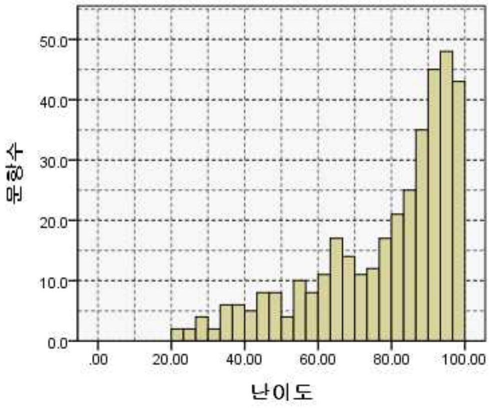

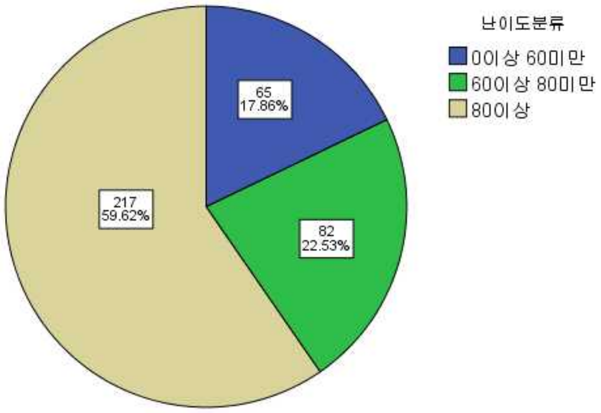

총점	난이도	표준편차
364	78.3	19.1

난이도	문항수	비율(%)
0~60미만	65	17.9
60~80미만	82	22.5
80~100	217	59.6
전체	364	100.0

(2) 전체 변별도1 분포도 및 비율분석

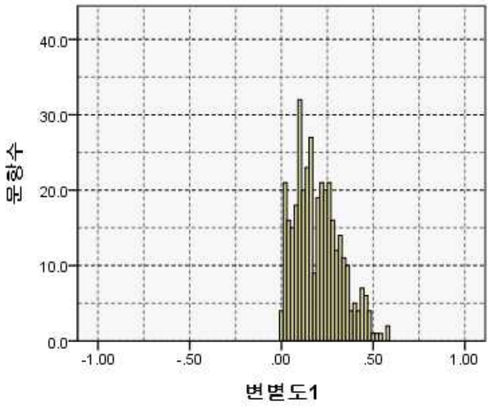

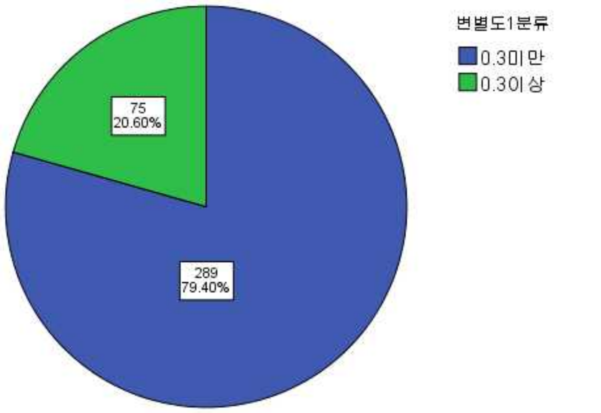

총점	변별도1	표준편차
364	.19	.12

변별도1	문항수	비율(%)
0.3미만	289	79.4
0.3이상	75	20.6
전체	364	100.0

(3) 전체 변별도2 분포도 및 비율분석

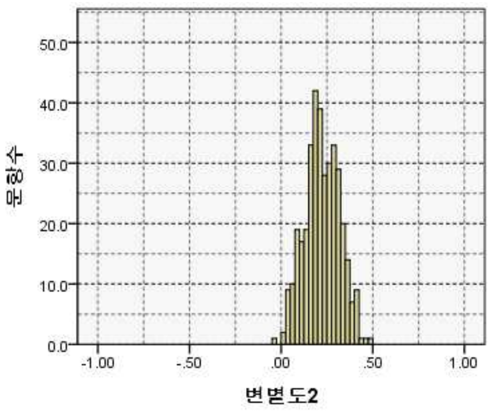

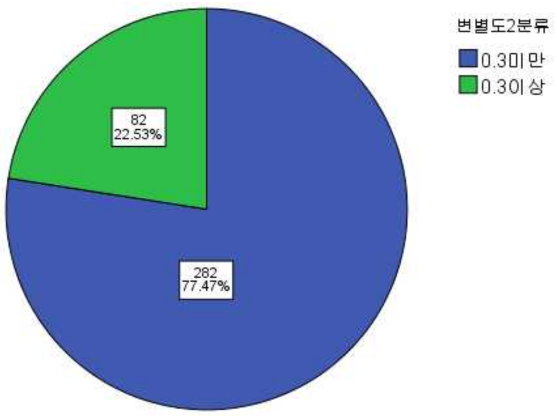

총점	변별도2	표준편차
364	.22	.09

변별도2	문항수	비율(%)
0.3미만	282	77.5
0.3이상	82	22.5
전체	364	100.0

해석

- 난이도 지수가 80 에서 100 사이인 문항이 전체 364 문항 중 217 문항으로 가장 많았으며, 차례로 60 이상 80 미만인 문항이 82 문항, 0 에서 60 미만인 문항이 65 문항인 것으로 나타남
- 변별도 1 지수를 기준으로 분류하였을 때, 0.3 미만인 문항이 289 문항으로 0.3 이상인 문항이 75 문항인 것에 비해 더 많이 나타남
- 변별도 2 지수를 기준으로 분류하였을 때, 0.3 미만인 문항이 282 문항으로 0.3 이상인 문항이 82 문항인 것에 비해 더 많이 나타남

2) 과목별 난이도와 변별도

가) 전회 대비 과목별 난이도와 변별도

(1) 전회 대비 구강악안면외과학 난이도와 변별도

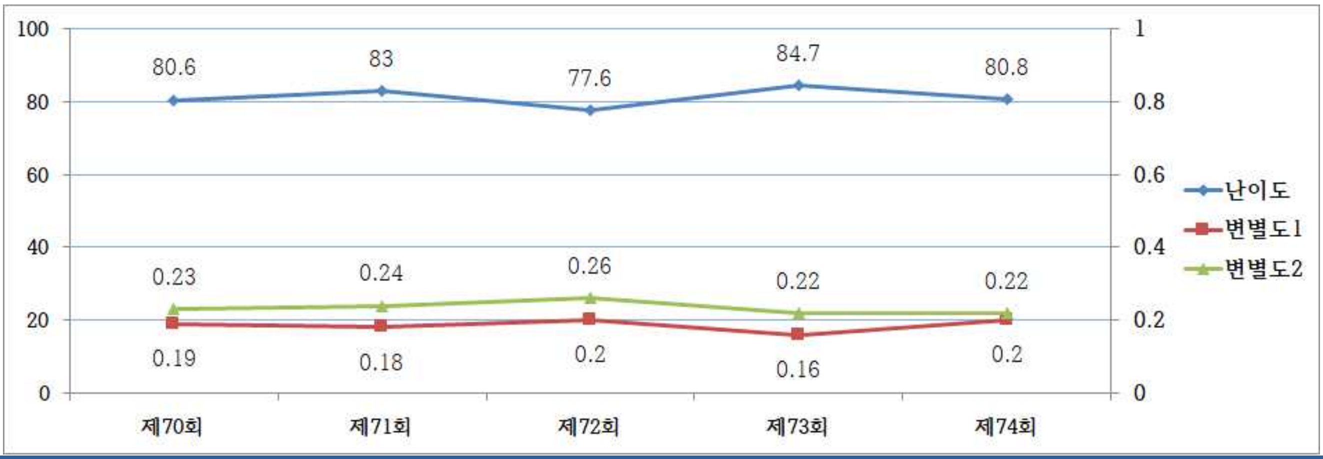

회차	난이도		변별도1		변별도2	
	평균	표준편차	평균	표준편차	평균	표준편차
제70회	80.6	15.1	.19	.09	.23	.09
제71회	83.0	17.1	.18	.13	.24	.10
제72회	77.6	21.4	.20	.13	.26	.10
제73회	84.7	11.9	.16	.12	.22	.11
제74회	80.8	15.5	.20	.13	.22	.10

해석

- 전회 대비 구강악안면외과학 과목의 난이도 지수는 3.9 감소함
- 전회 대비 구강악안면외과학 과목의 변별도 1 지수는 .04 증가함
- 전회 대비 구강악안면외과학 과목의 변별도 2 지수는 변하지 않음

(2) 전회 대비 치과보존학 난이도와 변별도

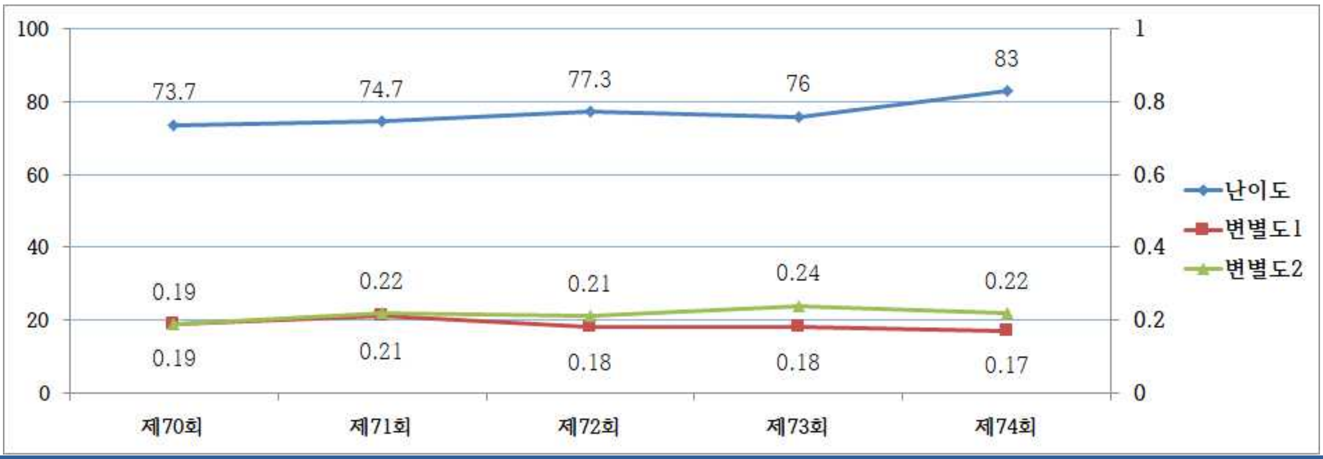

회차	난이도		변별도1		변별도2	
	평균	표준편차	평균	표준편차	평균	표준편차
제70회	73.7	21.2	.19	.10	.19	.07
제71회	74.7	18.9	.21	.12	.22	.09
제72회	77.3	17.0	.18	.11	.21	.08
제73회	76.0	23.4	.18	.13	.24	.10
제74회	83.0	17.9	.17	.11	.22	.08

해석

- 전회 대비 치과보존학 과목의 난이도 지수는 7.0 증가함
- 전회 대비 치과보존학 과목의 변별도 1 지수는 .07 감소함
- 전회 대비 치과보존학 과목의 변별도 2 지수는 .02 감소함

(3) 전회 대비 치과보철학 난이도와 변별도

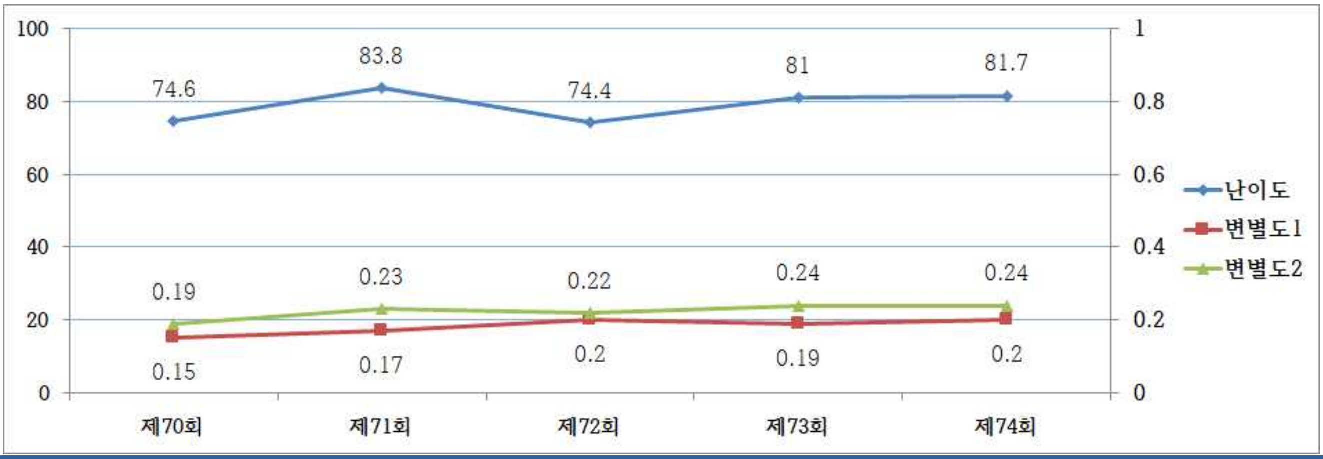

회차	난이도		변별도1		변별도2	
	평균	표준편차	평균	표준편차	평균	표준편차
제70회	74.6	25.9	.15	.11	.19	.09
제71회	83.8	14.6	.17	.11	.23	.08
제72회	74.4	20.4	.20	.12	.22	.09
제73회	81.0	15.7	.19	.12	.24	.09
제74회	81.7	18.3	.20	.12	.24	.09

해석

- 전회 대비 치과보철학 과목의 난이도 지수는 0.7 증가함
- 전회 대비 치과보철학 과목의 변별도 1 지수는 .01 증가함
- 전회 대비 치과보철학 과목의 변별도 2 지수는 변하지 않음

(4) 전회 대비 소아치과학 난이도와 변별도

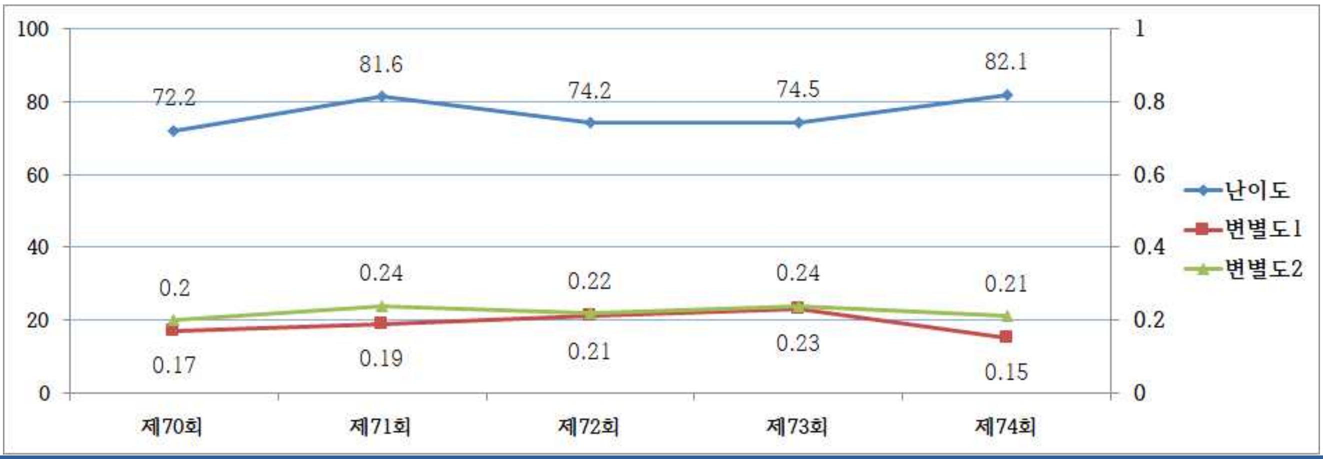

회차	난이도		변별도1		변별도2	
	평균	표준편차	평균	표준편차	평균	표준편차
제70회	72.2	24.5	.17	.11	.20	.08
제71회	81.6	16.2	.19	.11	.24	.09
제72회	74.2	18.0	.21	.11	.22	.09
제73회	74.5	19.8	.23	.13	.24	.08
제74회	82.1	19.5	.15	.12	.21	.09

해석

- 전회 대비 소아치과학 과목의 난이도 지수는 7.6 증가함
- 전회 대비 소아치과학 과목의 변별도 1 지수는 .08 감소함
- 전회 대비 소아치과학 과목의 변별도 2 지수는 .03 감소함

(5) 전회 대비 영상치의학 난이도와 변별도

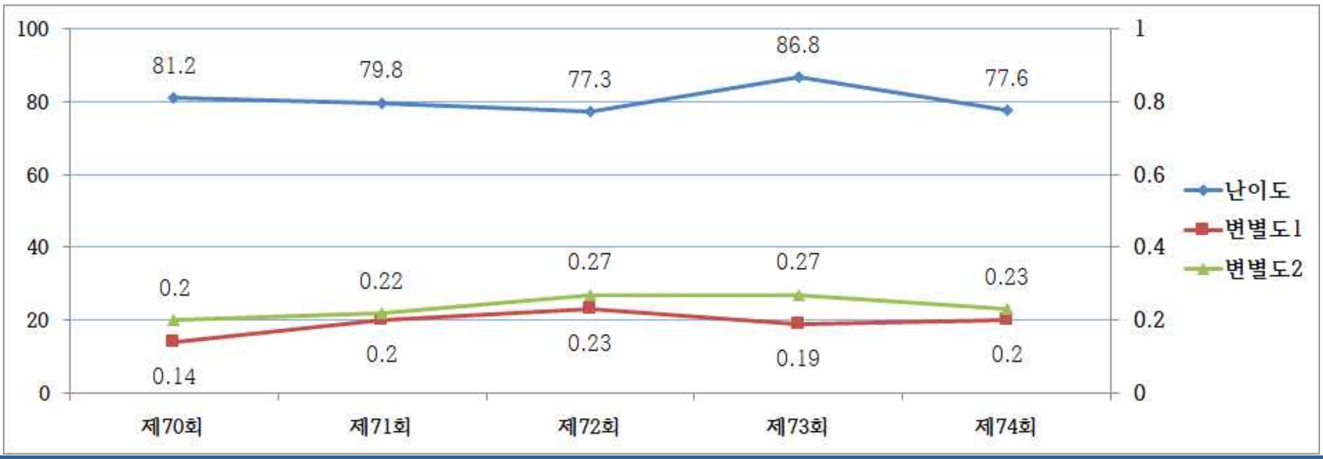

회차	난이도		변별도1		변별도2	
	평균	표준편차	평균	표준편차	평균	표준편차
제70회	81.2	22.6	.14	.12	.20	.08
제71회	79.8	15.4	.20	.14	.22	.09
제72회	77.3	19.8	.23	.14	.27	.08
제73회	86.8	9.9	.19	.13	.27	.10
제74회	77.6	17.9	.20	.12	.23	.10

해석

- 전회 대비 영상치의학 과목의 난이도 지수는 9.2 감소함
- 전회 대비 영상치의학 과목의 변별도 1 지수는 .01 증가함
- 전회 대비 영상치의학 과목의 변별도 2 지수는 .04 감소

(6) 전회 대비 치주과학 난이도와 변별도

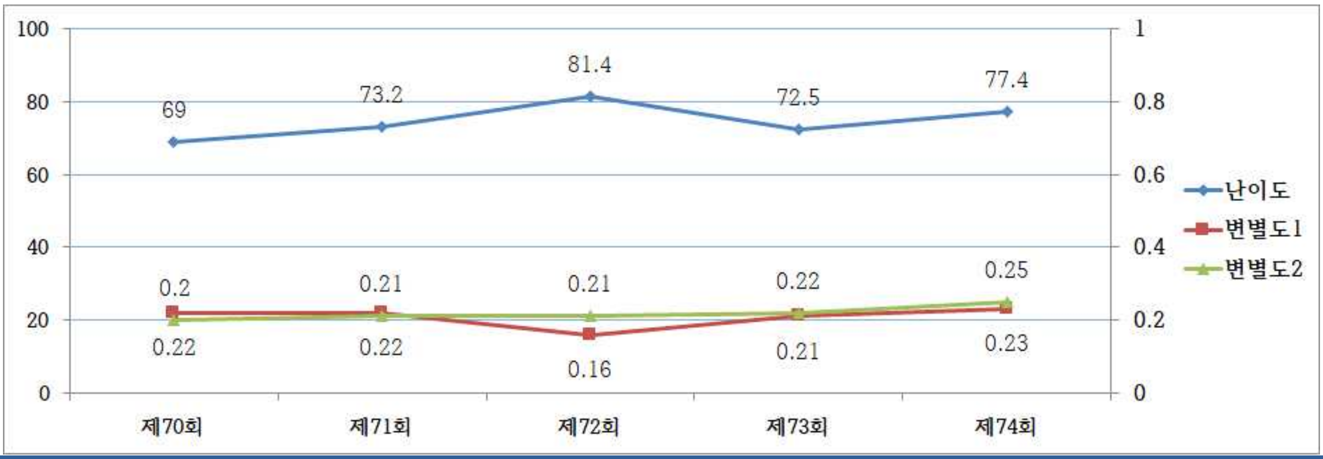

회차	난이도		변별도1		변별도2	
	평균	표준편차	평균	표준편차	평균	표준편차
제70회	69.0	21.8	.22	.13	.20	.10
제71회	73.2	18.4	.22	.14	.21	.10
제72회	81.4	13.6	.16	.09	.21	.09
제73회	72.5	20.4	.21	.16	.22	.13
제74회	77.4	18.6	.23	.14	.25	.11

해석

- 전회 대비 치주과학 과목의 난이도 지수는 4.9 증가함
- 전회 대비 치주과학 과목의 변별도 1 지수는 .02 증가함
- 전회 대비 치주과학 과목의 변별도 2 지수는 .03 증가함

(7) 전회 대비 구강내과학 난이도와 변별도

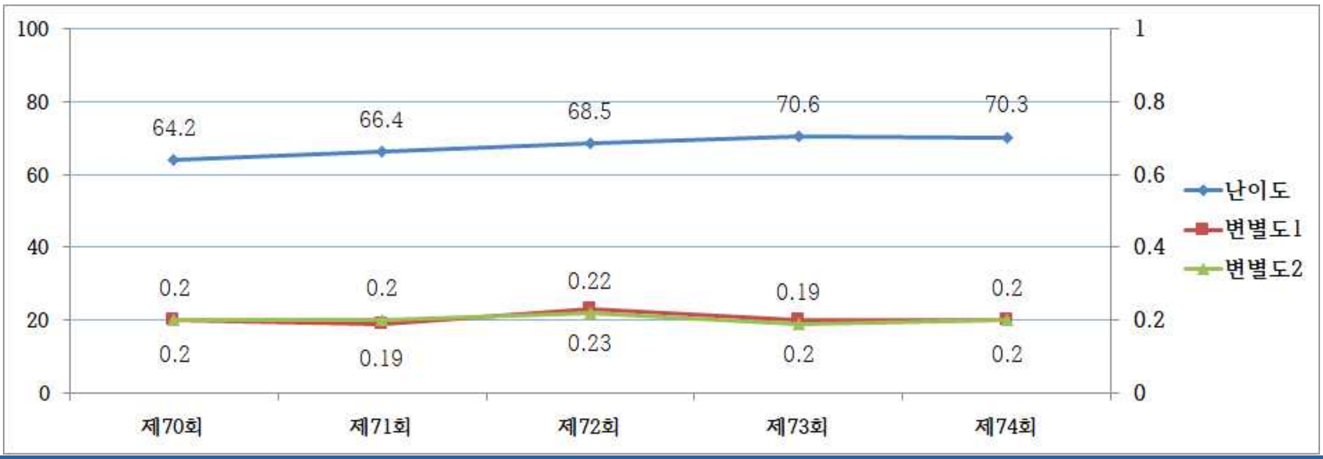

회차	난이도		변별도1		변별도2	
	평균	표준편차	평균	표준편차	평균	표준편차
제70회	64.2	26.5	.20	.12	.20	.11
제71회	66.4	25.3	.19	.09	.20	.08
제72회	68.5	20.1	.23	.13	.22	.12
제73회	70.6	21.9	.20	.16	.19	.13
제74회	70.3	18.2	.20	.15	.20	.12

해석

- 전회 대비 구강내과학 과목의 난이도 지수는 0.3 감소함
- 전회 대비 구강내과학 과목의 변별도 1 지수는 변하지 않음
- 전회 대비 구강내과학 과목의 변별도 2 지수는 .01 증가함

(8) 전회 대비 치과재료학 난이도와 변별도

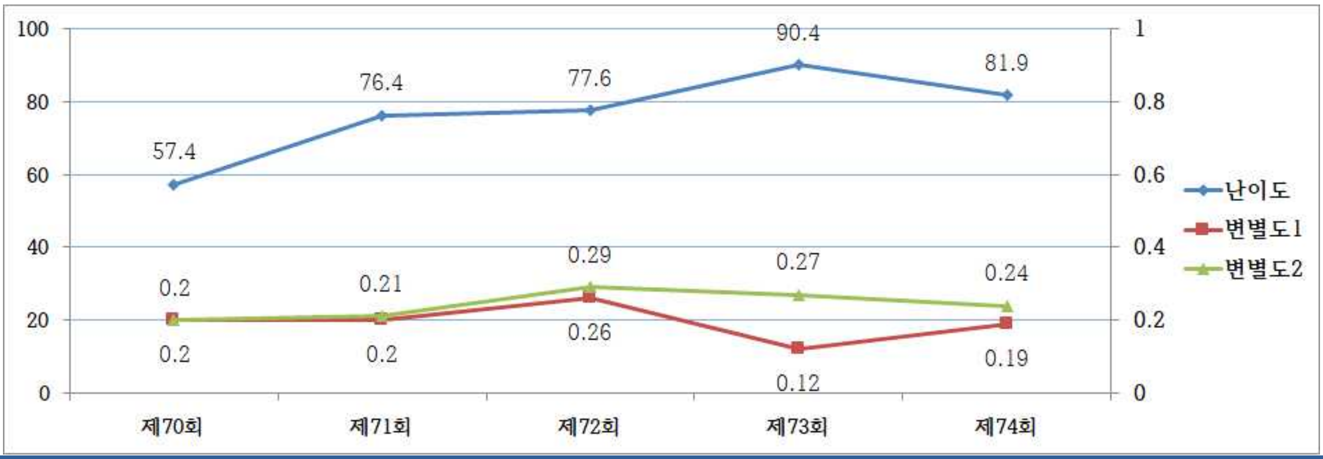

회차	난이도		변별도1		변별도2	
	평균	표준편차	평균	표준편차	평균	표준편차
제70회	57.4	30.0	.20	.11	.20	.11
제71회	76.4	22.4	.20	.09	.21	.07
제72회	77.6	17.5	.26	.12	.29	.07
제73회	90.4	13.7	.12	.07	.27	.09
제74회	81.9	18.4	.19	.14	.24	.08

해석

- 전회 대비 치과재료학 과목의 난이도 지수는 8.5 감소함
- 전회 대비 치과재료학 과목의 변별도 1 지수는 .07 증가함
- 전회 대비 치과재료학 과목의 변별도 2 지수는 .03 감소함

(9) 전회 대비 치과교정학 난이도와 변별도

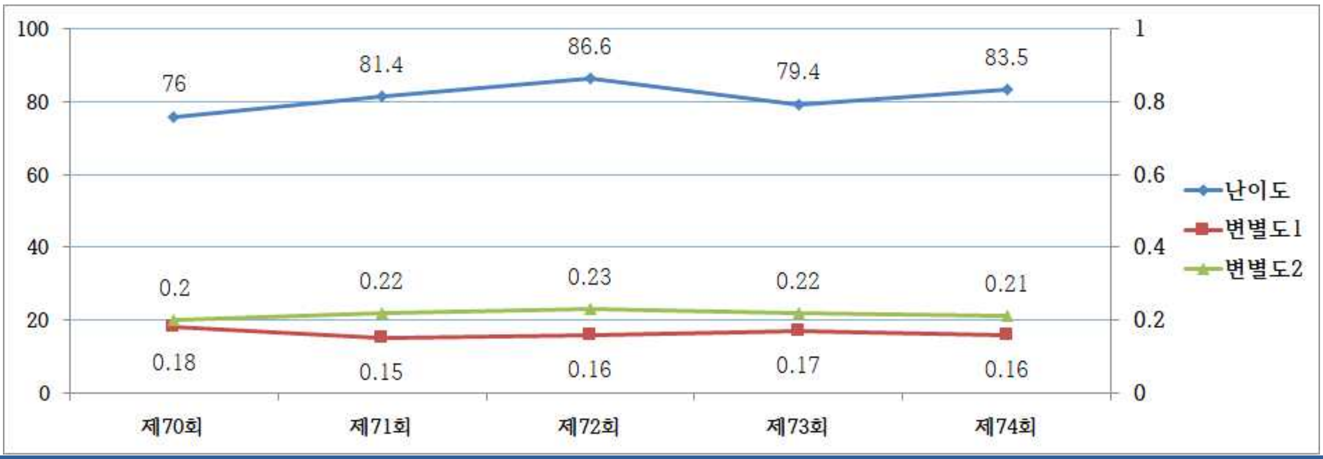

회차	난이도		변별도1		변별도2	
	평균	표준편차	평균	표준편차	평균	표준편차
제70회	76.0	17.9	.18	.11	.20	.08
제71회	81.4	21.5	.15	.11	.22	.09
제72회	86.6	12.5	.16	.10	.23	.07
제73회	79.4	21.7	.17	.14	.22	.12
제74회	83.5	14.1	.16	.10	.21	.08

해석

- 전회 대비 치과교정학 과목의 난이도 지수는 4.1 증가함
- 전회 대비 치과교정학 과목의 변별도 1 지수는 .01 감소함
- 전회 대비 치과교정학 과목의 변별도 2 지수는 .01 감소함

(10) 전회 대비 구강병리학 난이도와 변별도

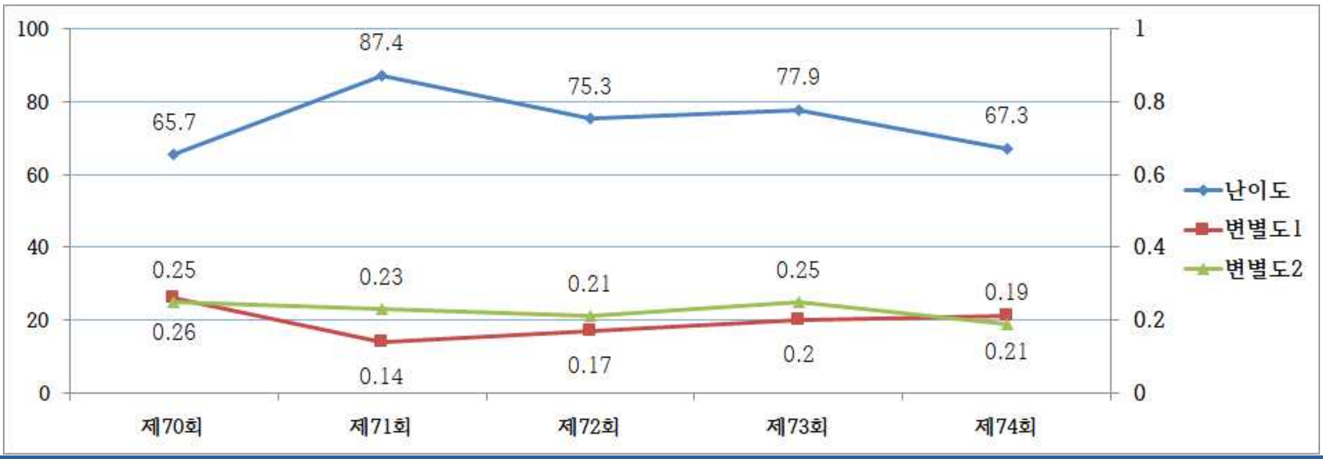

회차	난이도		변별도1		변별도2	
	평균	표준편차	평균	표준편차	평균	표준편차
제70회	65.7	21.5	.26	.17	.25	.14
제71회	87.4	11.1	.14	.08	.23	.08
제72회	75.3	23.8	.17	.08	.21	.08
제73회	77.9	19.5	.20	.10	.25	.10
제74회	67.3	21.2	.21	.12	.19	.08

해석

- 전회 대비 구강병리학 과목의 난이도 지수는 10.6 감소함
- 전회 대비 구강병리학 과목의 변별도 1 지수는 .01 증가함
- 전회 대비 구강병리학 과목의 변별도 2 지수는 .06 감소함

(11) 전회 대비 구강보건학 난이도와 변별도

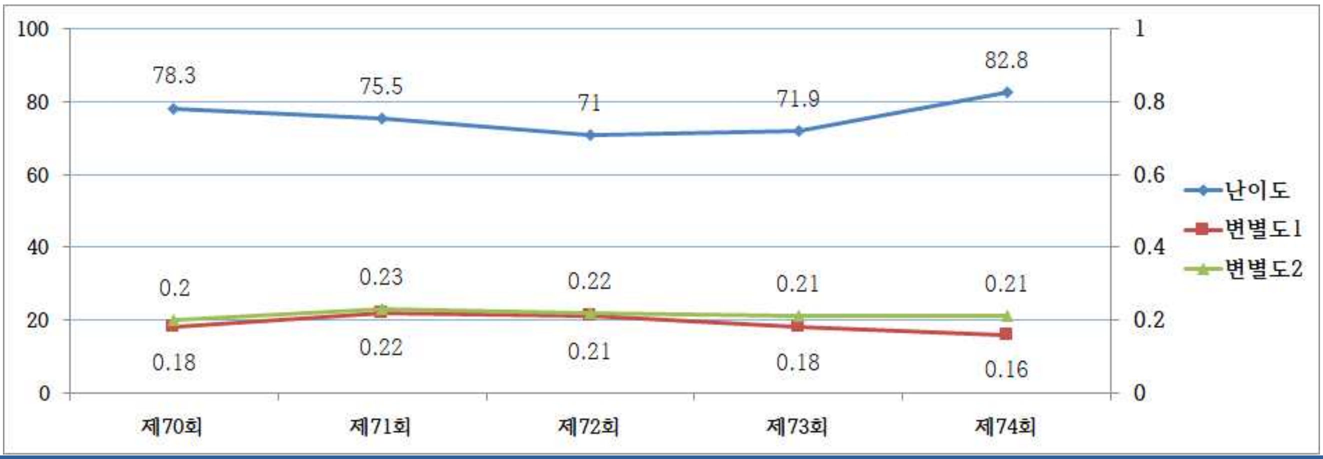

회차	난이도		변별도1		변별도2	
	평균	표준편차	평균	표준편차	평균	표준편차
제70회	78.3	16.2	.18	.09	.20	.08
제71회	75.5	18.7	.22	.14	.23	.09
제72회	71.0	23.2	.21	.10	.22	.08
제73회	71.9	26.2	.18	.13	.21	.13
제74회	82.8	18.7	.16	.11	.21	.10

해석

- 전회 대비 구강보건학 과목의 난이도 지수는 10.9 증가함
- 전회 대비 구강보건학 과목의 변별도 1 지수는 .02 감소함
- 전회 대비 구강보건학 과목의 변별도 2 지수는 변하지 않음

(12) 전회 대비 구강생물학 난이도와 변별도

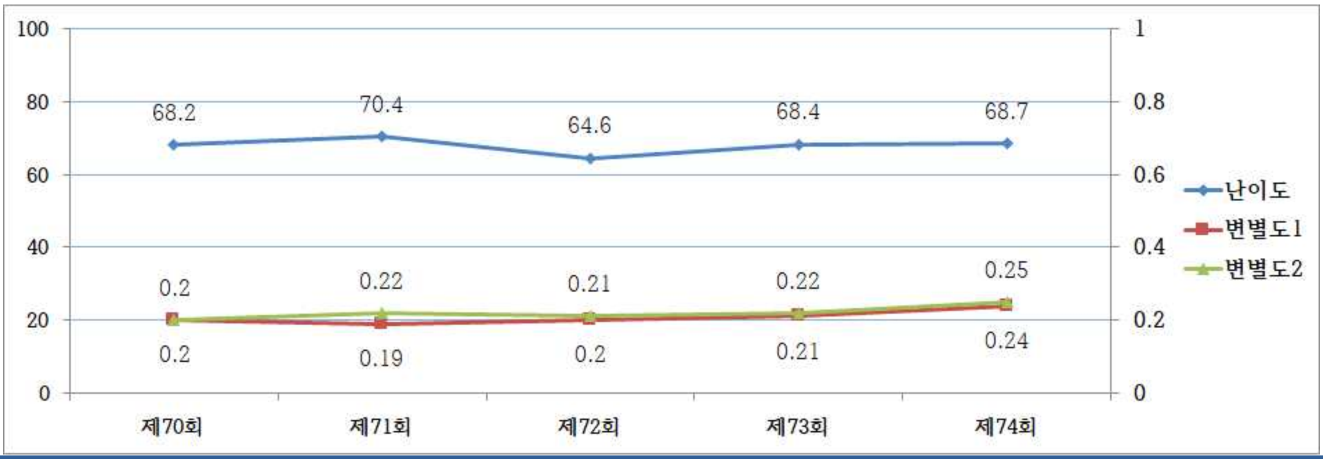

회차	난이도		변별도1		변별도2	
	평균	표준편차	평균	표준편차	평균	표준편차
제70회	68.2	19.2	.20	.12	.20	.10
제71회	70.4	22.3	.19	.11	.22	.09
제72회	64.6	24.4	.20	.13	.21	.09
제73회	68.4	-	.21	-	.22	-
제74회	68.7	22.1	.24	.14	.25	.11

해석

- 전회 대비 구강생물학 과목의 난이도 지수는 0.3 증가함
- 전회 대비 구강생물학 과목의 변별도 1 지수는 .03 증가함
- 전회 대비 구강생물학 과목의 변별도 2 지수는 .03 증가함

(13) 전회 대비 보건의약관계법규 난이도와 변별도

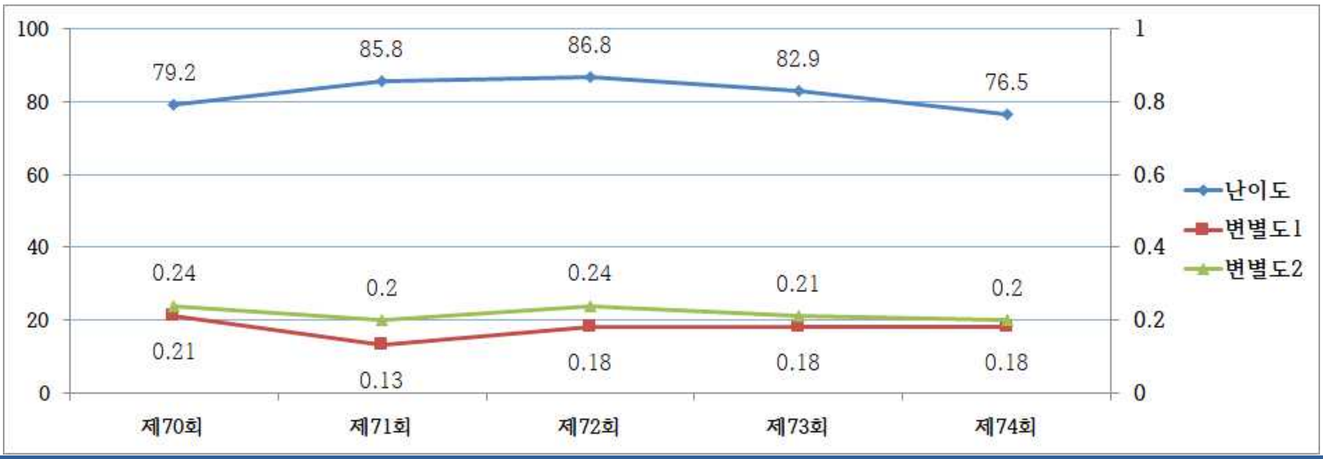

회차	난이도		변별도1		변별도2	
	평균	표준편차	평균	표준편차	평균	표준편차
제70회	79.2	16.2	.21	.11	.24	.07
제71회	85.8	16.3	.13	.10	.20	.08
제72회	86.8	10.5	.18	.12	.24	.09
제73회	82.9	12.9	.18	.09	.21	.10
제74회	76.5	22.6	.18	.12	.20	.09

해석

- 전회 대비 보건의약관계법규 과목의 난이도 지수는 6.4 감소함
- 전회 대비 보건의약관계법규 과목의 변별도 1 지수는 변하지 않음
- 전회 대비 보건의약관계법규 과목의 변별도 2 지수는 .01 감소함

나) 과목별 난이도와 변별도 분포도 및 비율분석

(1) 구강악안면외과학 난이도와 변별도 분포도 및 비율분석

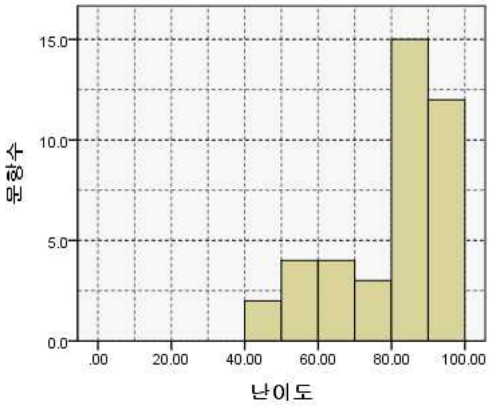

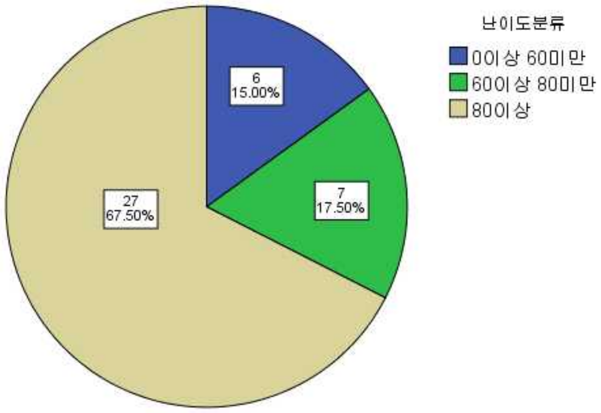

총점	난이도	표준편차
40	80.8	15.5

난이도	문항수	비율(%)
0~60미만	6	15.0
60~80미만	7	17.5
80~100	27	67.5
전체	40	100.0

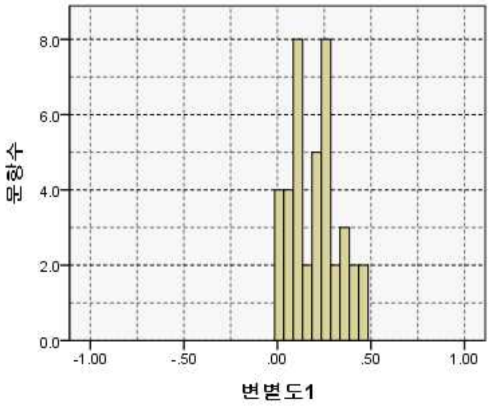

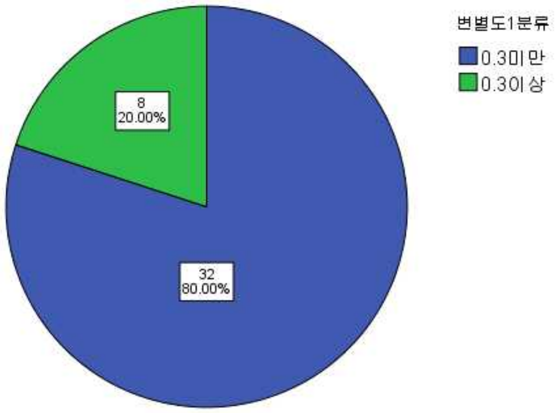

총점	변별도1	표준편차
40	.20	.13

변별도1	문항수	비율(%)
0.3미만	32	80.0
0.3이상	8	20.0
전체	40	100.0

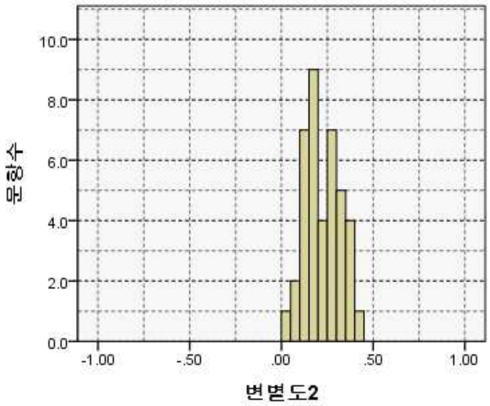

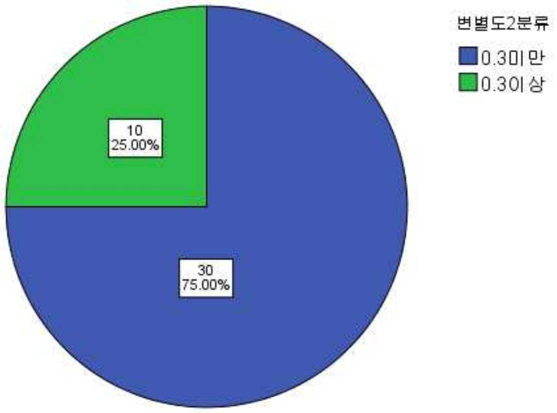

총점	변별도2	표준편차
40	.22	.10

변별도2	문항수	비율(%)
0.3미만	30	75.0
0.3이상	10	25.0
전체	40	100.0

해석

- 구강악안면외과학 과목에서 난이도 지수가 80 에서 100 사이인 문항이 전체 40 문항 중 27 문항으로 가장 많았으며, 차례로 60 이상 80 미만인 문항이 7 문항, 0 에서 60 미만인 문항이 6 문항으로 나타남
- 변별도 1 지수를 기준으로 분류하였을 때, 0.3 미만인 문항이 32 문항으로 0.3 이상인 문항이 8 문항인 것에 비해 더 많이 나타남
- 변별도 2 지수를 기준으로 분류하였을 때, 0.3 미만인 문항이 30 문항으로 0.3 이상인 문항이 10 문항인 것에 비해 더 많이 나타남

(2) 치과보존학 난이도와 변별도 분포도 및 비율분석

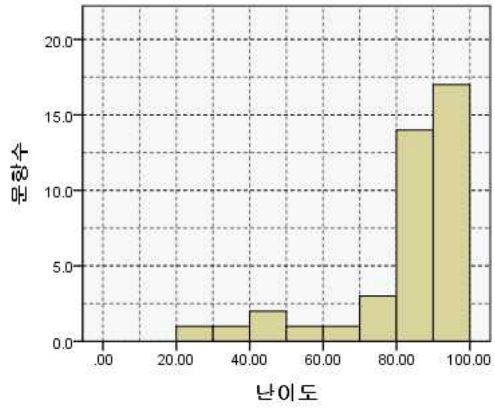

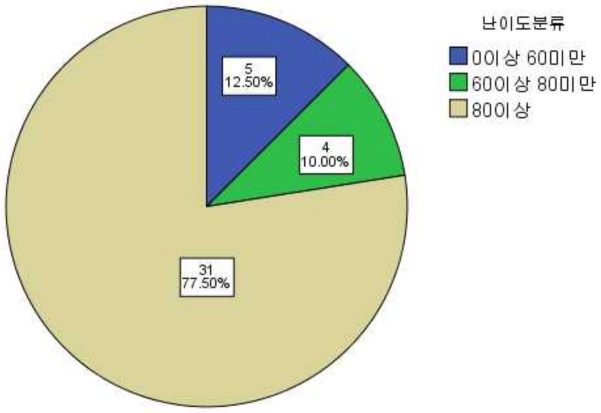

총점	난이도	표준편차
40	83.0	17.9

난이도	문항수	비율(%)
0~60미만	5	12.5
60~80미만	4	10.0
80~100	31	77.5
전체	40	100.0

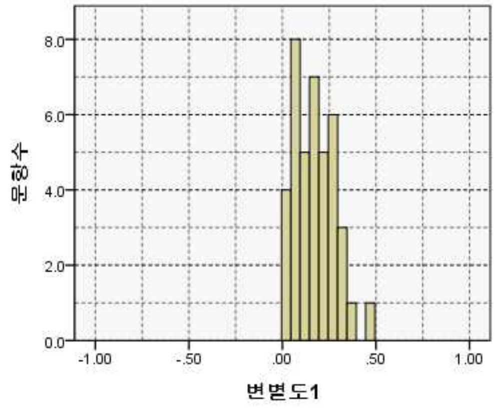

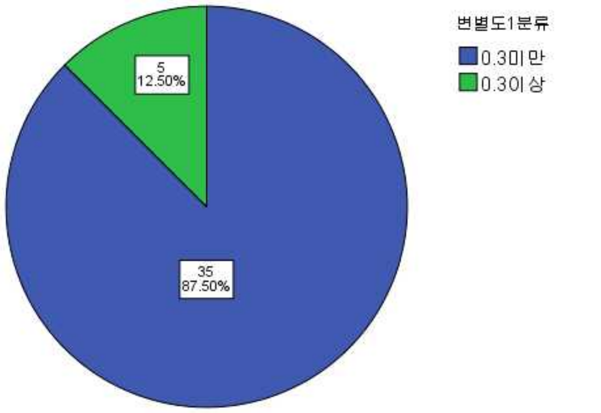

총점	변별도1	표준편차
40	.17	.11

변별도1	문항수	비율(%)
0.3미만	35	87.5
0.3이상	5	12.5
전체	40	100.0

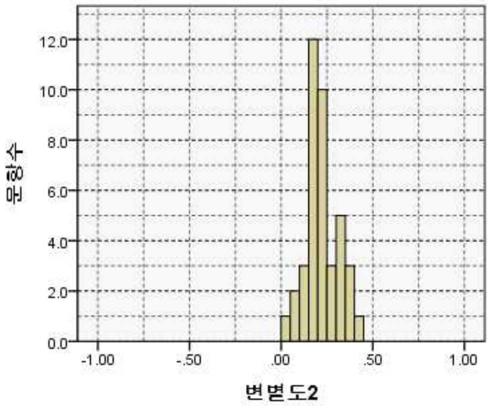

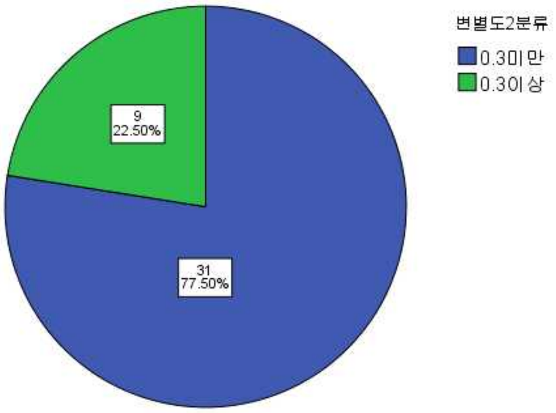

총점	변별도2	표준편차
40	.22	.08

변별도2	문항수	비율(%)
0.3미만	31	77.5
0.3이상	9	22.5
전체	40	100.0

해석

- 치과보존학 과목에서 난이도 지수가 80 에서 100 사이인 문항이 전체 40 문항 중 31 문항이었으며, 차례로 0 에서 60 미만인 문항이 4 문항, 60 이상 80 미만인 문항이 5 문항으로 나타남
- 변별도 1 지수를 기준으로 분류하였을 때, 0.3 미만인 문항이 35 문항으로 0.3 이상인 문항이 5 문항인 것에 비해 더 많이 나타남
- 변별도 2 지수를 기준으로 분류하였을 때, 0.3 미만인 문항이 31 문항으로 0.3 이상인 문항이 9 문항인 것에 비해 더 많이 나타남

(3) 치과보철학 난이도와 변별도 분포도 및 비율분석

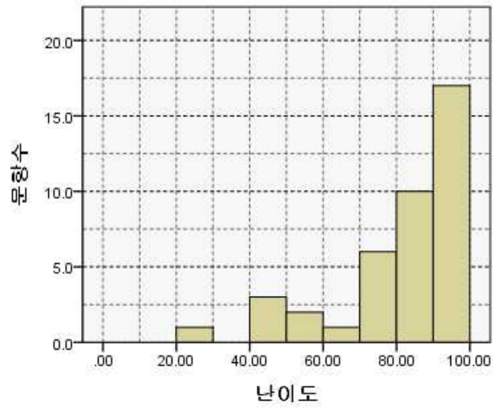

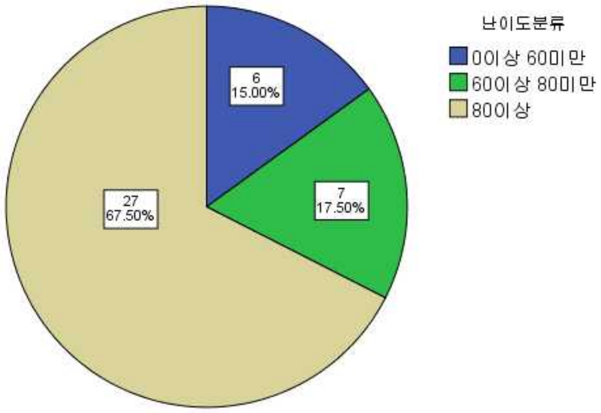

총점	난이도	표준편차
40	81.7	18.3

난이도	문항수	비율(%)
0~60미만	6	15.0
60~80미만	7	17.5
80~100	27	67.5
전체	40	100.0

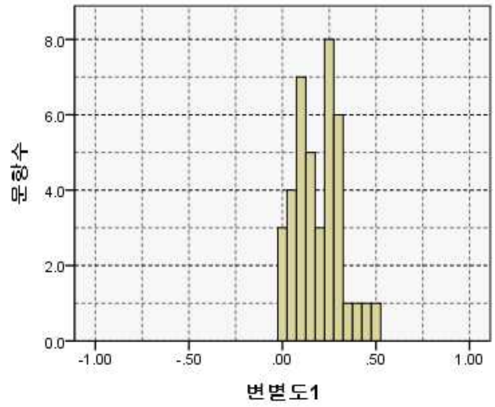

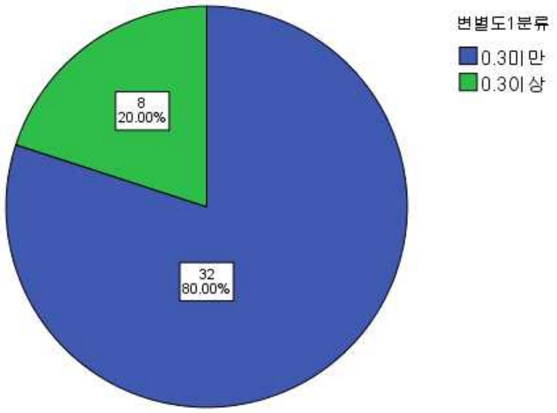

총점	변별도1	표준편차
40	.20	.12

변별도1	문항수	비율(%)
0.3미만	32	80.0
0.3이상	8	20.0
전체	40	100.0

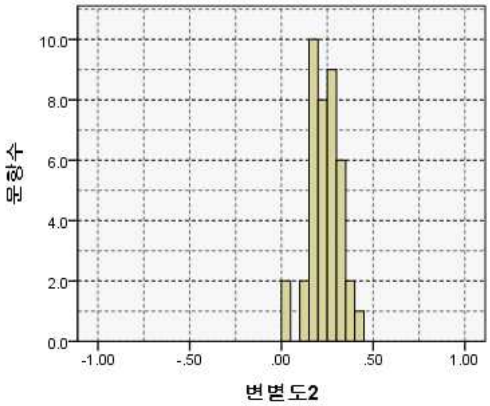

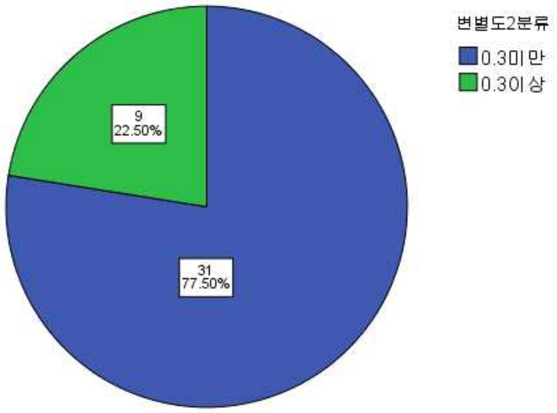

총점	변별도2	표준편차
40	.24	.09

변별도2	문항수	비율(%)
0.3미만	31	77.5
0.3이상	9	22.5
전체	40	100.0

해석

- 치과보철학 과목에서 난이도 지수가 80 에서 100 사이인 문항이 전체 40 문항 중 27 문항이었으며, 차례로 60 이상 80 미만인 문항이 7 문항, 0 에서 60 미만인 문항이 6 문항으로 나타남
- 변별도 1 지수를 기준으로 분류하였을 때, 0.3 미만인 문항이 32 문항으로 0.3 이상인 문항이 8 문항인 것에 비해 더 많이 나타남
- 변별도 2 지수를 기준으로 분류하였을 때, 0.3 미만인 문항이 31 문항으로 0.3 이상인 문항이 9 문항인 것에 비해 더 많이 나타남

(4) 소아치과학 난이도와 변별도 분포도 및 비율분석

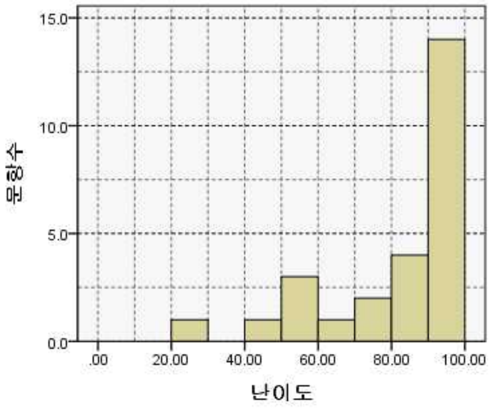

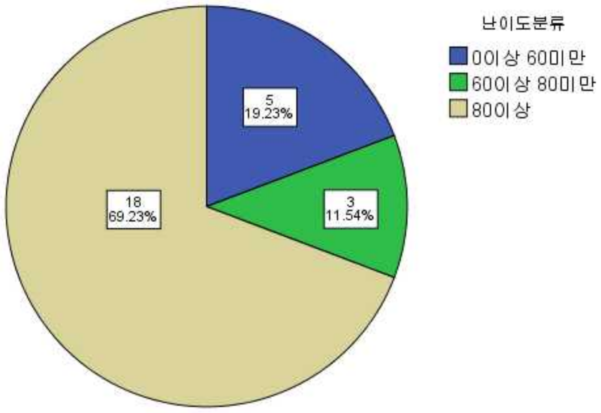

총점	난이도	표준편차
26	82.1	19.5

난이도	문항수	비율(%)
0~60미만	5	19.2
60~80미만	3	11.5
80~100	18	69.2
전체	26	100.0

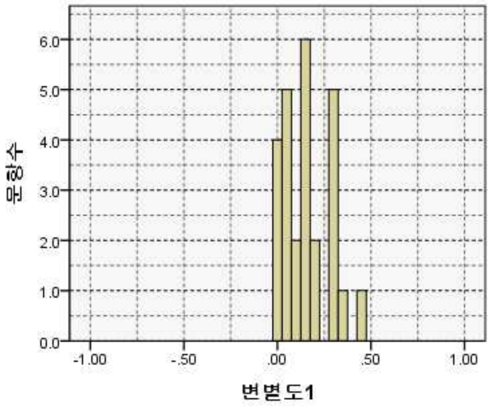

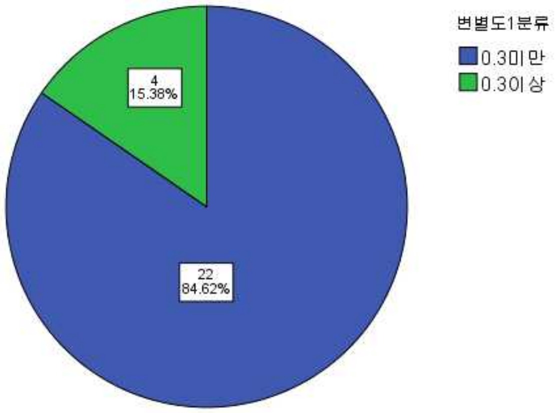

총점	변별도1	표준편차
26	.15	.12

변별도1	문항수	비율(%)
0.3미만	22	84.6
0.3이상	4	15.4
전체	26	100.0

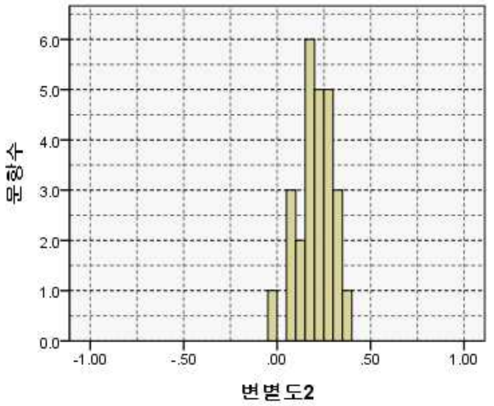

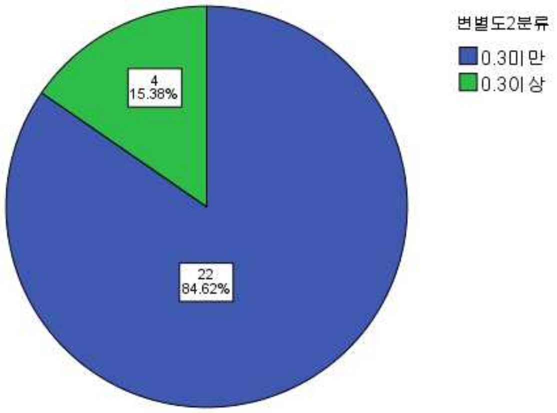

총점	변별도2	표준편차
26	.21	.09

변별도2	문항수	비율(%)
0.3미만	22	84.6
0.3이상	4	15.4
전체	26	100.0

해석

- 소아치과학 과목에서 난이도 지수가 80 에서 100 사이인 문항이 전체 26 문항 중 18 문항으로 가장 많았으며, 차례로 0 에서 60 미만인 문항이 3 문항, 60 이상 80 미만인 문항이 5 문항으로 나타남
- 변별도 1 지수를 기준으로 분류하였을 때, 0.3 미만인 문항이 22 문항으로 0.3 이상인 문항이 4 문항인 것에 비해 더 많이 나타남
- 변별도 2 지수를 기준으로 분류하였을 때, 0.3 미만인 문항이 22 문항으로 0.3 이상인 문항이 4 문항인 것에 비해 더 많이 나타남

(5) 영상치의학 난이도와 변별도 분포도 및 비율분석

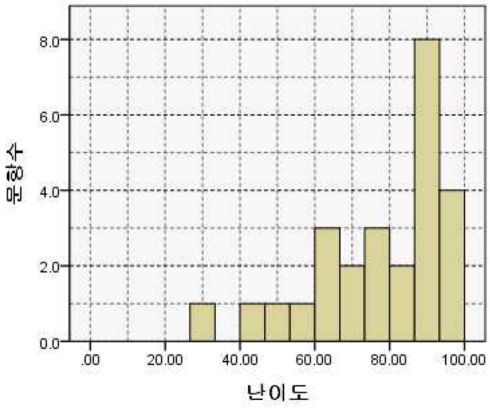

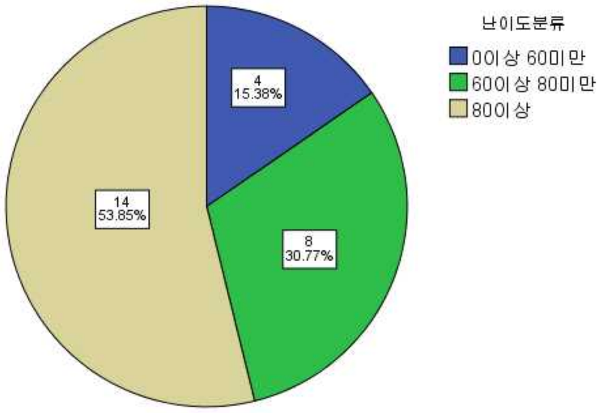

총점	난이도	표준편차
26	77.6	17.9

난이도	문항수	비율(%)
0~60미만	4	15.4
60~80미만	8	30.8
80~100	14	53.8
전체	26	100.0

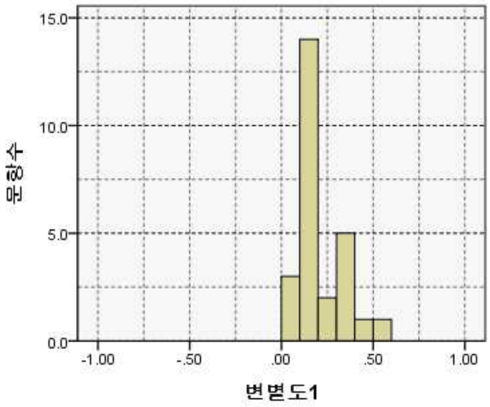

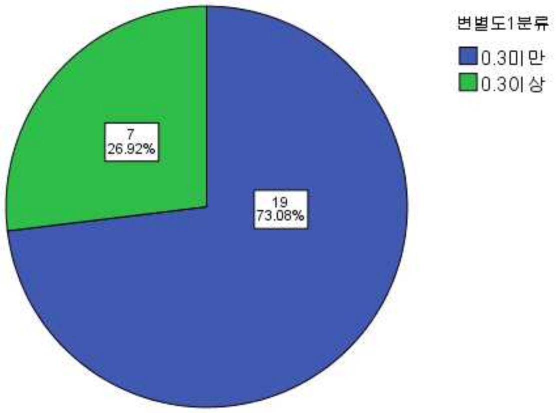

총점	변별도1	표준편차
26	.20	.12

변별도1	문항수	비율(%)
0.3미만	19	73.1
0.3이상	7	26.9
전체	26	100.0

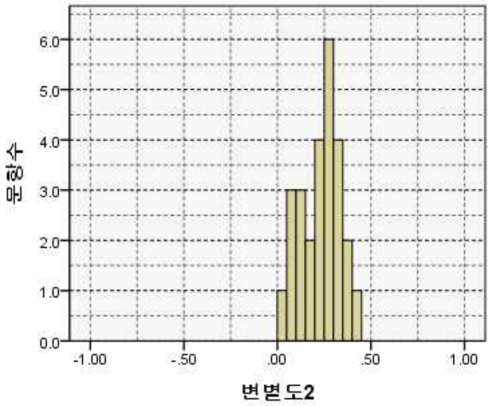

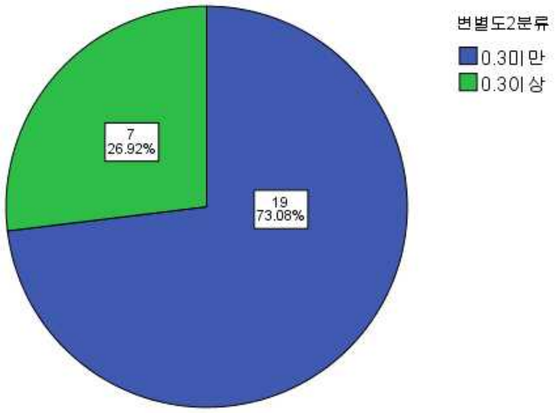

총점	변별도2	표준편차
26	.23	.10

변별도2	문항수	비율(%)
0.3미만	19	73.1
0.3이상	7	26.9
전체	26	100.0

해석

- 영상치의학 과목에서 난이도 지수가 80 에서 100 사이인 문항이 전체 26 문항 중 14 문항이었으며, 차례로 60 이상 80 미만인 문항이 8 문항, 0 에서 60 미만인 문항이 4 문항으로 나타남
- 변별도 1 지수를 기준으로 분류하였을 때, 0.3 미만인 문항이 19 문항으로 0.3 이상인 문항이 7 문항인 것에 비해 더 많이 나타남
- 변별도 2 지수를 기준으로 분류하였을 때, 0.3 미만인 문항이 19 문항으로 0.3 이상인 문항이 7 문항인 것에 비해 더 많이 나타남

(6) 치주과학 난이도와 변별도 분포도 및 비율분석

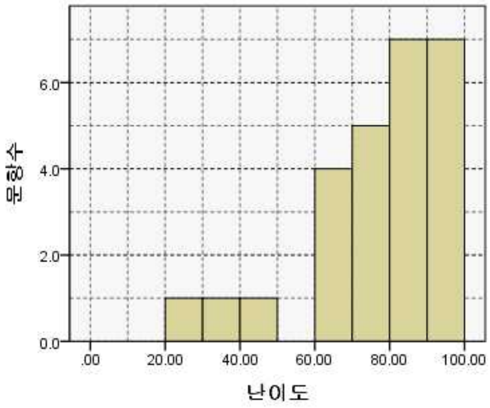

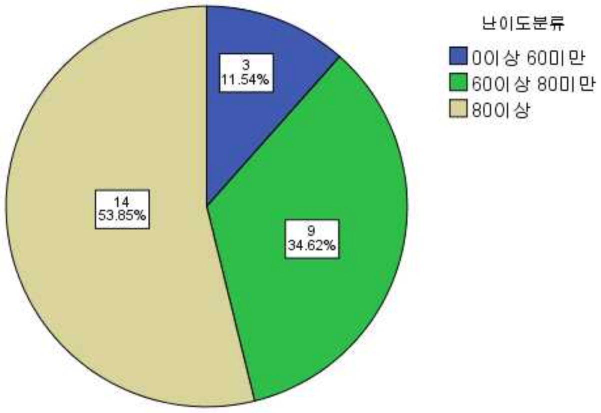

총점	난이도	표준편차
26	77.4	18.6

난이도	문항수	비율(%)
0~60미만	3	11.5
60~80미만	9	34.6
80~100	14	53.8
전체	26	100.0

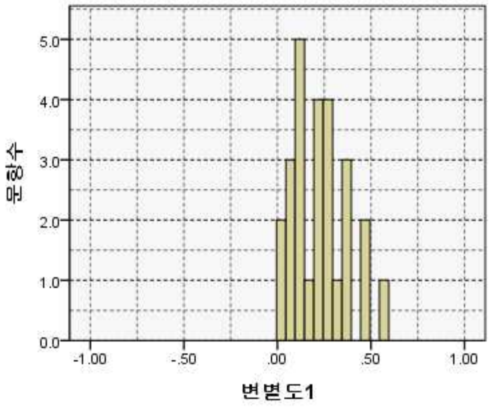

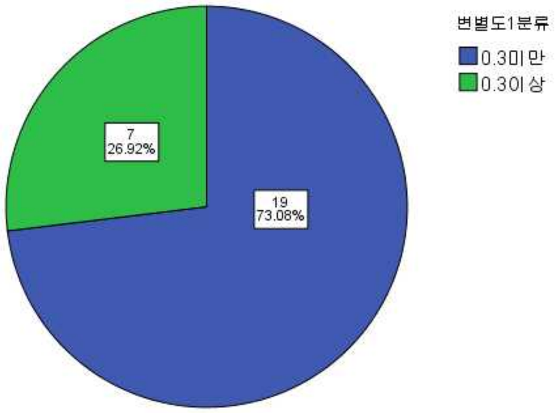

총점	변별도1	표준편차
26	.23	.14

변별도1	문항수	비율(%)
0.3미만	19	73.1
0.3이상	7	26.9
전체	26	100.0

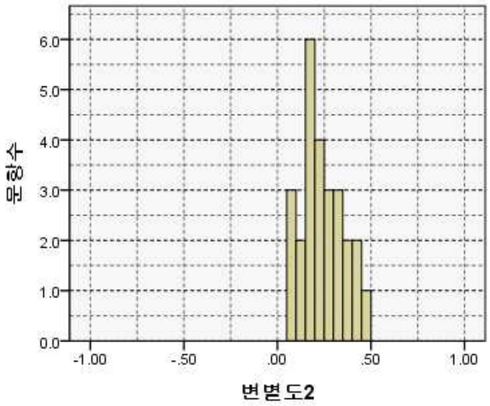

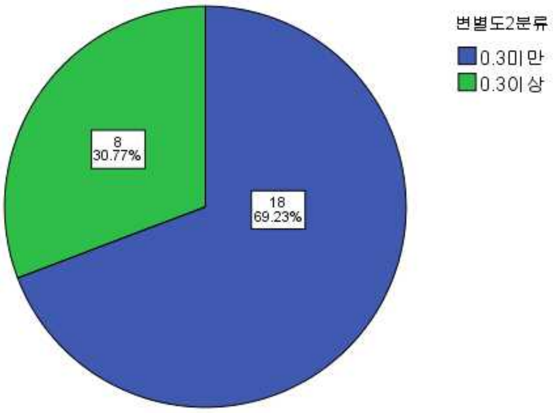

총점	변별도2	표준편차
26	.25	.11

변별도2	문항수	비율(%)
0.3미만	18	69.2
0.3이상	8	30.8
전체	26	100.0

해석

- 치주과학 과목에서 난이도 지수가 80 에서 100 사이인 문항이 전체 26 문항 중 14 문항, 60 이상 80 미만인 문항이 9 문항으로 가장 많았으며 차례로 0 에서 60 미만인 문항이 3 문항으로 나타남
- 변별도 1 지수를 기준으로 분류하였을 때, 0.3 미만인 문항이 19 문항으로 0.3 이상인 문항이 7 문항인 것에 비해 더 많이 나타남
- 변별도 2 지수를 기준으로 분류하였을 때, 0.3 미만인 문항이 18 문항으로 0.3 이상인 문항이 8 문항인 것에 비해 더 많이 나타남

(7) 구강내과학 난이도와 변별도 분포도 및 비율분석

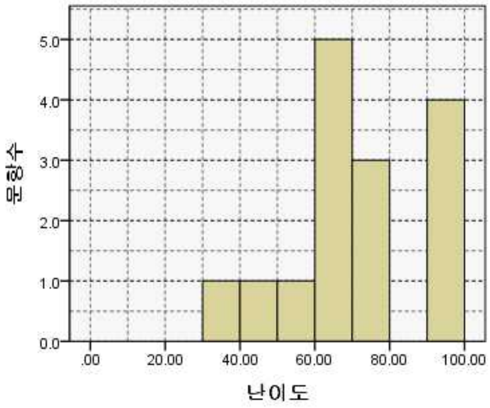

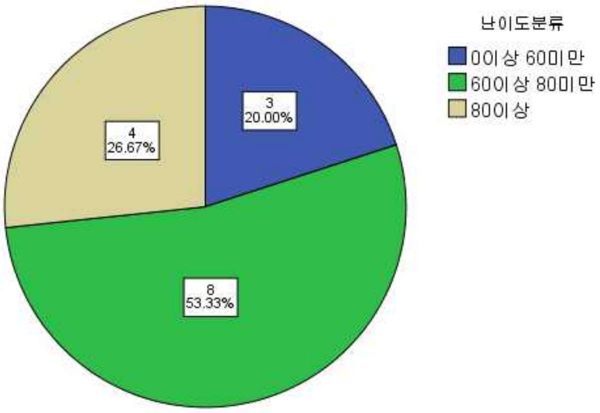

총점	난이도	표준편차
15	70.3	18.2

난이도	문항수	비율(%)
0~60미만	3	20.0
60~80미만	8	53.3
80~100	4	26.7
전체	15	100.0

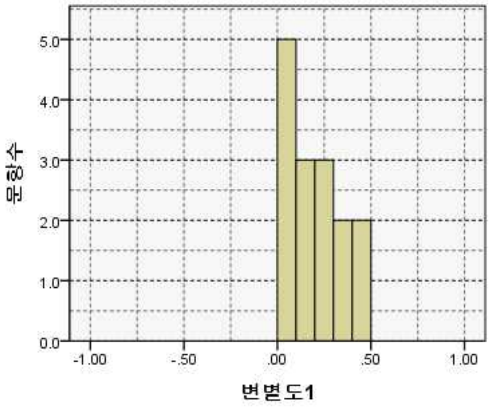

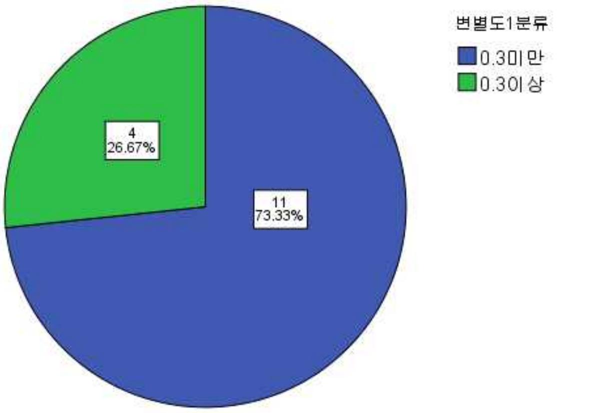

총점	변별도1	표준편차
15	.20	.15

변별도1	문항수	비율(%)
0.3미만	11	73.3
0.3이상	4	26.7
전체	15	100.0

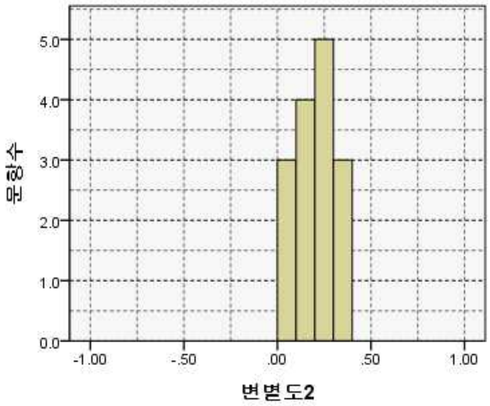

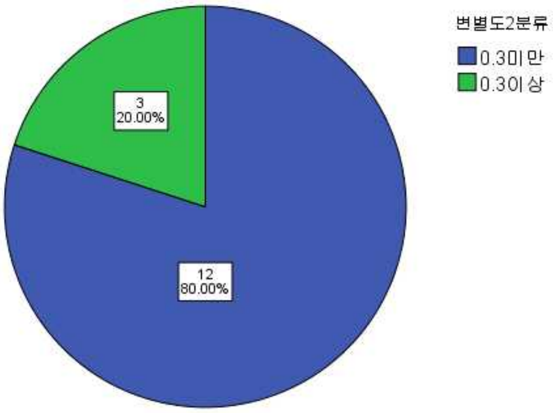

총점	변별도2	표준편차
15	.20	.12

변별도2	문항수	비율(%)
0.3미만	12	80.0
0.3이상	3	20.0
전체	15	100.0

해석

- 구강내과학 과목에서 난이도 지수가 80 에서 100 사이인 문항이 전체 15 문항 중 4 문항이었으며, 차례로 0 에서 60 미만인 문항이 8 문항, 60 이상 80 미만인 문항이 3 문항으로 나타남
- 변별도 1 지수를 기준으로 분류하였을 때, 0.3 미만인 문항이 11 문항으로 0.3 이상인 문항이 4 문항인 것에 비해 더 많이 나타남
- 변별도 2 지수를 기준으로 분류하였을 때, 0.3 미만인 문항이 12 문항으로 0.3 이상인 문항이 3 문항인 것에 비해 더 많이 나타남

(8) 치과재료학 난이도와 변별도 분포도 및 비율분석

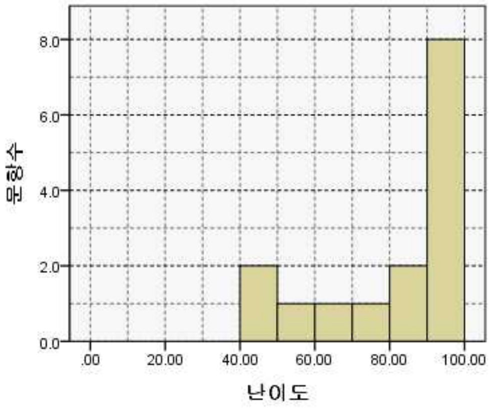

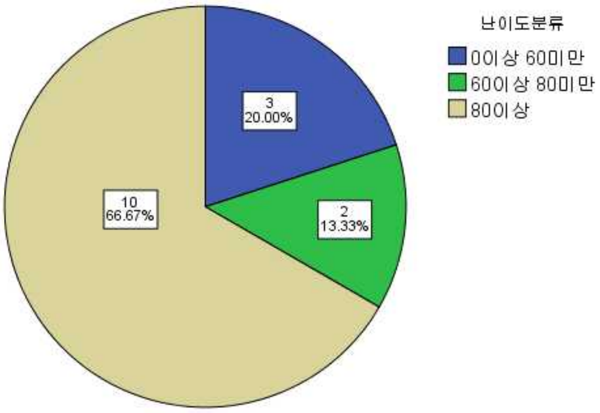

총점	난이도	표준편차
15	81.9	18.4

난이도	문항수	비율(%)
0~60미만	3	20.0
60~80미만	2	13.3
80~100	10	66.7
전체	15	100.0

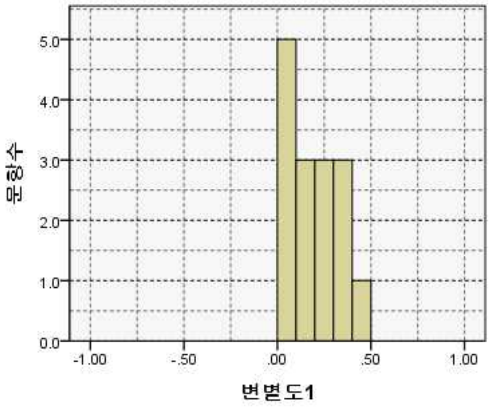

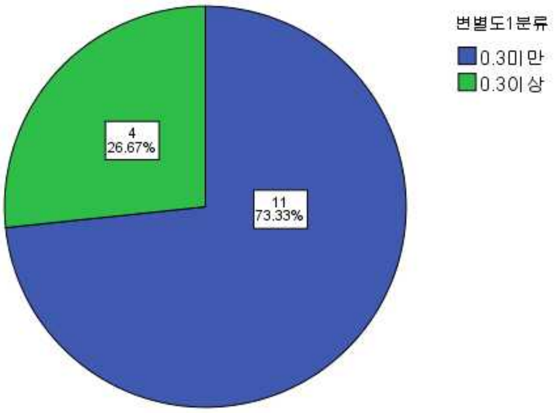

총점	변별도1	표준편차
15	.19	.14

변별도1	문항수	비율(%)
0.3미만	11	73.3
0.3이상	4	26.7
전체	15	100.0

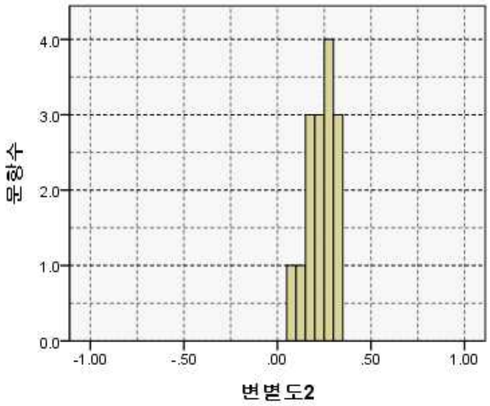

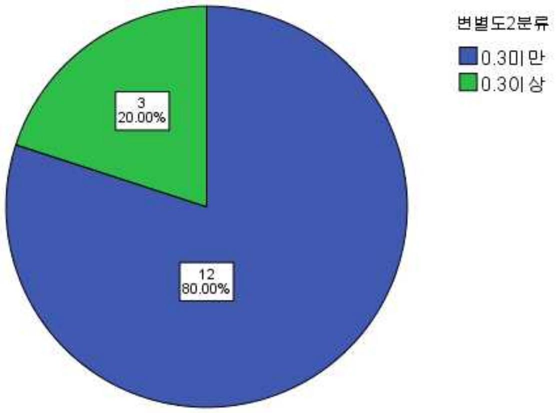

총점	변별도2	표준편차
15	.24	.08

변별도2	문항수	비율(%)
0.3미만	12	80.0
0.3이상	3	20.0
전체	15	100.0

해석

- 치과재료학 과목에서 난이도 지수가 80 에서 100 사이인 문항이 전체 15 문항 중 10 문항이었으며, 차례로 0 에서 60 미만인 문항이 2 문항, 60 이상 80 미만인 문항이 3 문항으로 나타남
- 변별도 1 지수를 기준으로 분류하였을 때, 0.3 미만인 문항이 11 문항으로 0.3 이상인 문항이 4 문항인 것에 비해 더 많이 나타남
- 변별도 2 지수를 기준으로 분류하였을 때, 0.3 미만인 문항이 12 문항으로 0.3 이상인 문항이 3 문항인 것에 비해 더 많이 나타남

(9) 치과교정학 난이도와 변별도 분포도 및 비율분석

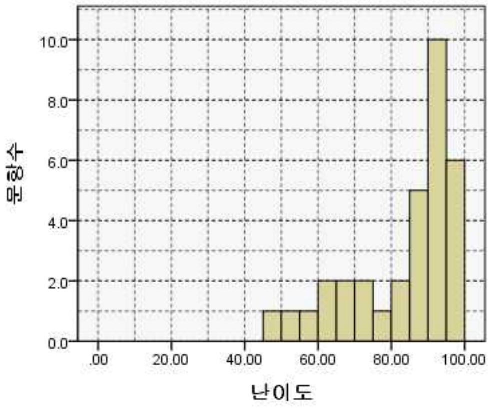

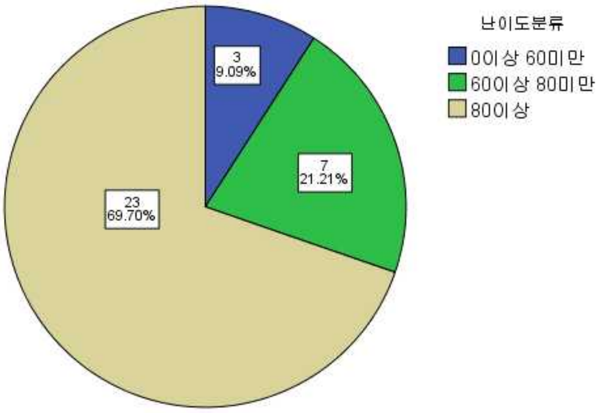

총점	난이도	표준편차
33	83.5	14.1

난이도	문항수	비율(%)
0~60미만	3	9.1
60~80미만	7	21.2
80~100	23	69.7
전체	33	100.0

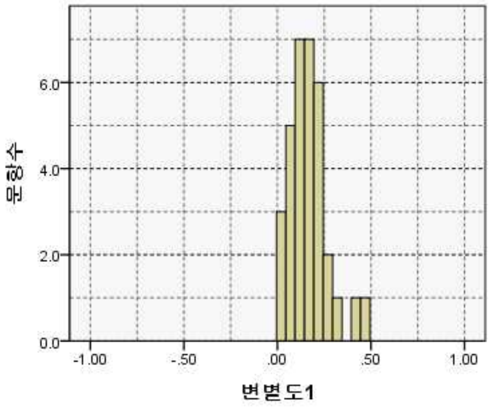

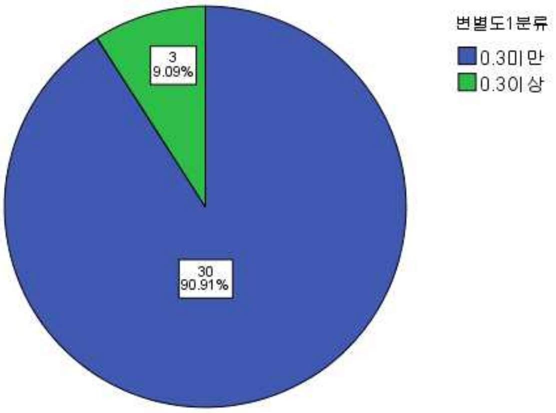

총점	변별도1	표준편차
33	.16	.10

변별도1	문항수	비율(%)
0.3미만	30	90.9
0.3이상	3	9.1
전체	33	100.0

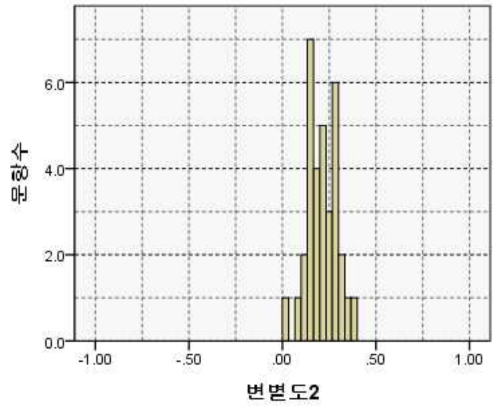

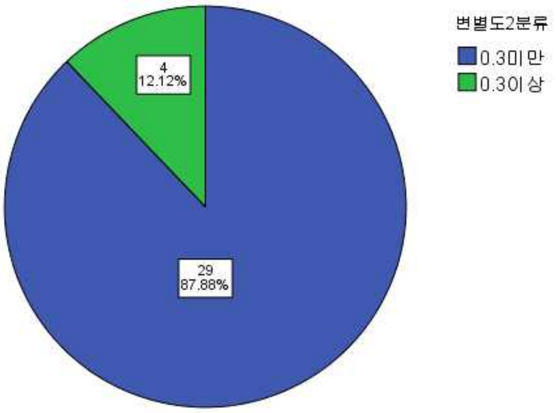

총점	변별도2	표준편차
33	.21	.08

변별도2	문항수	비율(%)
0.3미만	29	87.9
0.3이상	4	12.1
전체	33	100.0

해석

- 치과교정학 과목에서 난이도 지수가 80 에서 100 사이인 문항이 전체 33 문항 중 23 문항이었으며 차례로 60 이상 80 미만인 문항이 7 문항, 0 에서 60 미만인 문항이 3 문항으로 나타남
- 변별도 1 지수를 기준으로 분류하였을 때, 0.3 미만인 문항이 30 문항으로 0.3 이상인 문항이 3 문항인 것에 비해 더 많이 나타남
- 변별도 2 지수를 기준으로 분류하였을 때, 0.3 미만인 문항이 29 문항으로 0.3 이상인 문항이 4 문항인 것에 비해 더 많이 나타남

(10) 구강병리학 난이도와 변별도 분포도 및 비율분석

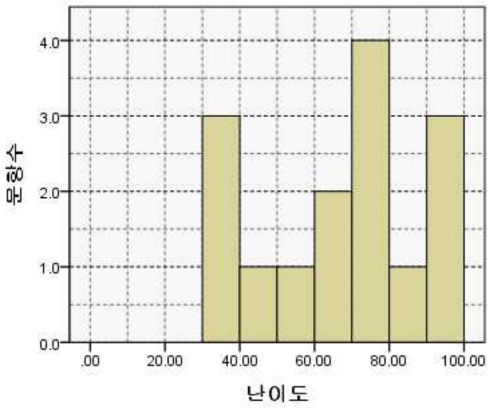

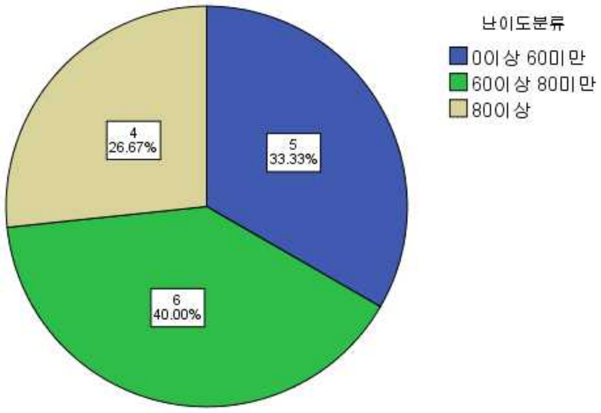

총점	난이도	표준편차
15	67.3	21.2

난이도	문항수	비율(%)
0~60미만	5	33.3
60~80미만	6	40.0
80~100	4	26.7
전체	15	100.0

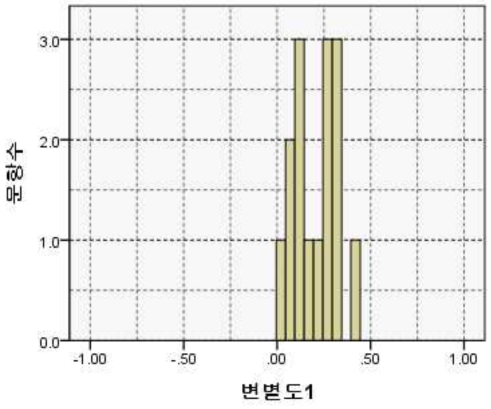

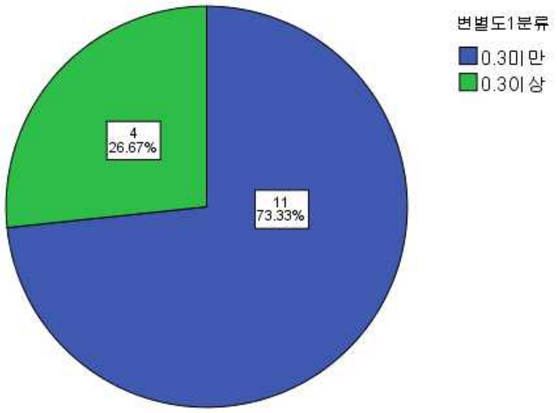

총점	변별도1	표준편차
15	.21	.12

변별도1	문항수	비율(%)
0.3미만	11	73.3
0.3이상	4	26.7
전체	15	100.0

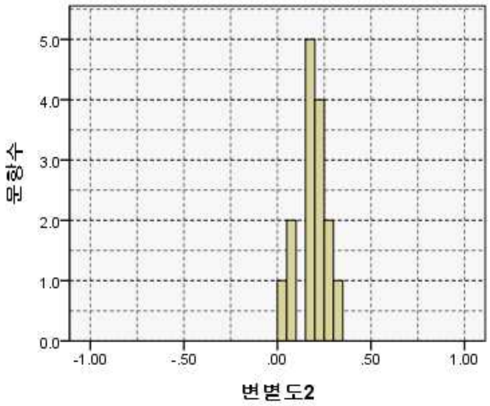

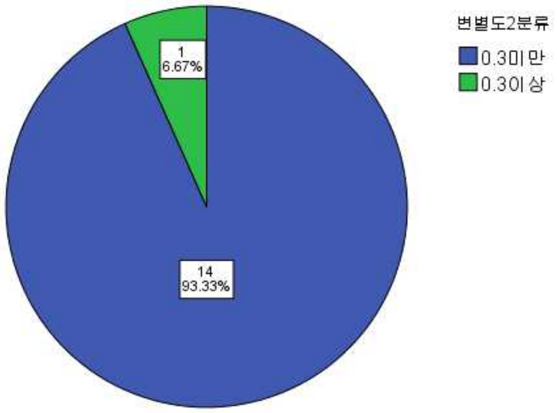

총점	변별도2	표준편차
15	.19	.08

변별도2	문항수	비율(%)
0.3미만	14	93.3
0.3이상	1	6.7
전체	15	100.0

해석

- 구강병리학 과목에서 난이도 지수가 80에서 100 사이인 문항이 전체 15문항 중 4문항이었으며, 차례로 0에서 60 미만인 문항이 6문항, 60 이상 80 미만인 문항이 5문항으로 나타남
- 변별도1 지수를 기준으로 분류하였을 때, 0.3 미만인 문항이 11문항으로 0.3 이상인 문항이 4문항인 것에 비해 더 많이 나타남
- 변별도2 지수를 기준으로 분류하였을 때, 0.3 미만인 문항이 14문항으로 0.3 이상인 문항이 1문항인 것에 비해 더 많이 나타남

(11) 구강보건학 난이도와 변별도 분포도 및 비율분석

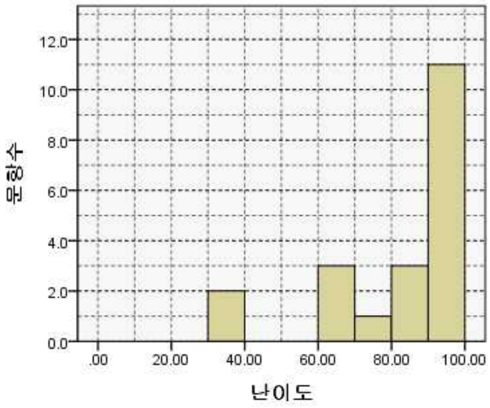

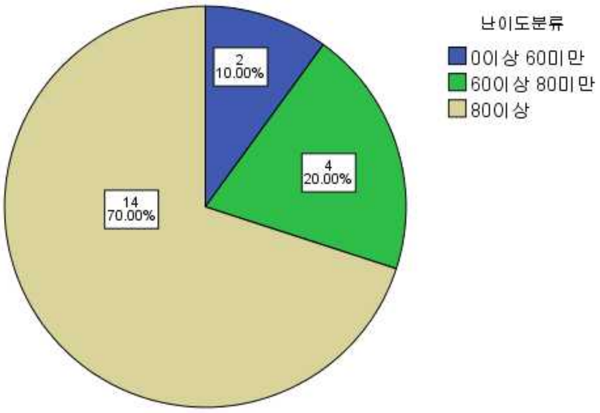

총점	난이도	표준편차
20	82.8	18.7

난이도	문항수	비율(%)
0~60미만	2	10.0
60~80미만	4	20.0
80~100	14	70.0
전체	20	100.0

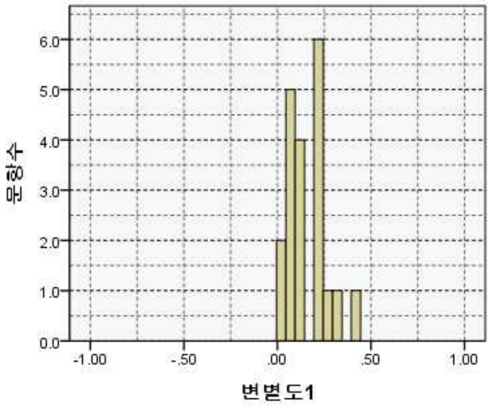

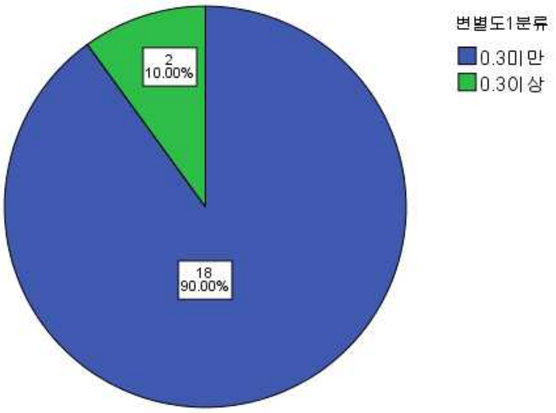

총점	변별도1	표준편차
20	.16	.11

변별도1	문항수	비율(%)
0.3미만	18	90.0
0.3이상	2	10.0
전체	20	100.0

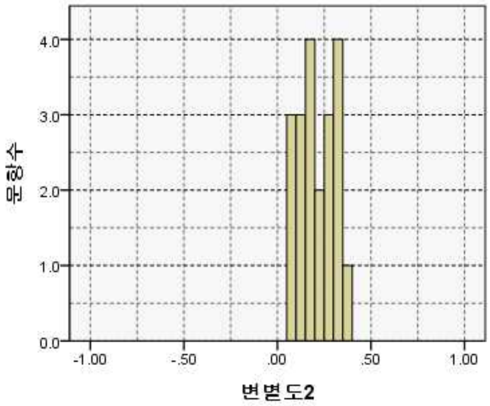

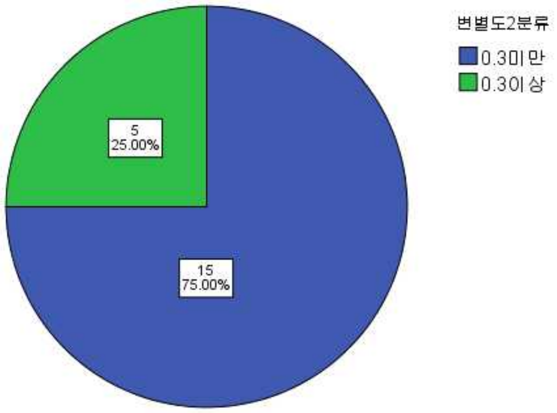

총점	변별도2	표준편차
20	.21	.10

변별도2	문항수	비율(%)
0.3미만	15	75.0
0.3이상	5	25.0
전체	20	100.0

해석

- 구강보건학 과목에서 난이도 지수가 80 에서 100 사이인 문항이 전체 20 문항 중 14 문항이었으며 차례로 0 에서 60 미만인 문항이 4 문항, 60 이상 80 미만인 문항이 2 문항으로 나타남
- 변별도 1 지수를 기준으로 분류하였을 때, 0.3 미만인 문항이 18 문항으로 0.3 이상인 문항이 2 문항인 것에 비해 더 많이 나타남
- 변별도 2 지수를 기준으로 분류하였을 때, 0.3 미만인 문항이 15 문항으로 0.3 이상인 문항이 5 문항인 것에 비해 더 많이 나타남

(12) 구강생물학 난이도와 변별도 분포도 및 비율분석

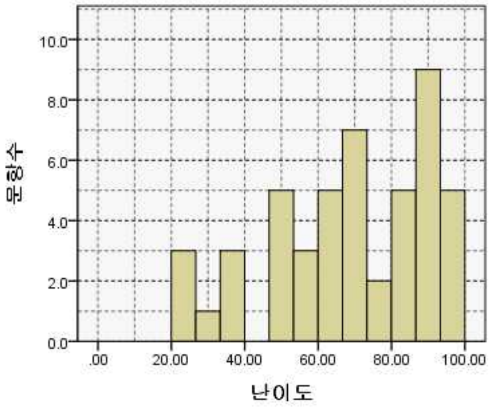

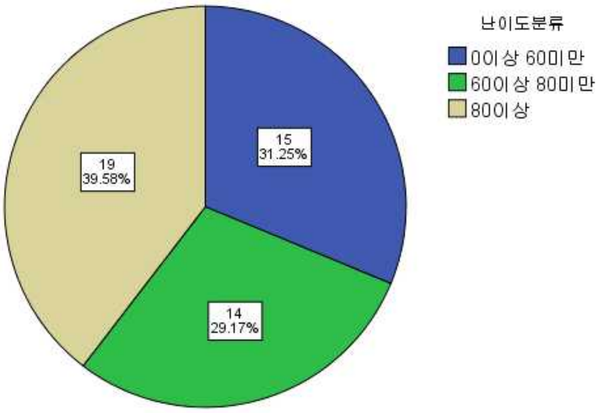

총점	난이도	표준편차
48	68.7	22.1

난이도	문항수	비율(%)
0~60미만	15	31.3
60~80미만	14	29.2
80~100	19	39.6
전체	48	100.0

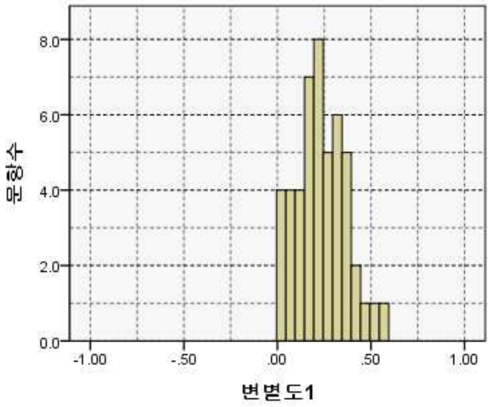

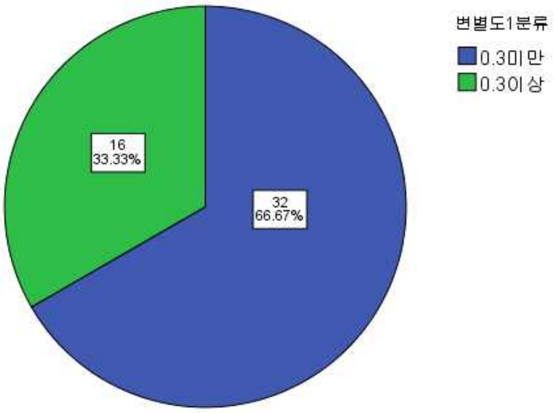

총점	변별도1	표준편차
48	.24	.14

변별도1	문항수	비율(%)
0.3미만	32	66.7
0.3이상	16	33.3
전체	48	100.0

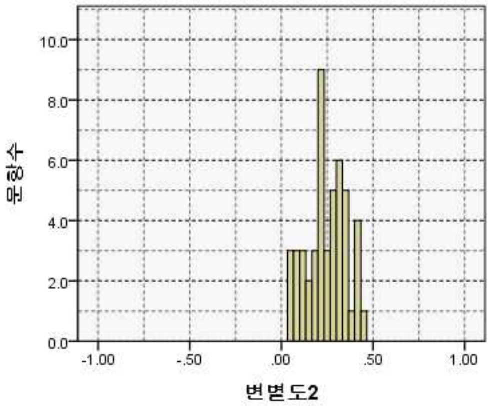

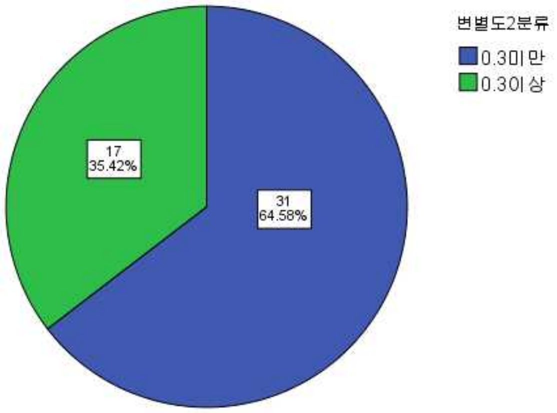

총점	변별도2	표준편차
48	.25	.11

변별도2	문항수	비율(%)
0.3미만	31	64.6
0.3이상	17	35.4
전체	48	100.0

해석

- 구강생물학 과목에서 난이도 지수가 80에서 100사이인 문항인 문항이 전체 48문항 중 19문항이었으며 차례로 0에서 60미만이 14문항, 60이상 80미만인 문항이 15문항으로 나타남
- 변별도1 지수를 기준으로 분류하였을 때, 0.3미만인 문항이 32문항으로 0.3이상인 문항이 16문항인 것에 비해 더 많이 나타남
- 변별도2 지수를 기준으로 분류하였을 때, 0.3미만인 문항이 31문항으로 0.3이상인 문항이 17문항인 것에 비해 더 많이 나타남

(13) 보건의약관계법규 난이도와 변별도 분포도 및 비율분석

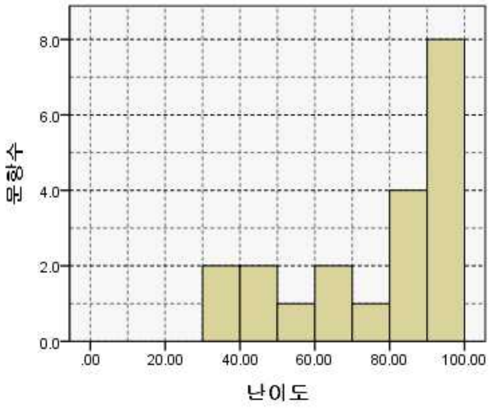

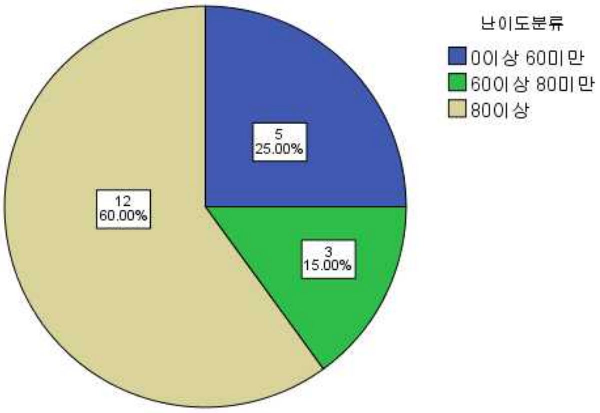

총점	난이도	표준편차
20	76.5	22.6

난이도	문항수	비율(%)
0~60미만	5	25.0
60~80미만	3	15.0
80~100	12	60.0
전체	20	100.0

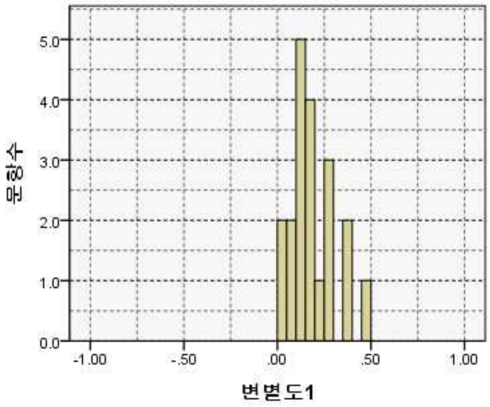

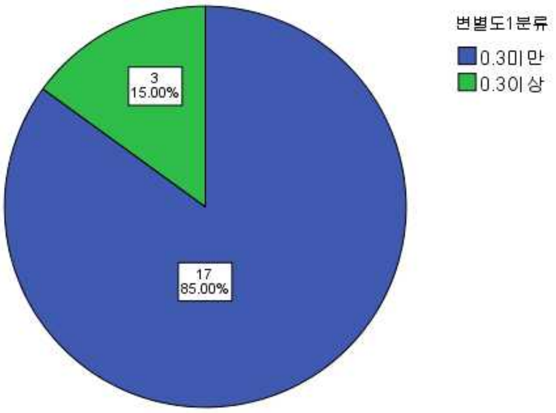

총점	변별도1	표준편차
20	.18	.12

변별도1	문항수	비율(%)
0.3미만	17	85.0
0.3이상	3	15.0
전체	20	100.0

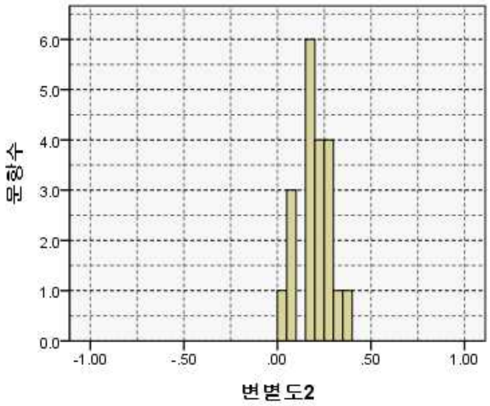

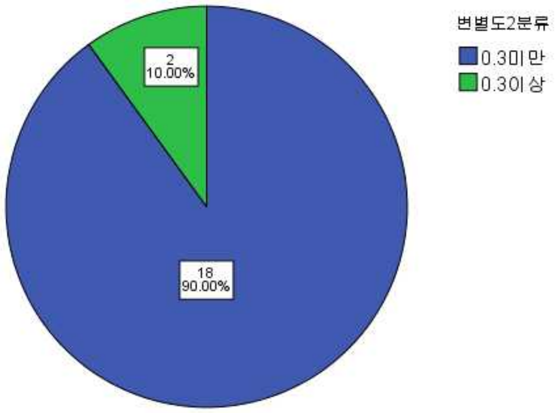

총점	변별도2	표준편차
20	.20	.09

변별도2	문항수	비율(%)
0.3미만	18	90.0
0.3이상	2	10.0
전체	20	100.0

해석

- 보건의약관계법규 과목에서 난이도 지수가 80 에서 100 사이인 문항이 전체 20 문항 중 12 문항이었으며 차례로 60 이상 80 미만인 문항이 3 문항, 0 에서 60 미만인 문항이 5 문항으로 나타남
- 변별도 1 지수를 기준으로 분류하였을 때, 0.3 미만인 문항이 17 문항으로 0.3 이상인 문항이 3 문항인 것에 비해 더 많이 나타남
- 변별도 2 지수를 기준으로 분류하였을 때, 0.3 미만인 문항이 18 문항으로 0.3 이상인 문항이 2 문항인 것에 비해 더 많이 나타남

3) 지식수준별 난이도와 변별도

가) 전회 대비 지식수준별 난이도와 변별도

(1) 전회 대비 암기형 난이도와 변별도

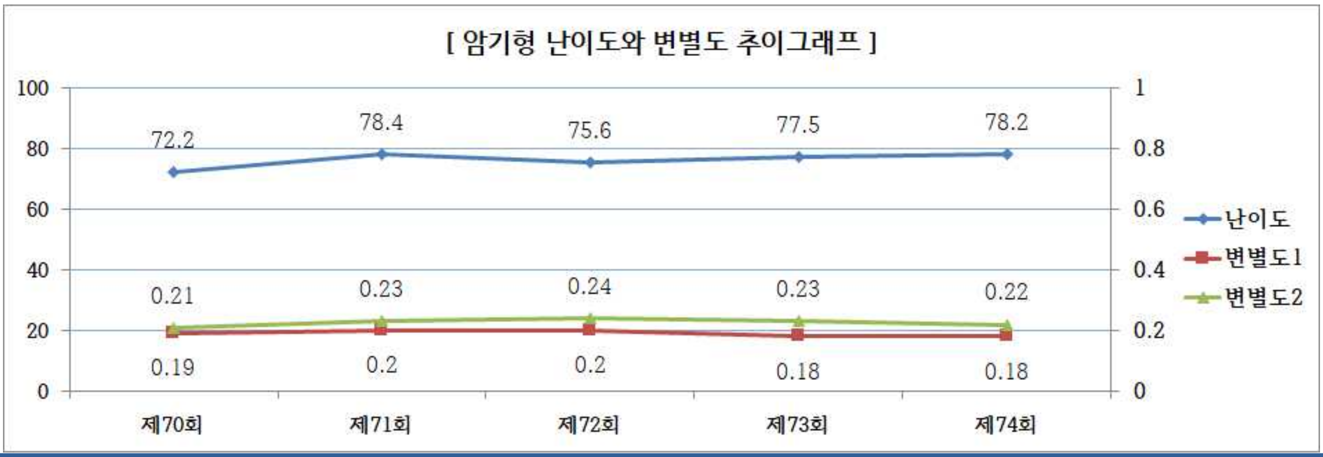

회차	난이도		변별도1		변별도2	
	평균	표준편차	평균	표준편차	평균	표준편차
제70회	72.2	22.8	.19	.12	.21	.09
제71회	78.4	19.0	.20	.13	.23	.10
제72회	75.6	22.1	.20	.12	.24	.08
제73회	77.5	20.9	.18	.13	.23	.11
제74회	78.2	20.1	.18	.11	.22	.09

해석

- 전회 대비 암기형 문항의 난이도 지수는 0.7 증가함
- 전회 대비 암기형 문항의 변별도 1 지수는 변하지 않음
- 전회 대비 암기형 문항의 변별도 2 지수는 .01 감소함

(2) 전회 대비 해석형 난이도와 변별도

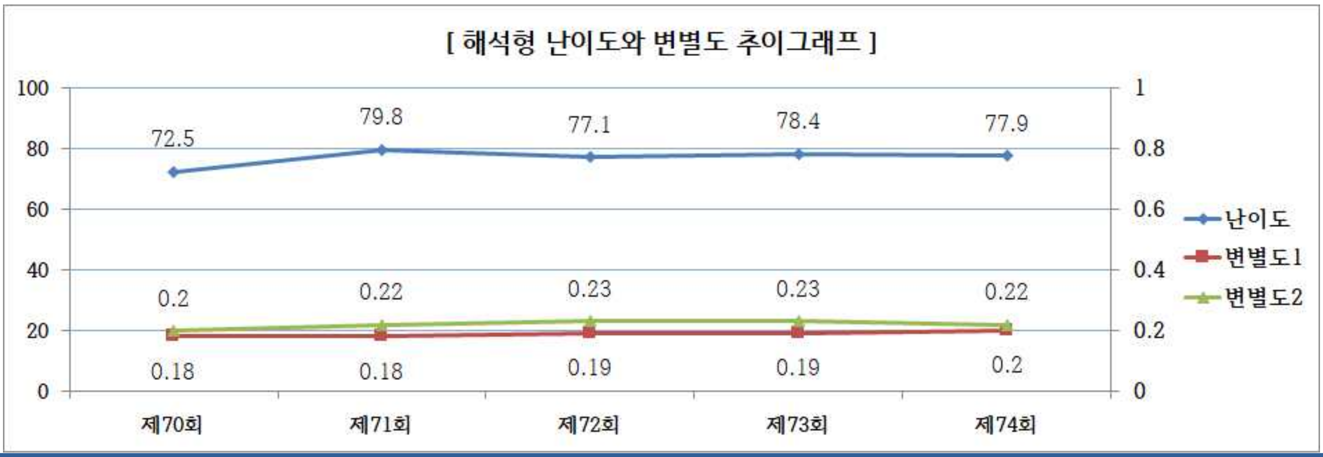

회차	난이도		변별도1		변별도2	
	평균	표준편차	평균	표준편차	평균	표준편차
제70회	72.5	22.5	.18	.12	.20	.09
제71회	79.8	18.1	.18	.11	.22	.08
제72회	77.1	19.9	.19	.12	.23	.09
제73회	78.4	20.8	.19	.13	.23	.10
제74회	77.9	18.6	.20	.13	.22	.10

해석

- 전회 대비 해석형 문항의 난이도 지수는 0.5 감소함
- 전회 대비 해석형 문항의 변별도 1 지수는 .01 증가함
- 전회 대비 해석형 문항의 변별도 2 지수는 .01 감소함

(3) 전회 대비 해결형 난이도와 변별도

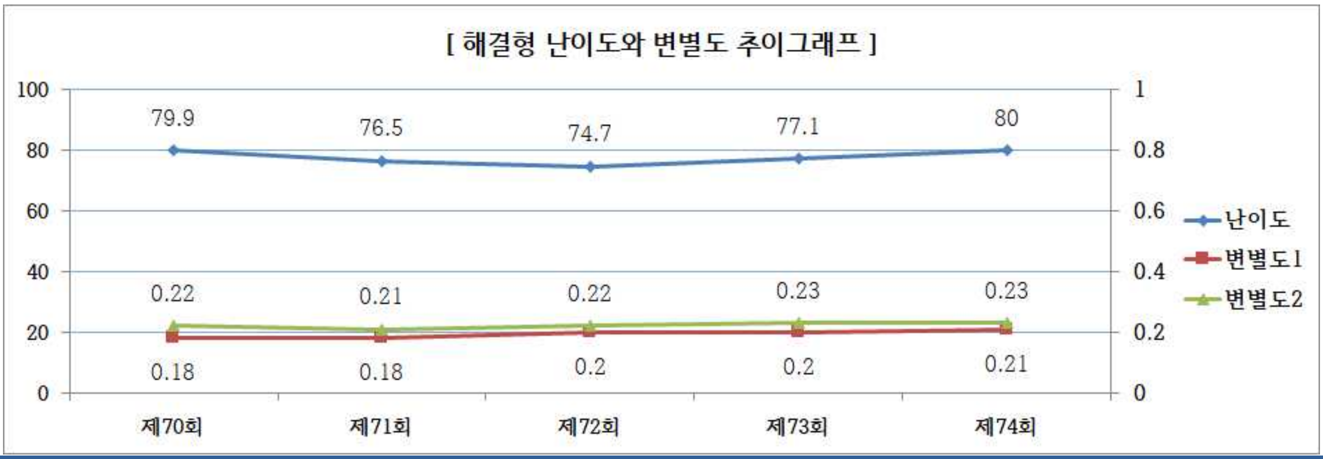

회차	난이도		변별도1		변별도2	
	평균	표준편차	평균	표준편차	평균	표준편차
제70회	79.9	15.8	.18	.10	.22	.08
제71회	76.5	21.5	.18	.12	.21	.09
제72회	74.7	18.6	.20	.13	.22	.10
제73회	77.1	17.7	.20	.13	.23	.10
제74회	80.0	18.1	.21	.13	.23	.09

해석

- 전회 대비 해결형 문항의 난이도 지수는 2.9 증가함
- 전회 대비 해결형 문항의 변별도 1 지수는 .01 증가함
- 전회 대비 해결형 문항의 변별도 2 지수는 변하지 않음

나) 지식수준별 난이도와 변별도 분포도 및 비율분석

(1) 암기형 난이도와 변별도 분포도 및 비율분석

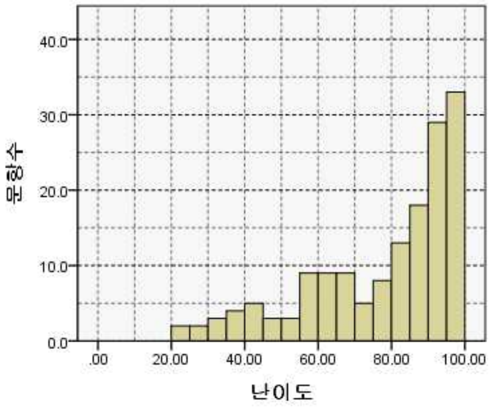

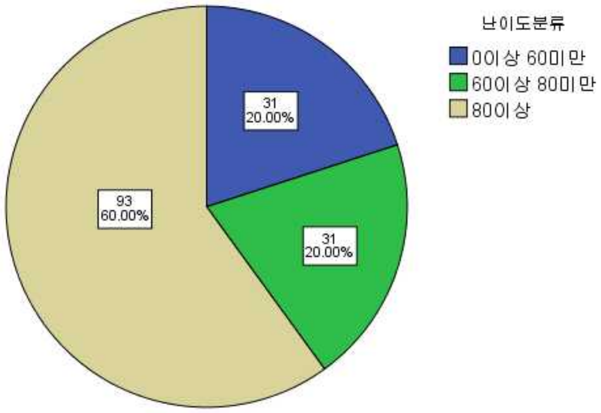

총점	난이도	표준편차
155	78.2	20.1

난이도	문항수	비율(%)
0~60미만	31	20.0
60~80미만	31	20.0
80~100	93	60.0
전체	155	100.0

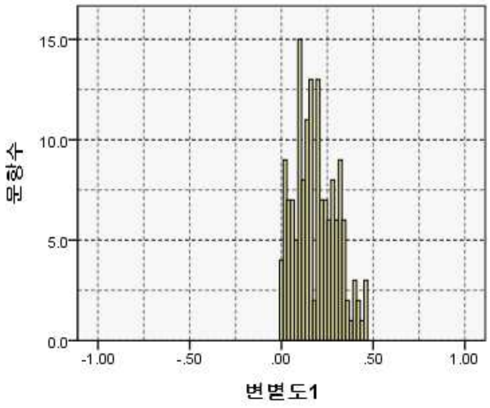

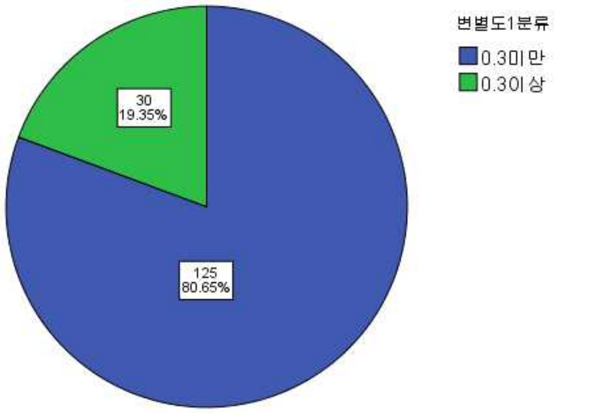

총점	변별도1	표준편차
155	.18	.11

변별도1	문항수	비율(%)
0.3미만	125	80.6
0.3이상	30	19.4
전체	155	100.0

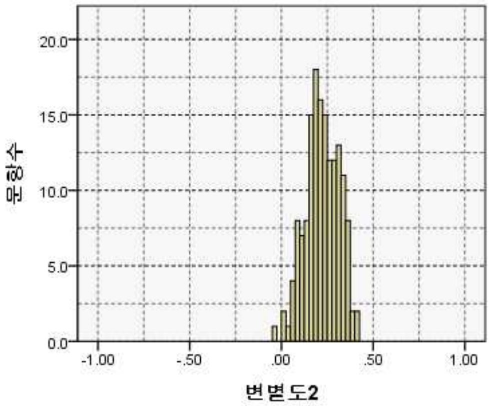

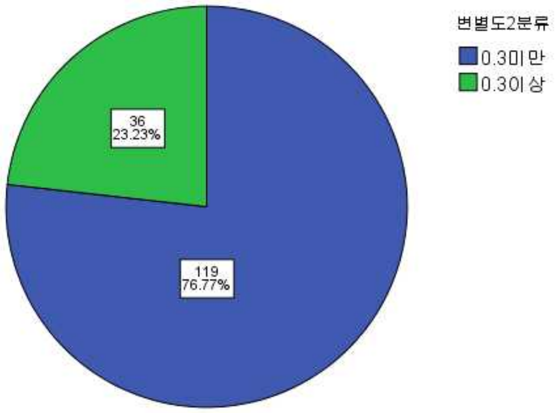

총점	변별도2	표준편차
155	.22	.09

변별도2	문항수	비율(%)
0.3미만	119	76.8
0.3이상	36	23.2
전체	155	100.0

해석

- 암기형 문항에서 난이도 지수가 80 에서 100 사이인 문항이 전체 155 문항 중 93 문항이었으며, 60 이상 80 미만인 문항이 31 문항, 0 에서 60 미만인 문항이 31 문항으로 나타남
- 변별도 1 지수를 기준으로 분류하였을 때, 0.3 미만인 문항이 125 문항으로 0.3 이상인 문항이 30 문항인 것에 비해 더 많이 나타남
- 변별도 2 지수를 기준으로 분류하였을 때, 0.3 미만인 문항이 119 문항으로 0.3 이상인 문항이 36 문항인 것에 비해 더 많이 나타남

(2) 해석형 난이도와 변별도 분포도 및 비율분석

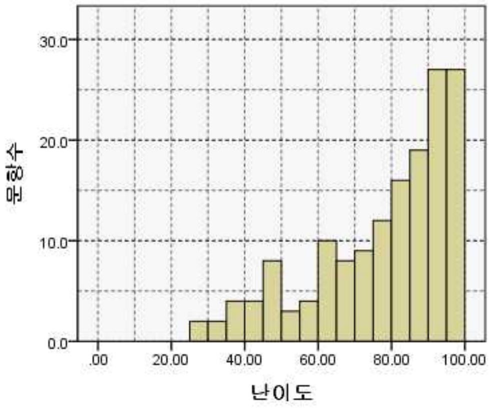

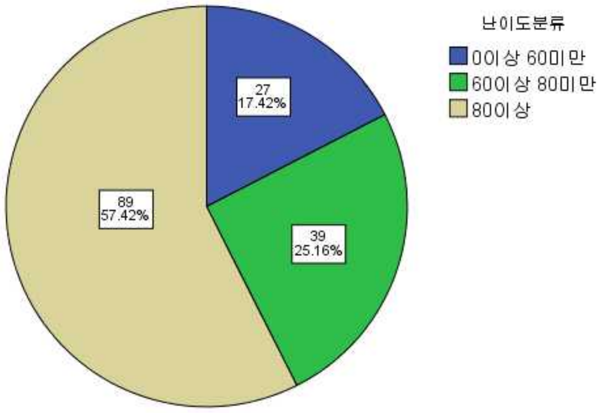

총점	난이도	표준편차
155	77.9	18.6

난이도	문항수	비율(%)
0~60미만	27	17.4
60~80미만	39	25.2
80~100	89	54.7
전체	155	100.0

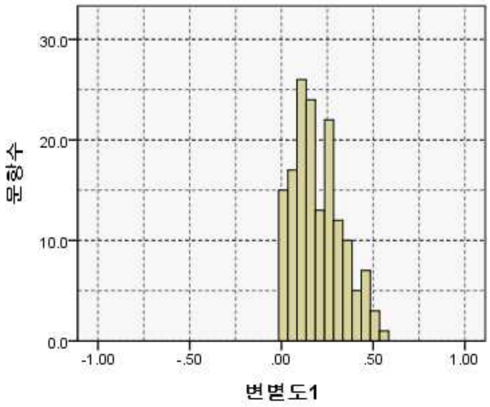

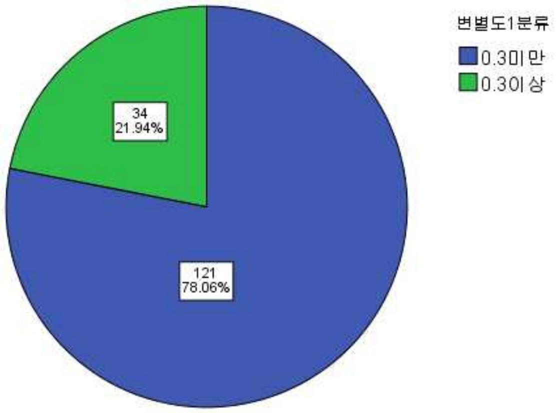

총점	변별도1	표준편차
155	.20	.13

변별도1	문항수	비율(%)
0.3미만	121	78.1
0.3이상	34	21.9
전체	155	100.0

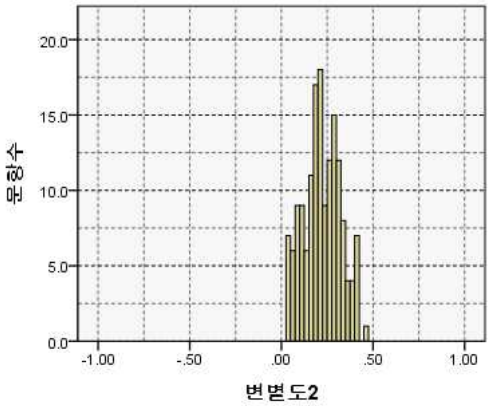

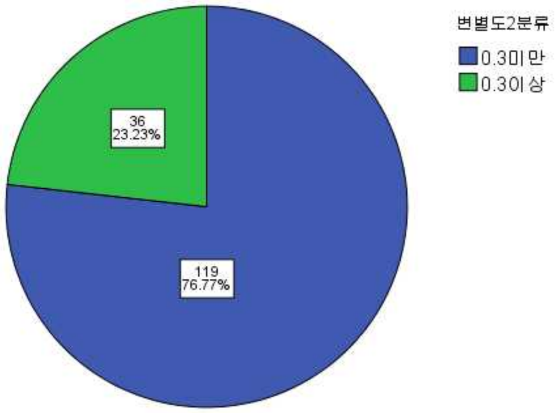

총점	변별도2	표준편차
155	.22	.10

변별도2	문항수	비율(%)
0.3미만	119	76.8
0.3이상	36	23.2
전체	155	100.0

해석

- 해석형 문항에서 난이도 지수가 80 에서 100 사이인 문항이 전체 155 문항 중 89 문항이었으며, 60 이상 80 미만인 문항이 39 문항, 0 에서 60 미만인 문항이 27 문항으로 나타남
- 변별도 1 지수를 기준으로 분류하였을 때, 0.3 미만인 문항이 121 문항으로 0.3 이상인 문항이 34 문항인 것에 비해 더 많이 나타남
- 변별도 2 지수를 기준으로 분류하였을 때, 0.3 미만인 문항이 119 문항으로 0.3 이상인 문항이 36 문항인 것에 비해 더 많이 나타남

(3) 해결형 난이도와 변별도 분포도 및 비율분석

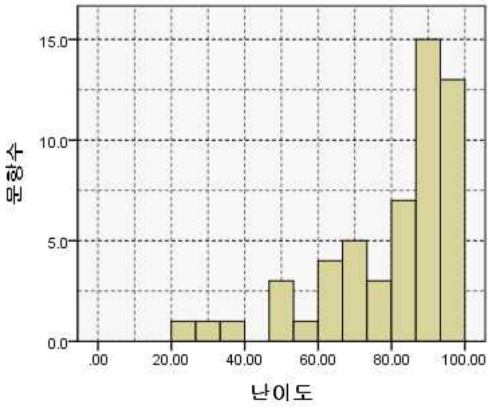

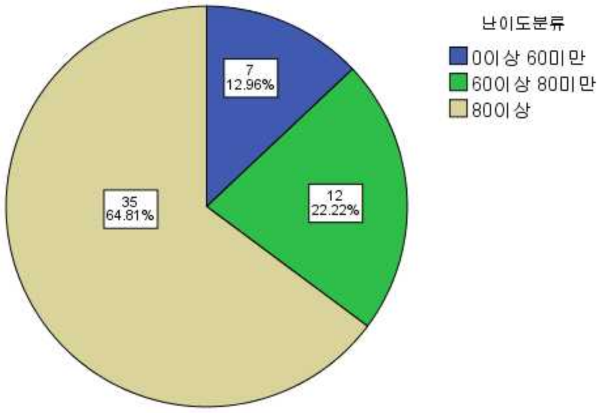

총점	난이도	표준편차
54	80.0	18.1

난이도	문항수	비율(%)
0~60미만	7	13.0
60~80미만	12	22.2
80~100	35	64.8
전체	54	100.0

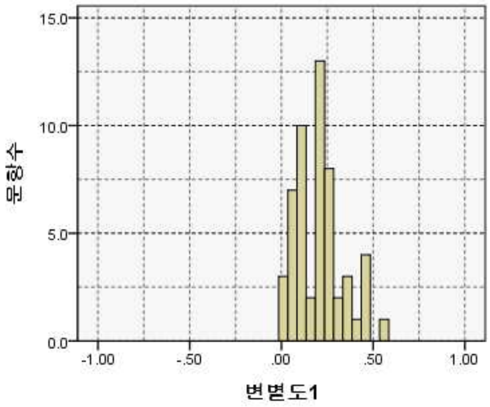

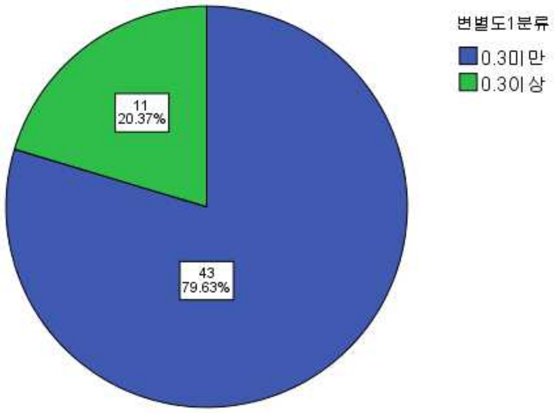

총점	변별도1	표준편차
54	.21	.13

변별도1	문항수	비율(%)
0.3미만	43	79.6
0.3이상	11	20.4
전체	54	100.0

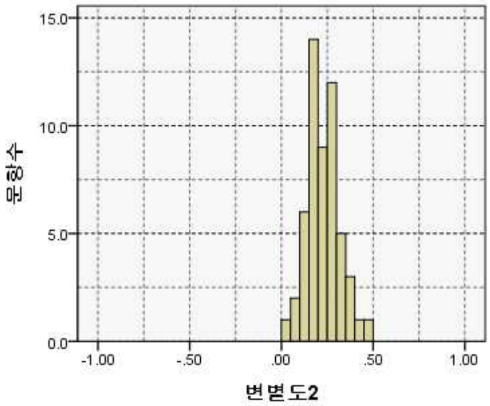

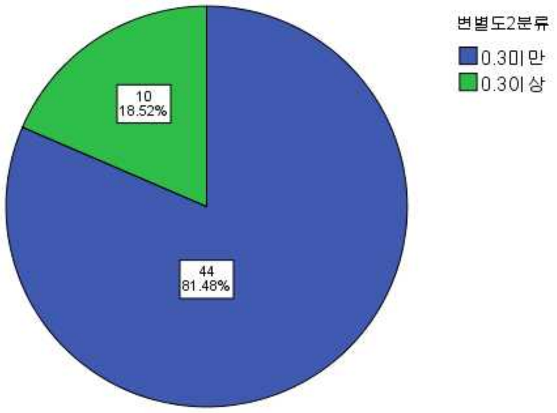

총점	변별도2	표준편차
54	.23	.09

변별도2	문항수	비율(%)
0.3미만	44	81.5
0.3이상	10	18.5
전체	54	100.0

해석

- 해결형 문항에서 난이도 지수가 80 에서 100 사이인 문항이 전체 54 문항 중 35 문항이었으며, 60 이상 80 미만인 문항이 12 문항, 0 에서 60 사이인 문항이 7 문항으로 나타남
- 변별도 1 지수를 기준으로 분류하였을 때, 0.3 미만인 문항이 43 문항으로 0.3 이상인 문항이 11 문항인 것에 비해 더 많이 나타남
- 변별도 2 지수를 기준으로 분류하였을 때, 0.3 미만인 문항이 44 문항으로 0.3 이상인 문항이 10 문항인 것에 비해 더 많이 나타남

3. 난이도와 변별도 간 산포도

1) 전체 난이도와 변별도 간 산포도

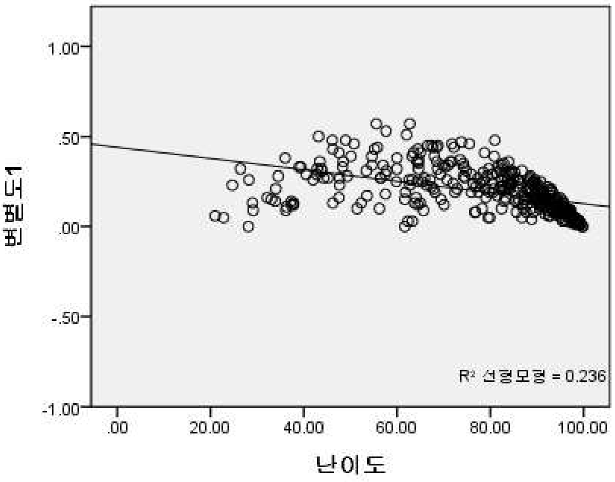

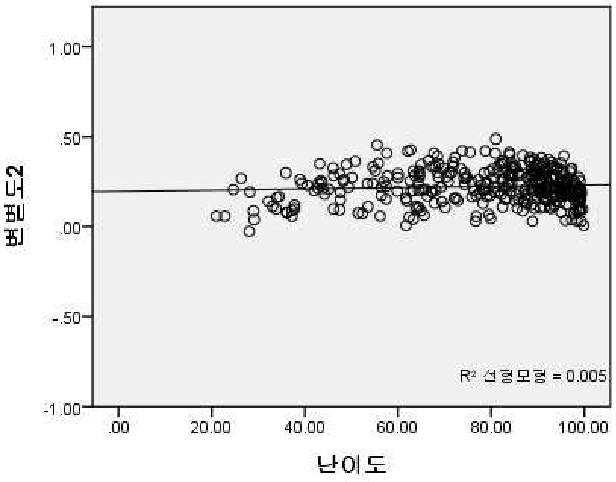

해석

- 전체 문항을 대상으로 난이도와 변별도 1 지수 간 상관은 -.485*로 난이도 지수가 높을수록 변별력이 낮아지는 것으로 나타남
- 난이도와 변별도 2 지수 간 상관은 .074 로 관련성이 없는 것으로 나타남

2) 과목별 난이도와 변별도 간 산포도

가) 구강악안면외과학 난이도와 변별도 간 산포도

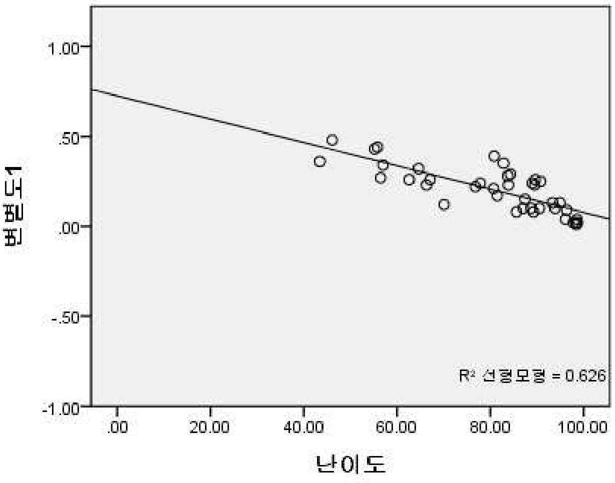

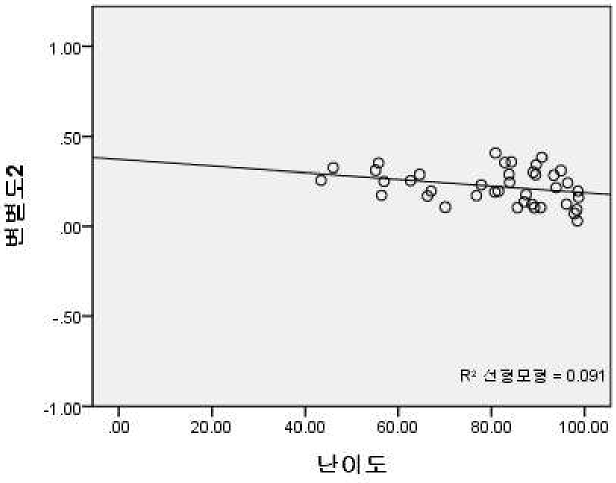

해석

- 구강악안면외과학 과목을 대상으로 난이도와 변별도 1 지수 간 상관은 -.791*로 난이도 지수가 높을수록 변별력이 낮아지는 것으로 나타남
- 난이도와 변별도 2 지수 간 상관은 -.301 로 관련성이 낮은 것으로 나타남

나) 치과보존학 난이도와 변별도 간 산포도

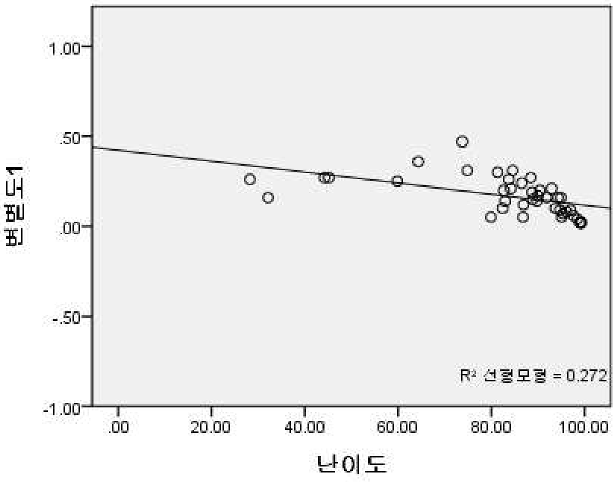

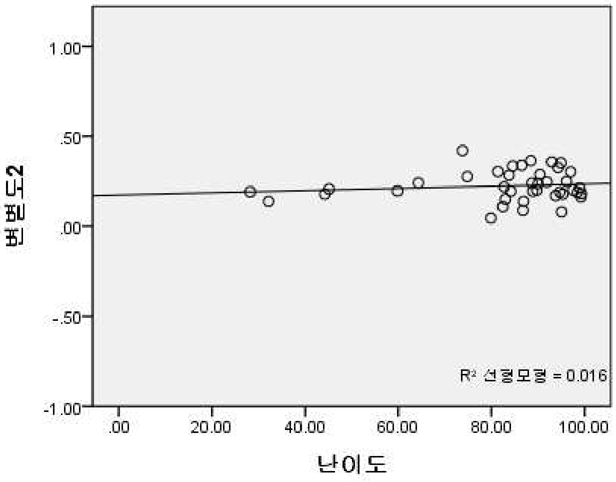

해석

- 치과보존학 과목을 대상으로 난이도와 변별도 1 지수 간 상관은 -.521*로 난이도 지수가 높을수록 변별력이 낮아지는 것으로 나타남
- 난이도와 변별도 2 지수 간 상관은 .128 로 관련성이 없는 것으로 나타남

다) 치과보철학 난이도와 변별도 간 산포도

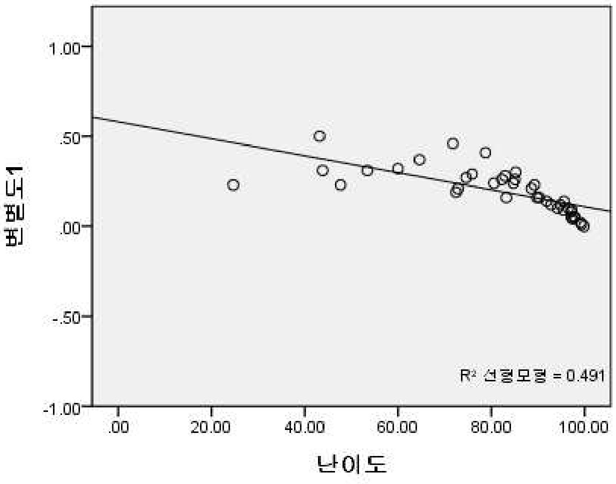

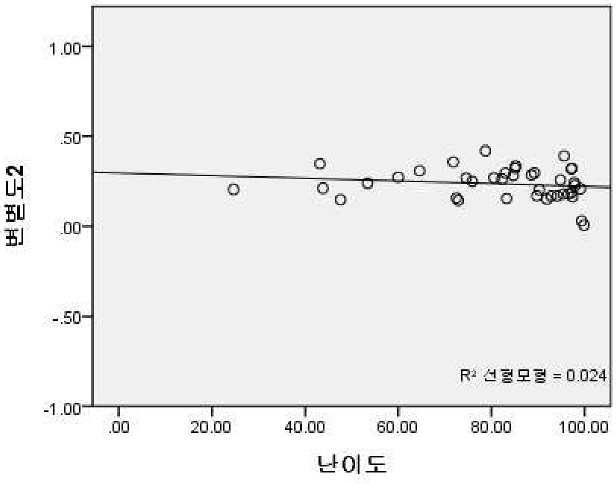

해석

- 치과보철학 과목을 대상으로 난이도와 변별도 1 지수 간 상관은 -.700*으로 난이도 지수가 높을수록 변별력이 낮아지는 것으로 나타남
- 난이도와 변별도 2 지수 간 상관은 -.156 으로 관련성이 없는 것으로 나타남

라) 소아치과학 난이도와 변별도 간 산포도

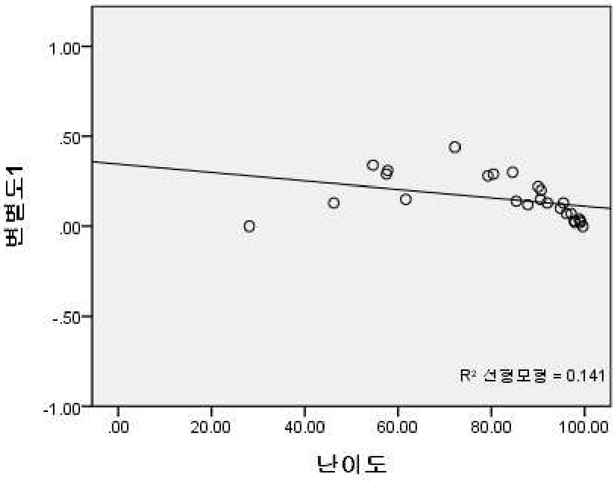

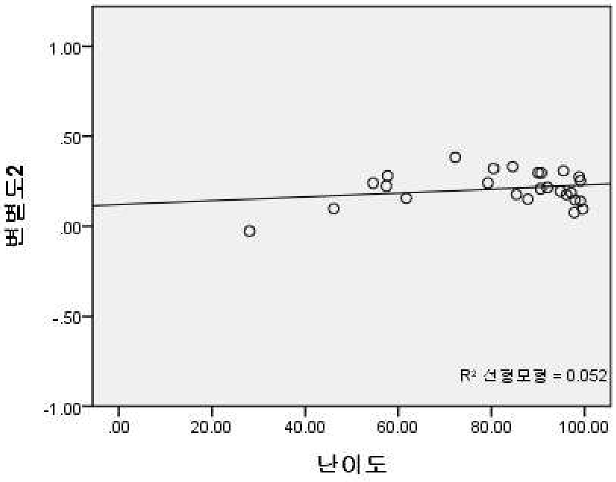

해석

- 소아치과학 과목을 대상으로 난이도와 변별도 1 지수 간 상관은 -.375 로 관련성이 낮은 것으로 나타남
- 난이도와 변별도 2 지수 간 상관은 .227 로 관련성이 없는 것으로 나타남

마) 영상치의학 난이도와 변별도 간 산포도

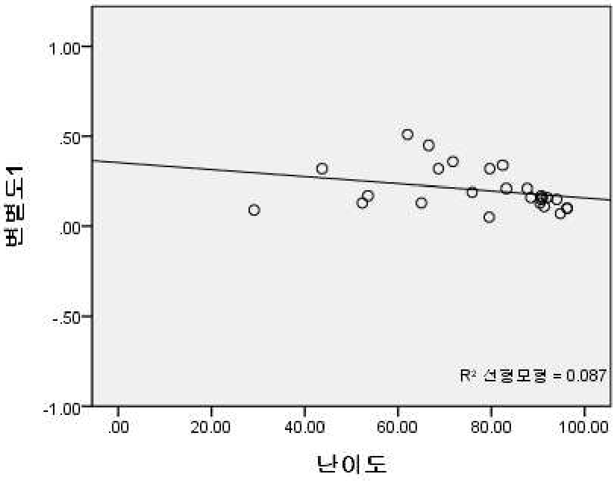

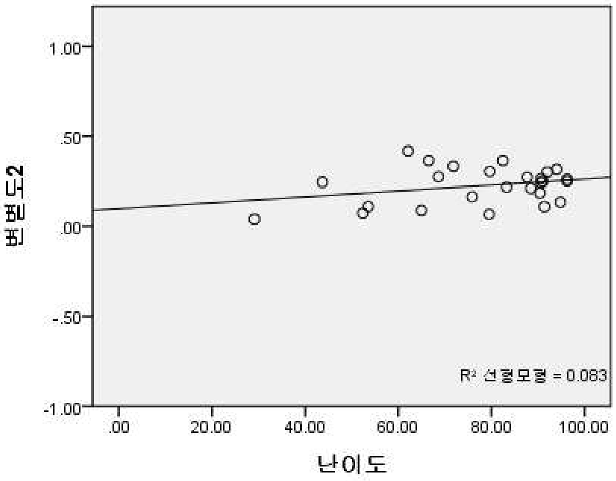

해석

- 영상치의학 과목을 대상으로 난이도와 변별도 1 지수 간 상관은 -.295 로 관련성이 없는 것으로 나타남
- 난이도와 변별도 2 지수 간 상관은 -.288 로 관련성이 없는 것으로 나타남

바) 치주과학 난이도와 변별도 간 산포도

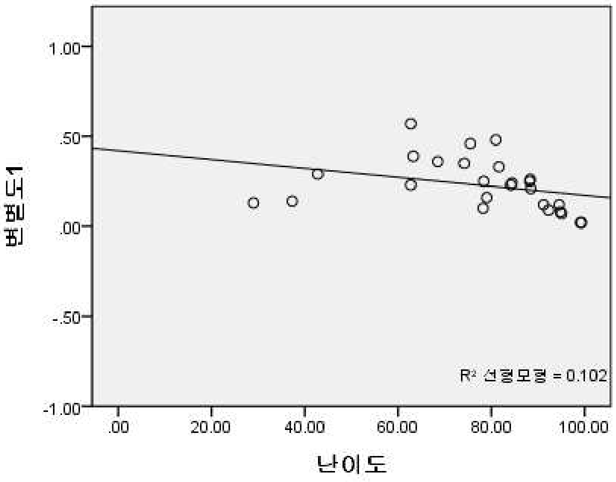

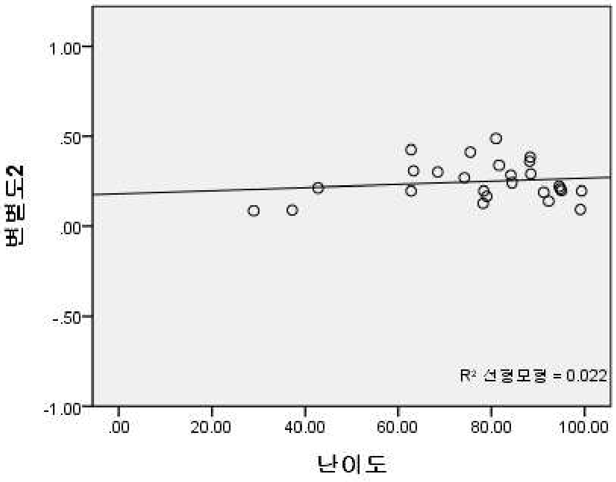

해석

- 치주과학 과목을 대상으로 난이도와 변별도 1 지수 간 상관은 -.319 로 관련성이 낮은 것으로 나타남
- 난이도와 변별도 2 지수 간 상관은 .147 로 관련성이 없는 것으로 나타남

사) 구강내과학 난이도와 변별도 간 산포도

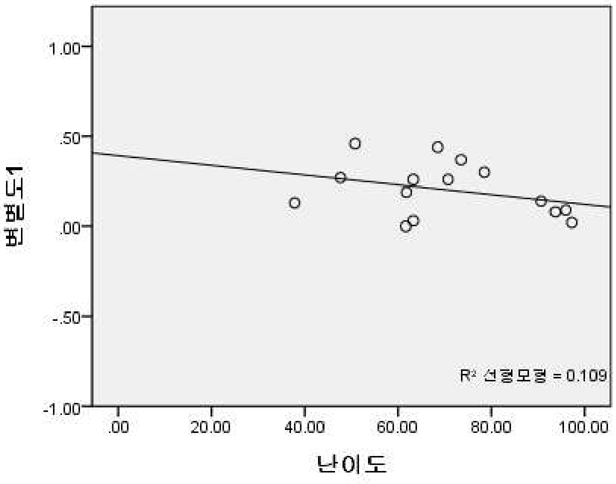

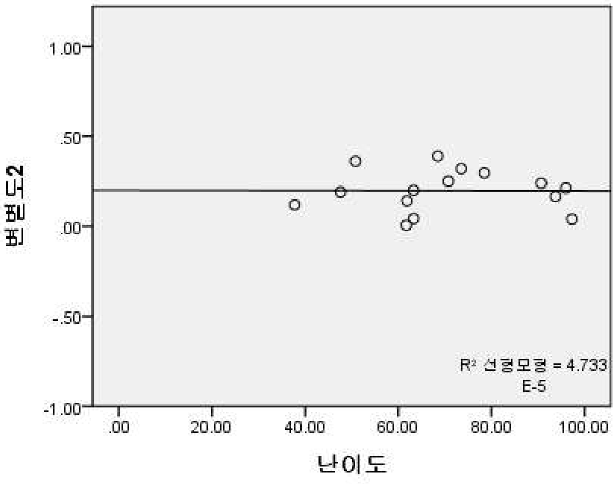

해석

- 구강내과학 과목을 대상으로 난이도와 변별도1 지수 간 상관은 -.330으로 관련성이 낮은 것으로 나타남
- 난이도와 변별도2 지수 간 상관은 -.007로 관련성이 없는 것으로 나타남

아) 치과재료학 난이도와 변별도 간 산포도

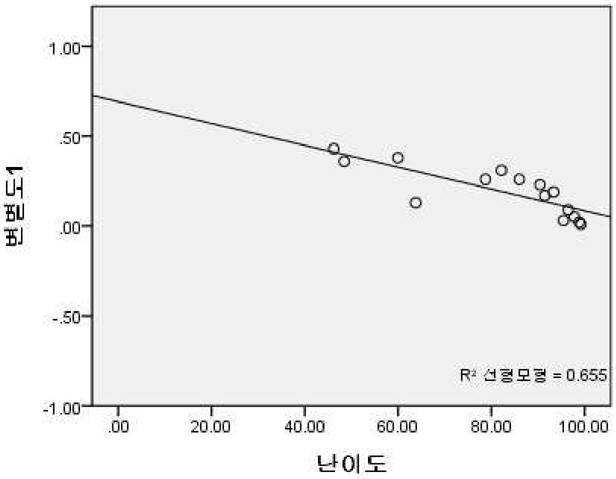

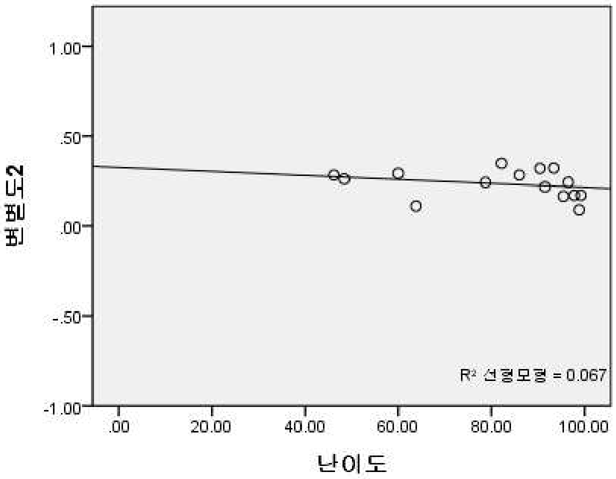

해석

- 치과재료학 과목을 대상으로 난이도와 변별도1 지수 간 상관은 -.809*로 난이도 지수가 높을수록 변별력이 낮아지는 것으로 나타남
- 난이도와 변별도2 지수 간 상관은 -.260로 관련성이 없는 것으로 나타남

자) 치과교정학 난이도와 변별도 간 산포도

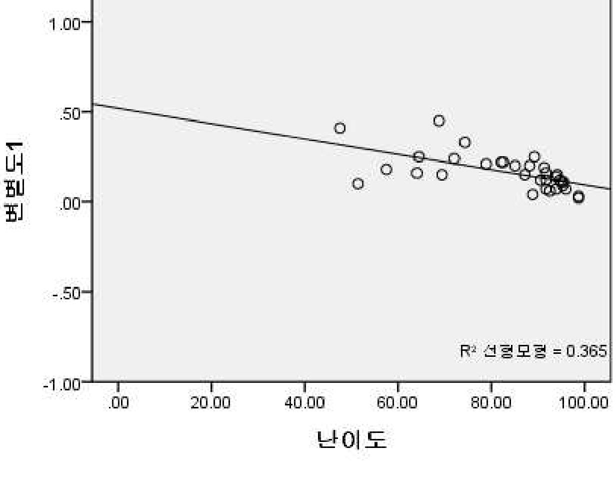

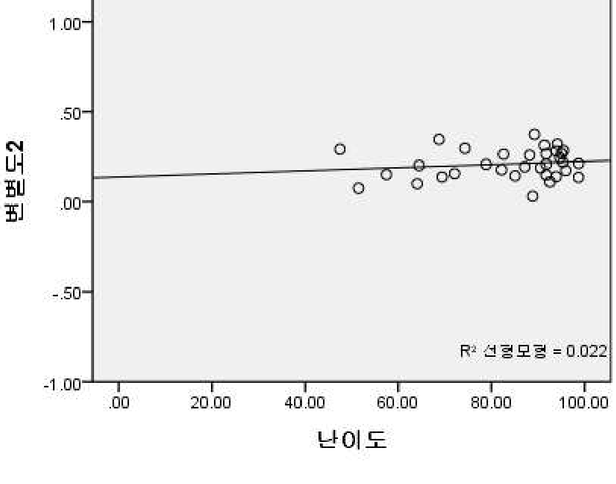

해석

- 치과교정학 과목을 대상으로 난이도와 변별도 1 지수 간 상관은 -.605*로 난이도 지수가 높을수록 변별력이 낮아지는 것으로 나타남
- 난이도와 변별도 2 지수 간 상관은 .153 으로 관련성이 없는 것으로 나타남

차) 구강병리학 난이도와 변별도 간 산포도

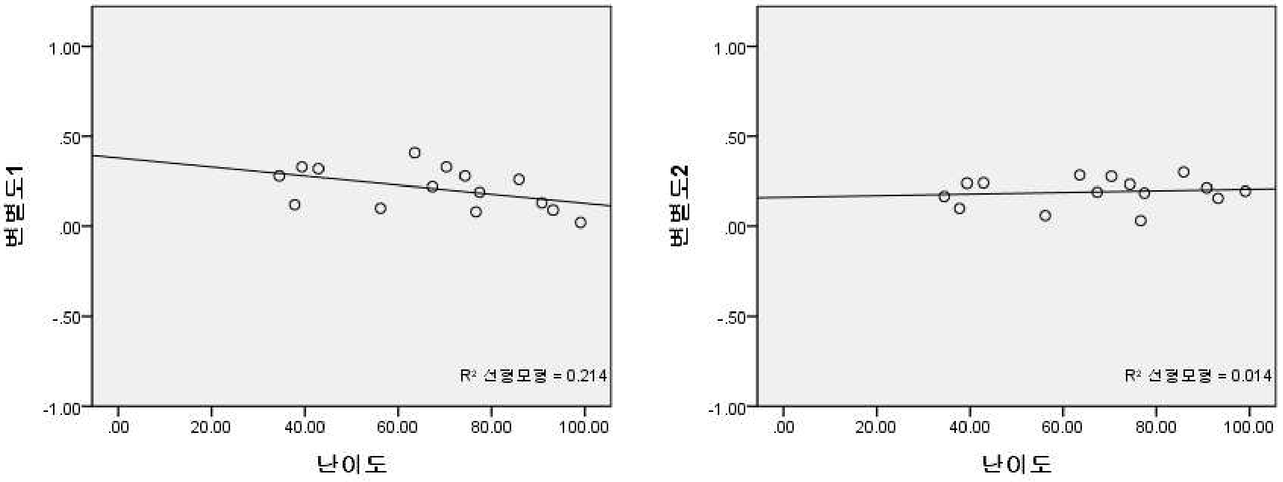

해석

- 구강병리학 과목을 대상으로 난이도와 변별도 1 지수 간 상관은 -.462 로 관련성이 낮은 것으로 나타남
- 난이도와 변별도 2 지수 간 상관은 .119 로 관련성이 없는 것으로 나타남

카) 구강보건학 난이도와 변별도 간 산포도

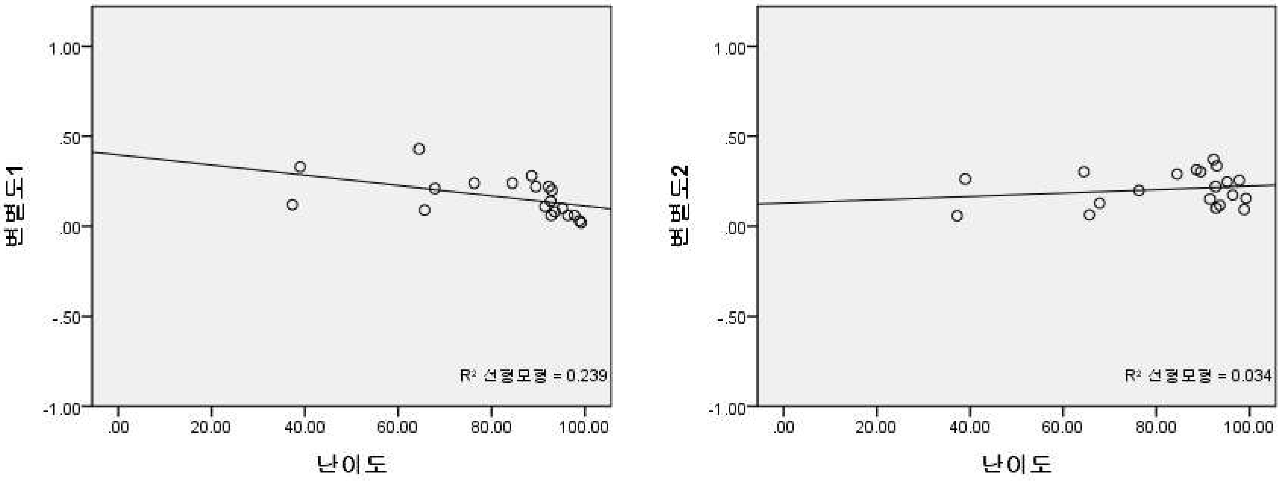

해석
- 구강보건학 과목을 대상으로 난이도와 변별도 1 지수 간 상관은 -.488*로 난이도 지수가 높을수록 변별력이 낮아지는 것으로 나타남<br>- 난이도와 변별도 2 지수 간 상관은 .184 로 관련성이 없는 것으로 나타남

타) 구강생물학 난이도와 변별도 간 산포도

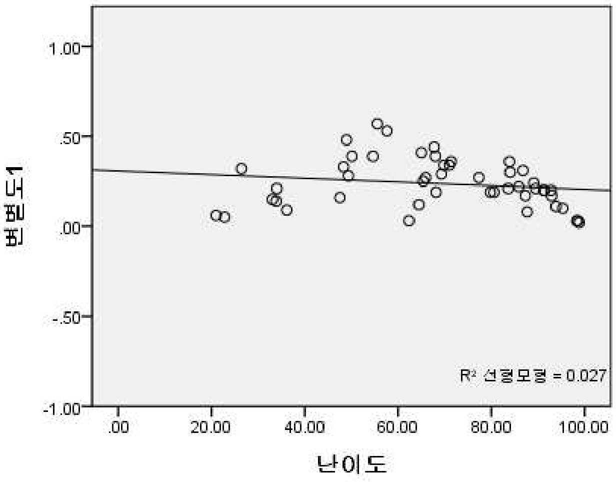

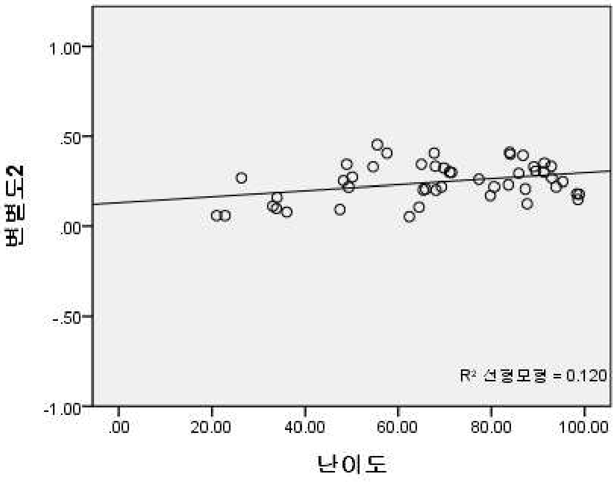

해석
- 구강생물학 과목을 대상으로 난이도와 변별도 1 지수 간 상관은 -.166 으로 관련성이 없는 것으로 나타남<br>- 난이도와 변별도 2 지수 간 상관은 .347*로 난이도 지수가 높을수록 변별력이 높아지는 것으로 나타남

파) 보건의약관계법규 난이도와 변별도 간 산포도

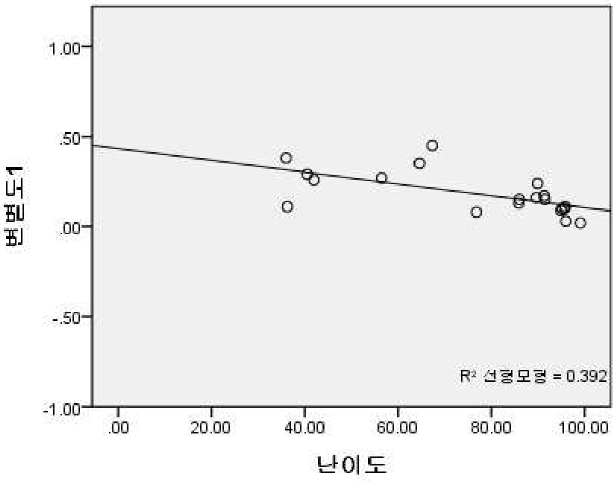

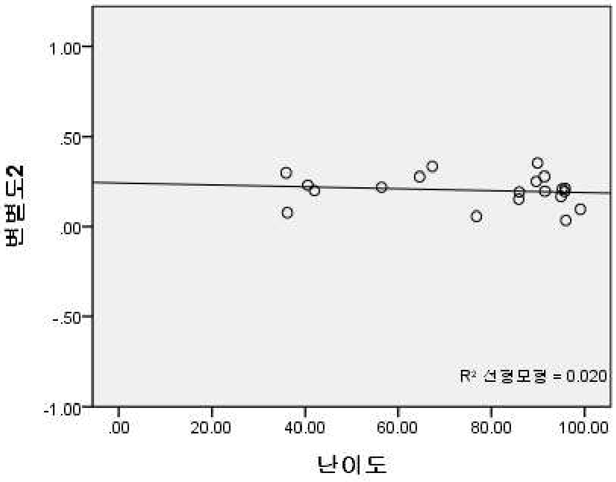

해석

- 보건의학관계법규 과목을 대상으로 난이도와 변별도 1 지수 간 상관은 -.626*로 난이도 지수가 높을수록 변별력이 낮아지는 것으로 나타남
- 난이도와 변별도 2 지수 간 상관은 .140 으로 관련성이 없는 것으로 나타남

4. 신뢰도 분석

과목명	문항수	제70회	제71회	제72회	제73회	제74회
전체	364	.939	.947	.949	.949	.949
구강악안면외과학	40	.703	.728	.733	.645	.700
치과보존학	40	.625	.675	.647	.683	.680
치과보철학	40	.590	.691	.679	.703	.714
소아치과학	26	.522	.609	.559	.639	.546
영상치의학	26	.560	.560	.667	.695	.576
치주과학	26	.571	.571	.533	.570	.645
구강내과학	15	.418	.368	.457	.409	.394
치과재료학	15	.432	.477	.594	.577	.474
치과교정학	33	.575	.631	.660	.603	.610
구강병리학	15	.529	.553	.421	.487	.365
구강보건학	20	.529	.599	.534	.496	.448
구강생물학	48	.701	.730	.736	.743	.776
보건의약관계법규	20	.607	.456	.620	.543	.532

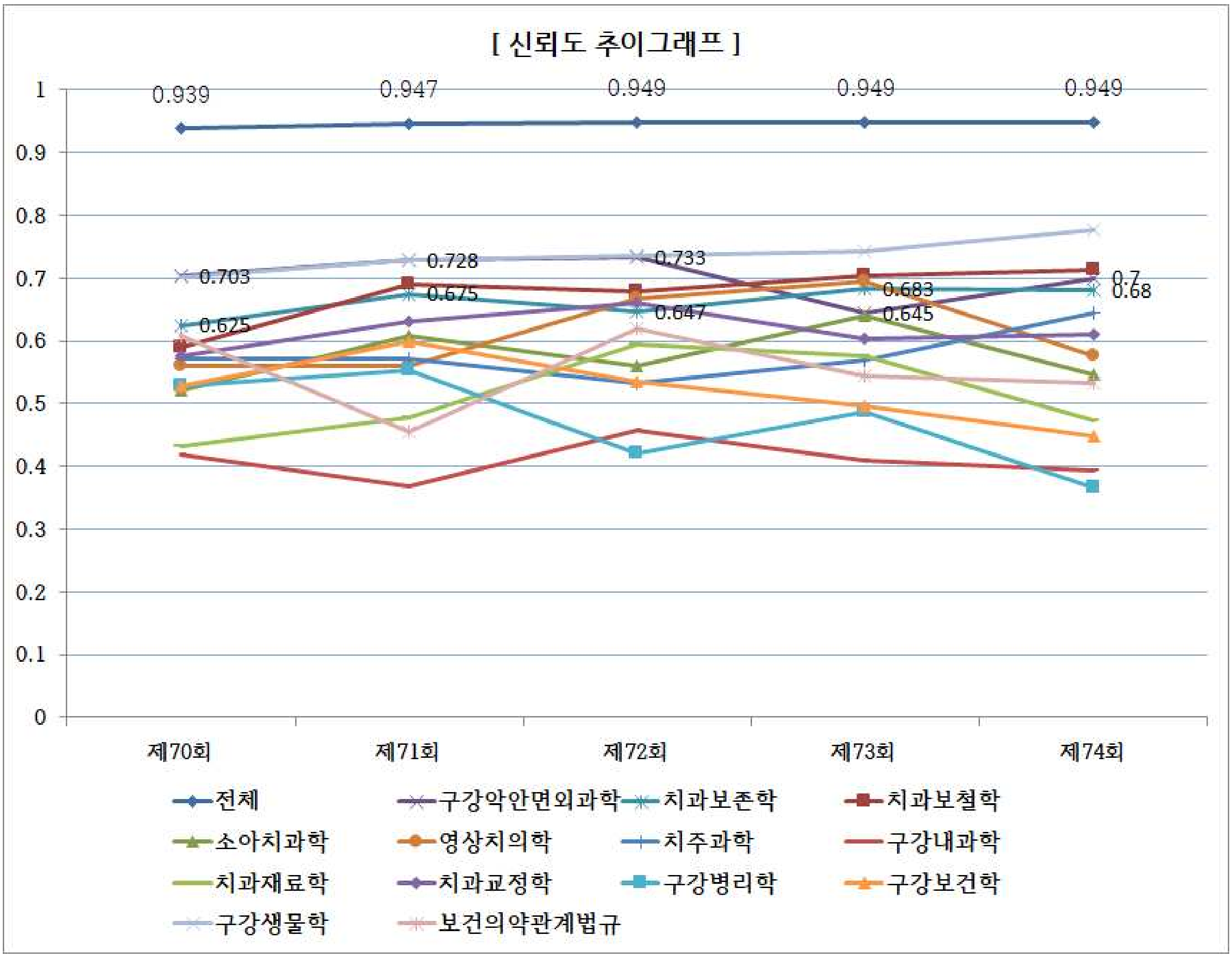

해석

- 전회 대비 신뢰도는 전체 문항에서는 동일했으며, 치과보존학, 소아치과학, 영상치의학, 구강내과학, 치과재료학, 구강병리학, 구강보건학, 보건의약관계 법규 과목에서 각각 .003, .093, .119, .015, .103, .122, .048, .011 감소함
- 구강악안면외과학, 치과보존학, 치과보철학, 치주과학, 치과교정학, 구강생물학 과목에서 신뢰도는 각각 .055, .011, .075, .007, .033 증가함

○ 분석결과 관련 문의 : 한국보건의료인국가시험원 연구개발본부 김준기 전임연구원
Tel : 02-2087-8957, FAX : 02-2087-8956
E-mail : tontates@kuksiwon.or.kr
