# Supplementary material for: Item difficulty index, discrimination index, and reliability of the 26 health professions licensing examinations in 2022, Korea: a psychometric study
Source: J Educ Eval Health Prof. 2023 Nov 22;20:31. doi: 10.3352/jeehp.2023.20.31 (PMC11959405; doi:10.3352/jeehp.2023.20.31)
Supplement: Supplementary file 1 — Supplement 1. Item analysis results of 26 health professions licensing examinations administered during late 2022 and early 2023. [file jeehp-20-31_Suppl1.zip › 2022│Γ╡╡ ┴a77╚╕ ╟╤└╟╗τ ▒╣░í╜├╟Φ ║╨╝«░ß░·.pdf]

2022년도 제77회 한의사 국가시험 문항분석 결과

국민이 신뢰하고 감동하는 시험평가기관
한국보건의료인국가시험원
KOREA HEALTH PERSONNEL LICENSING EXAMINATION INSTITUTE

일반 용어 정의

□ **평균**

- 집단에서의 대표적 경향값으로 전체 값을 더하여 총 응시자로 나눈 값

□ **표준편차**

- 평균과 각 점수의 차이인 편차들의 평균으로 점수가 흩어져 분포되어 있는 정도

□ **추정난이도**

- 문항개발자가 예측한 정답률

□ **검사이론**

- 검사와 검사를 구성하고 있는 문항의 양호도를 분석 및 평가하는 방법을 정의한 이론체계
- 대표적으로 고전검사이론과 문항반응이론이 있음

고전검사이론 용어 정의

□ **고전검사이론(Classical Test Theory; CTT)**

- 검사의 질을 분석하는 검사이론 중 한 가지로 19세기 말부터 전개되어 현재까지 주로 사용되고 있는 검사이론임
- 고전검사이론에 의한 문항과 응시자 능력 추정치는 다음과 같음

○ **문항난이도**

· 검사 문항의 쉽고 어려운 정도를 나타내는 지수
· 난이도 지수는 총 반응 수에 대한 정답 반응 수의 비율로 문항의 정답률임
· 문항난이도는 0~100까지의 값을 가짐
· 난이도 값이 큰 경우, 쉬운 문항으로 '난이도가 낮다'라고 해석하며, 난이도 값이 작은 경우, 어려운 문항으로 '난이도가 높다'라고 해석함

○ **문항변별도**

· 각 문항이 응시자의 능력 수준을 변별할 수 있는 정도를 나타내는 지수
· 문항변별도는 -1~+1까지의 값을 가지며, 1에 가까울수록 변별력 크다고 해석함
· 일반적으로 문항변별도가 0.3 이상이면 우수한 문항으로 평가함
· 구하는 방식에는 '상하위집단 구분법', '문항-총점 상관계수' 등이 있음
  1) 변별도 1(상하위구분법): 상위 27%와 하위 27% 집단의 난이도 차이를 구하는 방식
  2) 변별도 2(상관계수법): 문항-총점과의 상관계수로 구하는 방식

○ **신뢰도**

· 시험이 평가하고자 하는 것을 일관성 있게 측정하는가로 시험이 오차없이 정확하게 측정한 정도를 의미함
· 국시원에서는 문항의 내적일관성(Cronbach α)으로 신뢰도를 추정하며 1에 가까울수록 신뢰도가 높다고 해석함

목 차

I. 시행 결과 ······ 6
1. 시험 현황 ······ 7
1) 시험명 ······ 7
2) 시험시행일 ······ 7
3) 응시현황 ······ 7
4) 과목별 문항 수, 배점 및 과락 점수 ······ 7
2. 합격률과 평균성적 ······ 7
1) 합격 및 불합격 현황 ······ 7
2) 과목별 과락자수 내역 ······ 7
3) 전회 대비 합격률과 평균성적 ······ 8

II. 문항분석 결과 ······ 10
1. 성적 ······ 11
1) 전체 성적분포도 ······ 12
2) 과목별 성적분포도 ······ 11
2. 난이도와 변별도 ······ 15
1) 전체 난이도와 변별도 ······ 15
2) 과목별 난이도와 변별도 ······ 18
3) 지식수준별 난이도와 변별도 ······ 51
4) 자료유형별 난이도와 변별도 ······ 59
3. 난이도와 변별도 간 산포도 ······ 65
1) 전체 난이도와 변별도 간 산포도 ······ 65
2) 과목별 난이도와 변별도 간 산포도 ······ 65
4. 신뢰도 분석 ······ 72

I. 시행 결과

1. 시험 현황

1) 시험명: 2022년도 제77회 한의사 국가시험

2) 시험시행일: 2022년 1월 14일

3) 응시현황

응시대상자수	결시자수	부정행위자수	응시자 준수사항 위반자 수		응시자수 (%)
			휴대폰 소지	신분증 미지참	
757	4	0	0	0	753 (99.5)

4) 과목별 문항 수, 배점 및 과락 점수

교 시	과 목 명	문제 수	배점	총점	합격자 점수기준	
					과목 과락기준	총점 합격기준
1교시	내과학	112	1	112	44.8점 미만	204점 이상
2교시	침구학	48	1	48	19.2점 미만	
	보건의약관계 법규	20	1	20	8점 미만	
3교시	외과학	16	1	16	19.2점 미만	
	신경정신과학	16	1	16		
	안이비인후과학	16	1	16		
	부인과학	32	1	32	22.4점 미만	
4교시	소아과학	24	1	24		
	예방의학	24	1	24	22.4점 미만	
	한방생리학	16	1	16		
	본초학	16	1	16		
계		340		340		

2. 합격률과 평균성적

1) 합격 및 불합격 현황

합격자수 (%)	불합격자수(%)				채점보류자수
	평락	과락	기권	계	
731 (97.1)	20 (2.7)	1 (0.1)	1 (0.1)	22 (2.9)	0 (0.0)

2) 과목별 과락자수 내역

과목명 / 과락자수	내과학	침구학	보건의약관계법규	외과학	신경정신과학	안이비인후과학	부인과학	소아과학	예방의학	한방생리학	본초학
과목별 과락자 수	0	0	1	0	0	0	0	0	0	0	0
전과목 과락자 수	1										

3) 전회 대비 합격률과 평균성적

회차	년도	합격률(%)	평균성적	표준편차	백분율 환산점수
제73회	2018	95.7	290.7	31.7	76.5
제74회	2019	96.6	260.9	27.5	76.7
제75회	2020	96.6	254.7	25.4	74.9
제76회	2021	96.4	257.0	24.7	75.6
제77회	2022	97.1	260.8	26.0	76.7

※제73회 380점만점, 제74회~제77회 340점만점

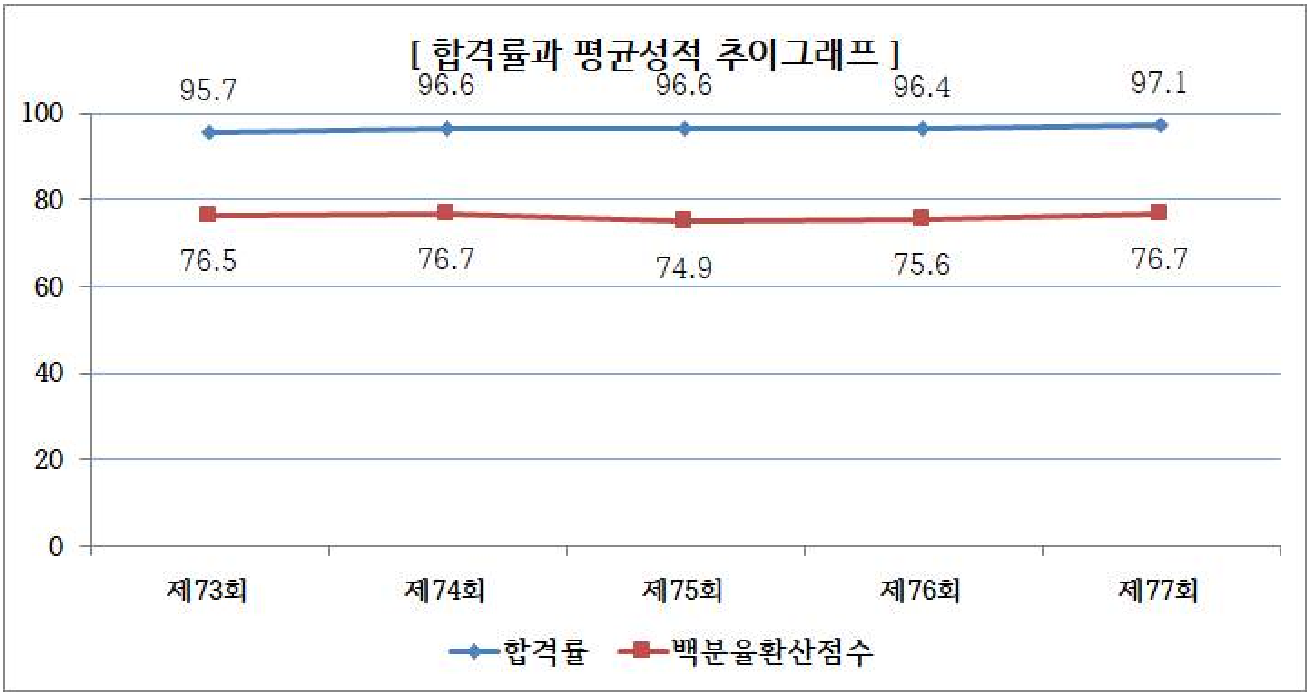

해석

- 전년 대비 합격률은 0.7 증가하였고, 백분율 환산점수는 1.1 점 상승함

Ⅱ. 문항분석 결과

1. 성적

1) 전체 성적분포도

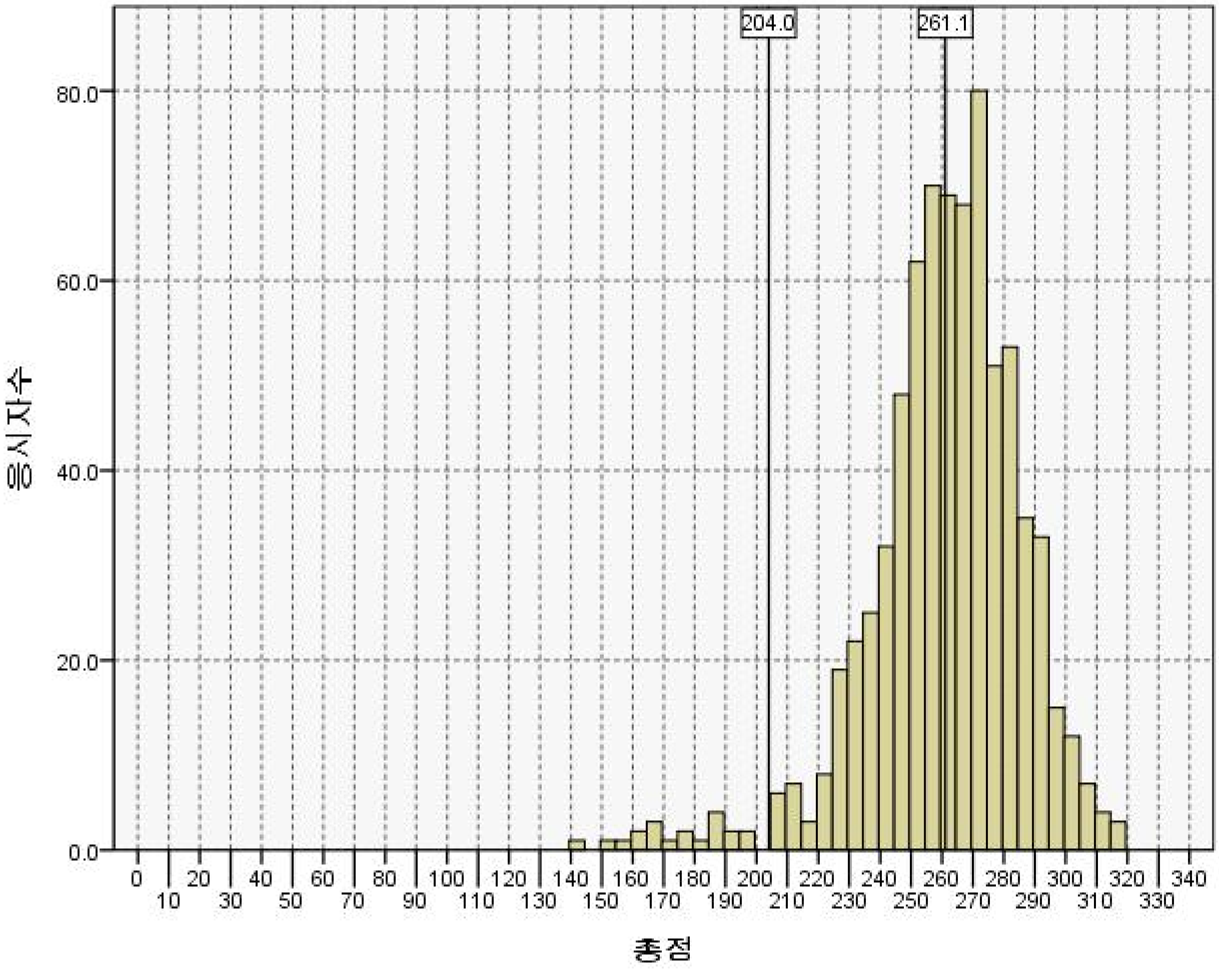

응시자	총점	합격선	평균성적	표준편차
752*	340	204	261.1	24.8

*기권자 1명 제외

2) 과목별 성적분포도

가) 내과학

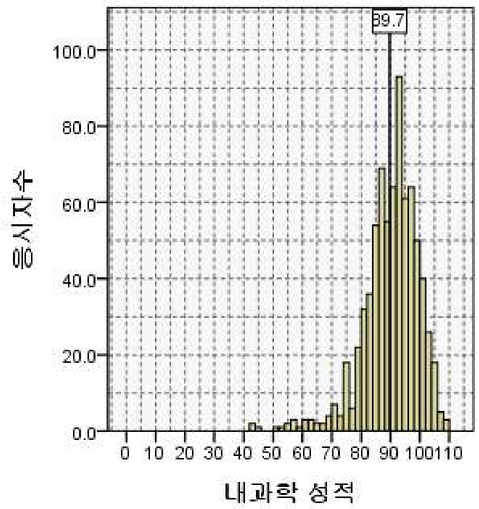

총점	과락선	평균성적	표준편차
112	44.8	89.7	9.5

나) 침구학

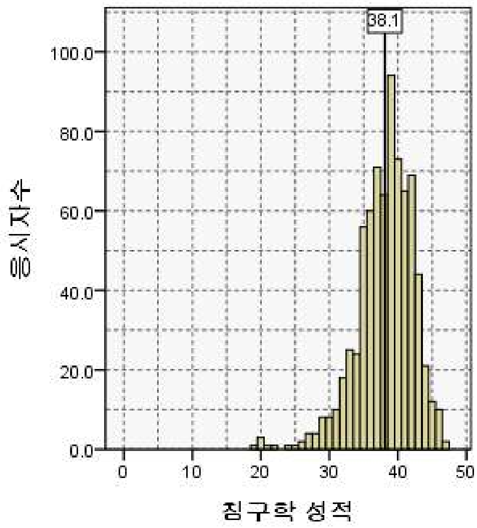

총점	과락선	평균성적	표준편차
48	19.2	38.1	4.1

다) 보건의약관계법규

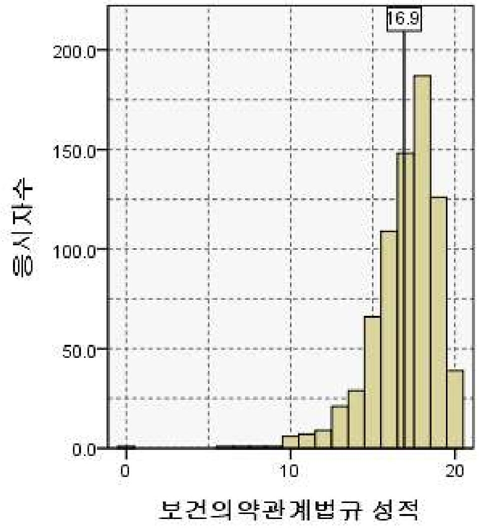

총점	과락선	평균성적	표준편차
20	8	16.9	2.1

라) 외과학

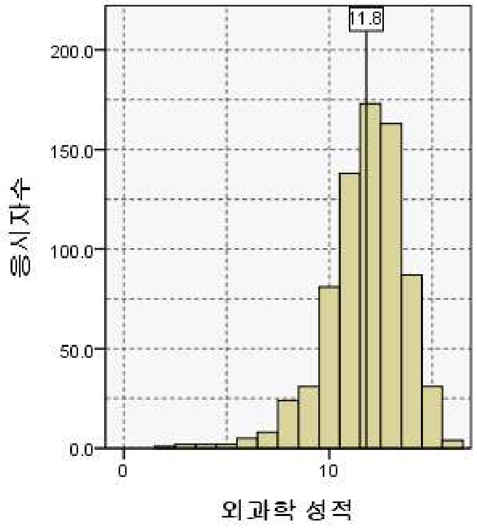

총점	과락선	평균성적	표준편차
16		11.8	1.9

마) 신경정신과학

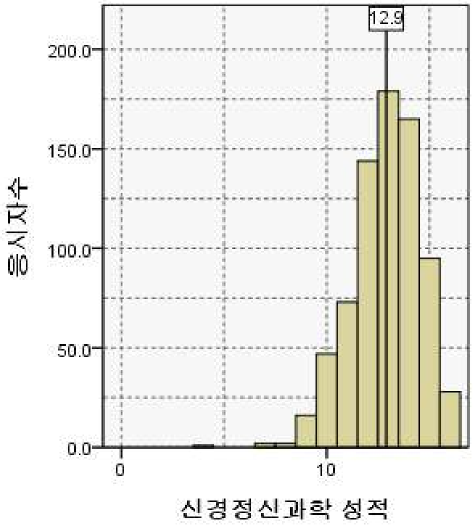

총점	과락선	평균성적	표준편차
16		12.9	1.7

바) 안이비인후과학

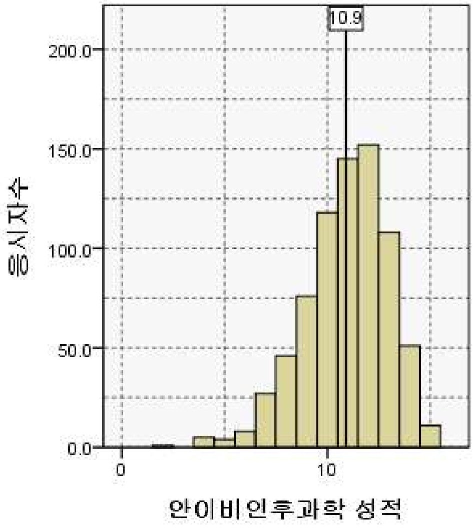

총점	과락선	평균성적	표준편차
16		10.9	2.0

사) 부인과학

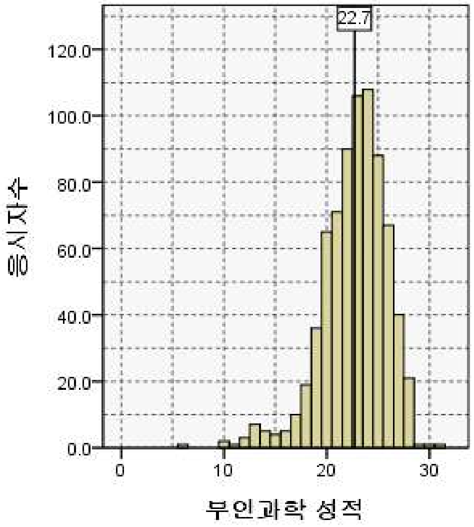

총점	과락선	평균성적	표준편차
32		22.7	3.1

아) 소아과학

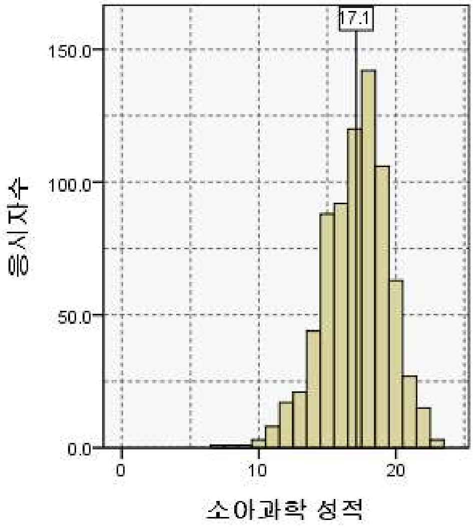

총점	과락선	평균성적	표준편차
24		17.1	2.4

자) 예방의학

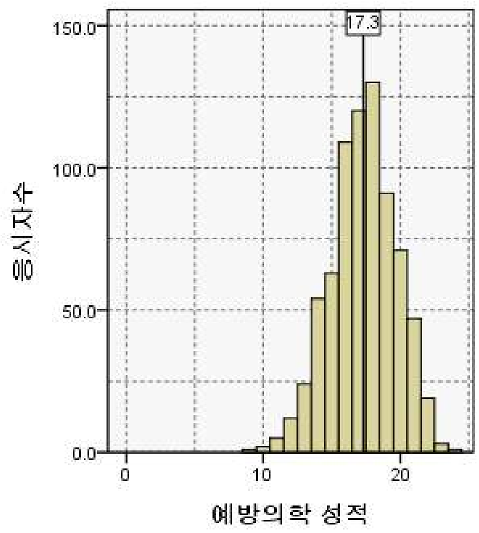

총점	과락선	평균성적	표준편차
24		17.3	2.4

차) 한방생리학

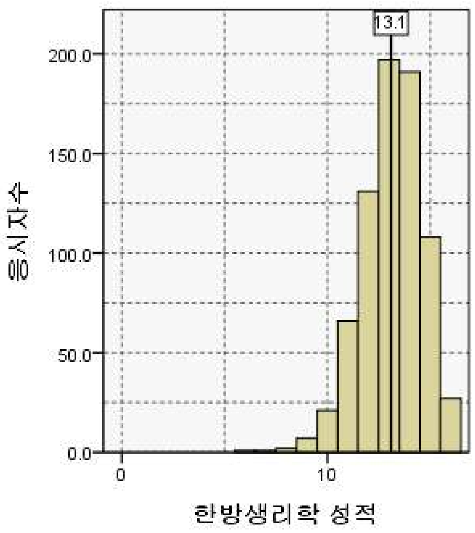

총점	과락선	평균성적	표준편차
16		13.1	1.5

카) 본초학

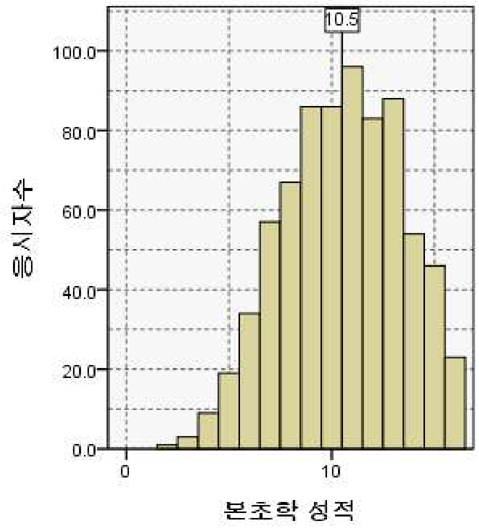

총점	과락선	평균성적	표준편차
16		10.5	2.9

※ 과락선은 매 과목 40% 미만. (세부적인 내용은 1.시험현황 참조)

2. 난이도와 변별도

1) 전체 난이도와 변별도

가) 전회 대비 전체 난이도와 변별도

회차	난이도		변별도1		변별도2	
	평균	표준편차	평균	표준편차	평균	표준편차
제73회	76.5	20.6	.19	.12	.23	.10
제74회	76.9	20.0	.17	.11	.21	.10
제75회	74.9	21.4	.17	.11	.20	.10
제76회	75.6	22.2	.17	.12	.20	.10
제77회	76.8	22.0	.17	.11	.20	.10

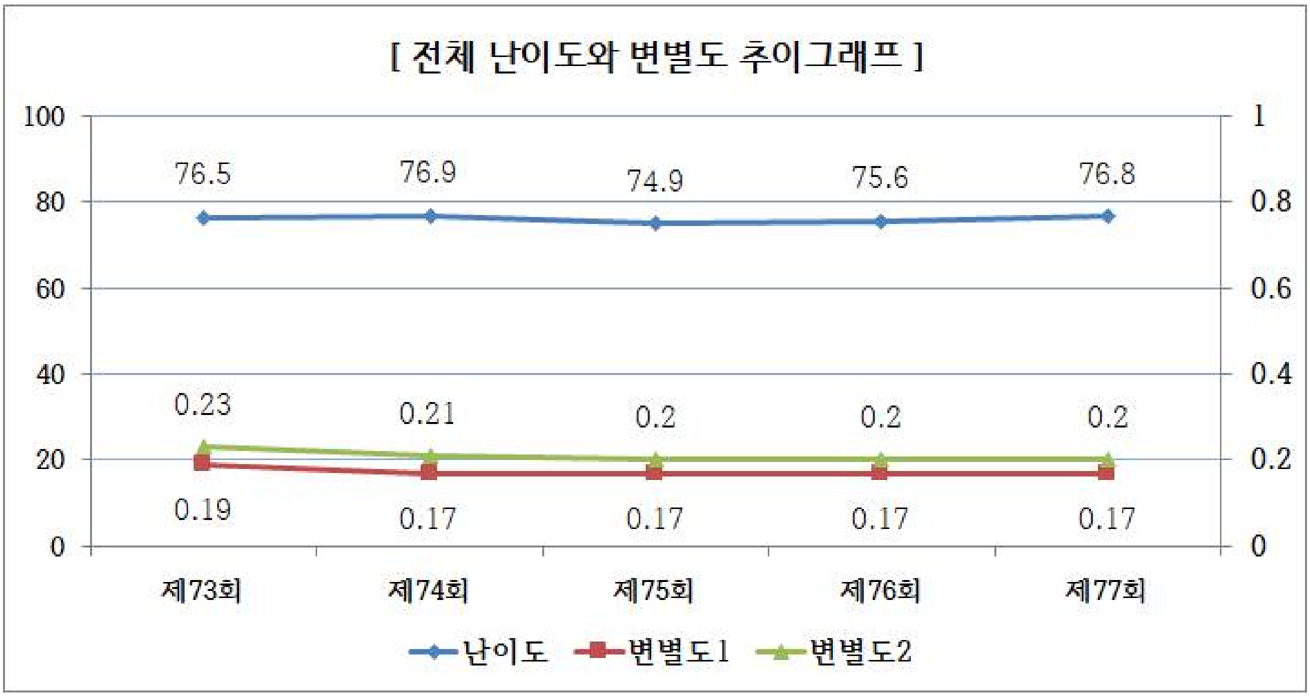

해석

- 전년 대비 난이도 지수는 1.2 상승함
- 변별도 1 지수는 변하지 않음
- 변별도 2 지수는 변하지 않음

나) 전체 난이도와 변별도 분포도 및 비율분석

(1) 전체 난이도 분포도 및 비율분석

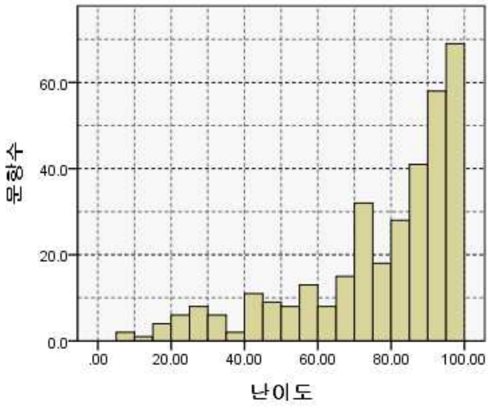

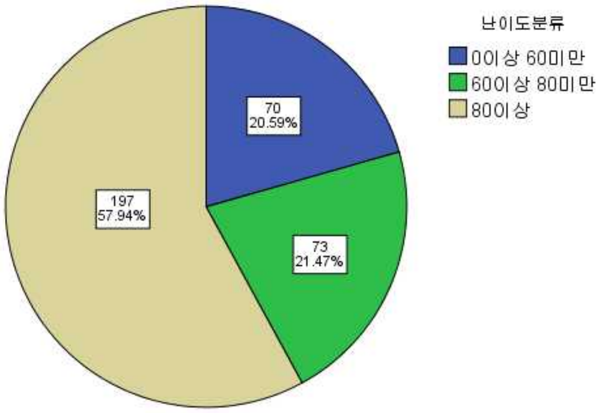

총점	난이도	표준편차
340	76.8	22.0

난이도	문항수	비율(%)
0~60미만	70	20.6
60~80미만	73	21.5
80~100	197	57.9
전체	340	100.0

(2) 전체 변별도1 분포도 및 비율분석

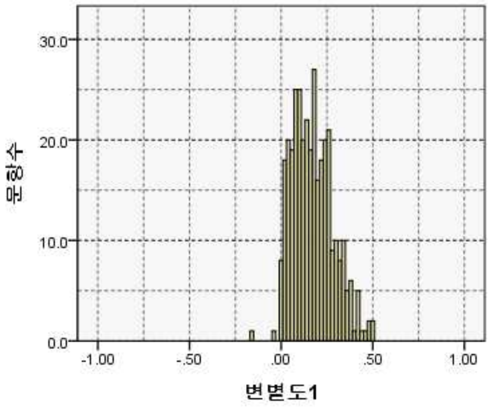

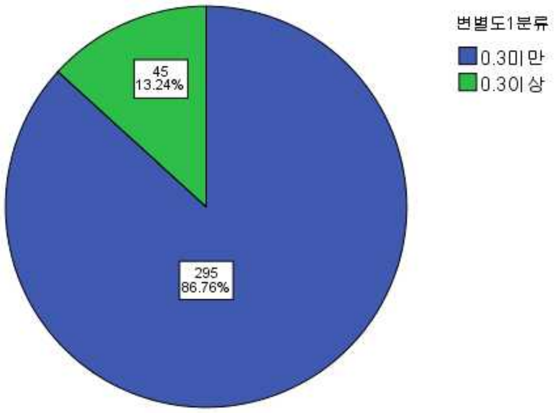

총점	변별도1	표준편차
340	.17	.11

변별도1	문항수	비율(%)
0.3미만	295	86.8
0.3이상	45	13.2
전체	340	100.0

(3) 전체 변별도2 분포도 및 비율분석

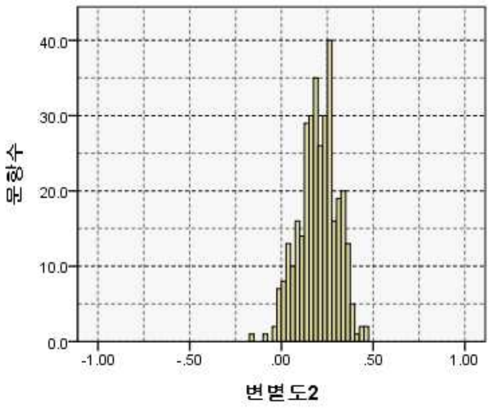

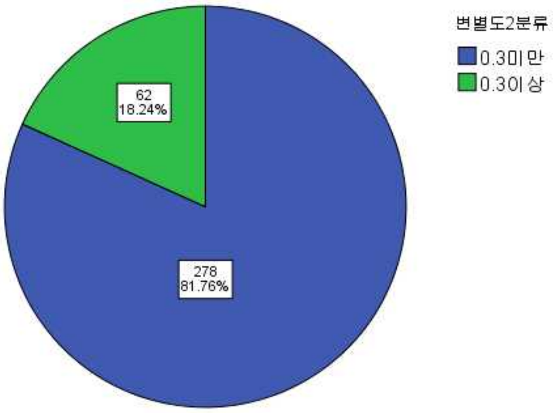

총점	변별도2	표준편차	변별도2	문항수	비율(%)
340	.20	.10	0.3미만	278	81.8
			0.3이상	62	18.2
			전체	340	100.0

해석

- 난이도 지수가 80에서 100사이인 문항이 전체 340문항 중 197문항으로 가장 많았으며, 차례로 60이상 80미만인 문항이 73문항, 60미만인 문항이 70문항인 것으로 나타남
- 변별도1 지수를 기준으로 분류하였을 때, 0.3 미만인 문항이 295문항으로 0.3 이상인 문항이 45문항인 것에 비해 더 많이 나타남
- 변별도2 지수를 기준으로 분류하였을 때, 0.3 미만인 문항이 278문항으로 0.3 이상인 문항이 62문항인 것에 비해 더 많이 나타남

2) 과목별 난이도와 변별도

가) 전회 대비 과목별 난이도와 변별도

(1) 전회 대비 내과학 난이도와 변별도

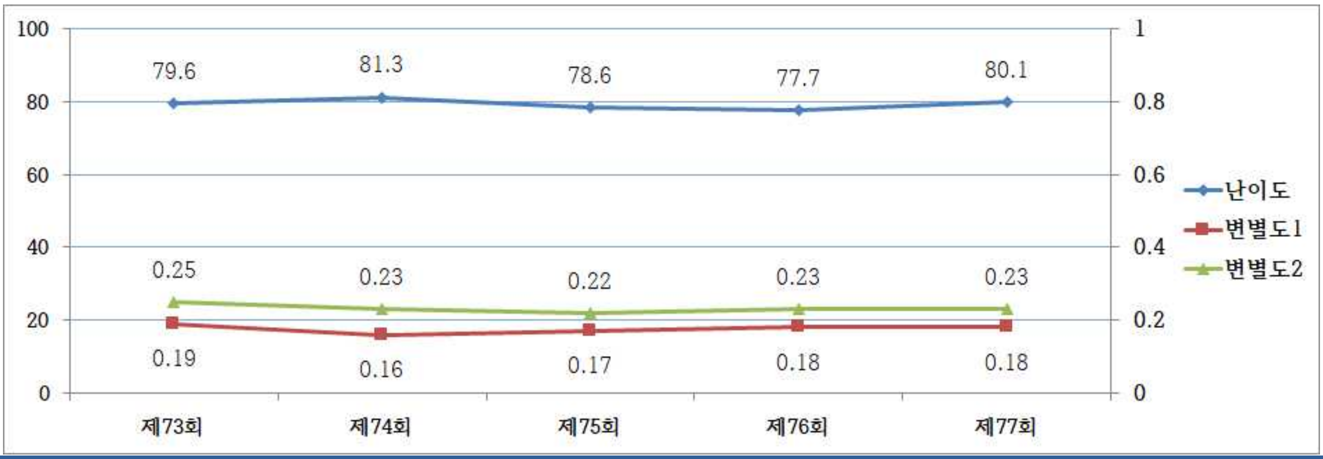

회차	난이도		변별도1		변별도2	
	평균	표준편차	평균	표준편차	평균	표준편차
제73회	79.6	18.9	.19	.12	.25	.09
제74회	81.3	19.4	.16	.11	.23	.10
제75회	78.6	19.2	.17	.11	.22	.10
제76회	77.7	22.0	.18	.12	.23	.10
제77회	80.1	19.9	.18	.11	.23	.10

해석

- 전회 대비 내과학 과목의 난이도 지수는 2.4 증가함
- 전회 대비 내과학 과목의 변별도 1 지수는 변하지 않음
- 전회 대비 내과학 과목의 변별도 2 지수는 변하지 않음

(2) 전회 대비 침구학 난이도와 변별도

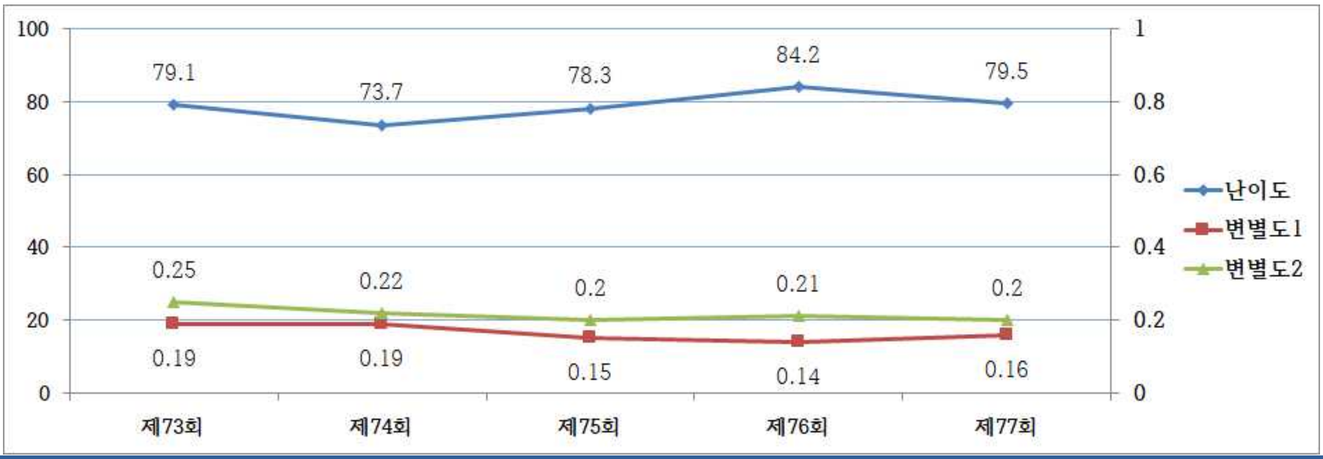

회차	난이도		변별도1		변별도2	
	평균	표준편차	평균	표준편차	평균	표준편차
제73회	79.1	19.0	.19	.10	.25	.09
제74회	73.7	20.9	.19	.11	.22	.11
제75회	78.3	20.0	.15	.11	.20	.10
제76회	84.2	17.1	.14	.11	.21	.09
제77회	79.5	21.6	.16	.11	.20	.09

해석

- 전회 대비 침구학 과목의 난이도 지수는 4.7 감소함
- 전회 대비 침구학 과목의 변별도 1 지수는 .02 증가함
- 전회 대비 침구학 과목의 변별도 2 지수는 .01 감소함

(3) 전회 대비 보건의약관계법규 난이도와 변별도

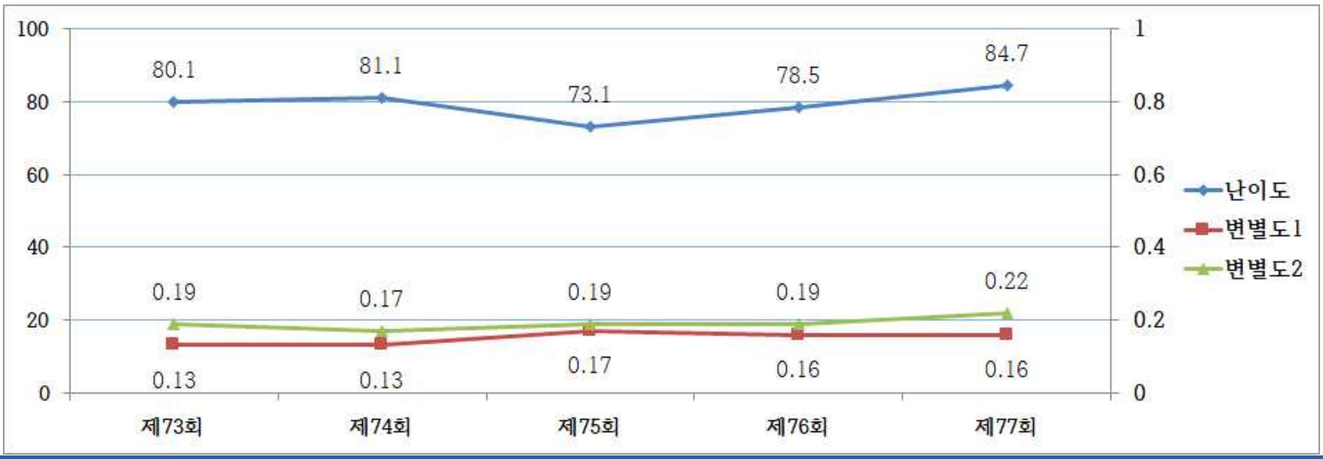

회차	난이도		변별도1		변별도2	
	평균	표준편차	평균	표준편차	평균	표준편차
제73회	80.1	24.1	.13	.10	.19	.10
제74회	81.1	17.9	.13	.09	.17	.09
제75회	73.1	22.9	.17	.09	.19	.08
제76회	78.5	19.4	.16	.12	.19	.08
제77회	84.7	15.9	.16	.09	.22	.08

해석

- 전회 대비 보건의약관계법규 과목의 난이도 지수는 6.2 증가함
- 전회 대비 보건의약관계법규 과목의 변별도 1 지수는 변하지 않음
- 전회 대비 보건의약관계법규 과목의 변별도 2 지수는 .03 증가함

(4) 전회 대비 외과학 난이도와 변별도

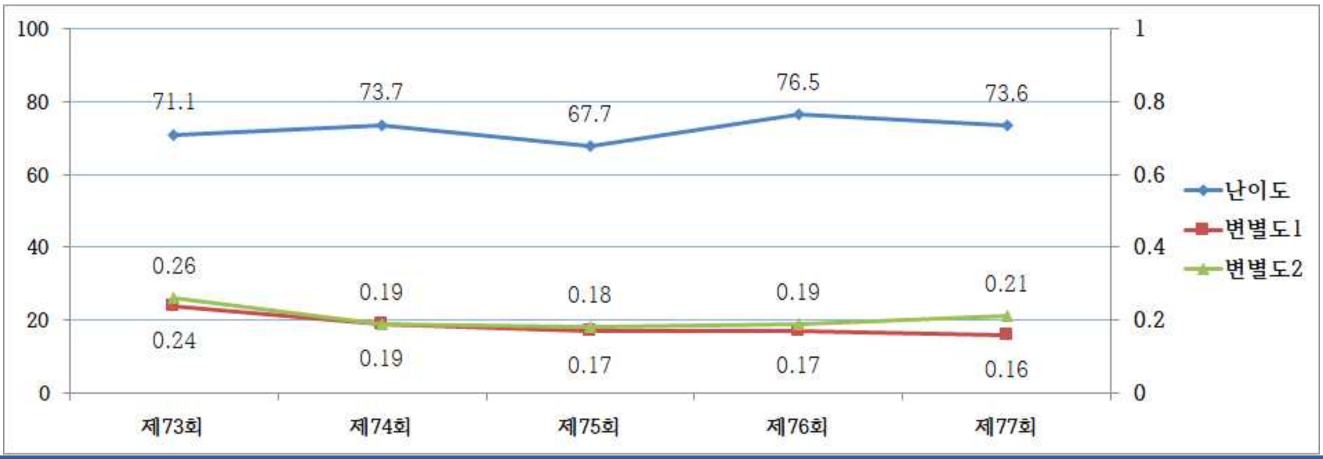

회차	난이도		변별도1		변별도2	
	평균	표준편차	평균	표준편차	평균	표준편차
제73회	71.1	21.6	.24	.17	.26	.13
제74회	73.7	16.5	.19	.12	.19	.10
제75회	67.7	25.9	.17	.13	.18	.13
제76회	76.5	18.2	.17	.09	.19	.08
제77회	73.6	26.5	.16	.09	.21	.11

해석

- 전회 대비 외과학 과목의 난이도 지수는 2.9 감소함
- 전회 대비 외과학 과목의 변별도 1 지수는 .01 감소함
- 전회 대비 외과학 과목의 변별도 2 지수는 .02 증가함

(5) 전회 대비 신경정신과학 난이도와 변별도

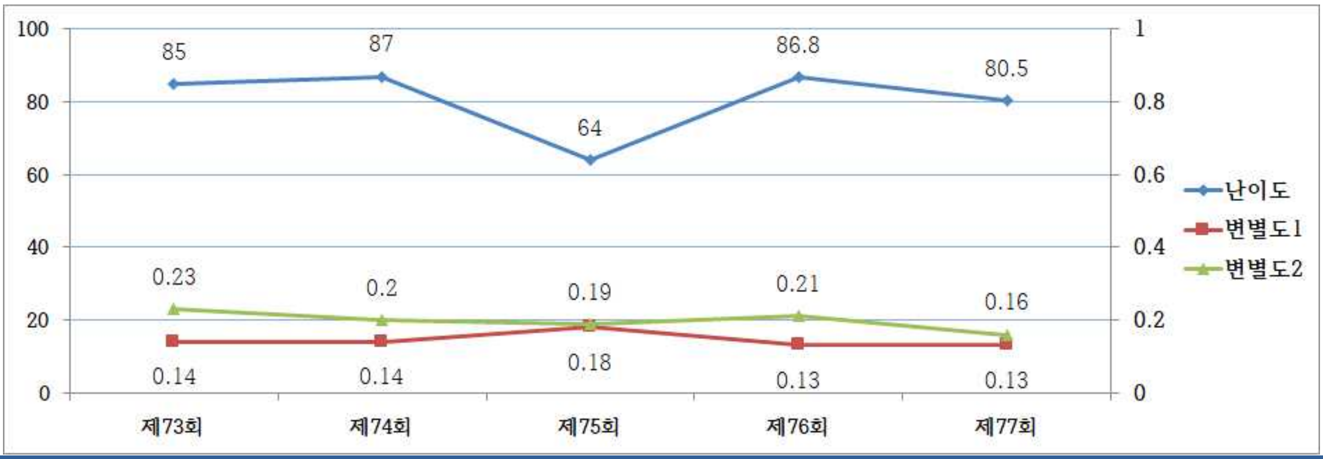

회차	난이도		변별도1		변별도2	
	평균	표준편차	평균	표준편차	평균	표준편차
제73회	85.0	14.7	.14	.11	.23	.11
제74회	87.0	9.4	.14	.10	.20	.11
제75회	64.0	25.8	.18	.14	.19	.14
제76회	86.8	12.1	.13	.07	.21	.09
제77회	80.5	18.3	.13	.11	.16	.11

해석

- 전회 대비 신경정신과학 과목의 난이도 지수는 6.3 감소함
- 전회 대비 신경정신과학 과목의 변별도 1 지수는 변하지 않음
- 전회 대비 신경정신과학 과목의 변별도 2 지수는 .05 감소함

(6) 전회 대비 안이비인후과학 난이도와 변별도

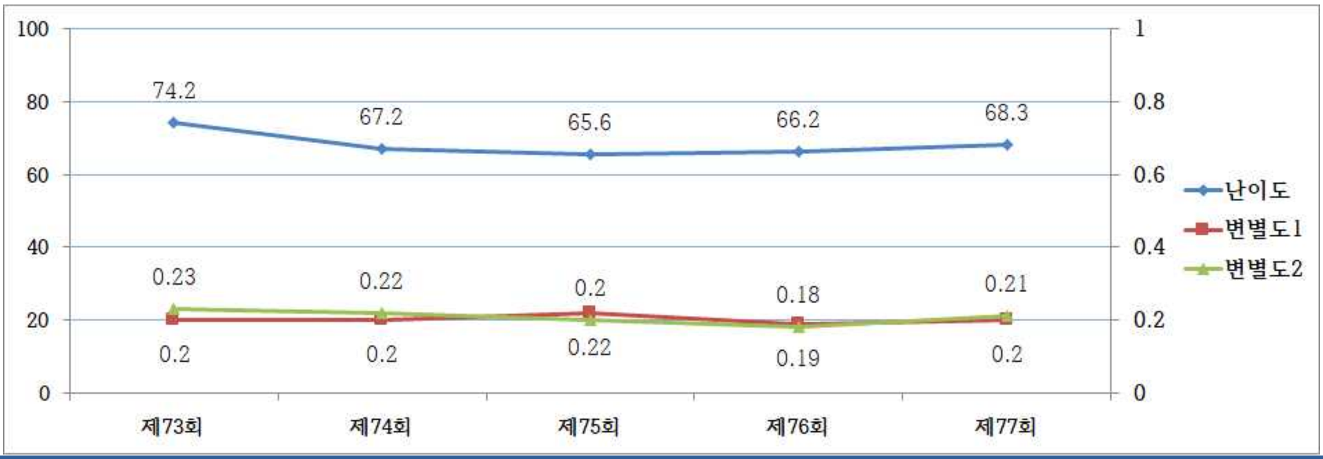

회차	난이도		변별도1		변별도2	
	평균	표준편차	평균	표준편차	평균	표준편차
제73회	74.2	22.5	.20	.13	.23	.10
제74회	67.2	24.2	.20	.11	.22	.09
제75회	65.6	21.0	.22	.12	.20	.10
제76회	66.2	22.0	.19	.17	.18	.14
제77회	68.3	24.8	.20	.11	.21	.11

해석

- 전회 대비 안이비인후과학 과목의 난이도 지수는 2.1 증가함
- 전회 대비 안이비인후과학 과목의 변별도 1 지수는 .01 증가함
- 전회 대비 안이비인후과학 과목의 변별도 2 지수는 .03 증가함

(7) 전회 대비 부인과학 난이도와 변별도

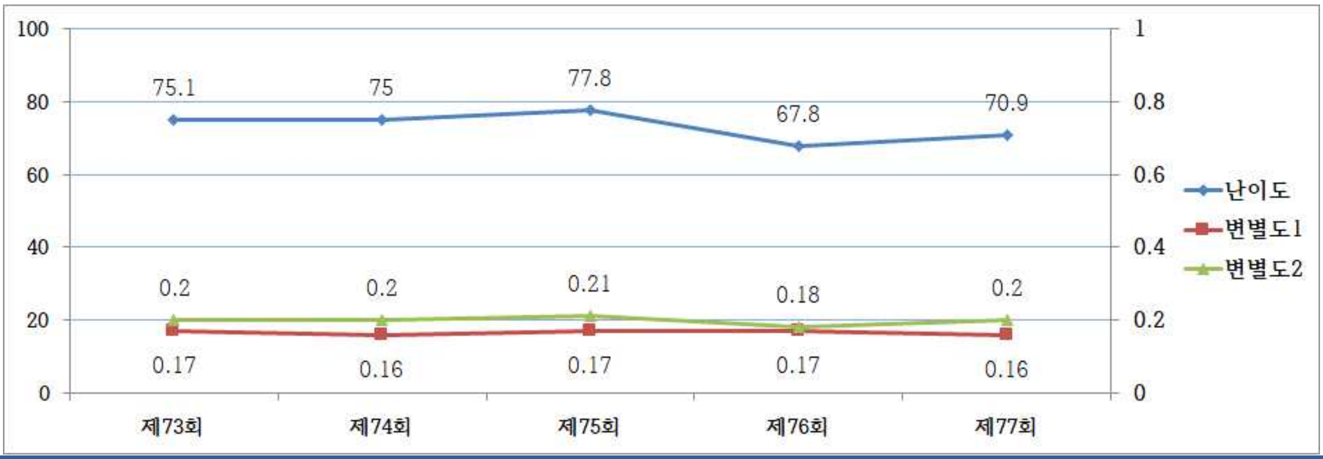

회차	난이도		변별도1		변별도2	
	평균	표준편차	평균	표준편차	평균	표준편차
제73회	75.1	20.1	.17	.14	.20	.12
제74회	75.0	17.8	.16	.12	.20	.12
제75회	77.8	20.7	.17	.10	.21	.11
제76회	67.8	24.7	.17	.14	.18	.12
제77회	70.9	26.9	.16	.13	.20	.14

해석

- 전회 대비 부인과학 과목의 난이도 지수는 3.1 증가함
- 전회 대비 부인과학 과목의 변별도 1 지수는 .01 감소함
- 전회 대비 부인과학 과목의 변별도 2 지수는 .02 증가함

(8) 전회 대비 소아과학 난이도와 변별도

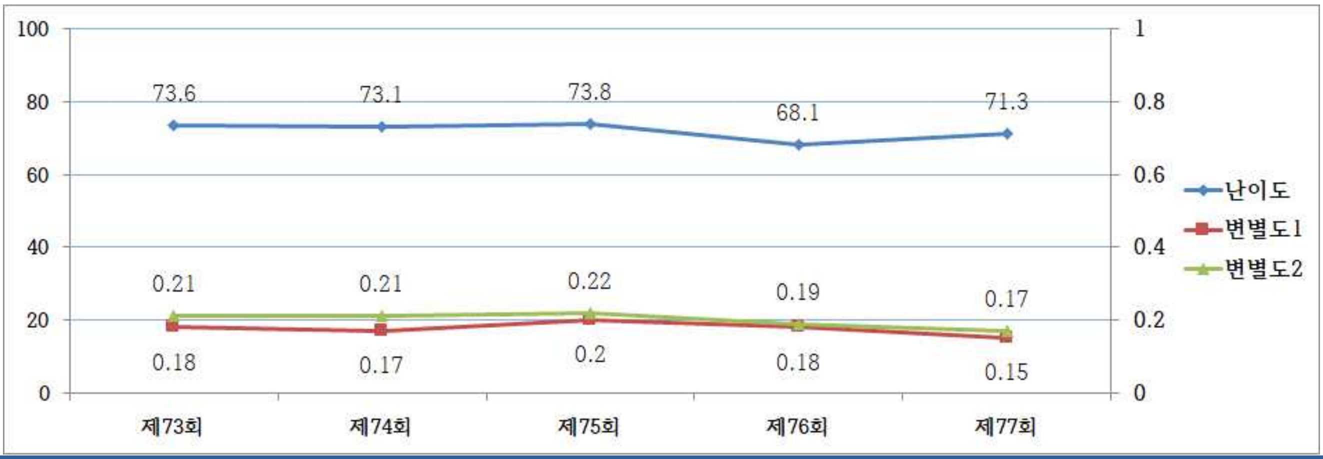

회차	난이도		변별도1		변별도2	
	평균	표준편차	평균	표준편차	평균	표준편차
제73회	73.6	20.4	.18	.16	.21	.13
제74회	73.1	25.3	.17	.12	.21	.08
제75회	73.8	20.7	.20	.13	.22	.10
제76회	68.1	25.3	.18	.13	.19	.12
제77회	71.3	25.7	.15	.10	.17	.10

해석

- 전회 대비 소아과학 과목의 난이도 지수는 3.2 증가함
- 전회 대비 소아과학 과목의 변별도 1 지수는 .03 감소함
- 전회 대비 소아과학 과목의 변별도 2 지수는 .02 감소함

(9) 전회 대비 예방의학 난이도와 변별도

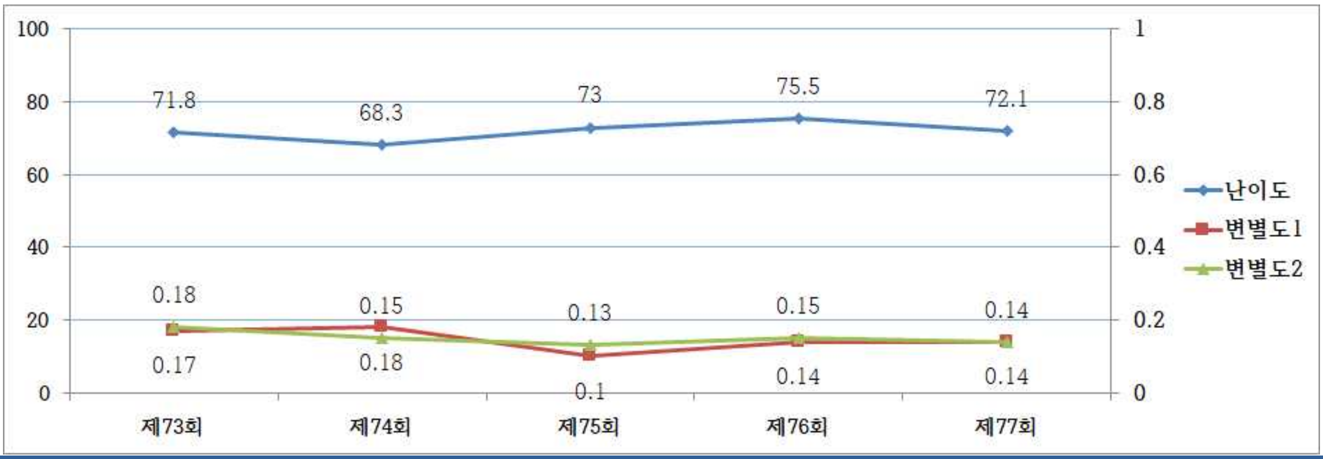

회차	난이도		변별도1		변별도2	
	평균	표준편차	평균	표준편차	평균	표준편차
제73회	71.8	21.7	.17	.10	.18	.09
제74회	68.3	21.2	.18	.11	.15	.10
제75회	73.0	25.3	.10	.07	.13	.08
제76회	75.5	26.1	.14	.13	.15	.11
제77회	72.1	23.2	.14	.09	.14	.08

해석

- 전회 대비 예방의학 과목의 난이도 지수는 3.4 감소함
- 전회 대비 예방의학 과목의 변별도 1 지수는 변하지 않음
- 전회 대비 예방의학 과목의 변별도 2 지수는 .01 감소함

(10) 전회 대비 한방생리학 난이도와 변별도

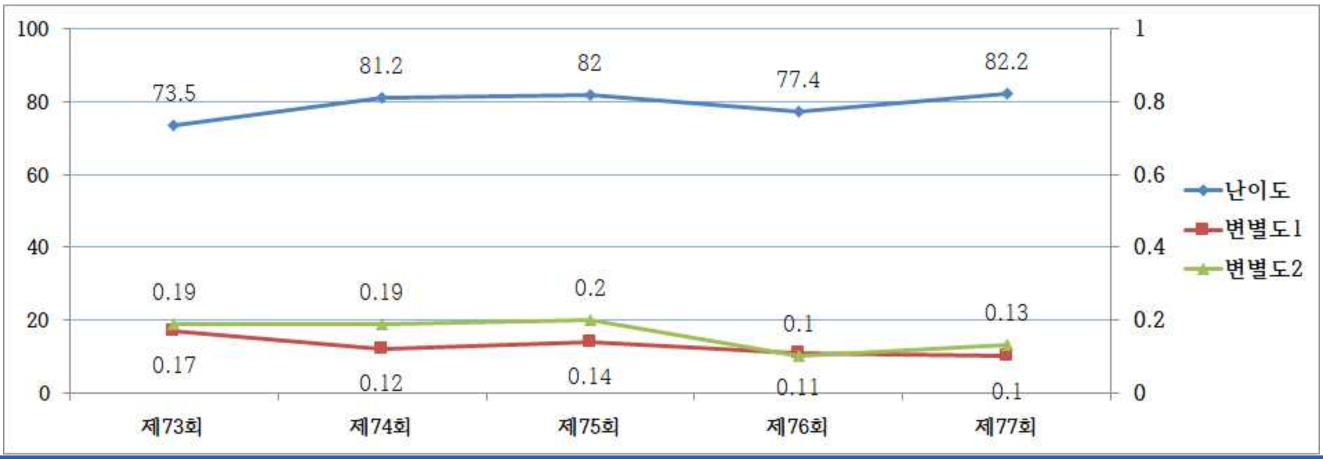

회차	난이도		변별도1		변별도2	
	평균	표준편차	평균	표준편차	평균	표준편차
제73회	73.5	24.4	.17	.16	.19	.12
제74회	81.2	19.2	.12	.10	.19	.08
제75회	82.0	18.6	.14	.08	.20	.06
제76회	77.4	19.5	.11	.09	.10	.10
제77회	82.2	18.6	.10	.07	.13	.10

해석

- 전회 대비 한방생리학 과목의 난이도 지수는 4.8 증가함
- 전회 대비 한방생리학 과목의 변별도 1 지수는 .01 감소함
- 전회 대비 한방생리학 과목의 변별도 2 지수는 .03 증가함

(11) 전회 대비 본초학 난이도와 변별도

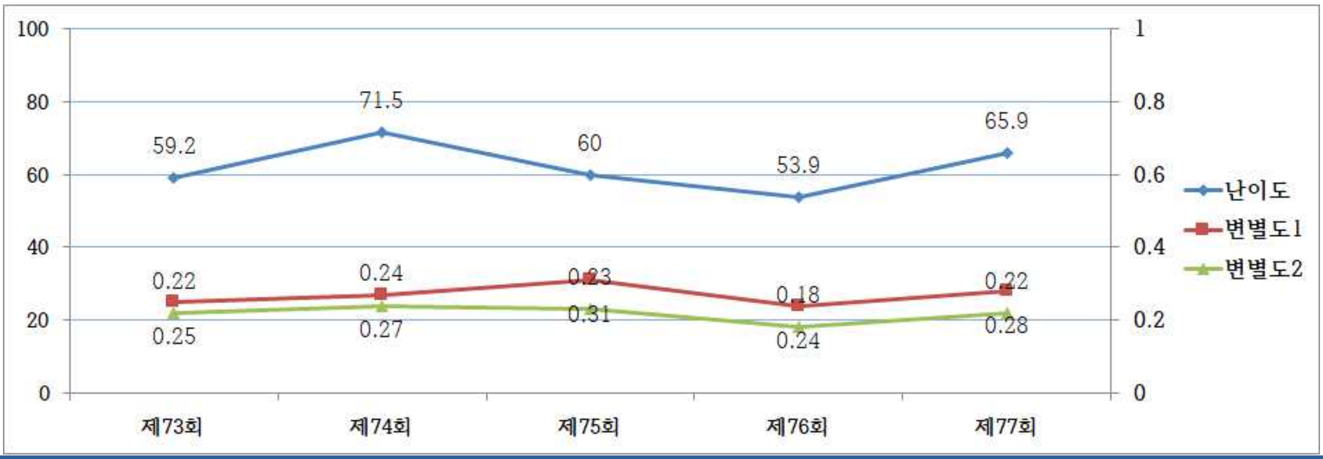

회차	난이도		변별도1		변별도2	
	평균	표준편차	평균	표준편차	평균	표준편차
제73회	59.2	22.9	.25	.09	.22	.08
제74회	71.5	15.5	.27	.11	.24	.06
제75회	60.0	16.5	.31	.09	.23	.07
제76회	53.9	18.1	.24	.10	.18	.08
제77회	65.9	16.2	.28	.12	.22	.08

해석

- 전회 대비 본초학 과목의 난이도 지수는 12.0 증가함
- 전회 대비 본초학 과목의 변별도 1 지수는 .04 증가함
- 전회 대비 본초학 과목의 변별도 2 지수는 .04 증가함

나) 과목별 난이도와 변별도 분포도 및 비율분석

(1) 내과학 난이도와 변별도 분석도 및 비율분석

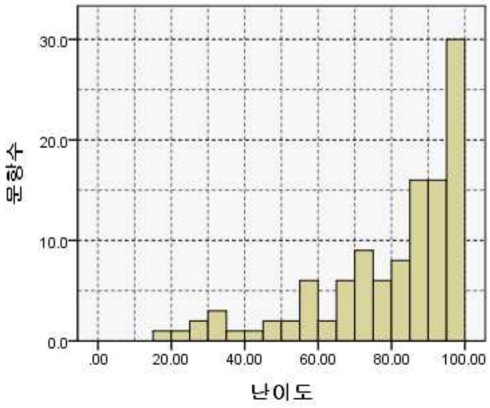

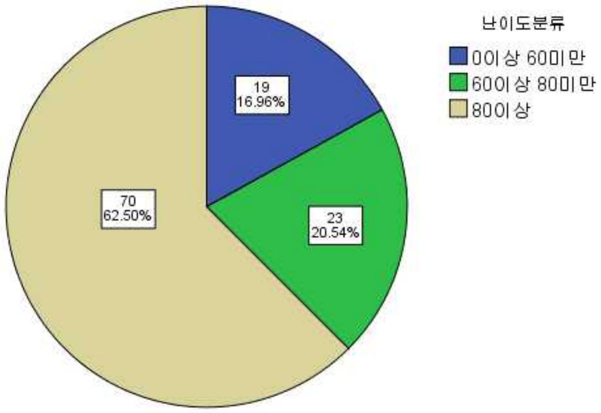

총점	난이도	표준편차	난이도	문항수	비율(%)
112	80.1	19.9	0~60미만	19	17.0
			60~80미만	23	20.5
			80~100	70	62.5
			전체	112	100.0

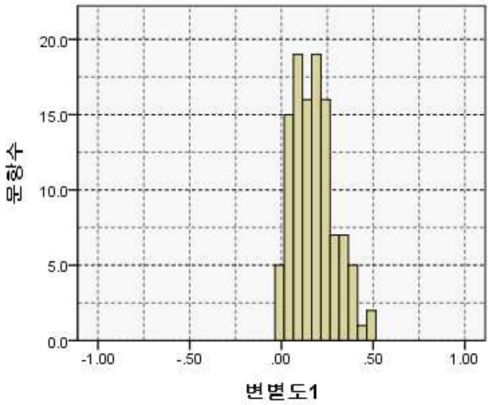

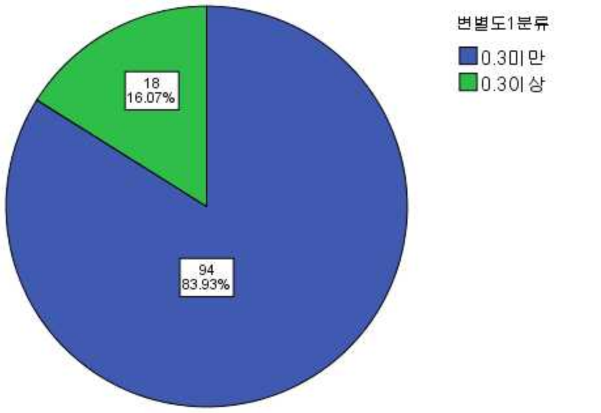

총점	변별도1	표준편차	변별도1	문항수	비율(%)
112	.18	.11	0.3미만	94	83.9
			0.3이상	18	16.1
			전체	112	100.0

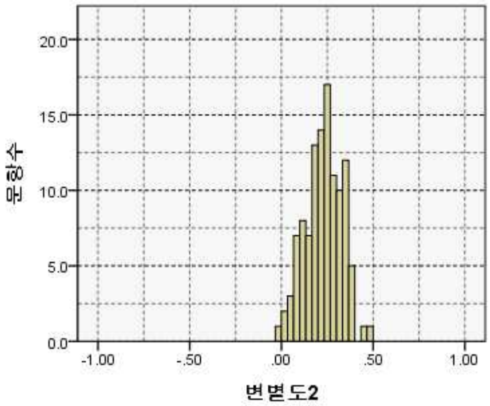

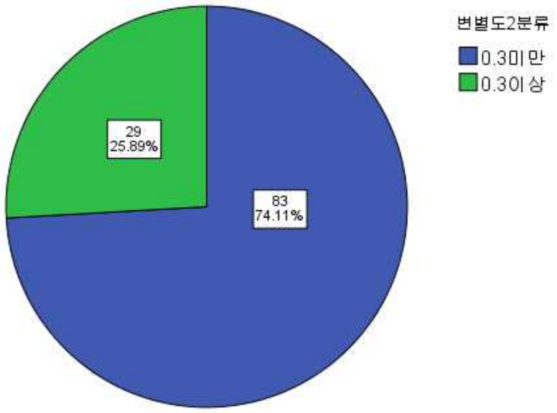

총점	변별도2	표준편차
112	.23	.10

변별도2	문항수	비율(%)
0.3미만	83	74.1
0.3이상	29	25.9
전체	112	100.0

해석

- 내과학 과목에서 난이도 지수가 80에서 100 사이인 문항이 전체 112문항 중 70문항으로 가장 많았으며, 다음으로 60이상 80미만인 문항이 23문항, 60 미만인 문항이 19문항으로 나타남
- 변별도1 지수를 기준으로 분류하였을 때, 0.3미만인 문항이 94문항으로 0.3 이상인 문항이 18문항인 것에 비해 더 많이 나타남
- 변별도2 지수를 기준으로 분류하였을 때, 0.3미만인 문항이 83문항으로 0.3 이상인 문항이 29문항인 것에 비해 더 많이 나타남

(2) 침구학 난이도와 변별도 분포도 및 비율분석

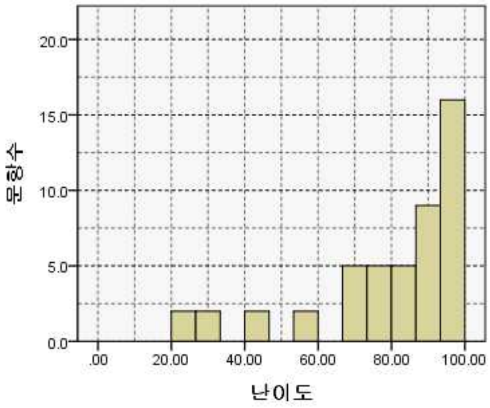

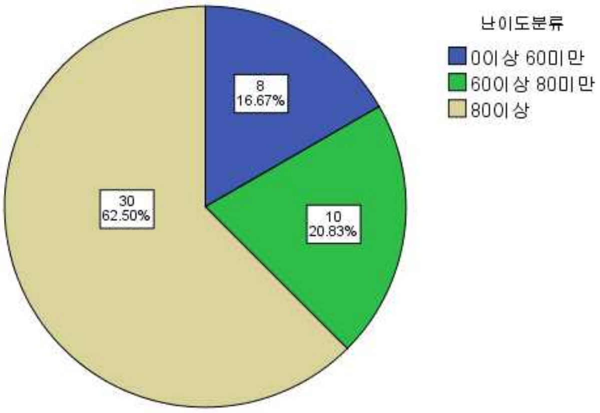

총점	난이도	표준편차
48	79.5	21.6

난이도	문항수	비율(%)
0~60미만	8	16.7
60~80미만	10	20.8
80~100	30	62.5
전체	48	100.0

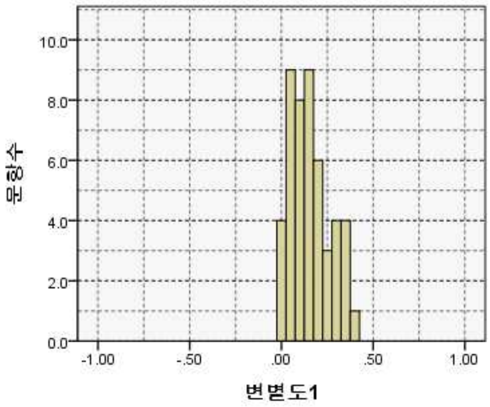

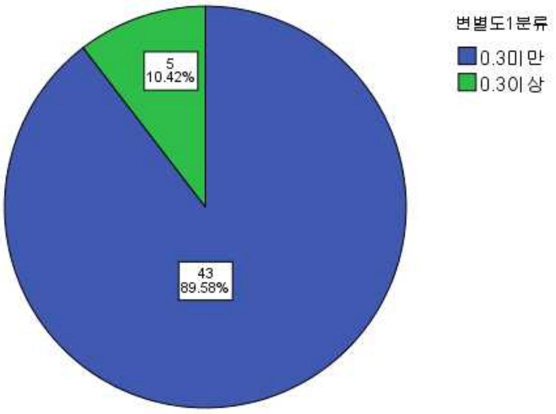

총점	변별도1	표준편차
48	.16	.11

변별도1	문항수	비율(%)
0.3미만	43	89.6
0.3이상	5	10.4
전체	48	100.0

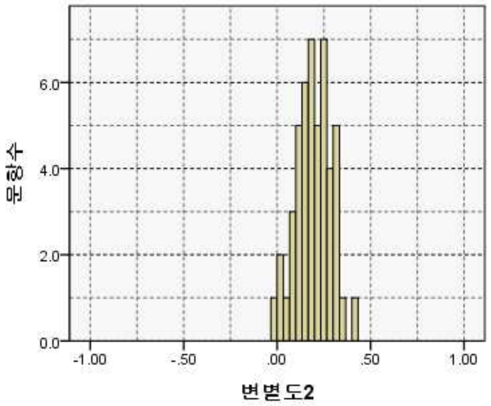

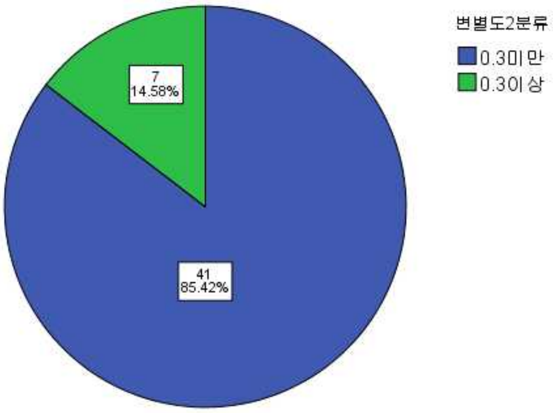

총점	변별도2	표준편차
48	.20	.09

변별도2	문항수	비율(%)
0.3미만	41	85.4
0.3이상	7	14.6
전체	48	100.0

해석

- 침구학 과목에서 난이도 지수가 80 에서 100 사이인 문항이 전체 48 문항 중 30 항으로 가장 많았으며, 다음으로 60 이상 80 미만인 문항이 10 항, 60 미만인 문항이 8 문항으로 나타남
- 변별도 1 지수를 기준으로 분류하였을 때, 0.3 미만인 문항이 43 문항으로 0.3 이상인 문항이 5 문항인 것에 비해 더 많이 나타남
- 변별도 2 지수를 기준으로 분류하였을 때, 0.3 미만인 문항이 41 문항으로 0.3 이상인 문항이 7 문항인 것에 비해 더 많이 나타남

(3) 보건의약관계법규 난이도와 변별도 분포도 및 비율분석

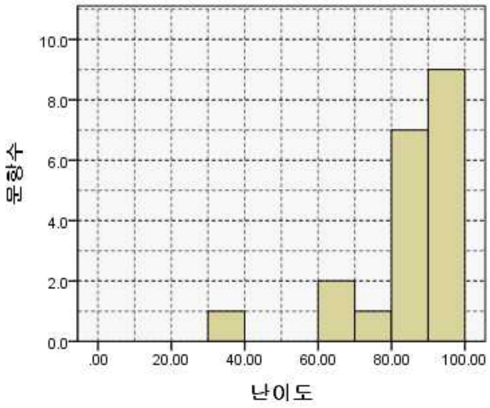

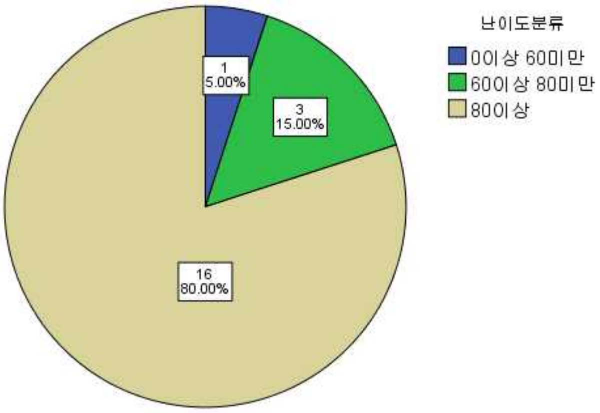

총점	난이도	표준편차
20	84.7	15.9

난이도	문항수	비율(%)
0~60미만	1	5.0
60~80미만	3	15.0
80~100	16	80.0
전체	20	100.0

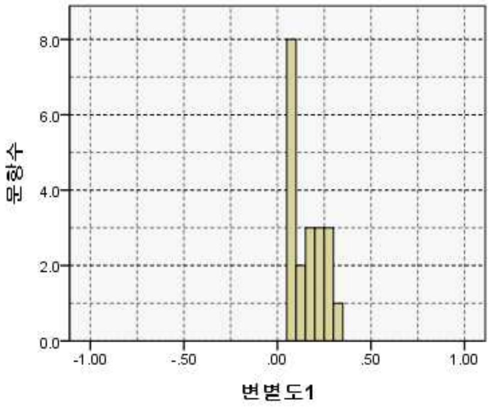

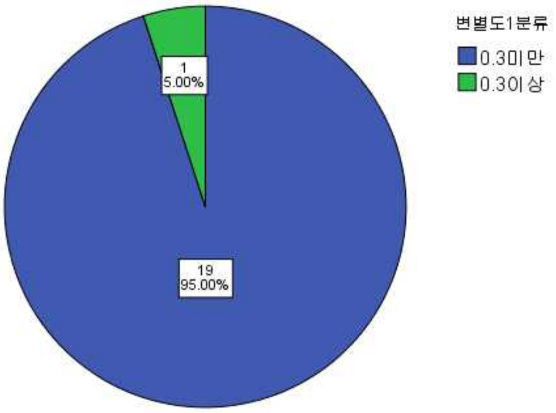

총점	변별도1	표준편차
20	.16	.09

변별도1	문항수	비율(%)
0.3미만	19	95.0
0.3이상	1	5.0
전체	20	100.0

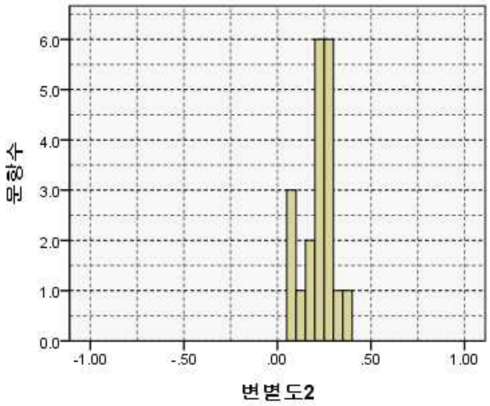

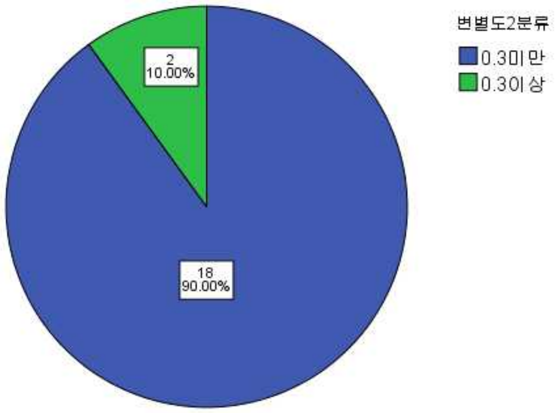

총점	변별도2	표준편차
20	.22	.08

변별도2	문항수	비율(%)
0.3미만	18	90.0
0.3이상	2	10.0
전체	20	100.0

해석

- 보건의약관계법규 과목에서 난이도 지수가 80에서 100 사이인 문항이 전체 20문항 중 16문항으로 가장 많았으며, 다음으로 60이상 80미만인 문항이 3문항, 60 미만인 문항이 1문항으로 나타남
- 변별도1 지수를 기준으로 분류하였을 때, 0.3미만인 문항이 19문항으로 0.3 이상인 문항이 1문항인 것에 비해 더 많이 나타남
- 변별도2 지수를 기준으로 분류하였을 때, 0.3미만인 문항이 18문항으로 0.3 이상인 문항이 2문항인 것에 비해 더 많이 나타남

(4) 외과학 난이도와 변별도 분포도 및 비율분석

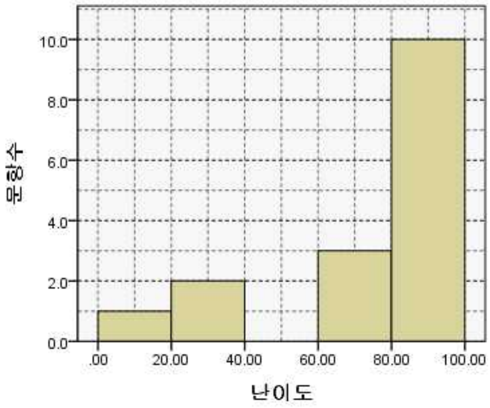

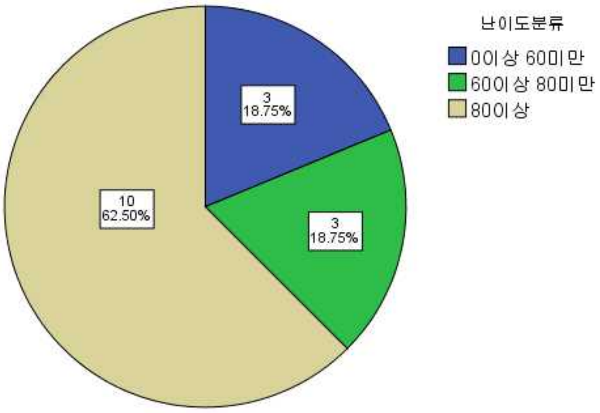

총점	난이도	표준편차
16	73.6	26.5

난이도	문항수	비율(%)
0~60미만	3	18.8
60~80미만	3	18.8
80~100	10	62.5
전체	16	100.0

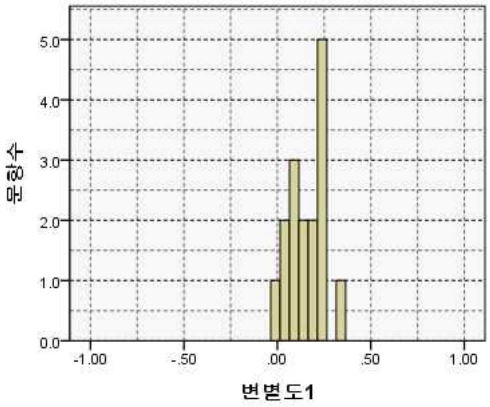

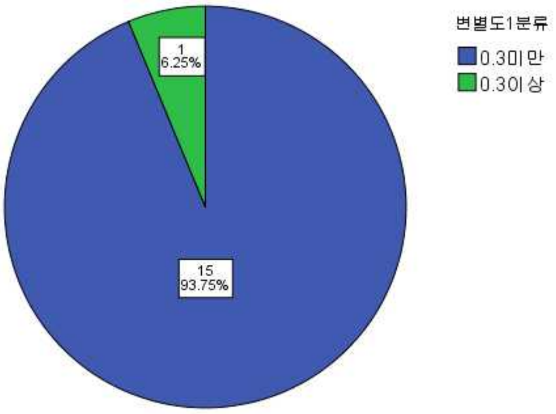

총점	변별도1	표준편차
16	.16	.09

변별도1	문항수	비율(%)
0.3미만	15	93.8
0.3이상	1	6.2
전체	16	100.0

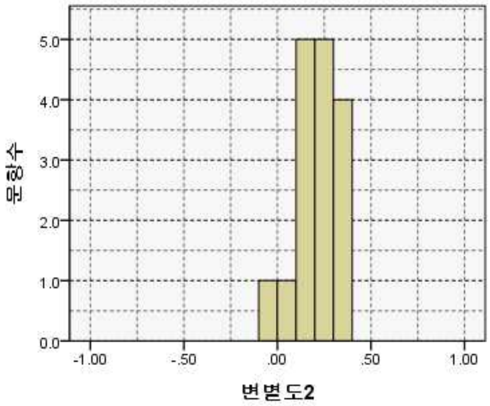

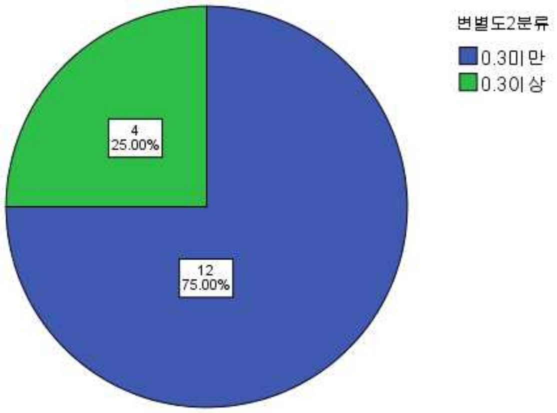

총점	변별도2	표준편차
16	.21	.11

변별도2	문항수	비율(%)
0.3미만	12	75.0
0.3이상	4	25.0
전체	16	100.0

해석

- 외과학 과목에서 난이도 지수가 80 에서 100 사이인 문항이 전체 16 문항 중 10 문항으로 가장 많았으며, 다음으로 60 이상 80 미만인 문항이 3 문항, 60 미만인 문항이 3 문항으로 나타남
- 변별도 1 지수를 기준으로 분류하였을 때, 0.3 미만인 문항이 15 문항으로 0.3 이상인 문항이 1 문항인 것에 비해 더 많이 나타남
- 변별도 2 지수를 기준으로 분류하였을 때, 0.3 미만인 문항이 12 문항으로 0.3 이상인 문항이 4 문항인 것에 비해 더 많이 나타남

(5) 신경정신과학 난이도와 변별도 분포도 및 비율분석

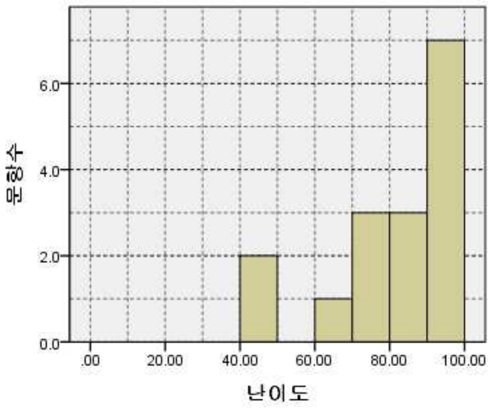

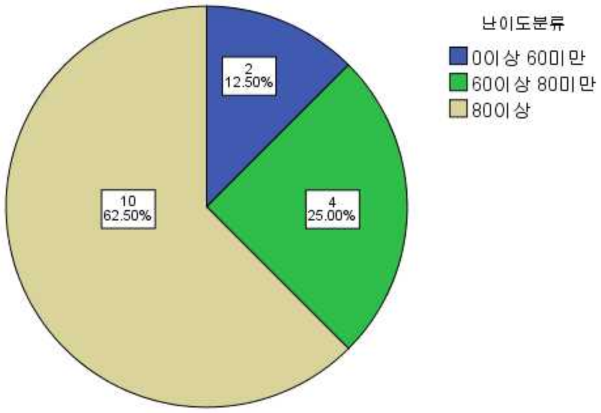

총점	난이도	표준편차	난이도	문항수	비율(%)
16	80.5	18.3	0~60미만	2	12.5
			60~80미만	4	25.0
			80~100	10	62.5
			전체	16	100.0

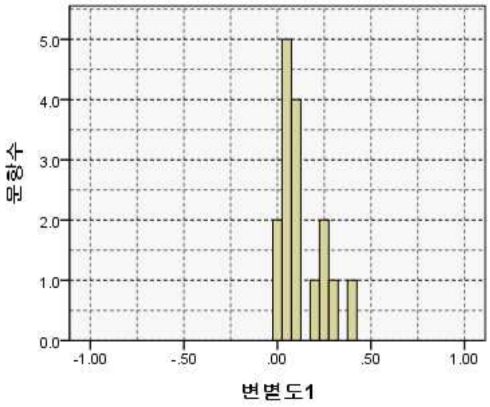

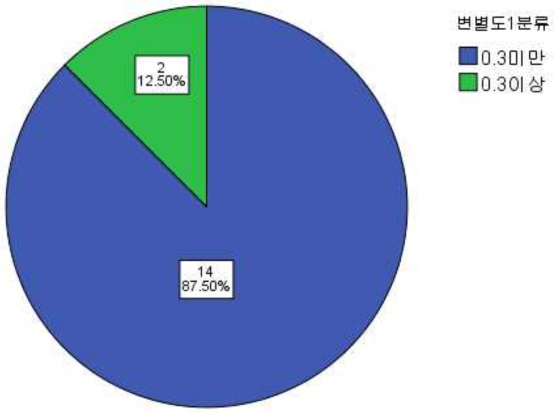

총점	변별도1	표준편차	변별도1	문항수	비율(%)
16	.13	.11	0.3미만	14	87.5
			0.3이상	2	12.5
			전체	16	100.0

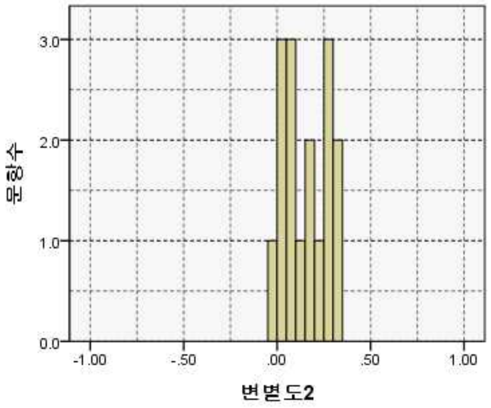

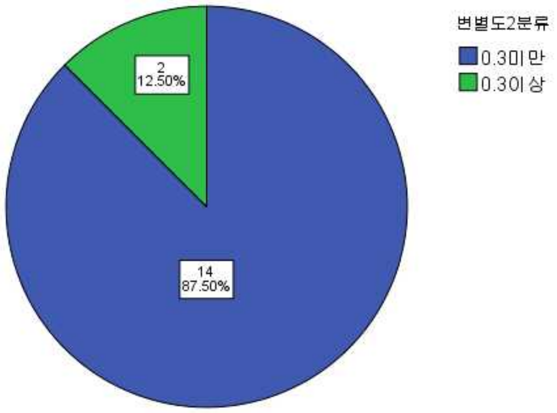

총점	변별도2	표준편차	변별도2	문항수	비율(%)
16	.16	.11	0.3미만	14	87.5
			0.3이상	2	12.5
			전체	16	100.0

해석

- 신경정신과학 과목에서 난이도 지수가 80에서 100 사이인 문항이 전체 16문항 중 10문항으로 가장 많았으며, 다음으로 60 이상 80 미만인 문항이 4문항, 60 미만인 문항이 2문항으로 나타남
- 변별도 1 지수를 기준으로 분류하였을 때, 0.3 미만인 문항이 14문항으로 0.3 이상인 문항이 2문항인 것에 비해 더 많이 나타남
- 변별도 2 지수를 기준으로 분류하였을 때, 0.3 미만인 문항이 14문항으로 0.3 이상인 문항이 2문항인 것에 비해 더 많이 나타남

(6) 안이비인후과 난이도와 변별도 분포도 및 비율분석

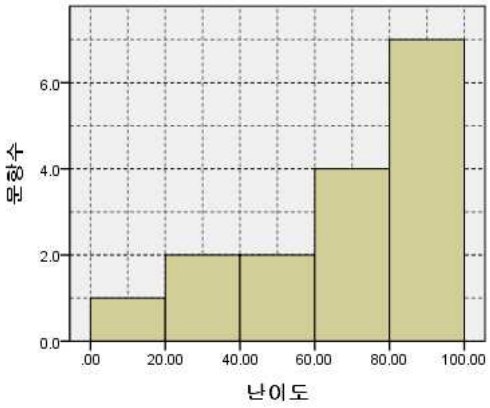

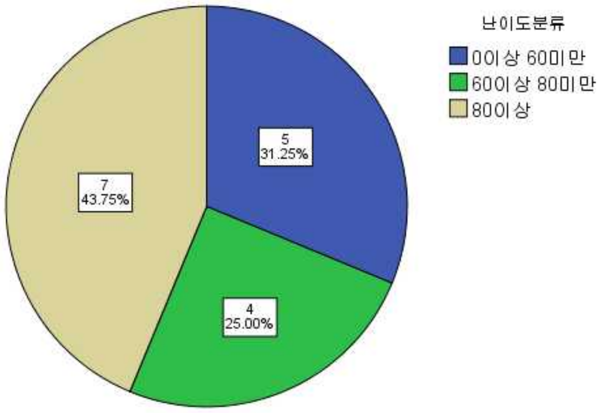

총점	난이도	표준편차
16	68.3	24.8

난이도	문항수	비율(%)
0~60미만	5	31.3
60~80미만	4	25.0
80~100	7	43.7
전체	16	100.0

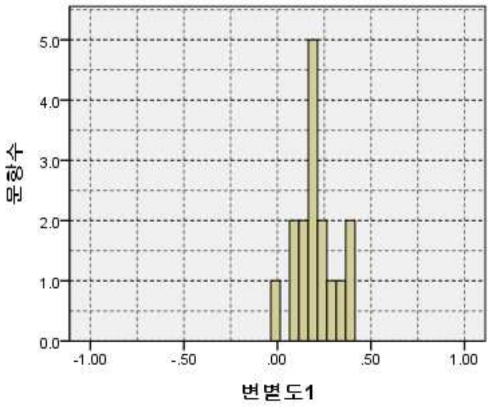

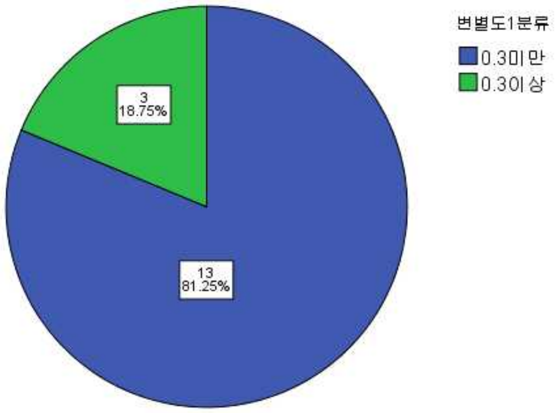

총점	변별도1	표준편차
16	.20	.11

변별도1	문항수	비율(%)
0.3미만	13	81.3
0.3이상	3	18.7
전체	16	100.0

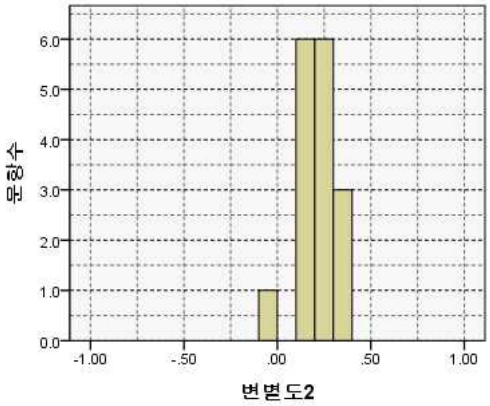

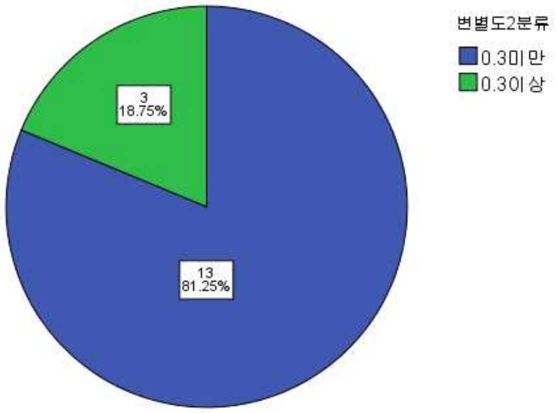

총점	변별도2	표준편차
16	.21	.11

변별도2	문항수	비율(%)
0.3미만	13	81.3
0.3이상	3	18.7
전체	16	100.0

해석

- 안이비인후과학 과목에서 난이도 지수가 80 에서 100 사이인 문항이 전체 16 문항 중 7 문항으로 나타났으며, 다음으로 60 이상 80 미만인 문항이 4 문항, 60 미만인 문항이 5 문항으로 나타남
- 변별도 1 지수를 기준으로 분류하였을 때, 0.3 미만인 문항이 13 문항으로 0.3 이상인 문항이 3 문항인 것에 비해 더 많이 나타남
- 변별도 2 지수를 기준으로 분류하였을 때, 0.3 미만인 문항이 13 문항으로 0.3 이상인 문항이 3 문항인 것에 비해 더 많이 나타남

(7) 부인과학 난이도와 변별도 분포도 및 비율분석

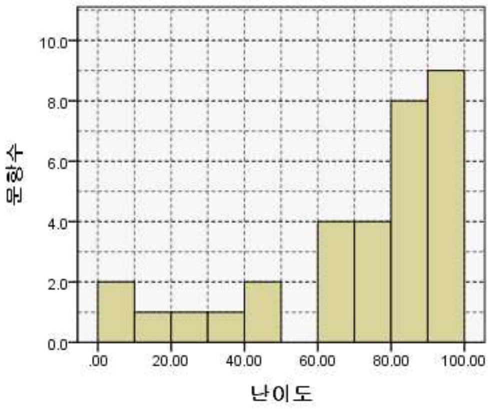

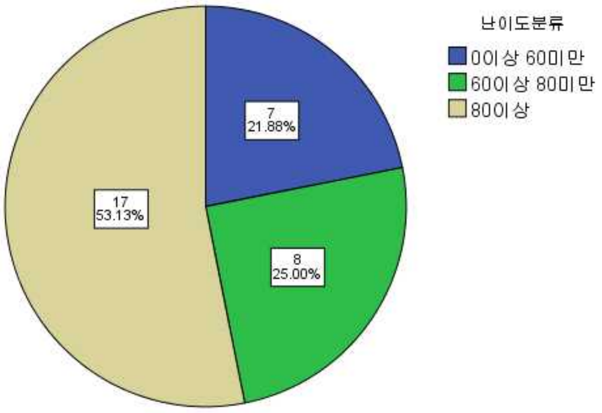

총점	난이도	표준편차
32	70.9	26.9

난이도	문항수	비율(%)
0~60미만	7	21.9
60~80미만	8	25.0
80~100	17	53.1
전체	32	100.0

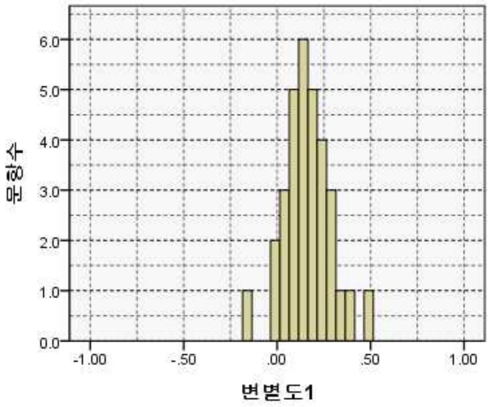

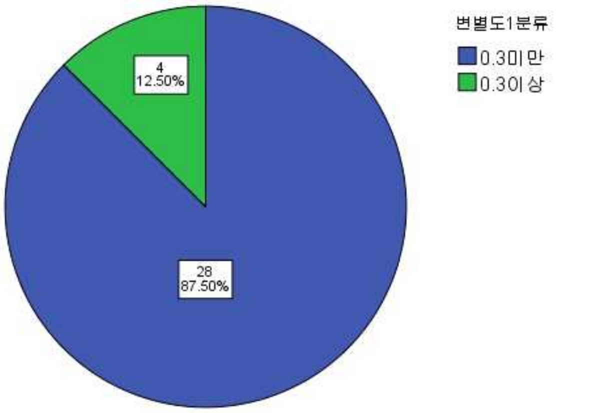

총점	변별도1	표준편차
32	.16	.13

변별도1	문항수	비율(%)
0.3미만	28	87.5
0.3이상	4	12.5
전체	32	100.0

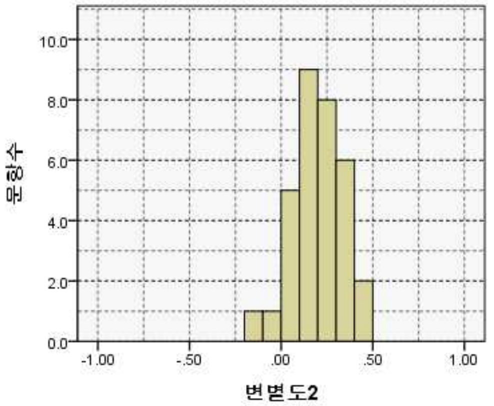

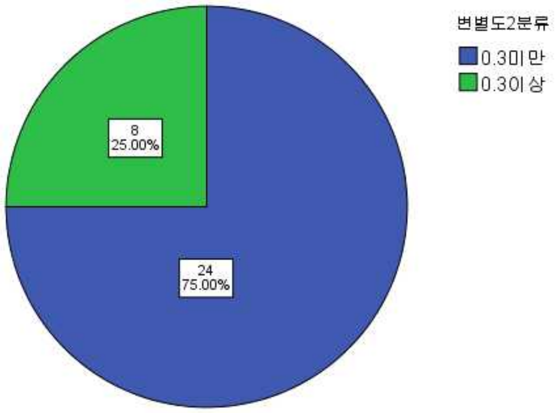

총점	변별도2	표준편차
32	.20	.14

변별도2	문항수	비율(%)
0.3미만	24	75.0
0.3이상	8	25.0
전체	32	100.0

해석

- 부인과학 과목에서 난이도 지수가 80 에서 100 사이인 문항이 전체 32 문항 중 17 문항으로 가장 많았으며, 60 이상 80 미만인 문항이 8 문항, 60 미만인 문항이 7 문항으로 나타남
- 변별도 1 지수를 기준으로 분류하였을 때, 0.3 미만인 문항이 28 문항으로 0.3 이상인 문항이 4 문항인 것에 비해 더 많이 나타남
- 변별도 2 지수를 기준으로 분류하였을 때, 0.3 미만인 문항이 24 문항으로 0.3 이상인 문항이 8 문항인 것에 비해 더 많이 나타남

(8) 소아과학 난이도와 변별도 분포도 및 비율분석

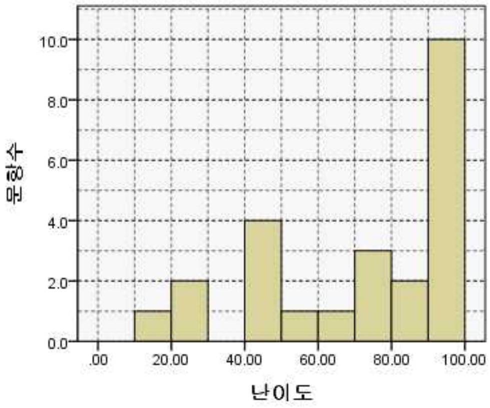

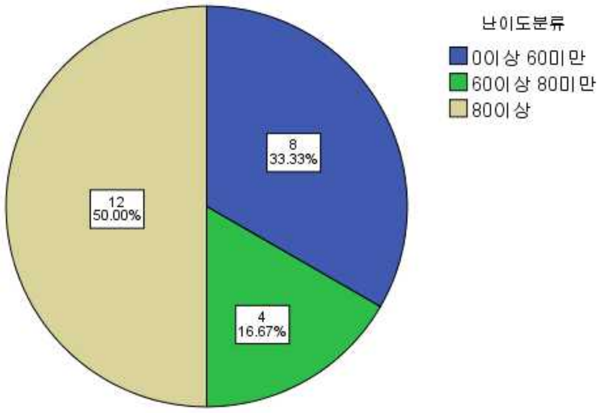

총점	난이도	표준편차
24	71.3	25.7

난이도	문항수	비율(%)
0~60미만	8	33.3
60~80미만	4	16.7
80~100	12	50.0
전체	24	100.0

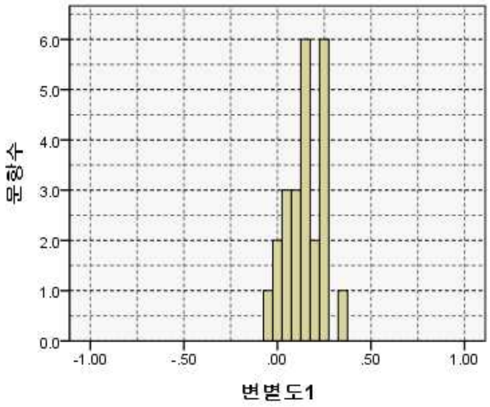

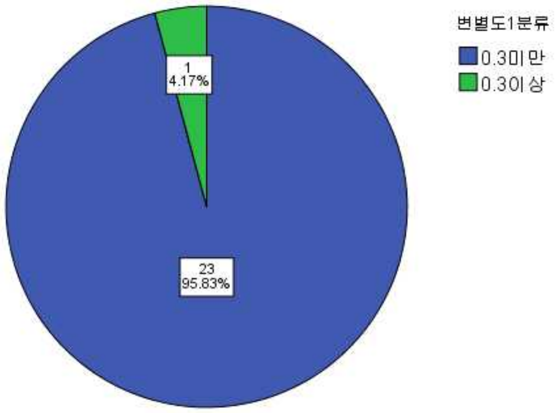

총점	변별도1	표준편차
24	.15	.10

변별도1	문항수	비율(%)
0.3미만	23	95.8
0.3이상	1	4.2
전체	24	100.0

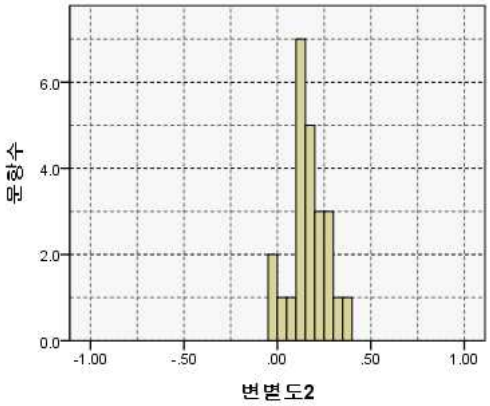

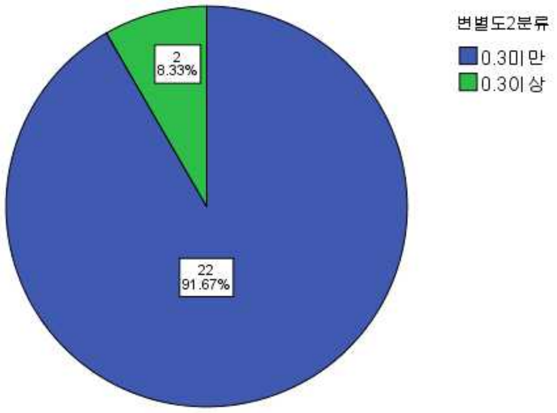

총점	변별도2	표준편차	변별도2	문항수	비율(%)
24	.17	.10	0.3미만	22	91.7
			0.3이상	2	8.3
			전체	24	100.0

해석

- 소아과학 과목에서 난이도 지수가 80 에서 100 사이인 문항이 전체 24 문항 중 12 문항이었으며, 60 이상 80 미만인 문항이 4 문항, 60 미만인 문항이 8 문항으로 나타남
- 변별도 1 지수를 기준으로 분류하였을 때, 0.3 미만인 문항이 23 문항으로 0.3 이상인 문항이 1 문항인 것에 비해 더 많이 나타남
- 변별도 2 지수를 기준으로 분류하였을 때, 0.3 미만인 문항이 22 문항으로 0.3 이상인 문항이 2 문항인 것에 비해 더 많이 나타남

(9) 예방의학 난이도와 변별도 분포도 및 비율분석

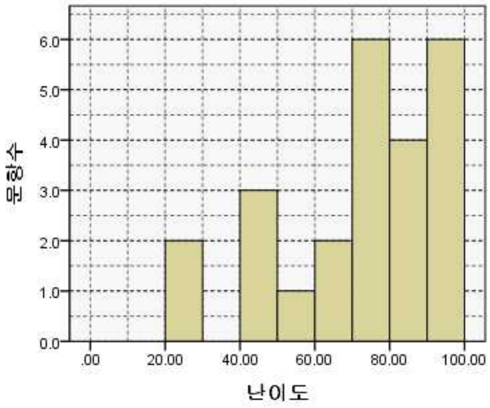

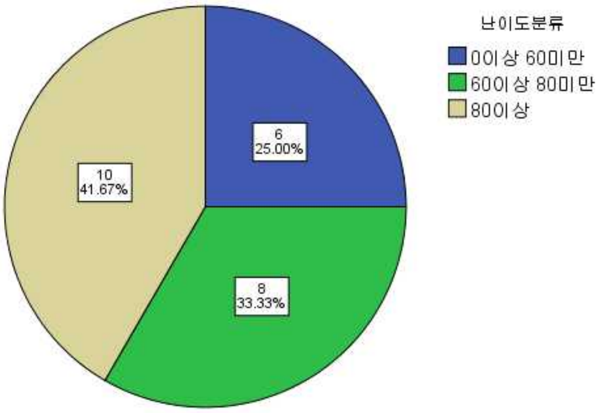

총점	난이도	표준편차
24	72.1	23.2

난이도	문항수	비율(%)
0~60미만	6	25.0
60~80미만	8	33.3
80~100	10	41.7
전체	24	100.0

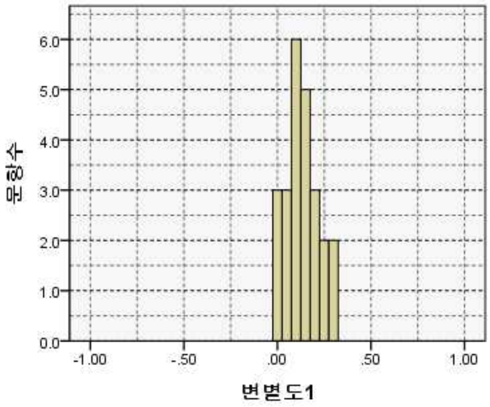

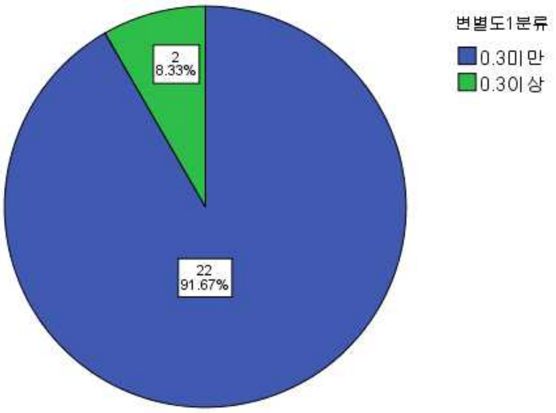

총점	변별도1	표준편차
24	.14	.09

변별도1	문항수	비율(%)
0.3미만	22	91.7
0.3이상	2	8.3
전체	24	100.0

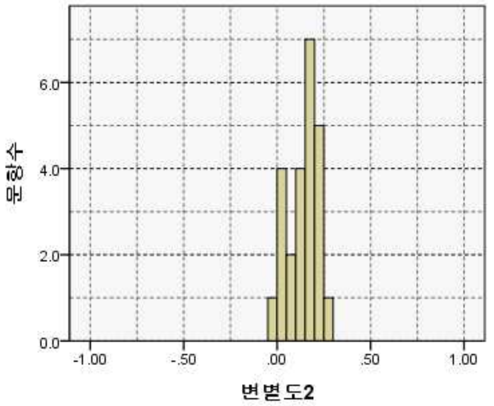

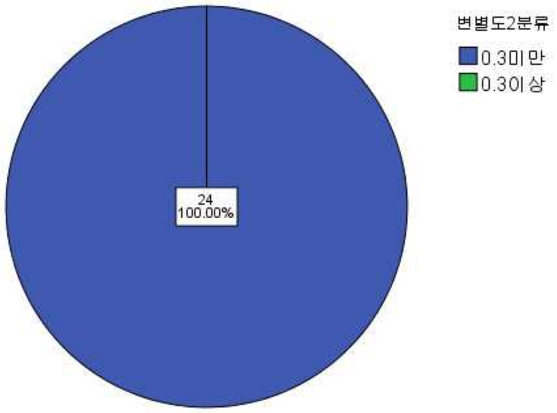

총점	변별도2	표준편차
24	.14	.08

변별도2	문항수	비율(%)
0.3미만	24	100.0
0.3이상	0	0.0
전체	24	100.0

해석

- 예방의학 과목에서 난이도 지수가 80 에서 100 사이인 문항이 전체 24 문항 중 10 문항으로 가장 많았으며, 60 이상 80 미만인 문항이 8 문항, 60 미만인 문항이 6 문항으로 나타남
- 변별도 1 지수를 기준으로 분류하였을 때, 0.3 미만인 문항이 22 문항으로 0.3 이상인 문항이 2 문항인 것에 비해 더 많이 나타남
- 변별도 2 지수를 기준으로 분류하였을 때, 0.3 미만인 문항이 24 문항으로 0.3 이상인 문항이 0 문항인 것에 비해 더 많이 나타남

(10) 한방생리학 난이도와 변별도 분포도 및 비율분석

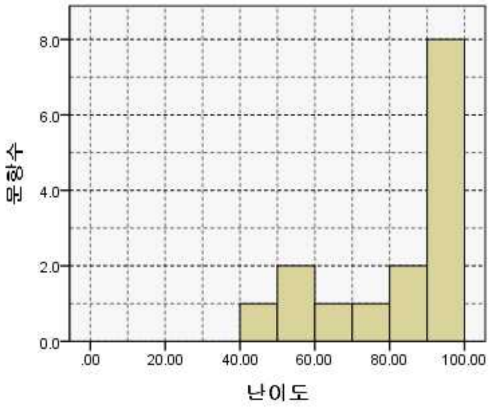

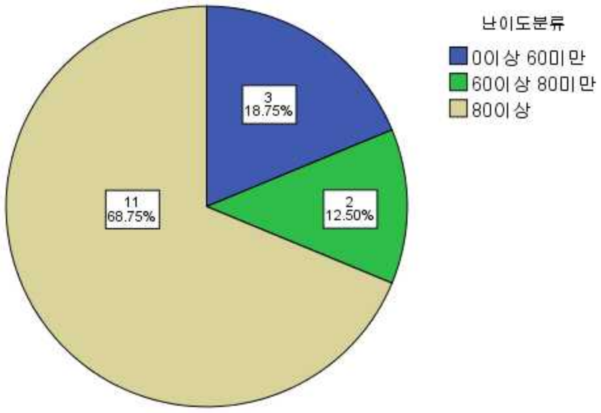

총점	난이도	표준편차	난이도	문항수	비율(%)
16	82.2	18.6	0~60미만	3	18.8
			60~80미만	2	12.5
			80~100	11	68.8
			전체	16	100.0

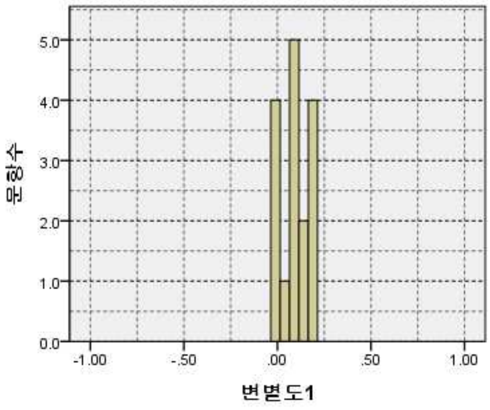

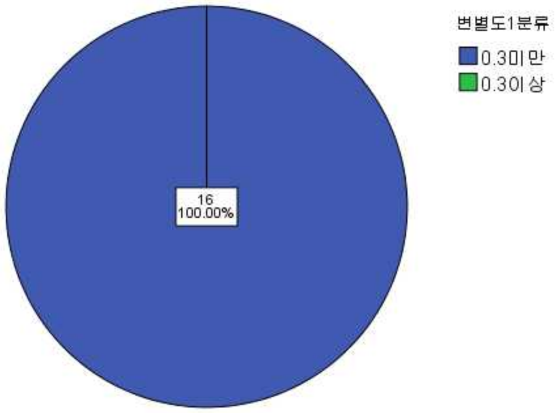

총점	변별도1	표준편차	변별도1	문항수	비율(%)
16	.10	.07	0.3미만	16	100.0
			0.3이상	0	0.0
			전체	16	100.0

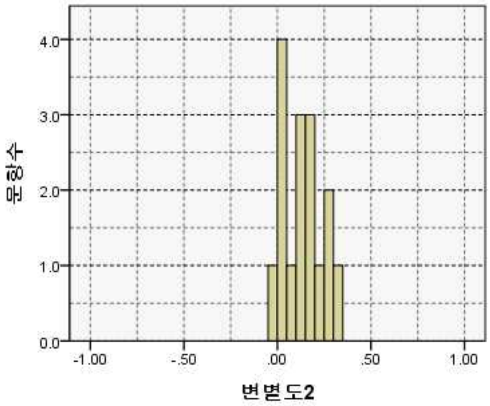

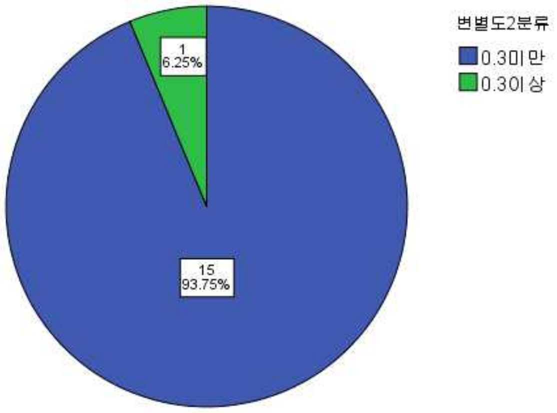

총점	변별도2	표준편차
16	.13	.10

변별도2	문항수	비율(%)
0.3미만	15	93.8
0.3이상	1	6.2
전체	16	100.0

해석

- 한방생리학 과목에서 난이도 지수가 80 에서 100 사이인 문항이 전체 16 문항 중 11 문항으로 가장 많았으며, 다음으로 60 이상 80 미만인 문항이 2 문항, 60 미만인 문항이 3 문항으로 나타남
- 변별도 1 지수를 기준으로 분류하였을 때, 0.3 미만인 문항이 16 문항으로 0.3 이상인 문항이 0 문항인 것에 비해 더 많이 나타남
- 변별도 2 지수를 기준으로 분류하였을 때, 0.3 미만인 문항이 15 문항으로 0.3 이상인 문항이 1 문항인 것에 비해 더 많이 나타남

(11) 본초학 난이도와 변별도 분포도 및 비율분석

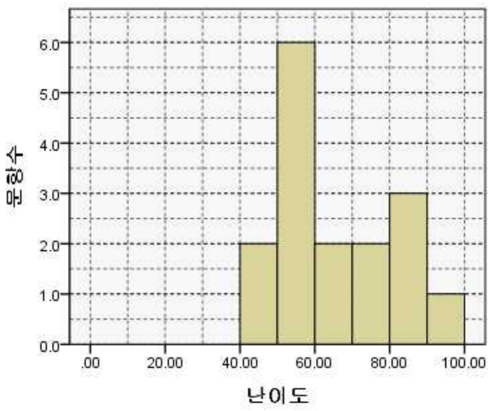

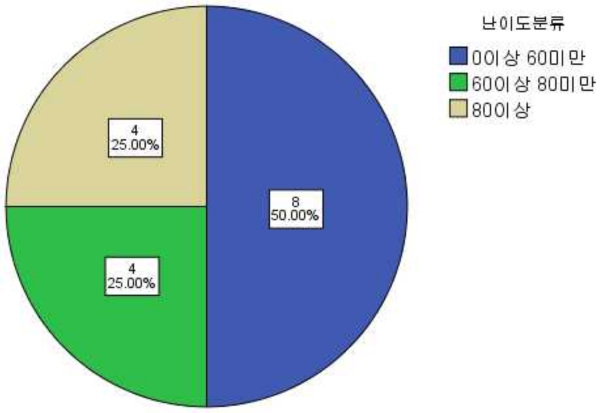

총점	난이도	표준편차
16	65.9	16.2

난이도	문항수	비율(%)
0~60미만	8	50.0
60~80미만	4	25.0
80~100	4	25.0
전체	16	100.0

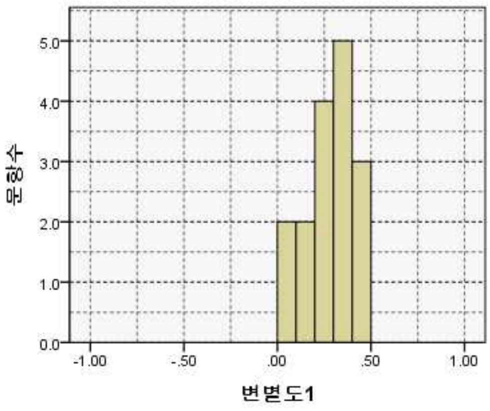

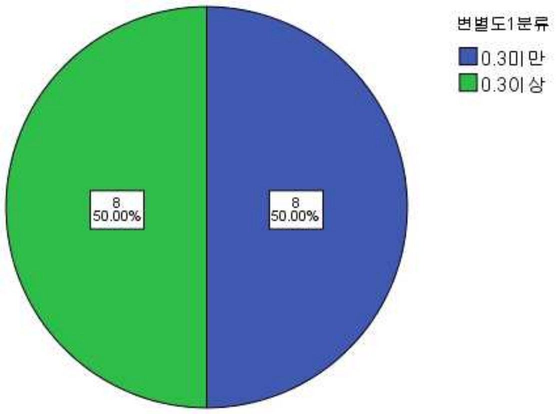

총점	변별도1	표준편차
16	.28	.12

변별도1	문항수	비율(%)
0.3미만	8	50.0
0.3이상	8	50.0
전체	16	100.0

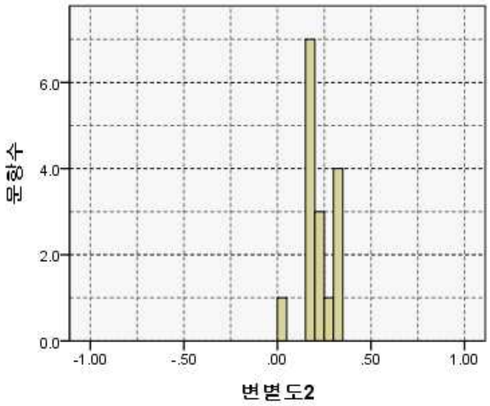

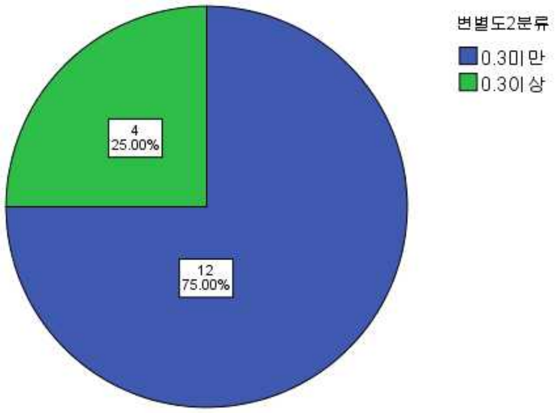

총점	변별도2	표준편차
16	.22	.08

변별도2	문항수	비율(%)
0.3미만	12	75.0
0.3이상	4	25.0
전체	16	100.0

해석

- 본초학 과목에서 난이도 지수가 80에서 100 사이인 문항이 전체 16문항 중 4문항이었으며, 60이상 80미만인 문항이 4문항, 60 미만인 문항이 8문항으로 나타남
- 변별도1 지수를 기준으로 분류하였을 때, 0.3미만인 문항이 8문항으로 0.3이상인 문항이 8문항인 것과 동일하게 나타남
- 변별도2 지수를 기준으로 분류하였을 때, 0.3미만인 문항이 12문항으로 0.3이상인 문항이 4문항인 것에 비해 더 많이 나타남

3) 지식수준별 난이도와 변별도

가) 전회 대비 지식수준별 난이도와 변별도

(1) 전회 대비 암기형 난이도와 변별도

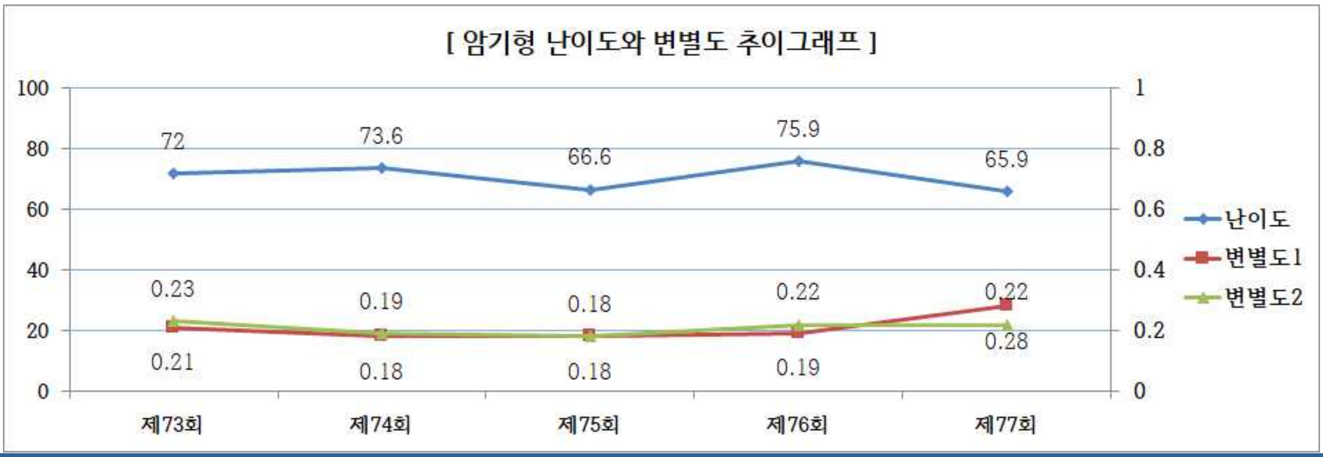

회차	난이도		변별도1		변별도2	
	평균	표준편차	평균	표준편차	평균	표준편차
제73회	72.0	22.5	.21	.12	.23	.11
제74회	73.6	22.5	.18	.12	.19	.10
제75회	66.6	24.7	.18	.11	.18	.09
제76회	75.9	21.5	.19	.11	.22	.10
제77회	65.9	16.2	.28	.12	.22	.08

(2) 전회 대비 해석형 난이도와 변별도

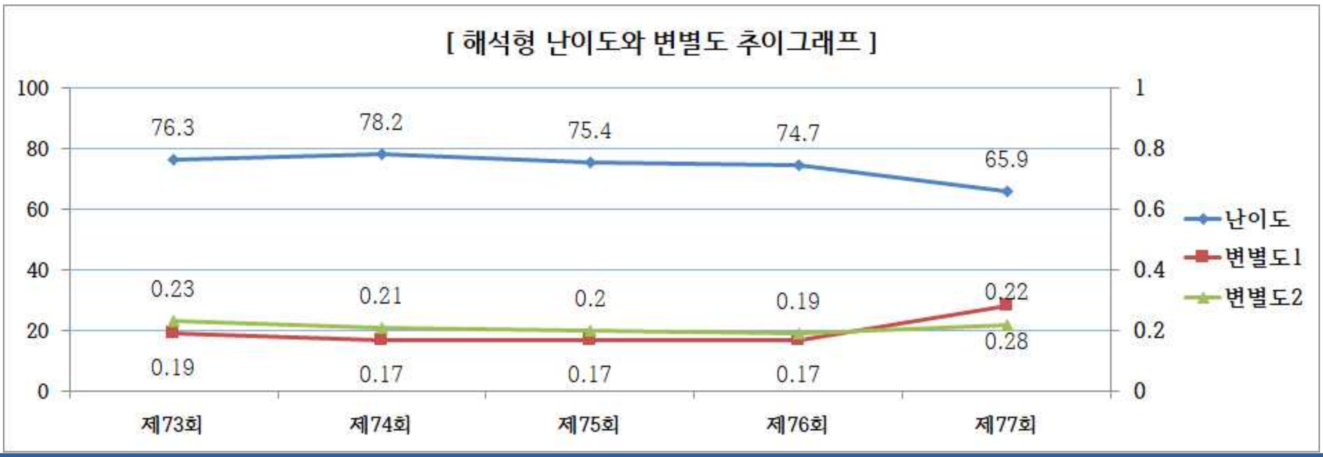

회차	난이도		변별도1		변별도2	
	평균	표준편차	평균	표준편차	평균	표준편차
제73회	76.3	21.5	.19	.12	.23	.10
제74회	78.2	19.0	.17	.11	.21	.09
제75회	75.4	20.5	.17	.12	.20	.10
제76회	74.7	22.4	.17	.13	.19	.11
제77회	65.9	16.2	.28	.12	.22	.08

(3) 전회 대비 해결형 난이도와 변별도

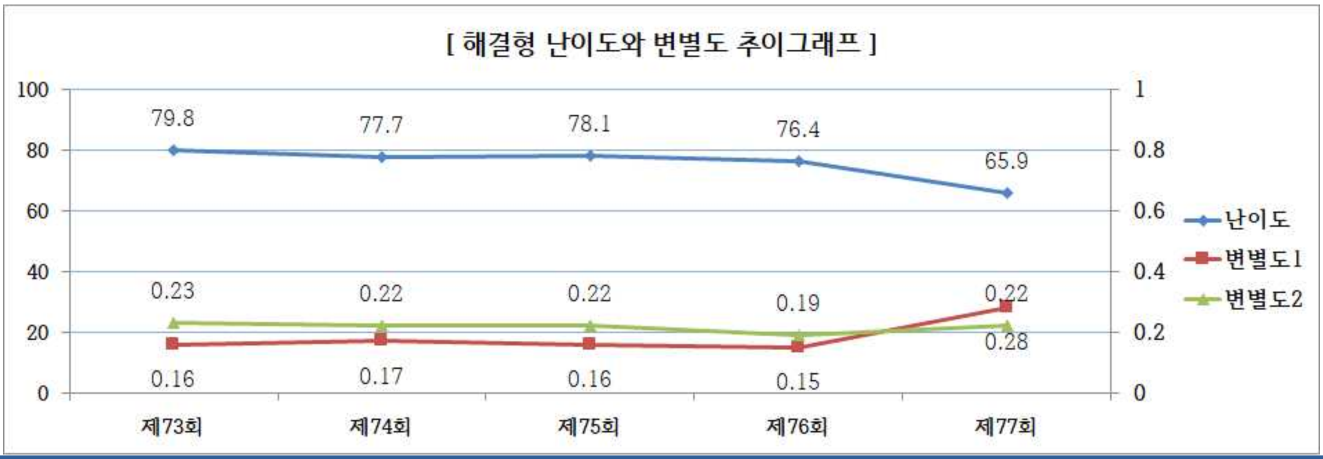

회차	난이도		변별도1		변별도2	
	평균	표준편차	평균	표준편차	평균	표준편차
제73회	79.8	18.0	.16	.12	.23	.11
제74회	77.7	19.3	.17	.11	.22	.11
제75회	78.1	19.6	.16	.11	.22	.10
제76회	76.4	22.5	.15	.11	.19	.11
제77회	65.9	16.2	.28	.12	.22	.08

해석

- 전회 대비 암기형 문항의 난이도 지수는 10.0 감소하였으며, 해석형 문항의 난이도 지수는 8.8 감소하였고 해결형 문항의 난이도 지수는 10.5 감소함
- 변별도 1 지수는 암기형 문항에서 .09 증가하였고, 해석형 문항에서는 .11 증가하였으며, 해결형 문항에서는 .13 증가함
- 변별도 2 지수는 암기형 문항에서 변하지 않았고, 해석형 문항에서는 .03 증가하였으며, 해결형 문항에서는 .03 증가함

나) 지식수준별 난이도와 변별도 분포도 및 비율분석

(1) 암기형 난이도와 변별도 분포도 및 비율분석

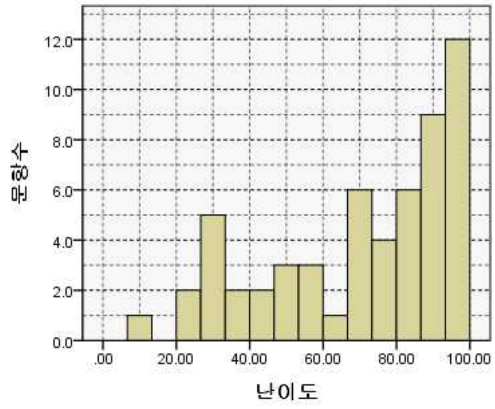

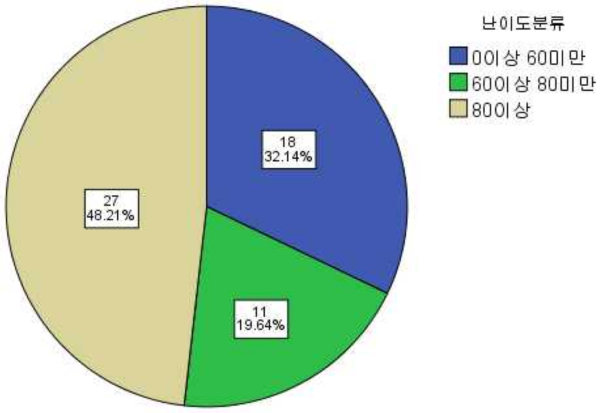

총점	난이도	표준편차
56	70.2	25.4

난이도	문항수	비율(%)
0~60미만	18	32.1
60~80미만	11	19.6
80~100	27	48.2
전체	56	100.0

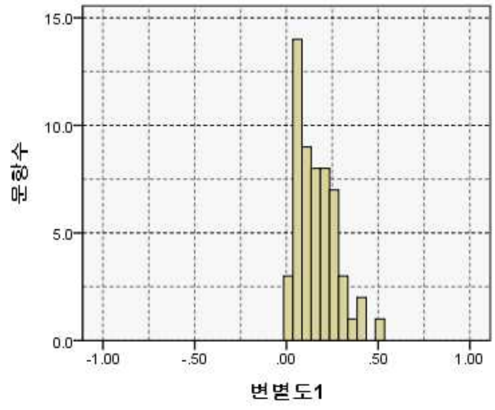

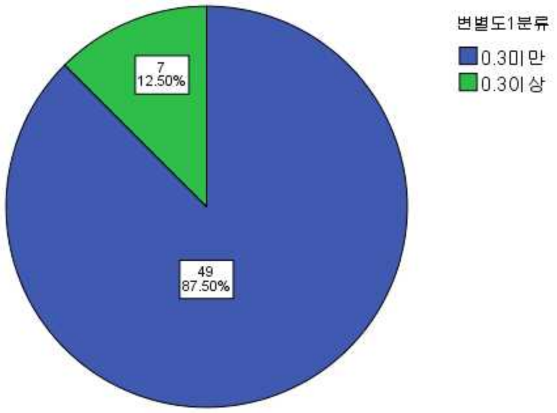

총점	변별도1	표준편차
56	.16	.11

변별도1	문항수	비율(%)
0.3미만	49	87.5
0.3이상	7	12.5
전체	56	100.0

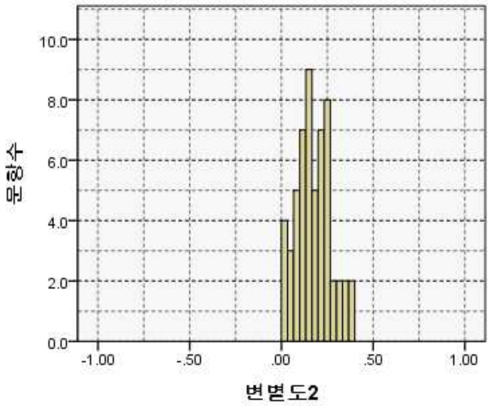

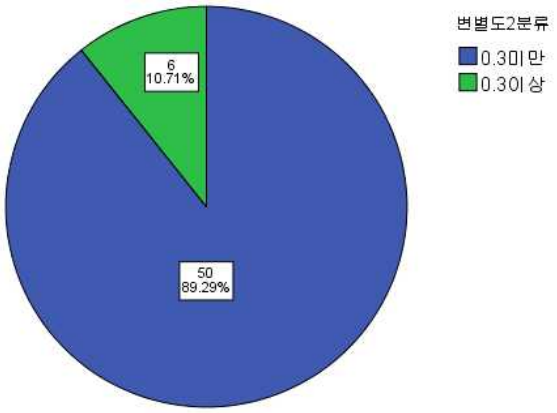

총점	변별도2	표준편차
56	.18	.09

변별도2	문항수	비율(%)
0.3미만	50	89.3
0.3이상	6	10.7
전체	56	100.0

해석

- 암기형 문항에서 난이도 지수가 80 에서 100 사이인 문항이 전체 56 문항 중 27 문항으로 가장 많았으며, 다음으로 60 이상 80 미만인 문항이 11 문항, 60 미만인 문항이 18 문항인 것으로 나타남
- 변별도 1 지수를 기준으로 하였을 때, 0.3 미만인 문항이 49 문항으로 0.3 이상인 문항이 7 문항인 것에 비해 더 많이 나타남
- 변별도 2 지수를 기준으로 분류하였을 때, 0.3 미만인 문항이 50 문항으로 0.3 이상인 문항이 6 문항인 것에 비해 더 많이 나타남

(2) 해석형 난이도와 변별도 분포도 및 비율분석

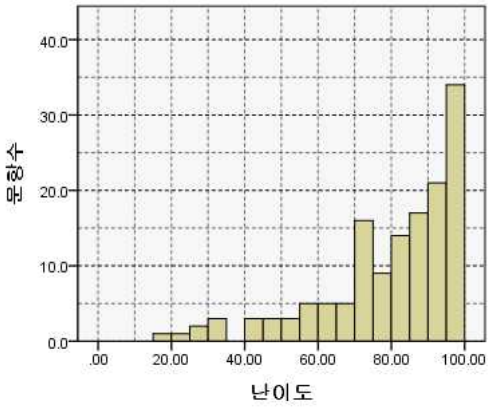

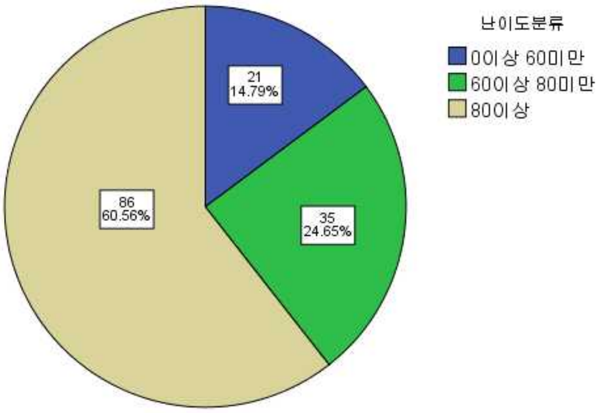

총점	난이도	표준편차
142	79.7	18.8

난이도	문항수	비율(%)
0~60미만	21	14.8
60~80미만	35	24.6
80~100	86	60.6
전체	142	100.0

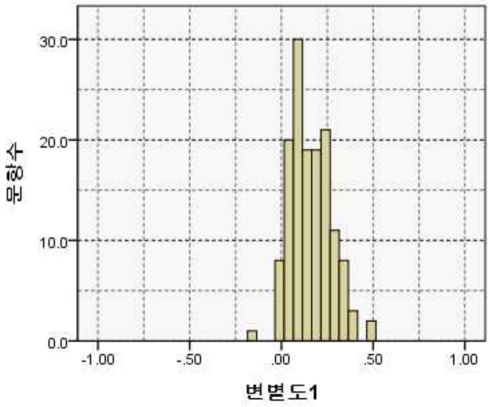

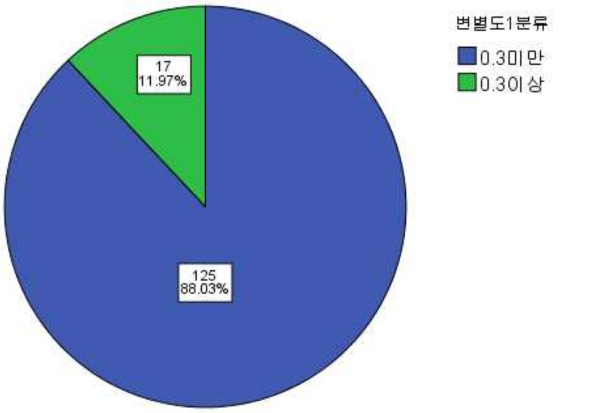

총점	변별도1	표준편차
142	.16	.11

변별도1	문항수	비율(%)
0.3미만	125	88.0
0.3이상	17	12.0
전체	142	100.0

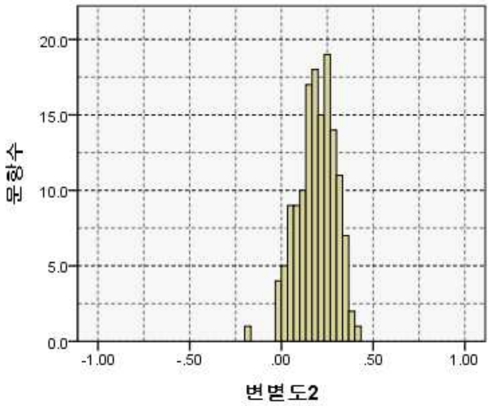

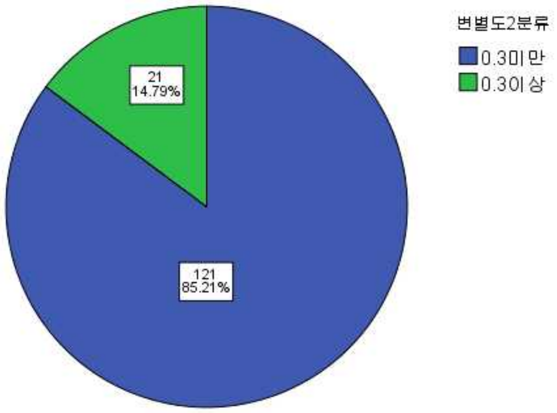

총점	변별도2	표준편차
142	.19	.10

변별도2	문항수	비율(%)
0.3미만	121	85.2
0.3이상	21	14.8
전체	142	100.0

해석

- 해석형 문항에서 난이도 지수가 80 에서 100 사이인 문항이 전체 142 문항 중 86 문항으로 가장 많았으며, 다음으로 60 이상 80 미만인 문항이 35 문항, 60 미만인 문항이 21 문항인 것으로 나타남
- 변별도 1 지수를 기준으로 하였을 때, 0.3 미만인 문항이 125 문항으로 0.3 이상인 문항이 17 문항인 것에 비해 더 많이 나타남
- 변별도 2 지수를 기준으로 분류하였을 때, 0.3 미만인 문항이 121 문항으로 0.3 이상인 문항이 21 문항인 것에 비해 더 많이 나타남

(3) 해결형 난이도와 변별도 분포도 및 비율분석

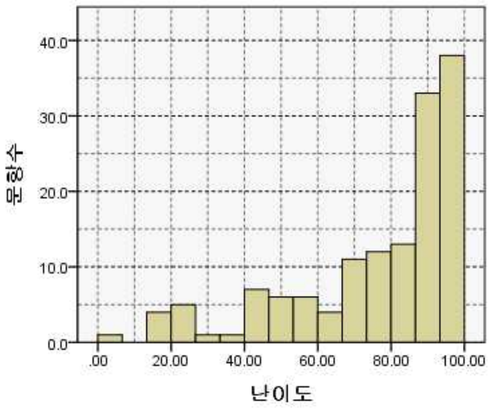

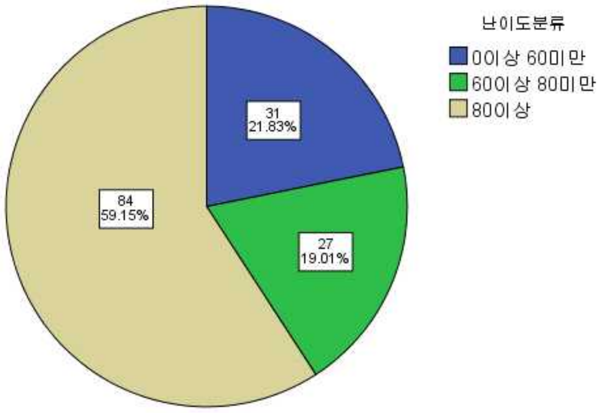

총점	난이도	표준편차
142	76.5	23.0

난이도	문항수	비율(%)
0~60미만	31	21.8
60~80미만	27	19.0
80~100	84	59.2
전체	142	100.0

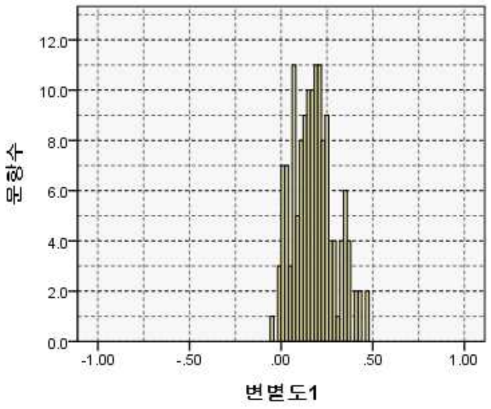

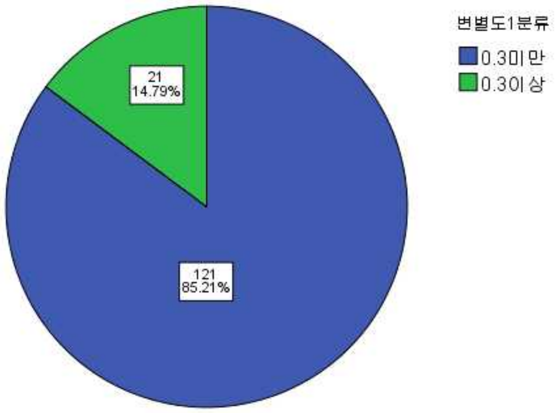

총점	변별도1	표준편차
142	.17	.11

변별도1	문항수	비율(%)
0.3미만	121	85.2
0.3이상	21	14.8
전체	142	100.0

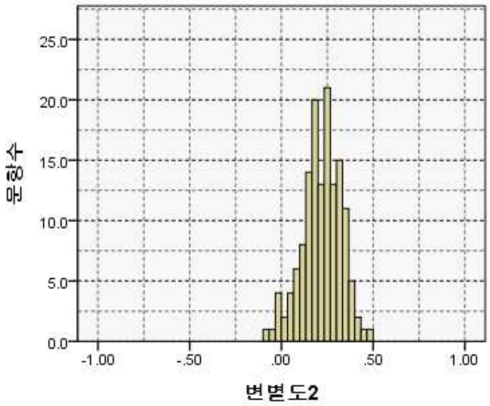

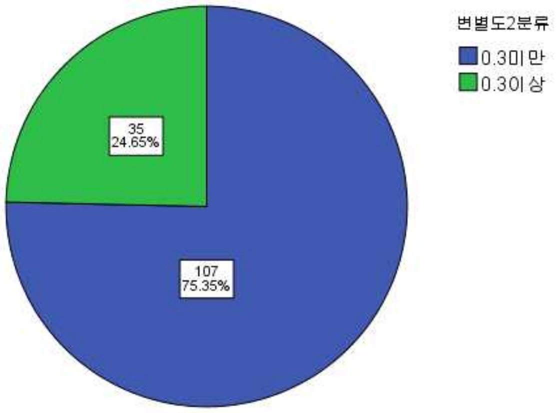

총점	변별도2	표준편차
142	.22	.11

변별도2	문항수	비율(%)
0.3미만	107	75.4
0.3이상	35	24.6
전체	142	100.0

해석

- 해결형 문항에서 난이도 지수가 80 에서 100 사이인 문항이 전체 142 문항 중 84 문항으로 가장 많았으며, 다음으로 60 이상 80 미만인 문항이 27 문항, 60 미만인 문항이 31 문항인 것으로 나타남
- 변별도 1 지수를 기준으로 하였을 때, 0.3 미만인 문항이 121 문항으로 0.3 이상인 문항이 21 문항인 것에 비해 더 많이 나타남
- 변별도 2 지수를 기준으로 분류하였을 때, 0.3 미만인 문항이 107 문항으로 0.3 이상인 문항이 35 문항인 것에 비해 더 많이 나타남

4) 자료유형별 난이도와 변별도

가) 전회 대비 자료유형별 난이도와 변별도

(1) 전회 대비 텍스트형 난이도와 변별도

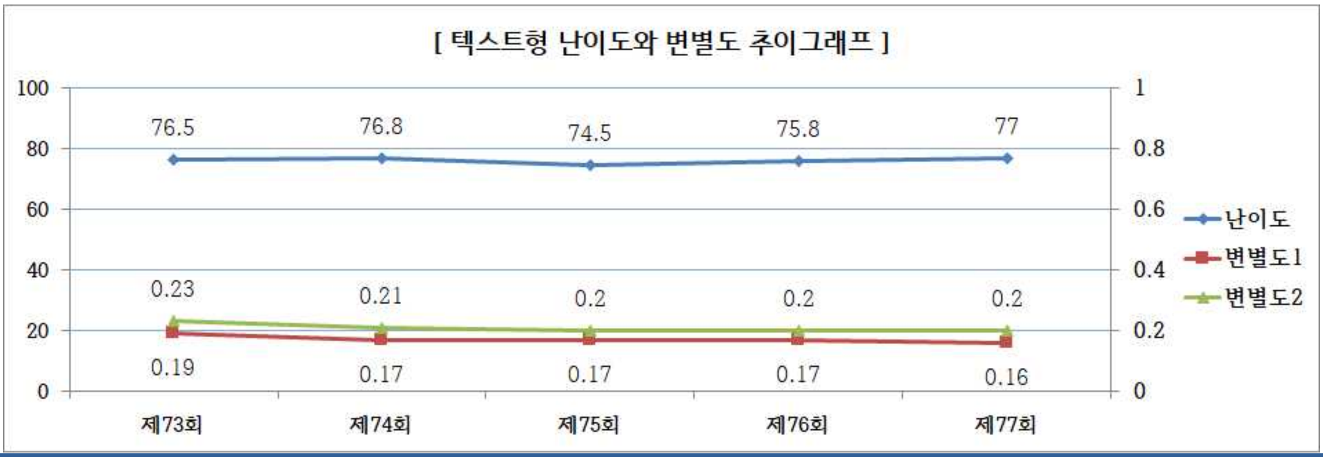

회차	난이도		변별도1		변별도2	
	평균	표준편차	평균	표준편차	평균	표준편차
제73회	76.5	20.6	.19	.12	.23	.10
제74회	76.8	20.0	.17	.11	.21	.10
제75회	74.5	21.5	.17	.11	.20	.10
제76회	75.8	22.3	.17	.12	.20	.10
제77회	77.0	21.9	.16	.11	.20	.11

(2) 전회 대비 자료제시형 난이도와 변별도

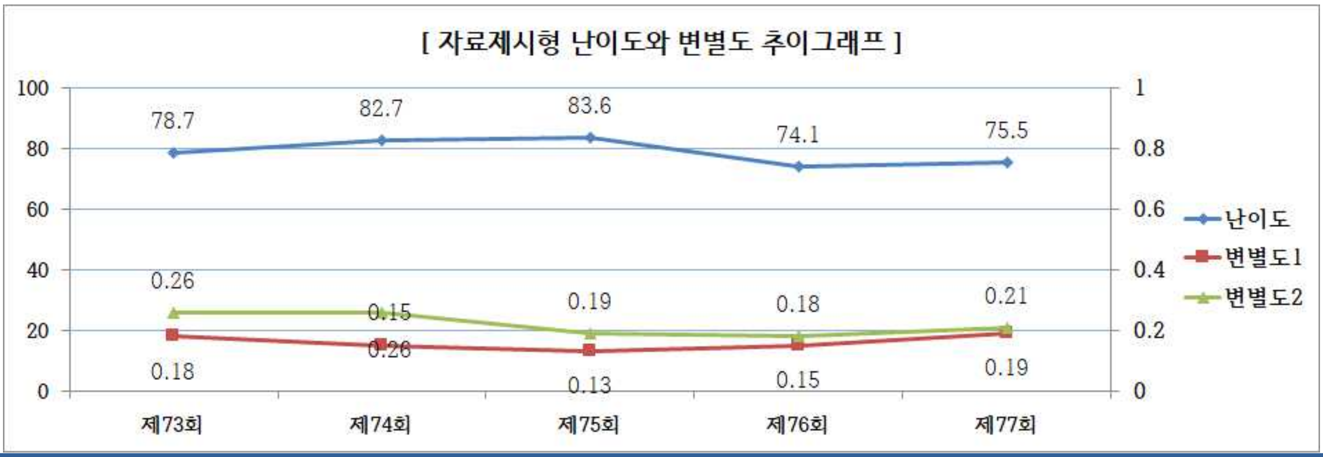

회차	난이도		변별도1		변별도2	
	평균	표준편차	평균	표준편차	평균	표준편차
제73회	78.7	25.5	.18	.12	.26	.08
제74회	82.7	20.0	.15	.10	.26	.10
제75회	83.6	15.2	.13	.10	.19	.12
제76회	74.1	22.0	.15	.12	.18	.11
제77회	75.5	22.3	.19	.12	.21	.09

해석

- 전회 대비 텍스트형 문항의 난이도 지수는 1.2 증가하였으며, 자료제시형 문항의 난이도 지수는 1.4 증가함
- 변별도 1 지수의 경우 텍스트형 문항에서는 .01 감소하였으며, 자료제시형 문항에서는 .04 증가함
- 변별도 2 지수의 경우 텍스트형 문항에서는 변하지 않았으며, 자료제시형 문항에서는 .03 증가함

나) 자료유형별 난이도와 변별도 분포도 및 비율분석

(1) 텍스트형 난이도와 변별도 분포도 및 비율분석

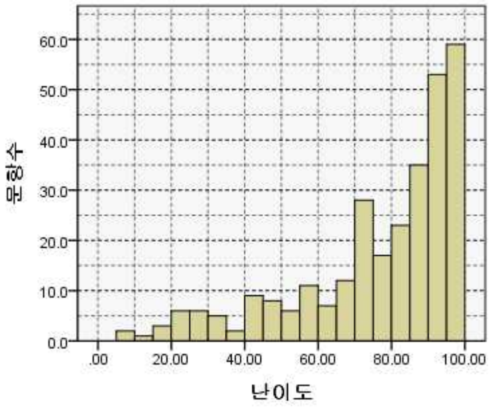

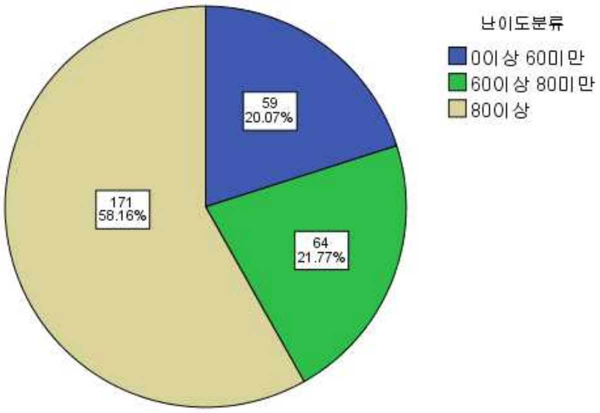

총점	난이도	표준편차
294	77.0	21.9

난이도	문항수	비율(%)
0~60미만	59	20.1
60~80미만	64	21.8
80~100	171	58.2
전체	294	100.0

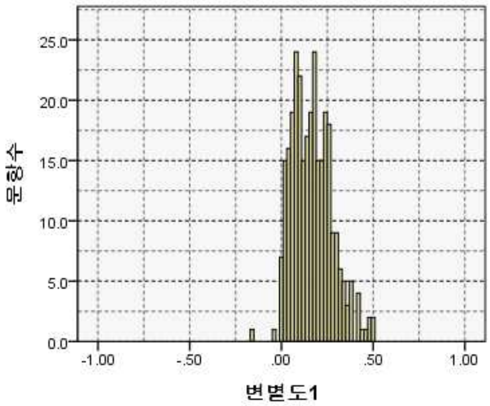

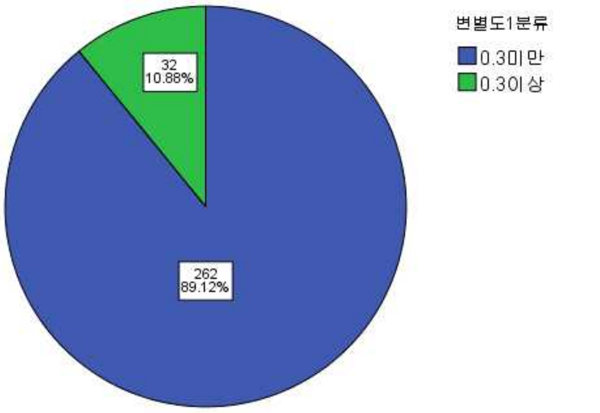

총점	변별도1	표준편차
294	.16	.11

변별도1	문항수	비율(%)
0.3미만	262	89.1
0.3이상	32	10.9
전체	294	100.0

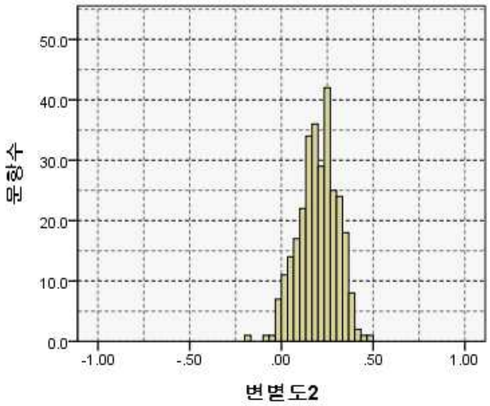

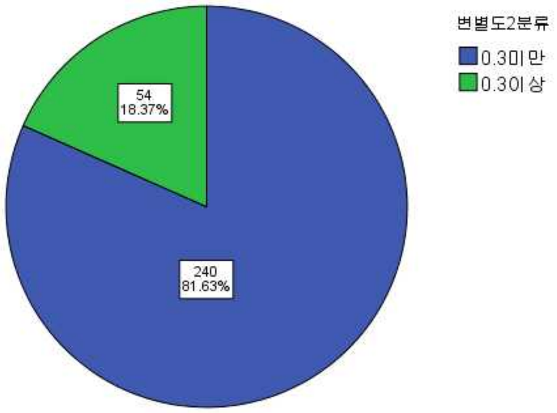

총점	변별도2	표준편차
294	.20	.11

변별도2	문항수	비율(%)
0.3미만	240	81.6
0.3이상	54	18.4
전체	294	100.0

해석

- 텍스트형 문항에서 난이도 지수가 80 에서 100 사이인 문항이 전체 294 문항 중 171 문항으로 가장 많았으며, 60 이상 80 미만인 문항이 64 문항, 60 미만인 문항이 59 문항인 것으로 나타남
- 변별도 1 지수를 기준으로 분류하였을 때, 0.3 미만인 문항이 262 문항으로 0.3 이상인 문항이 32 문항인 것에 비해 더 많이 나타남
- 변별도 2 지수를 기준으로 분류하였을 때, 0.3 미만인 문항이 240 문항으로 0.3 이상인 문항이 54 문항인 것에 비해 더 많이 나타남

(2) 자료제시형 난이도와 변별도 분포도 및 비율분석

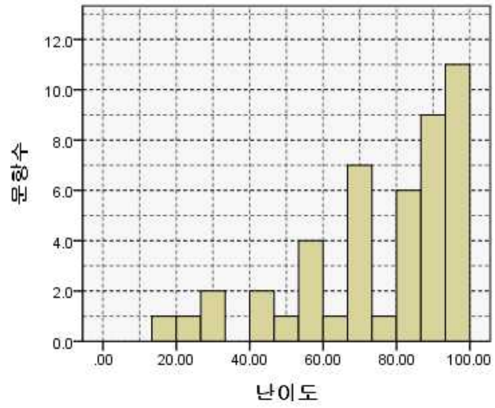

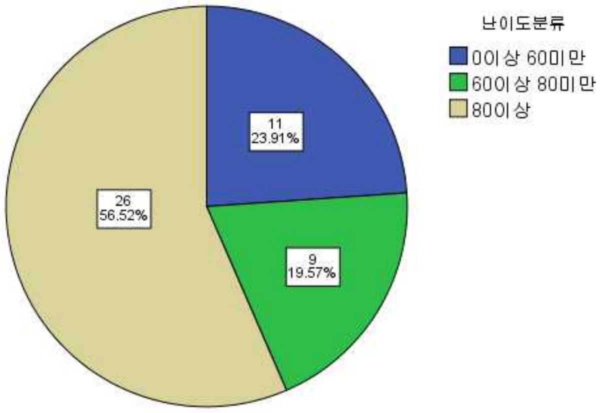

총점	난이도	표준편차
46	75.5	22.3

난이도	문항수	비율(%)
0~60미만	11	23.9
60~80미만	9	19.6
80~100	26	56.5
전체	46	100.0

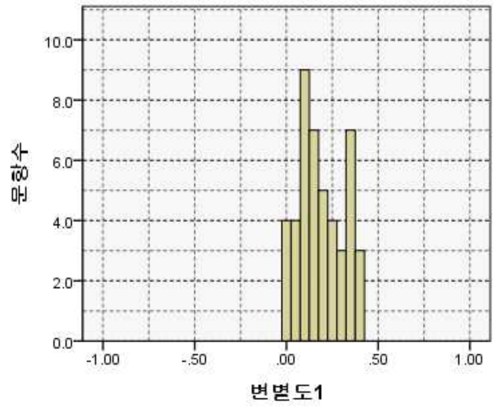

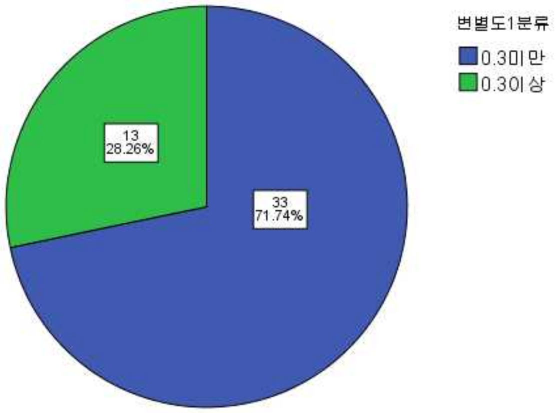

총점	변별도1	표준편차
46	.19	.12

변별도1	문항수	비율(%)
0.3미만	33	71.7
0.3이상	13	28.3
전체	46	100.0

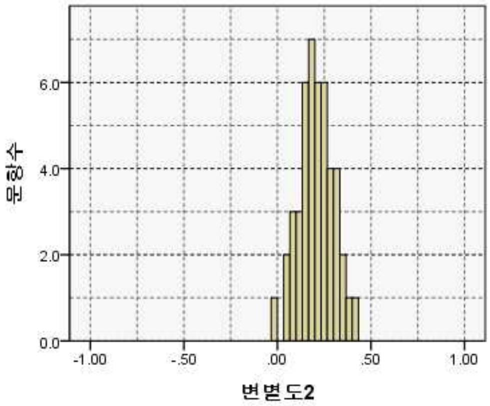

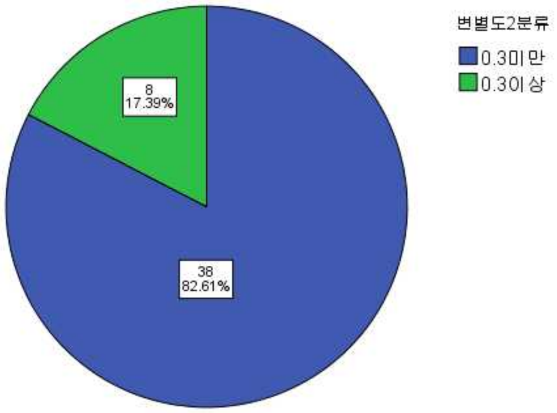

총점	변별도2	표준편차
46	.21	.09

변별도2	문항수	비율(%)
0.3미만	38	82.6
0.3이상	8	17.4
전체	46	100.0

해석

- 자료제시형 문항에서 난이도 지수가 80 에서 100 사이인 문항이 전체 46 문항 중 26 문항으로 가장 많았으며, 60 이상 80 미만인 문항이 9 문항, 60 미만인 문항이 11 문항인 것으로 나타남
- 변별도 1 지수를 기준으로 분류하였을 때, 0.3 미만인 문항이 33 문항으로 0.3 이상인 문항이 13 문항인 것에 비해 더 많이 나타남
- 변별도 2 지수를 기준으로 분류하였을 때, 0.3 미만인 문항이 38 문항으로 0.3 이상인 문항이 8 문항인 것에 비해 더 많이 나타남

3. 난이도와 변별도 간 산포도

1) 전체 난이도와 변별도 간 산포도

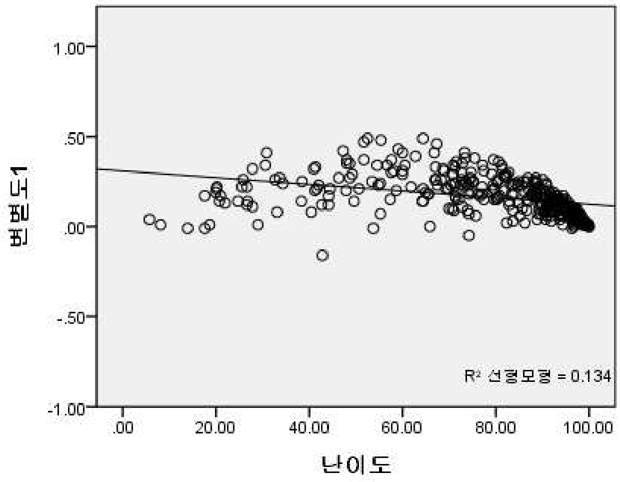

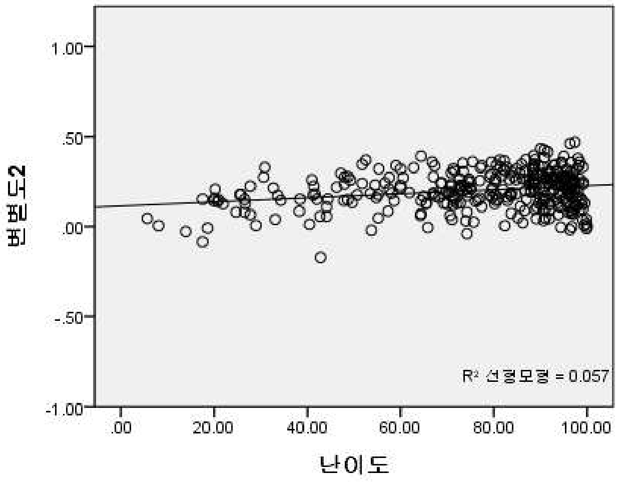

해석

- 난이도 지수와 변별도1 지수 간 상관은 -.366*로 난이도 지수가 높을수록 변별력이 낮아지는 것으로 나타남
- 난이도 지수와 변별도2 지수 간 상관은 .238*로 난이도 지수가 높을수록 변별력이 높아지는 것으로 나타남

2) 과목별 난이도와 변별도 간 산포도

가) 내과학 난이도와 변별도 간 산포도

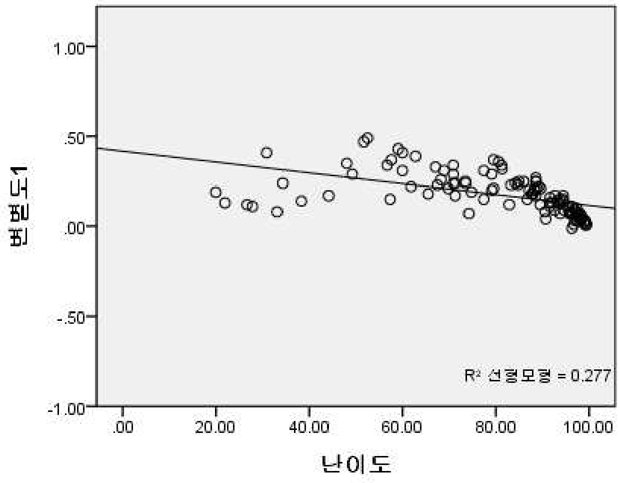

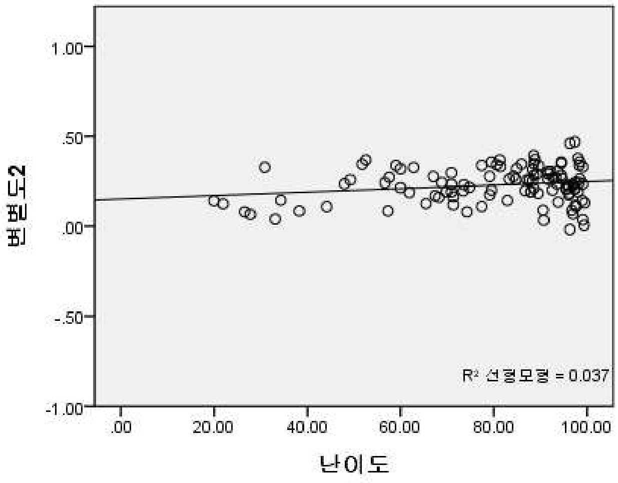

해석

- 난이도 지수와 변별도 1 지수 간 상관은 -.526*로 난이도 지수가 높을수록 변별력이 낮아지는 것으로 나타남
- 난이도 지수와 변별도 2 지수 간 상관은 .193*로 난이도 지수가 높을수록 변별력이 높아지는 것으로 나타남

나) 침구학 난이도와 변별도 간 산포도

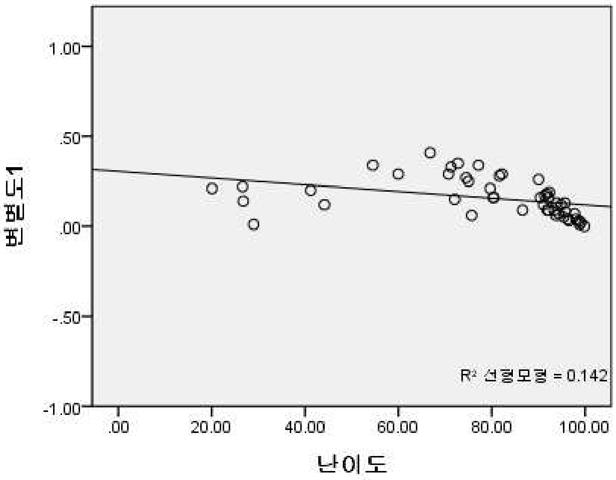

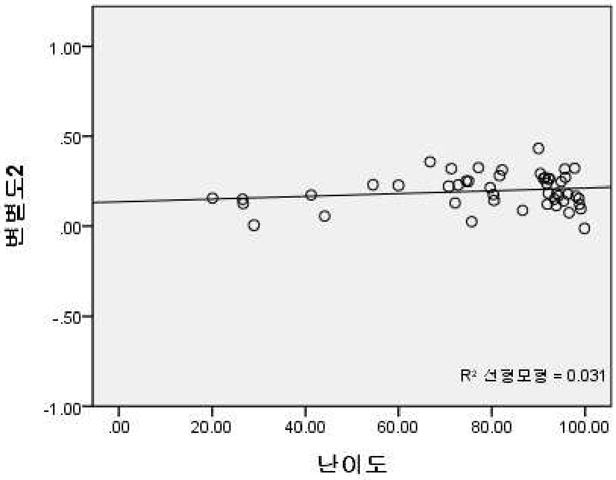

해석

- 난이도 지수와 변별도 1 지수 간 상관은 -.377*로 난이도 지수가 높을수록 변별력이 낮아지는 것으로 나타남
- 난이도 지수와 변별도 2 지수 간 상관은 .176 로 관련성이 없는 것으로 나타남

다) 보건의약관계법규 난이도와 변별도 간 산포도

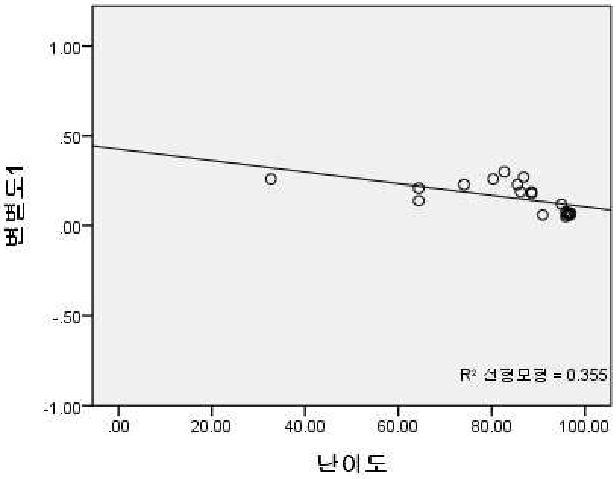

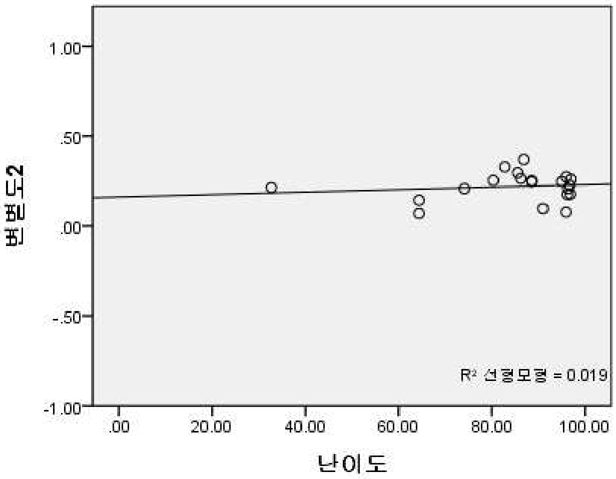

해석
- 난이도 지수와 변별도 1 지수 간 상관은 -.596*으로 난이도 지수가 높을수록 변별력이 낮아지는 것으로 나타남<br>- 난이도 지수와 변별도 2 지수 간 상관은 .138 로 관련성이 없는 것으로 나타남

라) 외과학 난이도와 변별도 간 산포도

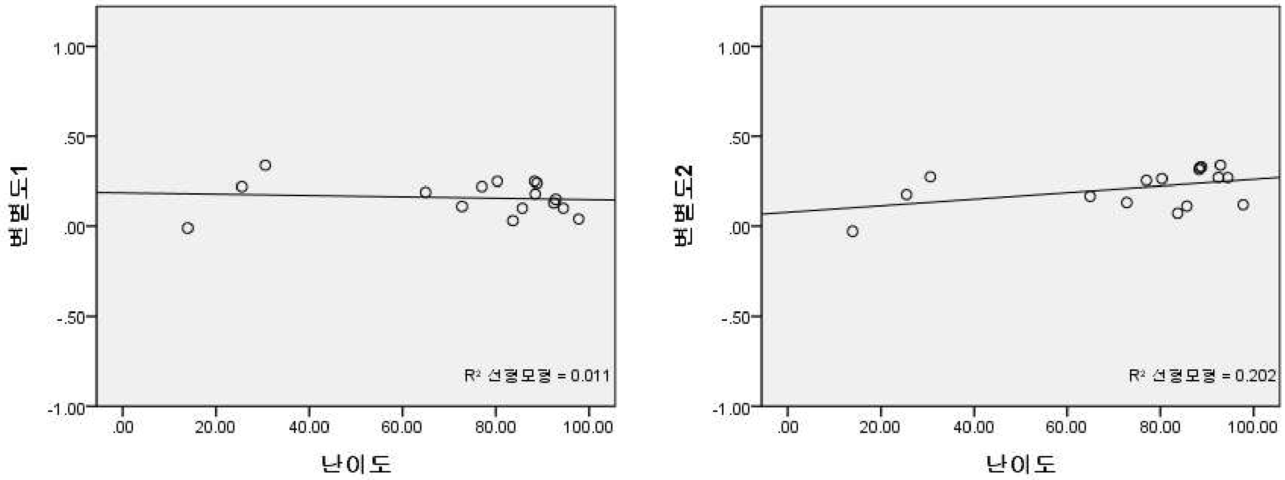

해석
- 난이도 지수와 변별도 1 지수 간 상관은 -.106 으로 관련성이 없는 것으로 나타남<br>- 난이도 지수와 변별도 2 지수 간 상관은 .449 로 관련성이 낮은 것으로 나타남

마) 신경정신과학 난이도와 변별도 간 산포도

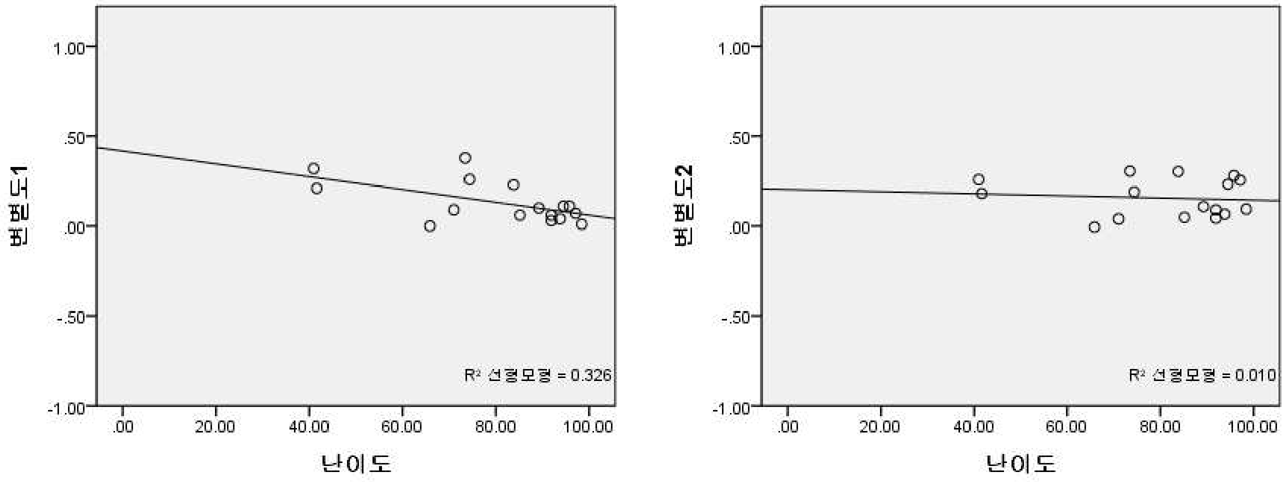

해석

- 난이도 지수와 변별도1 지수 간 상관은 -.571*로 난이도 지수가 높을수록 변별력이 낮아지는 것으로 나타남
- 난이도 지수와 변별도2 지수 간 상관은 .101로 관련성이 없는 것으로 나타남

바) 안이비인후과학 난이도와 변별도 간 산포도

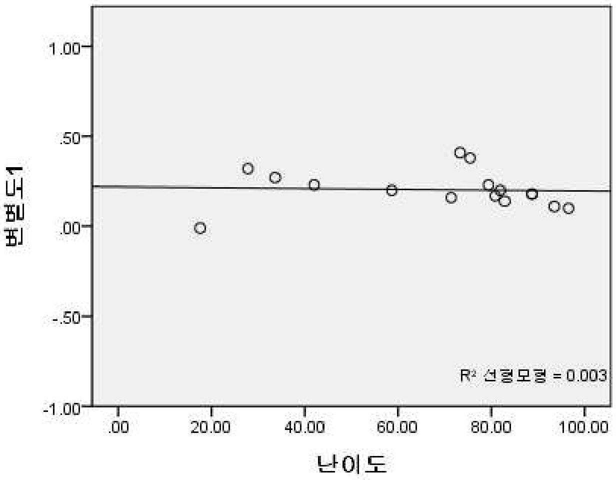

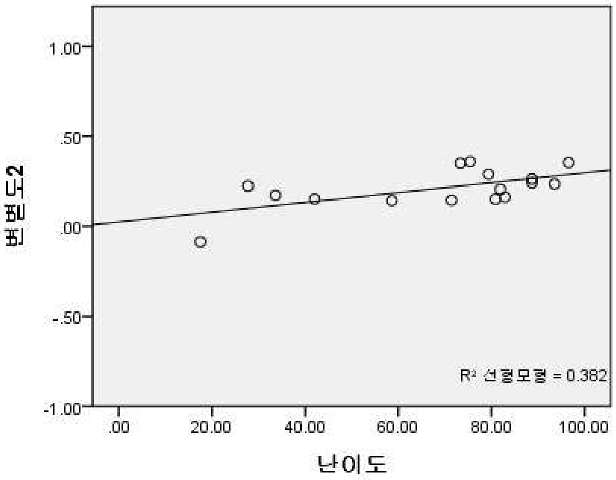

해석

- 난이도 지수와 변별도1 지수 간 상관은 -.052로 관련성이 없는 것으로 나타남
- 난이도 지수와 변별도2 지수 간 상관은 .618*로 난이도 지수가 높을수록 변별력이 높아지는 것으로 나타남

사) 부인과학 난이도와 변별도 간 산포도

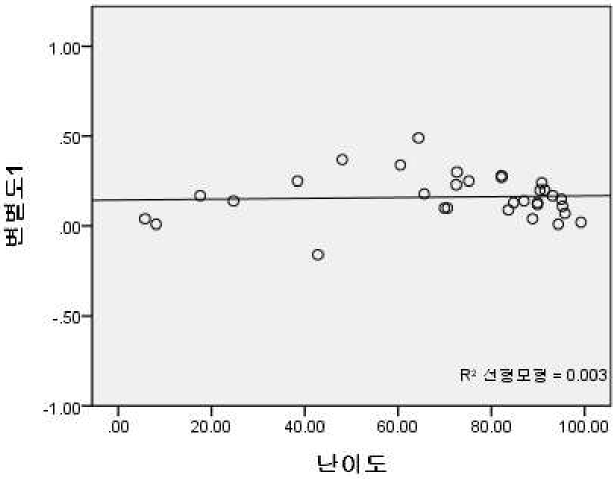

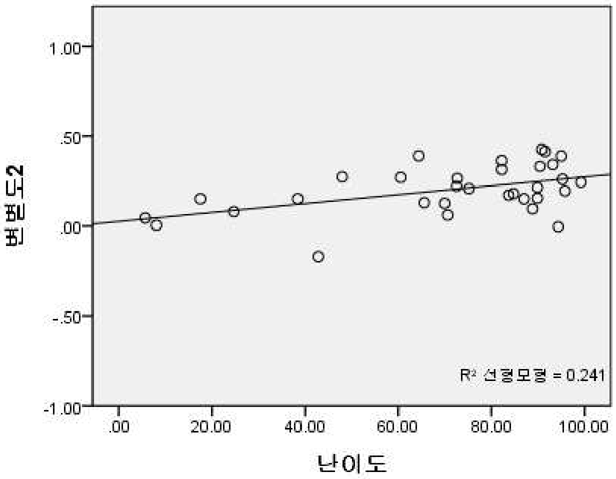

해석

- 난이도 지수와 변별도 1 지수 간 상관은 -.051 로 관련성이 없는 것으로 나타남
- 난이도 지수와 변별도 2 지수 간 상관은 .490*으로 난이도 지수가 높을수록 변별력이 높아지는 것으로 나타남

아) 소아과학 난이도와 변별도 간 산포도

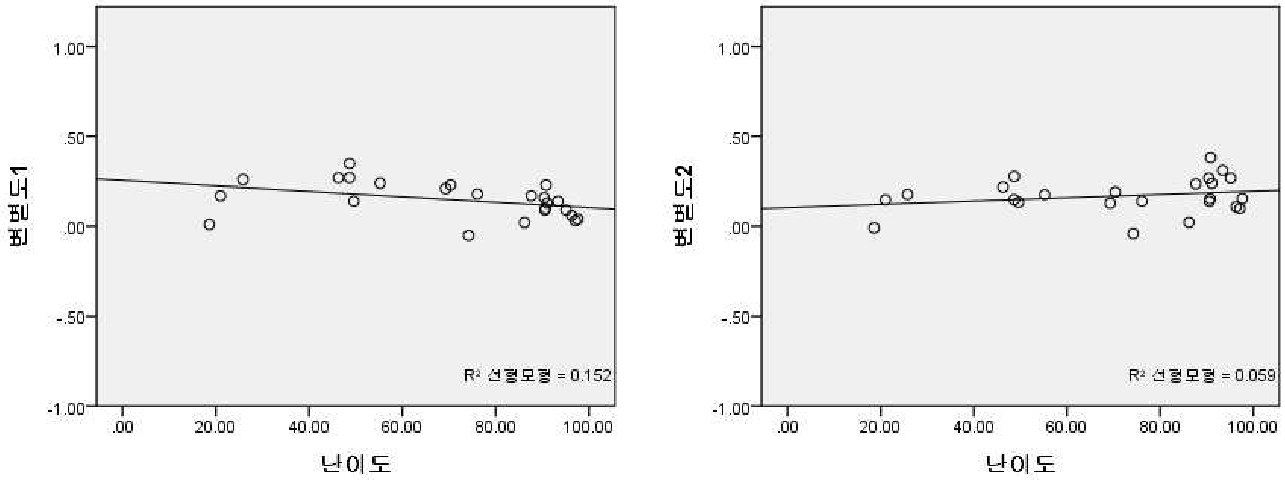

해석

- 난이도 지수와 변별도 1 지수 간 상관은 -.390 으로 관련성이 낮은 것으로 나타남
- 난이도 지수와 변별도 2 지수 간 상관은 .242 로 관련성이 없는 것으로 나타남

자) 예방의학 난이도와 변별도 간 산포도

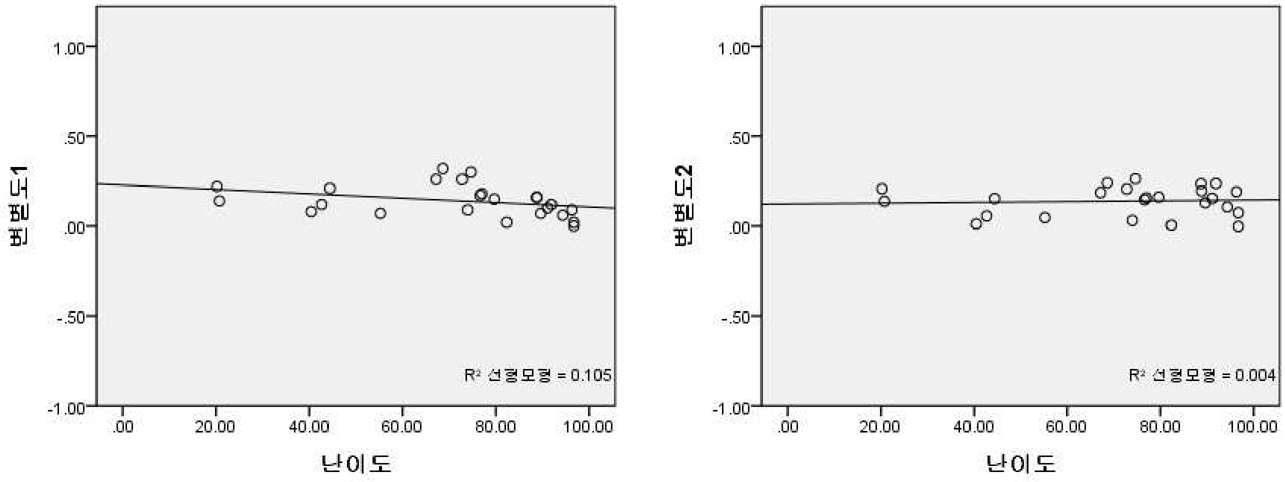

해석
- 난이도 지수와 변별도 1 지수 간 상관은 -.324 로 관련성이 낮은 것으로 나타남<br>- 난이도 지수와 변별도 2 지수 간 상관은 .063 으로 관련성이 없는 것으로 나타남

차) 한방생리학 난이도와 변별도 간 산포도

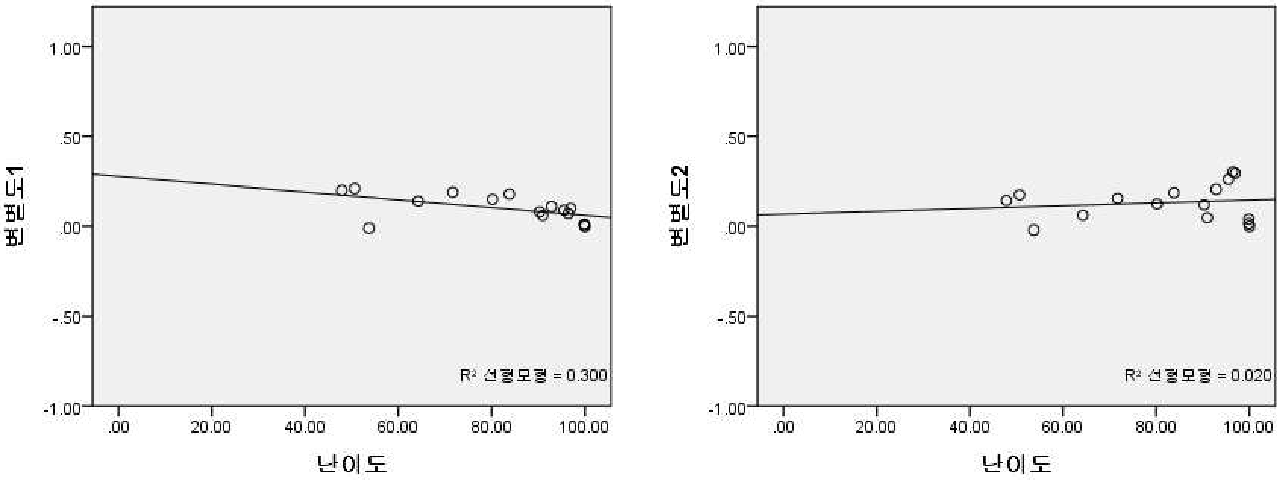

해석
- 난이도 지수와 변별도 1 지수 간 상관은 -.548*으로 난이도 지수가 높을수록 변별력이 낮아지는 것으로 나타남<br>- 난이도 지수와 변별도 2 지수 간 상관은 .143 으로 관련성이 없는 것으로 나타남

카) 본초학 난이도와 변별도 간 산포도

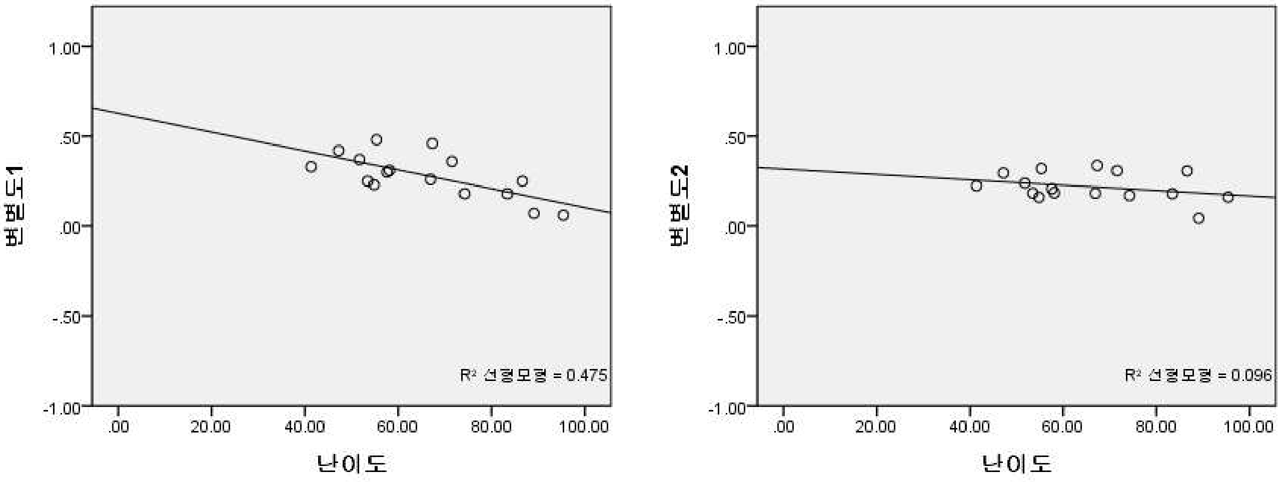

해석

- 난이도 지수와 변별도1 지수 간 상관은 -.689*으로 난이도 지수가 높을수록 변별력이 낮아지는 것으로 나타남
- 난이도 지수와 변별도2 지수 간 상관은 -.309로 관련성이 낮은 것으로 나타남

4. 신뢰도 분석

과목명	문항수	제73회	제74회	제75회	제76회	제77회
전체	340	.951	.941	.928	.927	.931
내과학	112	.892	.859	.834	.854	.858
침구학	48	.783	.714	.655	.674	.683
보건의약관계법규	20	.503	.470	.496	.463	.567
외과학	16	.616	.454	.391	.370	.478
신경정신과학	16	.435	.471	.379	.456	.302
안이비인후과학	16	.489	.472	.435	.365	.419
부인과학	32	.583	.610	.598	.557	.577
소아과학	24	.529	.576	.507	.497	.427
예방의학	24	.537	.513	.264	.461	.392
한방생리학	16	.379	.473	.357	.241	.198
본초학	16	.625	.654	.639	.560	.656

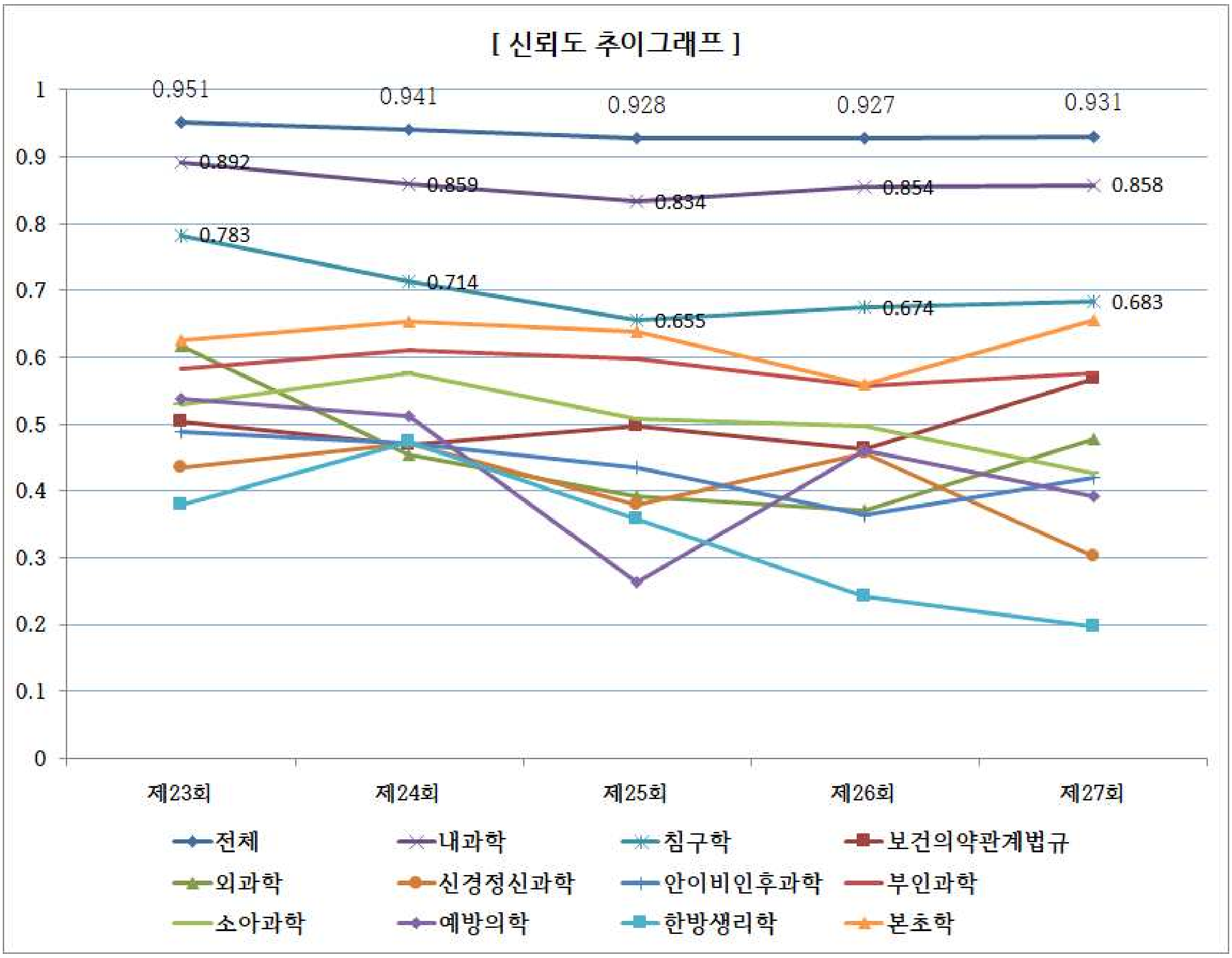

해석

- 전회 대비 전체문항의 신뢰도는 .004 증가함
- 전회 대비 내과학 과목 문항의 신뢰도는 .004 증가함
- 전회 대비 침구학 과목 문항의 신뢰도는 .009 증가함
- 전회 대비 보건의약관계법규 과목 문항의 신뢰도는 .104 증가함
- 전회 대비 외과학 과목 문항의 신뢰도는 .108 증가함
- 전회 대비 신경정신과학 과목 문항의 신뢰도는 .154 감소함
- 전회 대비 안이비인후과학 과목 문항의 신뢰도는 .054 감소함
- 전회 대비 부인과학 과목 문항의 신뢰도는 .020 증가함
- 전회 대비 소아과학 과목문항의 신뢰도는 .070 감소함
- 전회 대비 예방의학 과목 문항의 신뢰도는 .069 감소함
- 전회 대비 한방생리학 과목 문항의 신뢰도는 .043 감소함
- 전회 대비 본초학 과목 문항의 신뢰도는 .096 증가함

○ 분석결과 관련 문의 : 한국보건의료인국가시험원 연구개발본부 김준기 전임연구원
Tel : 02-2087-8956, FAX : 02-2087-8885
E-mail : tontates@kuksiwon.or.kr
