# Supplementary material for: Item difficulty index, discrimination index, and reliability of the 26 health professions licensing examinations in 2022, Korea: a psychometric study
Source: J Educ Eval Health Prof. 2023 Nov 22;20:31. doi: 10.3352/jeehp.2023.20.31 (PMC11959405; doi:10.3352/jeehp.2023.20.31)
Supplement: Supplementary file 1 — Supplement 1. Item analysis results of 26 health professions licensing examinations administered during late 2022 and early 2023. [file jeehp-20-31_Suppl1.zip › 2022│Γ╡╡ ┴a86╚╕ └╟╗τ ▒╣░í╜├╟Φ(╟╩▒Γ) ║╨╝«░ß░·.pdf]

2022년도 제86회
의사 국가시험(필기)
문항분석 결과

국민이 신뢰하고 감동하는 시험평가기관
한국보건의료인국가시험원
KOREA HEALTH PERSONNEL LICENSING EXAMINATION INSTITUTE

일반 용어 정의

□ **평균**

- 집단에서의 대표적 경향값으로 전체 값을 더하여 총 응시자로 나눈 값

□ **표준편차**

- 평균과 각 점수의 차이인 편차들의 평균으로 점수가 흩어져 분포되어 있는 정도

□ **검사이론**

- 검사와 검사를 구성하고 있는 문항의 양호도를 분석 및 평가하는 방법을 정의한 이론체계
- 대표적으로 고전검사이론과 문항반응이론이 있음

고전검사이론 용어 정의

□ **고전검사이론(Classical Test Theory; CTT)**

- 검사의 질을 분석하는 검사이론 중 한 가지로 19세기 말부터 전개되어 현재까지 주로 사용되고 있는 검사이론임
- 고전검사이론에 의한 문항과 응시자 능력 추정치는 다음과 같음

○ **문항난이도**

· 검사 문항의 쉽고 어려운 정도를 나타내는 지수
· 난이도 지수는 총 반응 수에 대한 정답 반응 수의 비율로 문항의 정답률임
· 문항난이도는 0~100까지의 값을 가짐
· 난이도 값이 큰 경우, 쉬운 문항으로 '난이도가 낮다'라고 해석하며, 난이도 값이 작은 경우, 어려운 문항으로 '난이도가 높다'라고 해석함

○ **문항변별도**

· 각 문항이 응시자의 능력 수준을 변별할 수 있는 정도를 나타내는 지수
· 문항변별도는 -1~+1까지의 값을 가지며, 1에 가까울수록 변별력 크다고 해석함
· 일반적으로 문항변별도가 0.3이상이면 우수한 문항으로 평가함
· 구하는 방식에는 '상하위집단 구분법', '문항-총점 상관계수' 등이 있음
  1) **변별도 1(상하위구분법):** 상위 27%와 하위 27% 집단의 난이도 차이를 구하는 방식
  2) **변별도 2(상관계수법):** 문항-총점과의 상관계수로 구하는 방식

○ **신뢰도**

· 시험이 평가하고자 하는 것을 일관성 있게 측정하는가로 시험이 오차없이 정확하게 측정한 정도를 의미함
· 국시원에서는 문항의 내적일관성(Cronbach α)으로 신뢰도를 추정하며 1에 가까울수록 신뢰도가 높다고 해석함

목 차

I. 시행 결과 ······ 5

1. 시험 현황 ······ 6

1) 시험명 ······ 6

2) 시험시행일 ······ 6

3) 응시현황 ······ 6

4) 과목별 문항 수, 배점 및 과락 점수 ······ 6

5) 교시별 문항 수 및 배점 ······ 6

2. 합격률과 평균성적 ······ 7

1) 합격 및 불합격 현황 ······ 7

2) 과목별 과락자수 내역 ······ 7

3) 전회 대비 합격률과 평균성적 ······ 8

II. 문항분석 결과 ······ 9

1. 성적 ······ 10

1) 전체 성적분포도 ······ 10

2) 과목별 성적분포도 ······ 11

2. 난이도와 변별도 ······ 12

1) 전체 난이도와 변별도 ······ 12

2) 과목별 난이도와 변별도 ······ 15

3) 지식수준별 난이도와 변별도 ······ 24

4) 자료유형별 난이도와 변별도 ······ 33

5) 문항형태별 난이도와 변별도 ······ 39

3. 난이도와 변별도 간 산포도 ······ 45

1) 전체 난이도와 변별도 간 산포도 ······ 45

2) 과목별 난이도와 변별도 간 산포도 ······ 45

4. 신뢰도 분석 ······ 48

I. 시행 결과

1. 시험 현황

1) 시험명: 2022년도 제86회 의사 국가시험(필기)

2) 시험시행일: 2022년 1월 6일 ~ 1월 7일

3) 응시현황

구분	응시대상자수	결시자수	부정행위자수	응시자 준수사항 위반자 수		응시자수 (%)
				휴대폰 소지	신분증 미지참	
실기시험 (상반기)	2,709	0	0	0	0	2,709 (100.0)
실기시험 (하반기)	3,377	12	0	0	0	3,365 (99.6)
**필기시험**	**※3,322**	**17**	**0**	**0**	**0**	**3,305 (99.4)**
종합	6,050	7	0	0	0	6,043 (99.9)

※ 응시결격자(졸업탈락) 2명 제외, 제85회 필기 면제 포기자 22명 포함

4) 과목별 문항 수, 배점 및 과락 점수

교 시	과 목 명	문제 수	배점	총점	합격자 점수기준	
					과목 과락기준	총점 합격기준
1교시	의학총론	60	1	60	24점 미만	192점 이상
2-5교시	의학각론	240	1	240	96점 미만	
1교시	보건의약관계법규	20	1	20	8점 미만	
계		320		320		

5) 교시별 문항 수 및 배점

교 시	과 목 명	문제 수	배점	총점
1교시	보건의약관계법규	20	1	20
	의학총론	60	1	60
2교시	의학각론1	80	1	80
3교시	의학각론2	80	1	80
4교시	의학각론3	48	1	48
	의학각론4(R형)	32	1	32
계		320		320

2. 합격률과 평균성적

1) 합격 및 불합격 현황

구분	합격자수 (%)	불합격자수(%)				채점보류자수
		평락	과락	기권	계	
실기시험 (상반기)	2,643 (97.6)				66 (2.4)	0 (0.0)
실기시험 (하반기)	3,228 (95.9)				137 (4.1)	0 (0.0)
필기시험	3,191 (96.6)	111 (3.4)	3 (0.1)	0 (0.0)	114 (3.4)	0 (0.0)
종합	5,786 (95.7)				257 (4.3)	0 (0.0)

2) 과목별 과락자수 내역

과목명 / 과락자수	의학총론	의학각론	보건의약관계법규
과목별 과락자 수	0	0	3
전과목 과락자 수	0		

3) 전회 대비 합격률과 평균성적

회차	년도	합격률(%)※	평균성적	표준편차	백분율 환산점수
제82회	2018	95.0	285.1	23.7	79.2
제83회	2019	94.2	276.5	25.6	76.8
제84회	2020	94.2	270.8	28.0	75.2
제85회	2021	12.8	285.1	26.8	79.2
**제86회**	**2022**	**95.7**	**249.9**	**27.6**	**78.1**

※ 필기시험과 실기시험을 종합한 합격률임

※ 제85회까지는 360점 만점, 제86회부터 320점 만점임

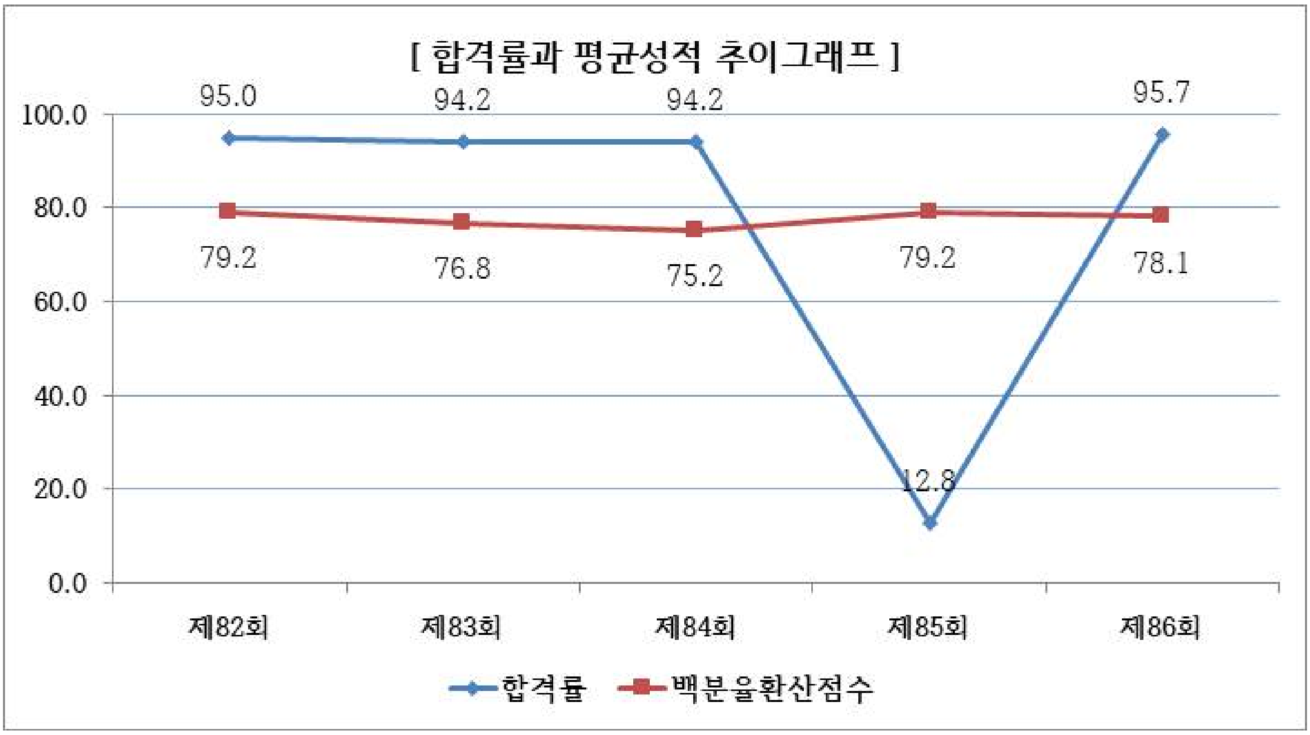

해석

- 전년 대비 합격률은 82.9 증가하였으며, 백분율 환산점수는 1.1 점 감소함
- 표준편차는 0.8 점 증가함

II. 문항분석 결과

1. 성적

1) 전체 성적분포도

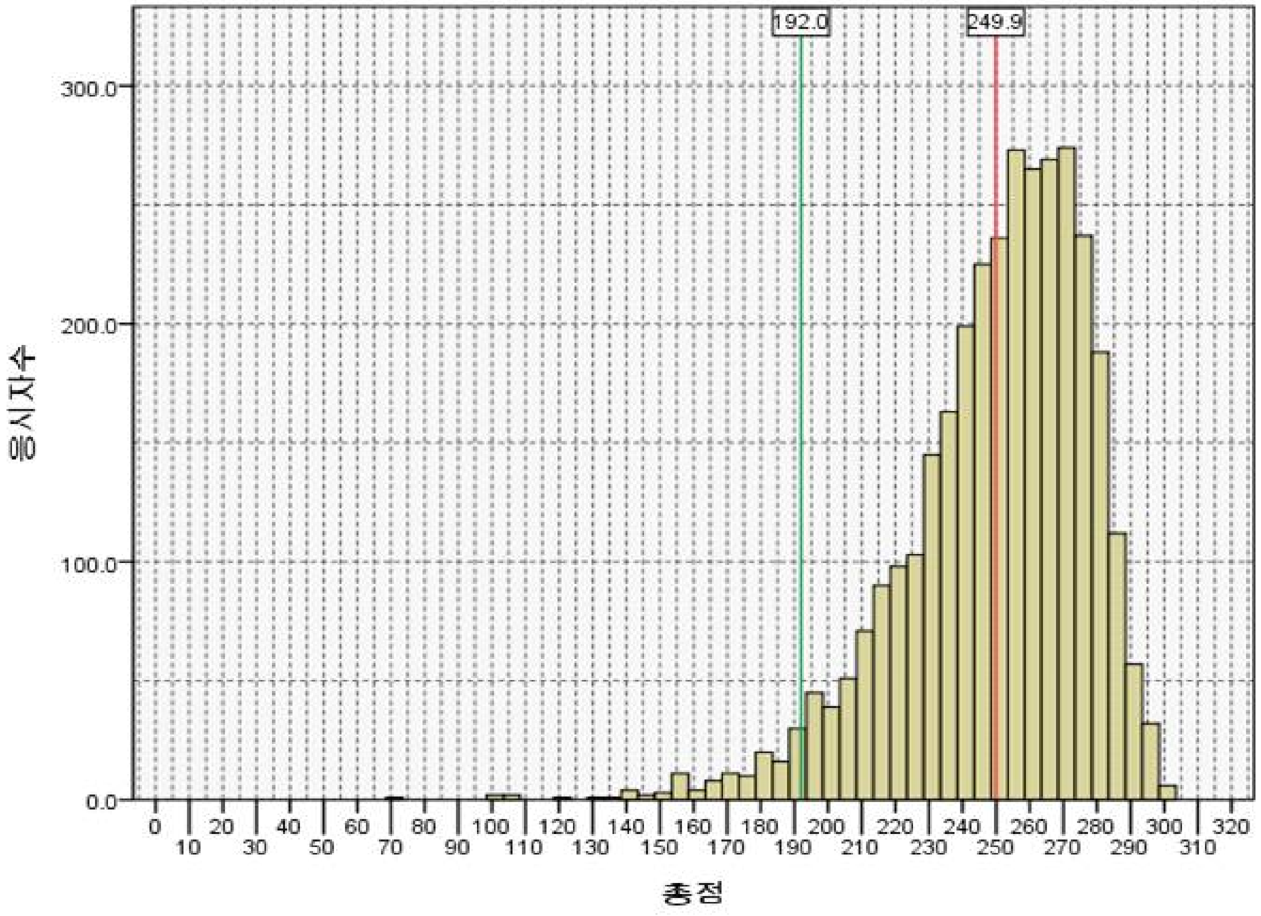

응시자	총점	합격선	평균성적	표준편차
3,305	320	192	249.9	27.6

2) 과목별 성적분포도

가) 의학총론

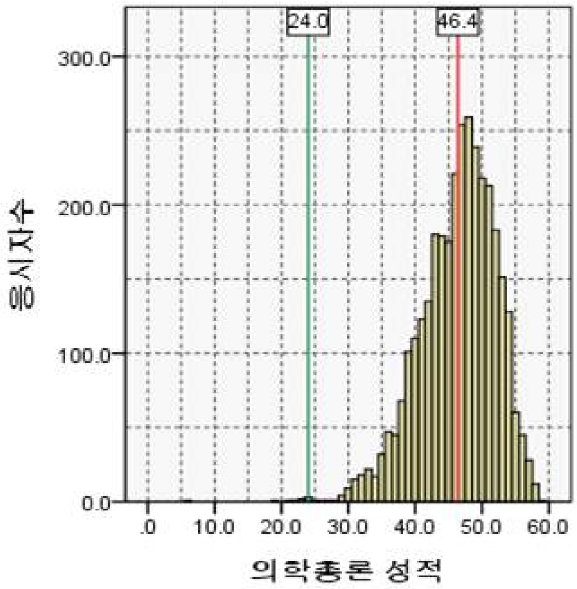

총점	과락선	평균성적	표준편차
60	24	46.4	5.7

나) 의학각론

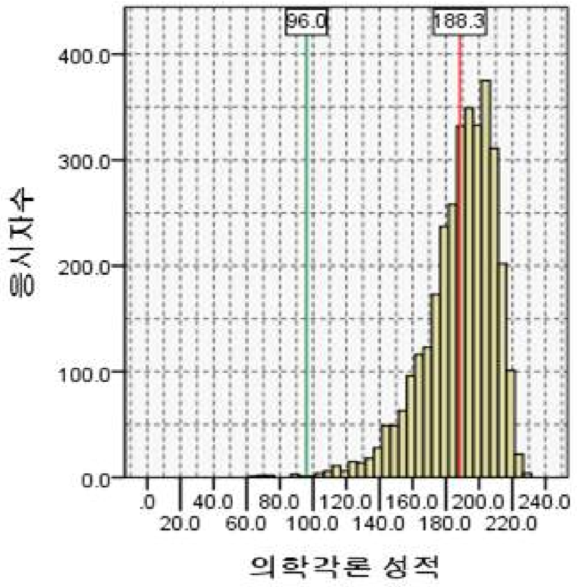

총점	과락선	평균성적	표준편차
240	96	188.3	21.6

다) 보건의약관계법규

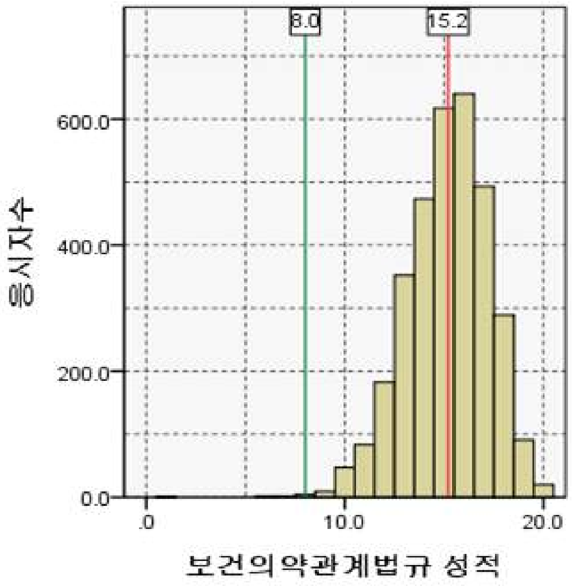

총점	과락선	평균성적	표준편차
20	8	15.2	2.1

2. 난이도와 변별도

1) 전체 난이도와 변별도

가) 전회 대비 전체 난이도와 변별도

회차	난이도		변별도1		변별도2	
	평균	표준편차	평균	표준편차	평균	표준편차
제82회	79.2	20.6	.15	.11	.18	.09
제83회	76.8	21.8	.16	.11	.19	.09
제84회	75.2	21.6	.18	.13	.20	.10
제85회	79.2	20.5	.17	.13	.21	.11
**제86회**	**78.1**	**20.6**	**.20**	**.14**	**.24**	**.11**

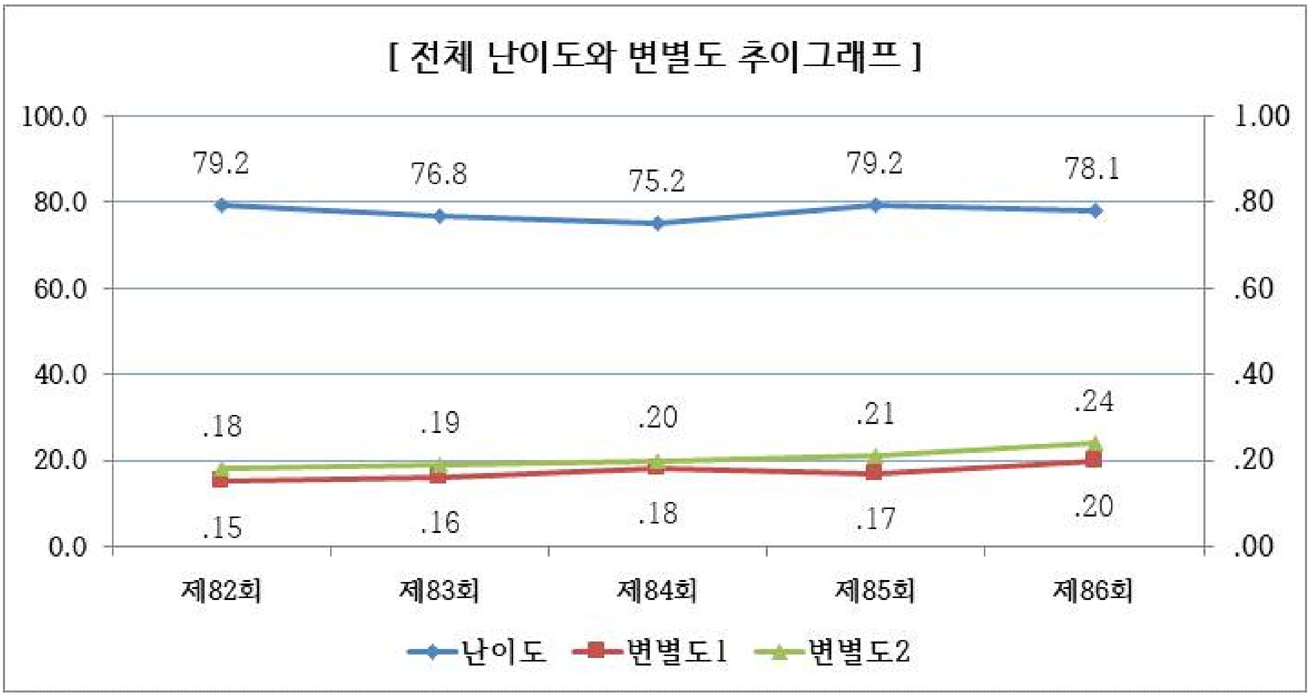

해석

- 전년 대비 난이도 지수는 1.1 감소함
- 변별도 1 지수와 변별도 2 지수는 각각 .03 증가함

나) 전체 난이도와 변별도 분포도 및 비율분석

(1) 전체 난이도 분포도 및 비율분석

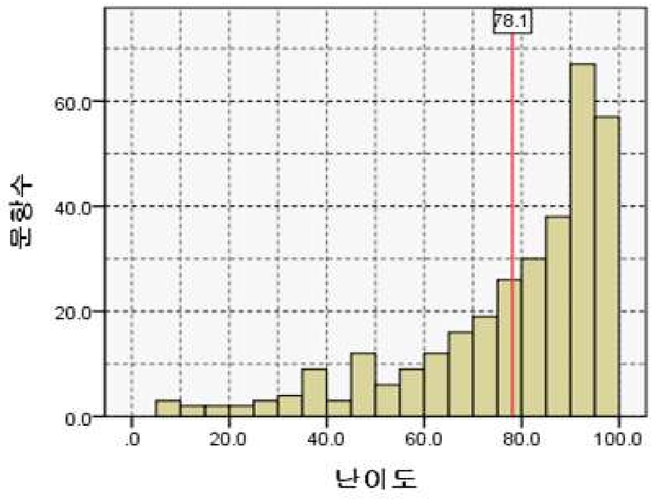

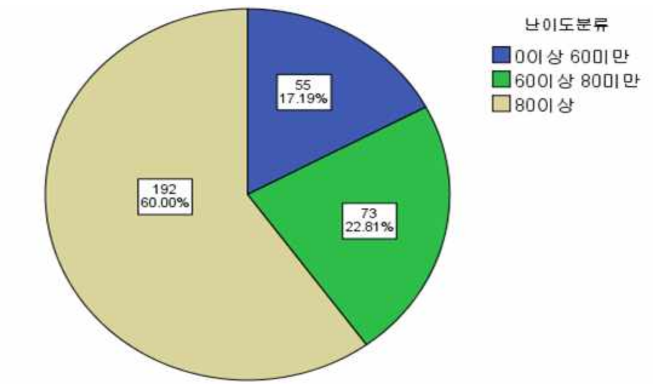

총점	난이도	표준편차
320	78.1	20.6

난이도	문항수	비율(%)
0~60미만	55	17.2
60~80미만	73	22.8
80~100	192	60.0
전체	320	100.0

(2) 전체 변별도1 분포도 및 비율분석

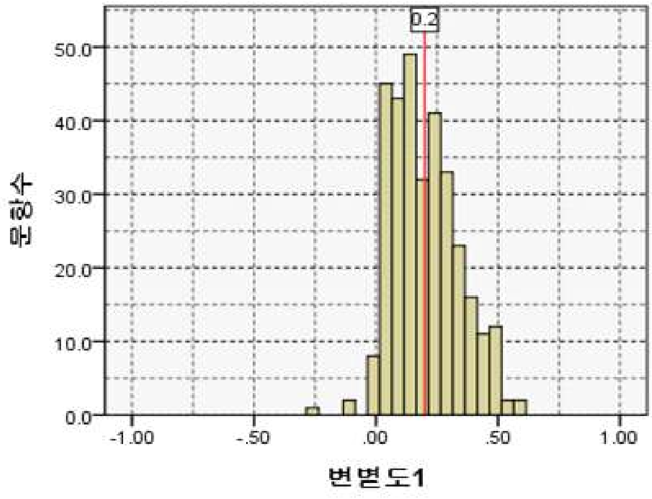

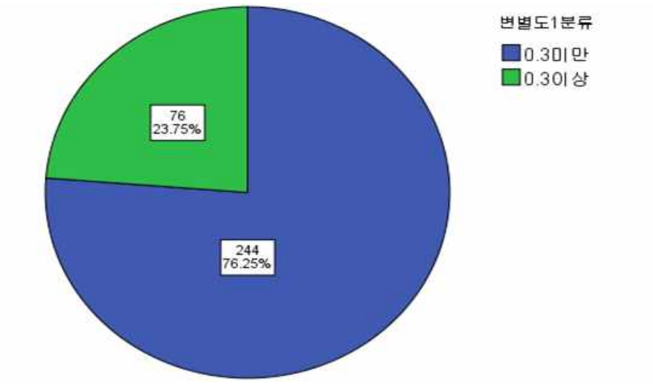

총점	변별도1	표준편차
320	.20	.14

변별도1	문항수	비율(%)
0.3미만	244	76.3
0.3이상	76	23.8
전체	320	100.0

(3) 전체 변별도2 분포도 및 비율분석

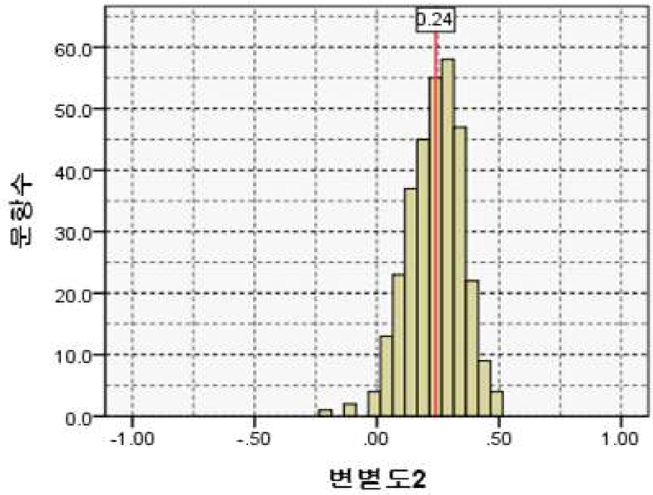

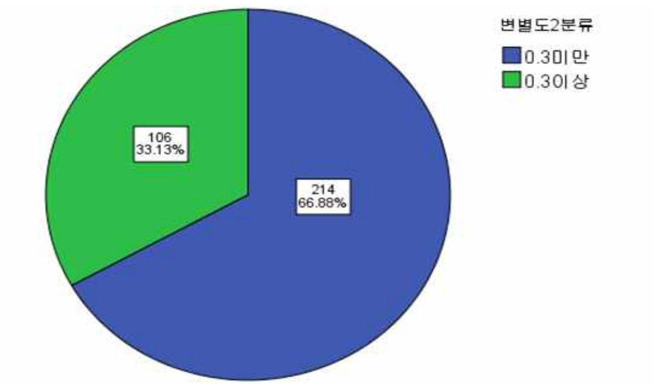

총점	변별도2	표준편차	변별도2	문항수	비율(%)
320	.24	.11	0.3미만	214	66.9
			0.3이상	106	33.1
			전체	320	100.0

해석

- 난이도 지수가 80 에서 100 사이인 문항이 전체 320 문항 중 192 문항으로 가장 많았으며, 차례로 60 이상 80 미만인 문항이 73 문항, 0 에서 60 미만인 문항이 55 문항인 것으로 나타남
- 변별도 1 지수를 기준으로 분류하였을 때, 0.3 미만인 문항이 244 문항으로 0.3 이상인 문항이 76 문항인 것에 비해 더 많이 나타남
- 변별도 2 지수를 기준으로 분류하였을 때, 0.3 미만인 문항이 214 문항으로 0.3 이상인 문항이 106 문항인 것에 비해 더 많이 나타남

2) 과목별 난이도와 변별도

가) 전회 대비 과목별 난이도와 변별도

(1) 전회 대비 의학총론 난이도와 변별도

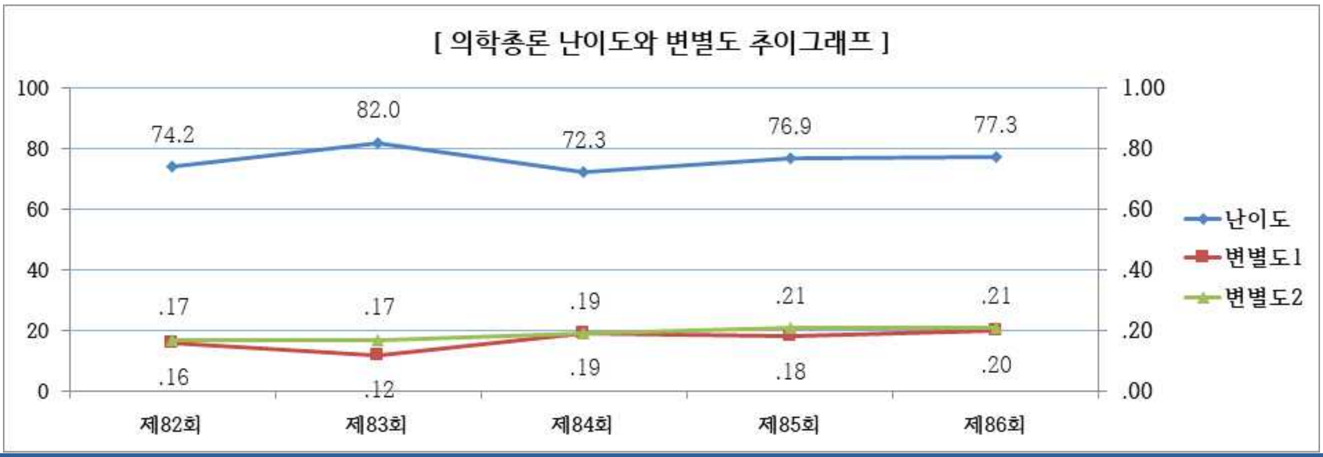

회차	난이도		변별도1		변별도2	
	평균	표준편차	평균	표준편차	평균	표준편차
제82회	74.2	20.8	.16	.11	.17	.09
제83회	82.0	20.1	.12	.10	.17	.09
제84회	72.3	22.4	.19	.13	.19	.11
제85회	76.9	21.6	.18	.16	.21	.11
**제86회**	**77.3**	**18.8**	**.20**	**.12**	**.21**	**.10**

해석

- 전회 대비 의학총론 과목의 난이도 지수는 0.4 증가함
- 전회 대비 의학총론 과목의 변별도 1 지수는 0.02 증가함
- 전회 대비 의학총론 과목의 변별도 2 지수는 동일함

(2) 전회 대비 의학각론 난이도와 변별도

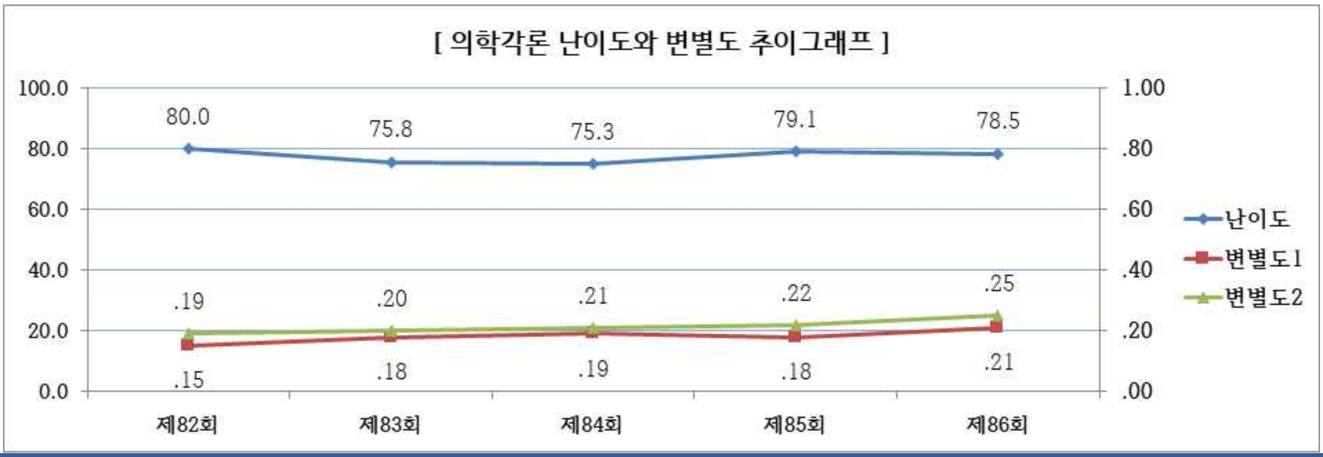

회차	난이도		변별도1		변별도2	
	평균	표준편차	평균	표준편차	평균	표준편차
제82회	80.0	20.3	.15	.11	.19	.09
제83회	75.8	21.7	.18	.11	.20	.09
제84회	75.3	21.8	.19	.13	.21	.10
제85회	79.1	20.4	.18	.13	.22	.11
**제86회**	**78.5**	**20.8**	**.21**	**.14**	**.25**	**.11**

해석

- 전회 대비 의학각론 과목의 난이도 지수는 0.6 감소함
- 전회 대비 의학각론 과목의 변별도 1 지수는 0.03 증가함
- 전회 대비 의학각론 과목의 변별도 2 지수는 0.03 증가함

(3) 전회 대비 보건의약관계법규 난이도와 변별도

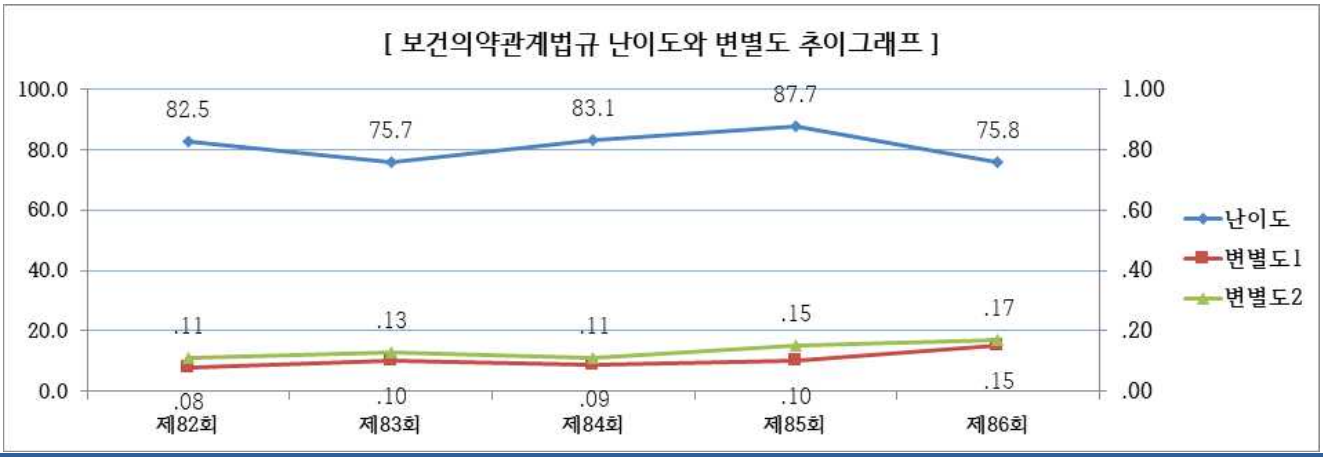

회차	난이도		변별도1		변별도2	
	평균	표준편차	평균	표준편차	평균	표준편차
제82회	82.5	22.8	.08	.10	.11	.08
제83회	75.7	27.3	.10	.09	.13	.08
제84회	83.1	15.7	.09	.09	.11	.08
제85회	87.7	15.0	.10	.09	.15	.09
**제86회**	**75.8**	**24.5**	**.15**	**.15**	**.17**	**.12**

해석

- 전회 대비 보건의약관계법규 과목의 난이도 지수는 11.9 감소함
- 전회 대비 보건의약관계법규 과목의 변별도 1 지수는 0.05 증가함
- 전회 대비 보건의약관계법규 과목의 변별도 2 지수는 0.02 증가함

나) 과목별 난이도와 변별도 분포도 및 비율분석

(1) 의학총론 난이도와 변별도 분포도 및 비율분석

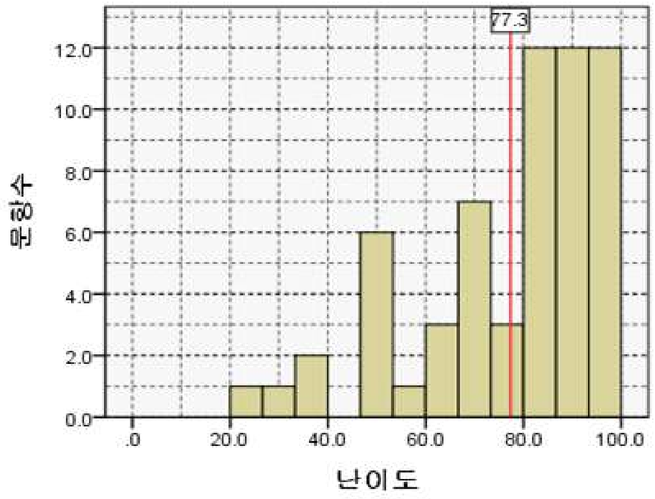

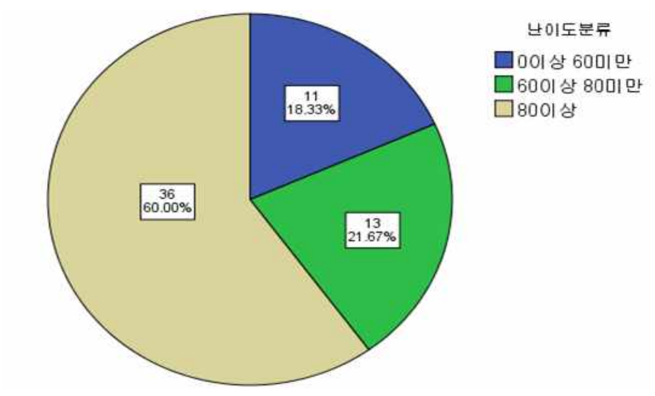

총점	난이도	표준편차	난이도	문항수	비율(%)
60	77.3	18.8	0~60미만	11	18.3
			60~80미만	13	21.7
			80~100	36	60.0
			전체	60	100.0

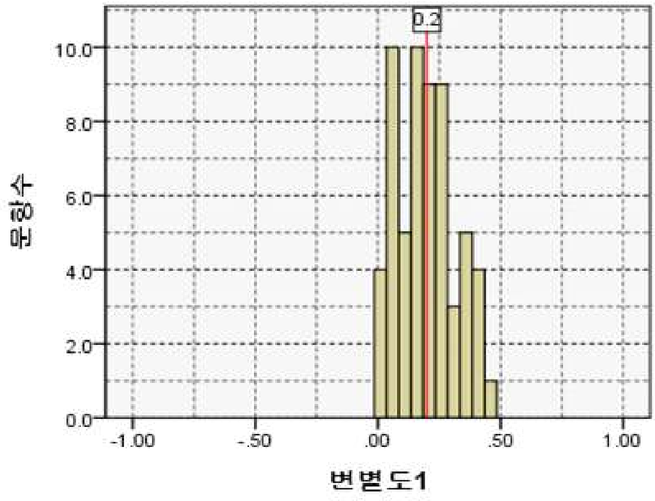

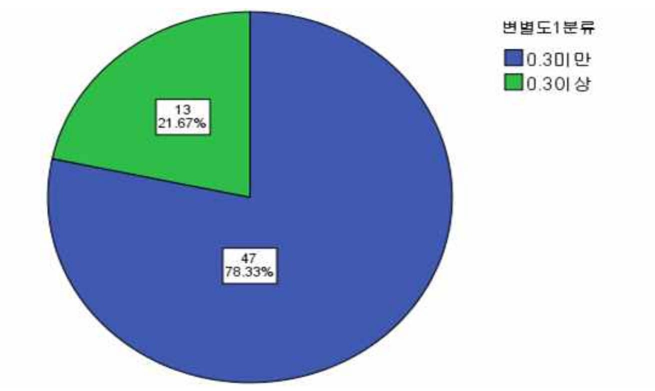

총점	변별도1	표준편차	변별도1	문항수	비율(%)
60	.20	.12	0.3미만	47	78.3
			0.3이상	13	21.7
			전체	60	100.0

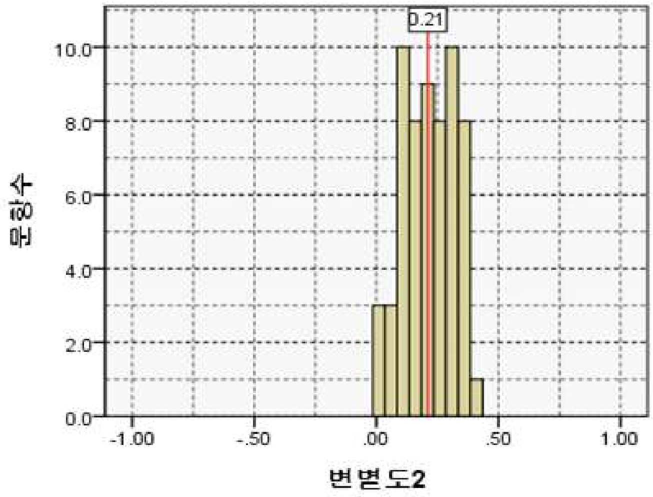

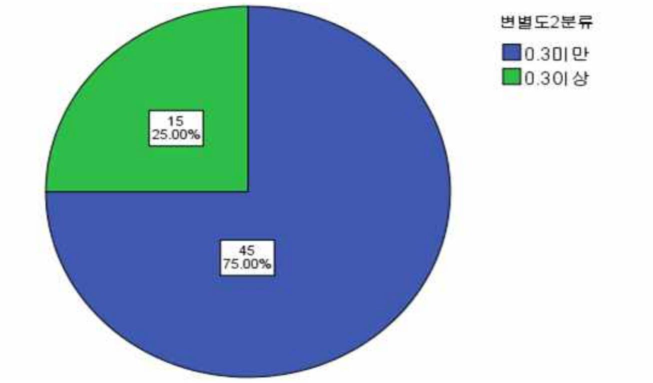

총점	변별도2	표준편차
60	.21	.10

변별도2	문항수	비율(%)
0.3미만	45	75.0
0.3이상	15	25.0
전체	60	100.0

해석

- 의학총론 과목에서 난이도 지수가 80 에서 100 사이인 문항이 전체 60 문항 중 36 문항으로 가장 많았으며, 차례로 60 이상 80 미만인 문항이 13 문항, 0 에서 60 미만인 문항이 11 문항인 것으로 나타남
- 변별도 1 지수를 기준으로 분류하였을 때, 0.3 미만인 문항이 47 문항으로 0.3 이상인 문항이 13 문항인 것에 비해 더 많이 나타남
- 변별도 2 지수를 기준으로 분류하였을 때, 0.3 미만인 문항이 45 문항으로 0.3 이상인 문항이 15 문항인 것에 비해 더 많이 나타남

(2) 의학각론 난이도와 변별도 분포도 및 비율분석

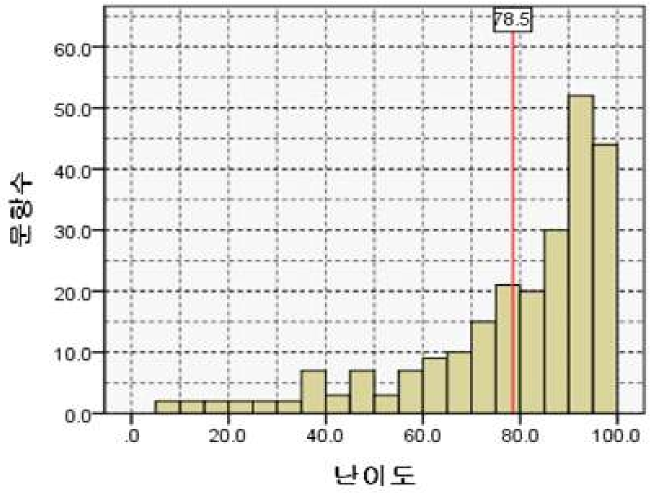

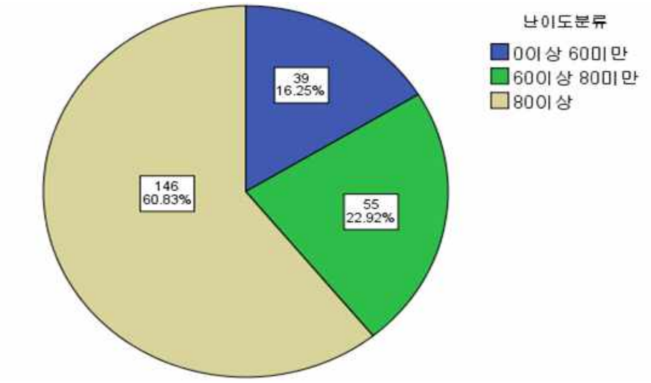

총점	난이도	표준편차
240	78.5	20.8

난이도	문항수	비율(%)
0~60미만	39	16.3
60~80미만	55	22.9
80~100	146	60.8
전체	240	100.0

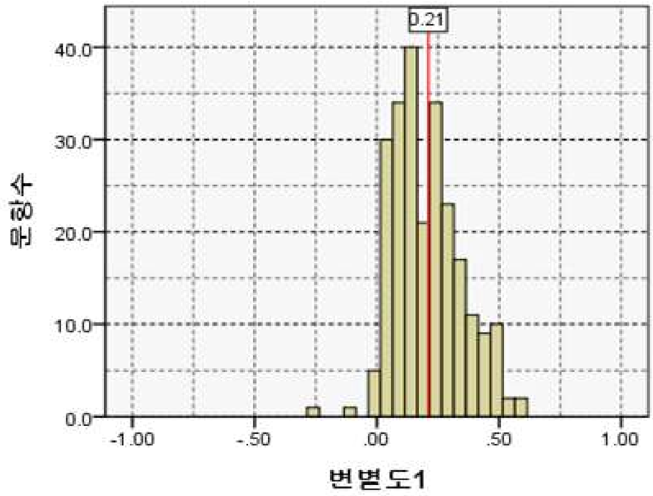

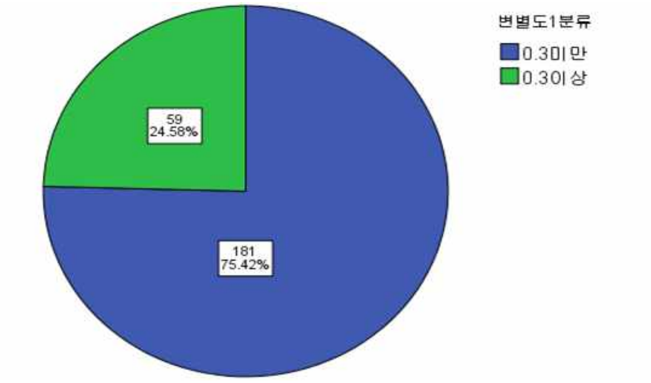

총점	변별도1	표준편차
240	.21	.14

변별도1	문항수	비율(%)
0.3미만	181	75.4
0.3이상	59	24.6
전체	240	100.0

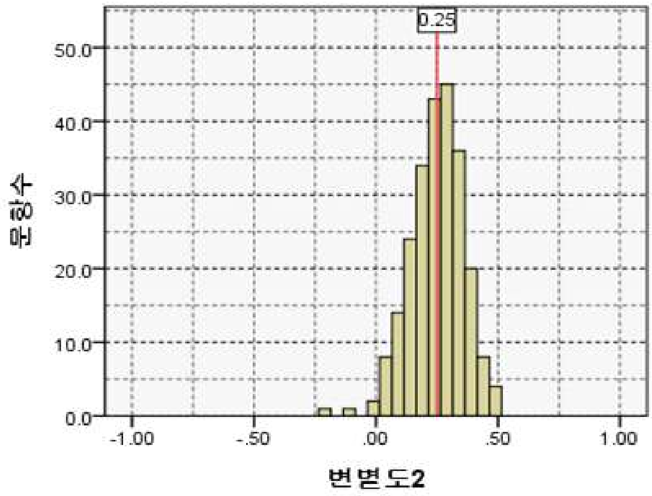

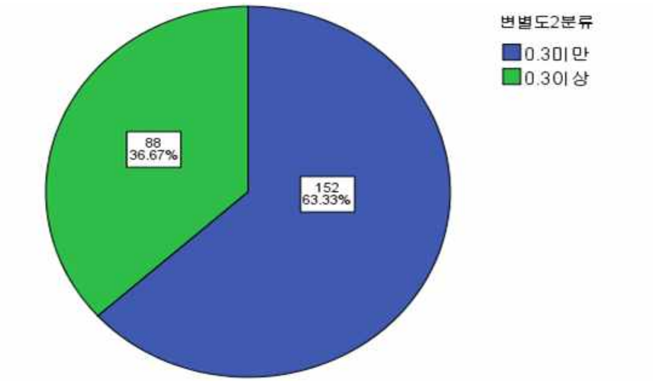

총점	변별도2	표준편차
240	.25	.11

변별도2	문항수	비율(%)
0.3미만	152	63.3
0.3이상	88	36.7
전체	240	100.0

해석

- 의학각론 과목에서 난이도 지수가 80 에서 100 사이인 문항이 전체 240 문항 중 146 문항으로 가장 많았으며, 차례로 60 이상 80 미만인 문항이 55 문항, 0 에서 60 미만인 문항이 39 문항인 것으로 나타남
- 변별도 1 지수를 기준으로 분류하였을 때, 0.3 미만인 문항이 181 문항으로 0.3 이상인 문항이 59 문항인 것에 비해 더 많이 나타남
- 변별도 2 지수를 기준으로 분류하였을 때, 0.3 미만인 문항이 152 문항으로 0.3 이상인 문항이 88 문항인 것에 비해 더 많이 나타남

(3) 보건의약관계법규 난이도와 변별도 분포도 및 비율분석

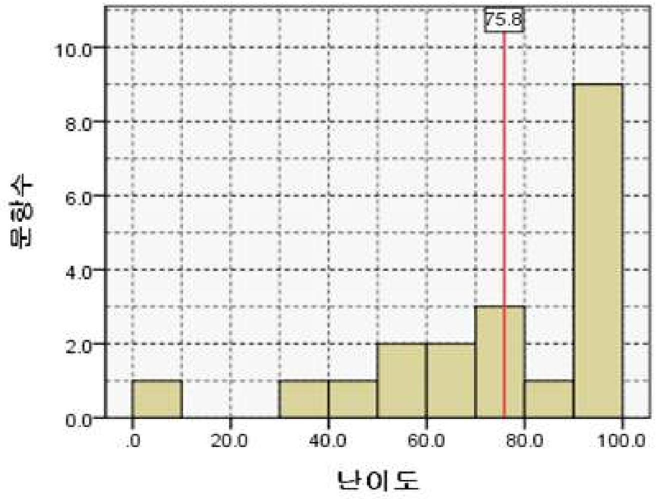

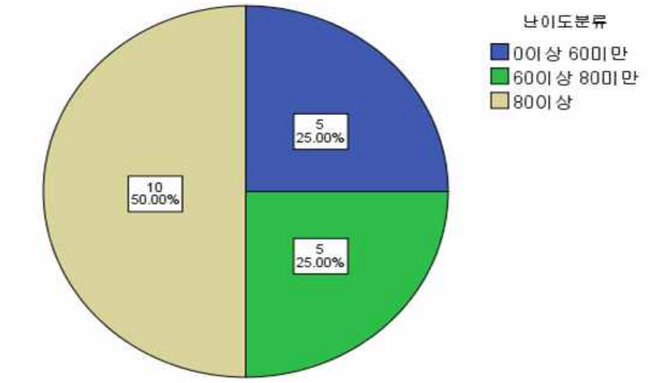

총점	난이도	표준편차
20	75.8	24.5

난이도	문항수	비율(%)
0~60미만	5	25.0
60~80미만	5	25.0
80~100	10	50.0
전체	20	100.0

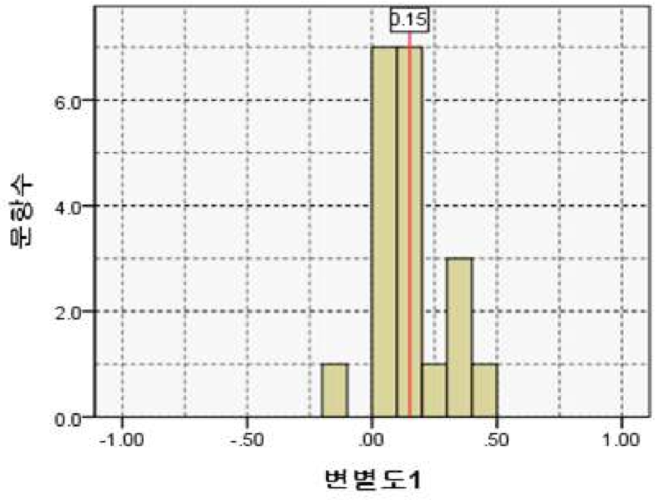

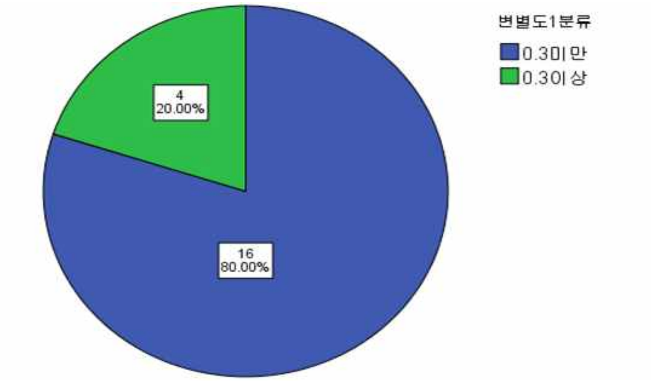

총점	변별도1	표준편차
20	.15	.15

변별도1	문항수	비율(%)
0.3미만	16	80.0
0.3이상	4	20.0
전체	20	100.0

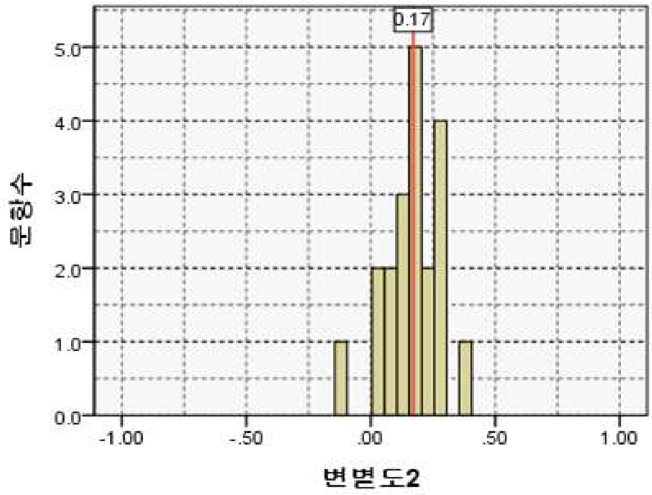

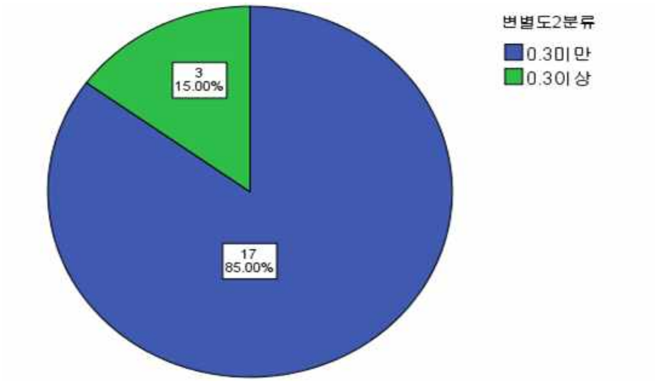

총점	변별도2	표준편차
20	.17	.12

변별도2	문항수	비율(%)
0.3미만	17	85.0
0.3이상	3	15.0
전체	20	100.0

해석

- 보건의약관계법규 과목에서 난이도 지수가 80 에서 100 사이인 문항이 전체 20 문항 중 10 문항으로 가장 많았으며, 차례로 60 이상 80 미만인 문항이 5 문항, 0 에서 60 미만인 문항이 5 문항인 것으로 나타남
- 변별도 1 지수를 기준으로 분류하였을 때, 0.3 미만인 문항이 16 문항으로 0.3 이상인 문항이 4 문항인 것에 비해 더 많이 나타남
- 변별도 2 지수를 기준으로 분류하였을 때, 0.3 미만인 문항이 17 문항으로 0.3 이상인 문항이 3 문항인 것에 비해 더 많이 나타남

3) 지식수준별 난이도와 변별도

가) 전회 대비 지식수준별 난이도와 변별도

(1) 전회 대비 암기형 난이도와 변별도

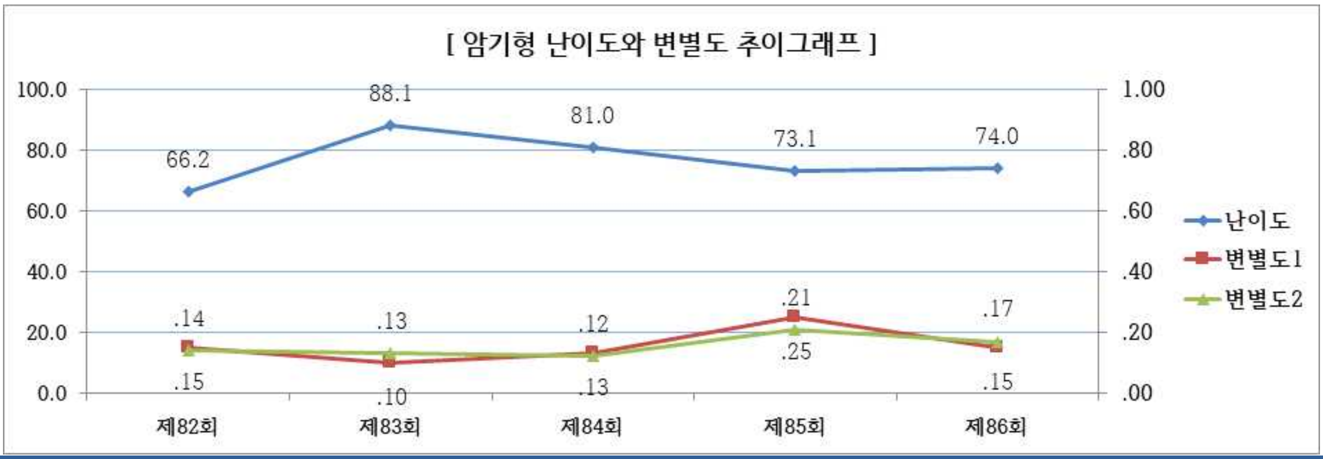

회차	난이도		변별도1		변별도2	
	평균	표준편차	평균	표준편차	평균	표준편차
제82회	66.2	26.2	.15	.14	.14	.11
제83회※	88.1	-	.10	-	.13	-
제84회	81.0	11.3	.13	.13	.12	.12
제85회	73.1	14.3	.25	.13	.21	.06
**제86회**	**74.0**	**32.5**	**.15**	**.13**	**.17**	**.11**

해석

- 전회 대비 암기형 문항의 난이도 지수는 0.9 증가함
- 전회 대비 암기형 문항의 변별도 1 지수는 0.10 감소함
- 전회 대비 암기형 문항의 변별도 2 지수는 0.04 감소함

※ 1개 문항 출제로 표준편차가 없음

(2) 전회 대비 해석형 난이도와 변별도

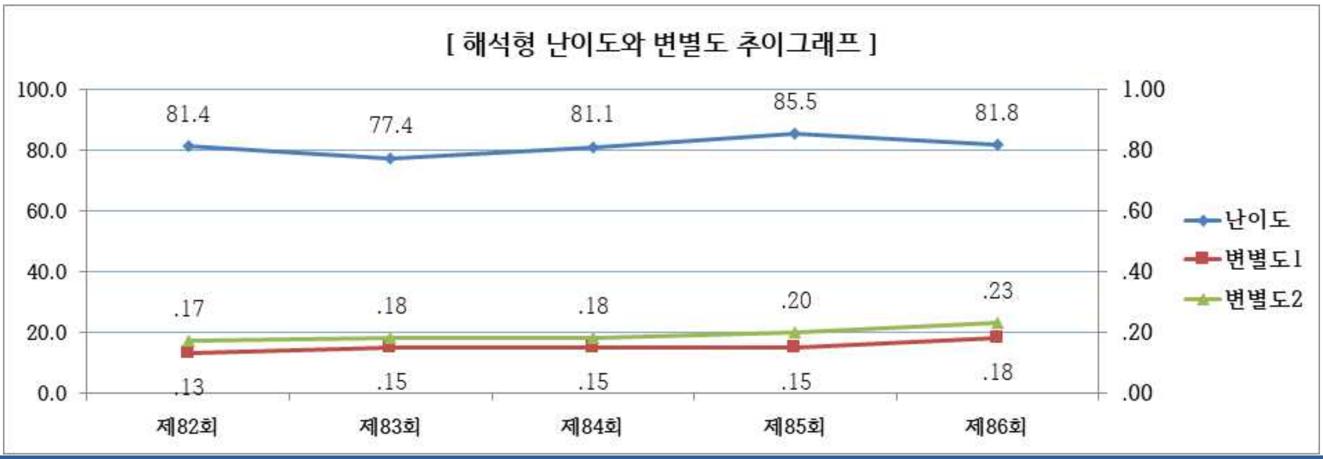

회차	난이도		변별도1		변별도2	
	평균	표준편차	평균	표준편차	평균	표준편차
제82회	81.4	20.6	.13	.11	.17	.09
제83회	77.4	22.7	.15	.11	.18	.09
제84회	81.1	18.4	.15	.12	.18	.09
제85회	85.5	17.1	.15	.13	.20	.10
**제86회**	**81.8**	**16.2**	**.18**	**.12**	**.23**	**.10**

해석

- 전회 대비 해석형 문항의 난이도 지수는 3.7 감소함
- 전회 대비 해석형 문항의 변별도 1 지수는 0.03 증가함
- 전회 대비 해석형 문항의 변별도 2 지수는 0.03 증가함

(3) 전회 대비 해결형 난이도와 변별도

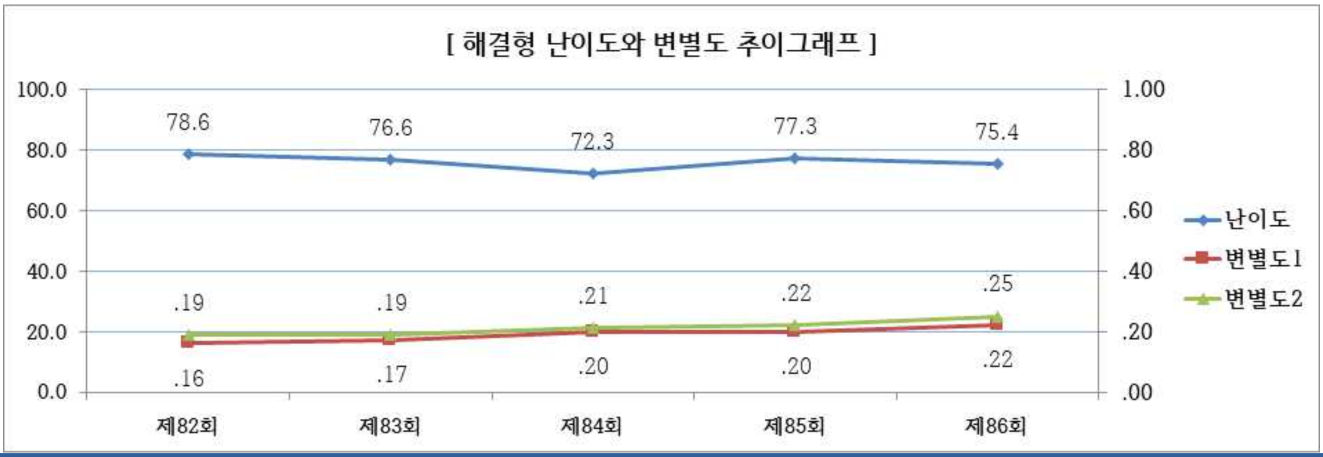

회차	난이도		변별도1		변별도2	
	평균	표준편차	평균	표준편차	평균	표준편차
제82회	78.6	20.5	.16	.11	.19	.09
제83회	76.6	21.5	.17	.11	.19	.09
제84회	72.3	22.7	.20	.14	.21	.11
제85회	77.3	21.1	.20	.15	.22	.11
**제86회**	**75.4**	**22.8**	**.22**	**.15**	**.25**	**.12**

해석

- 전회 대비 해결형 문항의 난이도 지수는 1.9 감소함
- 전회 대비 해결형 문항의 변별도 1 지수는 0.02 증가함
- 전회 대비 해결형 문항의 변별도 2 지수는 0.03 증가함

나) 지식수준별 난이도와 변별도 분포도 및 비율분석

(1) 암기형 난이도와 변별도 분포도 및 비율분석

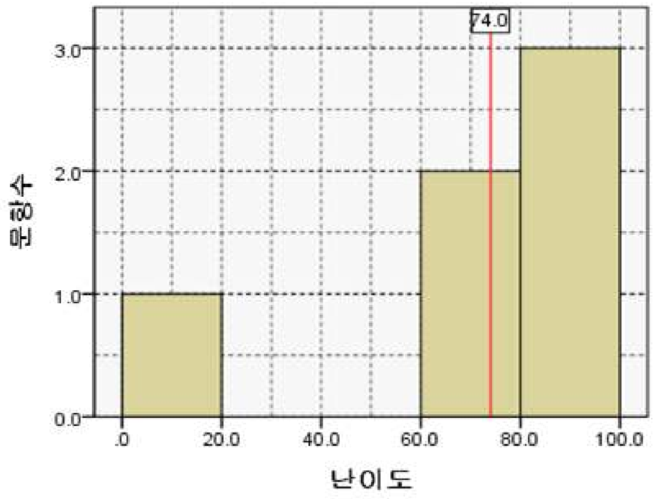

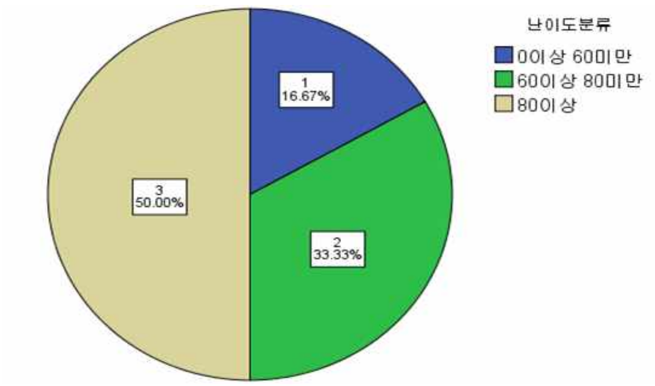

총점	난이도	표준편차
6	74.0	32.5

난이도	문항수	비율(%)
0~60미만	1	16.7
60~80미만	2	33.3
80~100	3	50.0
전체	6	100.0

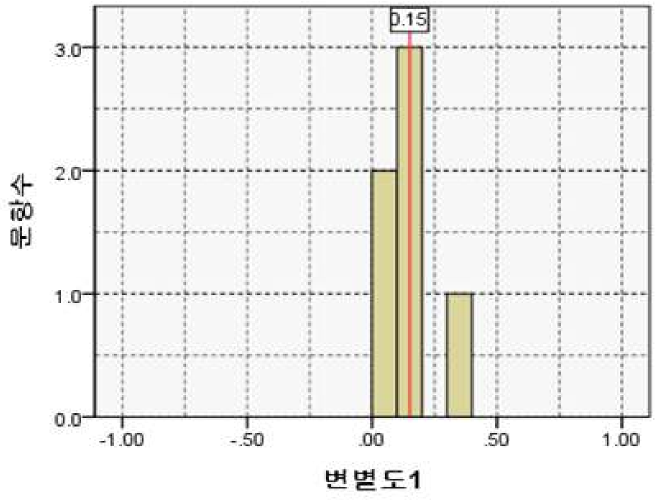

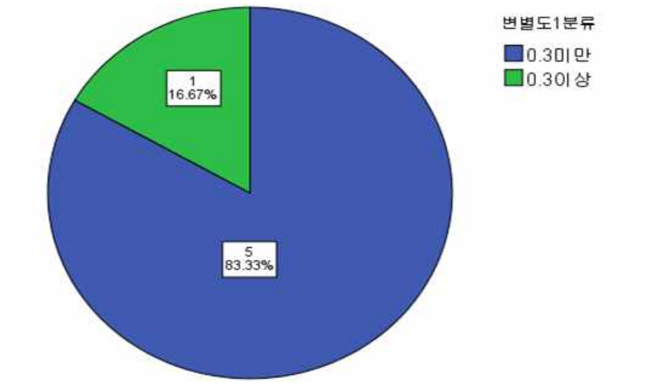

총점	변별도1	표준편차
6	.15	.13

변별도1	문항수	비율(%)
0.3미만	5	83.3
0.3이상	1	16.7
전체	6	100.0

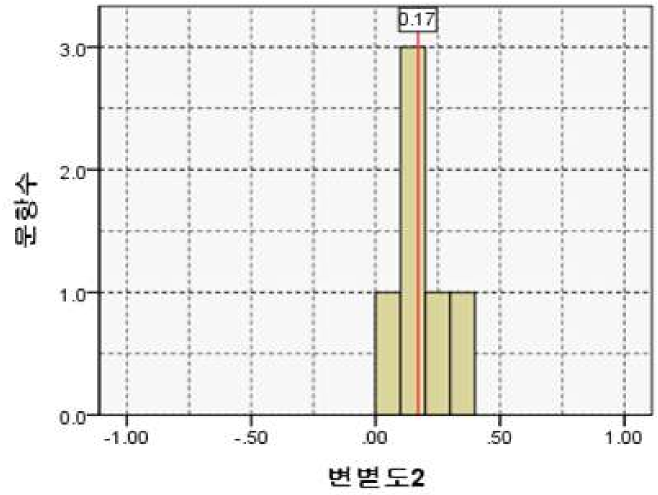

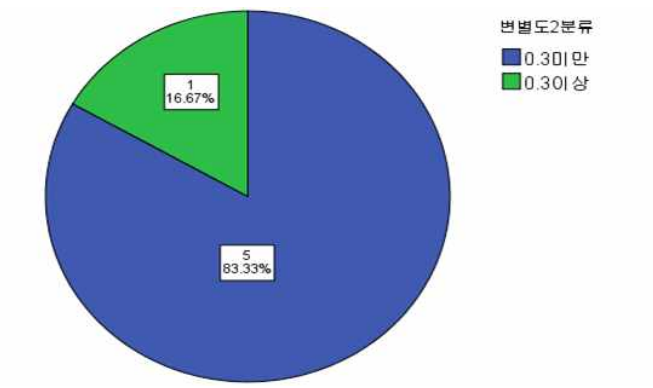

총점	변별도2	표준편차
6	.17	.11

변별도2	문항수	비율(%)
0.3미만	5	83.3
0.3이상	1	16.7
전체	6	100.0

해석

- 암기형 문항에서 난이도 지수가 80 에서 100 사이인 문항이 전체 6 문항 중 3 문항으로 가장 많았으며, 차례로 60 이상 80 미만인 문항이 2 문항, 0 에서 60 미만인 문항이 1 문항인 것으로 나타남
- 변별도 1 지수를 기준으로 분류하였을 때, 0.3 미만인 문항이 5 문항으로 0.3 이상인 문항이 1 문항인 것에 비해 더 많이 나타남
- 변별도 2 지수를 기준으로 분류하였을 때, 0.3 미만인 문항이 5 문항으로 0.3 이상인 문항이 1 문항인 것에 비해 더 많이 나타남

(2) 해석형 난이도와 변별도 분포도 및 비율분석

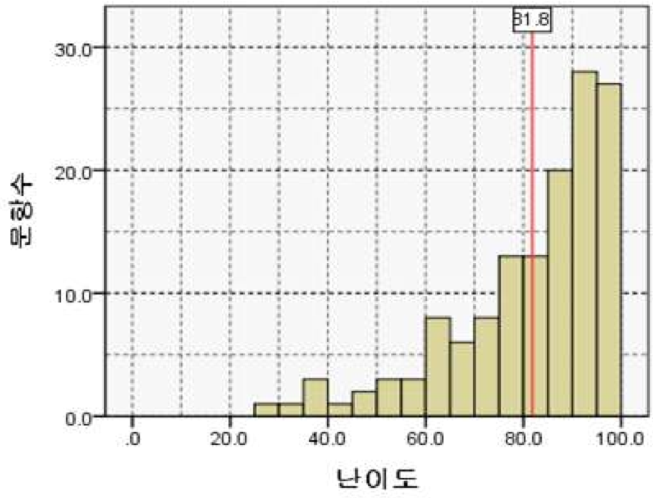

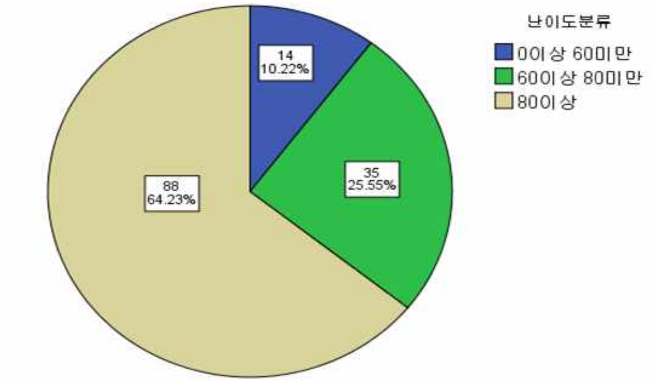

총점	난이도	표준편차
137	81.8	16.2

난이도	문항수	비율(%)
0~60미만	14	10.2
60~80미만	35	25.5
80~100	88	64.2
전체	137	100.0

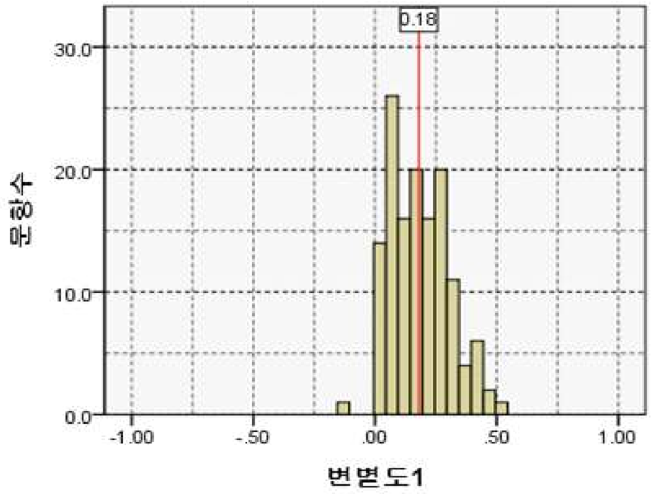

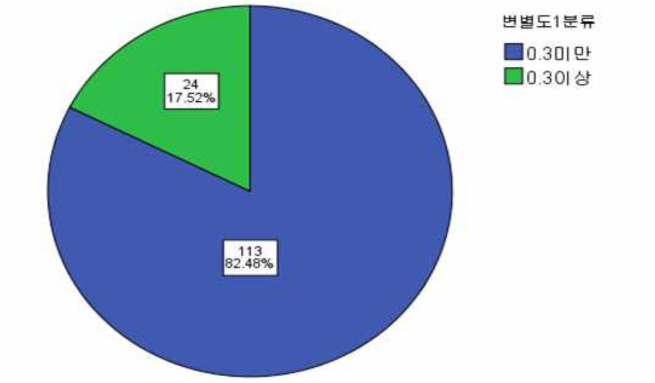

총점	변별도1	표준편차
137	.18	.12

변별도1	문항수	비율(%)
0.3미만	113	82.5
0.3이상	24	17.5
전체	137	100.0

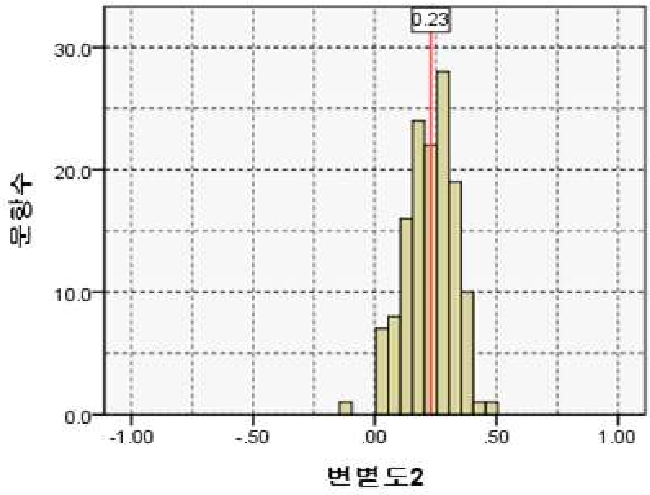

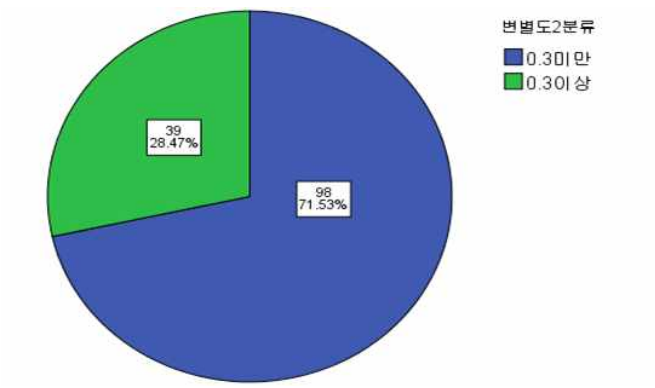

총점	변별도2	표준편차
137	.23	.10

변별도2	문항수	비율(%)
0.3미만	98	71.5
0.3이상	39	28.5
전체	137	100.0

해석

- 해석형 문항에서 난이도 지수가 80 에서 100 사이인 문항이 전체 137 문항 중 88 문항으로 가장 많았으며, 차례로 60 이상 80 미만인 문항이 35 문항, 0 에서 60 미만인 문항이 14 문항인 것으로 나타남
- 변별도 1 지수를 기준으로 분류하였을 때, 0.3 미만인 문항이 113 문항으로 0.3 이상인 문항이 24 문항인 것에 비해 더 많이 나타남
- 변별도 2 지수를 기준으로 분류하였을 때, 0.3 미만인 문항이 98 문항으로 0.3 이상인 문항이 39 문항인 것에 비해 더 많이 나타남

(3) 해결형 난이도와 변별도 분포도 및 비율분석

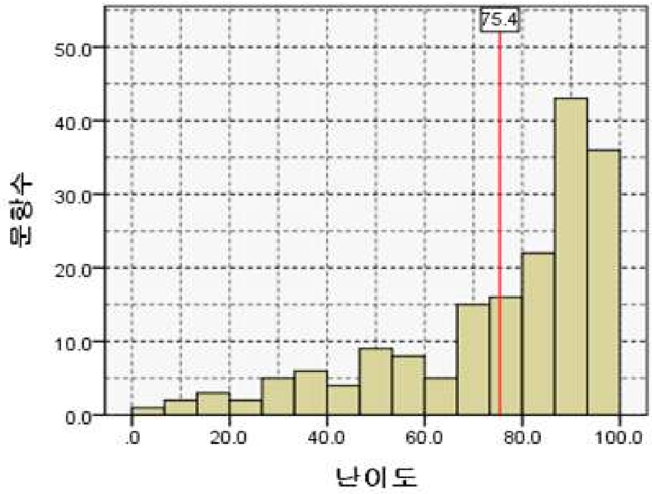

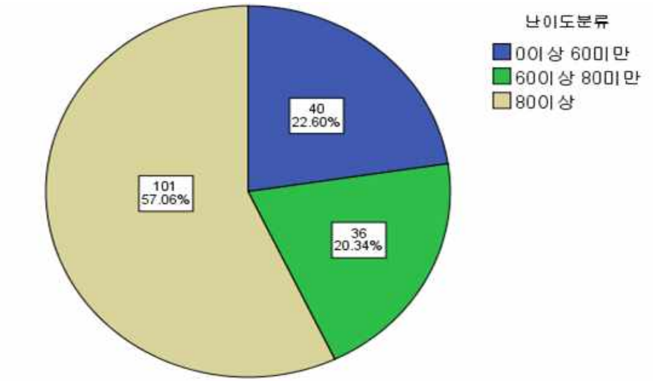

총점	난이도	표준편차
177	75.4	22.8

난이도	문항수	비율(%)
0~60미만	40	22.6
60~80미만	36	20.3
80~100	101	57.1
전체	177	100.0

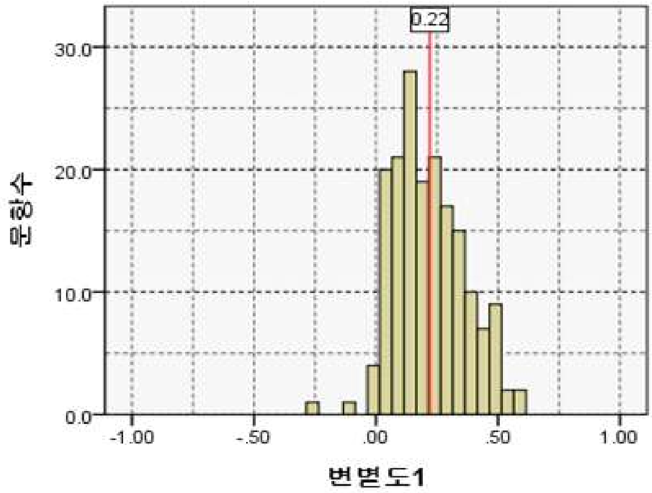

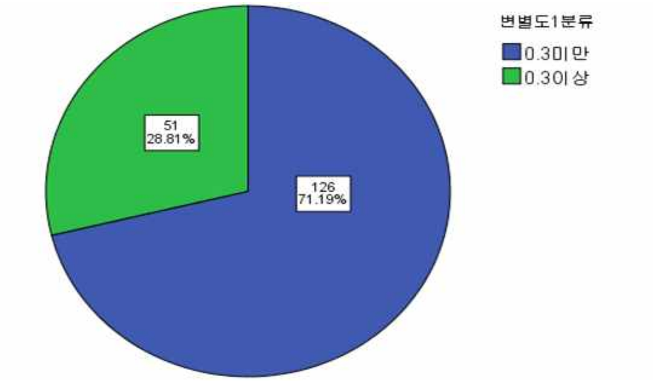

총점	변별도1	표준편차
177	.22	.15

변별도1	문항수	비율(%)
0.3미만	126	71.2
0.3이상	51	28.8
전체	177	100.0

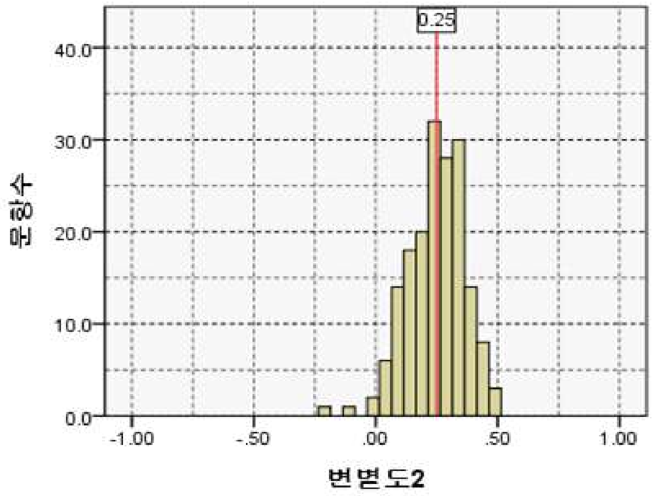

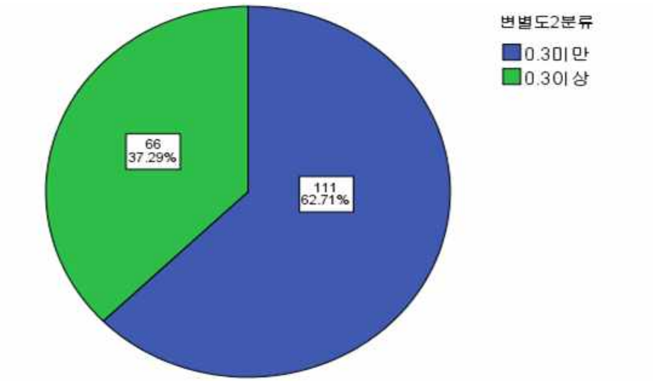

총점	변별도2	표준편차
177	.25	.12

변별도2	문항수	비율(%)
0.3미만	111	62.7
0.3이상	66	37.3
전체	177	100.0

해석

- 해결형 문항에서 난이도 지수가 80에서 100사이인 문항이 전체 177문항 중 101문항으로 가장 많았으며, 차례로 0에서 60미만인 문항이 40문항, 60이상 80미만인 문항이 36문항인 것으로 나타남
- 변별도1 지수를 기준으로 분류하였을 때, 0.3미만인 문항이 126문항으로 0.3 이상인 문항이 51문항인 것에 비해 더 많이 나타남
- 변별도2 지수를 기준으로 분류하였을 때, 0.3미만인 문항이 111문항으로 0.3 이상인 문항이 66문항인 것에 비해 더 많이 나타남

4) 자료유형별 난이도와 변별도

가) 전회 대비 자료유형별 난이도와 변별도

(1) 전회 대비 텍스트형 난이도와 변별도

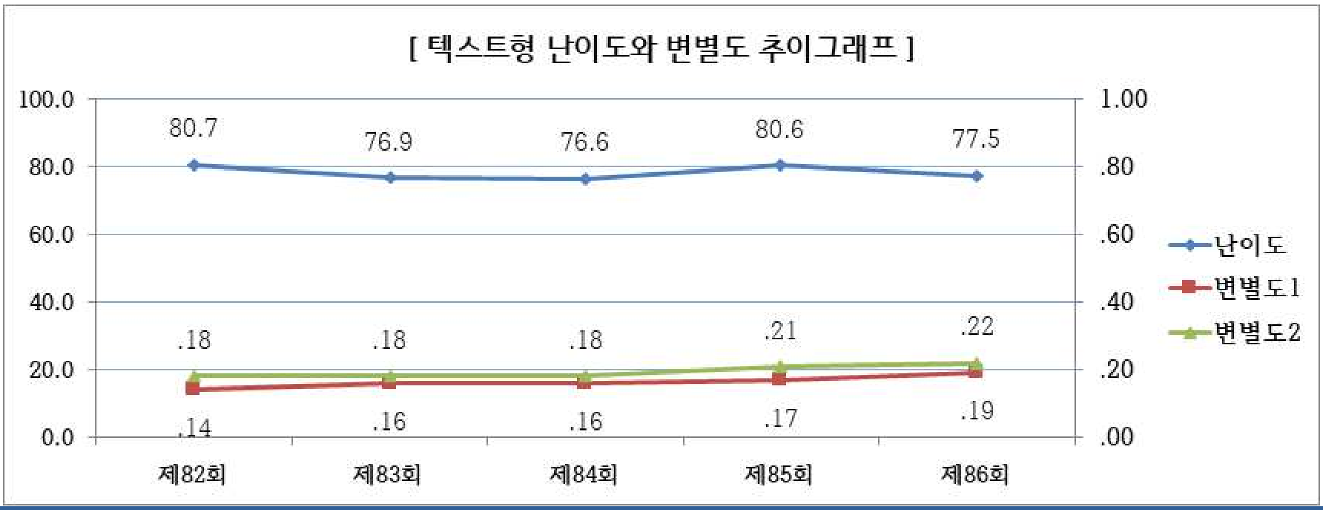

회차	난이도		변별도1		변별도2	
	평균	표준편차	평균	표준편차	평균	표준편차
제82회	80.7	20.3	.14	.11	.18	.09
제83회	76.9	22.9	.16	.12	.18	.09
제84회	76.6	22.0	.16	.12	.18	.10
제85회	80.6	20.0	.17	.14	.21	.11
**제86회**	**77.5**	**21.3**	**.19**	**.14**	**.22**	**.12**

해석

- 전회 대비 텍스트형 문항의 난이도 지수는 3.1 감소함
- 전회 대비 텍스트형 문항의 변별도 1 지수는 0.02 증가함
- 전회 대비 텍스트형 문항의 변별도 2 지수는 0.01 증가함

(2) 전회 대비 자료제시형 난이도와 변별도

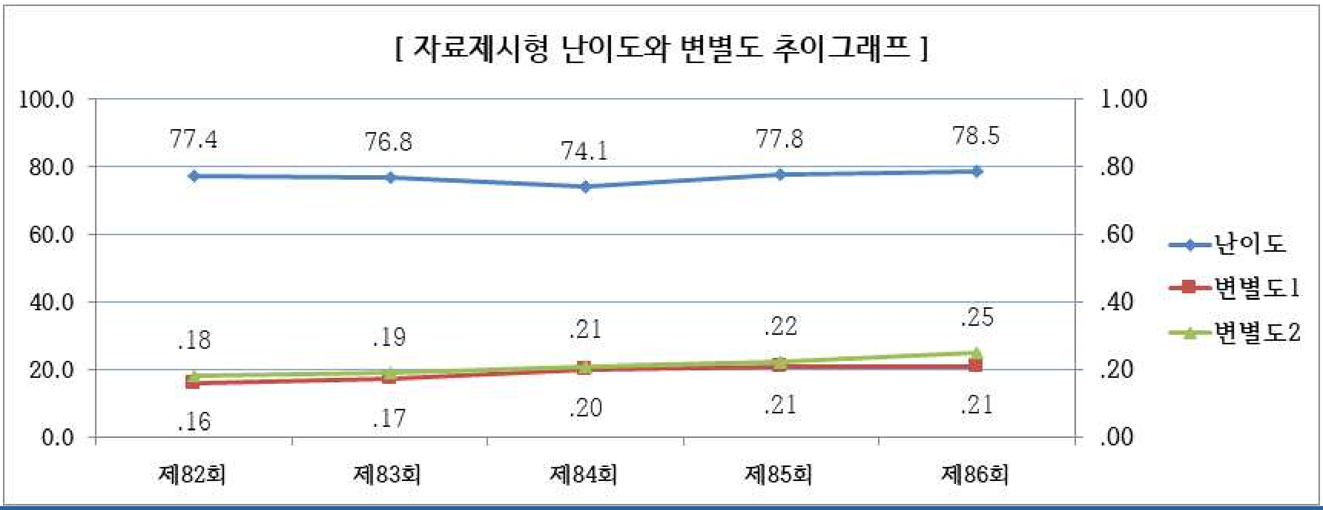

회차	난이도		변별도1		변별도2	
	평균	표준편차	평균	표준편차	평균	표준편차
제82회	77.4	20.9	.16	.11	.18	.09
제83회	76.8	20.7	.17	.11	.19	.09
제84회	74.1	21.3	.20	.14	.21	.10
제85회	77.8	20.8	.21	.15	.22	.11
**제86회**	**78.5**	**20.1**	**.21**	**.13**	**.25**	**.10**

해석

- 전회 대비 자료제시형 문항의 난이도 지수는 0.7 증가함
- 전회 대비 자료제시형 문항의 변별도 1 지수는 동일함
- 전회 대비 자료제시형 문항의 변별도 2 지수는 0.03 증가함

나) 자료유형별 난이도와 변별도 분포도 및 비율분석

(1) 텍스트형 난이도와 변별도 분포도 및 비율분석

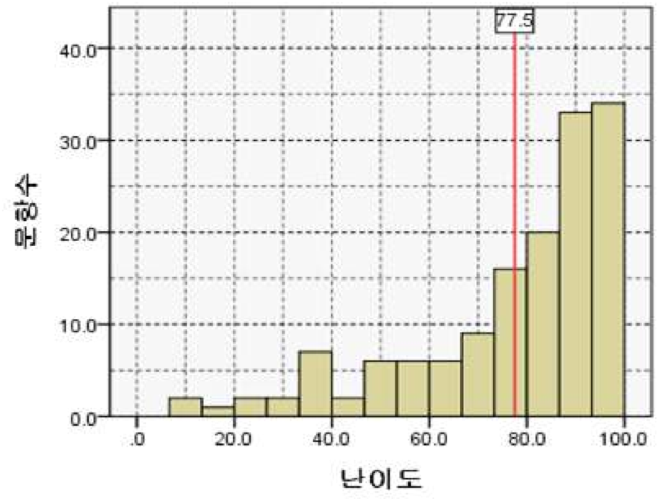

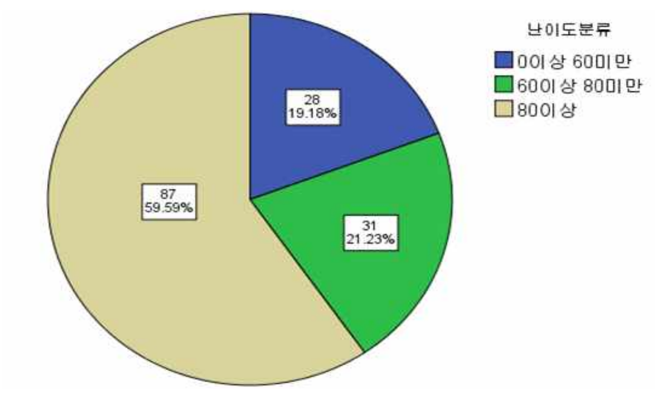

총점	난이도	표준편차
146	77.5	21.3

난이도	문항수	비율(%)
0~60미만	28	19.2
60~80미만	31	21.2
80~100	87	59.6
전체	146	100.0

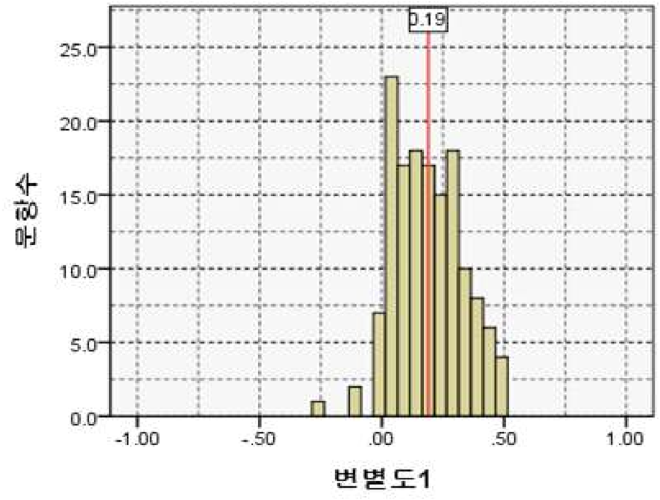

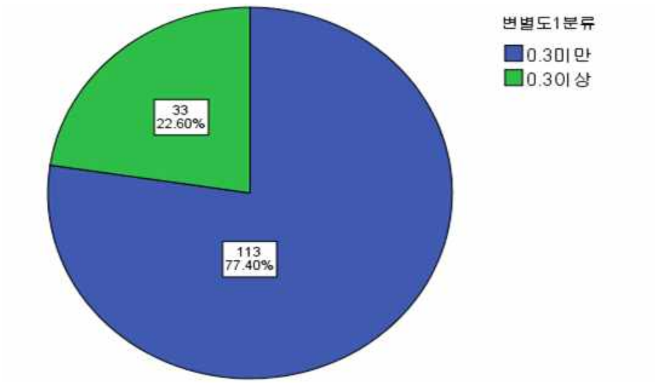

총점	변별도1	표준편차
146	.19	.14

변별도1	문항수	비율(%)
0.3미만	113	77.4
0.3이상	33	22.6
전체	146	100.0

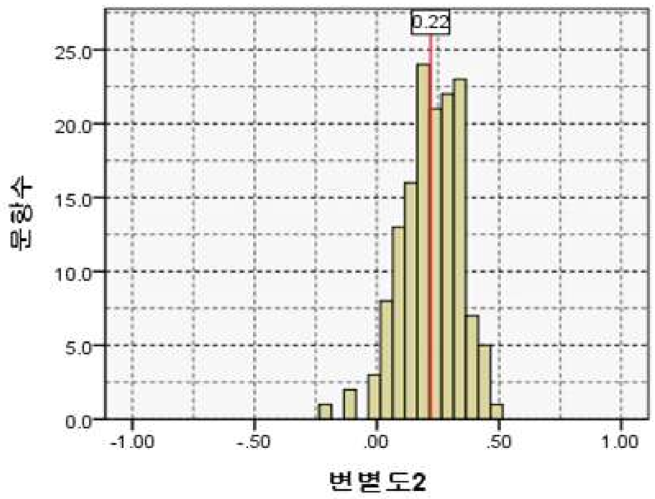

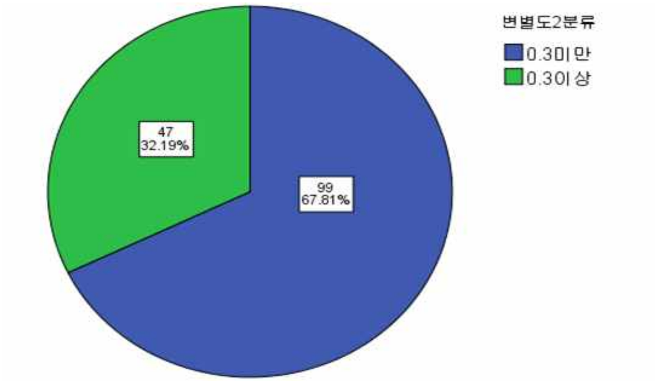

총점	변별도2	표준편차
146	.22	.12

변별도2	문항수	비율(%)
0.3미만	99	67.8
0.3이상	47	32.2
전체	146	100.0

해석

- 텍스트형 문항에서 난이도 지수가 80 에서 100 사이인 문항이 전체 146 문항 중 87 문항으로 가장 많았으며, 차례로 60 이상 80 미만인 문항이 31 문항, 0 에서 60 미만인 문항이 28 문항인 것으로 나타남
- 변별도 1 지수를 기준으로 분류하였을 때, 0.3 미만인 문항이 113 문항으로 0.3 이상인 문항이 33 문항인 것에 비해 더 많이 나타남
- 변별도 2 지수를 기준으로 분류하였을 때, 0.3 미만인 문항이 99 문항으로 0.3 이상인 문항이 47 문항인 것에 비해 더 많이 나타남

(2) 자료제시형 난이도와 변별도 분포도 및 비율분석

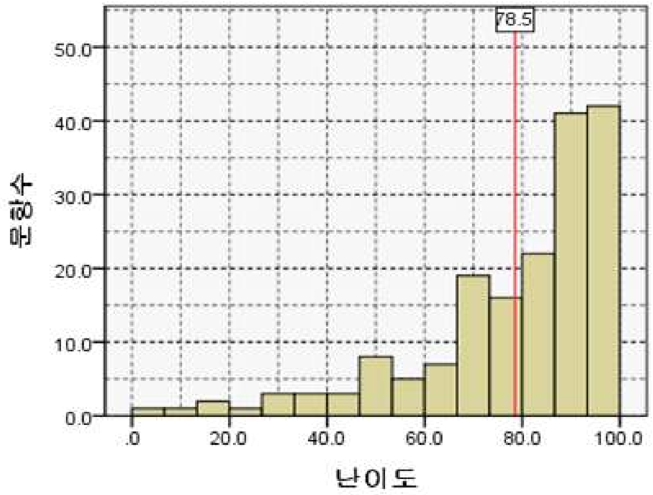

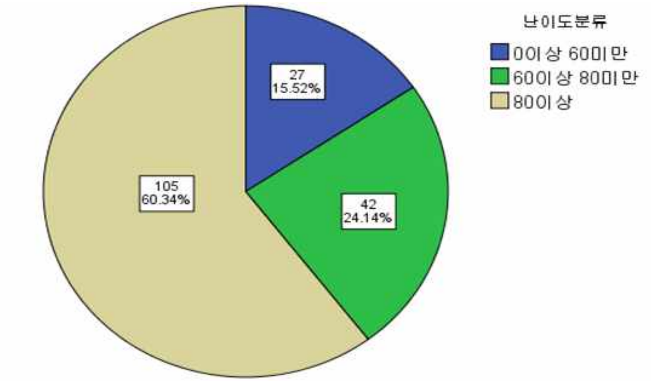

총점	난이도	표준편차
174	78.5	20.1

난이도	문항수	비율(%)
0~60미만	27	15.5
60~80미만	42	24.1
80~100	105	60.3
전체	174	100.0

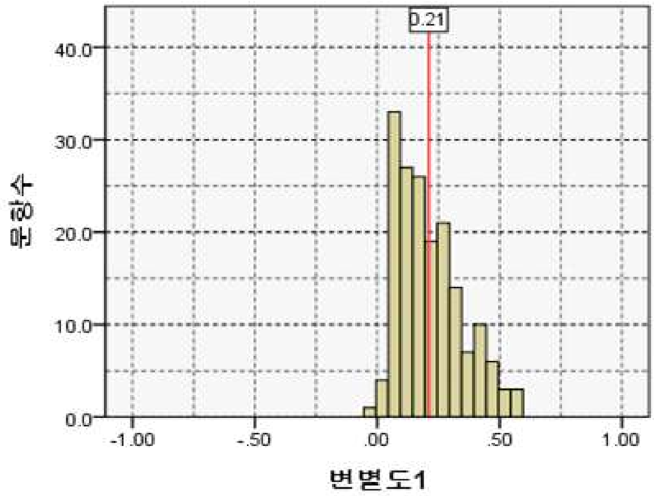

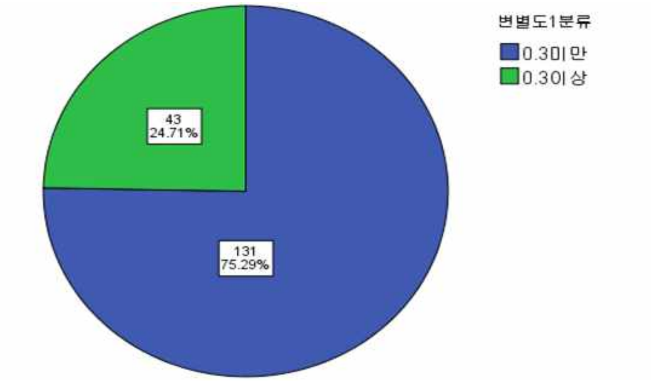

총점	변별도1	표준편차
174	.21	.13

변별도1	문항수	비율(%)
0.3미만	131	75.3
0.3이상	43	24.7
전체	174	100.0

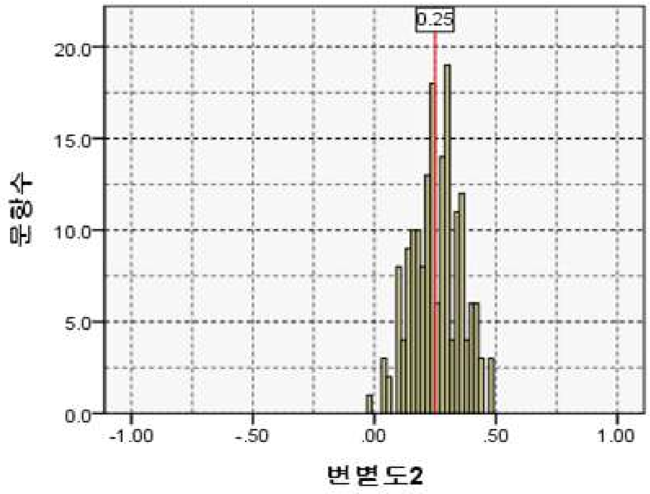

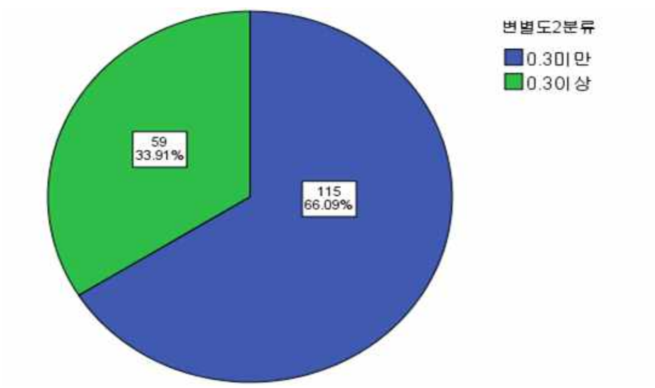

총점	변별도2	표준편차
174	.25	.10

변별도2	문항수	비율(%)
0.3미만	115	66.1
0.3이상	59	33.9
전체	174	100.0

해석

- 자료제시형 문항에서 난이도 지수가 80 에서 100 사이인 문항이 전체 174 문항 중 105 문항으로 가장 많았으며, 차례로 60 이상 80 미만인 문항이 42 문항, 0 에서 60 미만인 문항이 27 문항인 것으로 나타남
- 변별도 1 지수를 기준으로 분류하였을 때, 0.3 미만인 문항이 131 문항으로 0.3 이상인 문항이 43 문항인 것에 비해 더 많이 나타남
- 변별도 2 지수를 기준으로 분류하였을 때, 0.3 미만인 문항이 115 문항으로 0.3 이상인 문항이 59 문항인 것에 비해 더 많이 나타남

5) 문항형태별 난이도와 변별도

가) 전회 대비 문항형태별 난이도와 변별도

(1) 전회 대비 A형 난이도와 변별도

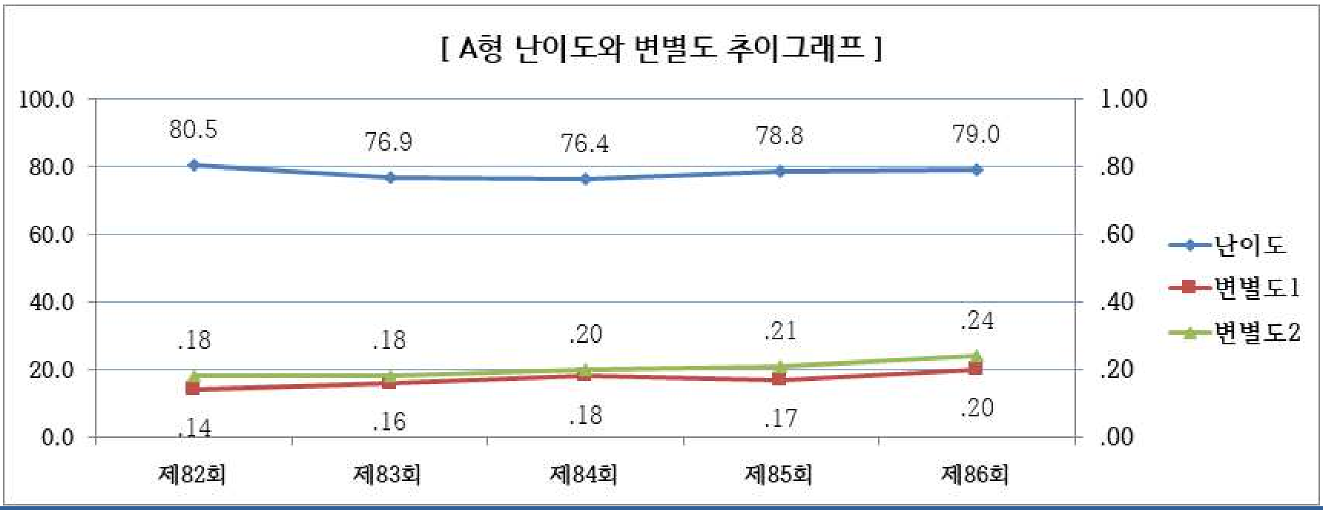

회차	난이도		변별도1		변별도2	
	평균	표준편차	평균	표준편차	평균	표준편차
제82회	80.5	19.4	.14	.10	.18	.09
제83회	76.9	22.2	.16	.11	.18	.09
제84회	76.4	21.1	.18	.13	.20	.10
제85회	78.8	20.9	.17	.13	.21	.11
**제86회**	**79.0**	**19.7**	**.20**	**.14**	**.24**	**.11**

해석

- 전회 대비 A형 문항의 난이도 지수는 0.2 증가함
- 전회 대비 A형 문항의 변별도1 지수는 0.03 증가함
- 전회 대비 A형 문항의 변별도2 지수는 0.03 증가함

(2) 전회 대비 R형 난이도와 변별도

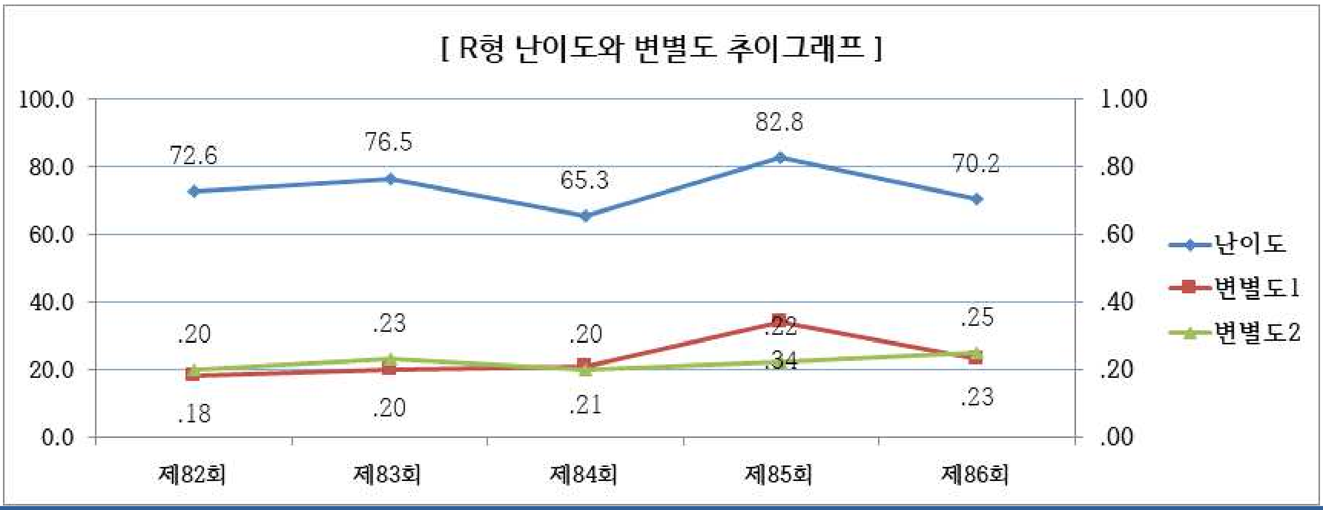

회차	난이도		변별도1		변별도2	
	평균	표준편차	평균	표준편차	평균	표준편차
제82회	72.6	25.1	.18	.13	.20	.09
제83회	76.5	18.5	.20	.11	.23	.08
제84회	65.3	24.5	.21	.13	.20	.10
제85회	82.8	16.1	.34	.20	.22	.11
**제86회**	**70.2**	**26.5**	**.23**	**.13**	**.25**	**.09**

해석

- 전회 대비 R 형 문항의 난이도 지수는 12.6 감소함
- 전회 대비 R 형 문항의 변별도 1 지수는 0.11 감소함
- 전회 대비 R 형 문항의 변별도 2 지수는 0.03 증가함

나) 문항형태별 난이도와 변별도 분포도 및 비율분석

(1) A형 난이도와 변별도 분포도 및 비율분석

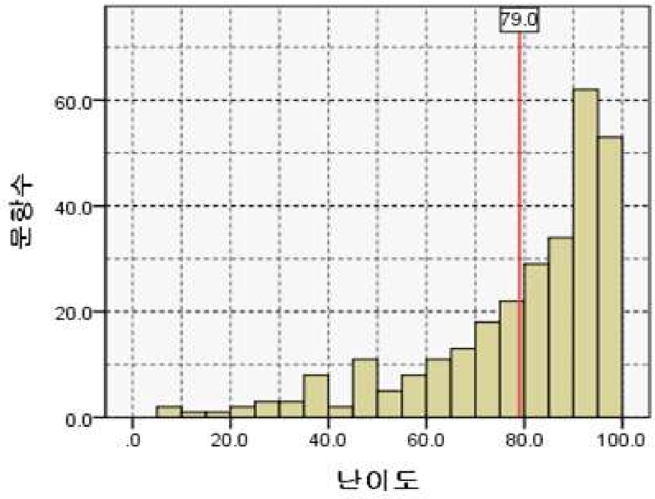

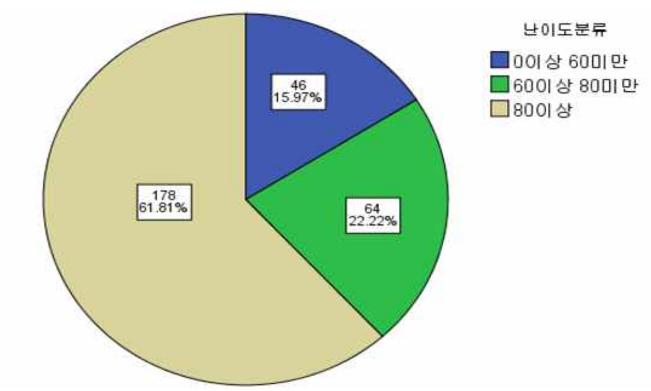

총점	난이도	표준편차
288	79.0	19.7

난이도	문항수	비율(%)
0~60미만	46	16.0
60~80미만	64	22.2
80~100	178	61.8
전체	288	100.0

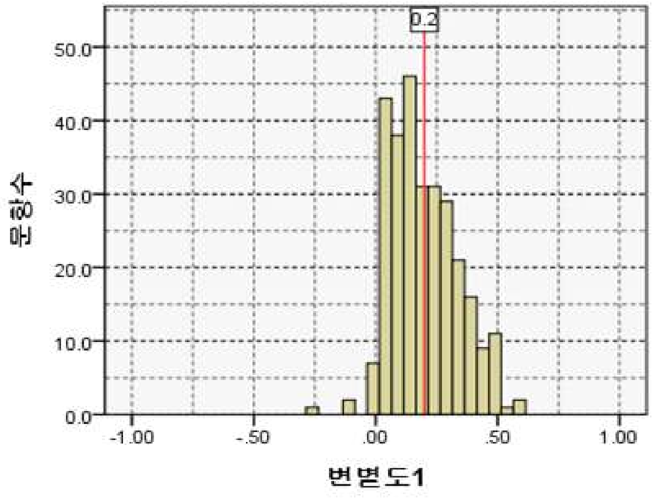

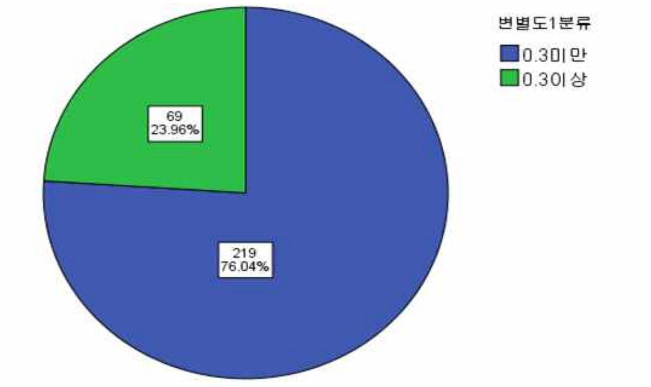

총점	변별도1	표준편차
288	.20	.14

변별도1	문항수	비율(%)
0.3미만	219	76.0
0.3이상	69	24.0
전체	288	100.0

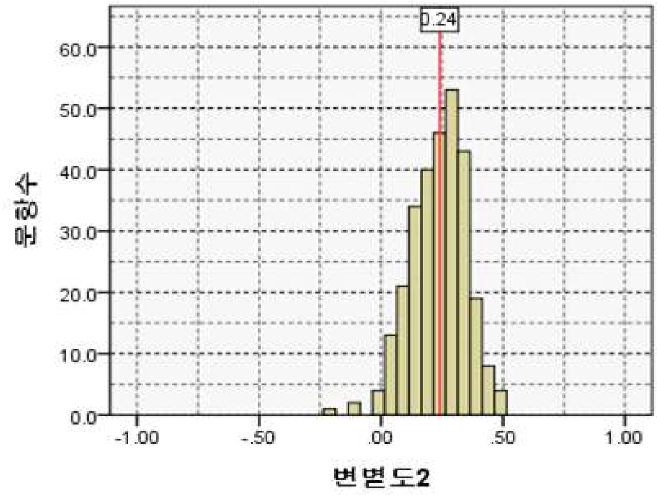

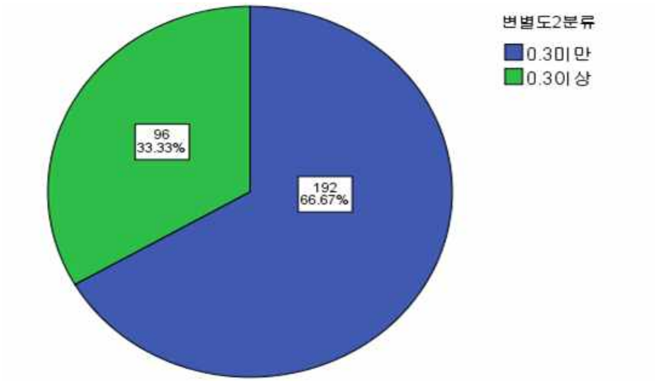

총점	변별도2	표준편차
288	.24	.11

변별도2	문항수	비율(%)
0.3미만	192	66.7
0.3이상	96	33.3
전체	288	100.0

해석

- A 형 문항에서 난이도 지수가 80 에서 100 사이인 문항이 전체 288 문항 중 178 문항으로 가장 많았으며, 차례로 60 이상 80 미만인 문항이 64 문항, 0 에서 60 미만인 문항이 46 문항인 것으로 나타남
- 변별도 1 지수를 기준으로 분류하였을 때, 0.3 미만인 문항이 219 문항으로 0.3 이상인 문항이 69 문항인 것에 비해 더 많이 나타남
- 변별도 2 지수를 기준으로 분류하였을 때, 0.3 미만인 문항이 192 문항으로 0.3 이상인 문항이 96 문항인 것에 비해 더 많이 나타남

(2) R형 난이도와 변별도 분포도 및 비율분석

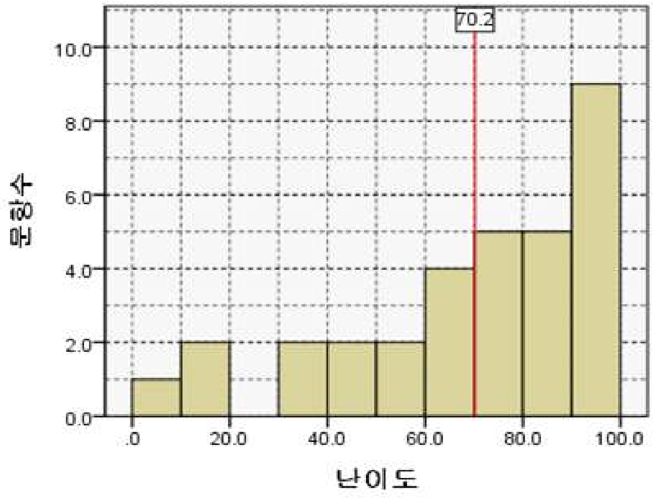

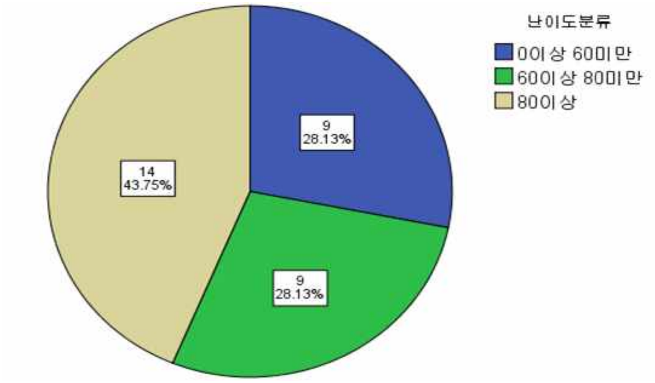

총점	난이도	표준편차
32	70.2	26.5

난이도	문항수	비율(%)
0~60미만	9	28.1
60~80미만	9	28.1
80~100	14	43.8
전체	32	100.0

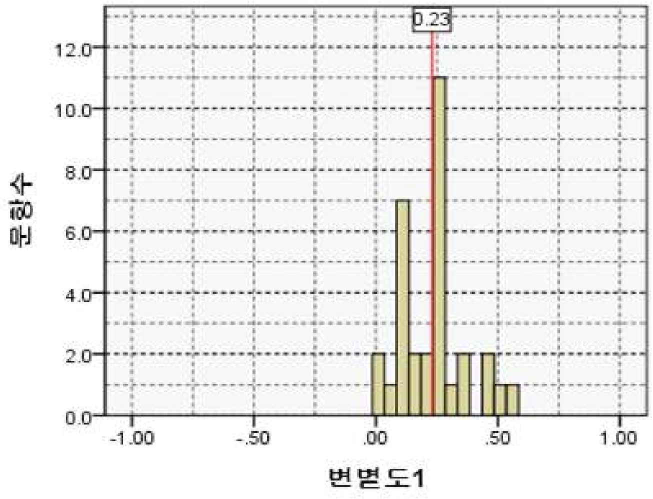

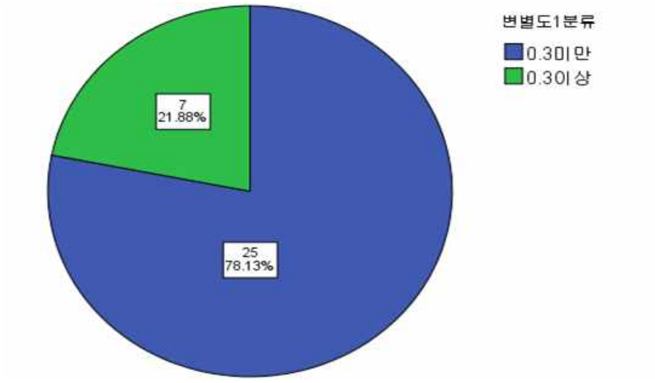

총점	변별도1	표준편차
32	.23	.13

변별도1	문항수	비율(%)
0.3미만	25	78.1
0.3이상	7	21.9
전체	32	100.0

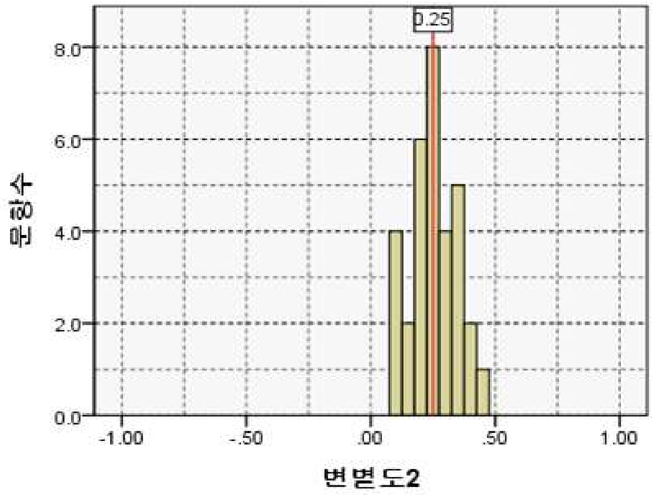

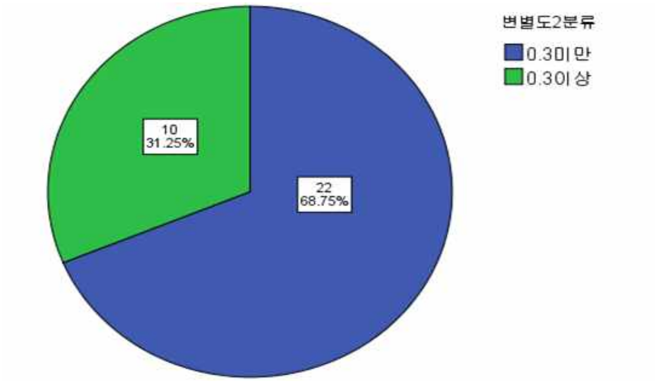

총점	변별도2	표준편차
32	.25	.09

변별도2	문항수	비율(%)
0.3미만	22	68.8
0.3이상	10	31.3
전체	32	100.0

해석

- R형 문항에서 난이도 지수가 80에서 100사이인 문항이 전체 32문항 중 14문항으로 가장 많았으며, 차례로 60이상 80미만인 문항이 9문항, 0에서 60미만인 문항이 9문항인 것으로 나타남
- 변별도1 지수를 기준으로 분류하였을 때, 0.3미만인 문항이 25문항으로 0.3이상인 문항이 7문항인 것에 비해 더 많이 나타남
- 변별도2 지수를 기준으로 분류하였을 때, 0.3미만인 문항이 22문항으로 0.3이상인 문항이 10문항인 것에 비해 더 많이 나타남

3. 난이도와 변별도 간 산포도

1) 전체 난이도와 변별도 간 산포도

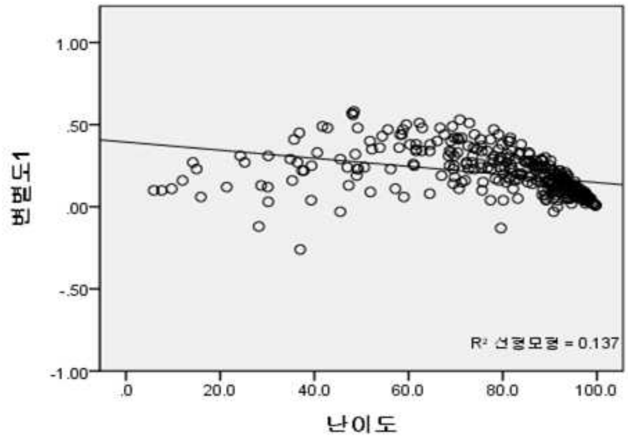

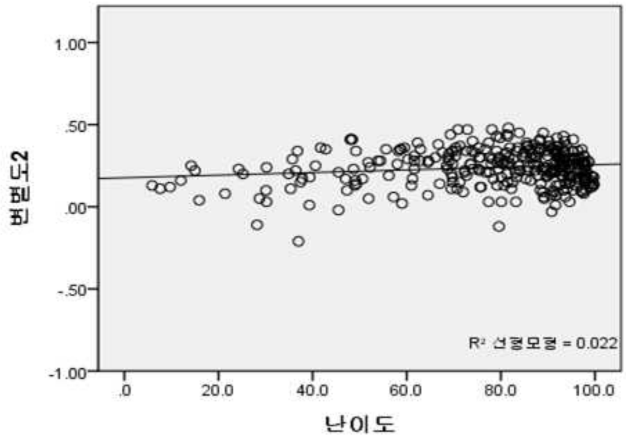

해석

- 전체 문항을 대상으로 난이도와 변별도 1 지수 간 상관은 -.371**로 문항 난이도가 쉬울수록 변별력이 낮아지는 것으로 나타남
- 난이도와 변별도 2 지수 간 상관은 .148**로 문항 난이도가 쉬울수록 변별력이 높아지는 것으로 나타남

2) 과목별 난이도와 변별도 간 산포도

가) 의학총론 난이도와 변별도 간 산포도

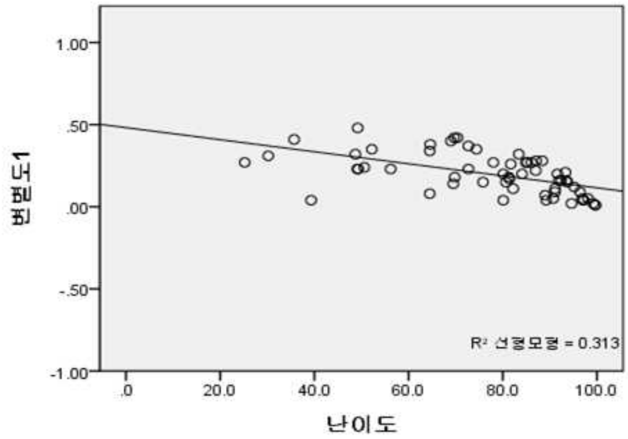

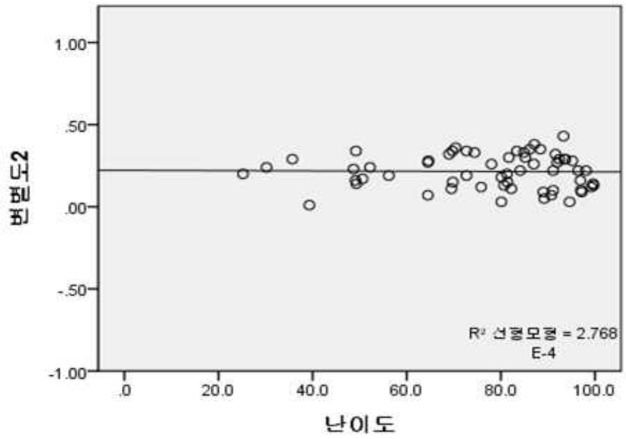

해석

- 의학총론 과목 문항을 대상으로 난이도와 변별도 1 지수 간 상관은 -.559** 로 문항 난이도가 쉬울수록 변별력이 낮아지는 것으로 나타남
- 난이도와 변별도 2 지수 간 상관은 -.017 로 문항 난이도와 변별력 간 관련성이 없는 것으로 나타남

나) 의학각론 난이도와 변별도 간 산포도

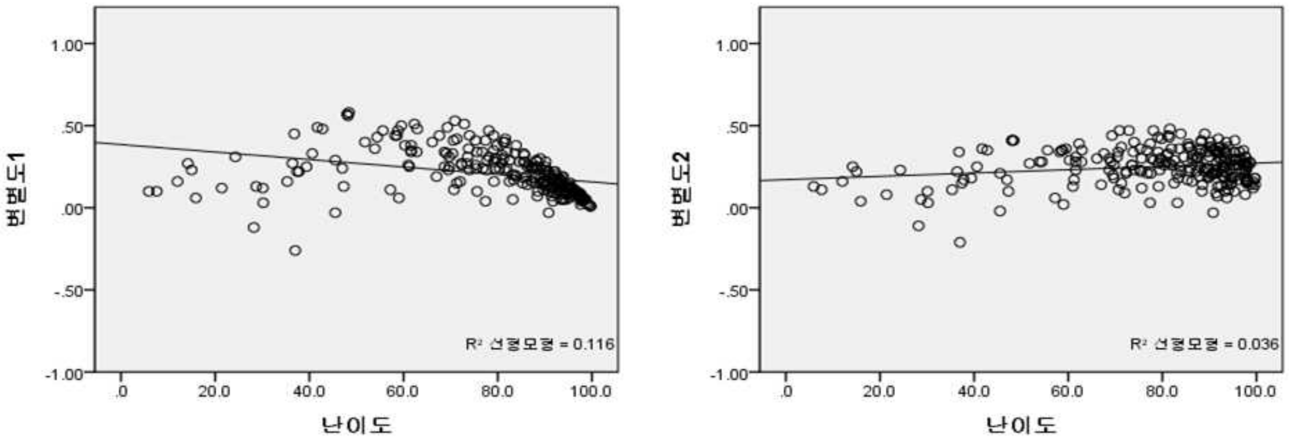

해석

- 의학각론 과목 문항을 대상으로 난이도와 변별도 1 지수 간 상관은 -.340** 으로 문항 난이도가 쉬울수록 변별력이 낮아지는 것으로 나타남
- 난이도와 변별도 2 지수 간 상관은 .191**로 문항 난이도가 쉬울수록 변별력이 높아지는 것으로 나타남

다) 보건의약관계법규 난이도와 변별도 간 산포도

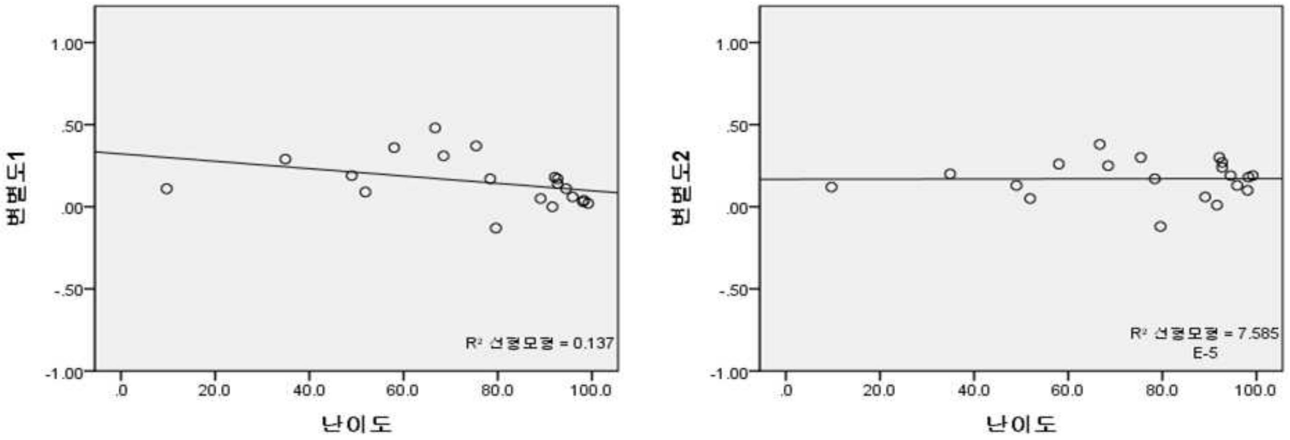

해석

- 보건의약관계법규 과목을 대상으로 난이도와 변별도 1 지수 간 상관은 -.370 으로 문항 난이도와 변별력 간 관련성이 낮은 것으로 나타남
- 난이도와 변별도 2 지수 간 상관은 .009 로 문항 난이도와 변별력 간 관련성이 없는 것으로 나타남

4. 신뢰도 분석

과목명	문항수	제83회	제83회	제84회	제85회	제86회
전체	320	.928	.928	.937	.941	**.949**
의학총론	60	.614	.614	.700	.729	**.749**
의학각론	240	.919	.919	.926	.929	**.939**
보건의약관계법규	20	.296	.296	.298	.393	**.429**

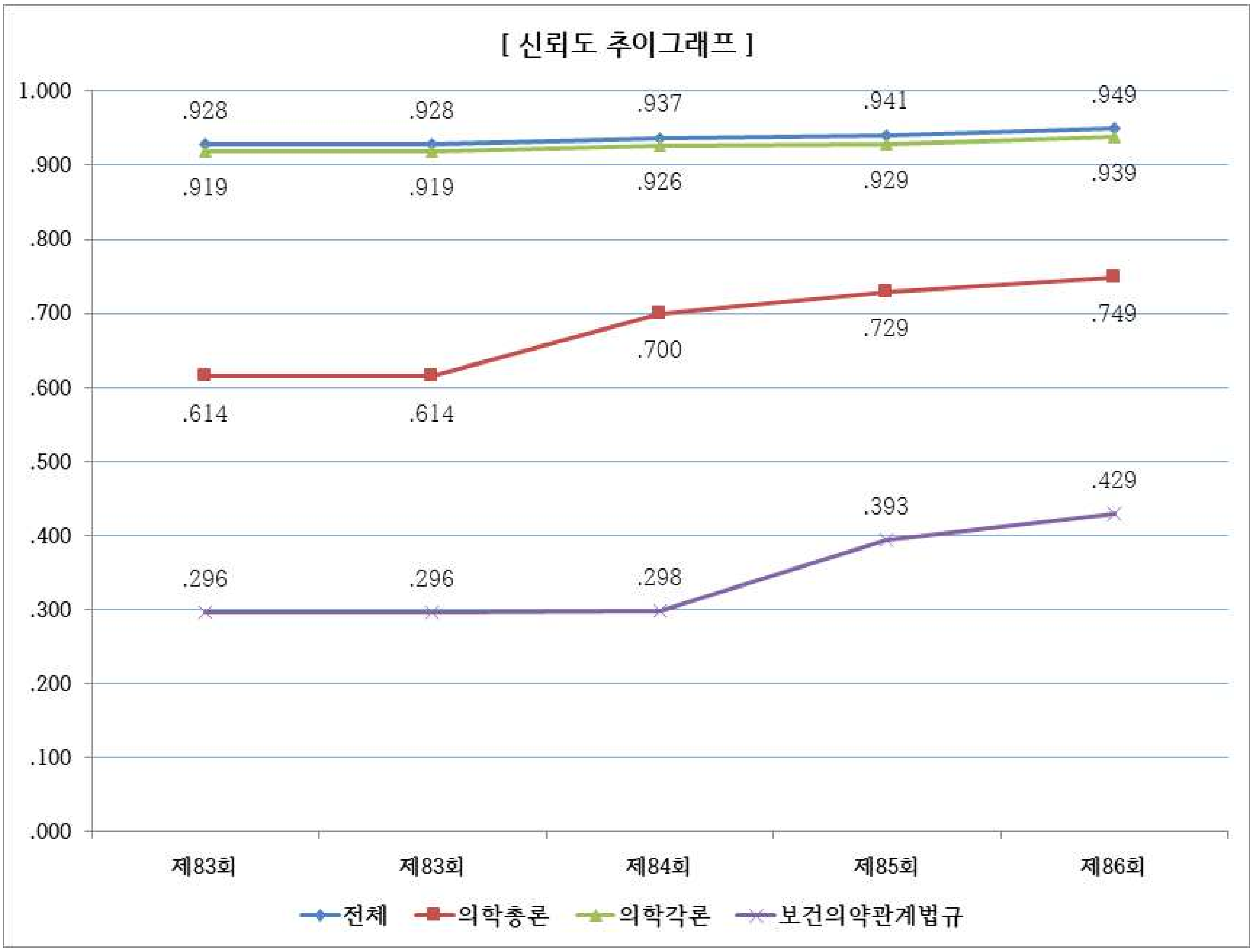

해석

- 제 86 회 의사 국가시험 전체 및 의학총론 과목, 의학각론 과목은 문항 모두 일관되게 해당 영역을 측정하고 있는 것으로 나타났으며, 보건의약관계법규 과목은 앞선 과목 대비 문항이 일관되게 해당 영역을 측정하고 있지 못한 것으로 나타남
- 전회 대비 신뢰도는 전체문항과 의학총론 과목, 의학각론 과목, 보건의약관계법규 과목이 각각 .008, .020, .010, .036 증가함

○ 분석결과 관련 문의 : 한국보건의료인국가시험원 연구개발본부 정보경 전임연구원
Tel : 02-2087-8957, FAX : 02-2087-8885
E-mail : luckys1004@kuksiwon.or.kr
