# Supplementary material for: Item difficulty index, discrimination index, and reliability of the 26 health professions licensing examinations in 2022, Korea: a psychometric study
Source: J Educ Eval Health Prof. 2023 Nov 22;20:31. doi: 10.3352/jeehp.2023.20.31 (PMC11959405; doi:10.3352/jeehp.2023.20.31)
Supplement: Supplementary file 1 — Supplement 1. Item analysis results of 26 health professions licensing examinations administered during late 2022 and early 2023. [file jeehp-20-31_Suppl1.zip › 2022│Γ╡╡ ╟╧╣▌▒Γ ░ú╚ú┴╢╣1⁄2╗τ ▒╣░í╜├╟Φ ║╨╝«░ß░·.pdf]

2022년도 하반기
간호조무사 국가시험
문항분석 결과

국민이 신뢰하고 감동하는 시험평가기관
한국보건의료인국가시험원
KOREA HEALTH PERSONNEL LICENSING EXAMINATION INSTITUTE

일반 용어 정의

□ **평균**

- 집단에서의 대표적 경향 값으로 전체 값을 더하여 총 응시자로 나눈 값

□ **표준편차**

- 평균과 각 점수의 차이인 편차들의 평균으로 점수가 흩어져 분포되어 있는 정도

□ **추정난이도**

- 문항개발자가 예측한 정답률

□ **검사이론**

- 검사와 검사를 구성하고 있는 문항의 양호도를 분석 및 평가하는 방법을 정의한 이론체계
- 대표적으로 고전검사이론과 문항반응이론이 있음

고전검사이론 용어 정의

□ 고전검사이론(Classical Test Theory; CTT)

- 검사의 질을 분석하는 검사이론 중 한 가지로 19세기 말부터 전개되어 현재까지 주로 사용되고 있는 검사이론임
- 고전검사이론에 의한 문항과 응시자 능력 추정치는 다음과 같음

○ **문항난이도**

· 검사 문항의 쉽고 어려운 정도를 나타내는 지수

· 난이도 지수는 총 반응 수에 대한 정답 반응 수의 비율로 문항의 정답률임

· 문항난이도는 0~100까지의 값을 가짐

· 난이도 값이 큰 경우, 쉬운 문항으로 '난이도가 낮다'라고 해석하며, 난이도 값이 작은 경우, 어려운 문항으로 '난이도가 높다'라고 해석함

○ **문항변별도**

· 각 문항이 응시자의 능력 수준을 변별할 수 있는 정도를 나타내는 지수

· 문항변별도는 -1~+1까지의 값을 가지며, 1에 가까울수록 변별력 크다고 해석함

· 일반적으로 문항변별도가 0.3 이상이면 우수한 문항으로 평가함

· 구하는 방식에는 '상하위집단 구분법', '문항-총점 상관계수' 등이 있음

1) **변별도 1(상하위구분법):** 상위 27%와 하위 27% 집단의 난이도 차이를 구하는 방식

2) **변별도 2(상관계수법):** 문항-총점과의 상관계수로 구하는 방식

○ **신뢰도**

· 시험이 평가하고자 하는 것을 일관성 있게 측정하는가로 시험이 오차없이 정확하게 측정한 정도를 의미함

· 국시원에서는 문항의 내적일관성(Cronbach α)으로 신뢰도를 추정하며 1에 가까울수록 신뢰도가 높다고 해석함

목 차

I. 시행 결과 ········ 6
1. 시험 현황 ········ 7
1) 시험명 ········ 7
2) 시험시행일 ········ 7
3) 응시현황 ········ 7
4) 과목별 문항 수, 배점 및 과락 점수 ········ 7
2. 합격률과 평균성적 ········ 7
1) 합격 및 불합격 현황 ········ 7
2) 과목별 과락자수 내역 ········ 7
3) 전회 대비 합격률과 평균성적 ········ 8

II. 문항분석 결과 ········ 9
1. 성적 ········ 10
1) 전체 성적분포도 ········ 10
2) 과목별 성적분포도 ········ 11
2. 난이도와 변별도 ········ 12
1) 전체 난이도와 변별도 ········ 12
2) 과목별 난이도와 변별도 ········ 15
3) 지식수준별 난이도와 변별도 ········ 26
4) 자료유형별 난이도와 변별도 ········ 34
3. 난이도와 변별도 간 산포도 ········ 40
1) 전체 난이도와 변별도 간 산포도 ········ 40
2) 과목별 난이도와 변별도 간 산포도 ········ 40
4. 신뢰도 분석 ········ 43

I. 시행 결과

1. 시험 현황

1) 시험명: 2022년도 하반기 간호조무사 자격시험
2) 시험시행일: 2022년 9월 24일
3) 응시현황

응시대상자수	결시자수	부정행위자수	응시자 준수사항 위반자 수		응시자수 (%)
			휴대폰 소지등	신분증 미지참	
20,774	988	-	-	-	17,840 (85.9)

4) 과목별 문항 수, 배점 및 과락 점수

교 시	과 목 명	문제 수	배점	총점	합격자 점수기준	
					과락기준	총점 합격기준
1교시	1. 기초간호학 개요	35	1	35	14	60점 이상
2교시	2. 보건간호학 개요	15	1	15	6	
3교시	3. 공중보건학개론	20	1	20	8	
4교시	4. 실기	30	1	30	12	
계		100		100		

2. 합격률과 평균성적

1) 합격 및 불합격 현황

합격자수 (%)	불합격자수(%)				채점보류자수
	평락	과락	기권	계	
14,812(83.0)	2,954(16.6)	74(0.4)	-	3,028(17.0)	1,946

2) 과목별 과락자수 내역

과목명 / 과락자수	기초간호학 개요	보건간호학 개요	공중보건학 개론	실기
과목별 과락자 수	-	57	8	9
2과목 과락자 수	-			

3) 전회 대비 합격률과 평균성적

회차	년도	합격률(%)	평균성적	표준편차	백분율 환산점수
2020년 하반기	2020	91.9	81.8	12.8	81.8
2021년 상반기	2021	85.7	76.4	14.1	76.4
2021년 하반기	2021	87.0	77.6	13.8	77.6
**2022년 상반기**	**2022**	**82.4**	**73.9**	**14.1**	**73.9**
2022년도 하반기	2022	83.0	76.2	14.2	76.2

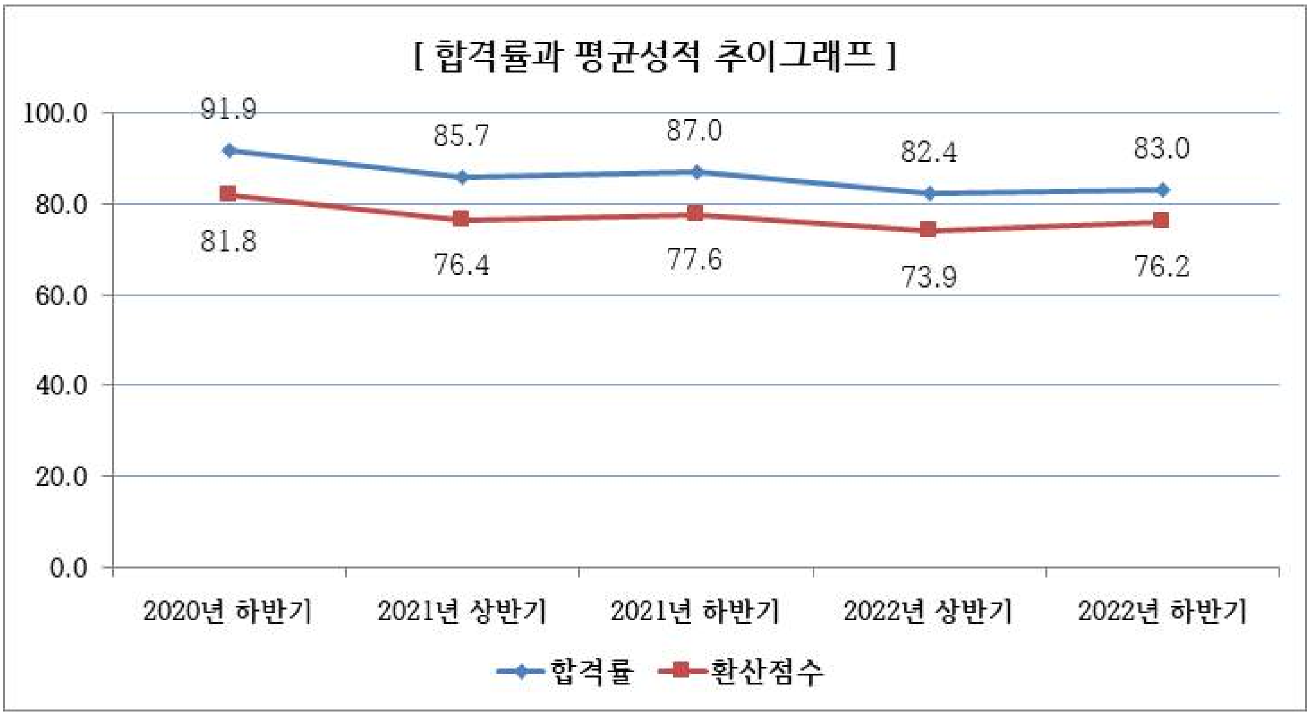

해석

- 전년 대비 합격률은 0.6 증가하였으며, 백분율 환산점수는 2.3 점 증가함
- 표준편차는 0.1 증가함

Ⅱ. 문항분석 결과

1. 성적

1) 전체 성적분포도

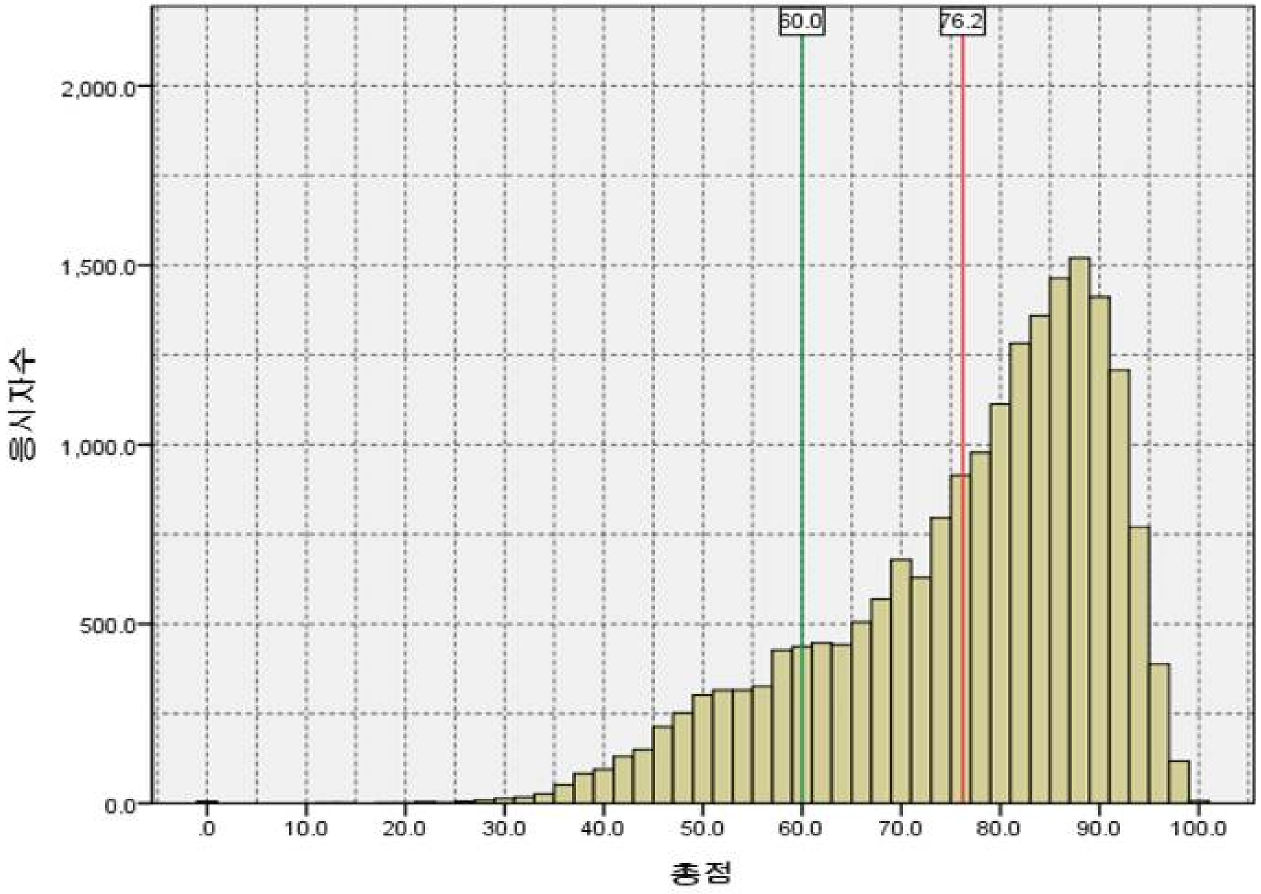

응시자	총점	합격선	평균성적	표준편차
19,784※	100	60	76.2	14.2

※ 19,784명은 응시자(17,840명)에 채점보류자(1,946명)를 포함하고 부정행위자 2명을 제외한 숫자임

2) 과목별 성적분포도

가) 기초간호학개요

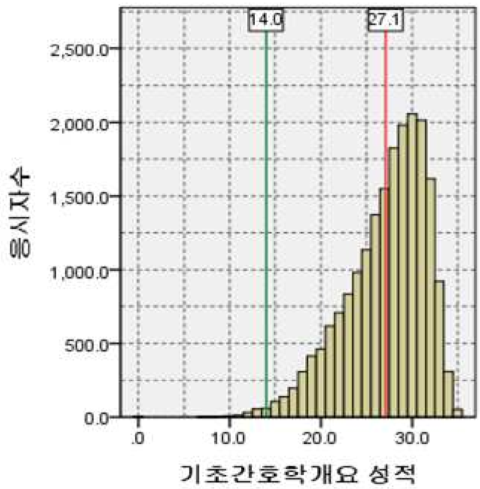

총점	과락선	평균성적	표준편차
35	14	27.1	4.4

나) 보건간호학개요

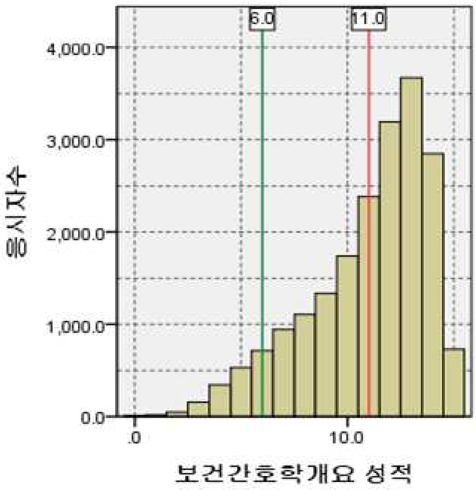

총점	과락선	평균성적	표준편차
15	6	11.0	2.8

다) 공중보건학개론

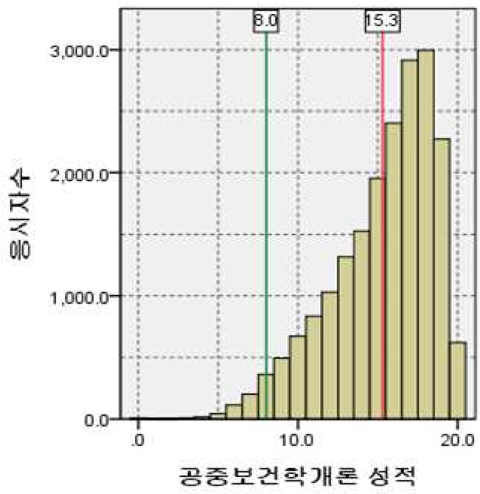

총점	과락선	평균성적	표준편차
20	8	15.3	3.2

실기

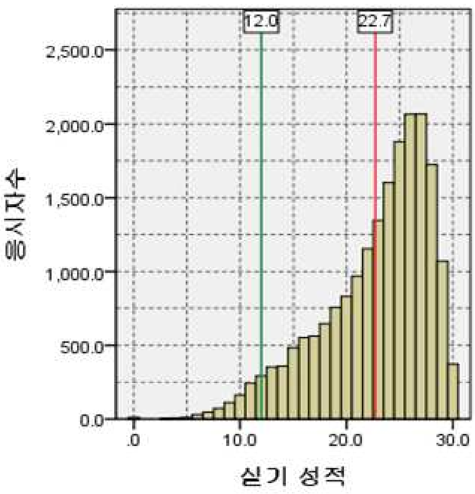

총점	과락선	평균성적	표준편차
30	12	22.7	5.1

2. 난이도와 변별도

1) 전체 난이도와 변별도

가) 전회 대비 전체 난이도와 변별도

회차	난이도		변별도1		변별도2	
	평균	표준편차	평균	표준편차	평균	표준편차
2020년 하반기	81.8	14.8	0.30	0.17	0.36	0.13
2021년 상반기	76.4	17.1	0.33	0.16	0.37	0.12
2021년 하반기	77.6	16.0	0.33	0.18	0.36	0.13
2022년 상반기	73.9	20.3	0.34	0.18	0.36	0.13
**2022년 하반기**	**76.2**	**18.3**	**0.34**	**0.16**	**0.37**	**0.13**

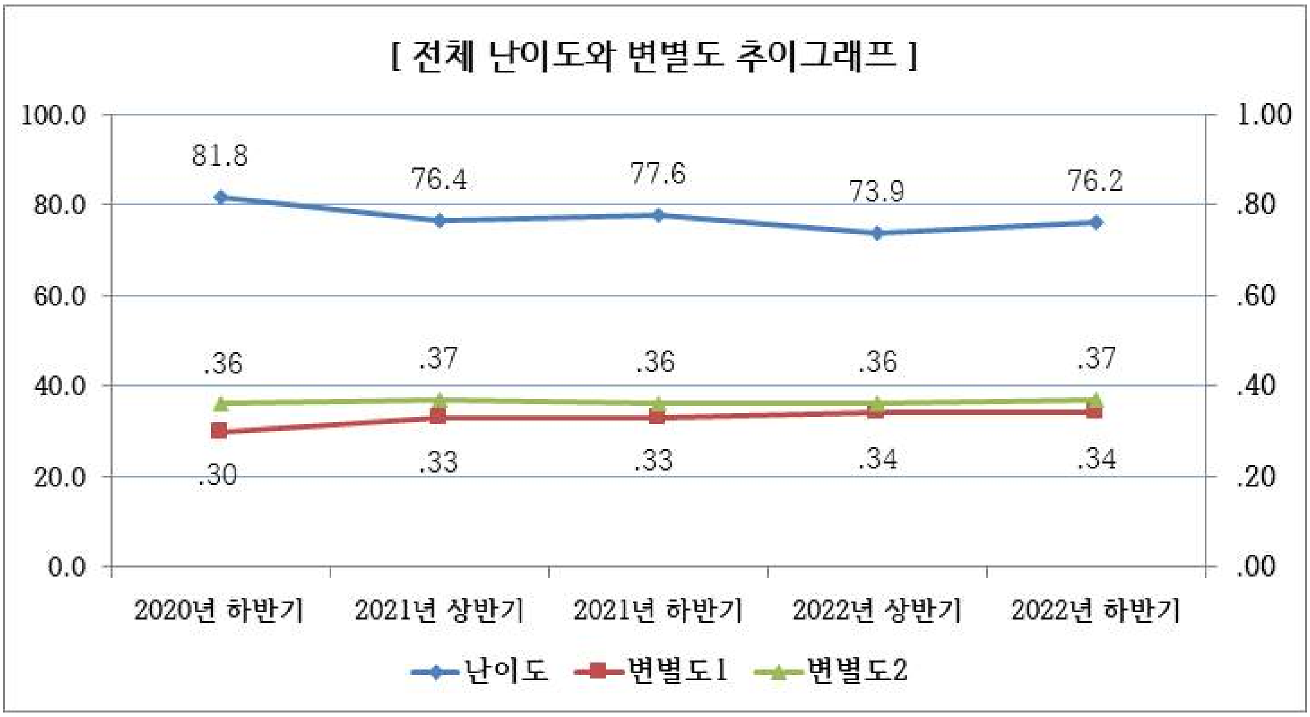

해석

- 전년 대비 난이도 지수는 2.3 증가함
- 변별도 1 지수는 변화 없었으며, 변별도 2 지수는 0.01 증가함

나) 전체 난이도와 변별도 분포도 및 비율분석

(1) 전체 난이도 분포도 및 비율분석

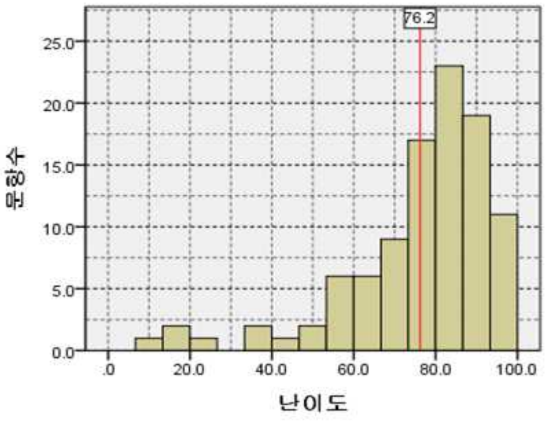

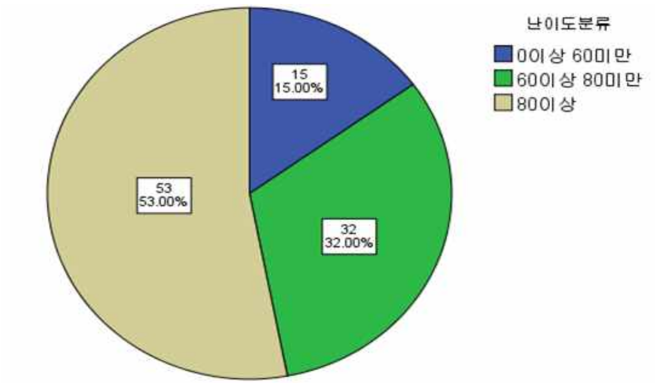

총점	난이도	표준편차
100	76.2	18.3

난이도	문항수	비율(%)
0~60미만	15	15.0
60~80미만	32	32.0
80~100	53	53.0
전체	100	100.0

(2) 전체 변별도1 분포도 및 비율분석

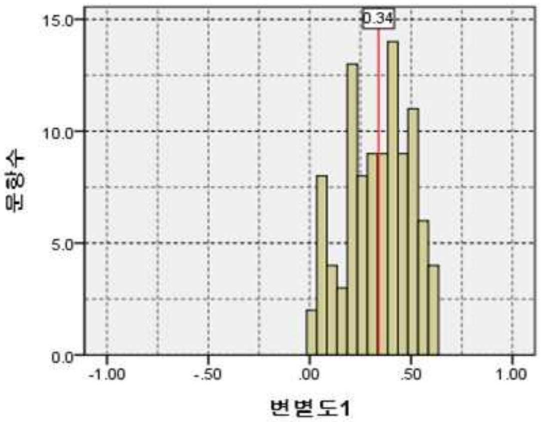

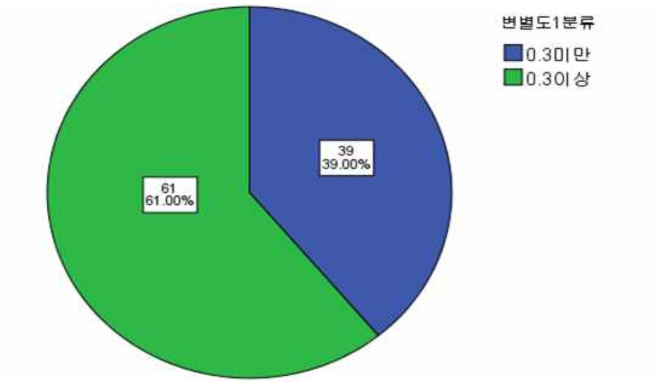

총점	변별도1	표준편차
100	.34	.16

변별도1	문항수	비율(%)
0.3미만	39	39.0
0.3이상	61	61.0
전체	100	100.0

(3) 전체 변별도2 분포도 및 비율분석

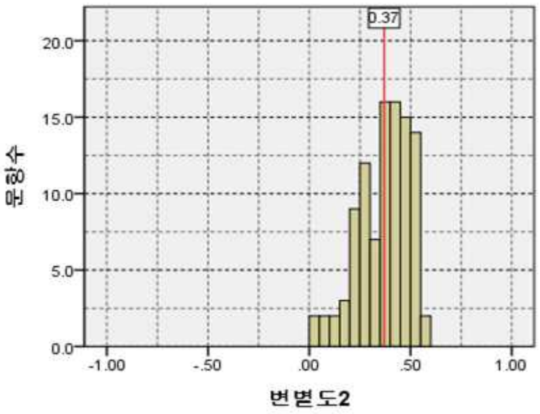

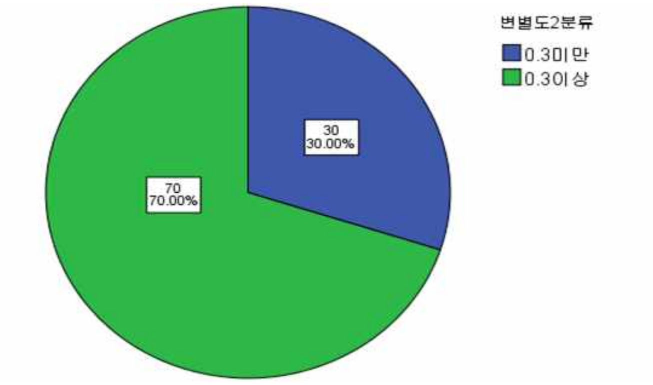

총점	변별도2	표준편차	변별도2	문항수	비율(%)
100	.37	.13	0.3미만	30	30.0
			0.3이상	70	70.0
			전체	100	100.0

해석

- 난이도 지수가 80 에서 100 사이인 문항이 전체 100 문항 중 53 문항으로 가장 많았으며, 차례로 60 이상 80 미만인 문항이 32 문항, 60 미만인 문항이 15 문항인 것으로 나타남
- 변별도 1 지수를 기준으로 분류하였을 때, 0.3 미만인 문항이 39 문항으로 0.3 이상인 문항이 61 문항인 것에 비해 더 적게 나타남
- 변별도 2 지수를 기준으로 분류하였을 때, 0.3 미만인 문항이 30 문항으로 0.3 이상인 문항이 70 문항인 것에 비해 더 적게 나타남

2) 과목별 난이도와 변별도

가) 전회 대비 과목별 난이도와 변별도

(1) 전회 대비 기초간호학개요 난이도와 변별도

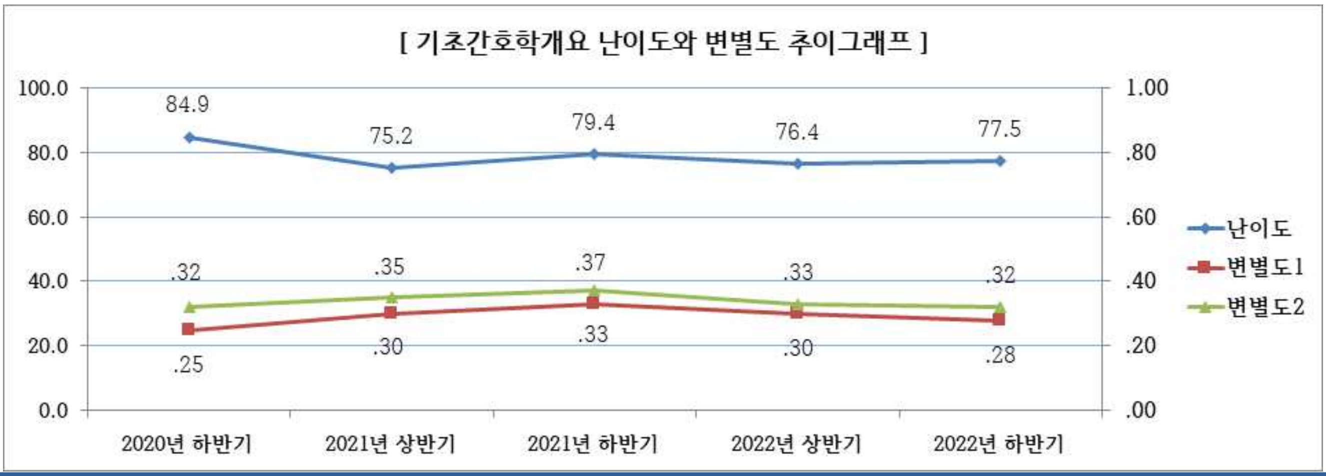

회차	난이도		변별도1		변별도2	
	평균	표준편차	평균	표준편차	평균	표준편차
2020년 하반기	84.9	14.4	0.25	0.17	0.32	0.11
2021년 상반기	75.2	20.6	0.30	0.15	0.35	0.10
2021년 하반기	79.4	16.1	0.33	0.20	0.37	0.11
2022년 상반기	76.4	22.0	0.30	0.19	0.33	0.12
**2022년 하반기**	**77.5**	**21.3**	**0.28**	**0.15**	**0.32**	**0.12**

(2) 전회 대비 보건간호학개요 난이도와 변별도

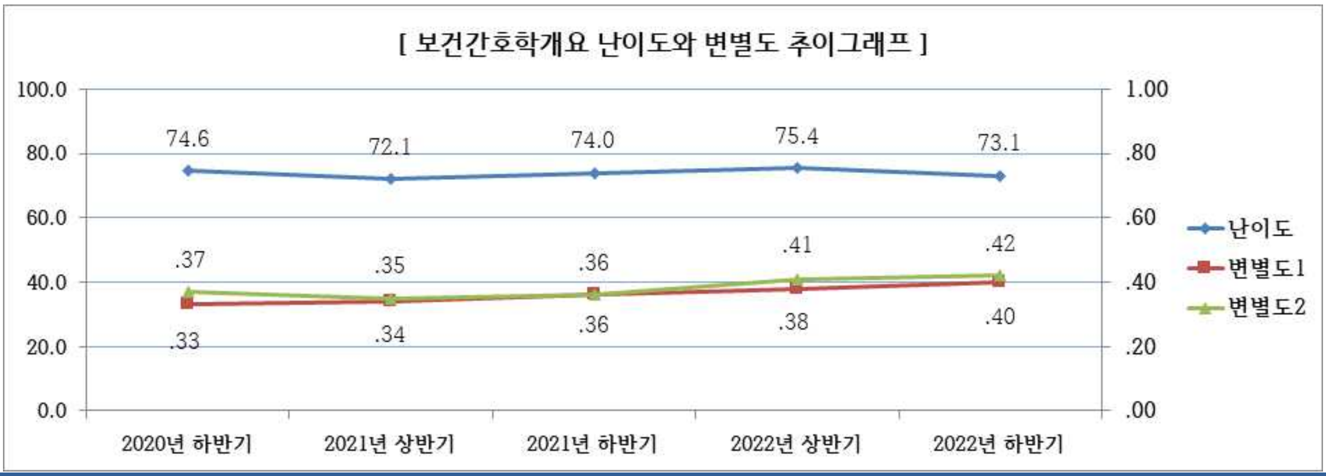

회차	난이도		변별도1		변별도2	
	평균	표준편차	평균	표준편차	평균	표준편차
2020년 하반기	74.6	20.0	0.33	0.17	0.37	0.13
2021년 상반기	72.1	20.6	0.34	0.15	0.35	0.11
2021년 하반기	74.0	11.7	0.36	0.18	0.36	0.15
2022년 상반기	75.4	18.1	0.38	0.16	0.41	0.10
**2022년 하반기**	**73.1**	**19.8**	**0.40**	**0.16**	**0.42**	**0.14**

(3) 전회 대비 공중보건학개론 난이도와 변별도

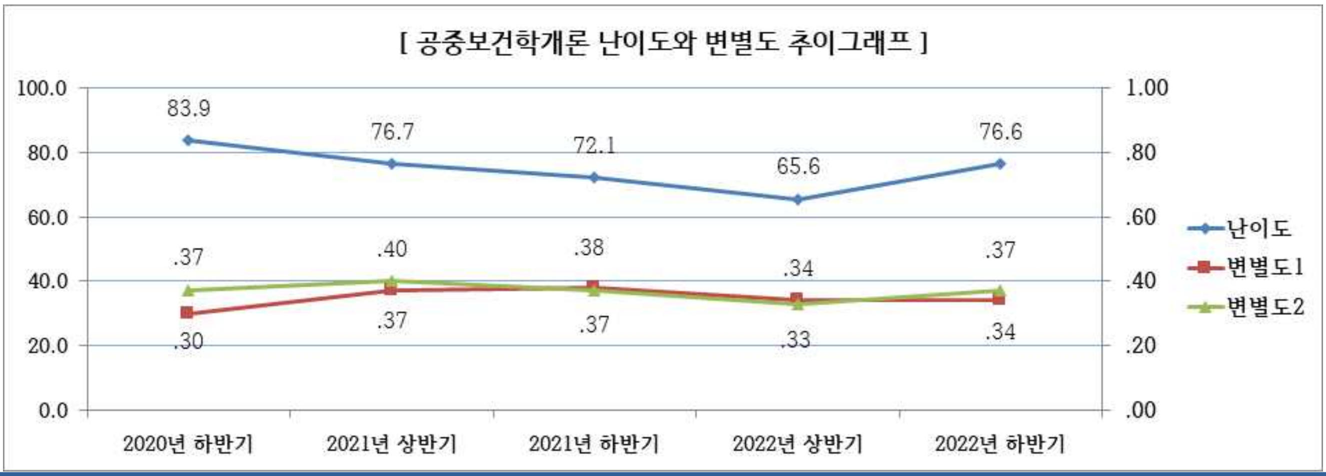

회차	난이도		변별도1		변별도2	
	평균	표준편차	평균	표준편차	평균	표준편차
2020년 하반기	83.9	11.3	0.30	0.18	0.37	0.13
2021년 상반기	76.7	13.4	0.37	0.18	0.40	0.14
2021년 하반기	72.1	12.4	0.38	0.17	0.37	0.14
2022년 상반기	65.6	22.1	0.34	0.18	0.33	0.14
**2022년 하반기**	**76.6**	**17.1**	**0.34**	**0.17**	**0.37**	**0.13**

(4) 전회 대비 실기 난이도와 변별도

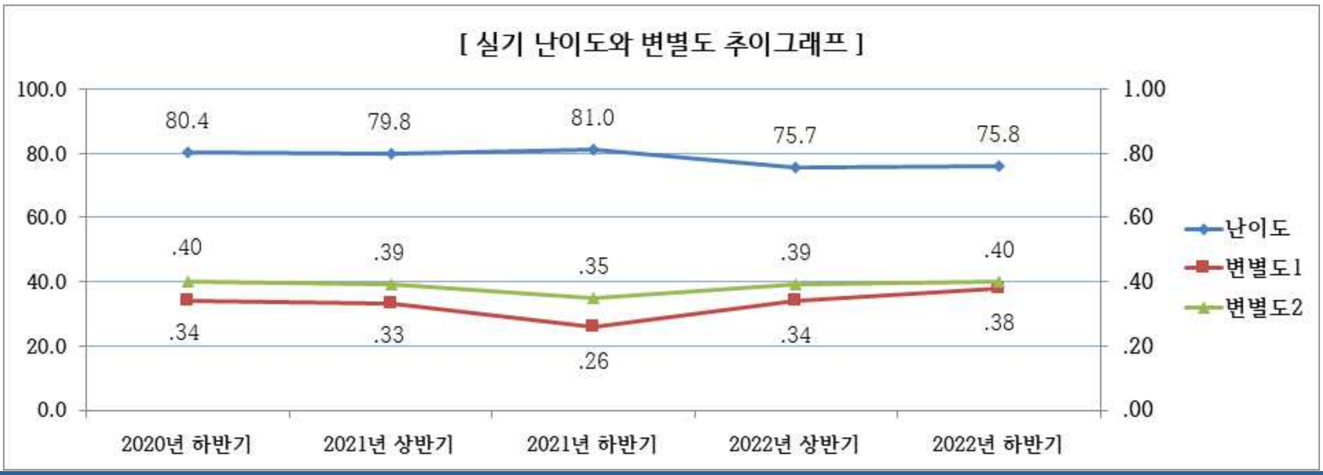

회차	난이도		변별도1		변별도2	
	평균	표준편차	평균	표준편차	평균	표준편차
2020년 하반기	80.4	13.5	0.34	0.17	0.40	0.13
2021년 상반기	79.8	12.6	0.33	0.16	0.39	0.14
2021년 하반기	81.0	19.1	0.26	0.16	0.35	0.13
2022년 상반기	75.7	17.6	0.34	0.18	0.39	0.13
**2022년 하반기**	**75.8**	**14.9**	**0.38**	**0.14**	**0.40**	**0.11**

해석

- 전회 대비 기초간호학개요, 공중보건학개론, 실기 과목의 난이도 지수는 각각 1.1, 11.0, 0.1 증가하였으며, 보건간호학개론 과목의 난이도 지수는 2.30 감소함
- 기초간호학개요 과목의 변별도 1 지수 및 변별도 2 지수는 각각 .02, .01 감소함
- 보건간호학개요 과목의 변별도 1 지수 및 변별도 2 지수는 각각 .02, .01 증가함
- 공중보건학개론 과목의 변별도 1 지수는 변화 없었으며, 변별도 2 지수는 .04 증가함
- 실기 과목의 변별도 1 지수 및 변별도 2 지수는 각각 .04, .01 증가함

나) 과목별 난이도와 변별도 분포도 및 비율분석

(1) 기초간호학개요 난이도와 변별도 분포도 및 비율분석

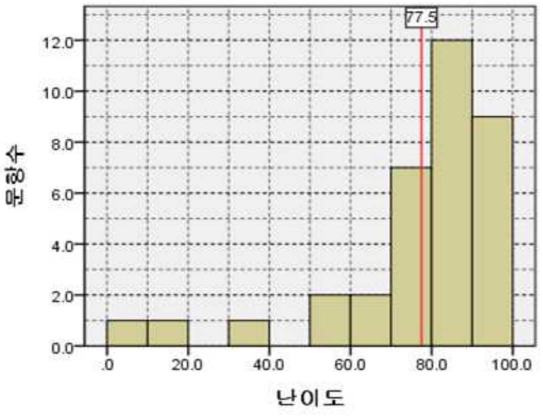

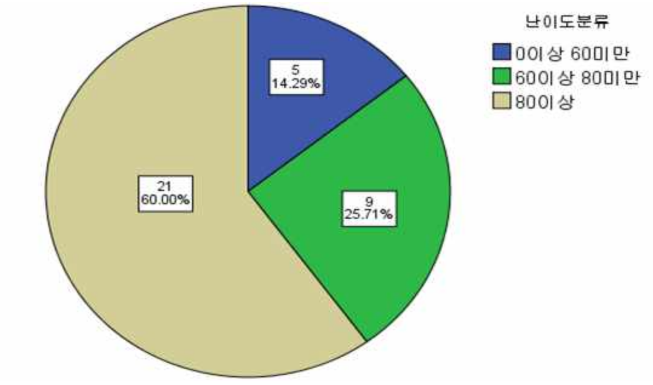

총점	난이도	표준편차	난이도	문항수	비율(%)
35	77.5	21.3	0~60미만	5	14.3
			60~80미만	9	25.7
			80~100	21	60.0
			전체	35	100.0

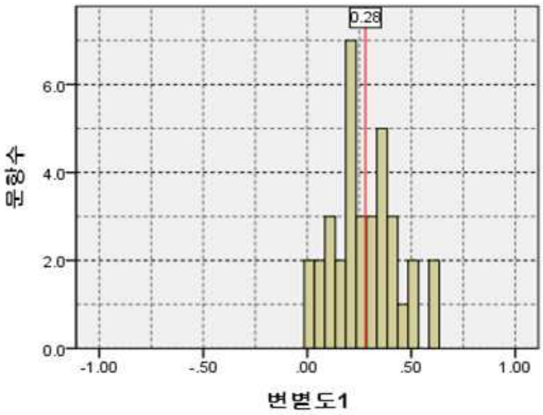

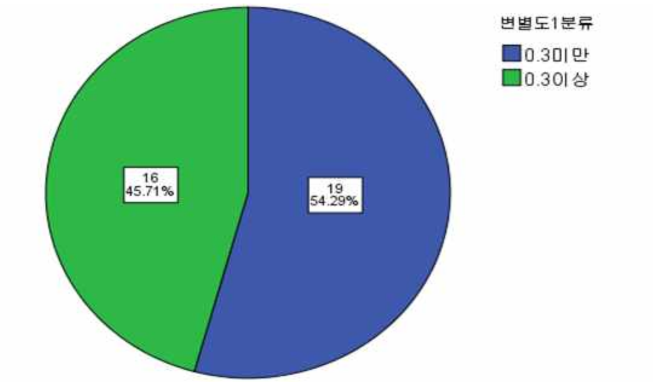

총점	변별도1	표준편차	변별도1	문항수	비율(%)
35	.28	.15	0.3미만	19	54.3
			0.3이상	16	45.7
			전체	35	100.0

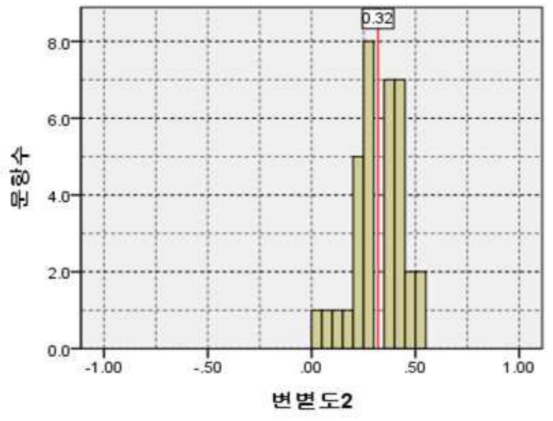

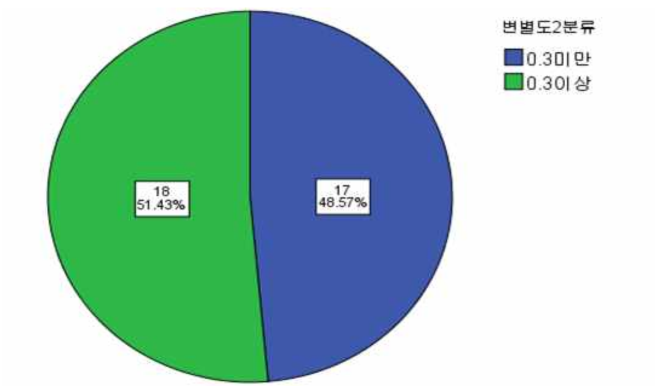

총점	변별도2	표준편차
35	.32	.12

변별도2	문항수	비율(%)
0.3미만	17	48.6
0.3이상	18	51.4
전체	35	100.0

해석

- 기초간호학개요 과목에서 난이도 지수가 80에서 100 사이인 문항이 전체 35문항 중 21문항으로 가장 많았으며, 다음으로 60이상 80미만인 문항이 9문항, 60미만인 문항이 5문항 인 것으로 나타남
- 변별도1 지수를 기준으로 분류하였을 때, 0.3미만인 문항이 19문항으로 0.3이상인 문항이 16문항인 것에 비해 더 많게 나타남
- 변별도2 지수를 기준으로 분류하였을 때, 0.3미만인 문항이 17문항으로 0.3이상인 문항이 18문항인 것에 비해 더 적게 나타남

(2) 보건간호학개요 난이도와 변별도 분포도 및 비율분석

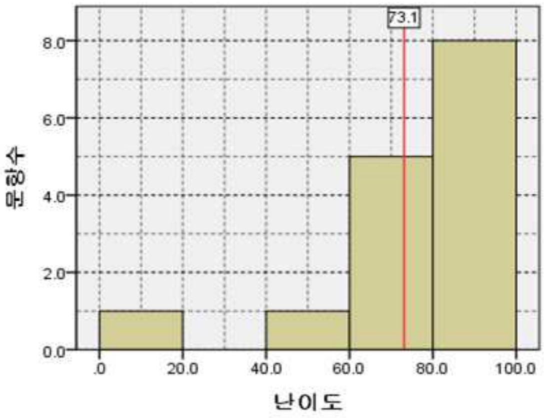

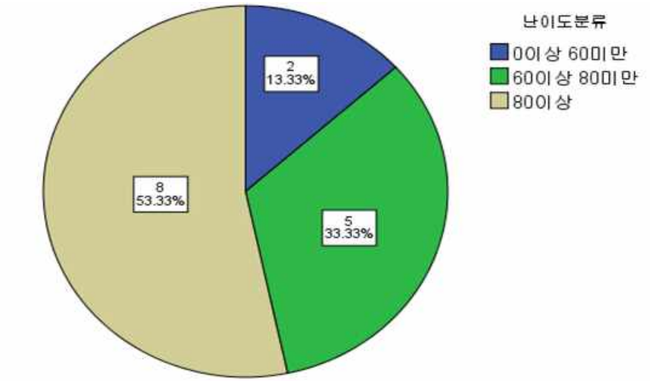

총점	난이도	표준편차
15	73.1	19.8

난이도	문항수	비율(%)
0~60미만	2	13.3
60~80미만	5	33.3
80~100	8	53.3
전체	15	100.0

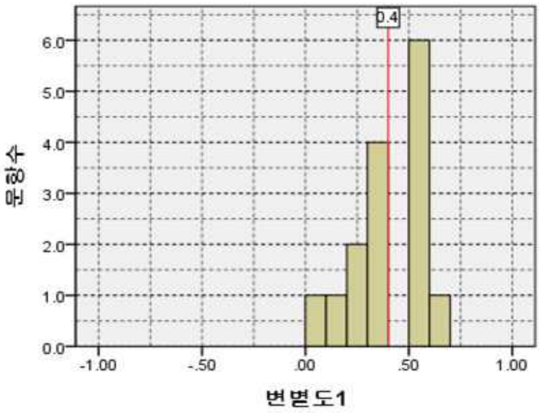

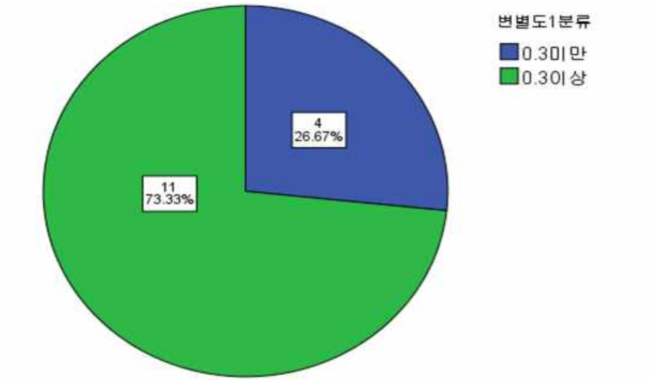

총점	변별도1	표준편차
15	.40	.16

변별도1	문항수	비율(%)
0.3미만	4	26.7
0.3이상	11	73.3
전체	15	100.0

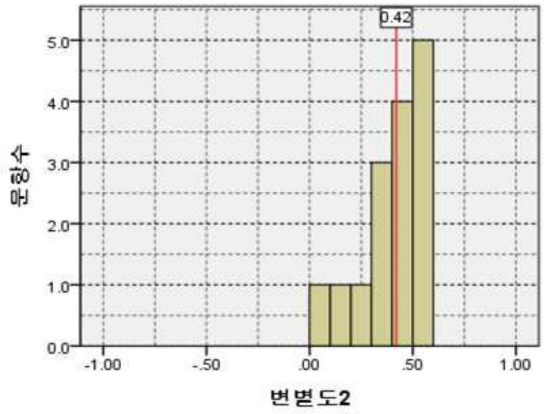

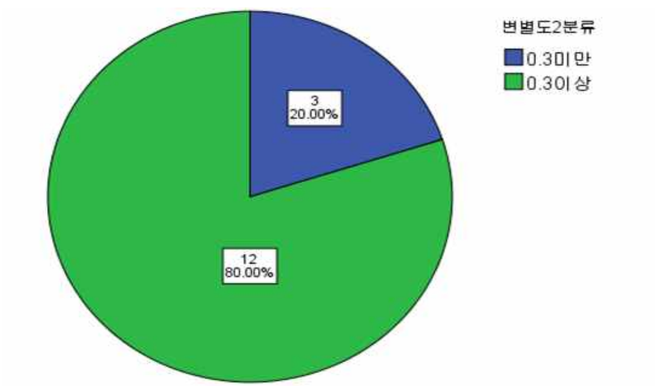

총점	변별도2	표준편차
15	.42	.14

변별도2	문항수	비율(%)
0.3미만	3	20.0
0.3이상	12	80.0
전체	15	100.0

해석

- 보건간호학개요 과목에서 난이도 지수가 80 에서 100 사이인 문항이 전체 15 문항 중 8 문항이었으며, 다음으로 60 이상 80 미만인 문항이 5 문항, 60 미만인 문항이 2 문항 인 것으로 나타남
- 변별도 1 지수를 기준으로 분류하였을 때, 0.3 미만인 문항이 4 문항으로 0.3 이상인 문항이 11 문항인 것에 비해 더 적게 나타남
- 변별도 2 지수를 기준으로 분류하였을 때, 0.3 미만인 문항이 3 문항으로 0.3 이상인 문항이 12 문항인 것에 비해 더 적게 나타남

(3) 공중보건학개론 난이도와 변별도 분포도 및 비율분석

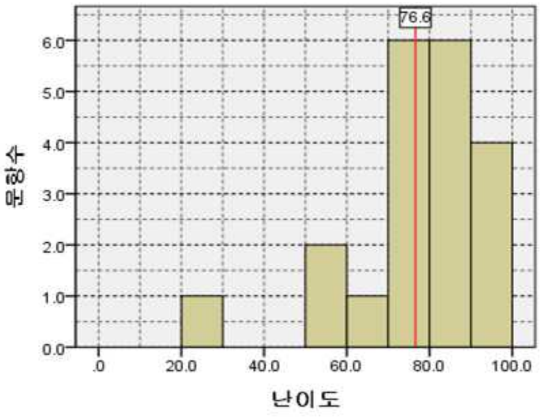

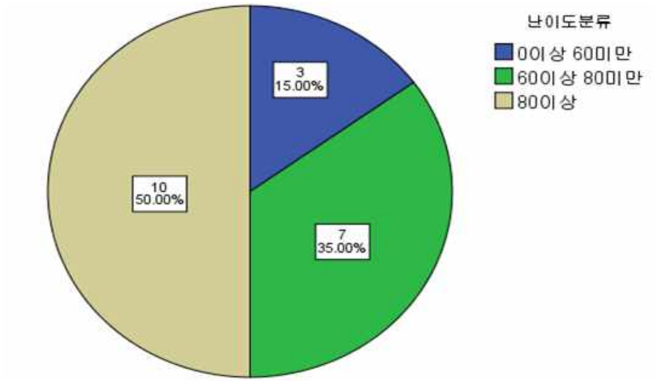

총점	난이도	표준편차
20	76.6	17.1

난이도	문항수	비율(%)
0~60미만	3	15.0
60~80미만	7	35.0
80~100	10	50.0
전체	20	100.0

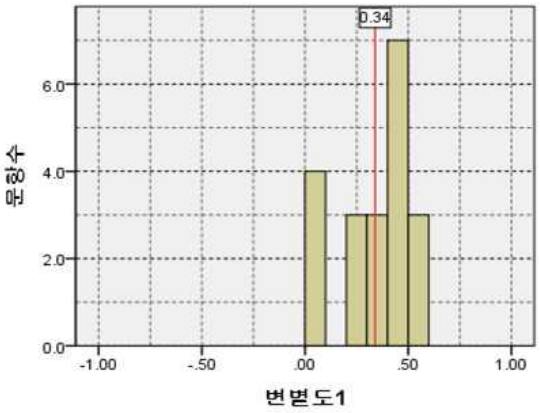

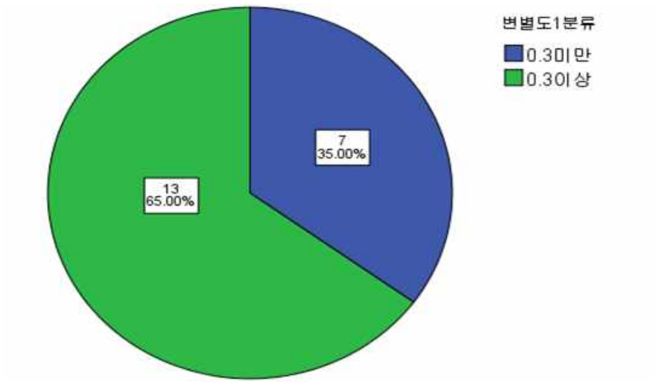

총점	변별도1	표준편차
20	.34	.17

변별도1	문항수	비율(%)
0.3미만	7	35.0
0.3이상	13	65.0
전체	20	100.0

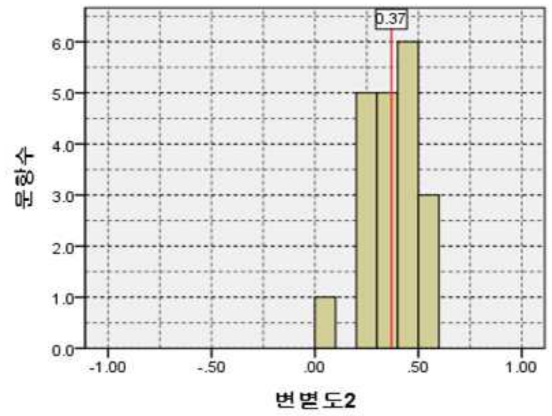

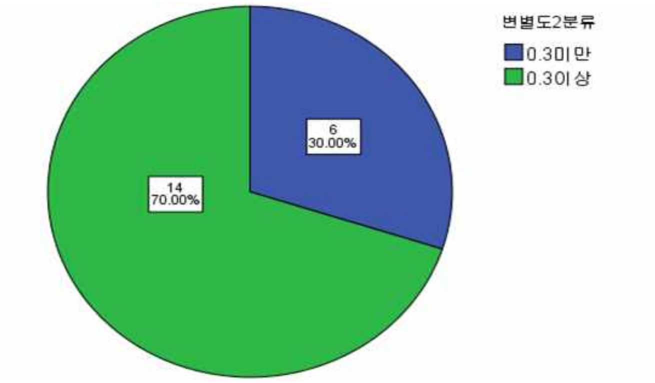

총점	변별도2	표준편차
20	.37	.13

변별도2	문항수	비율(%)
0.3미만	6	30.0
0.3이상	14	70.0
전체	20	100.0

해석

- 공중보건학개론 과목에서 난이도 지수가 80 에서 100 사이인 문항이 전체 20 문항 중 10 문항으로 나타났으며, 다음으로 60 에서 80 사이인 문항이 7 문항, 60 미만인 문항이 3 문항으로 나타남
- 변별도 1 지수를 기준으로 분류하였을 때, 0.3 미만인 문항이 7 문항으로 0.3 이상인 문항이 13 문항인 것에 비해 더 적게 나타남
- 변별도 2 지수를 기준으로 분류하였을 때, 0.3 미만인 문항이 6 문항으로 0.3 이상인 문항이 14 문항인 것에 비해 더 적게 나타남

(4) 실기 난이도와 변별도 분포도 및 비율분석

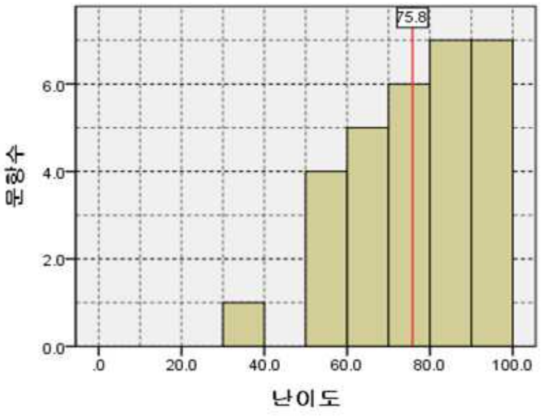

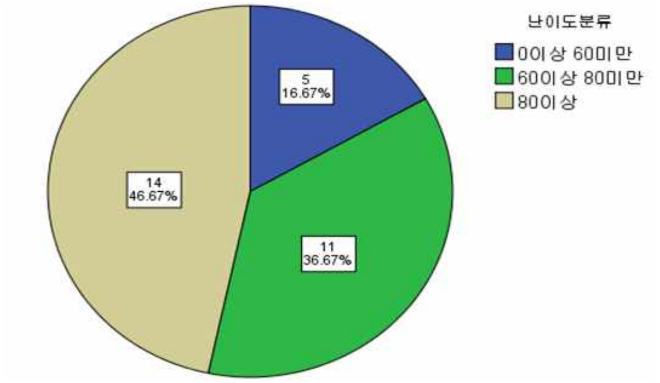

총점	난이도	표준편차
30	75.8	14.9

난이도	문항수	비율(%)
0~60미만	5	16.7
60~80미만	11	36.7
80~100	14	46.7
전체	30	100.0

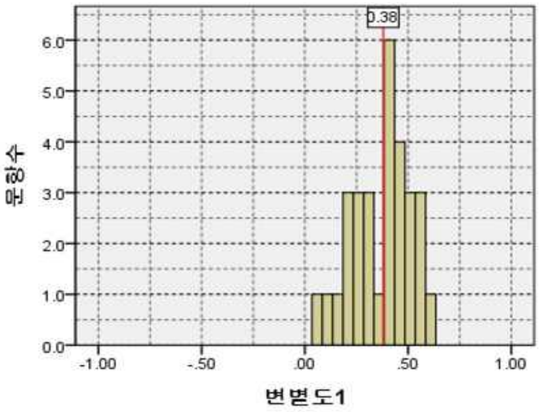

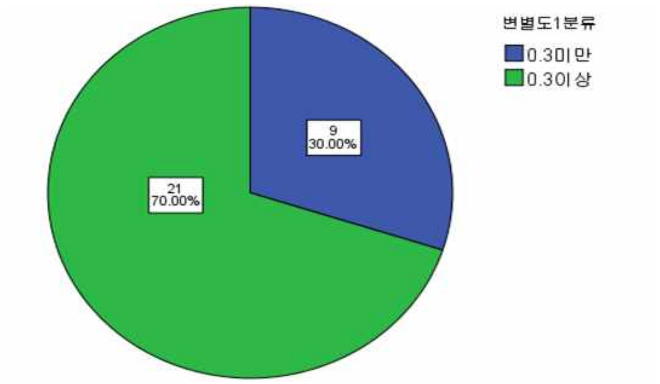

총점	변별도1	표준편차
30	.38	.14

변별도1	문항수	비율(%)
0.3미만	9	30.0
0.3이상	21	70.0
전체	30	100.0

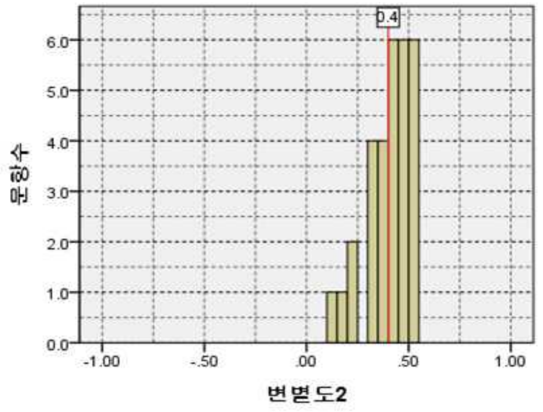

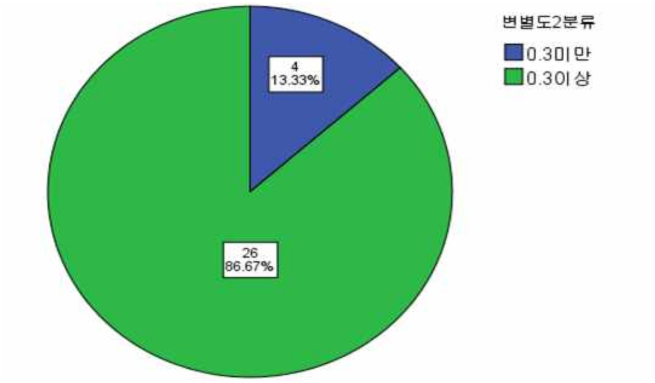

총점	변별도2	표준편차
30	.40	.11

변별도2	문항수	비율(%)
0.3미만	4	13.3
0.3이상	26	86.7
전체	30	100.0

해석

- 실기 과목에서 난이도 지수가 60 이상 80 미만인 문항이 전체 30 문항 중 14 문항으로 가장 많았으며, 다음으로 80 에서 100 사이인 문항 11 문항, 60 미만인 문항이 5 문항 인 것으로 나타남
- 변별도 1 지수를 기준으로 분류하였을 때, 0.3 미만인 문항이 9 문항으로 0.3 이상인 문항이 21 문항인 것에 비해 적게 나타남
- 변별도 2 지수를 기준으로 분류하였을 때, 0.3 미만인 문항이 4 문항으로 0.3 이상인 문항이 26 문항인 것에 비해 더 적게 나타남

3) 지식수준별 난이도와 변별도

가) 전회 대비 지식수준별 난이도와 변별도

(1) 전회 대비 암기형 난이도와 변별도

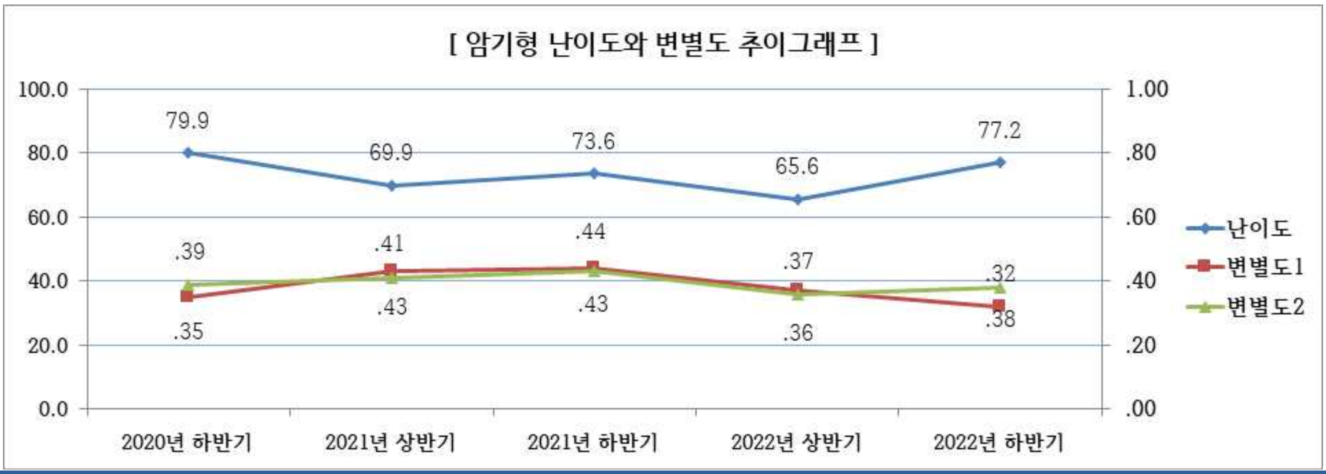

회차	난이도		변별도1		변별도2	
	평균	표준편차	평균	표준편차	평균	표준편차
2021년 상반기	69.9	17.2	0.43	0.15	0.41	0.11
2021년 하반기	73.6	14.5	0.44	0.17	0.43	0.10
2021년 하반기	73.6	14.5	0.44	0.17	0.43	0.10
2022년 상반기	65.6	21.8	0.37	0.19	0.36	0.15
**2022년 하반기**	**77.2**	**21.5**	**0.32**	**0.16**	**0.38**	**0.14**

(2) 전회 대비 해석형 난이도와 변별도

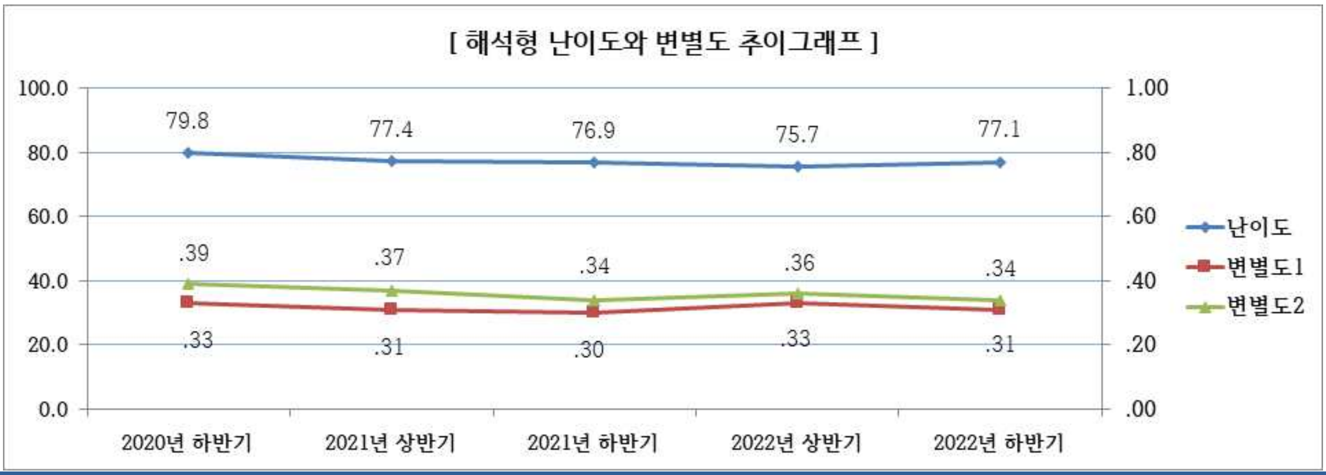

회차	난이도		변별도1		변별도2	
	평균	표준편차	평균	표준편차	평균	표준편차
2020년 하반기	79.8	16.3	0.33	0.17	0.39	0.12
2021년 상반기	77.4	16.8	0.31	0.14	0.37	0.12
2021년 하반기	76.9	17.7	0.30	0.18	0.34	0.13
2022년 상반기	75.7	19.3	0.33	0.18	0.36	0.15
**2022년 하반기**	**77.1**	**17.6**	**0.31**	**0.17**	**0.34**	**0.14**

(3) 전회 대비 해결형 난이도와 변별도

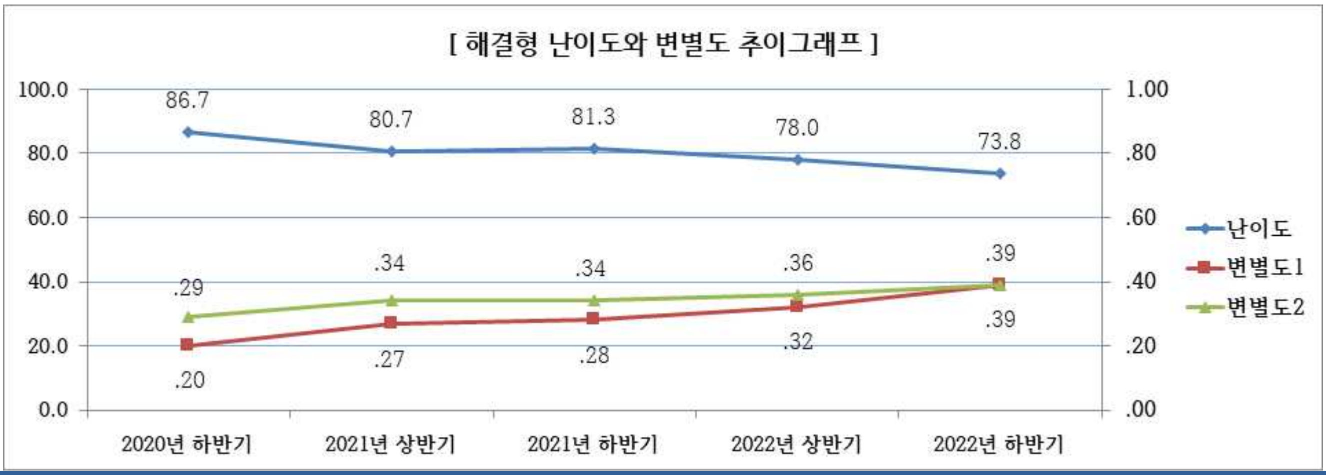

회차	난이도		변별도1		변별도2	
	평균	표준편차	평균	표준편차	평균	표준편차
2020년 하반기	86.7	13.9	0.20	0.15	0.29	0.11
2021년 상반기	80.7	16.2	0.27	0.16	0.34	0.13
2021년 하반기	81.3	14.3	0.28	0.16	0.34	0.12
2022년 상반기	78.0	19.3	0.32	0.19	0.36	0.13
**2022년 하반기**	**73.8**	**15.3**	**0.39**	**0.13**	**0.39**	**0.09**

해석

- 전회 대비 암기형, 해석형 문항의 난이도 지수는 각각 11.6, 1.4 증가하였으며, 해결형 문항의 난이도 지수는 4.2 감소함
- 암기형 문항의 변별도 1 지수는 .05 감소하였으나, 변별도 2 지수는 .02 증가함
- 해석형 문항의 변별도 1 지수와 변별도 2 지수는 각각 .02 감소함
- 해결형 문항의 변별도 1 지수와 변별도 2 지수는 각각 .07, .03 증가함

나) 지식수준별 난이도와 변별도 분포도 및 비율분석

(1) 암기형 난이도와 변별도 분포도 및 비율분석

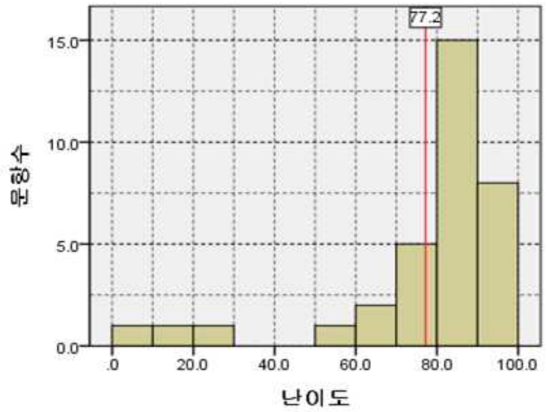

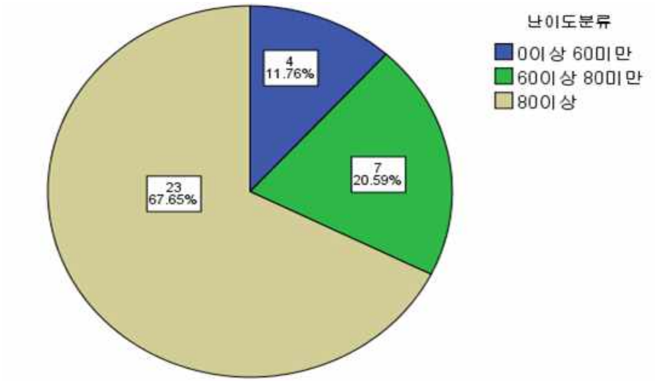

총점	난이도	표준편차
34	77.2	21.5

난이도	문항수	비율(%)
0~60미만	4	11.8
60~80미만	7	20.6
80~100	23	67.6
전체	34	100.0

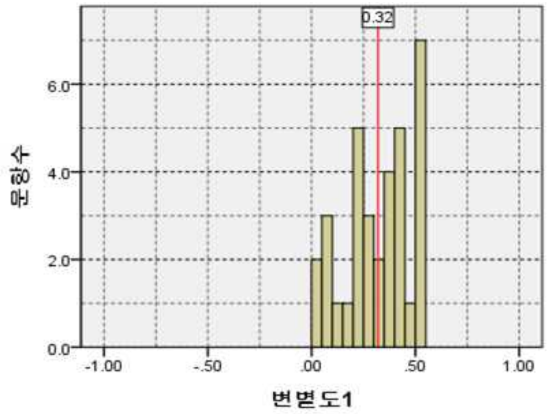

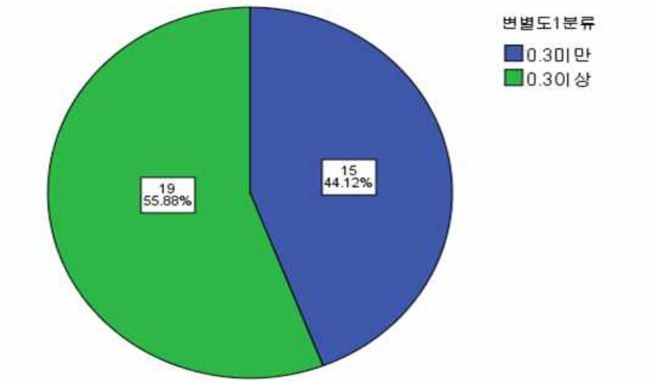

총점	변별도1	표준편차
34	.32	.16

변별도1	문항수	비율(%)
0.3미만	15	44.1
0.3이상	19	55.9
전체	34	100.0

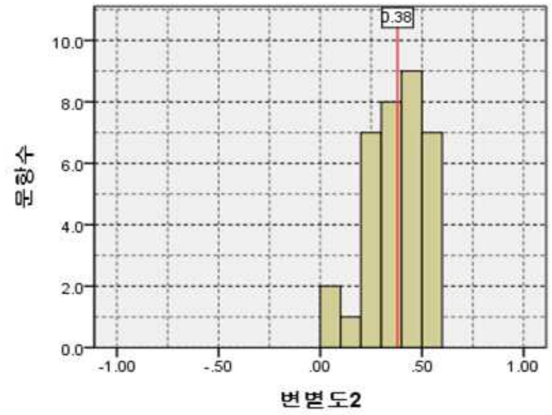

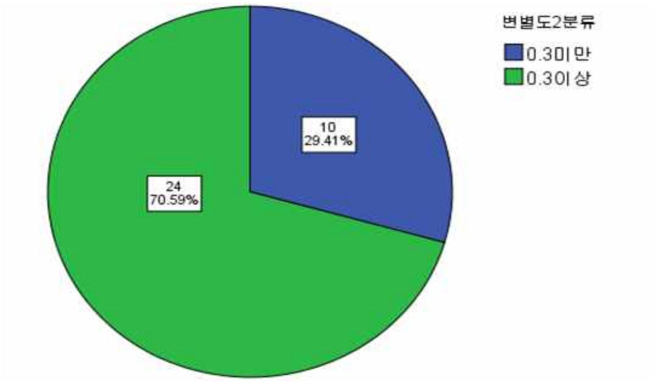

총점	변별도2	표준편차
34	.38	.14

변별도2	문항수	비율(%)
0.3미만	10	29.4
0.3이상	24	70.6
전체	34	100.0

해석

- 암기형 문항에서 난이도 지수가 80 에서 100 사이인 문항이 전체 34 문항 중 23 문항 이였으며, 다음으로 60 이상 80 미만인 문항이 7 문항, 60 미만인 문항이 4 문항인 것으로 나타남
- 변별도 1 지수를 기준으로 분류하였을 때, 0.3 미만인 문항이 15 문항으로 0.3 이상인 문항이 19 문항인 것에 비해 더 적게 나타남
- 변별도 2 지수를 기준으로 분류하였을 때, 0.3 미만인 문항이 10 문항으로 0.3 이상인 문항이 24 문항인 것에 비해 더 적게 나타남

(2) 해석형 난이도와 변별도 분포도 및 비율분석

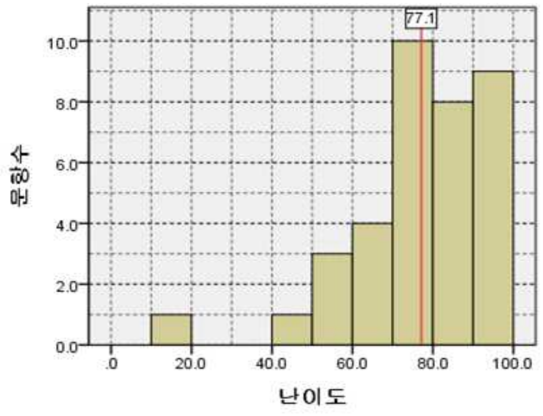

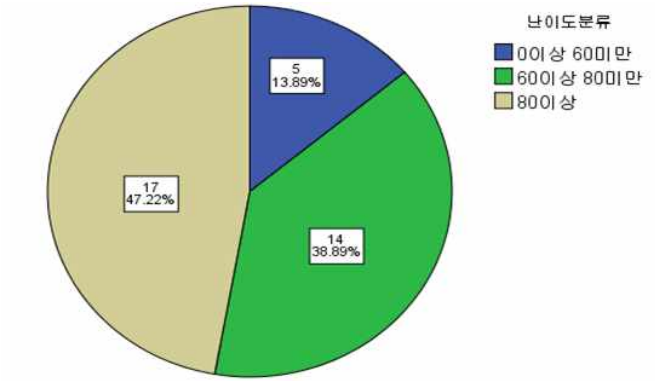

총점	난이도	표준편차
36	77.1	17.6

난이도	문항수	비율(%)
0~60미만	5	13.9
60~80미만	14	38.9
80~100	17	47.2
전체	36	100.0

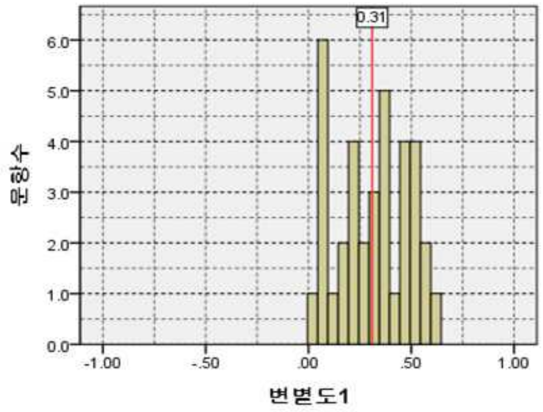

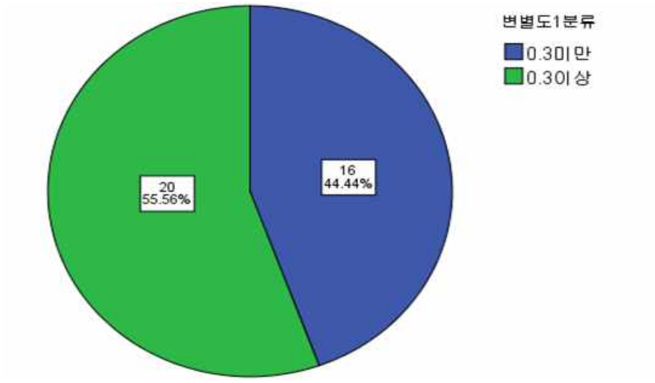

총점	변별도1	표준편차
36	.31	.17

변별도1	문항수	비율(%)
0.3미만	16	44.4
0.3이상	20	55.6
전체	36	100.0

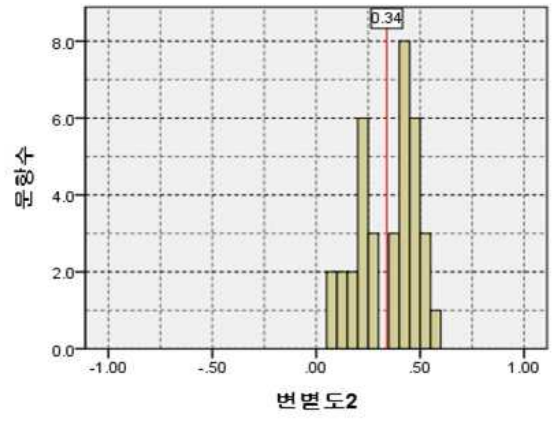

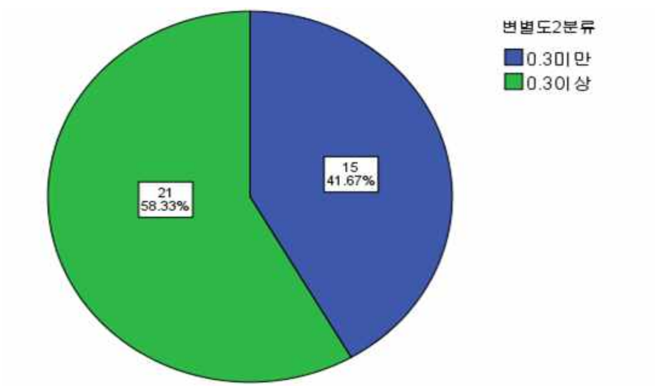

총점	변별도2	표준편차
36	.34	.14

변별도2	문항수	비율(%)
0.3미만	15	41.7
0.3이상	21	58.3
전체	36	100.0

해석

- 해석형 문항에서 난이도 지수가 80 에서 100 사이인 문항이 전체 36 문항 중 17 문항이었으며, 다음으로 60 이상 80 미만인 문항이 14 문항, 60 미만인 문항이 5 문항인 것으로 나타남
- 변별도 1 지수를 기준으로 분류하였을 때, 0.3 미만인 문항이 16 문항으로 0.3 이상인 문항이 20 문항인 것에 비해 더 적게 나타남
- 변별도 2 지수를 기준으로 분류하였을 때, 0.3 미만인 문항이 15 문항으로 0.3 이상인 문항이 21 문항인 것에 비해 더 적게 나타남

(3) 해결형 난이도와 변별도 분포도 및 비율분석

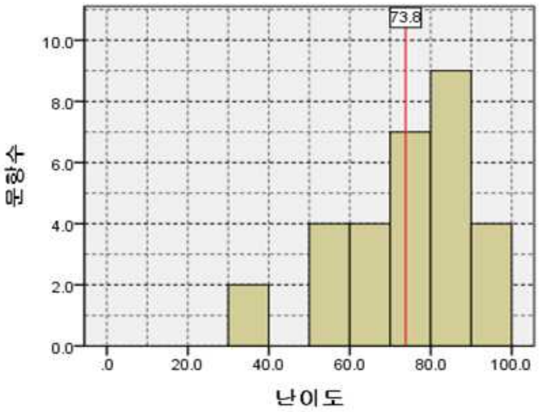

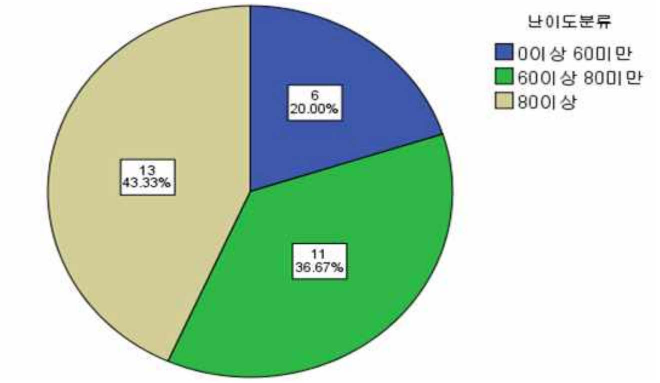

총점	난이도	표준편차
30	73.8	15.3

난이도	문항수	비율(%)
0~60미만	6	20.0
60~80미만	11	36.7
80~100	13	43.3
전체	30	100.0

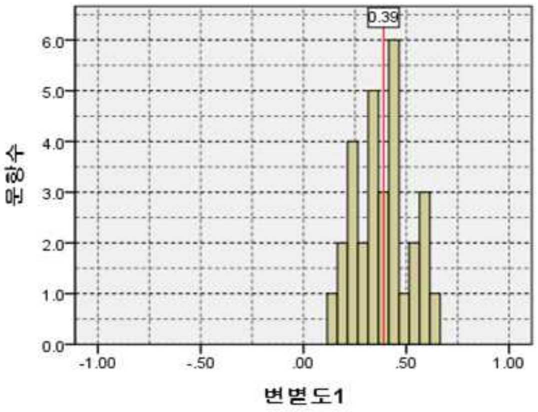

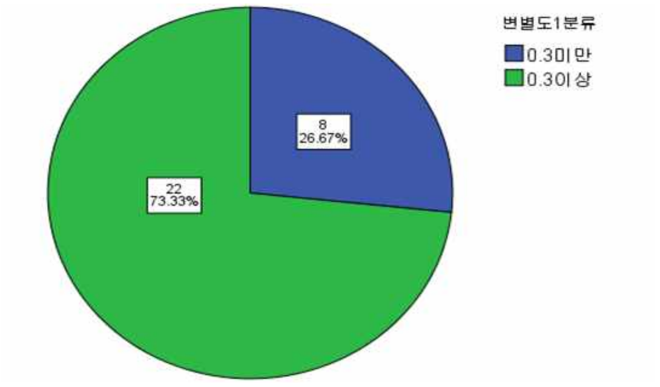

총점	변별도1	표준편차
30	.39	.13

변별도1	문항수	비율(%)
0.3미만	8	26.7
0.3이상	22	73.3
전체	30	100.0

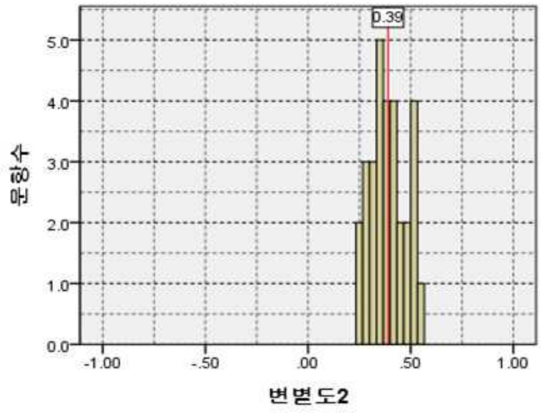

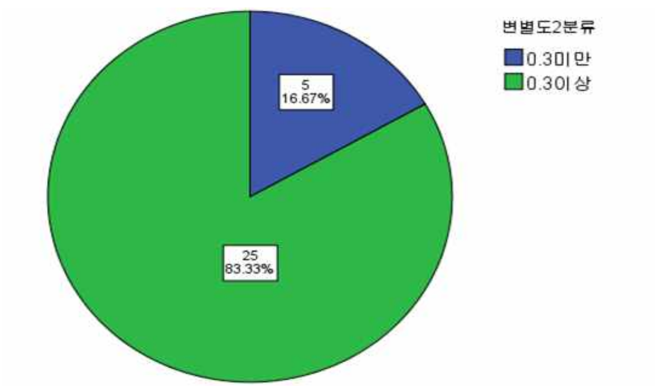

총점	변별도2	표준편차
30	.39	.09

변별도2	문항수	비율(%)
0.3미만	5	16.7
0.3이상	25	83.3
전체	30	100.0

해석

- 해결형 문항에서 난이도 지수가 80에서 100 사이인 문항이 전체 30문항 중 13문항으로 가장 많았으며, 다음으로 60이상 80미만인 문항이 11문항, 60 미만인 문항이 6문항인 것으로 나타남
- 변별도1 지수를 기준으로 분류하였을 때, 0.3미만인 문항이 8문항으로 0.3이상인 문항이 22문항인 것에 비해 더 많게 나타남
- 변별도2 지수를 기준으로 분류하였을 때, 0.3미만인 문항이 5문항으로 0.3이상인 문항이 25문항인 것에 비해 더 적게 나타남

4) 자료유형별 난이도와 변별도

가) 전회 대비 자료유형별 난이도와 변별도

(1) 전회 대비 텍스트형 난이도와 변별도

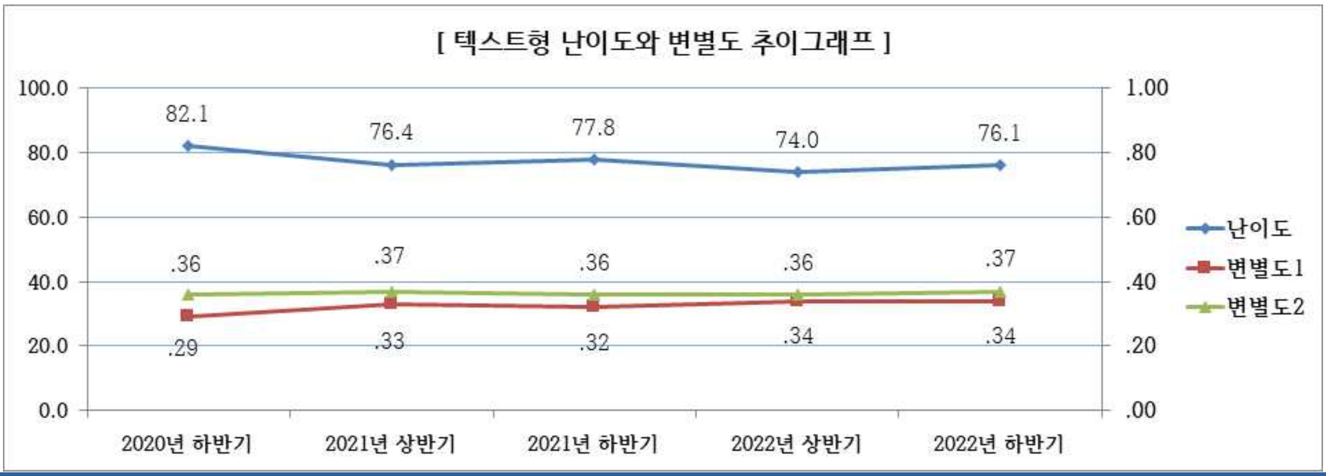

회차	난이도		변별도1		변별도2	
	평균	표준편차	평균	표준편차	평균	표준편차
2020년 하반기	82.1	14.6	0.29	0.17	0.36	0.13
2021년 상반기	76.4	17.1	0.33	0.16	0.37	0.12
2021년 하반기	77.8	16.0	0.32	0.18	0.36	0.13
2022년 상반기	74.0	20.1	0.34	0.19	0.36	0.13
**22년 하반기**	**76.1**	**18.4**	**0.34**	**0.16**	**0.37**	**0.13**

(2) 전회 대비 자료제시형 난이도와 변별도

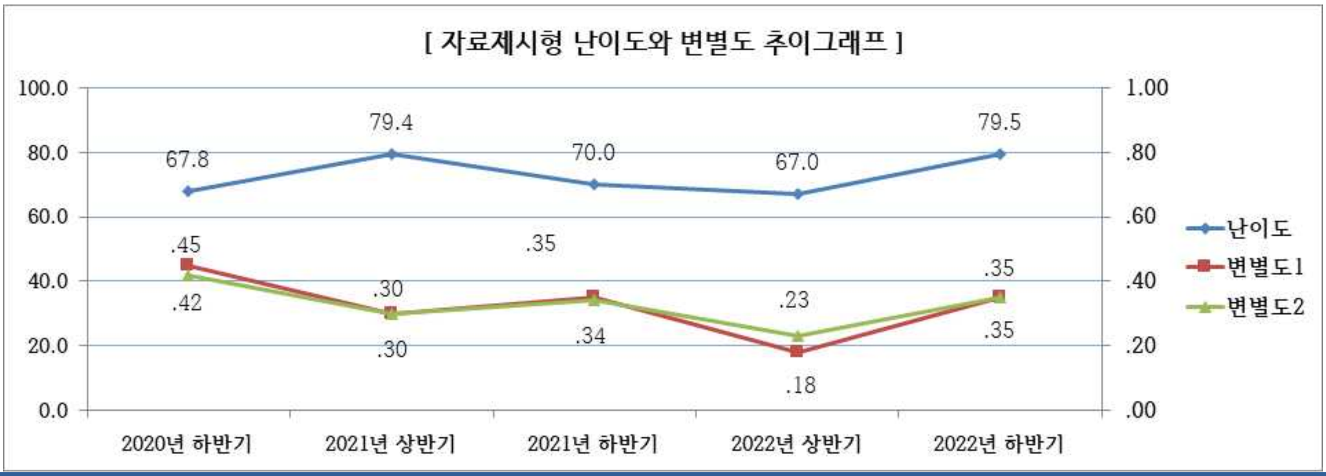

회차	난이도		변별도1		변별도2	
	평균	표준편차	평균	표준편차	평균	표준편차
2020년 하반기	67.8	22.9	0.45	0.14	0.42	0.01
2021년 상반기	79.4	22.6	0.30	0.30	0.30	0.17
2021년 하반기	70.0	21.7	0.35	0.10	0.34	0.00
2022년 상반기	67.0	40.6	0.18	0.08	0.23	0.07
**22년 하반기**	**79.5**	**19.3**	**0.35**	**0.32**	**0.35**	**0.19**

해석

- 전회 대비 텍스트형 및 자료제시형 문항의 난이도 지수는 각각 2.1, 12.5 증가함
- 텍스트형의 변별도 1 지수는 변화 없었으며, 변별도 2 지수는 .01 증가함
- 자료제시형의 변별도 1 지수와 변별도 2 지수는 각각 .17, .12 증가함

나) 자료유형별 난이도와 변별도 분포도 및 비율분석

(1) 텍스트형 난이도와 변별도 분포도 및 비율분석

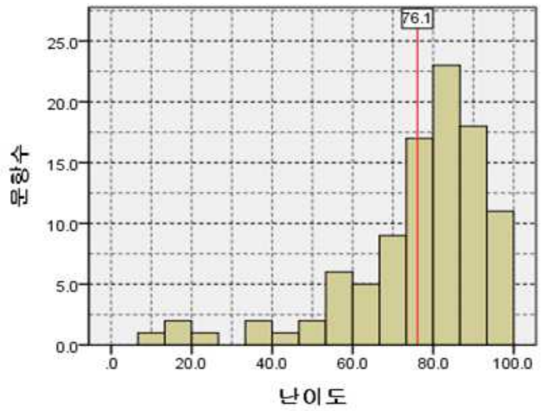

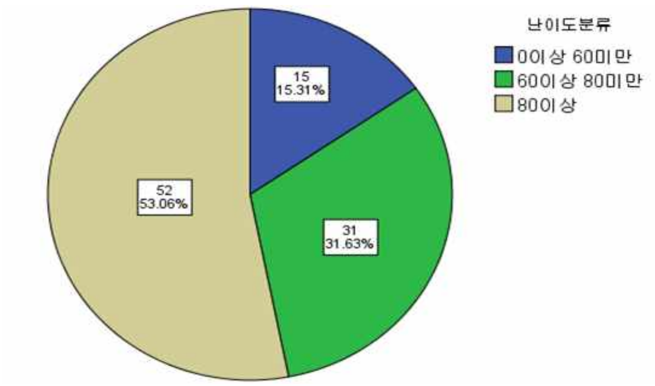

총점	난이도	표준편차
98	76.1	18.4

난이도	문항수	비율(%)
0~60미만	15	15.3
60~80미만	31	31.6
80~100	52	53.1
전체	98	100.0

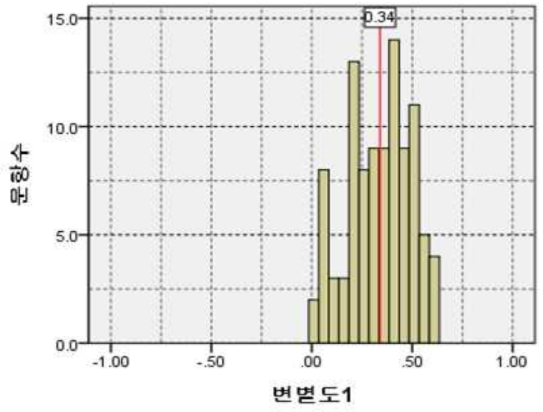

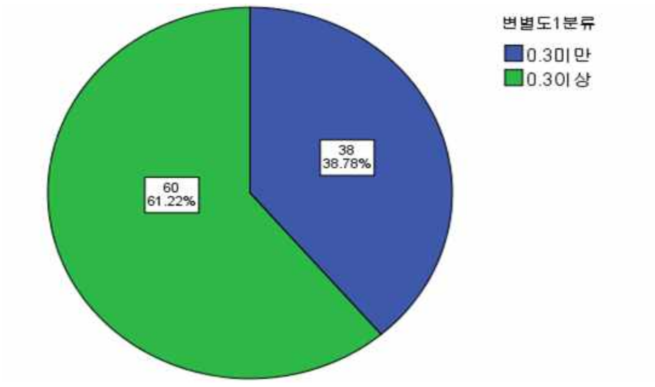

총점	변별도1	표준편차
98	.34	.16

변별도1	문항수	비율(%)
0.3미만	38	38.8
0.3이상	60	61.2
전체	98	100.0

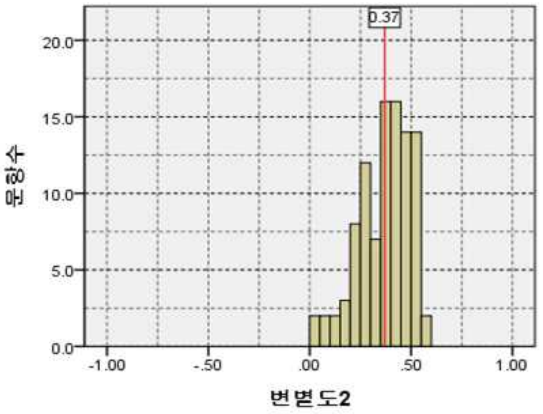

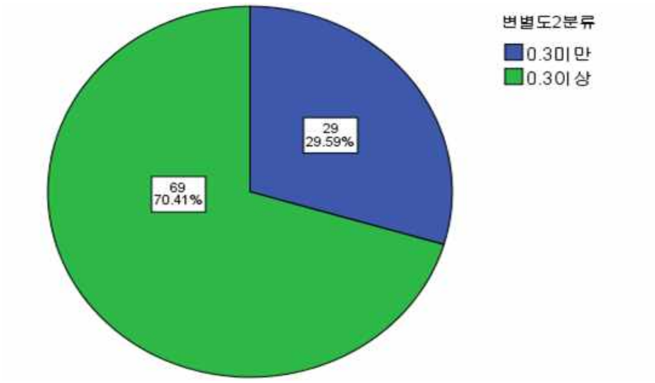

총점	변별도2	표준편차	변별도2	문항수	비율(%)
98	.37	.13	0.3미만	29	29.6
			0.3이상	69	70.4
			전체	98	100.0

해석

- 텍스트형 문항에서 난이도 지수가 80 에서 100 사이인 문항이 전체 98 문항 중 52 문항으로 가장 많았으며, 다음으로 60 이상 80 미만인 문항이 31 문항, 60 미만인 문항이 15 문항인 것으로 나타남
- 변별도 1 지수를 기준으로 분류하였을 때, 0.3 미만인 문항이 38 문항으로 0.3 이상인 문항이 60 문항으로 더 적게 나타남
- 변별도 2 지수를 기준으로 분류하였을 때, 0.3 미만인 문항이 29 문항으로 0.3 이상인 문항이 69 문항에 비해 더 적게 나타남

(2) 자료제시형 난이도와 변별도 분포도 및 비율분석

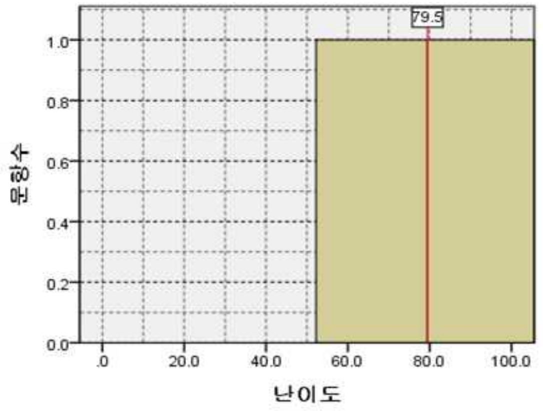

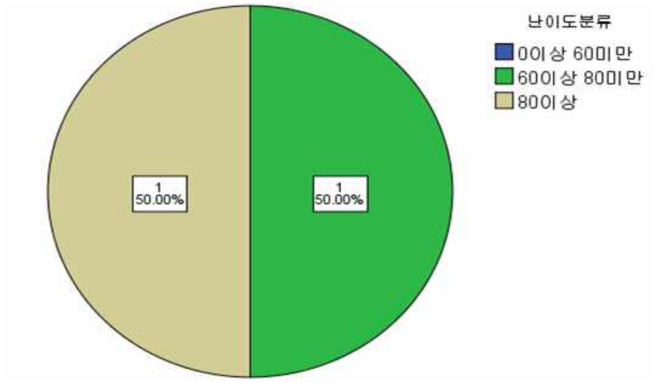

총점	난이도	표준편차
2	79.5	19.3

난이도	문항수	비율(%)
0~60미만	-	-
60~80미만	1	50.0
80~100	1	50.0
전체	2	100.0

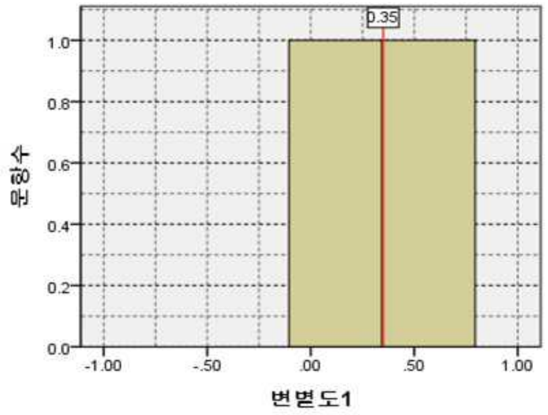

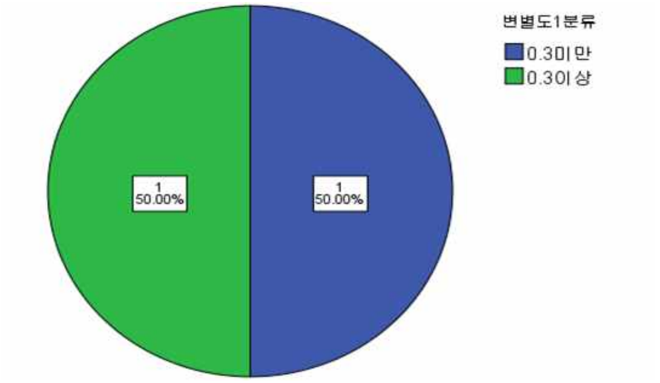

총점	변별도1	표준편차
2	.35	.32

변별도1	문항수	비율(%)
0.3미만	1	50.0
0.3이상	1	50.0
전체	2	100.0

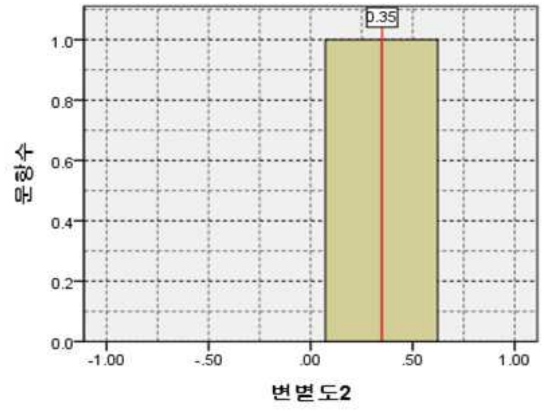

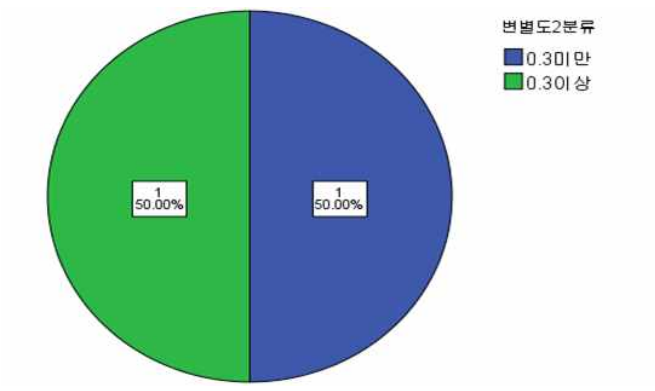

총점	변별도2	표준편차
2	.35	.19

변별도2	문항수	비율(%)
0.3미만	1	50.0
0.3이상	1	50.0
전체	2	100.0

해석

- 자료제시형 문항의 난이도 지수가 80 에서 100 사이인 문항이 1 문항, 60 이상 80 미만인 문항이 각 1 문항인 것으로 나타남
- 변별도 1 지수를 기준으로 분류하였을 때, 0.3 미만인 문항과 0.3 이상인 문항이 각각 1 문항으로 나타남
- 변별도 2 지수를 기준으로 분류하였을 때, 0.3 미만인 문항과 0.3 이상인 문항이 각각 1 문항으로 나타남

3. 난이도와 변별도 간 산포도

1) 전체 난이도와 변별도 간 산포도

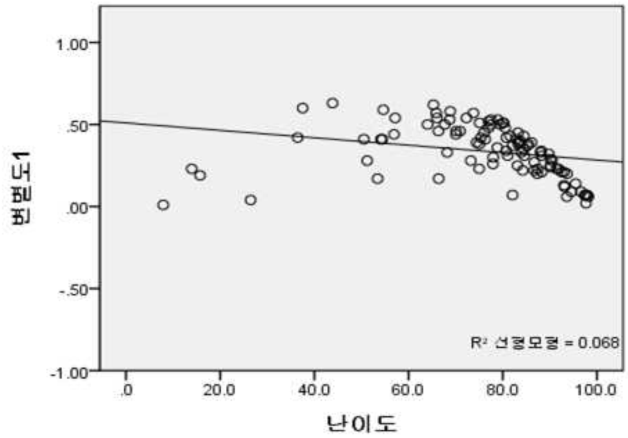

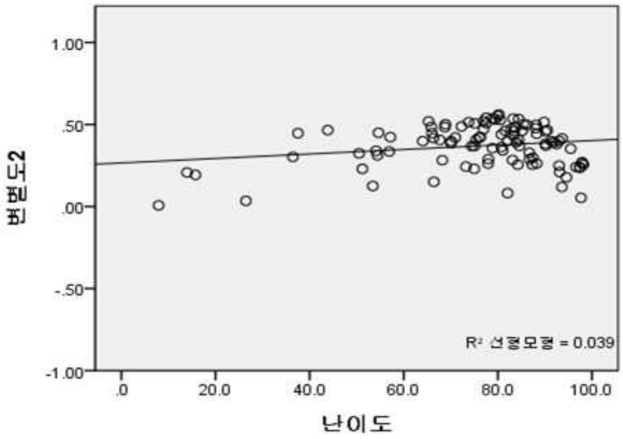

해석

- 전체 문항을 대상으로 난이도와 변별도 1 지수 간 상관은 -.260*로 문항 난이도가 쉬울수록 변별력이 낮아지는 것으로 나타남
- 난이도와 변별도 2 지수 간 상관은 .197 로 문항 난이도와 변별력 간 관련성이 낮은 것으로 나타남

2) 과목별 난이도와 변별도 간 산포도

가) 기초간호학개요 난이도와 변별도 간 산포도

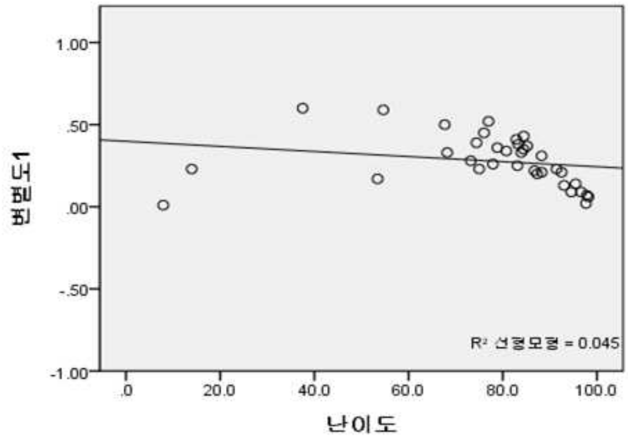

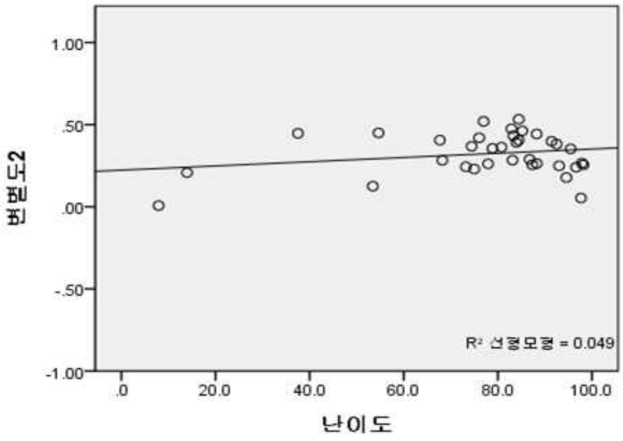

해석

- 기초간호학개론 과목 문항을 대상으로 난이도와 변별도1 지수 간 상관은 -.213으로 문항 난이도와 변별력 간 관련성이 낮은 것으로 나타남
- 난이도와 변별도2 지수 간 상관은 .221로 문항 난이도와 변별력 간 관련성이 낮은 것으로 나타남

나) 보건간호학개요 난이도와 변별도 간 산포도

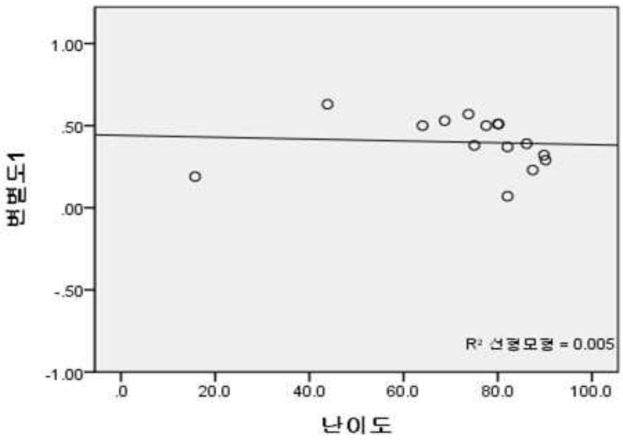

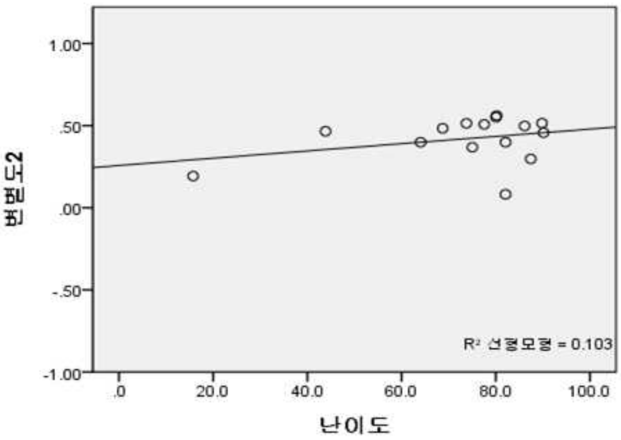

해석

- 보건간호학개요 과목 문항을 대상으로 난이도와 변별도1 지수 간 상관은 -.071로 문항 난이도와 변별력 간 관련성이 없는 것으로 나타남
- 난이도와 변별도2 지수 간 상관은 .321로 문항 난이도와 변별력 간 관련성이 낮은 것으로 나타남

다) 공중보건학개론 난이도와 변별도 간 산포도

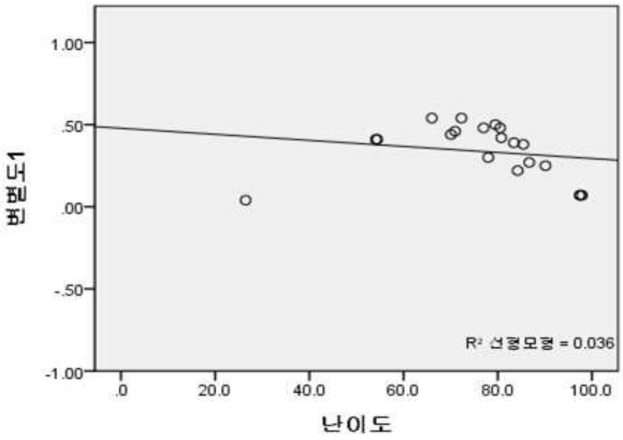

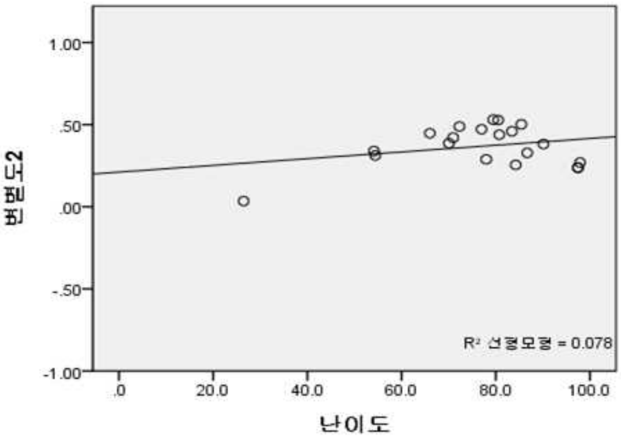

해석

- 공중보건학개론 과목 문항을 대상으로 난이도와 변별도1 지수 간 상관은 -.189로 문항 난이도와 변별력 간 관련성이 낮은 것으로 나타남
- 난이도와 변별도2 지수 간 상관은 .279로 문항 난이도와 변별력 간 관련성이 낮은 것으로 나타남

라) 실기 난이도와 변별도 간 산포도

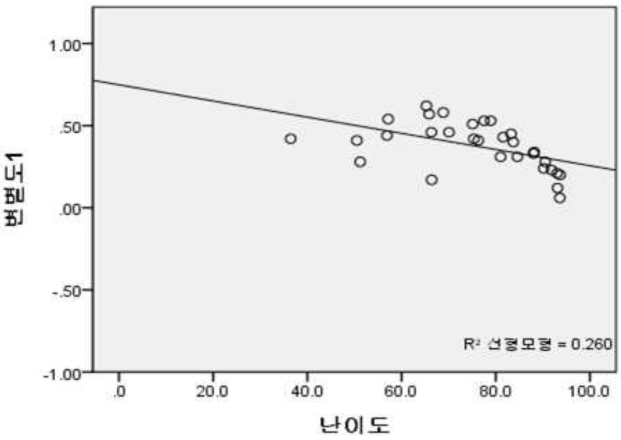

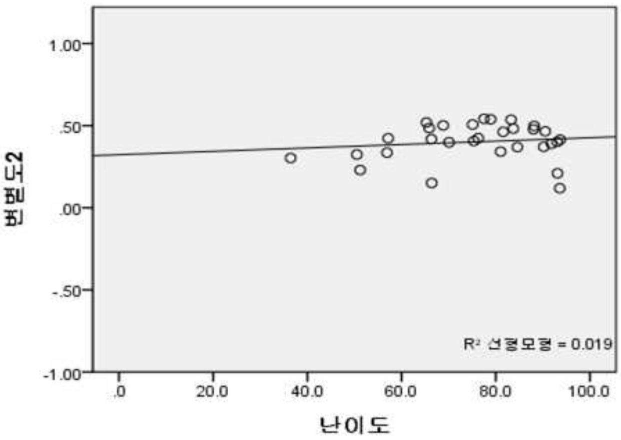

해석

- 실기 과목 문항을 대상으로 난이도와 변별도1 지수 간 상관은 -.510*으로 문항 난이도가 쉬울수록 변별력이 낮아지는 것으로 나타남
- 난이도와 변별도2 지수 간 상관은 .138로 문항 난이도와 변별력 간 관련성이 낮은 것으로 나타남

4. 신뢰도 분석

과목명	문항수	2020년 하반기	2021년 상반기	2021년 하반기	2022년 상반기	22년 하반기
**전체**	**100**	**.931**	**.933**	**.931**	.932	.935
기초간호학개요	35	.786	.808	.843	.805	**.789**
보건간호학개요	15	.661	.656	.664	.740	**.750**
공중보건학개론	20	.751	.775	.737	.697	**.744**
실기	30	.838	.830	.792	.832	**.841**

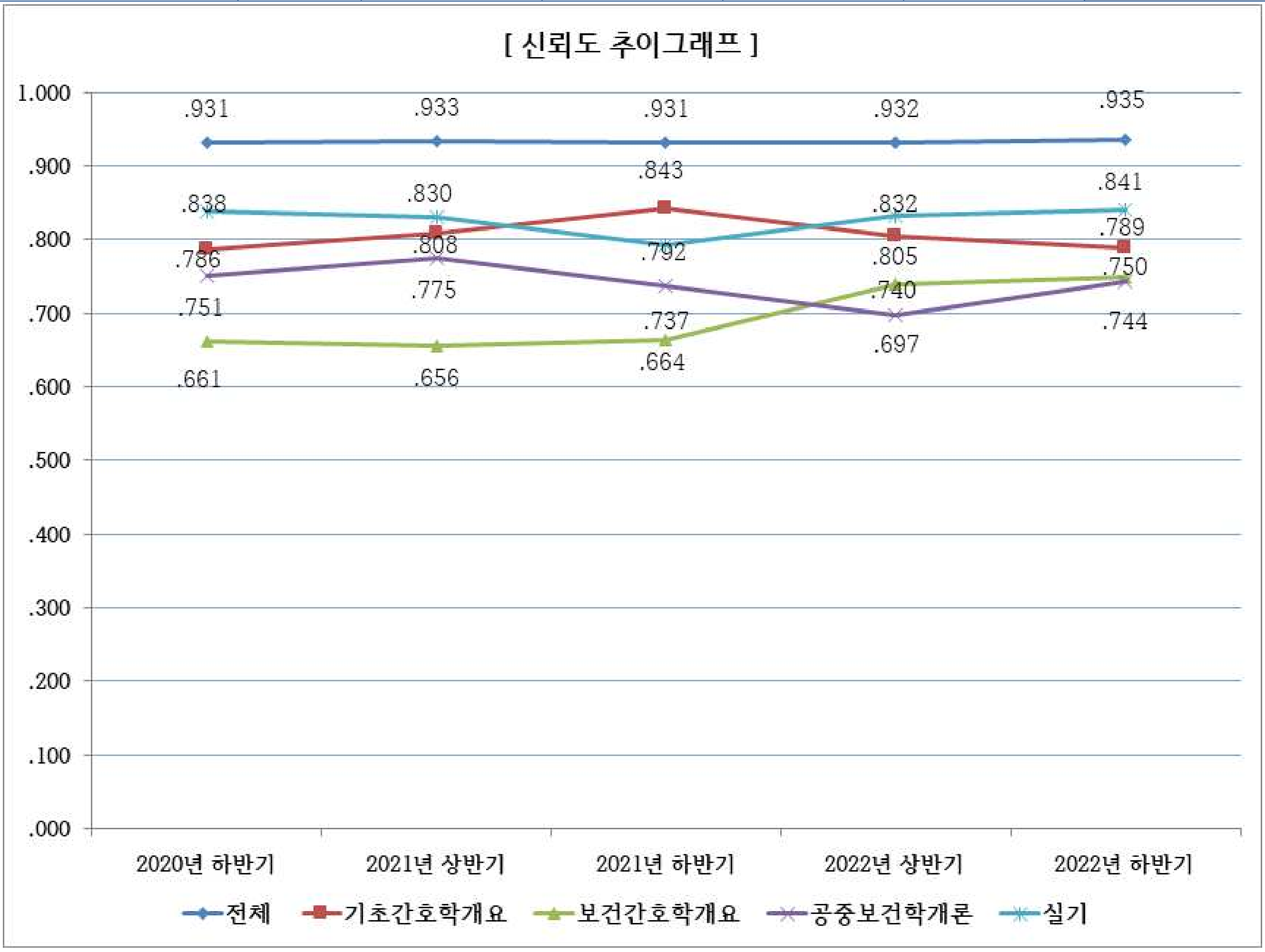

해석

- 간호조무사 자격시험 전체 문항에서 일관되게 해당 영역을 측정하고 있는 것으로 나타남
- 시험 전체, 보건간호학 개요, 공중보건학개론, 실기 과목을 대상으로 했을 때, 전회대비 신뢰도는 각각 .003, .010, .047, .009 증가함
- 기초간호학개요 과목을 대상으로 했을 때, 전회 대비 신뢰도는 .016 감소함.
